# Supplementary figures and images for: Distinct Cohorts of Aspergillus fumigatus Transcription Factors Are Required for Epithelial Damage Occurring via Contact- or Soluble Effector-Mediated Mechanisms
Source: Front Cell Infect Microbiol. 2022 Jul 28;12:907519. doi: 10.3389/fcimb.2022.907519 (PMC9379863; doi:10.3389/fcimb.2022.907519)

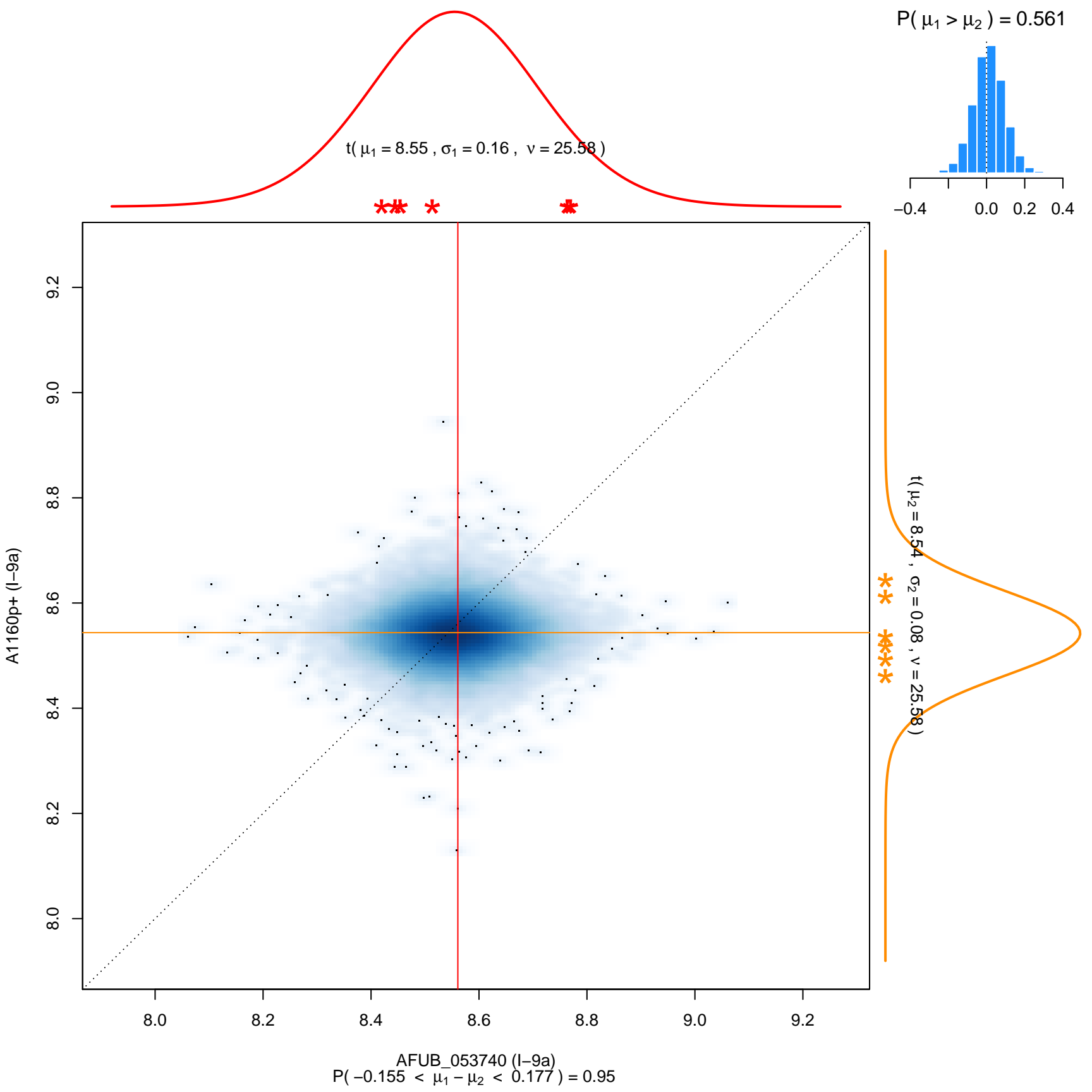

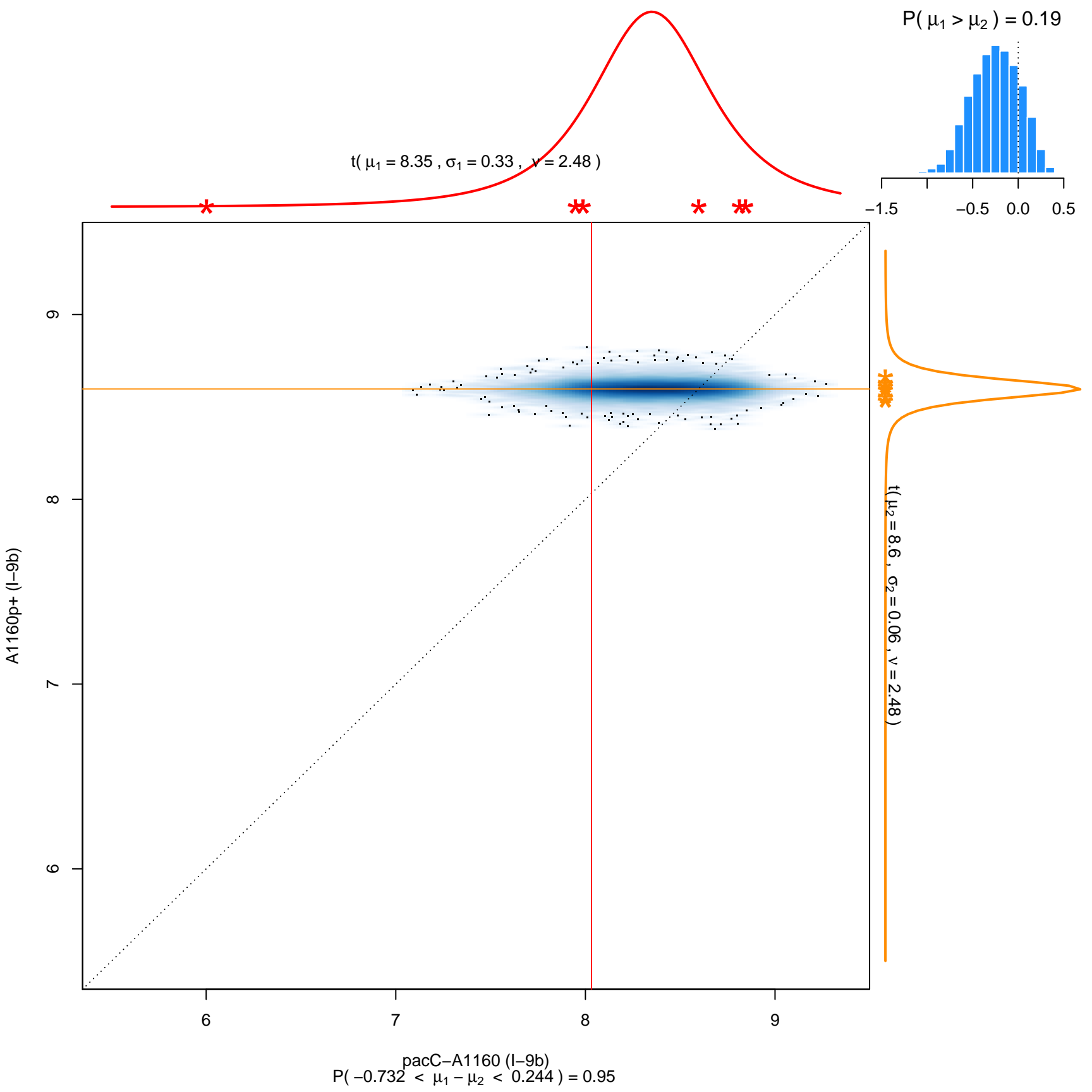

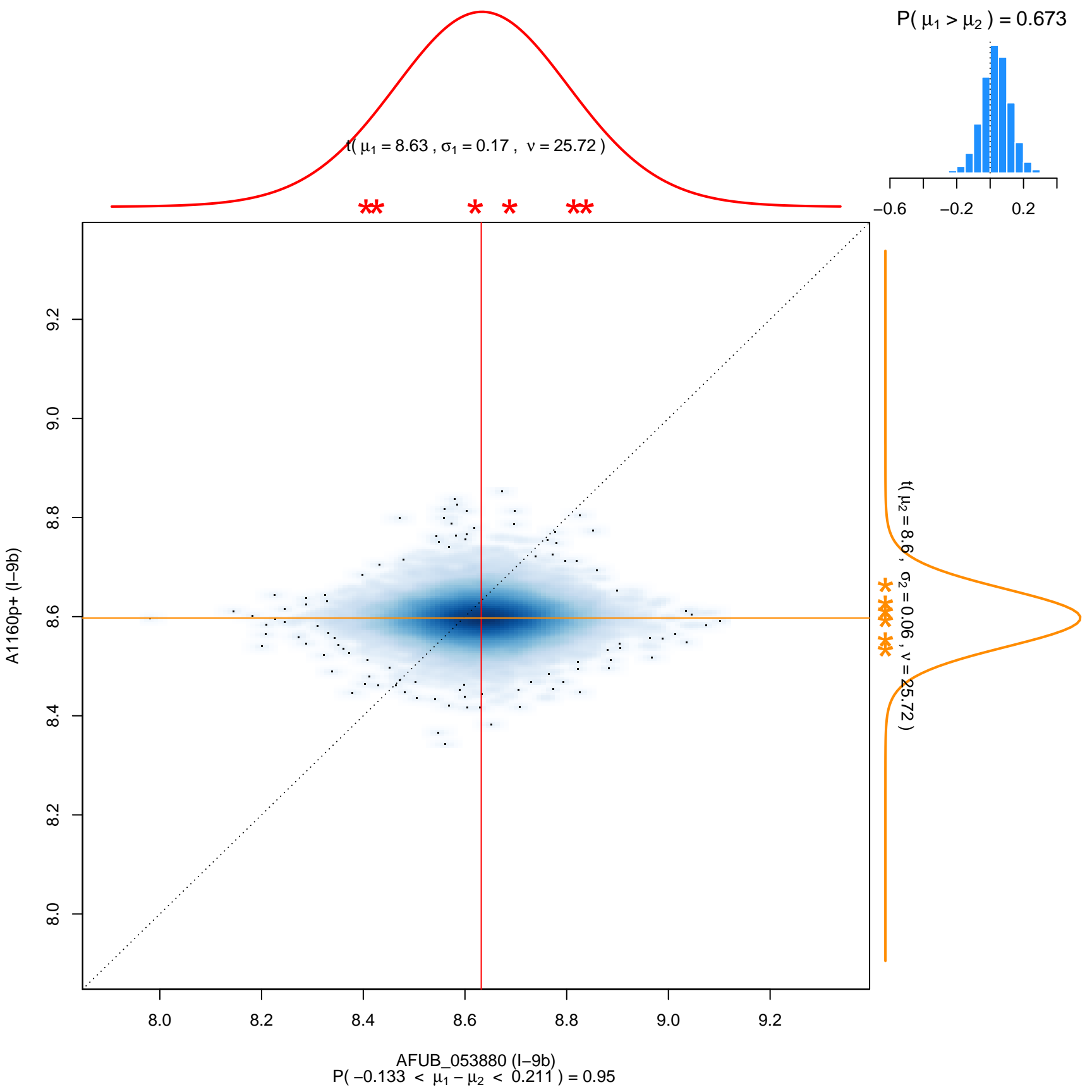

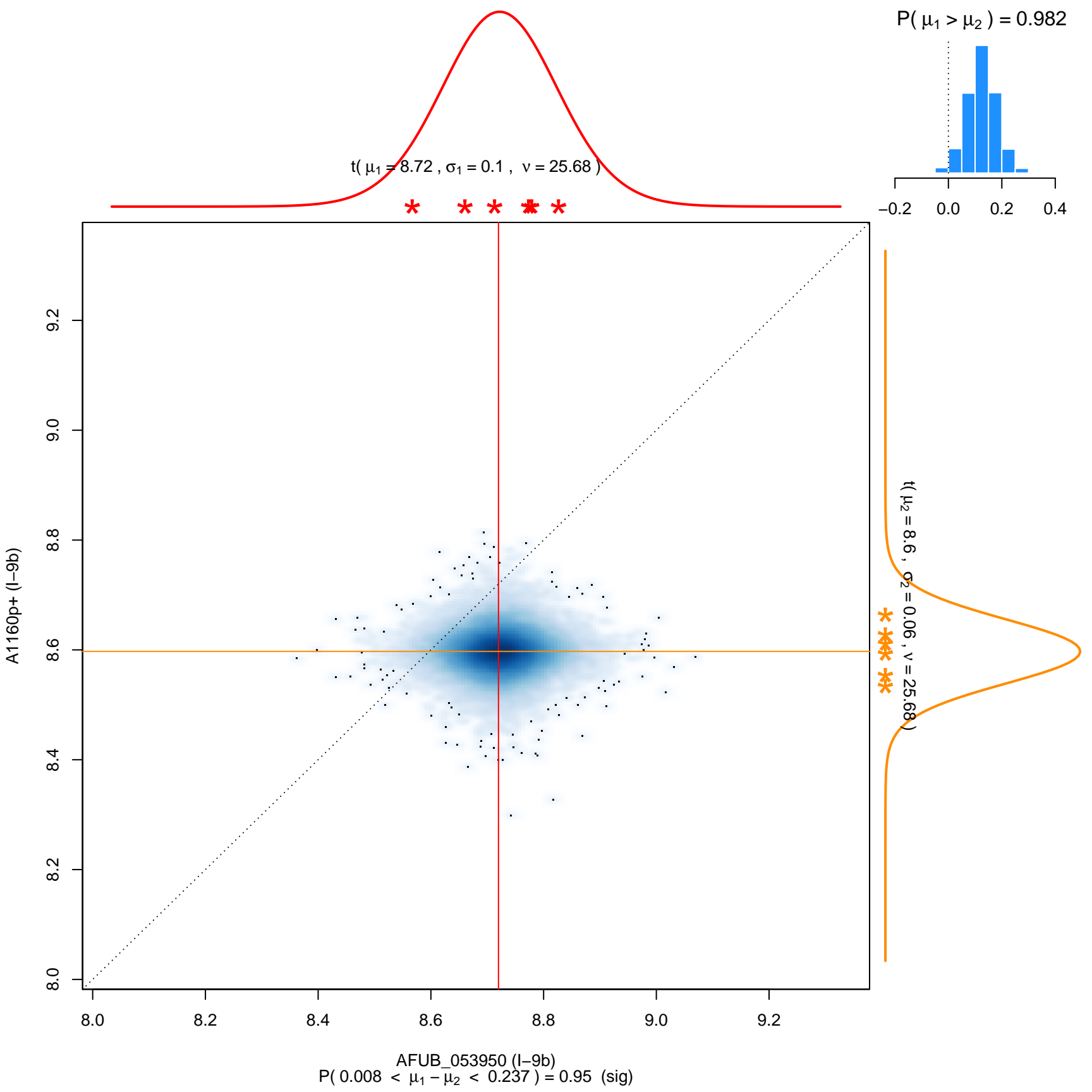

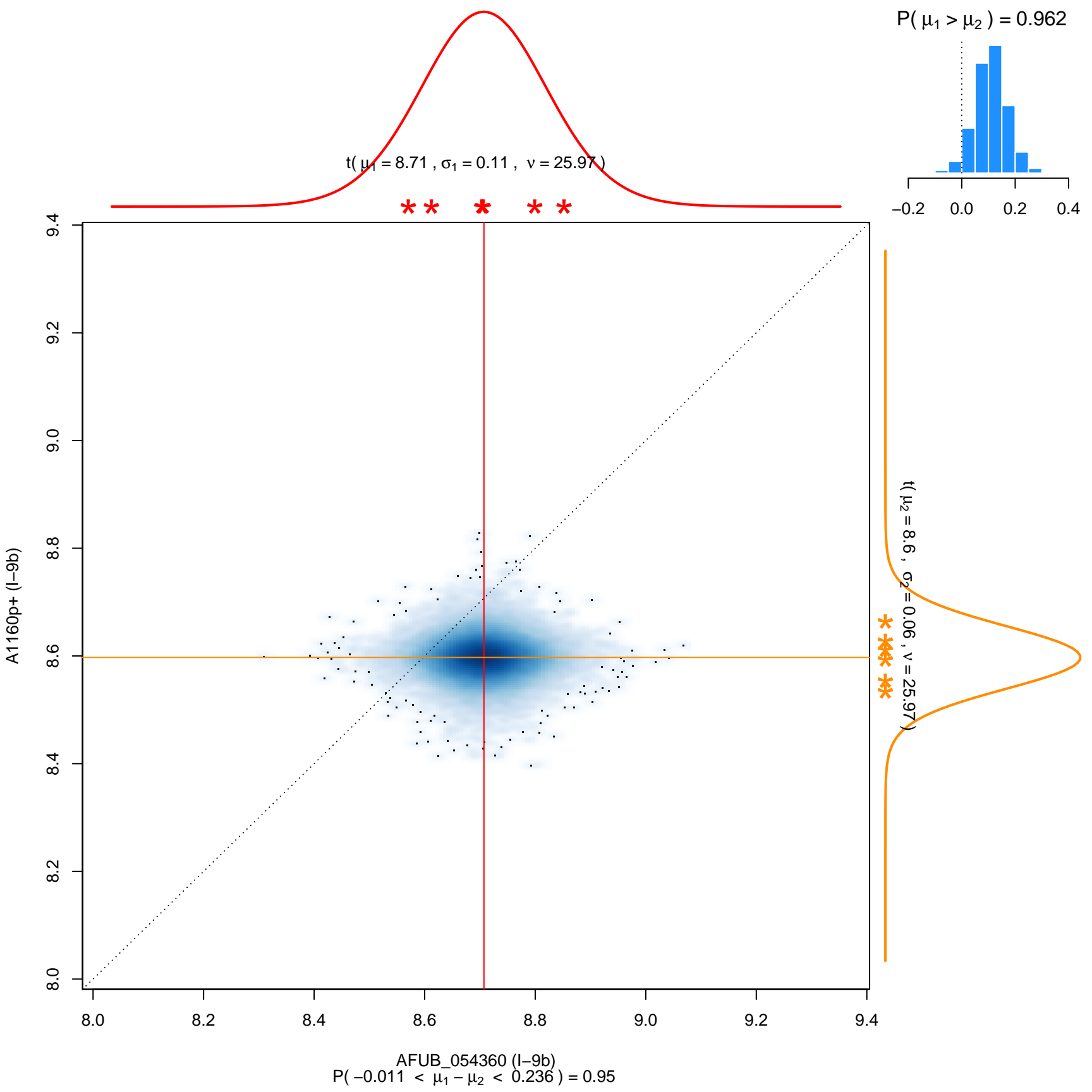

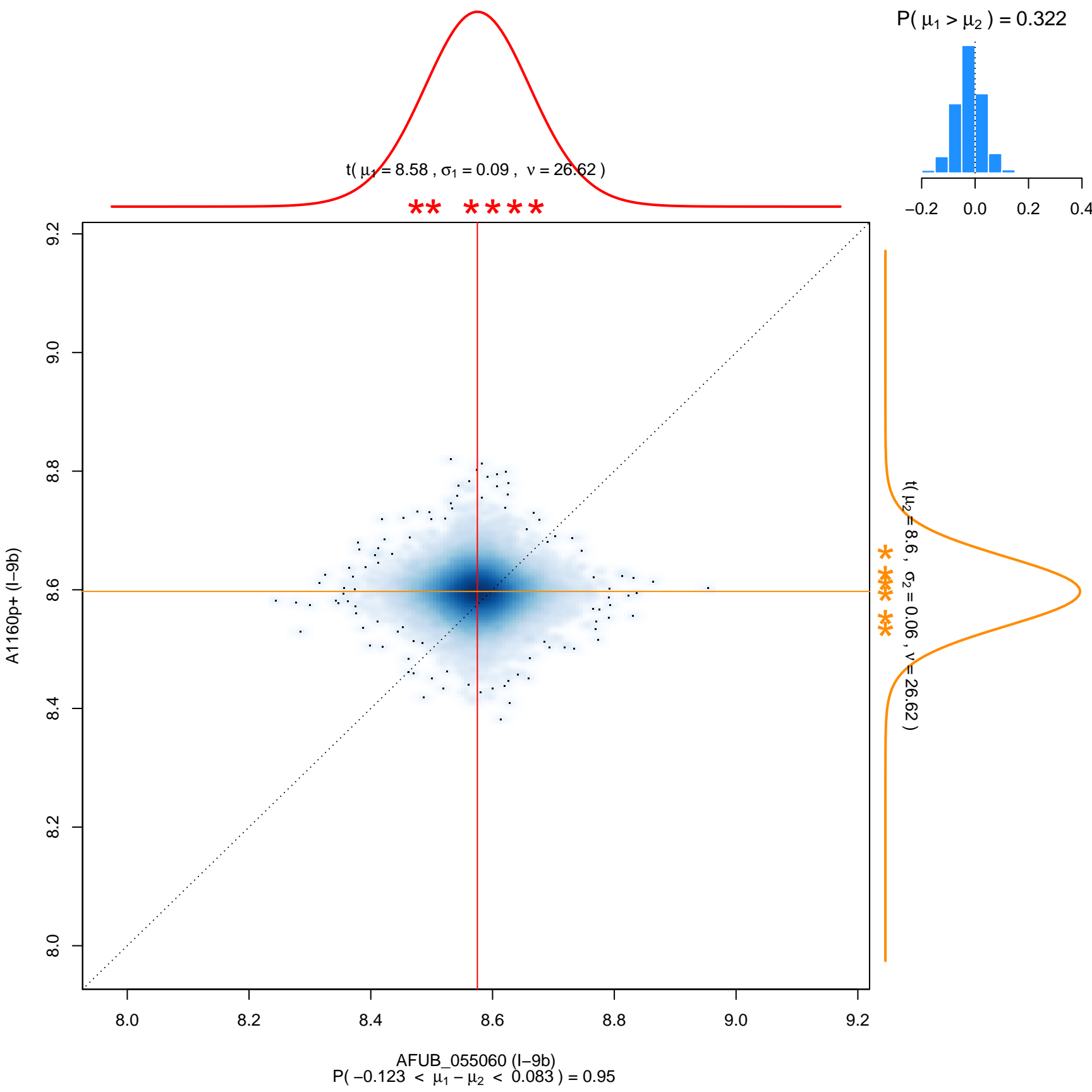

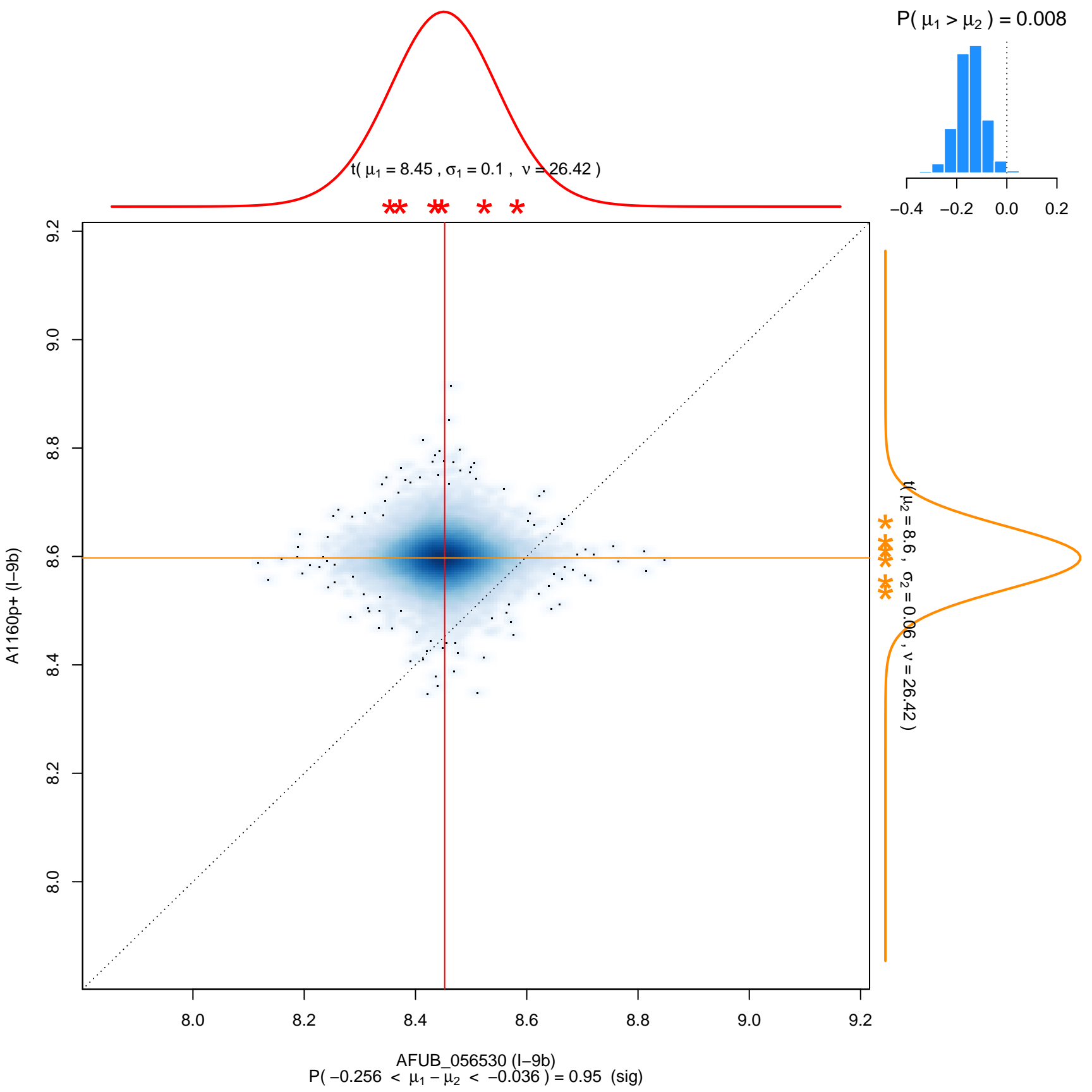

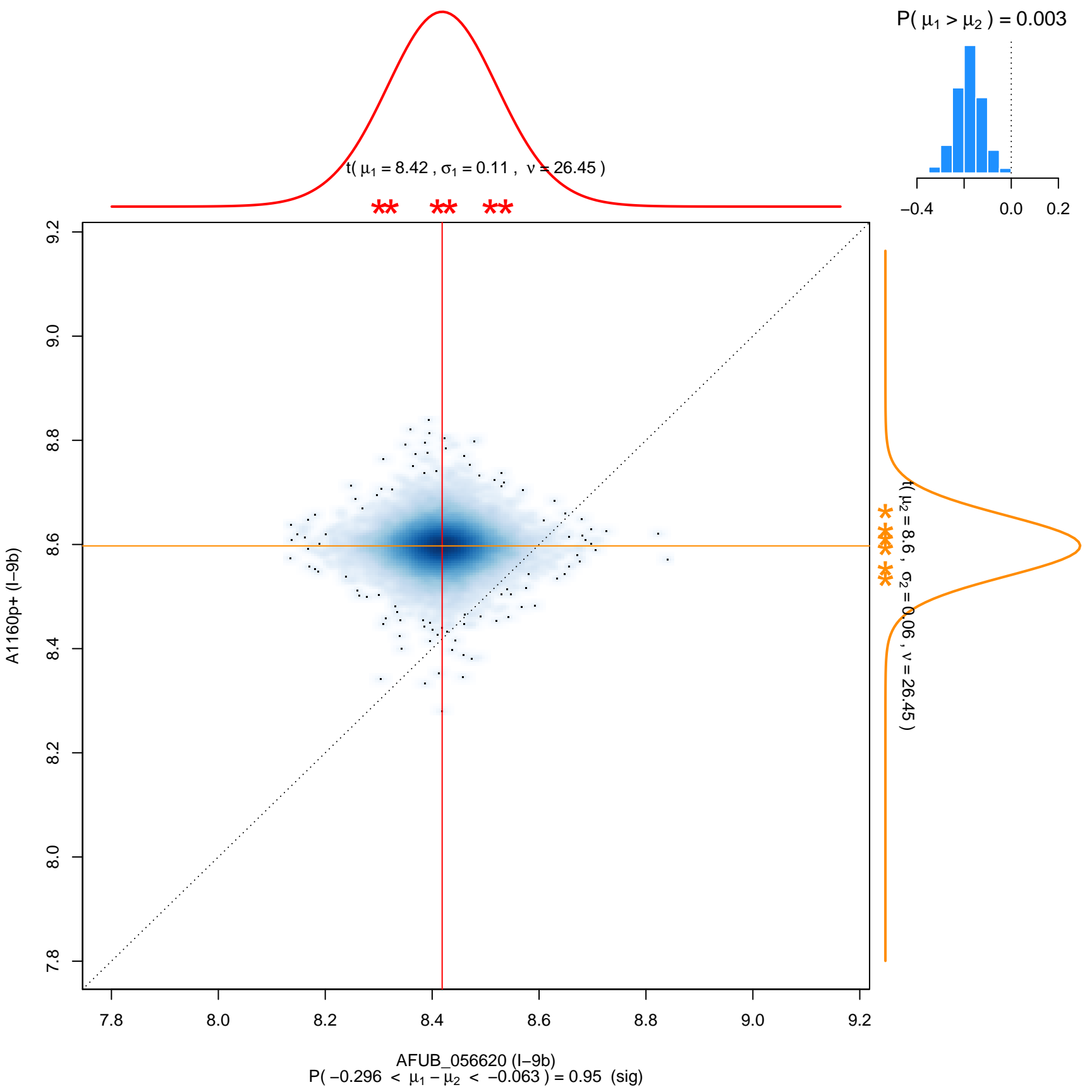

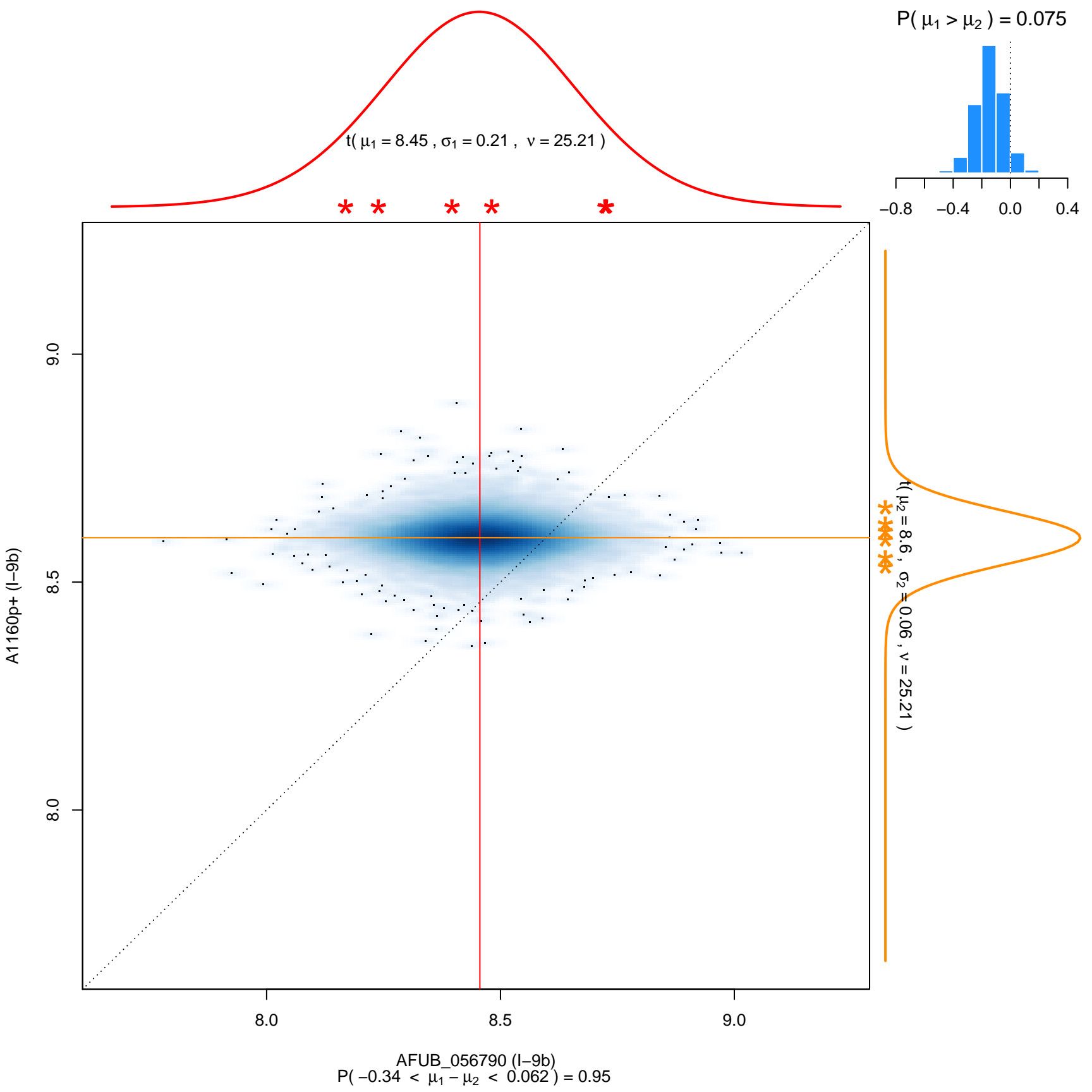

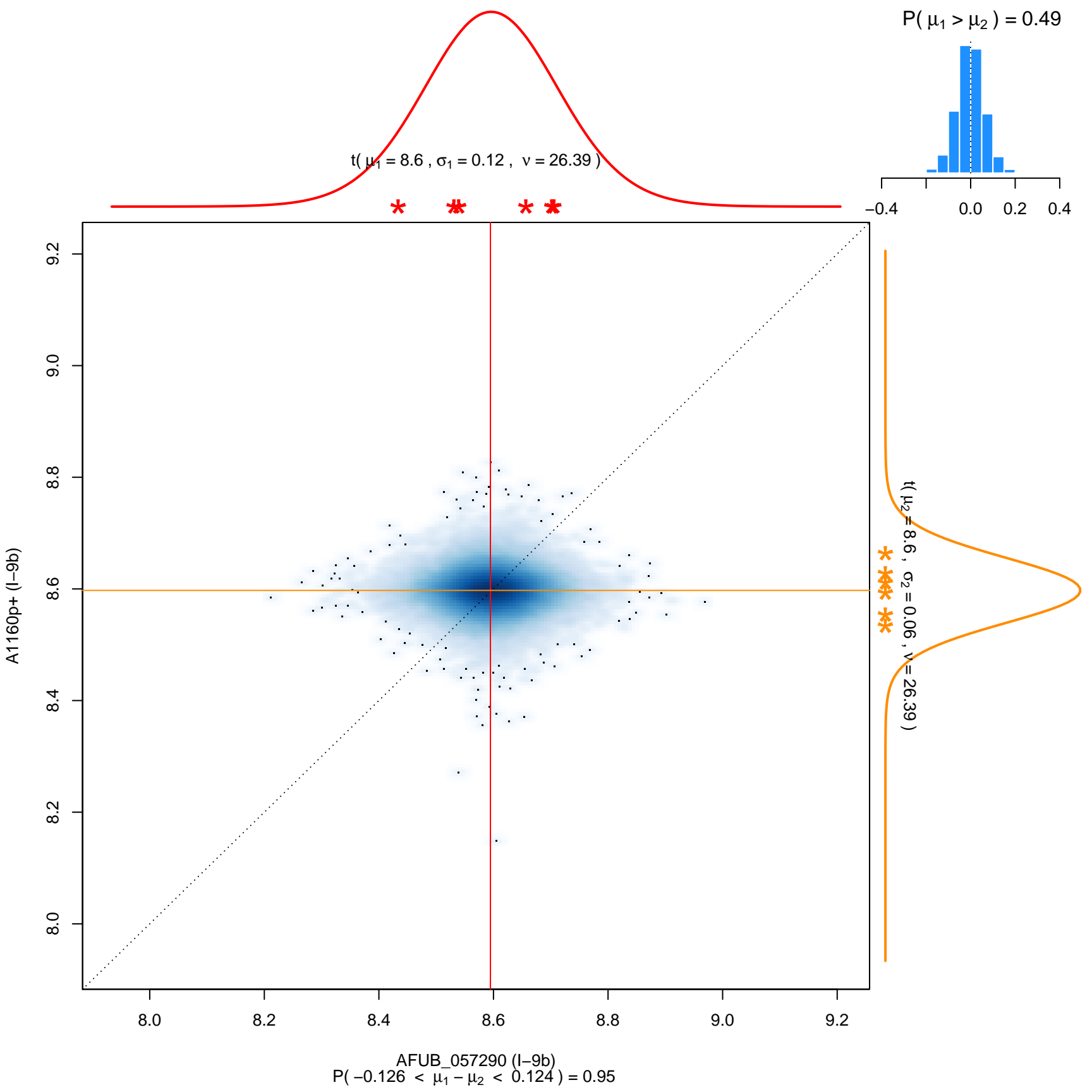

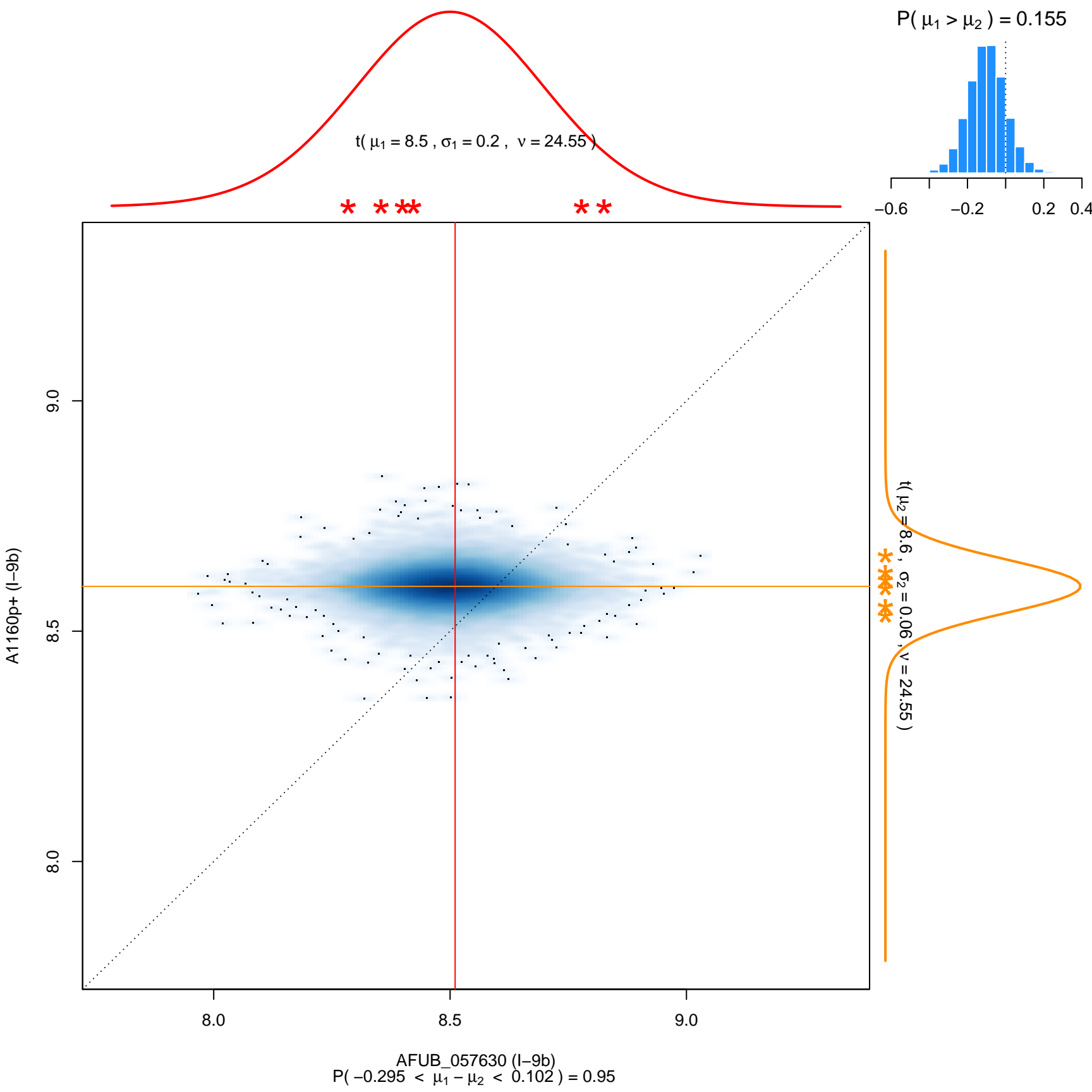

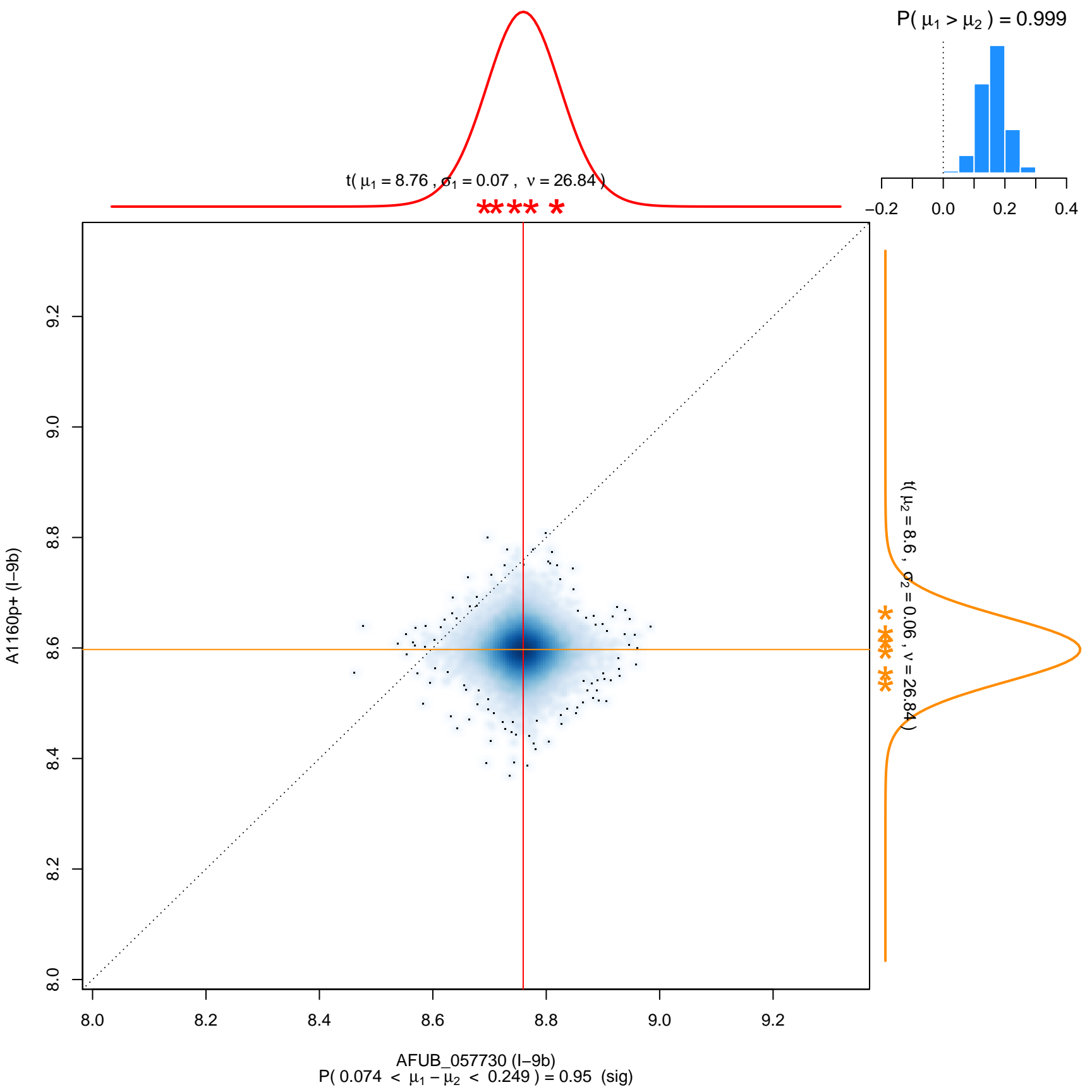

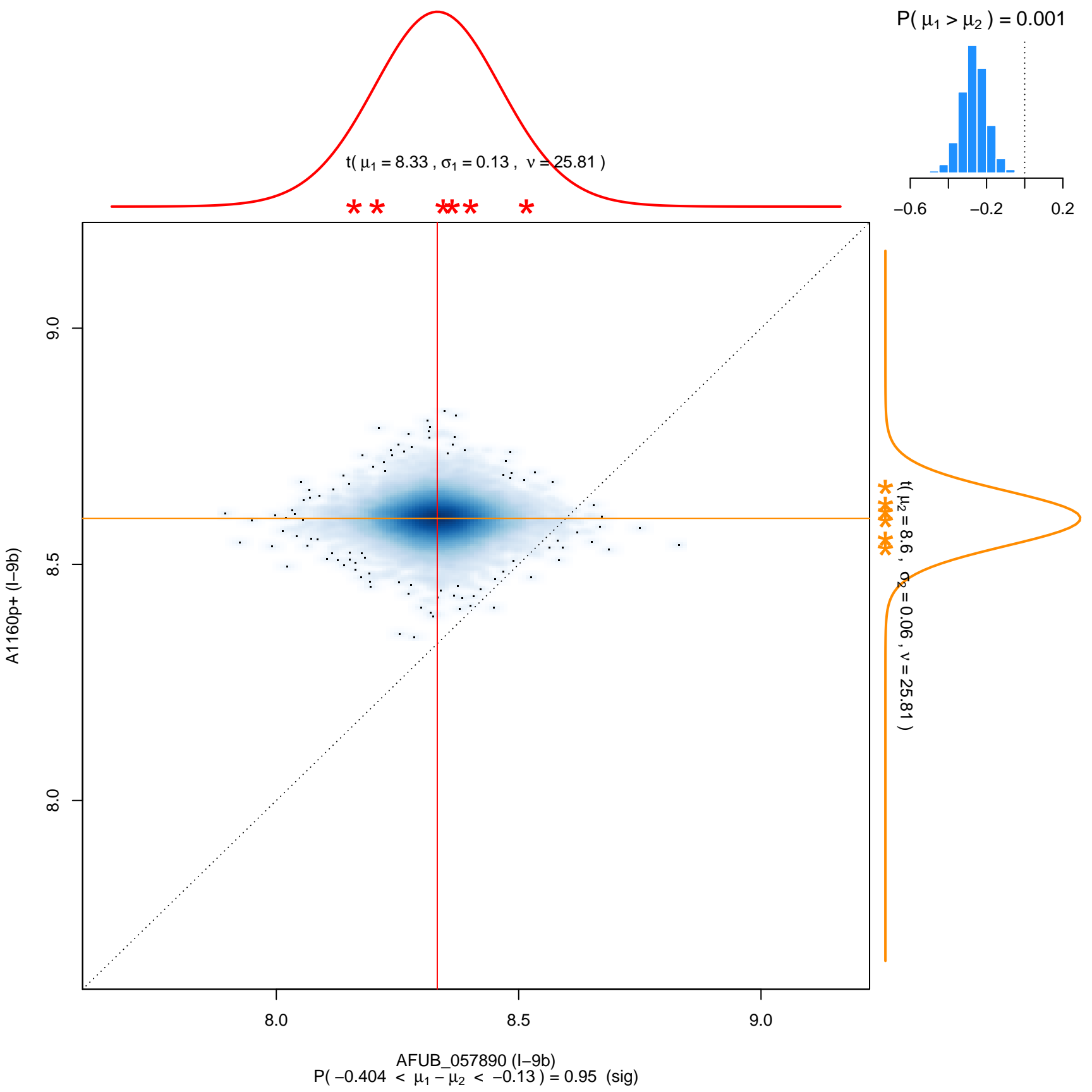

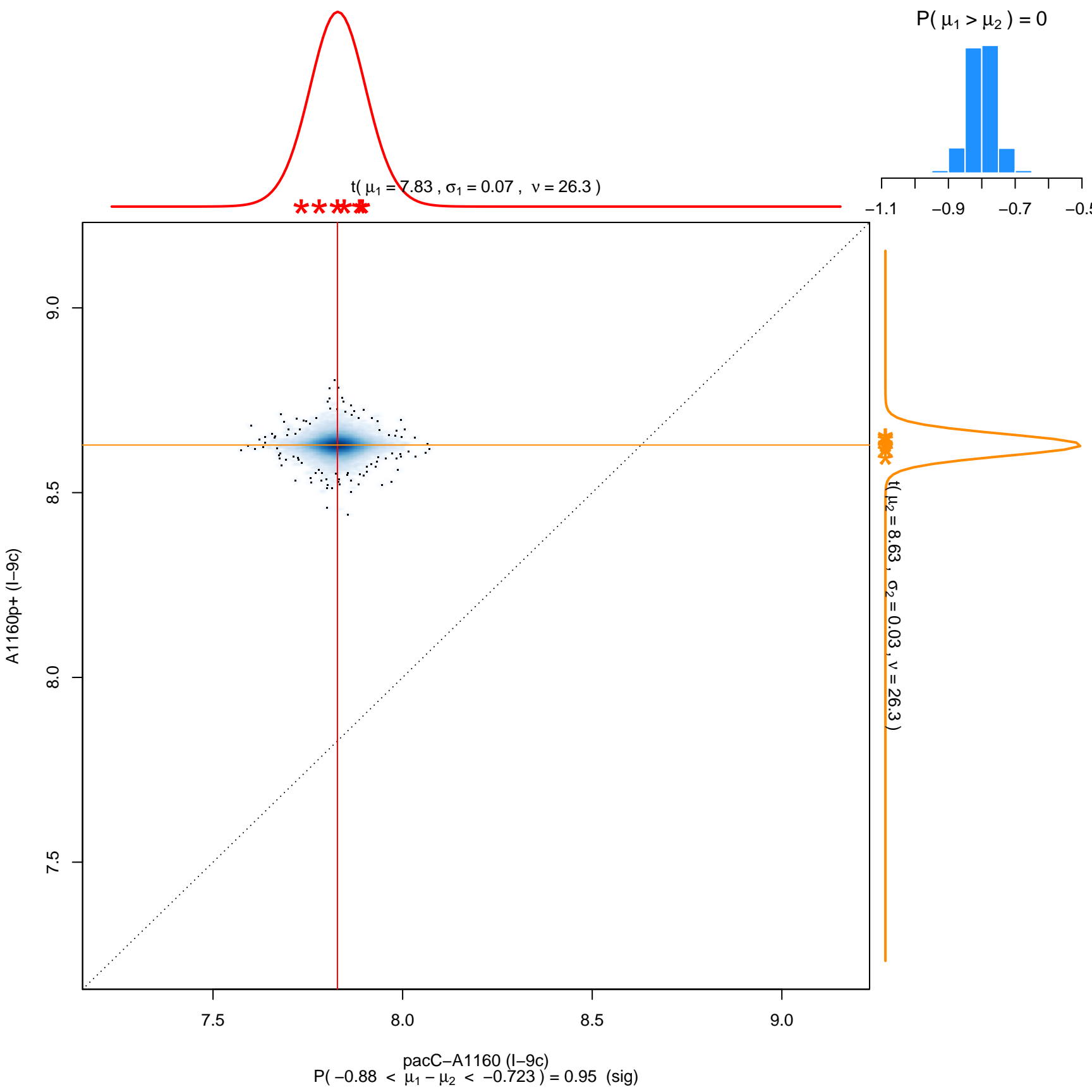

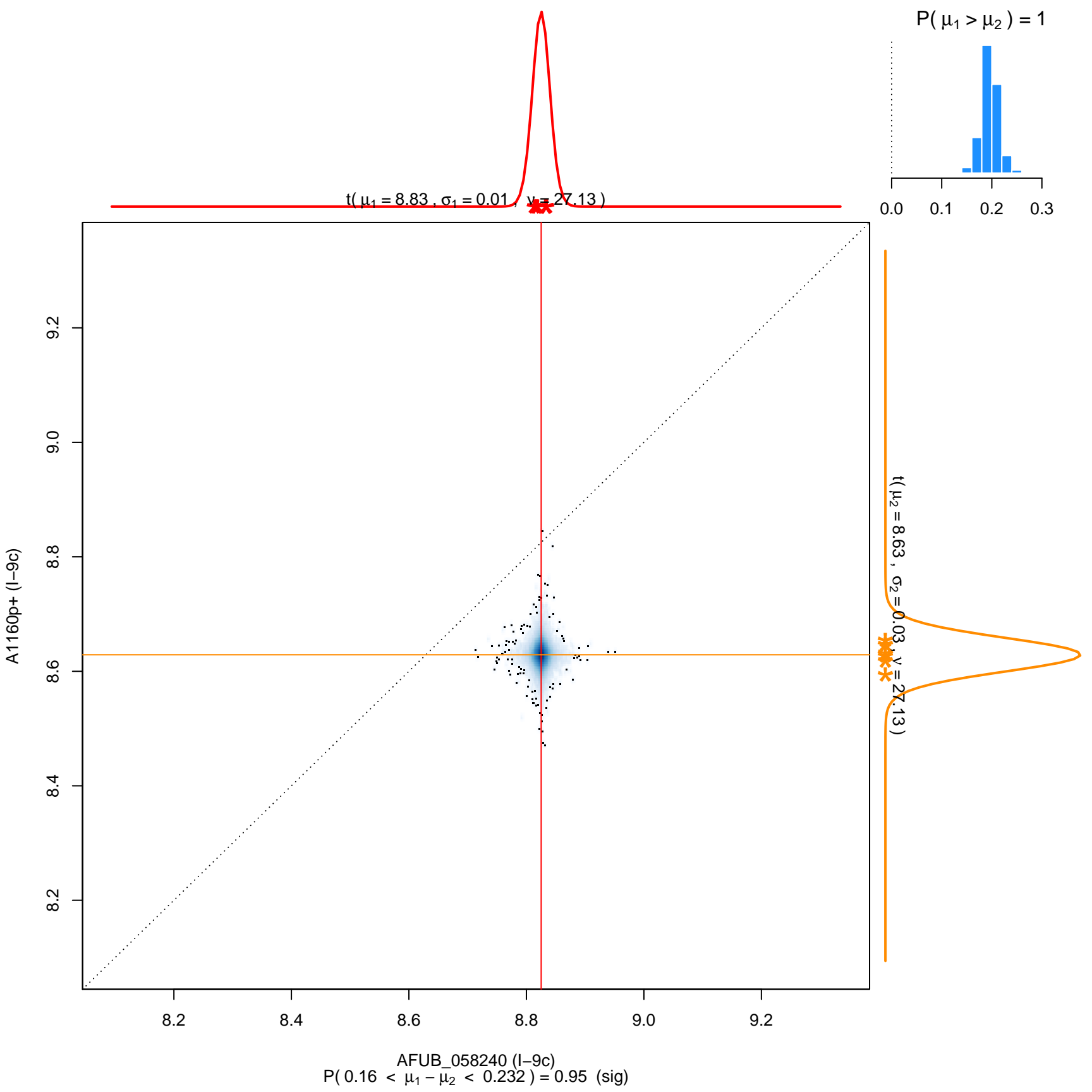

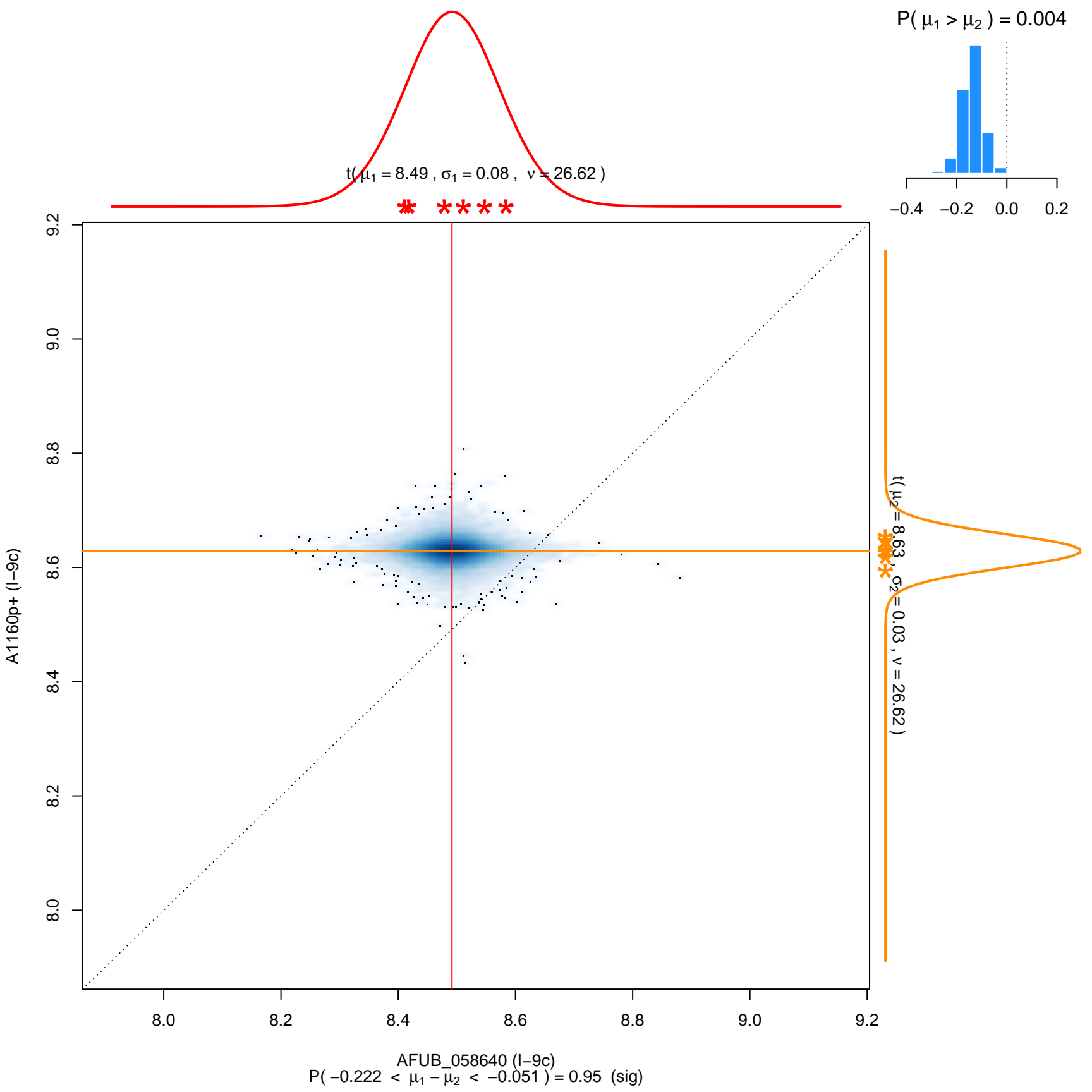

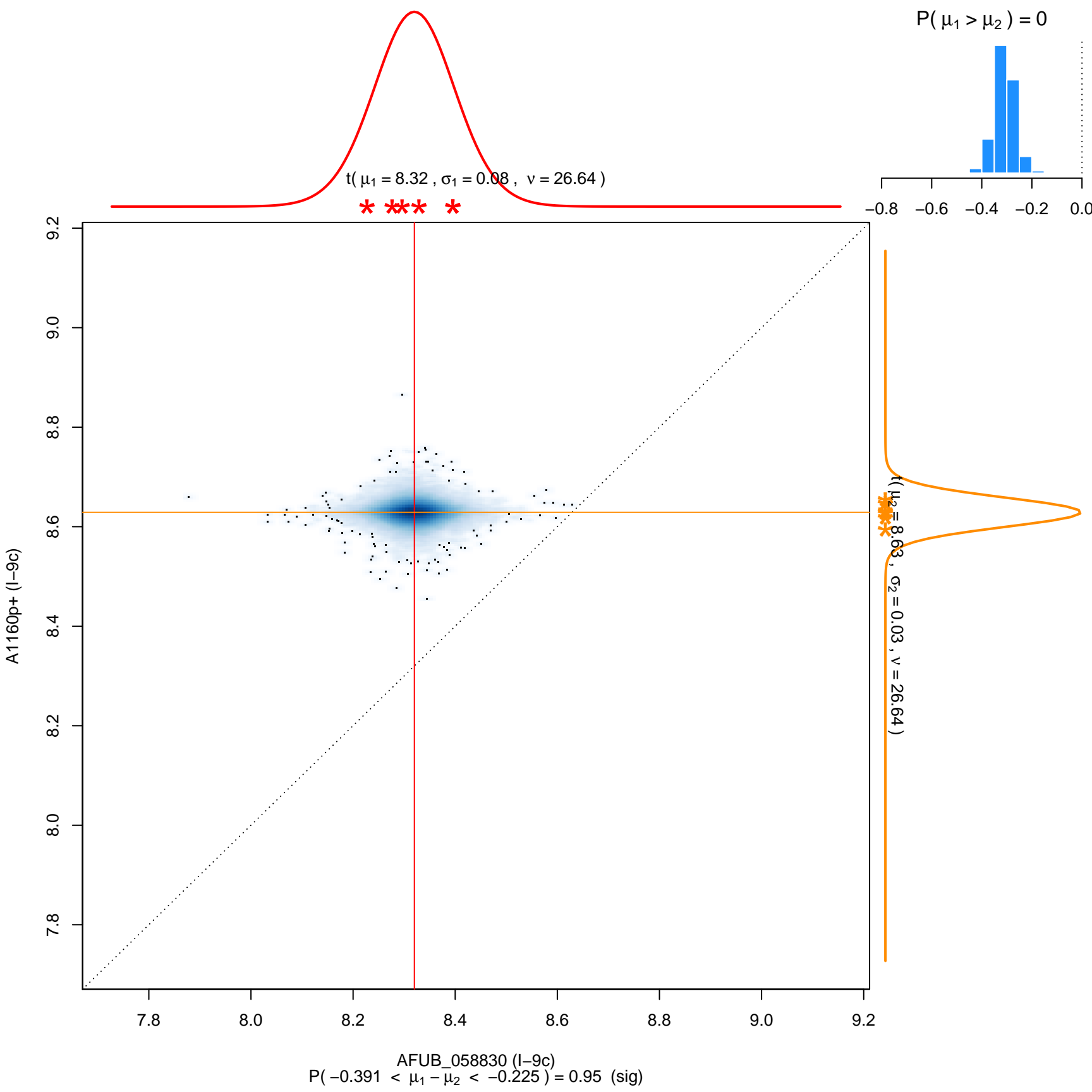

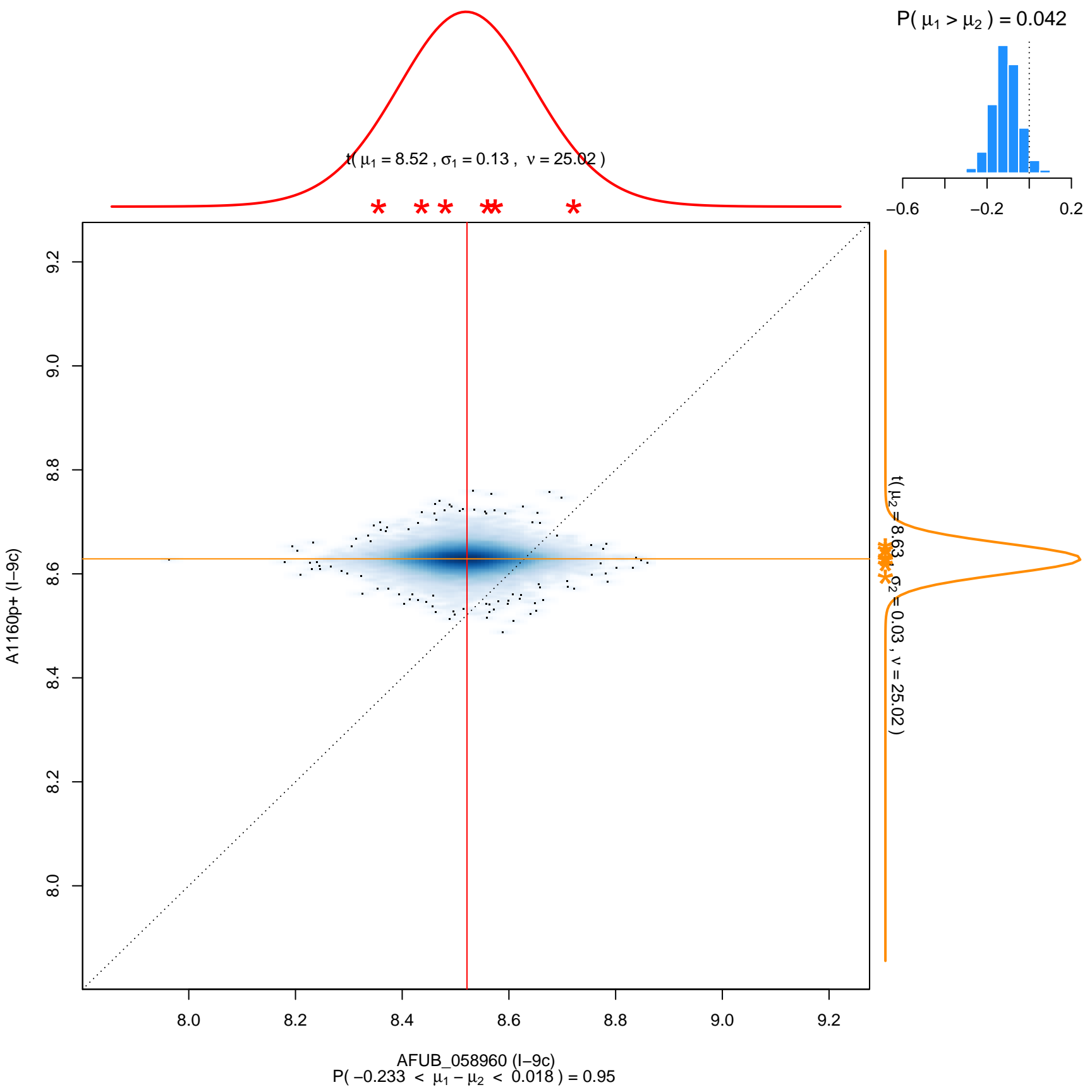

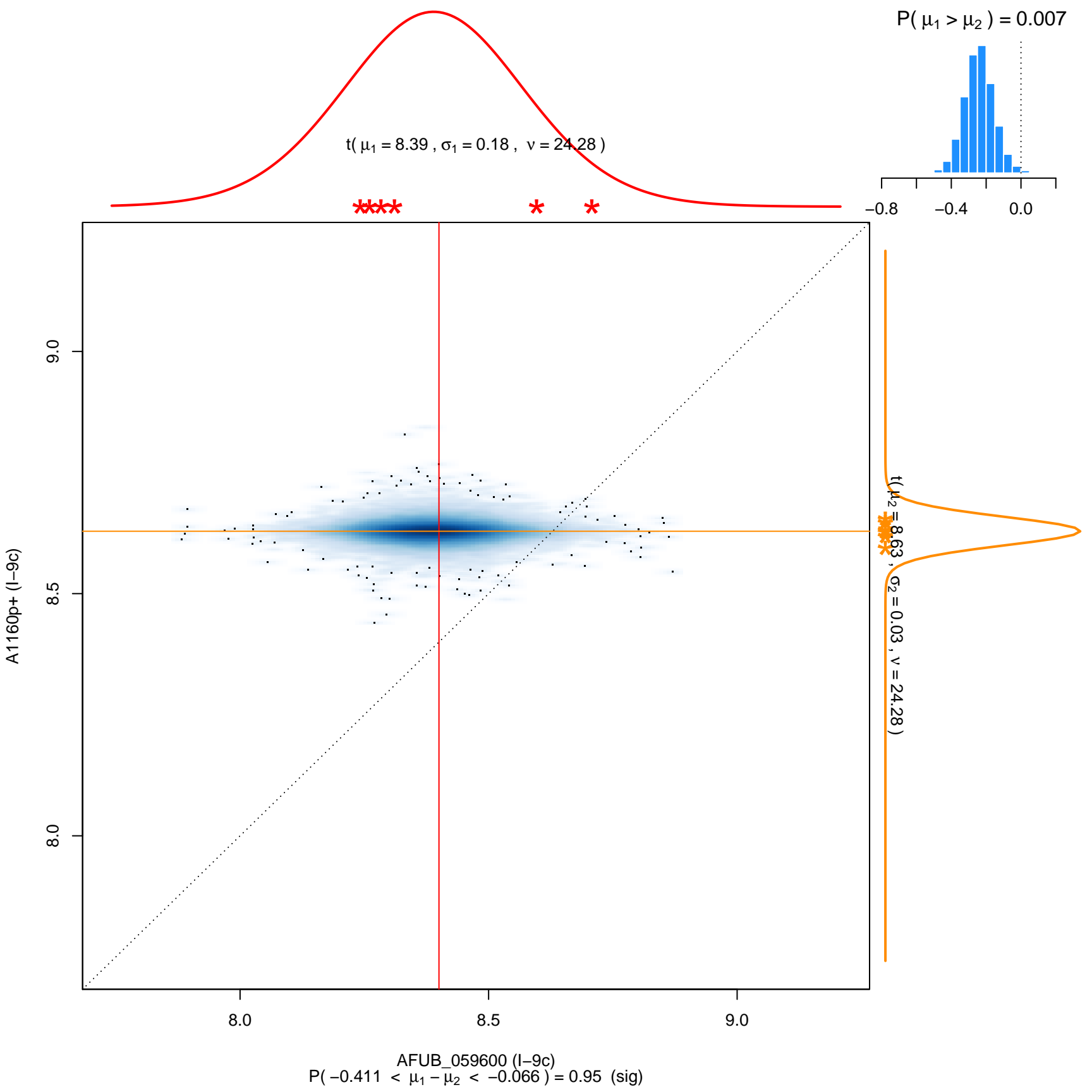

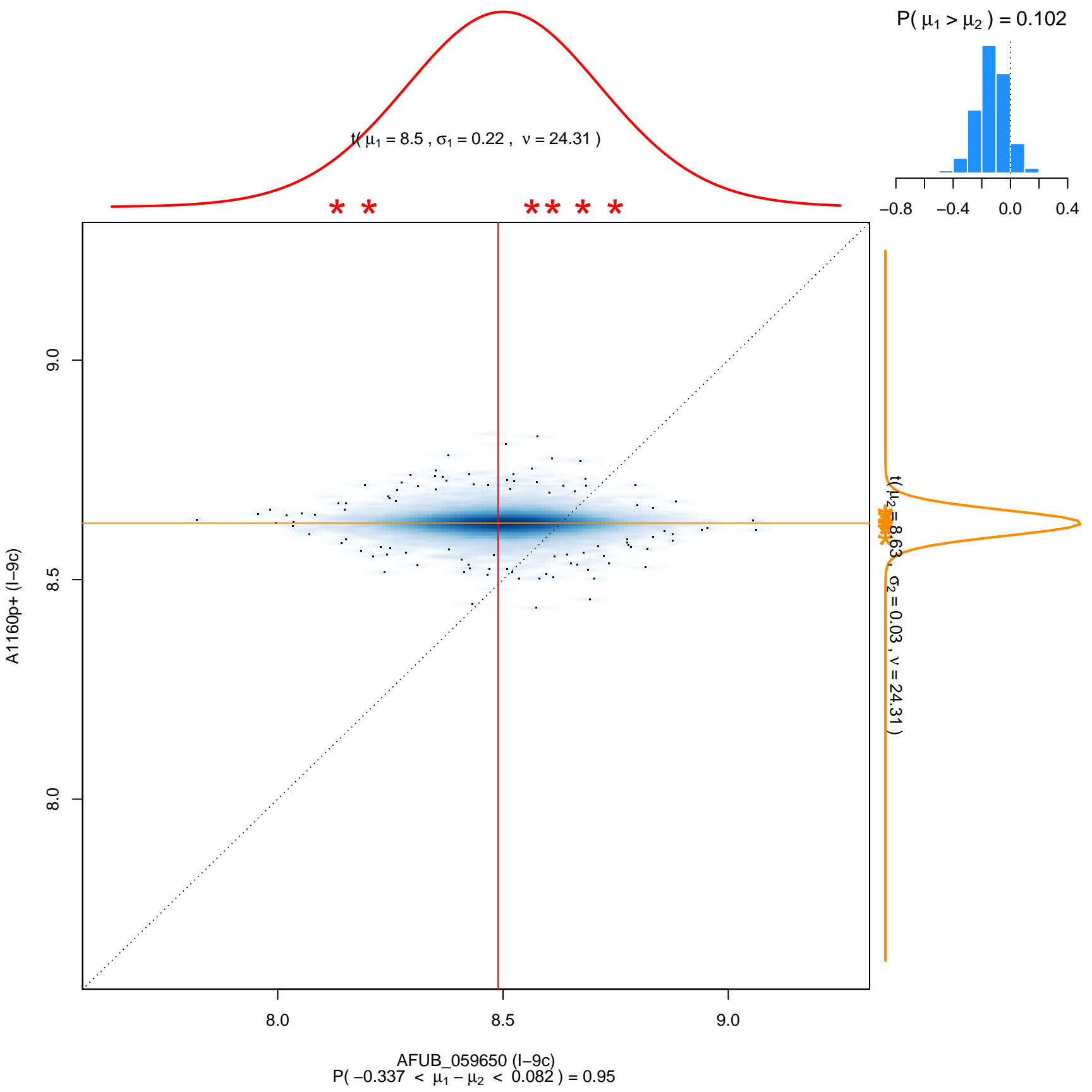

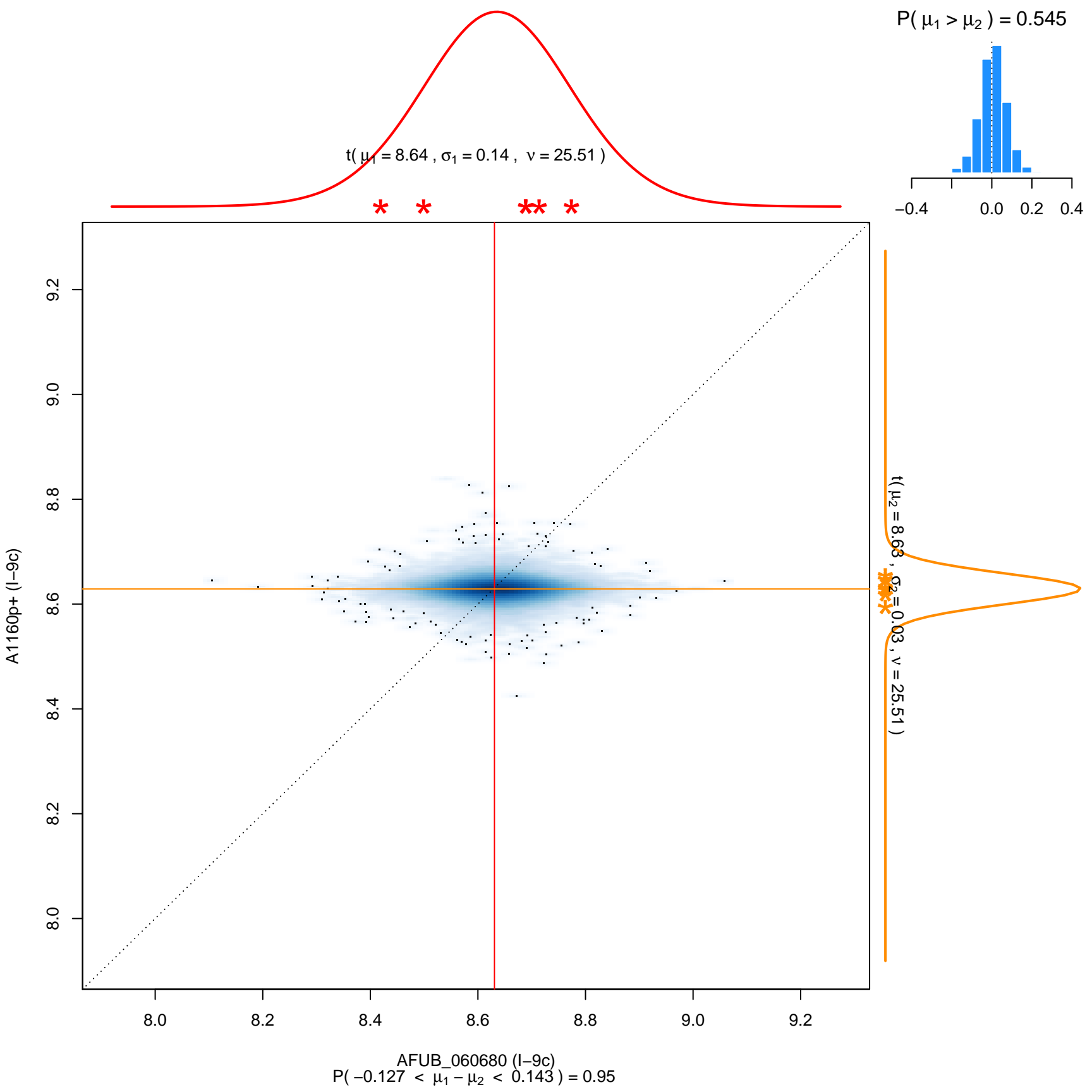

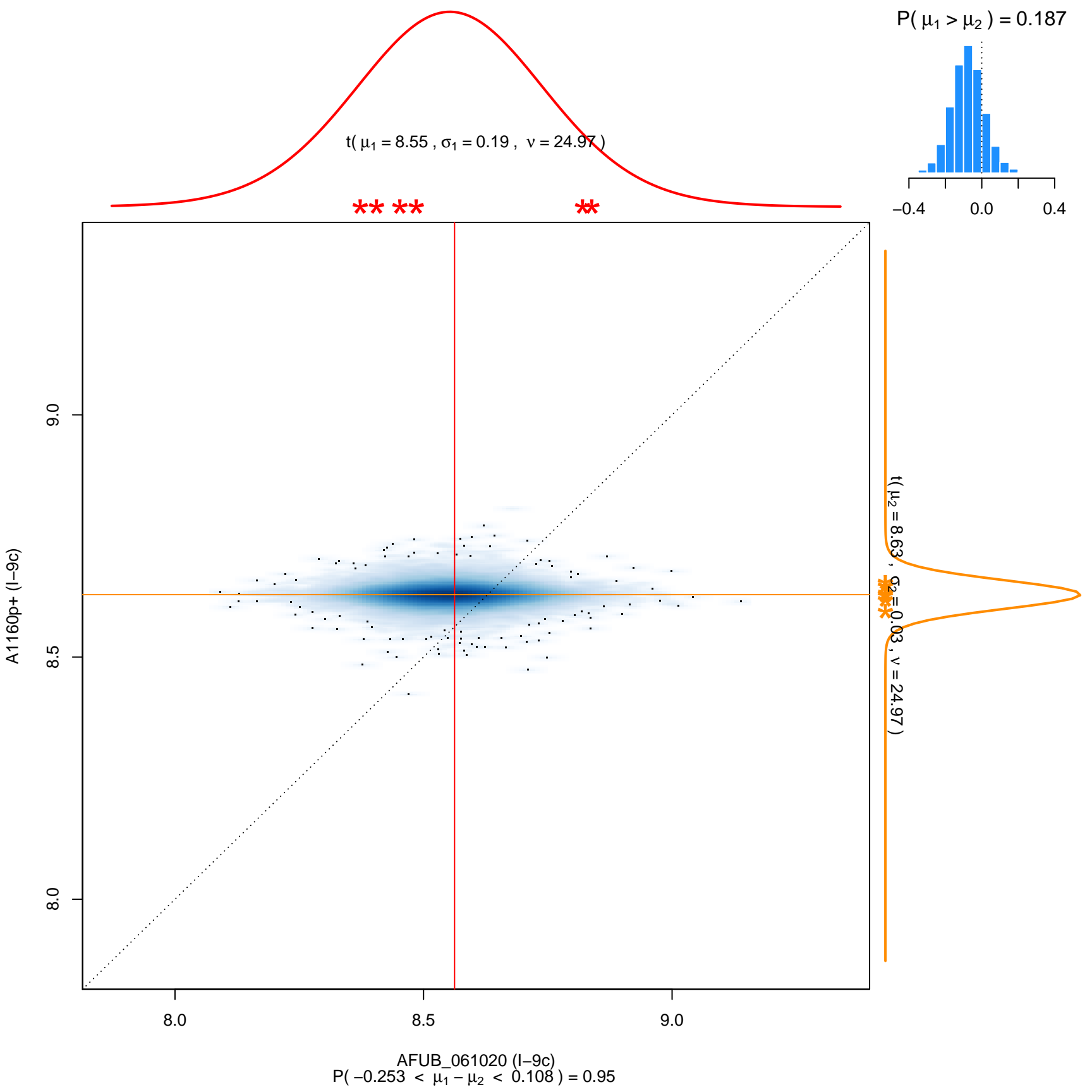

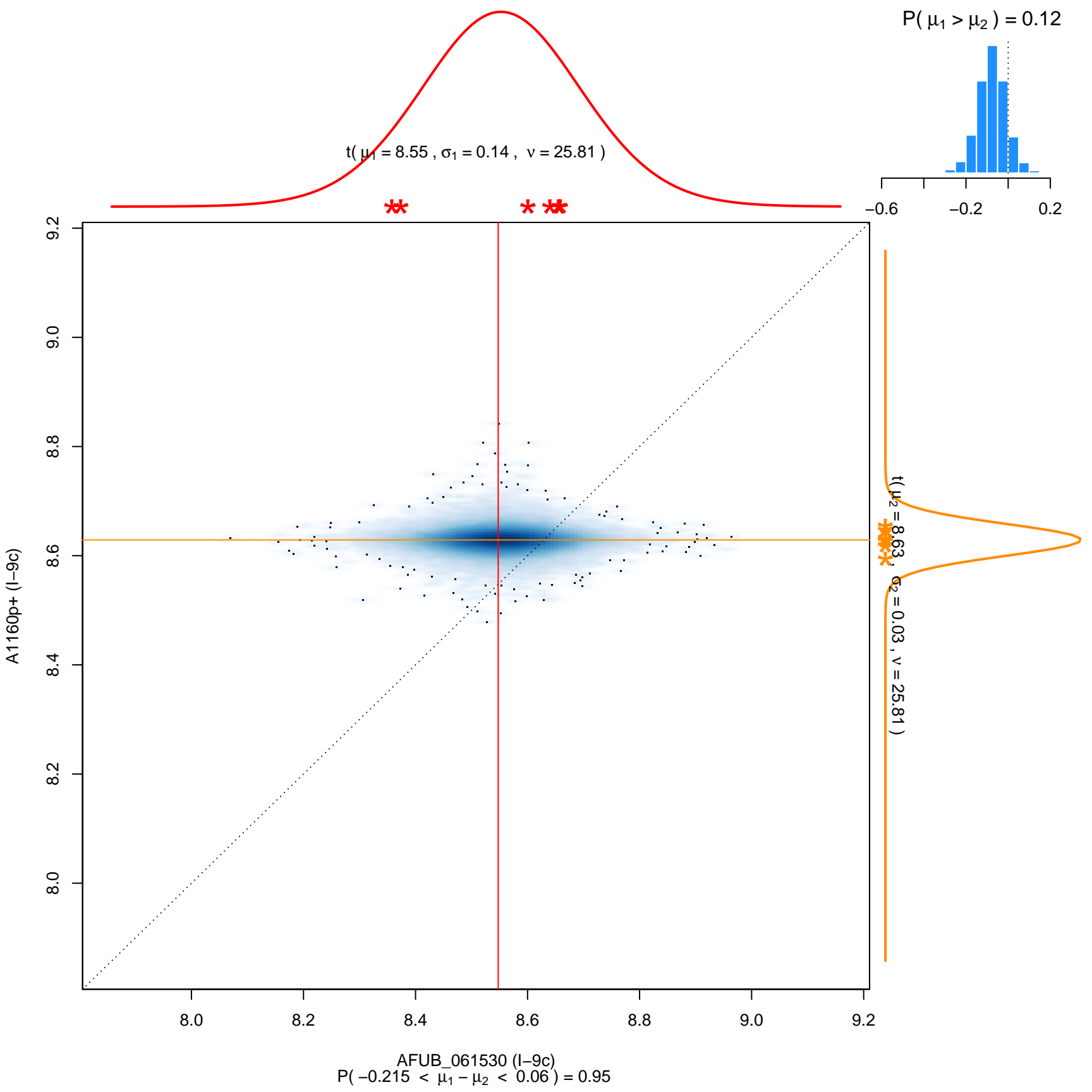

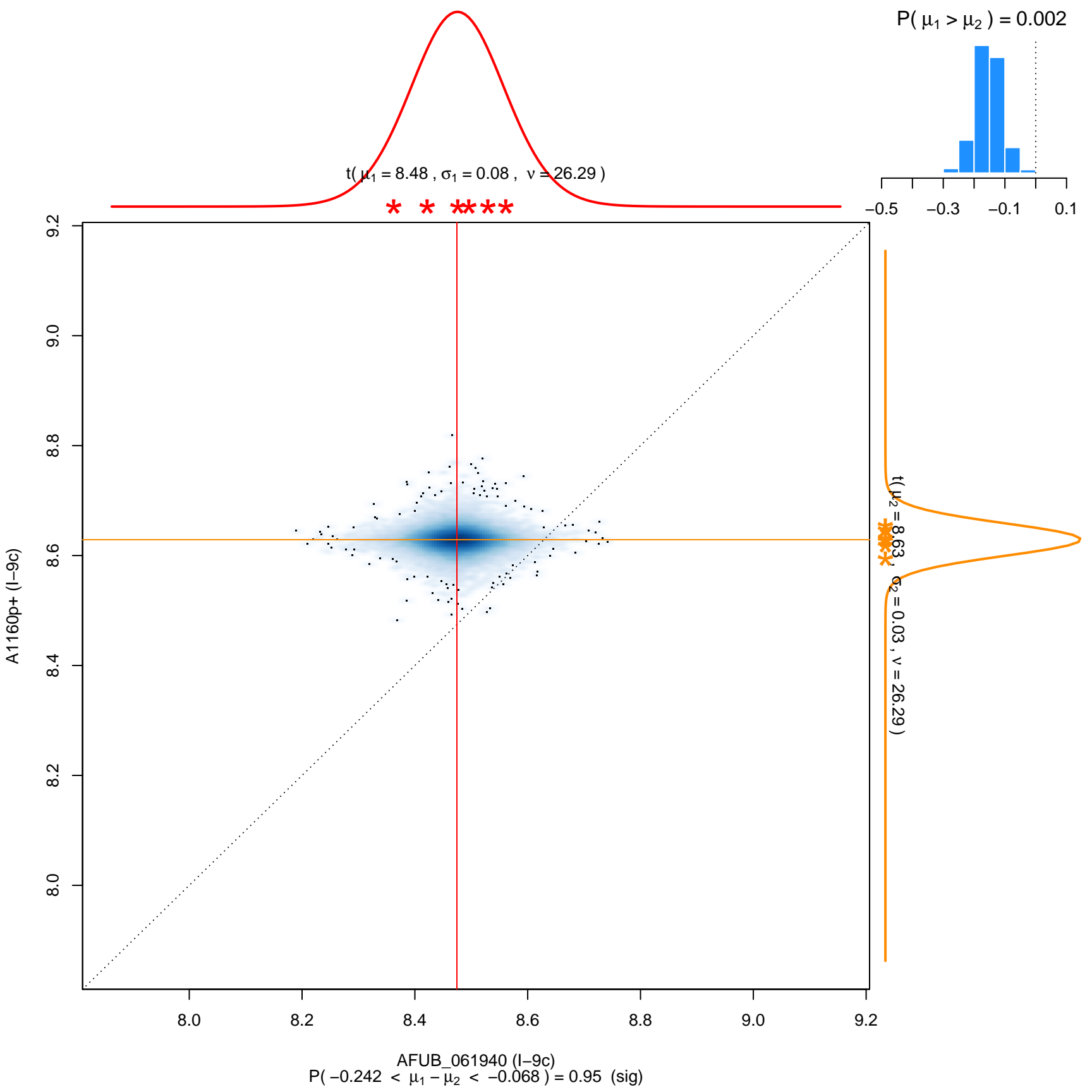

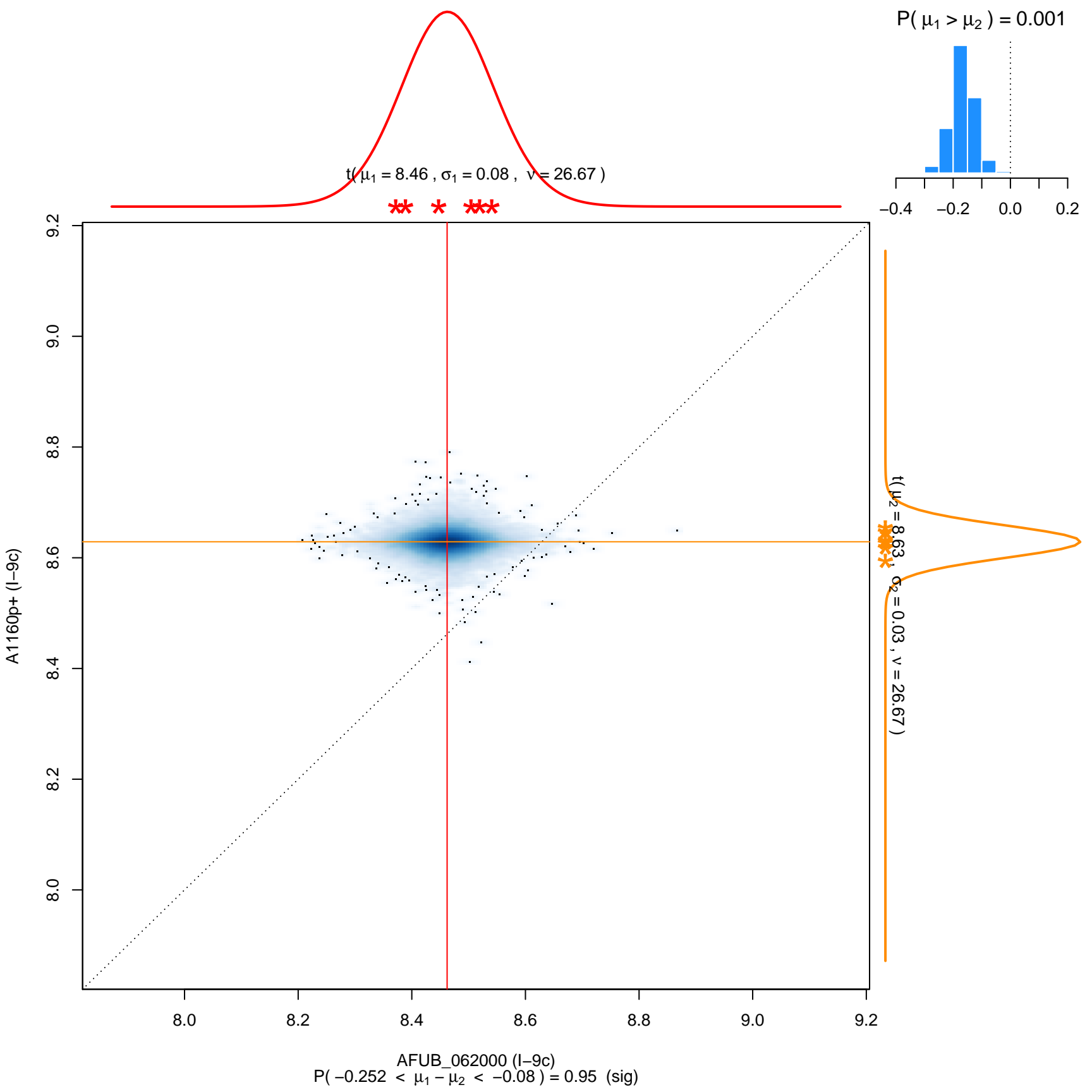

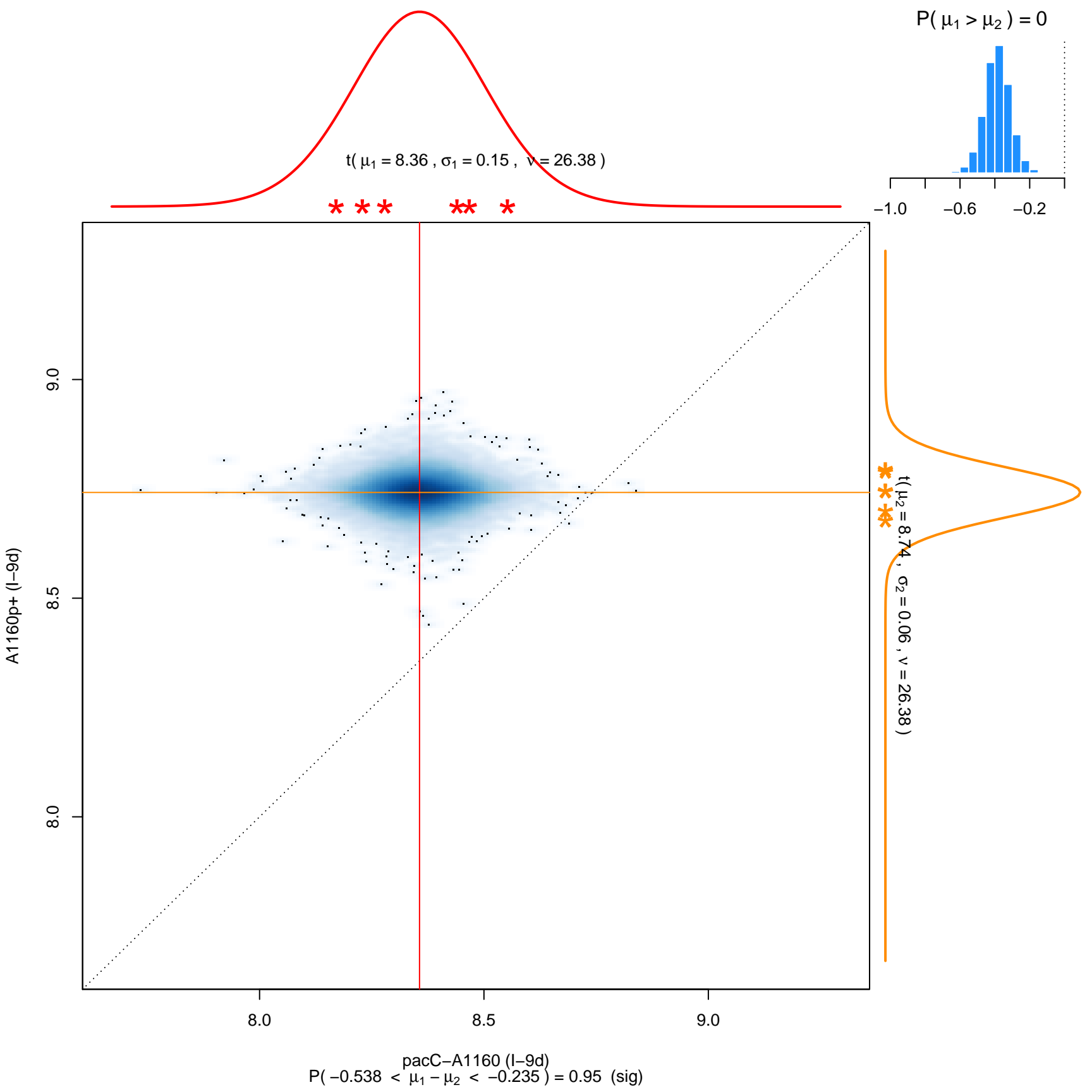

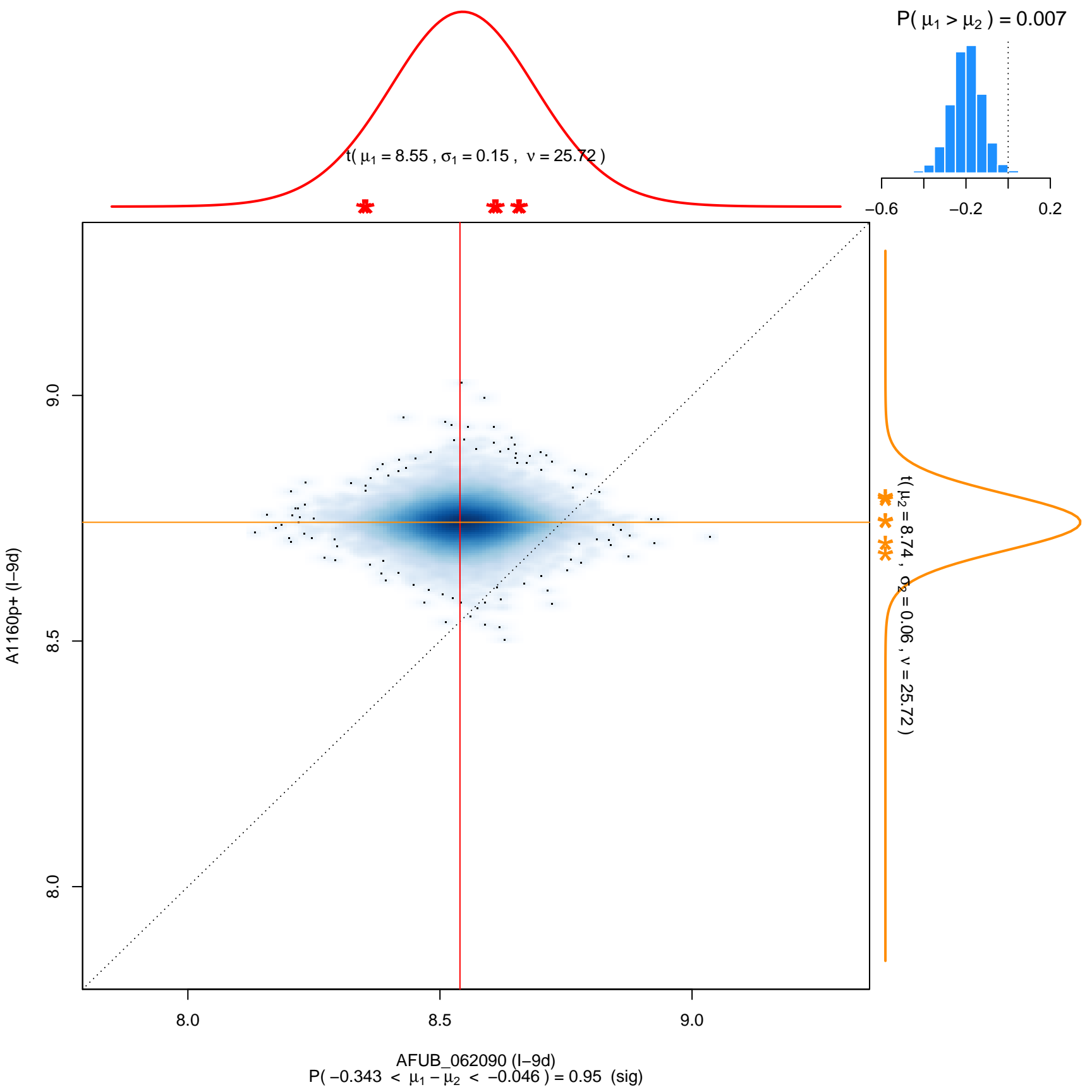

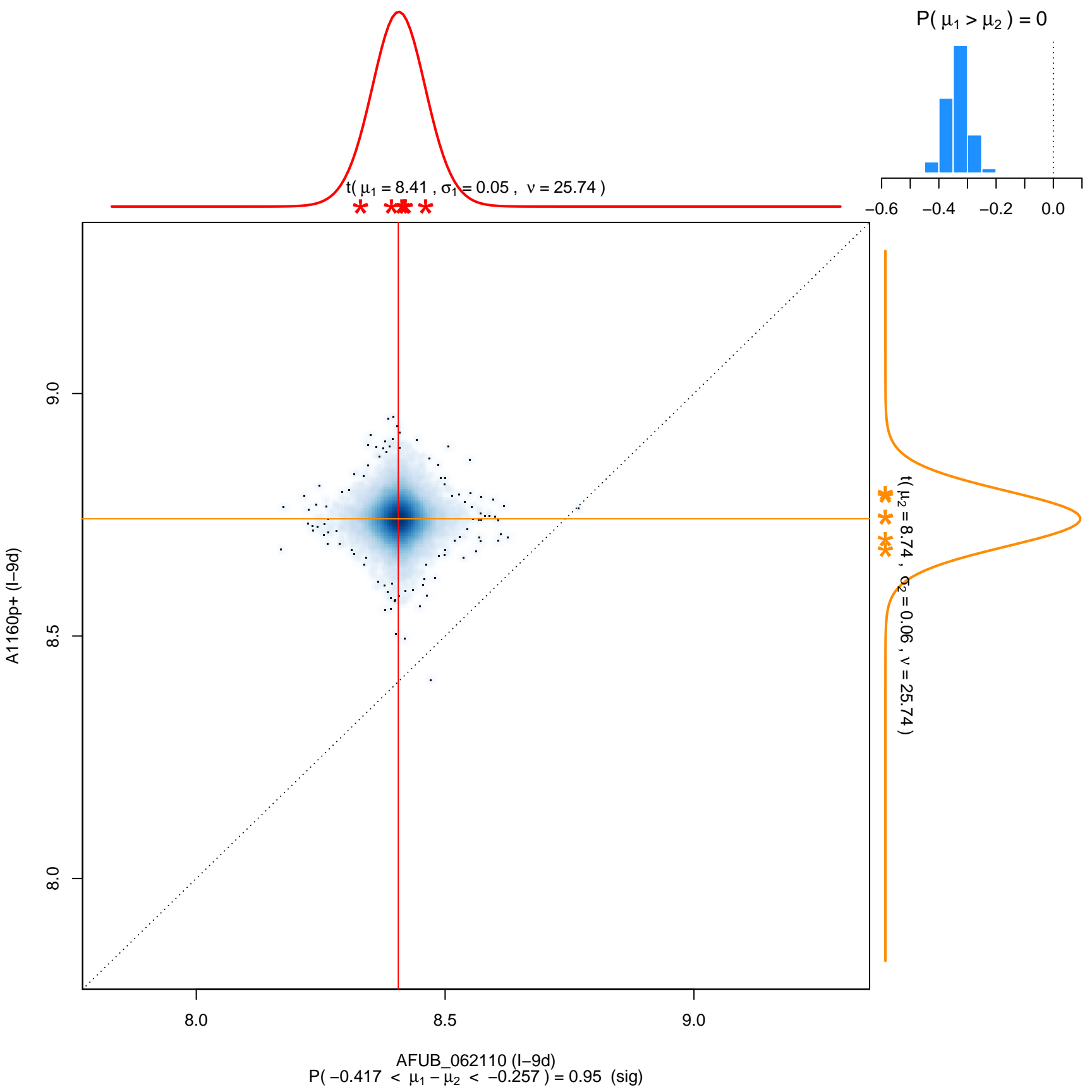

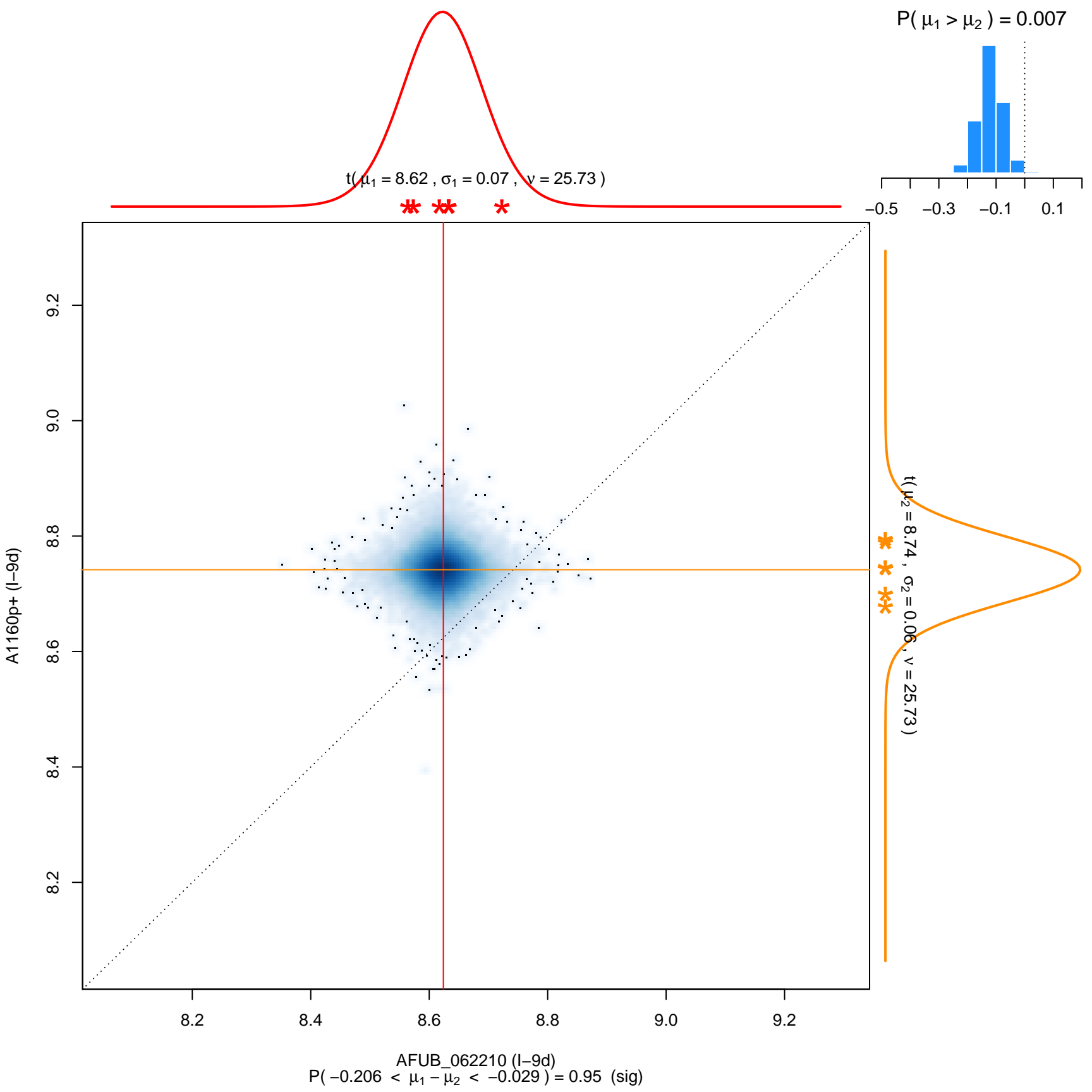

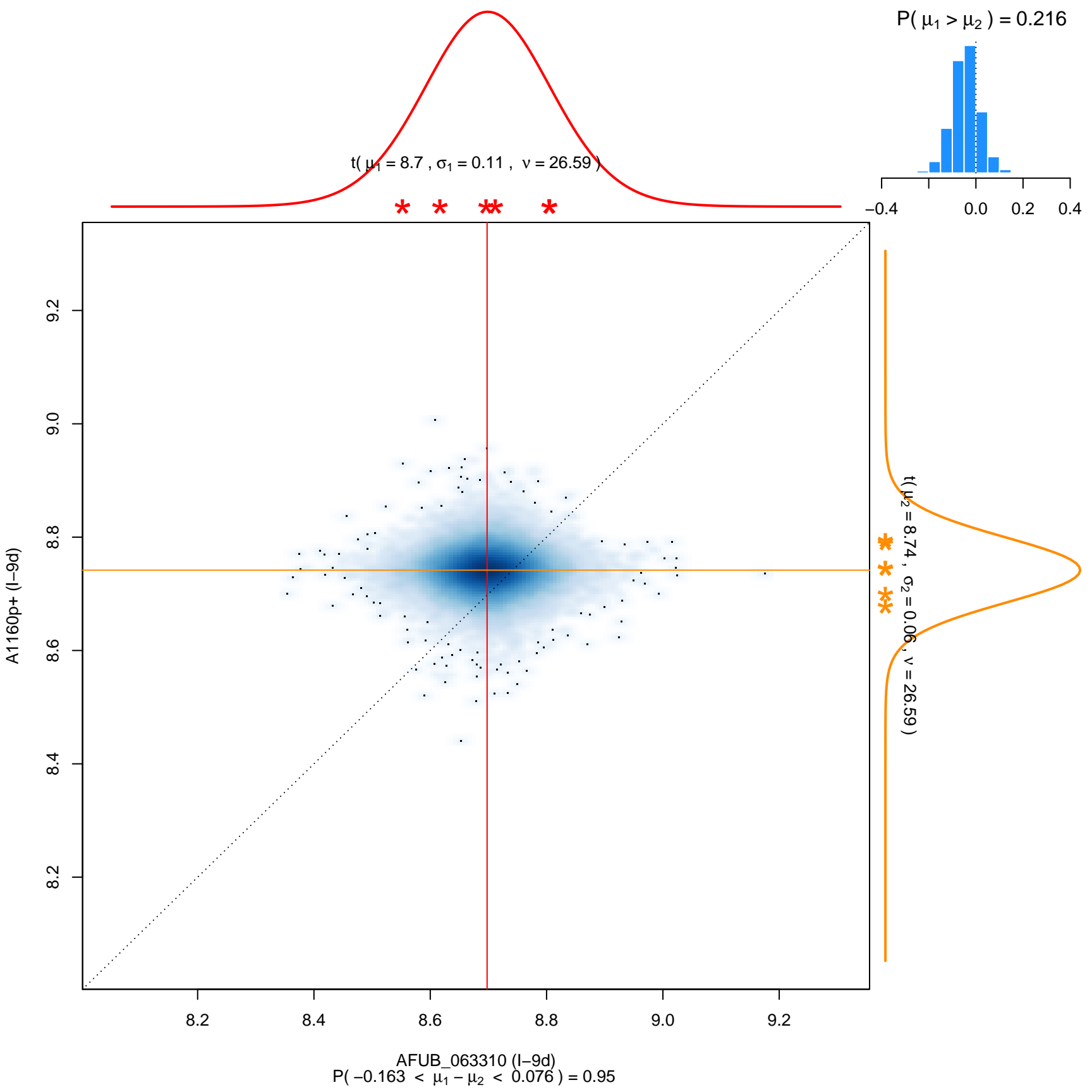

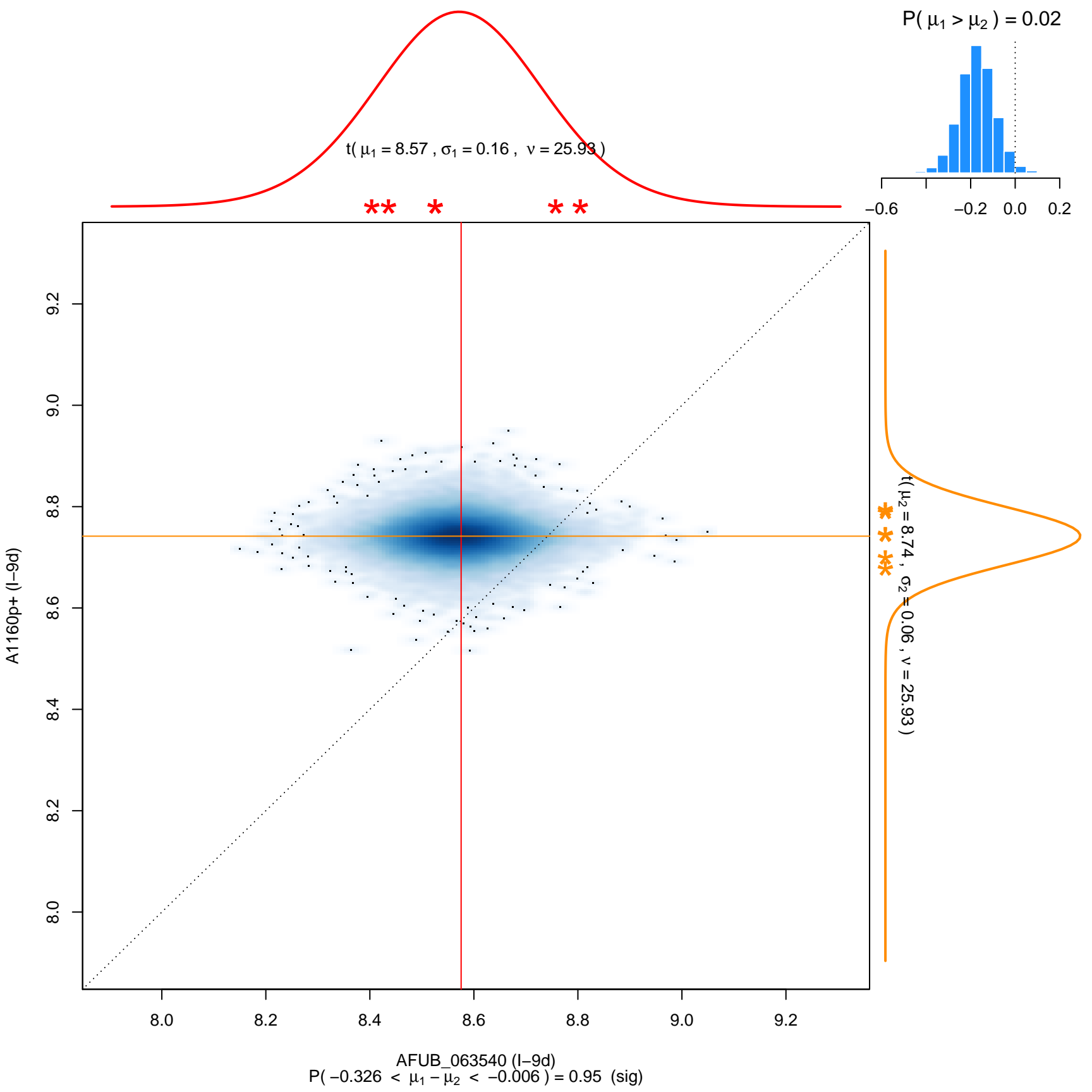

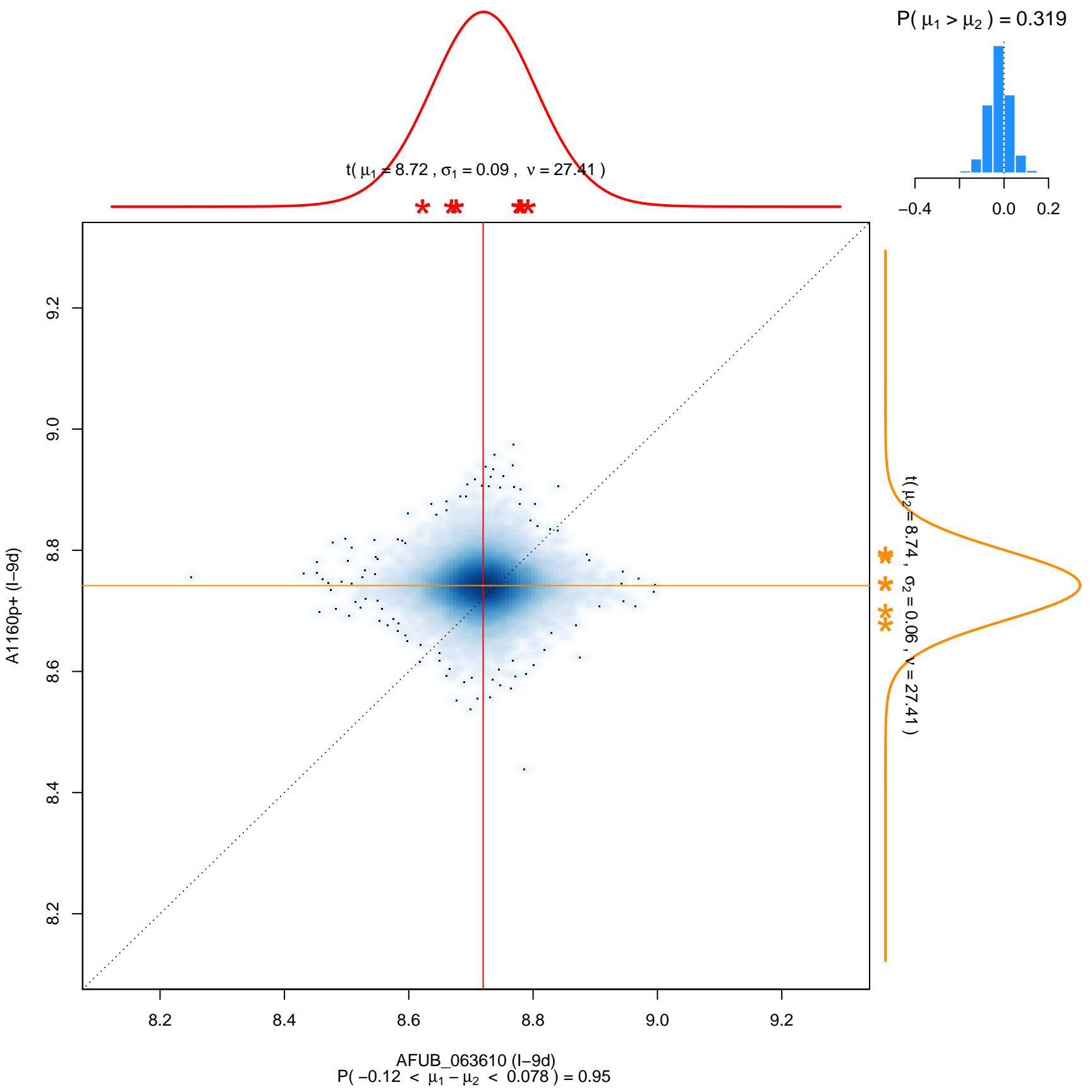

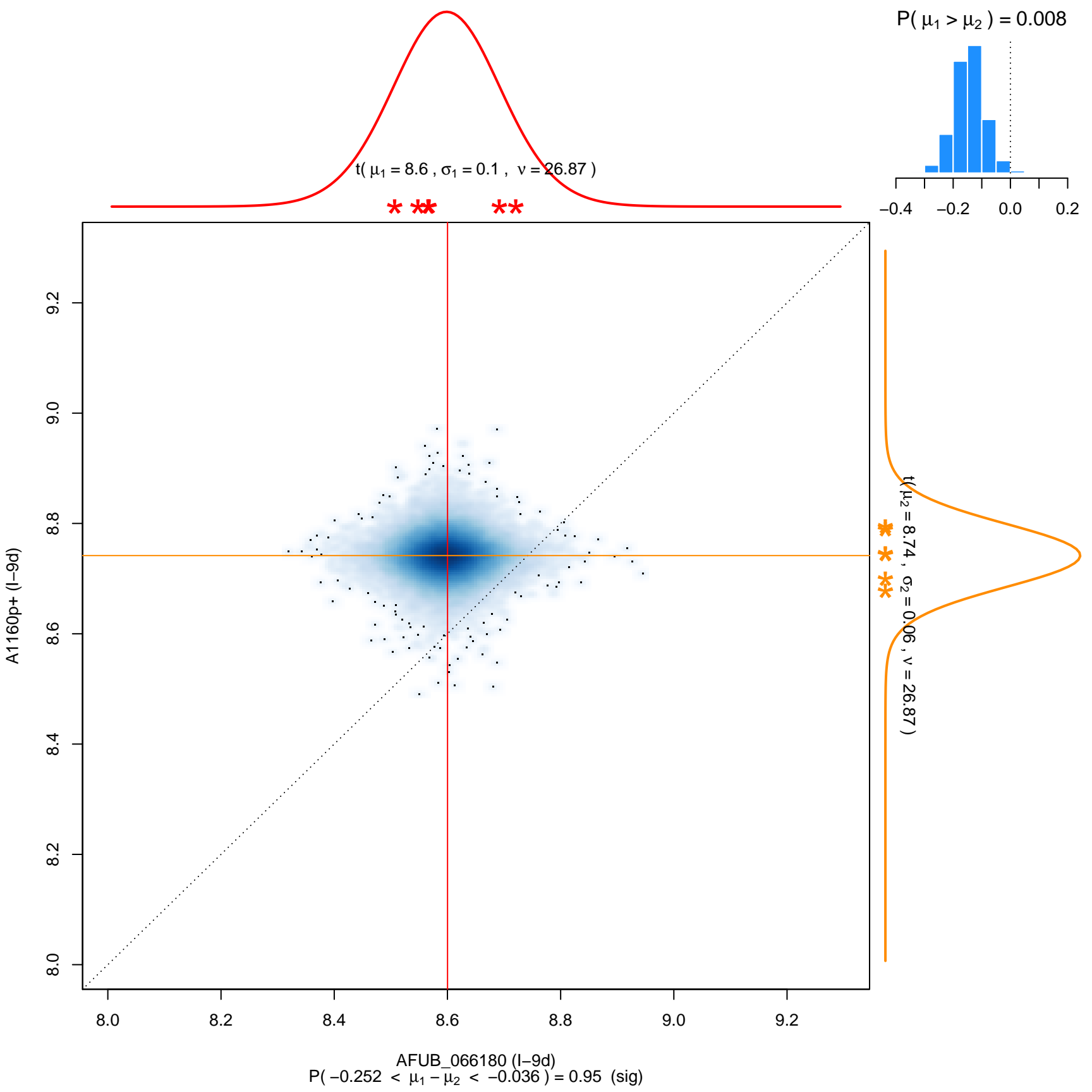

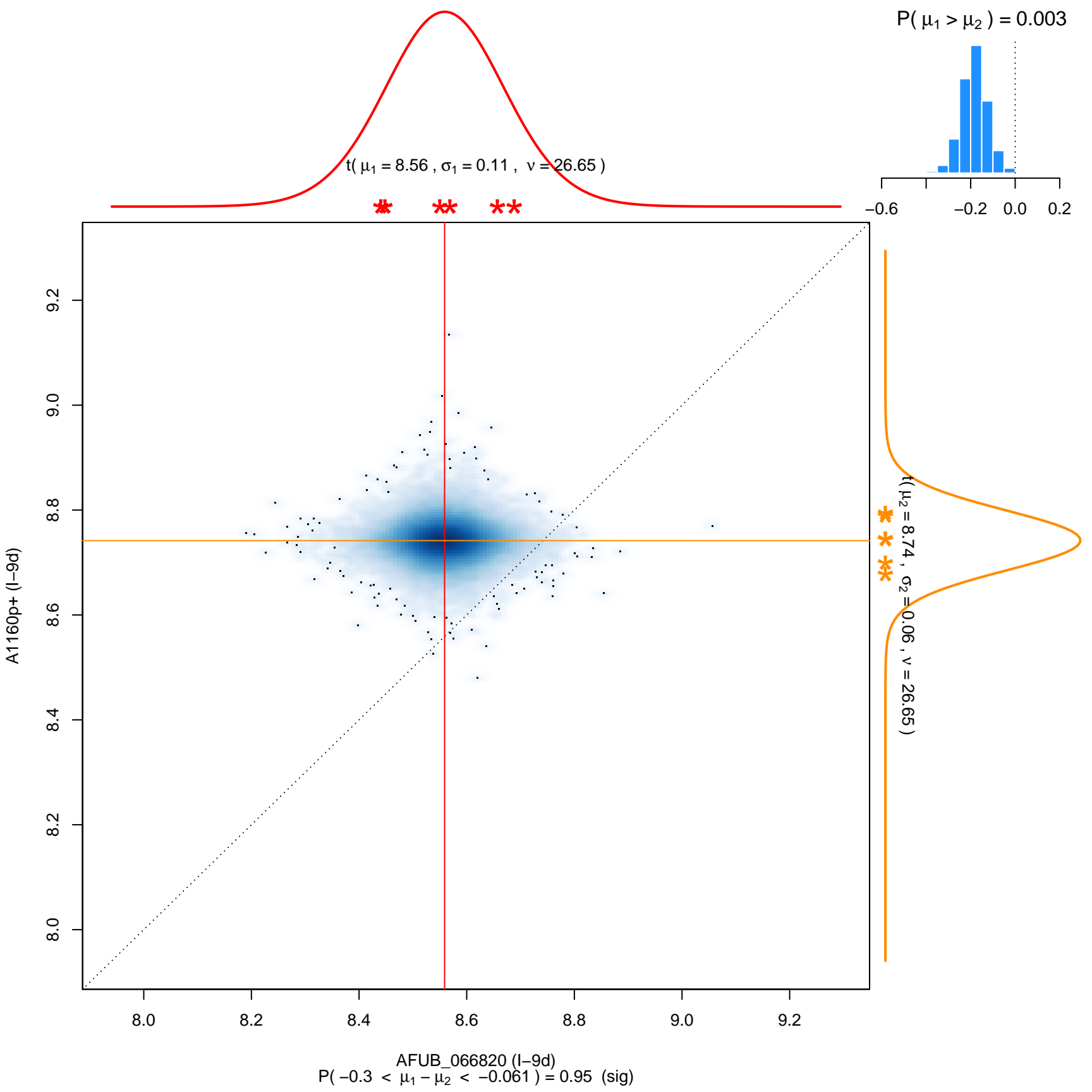

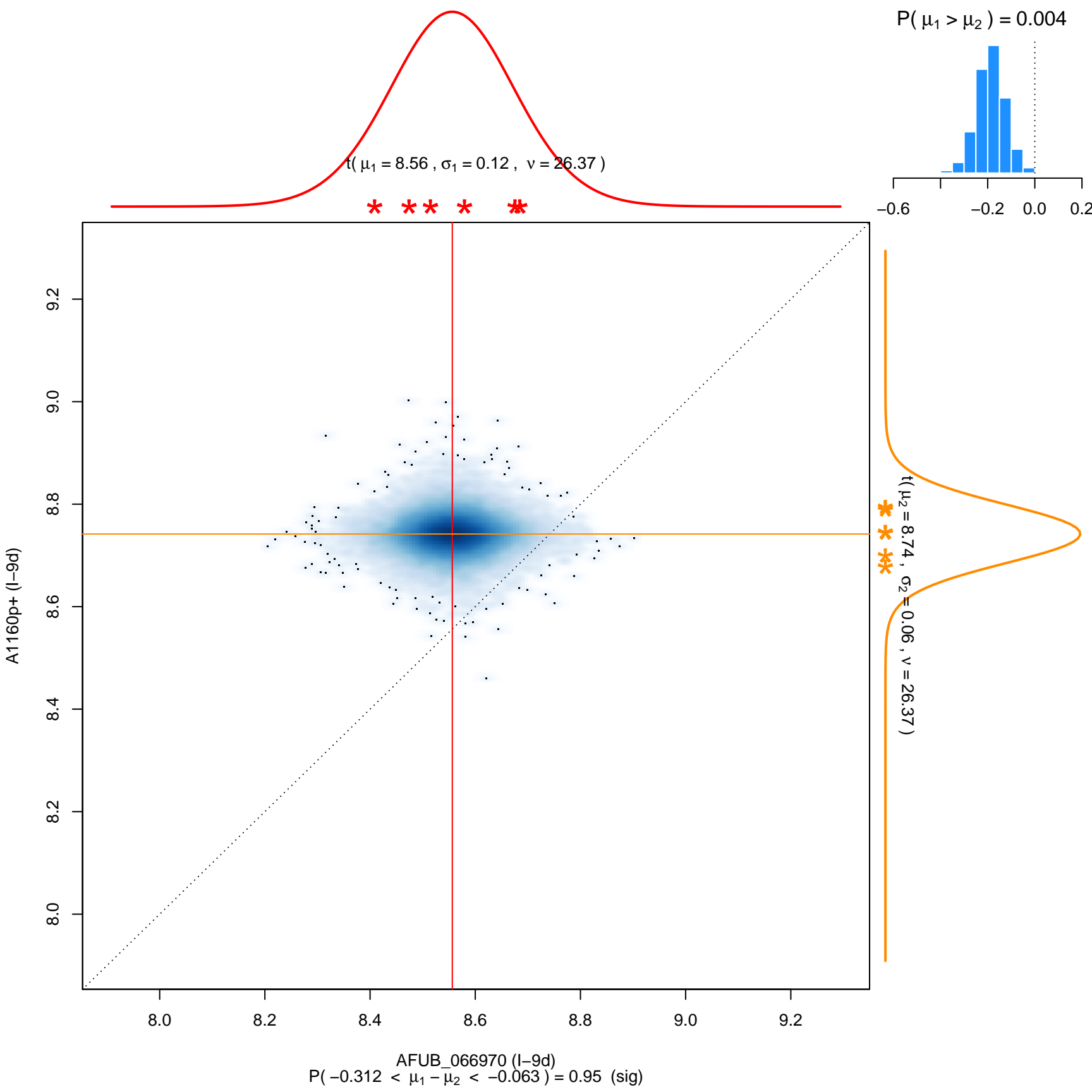

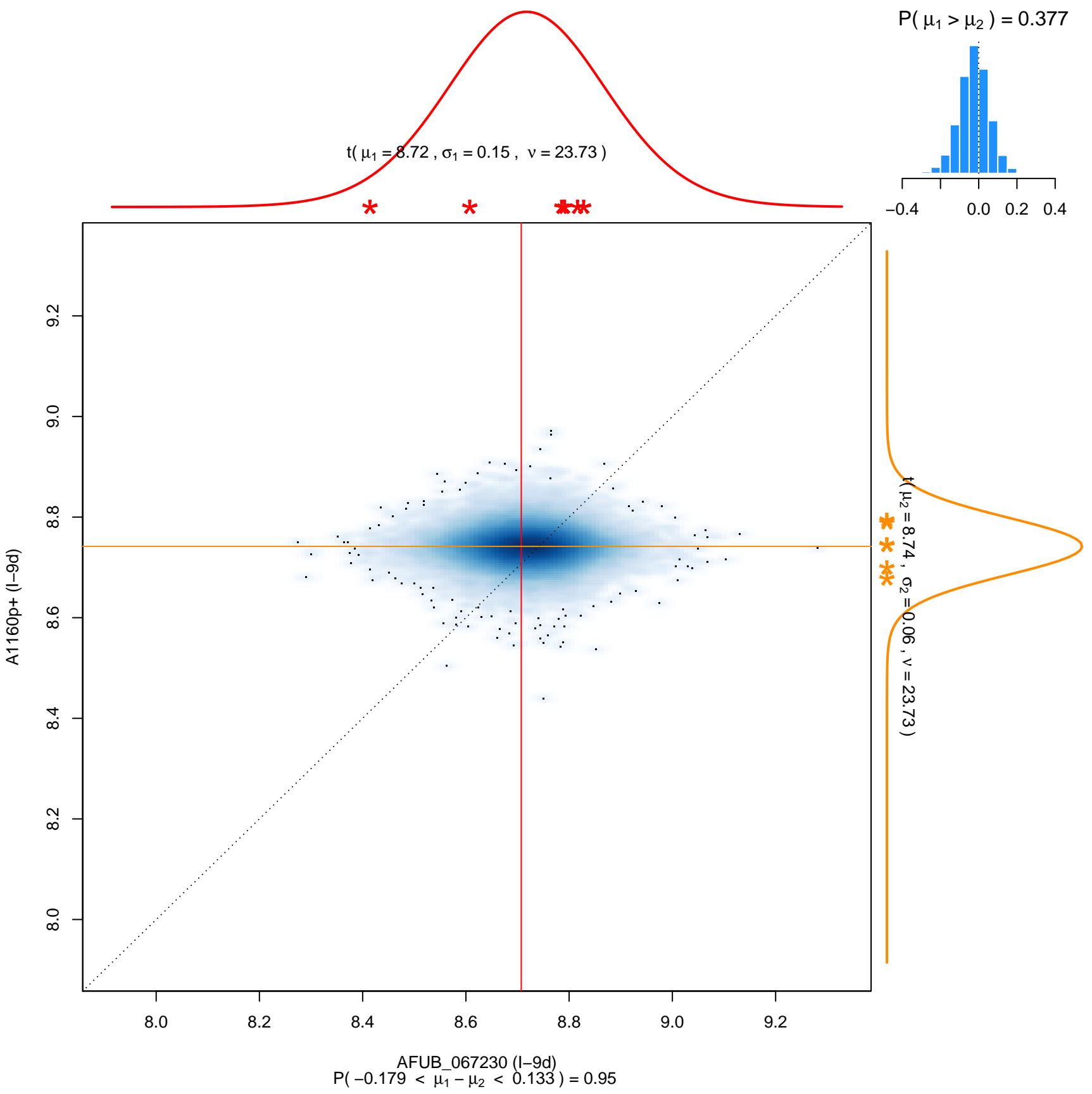

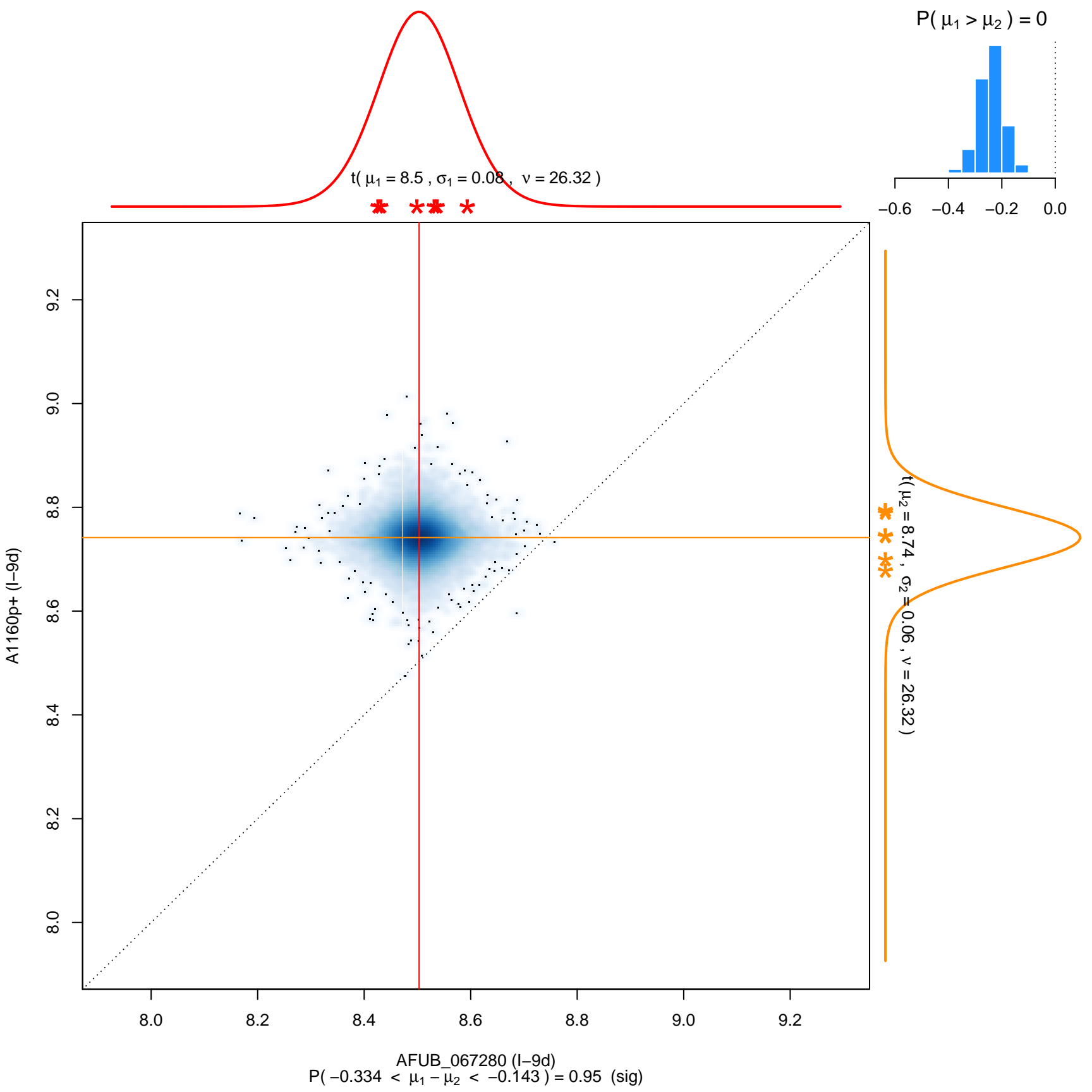

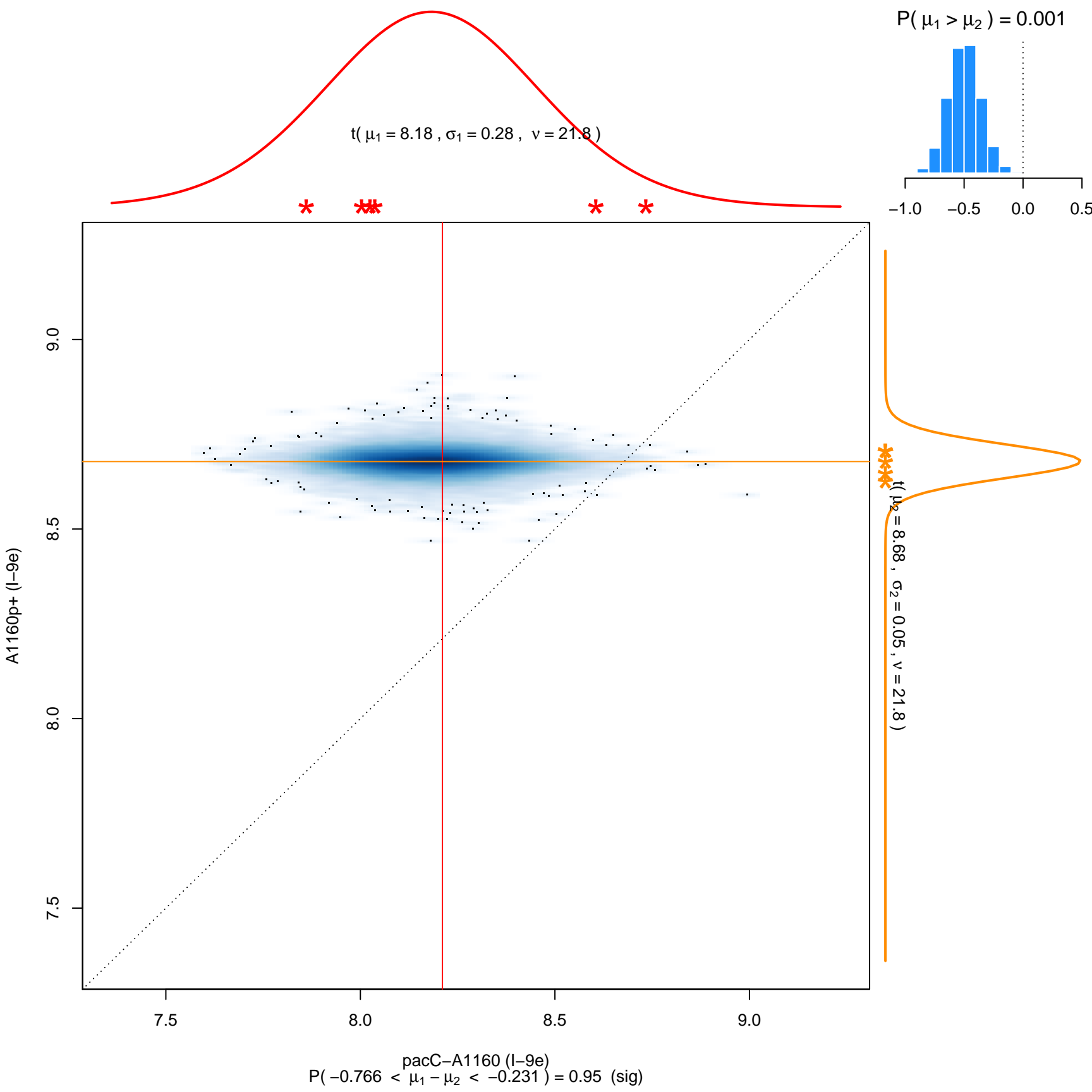

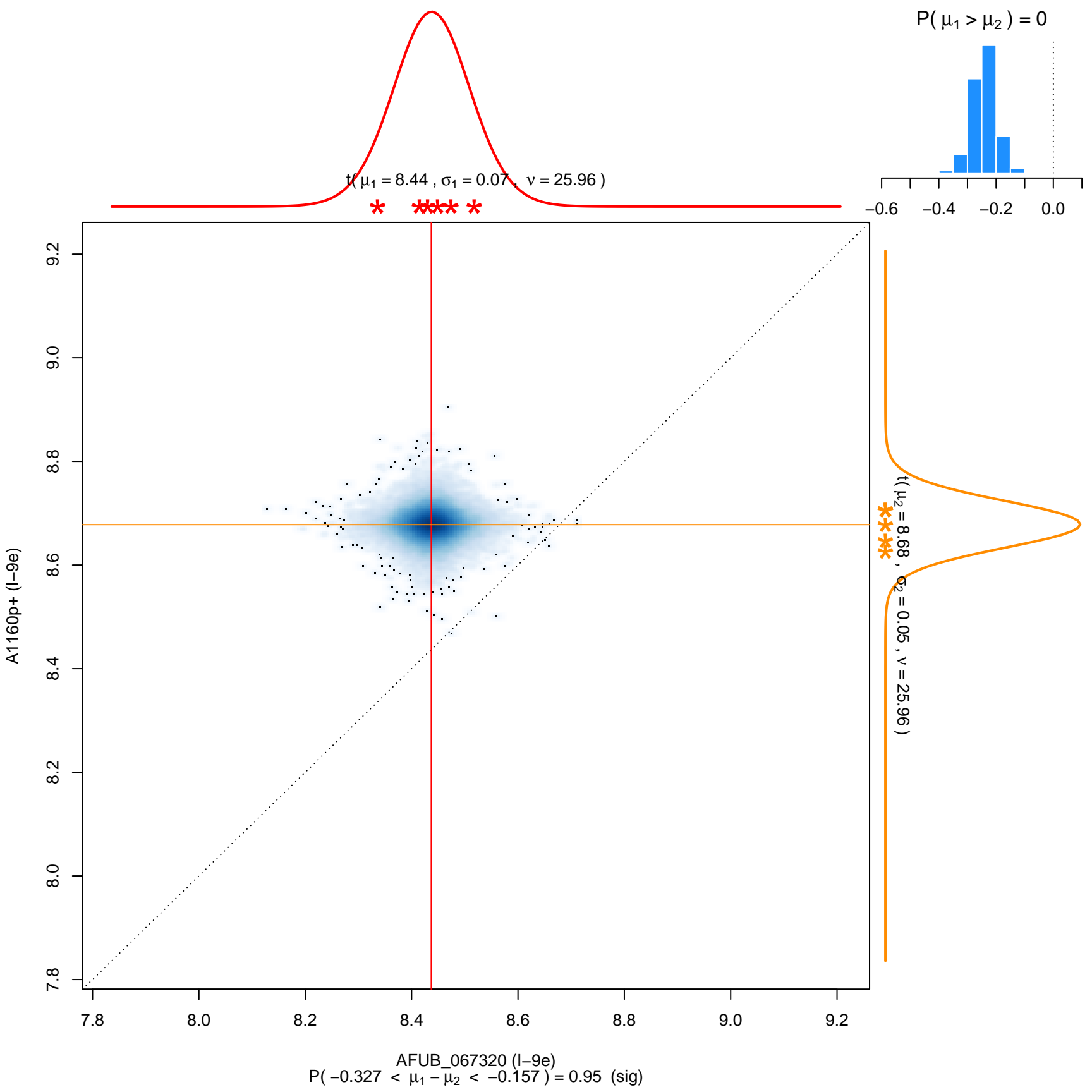

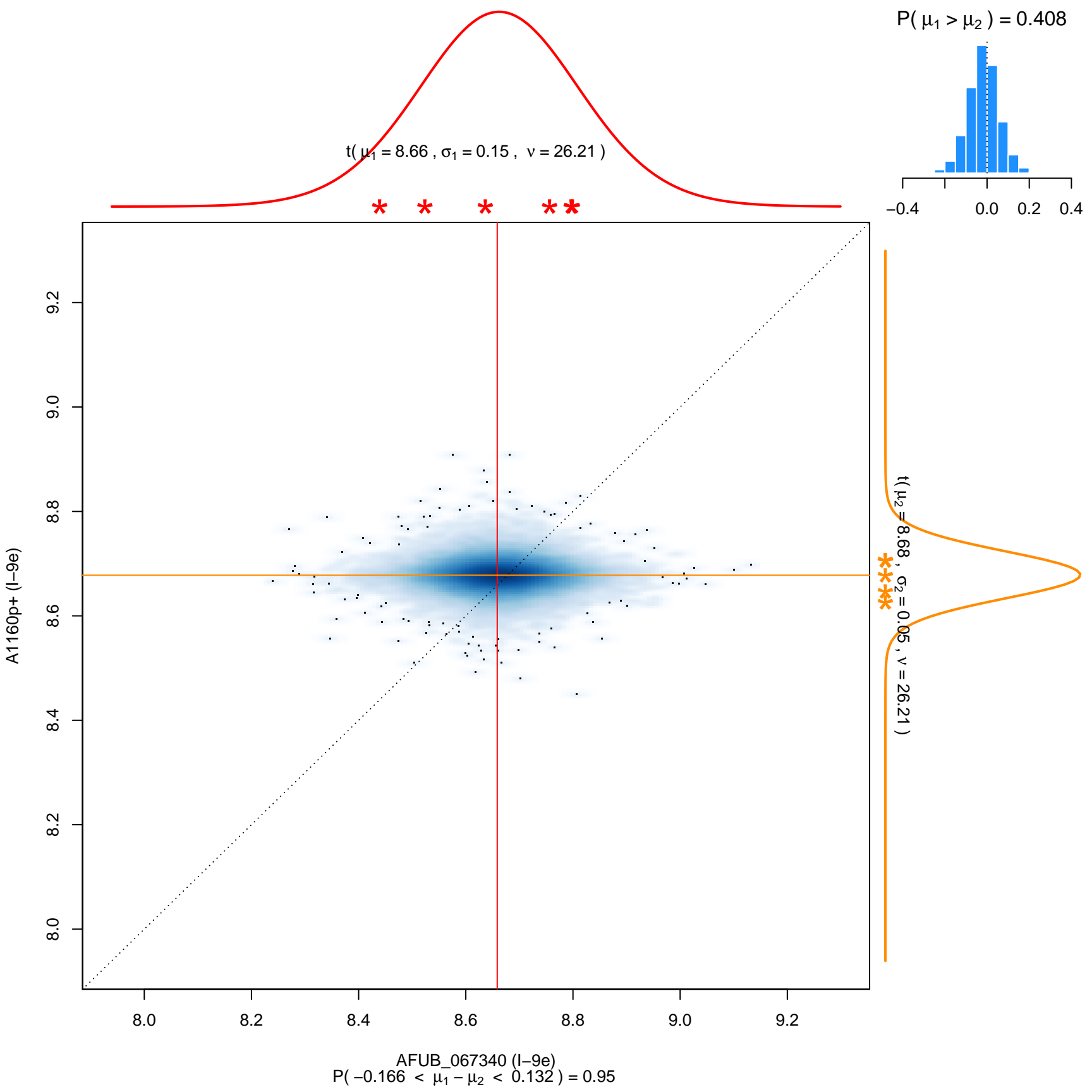

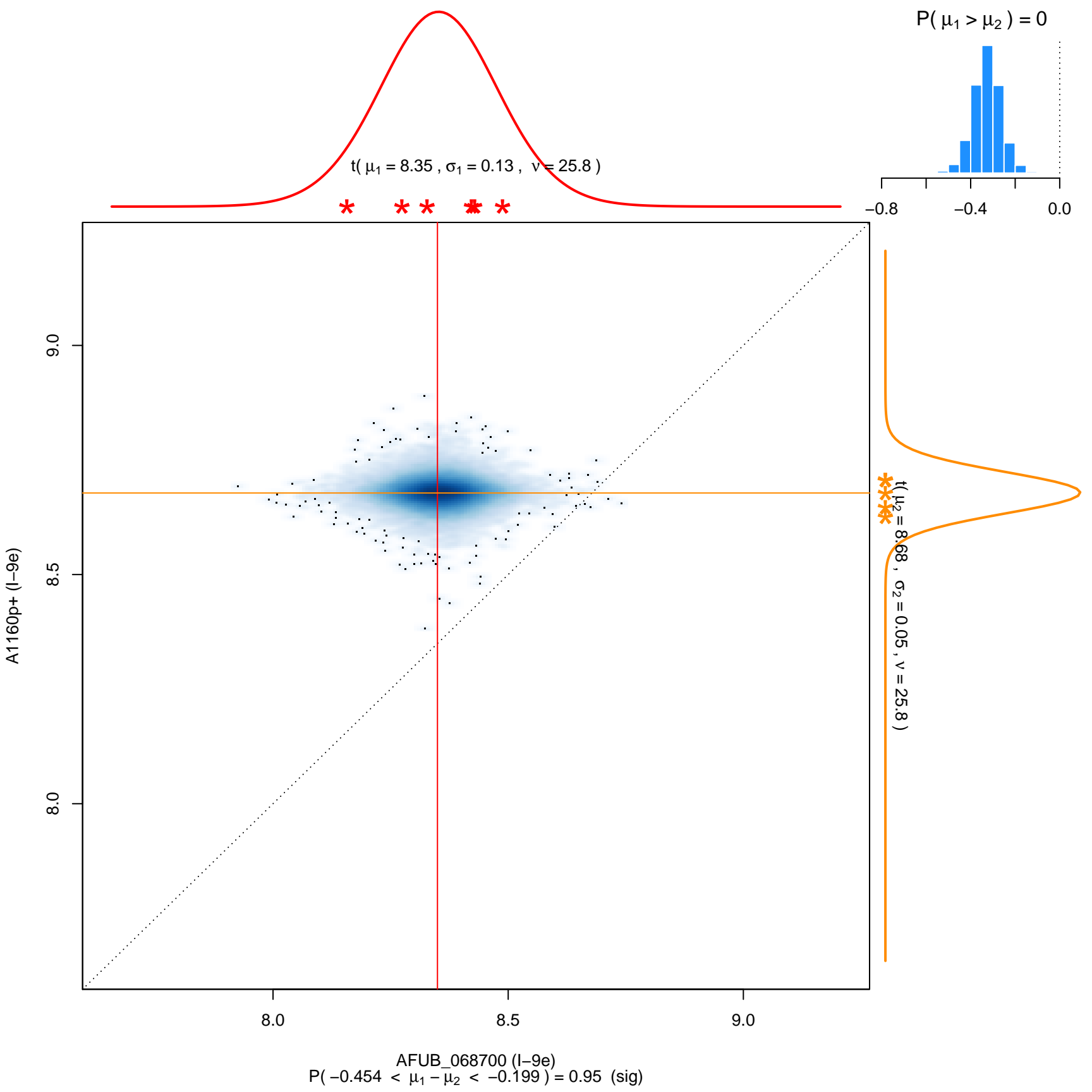

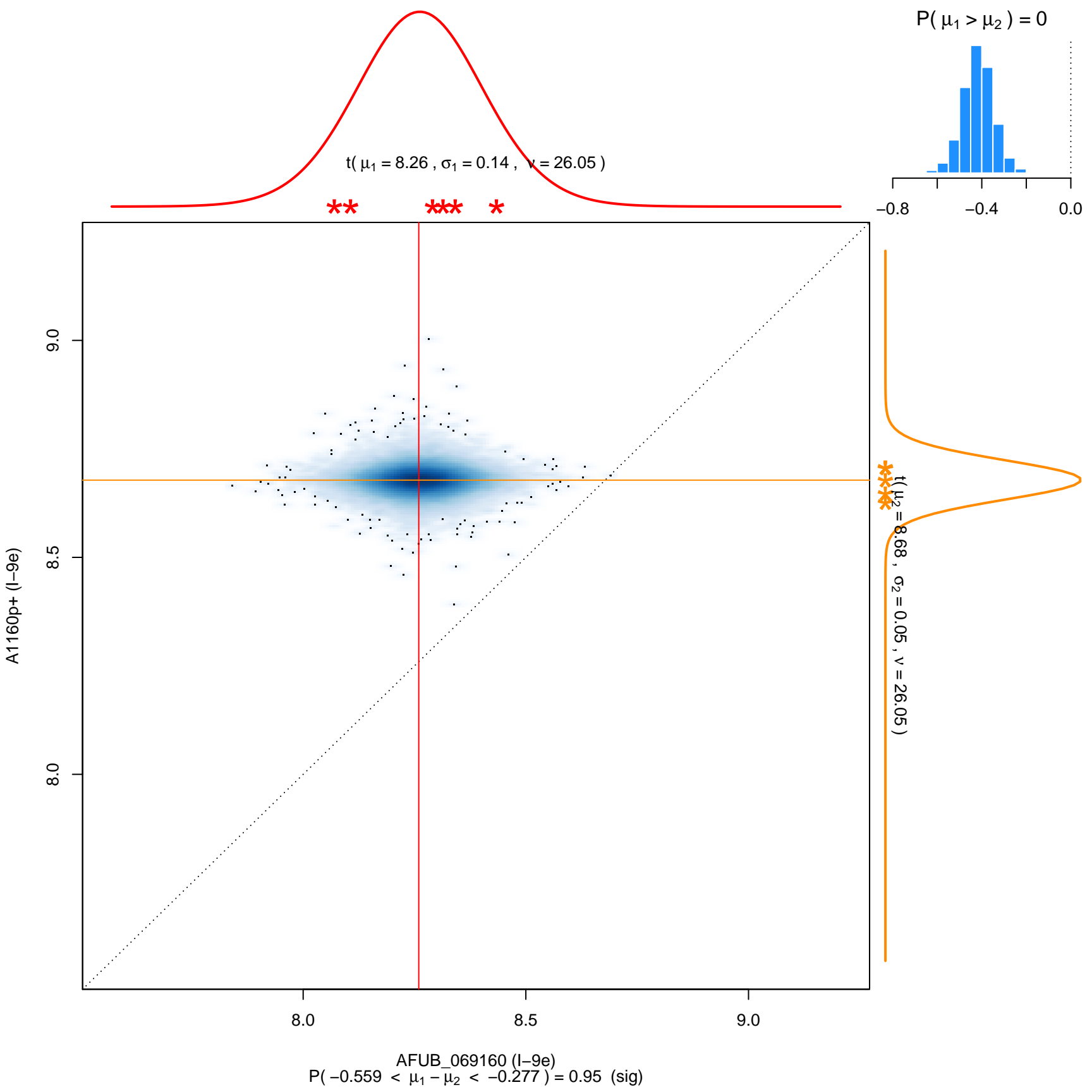

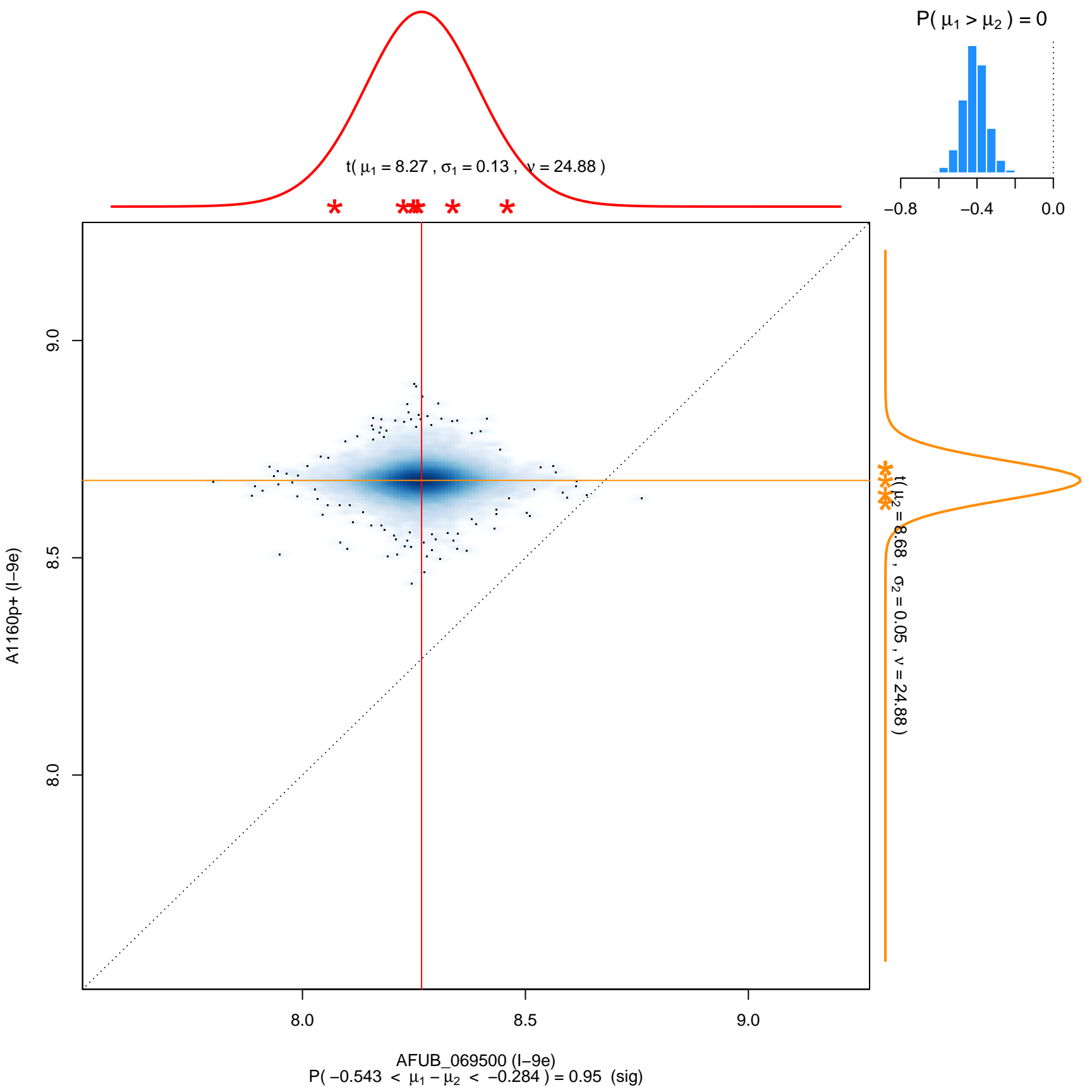

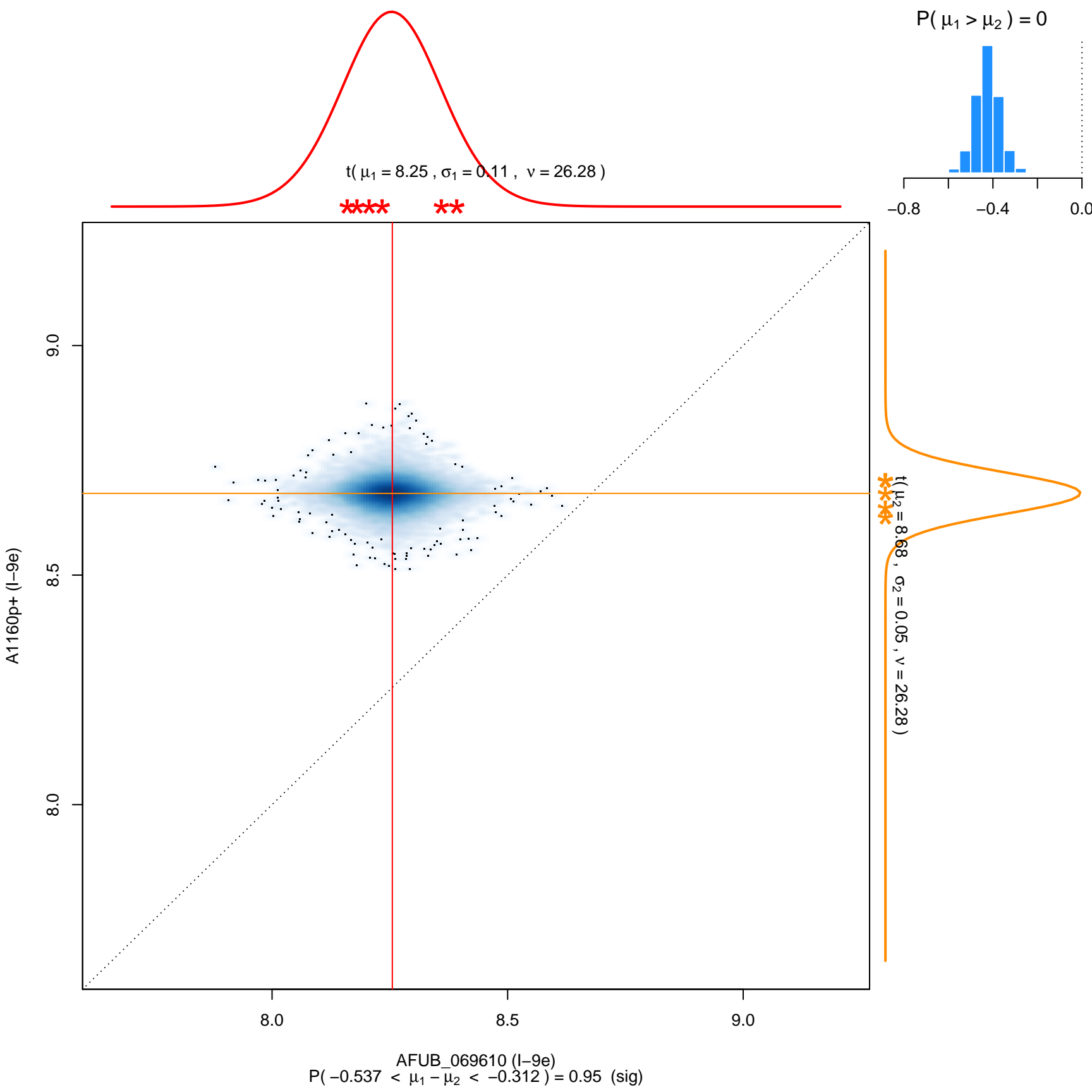

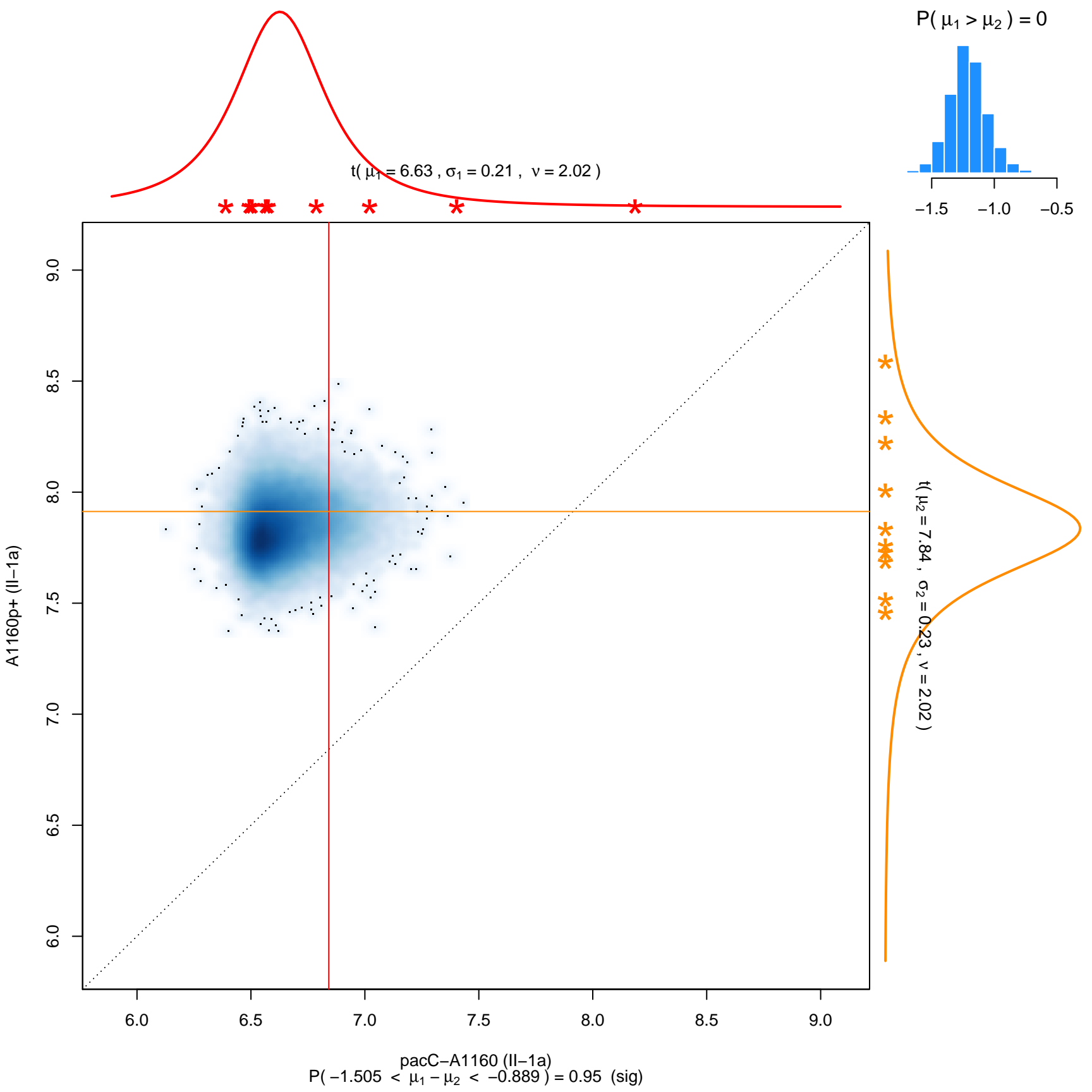

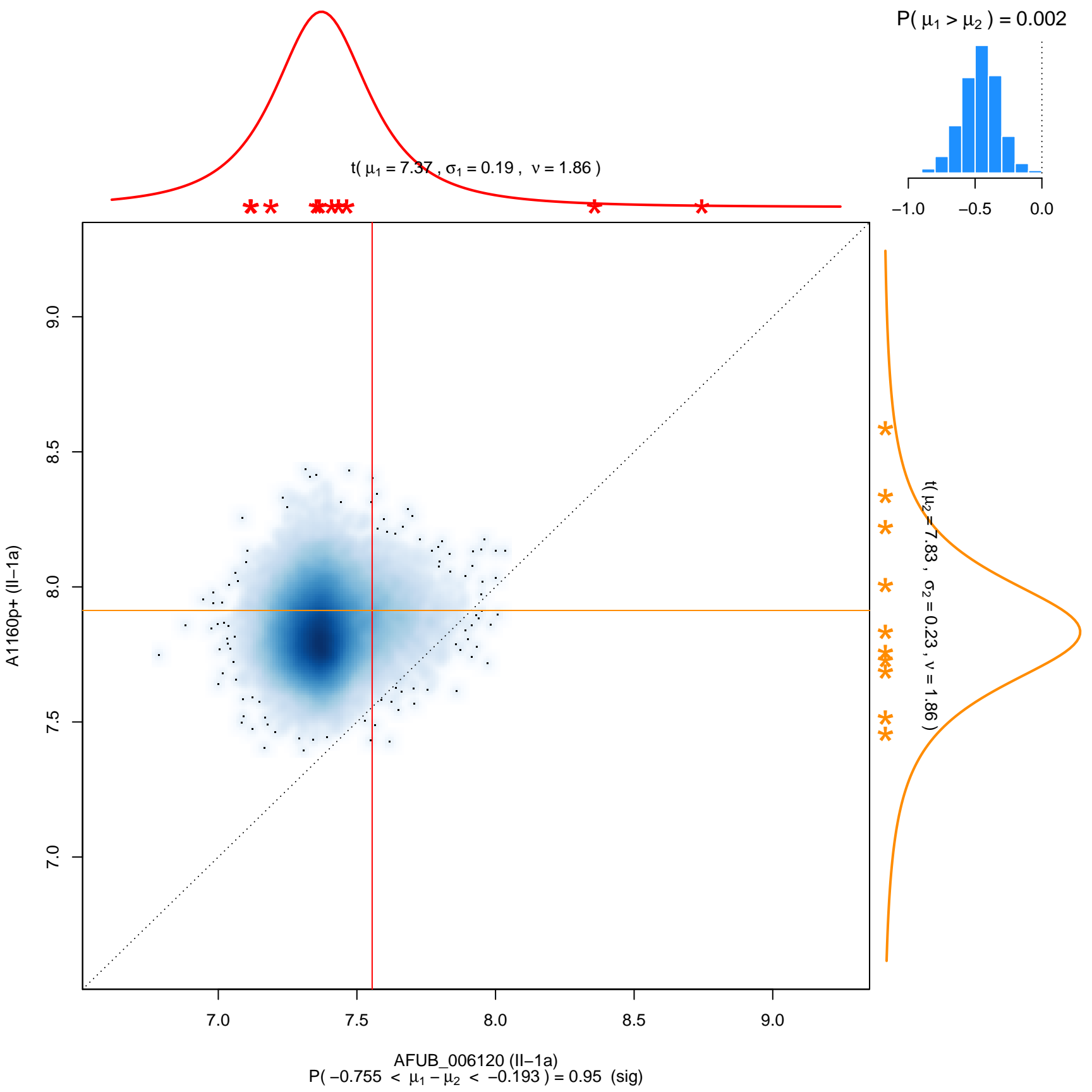

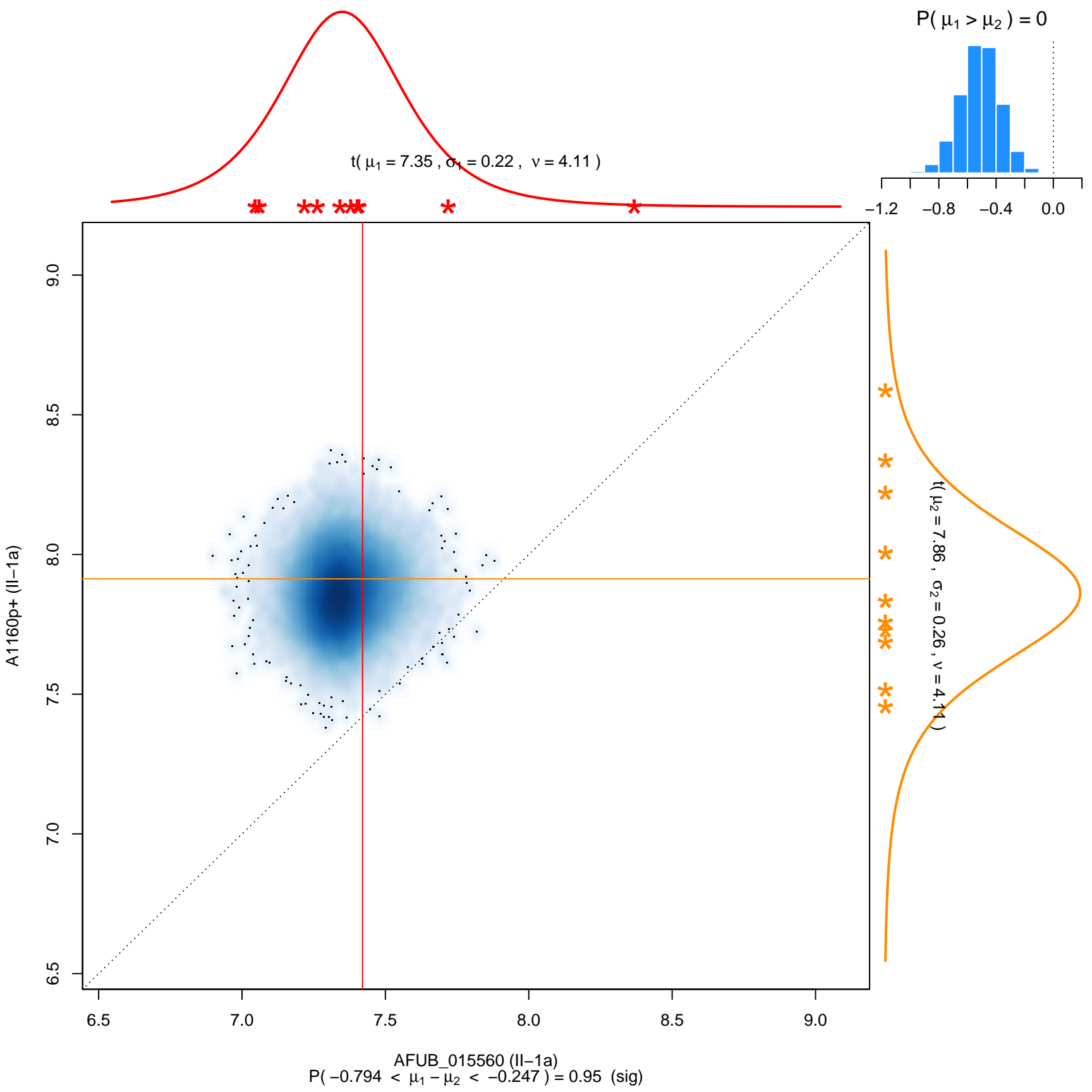

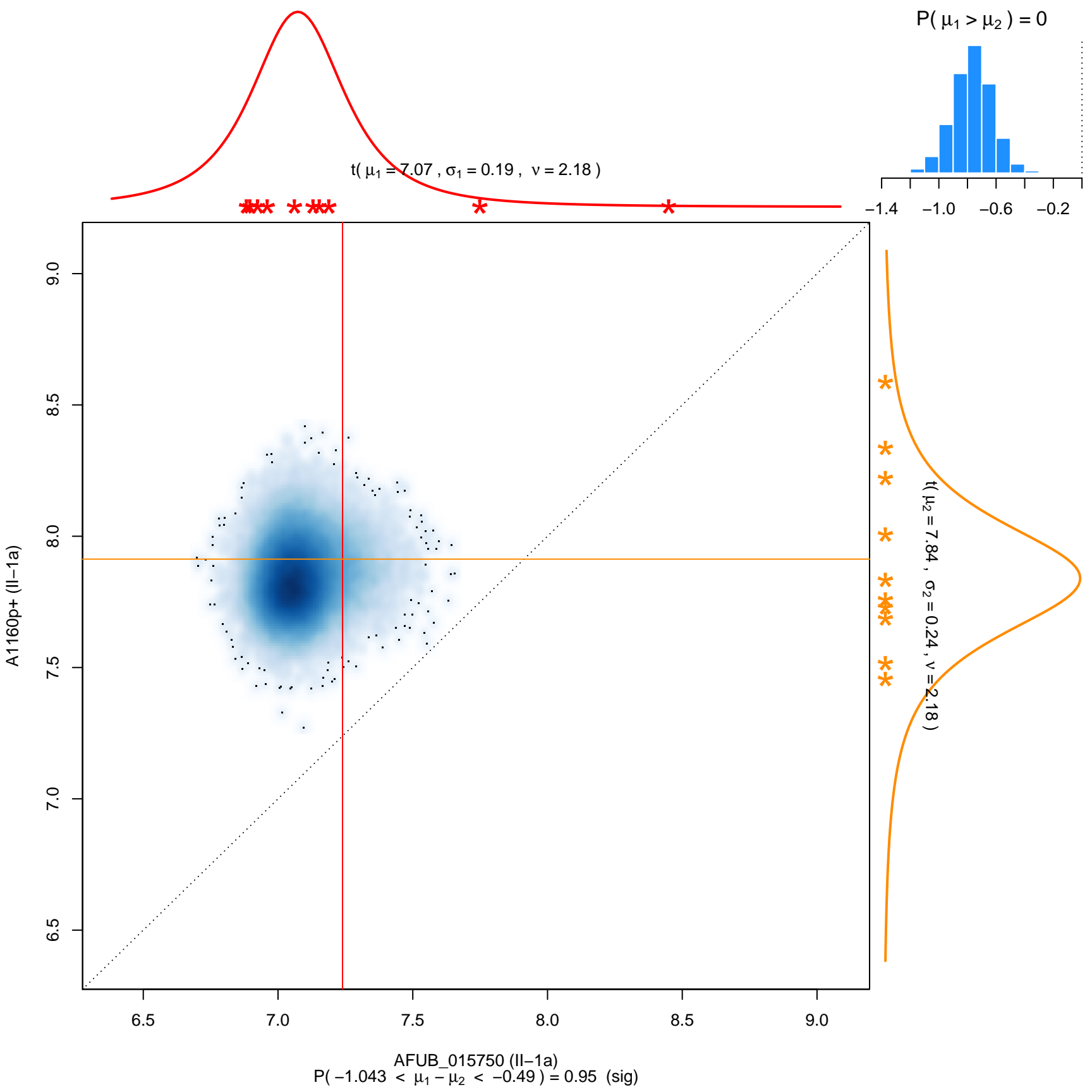

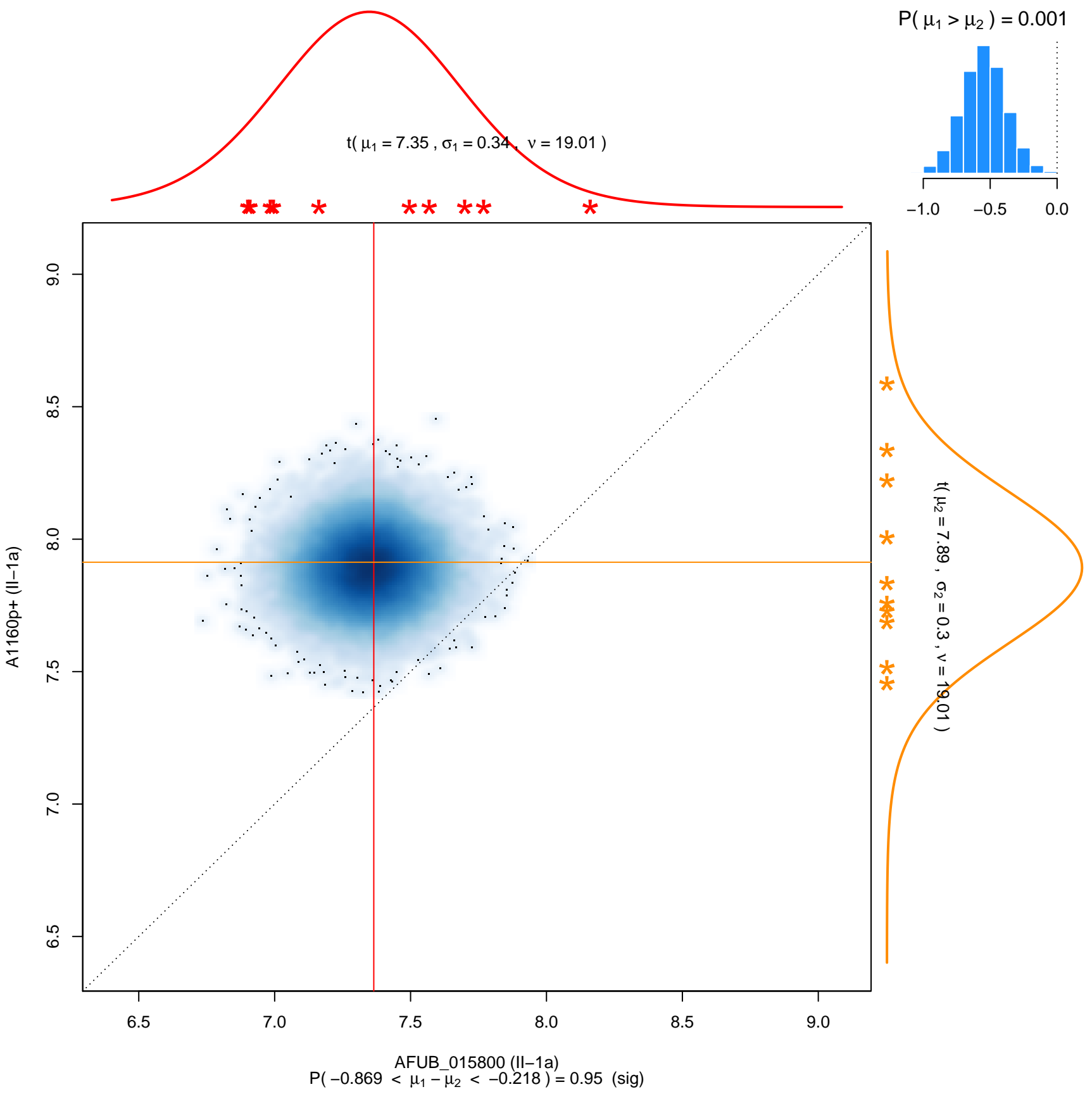

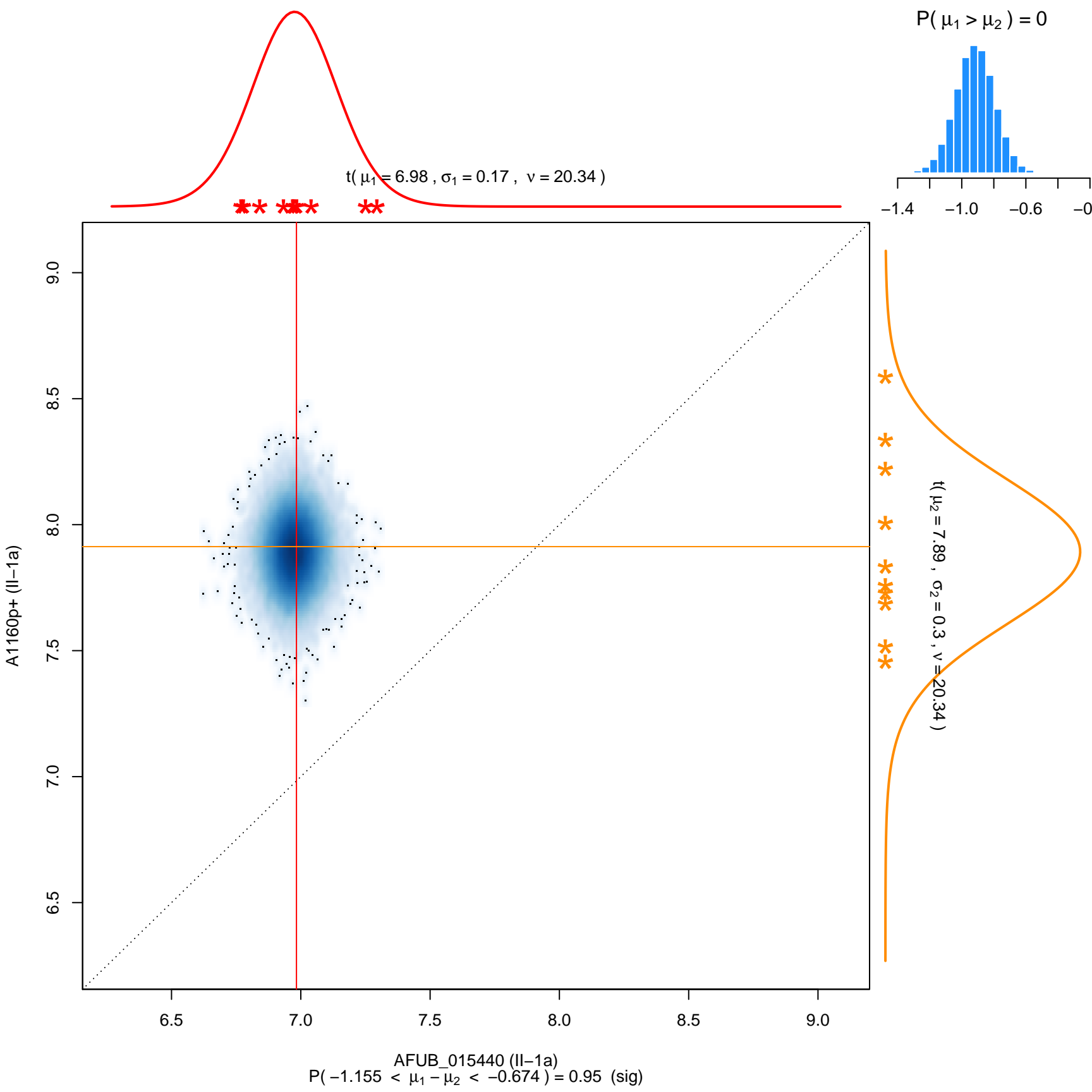

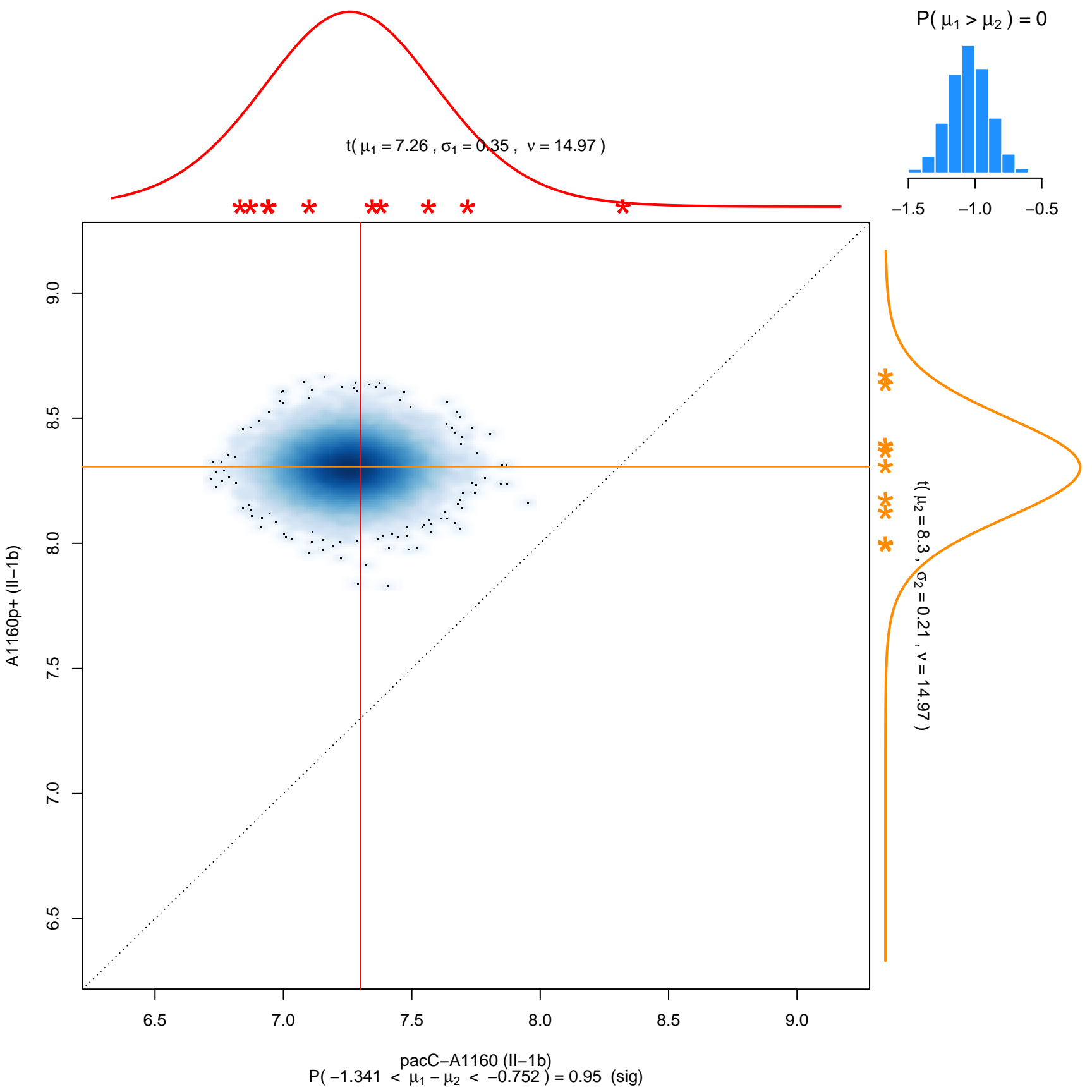

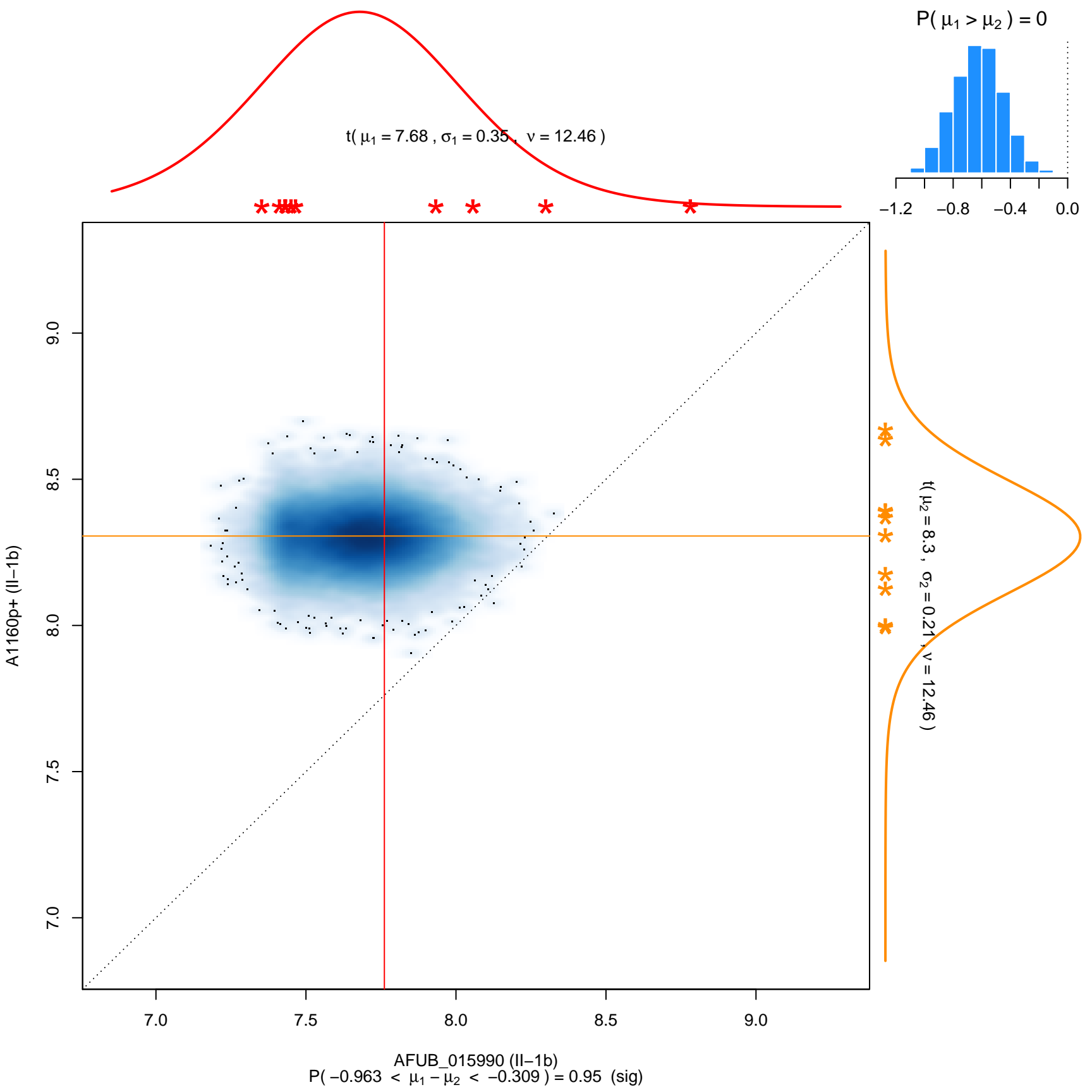

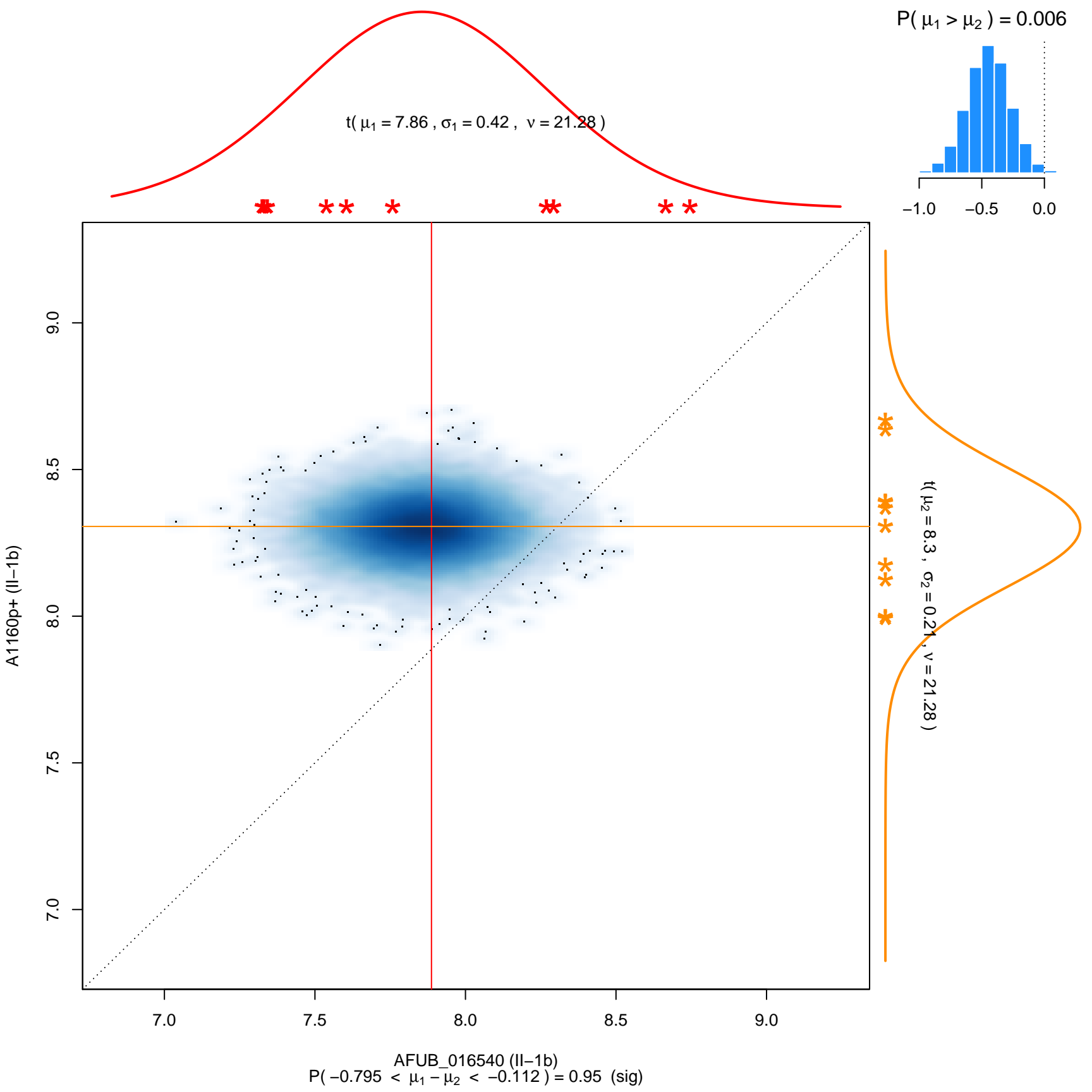

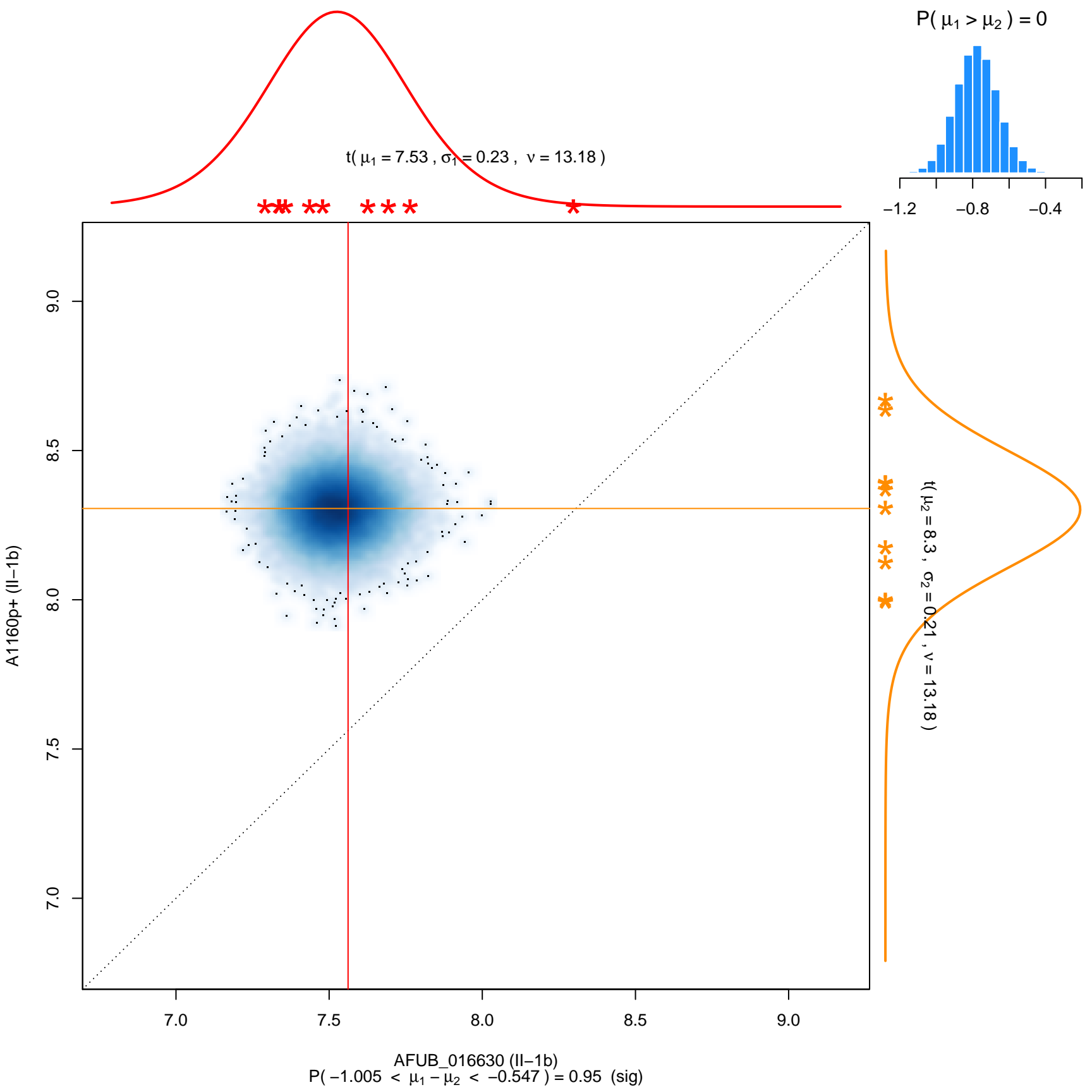

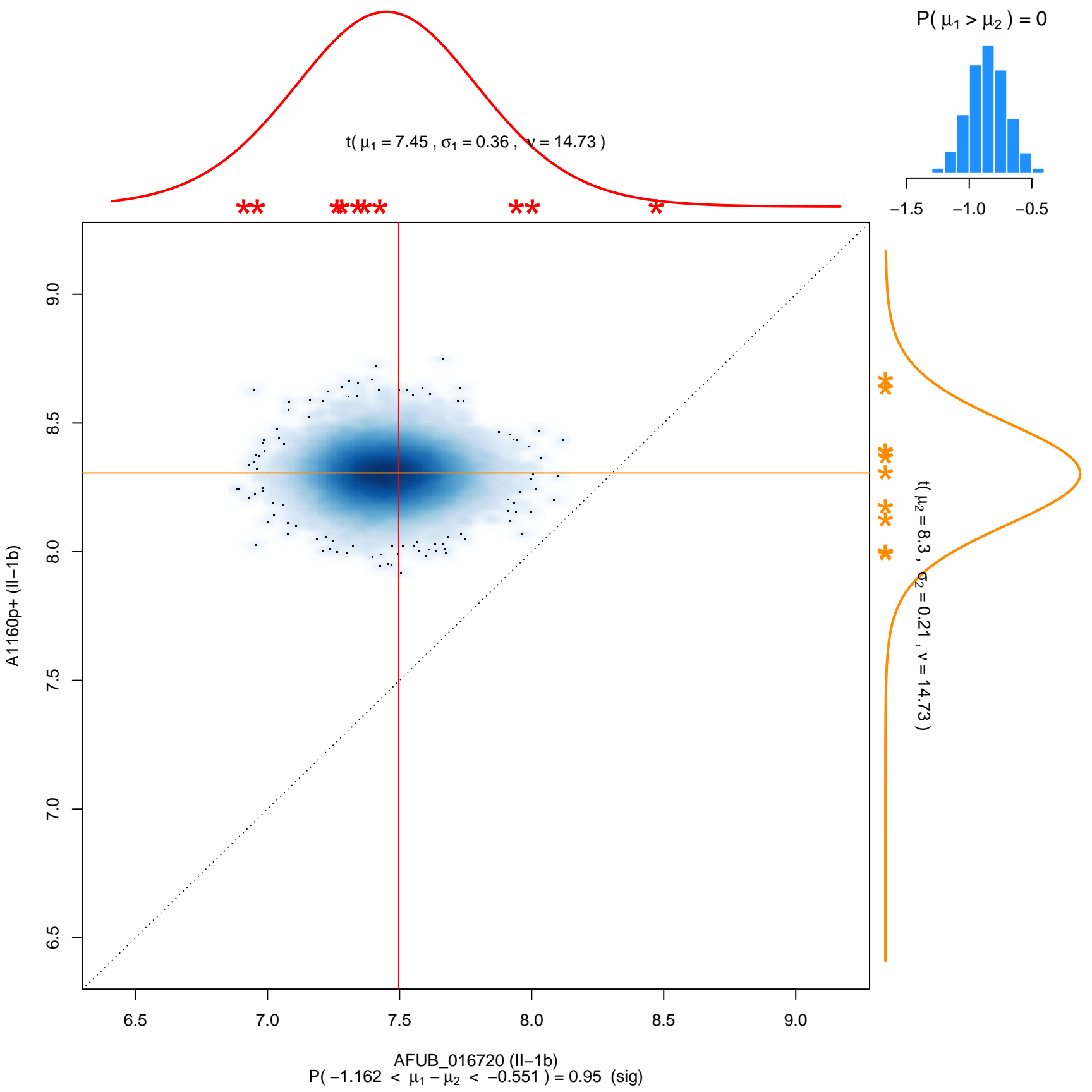

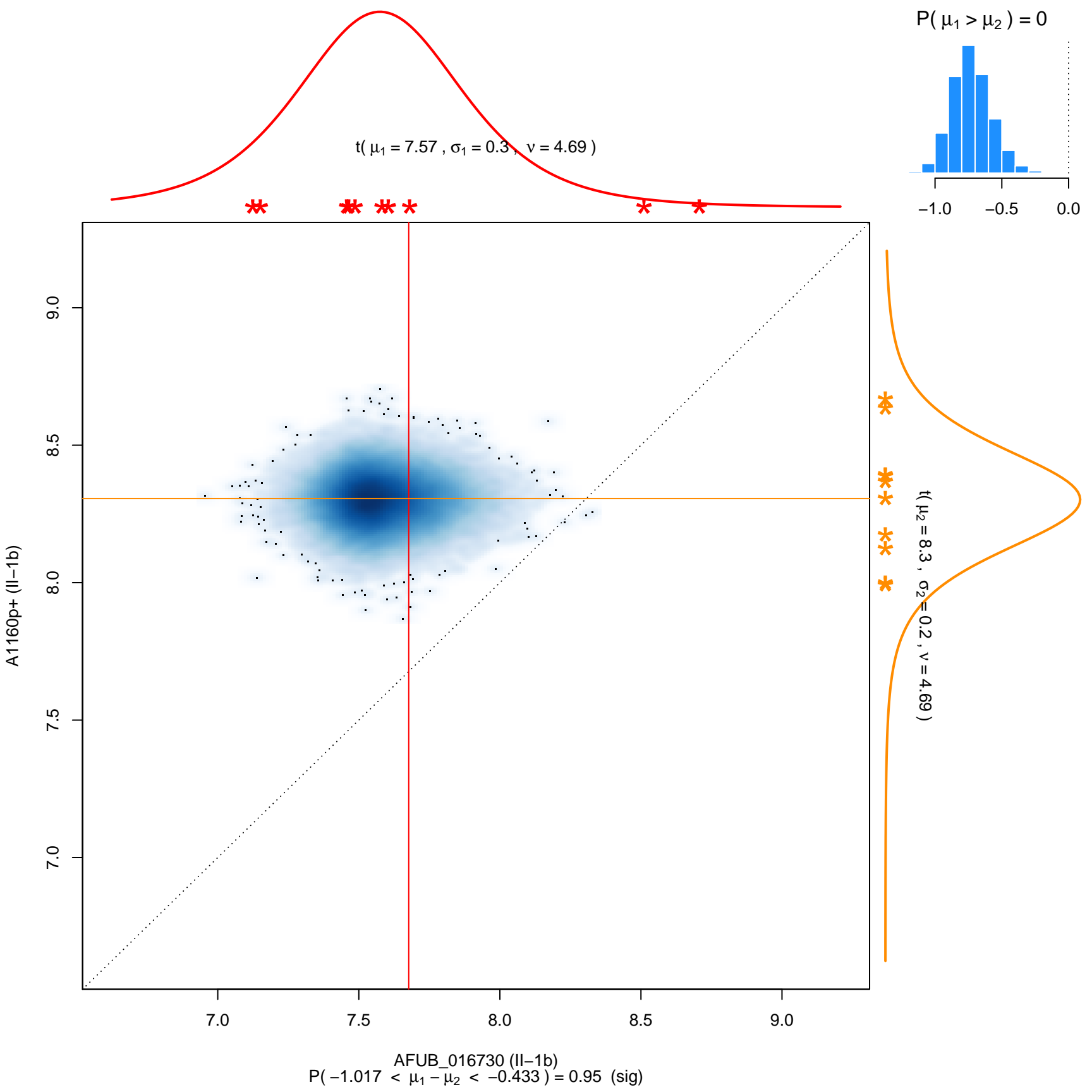

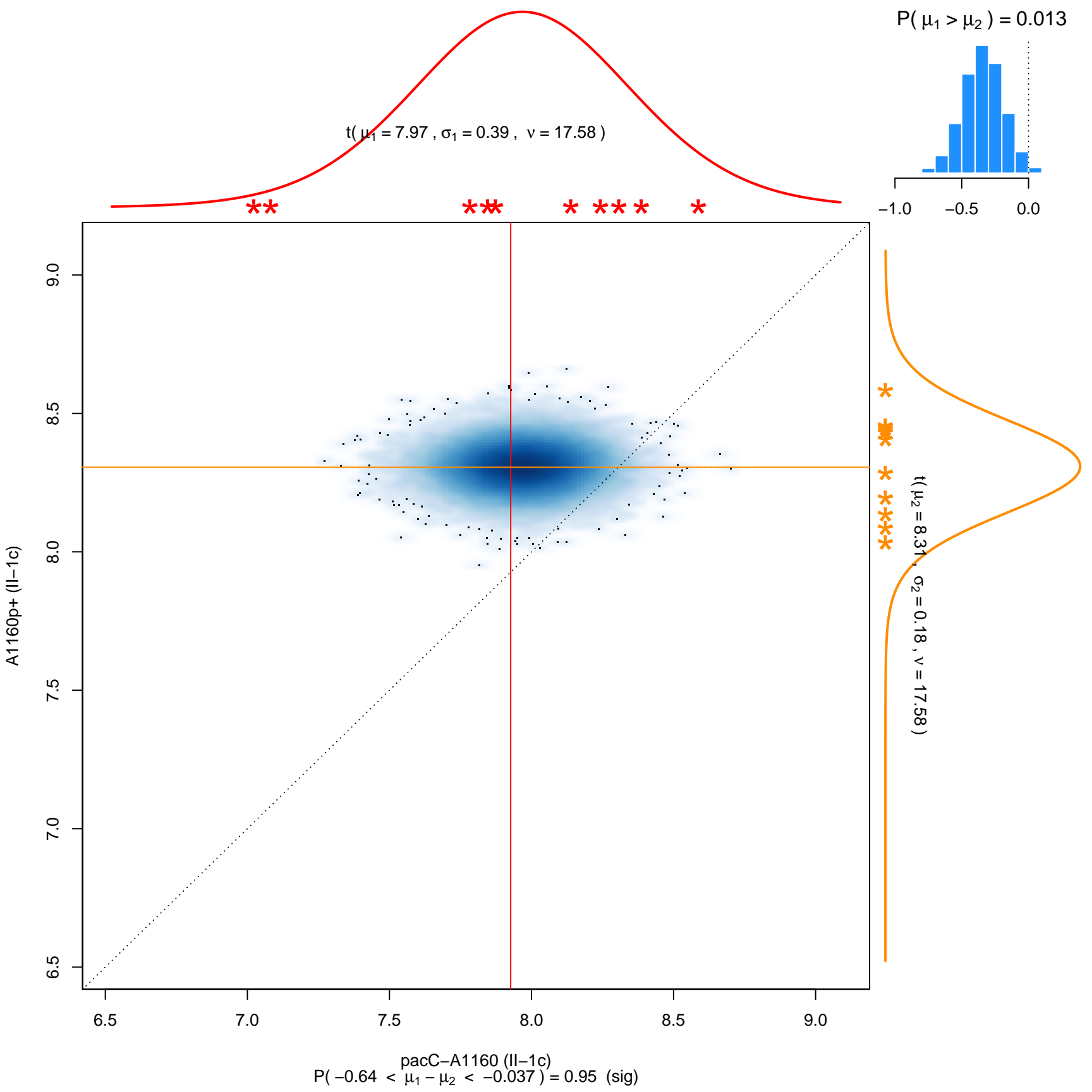

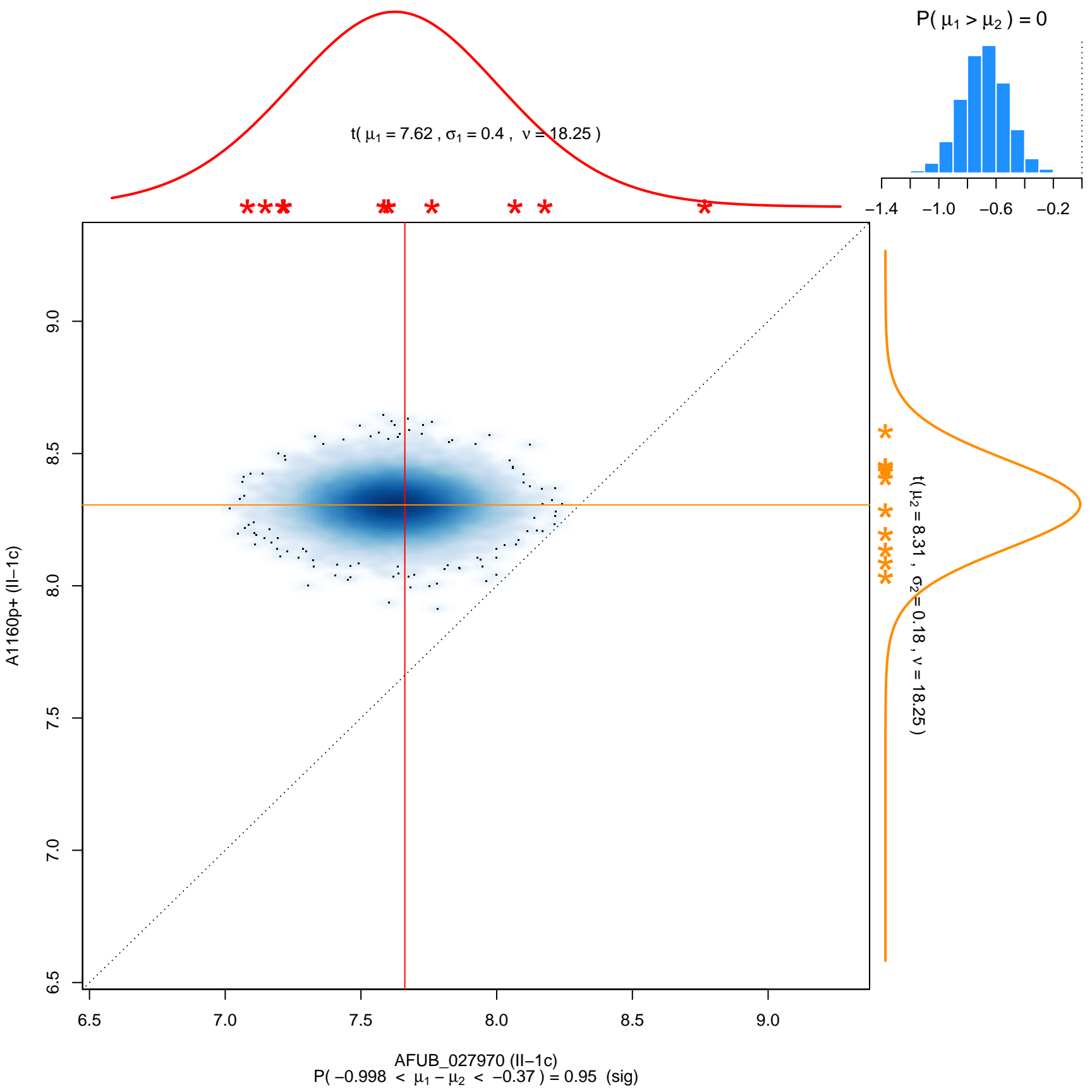

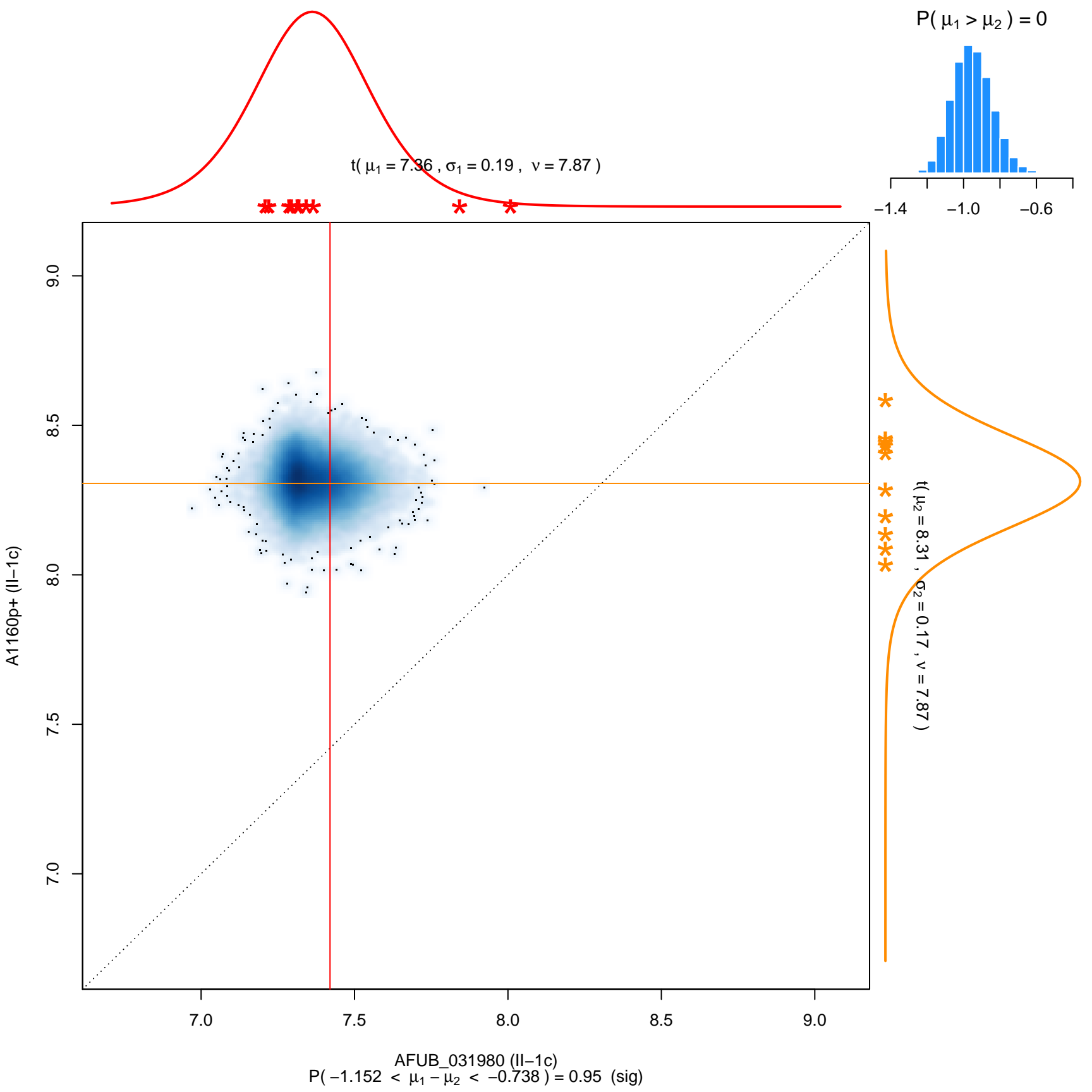

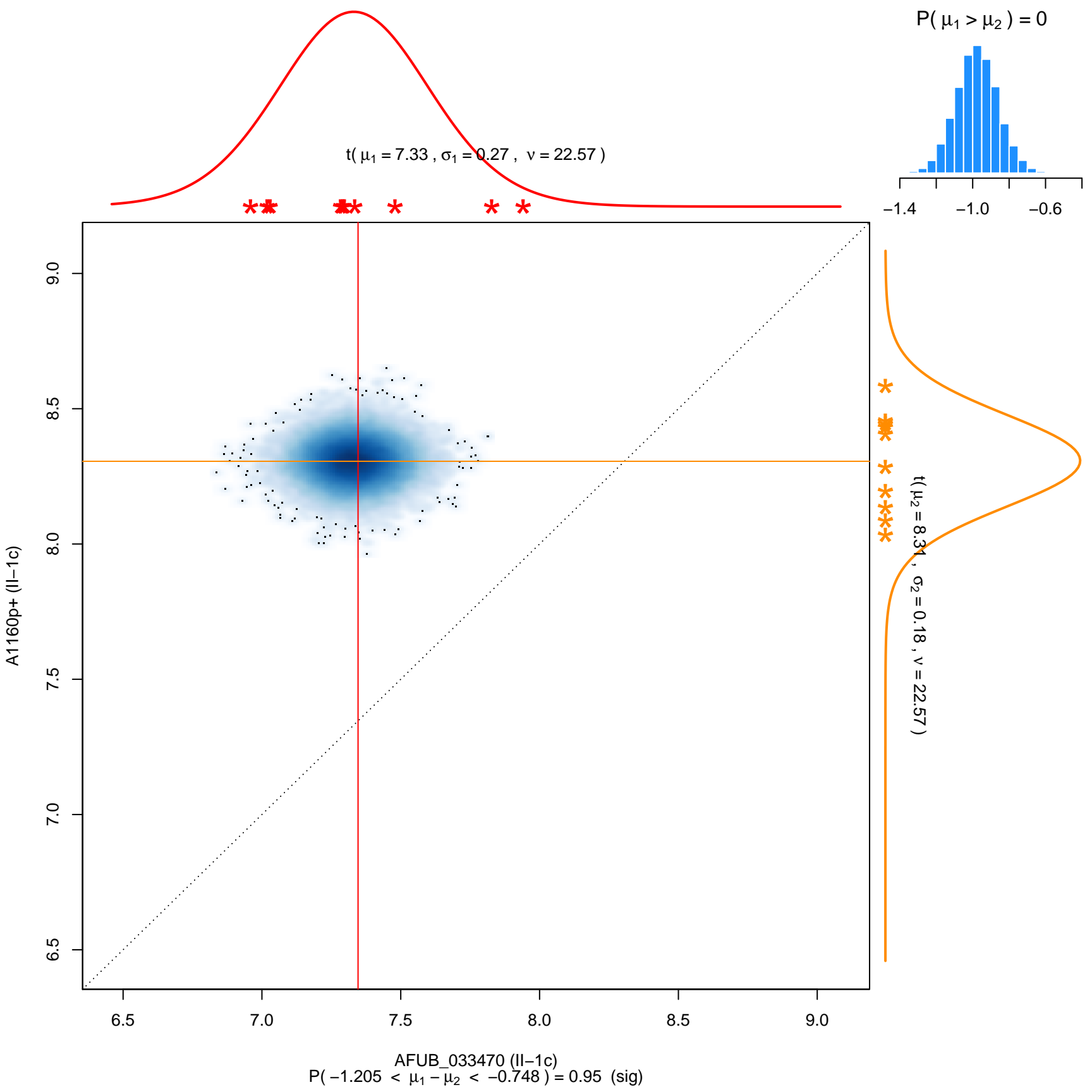

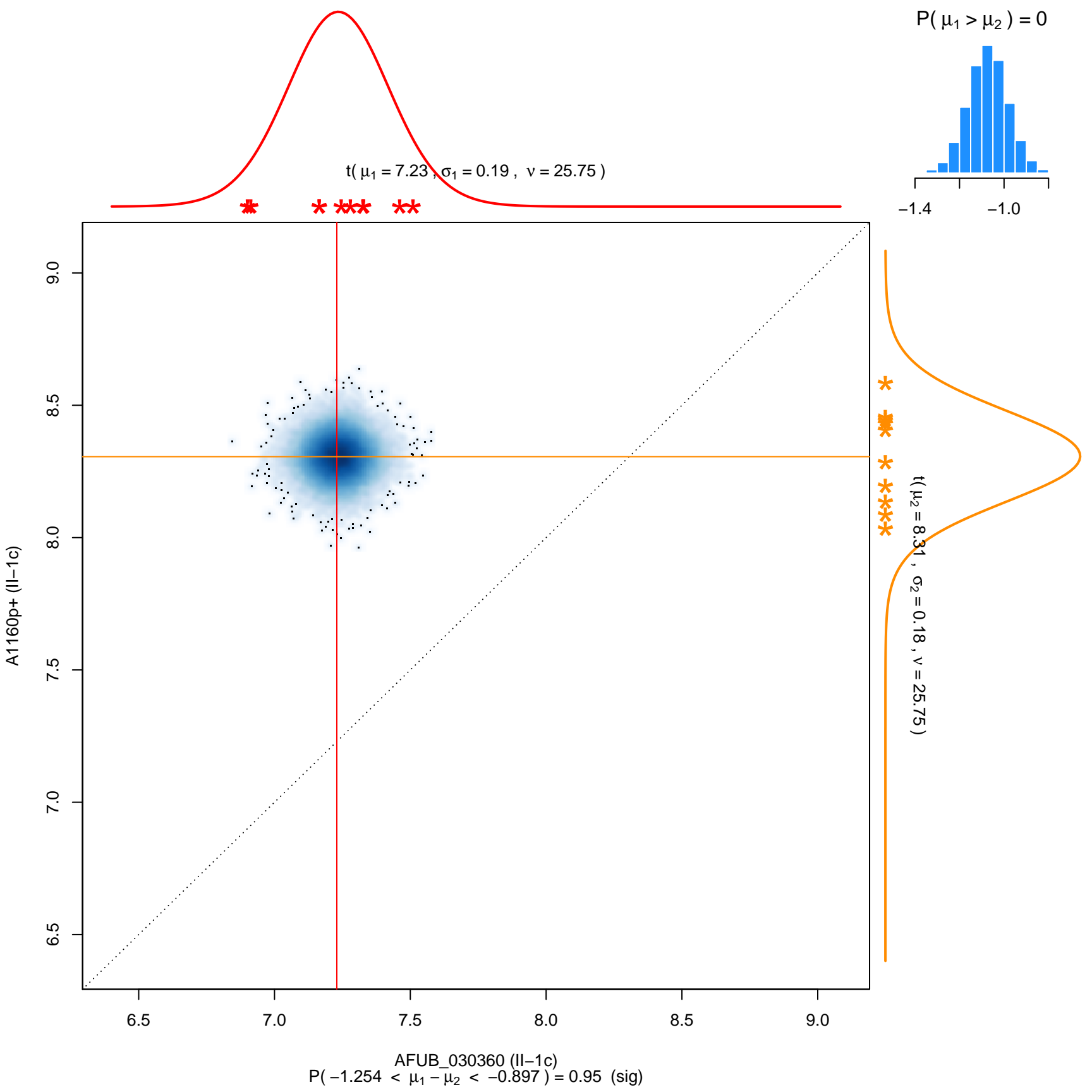

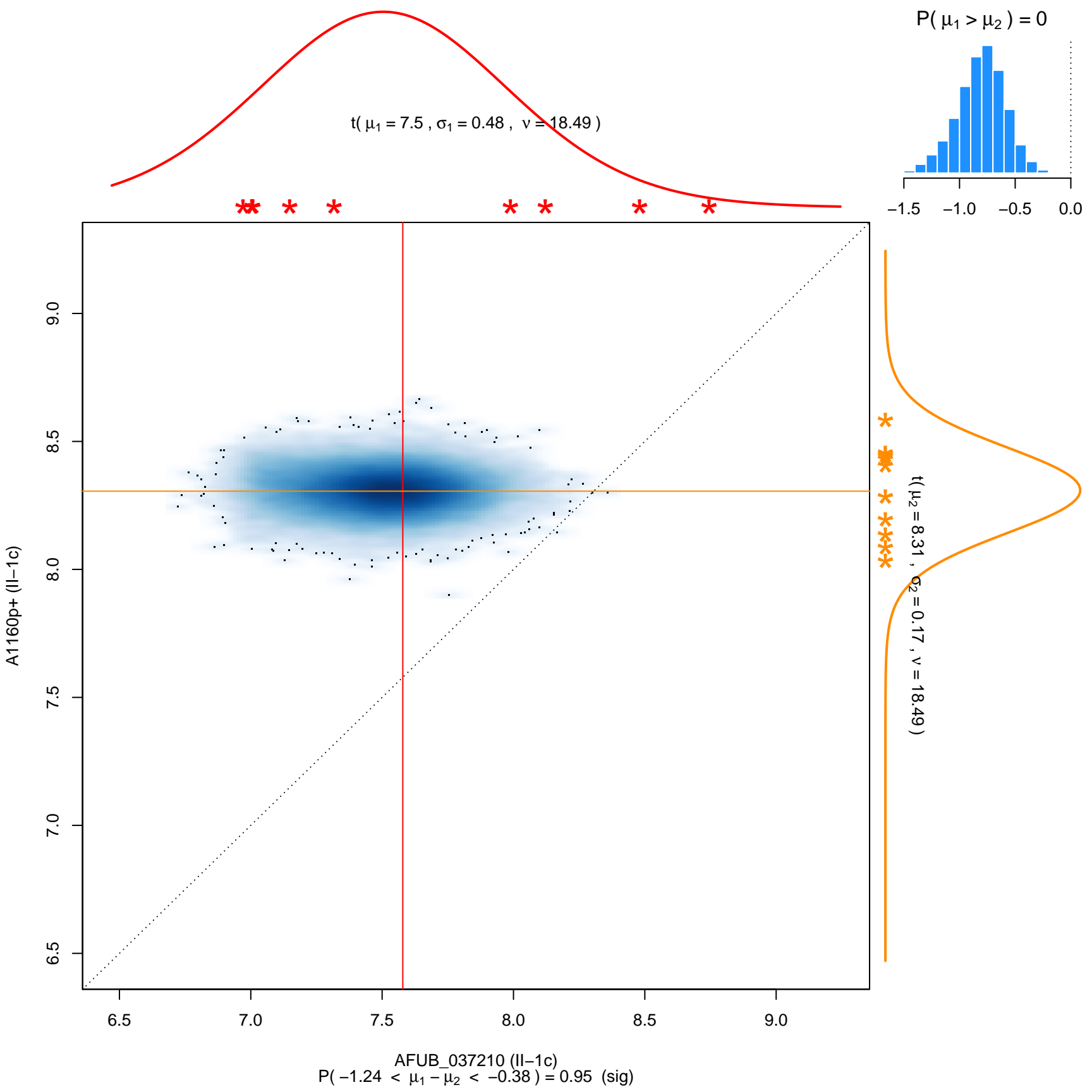

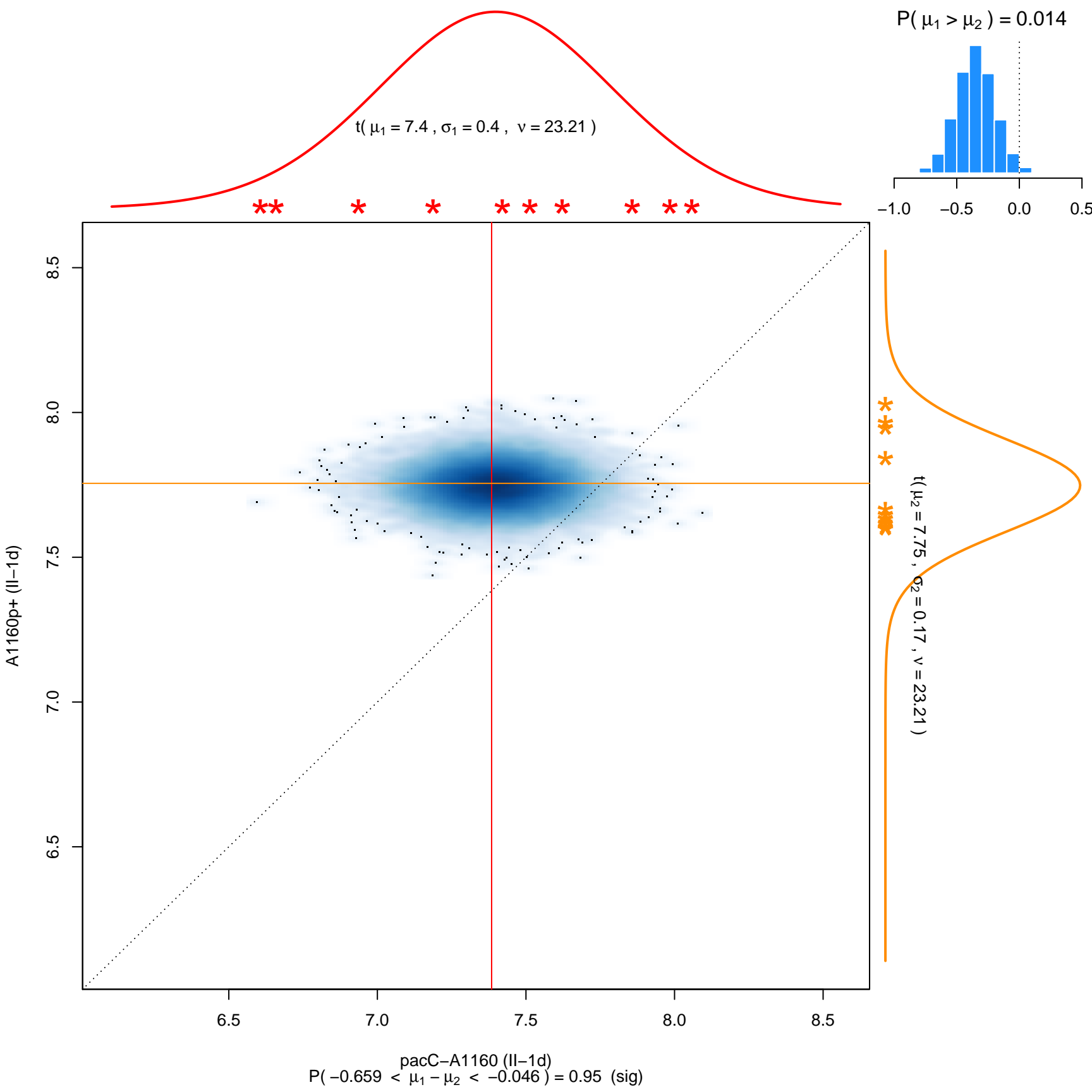

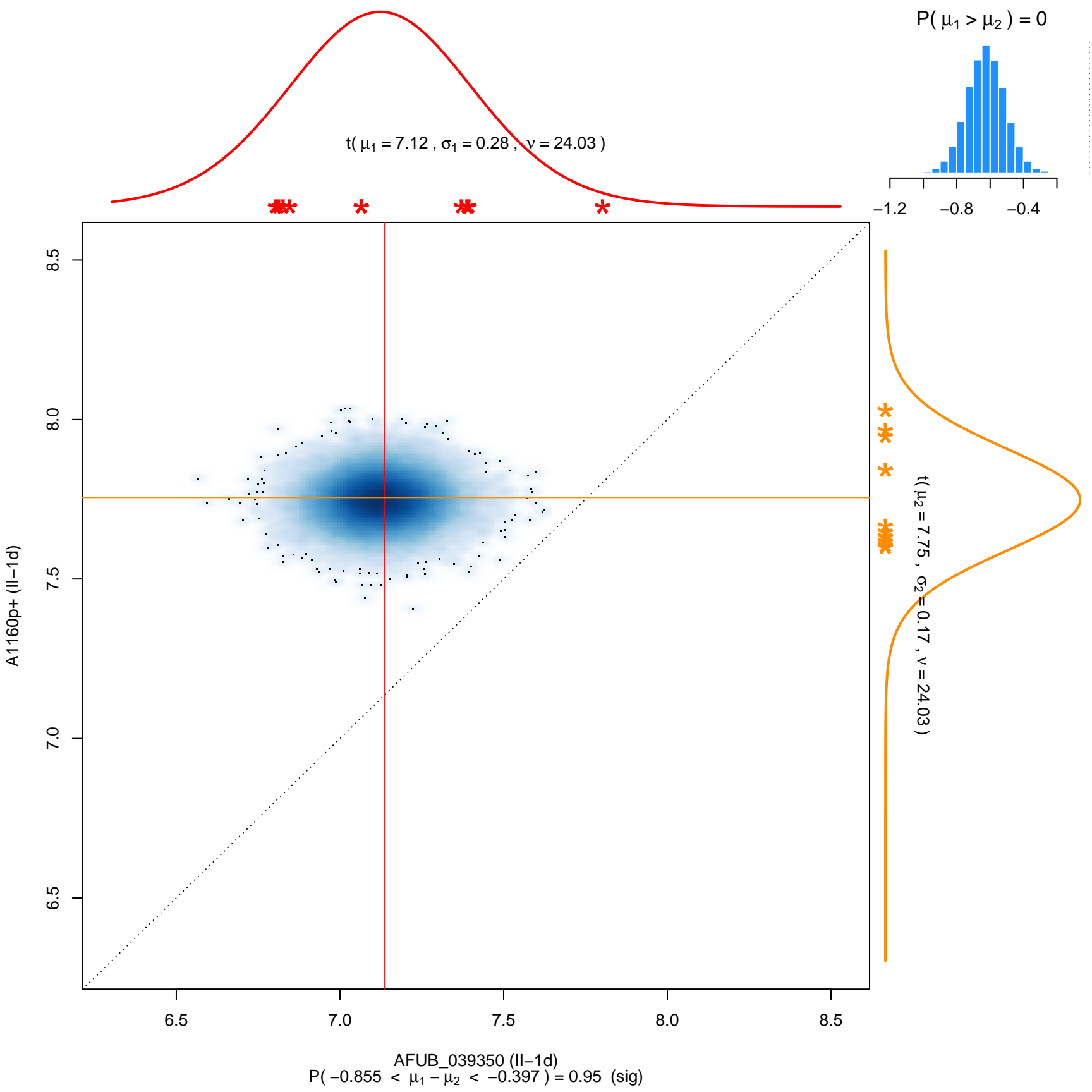

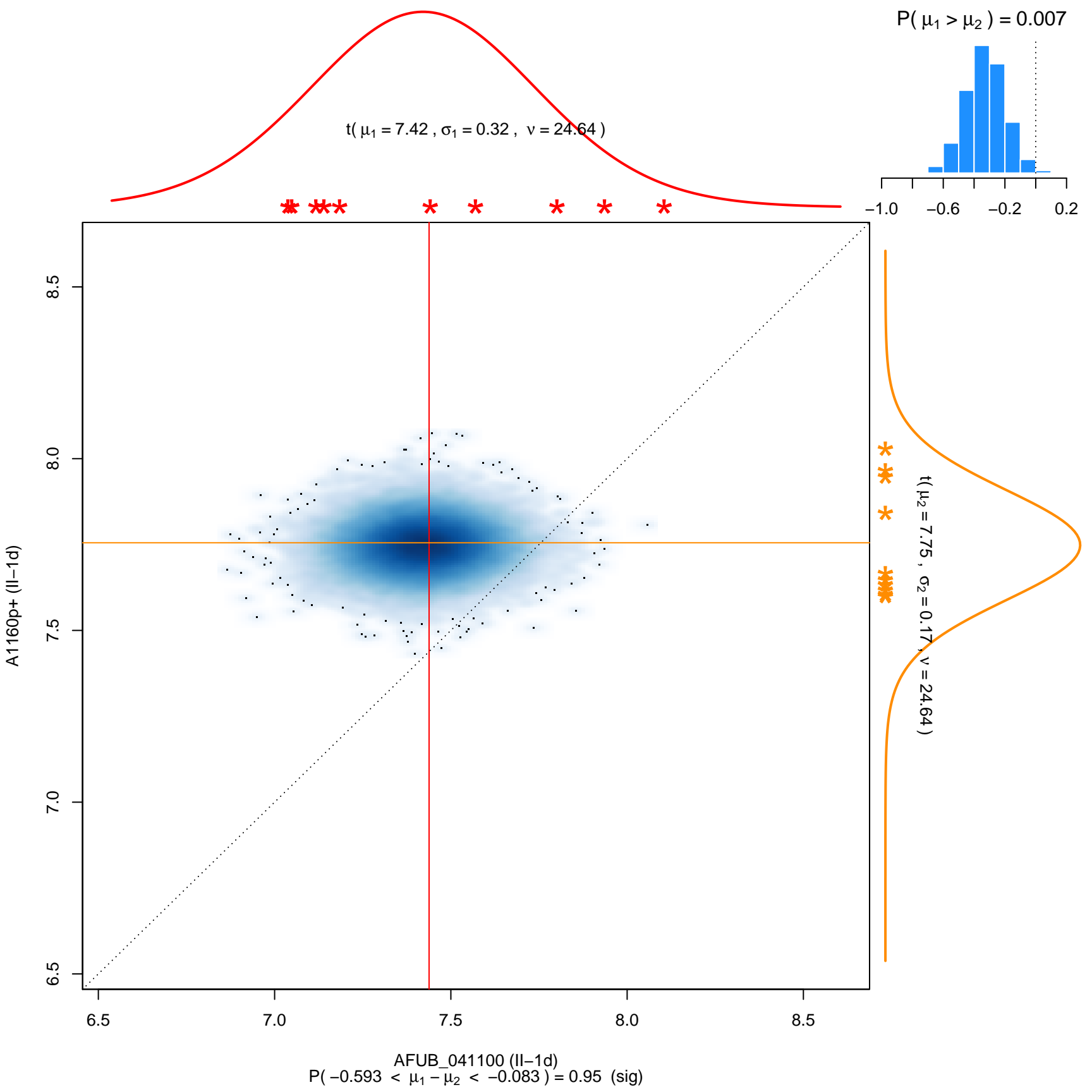

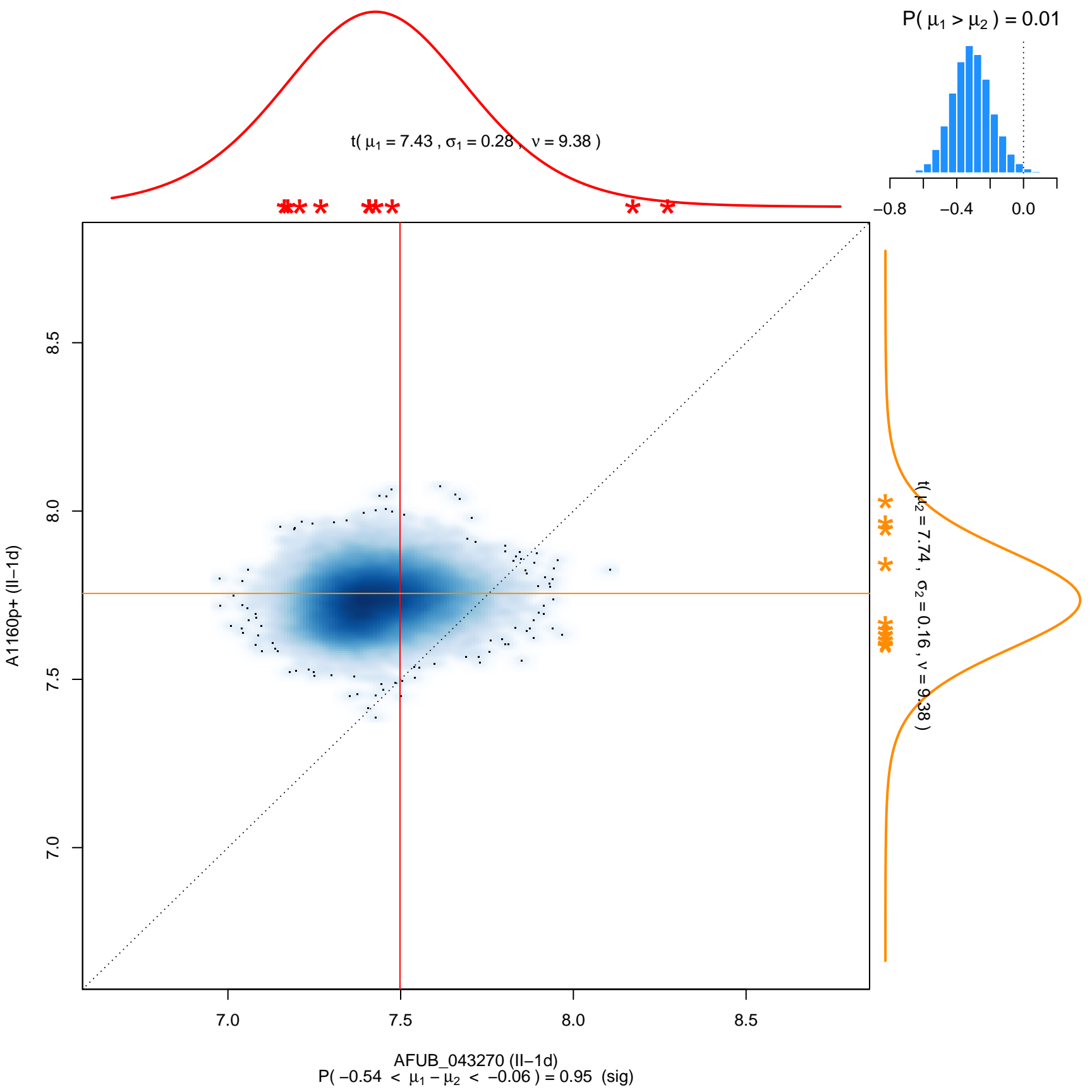

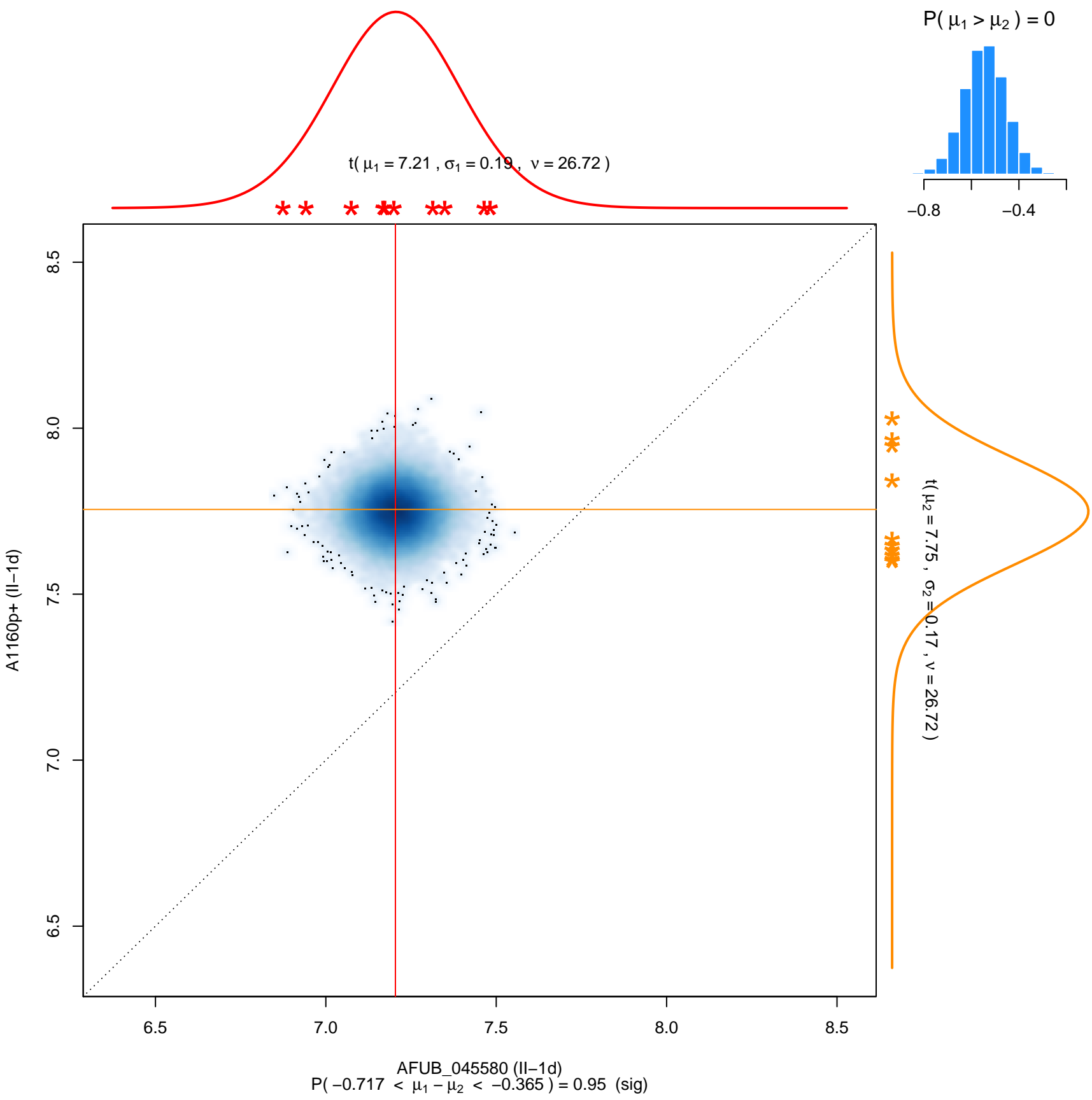

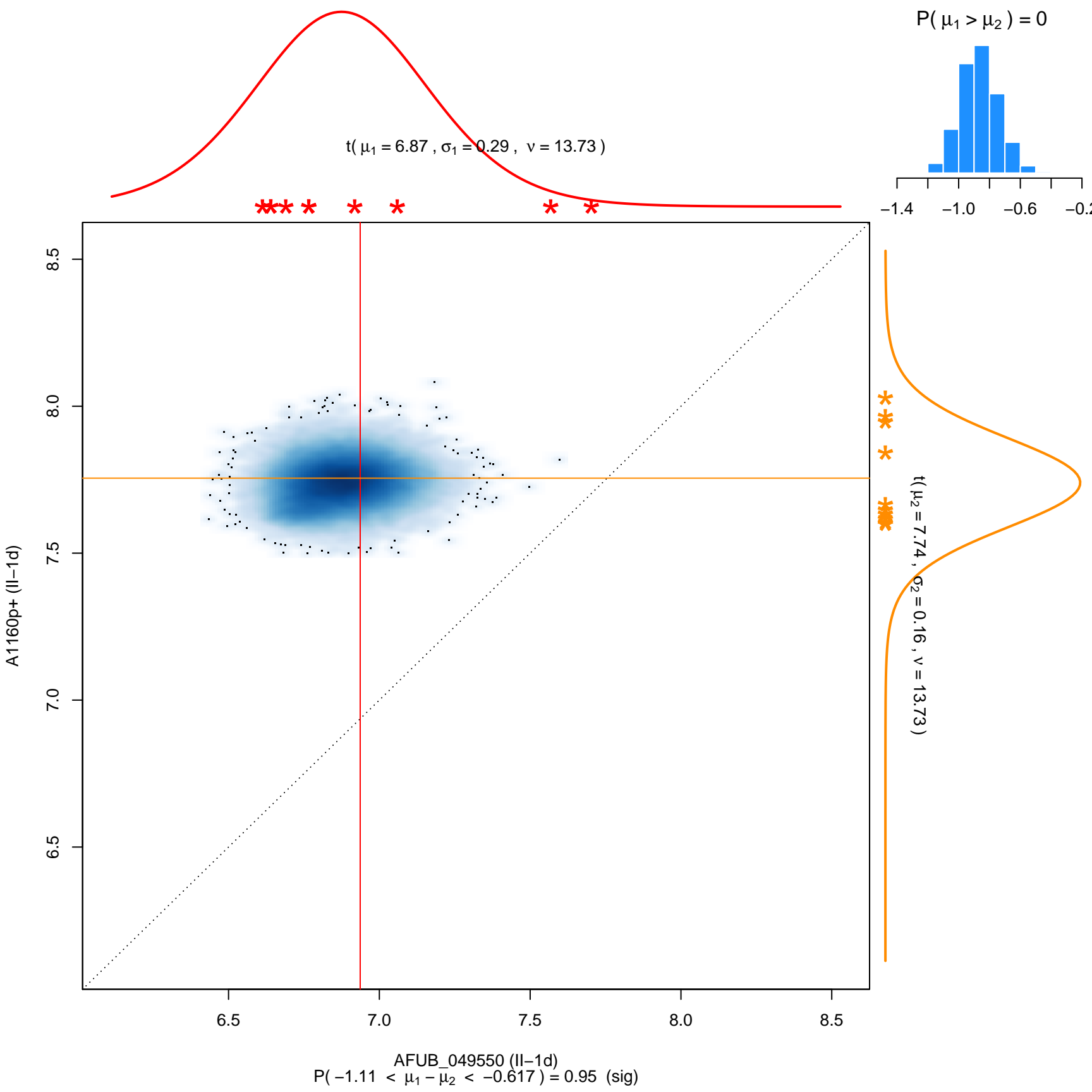

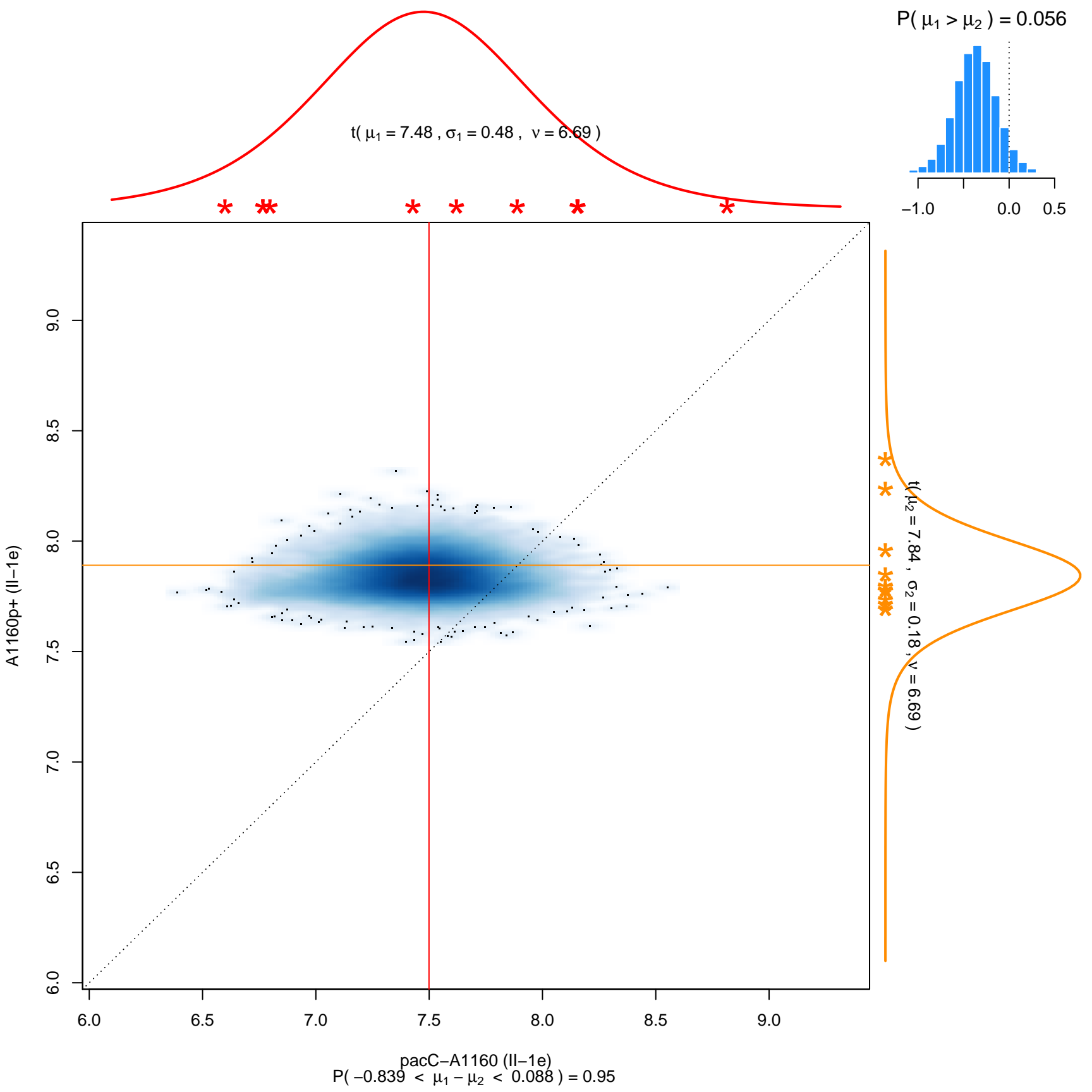

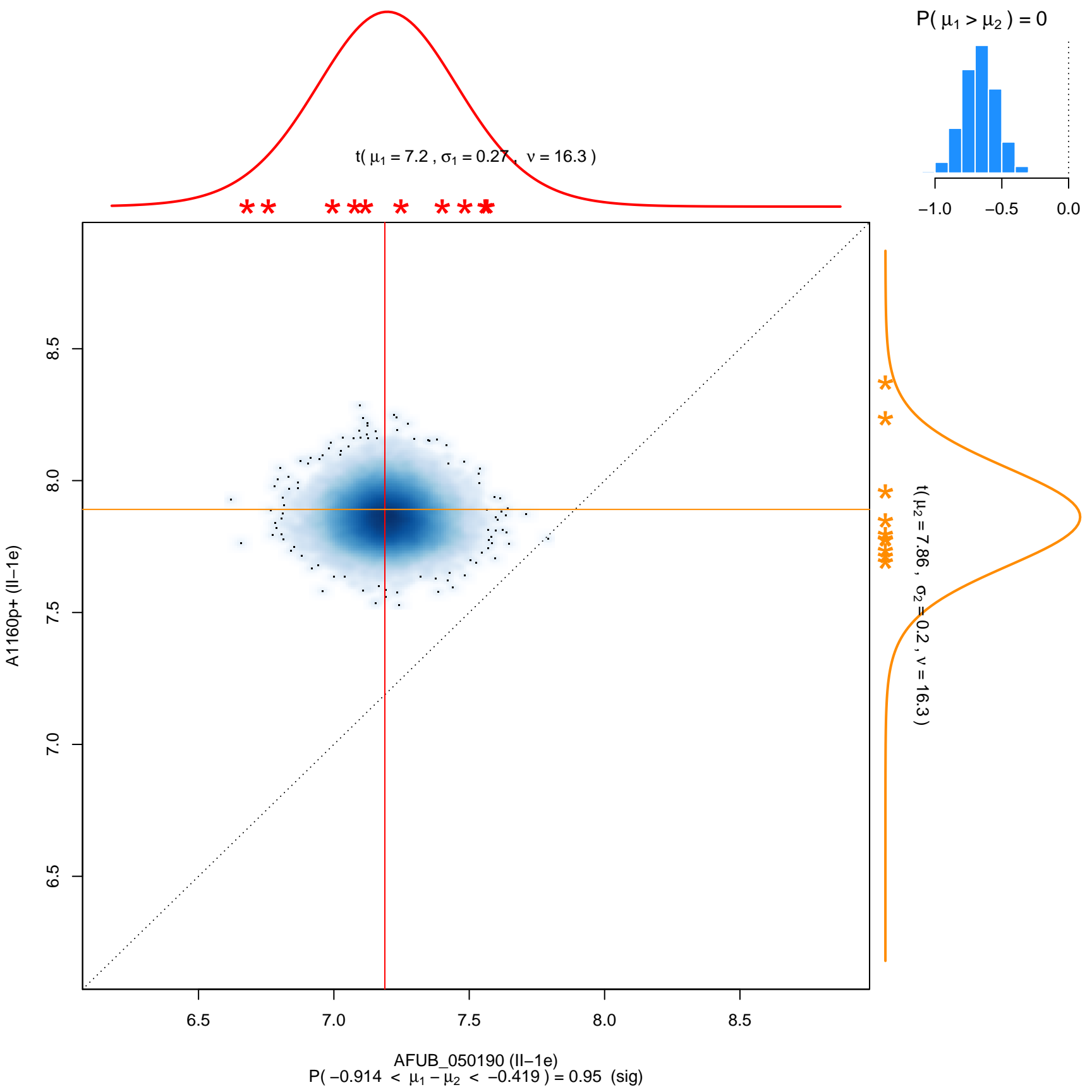

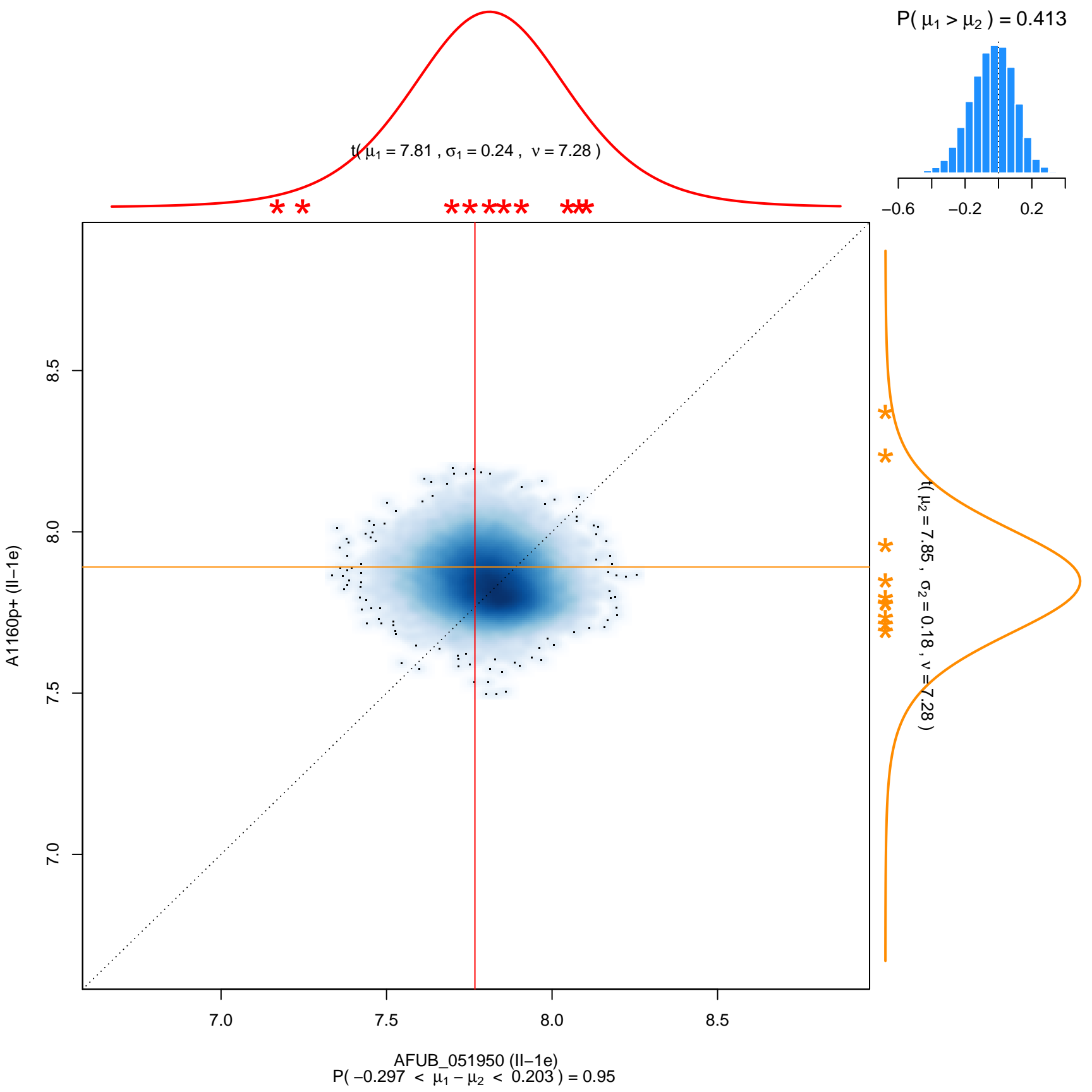

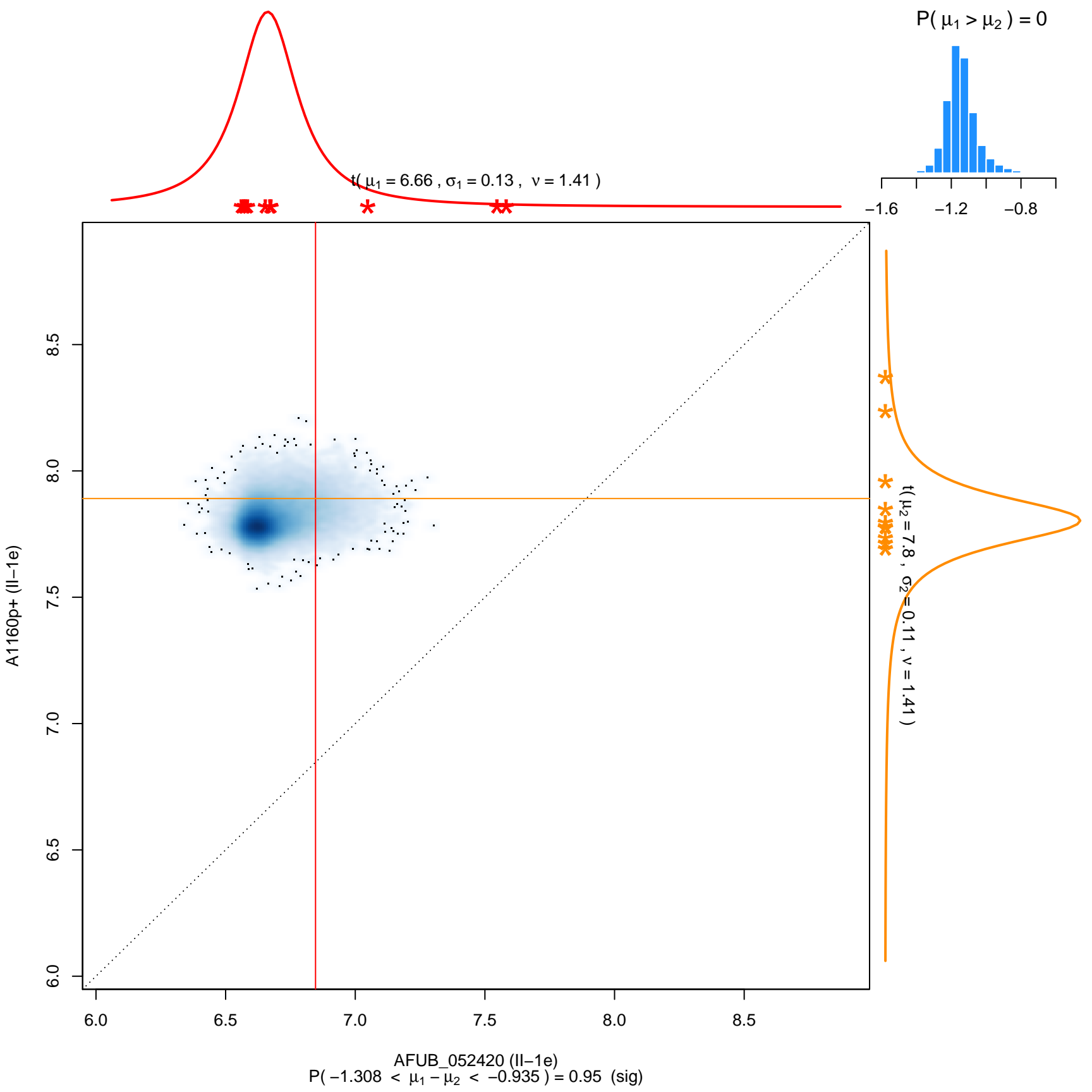

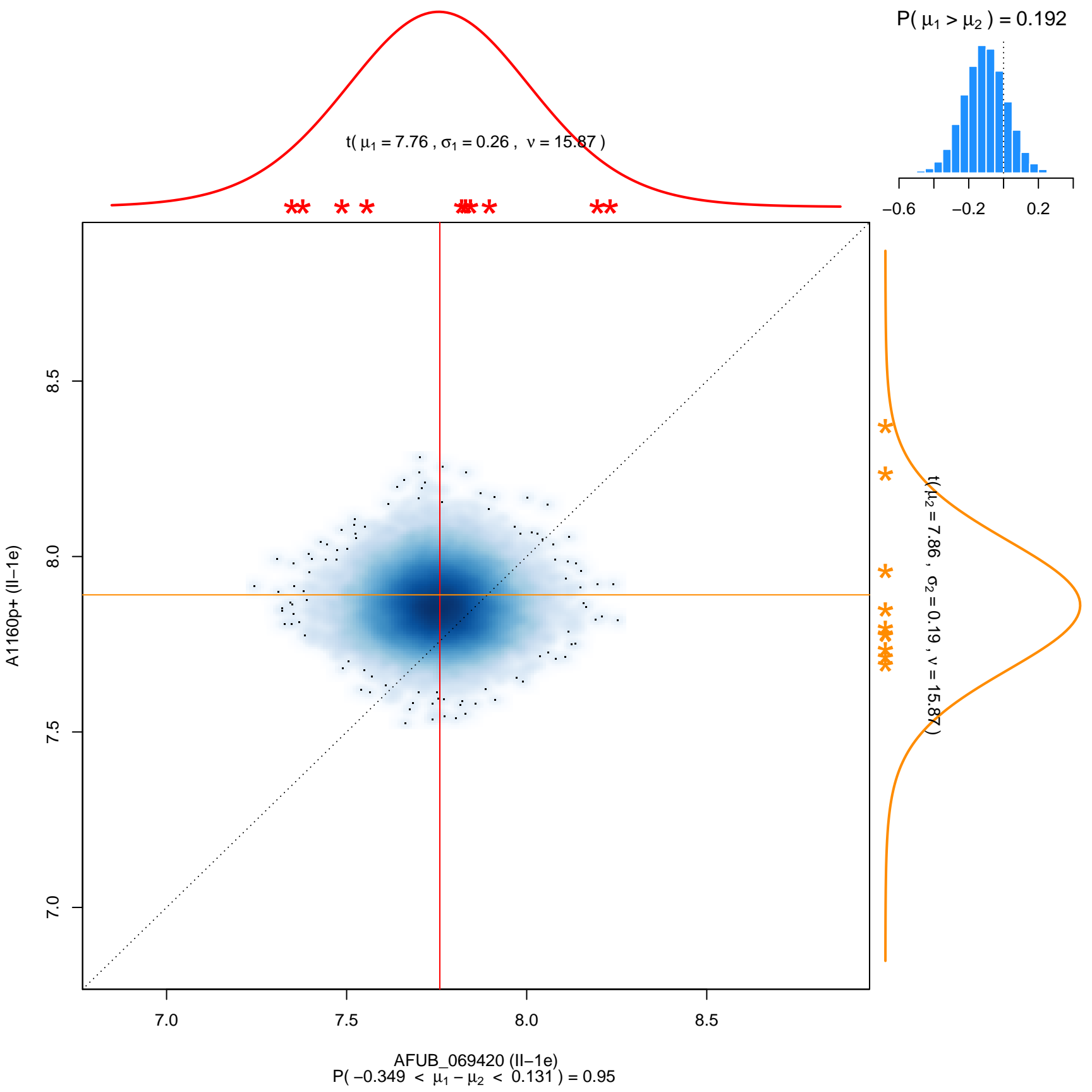

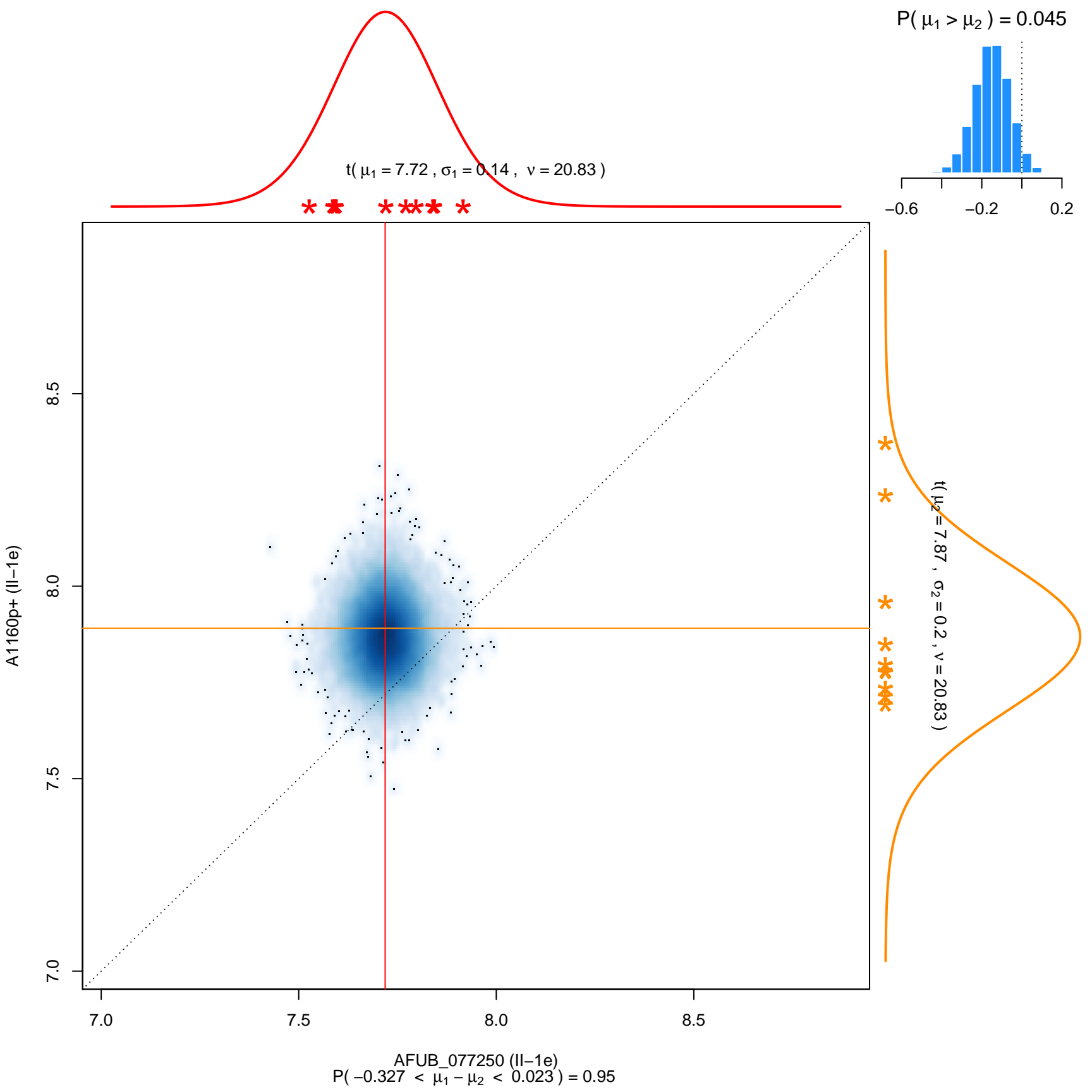

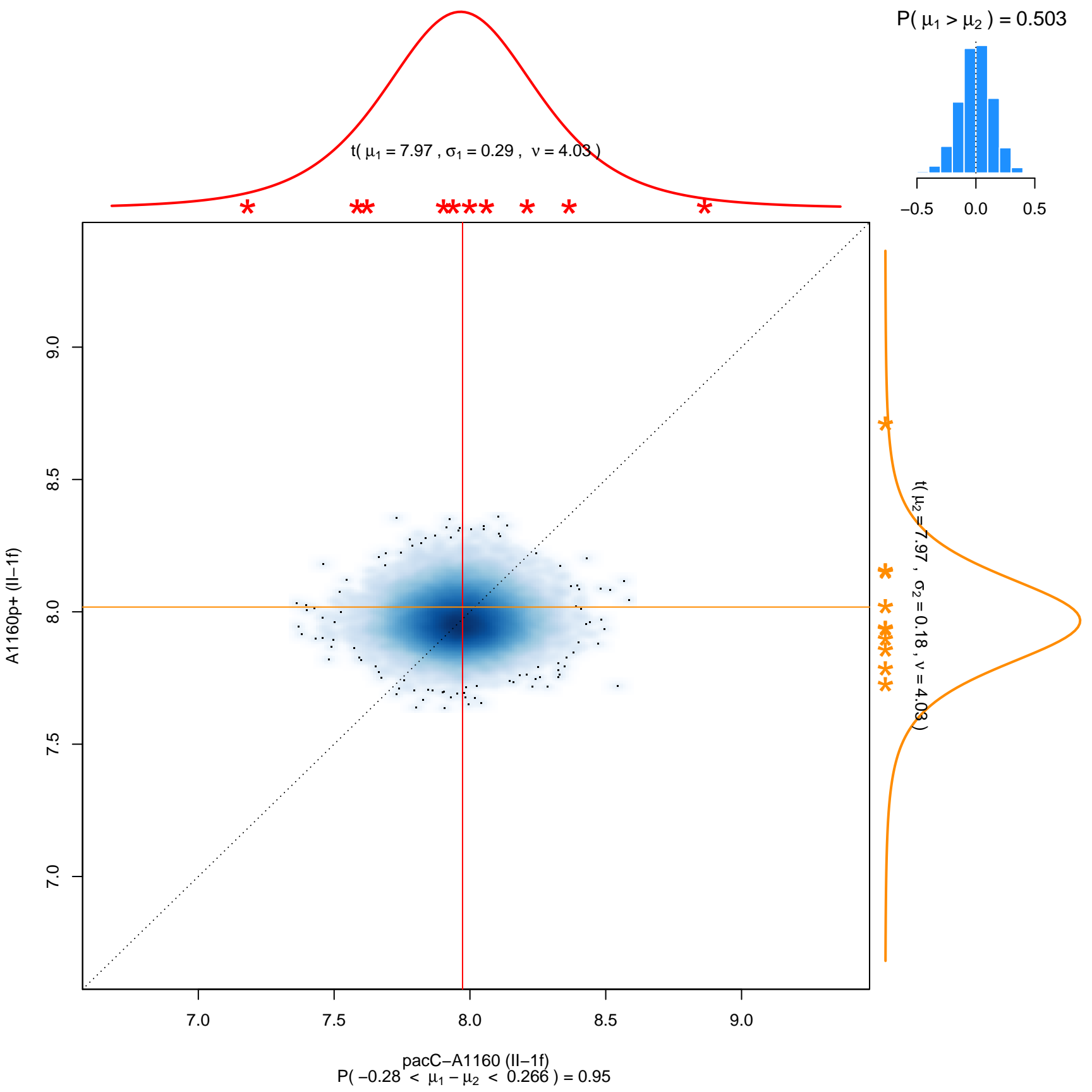

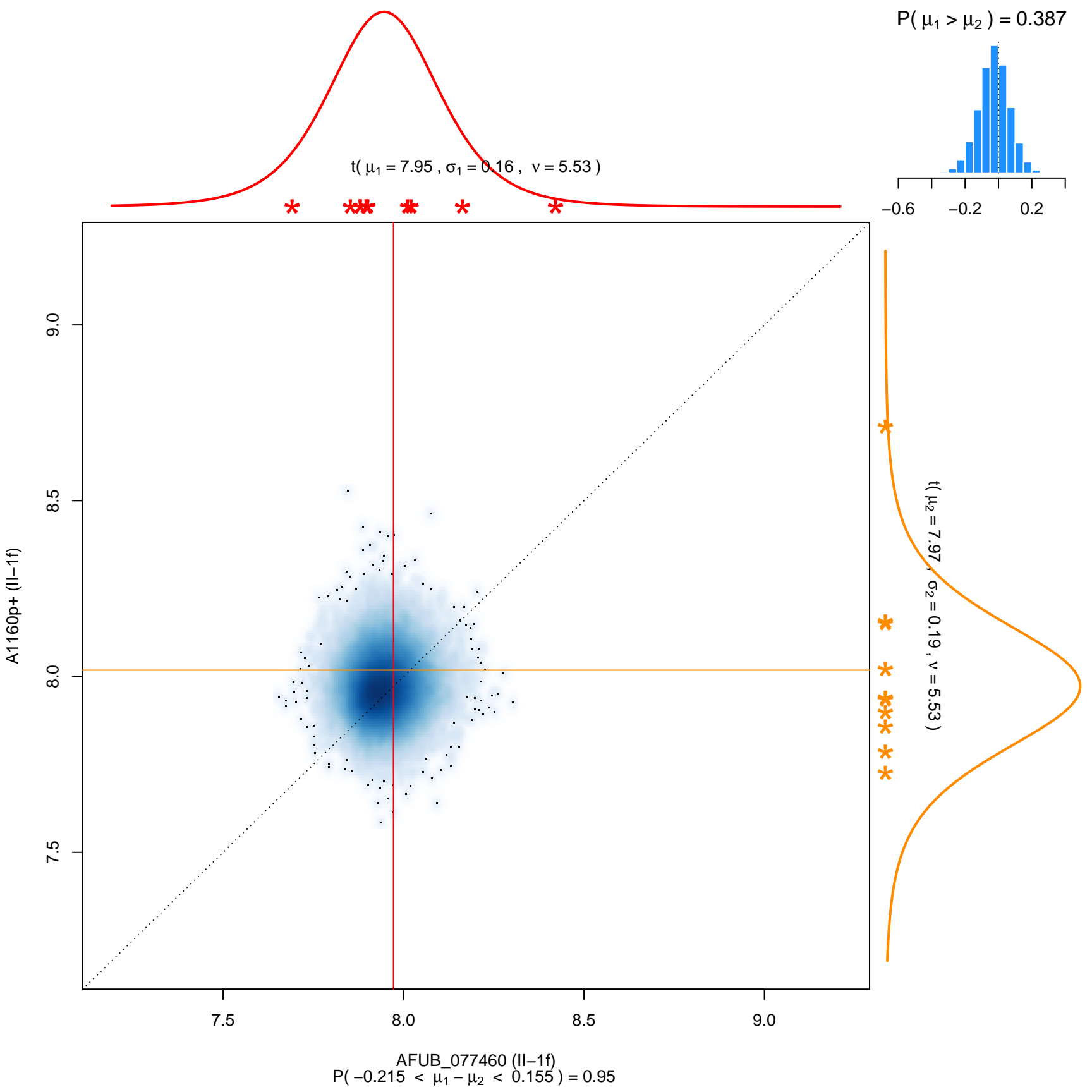

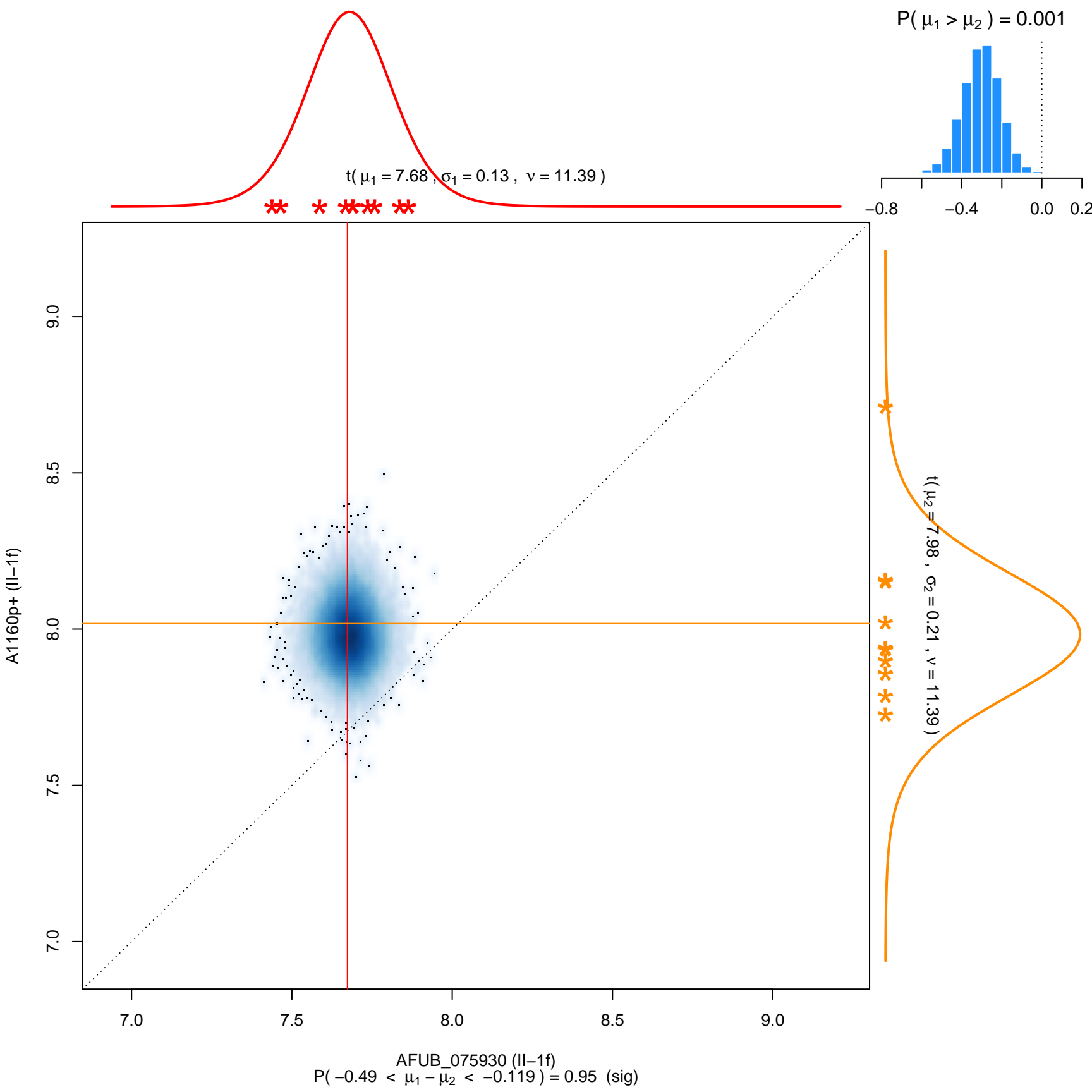

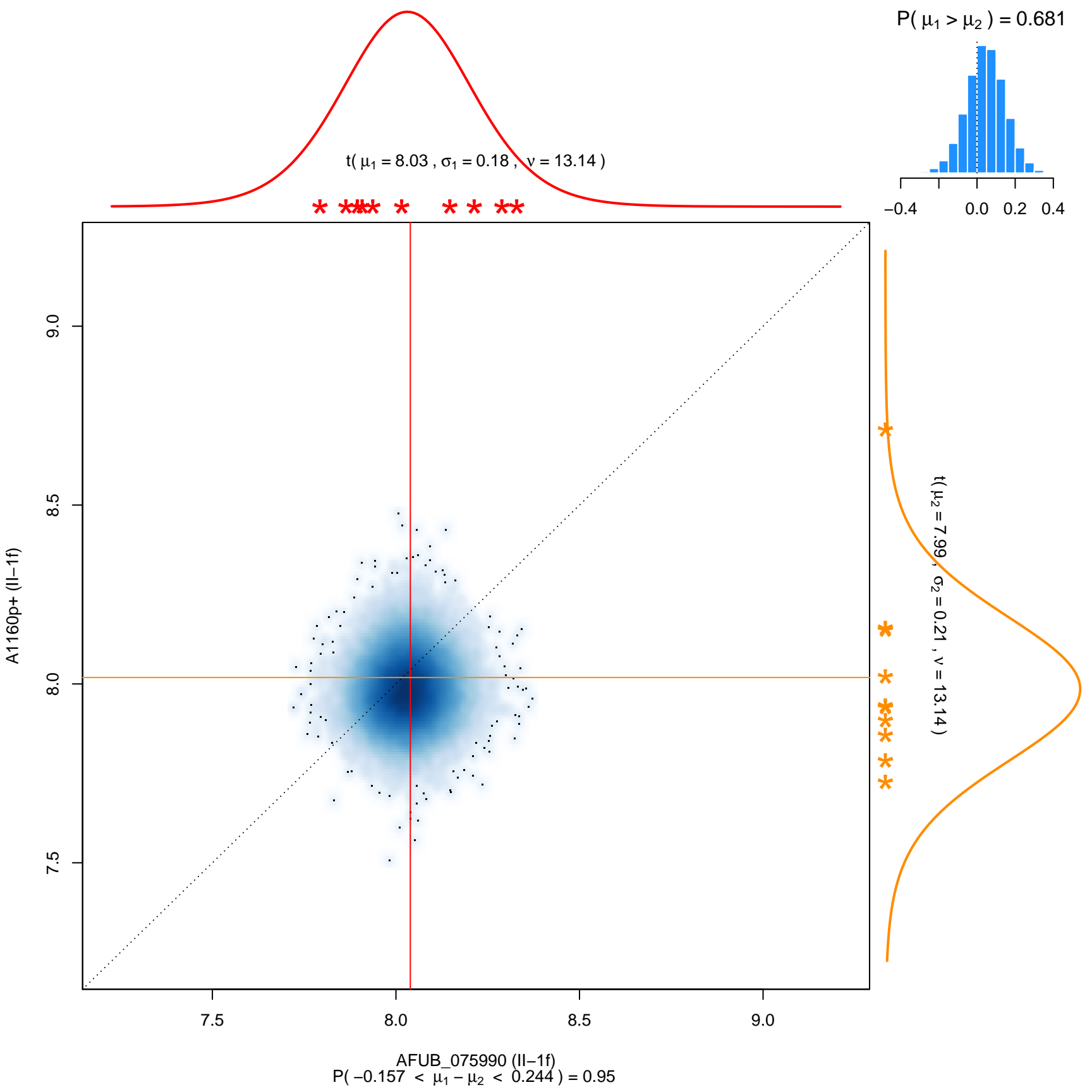

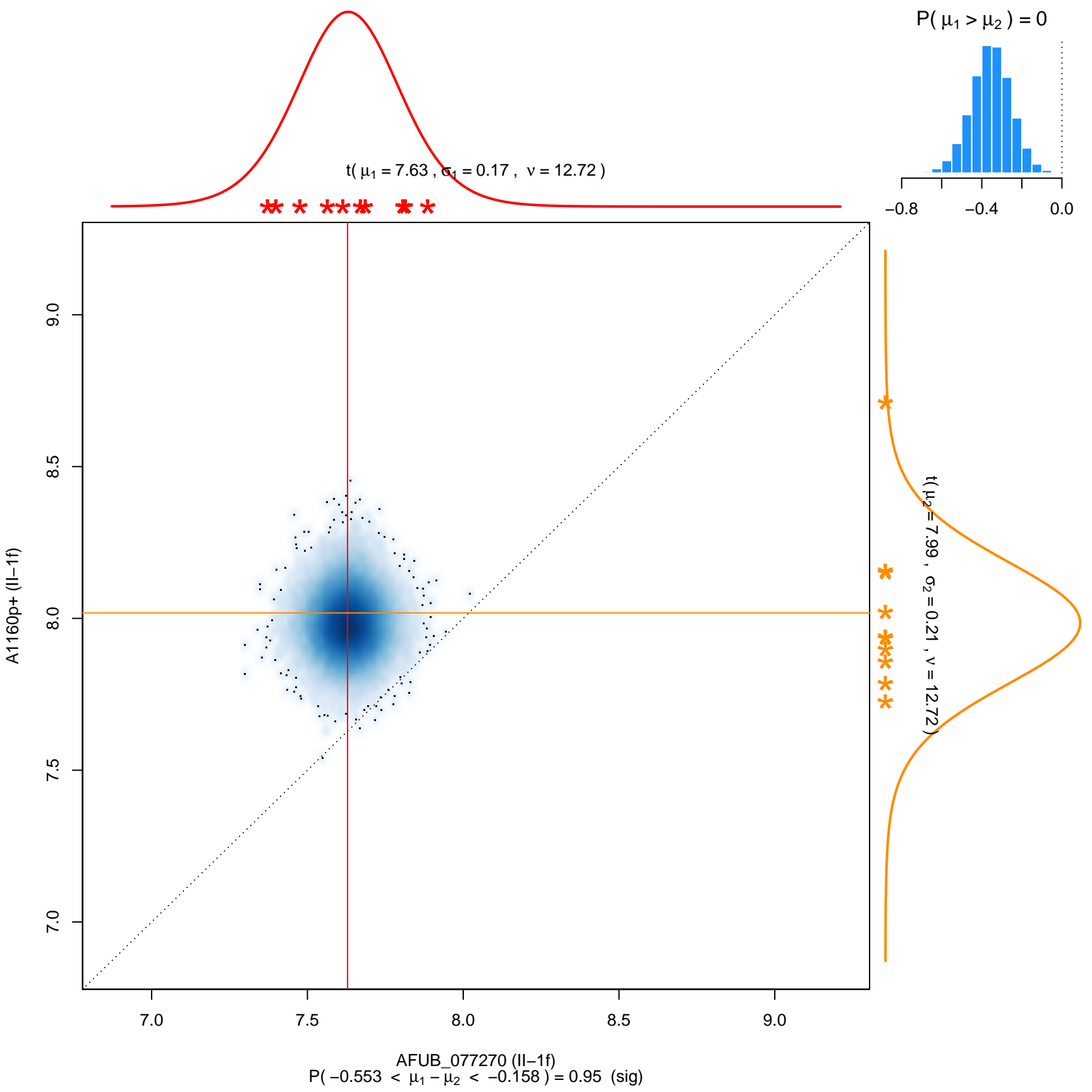

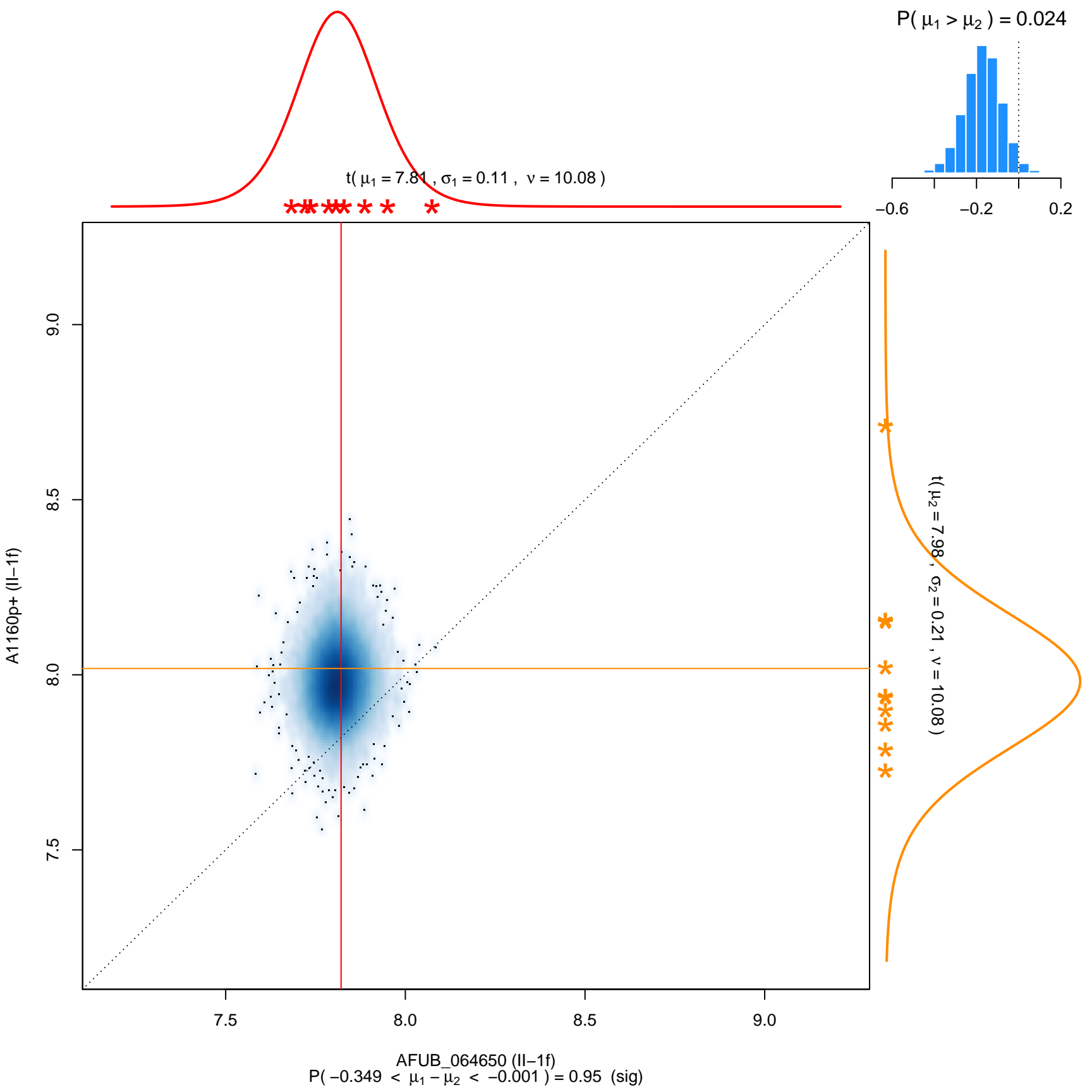

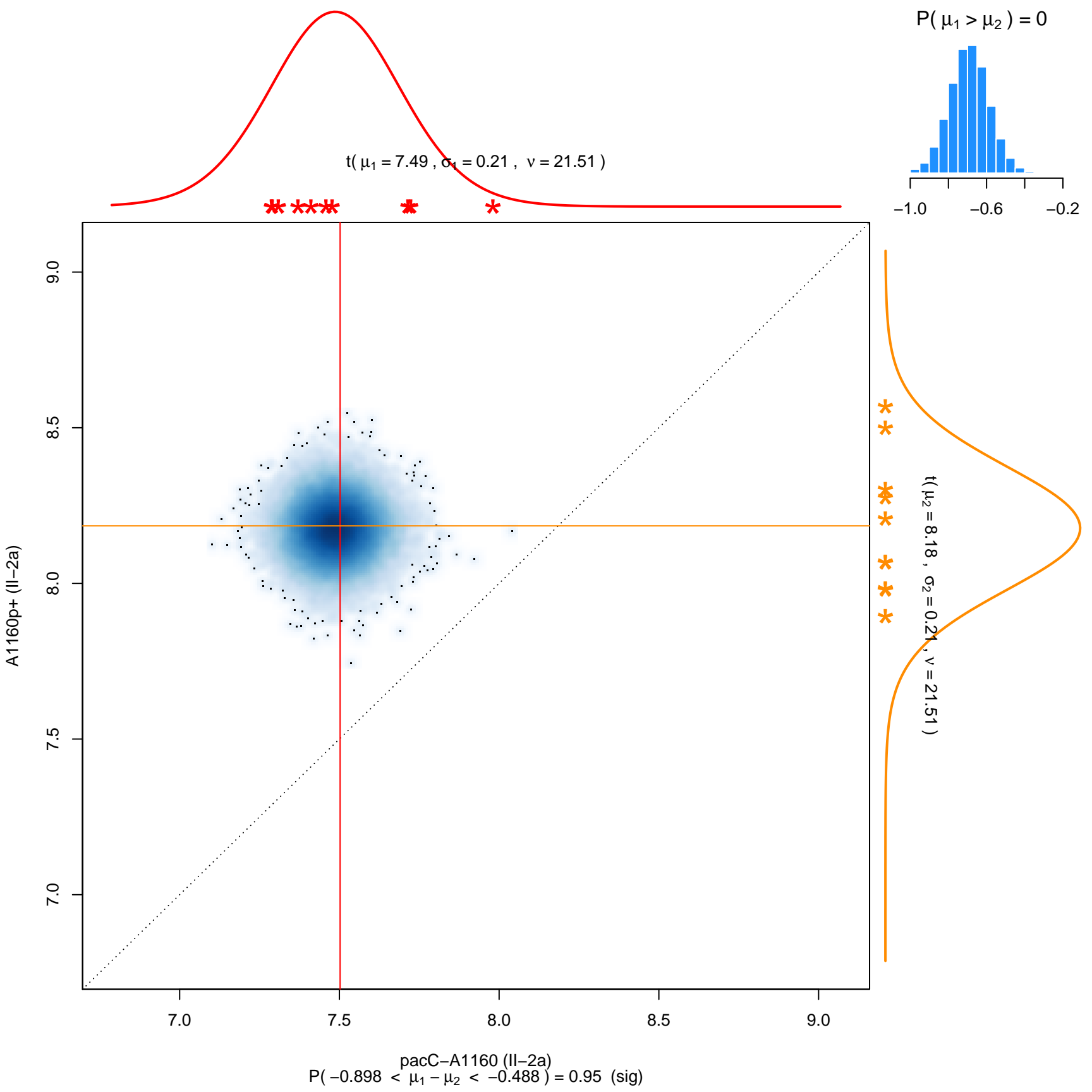

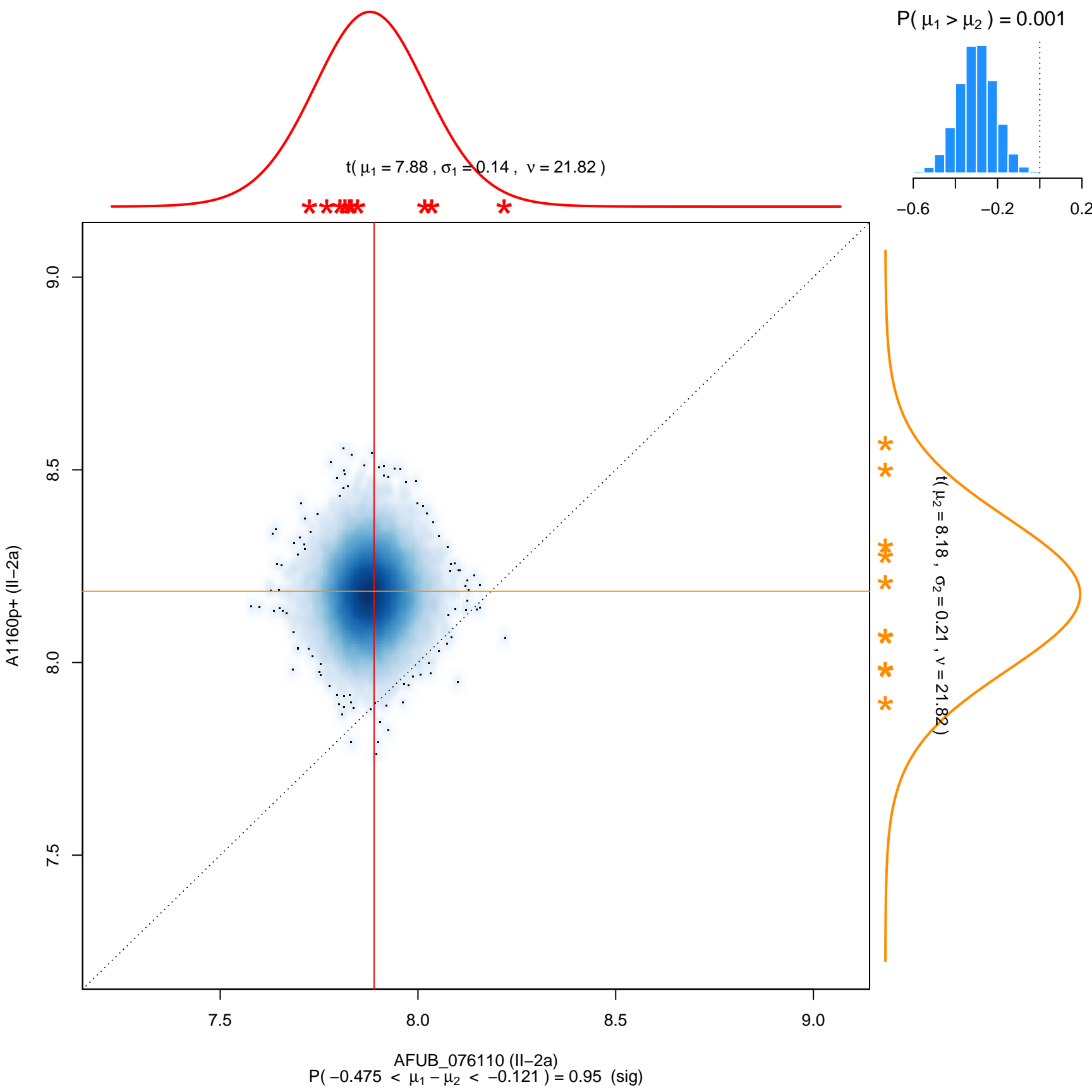

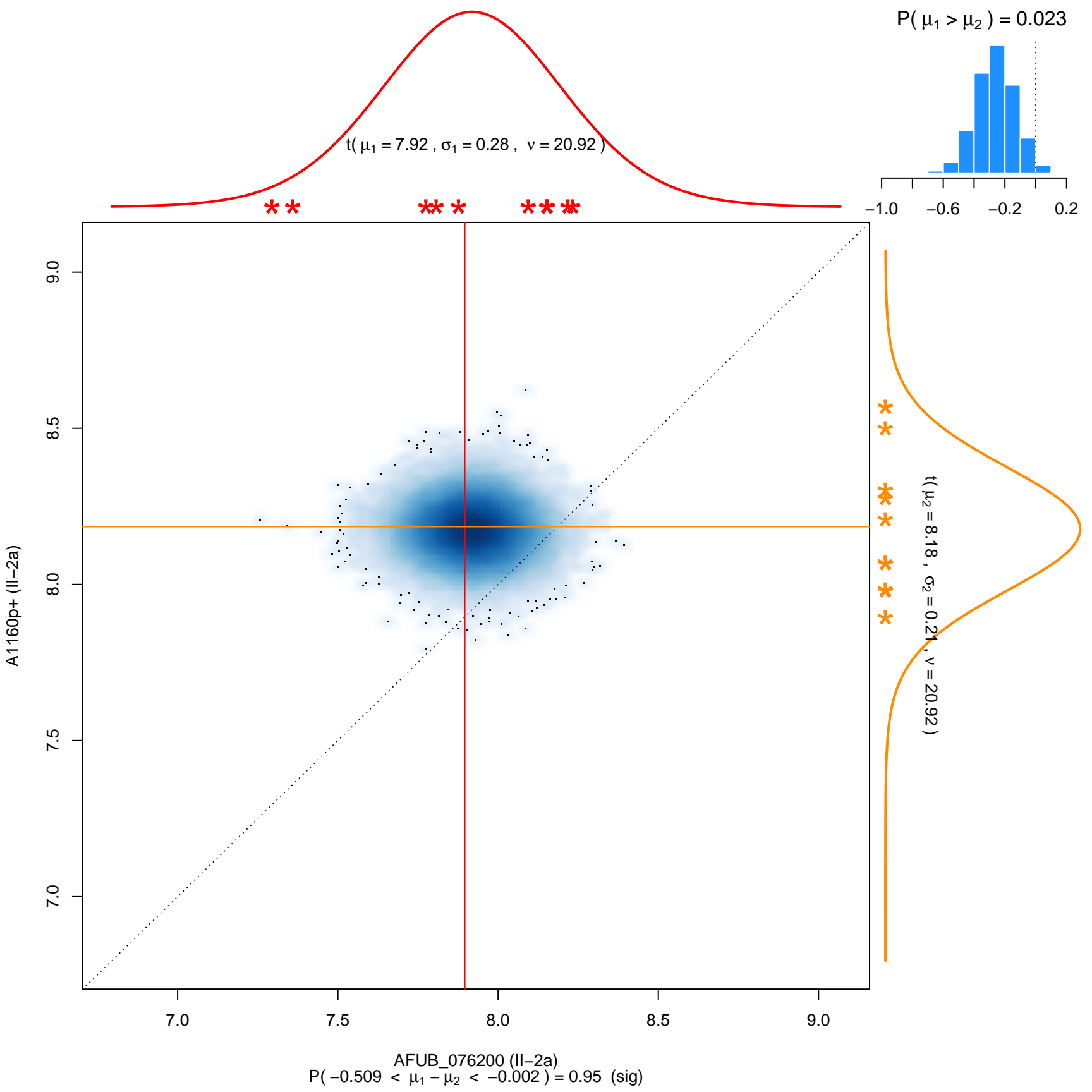

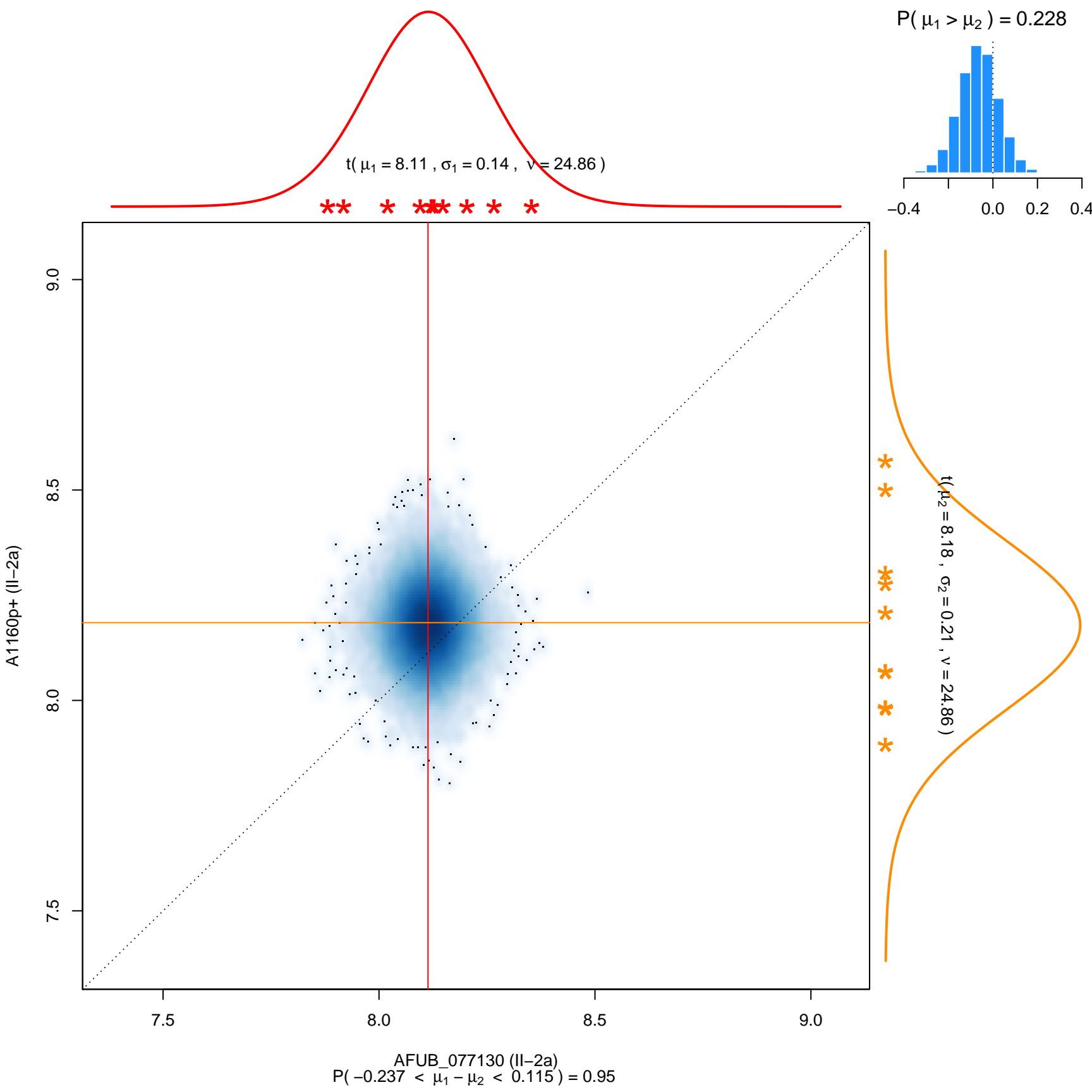

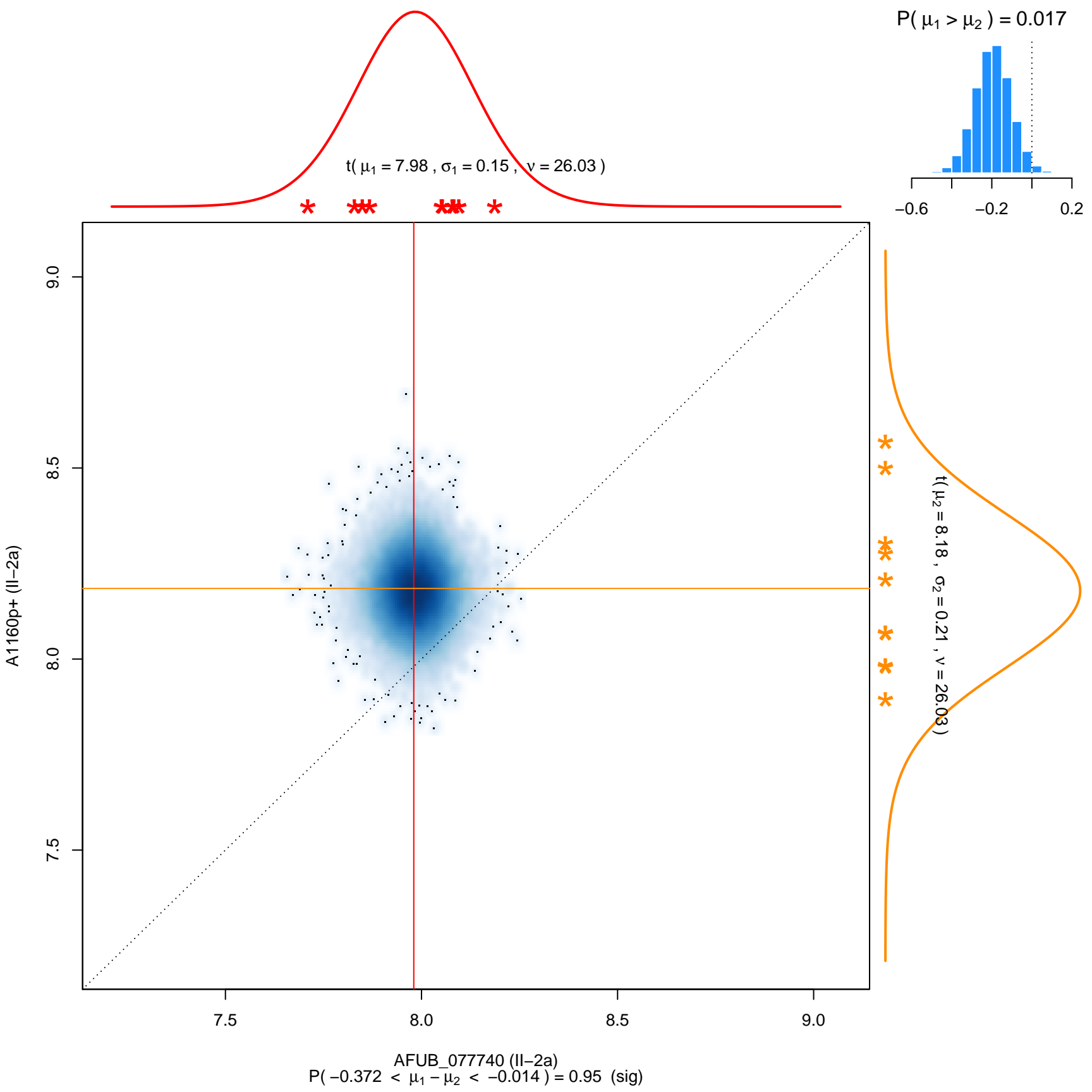

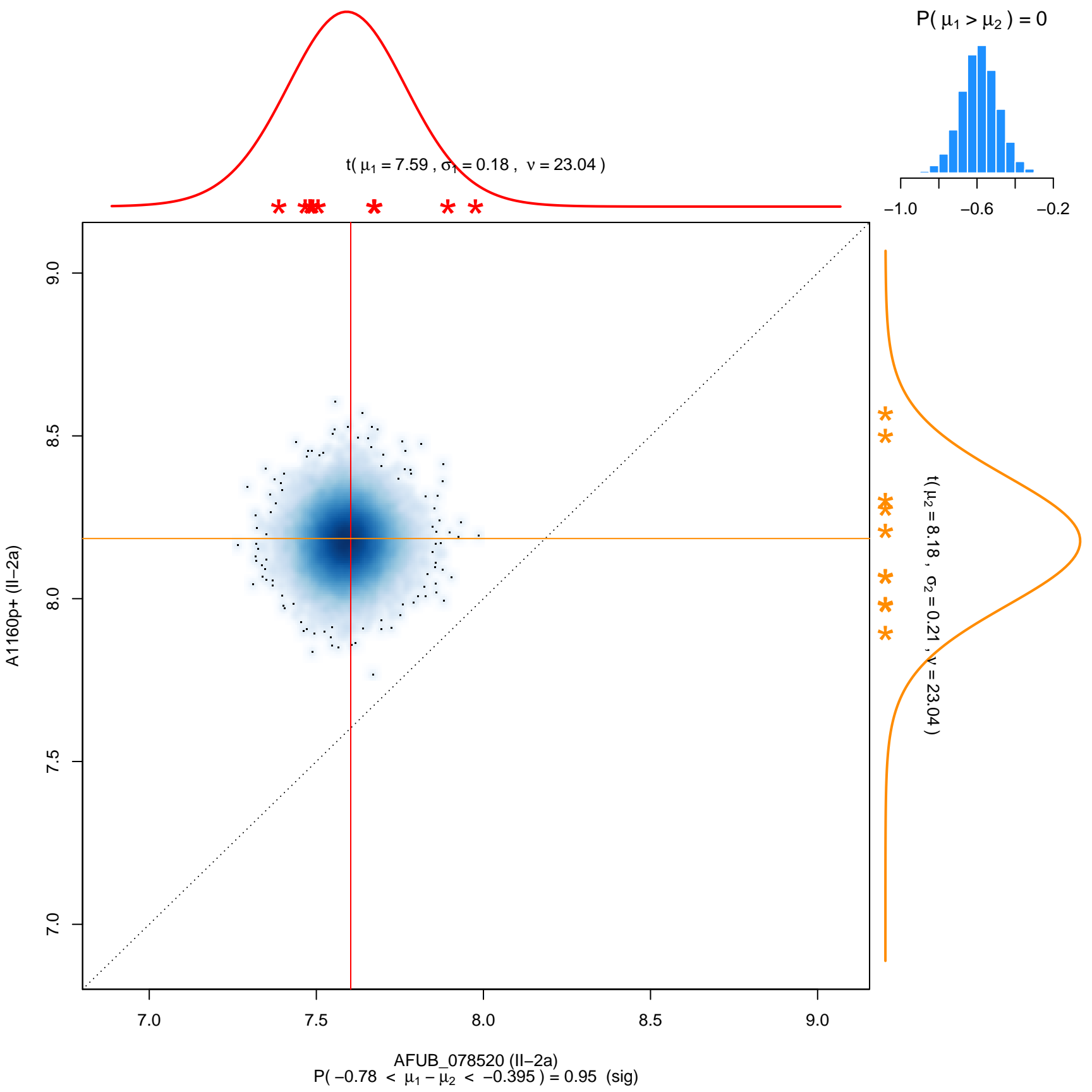

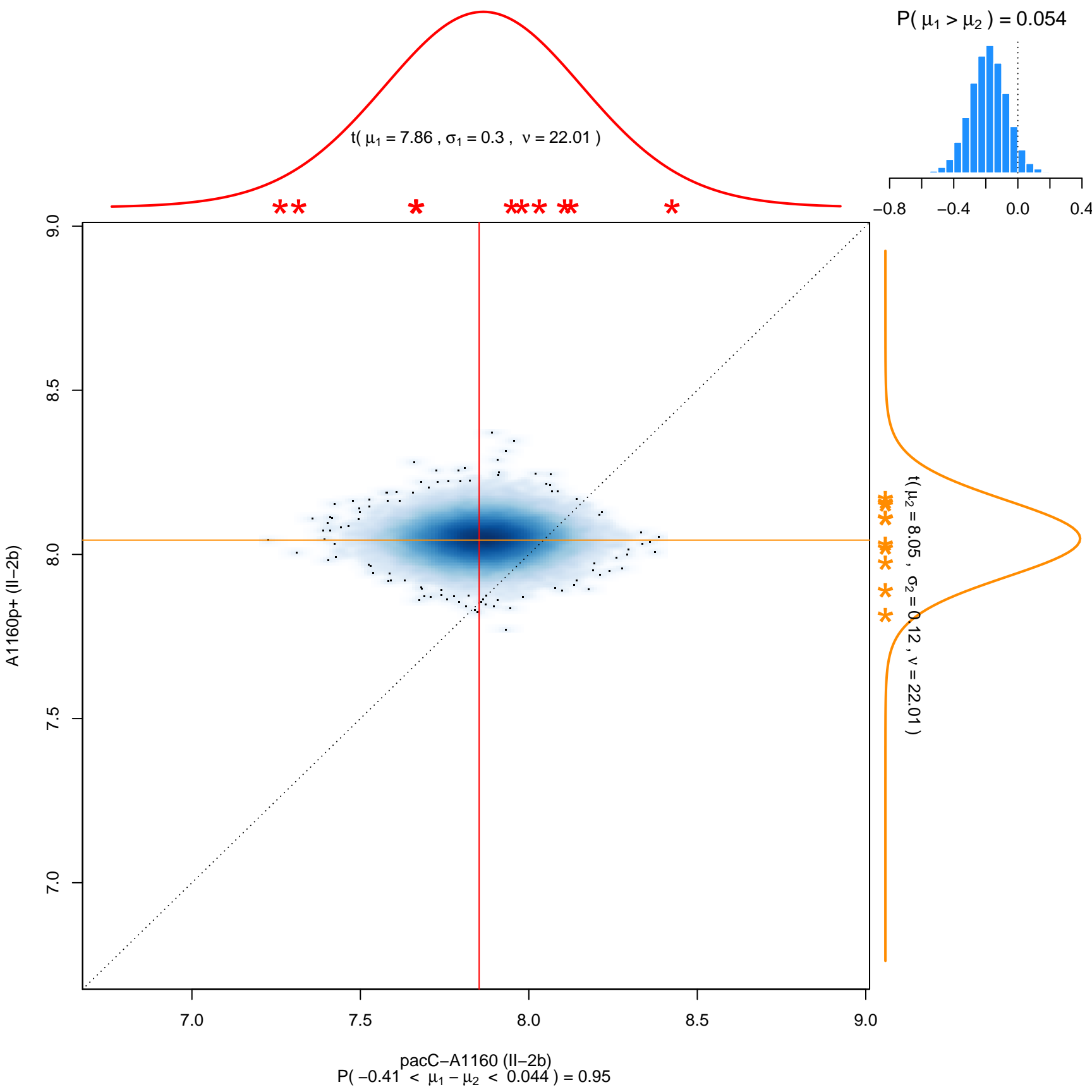

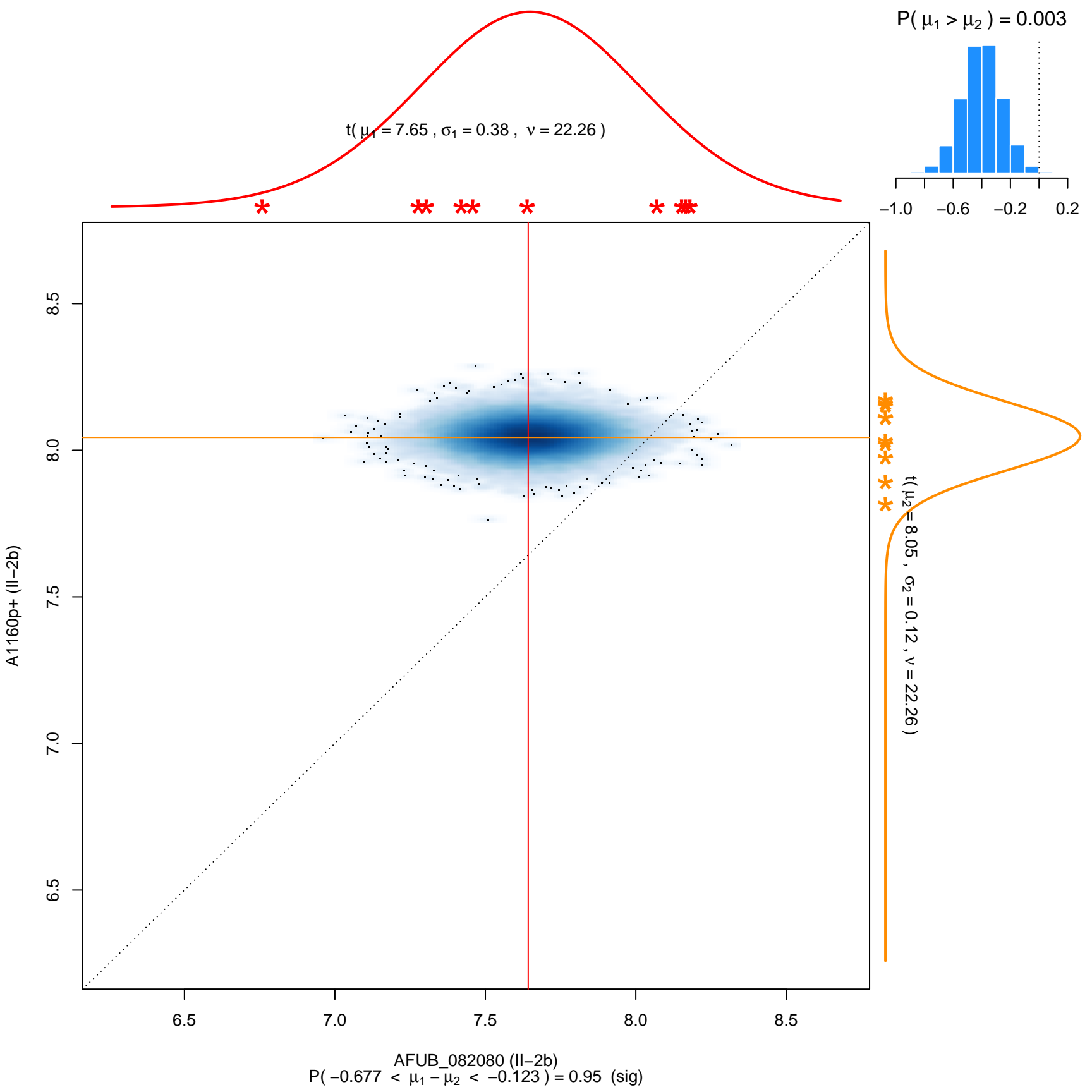

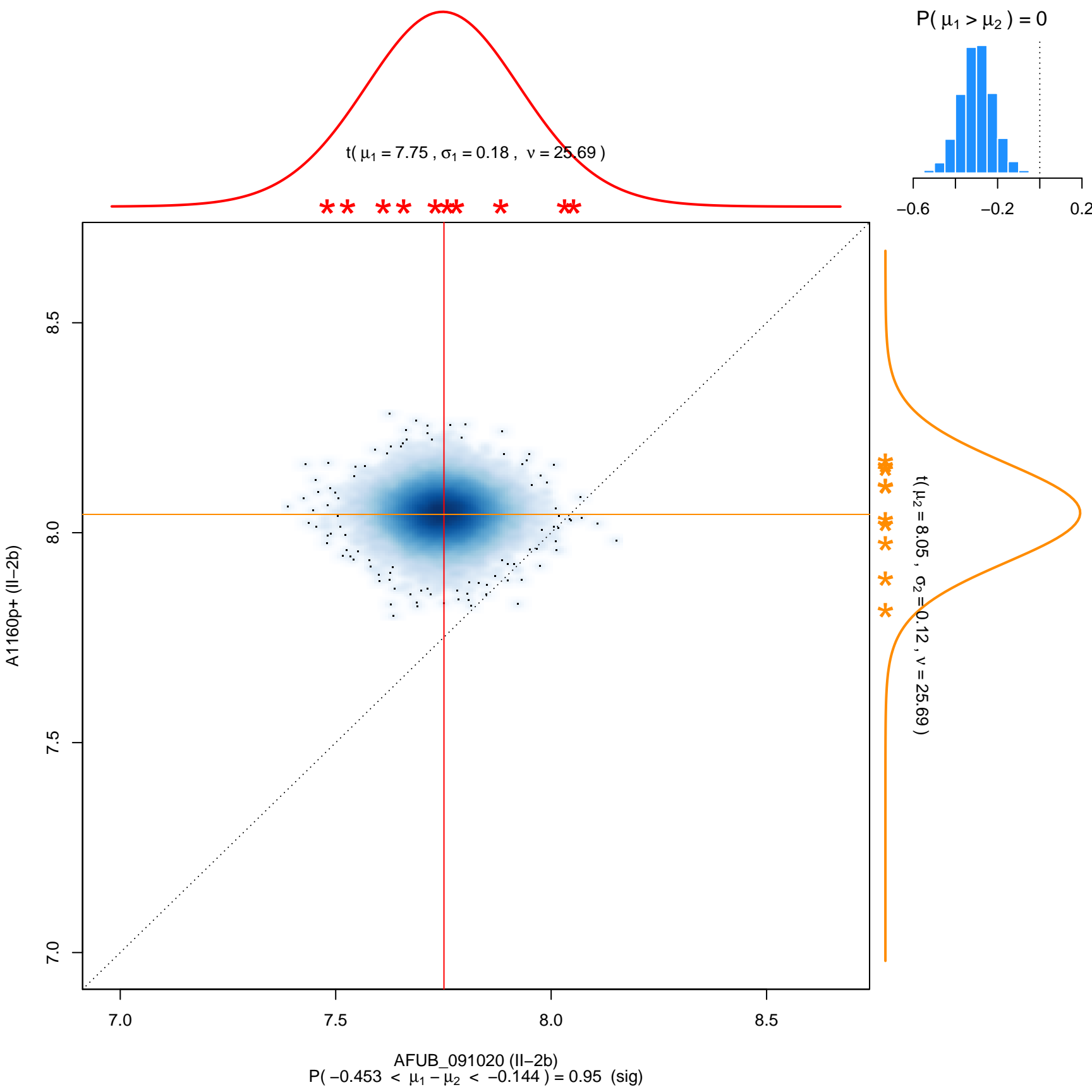

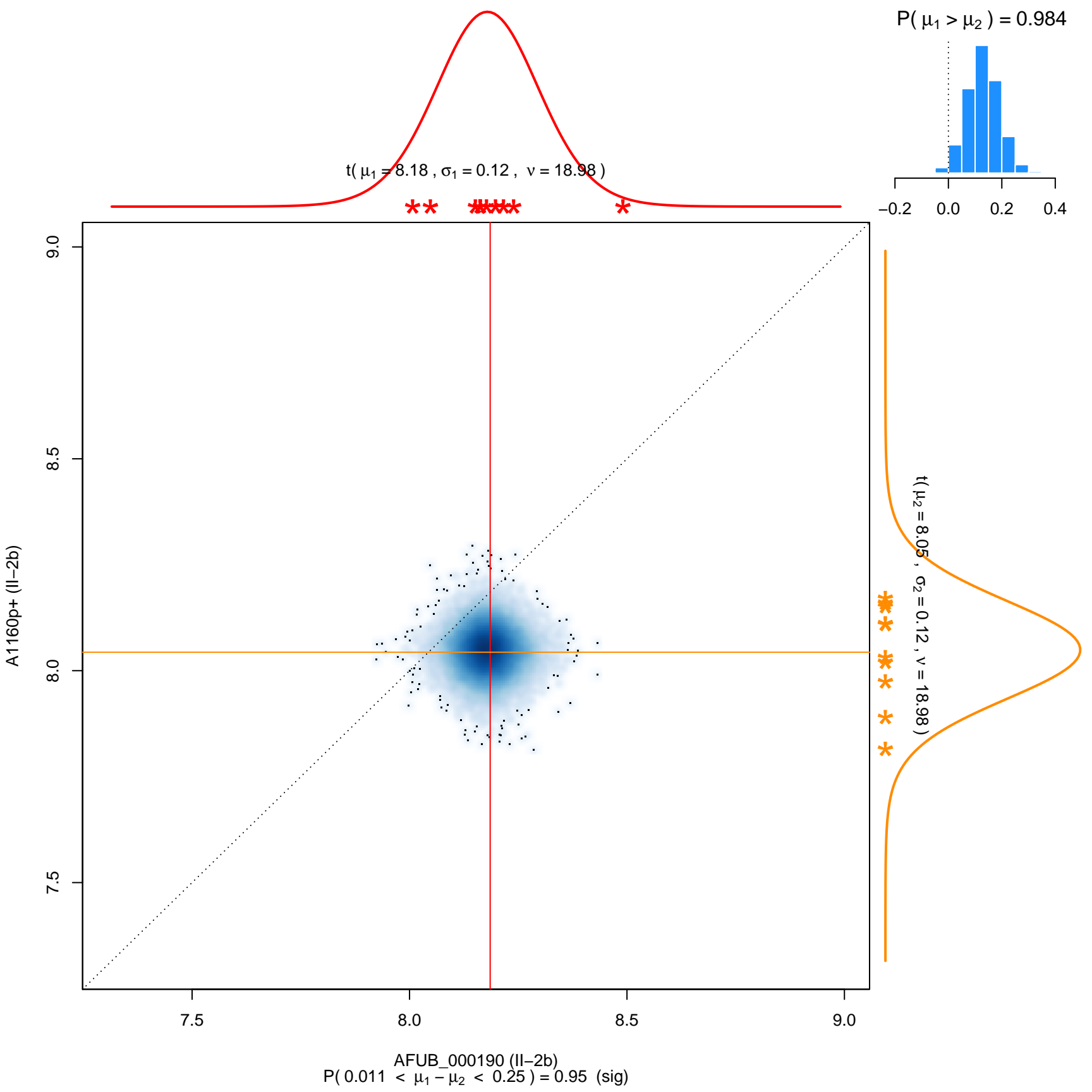

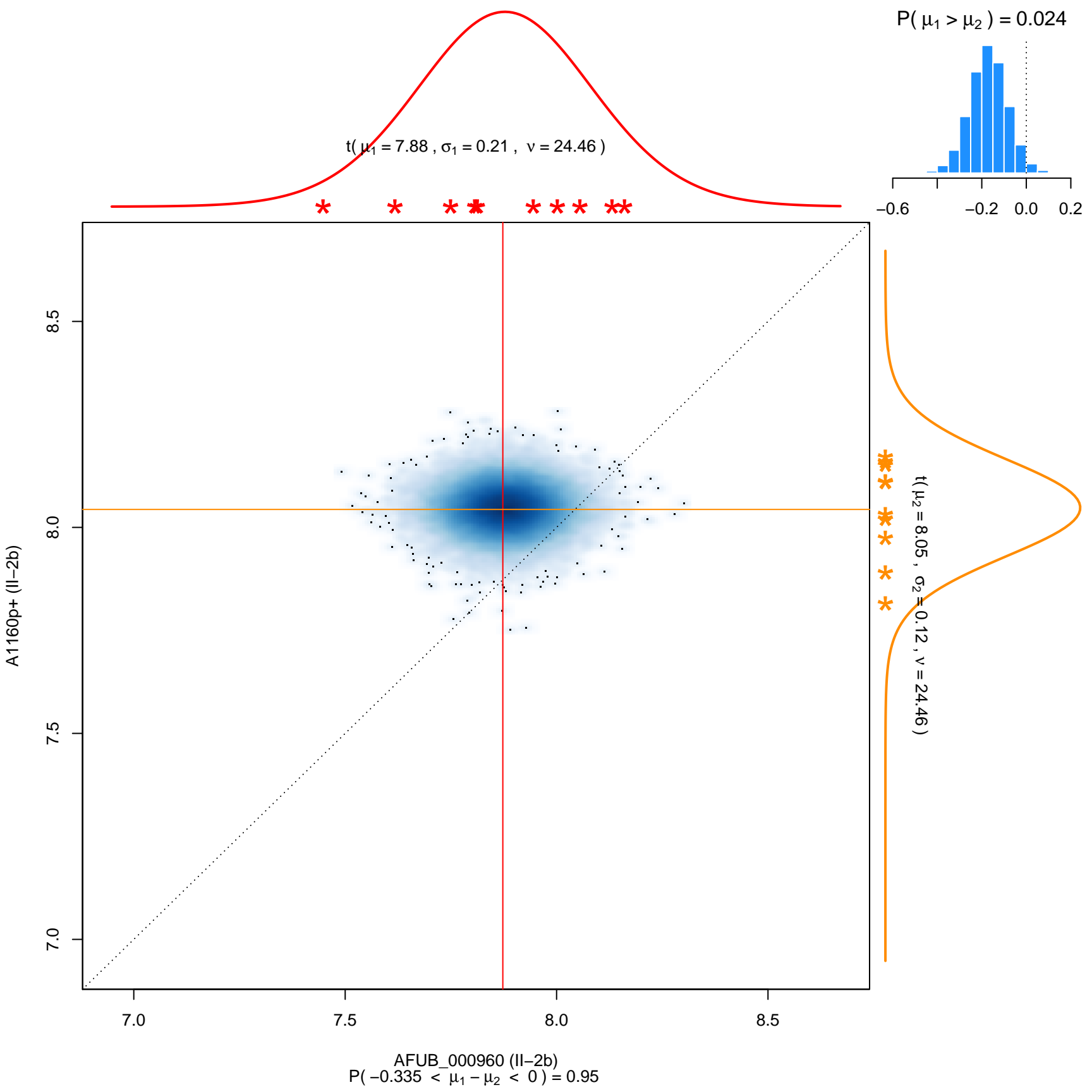

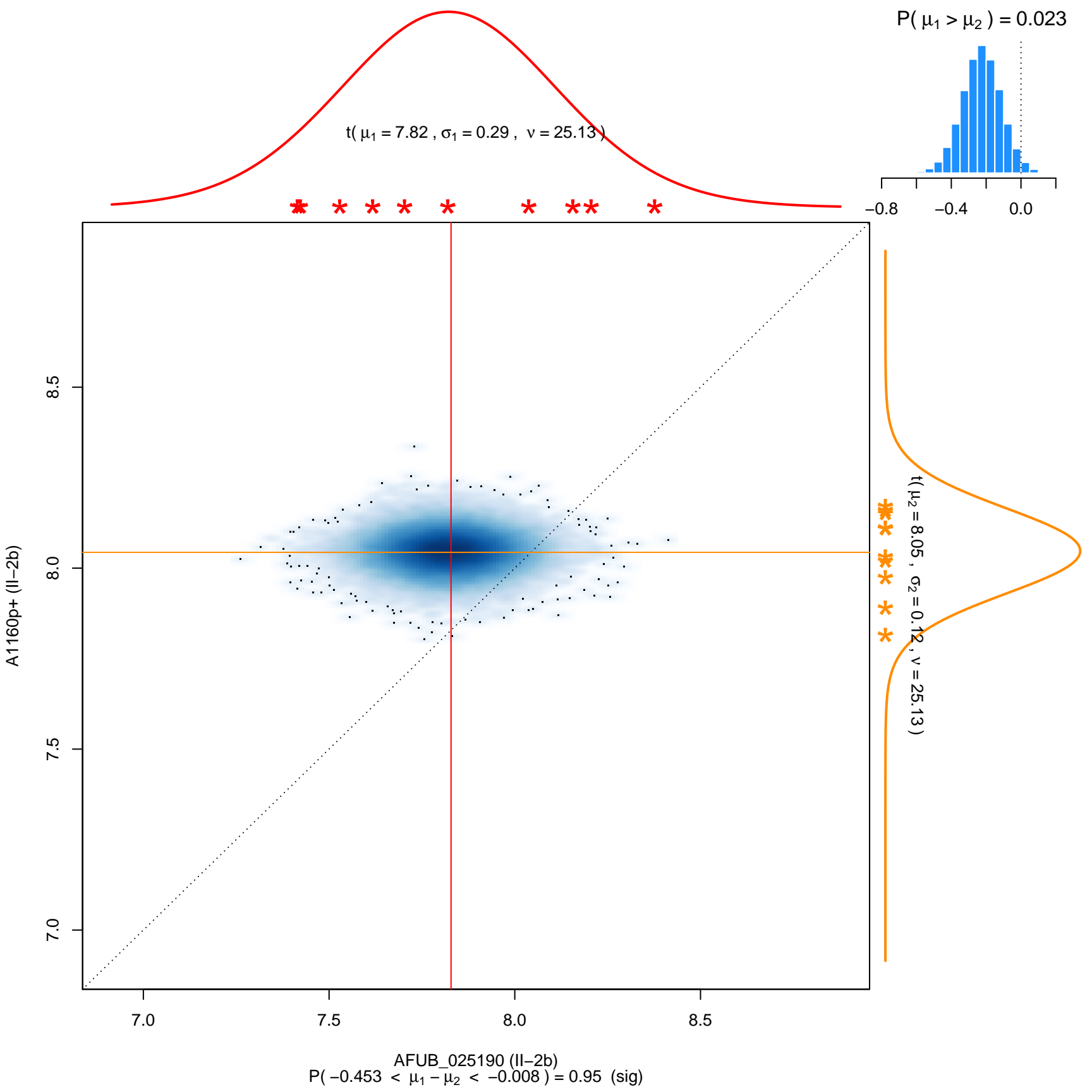

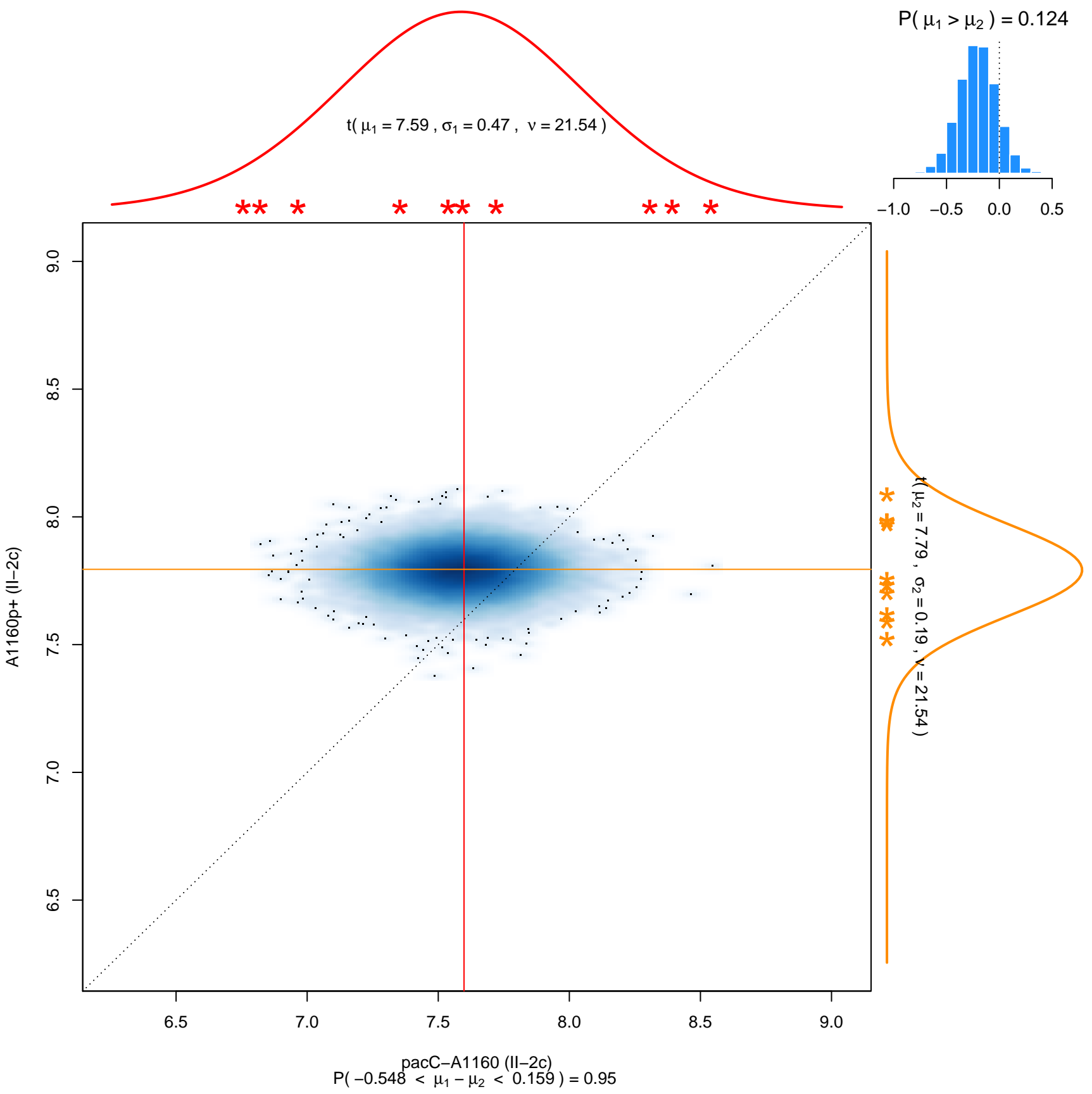

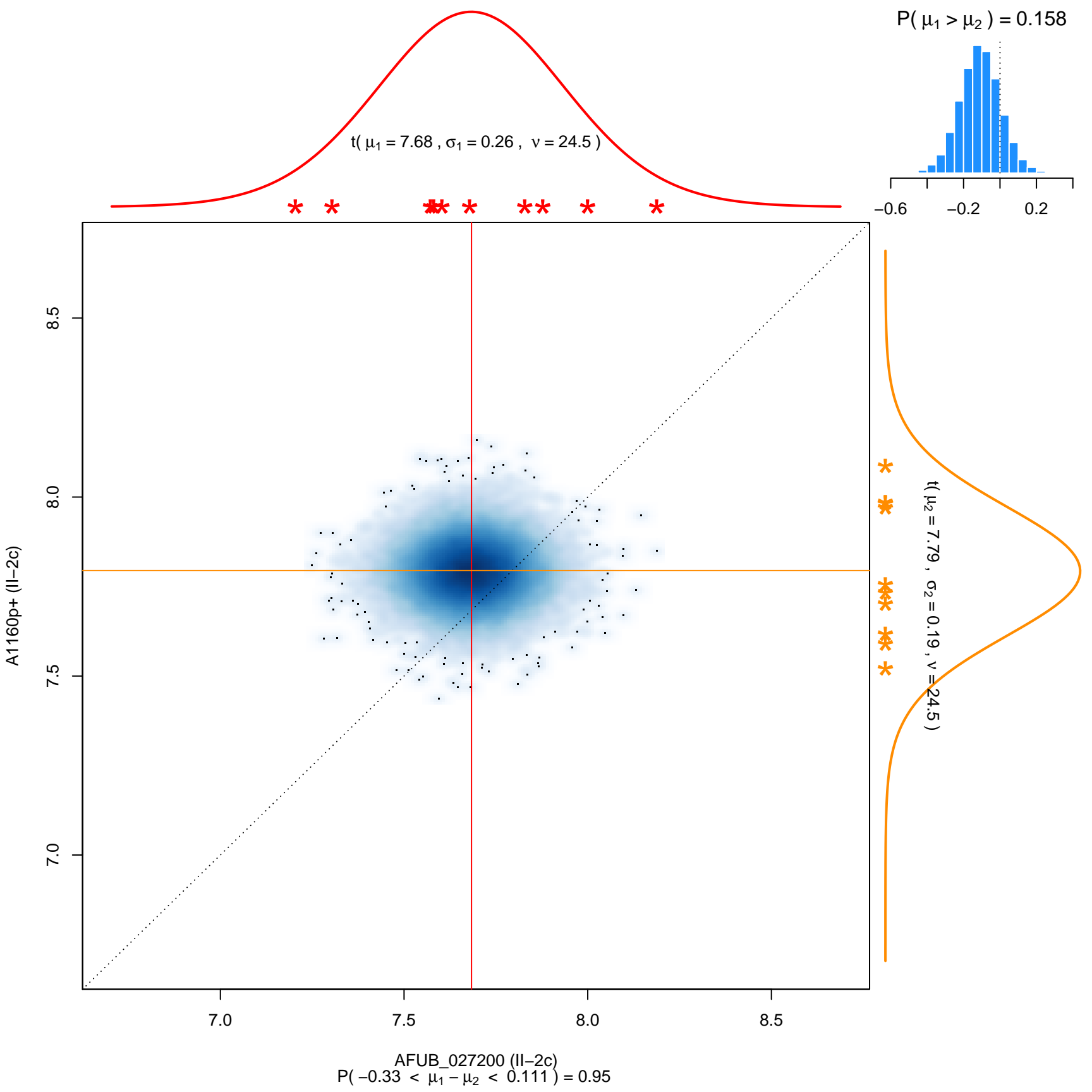

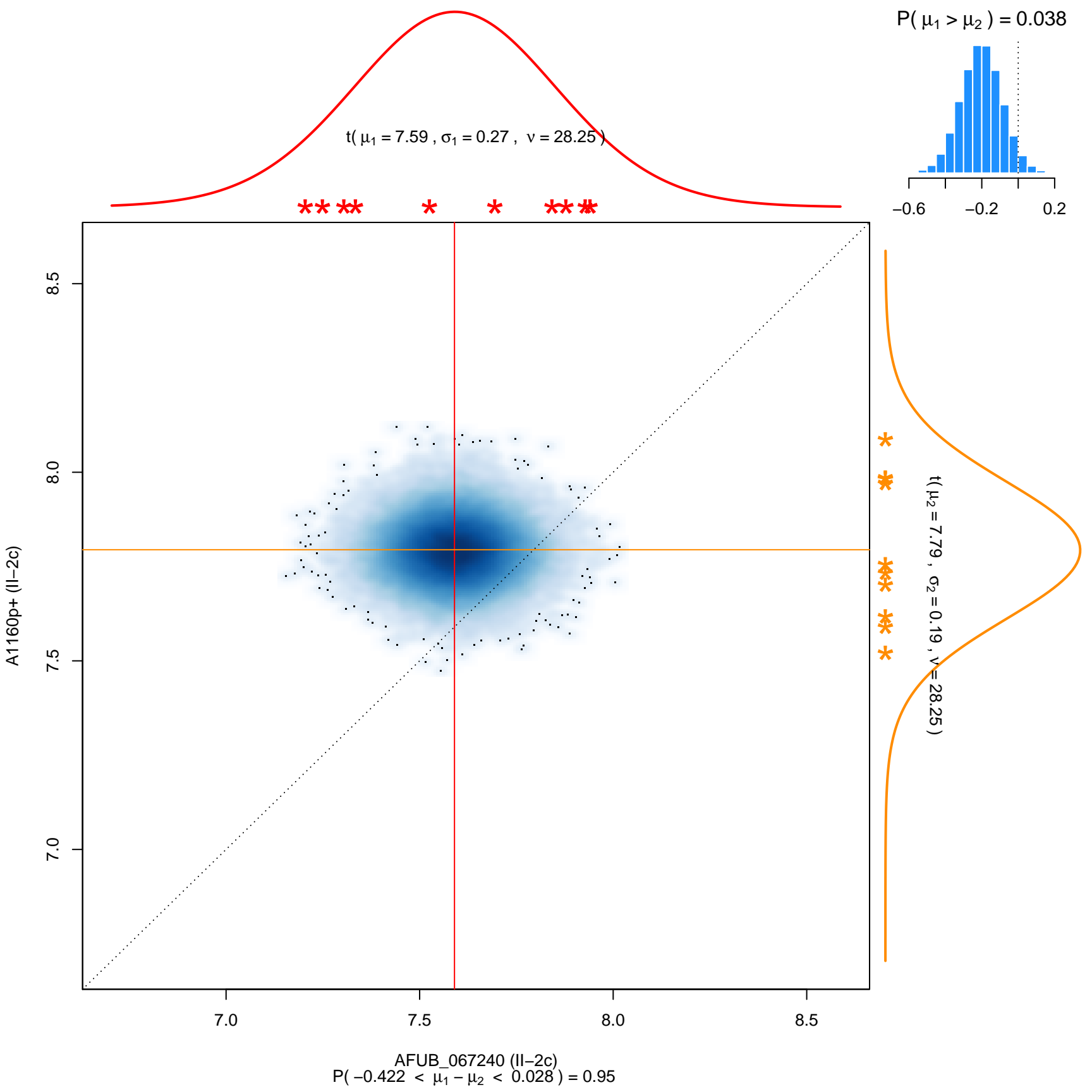

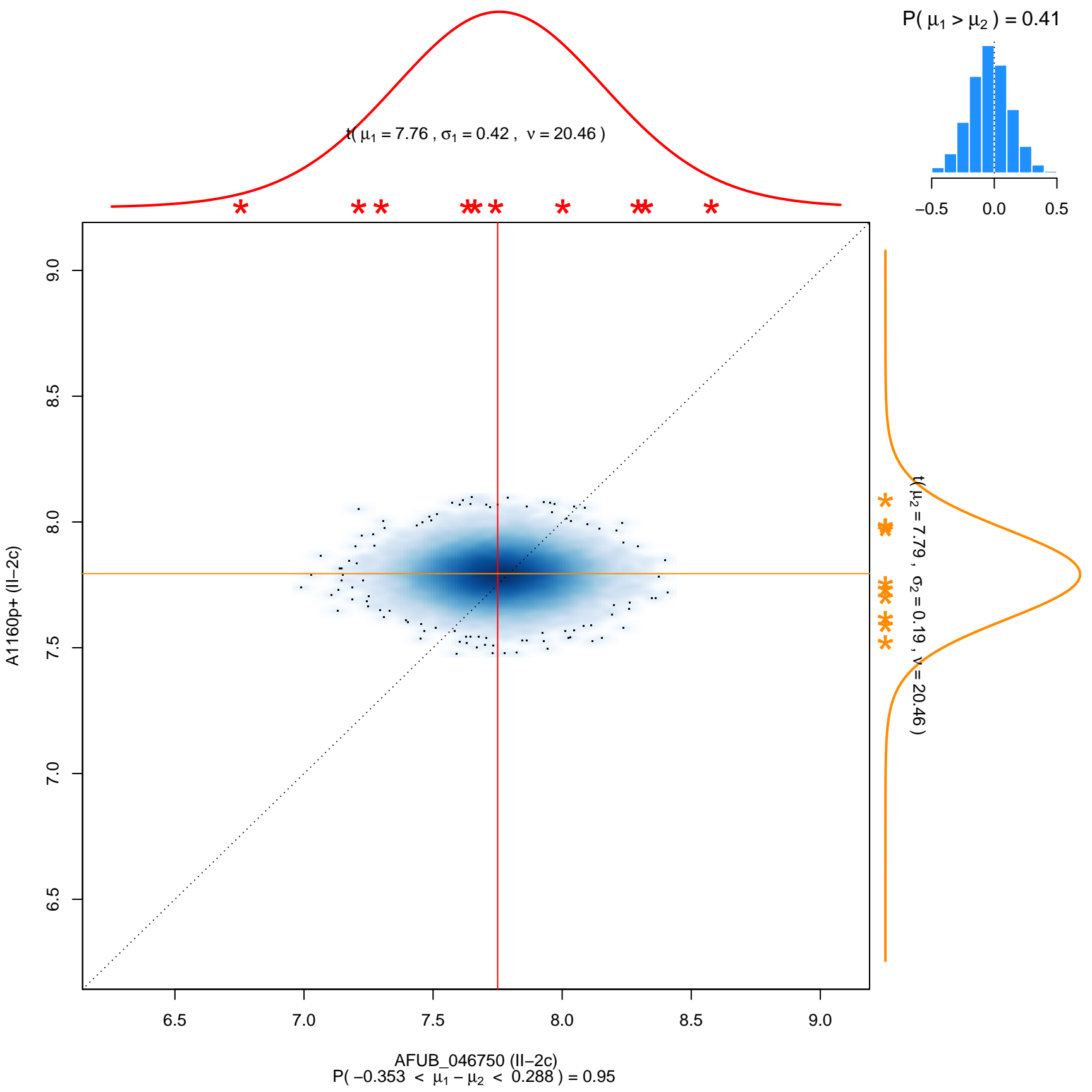

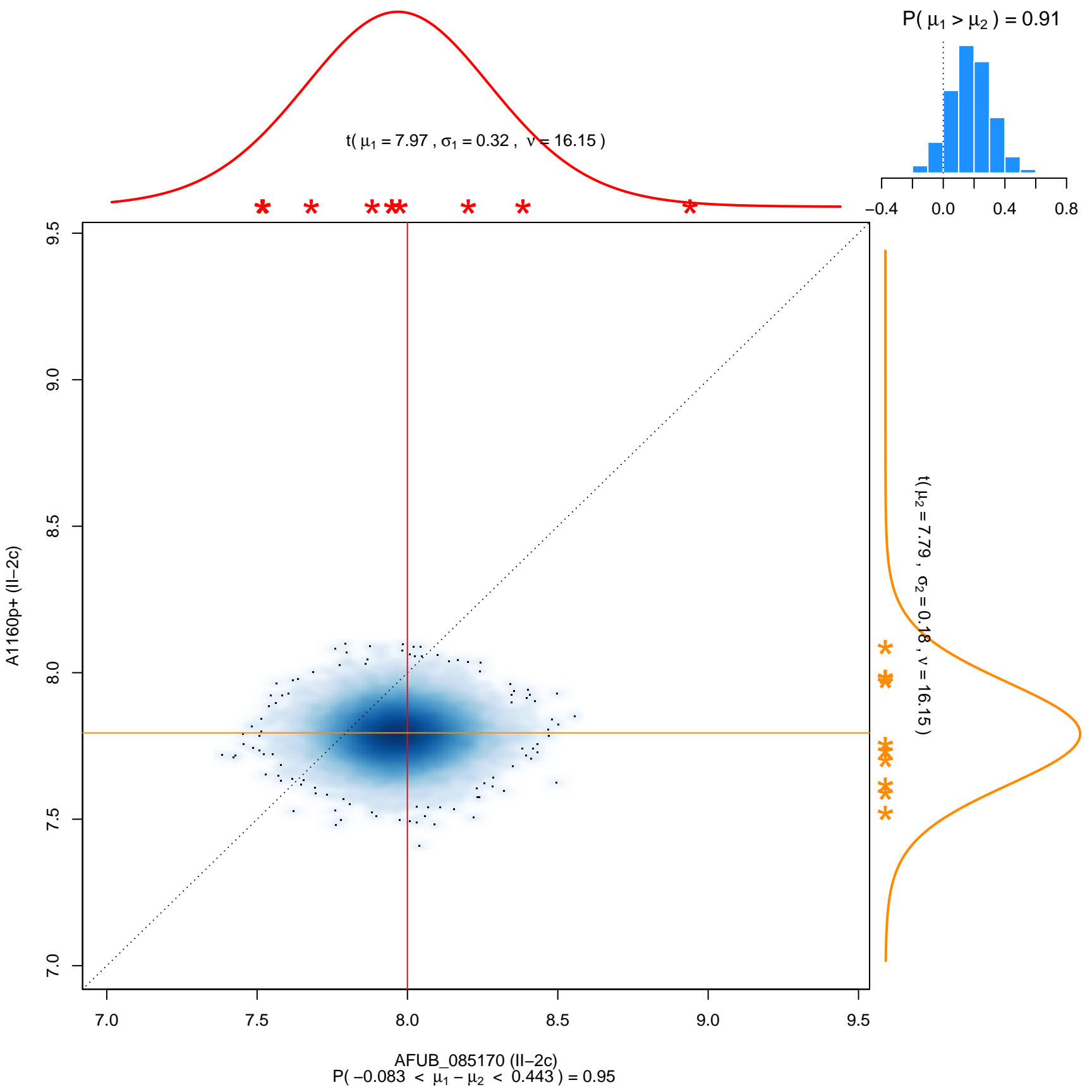

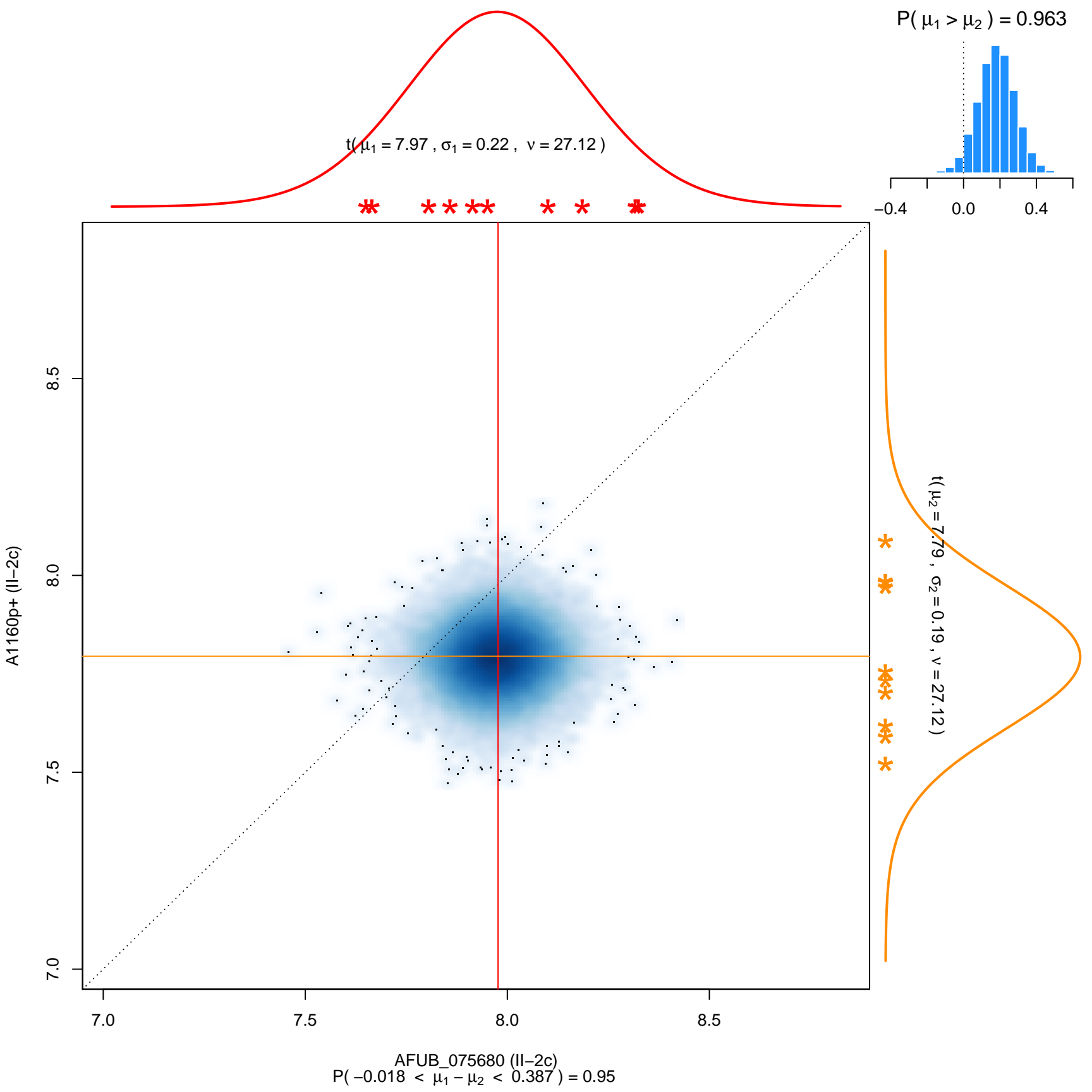

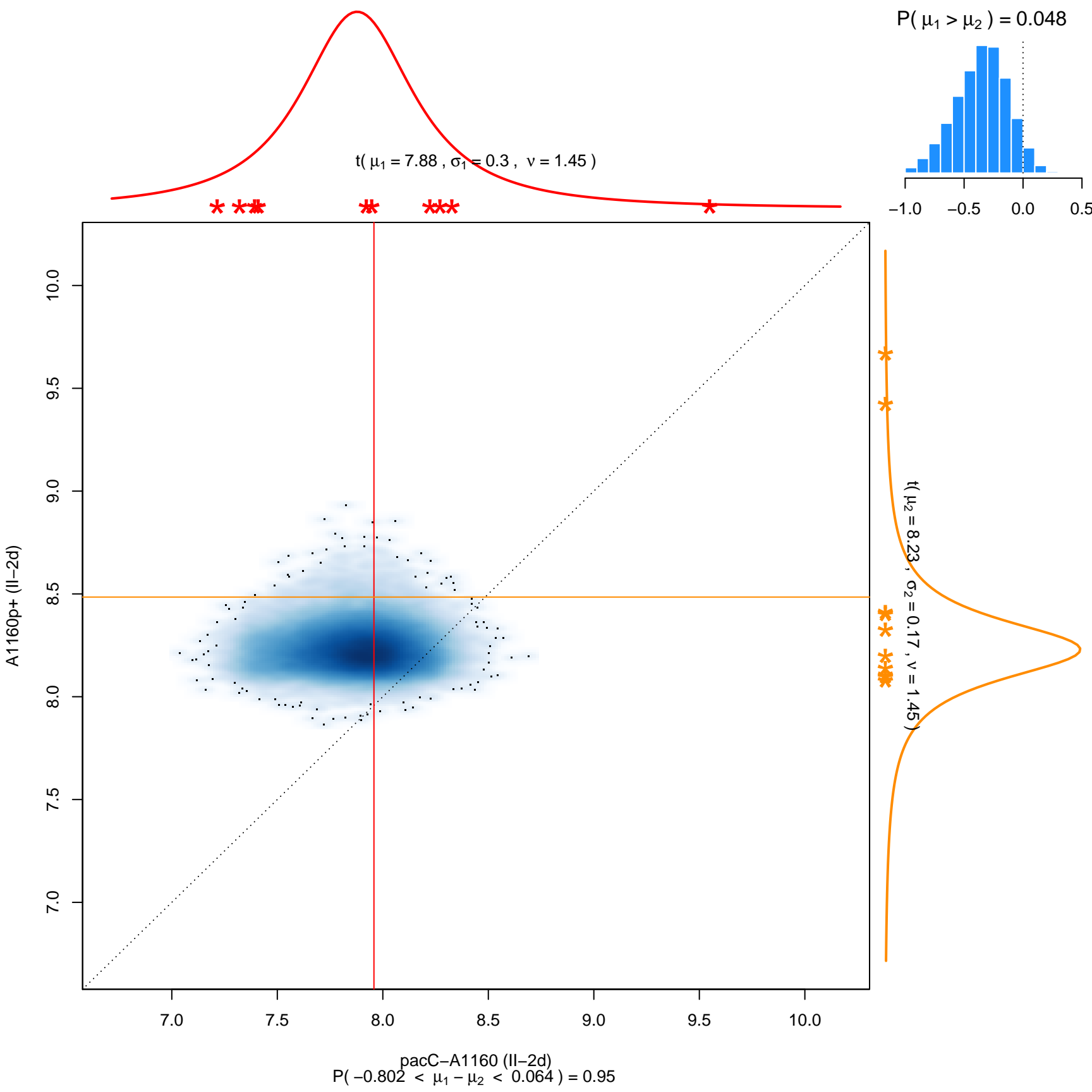

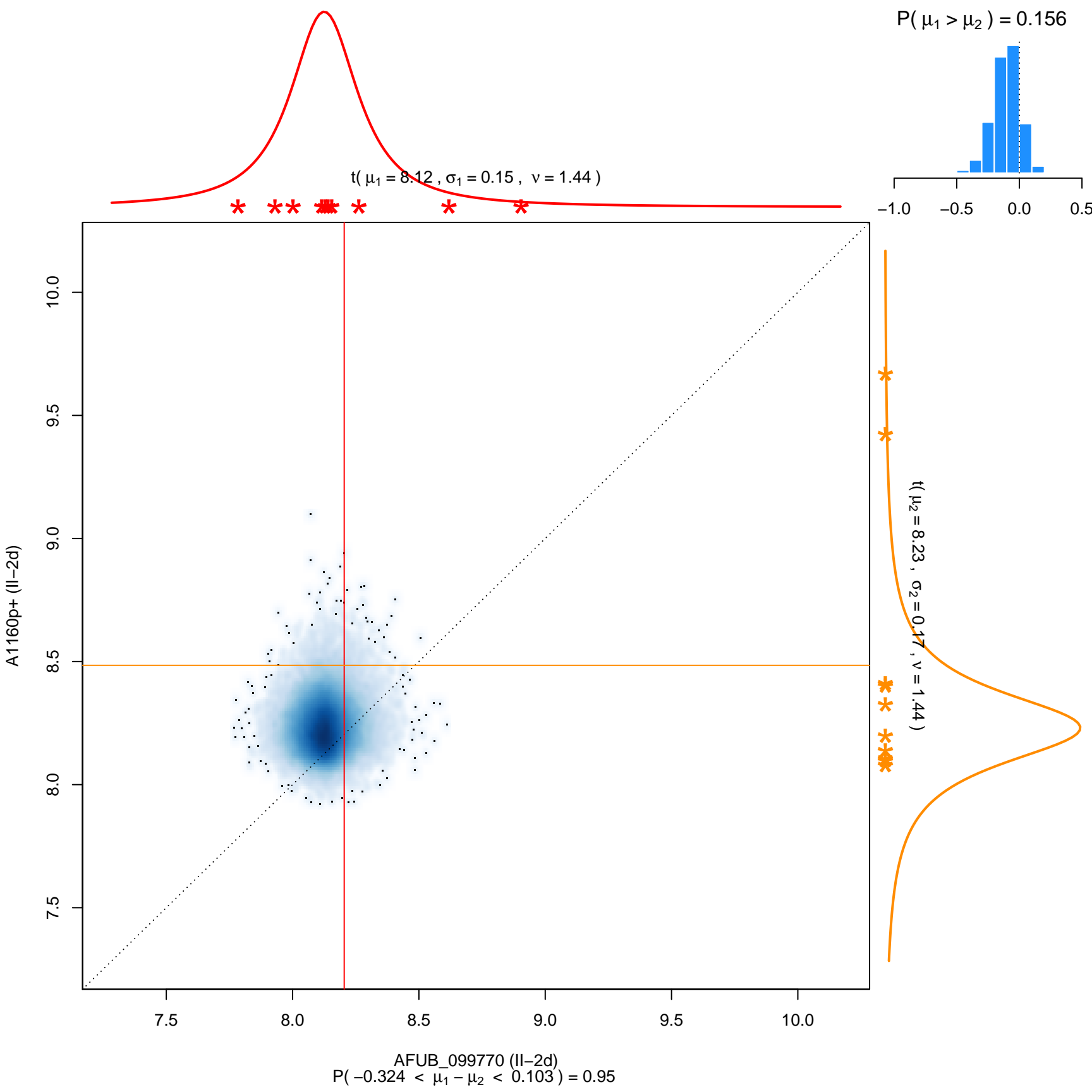

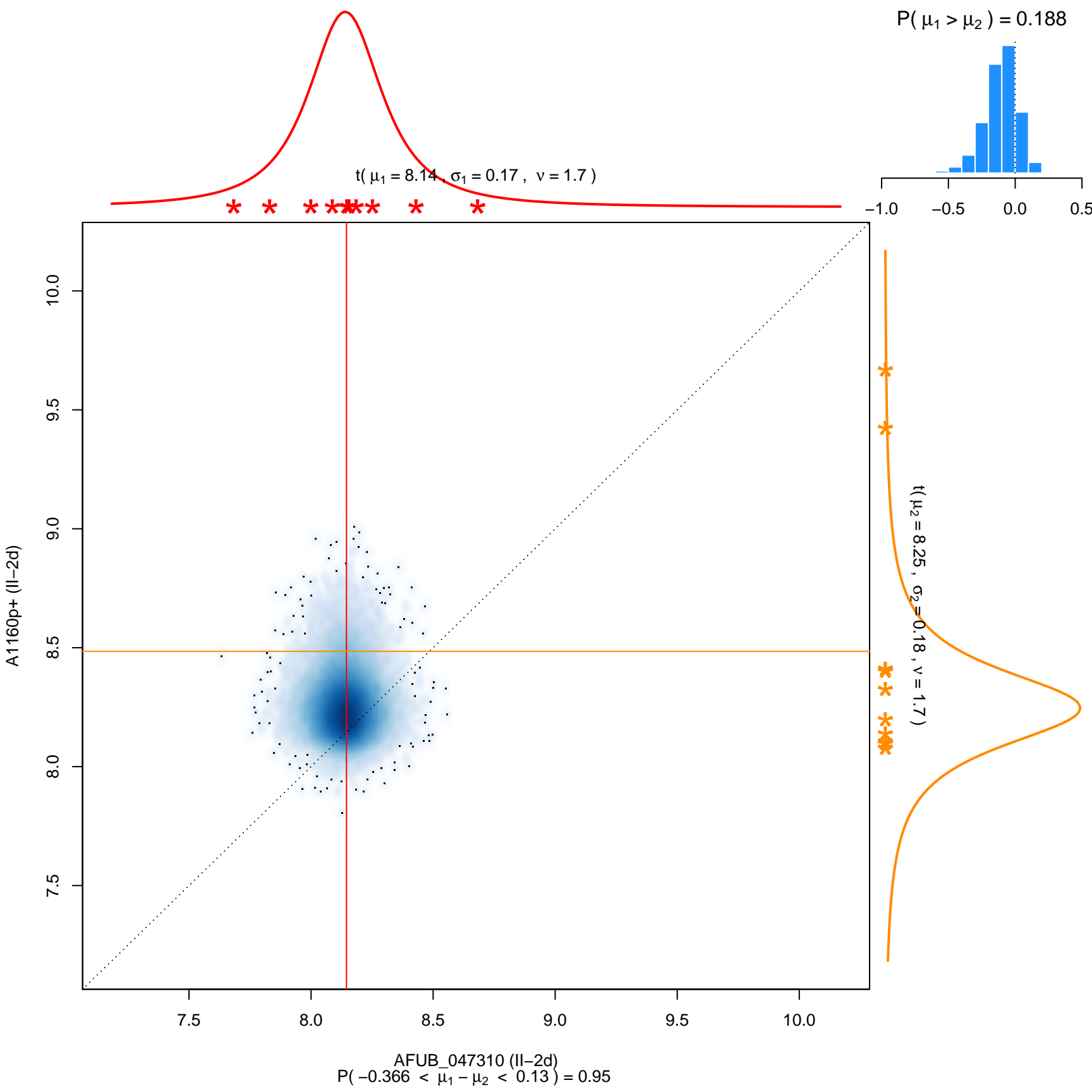

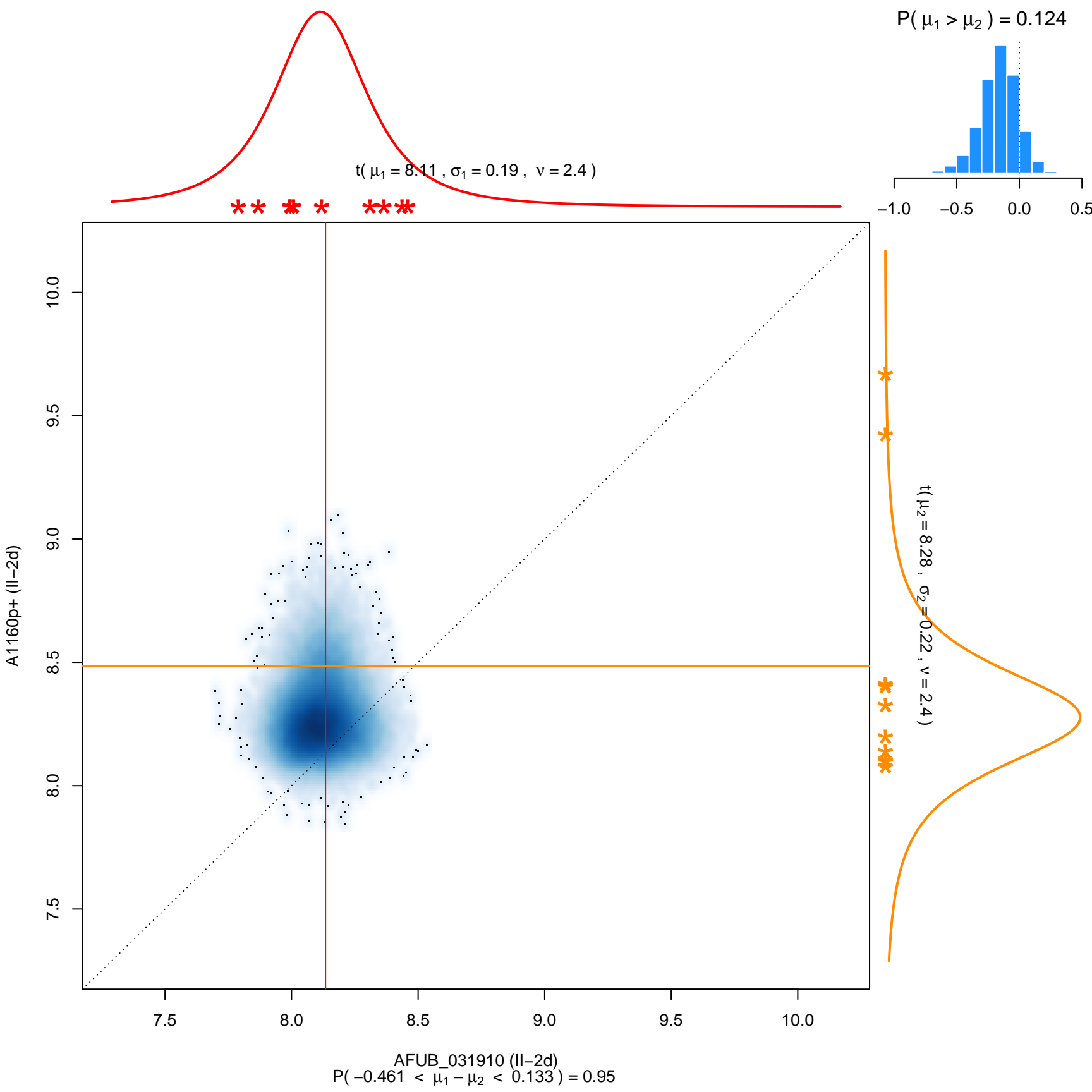

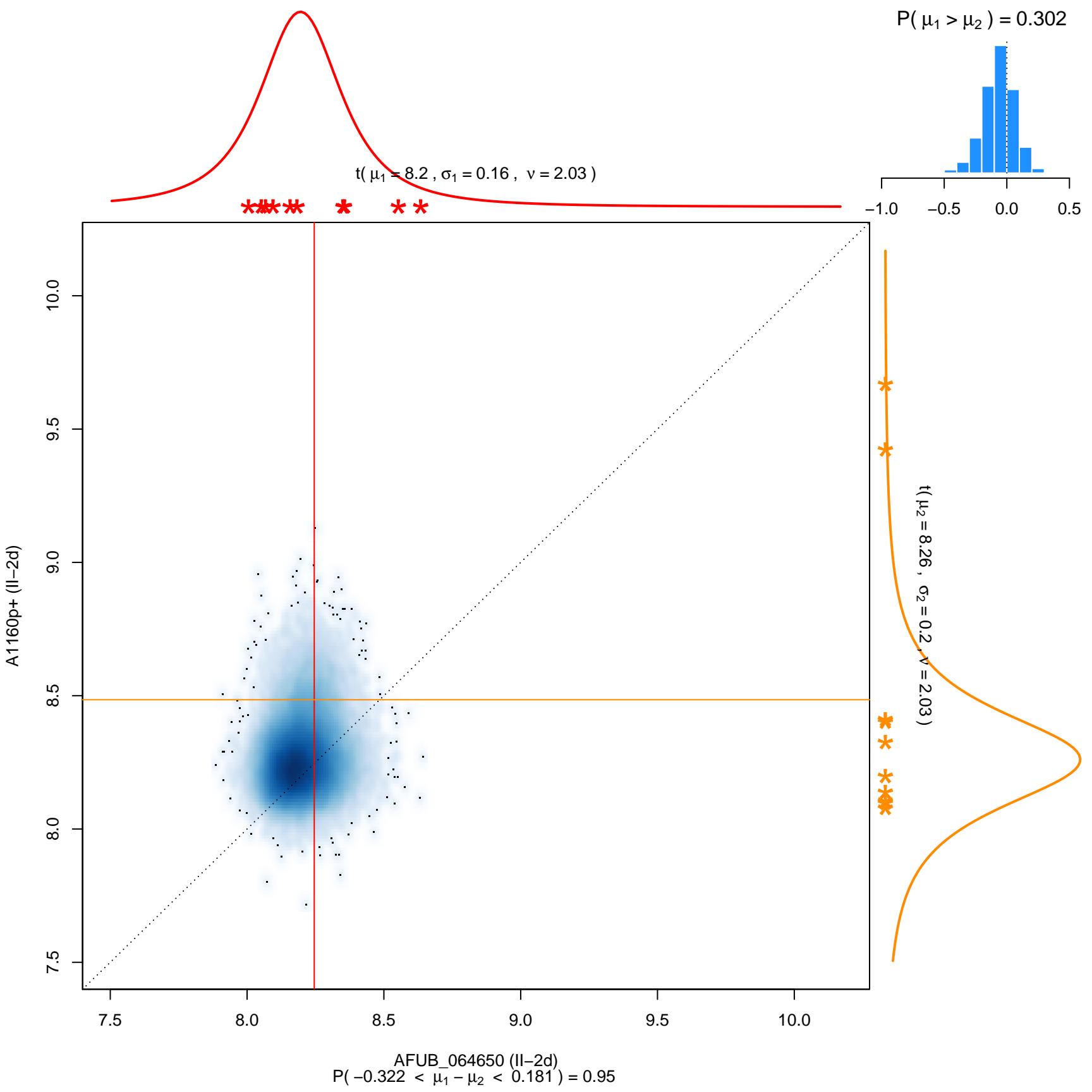

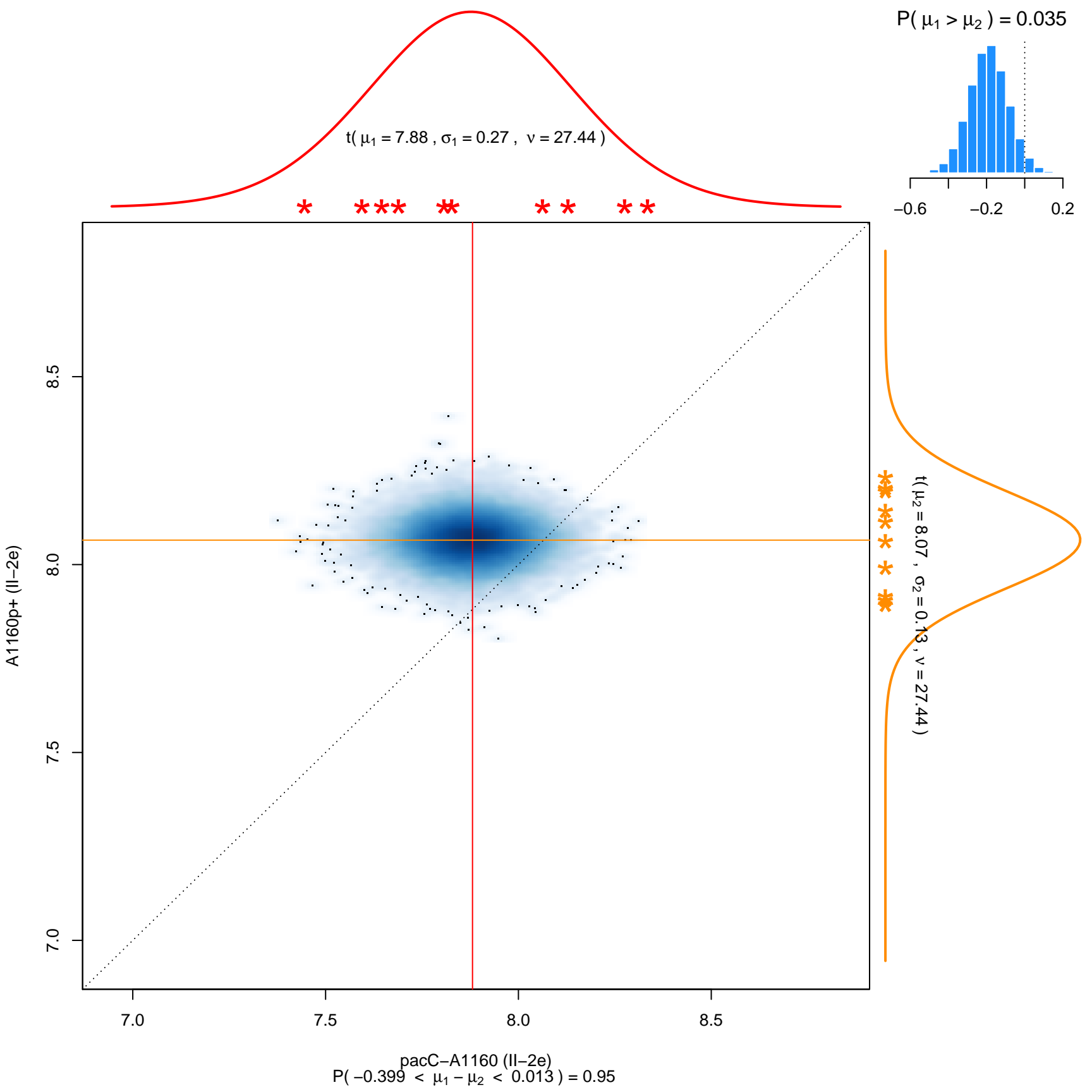

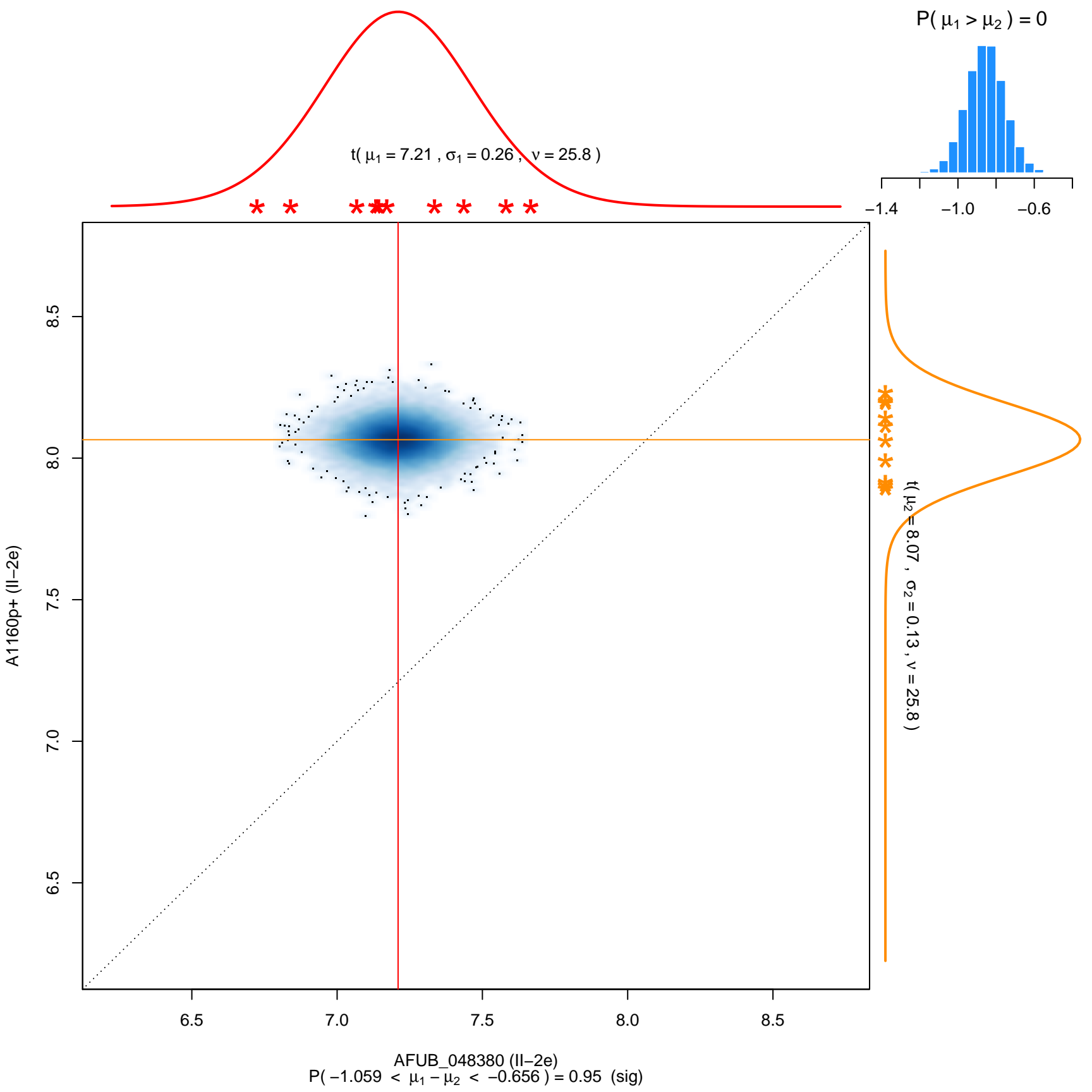

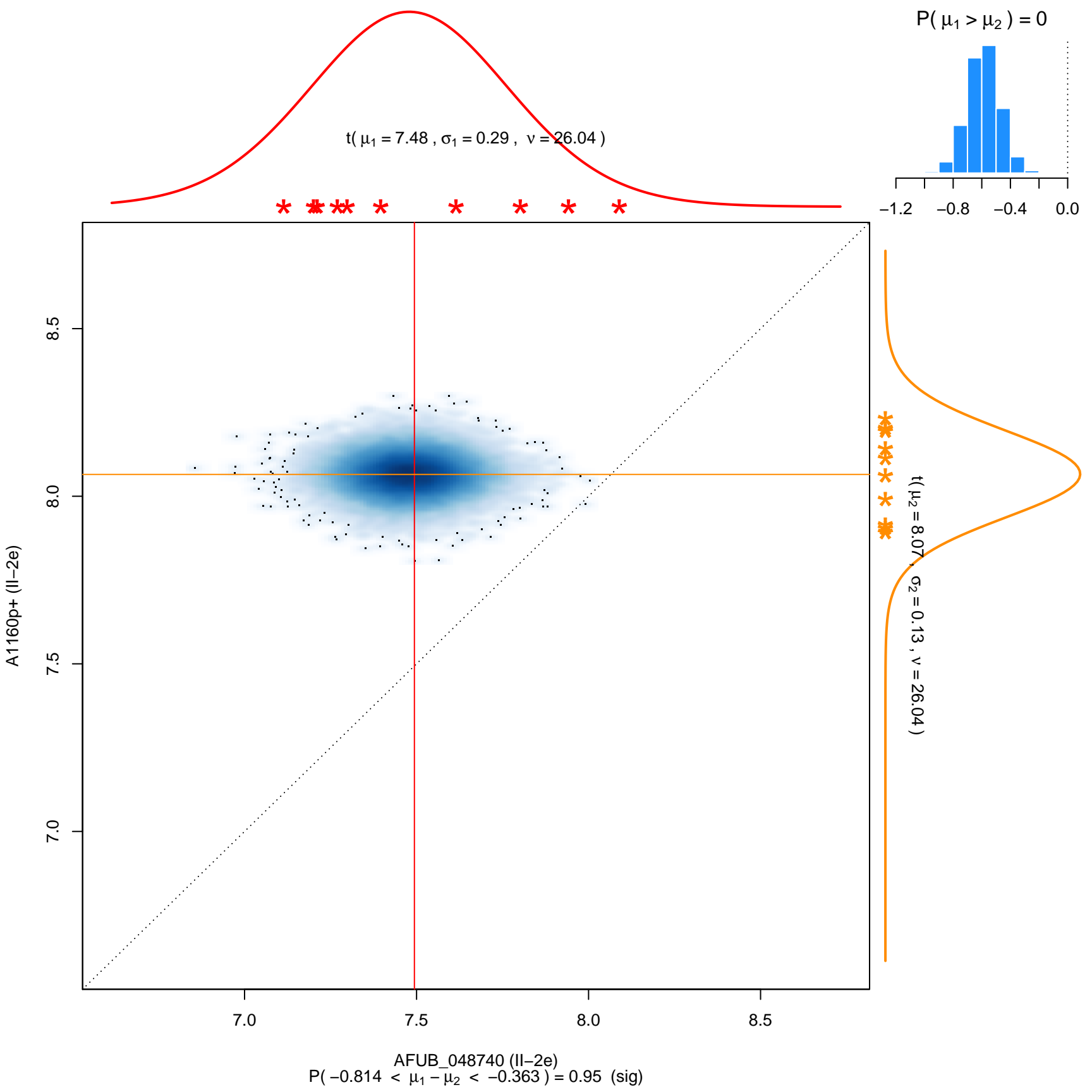

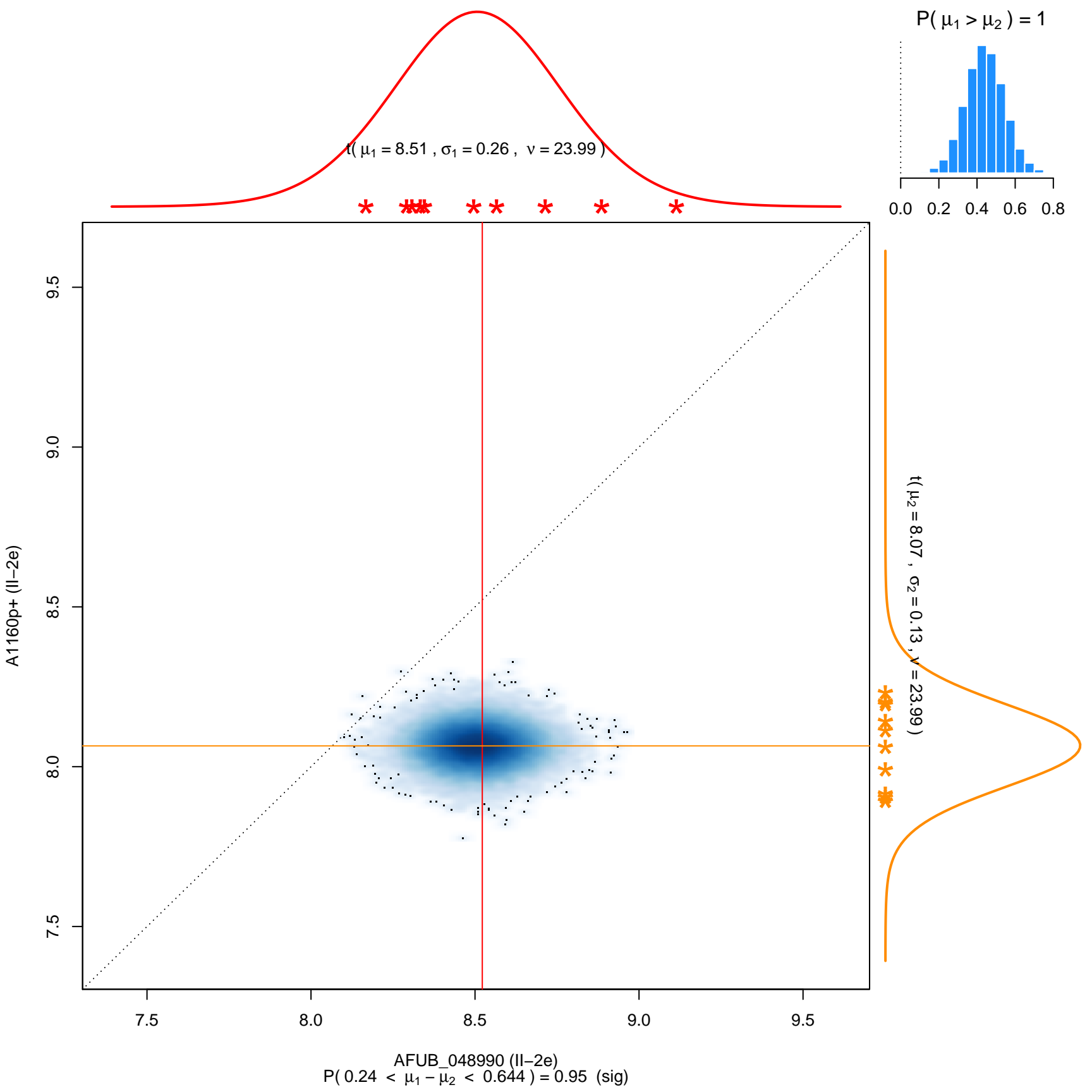

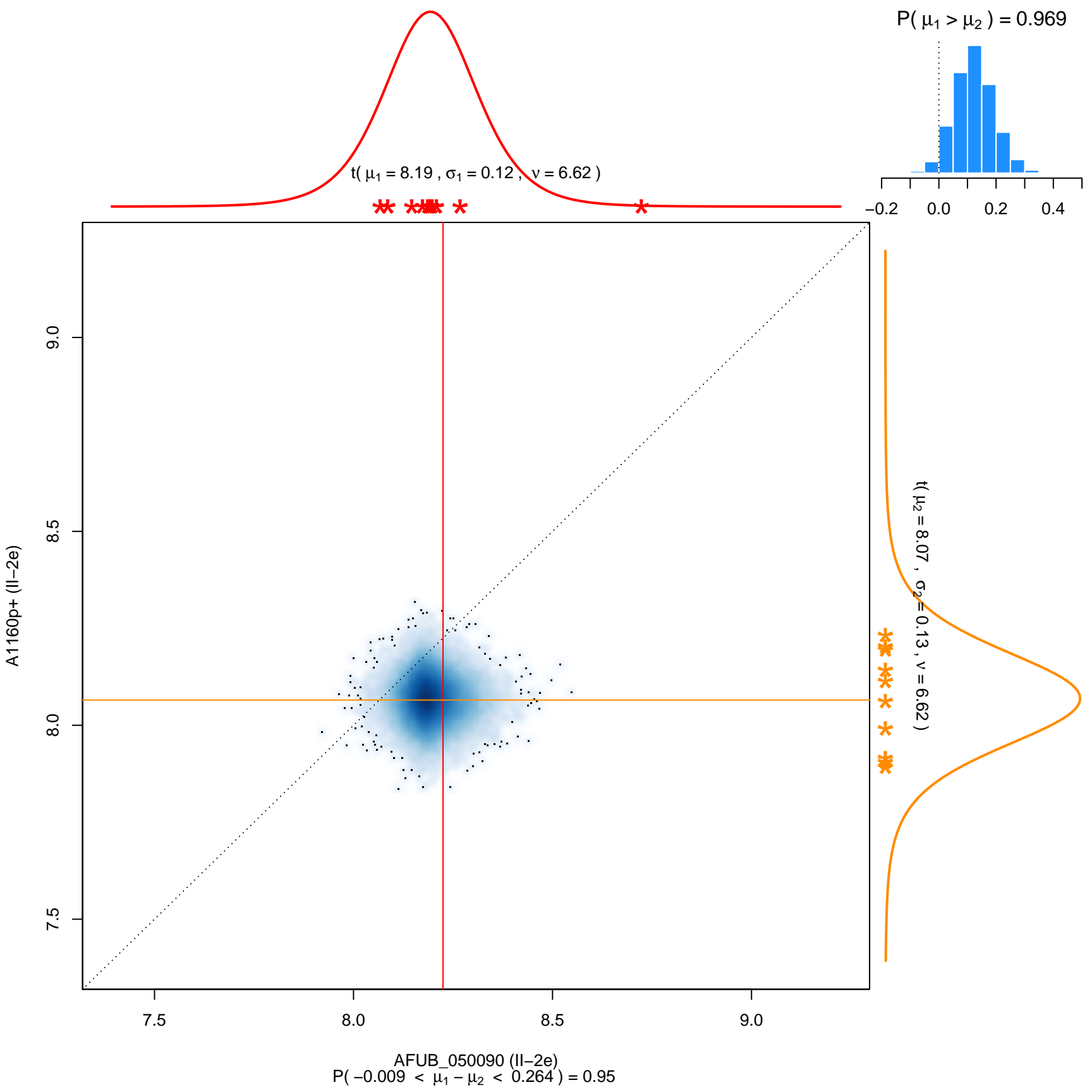

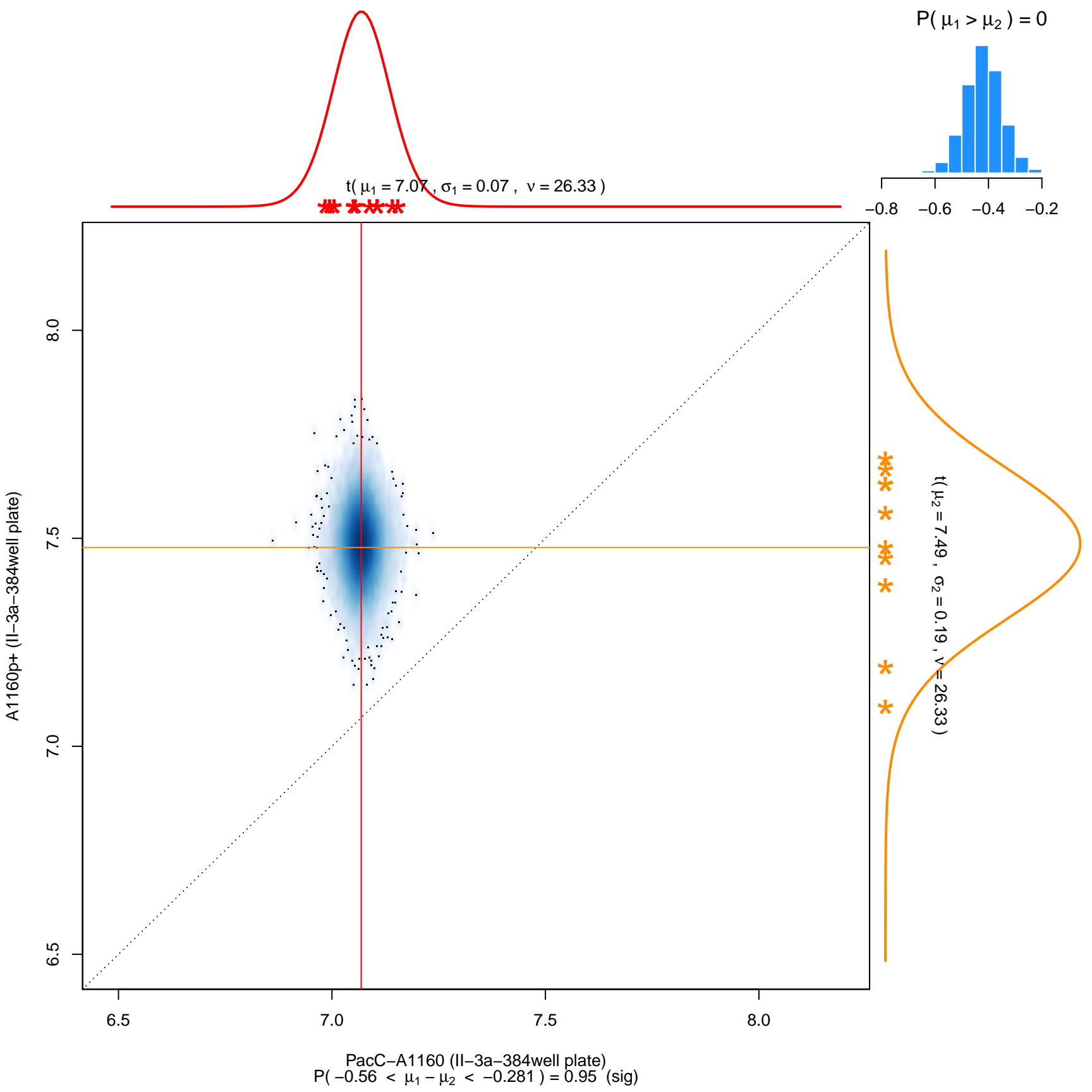

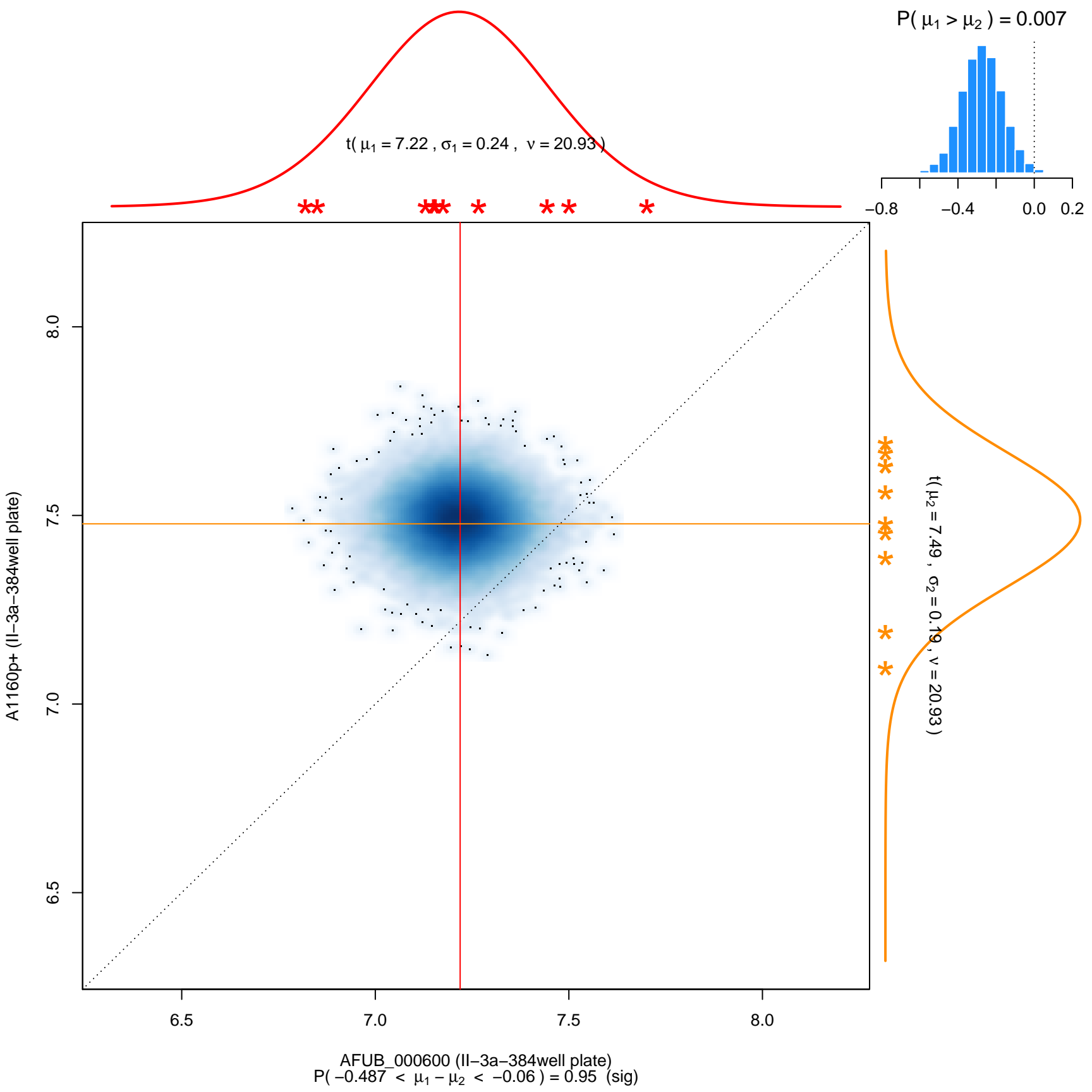

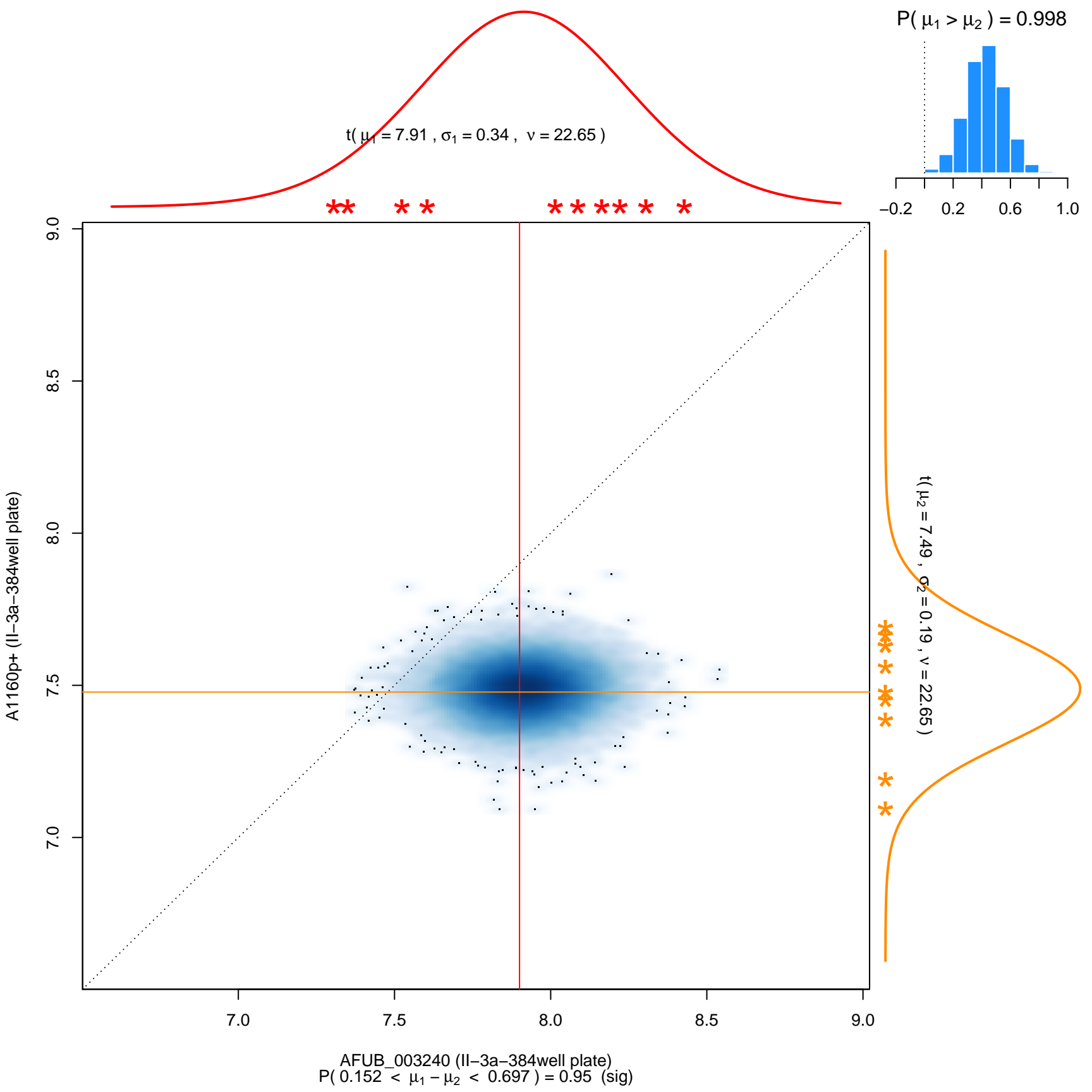

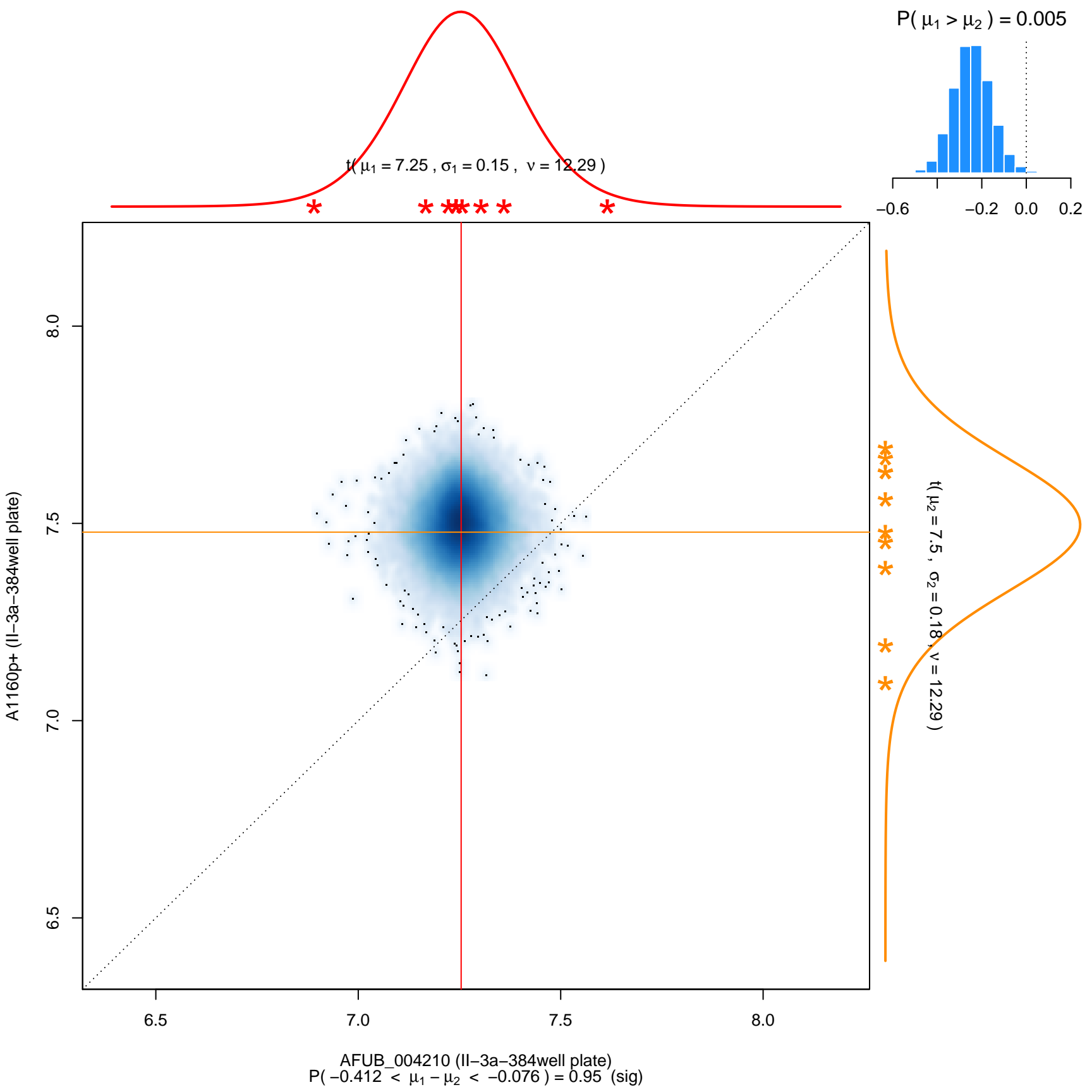

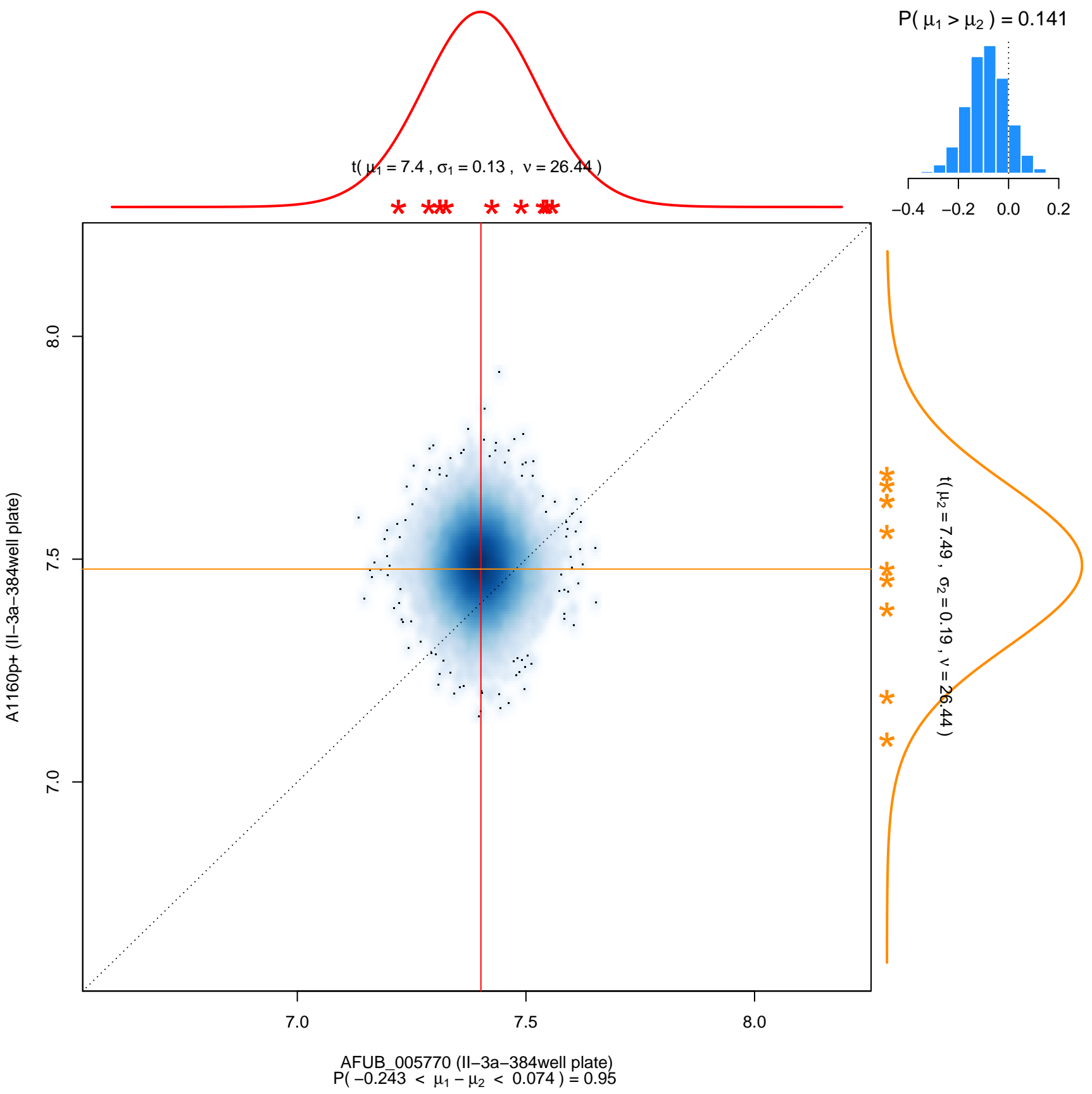

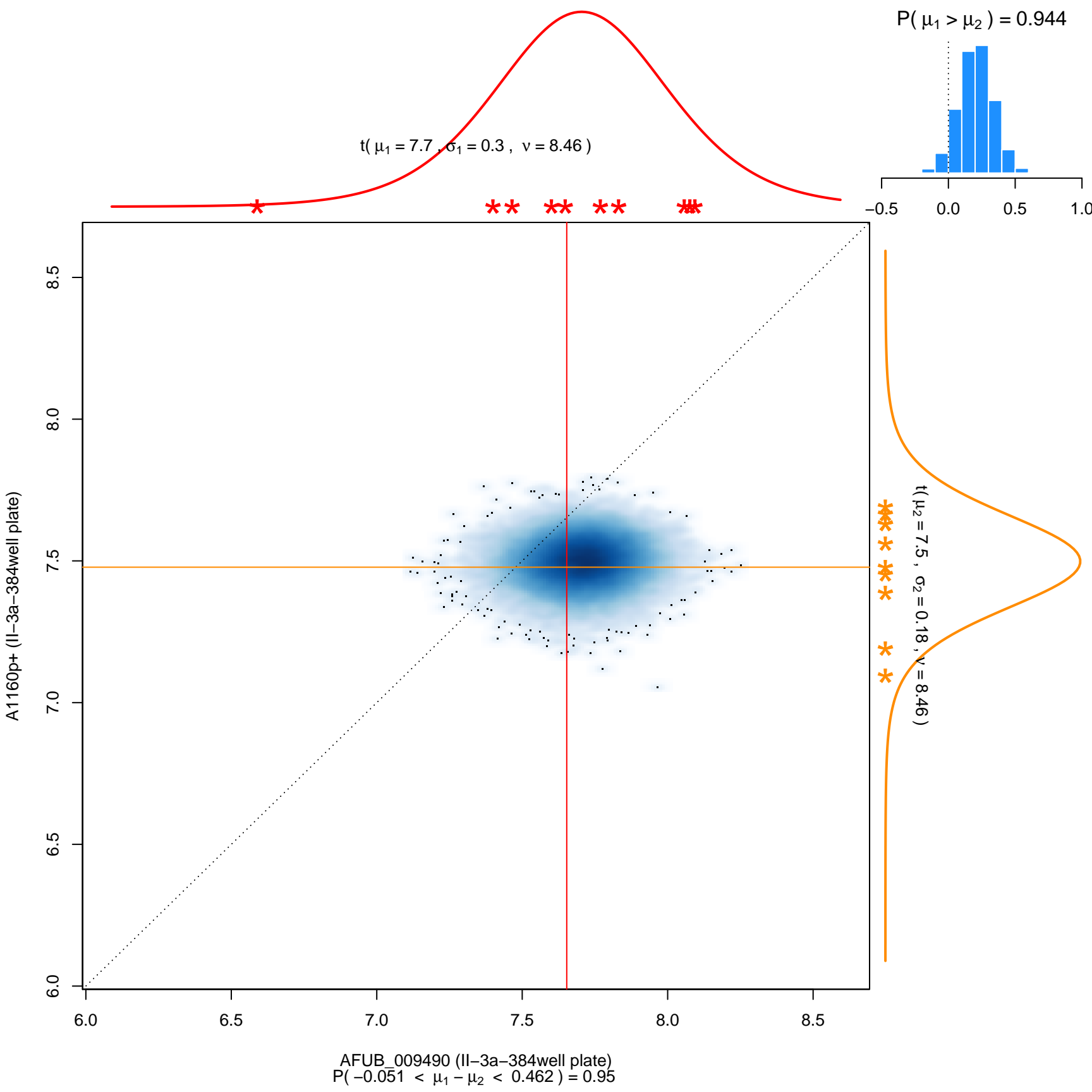

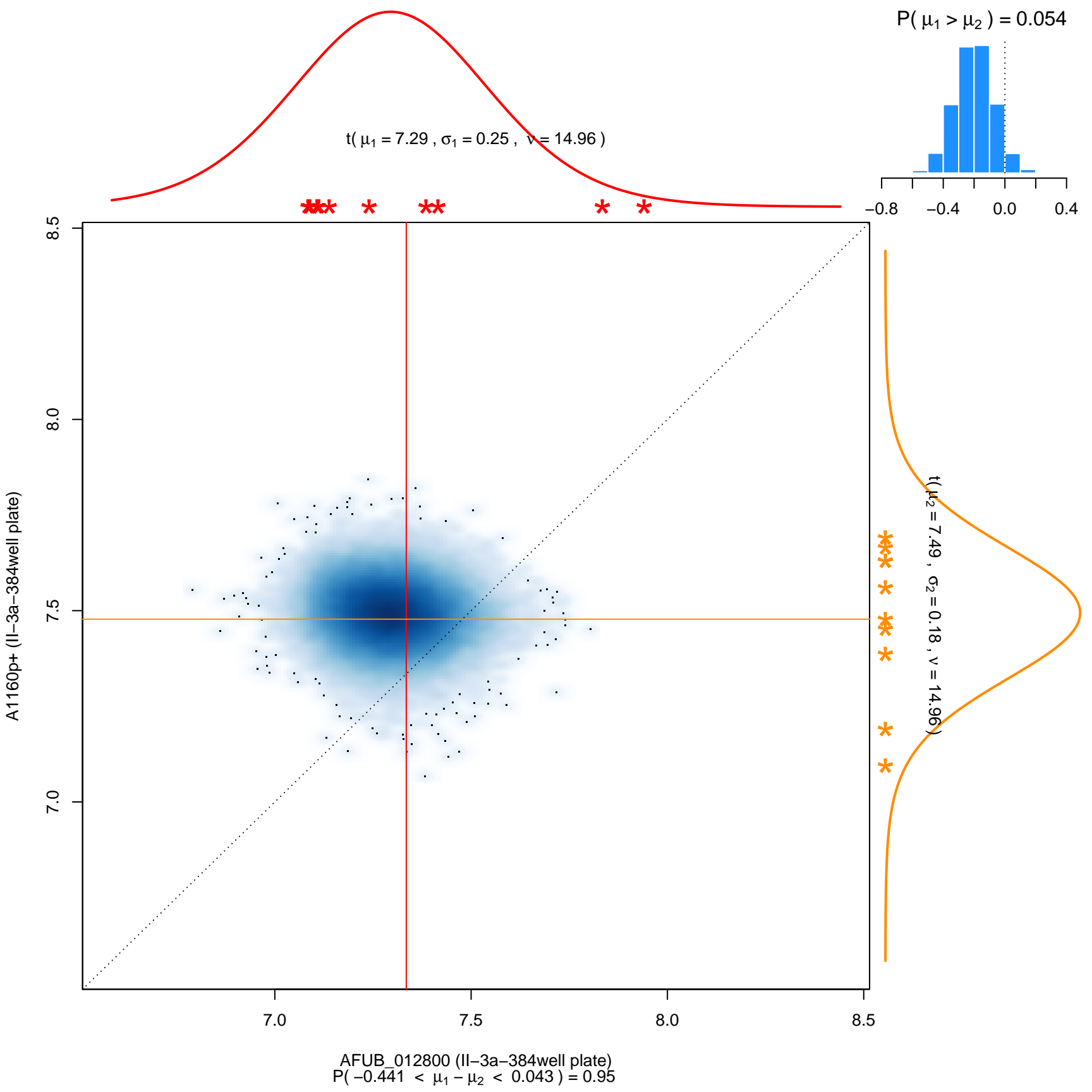

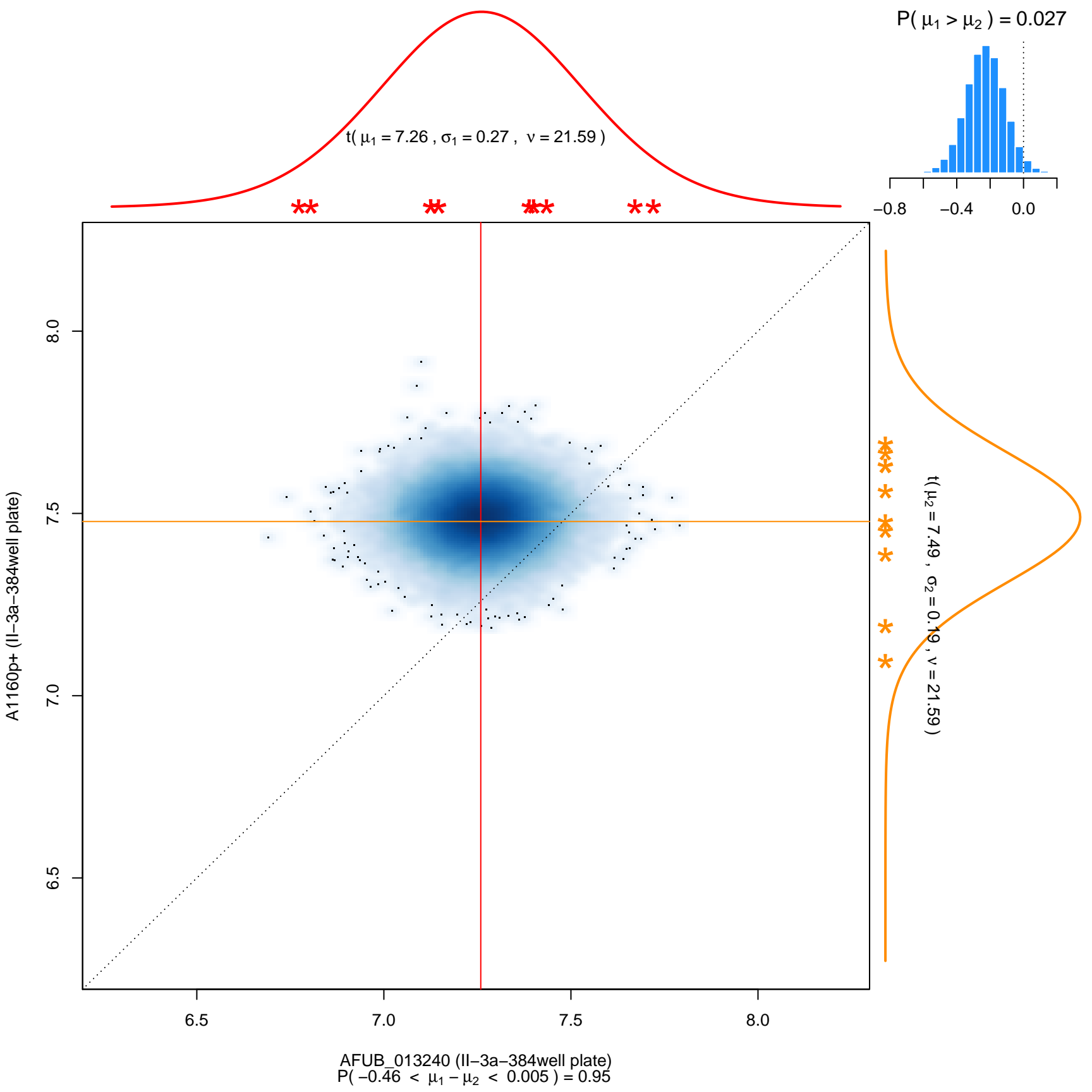

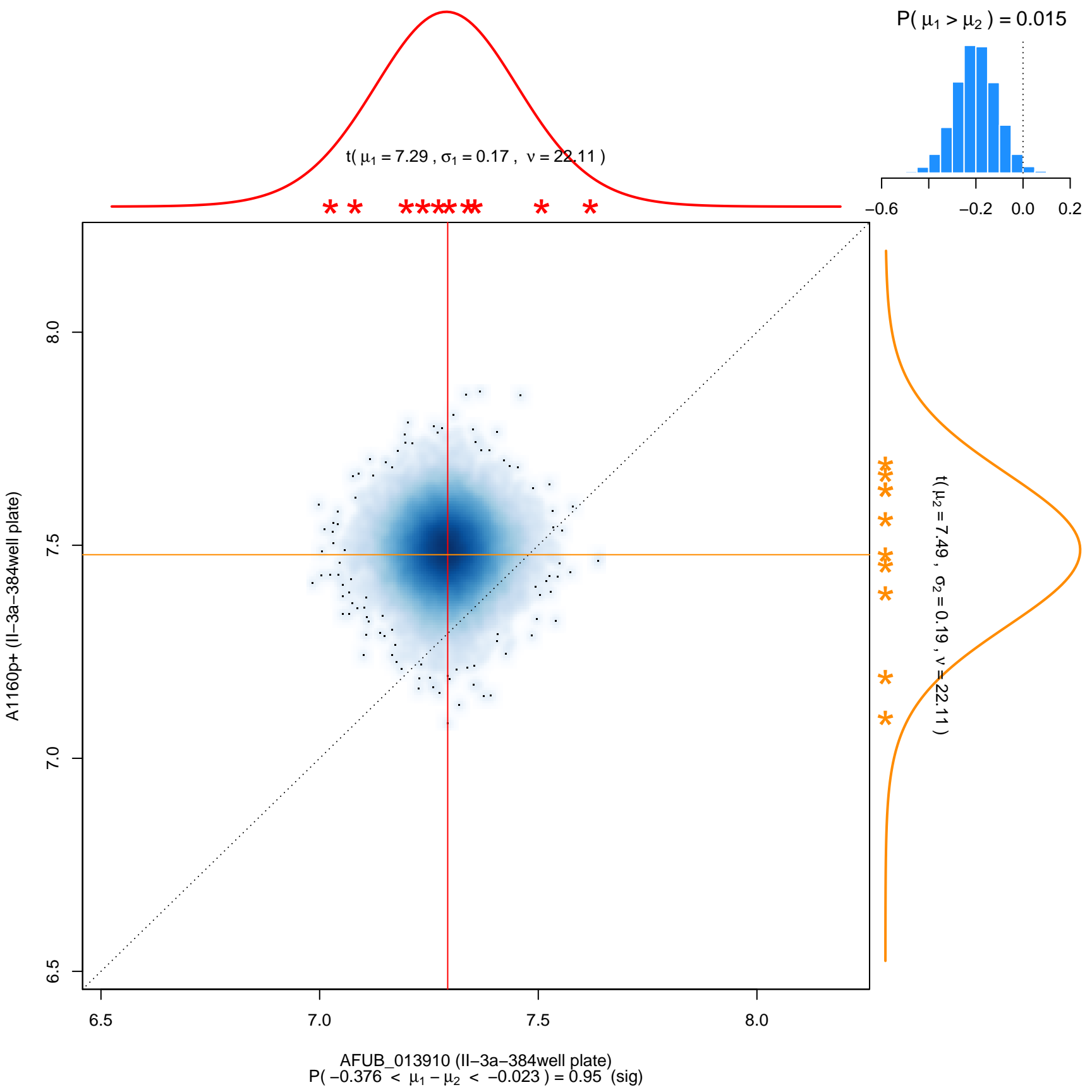

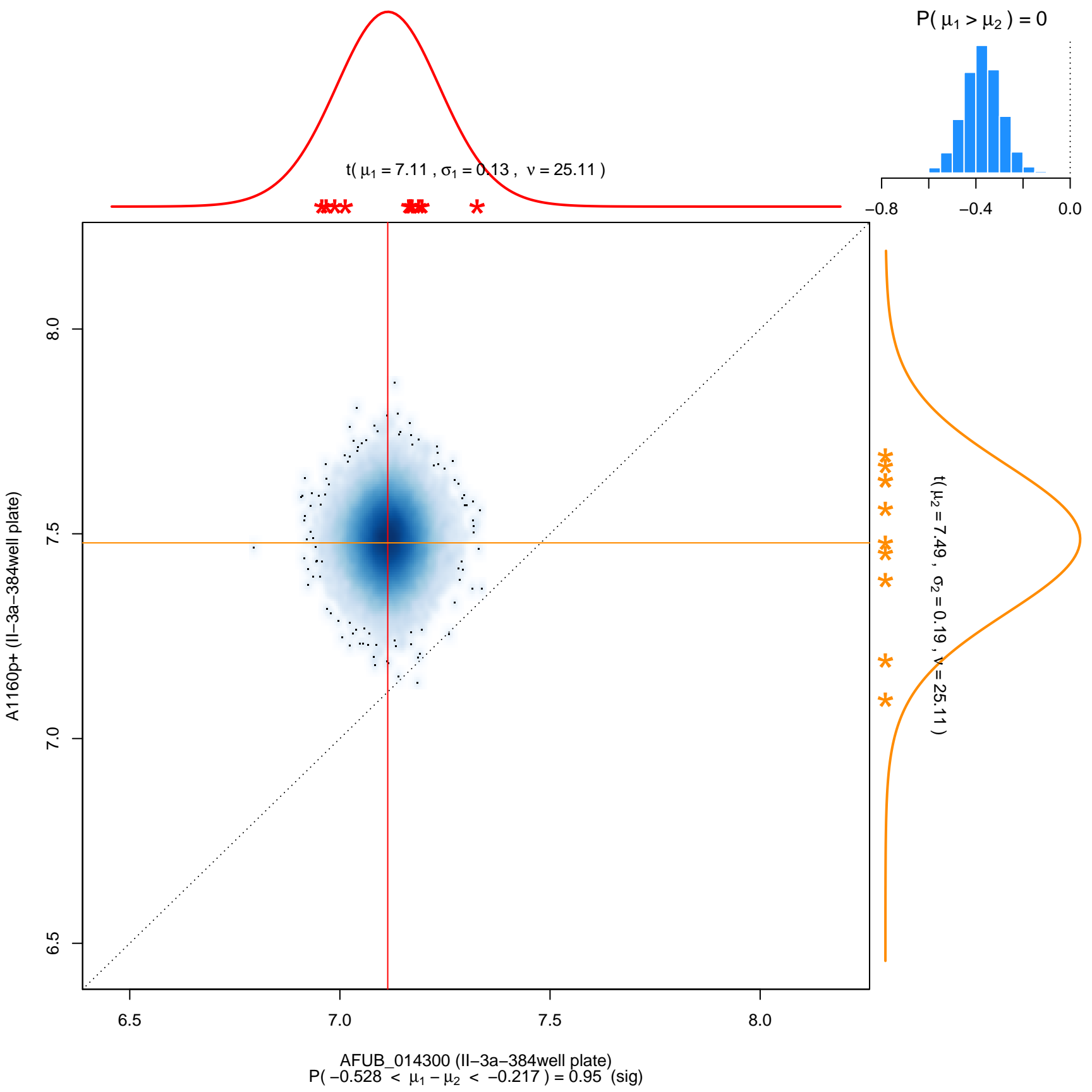

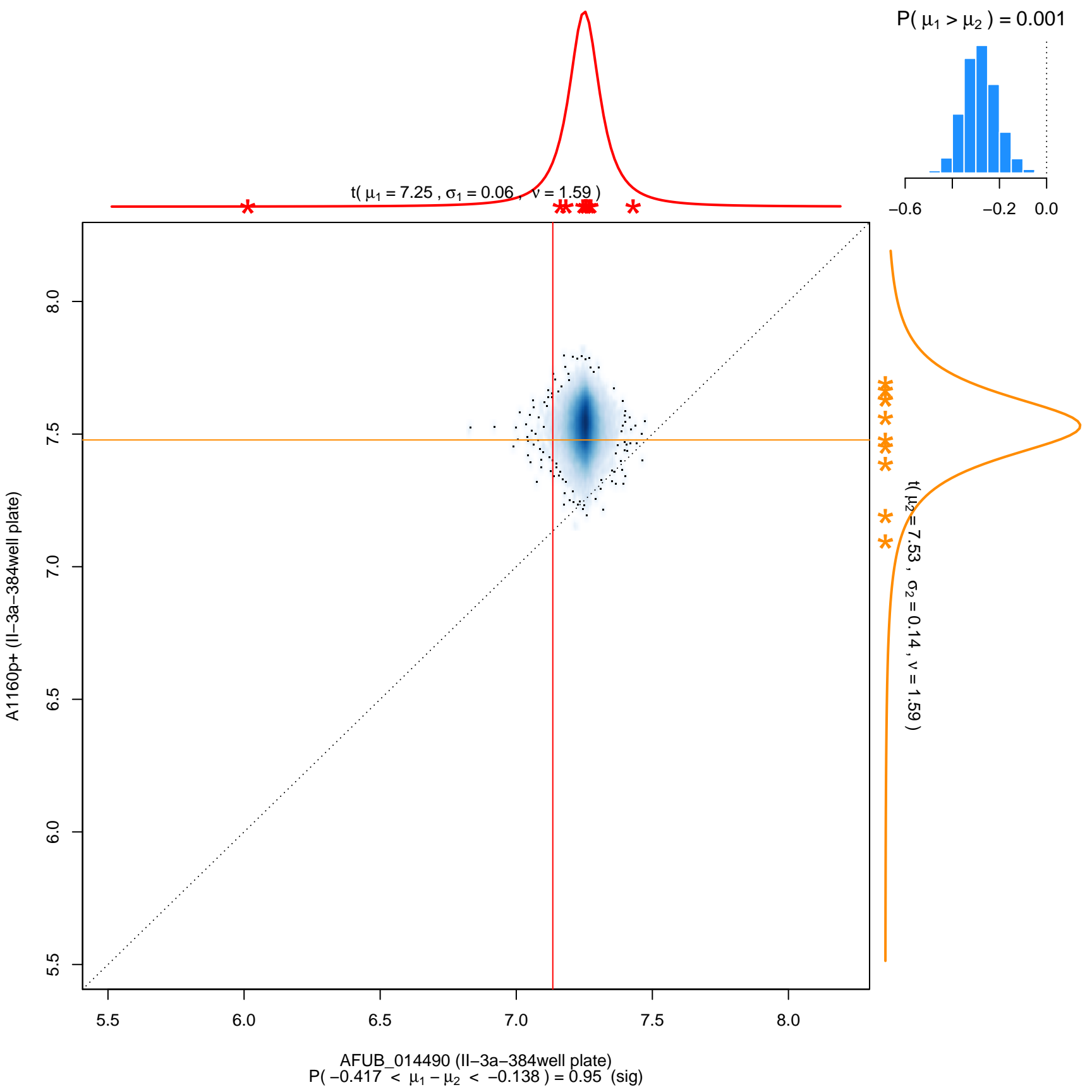

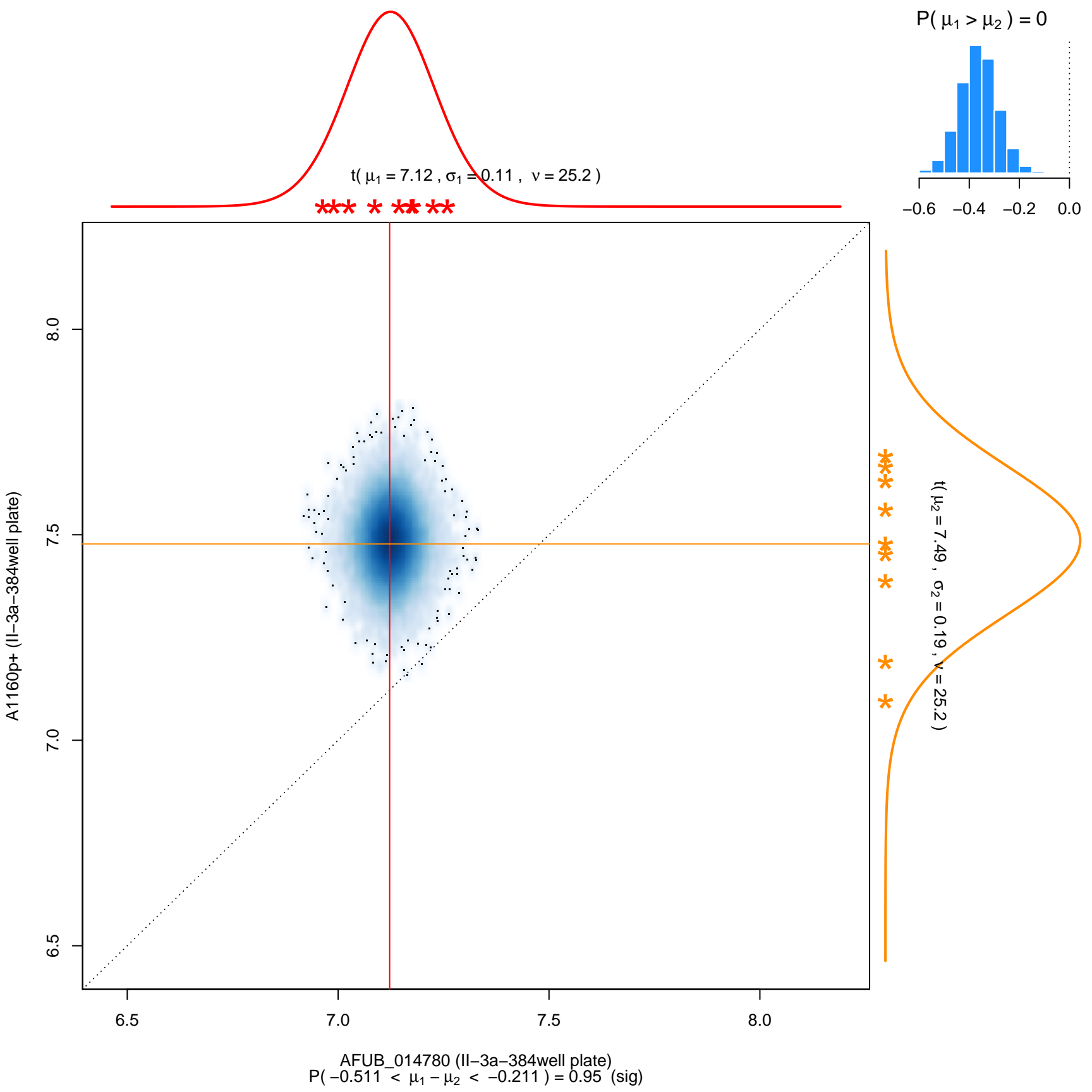

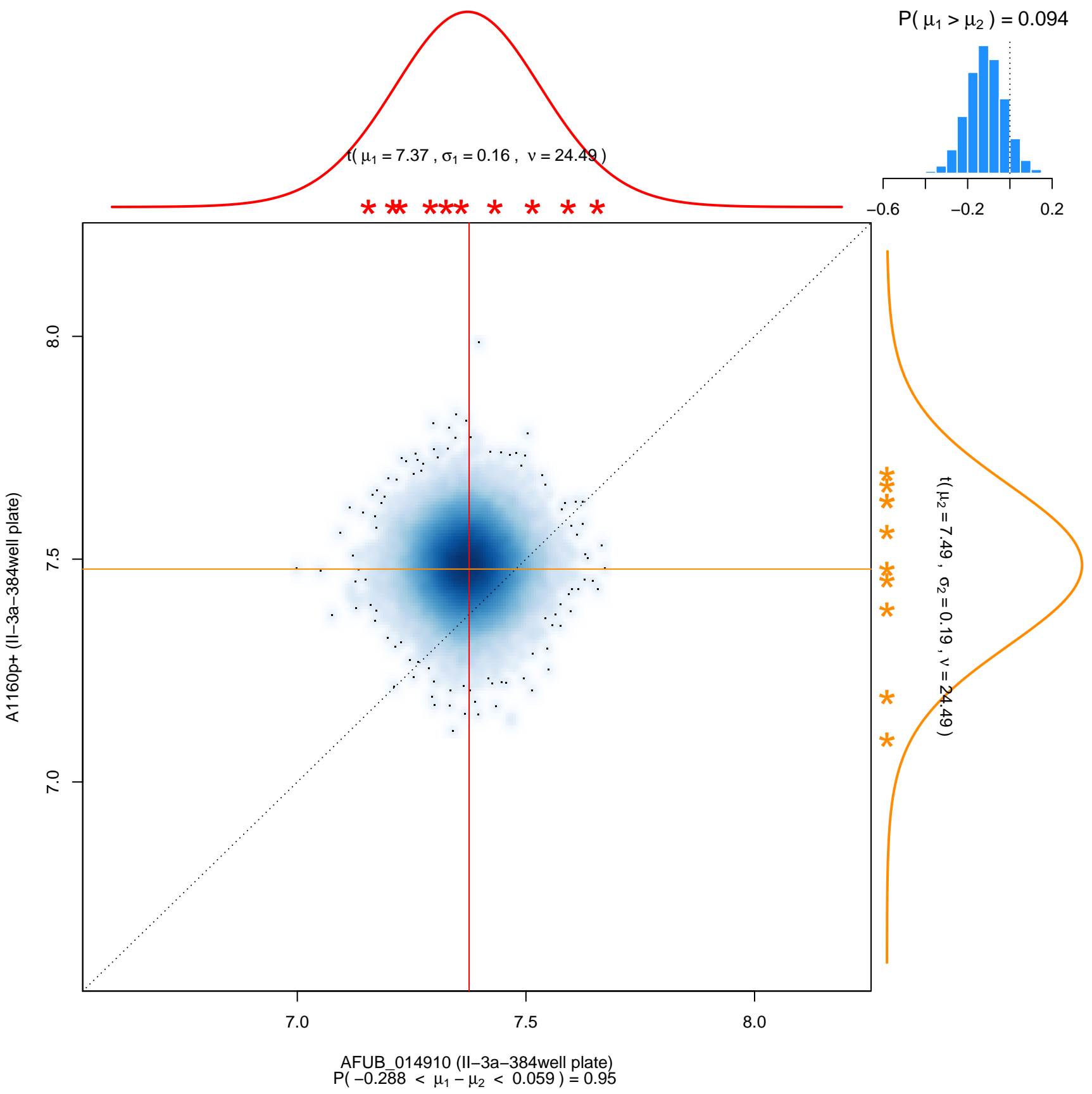

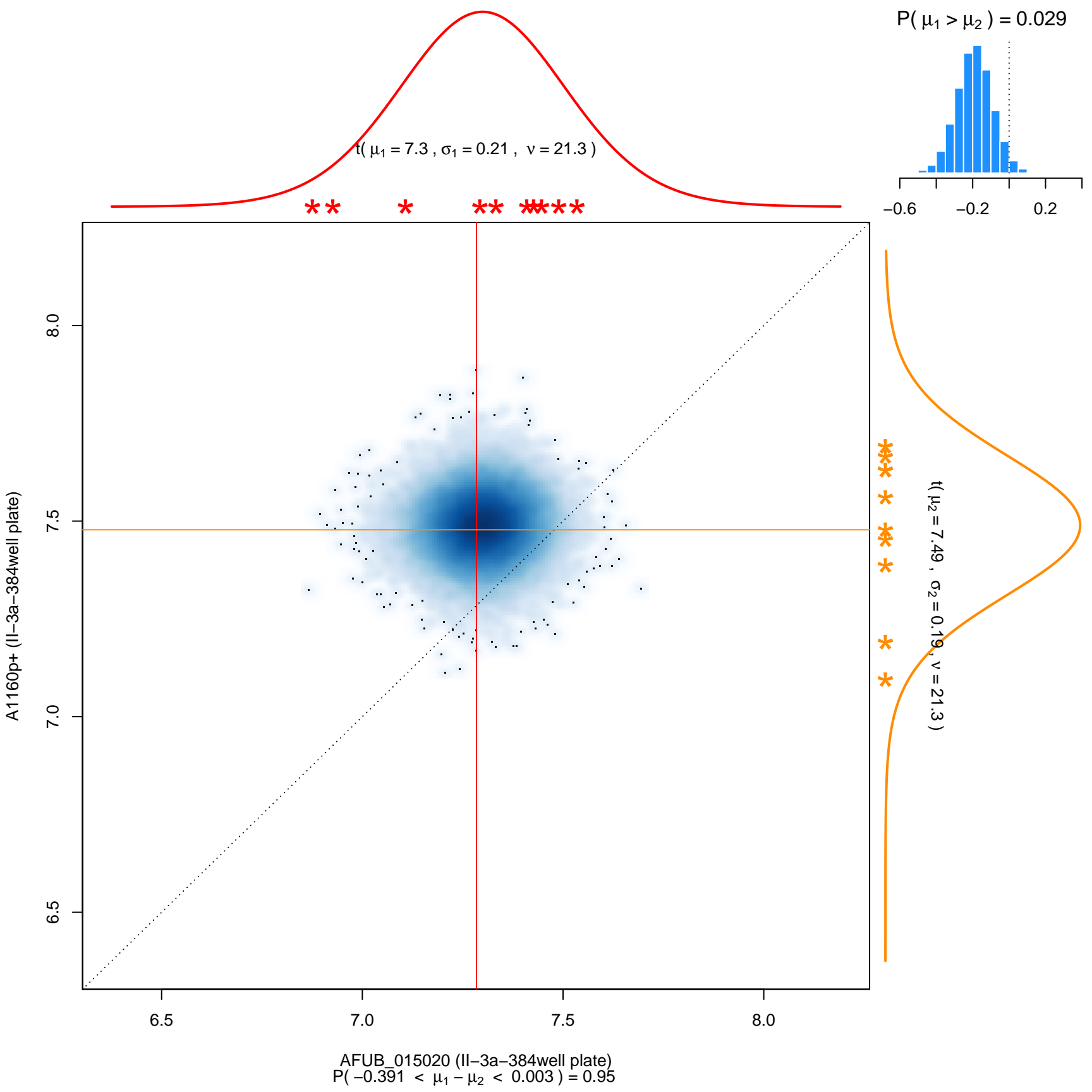

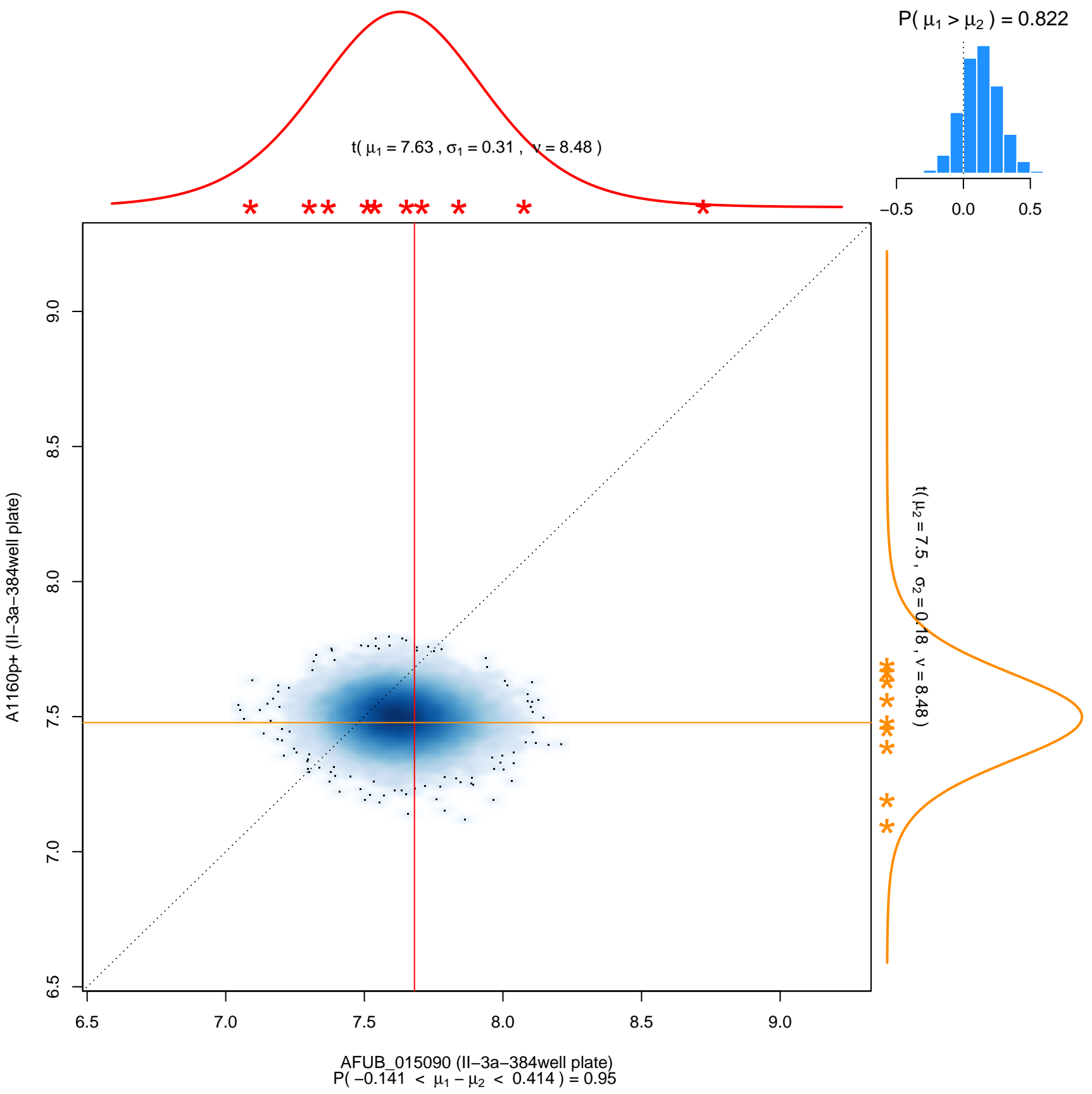

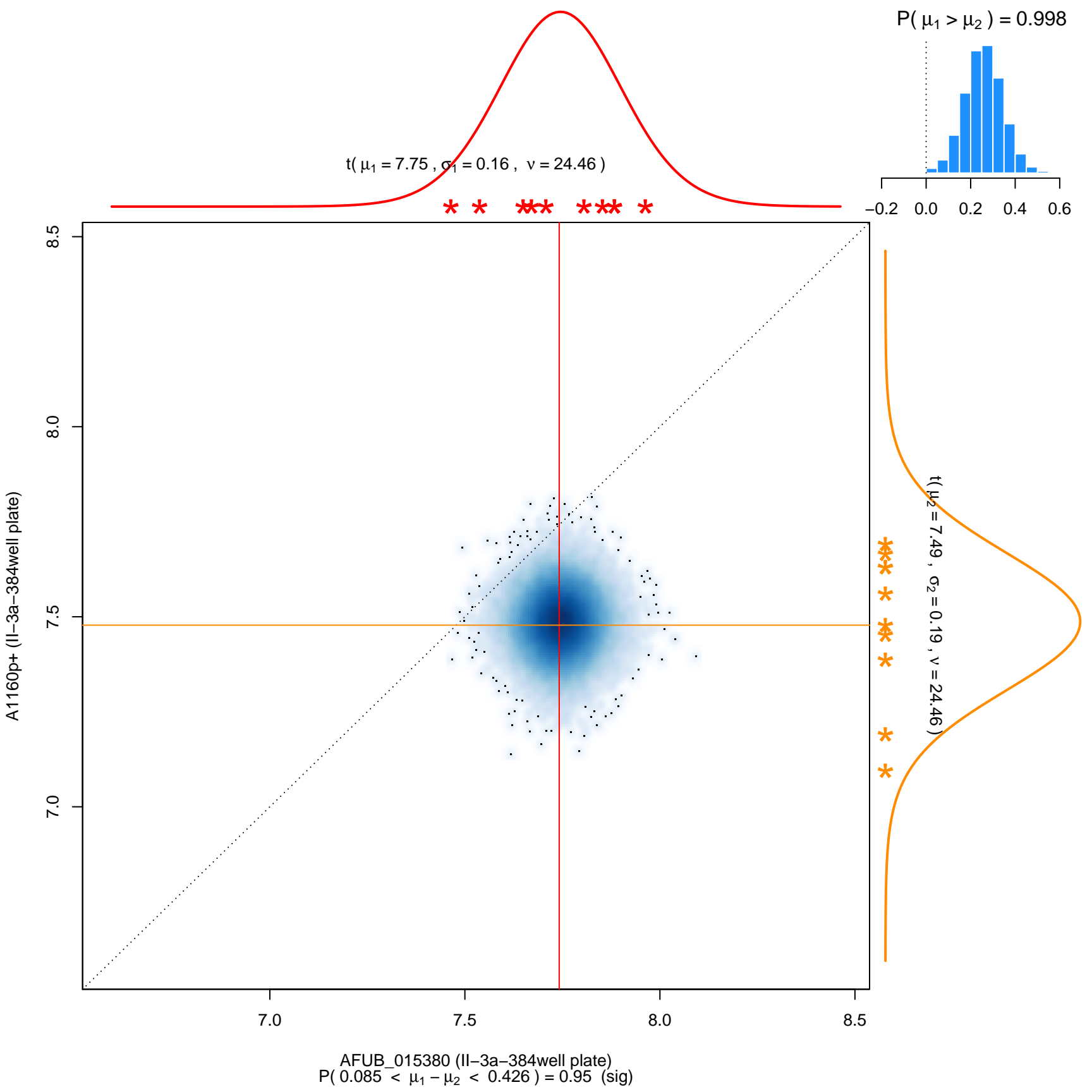

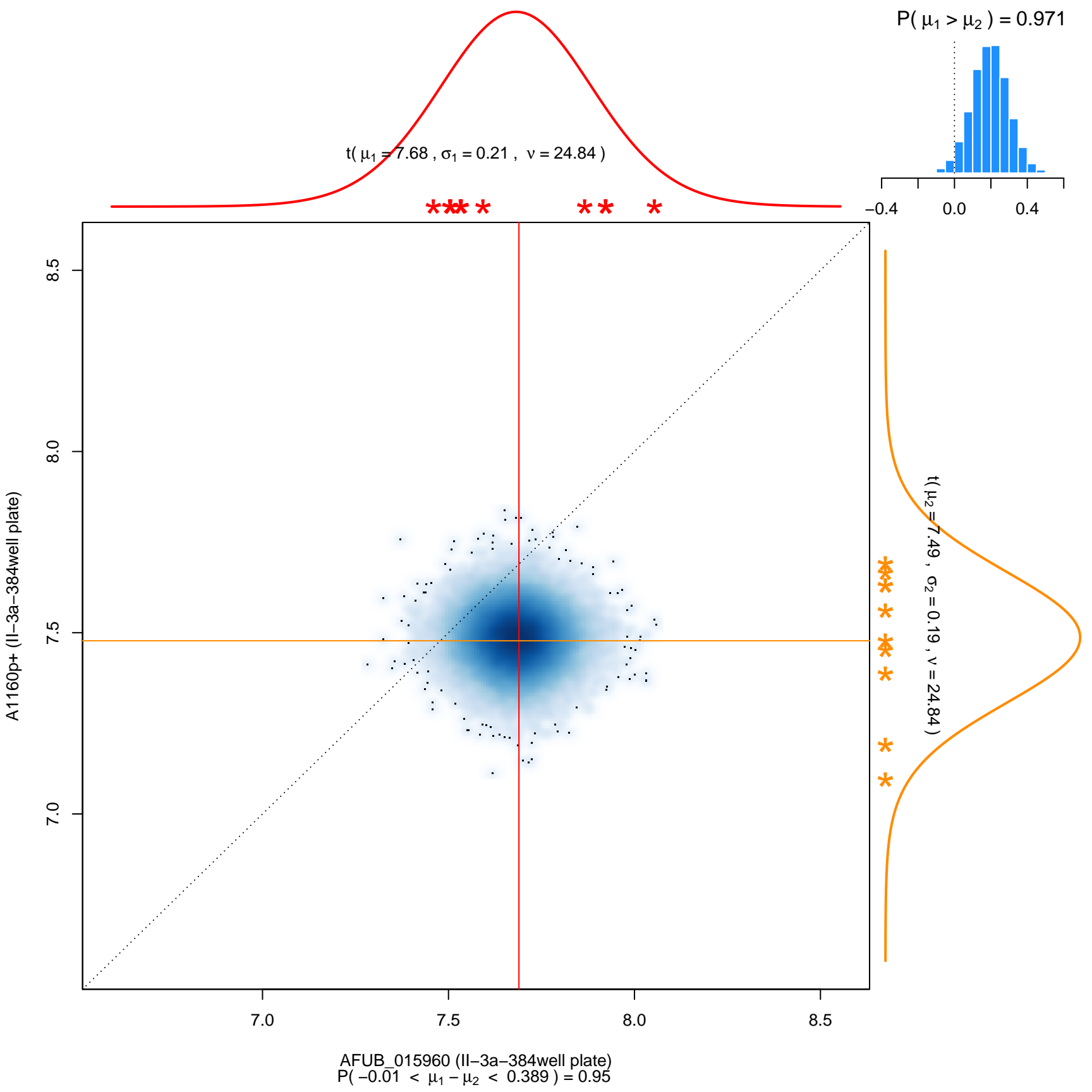

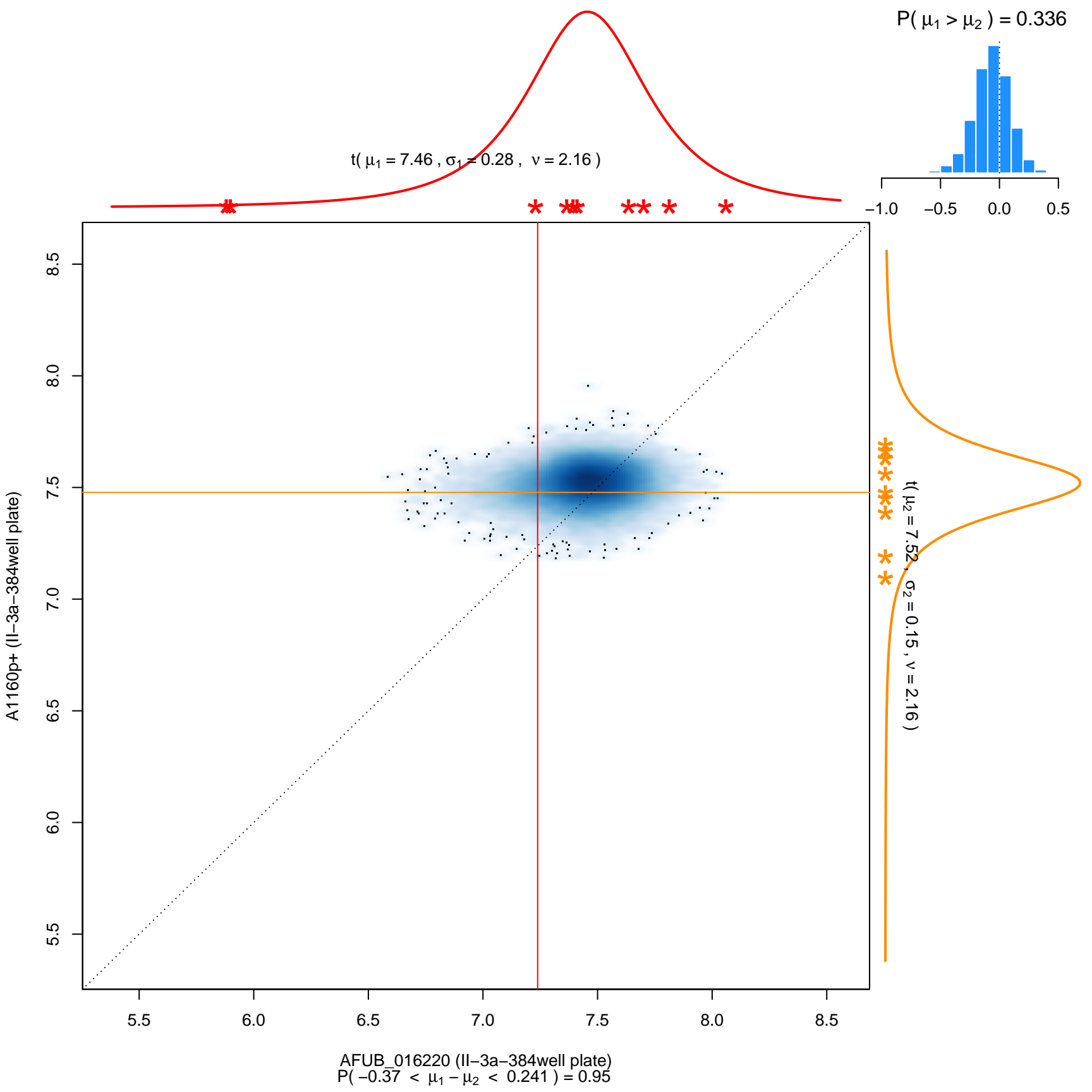

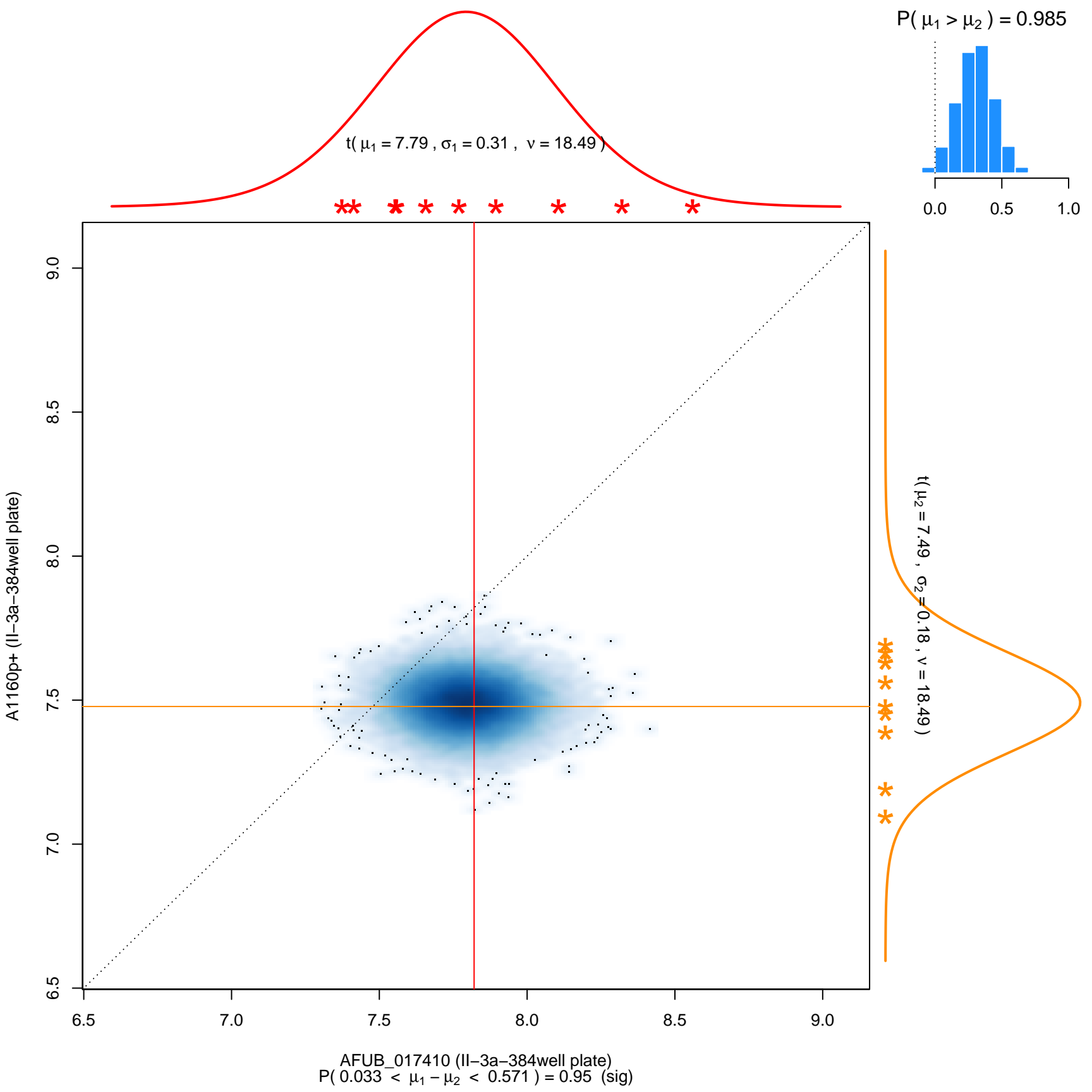

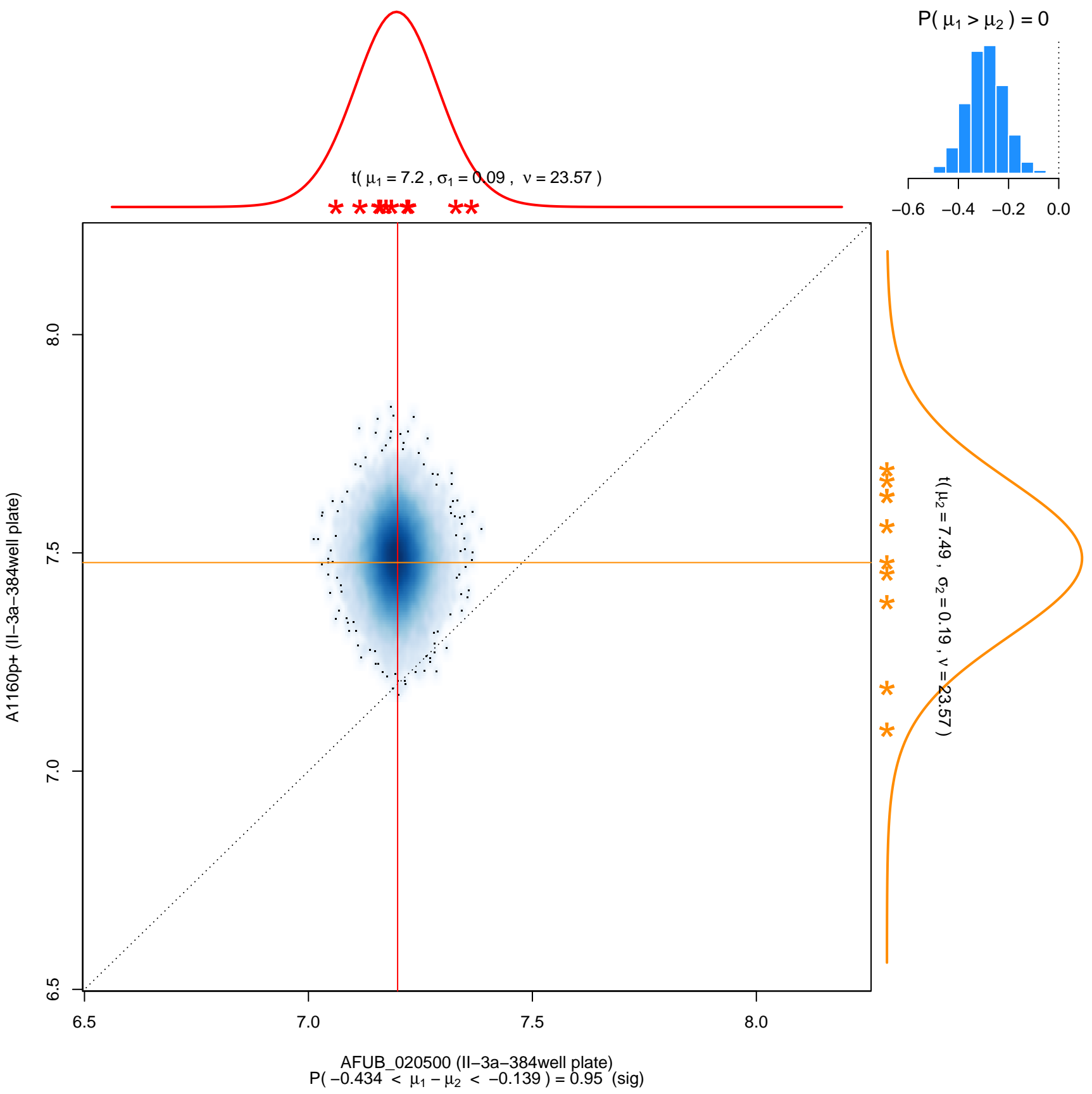

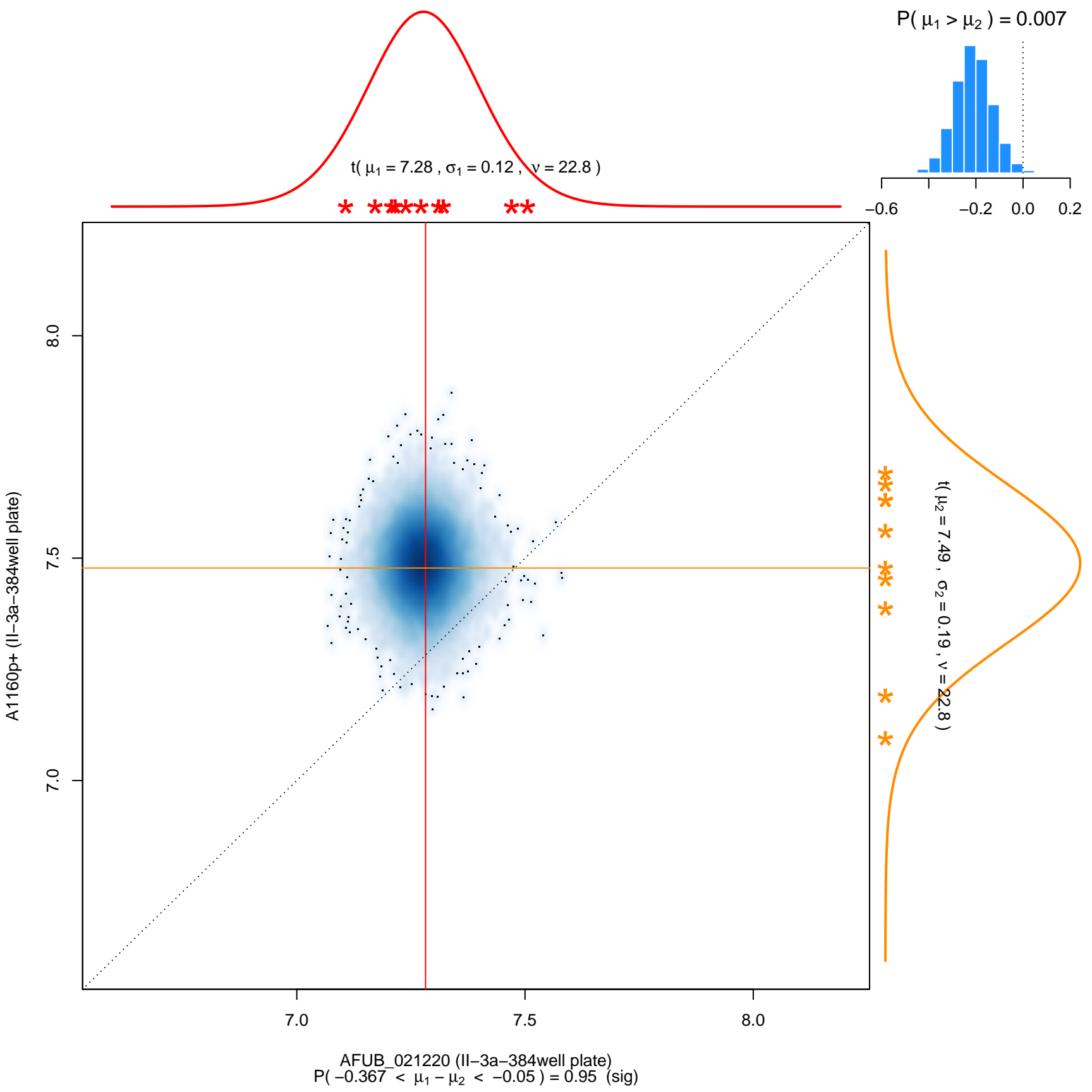

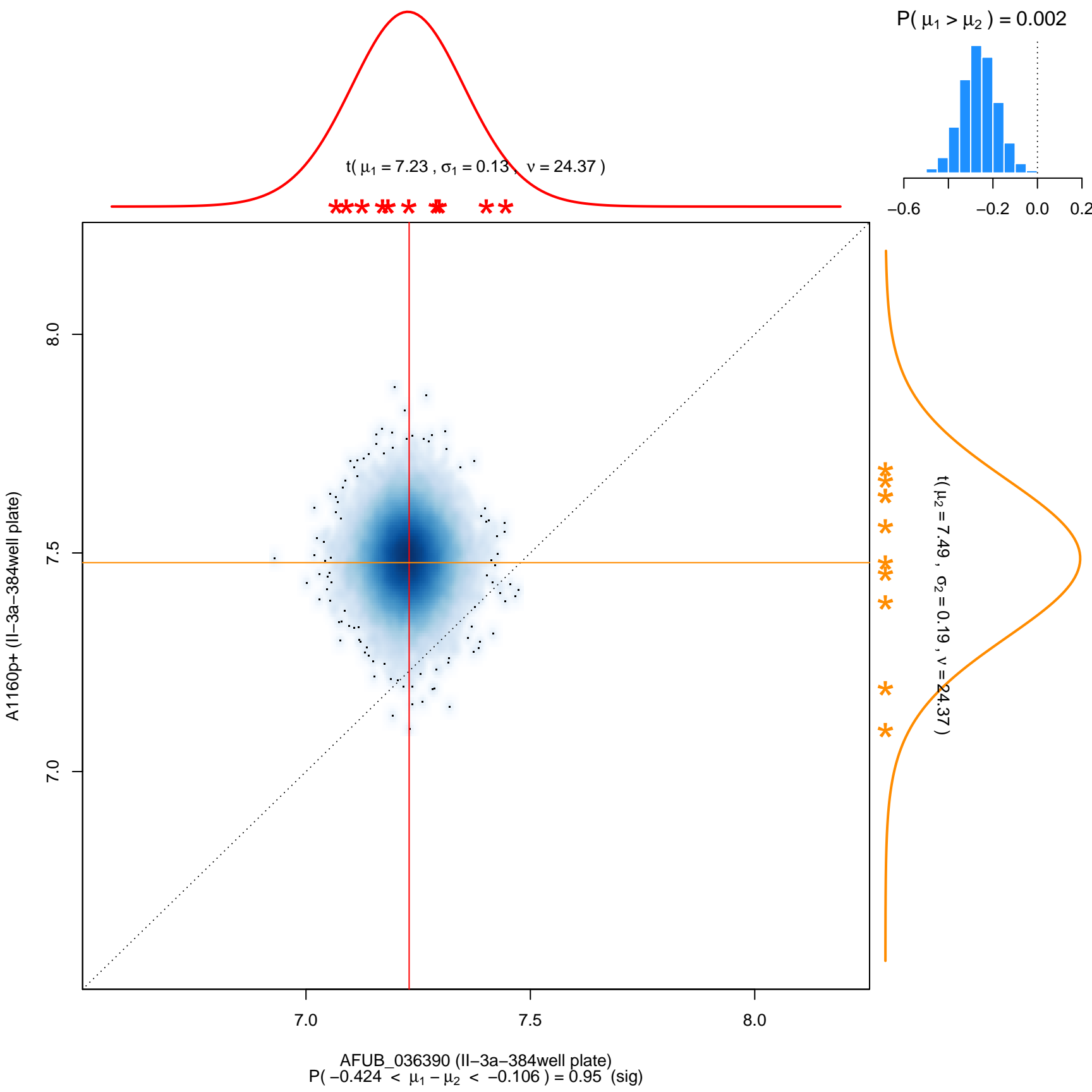

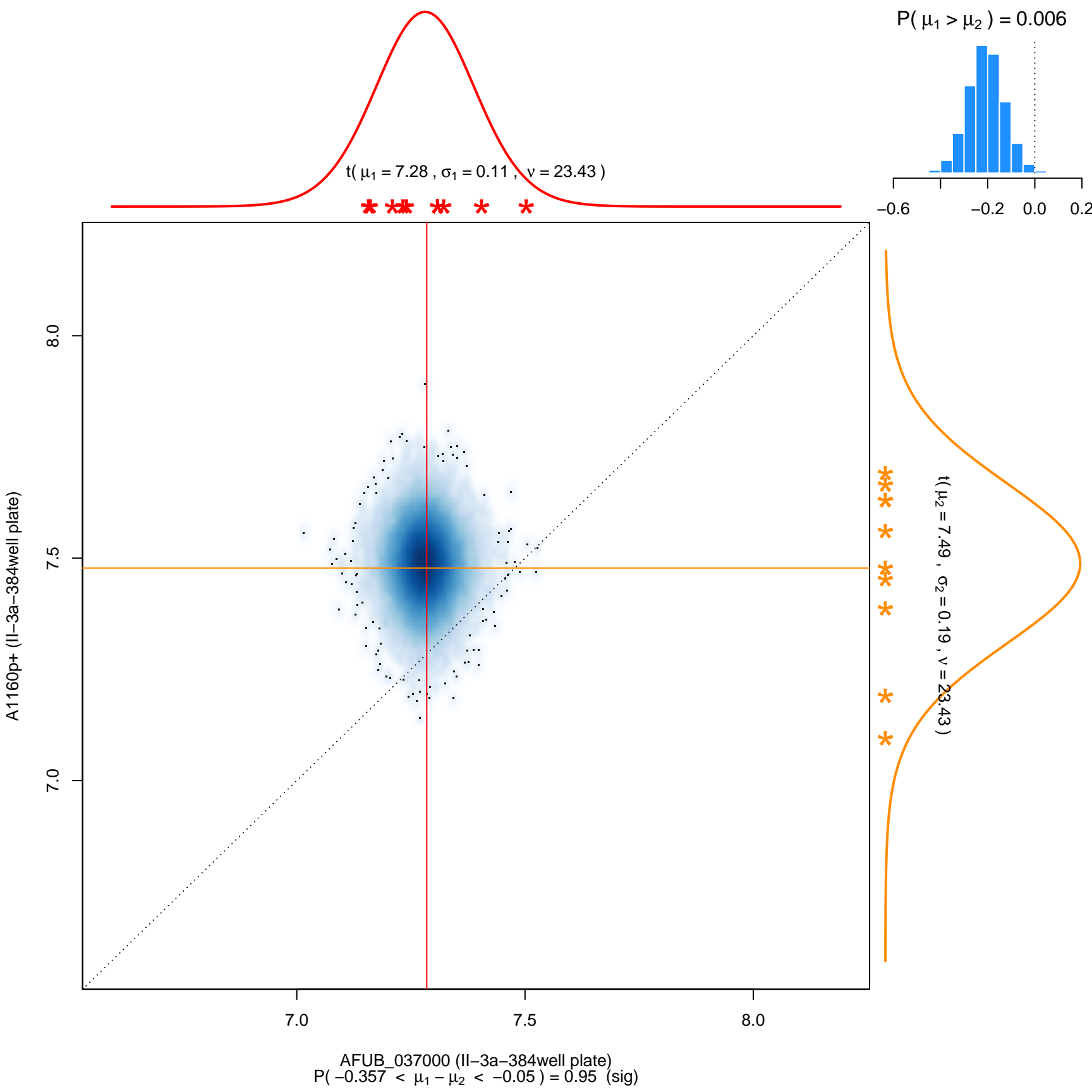

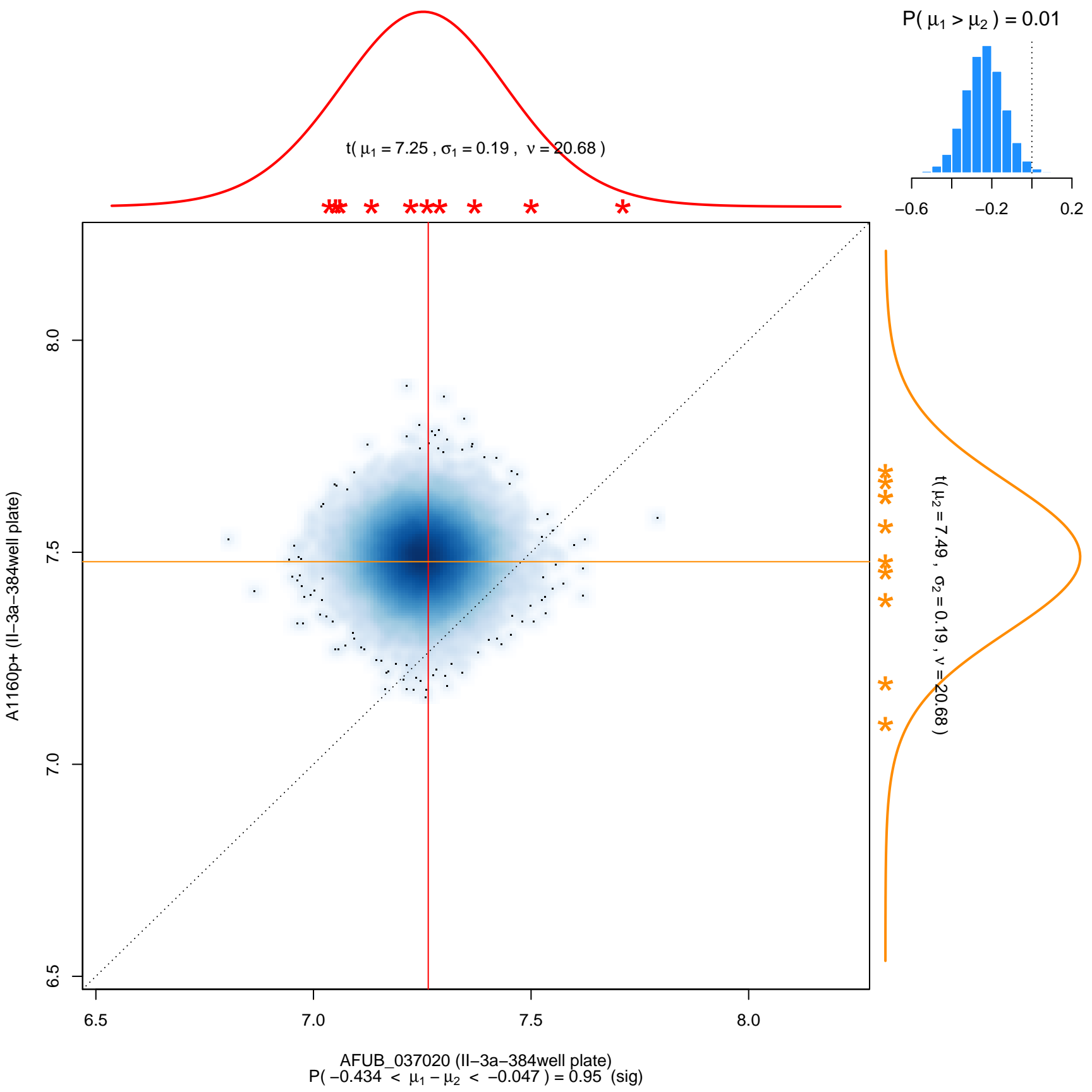

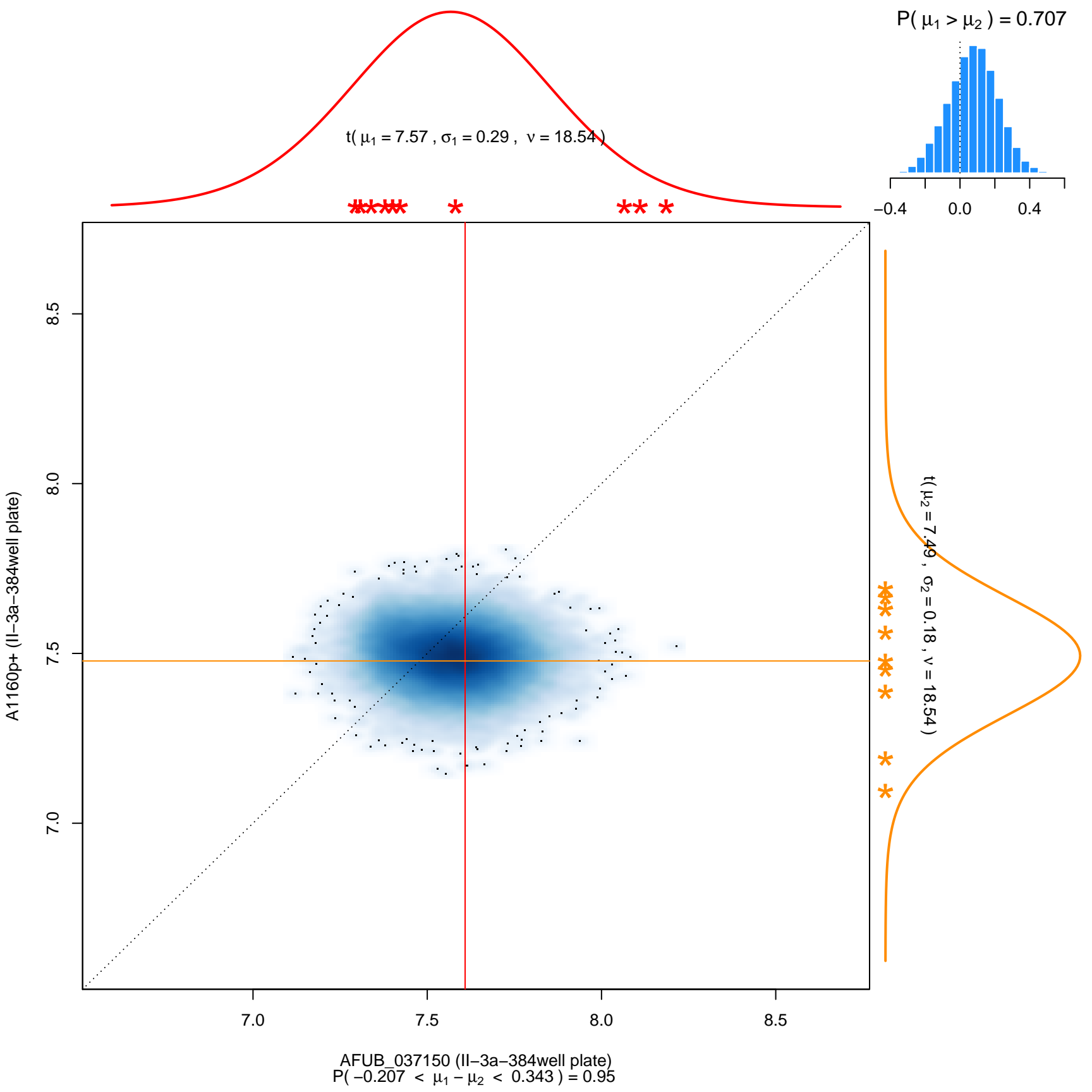

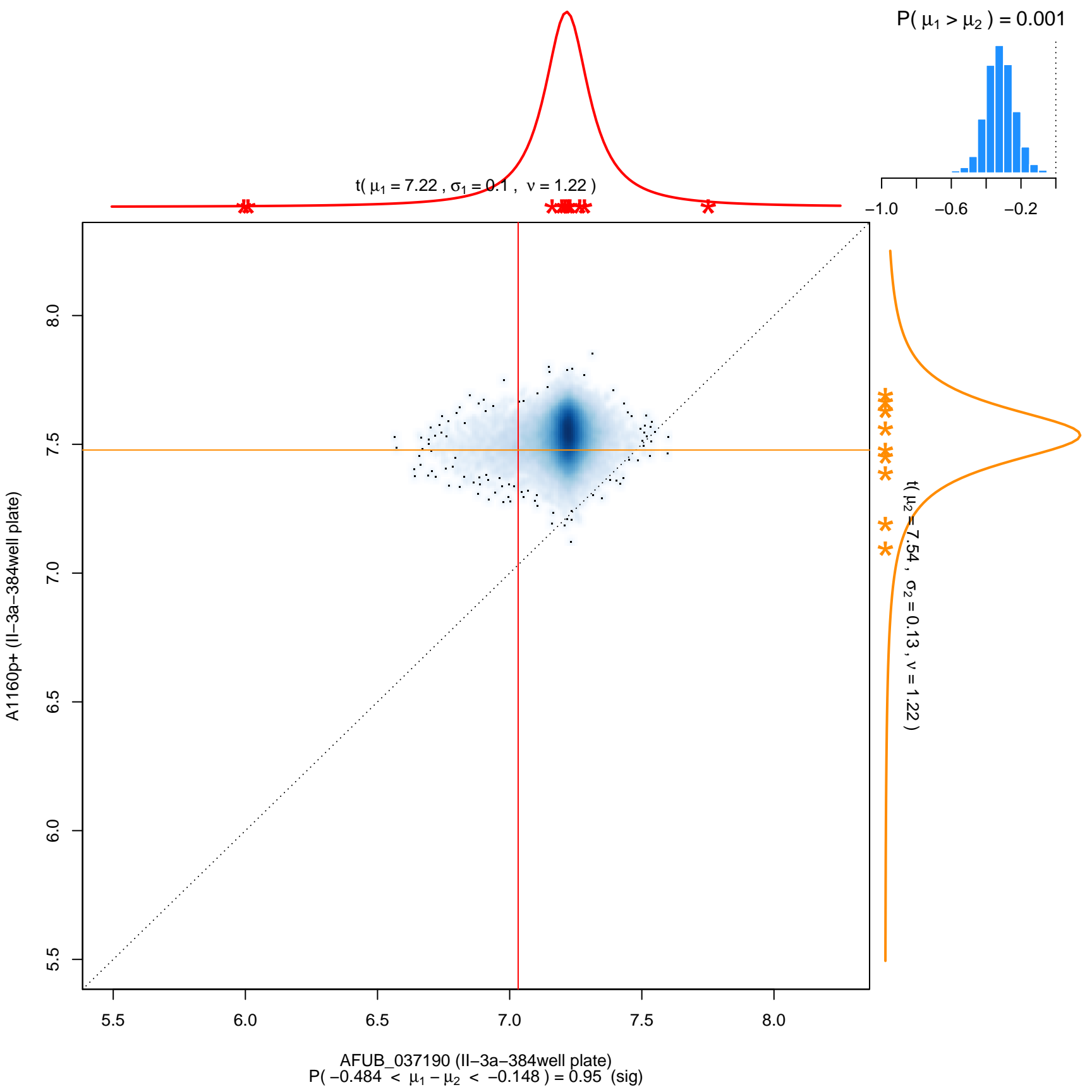

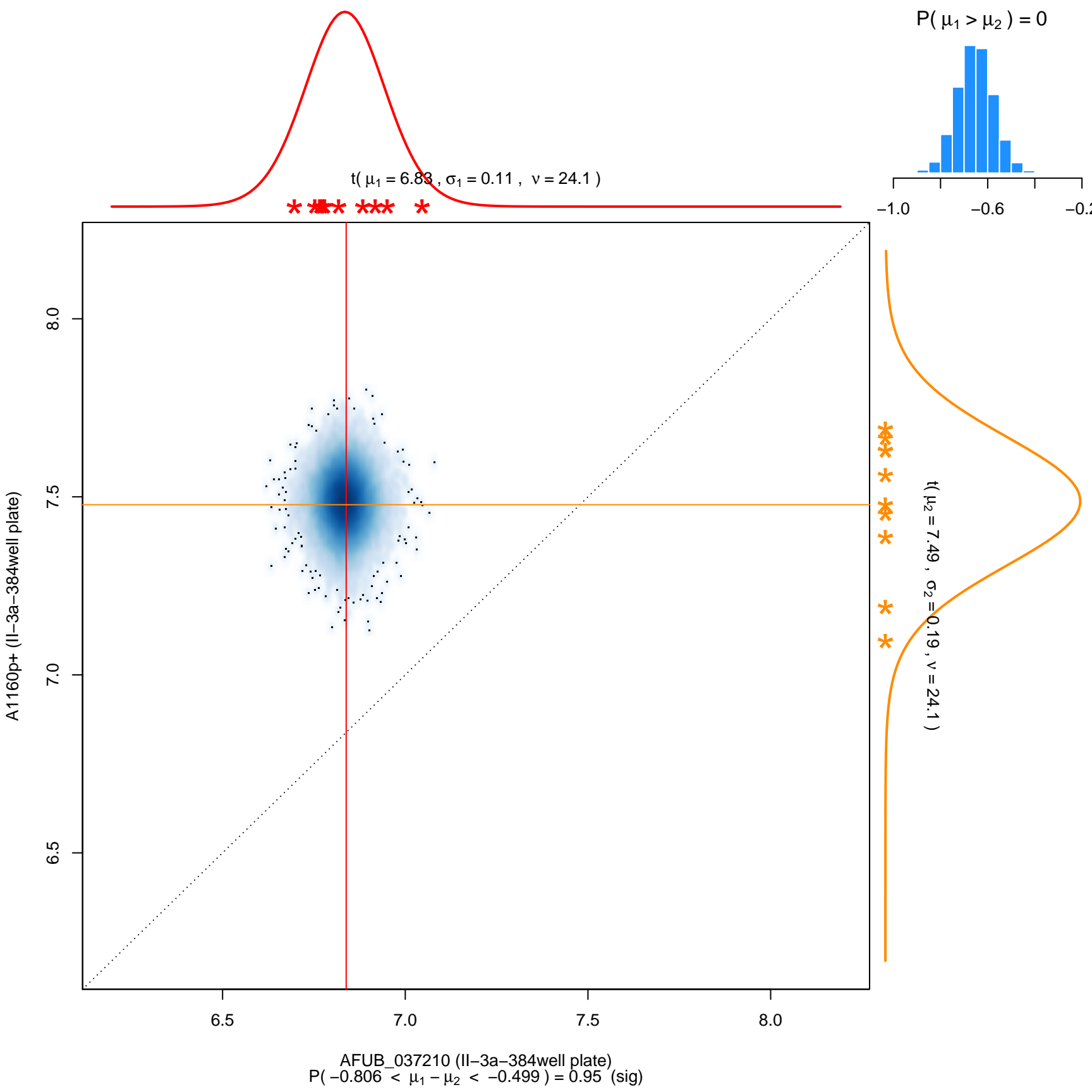

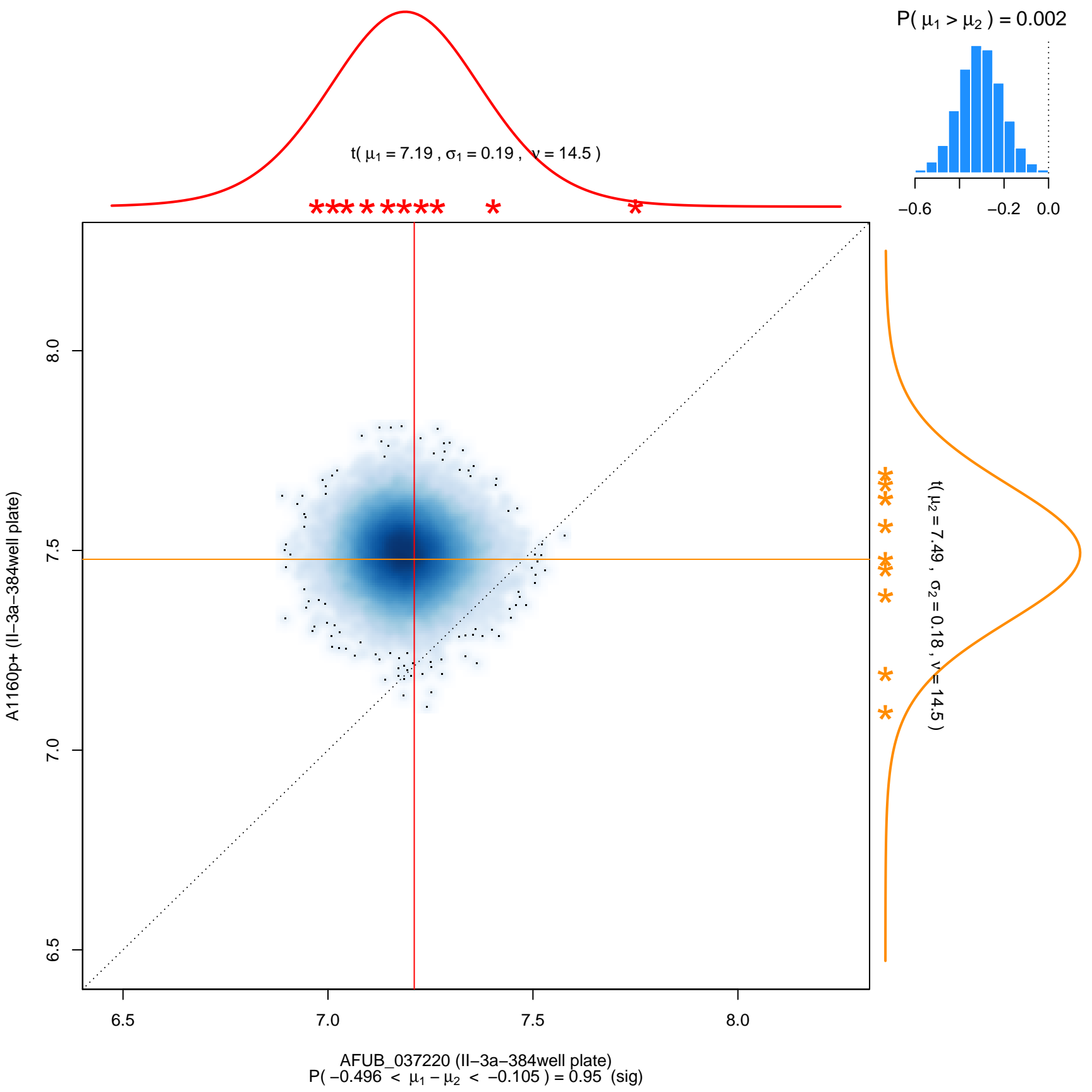

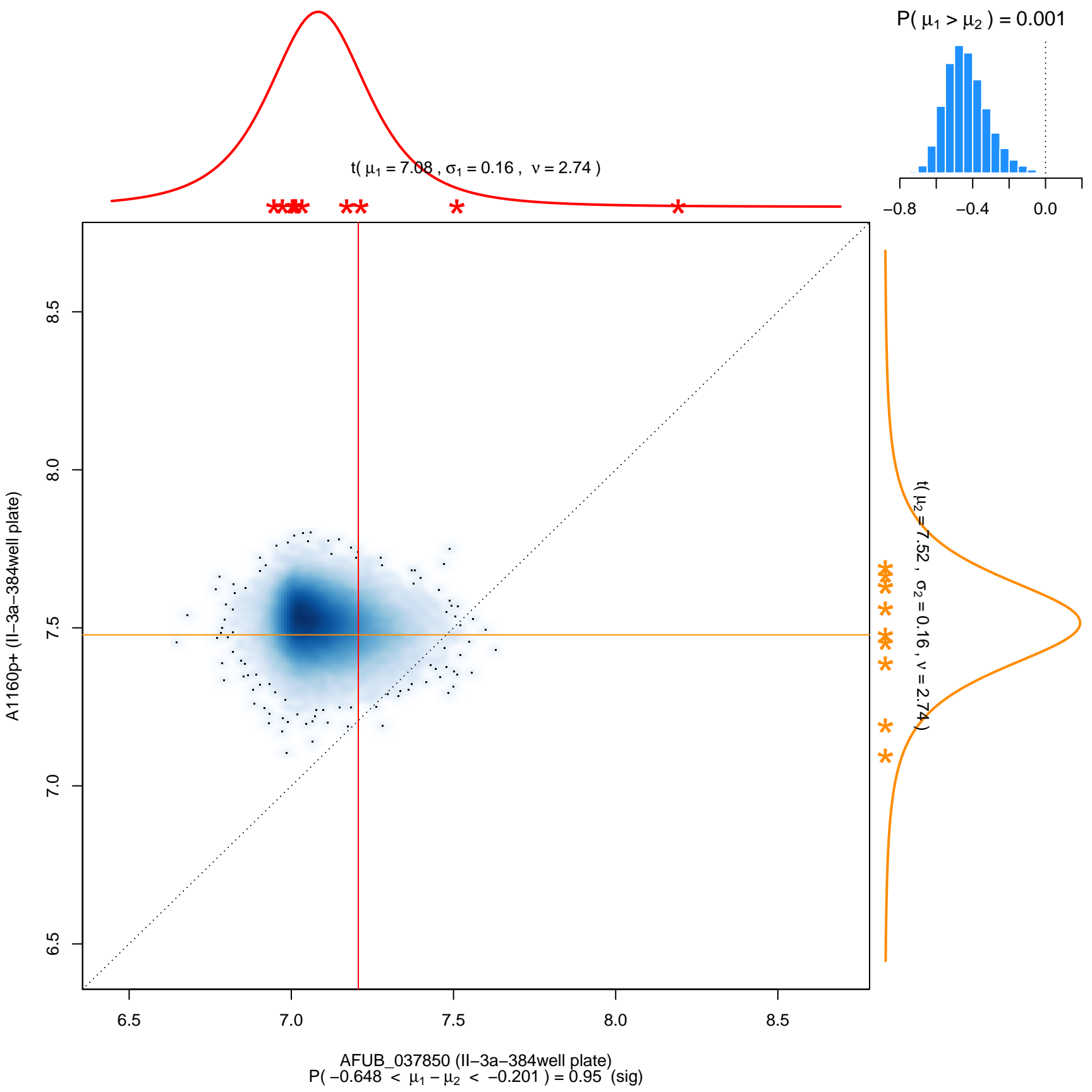

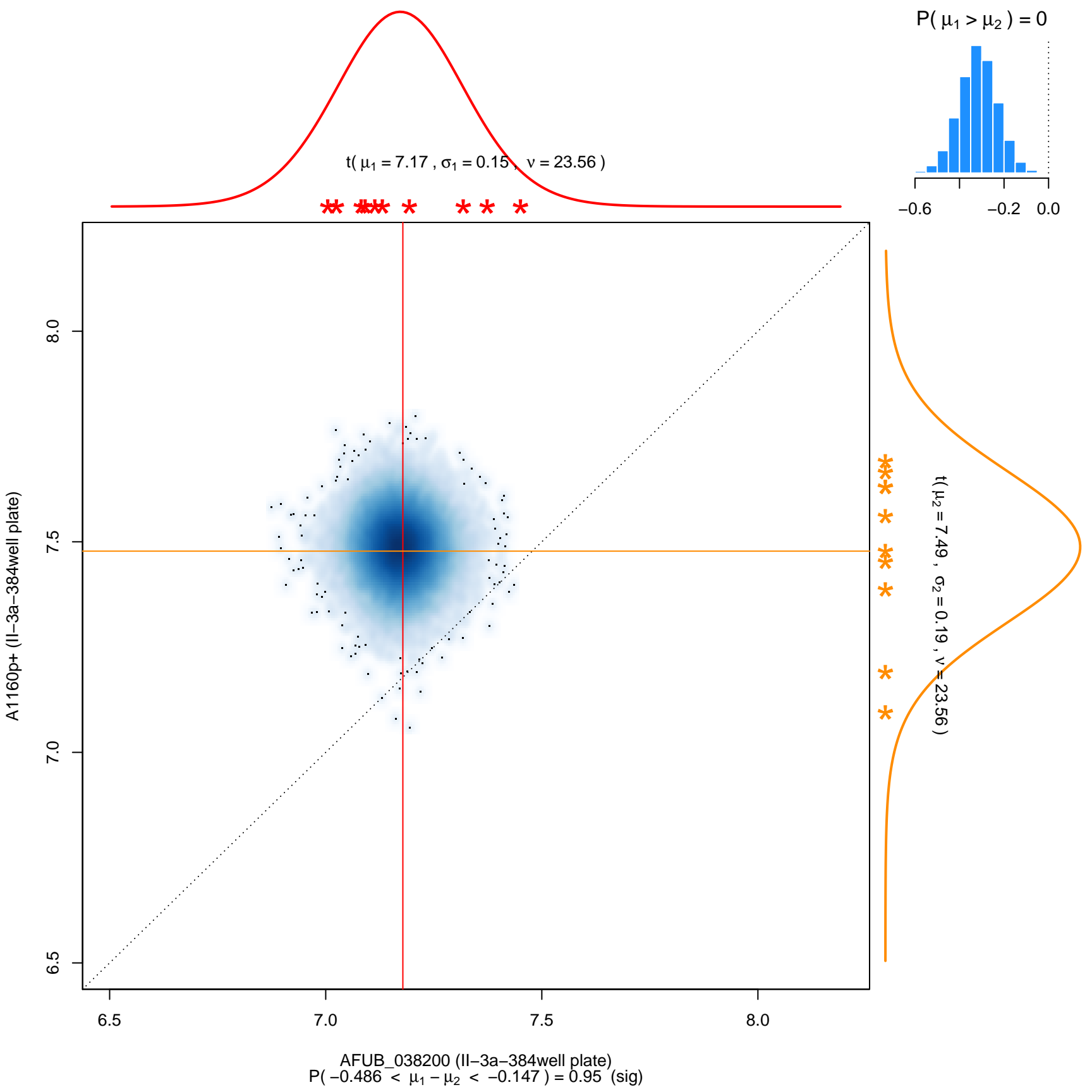

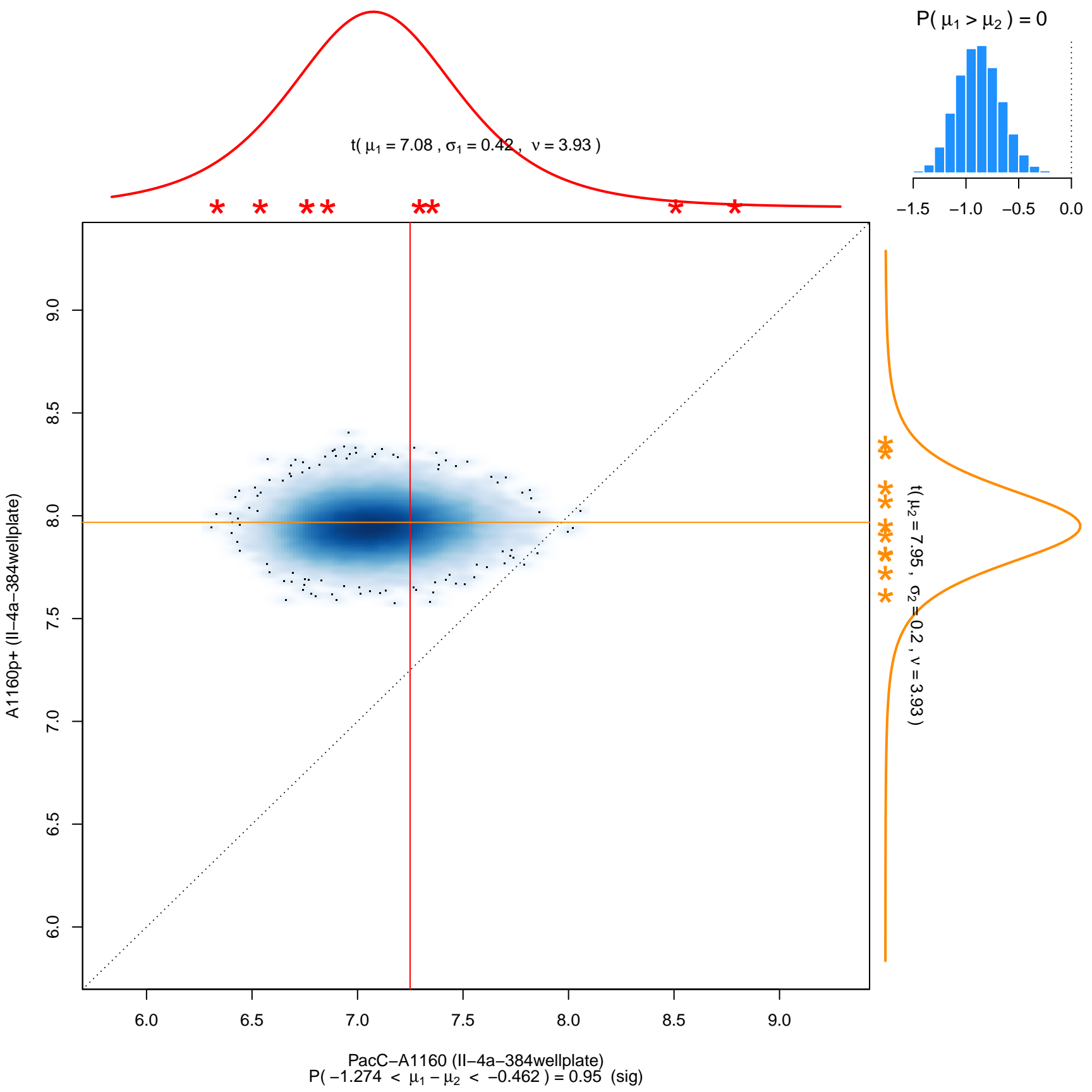

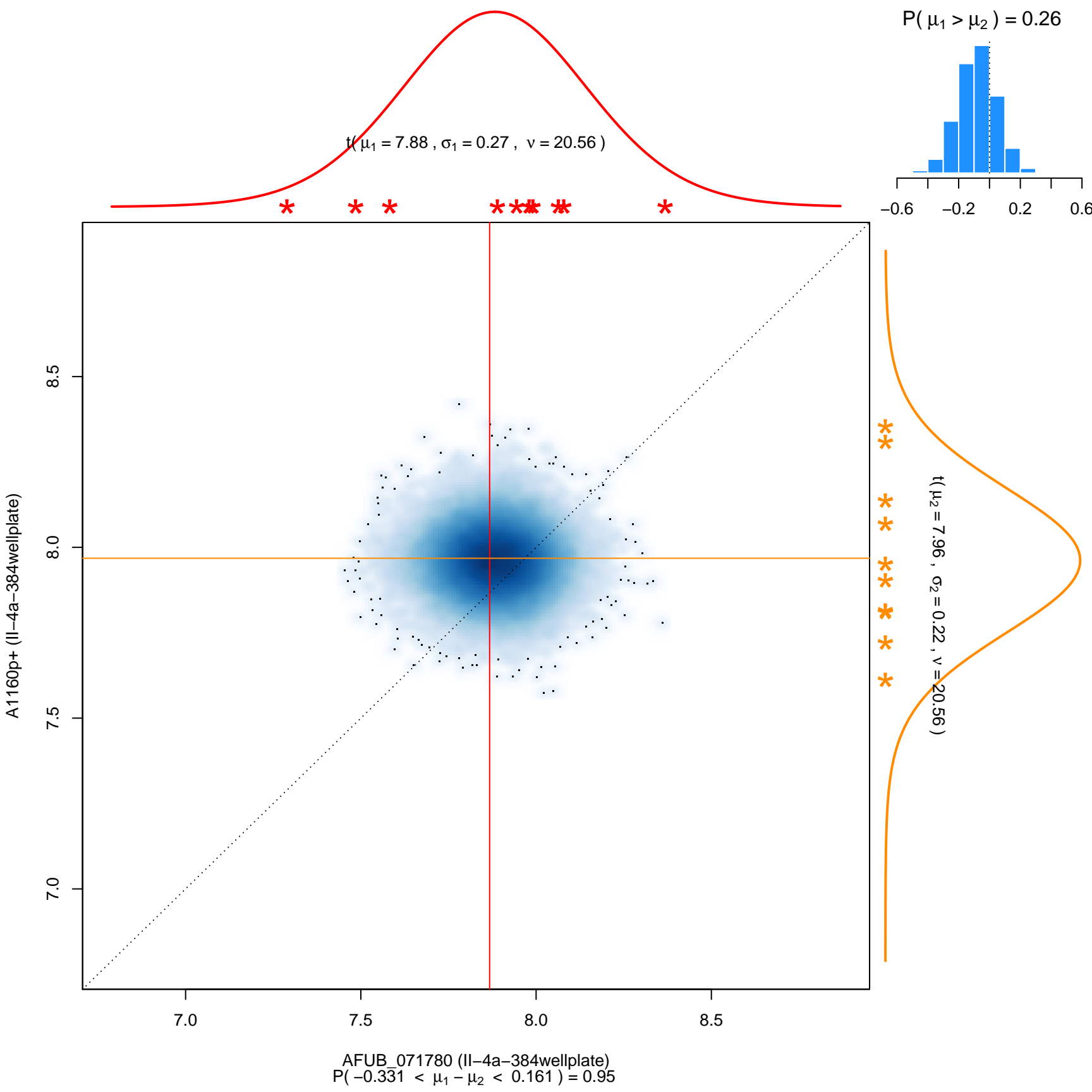

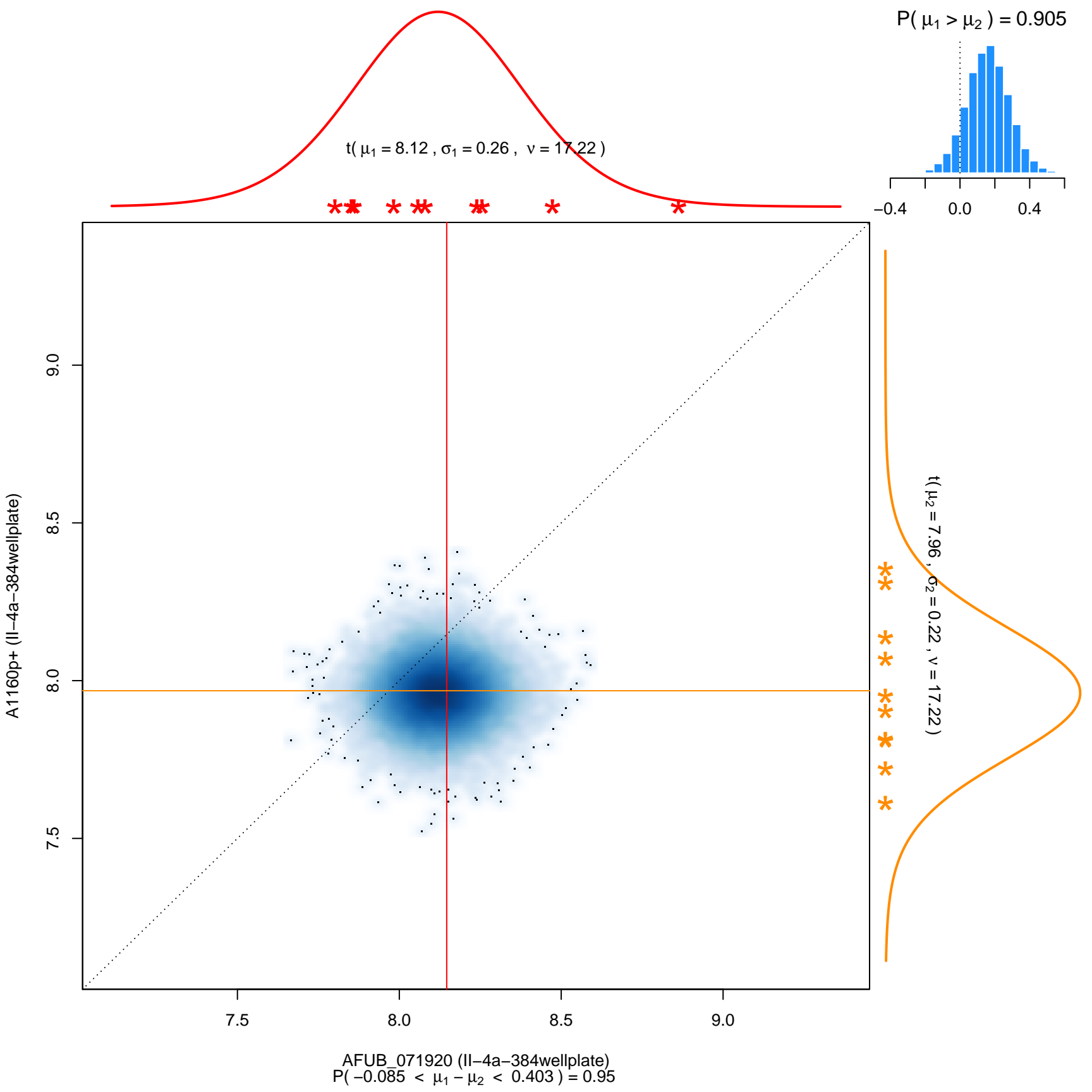

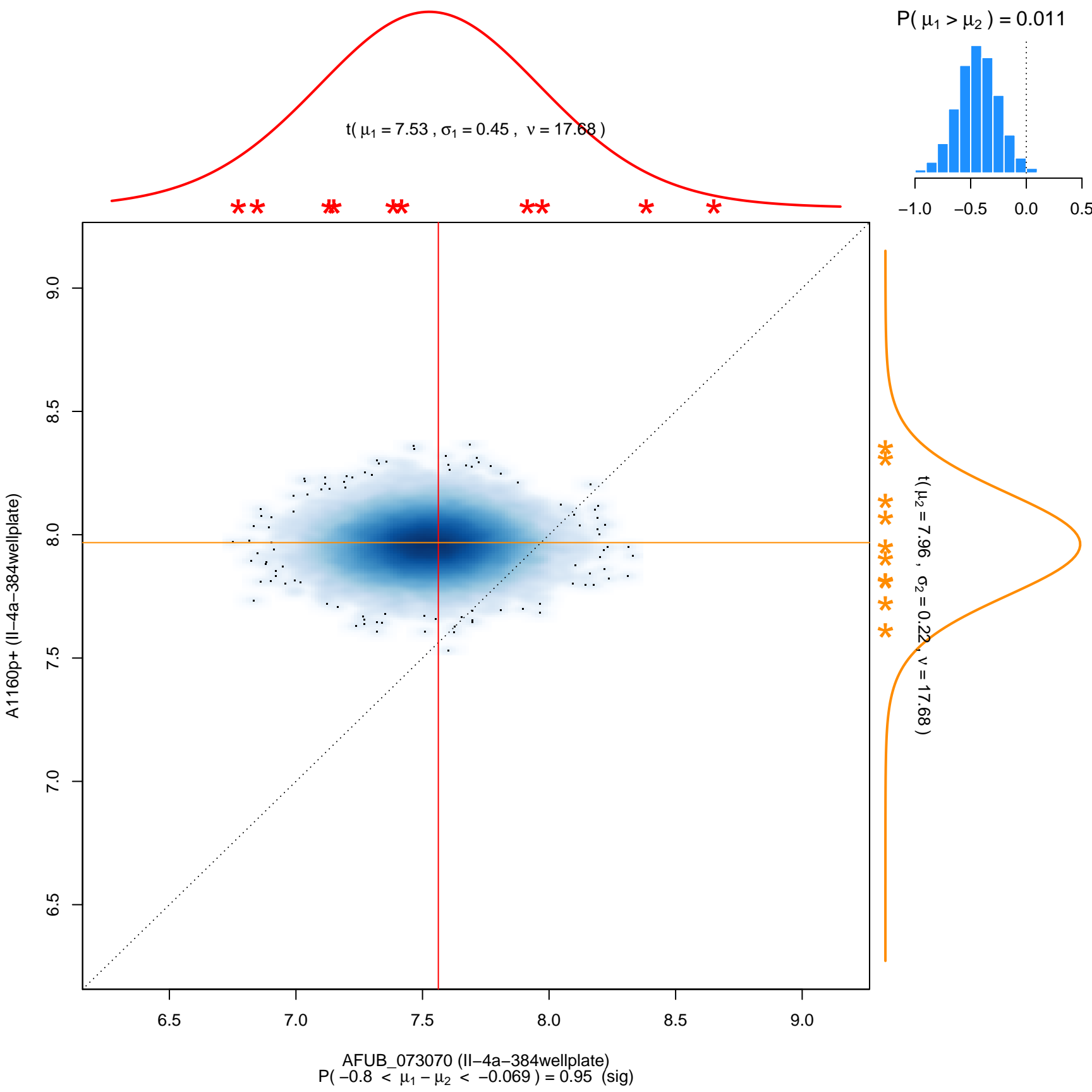

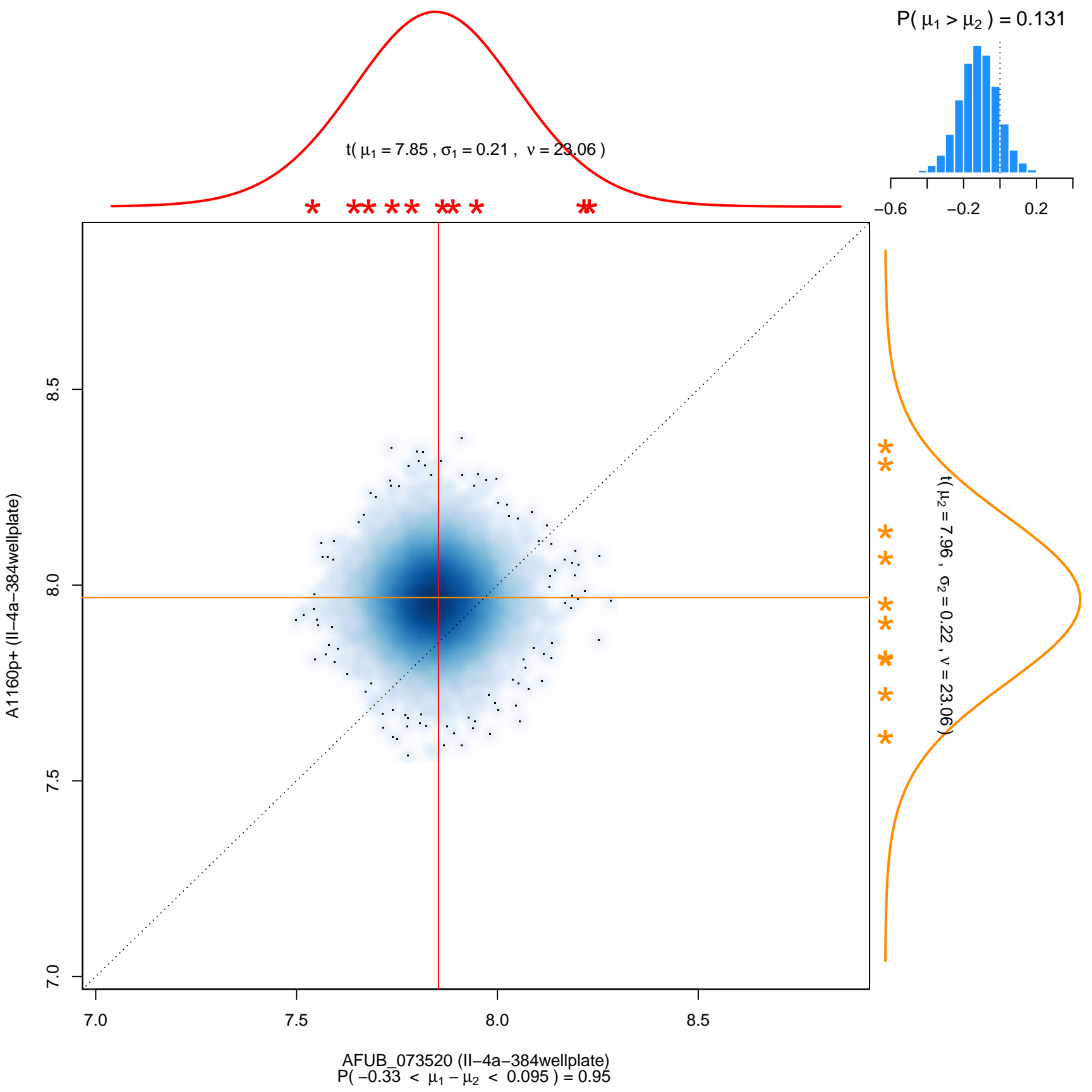

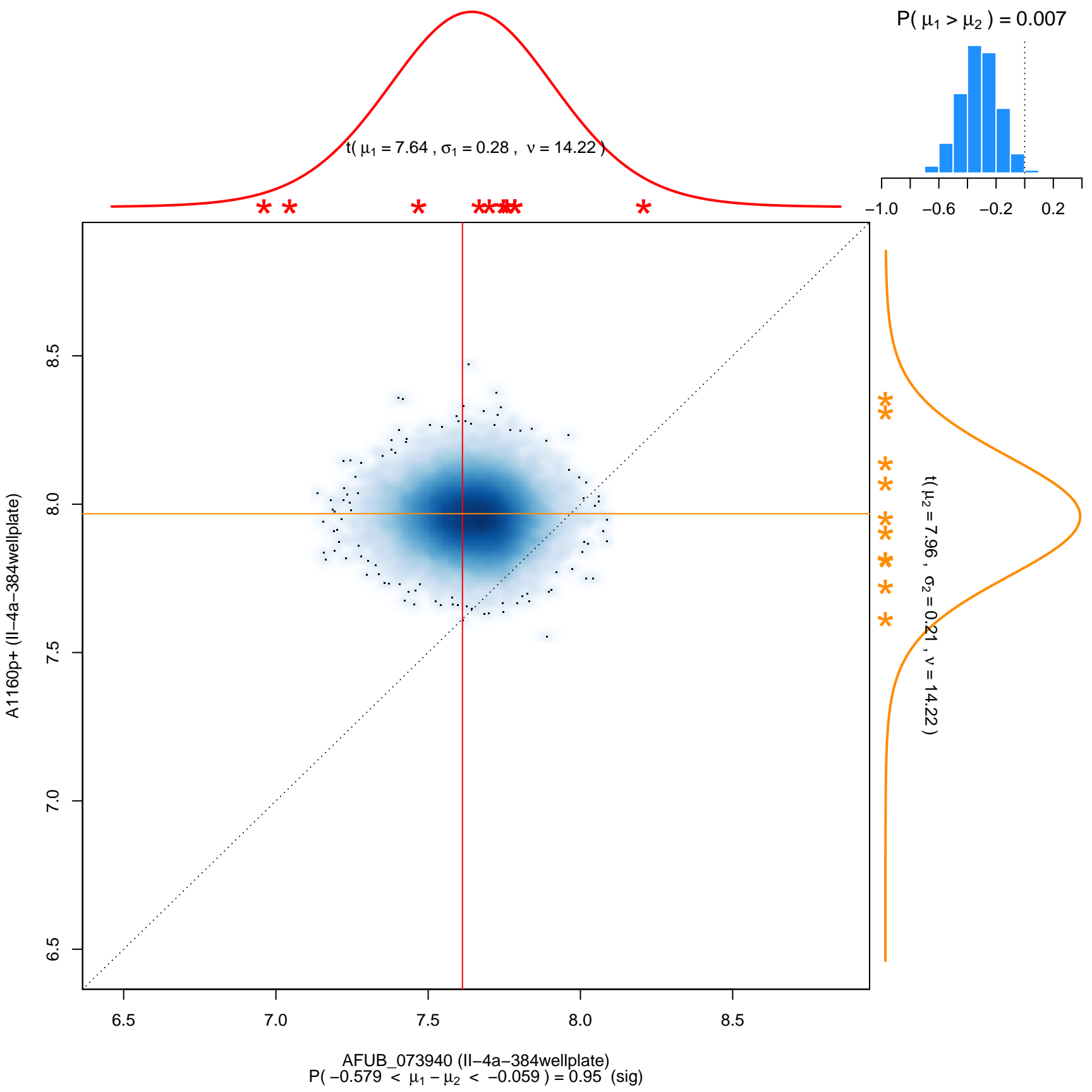

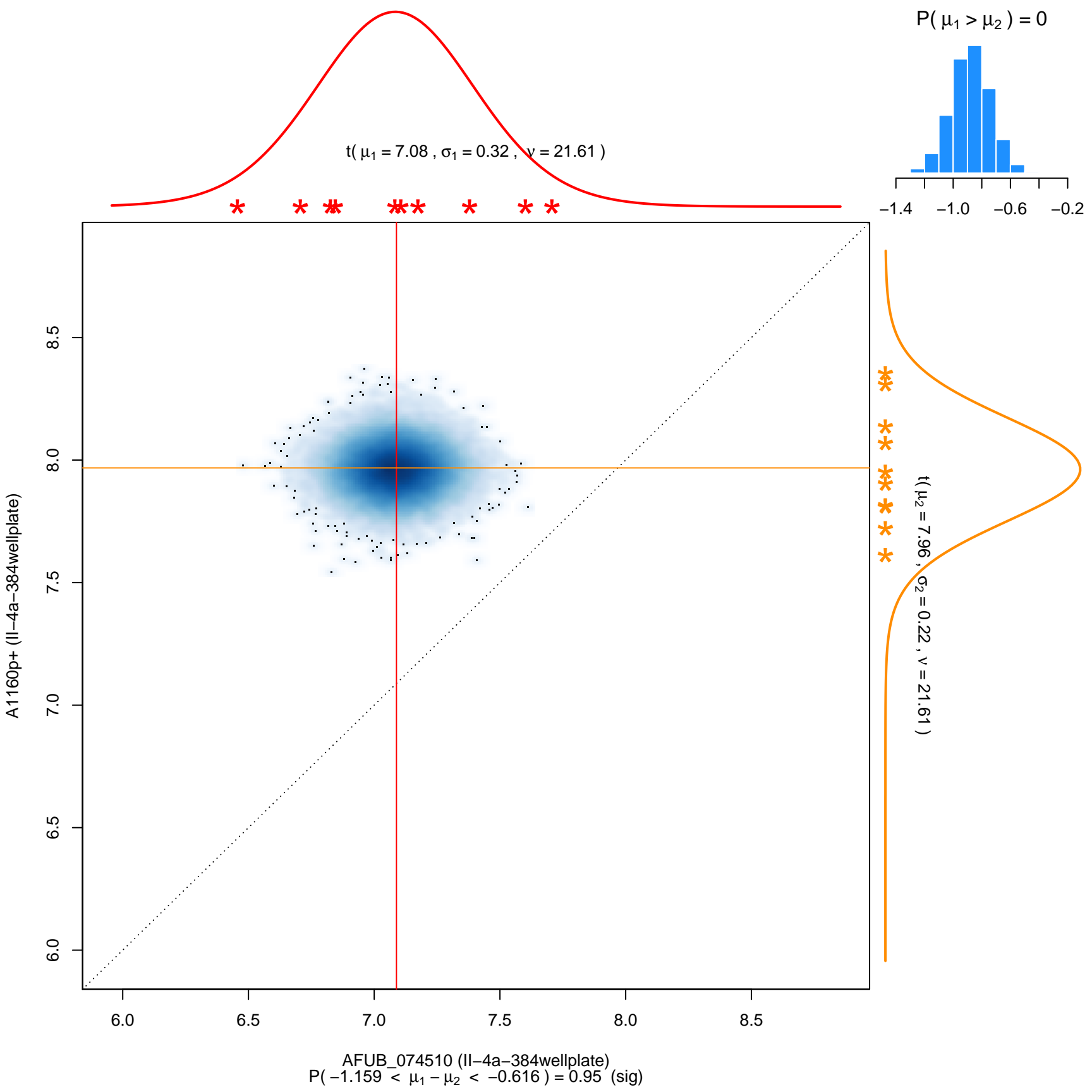

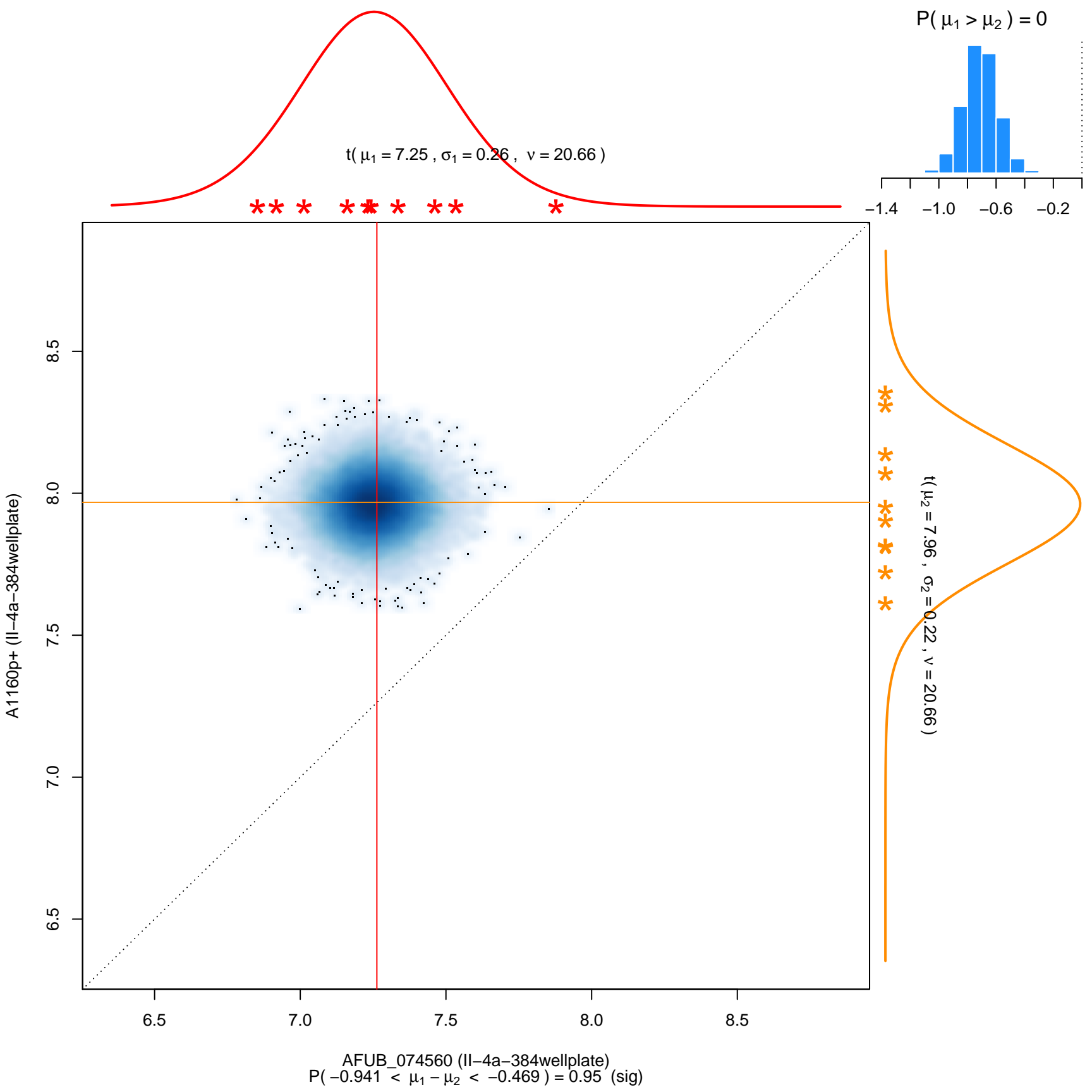

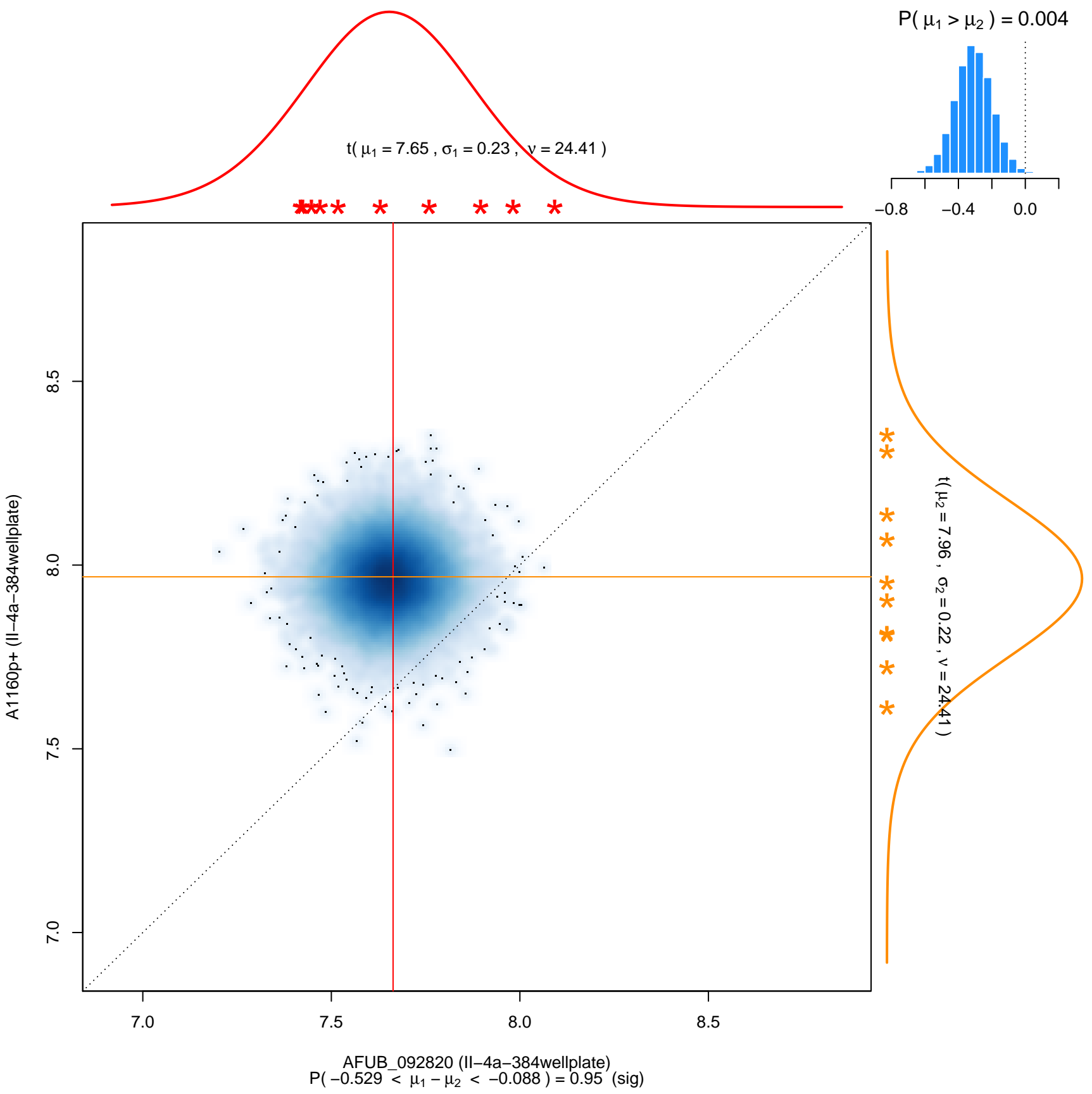

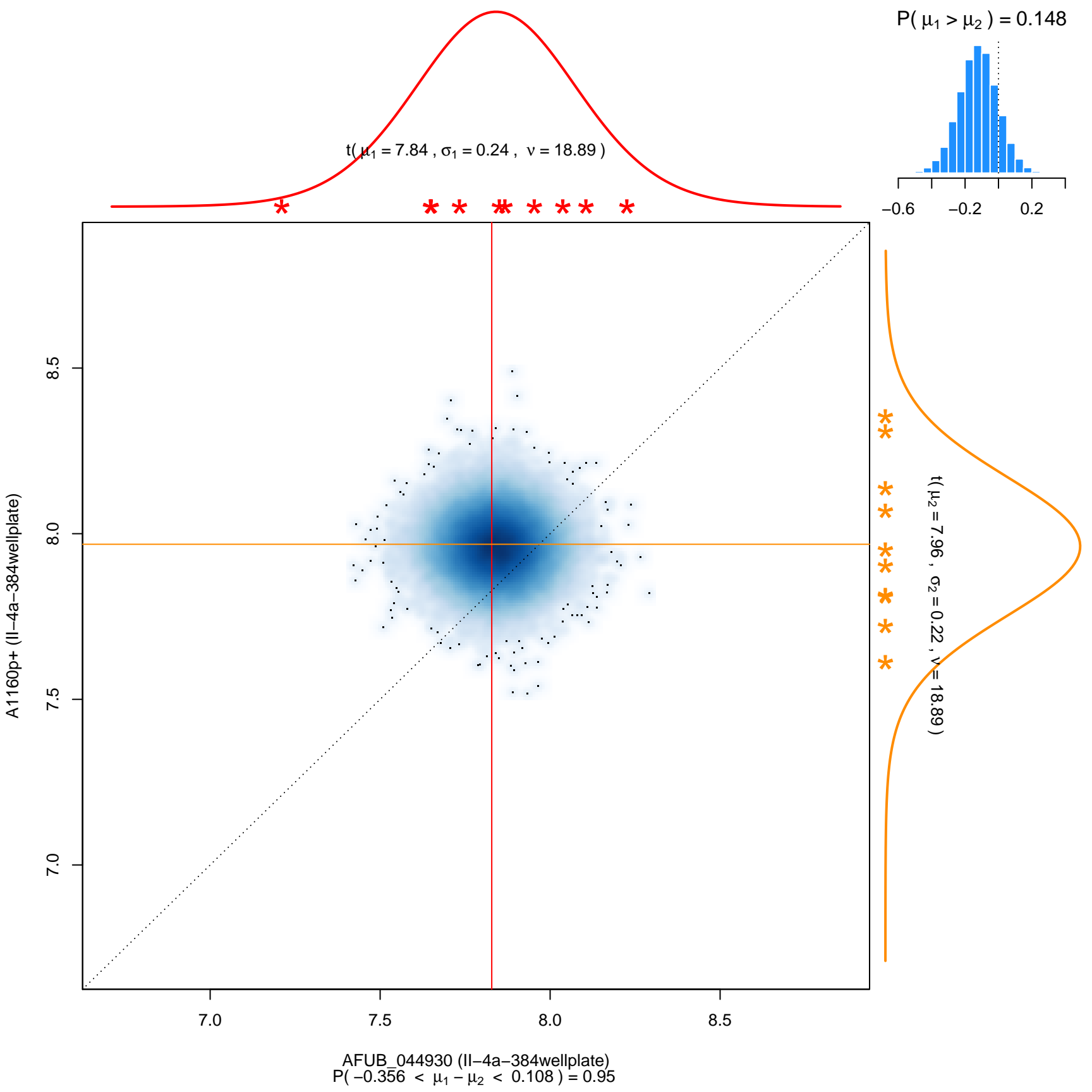

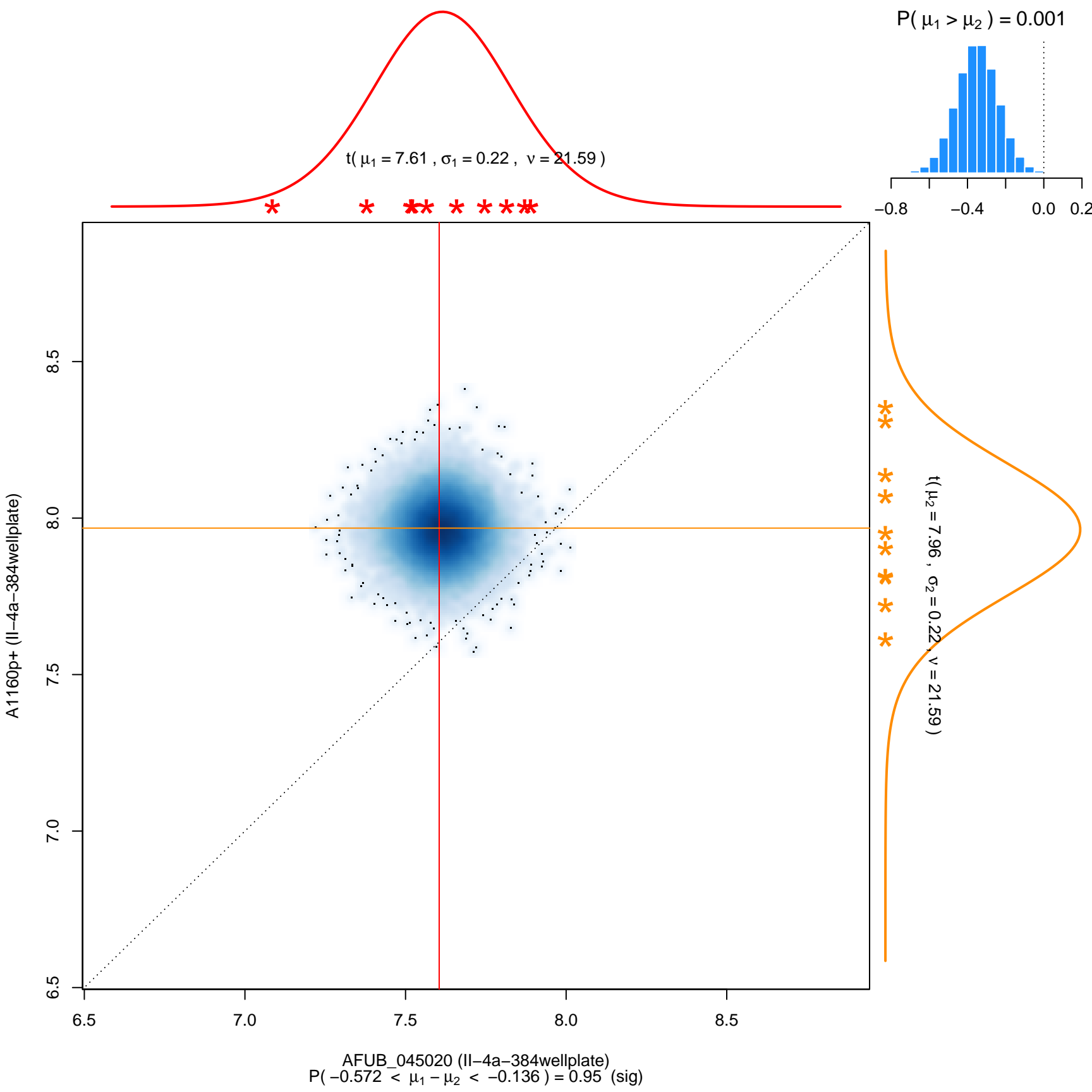

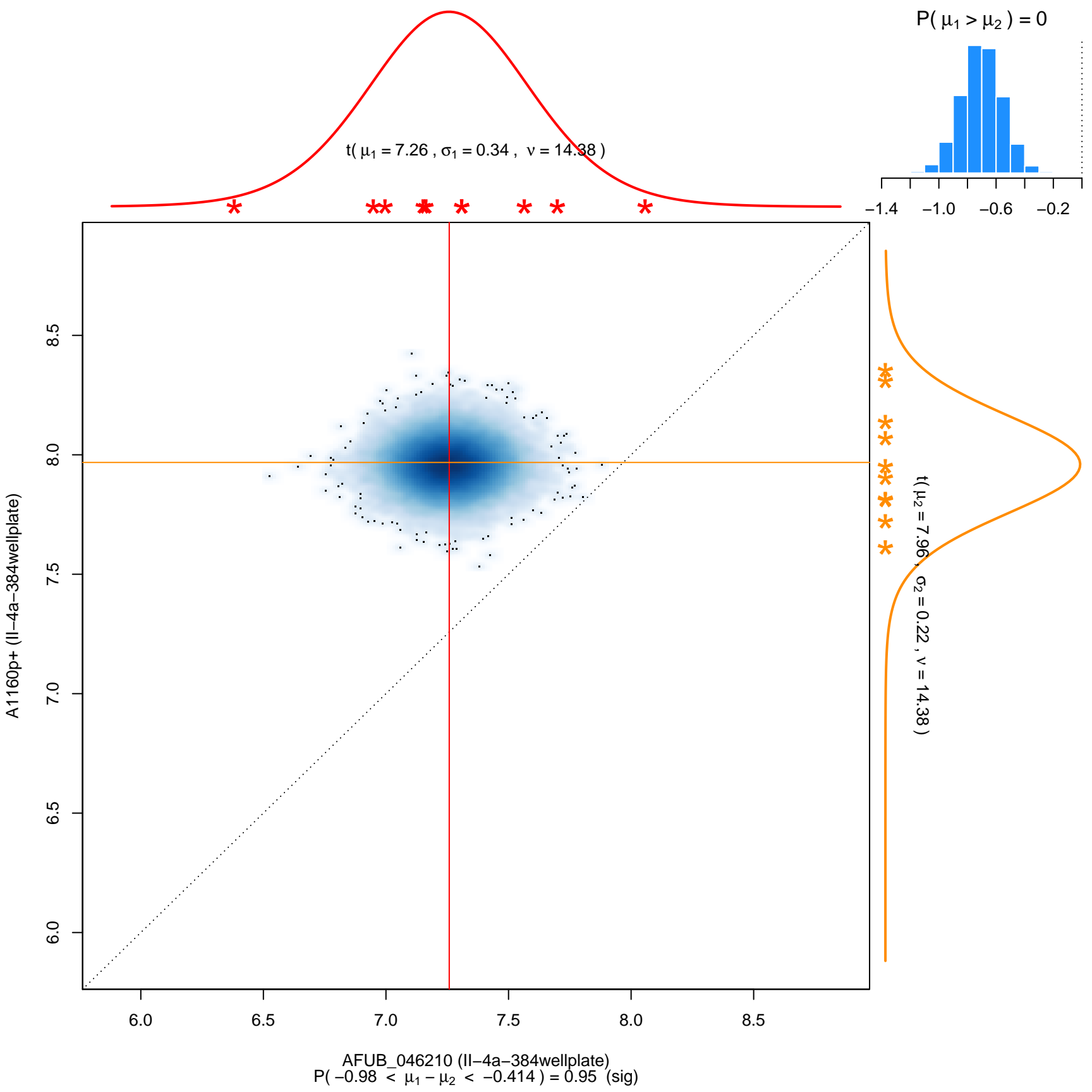

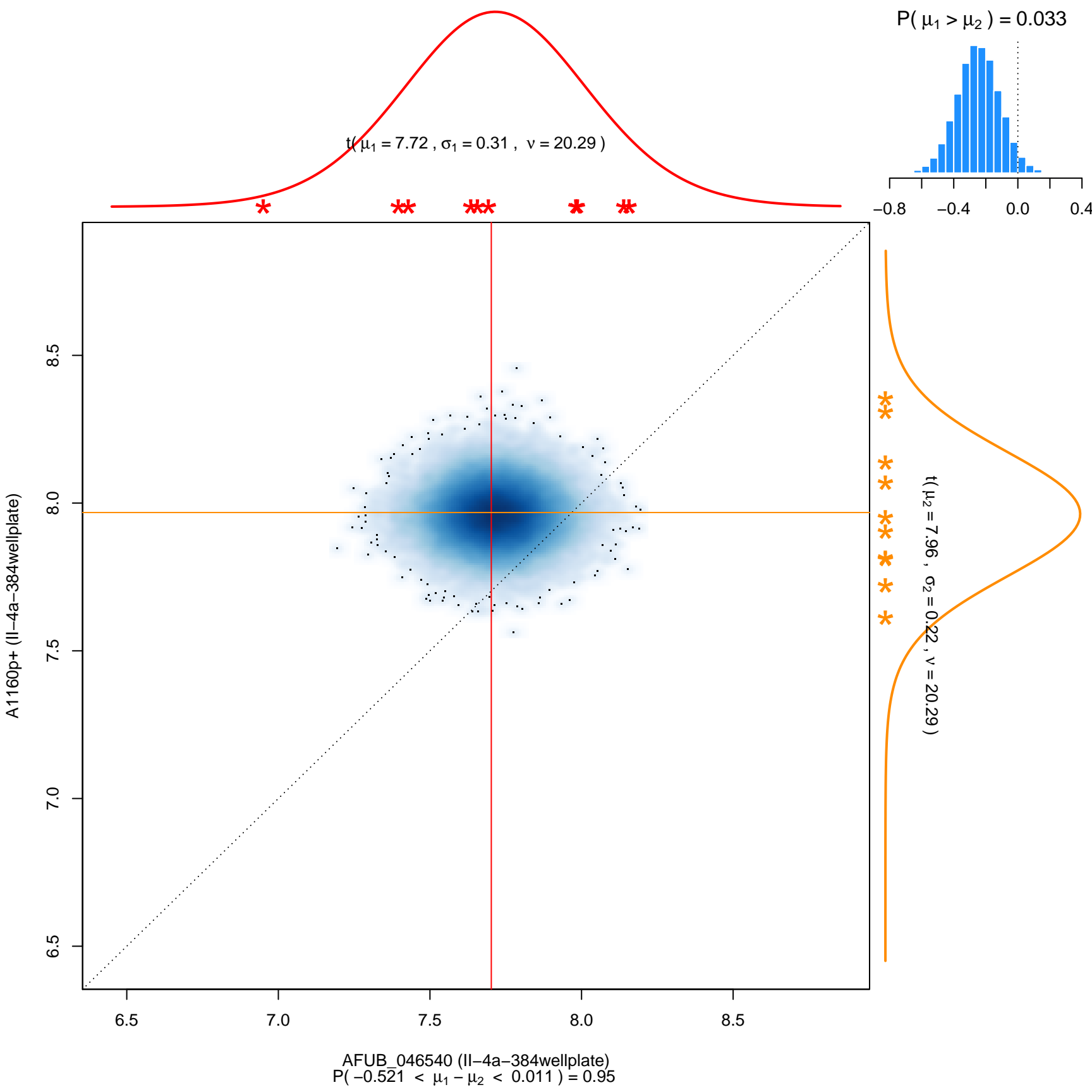

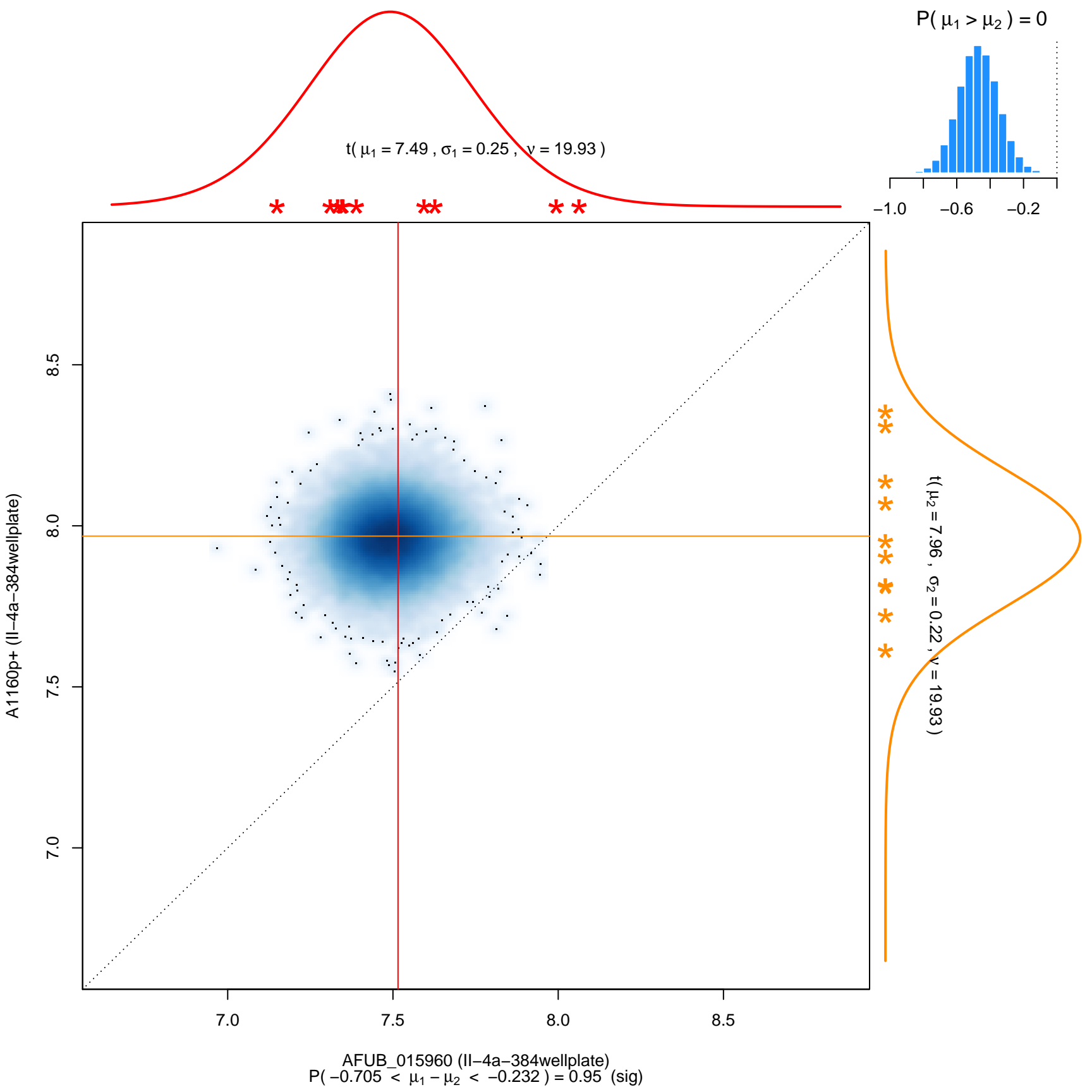

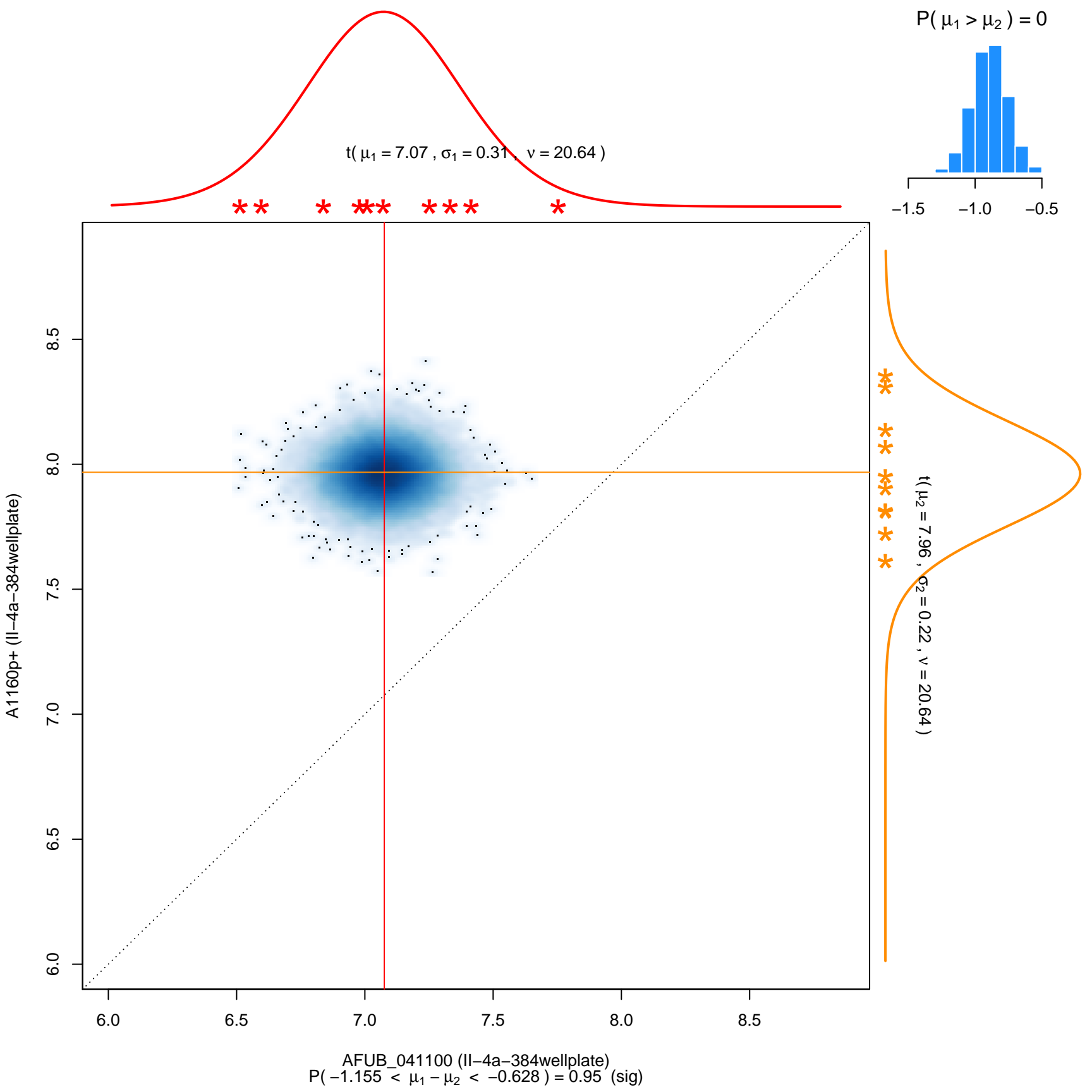

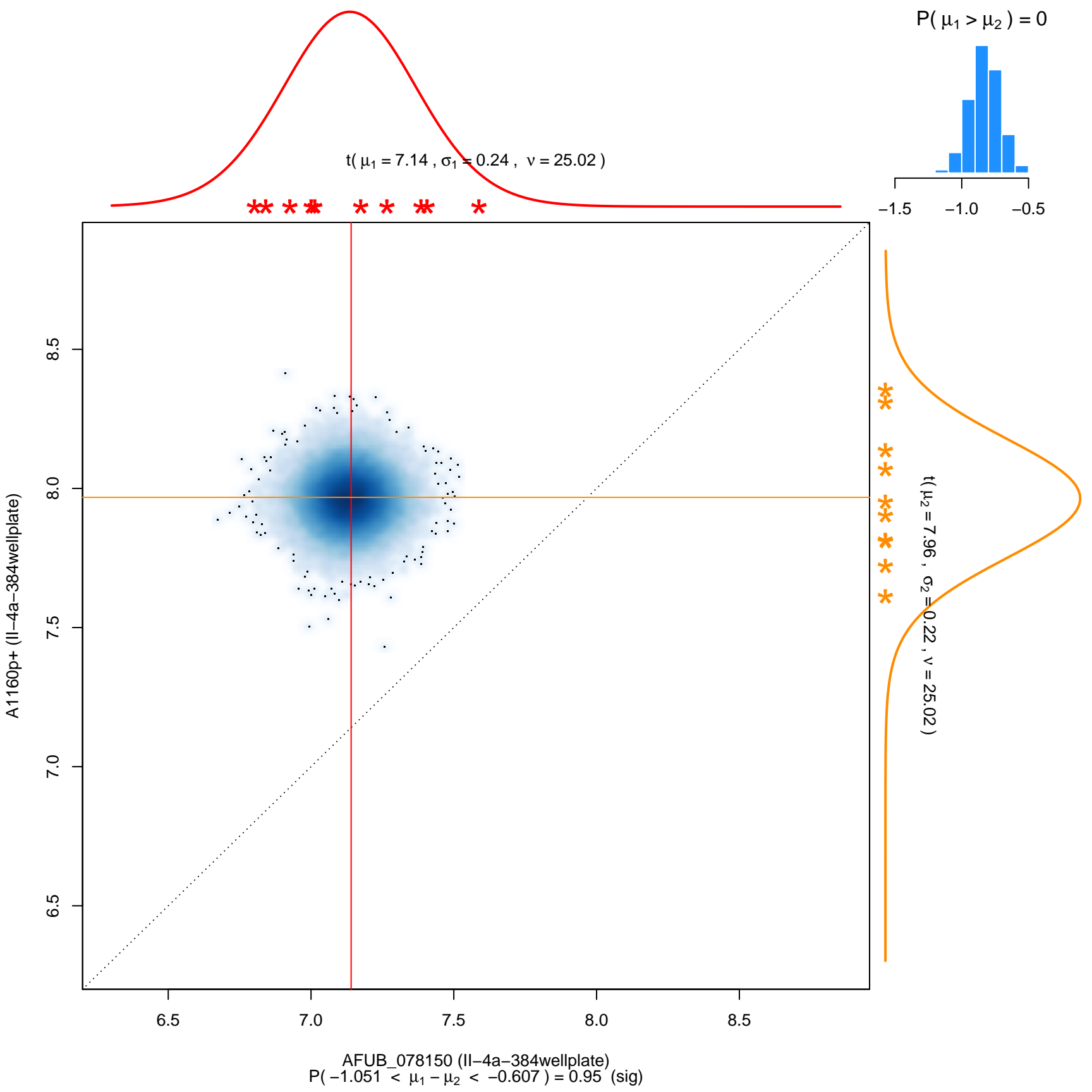

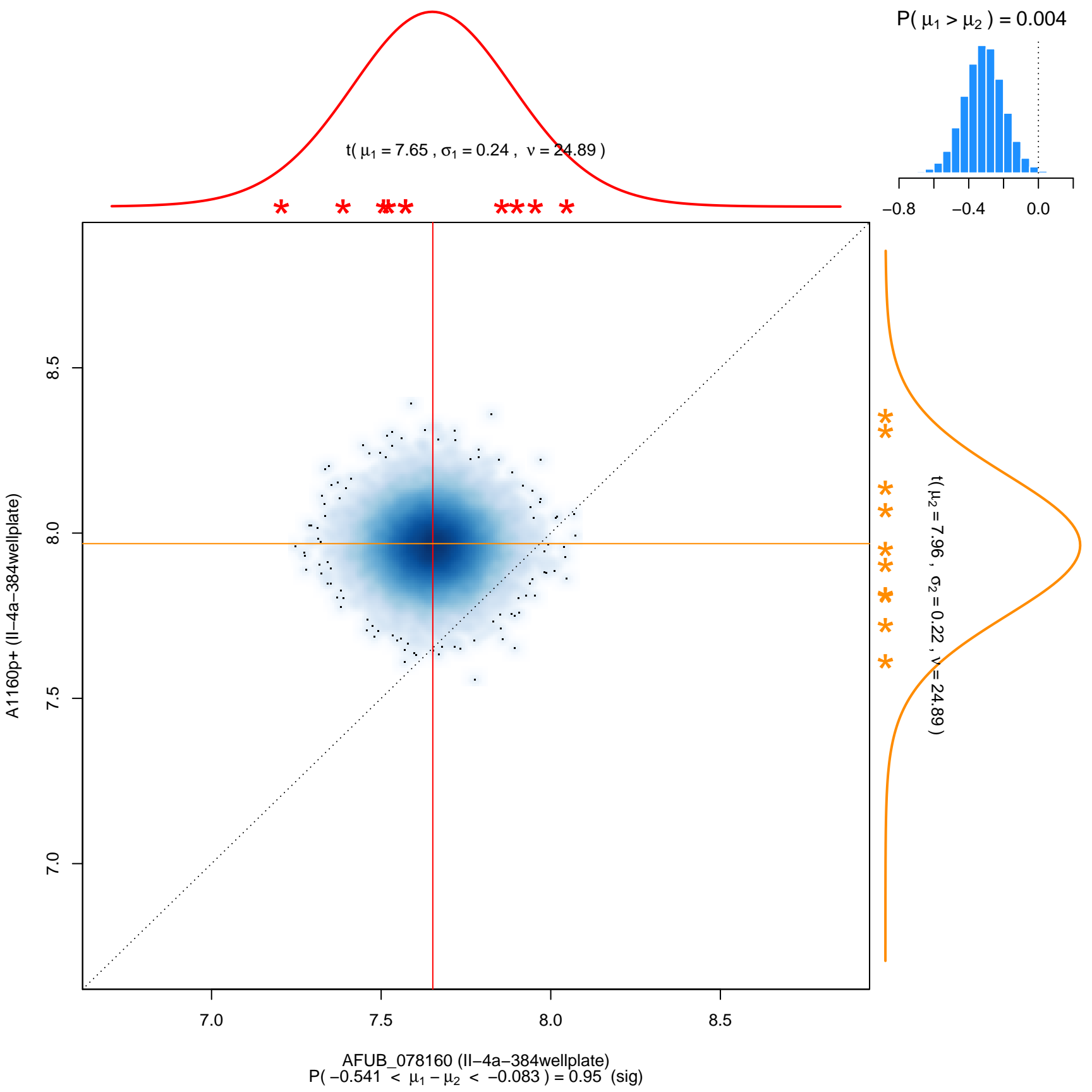

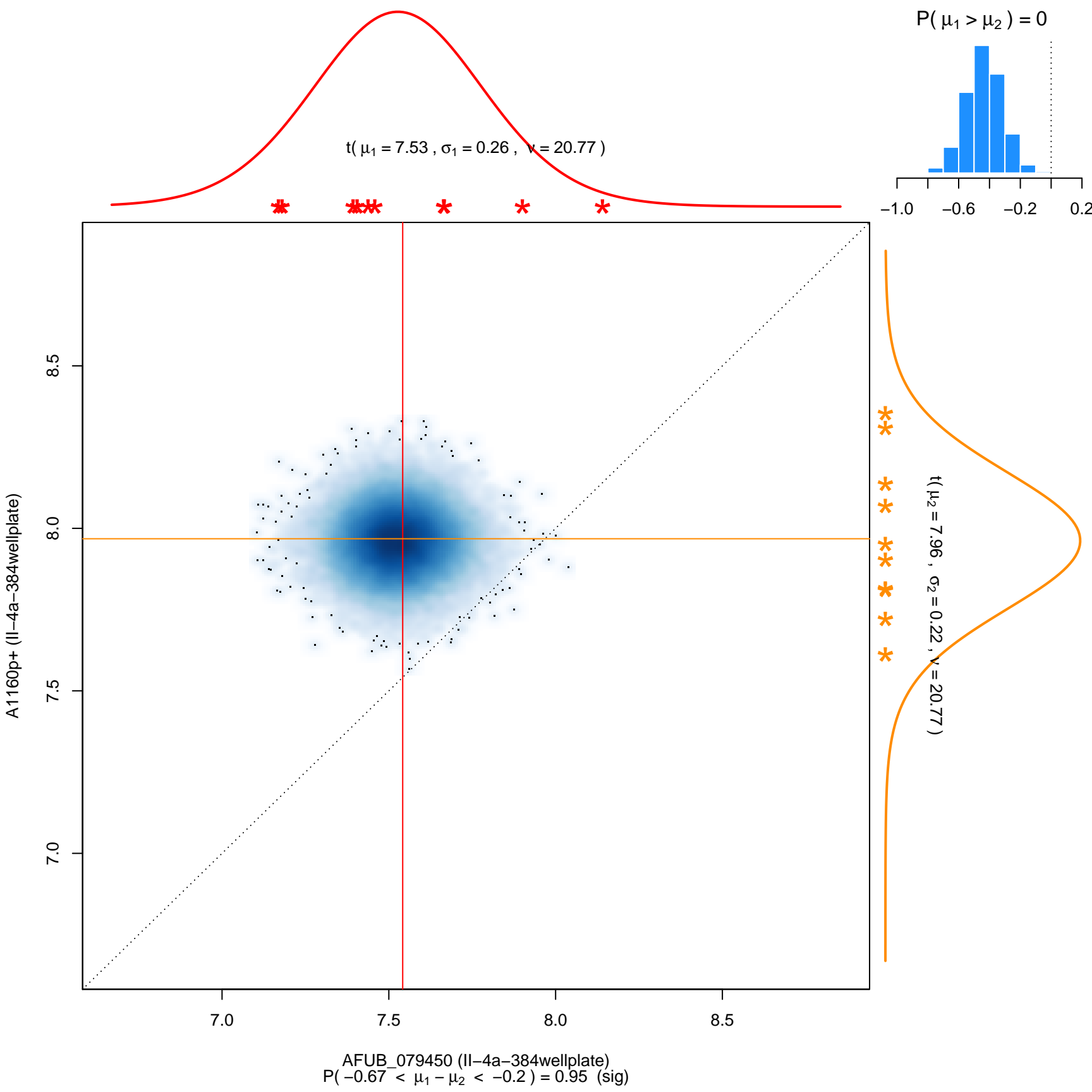

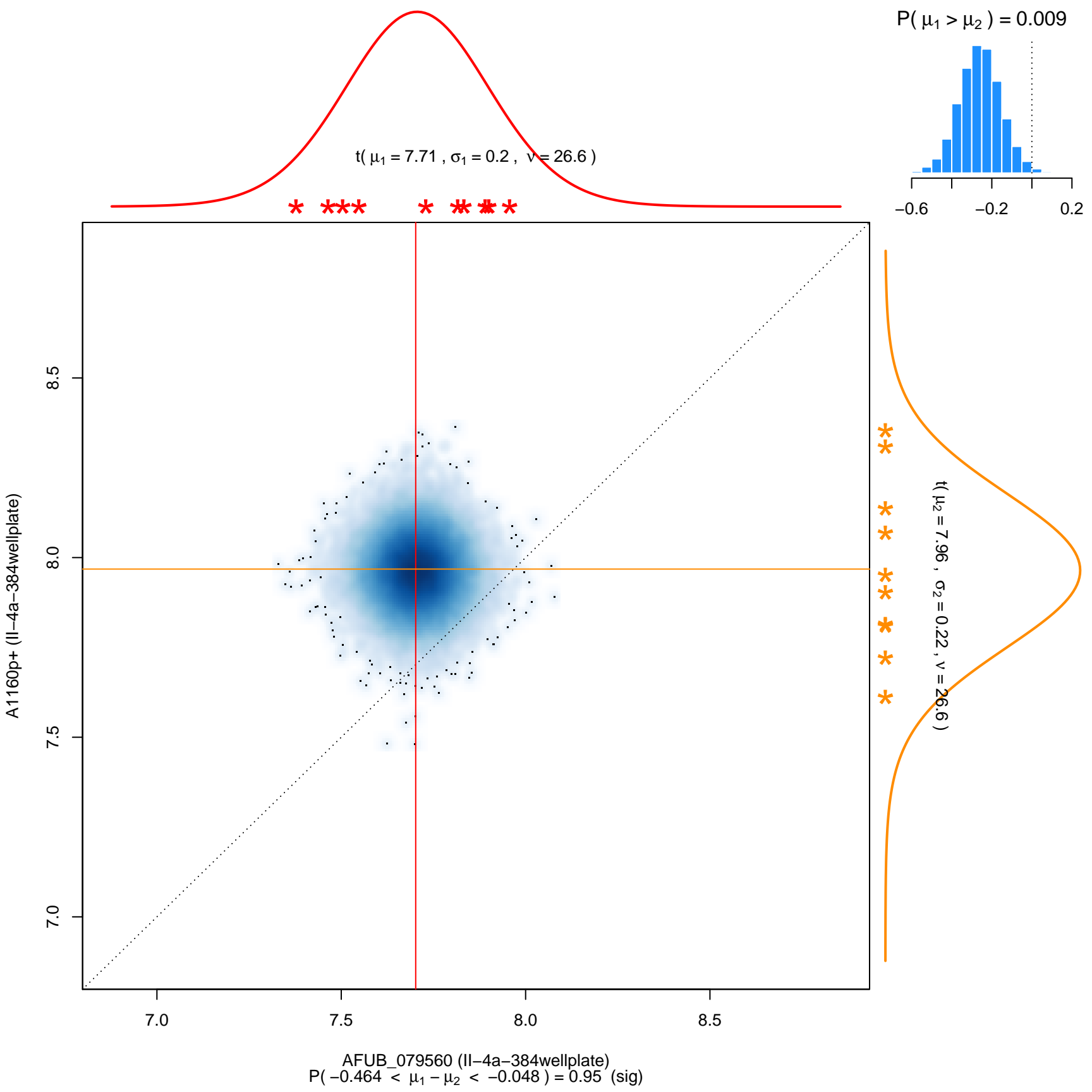

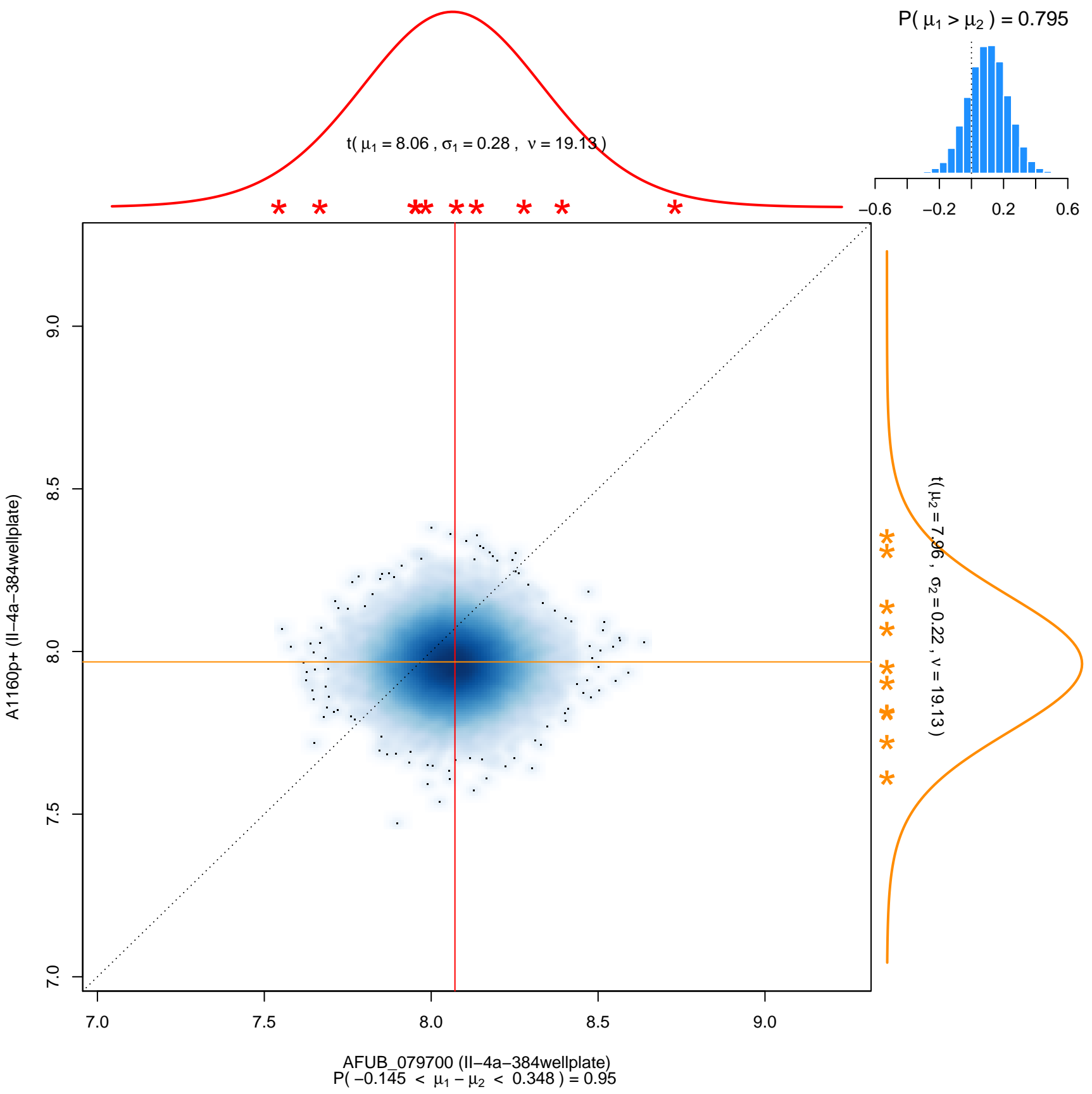

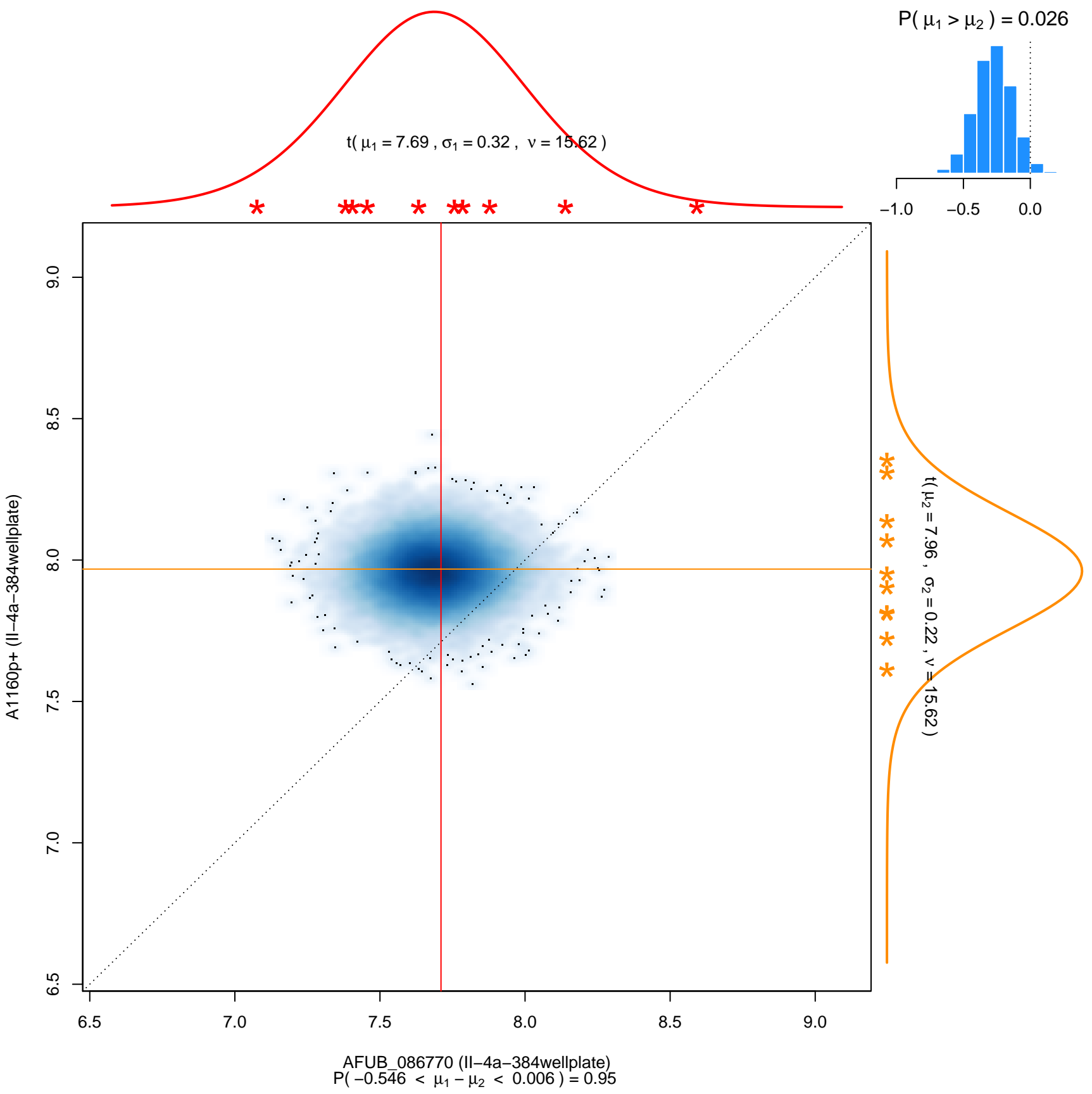

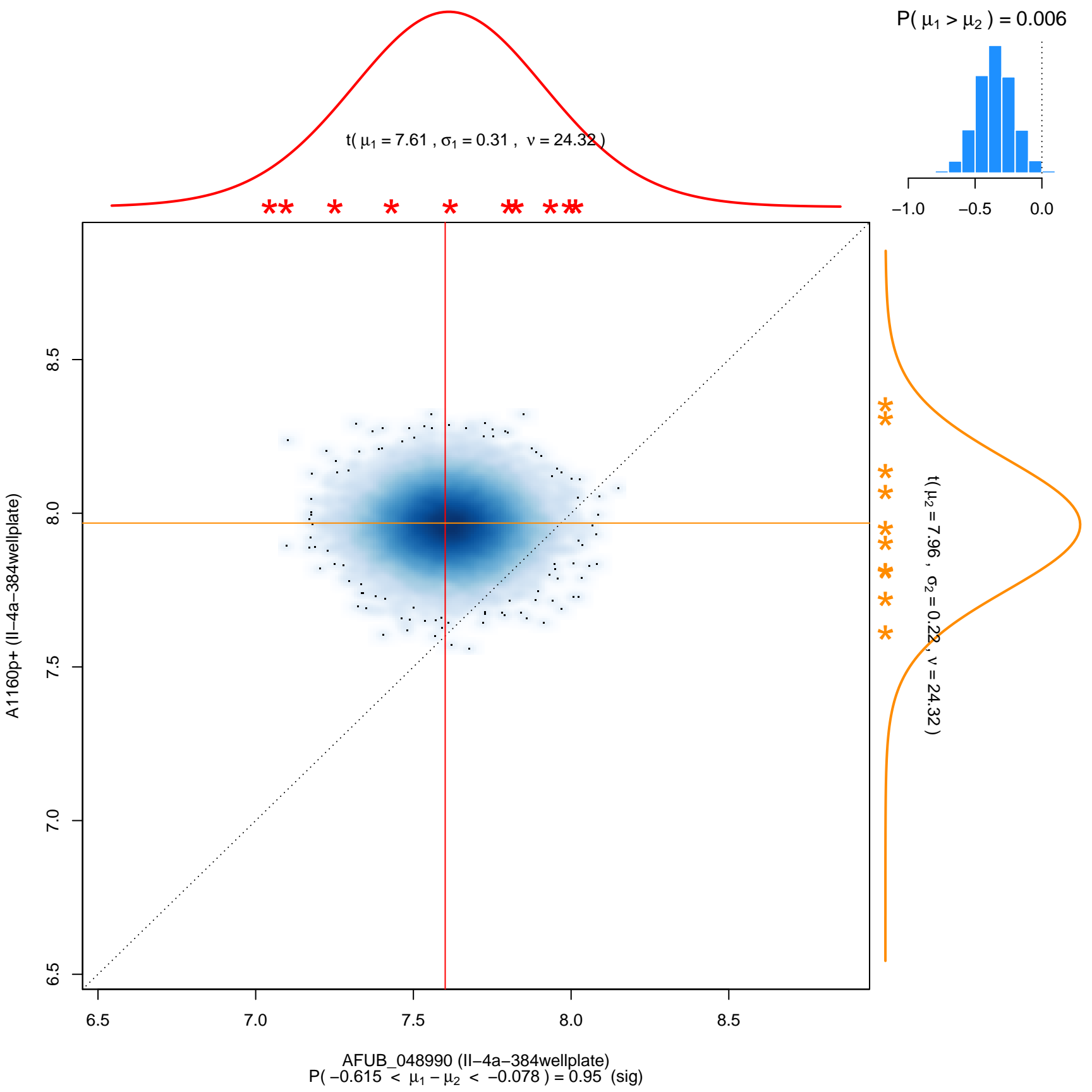

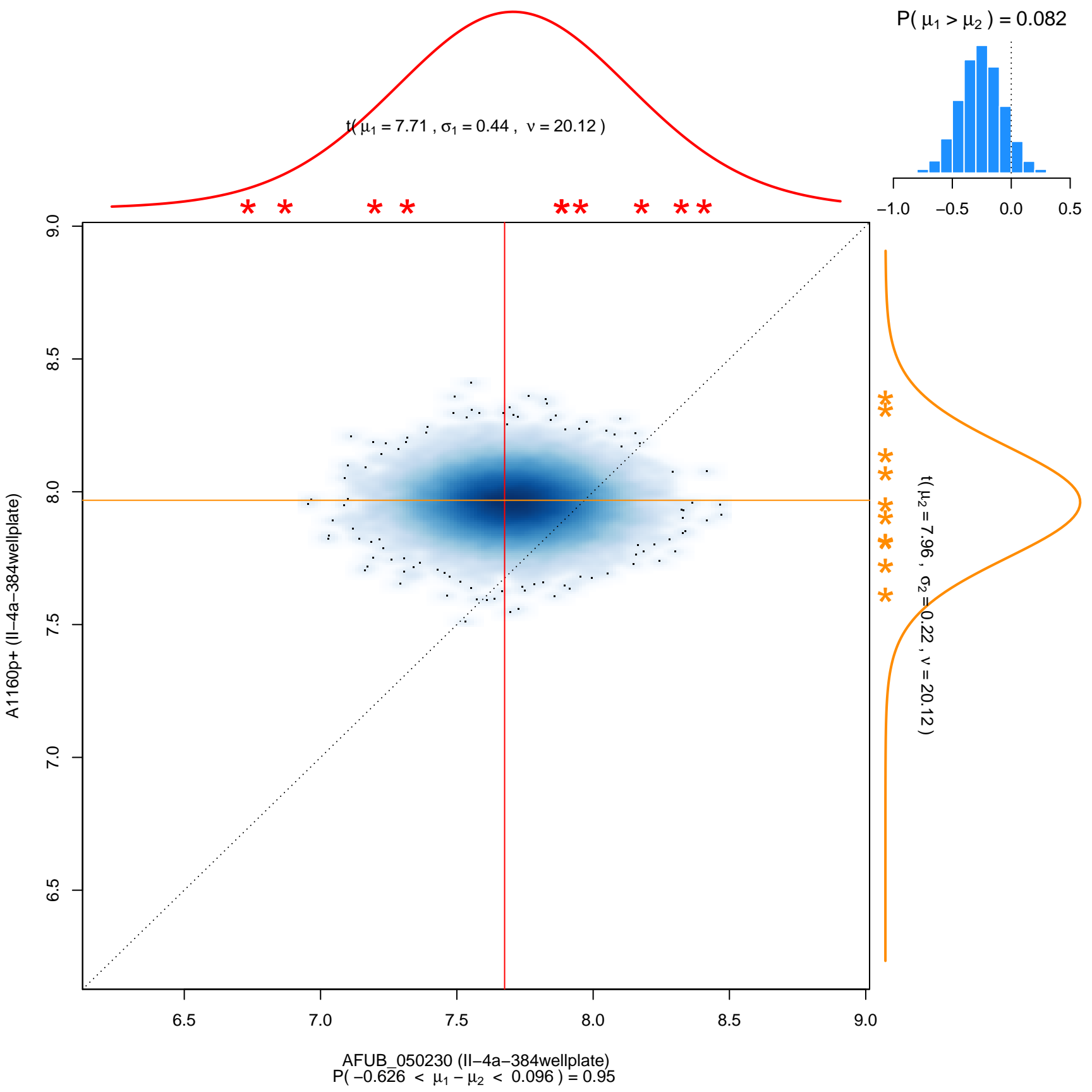

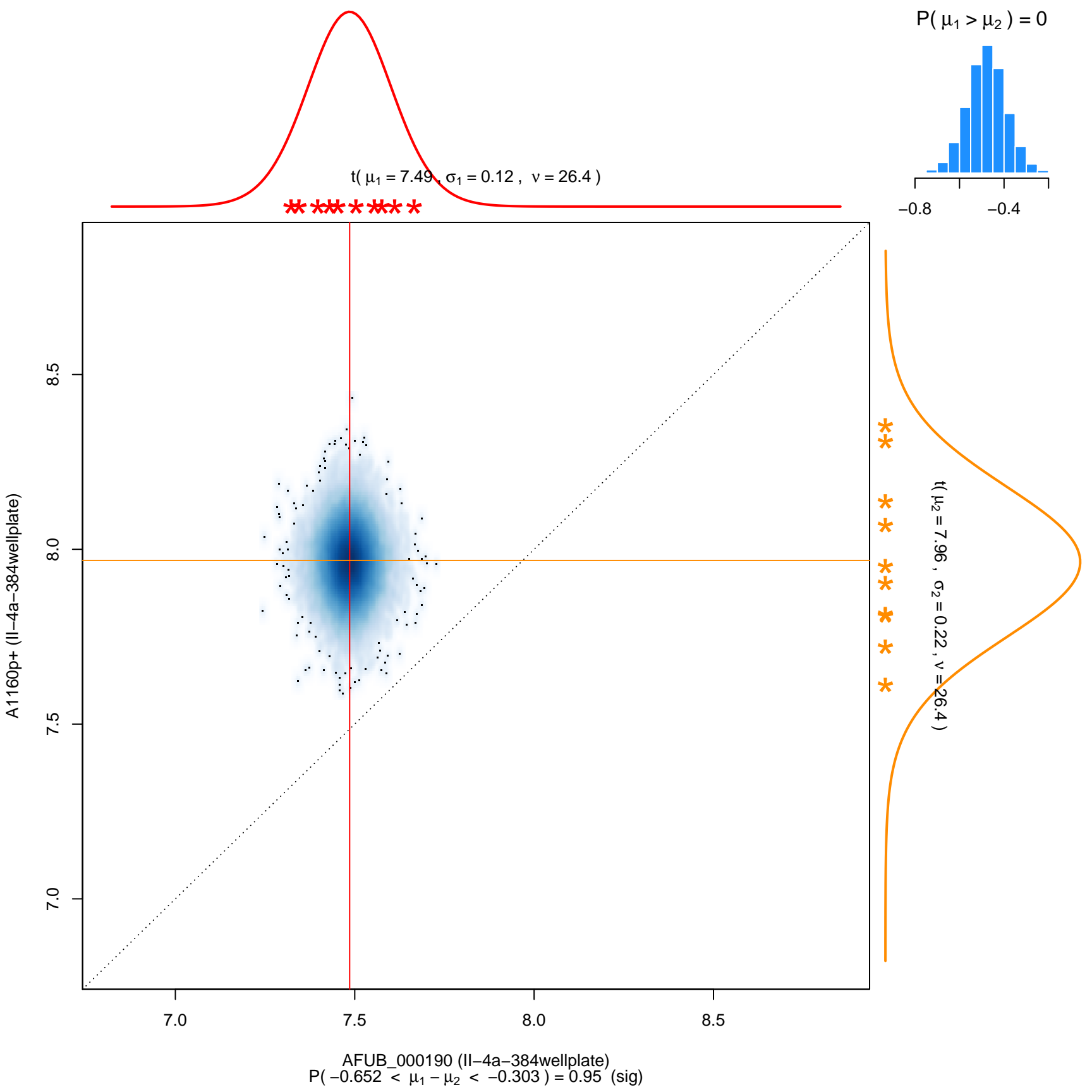

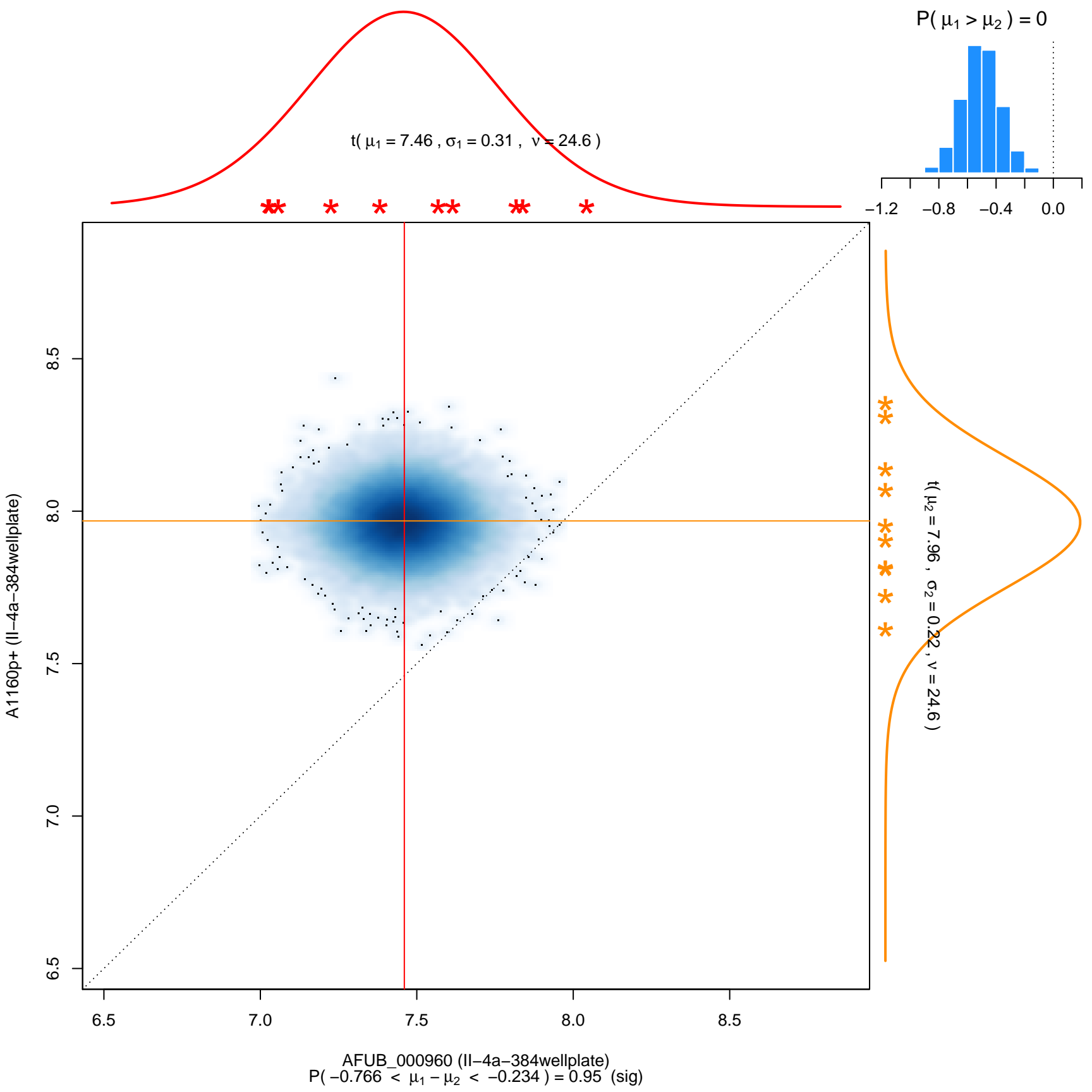

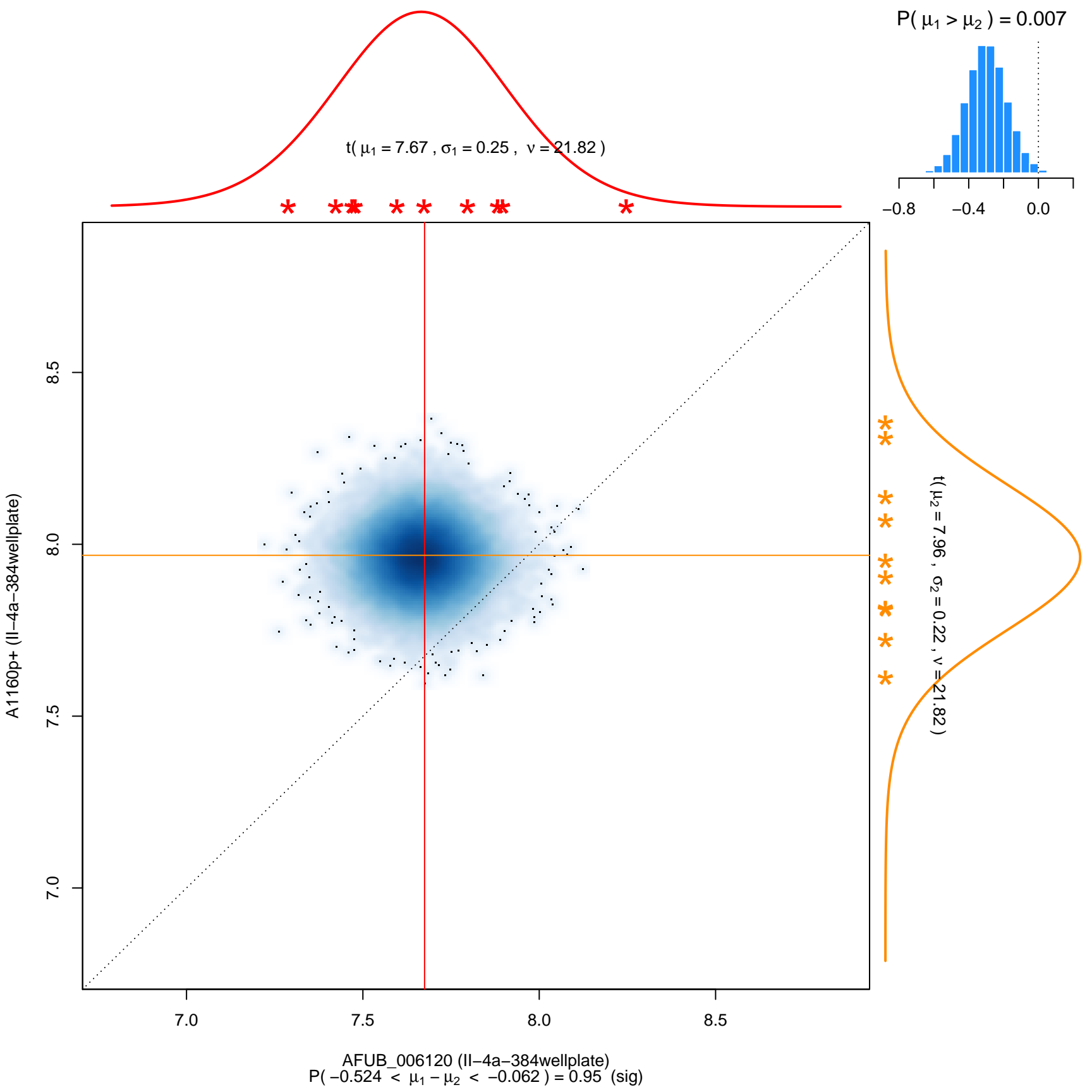

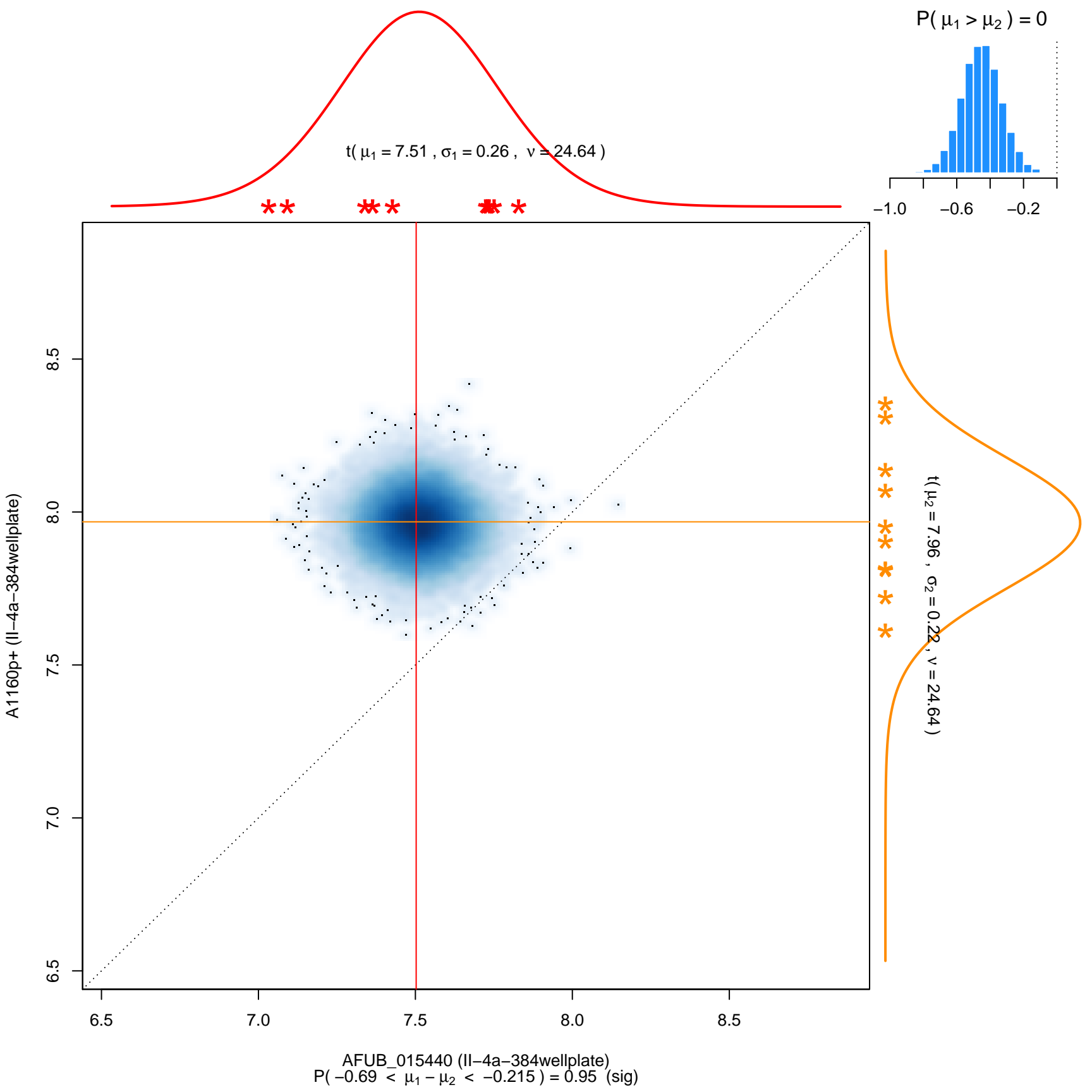

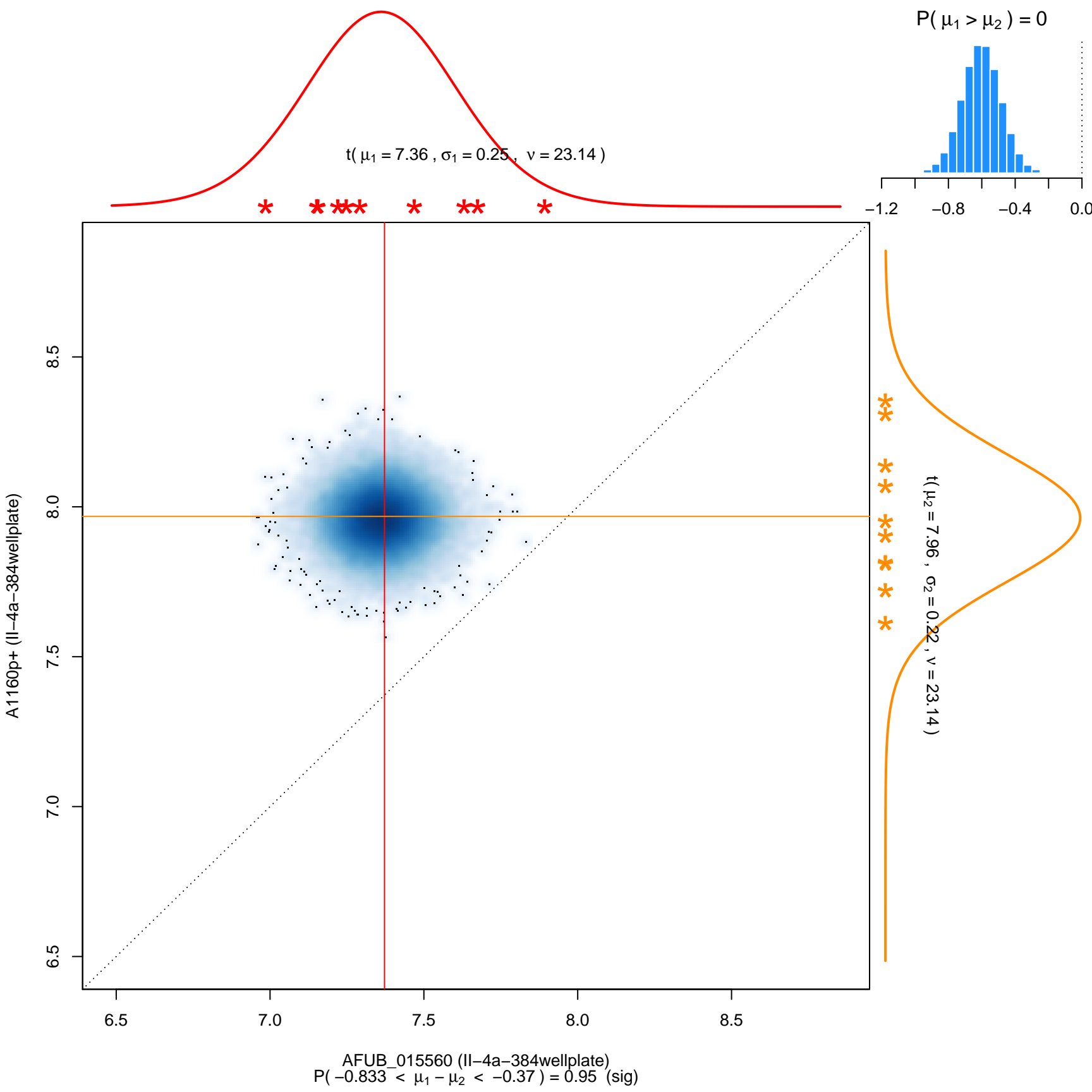

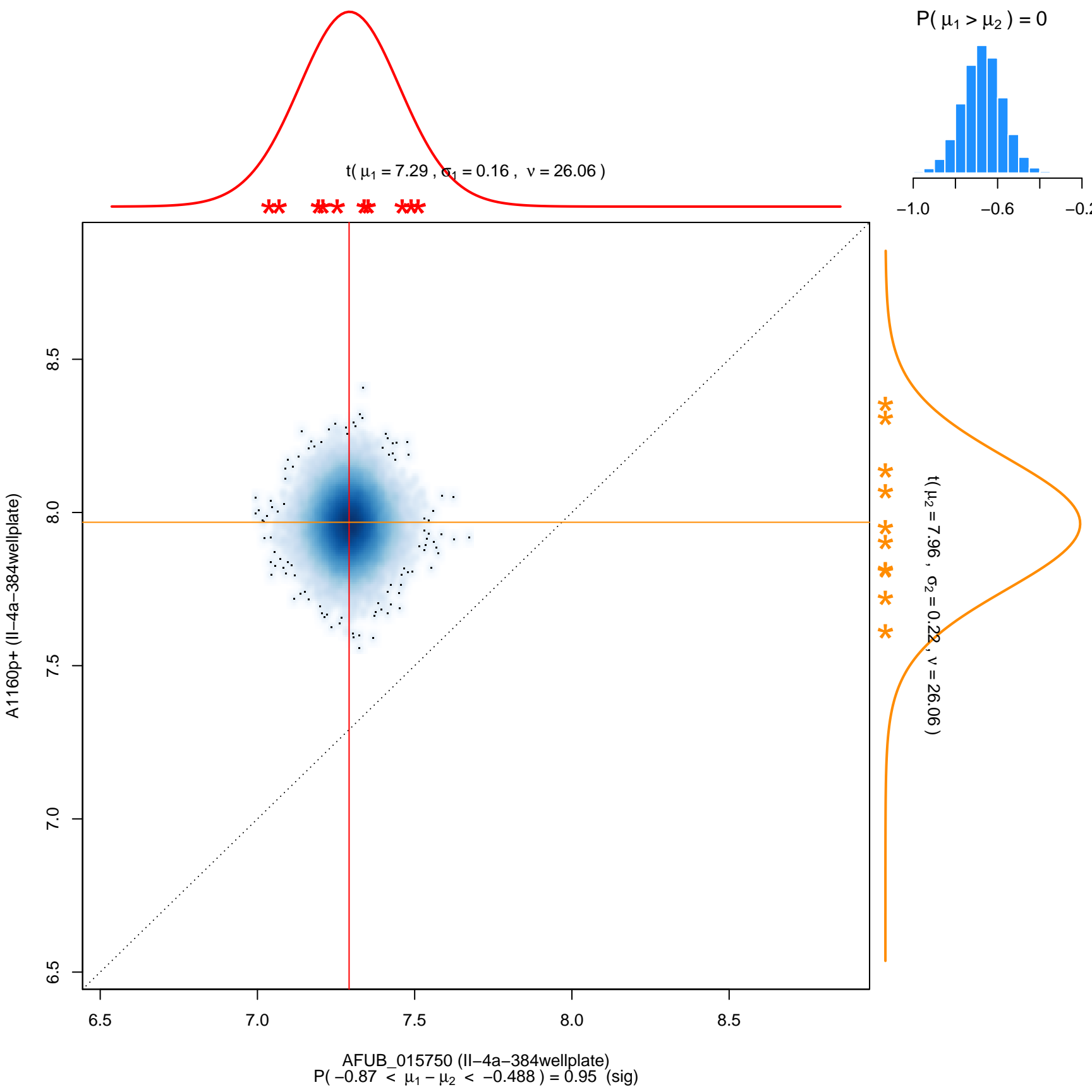

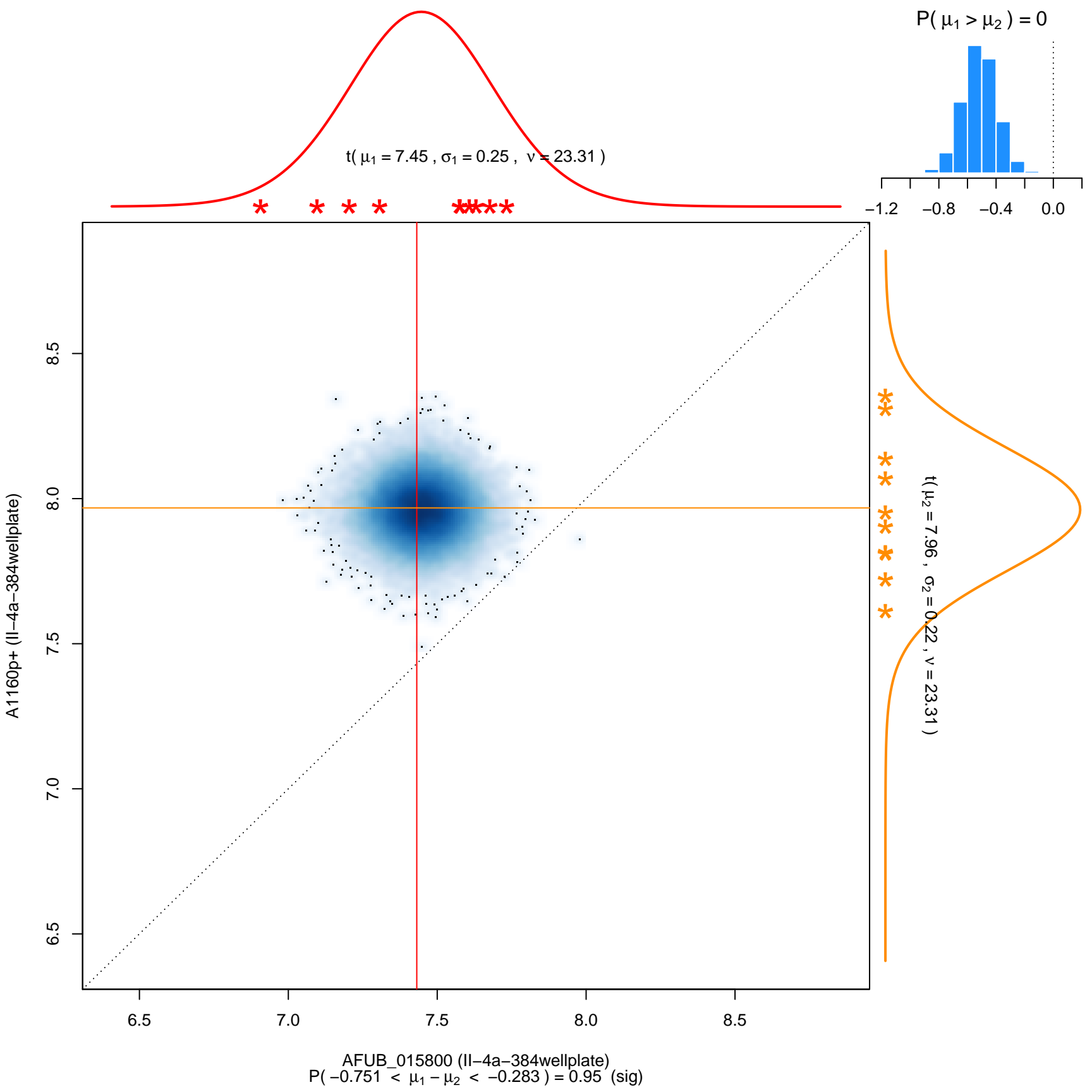

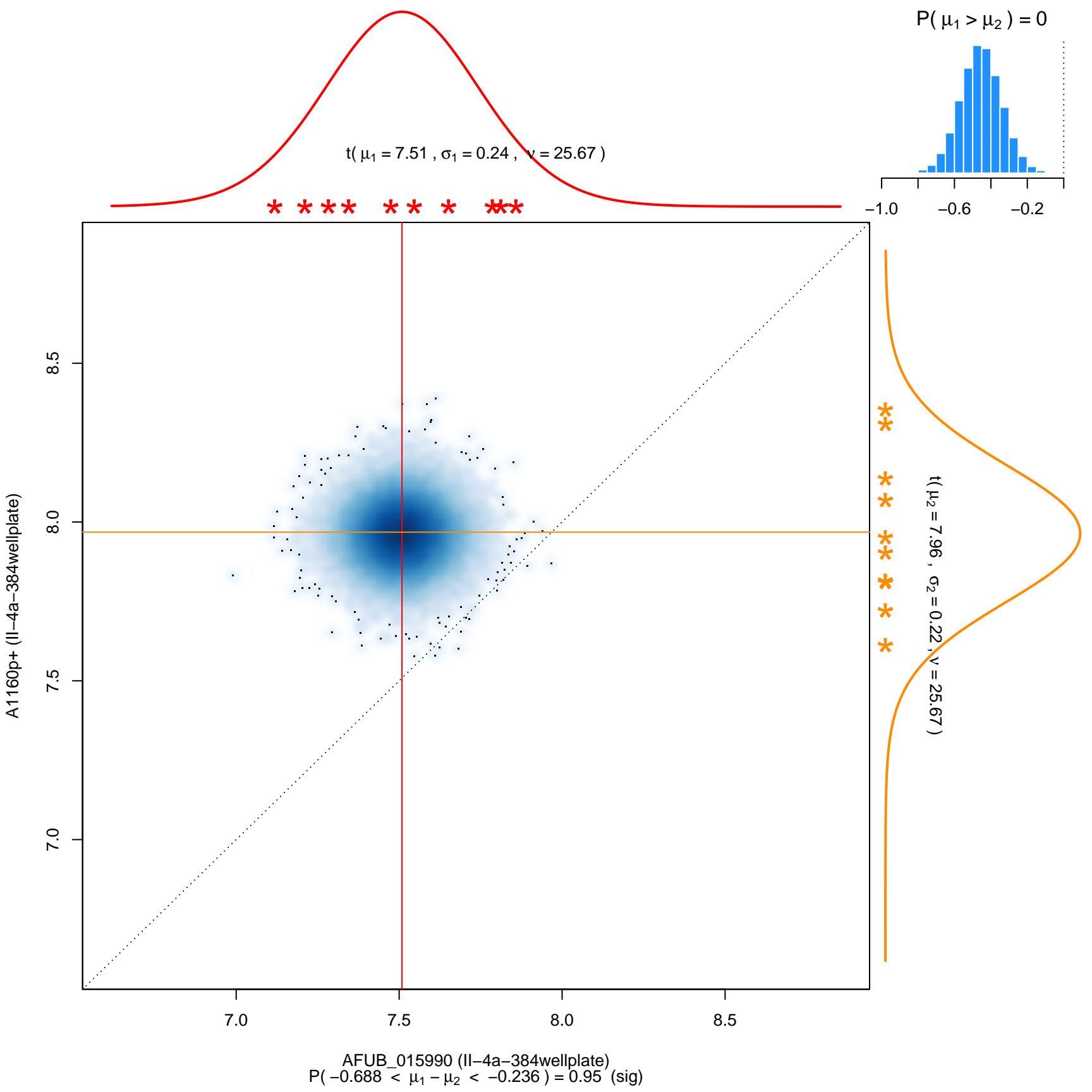

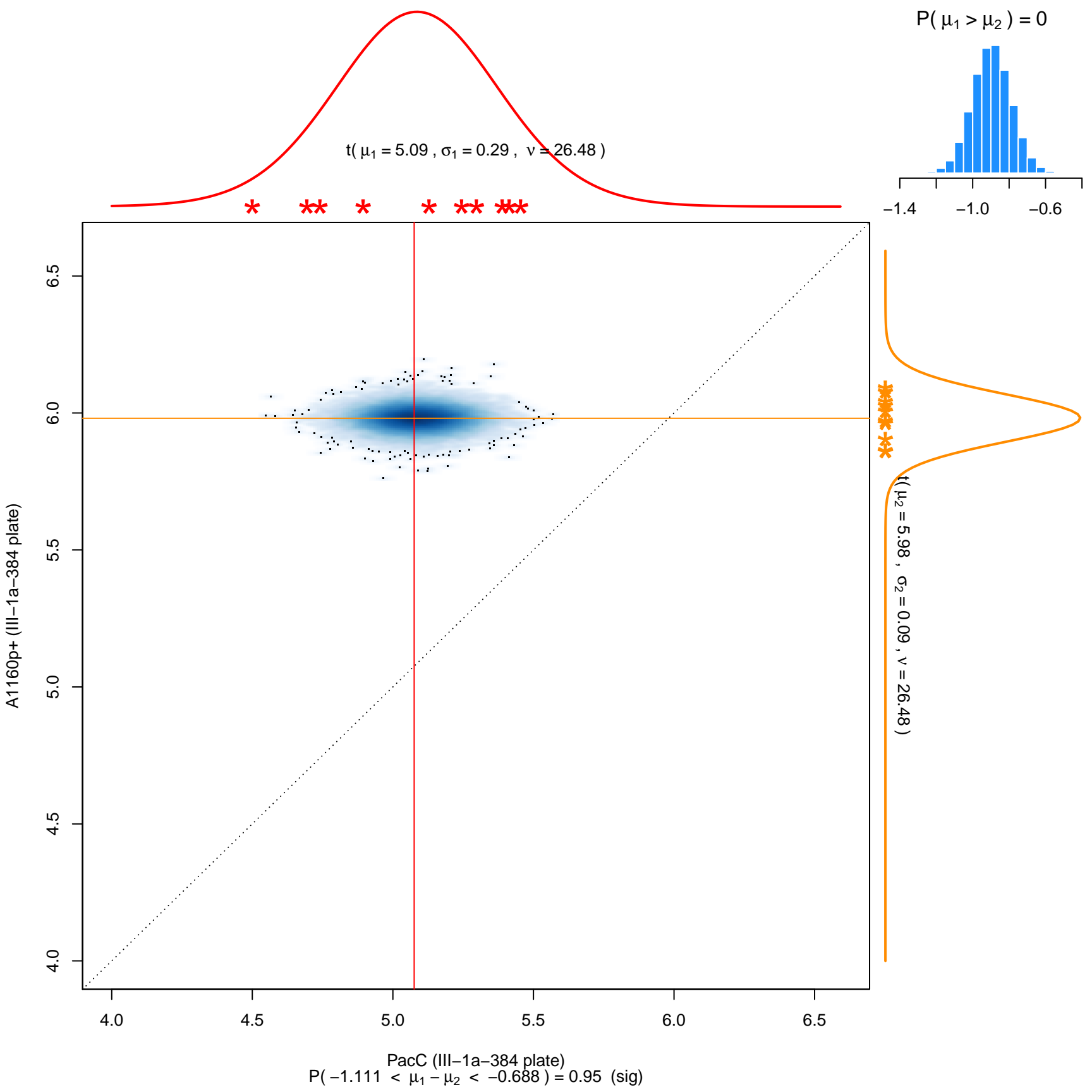

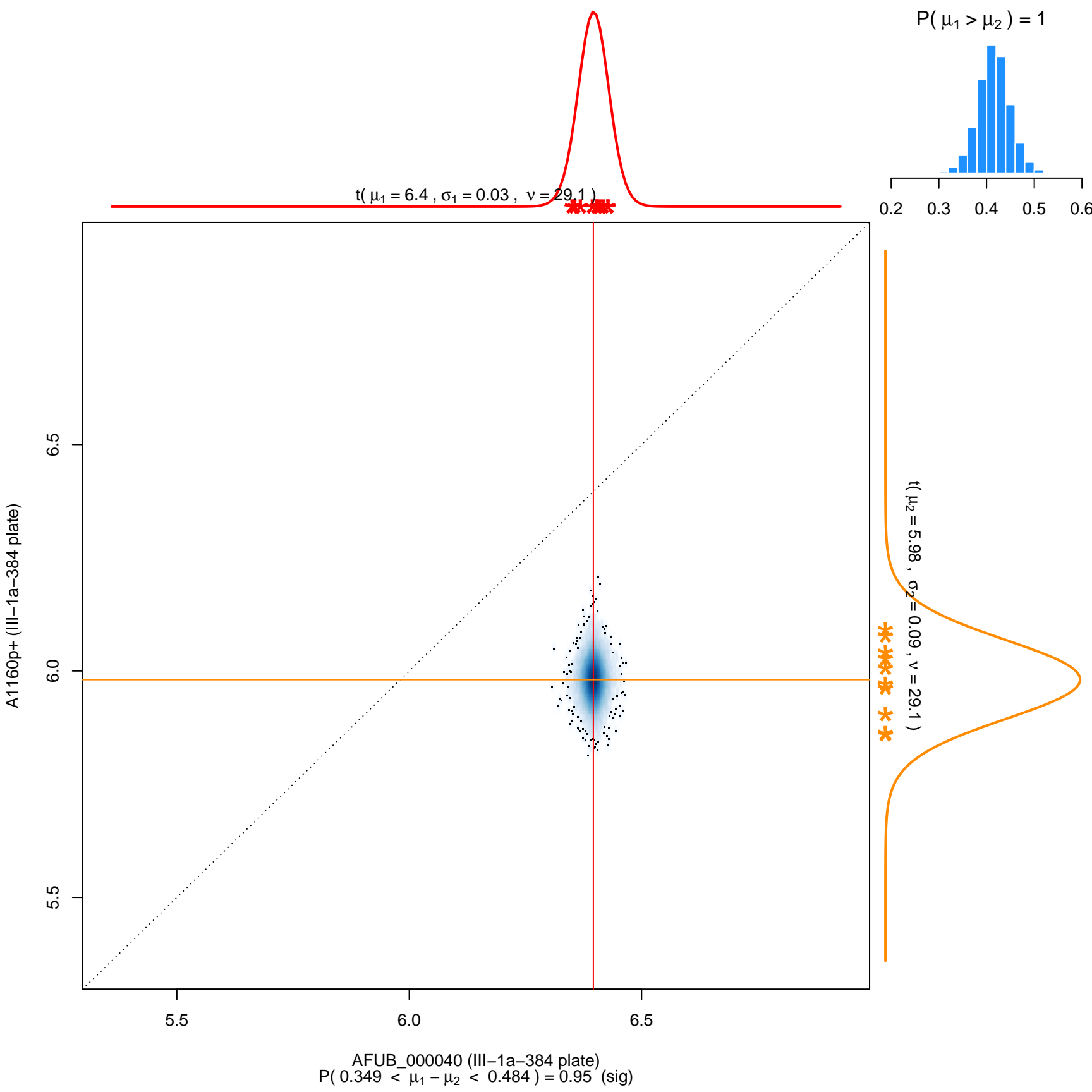

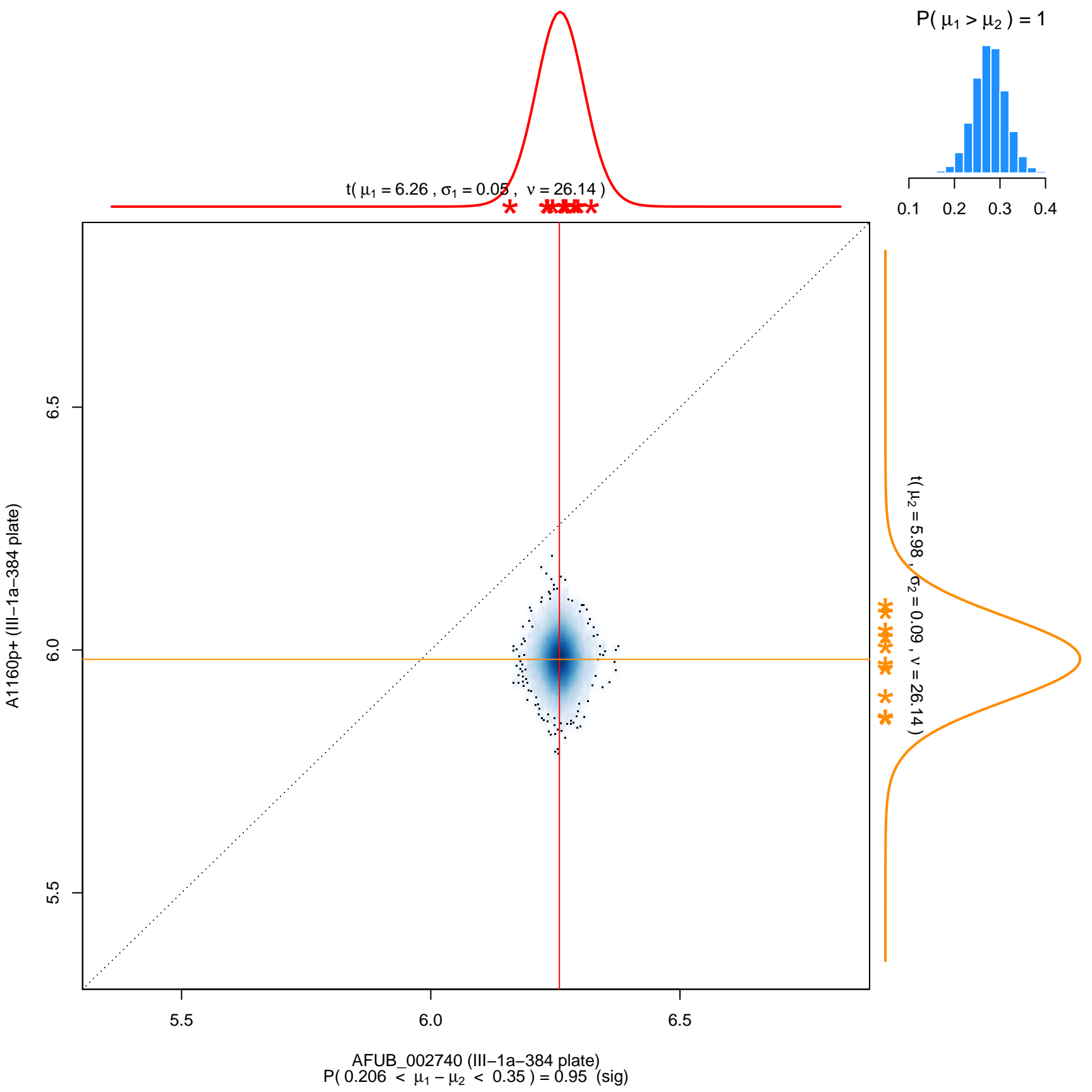

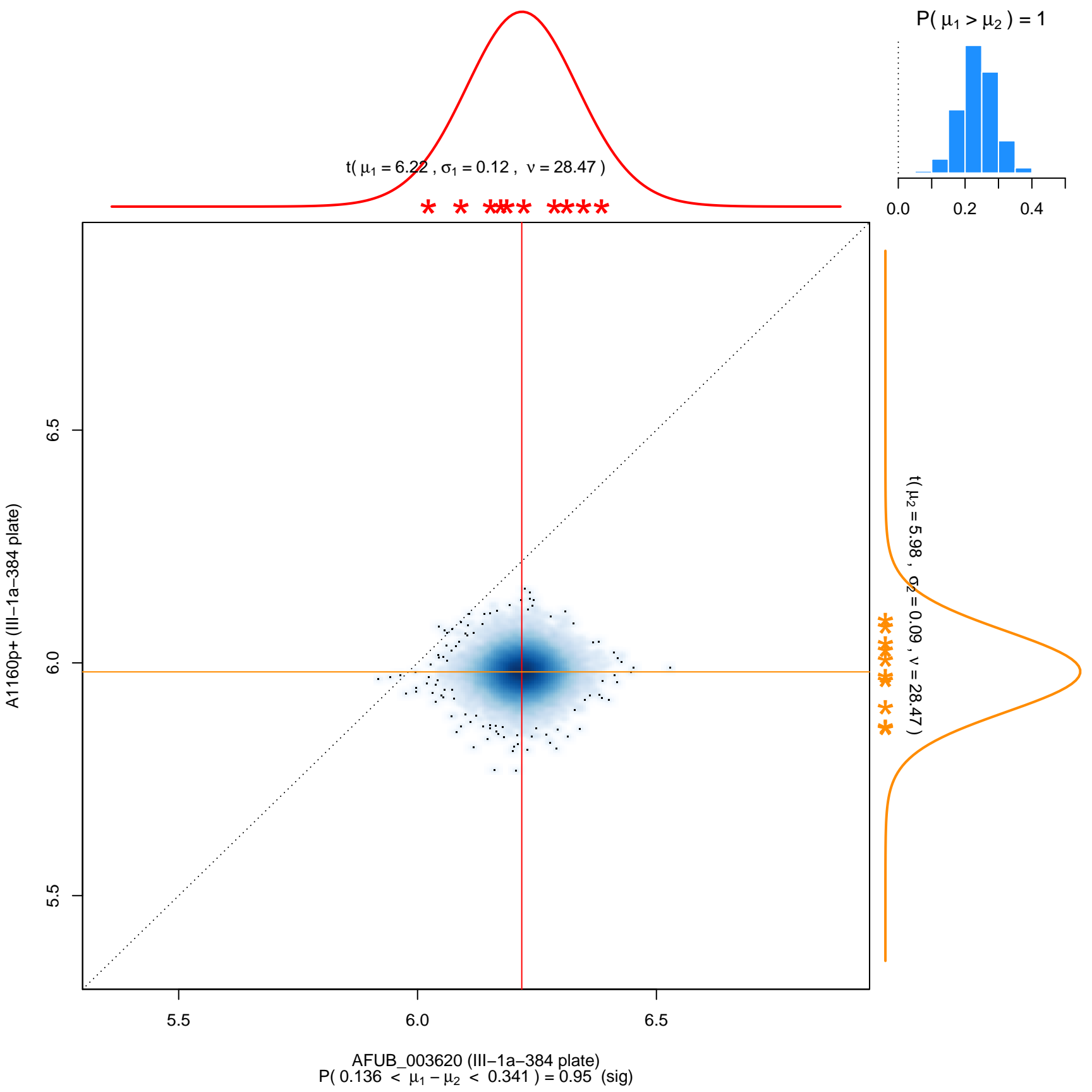

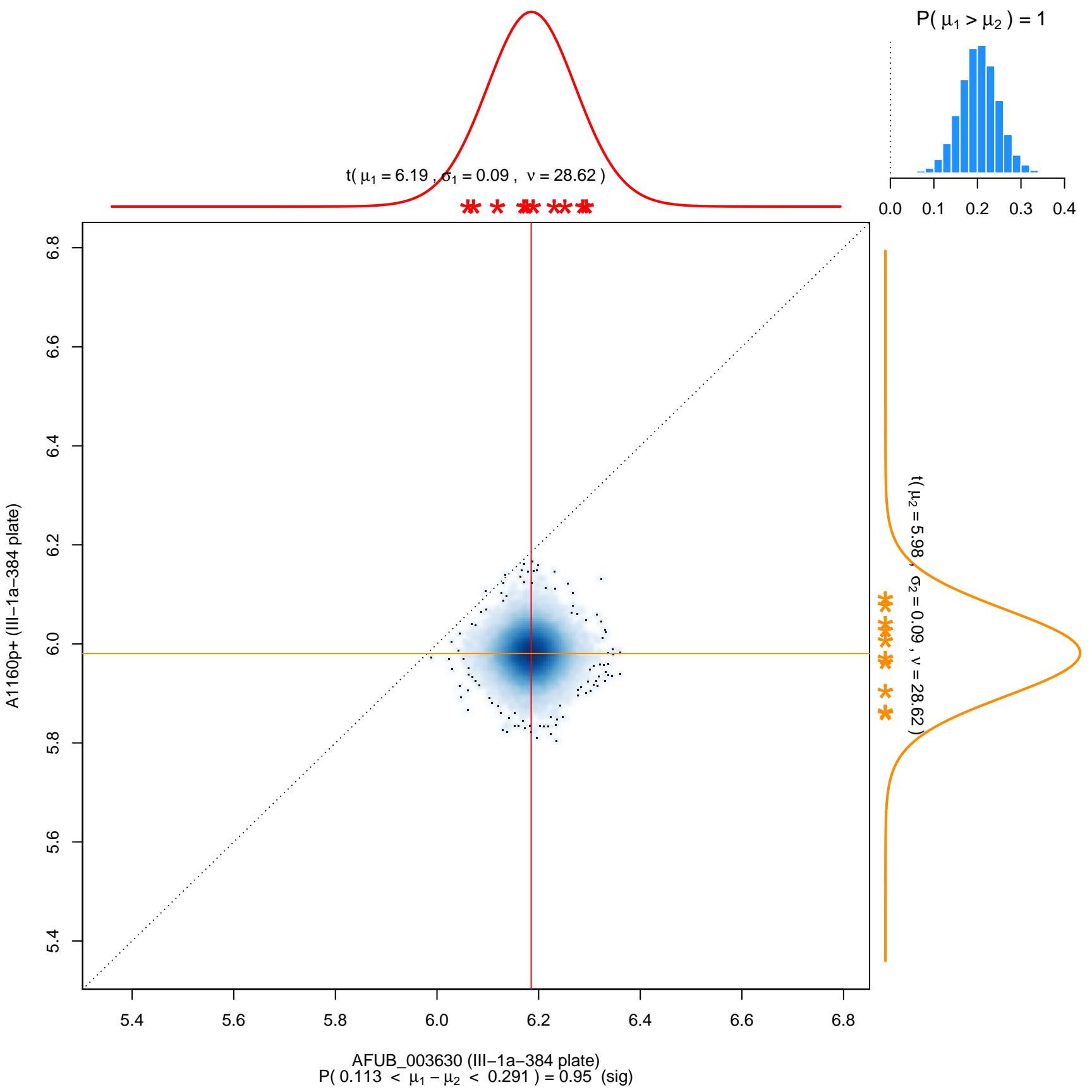

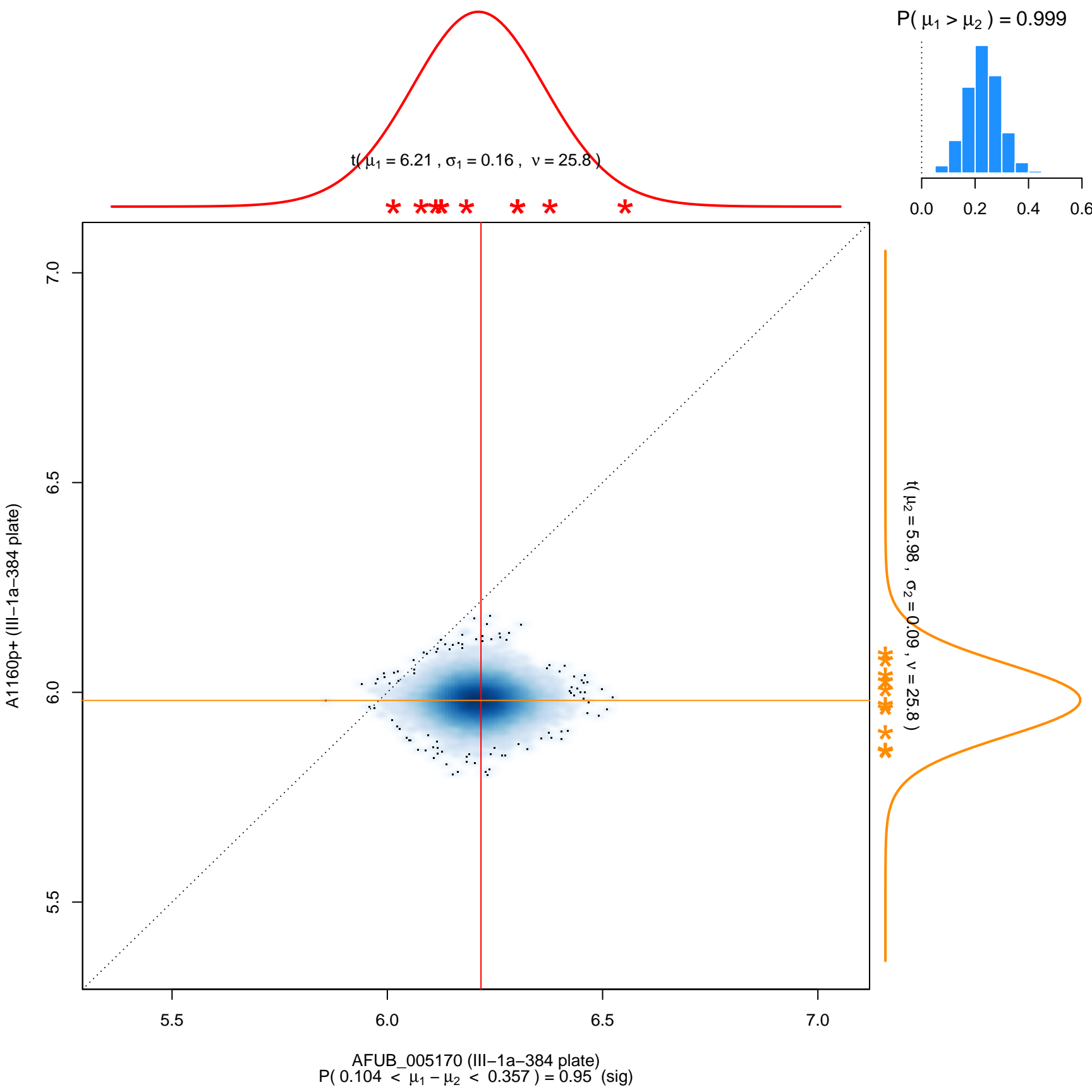

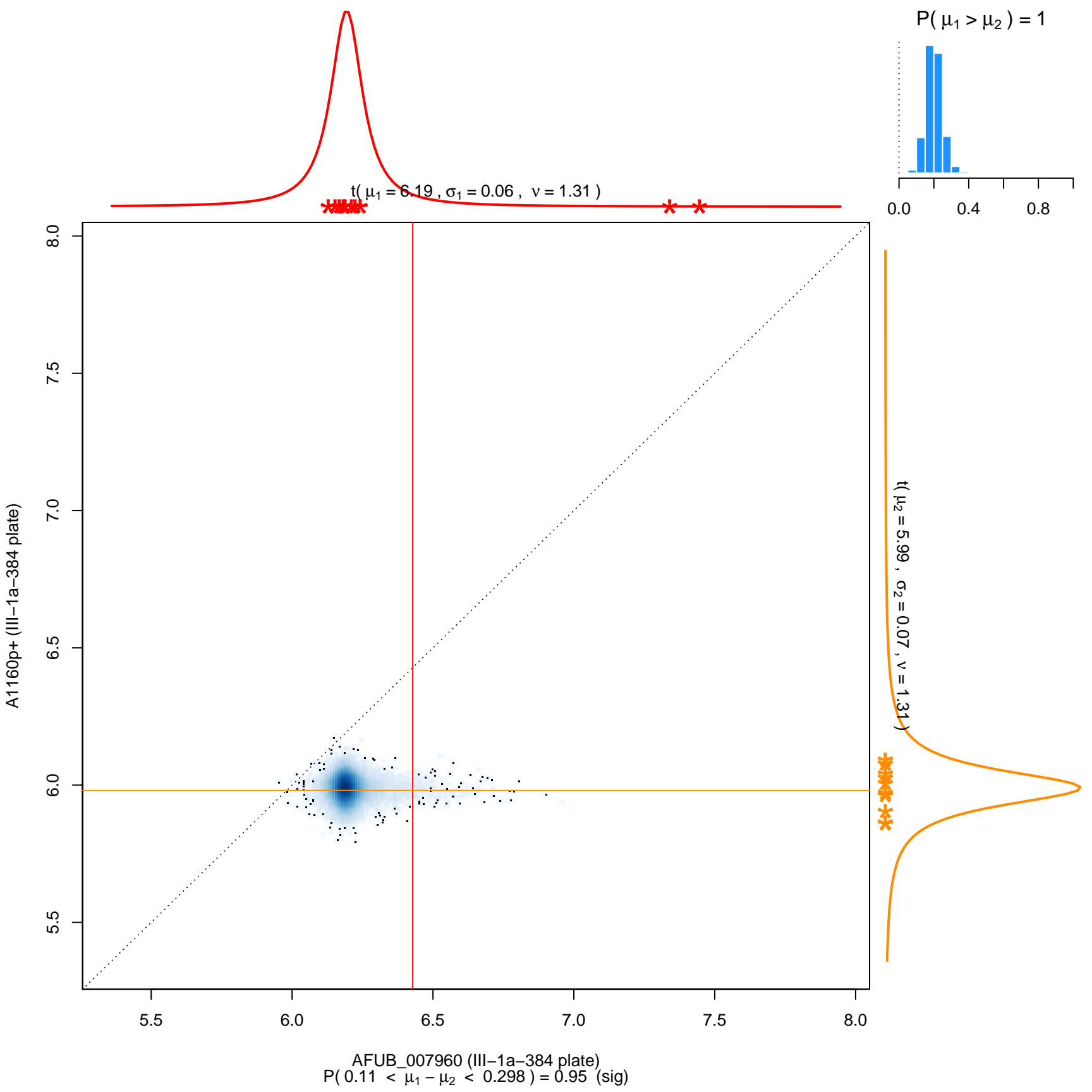

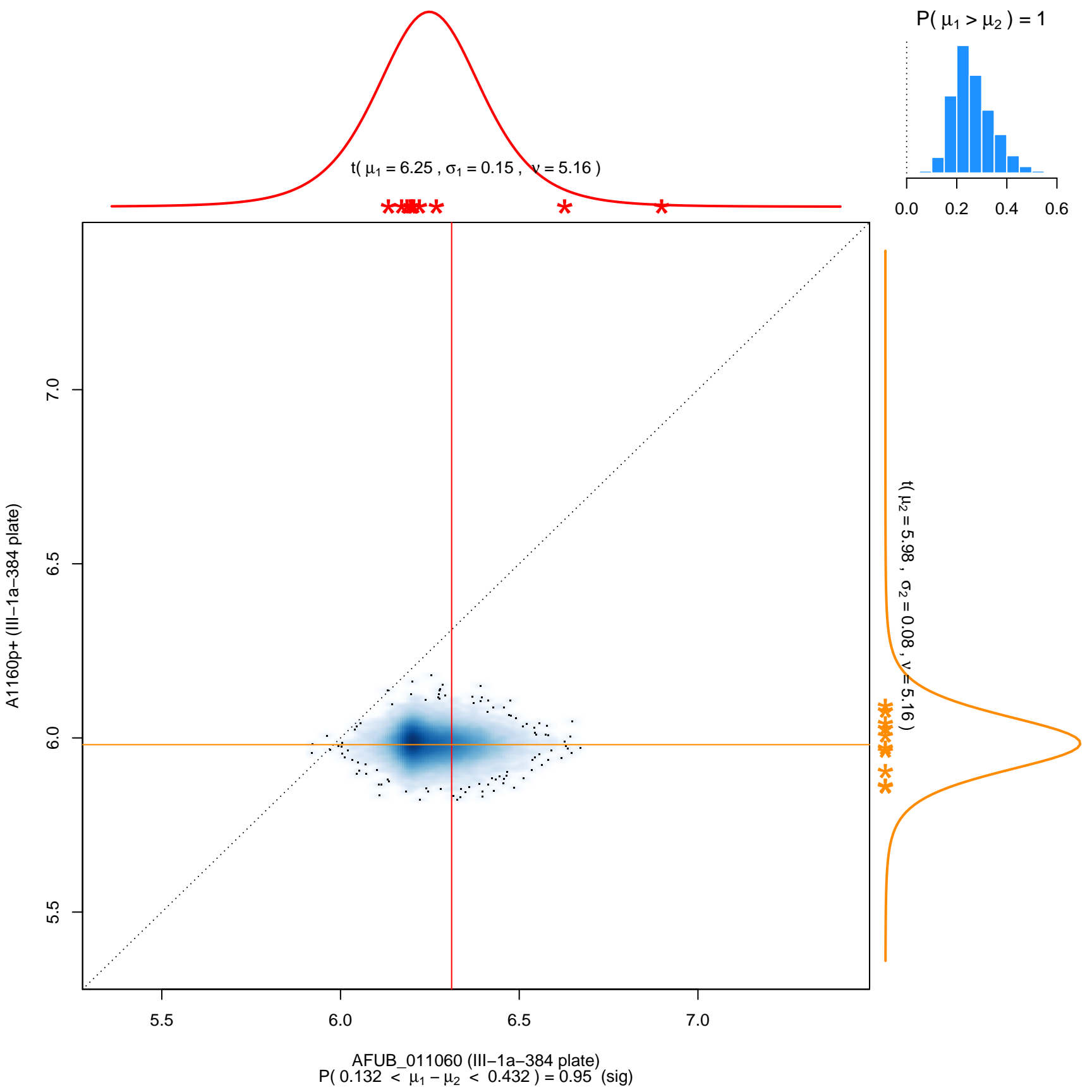

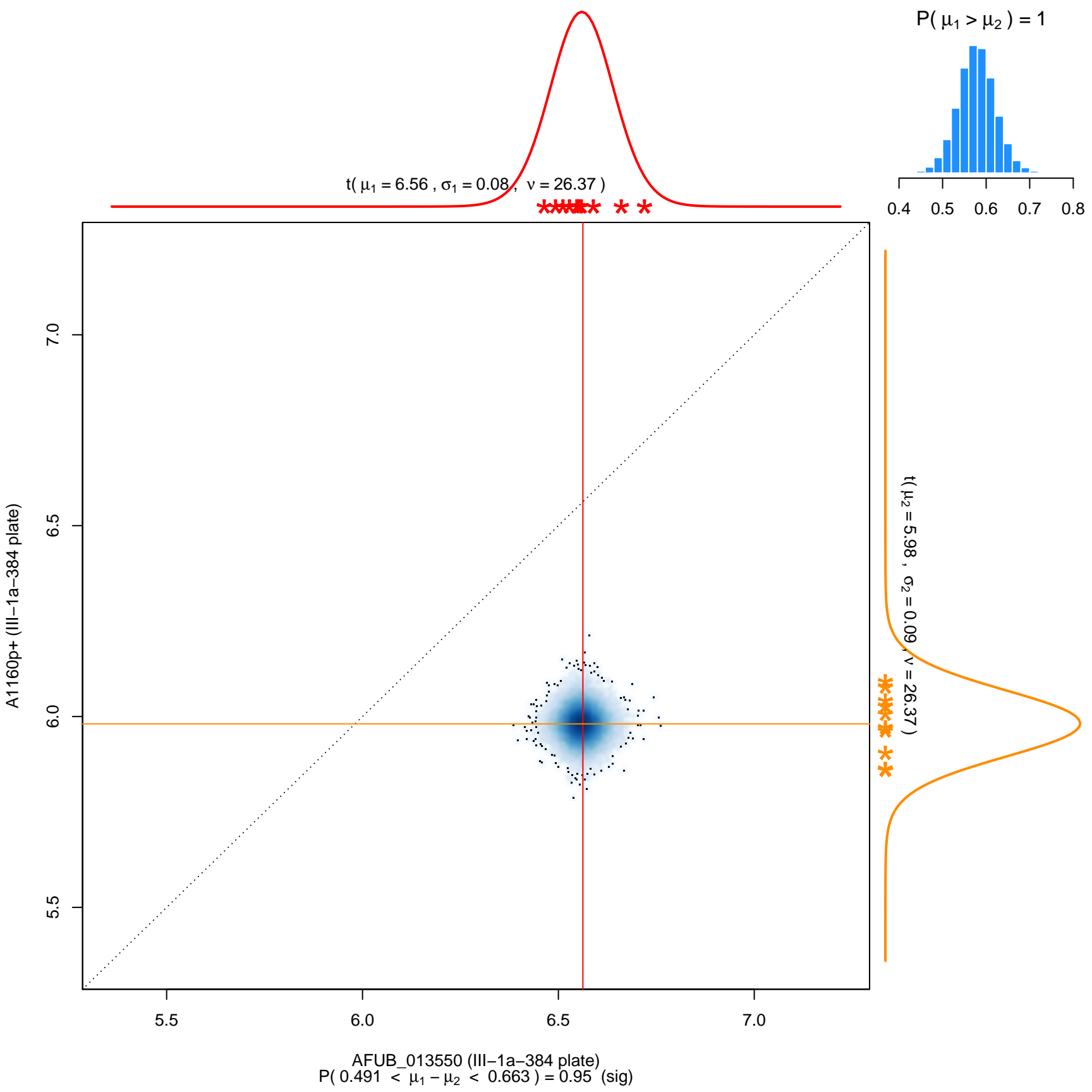

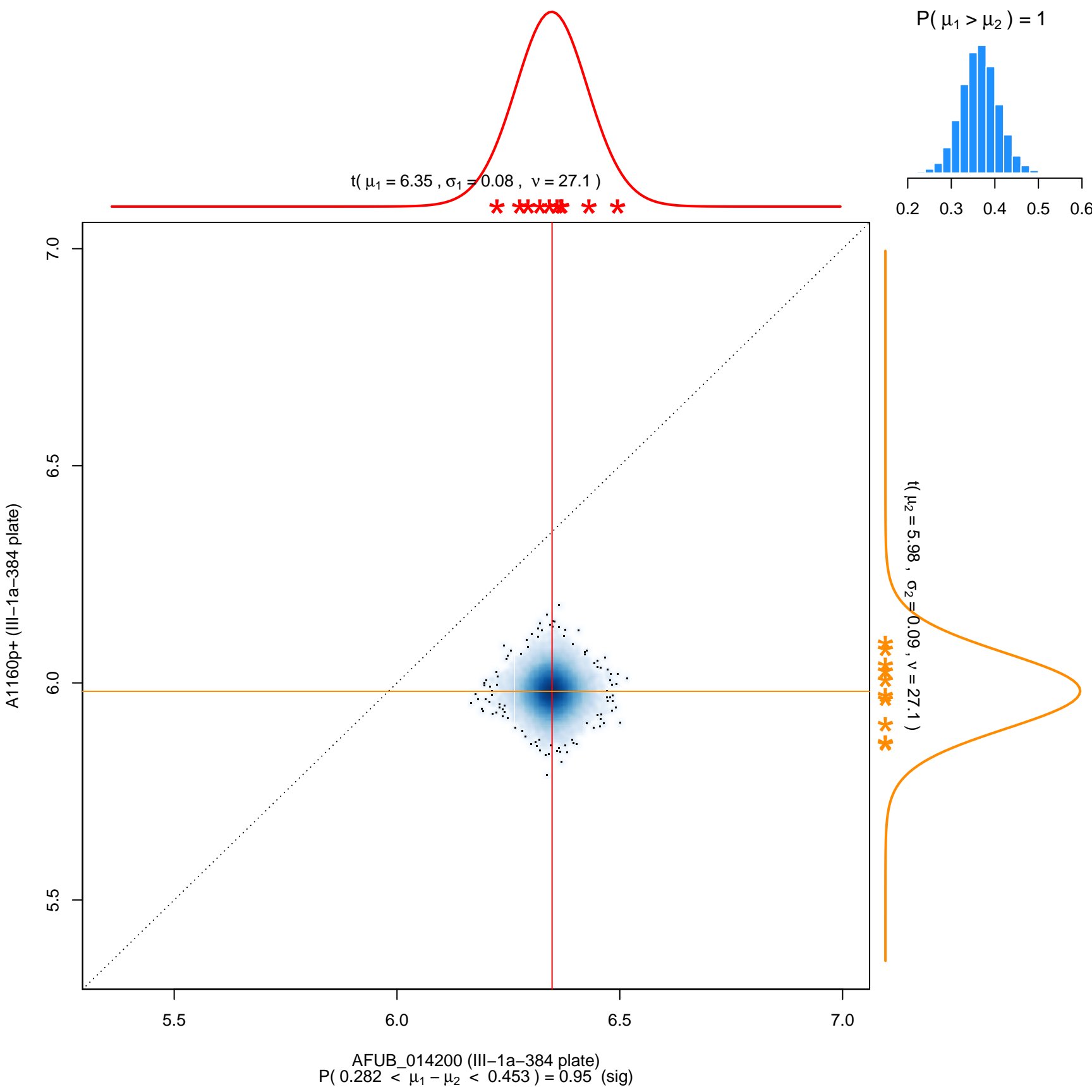

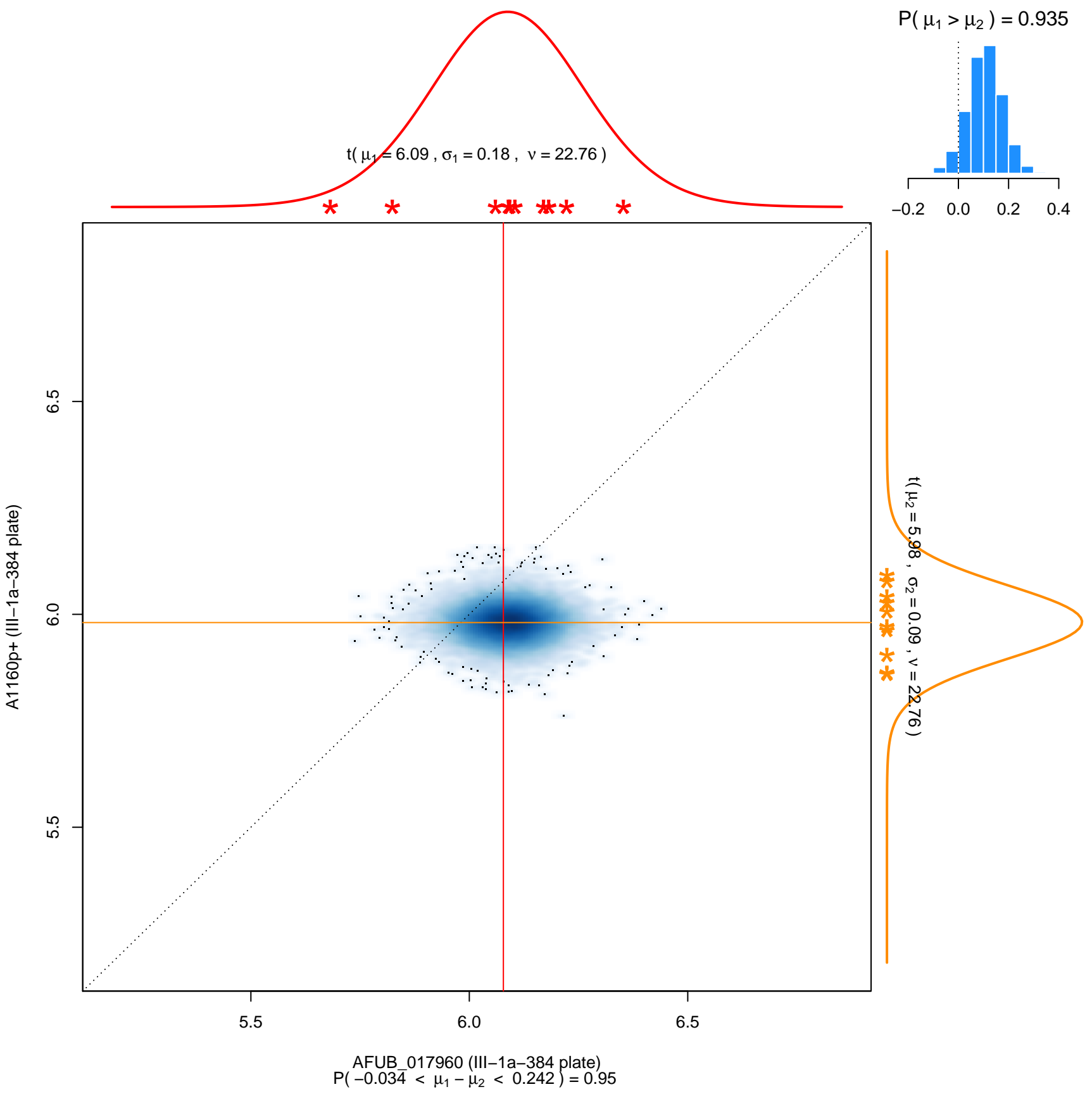

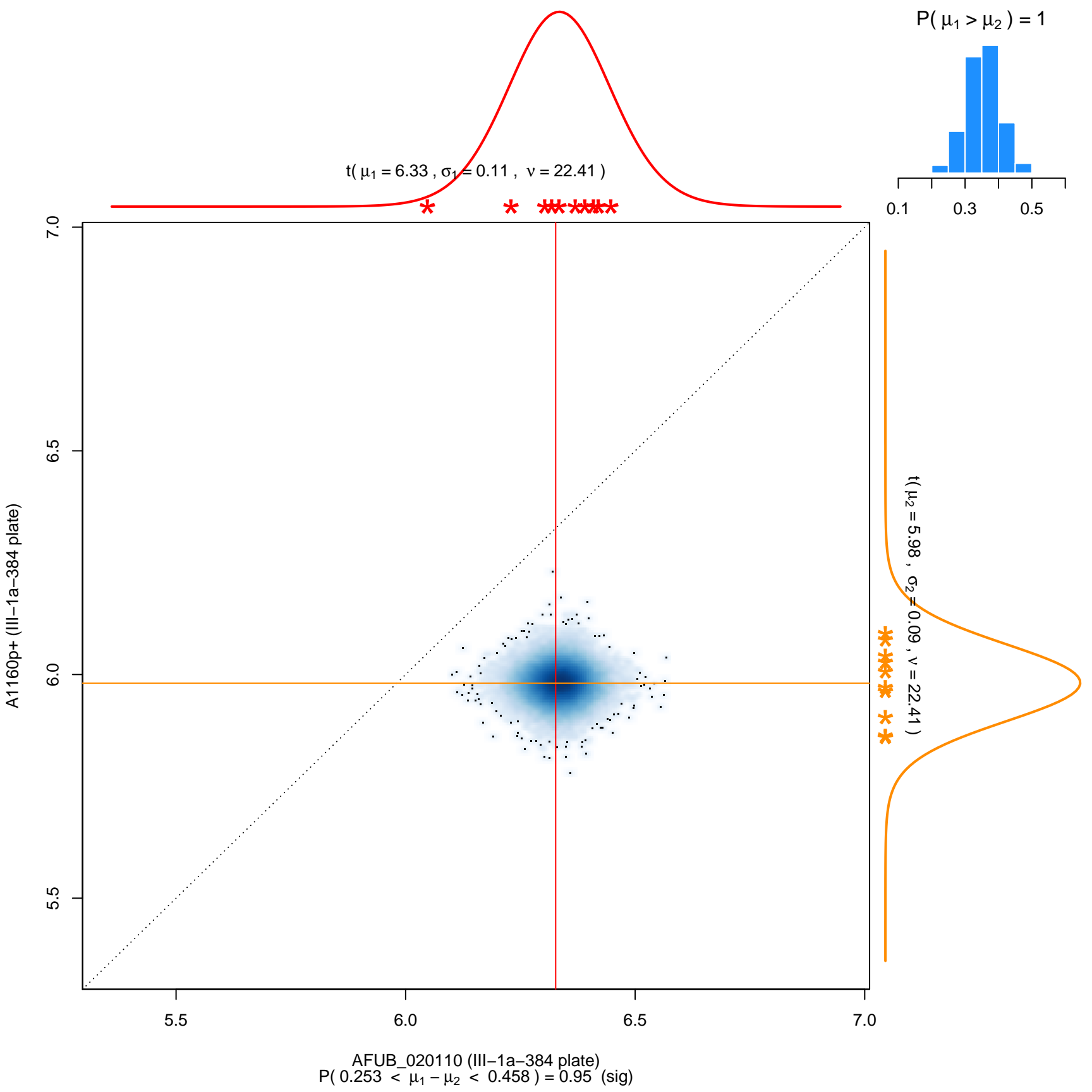

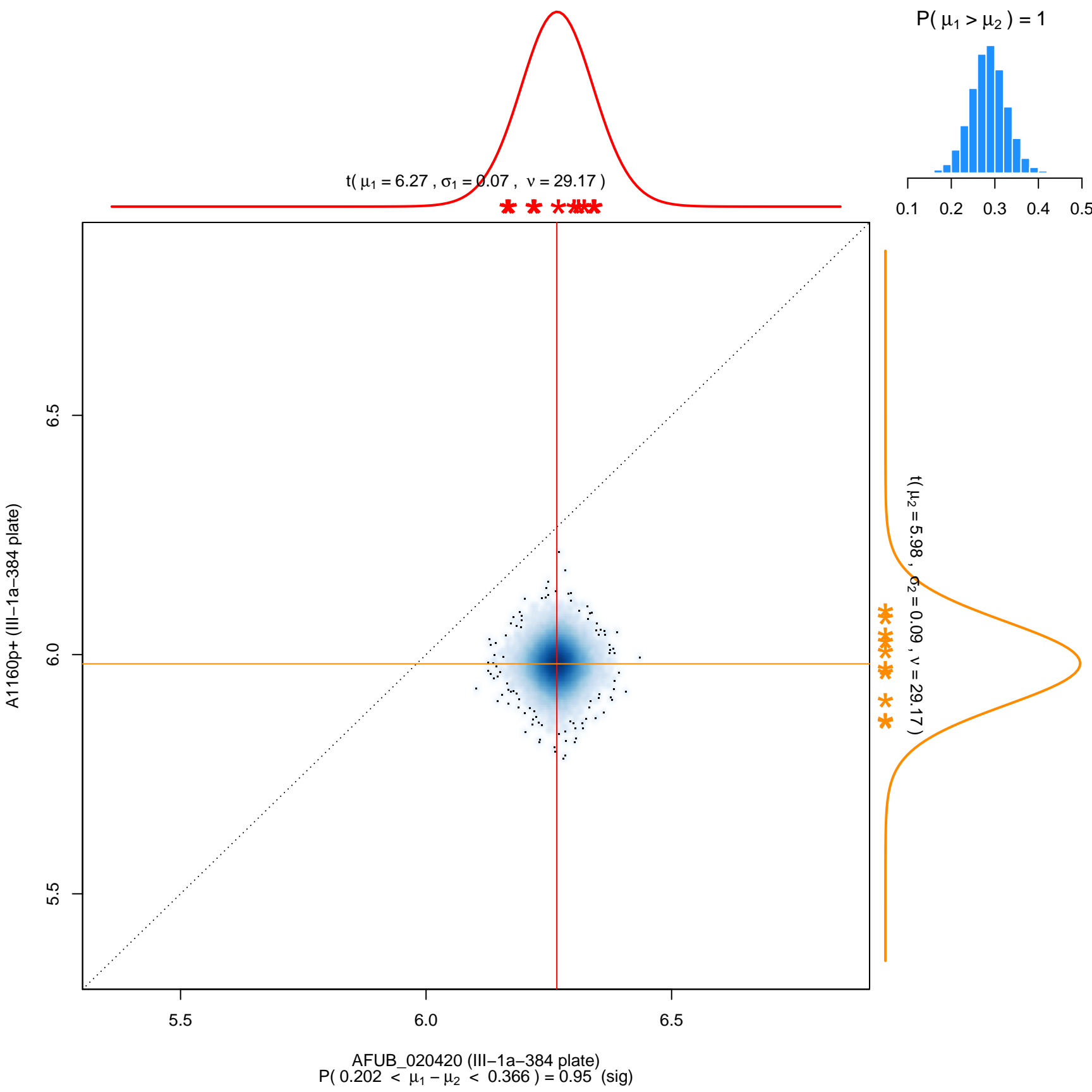

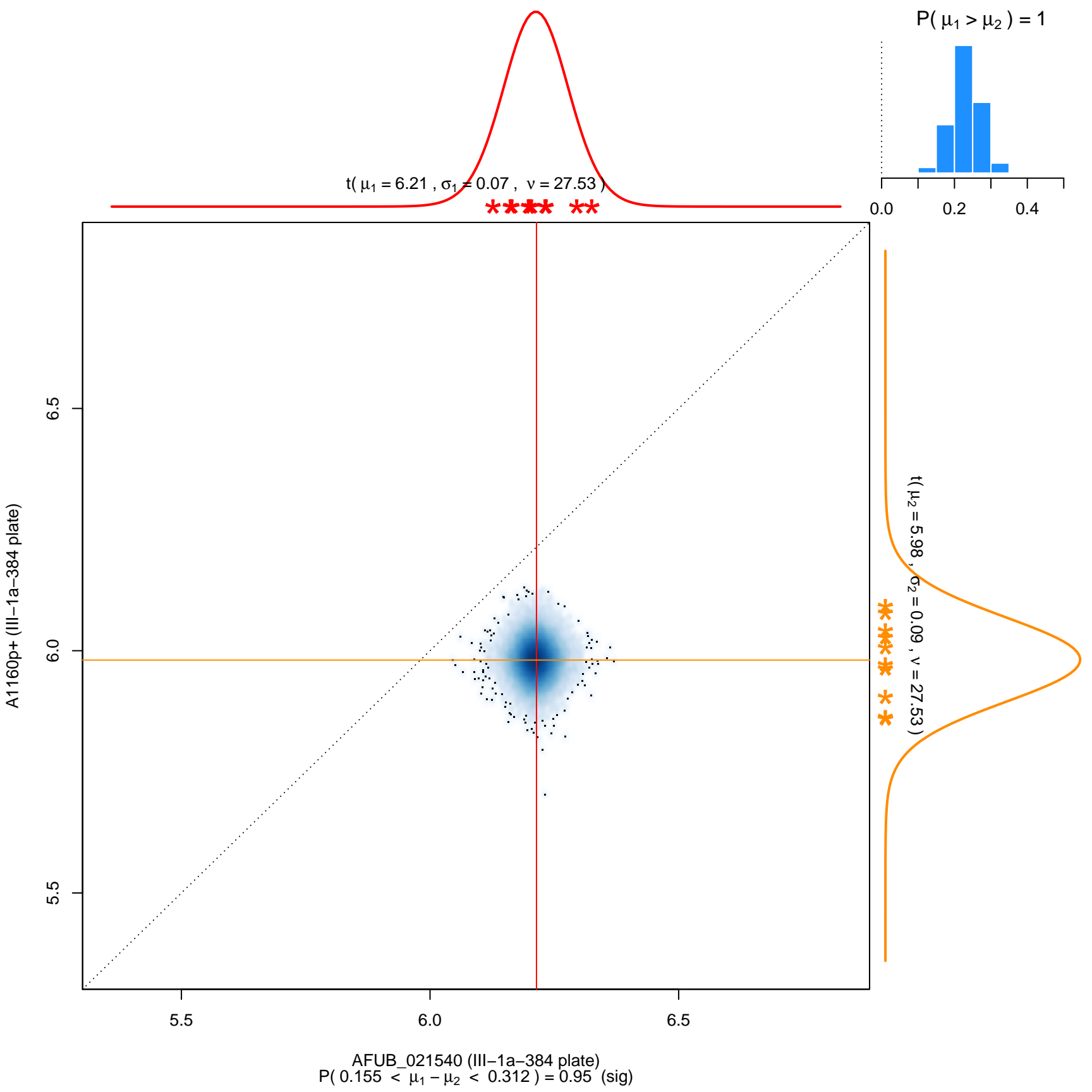

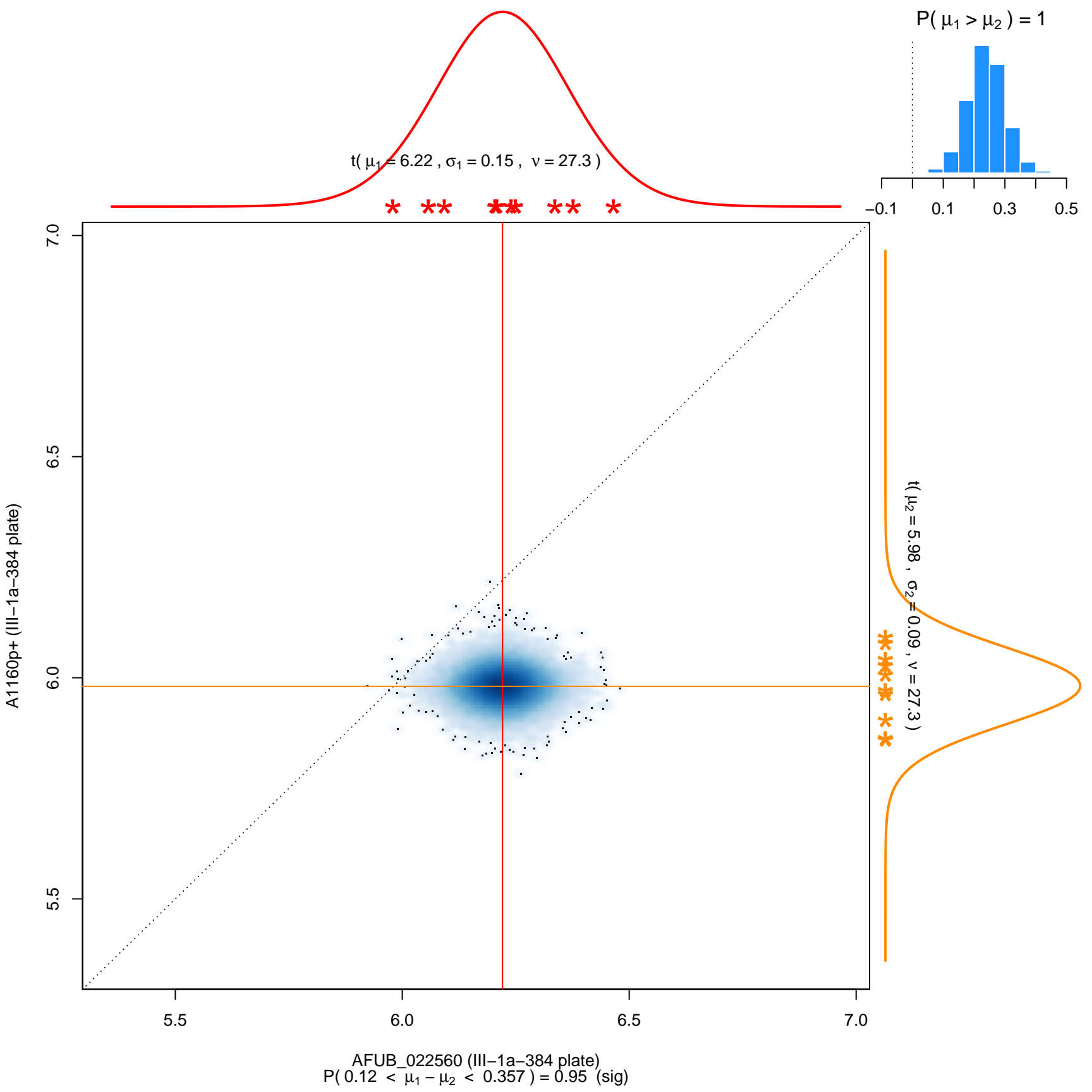

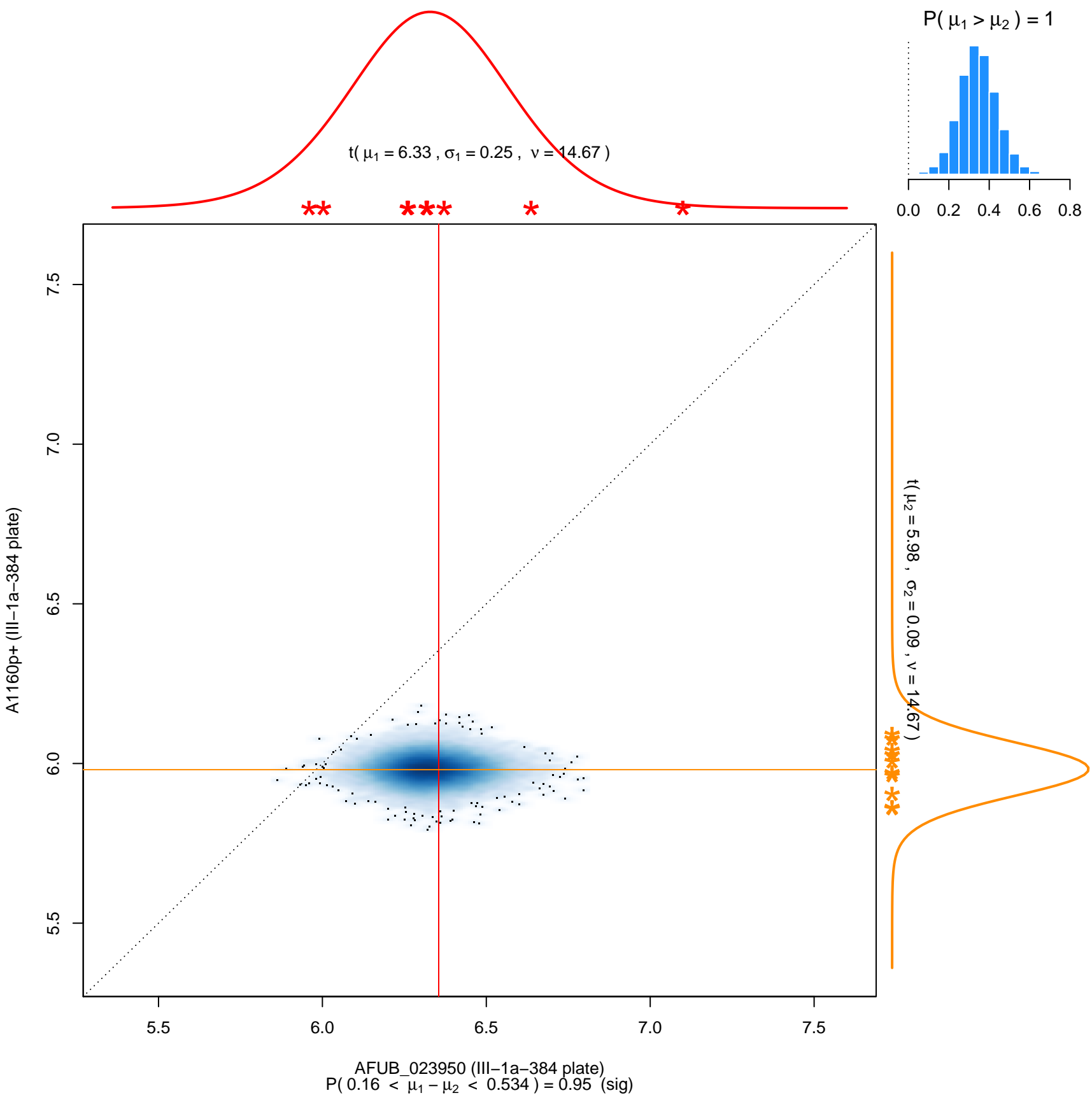

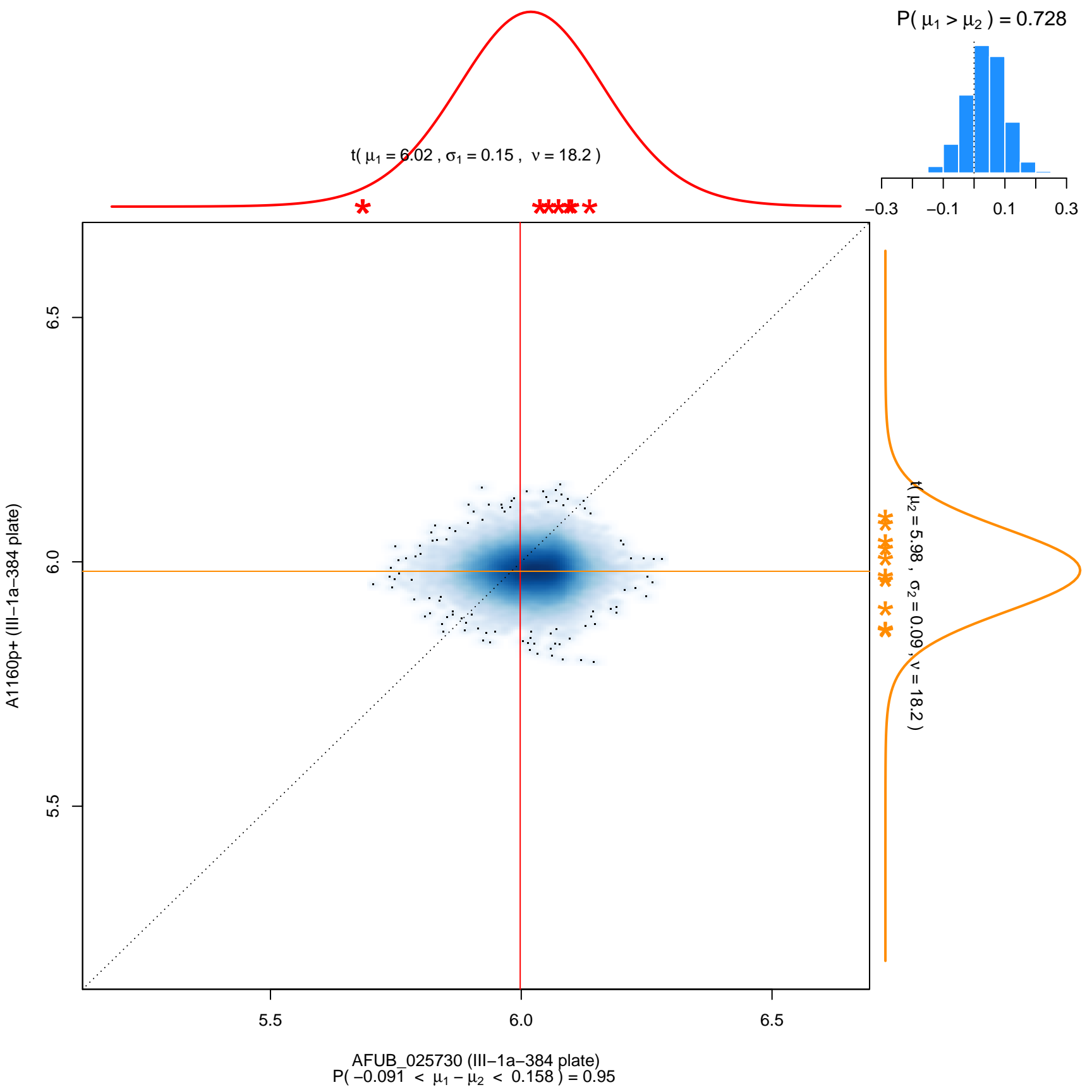

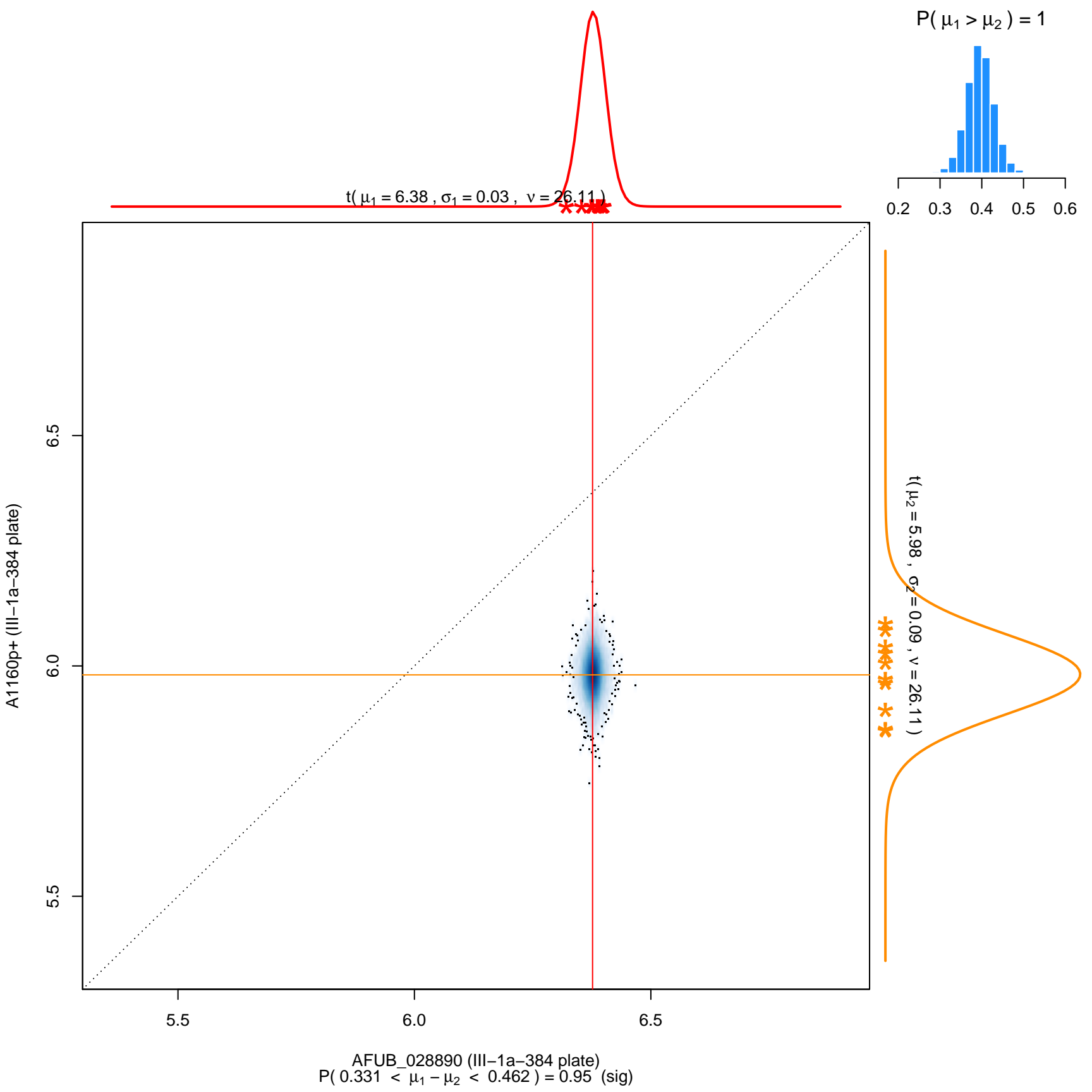

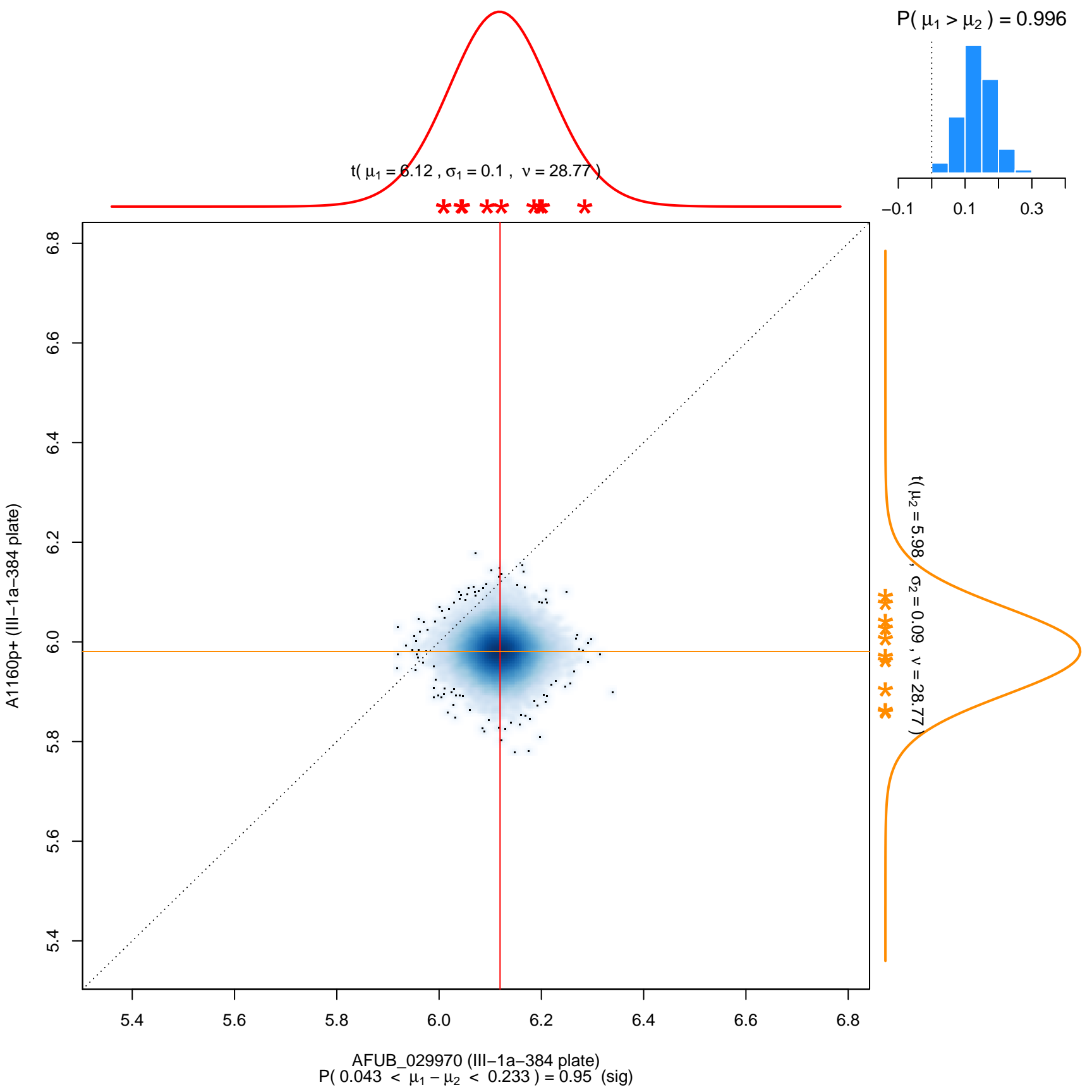

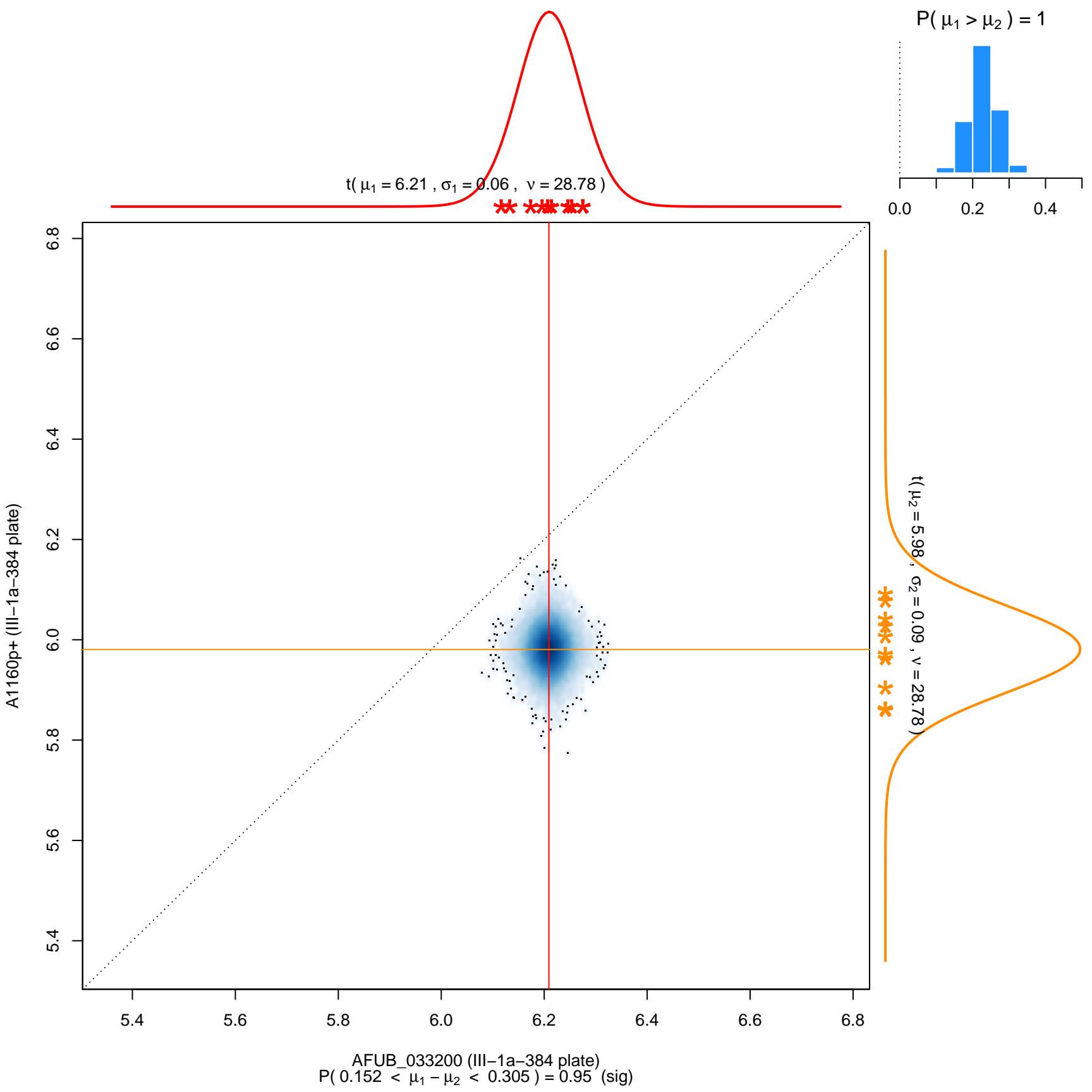

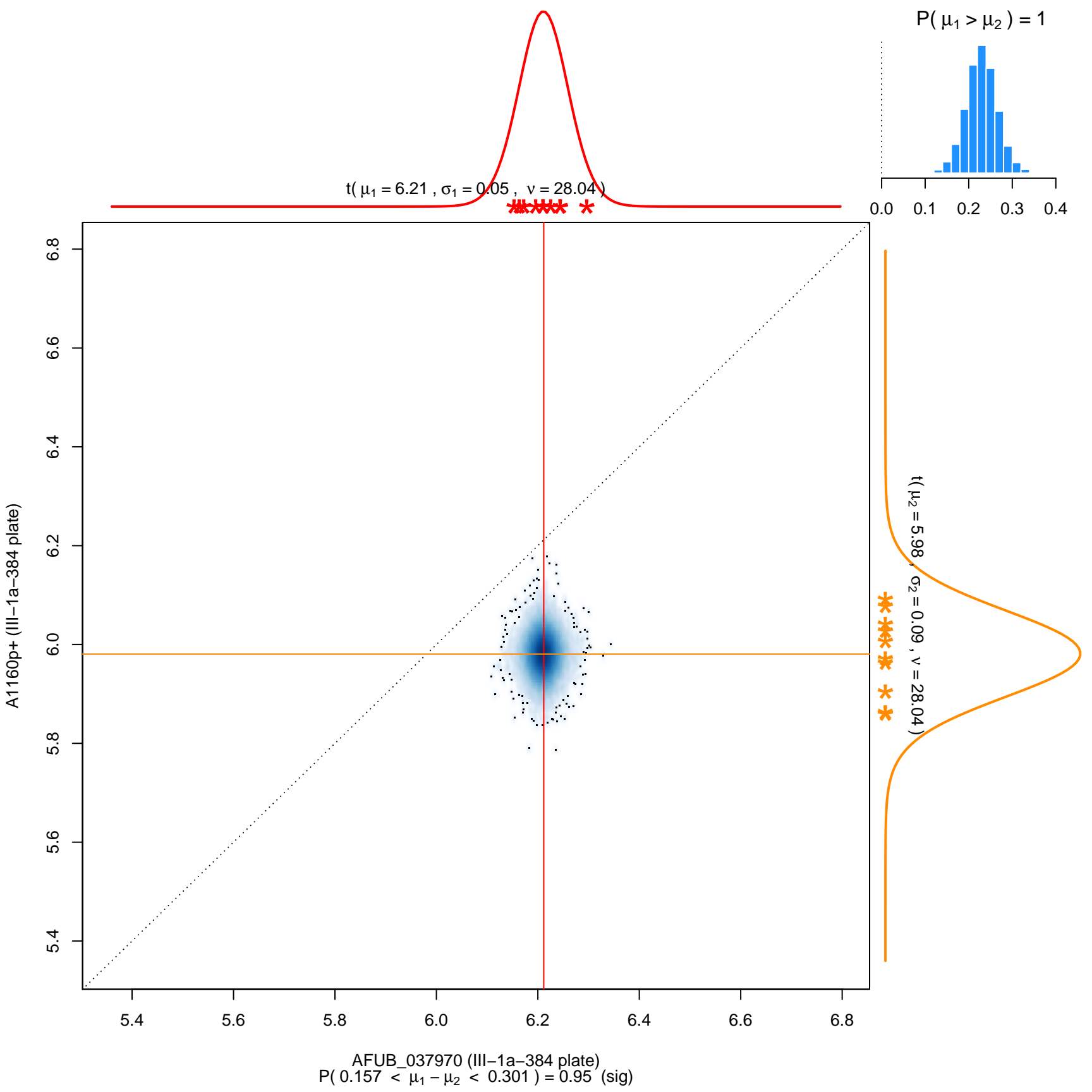

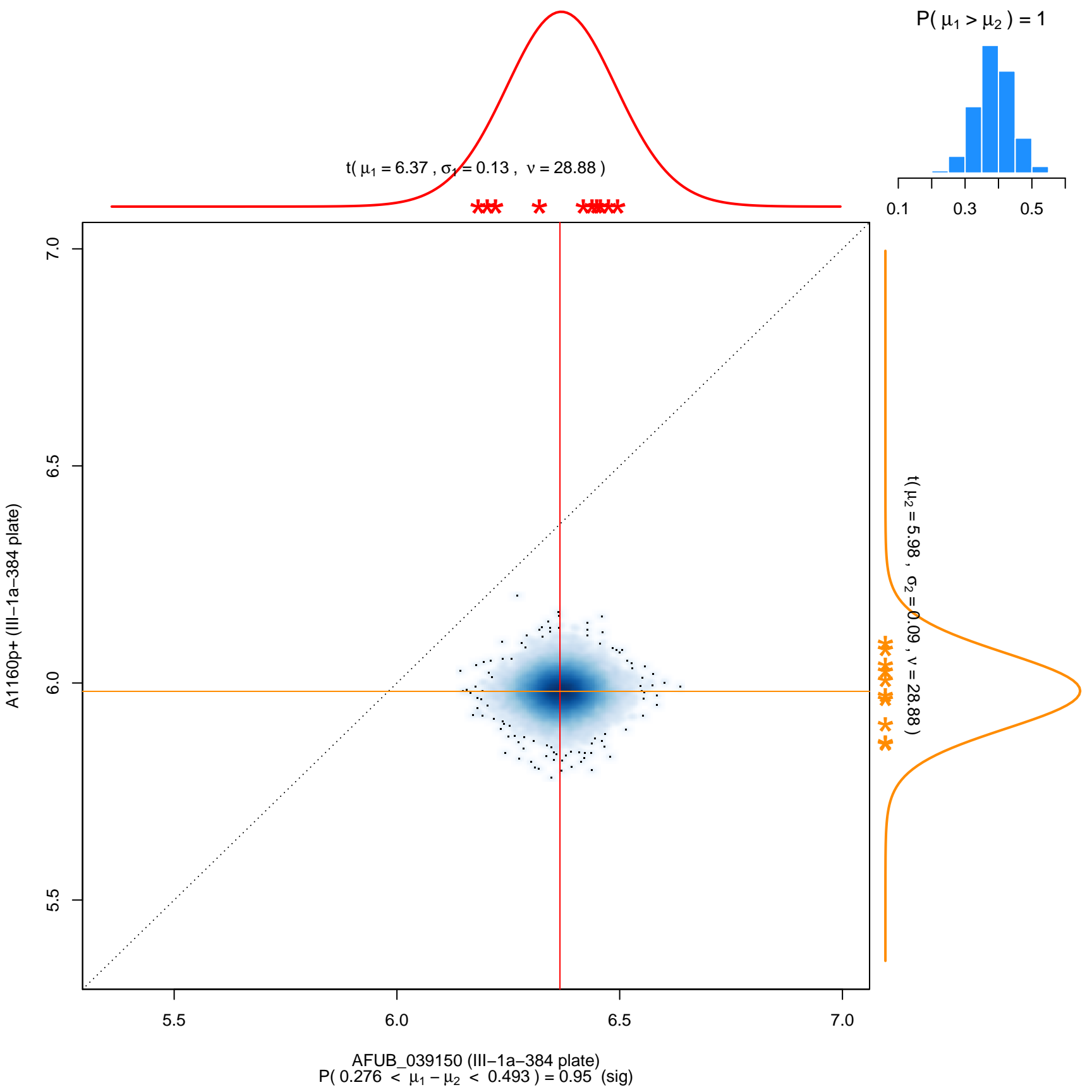

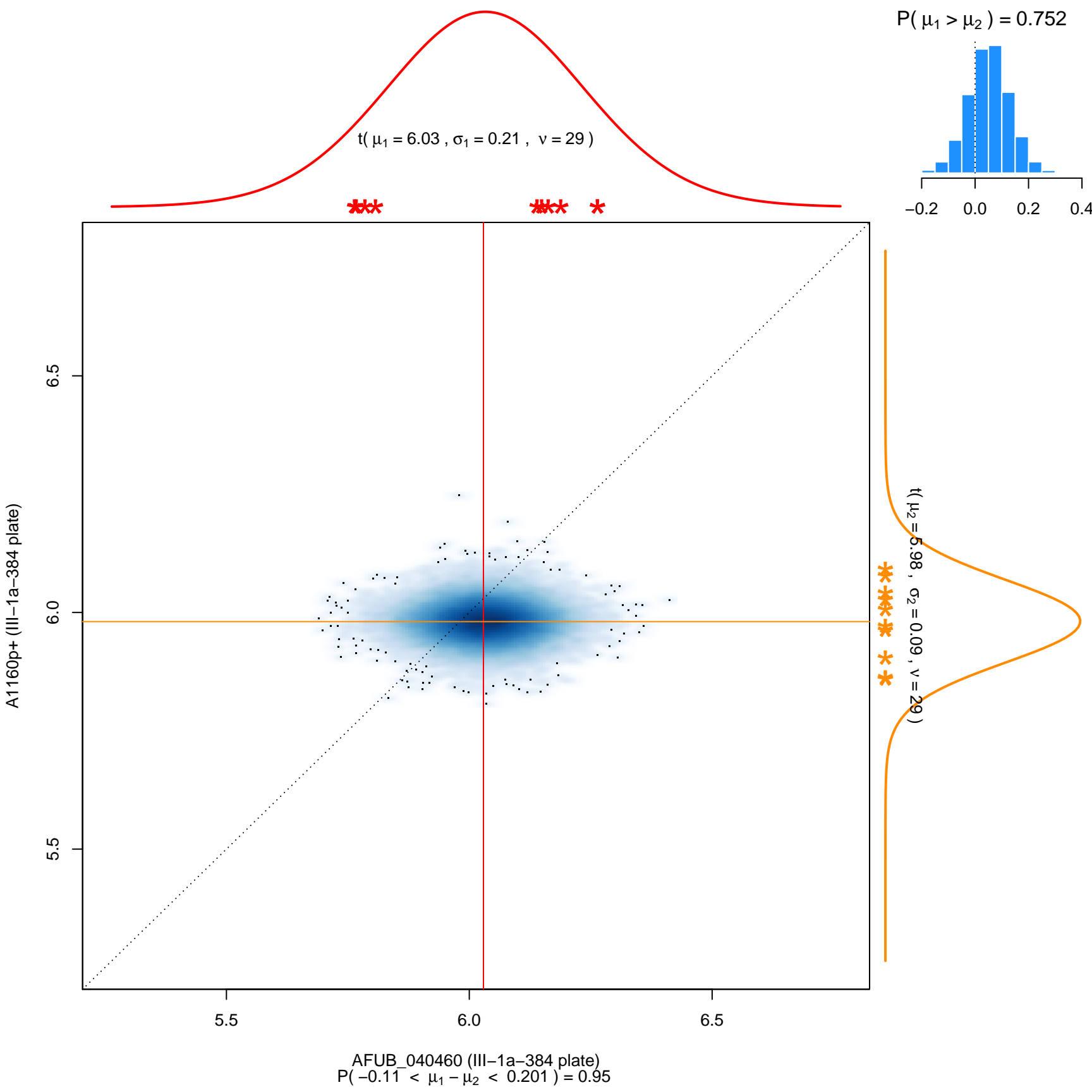

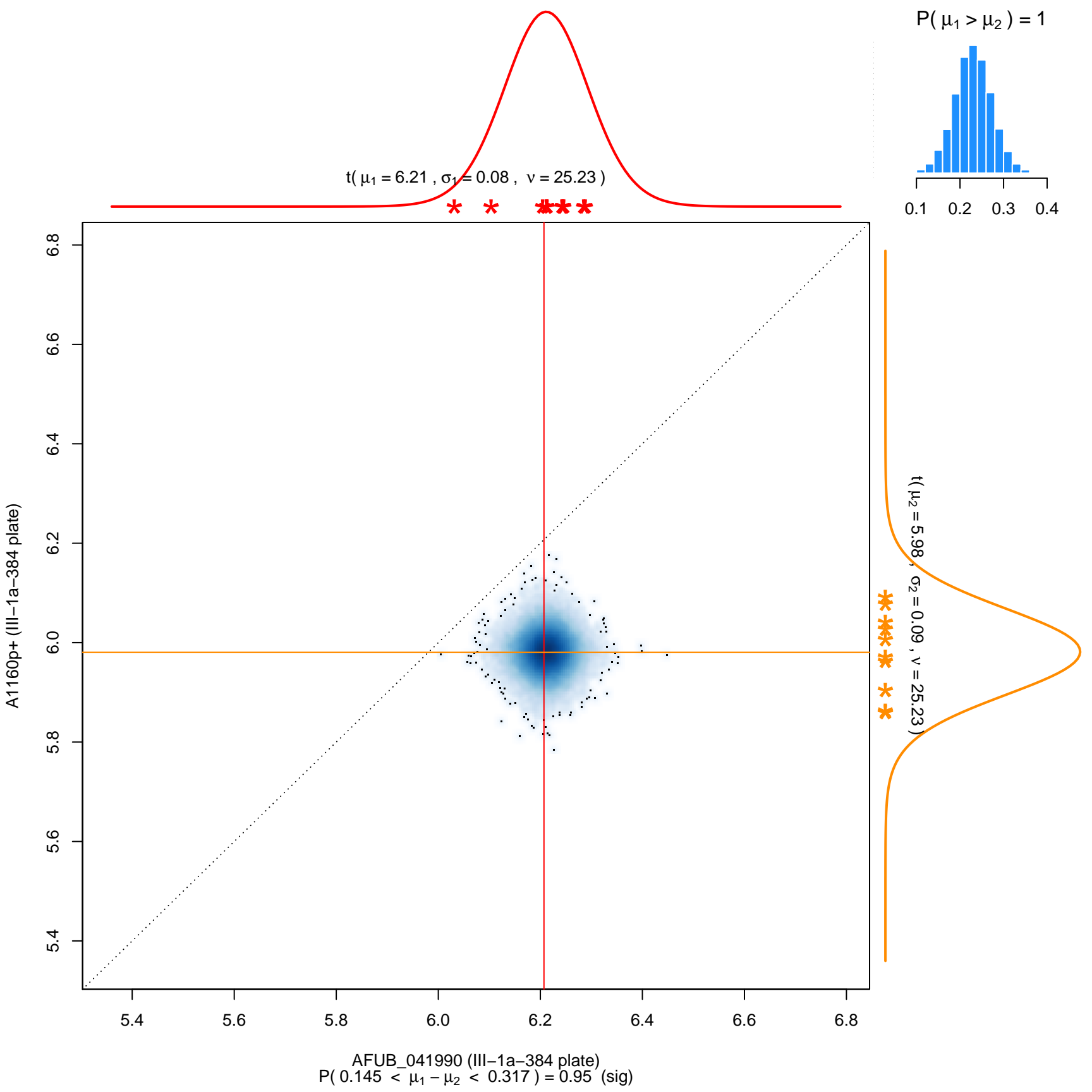

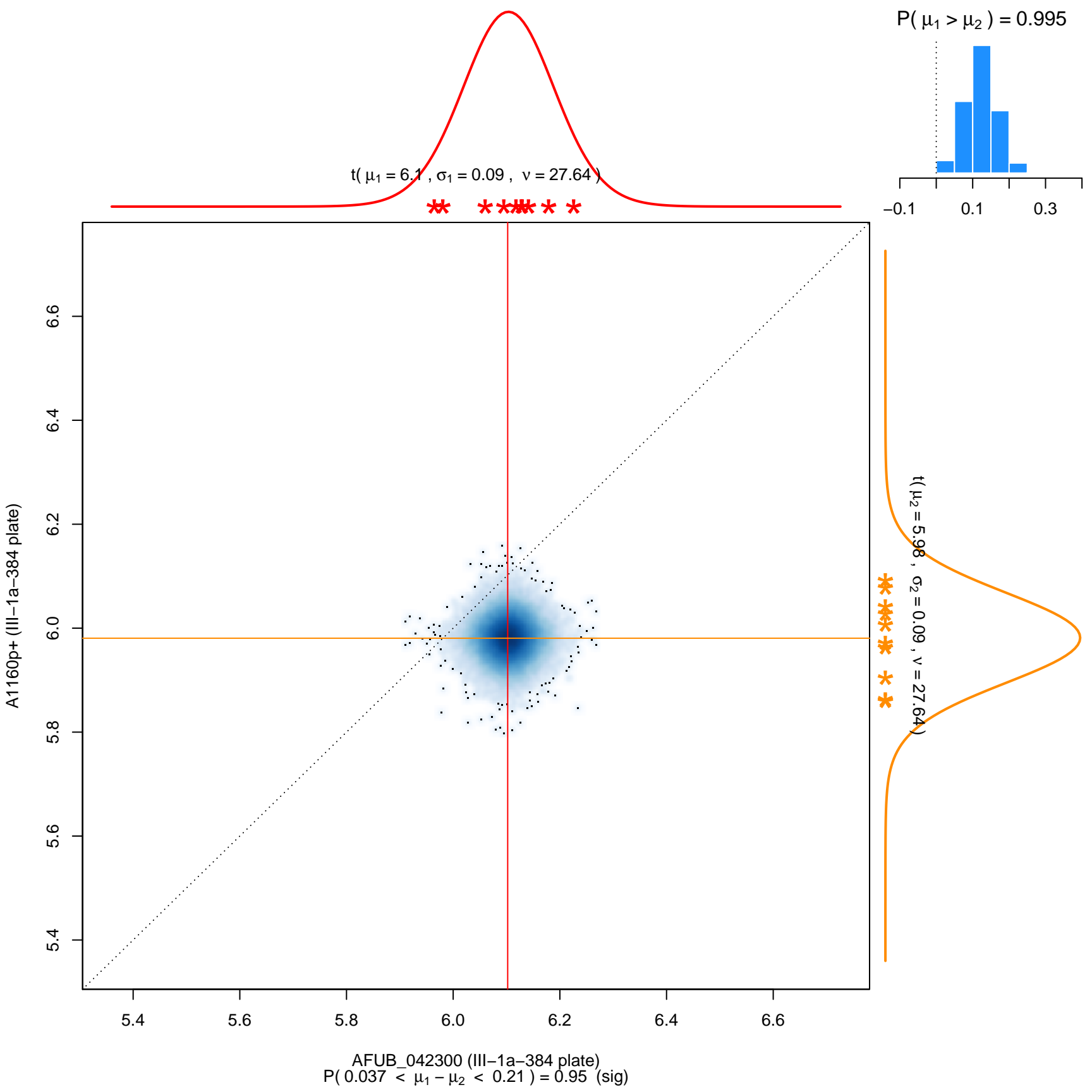

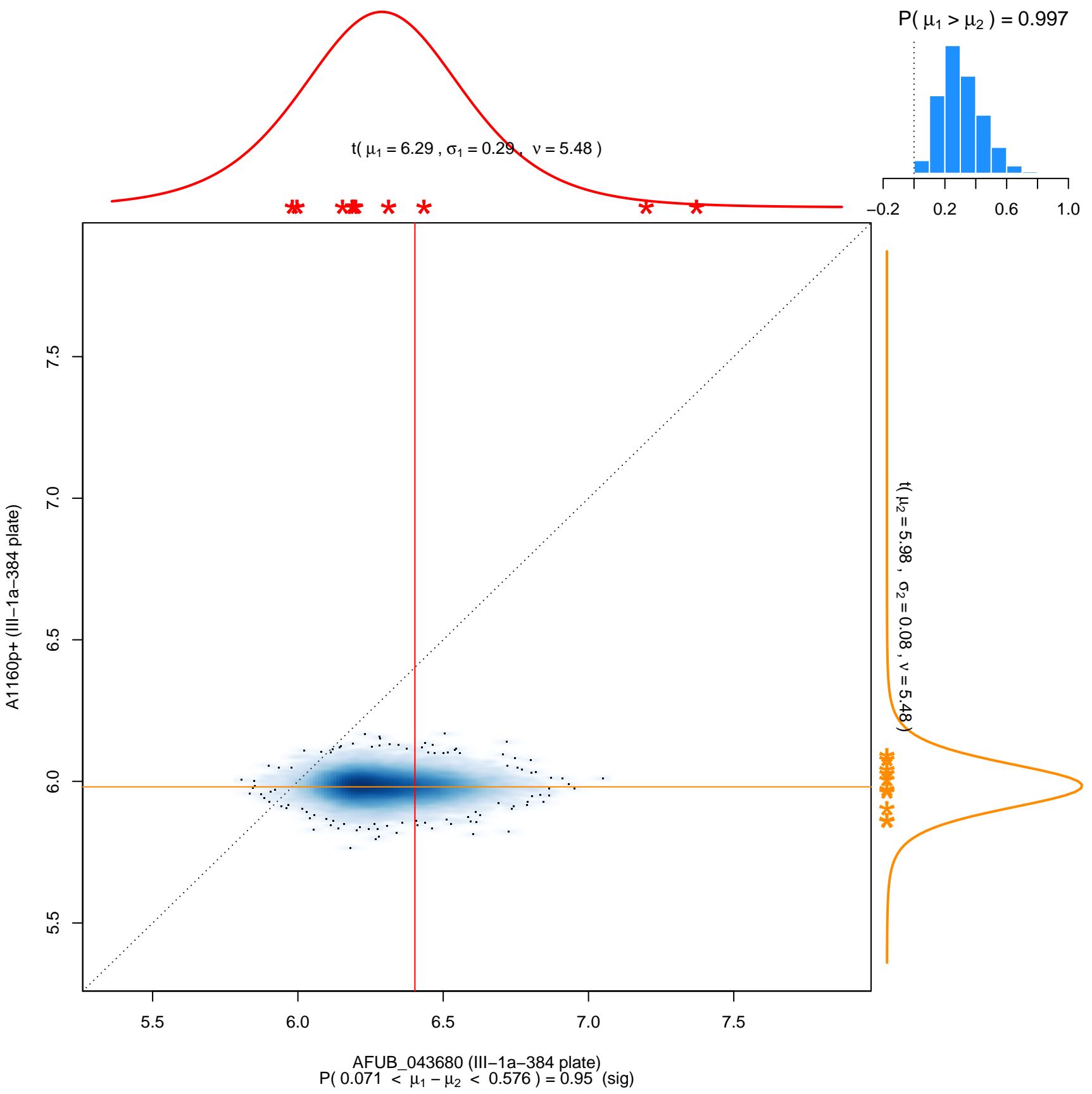

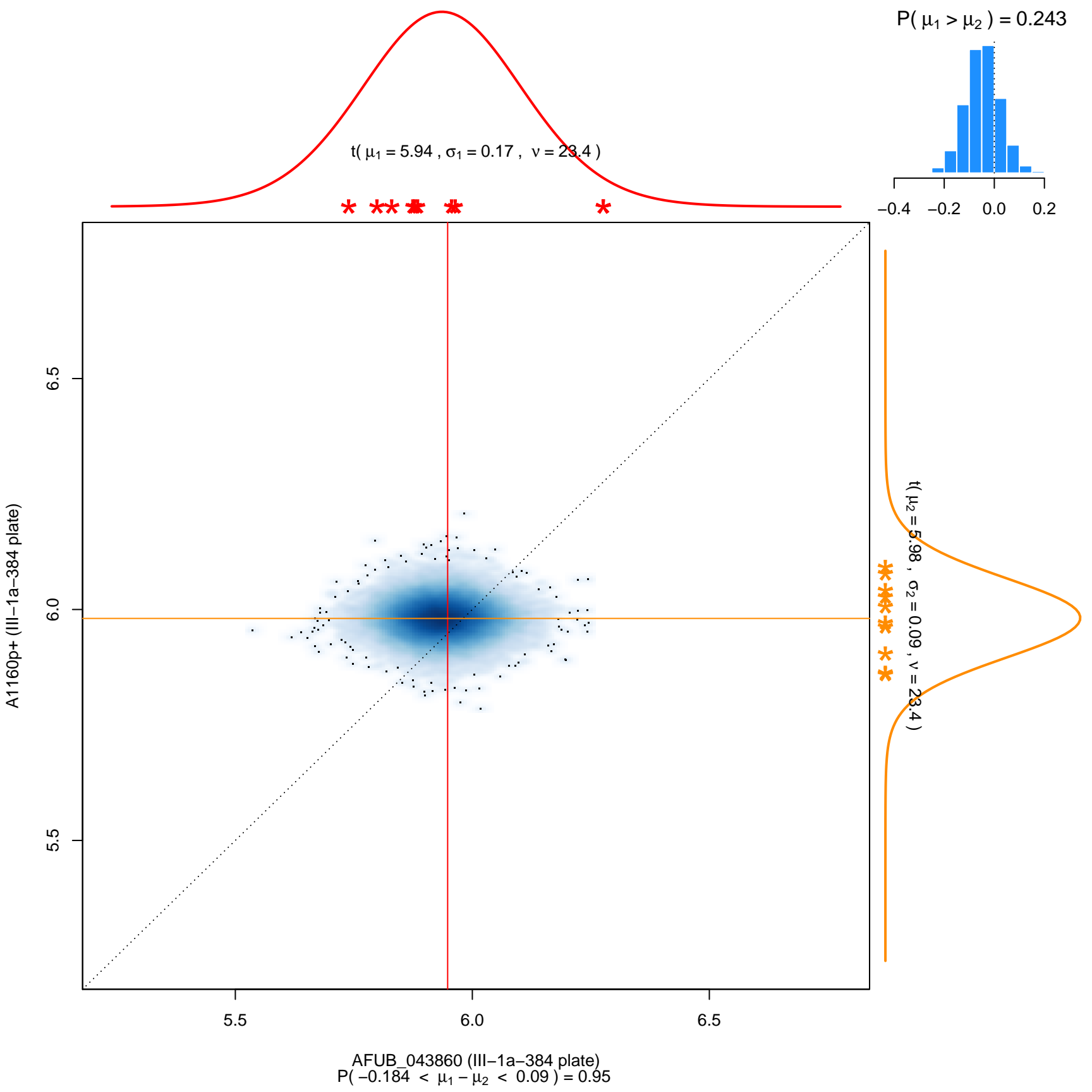

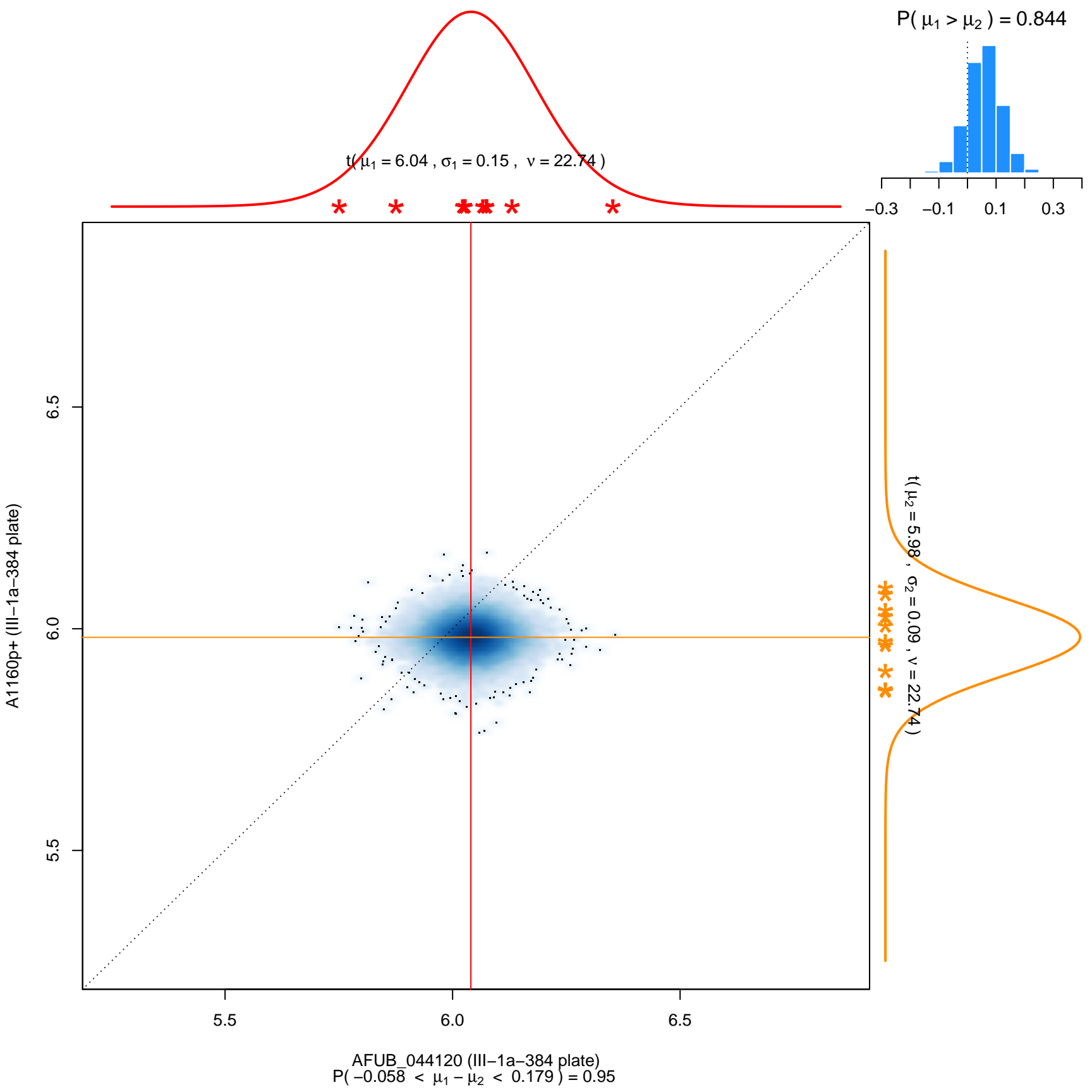

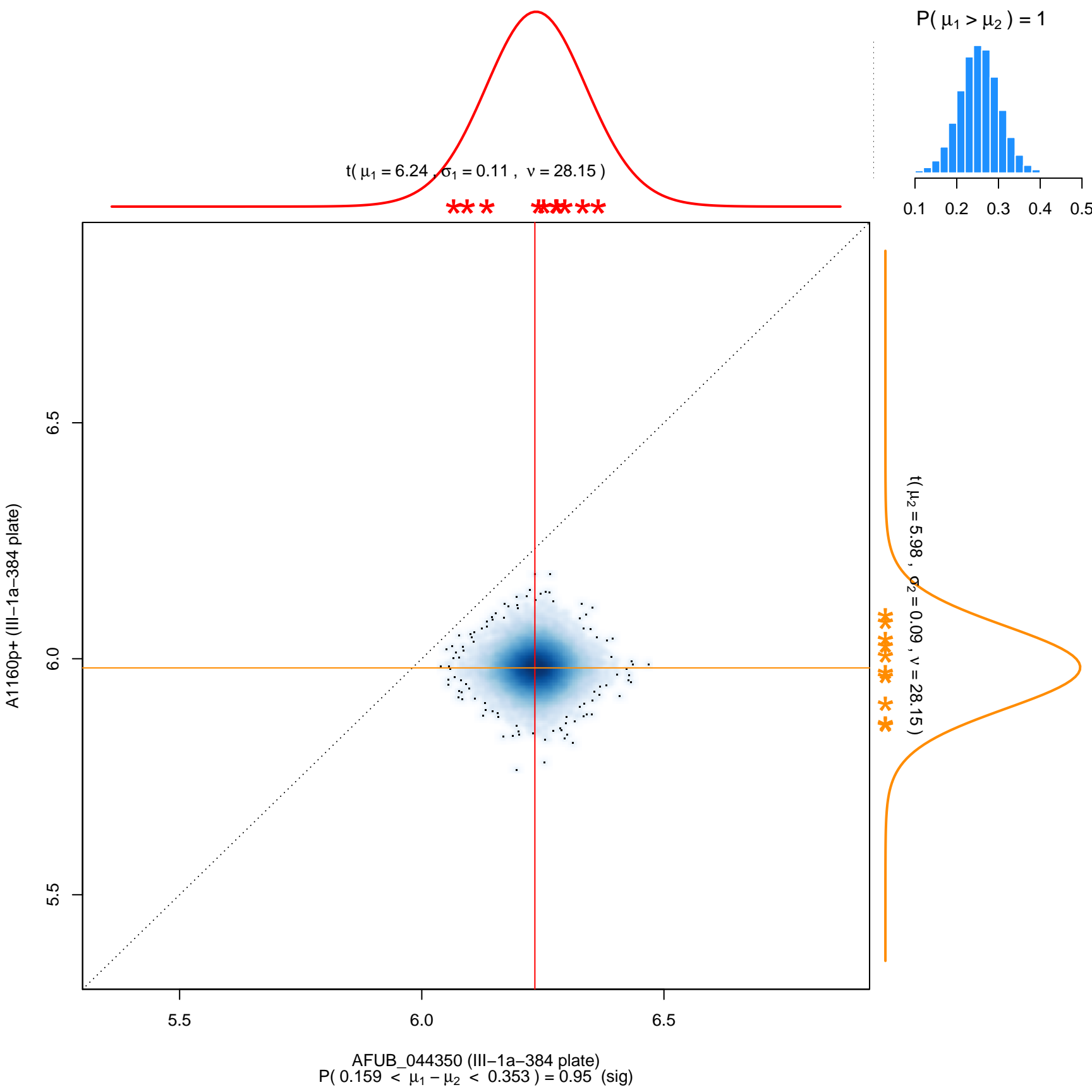

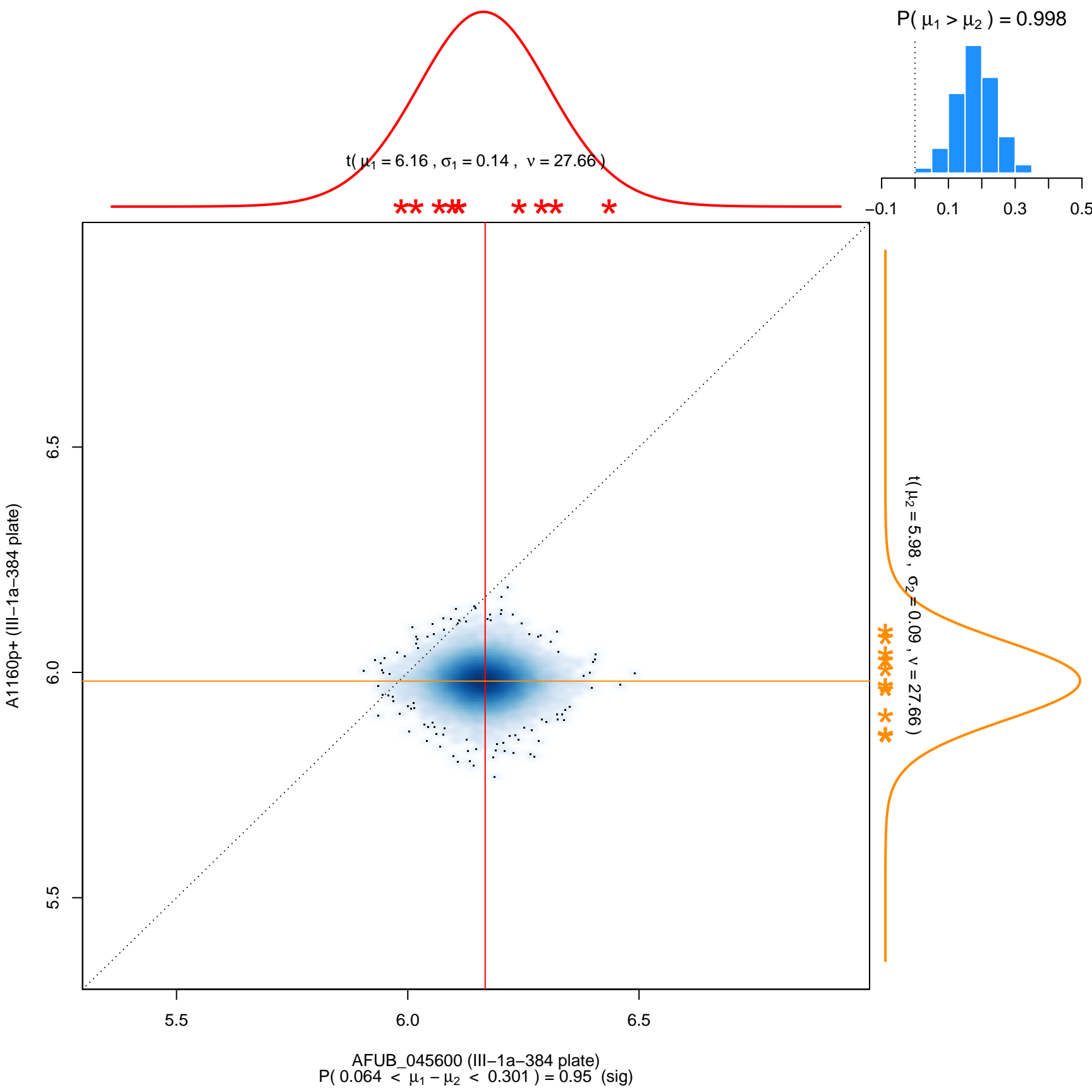

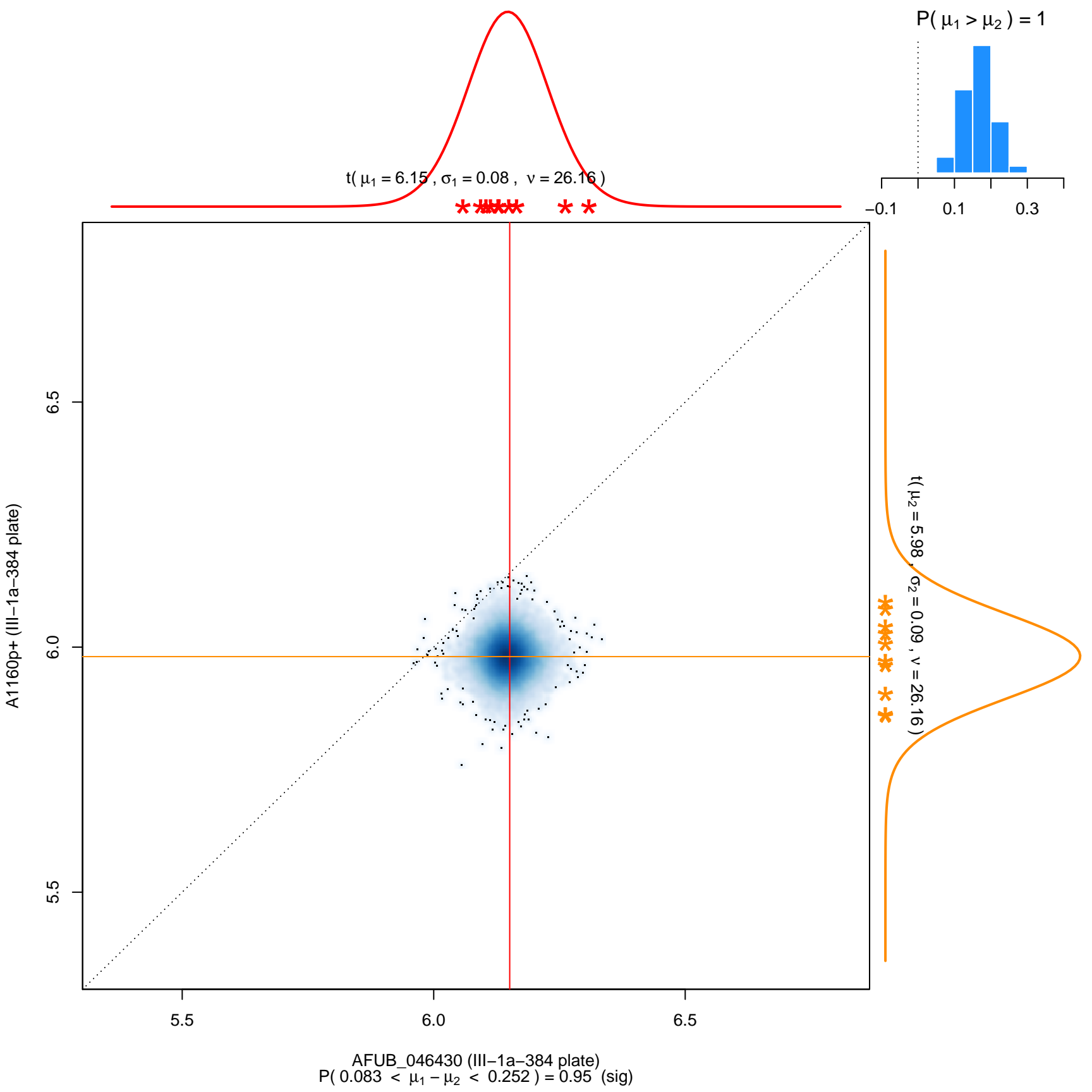

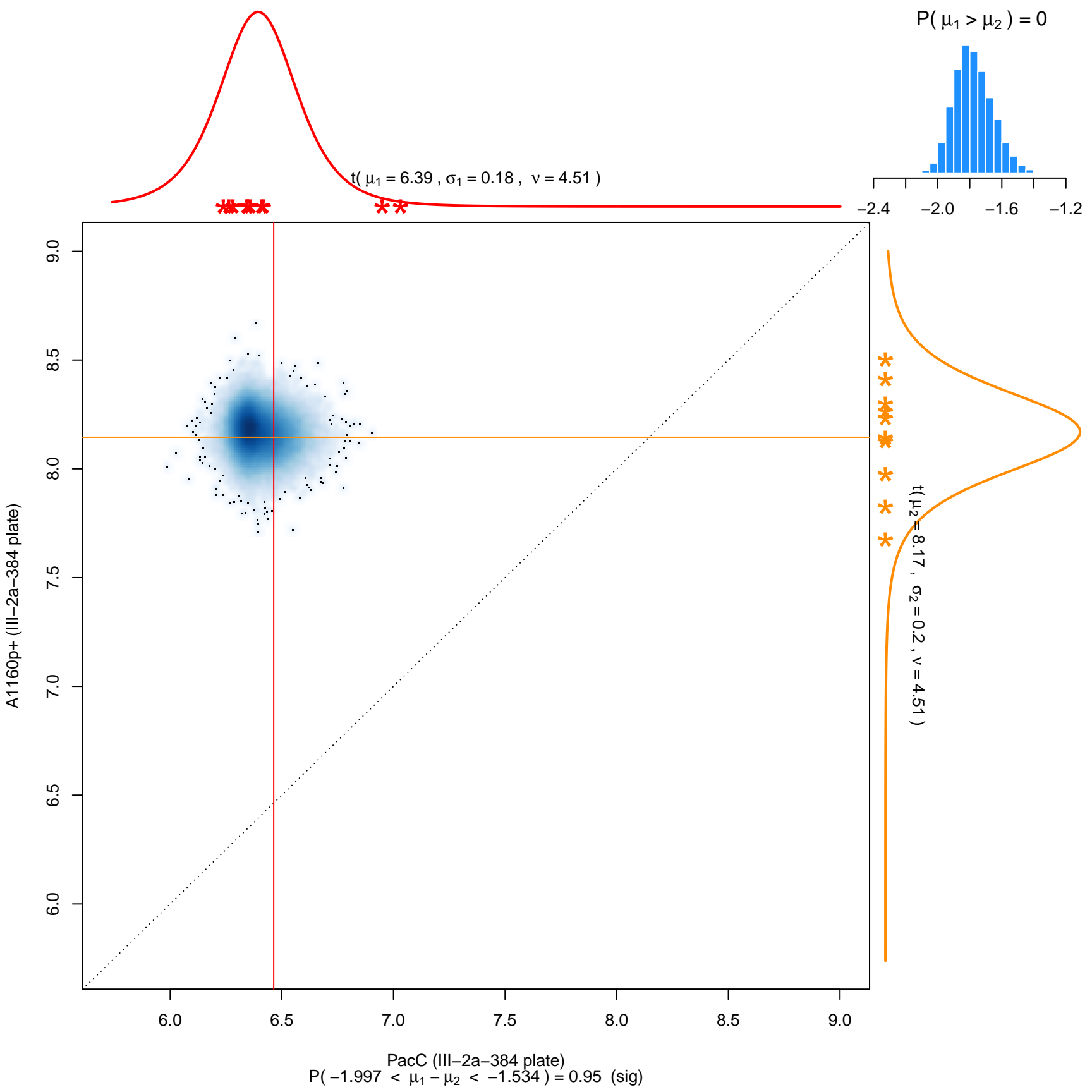

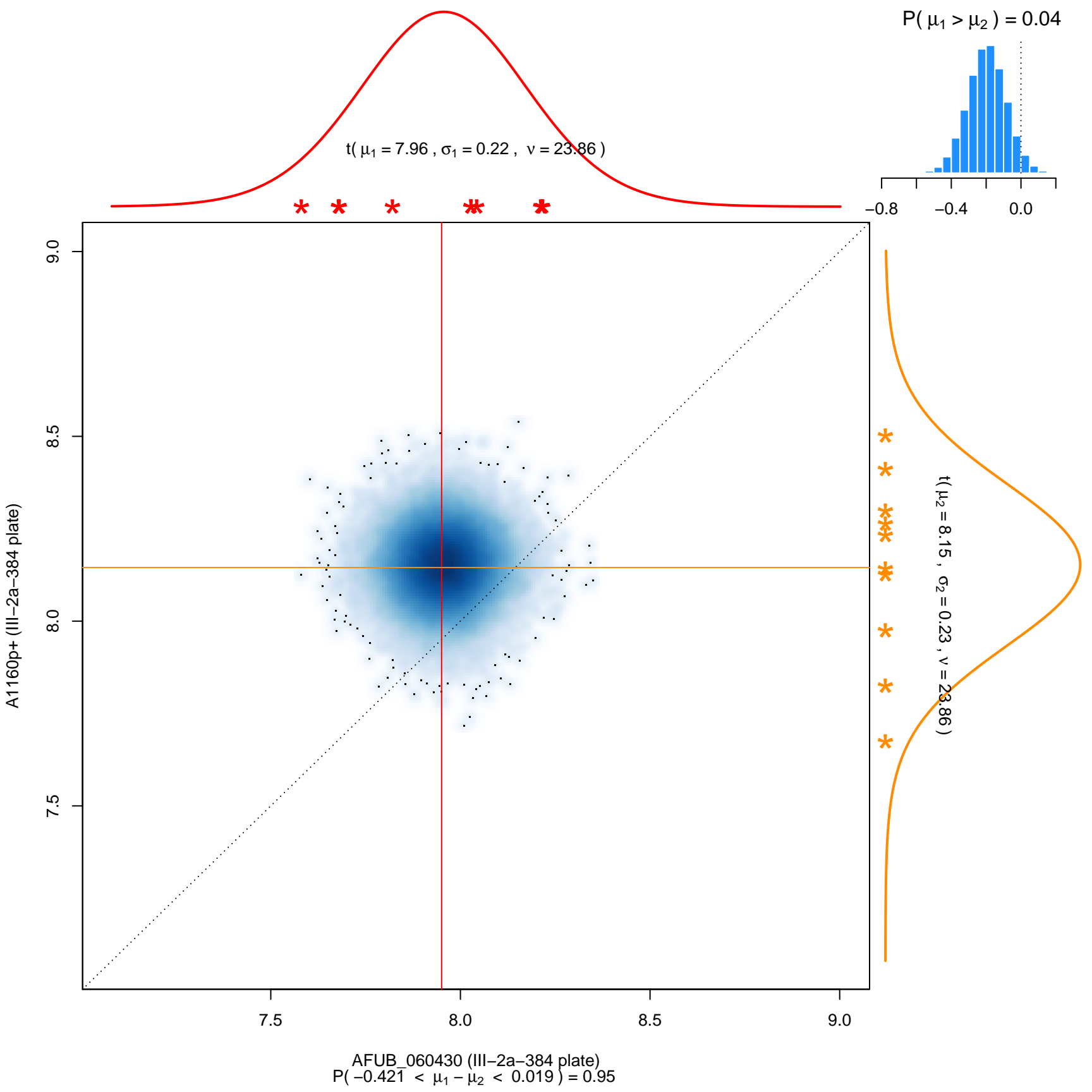

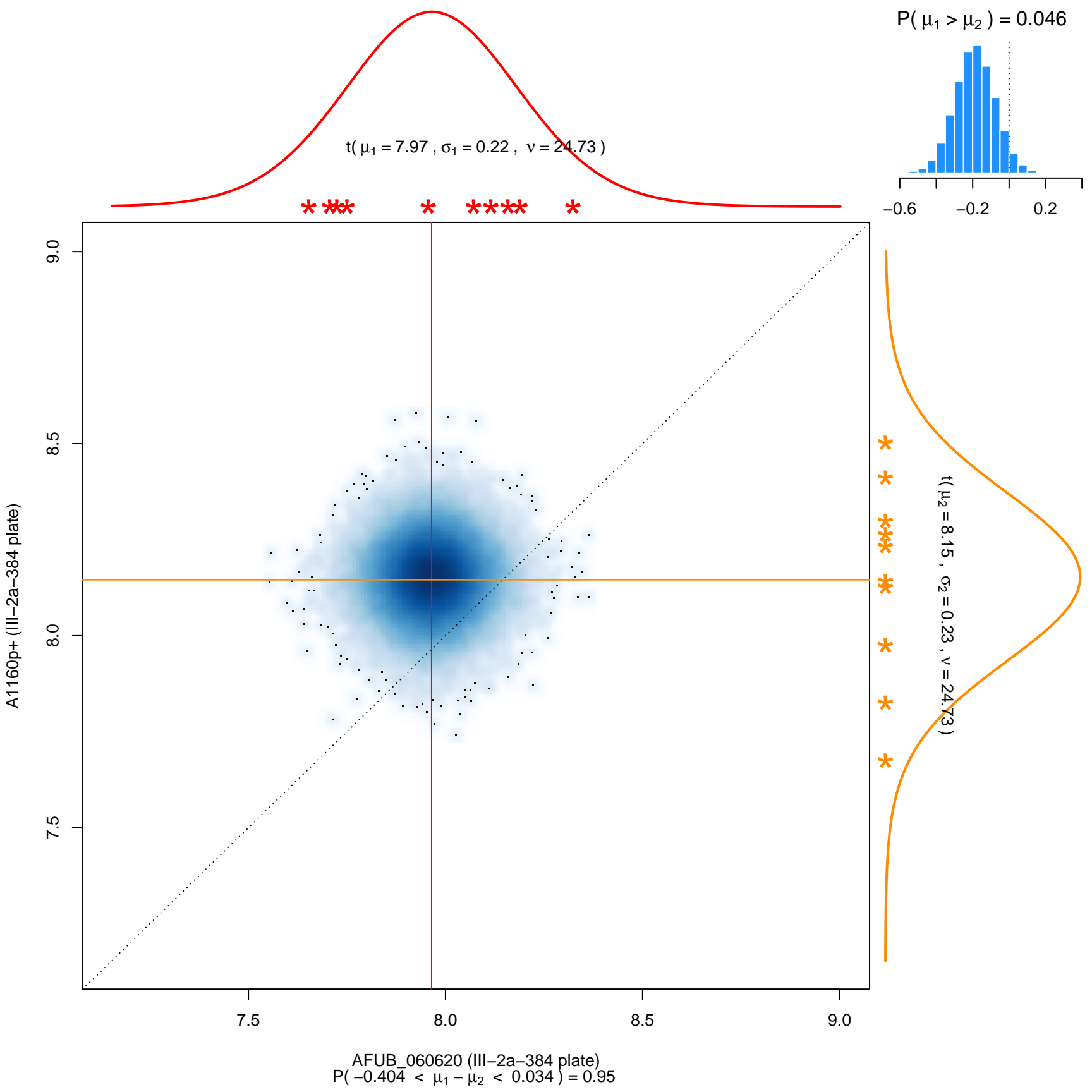

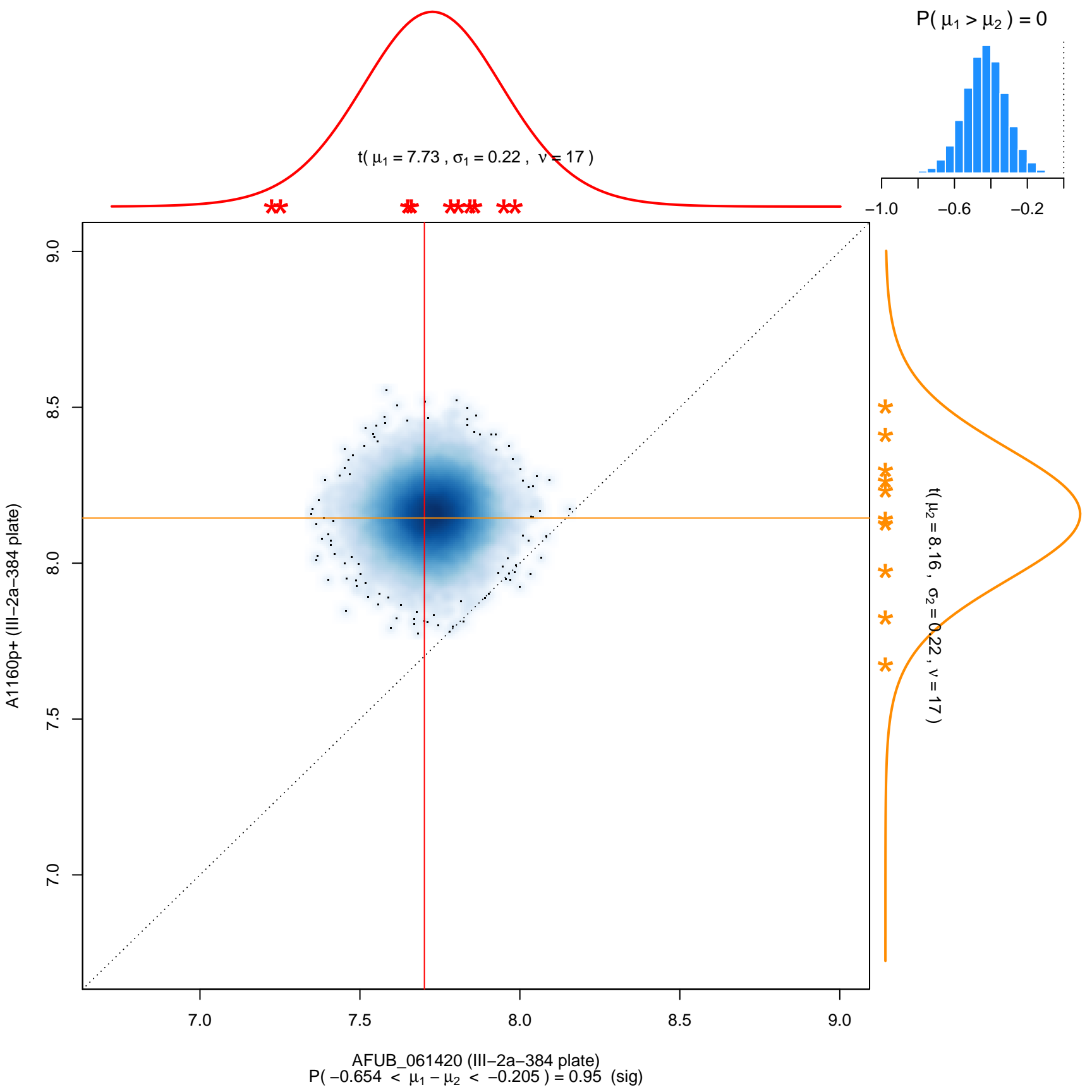

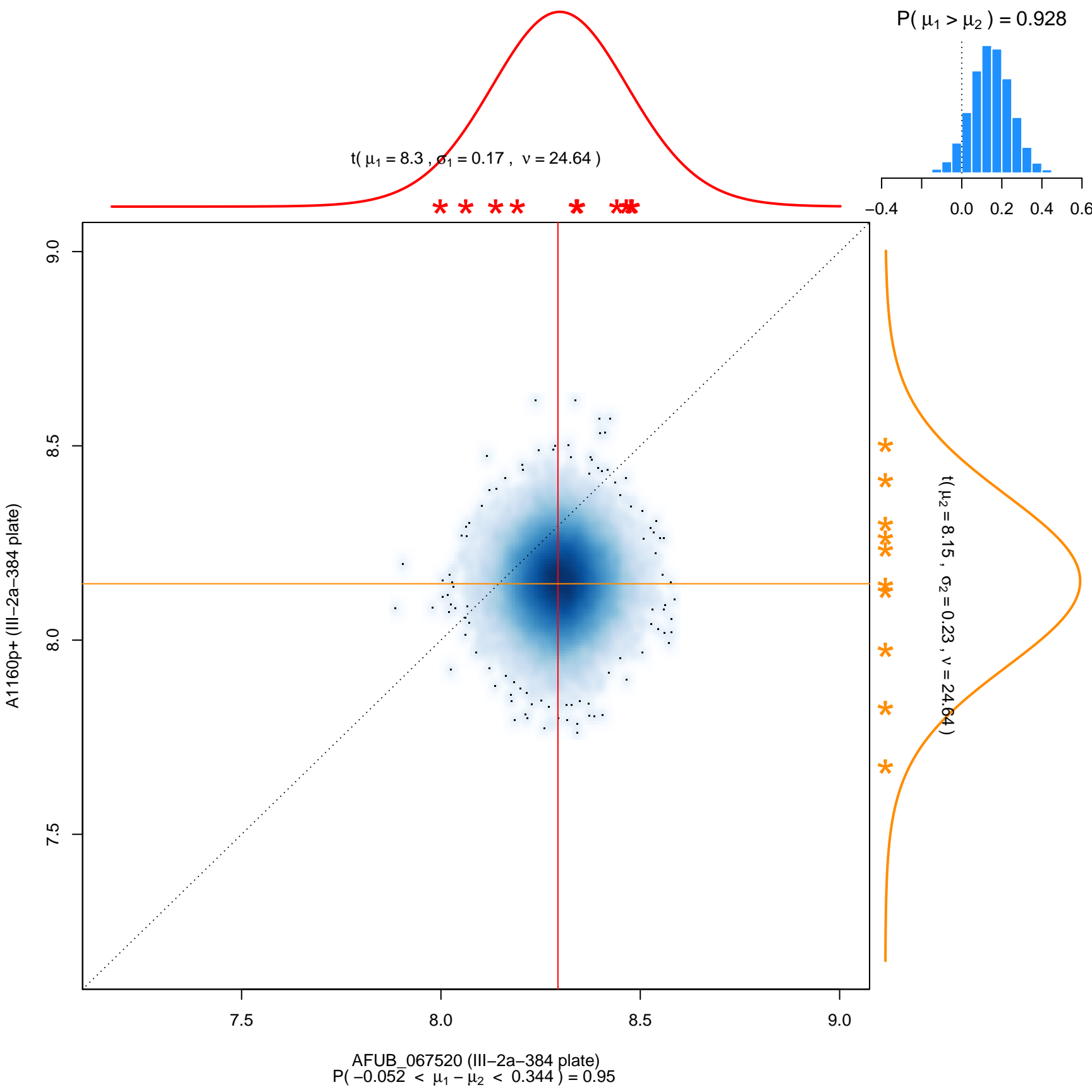

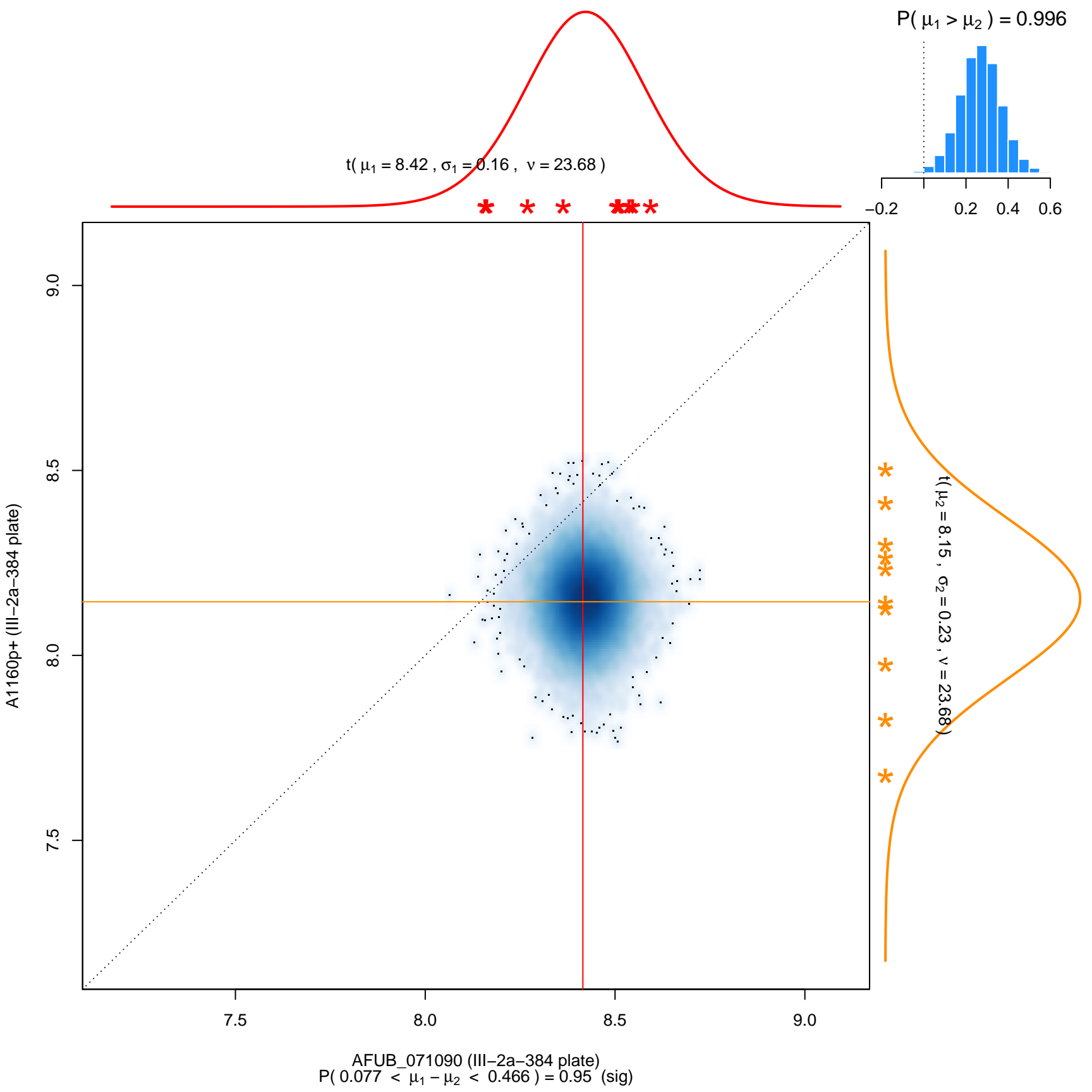

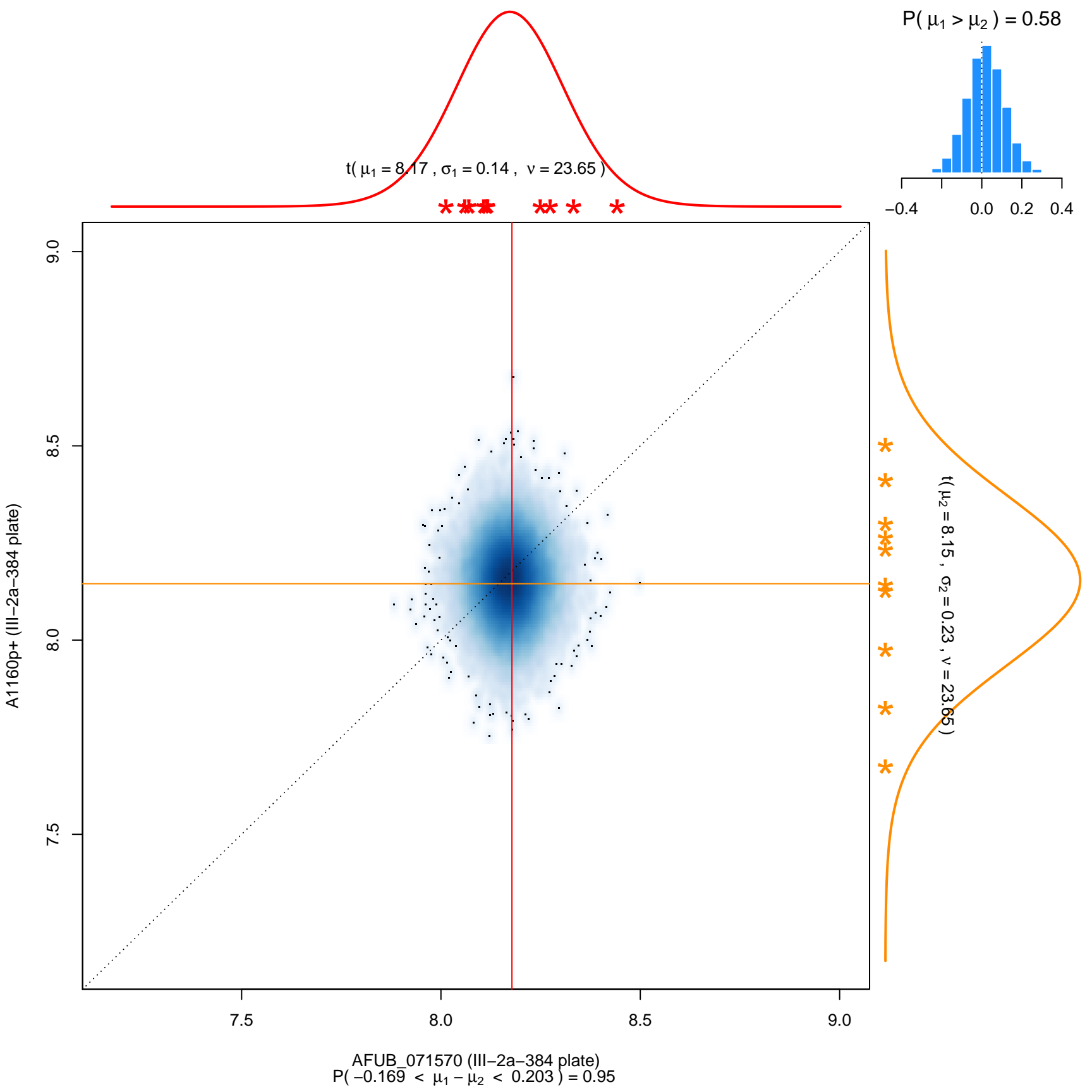

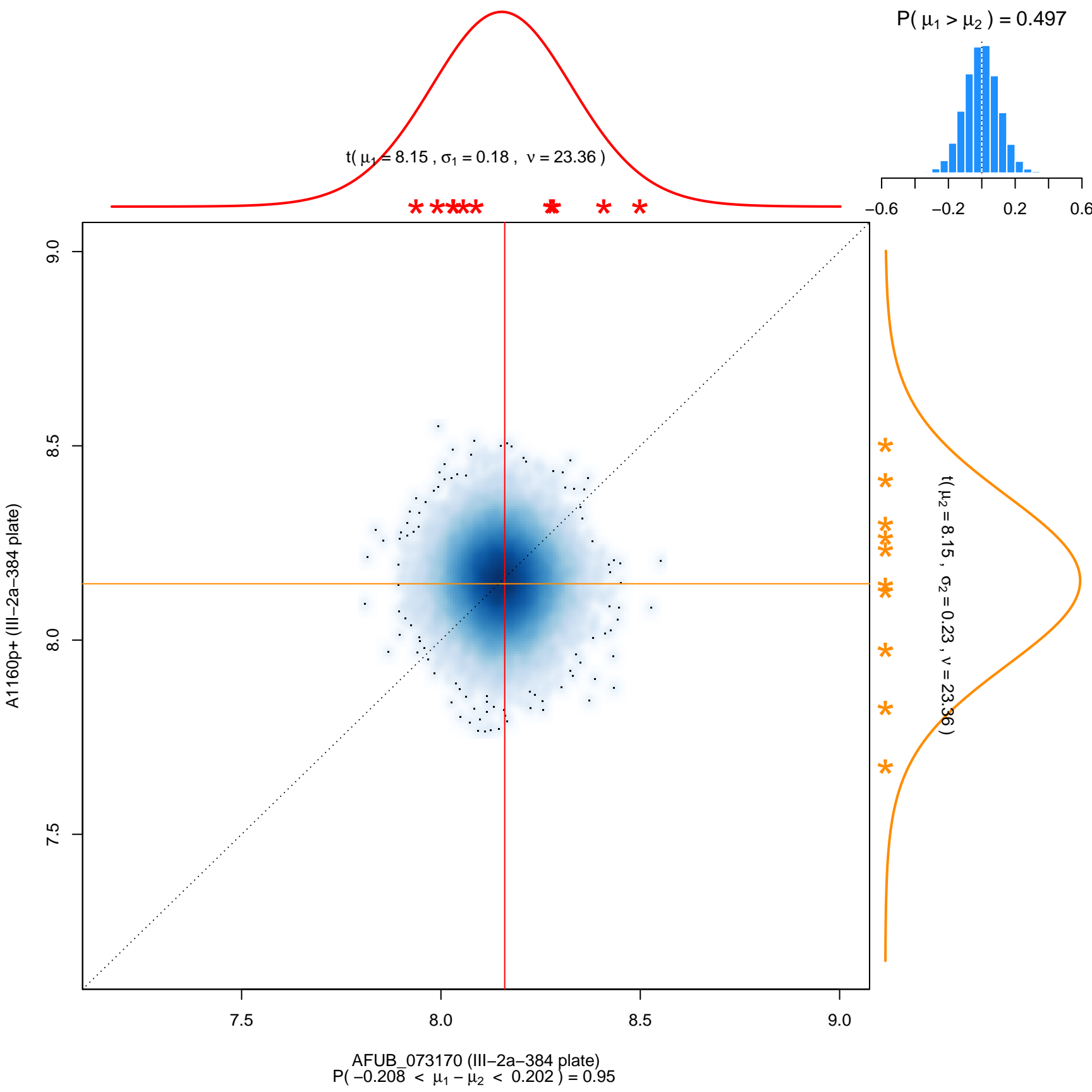

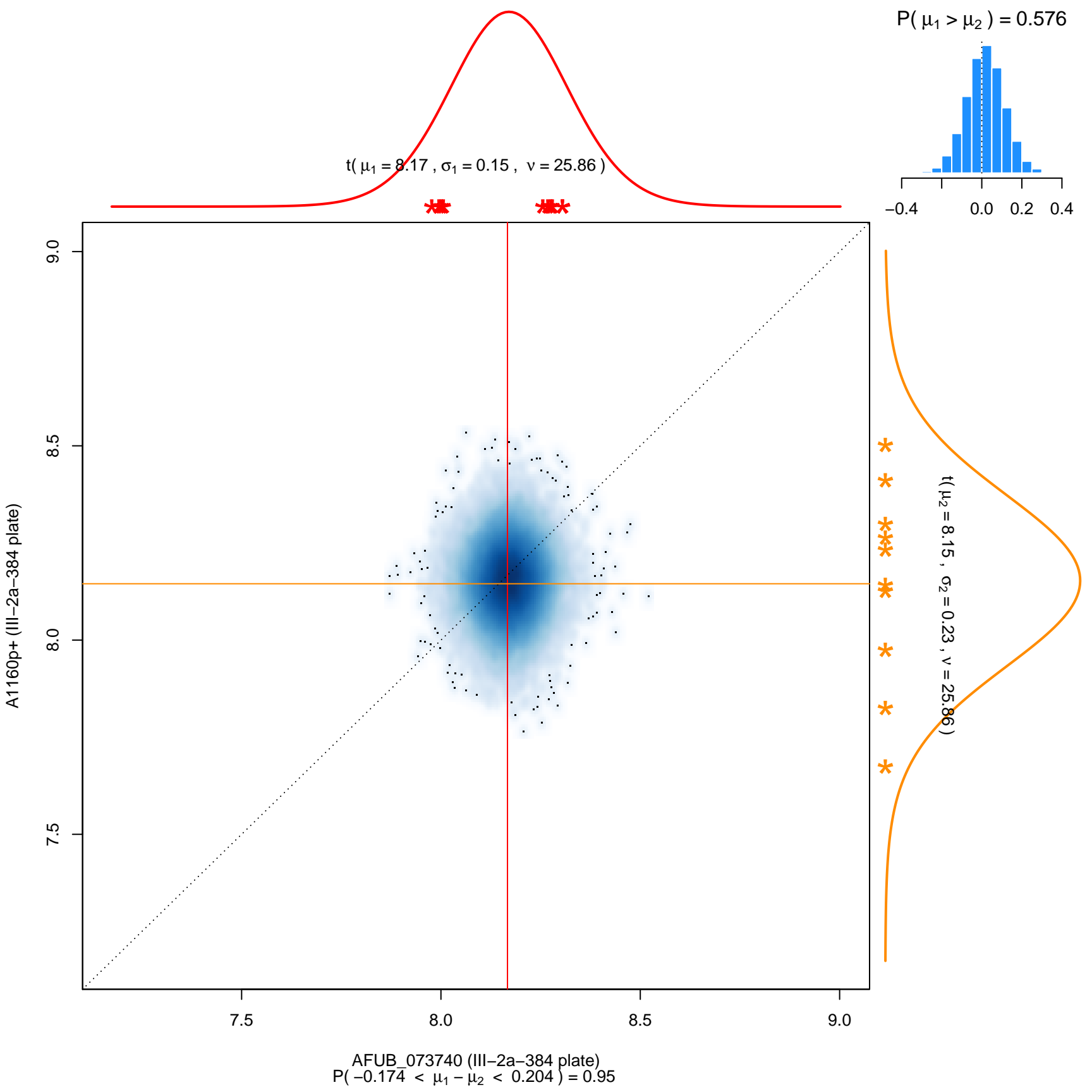

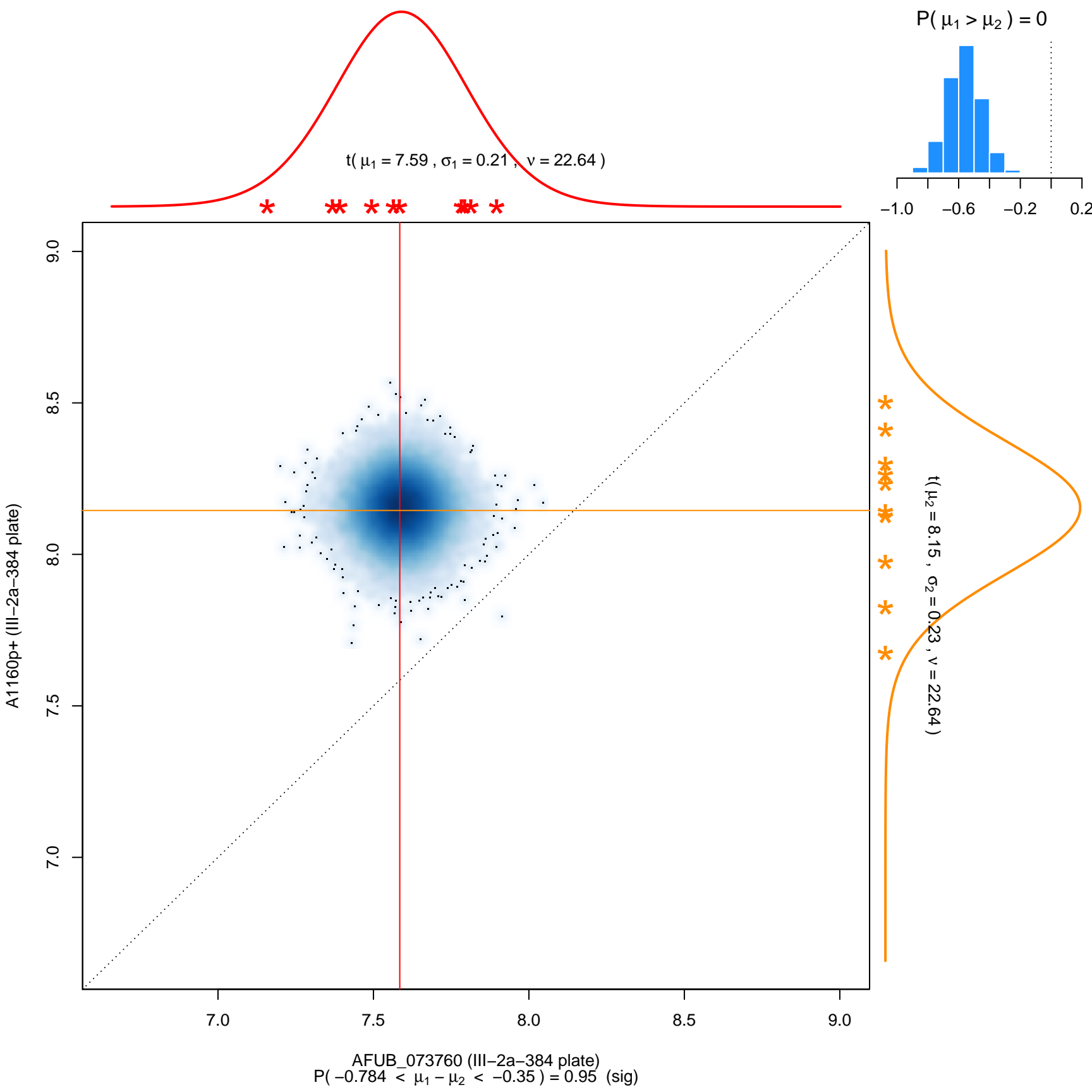

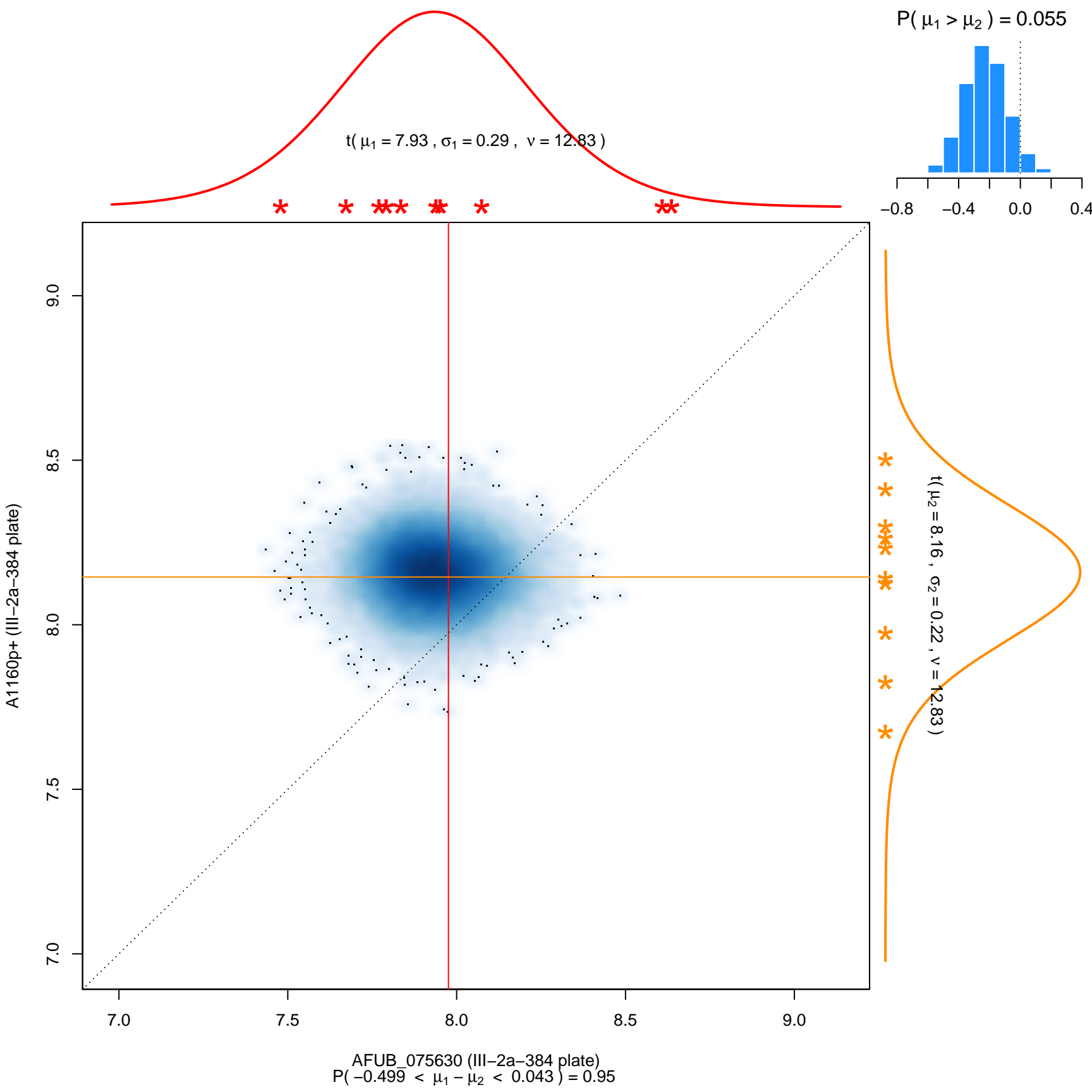

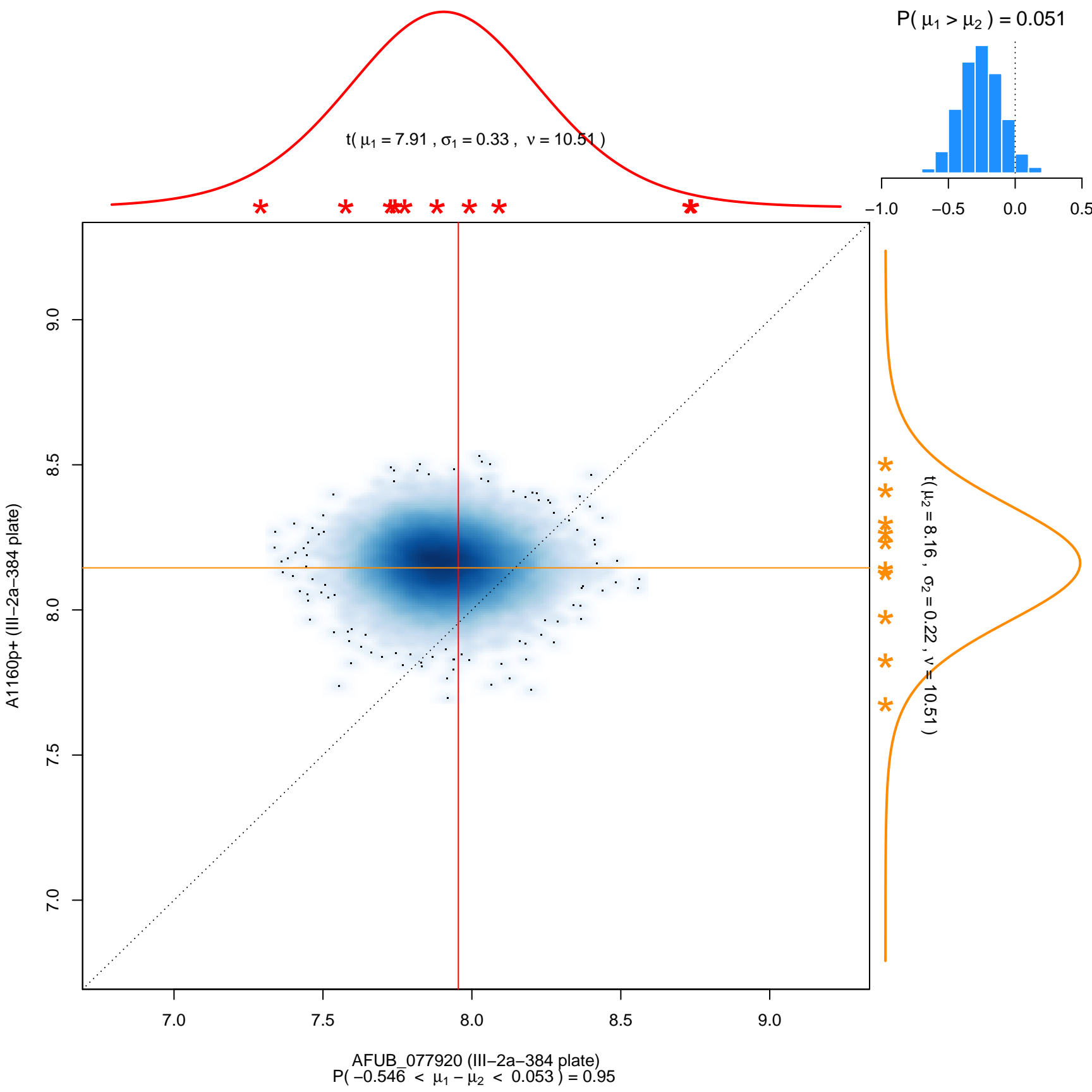

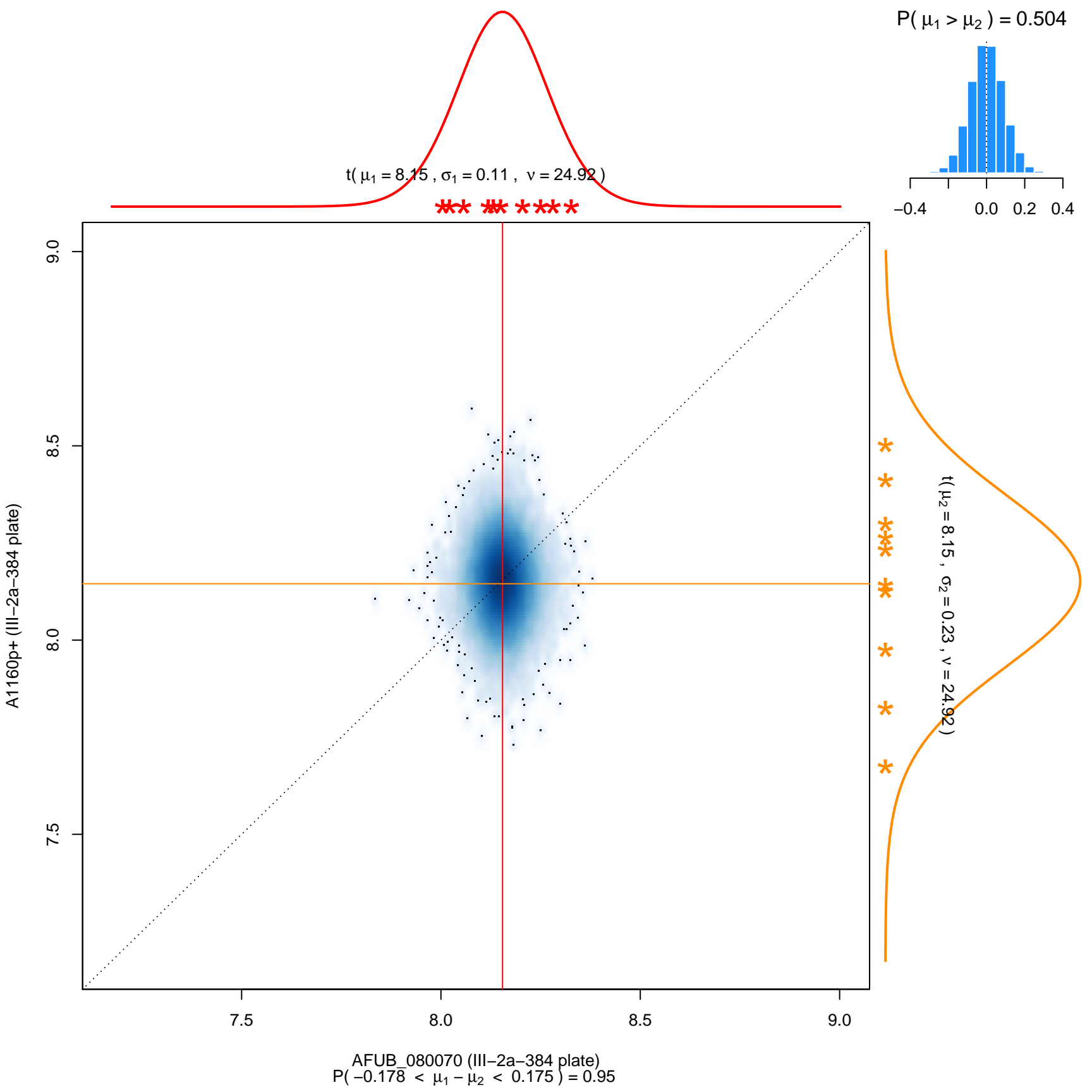

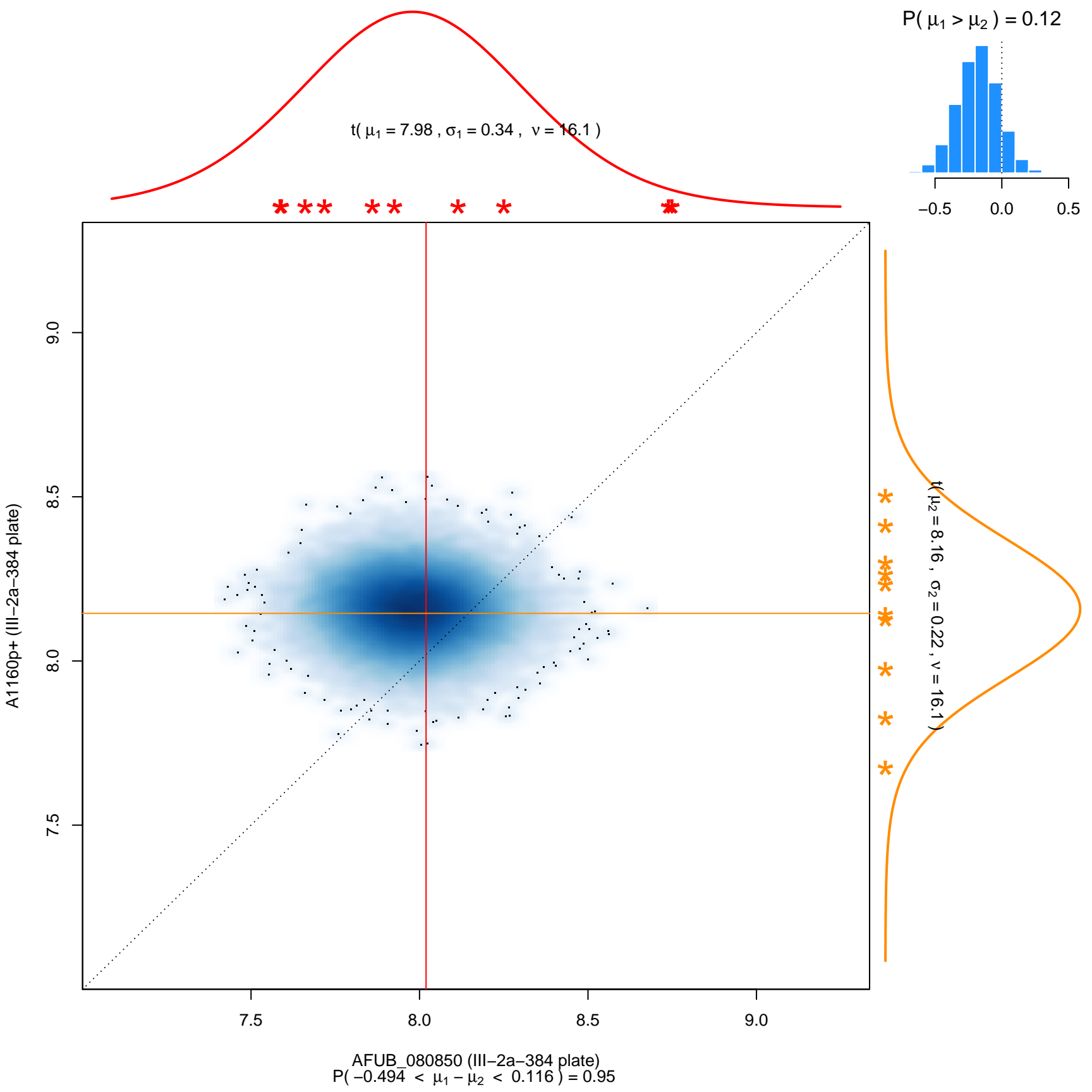

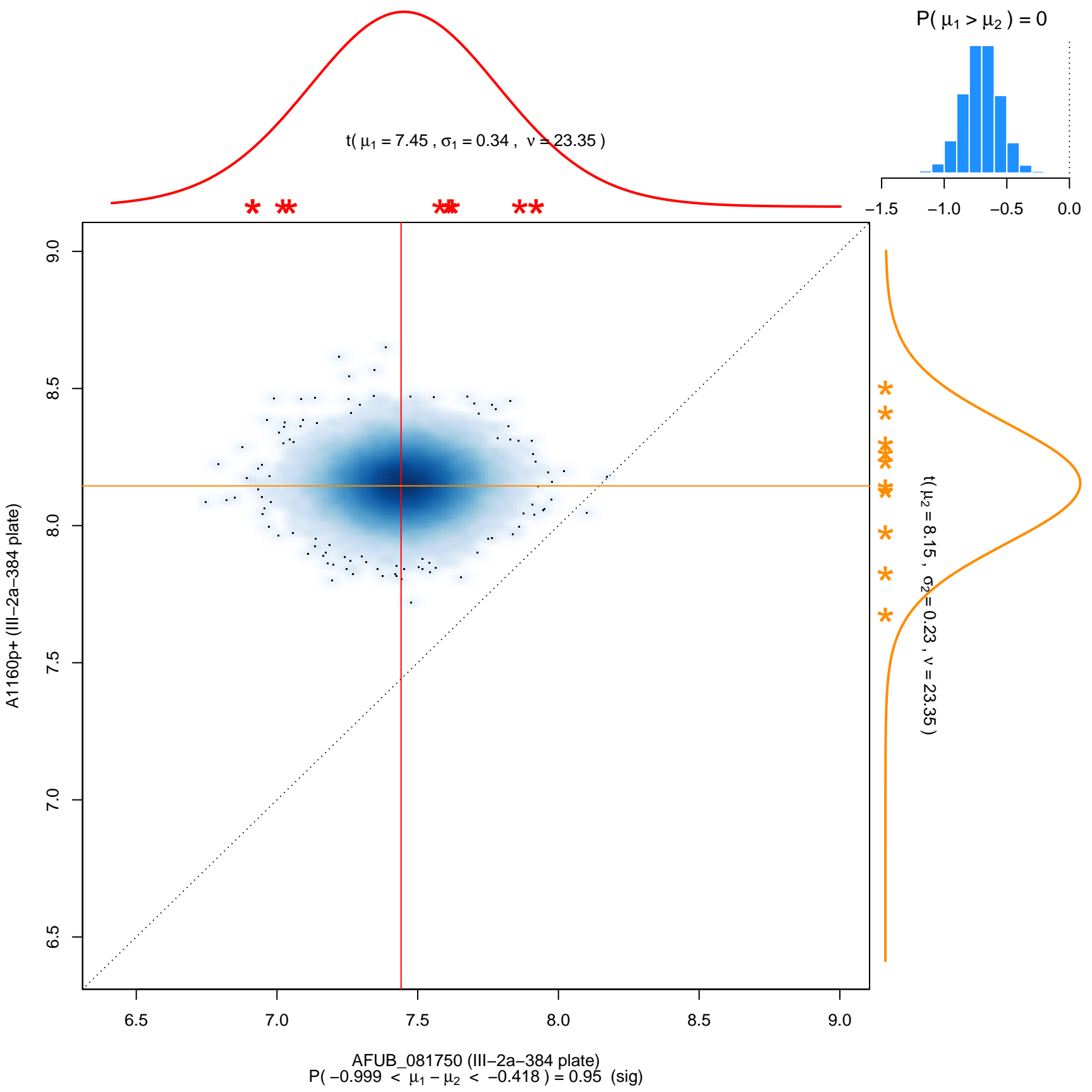

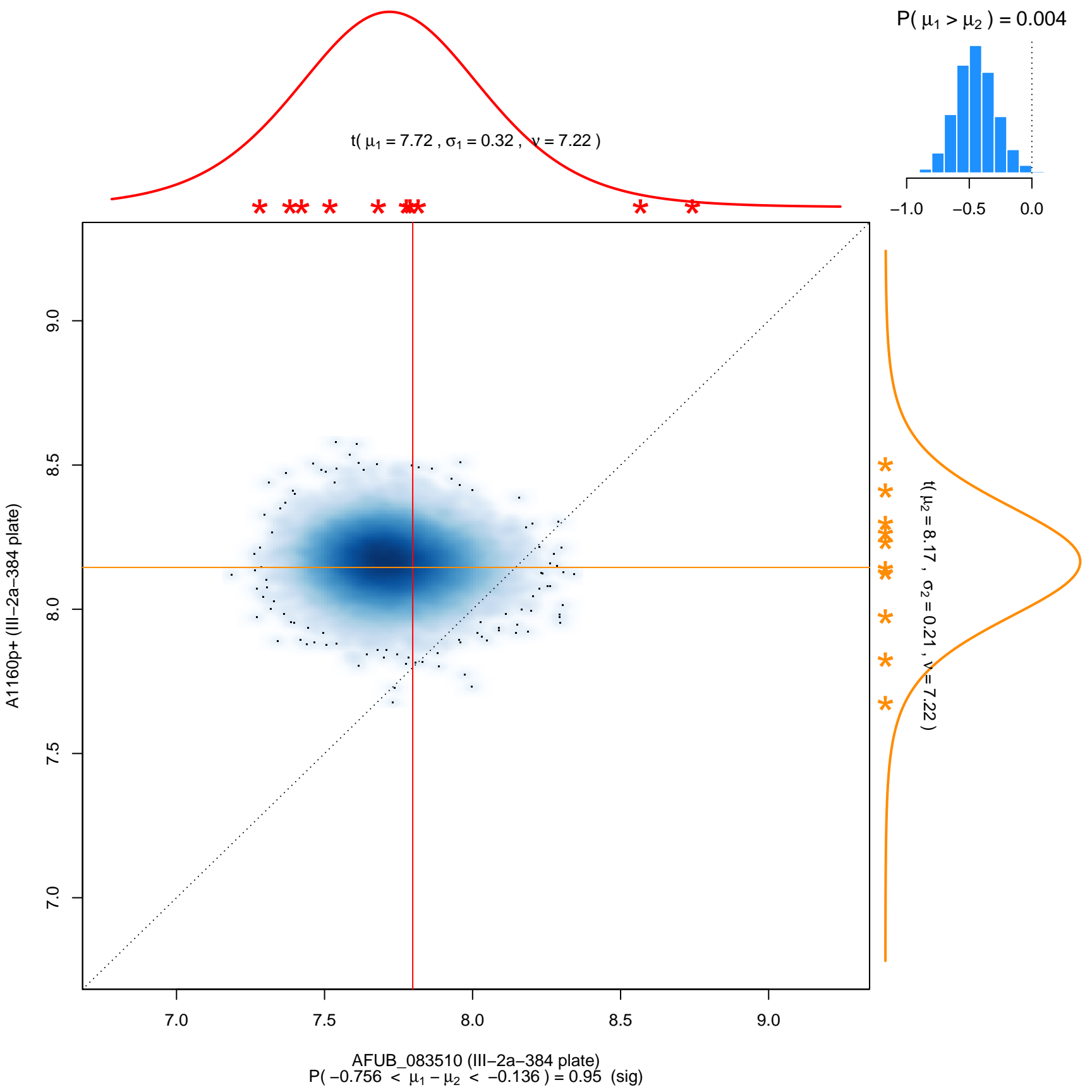

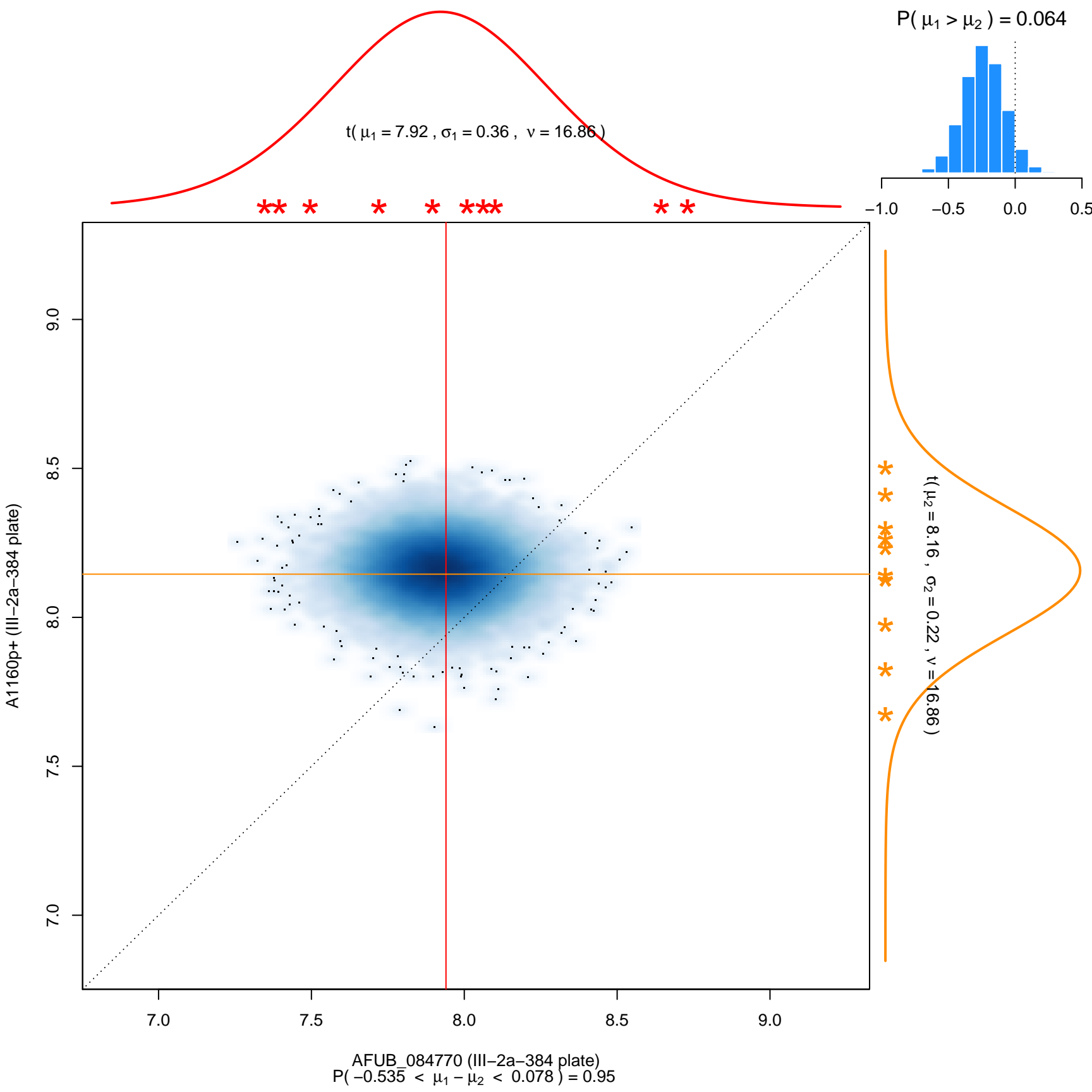

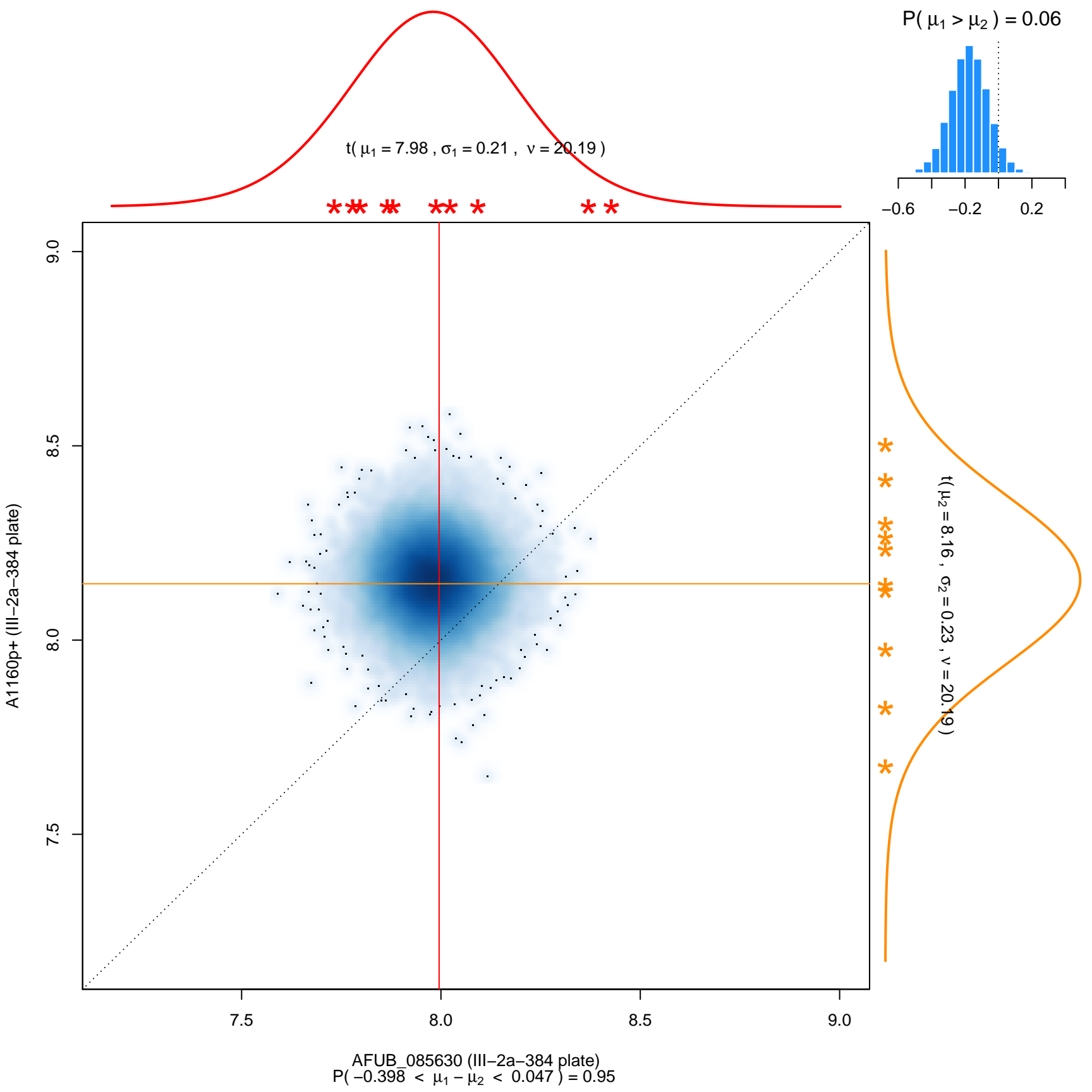

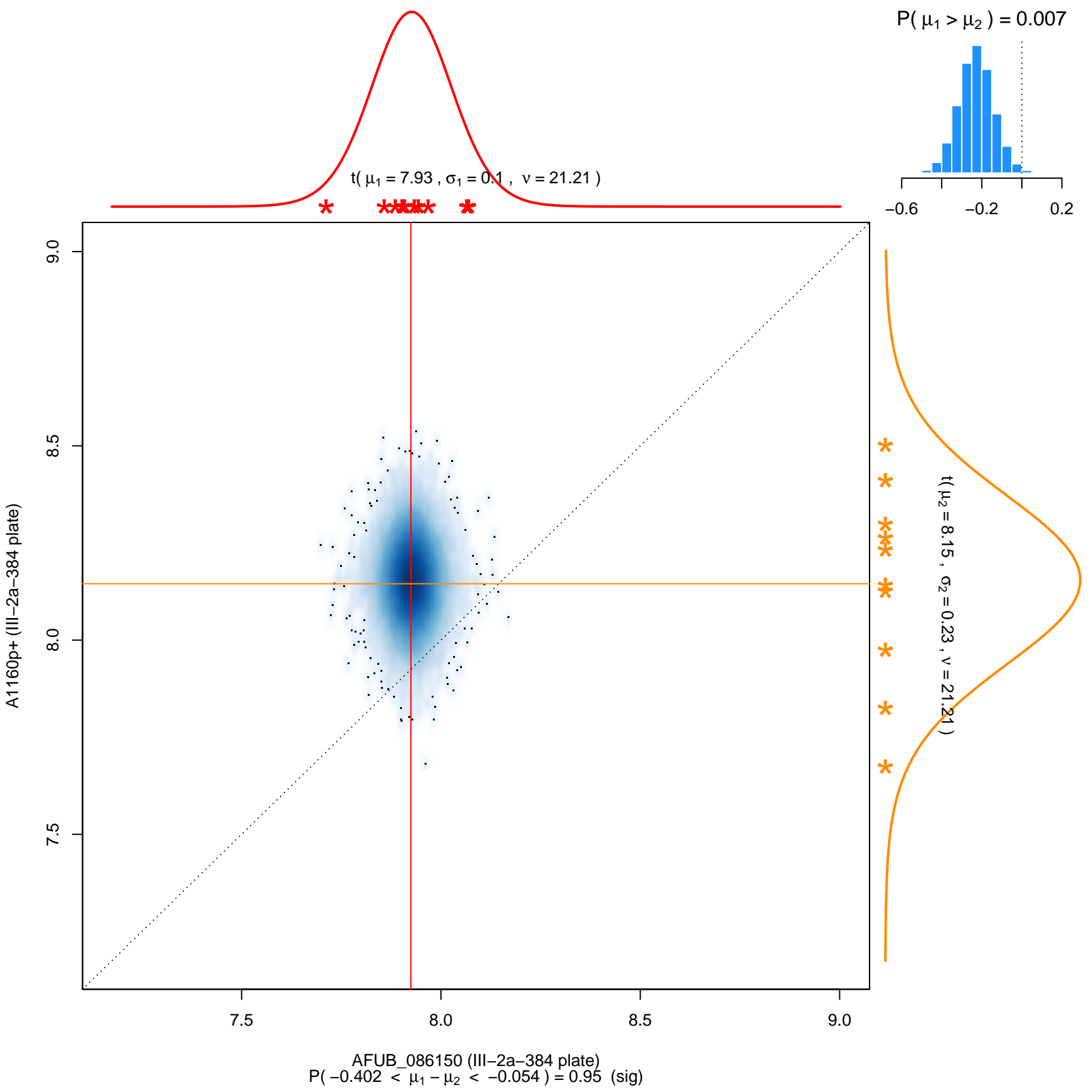

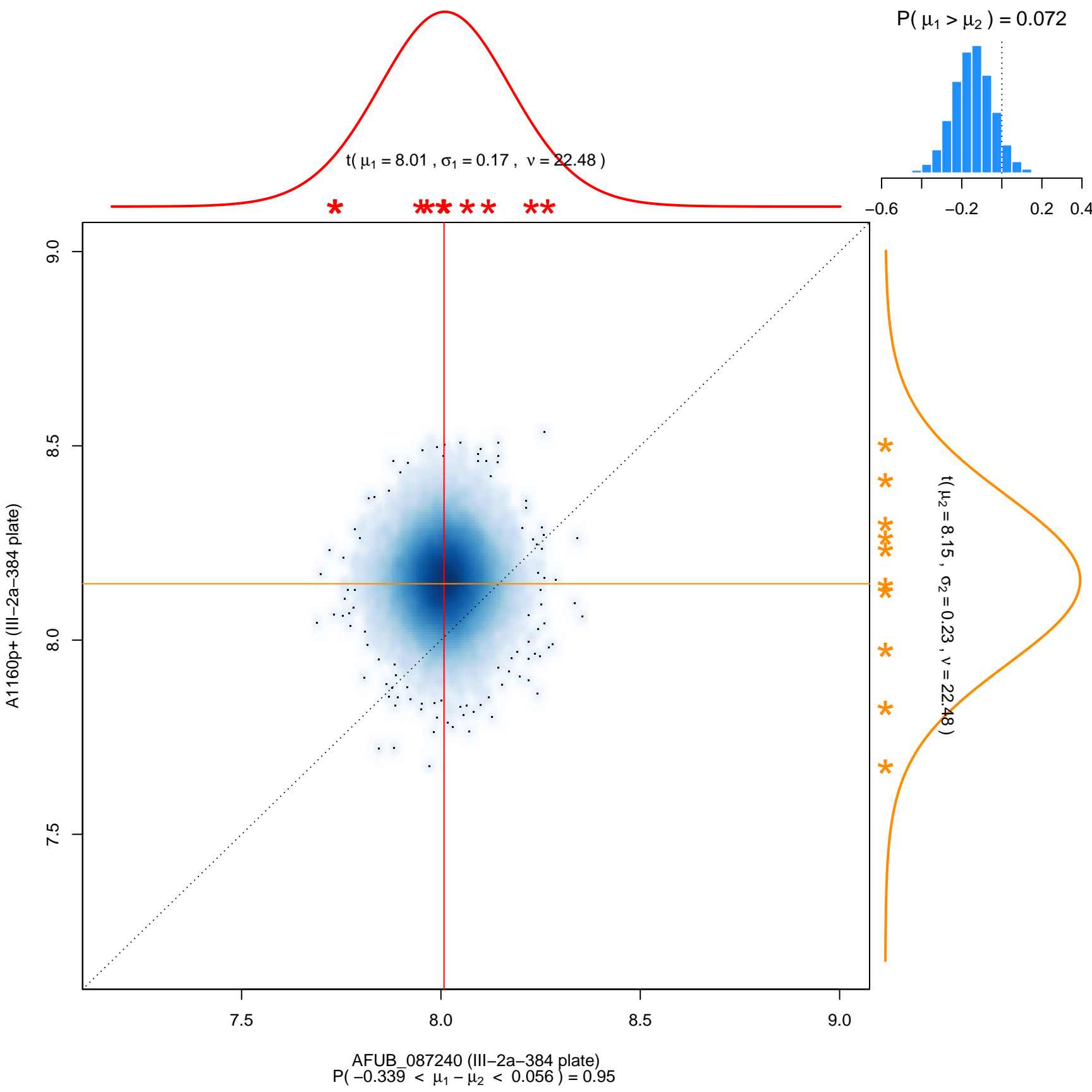

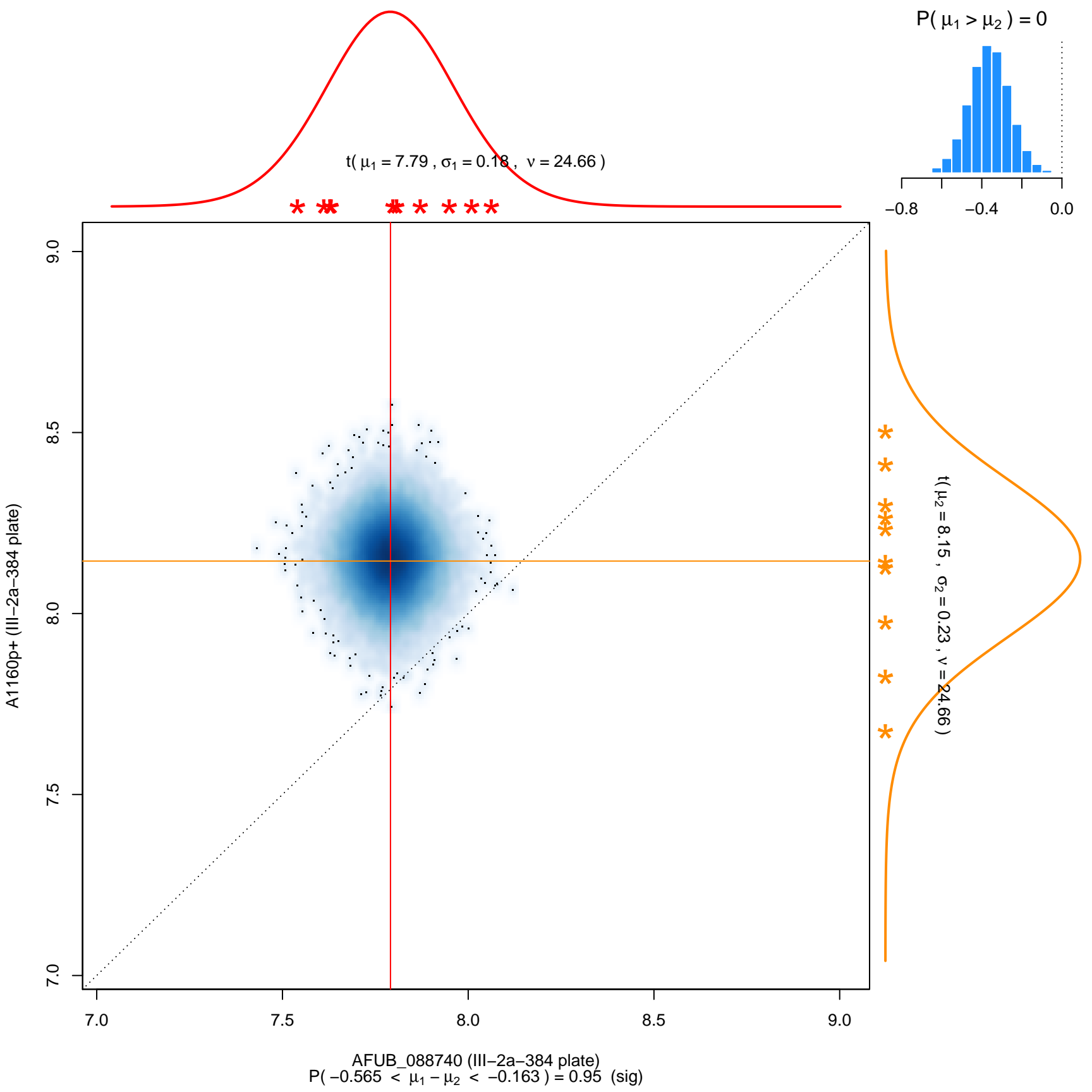

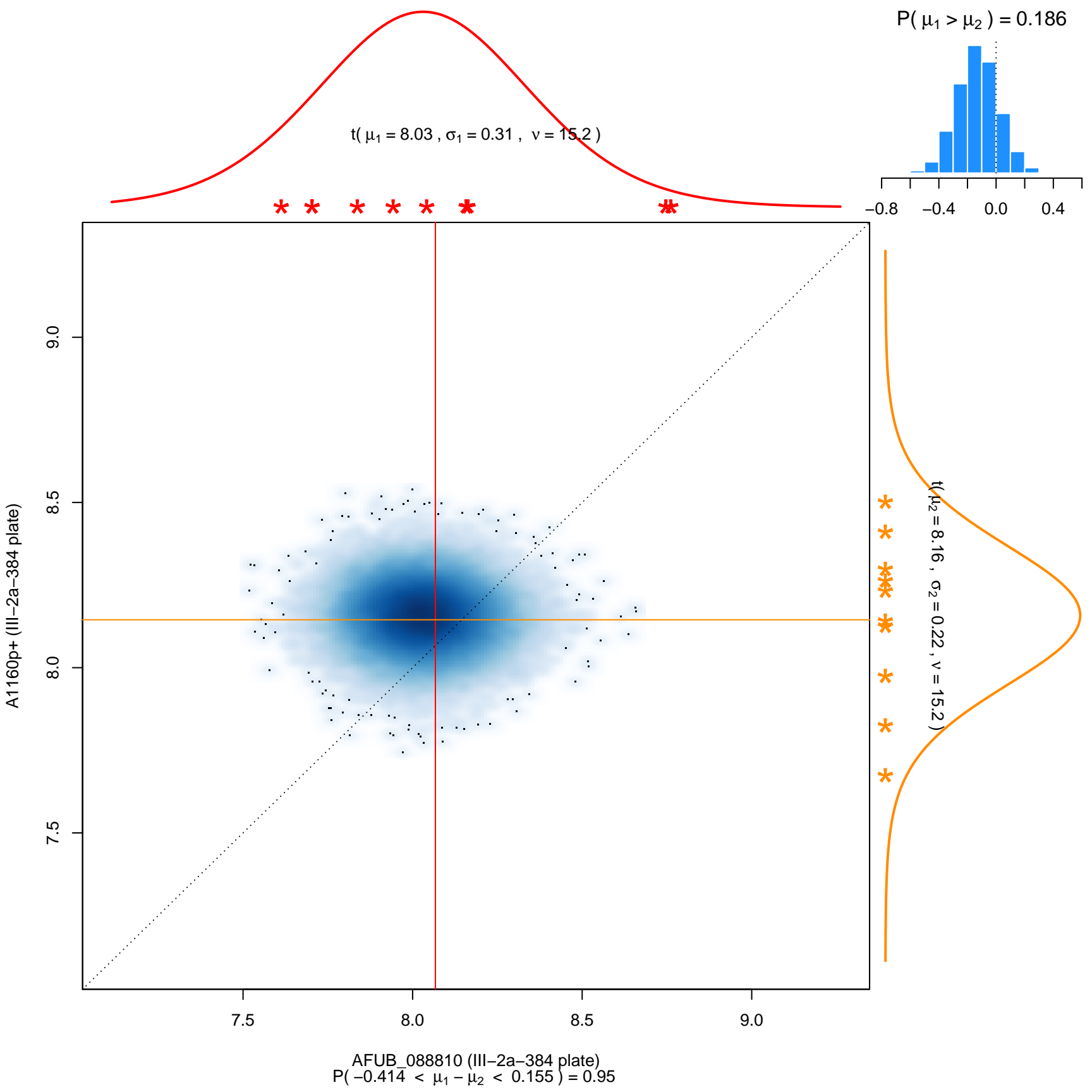

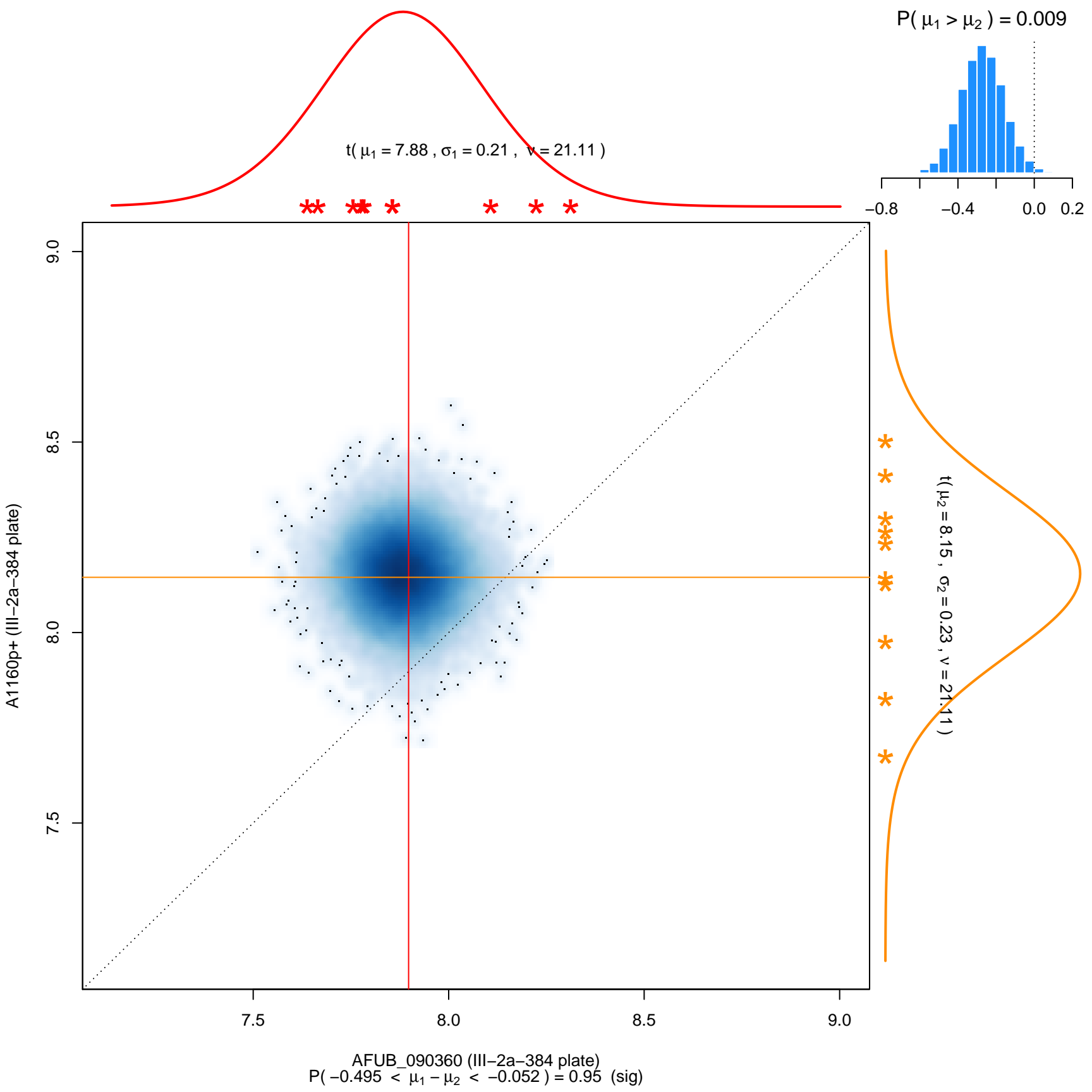

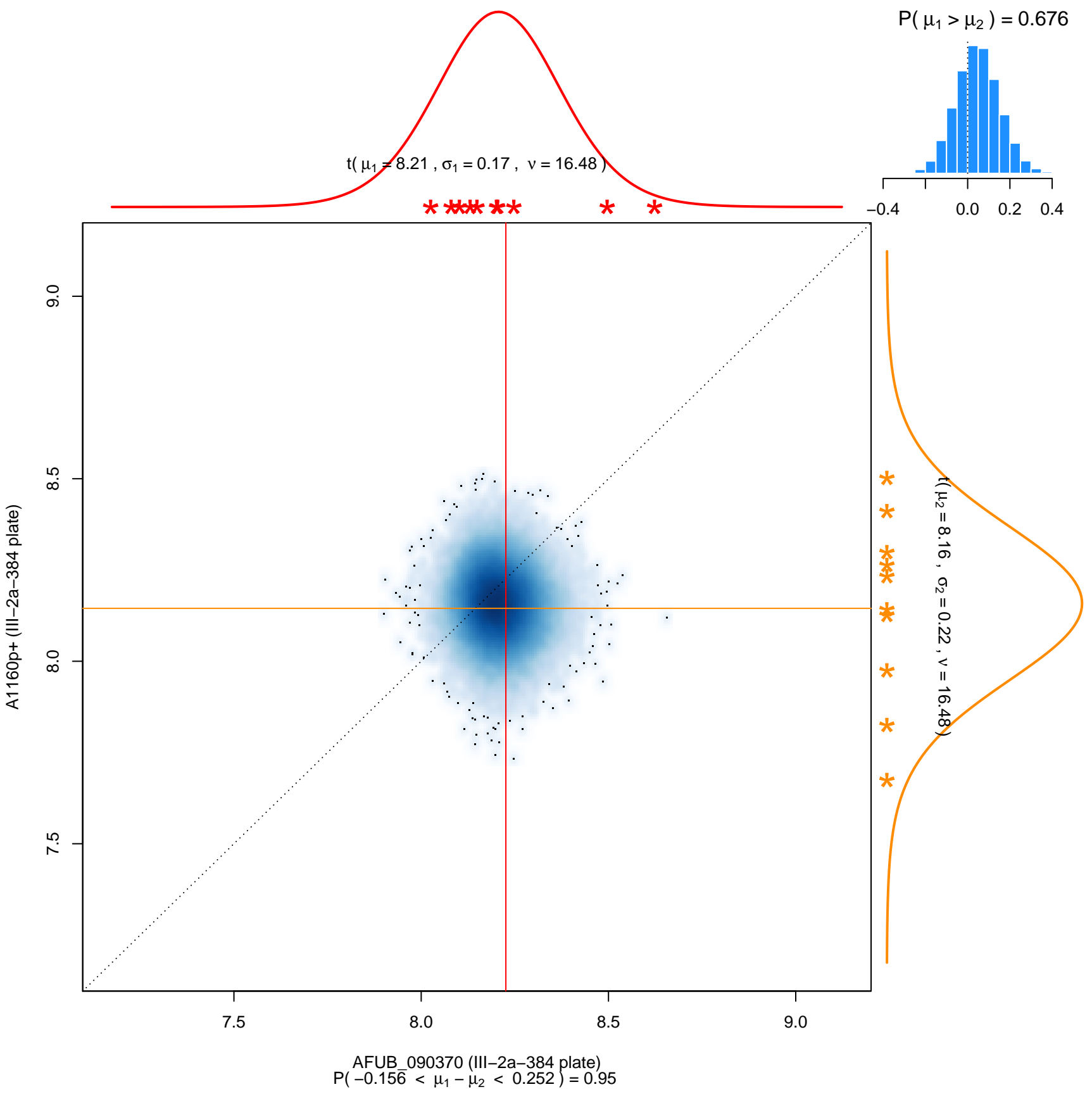

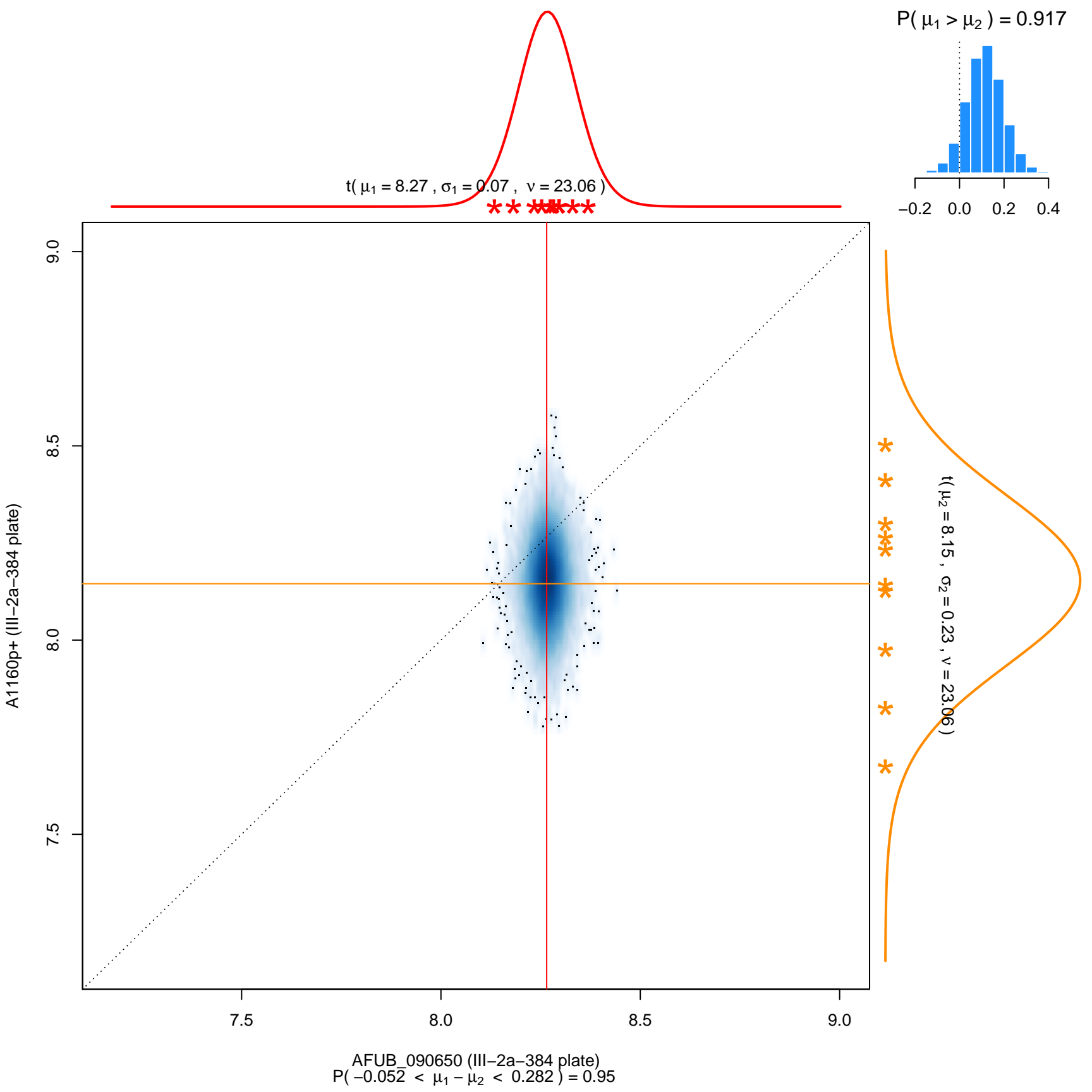

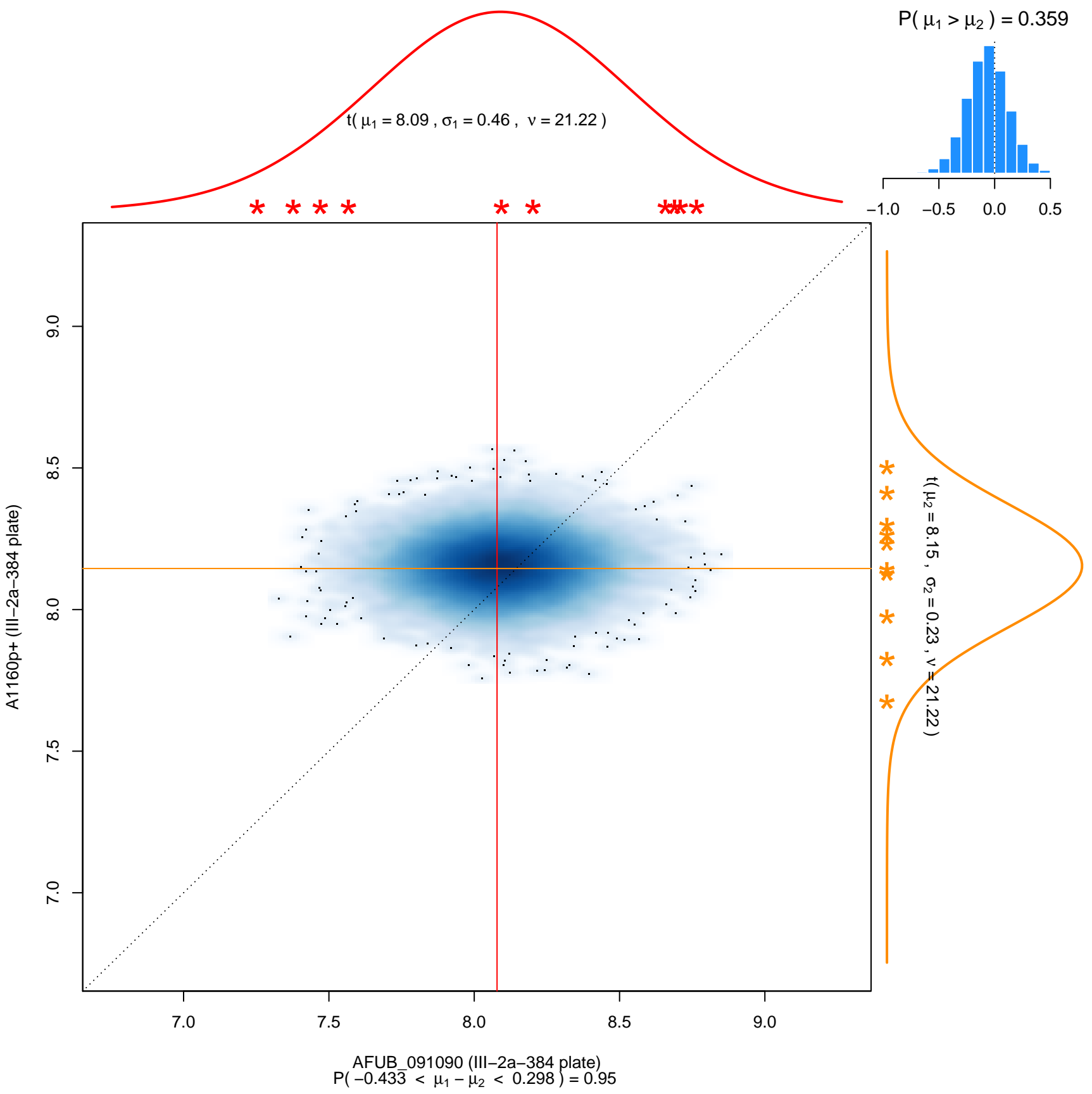

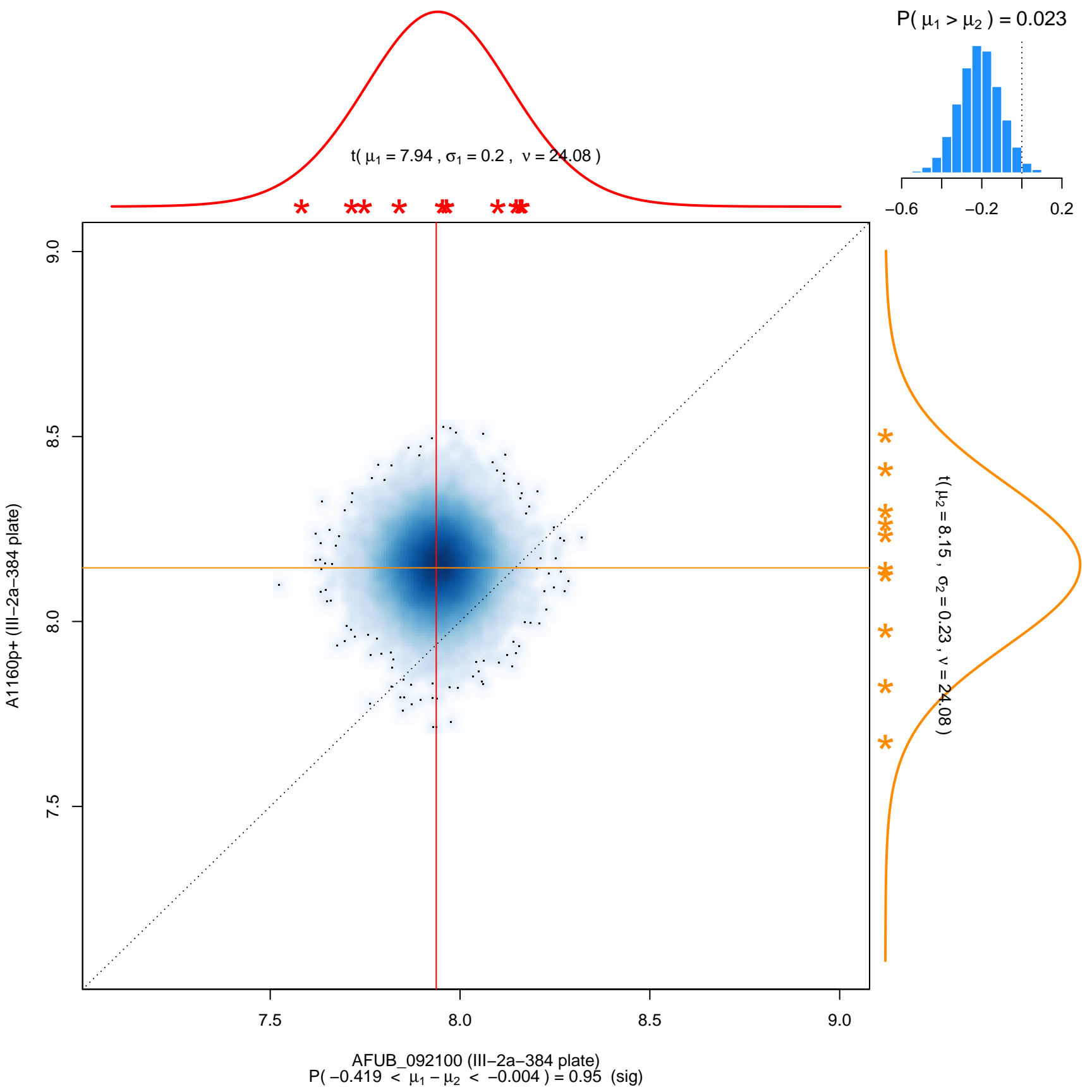

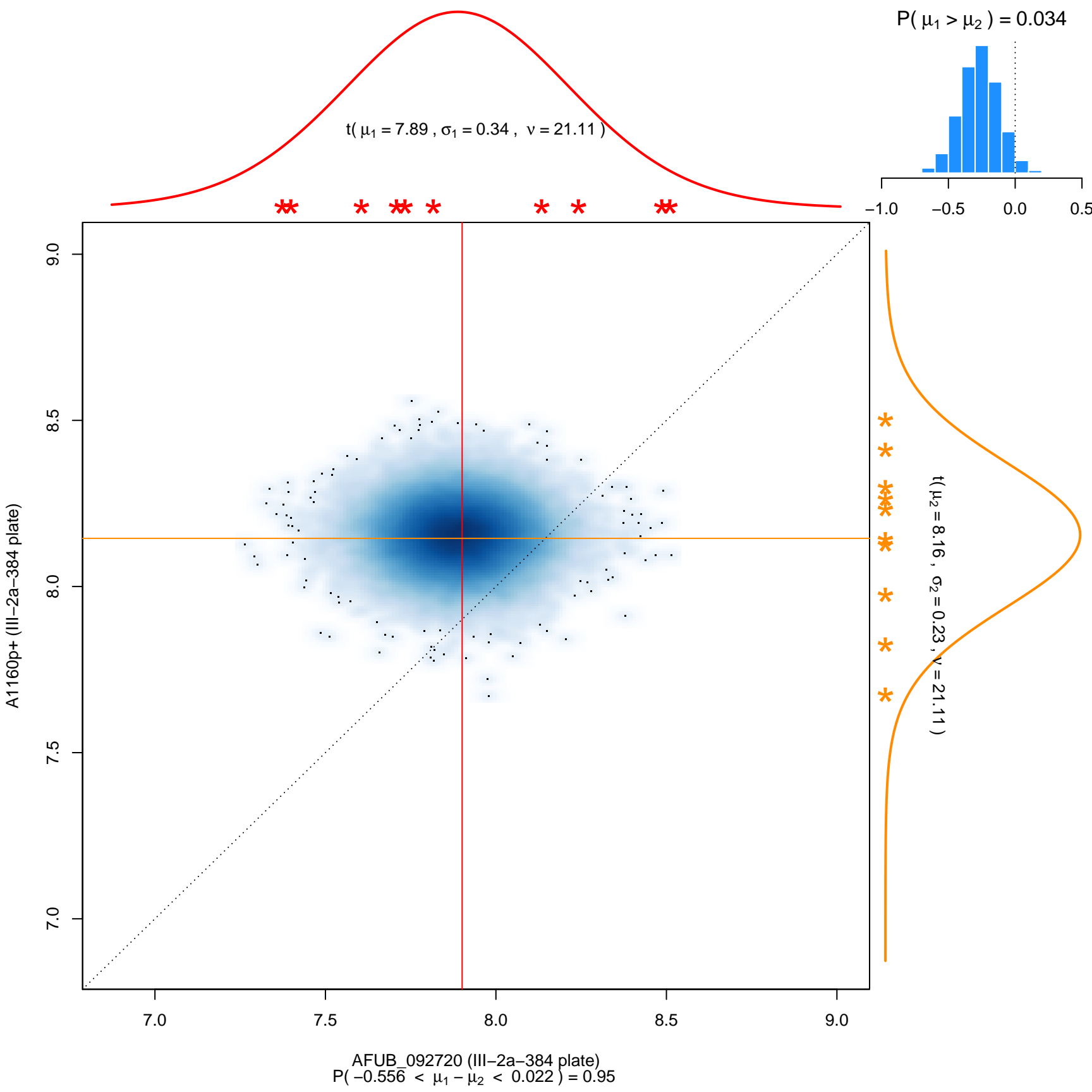

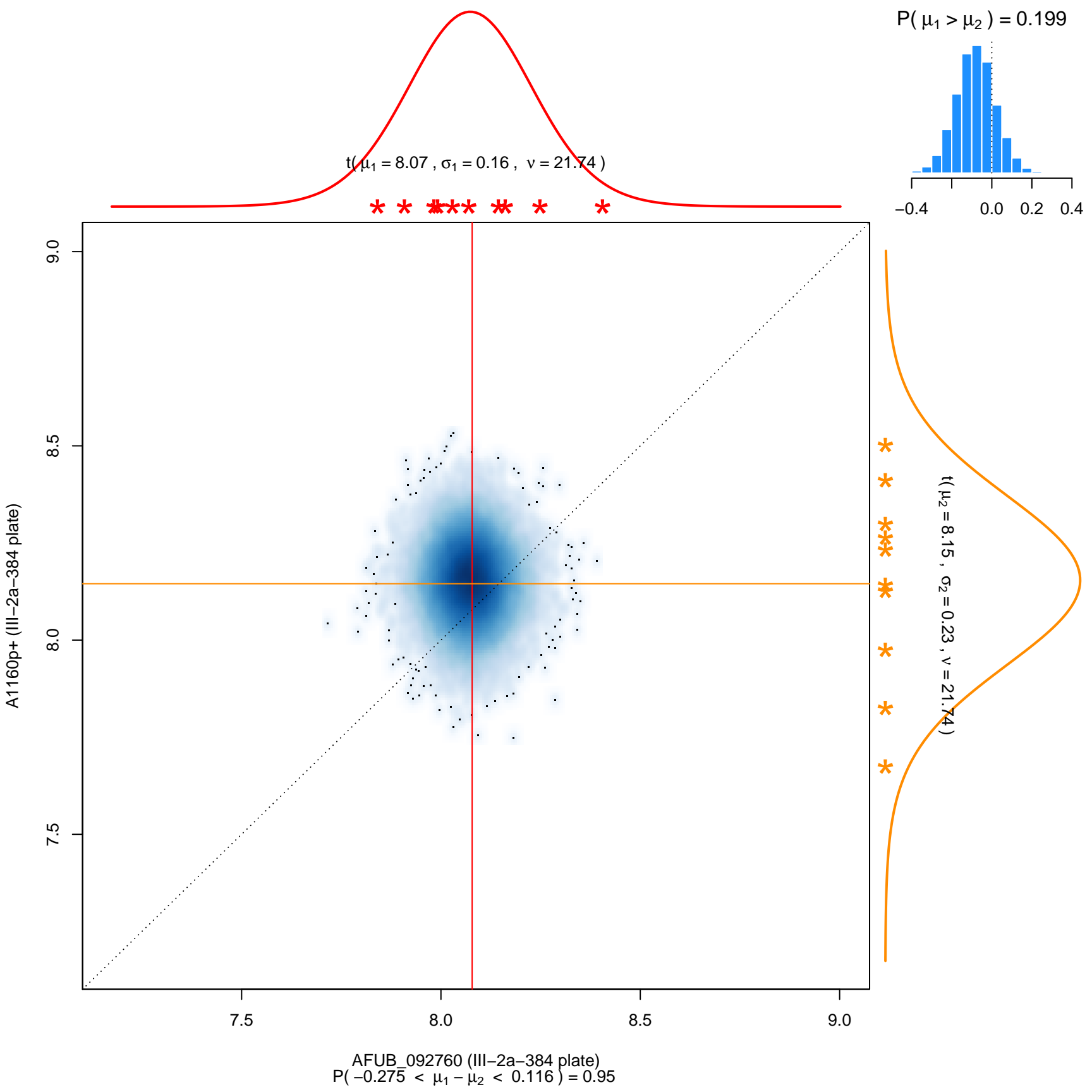

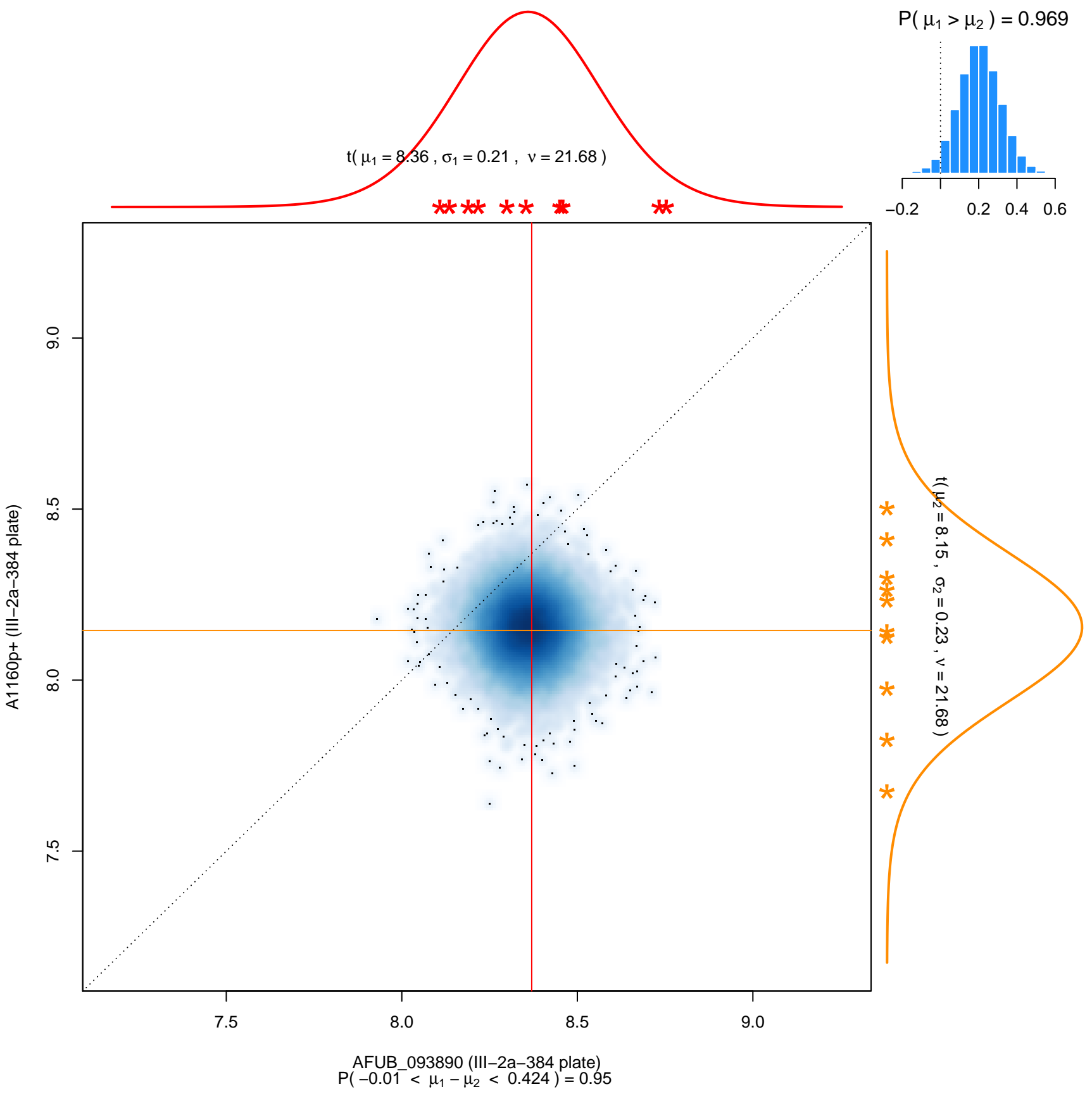

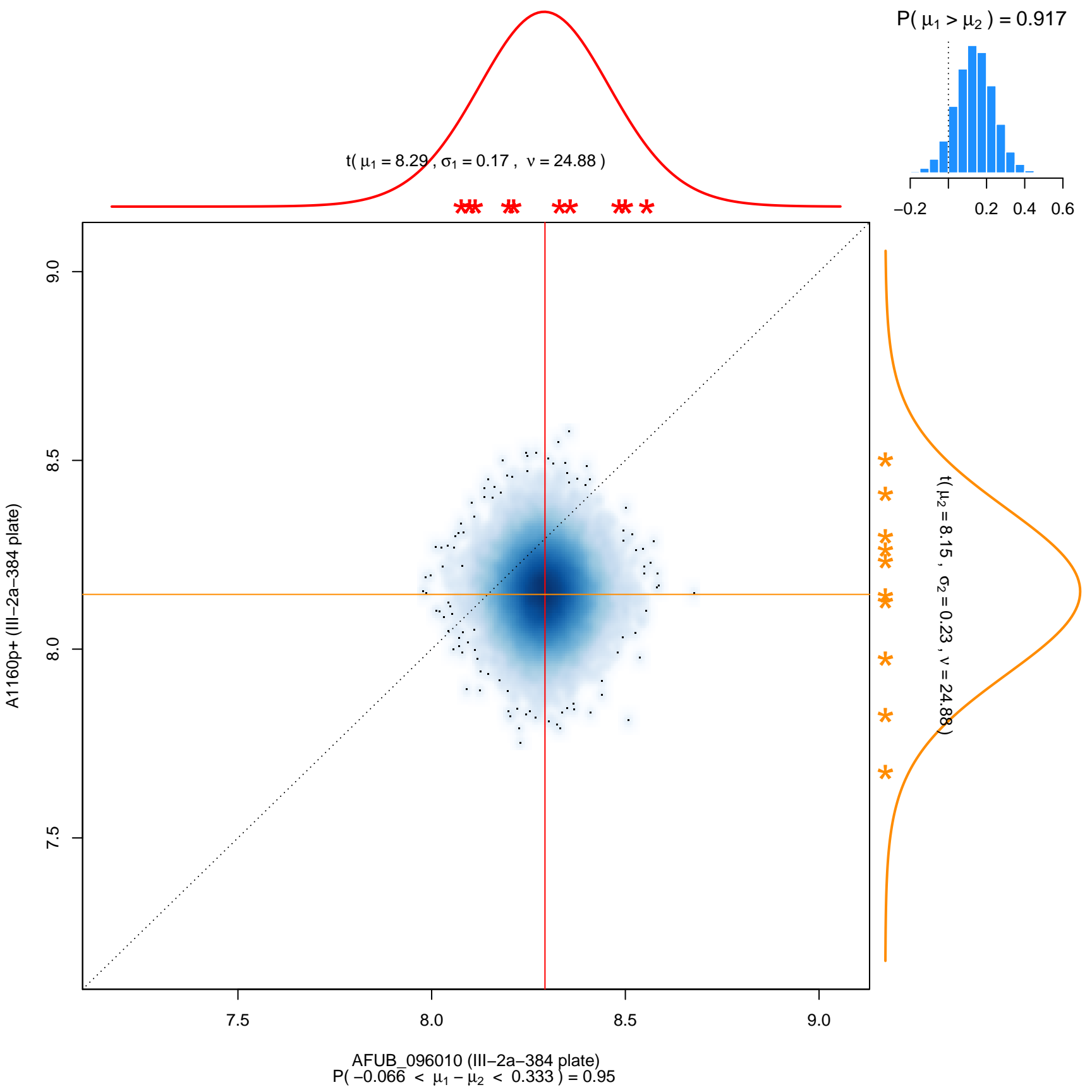

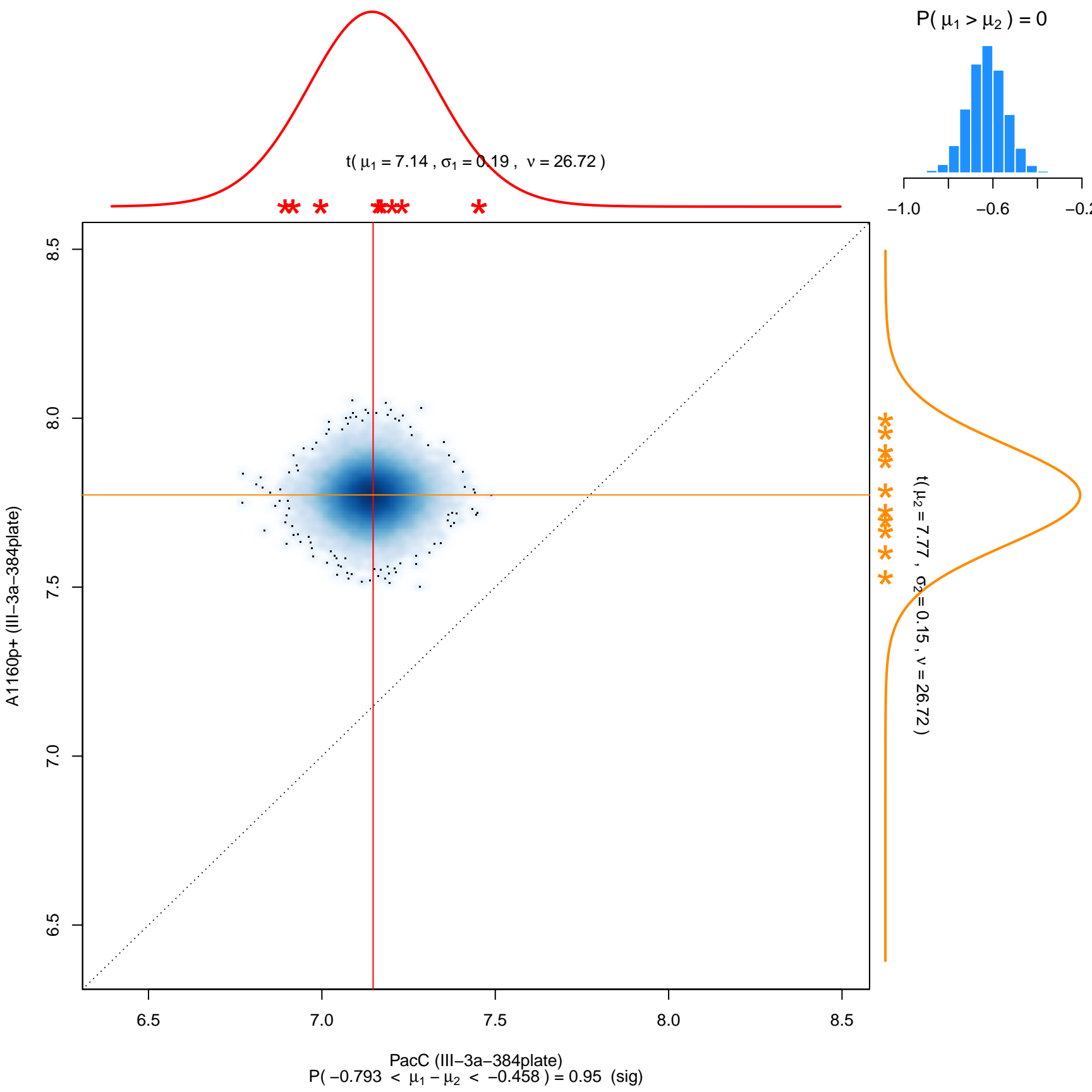

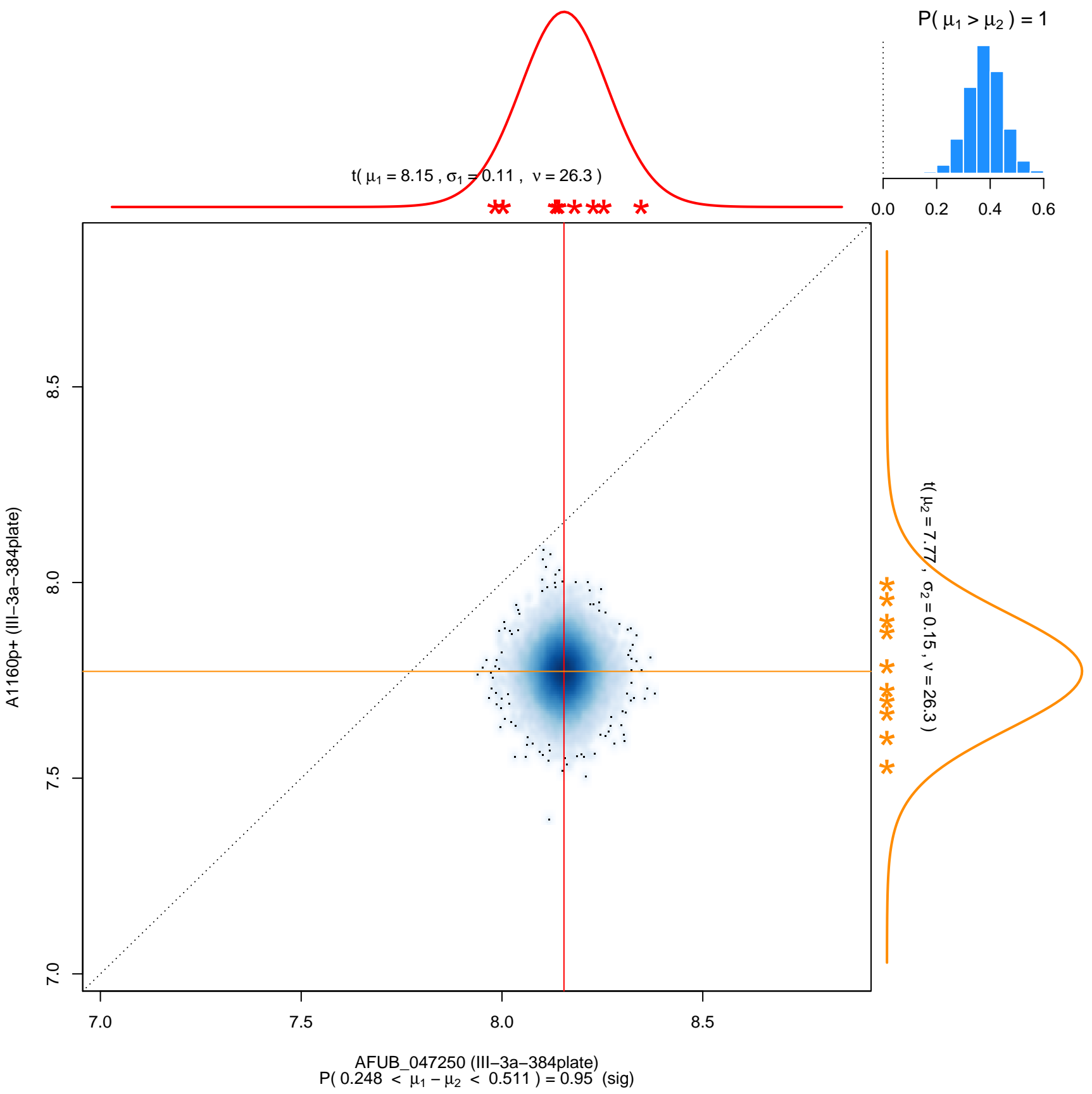

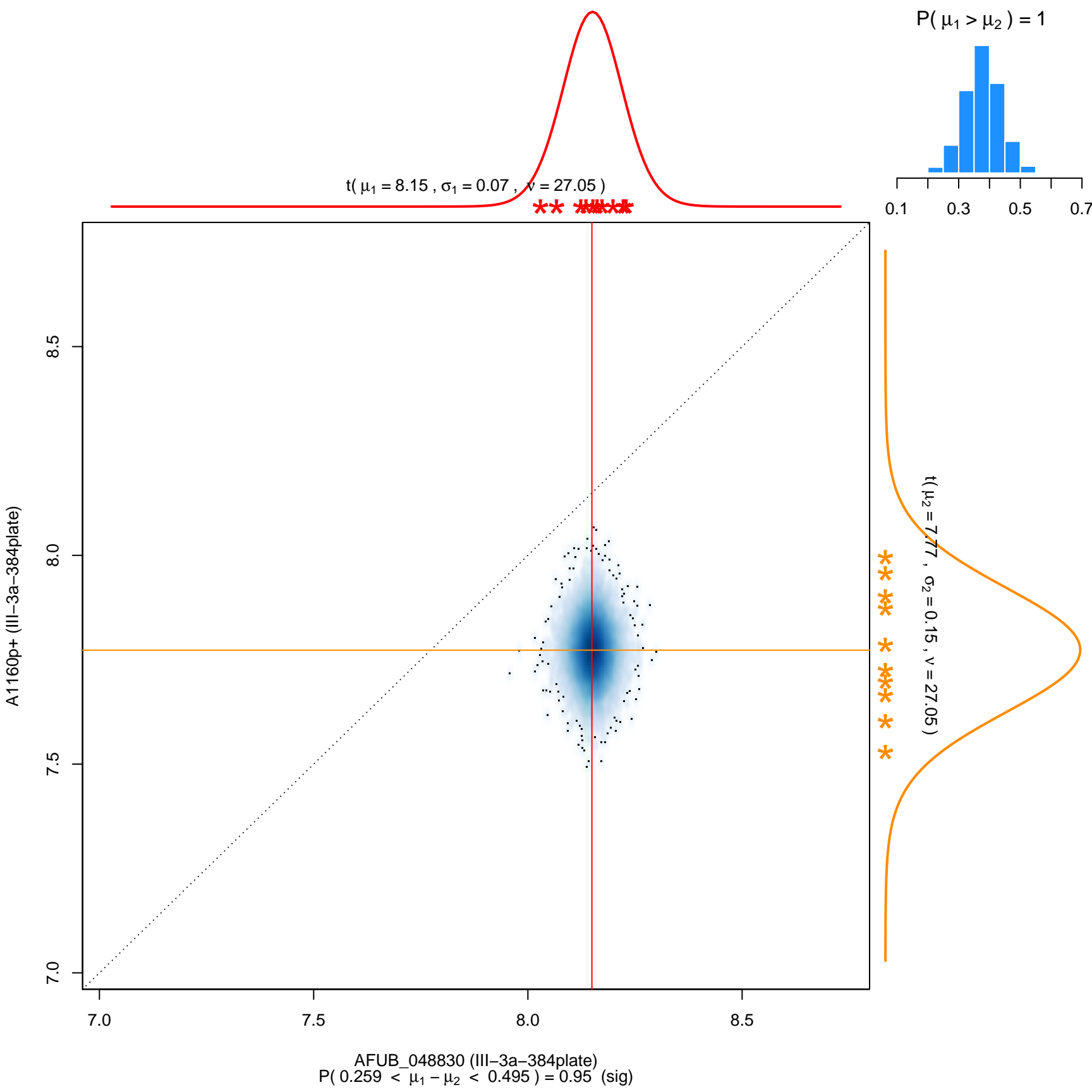

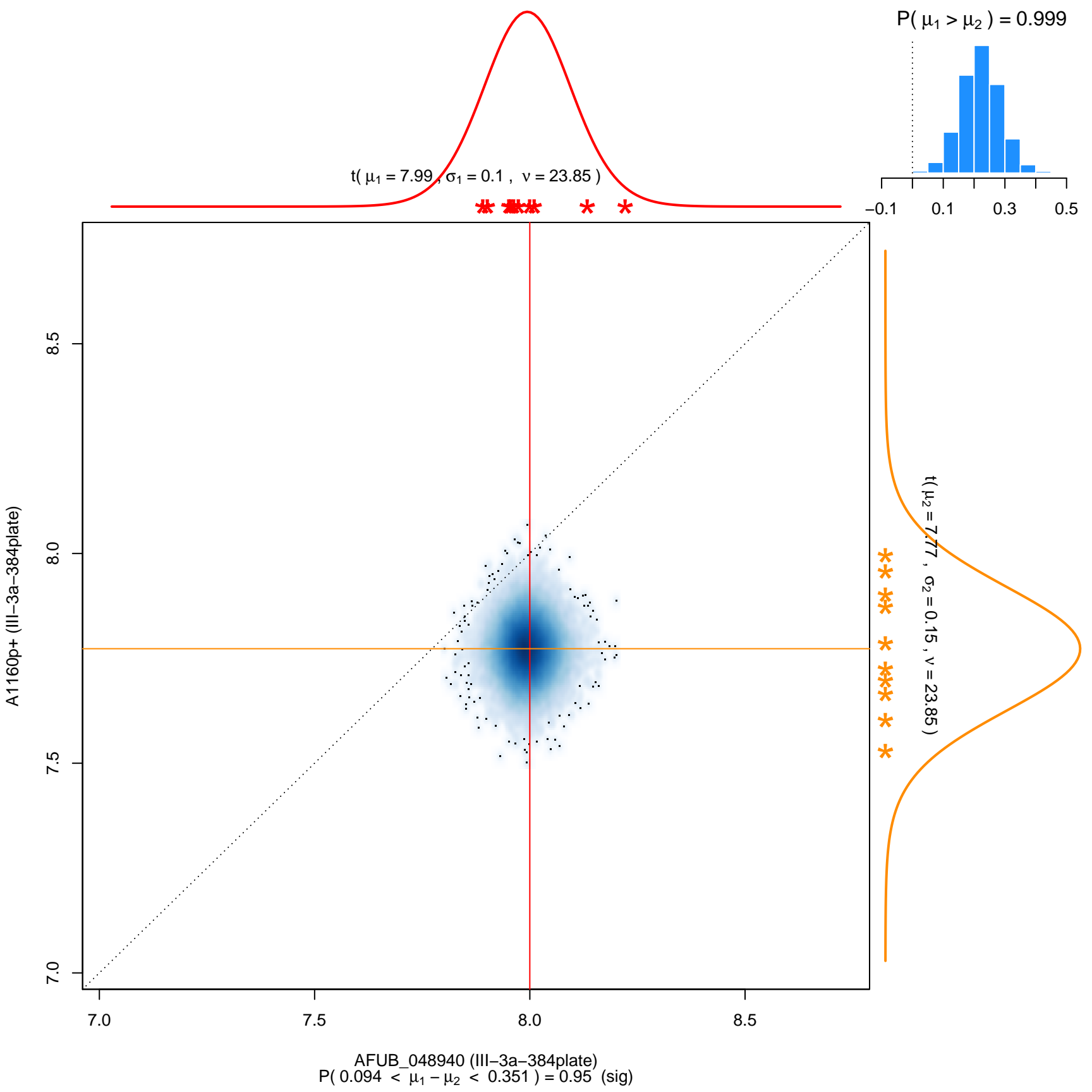

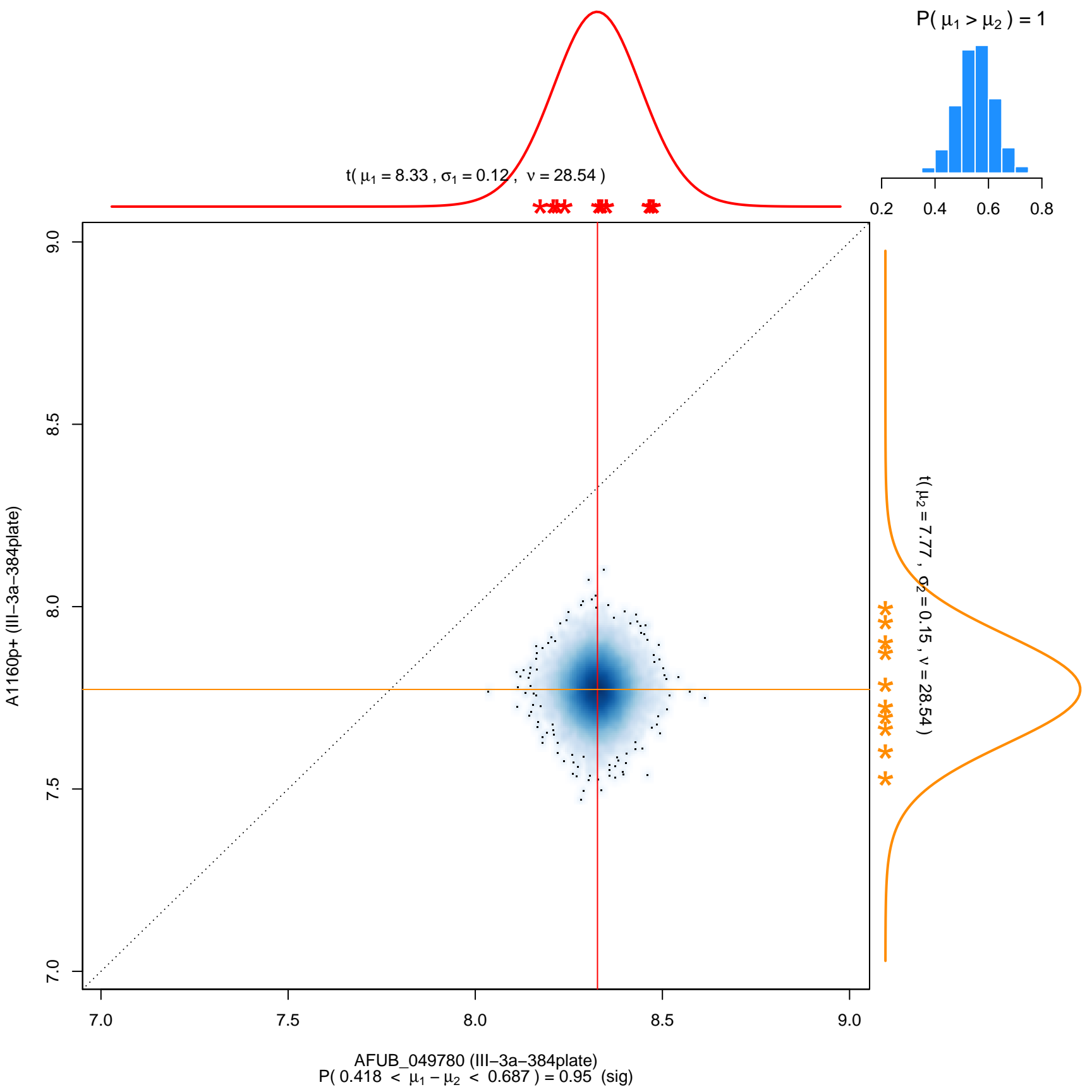

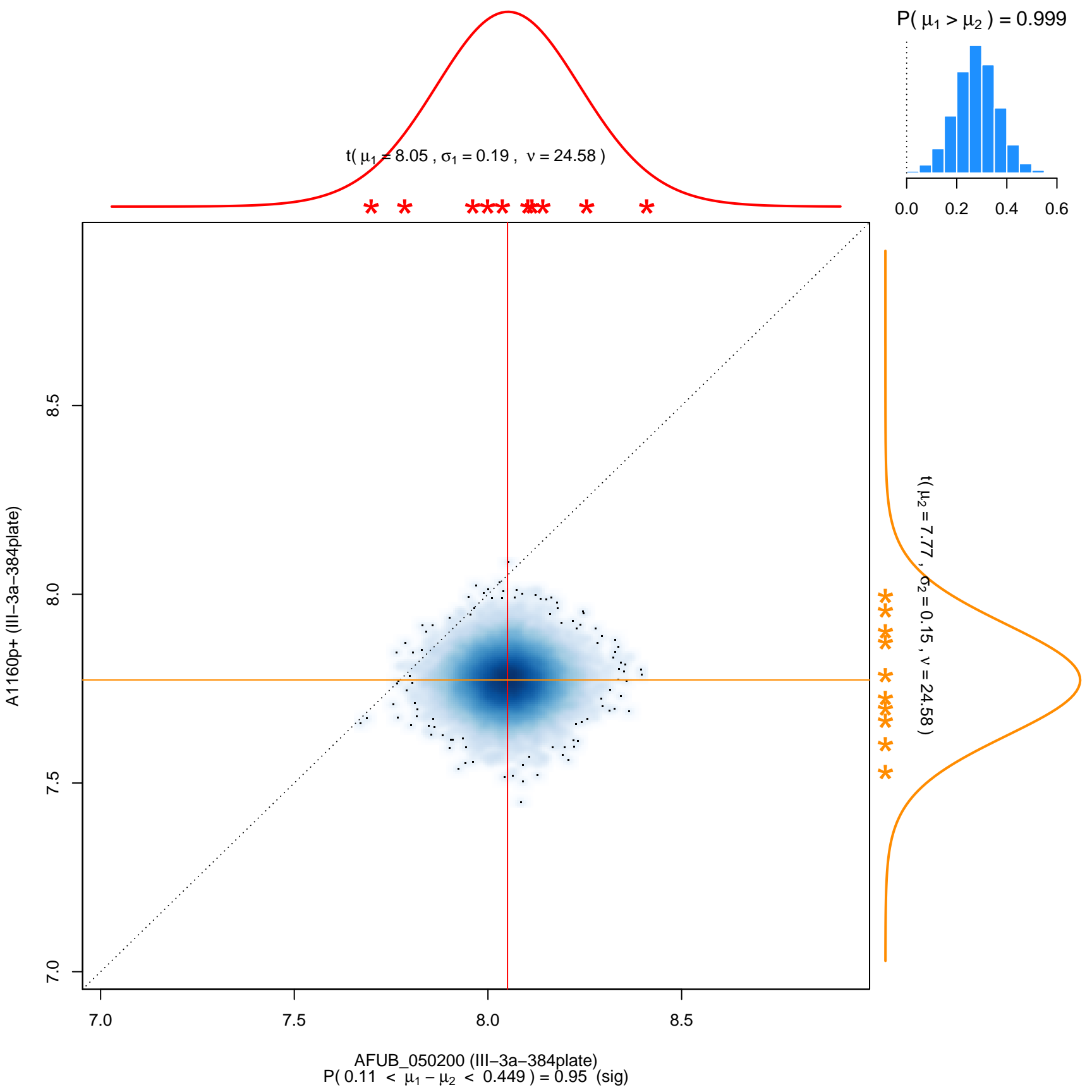

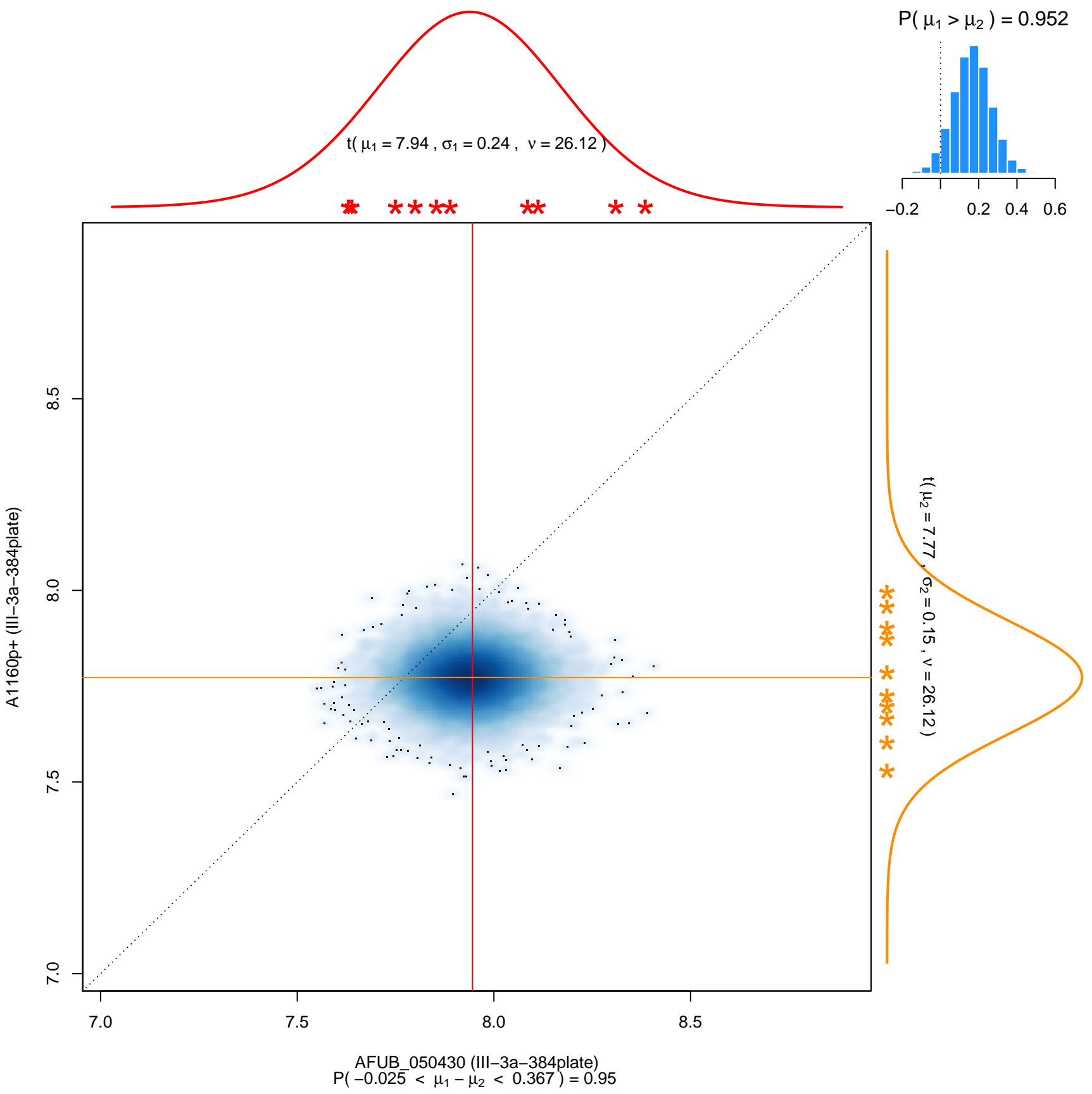

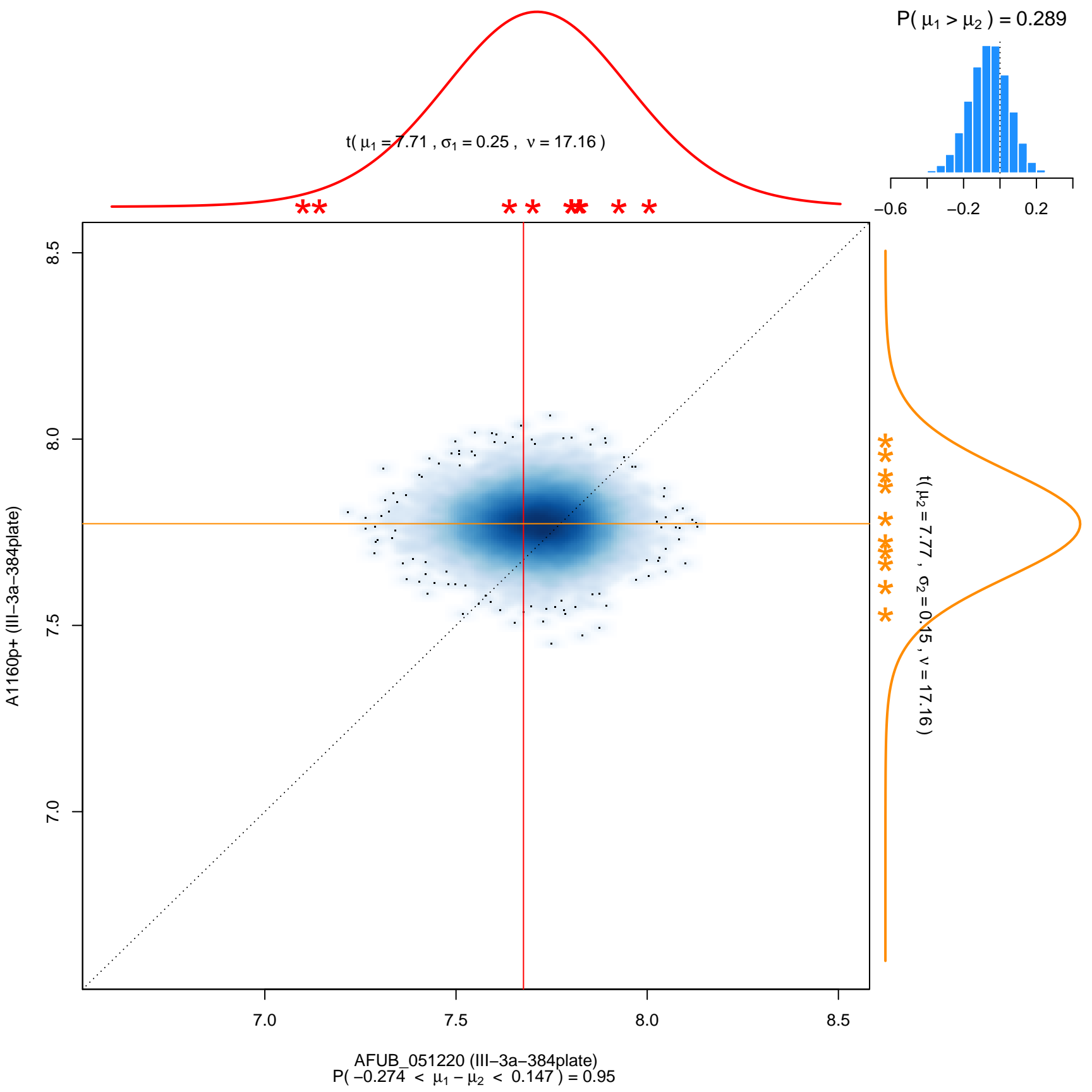

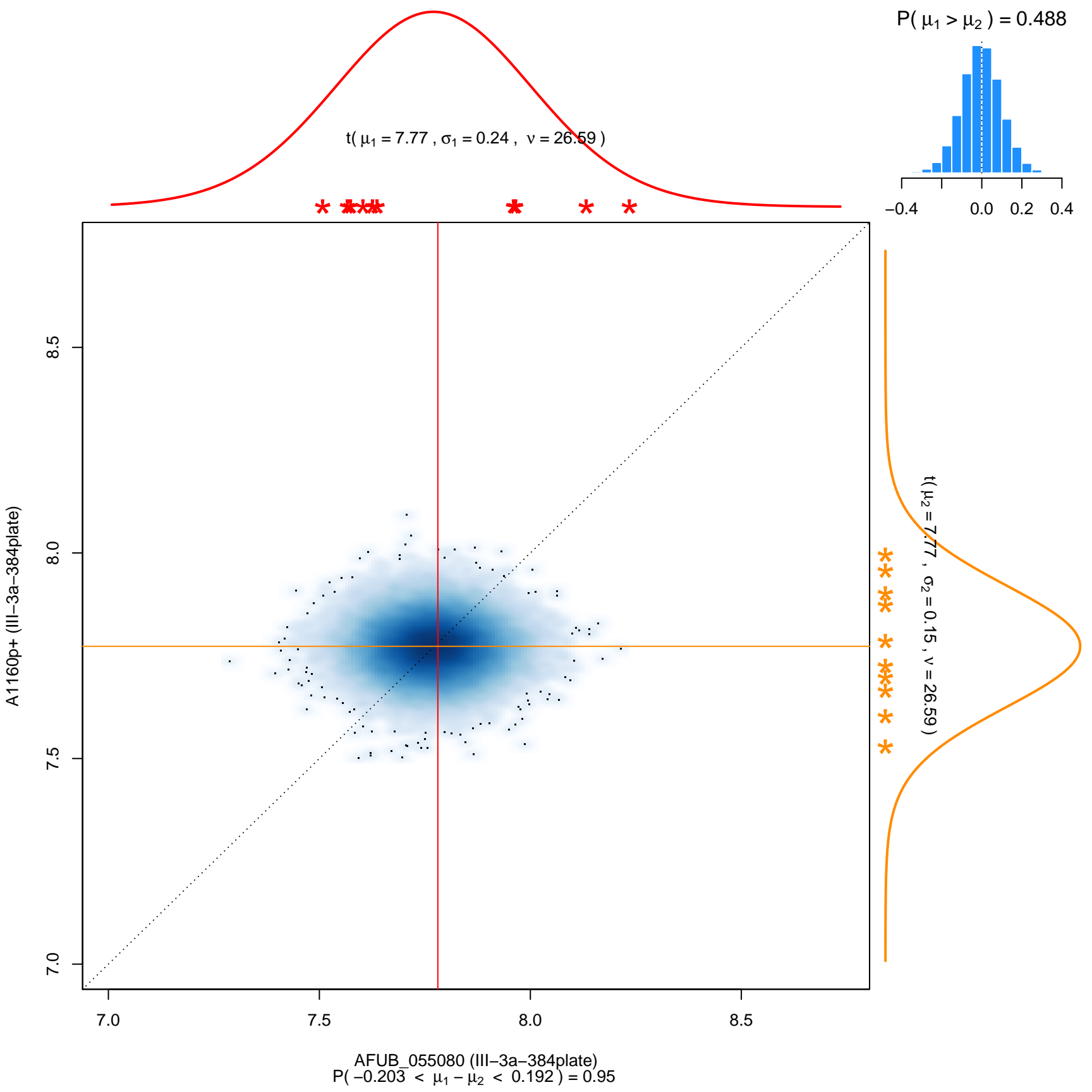

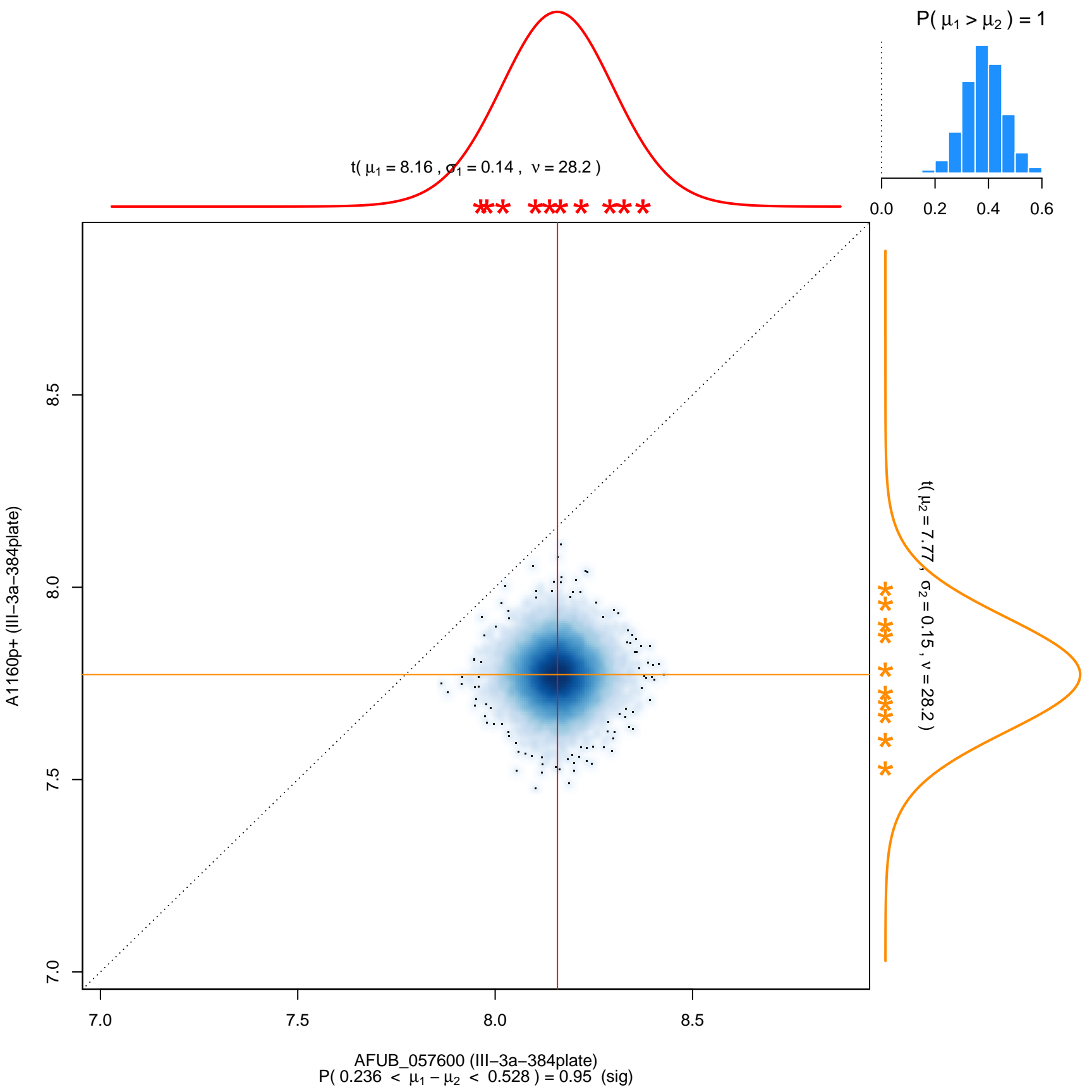

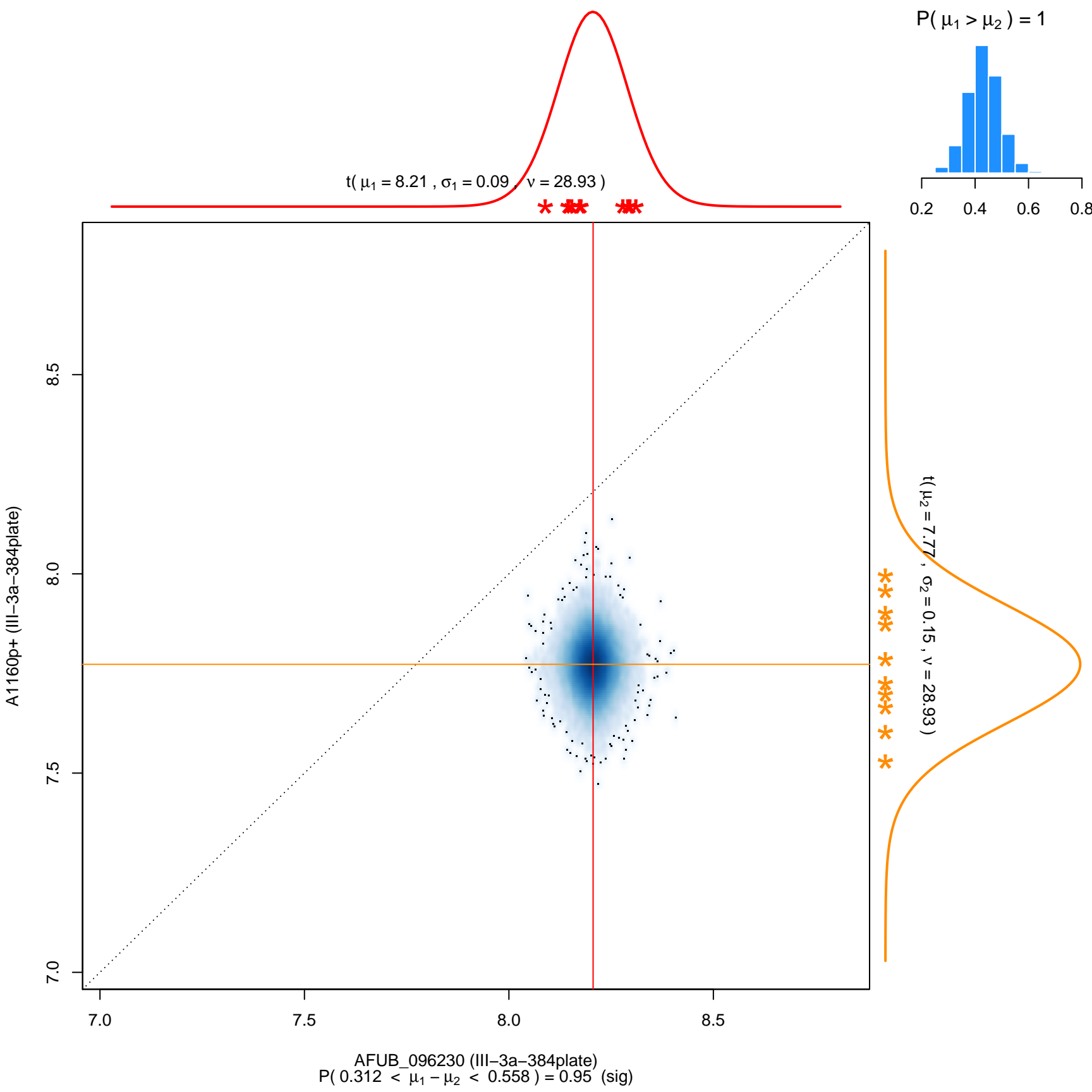

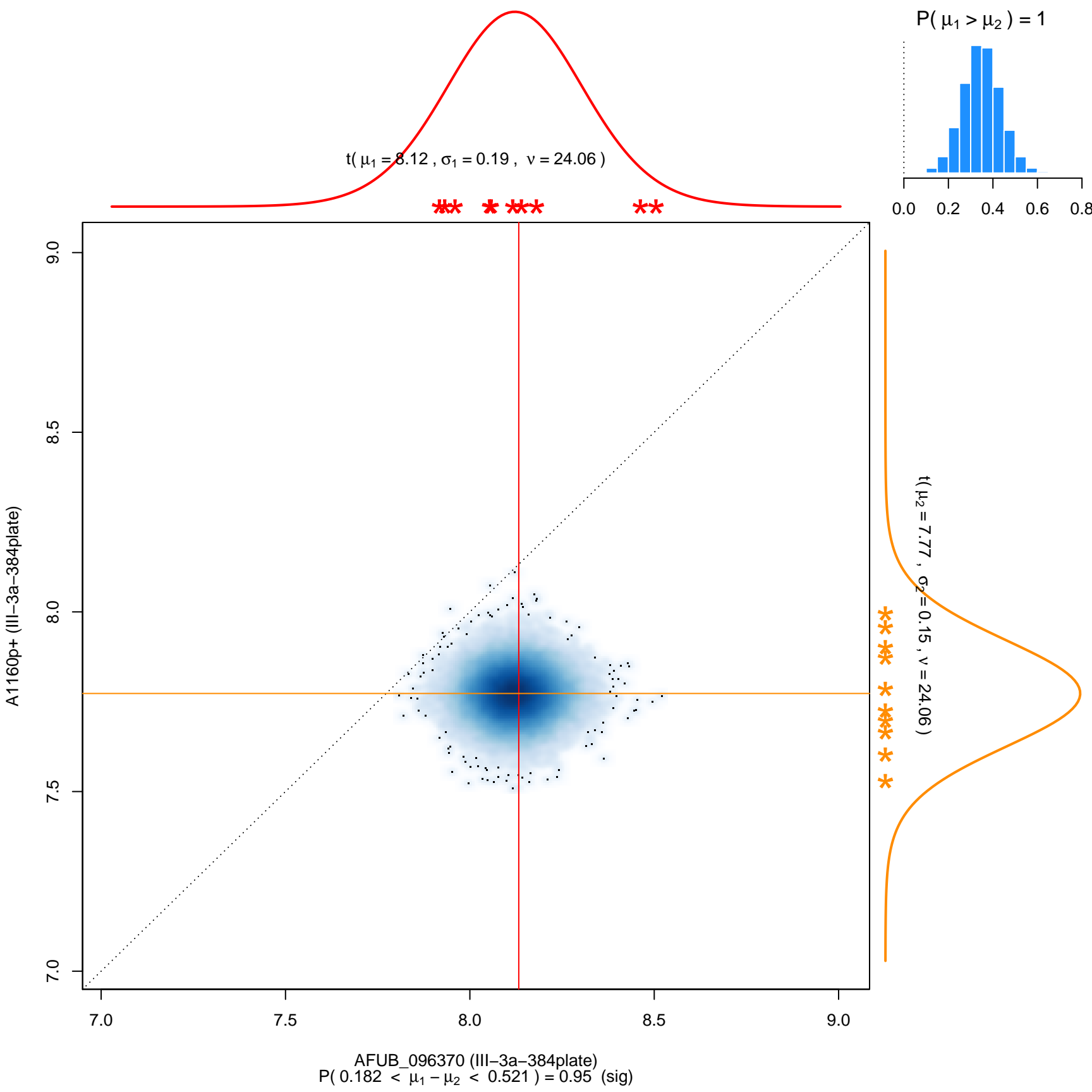

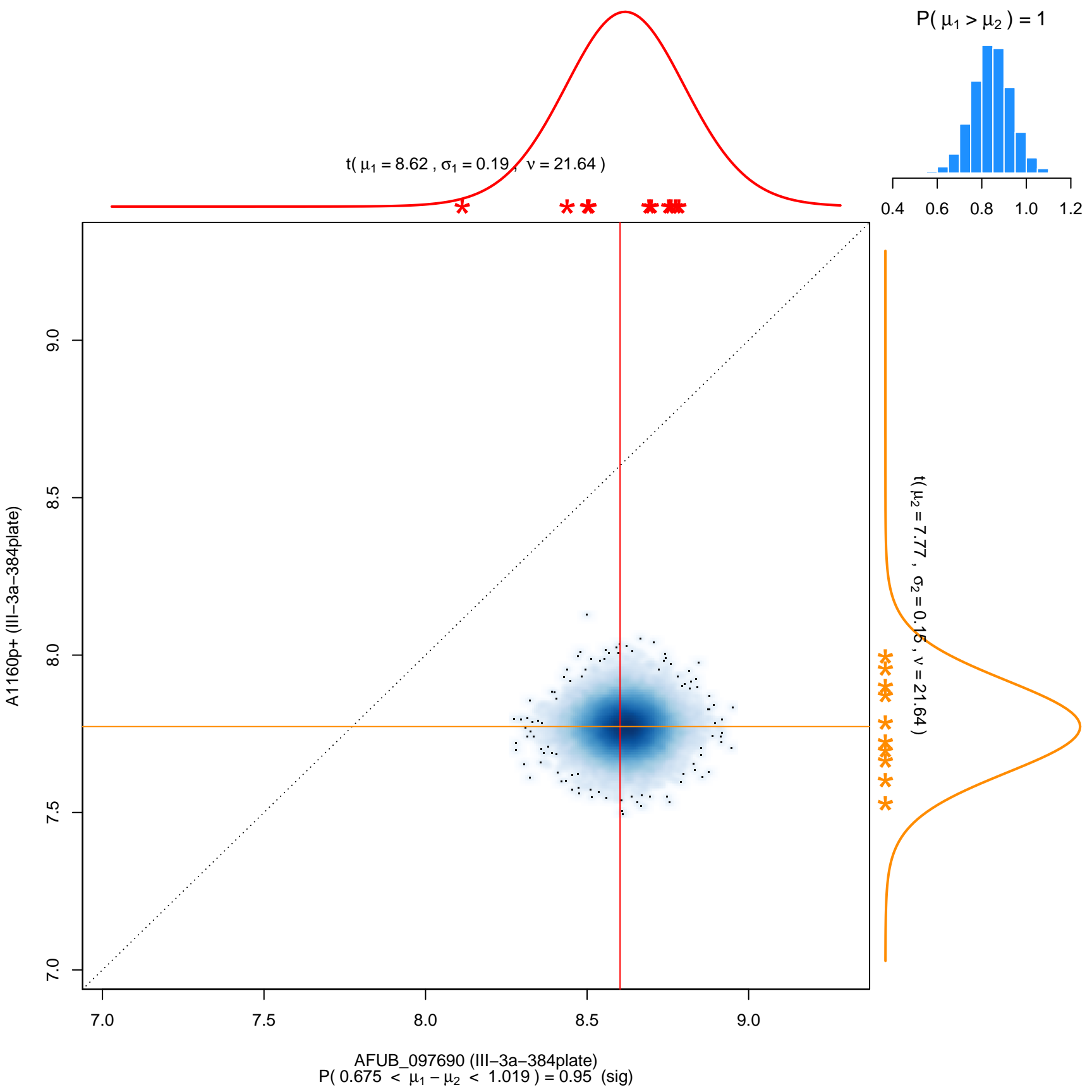

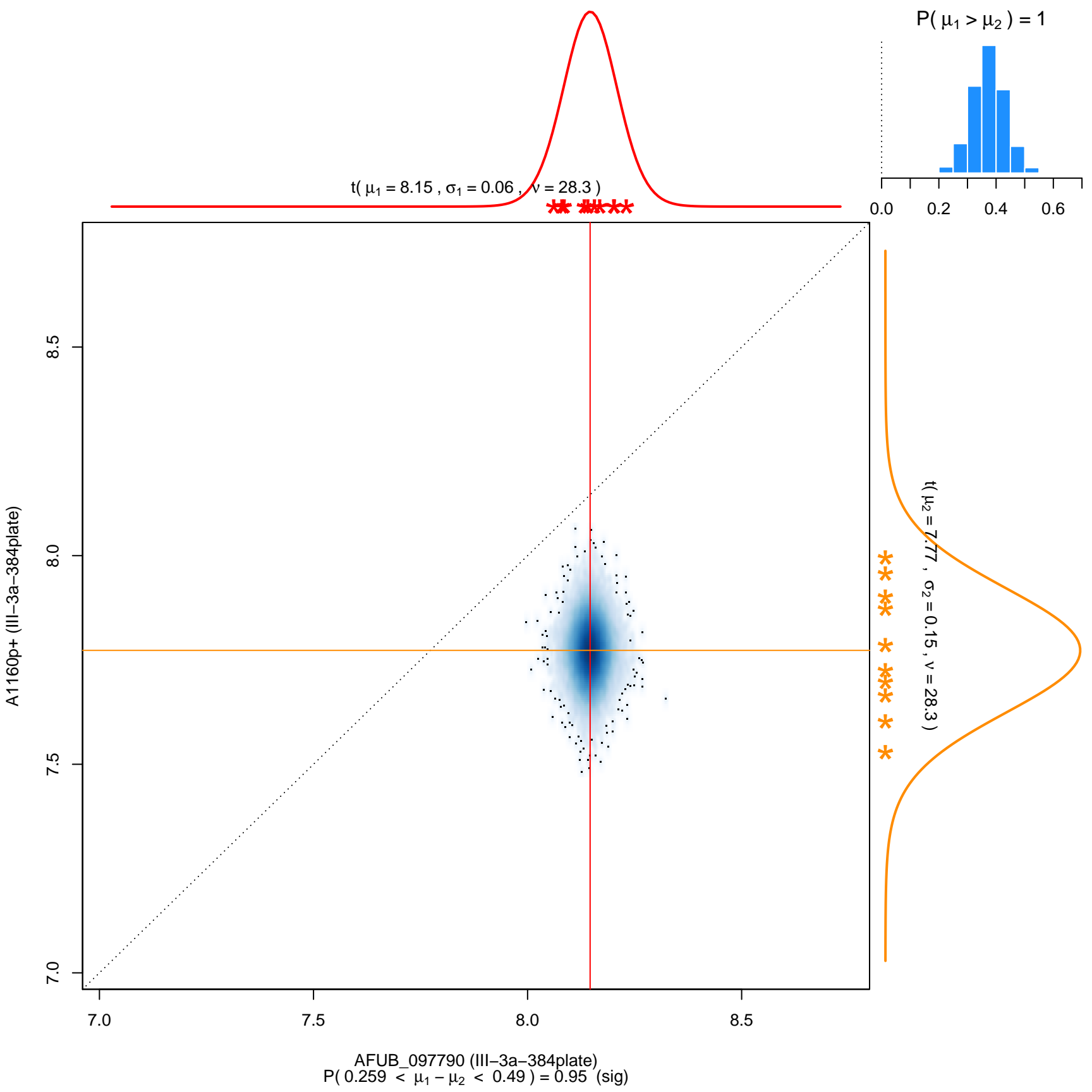

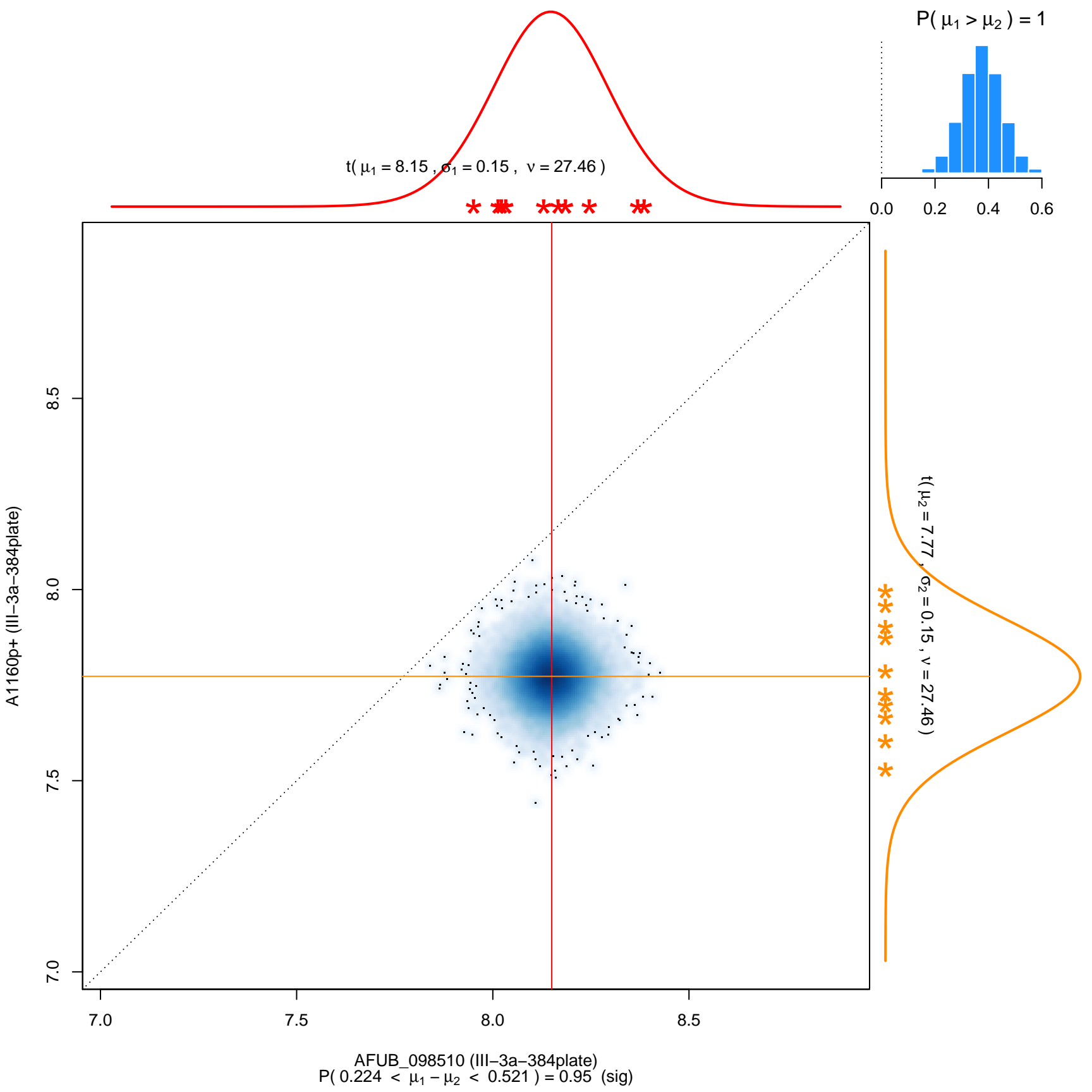

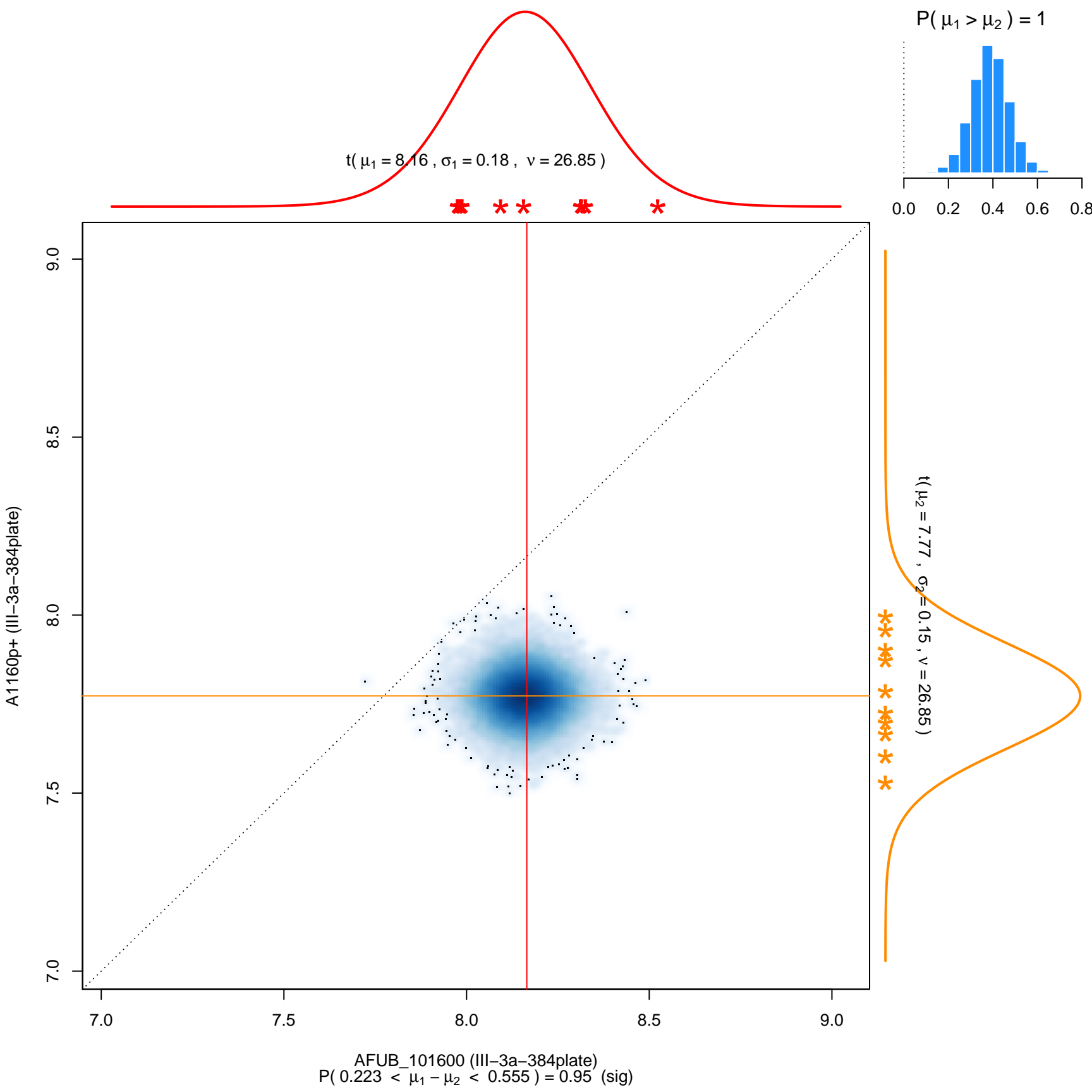

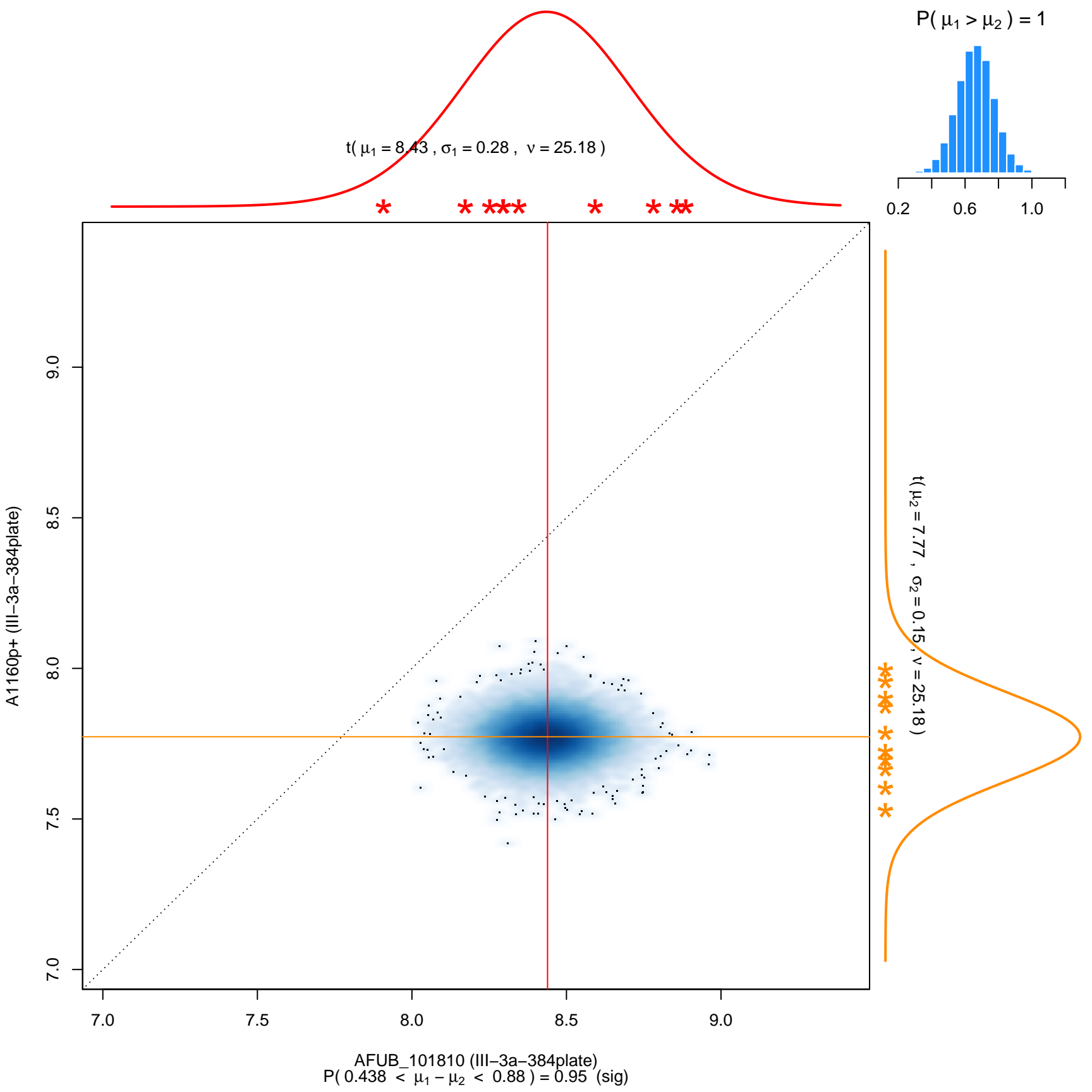

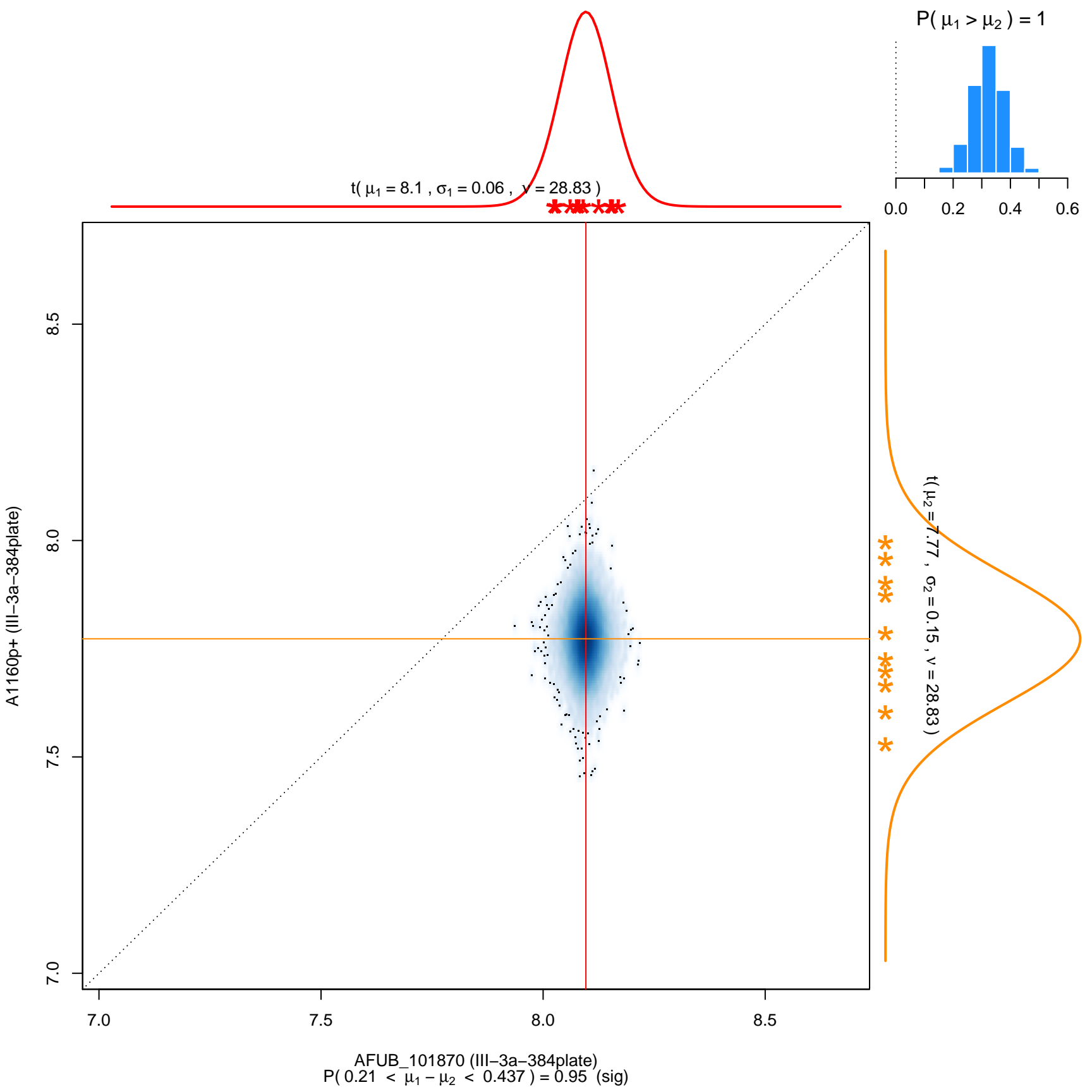

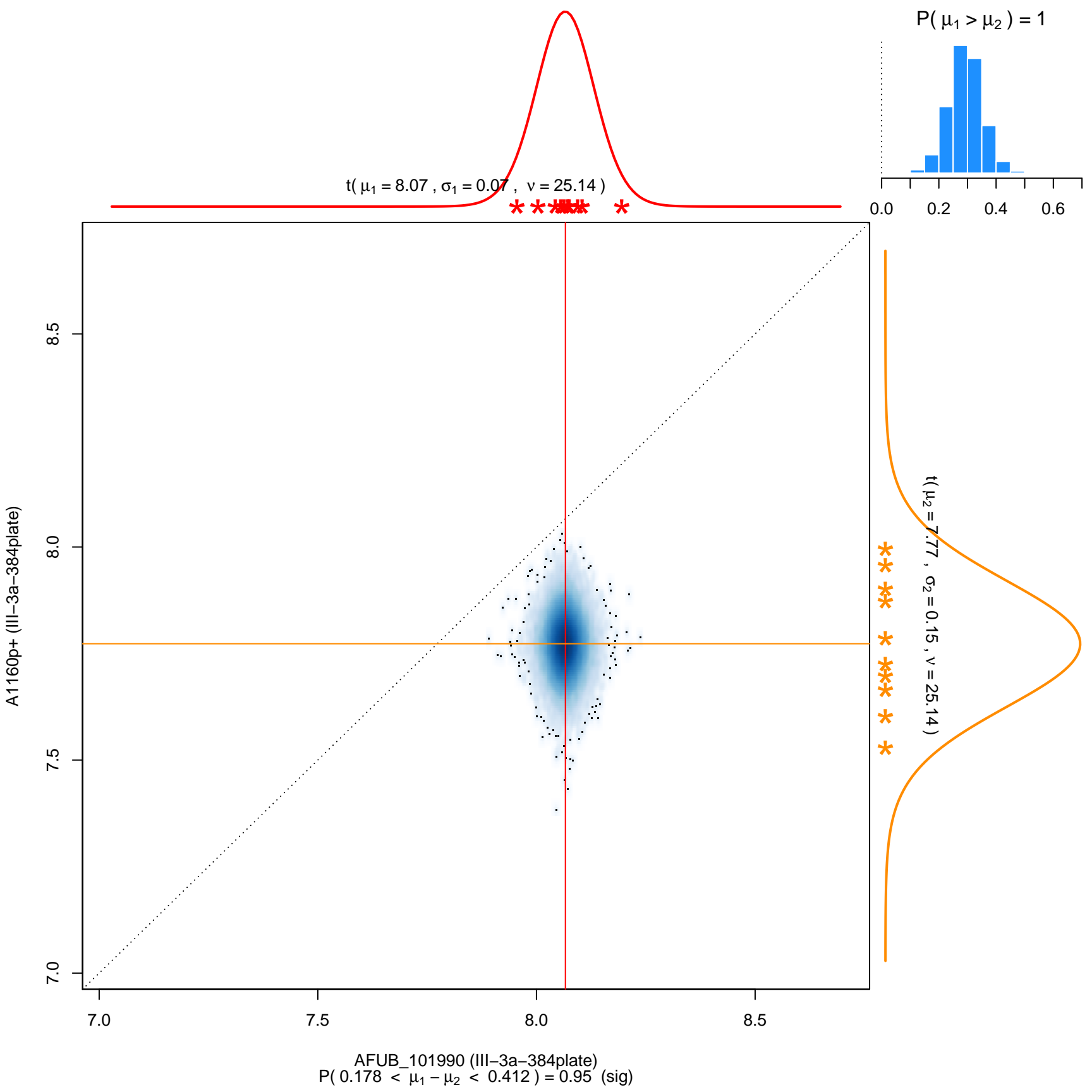

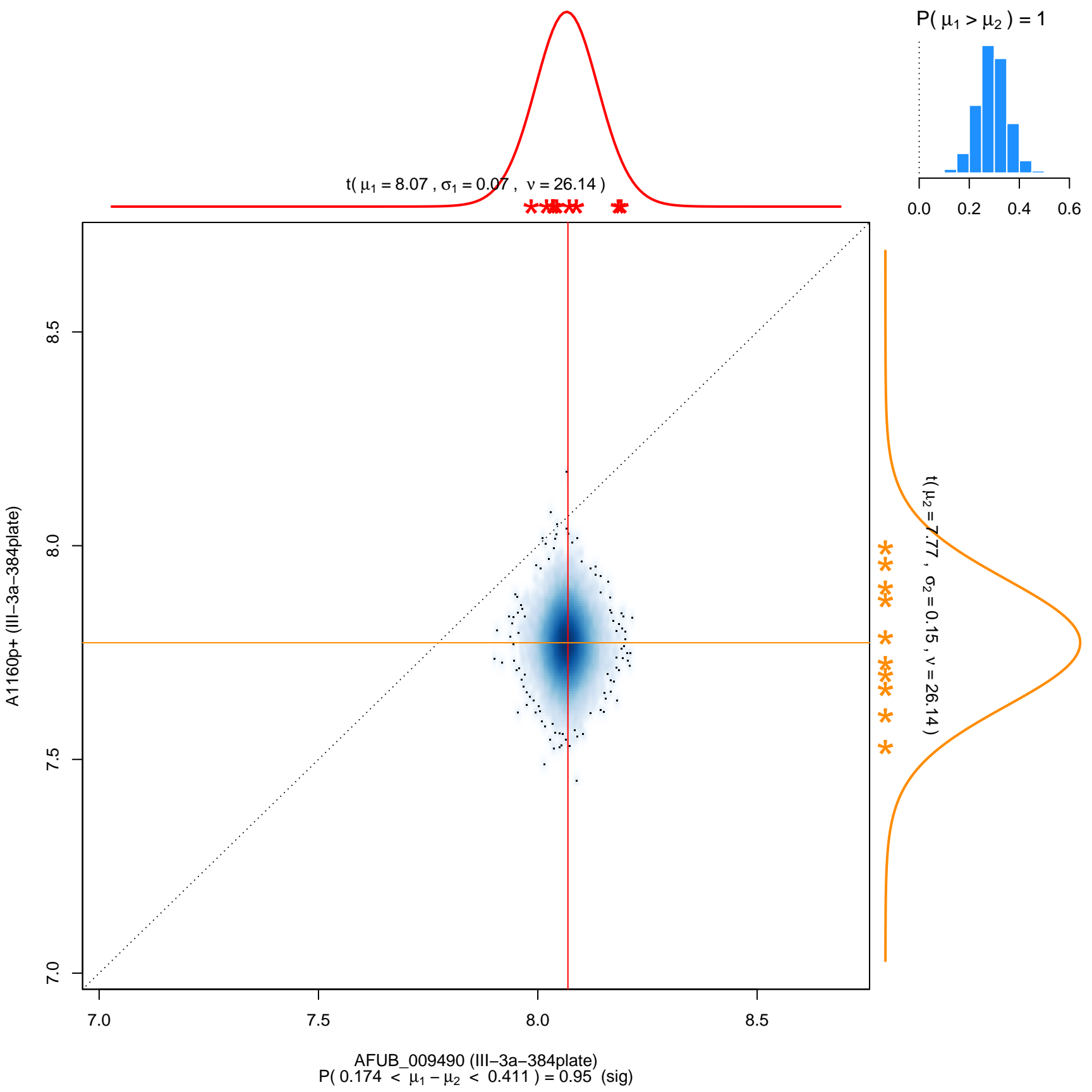

Supplement: Supplementary file 1 [file DataSheet_3.pdf]

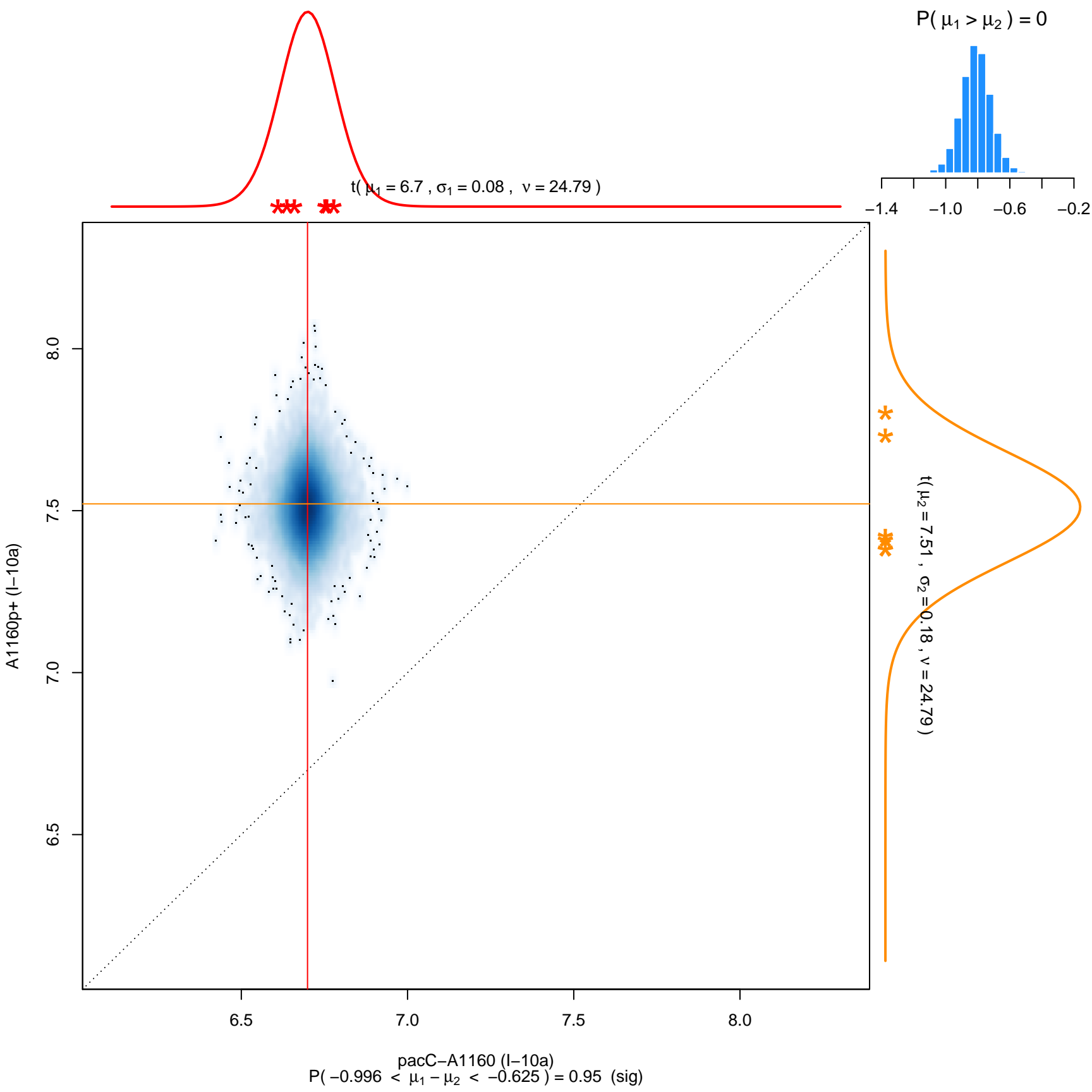

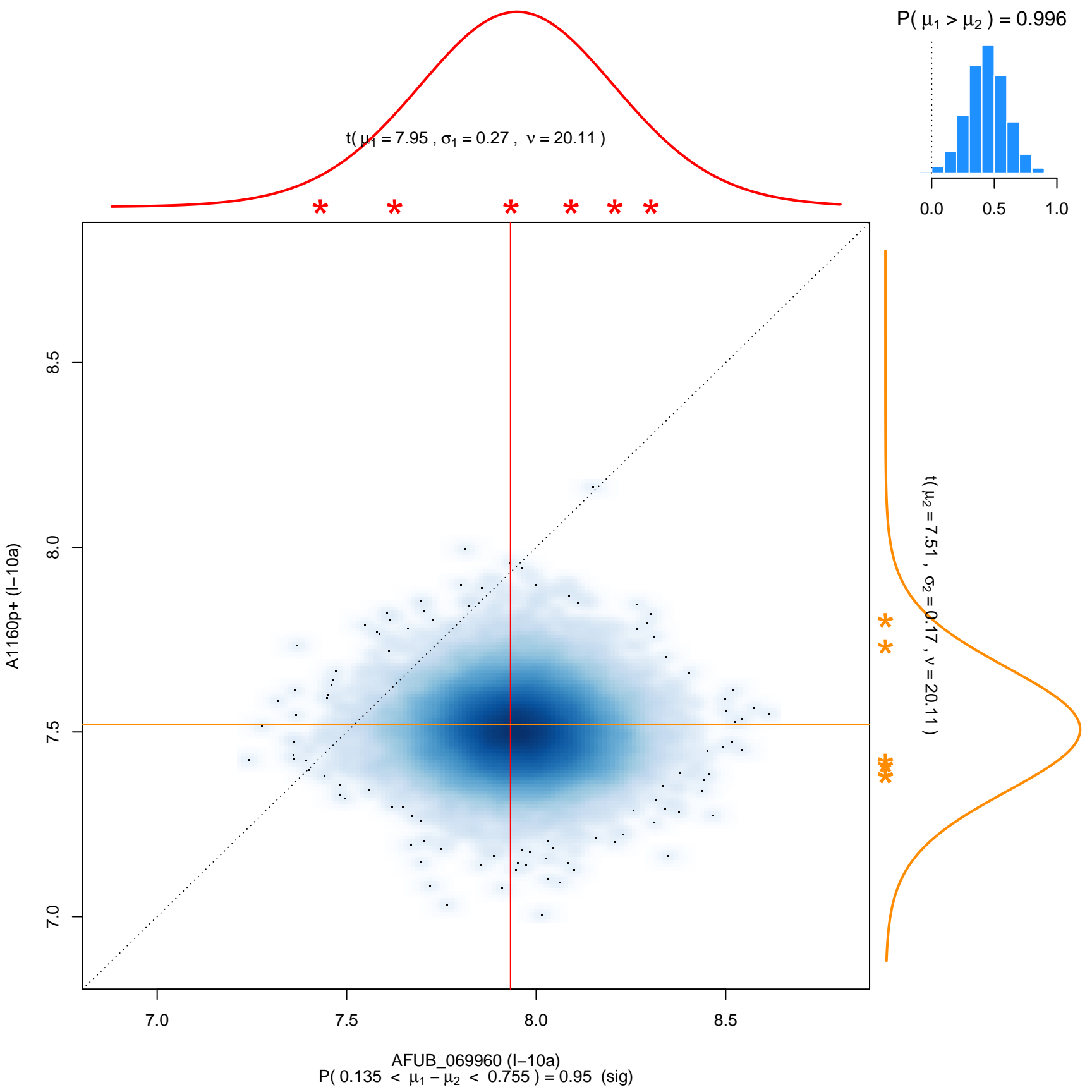

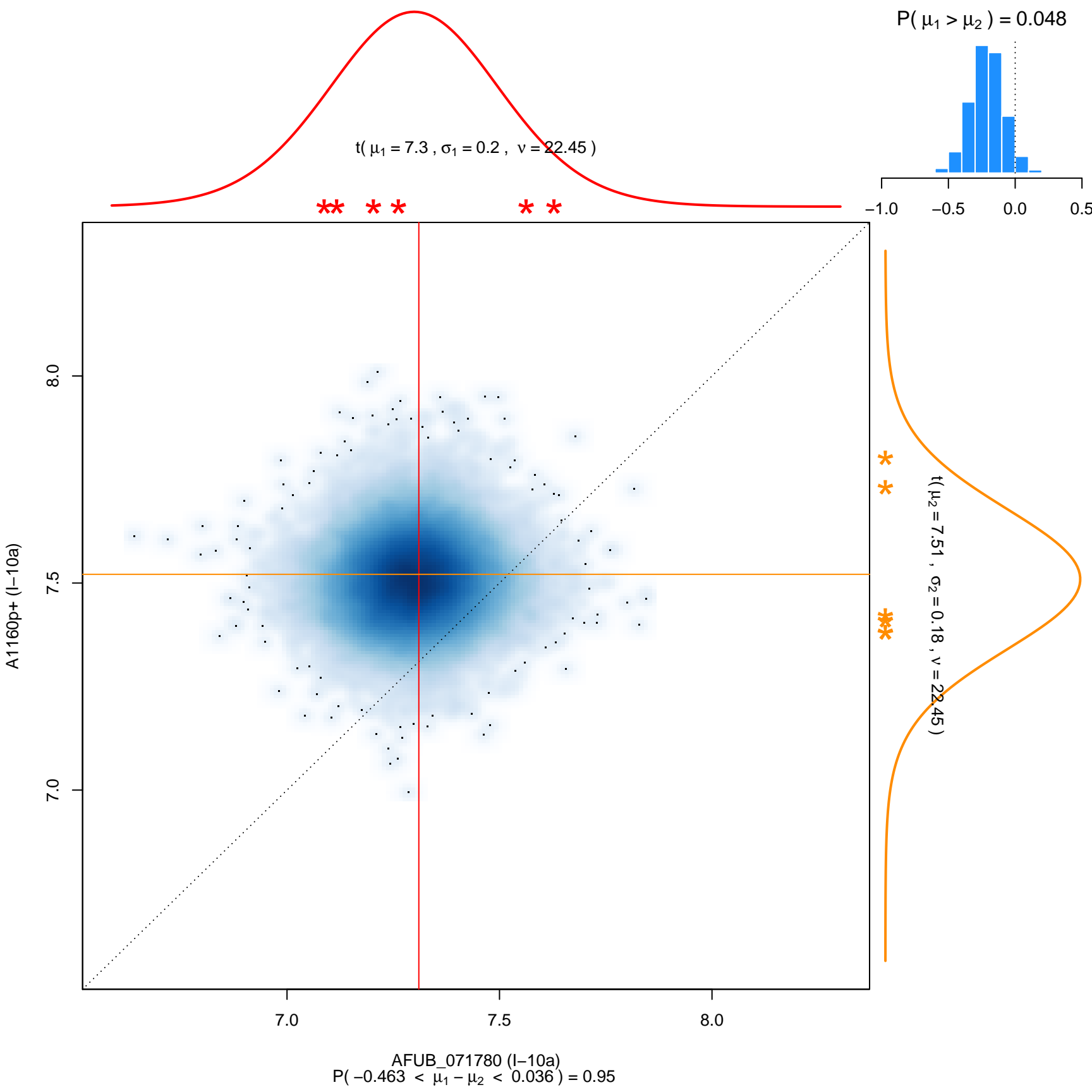

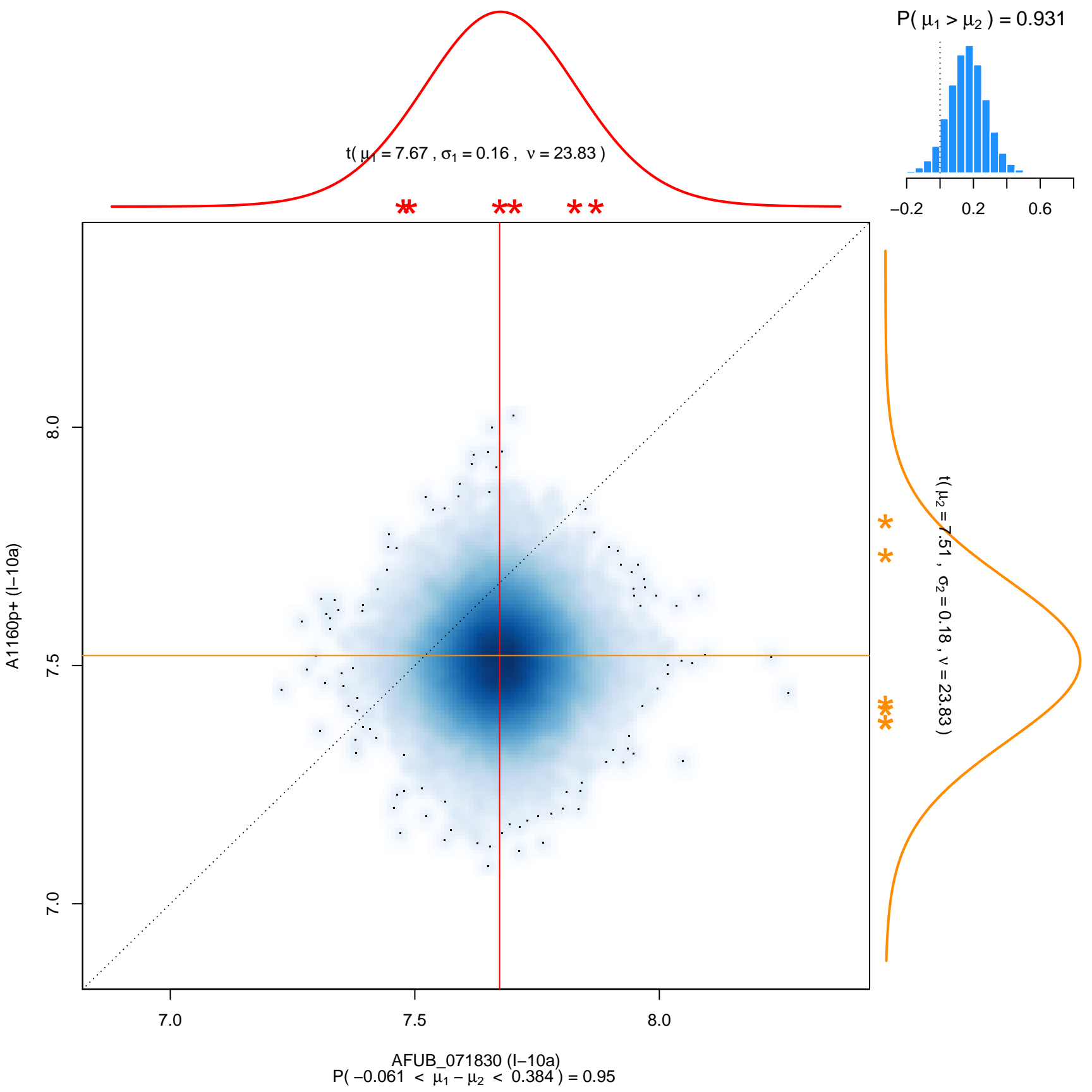

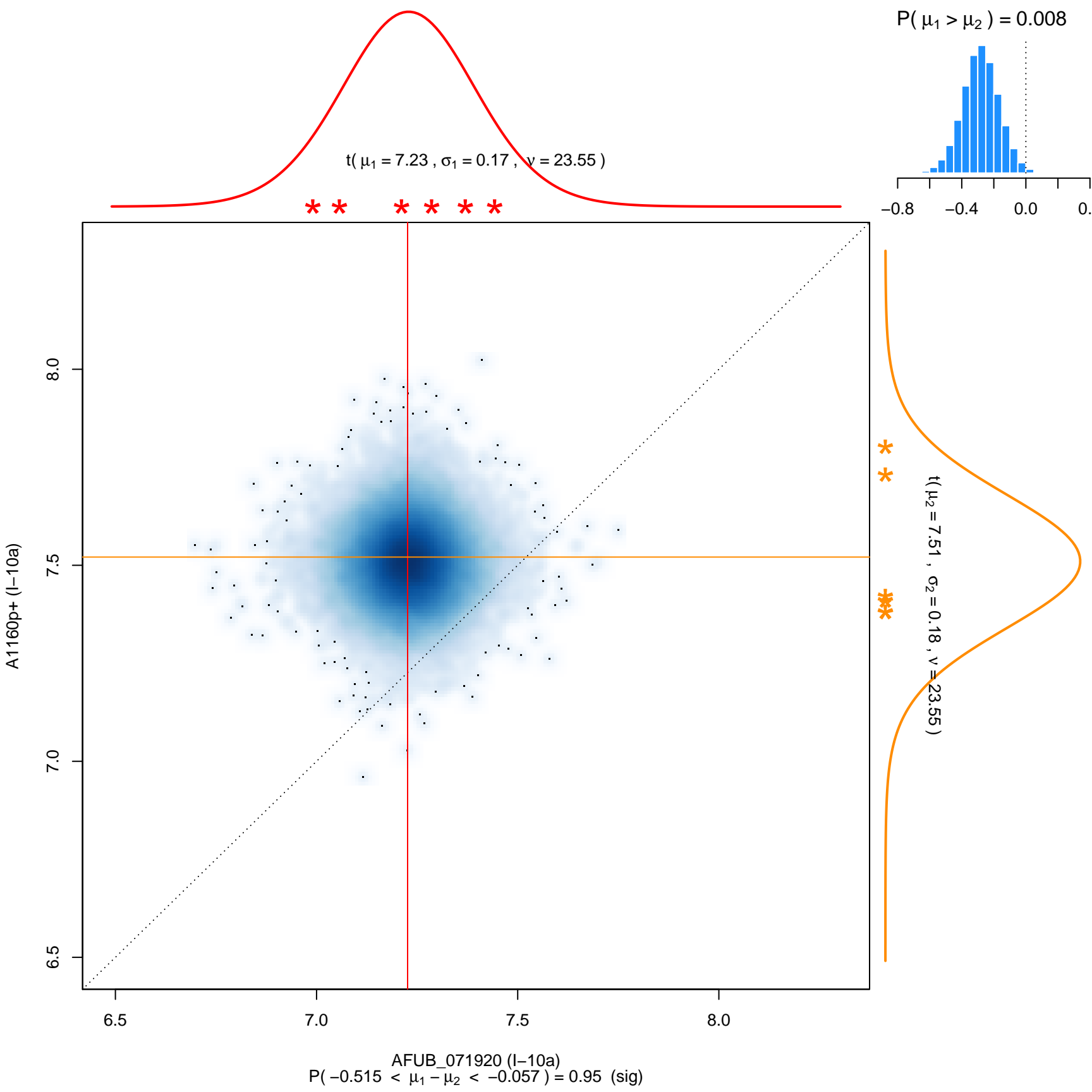

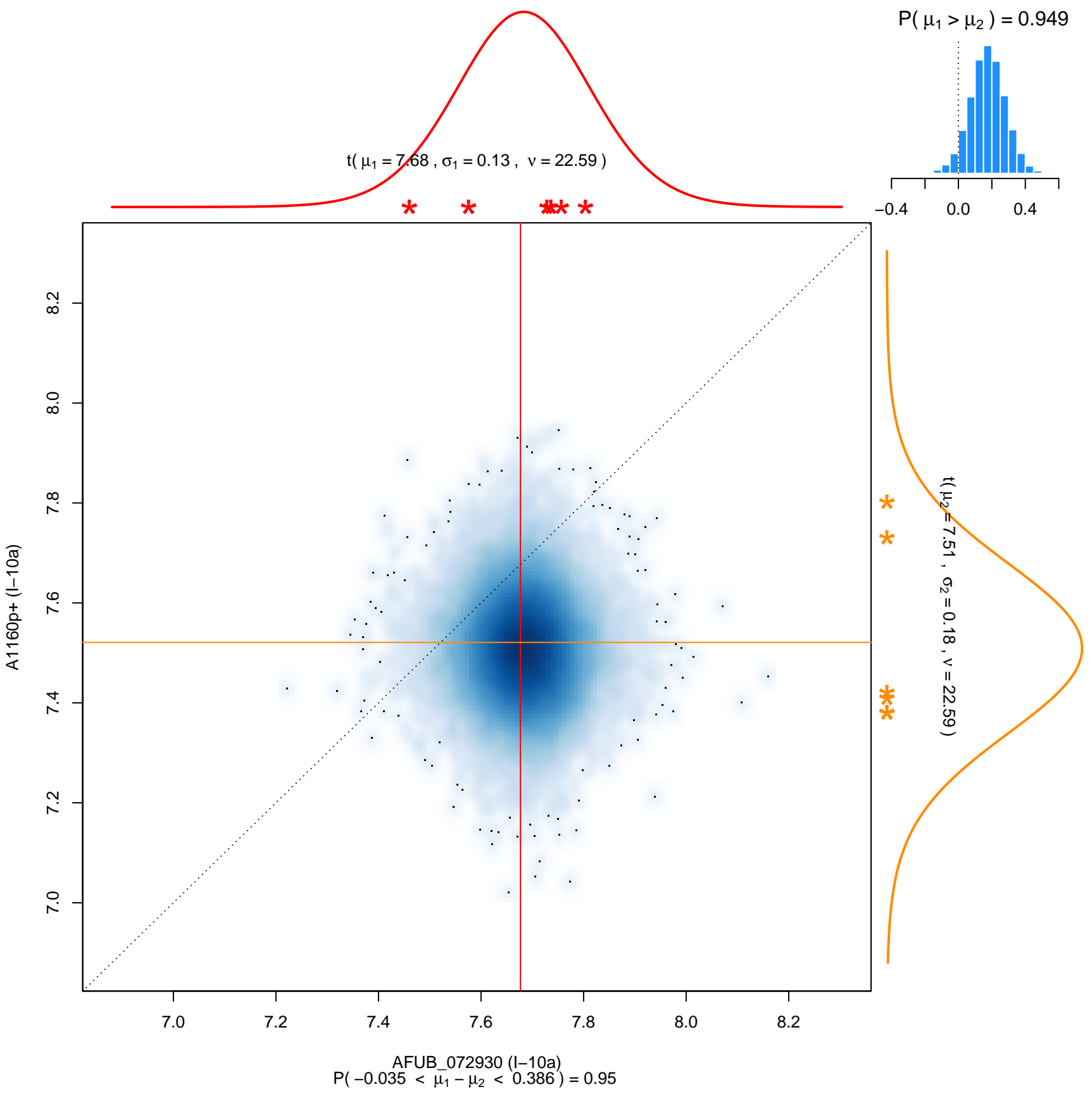

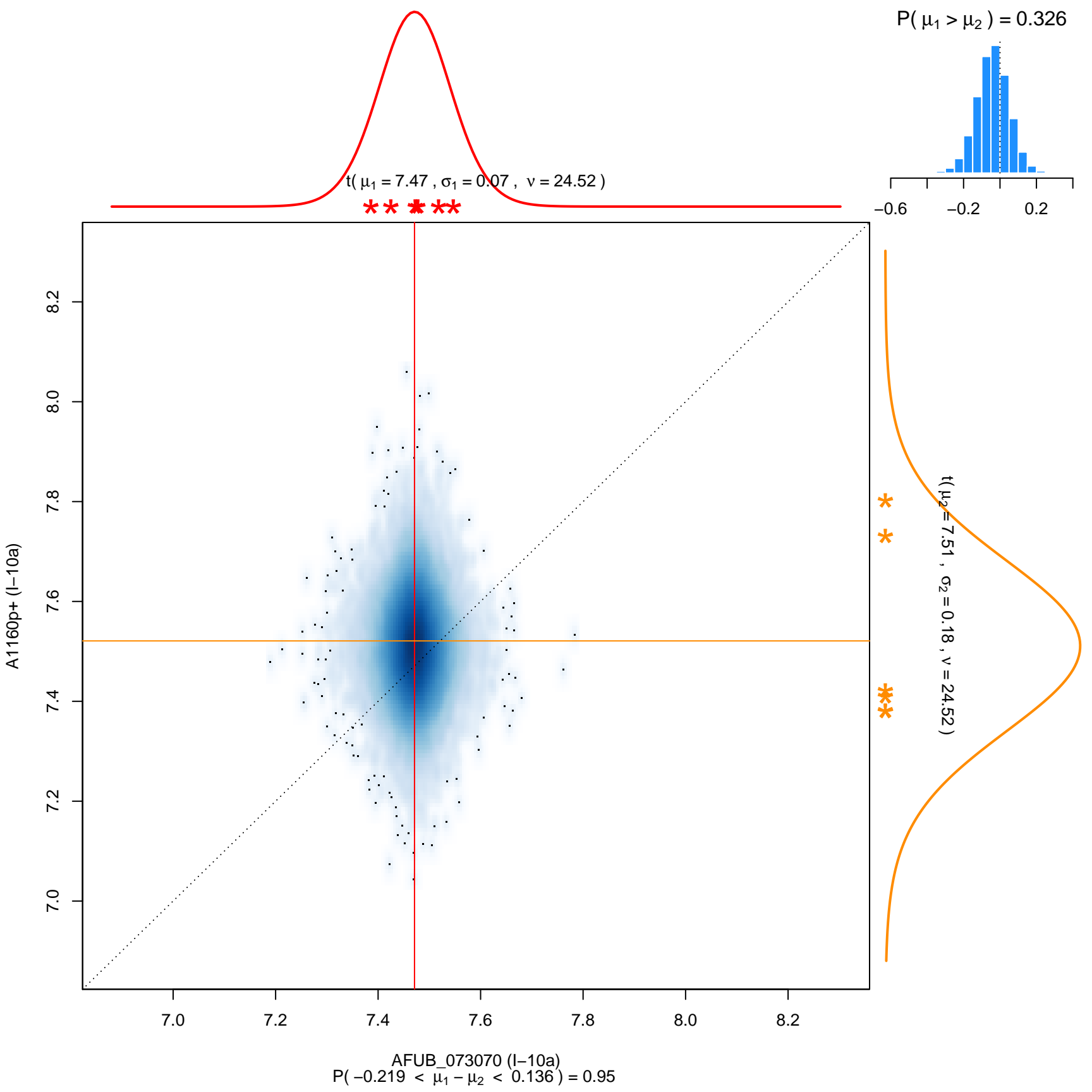

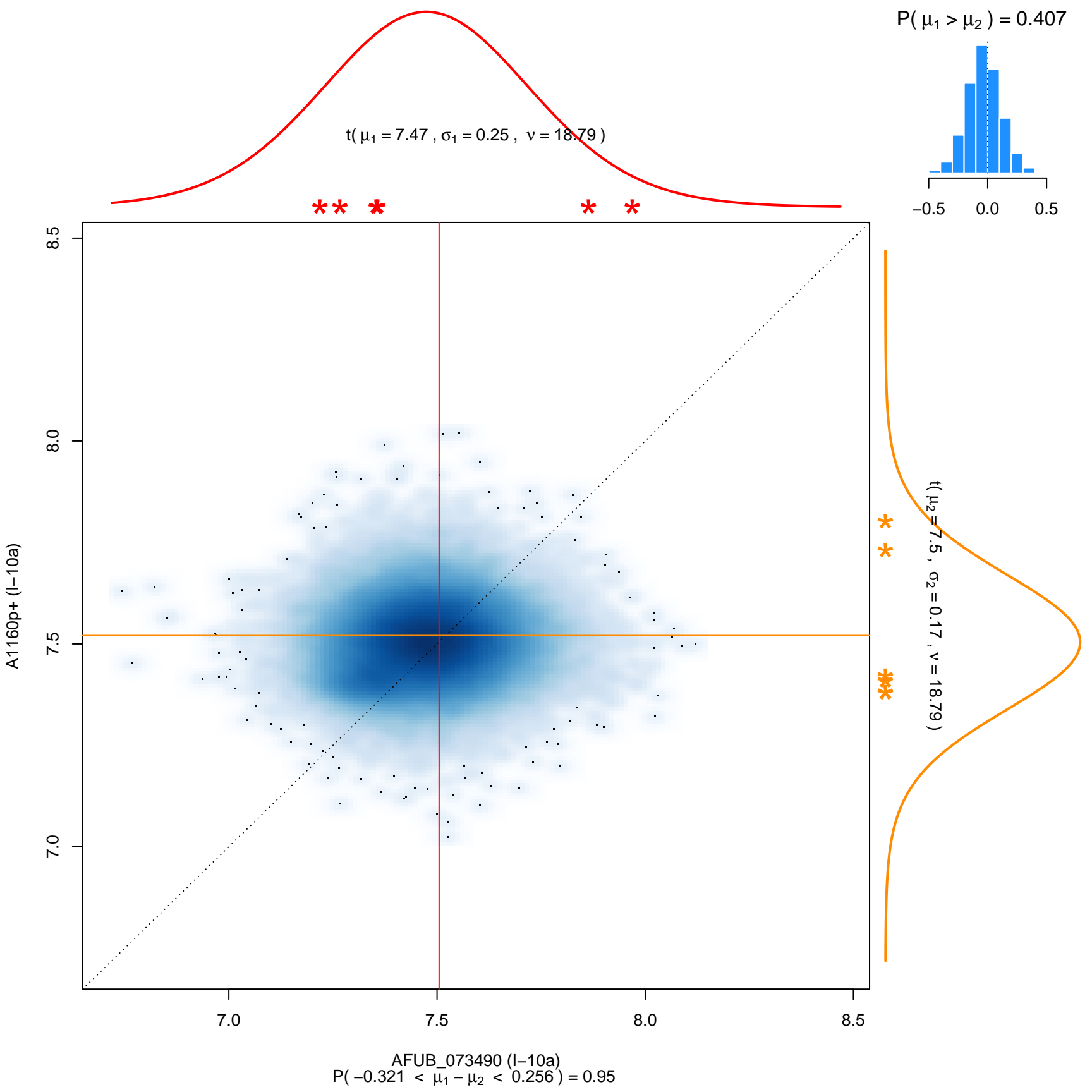

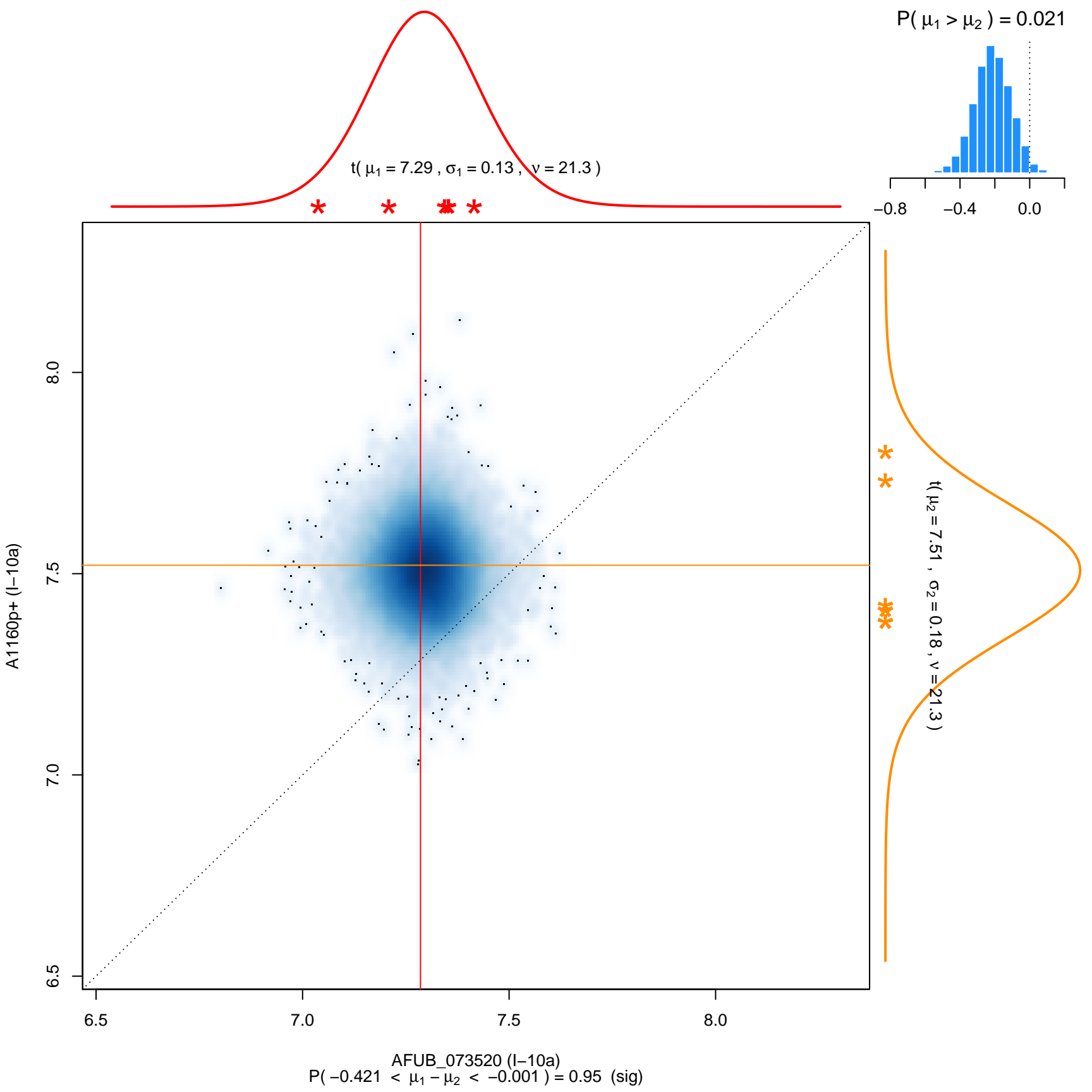

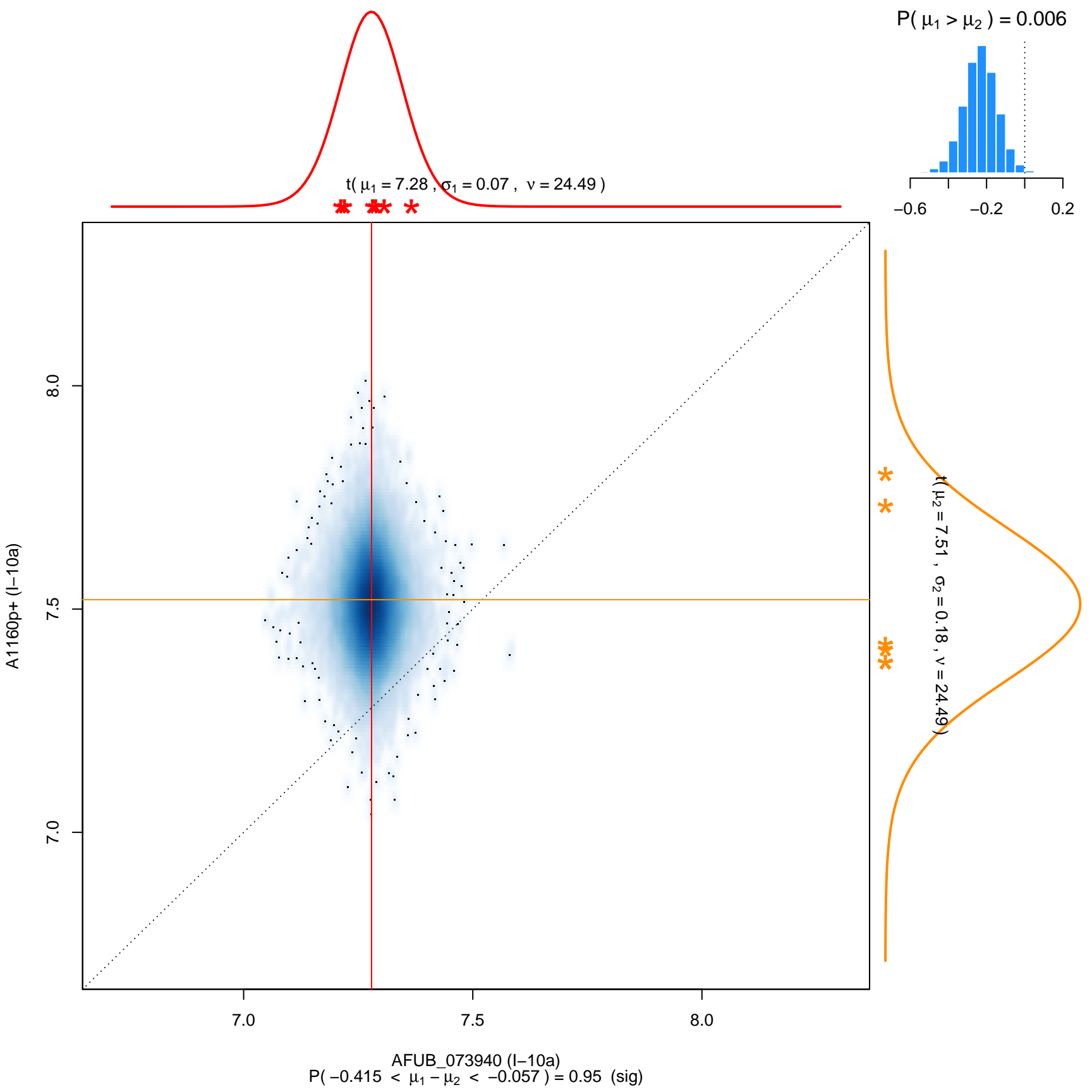

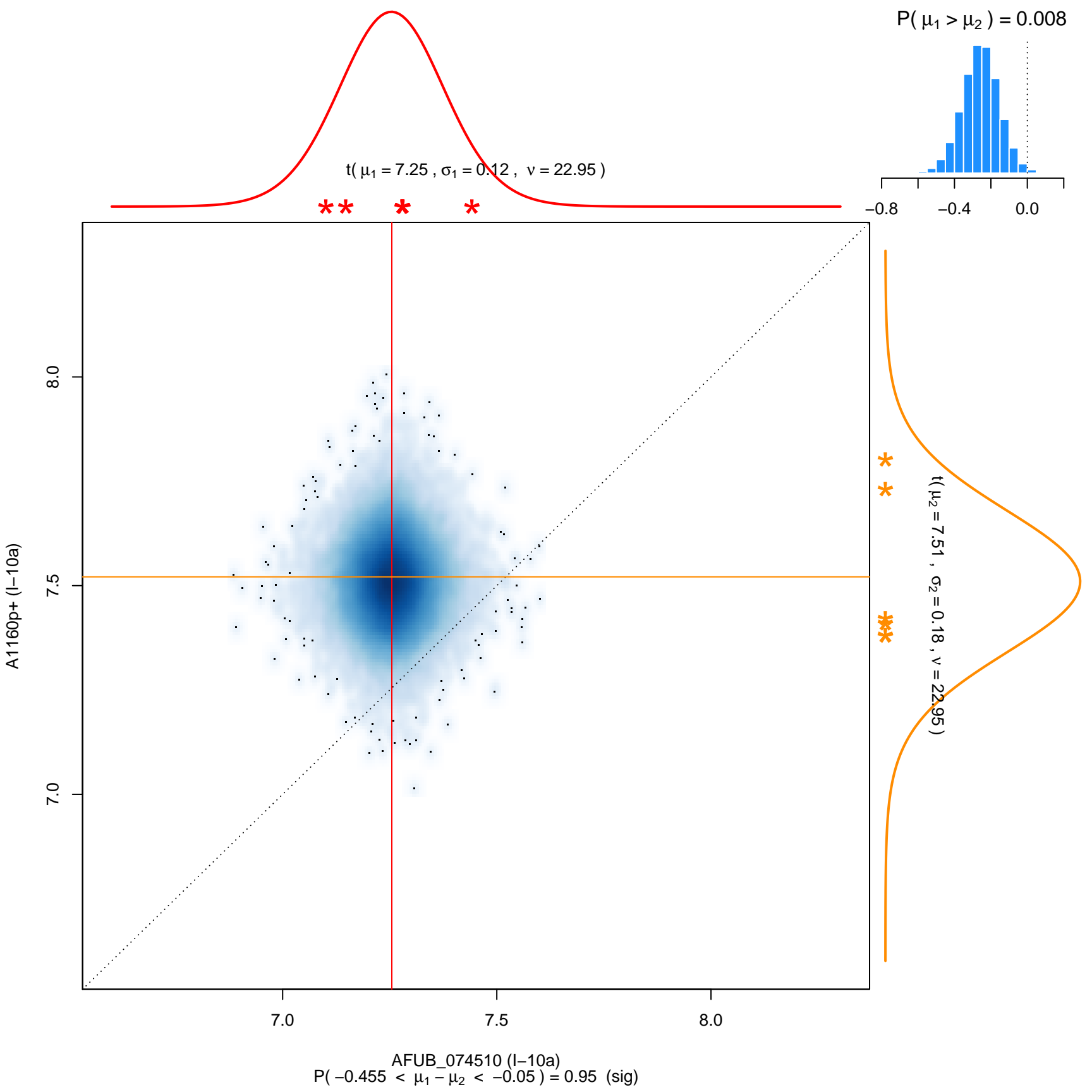

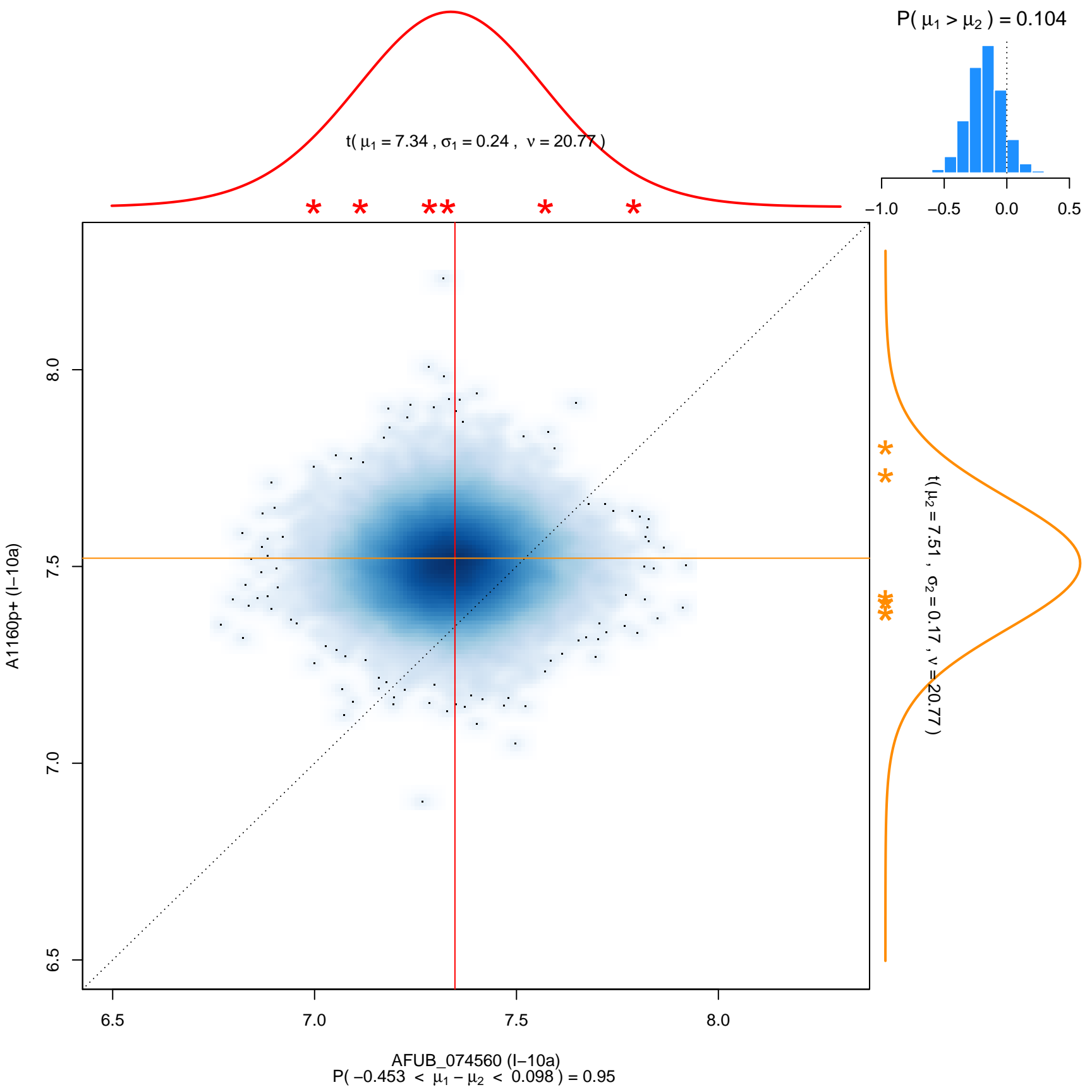

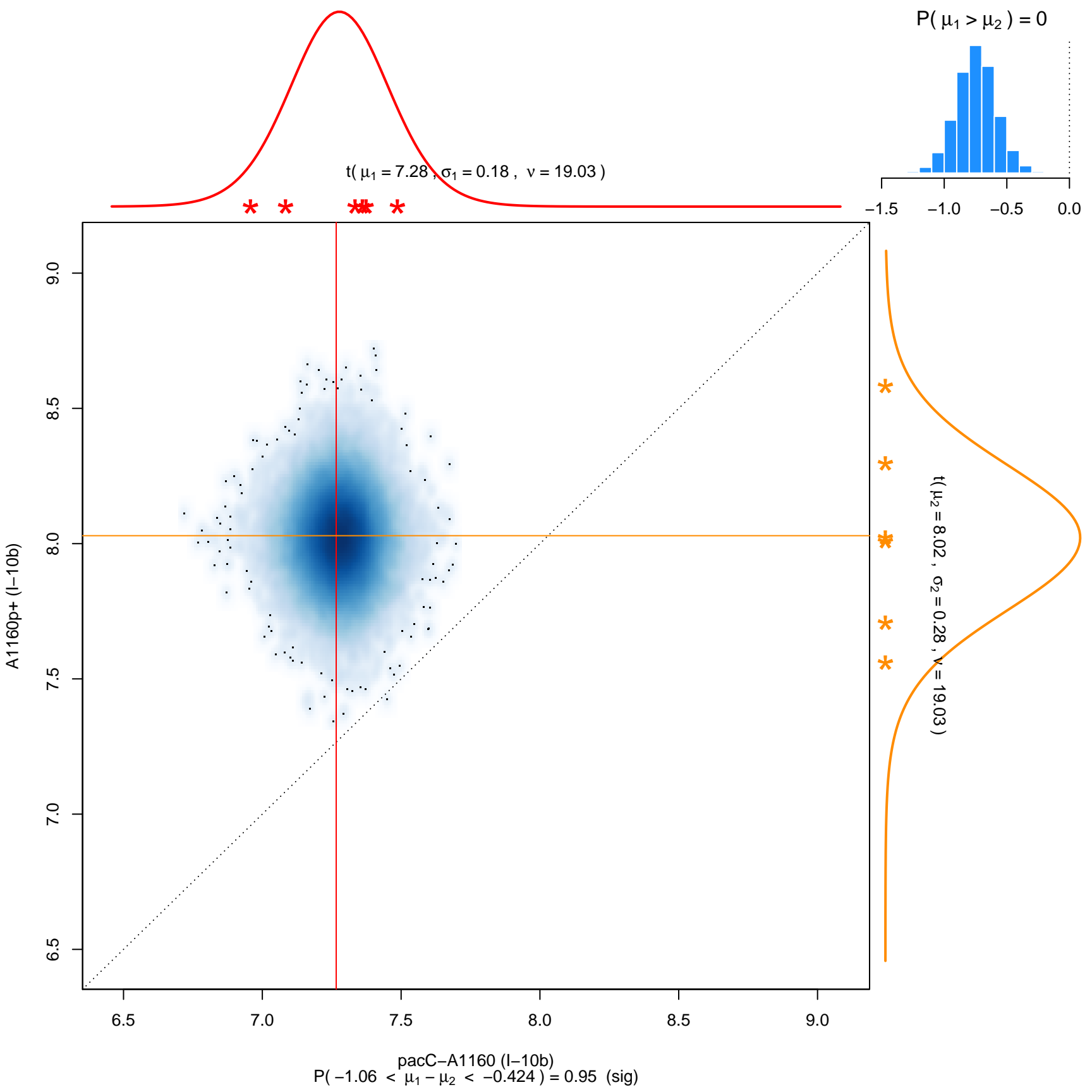

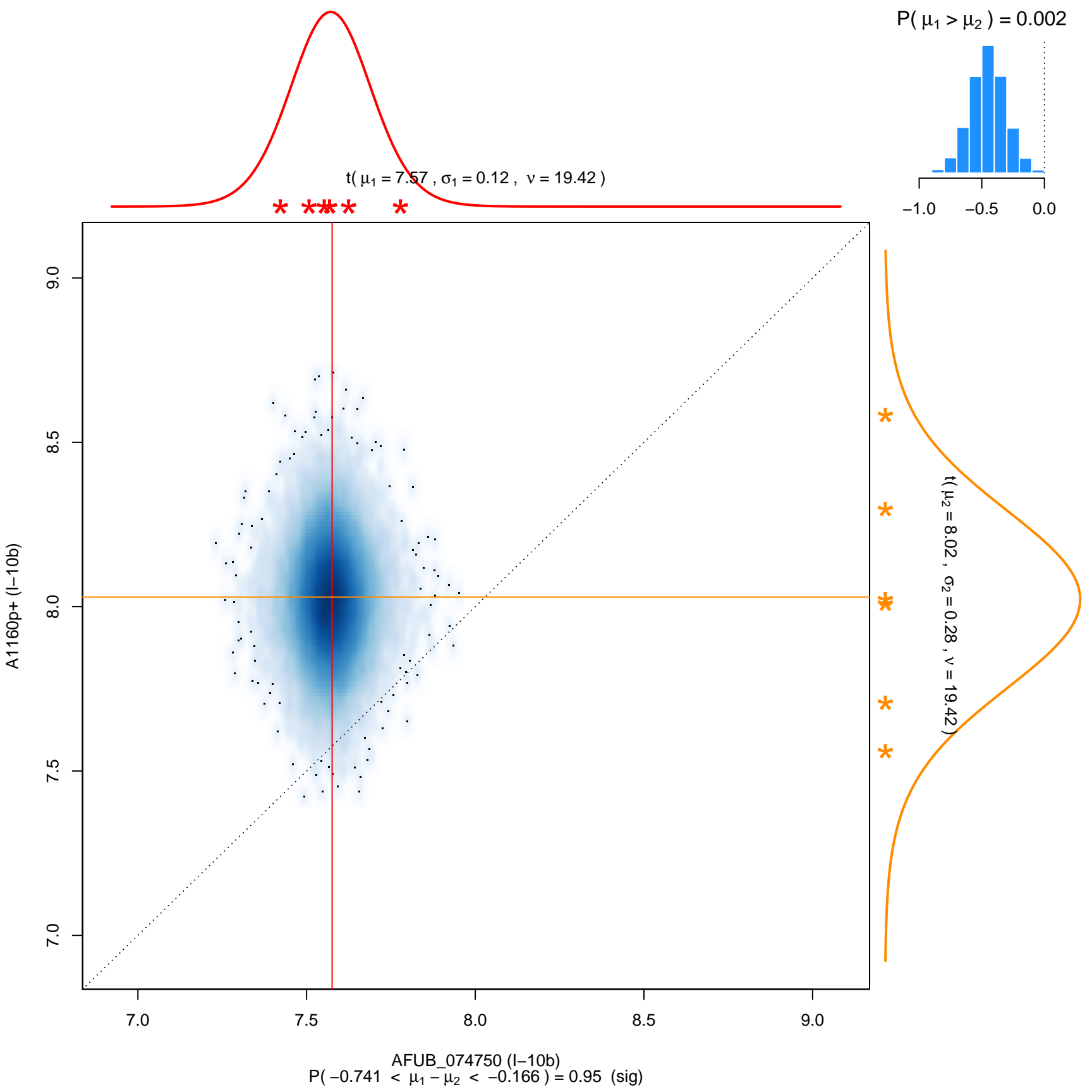

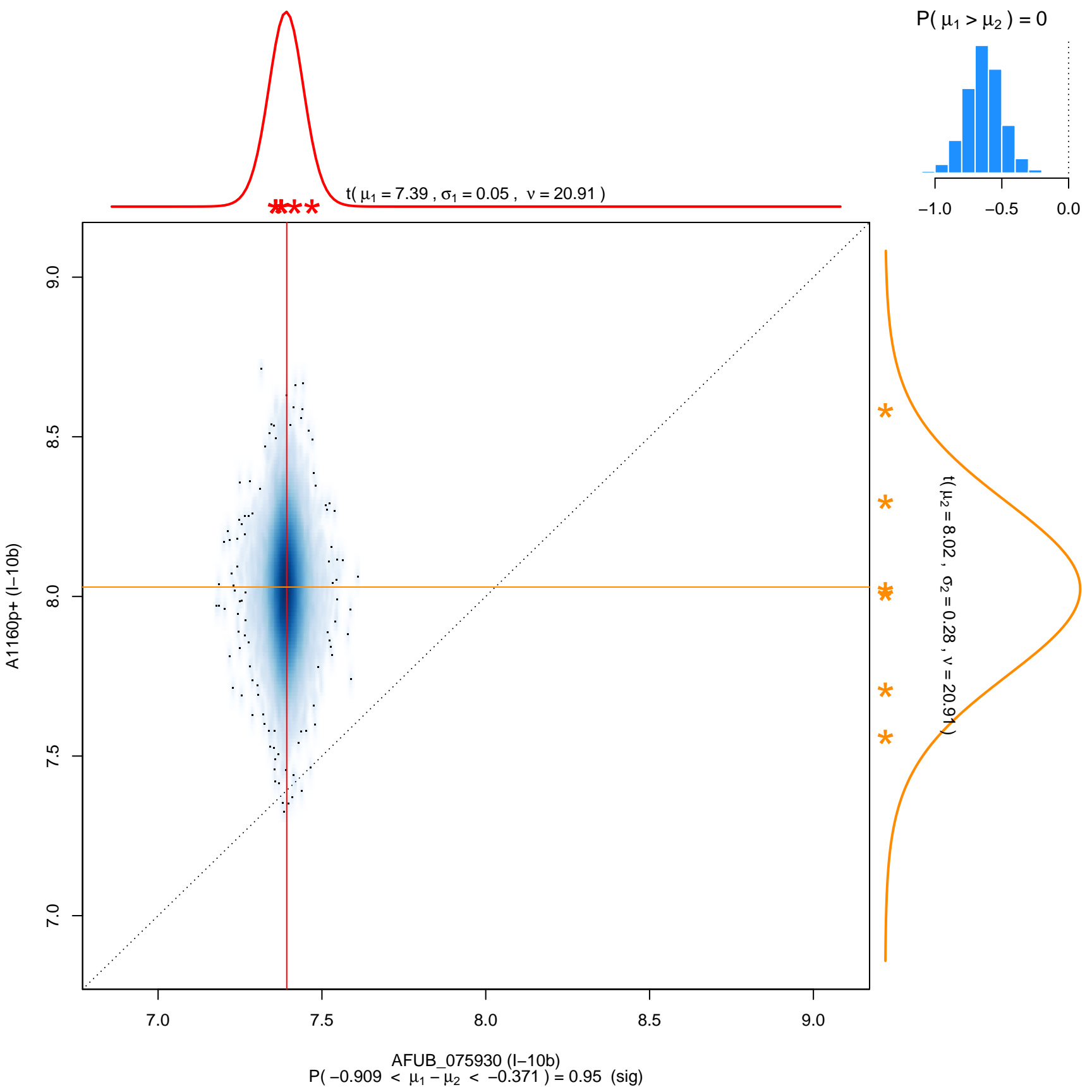

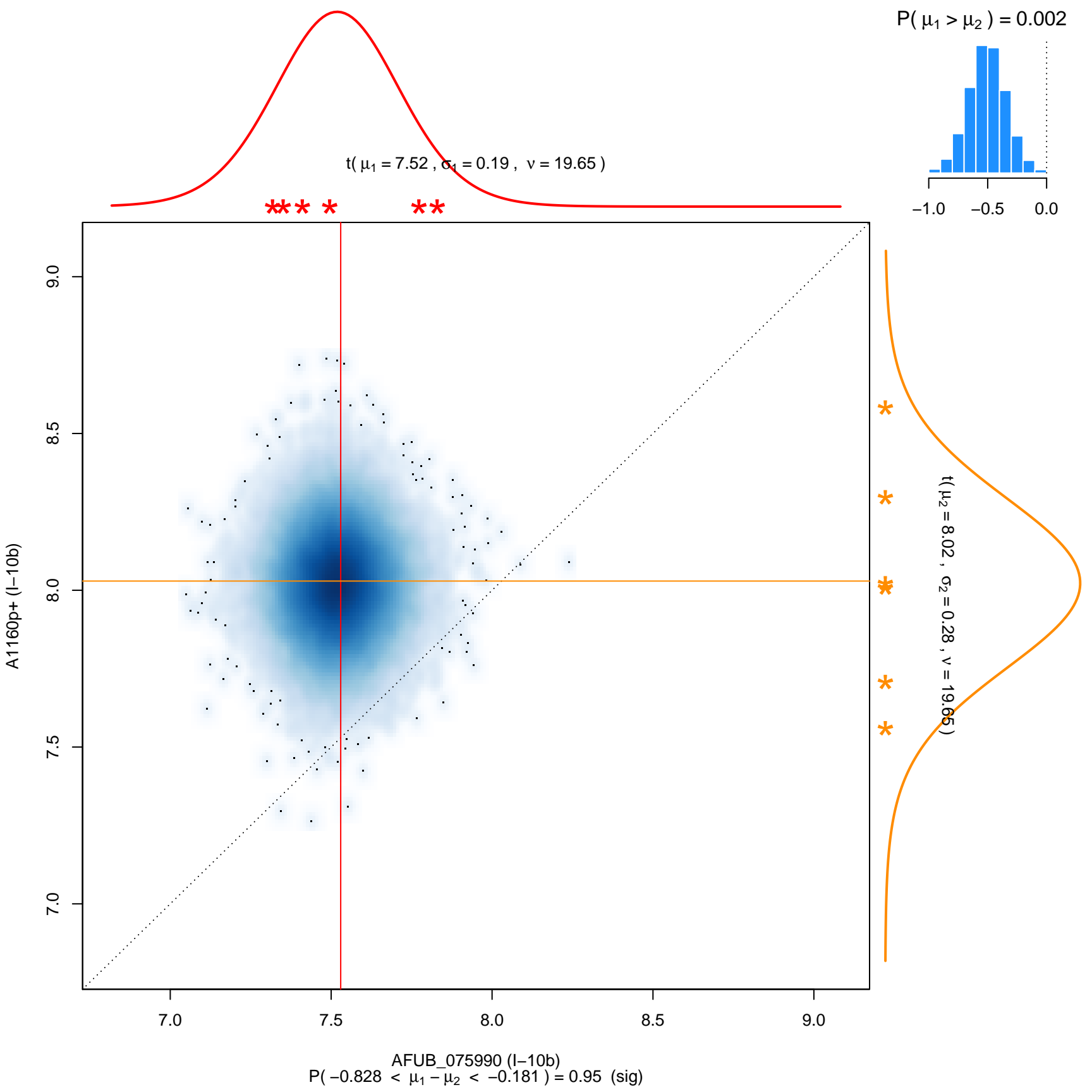

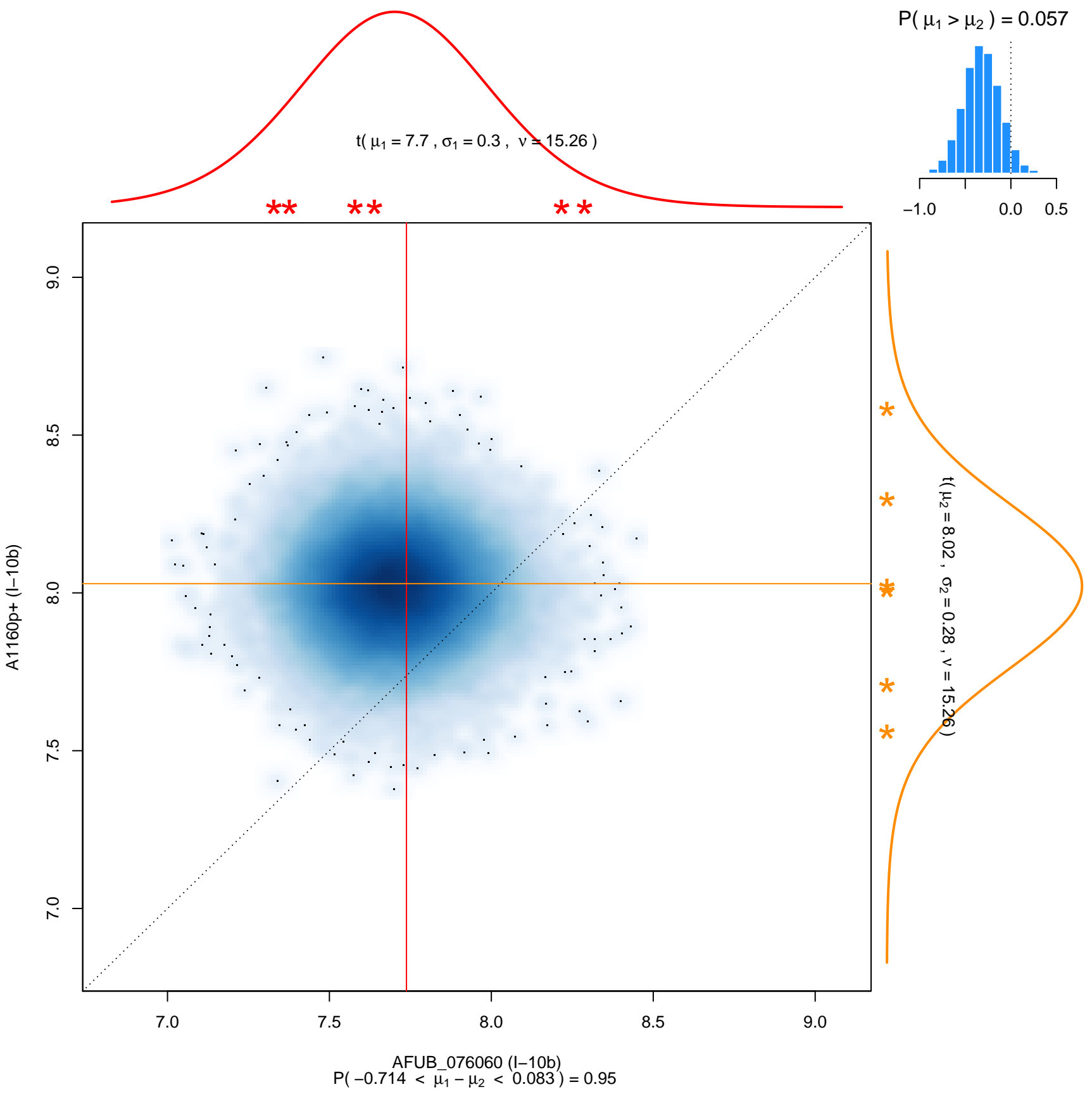

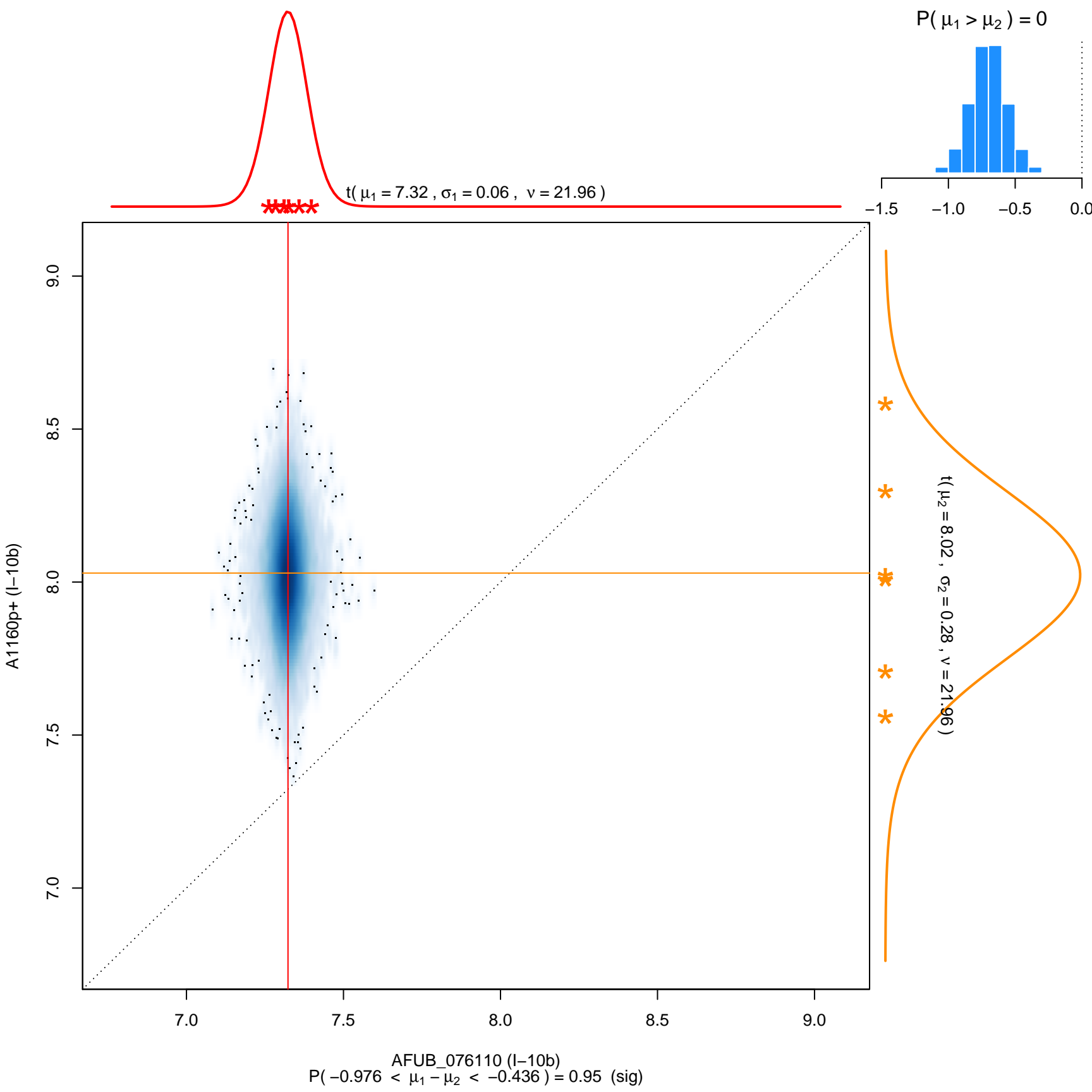

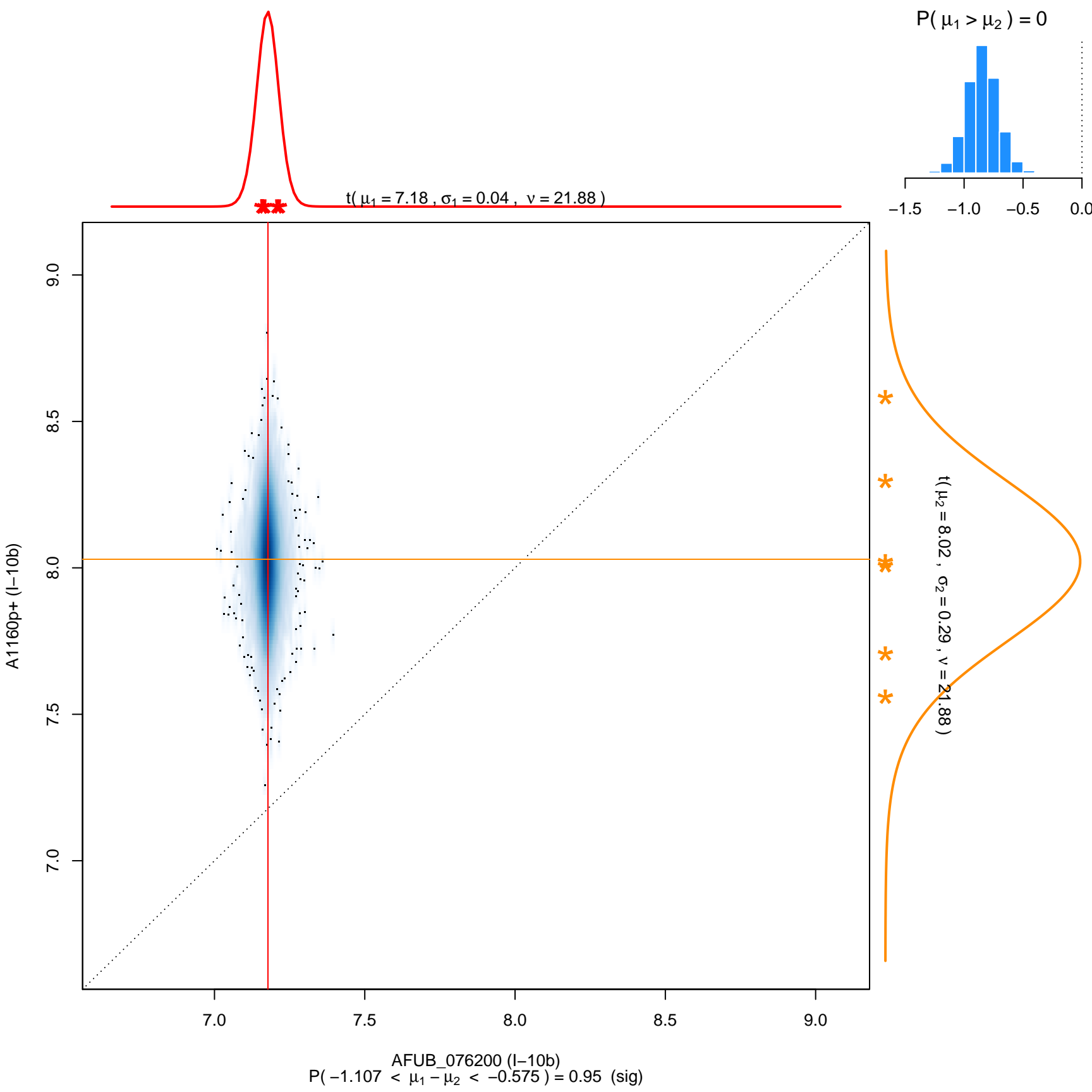

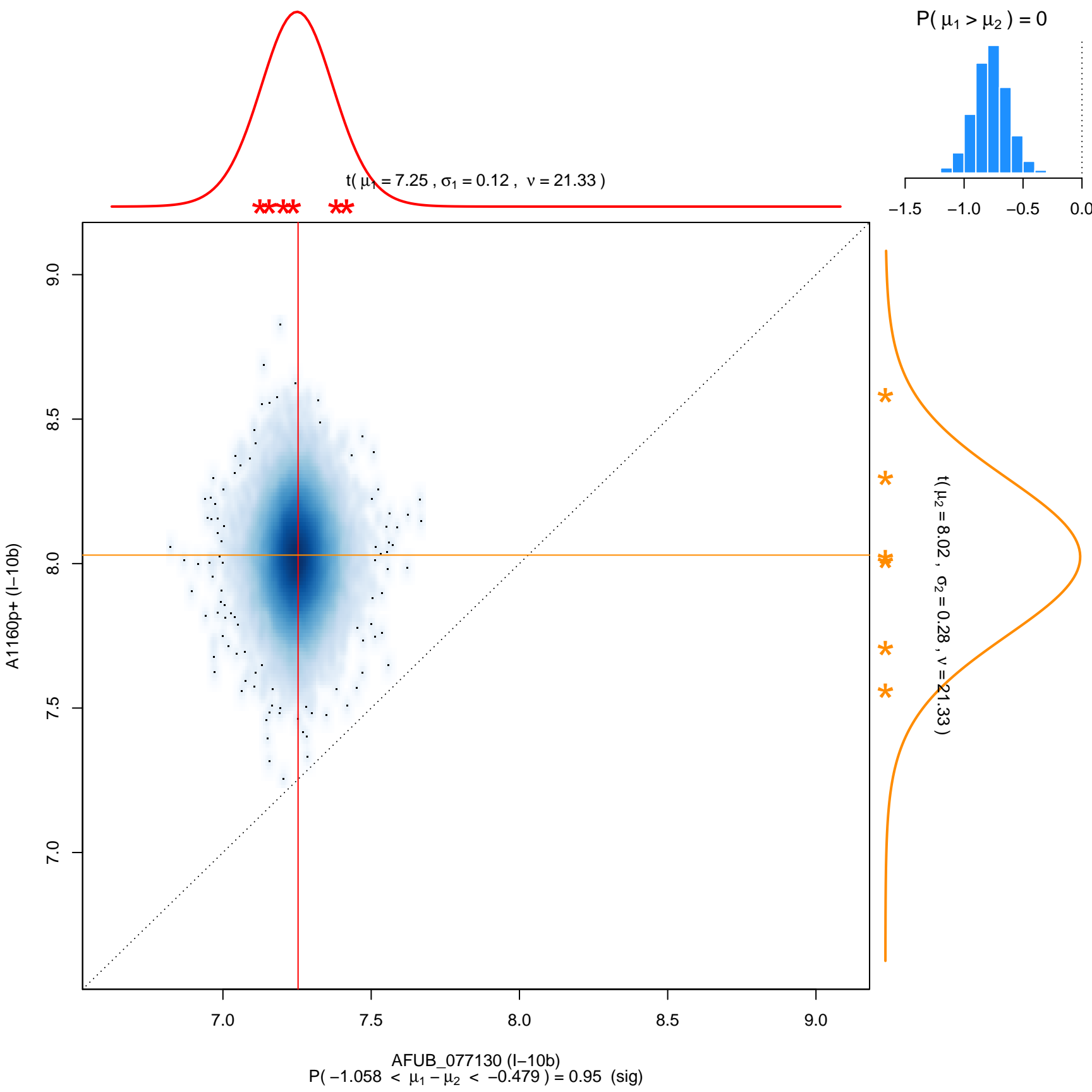

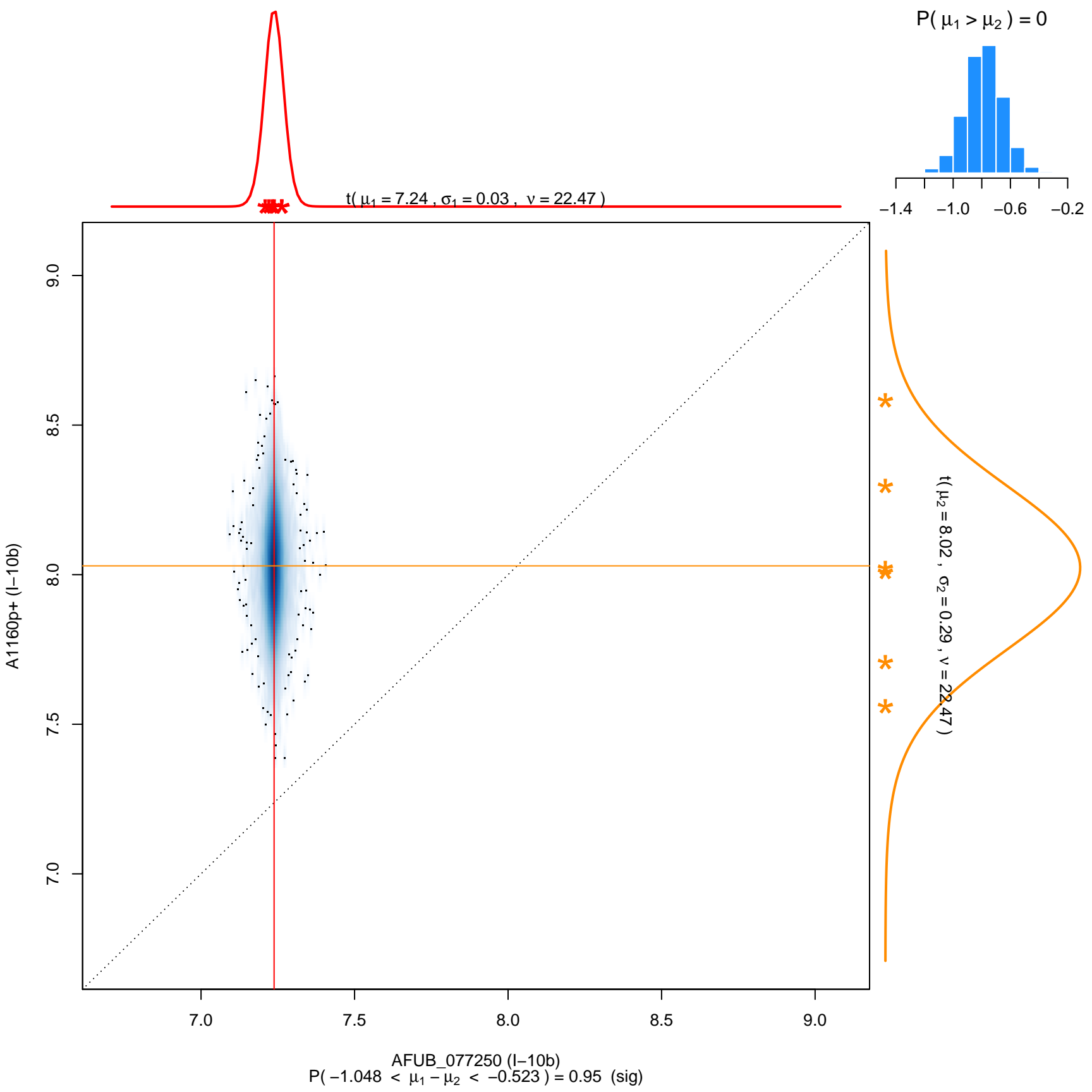

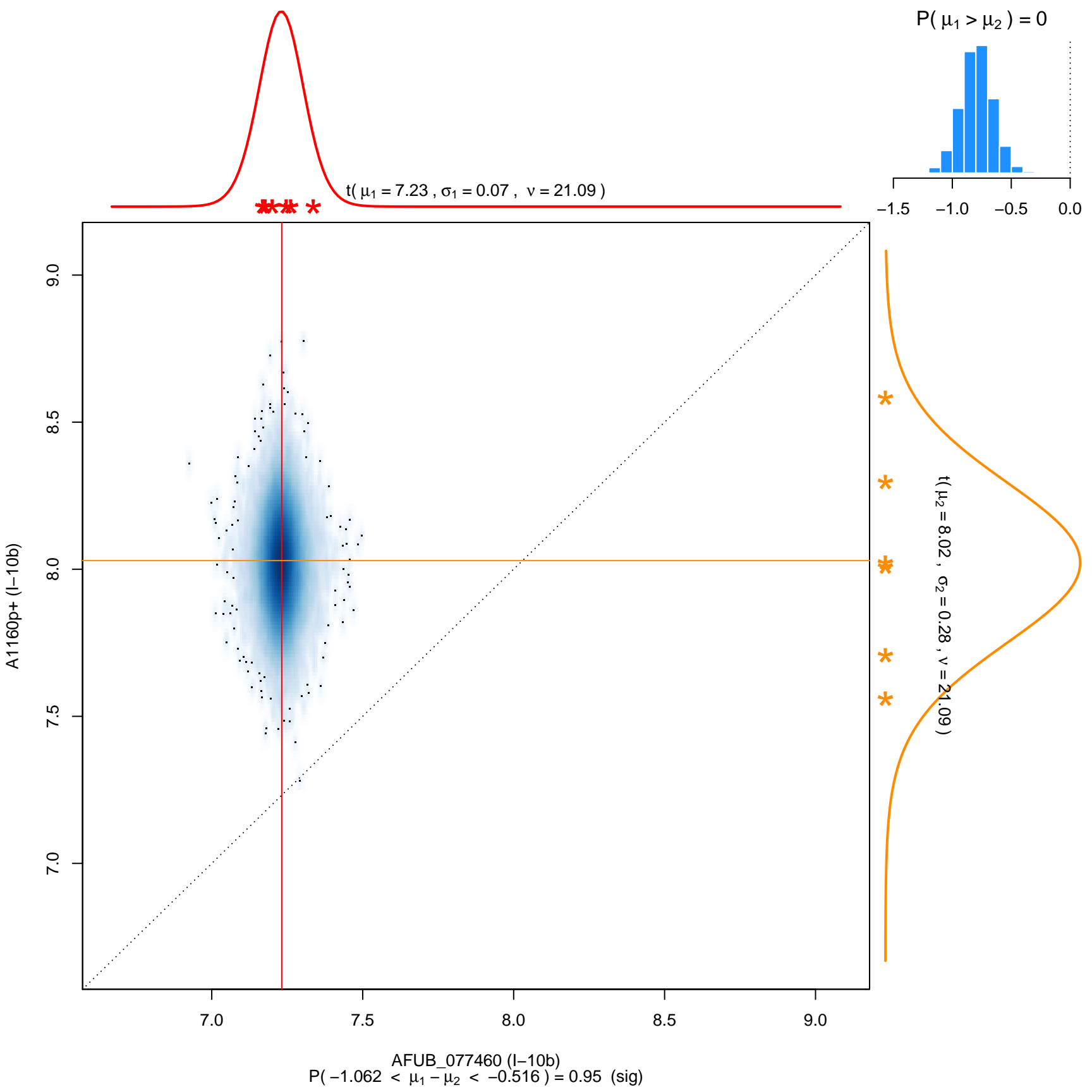

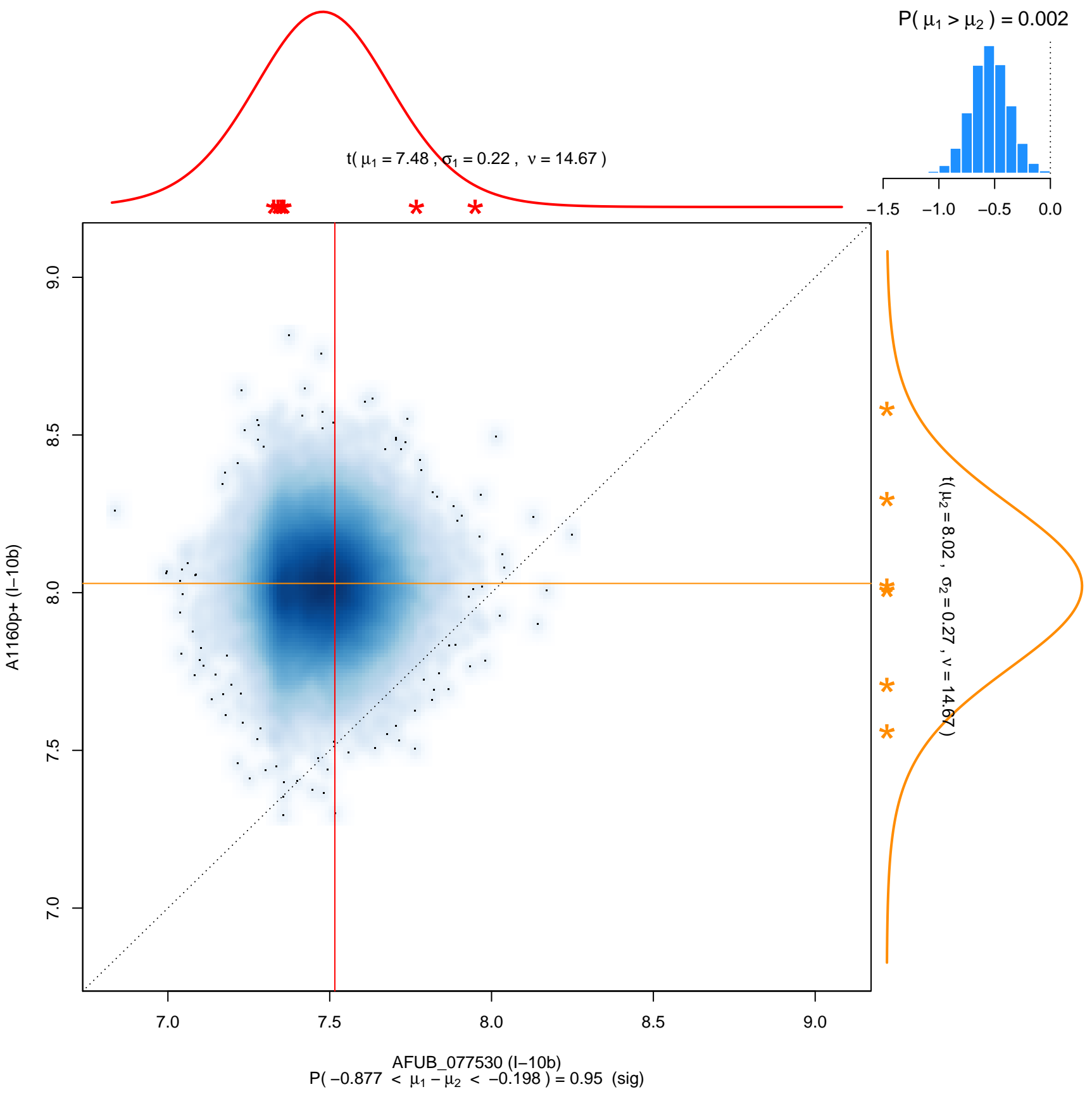

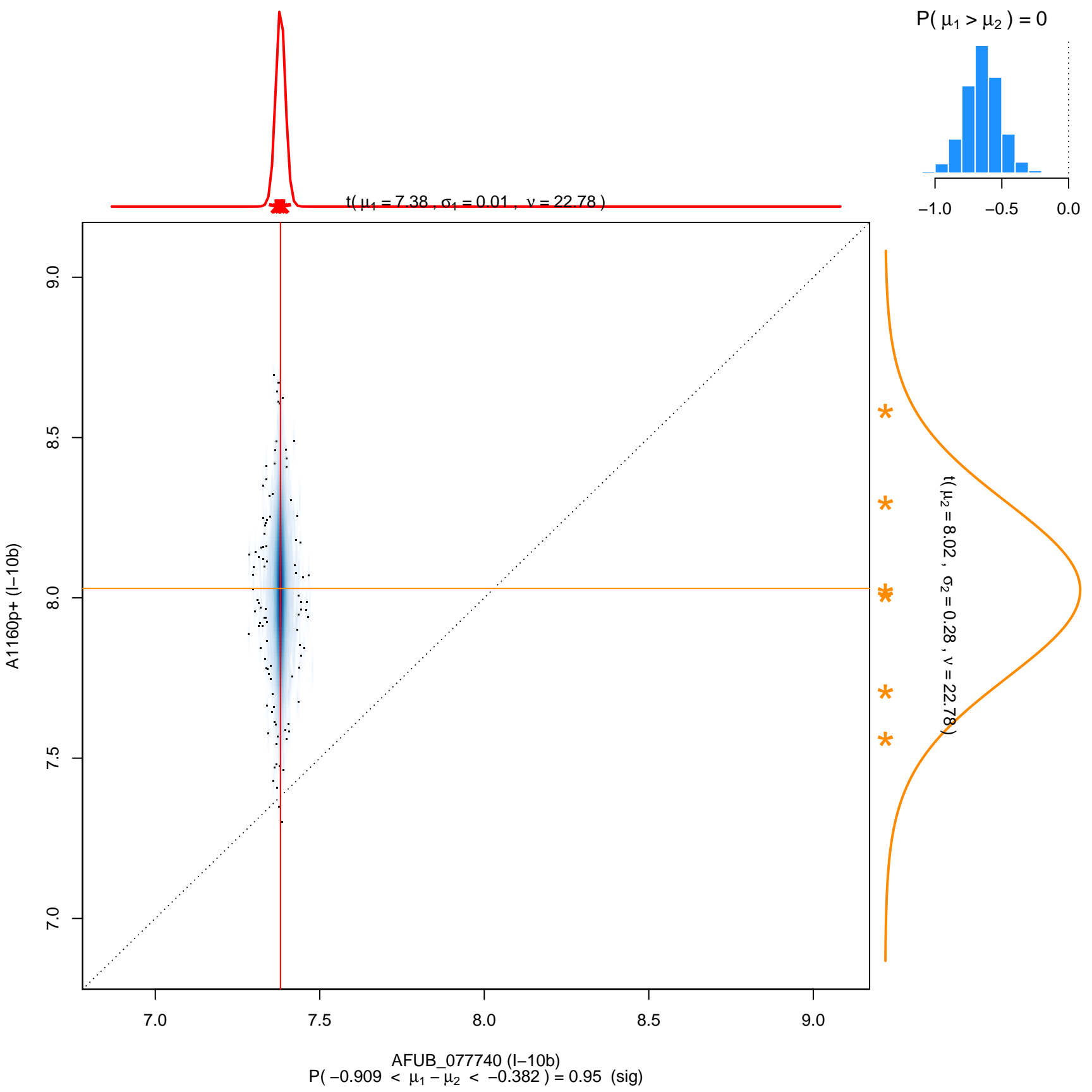

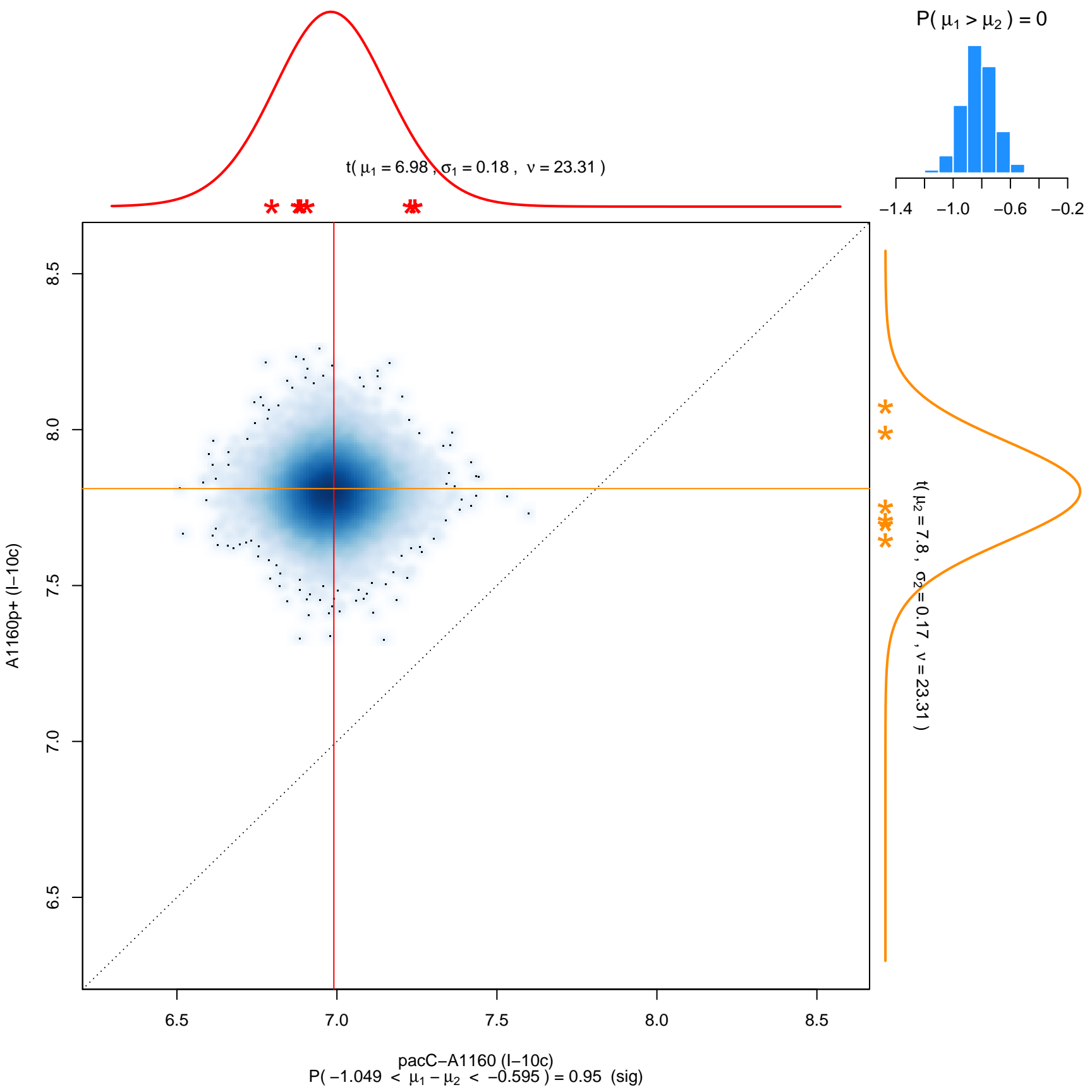

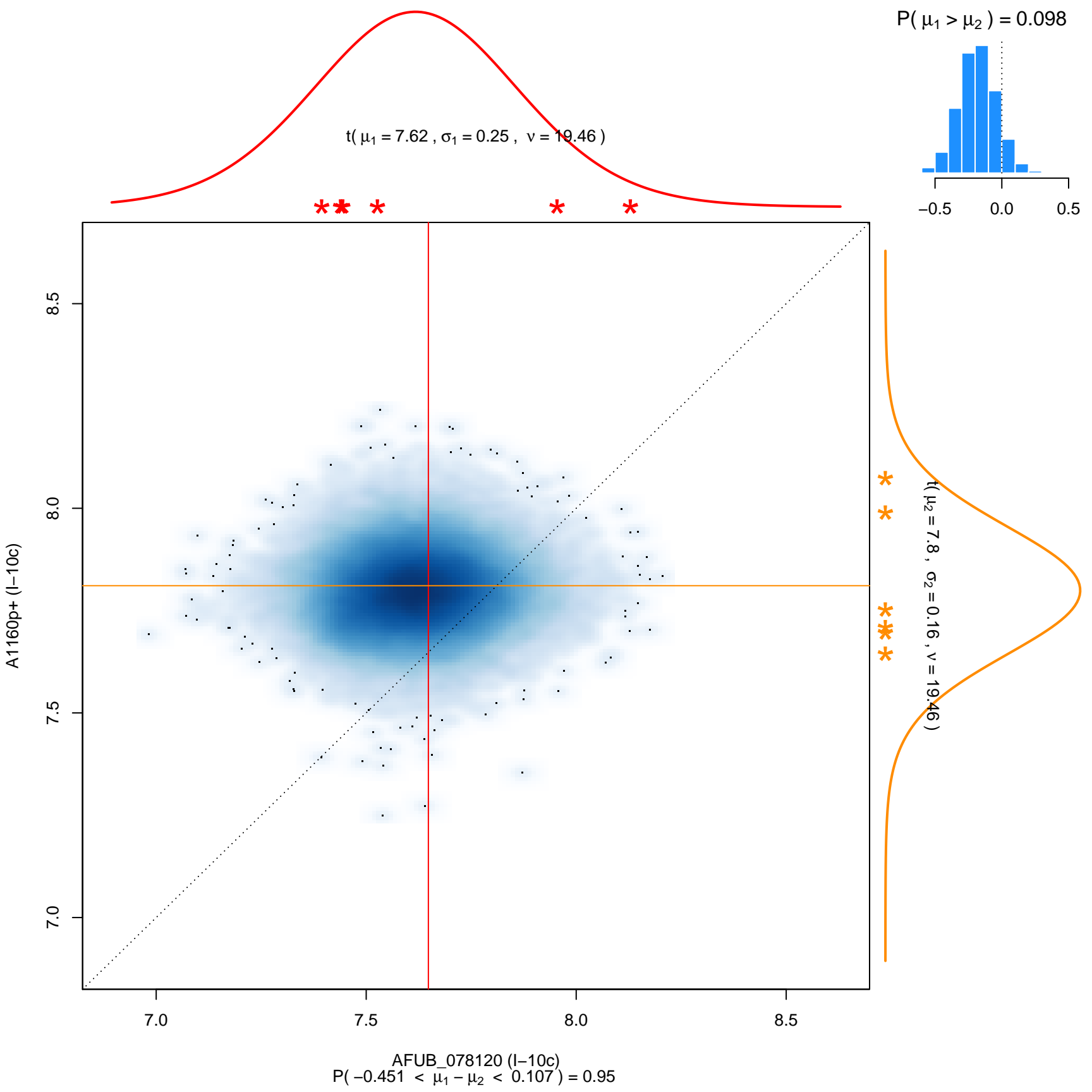

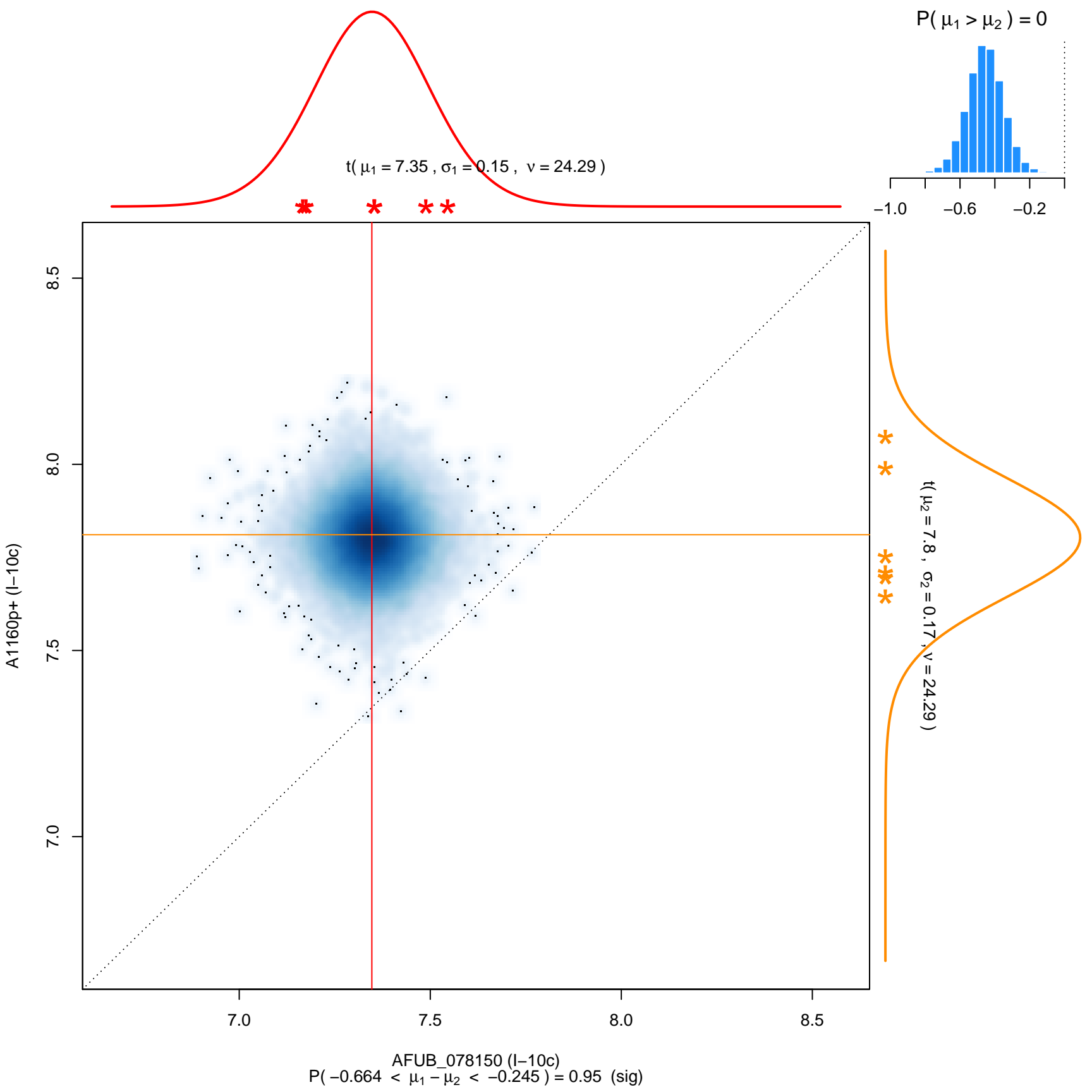

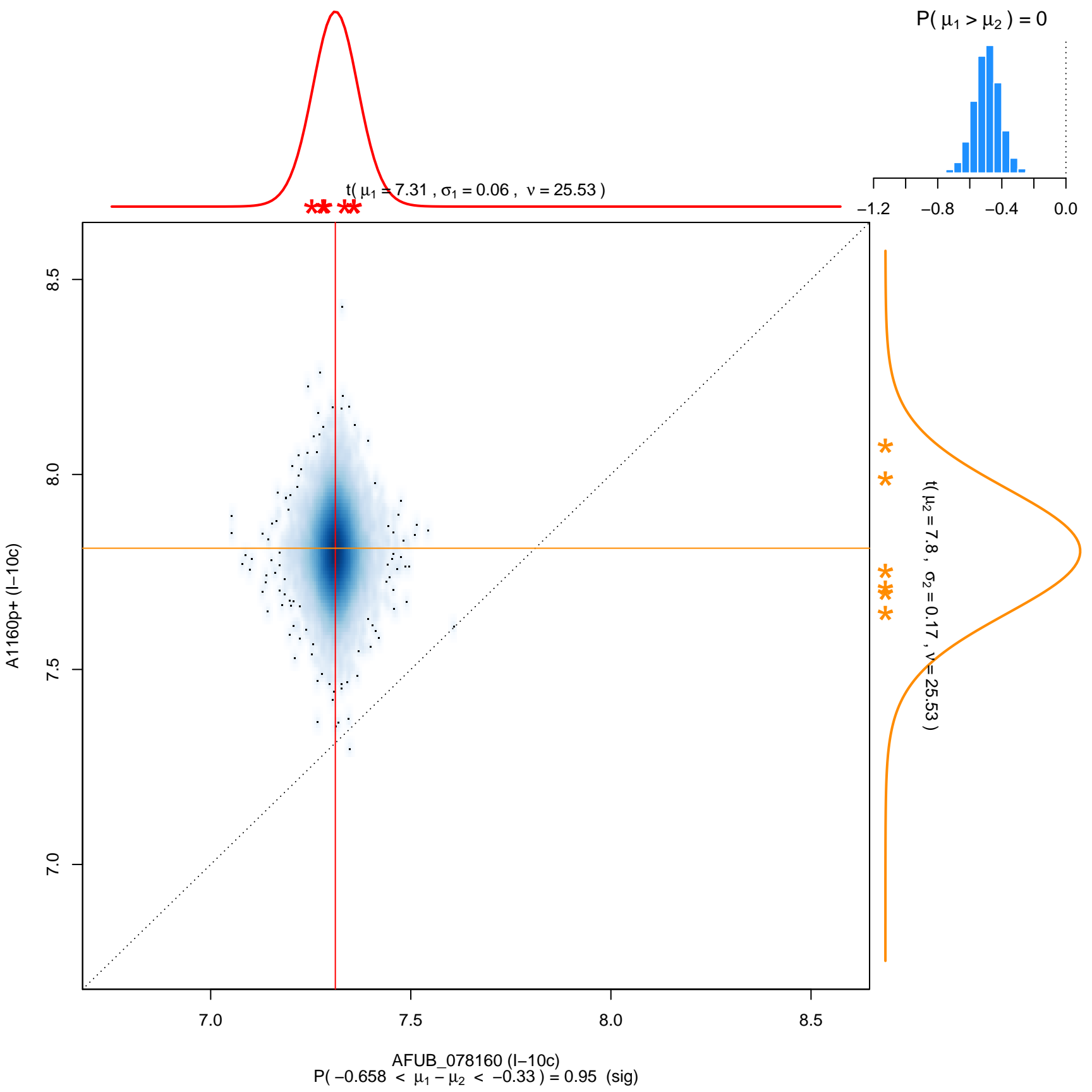

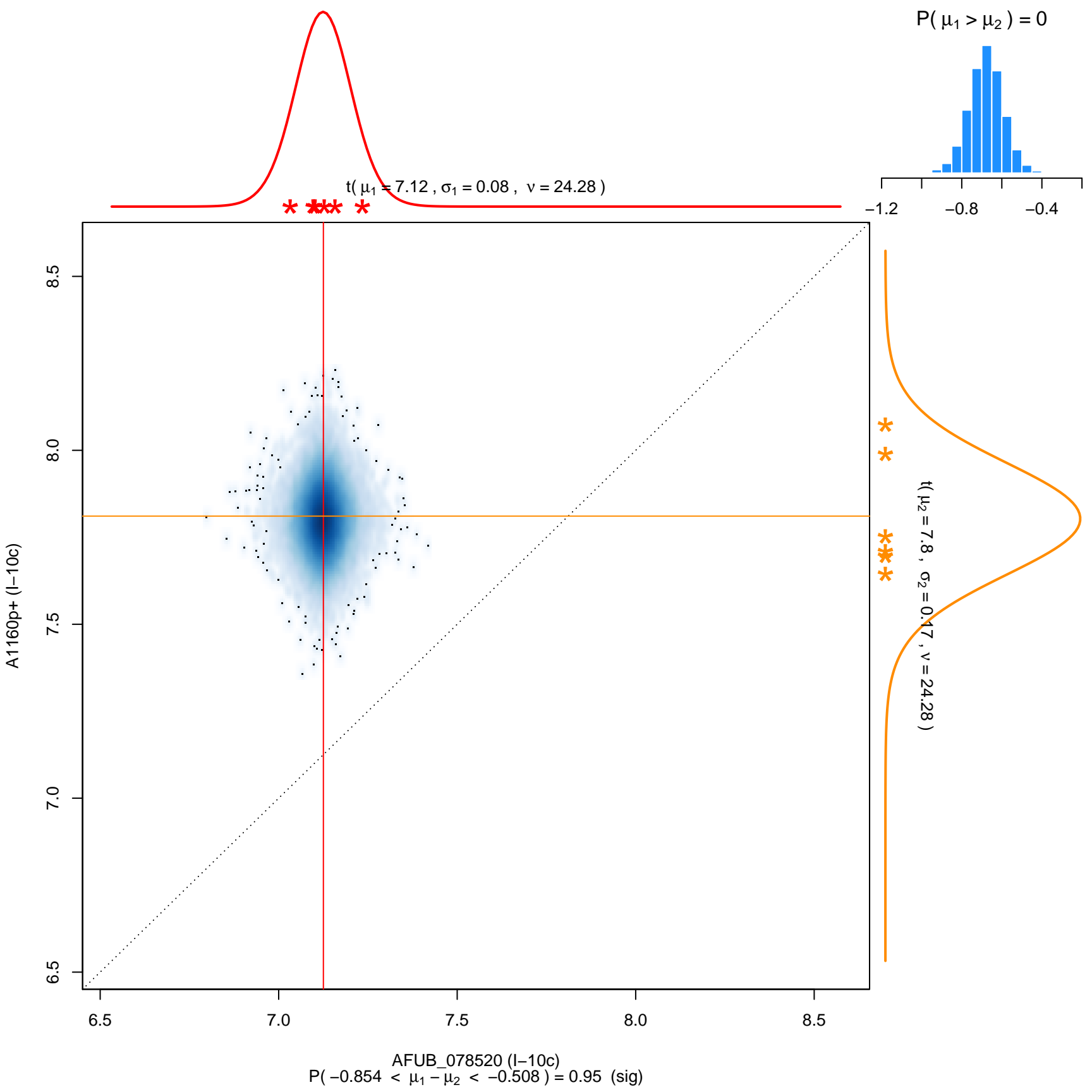

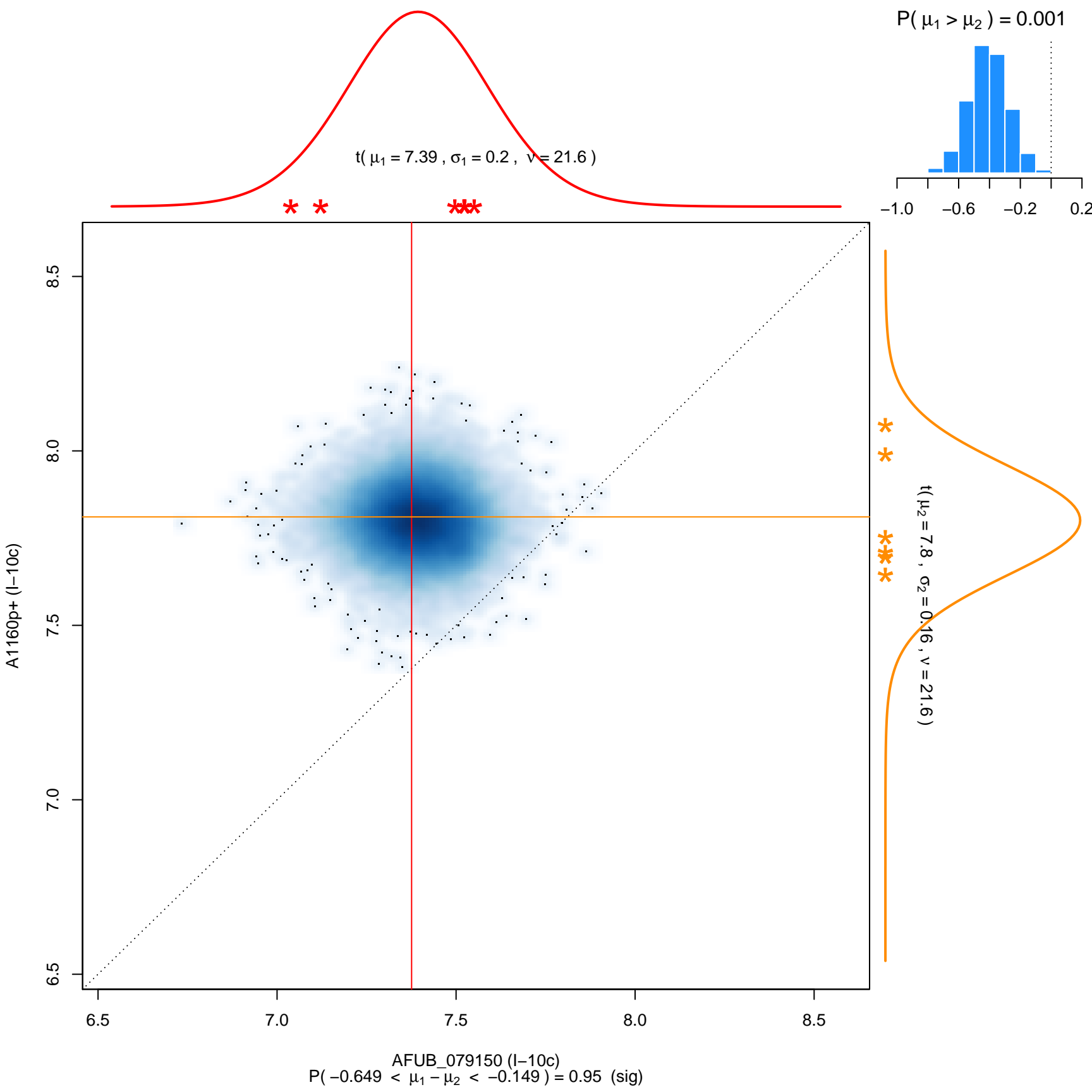

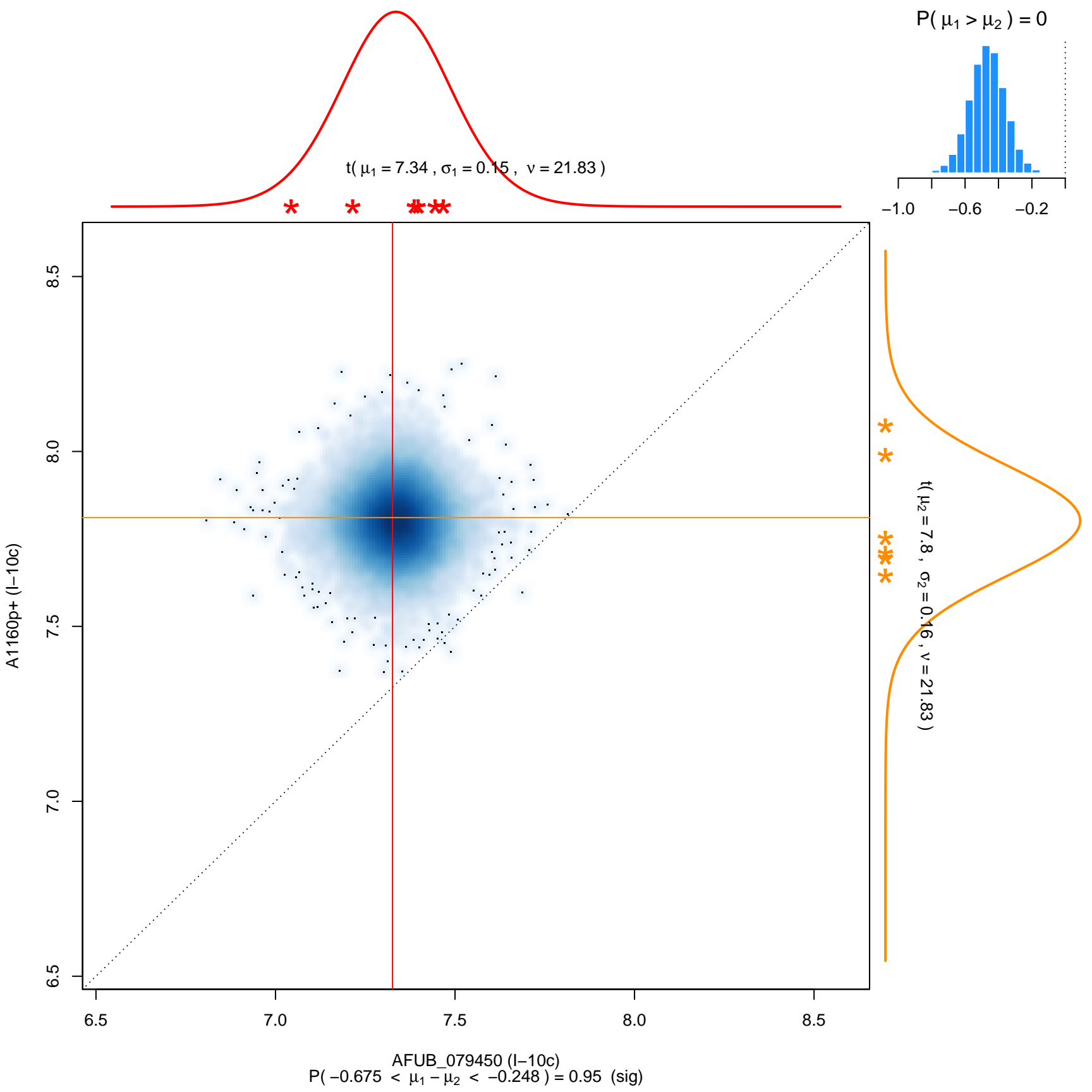

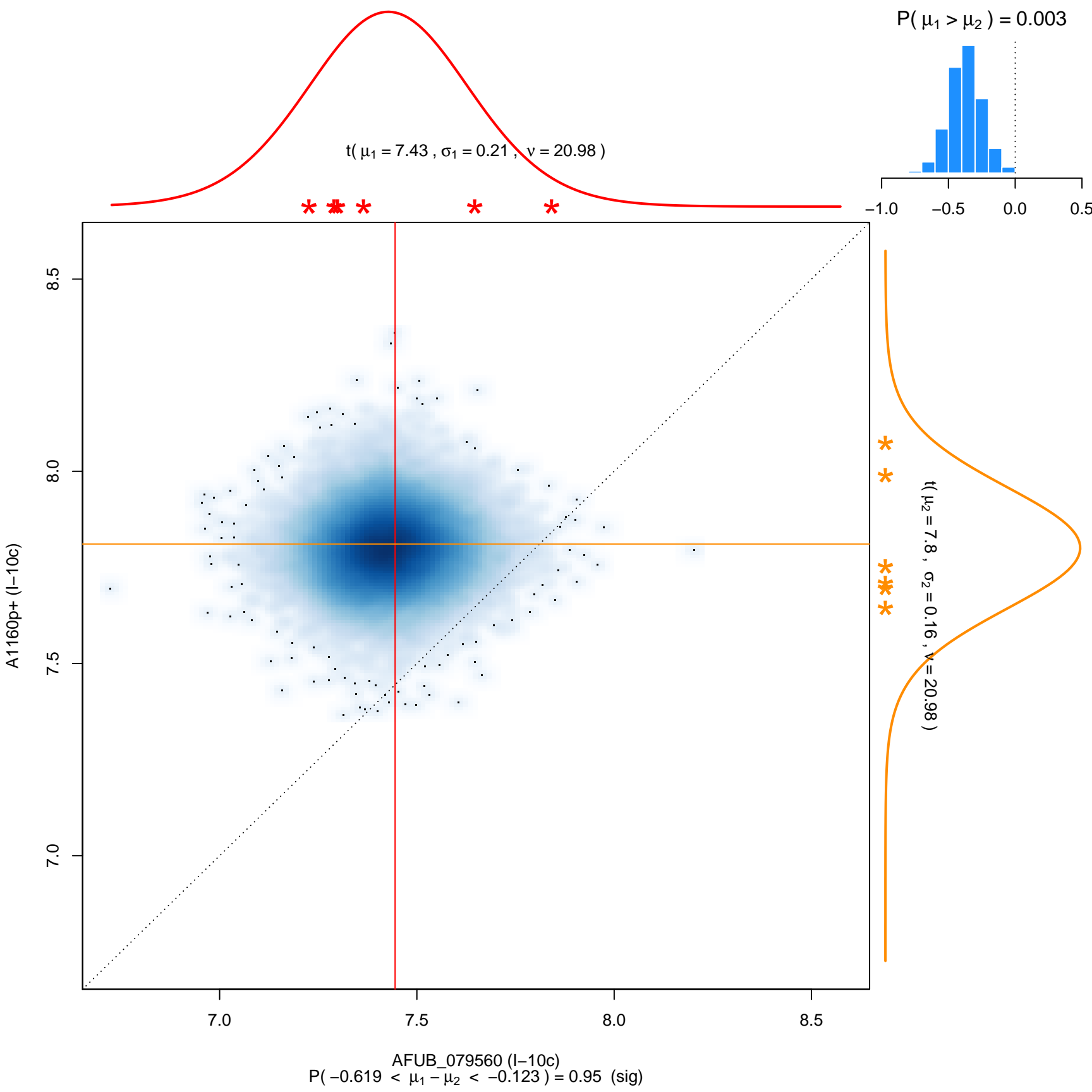

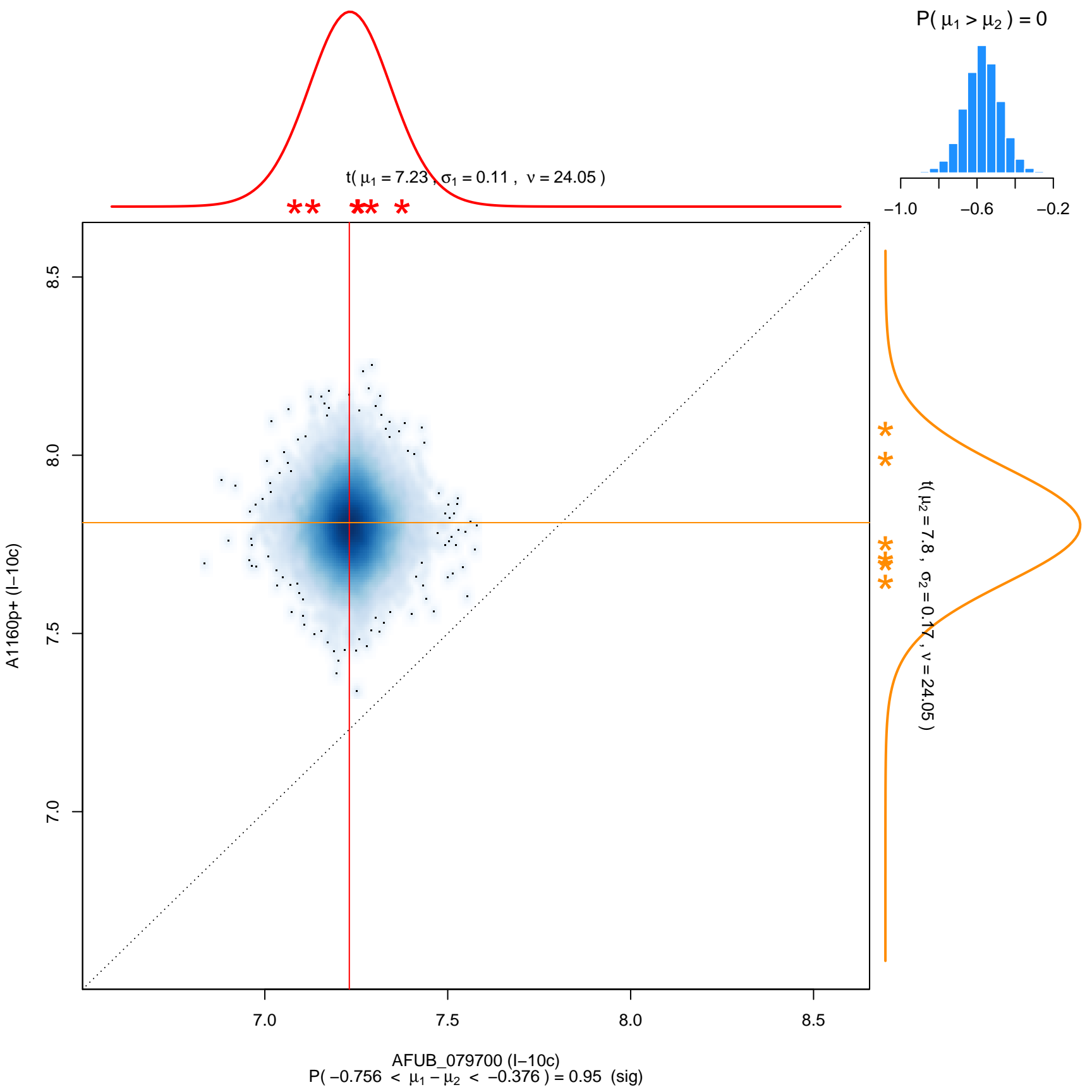

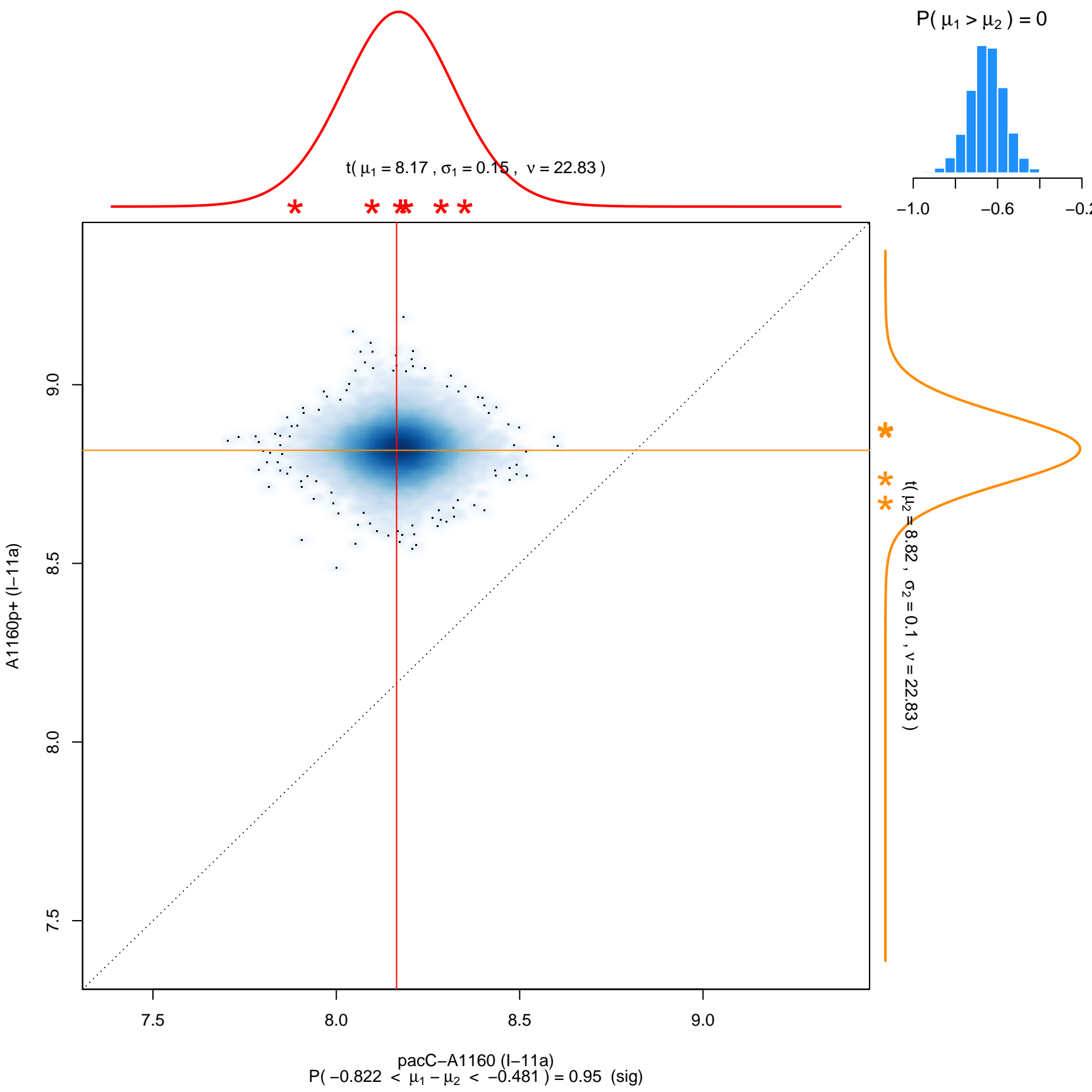

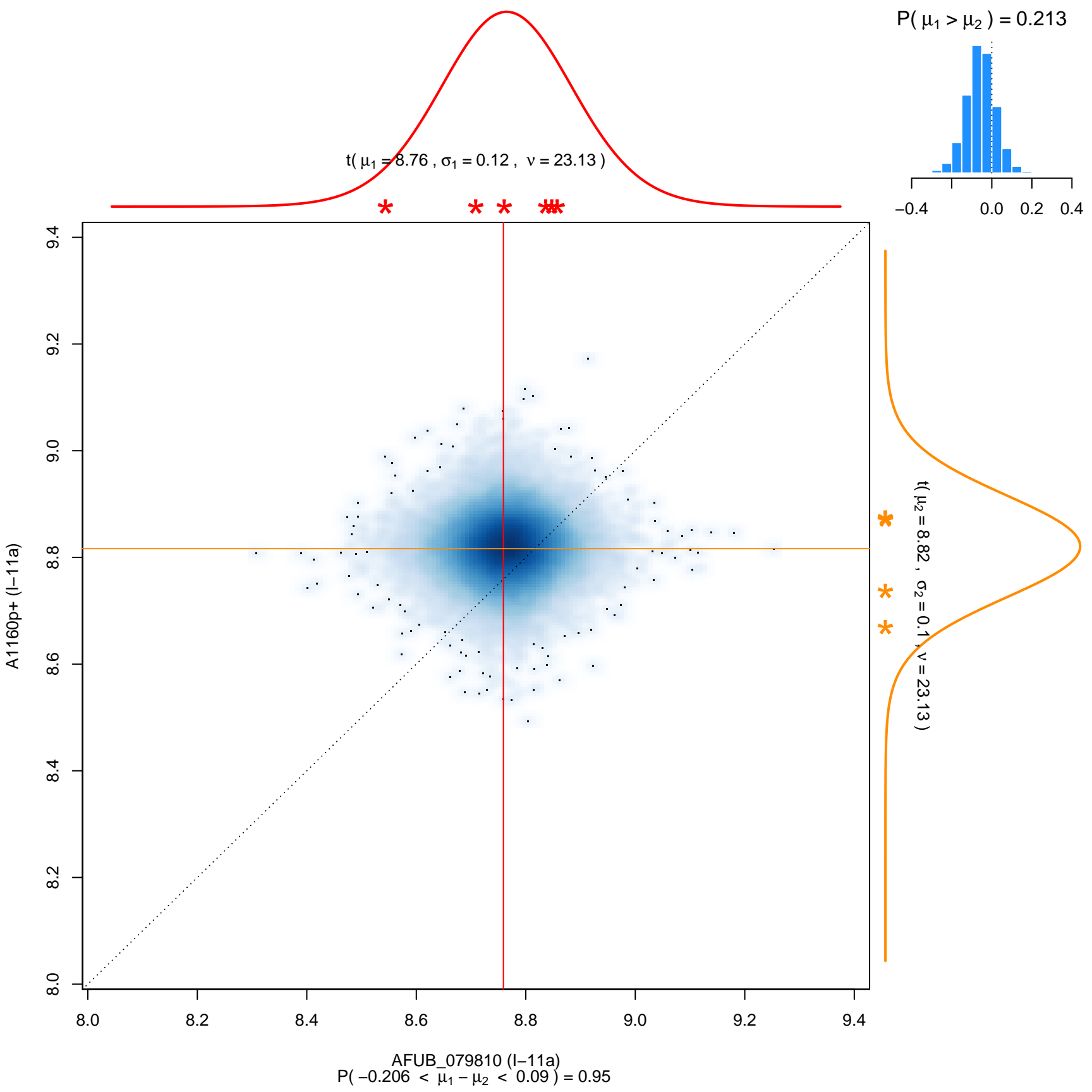

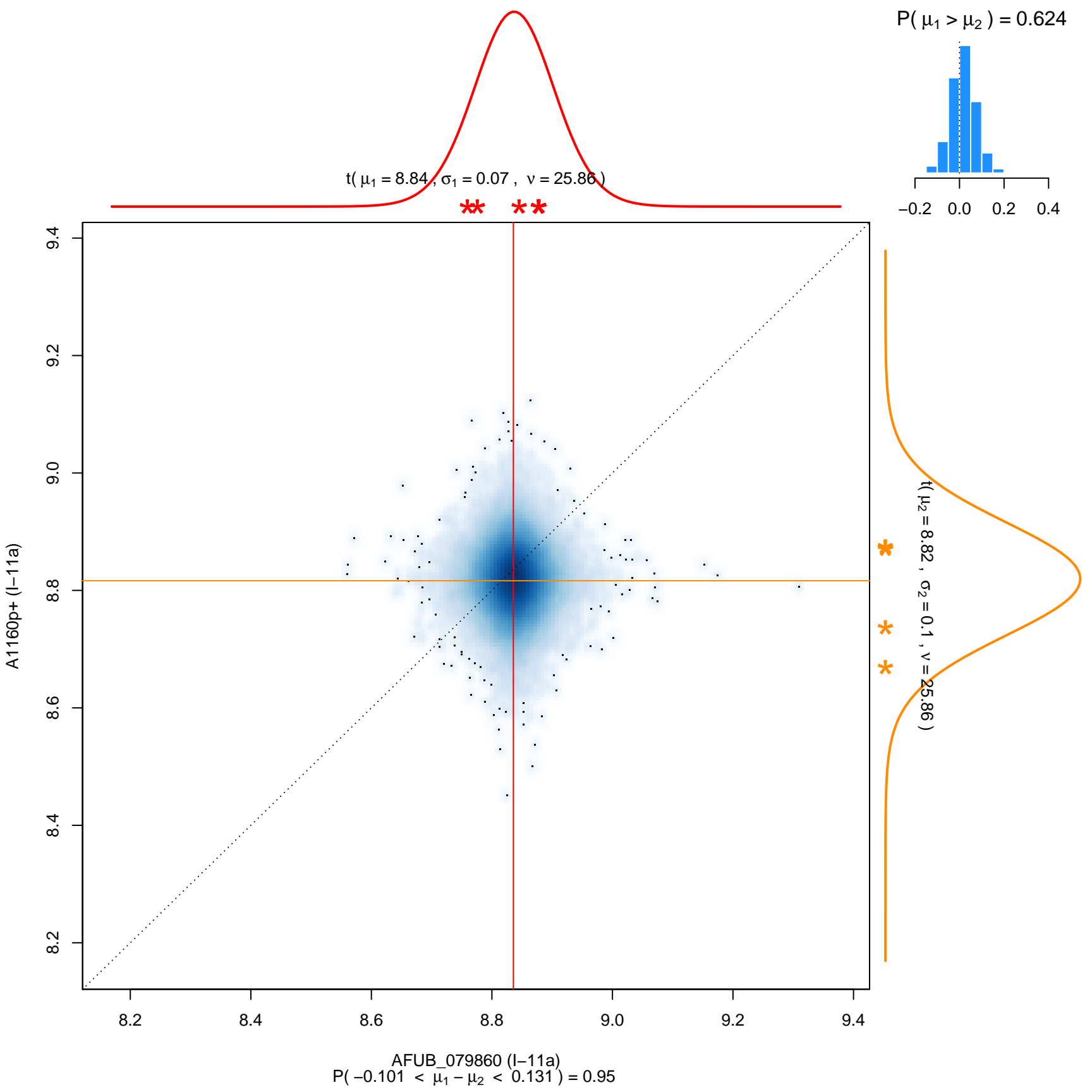

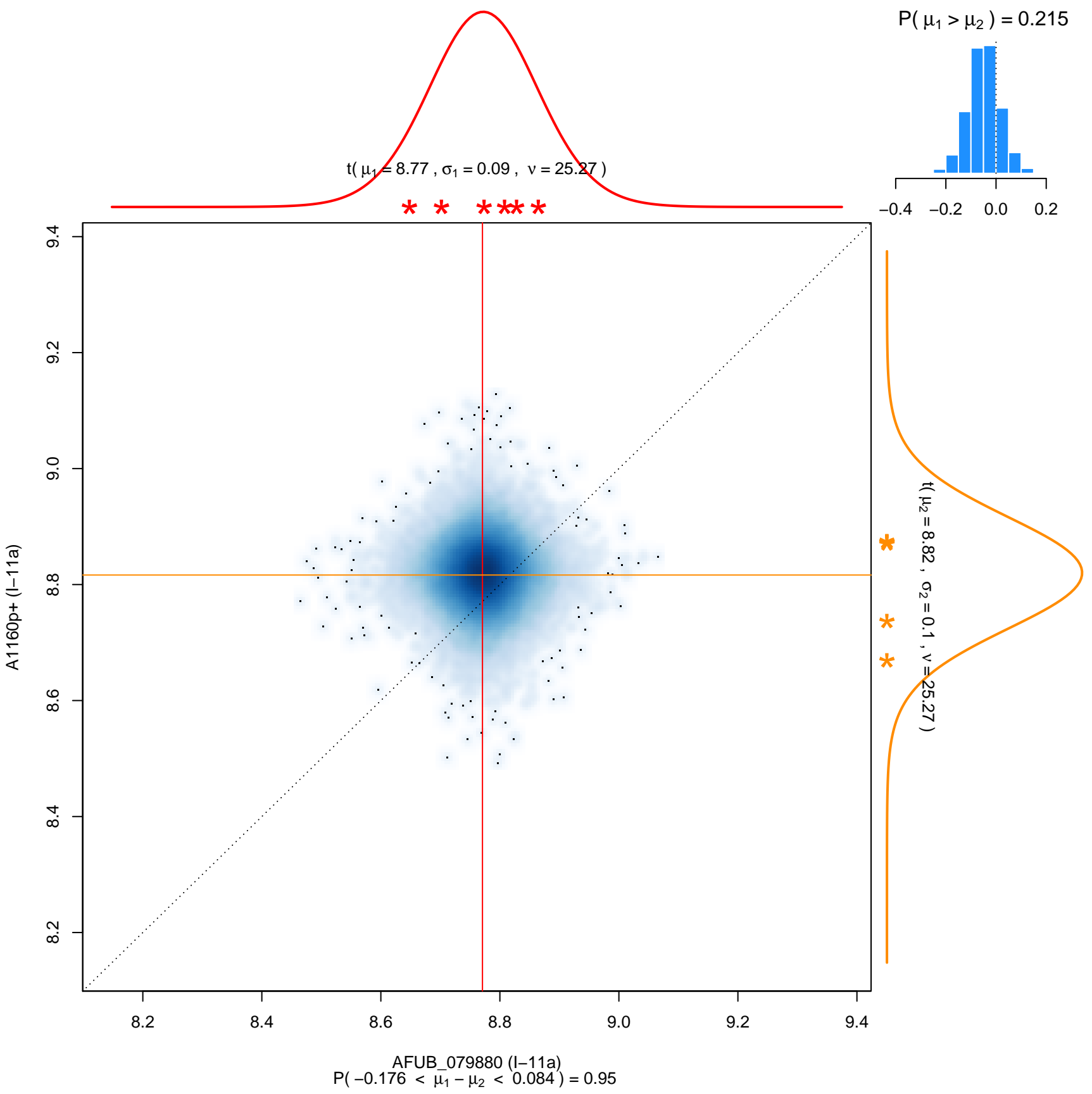

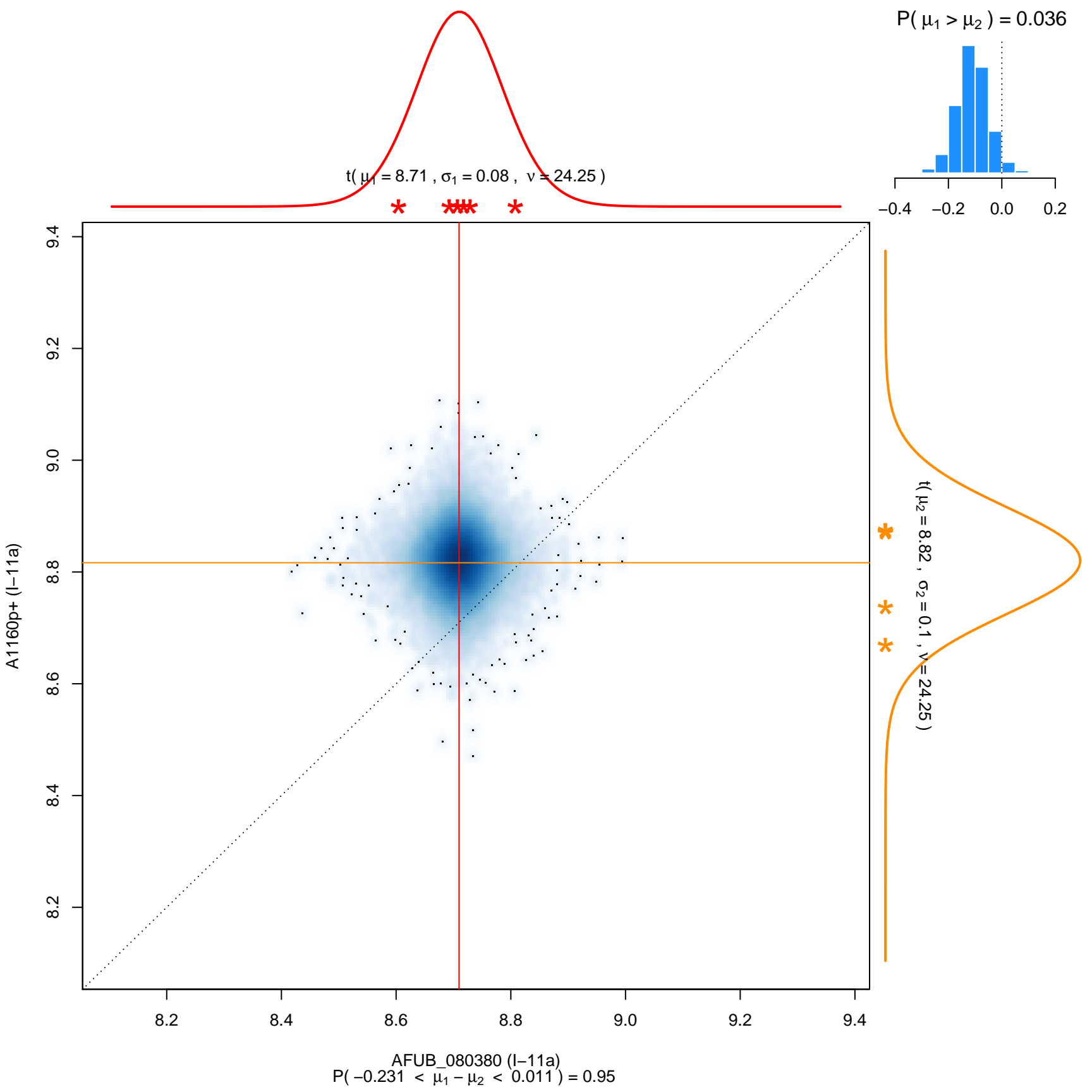

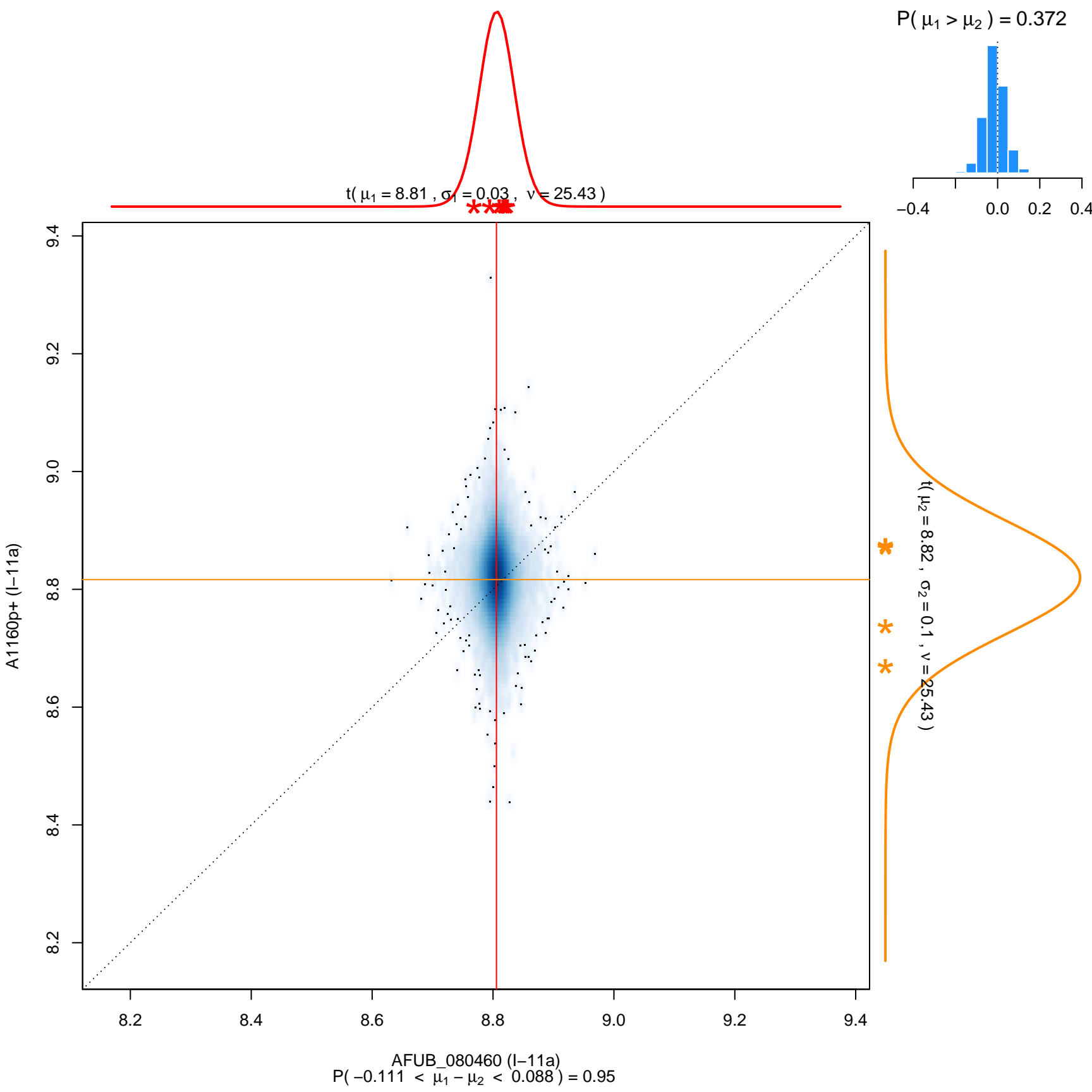

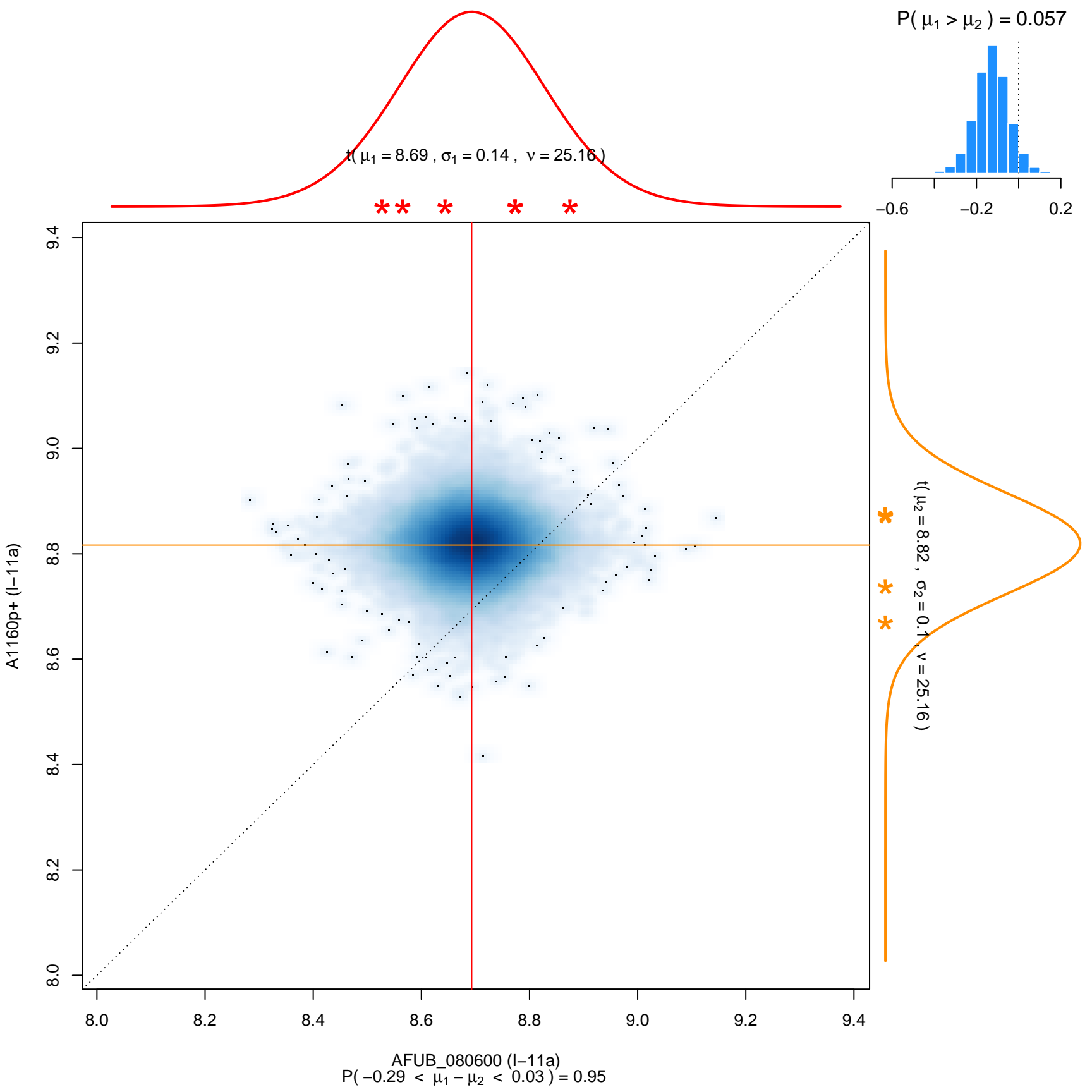

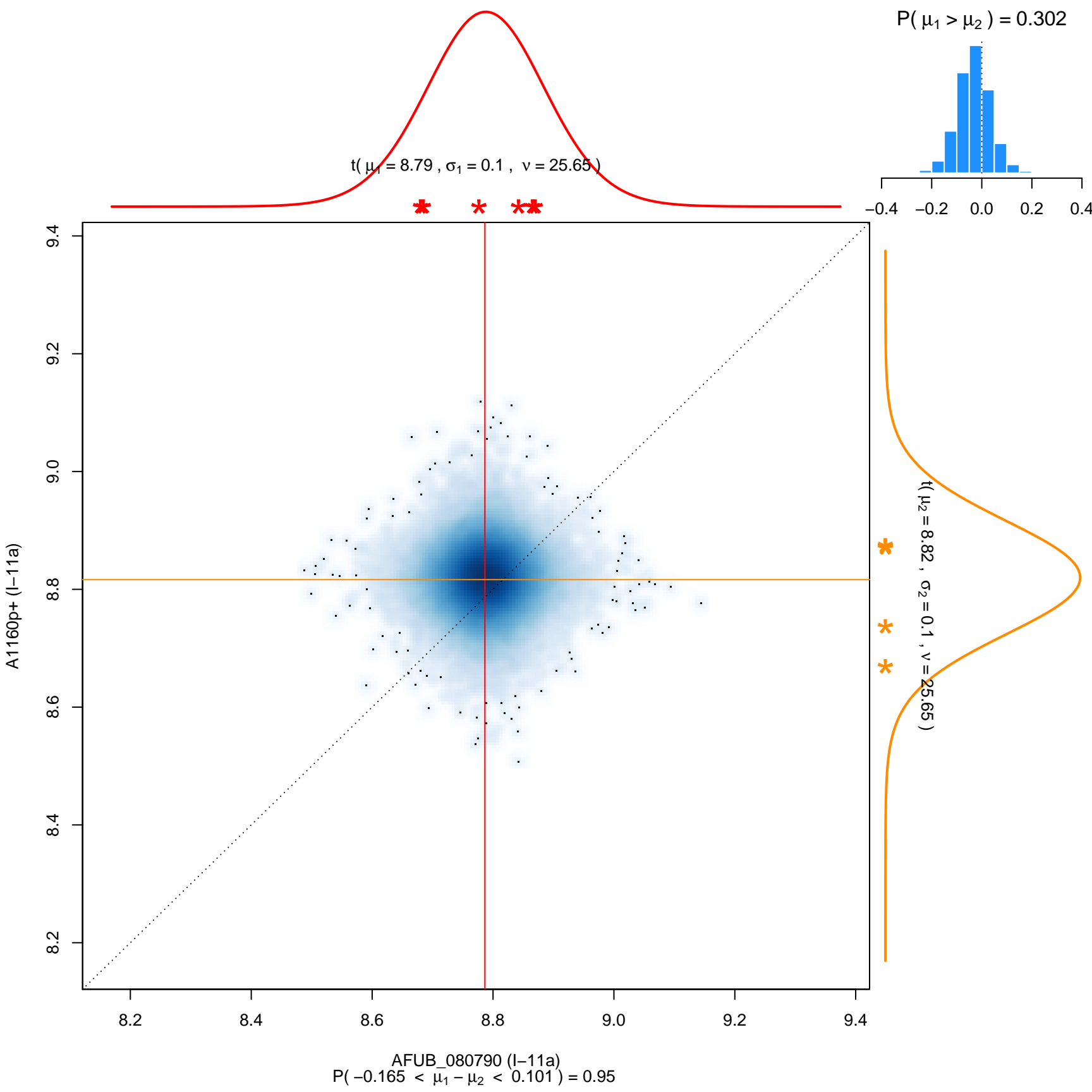

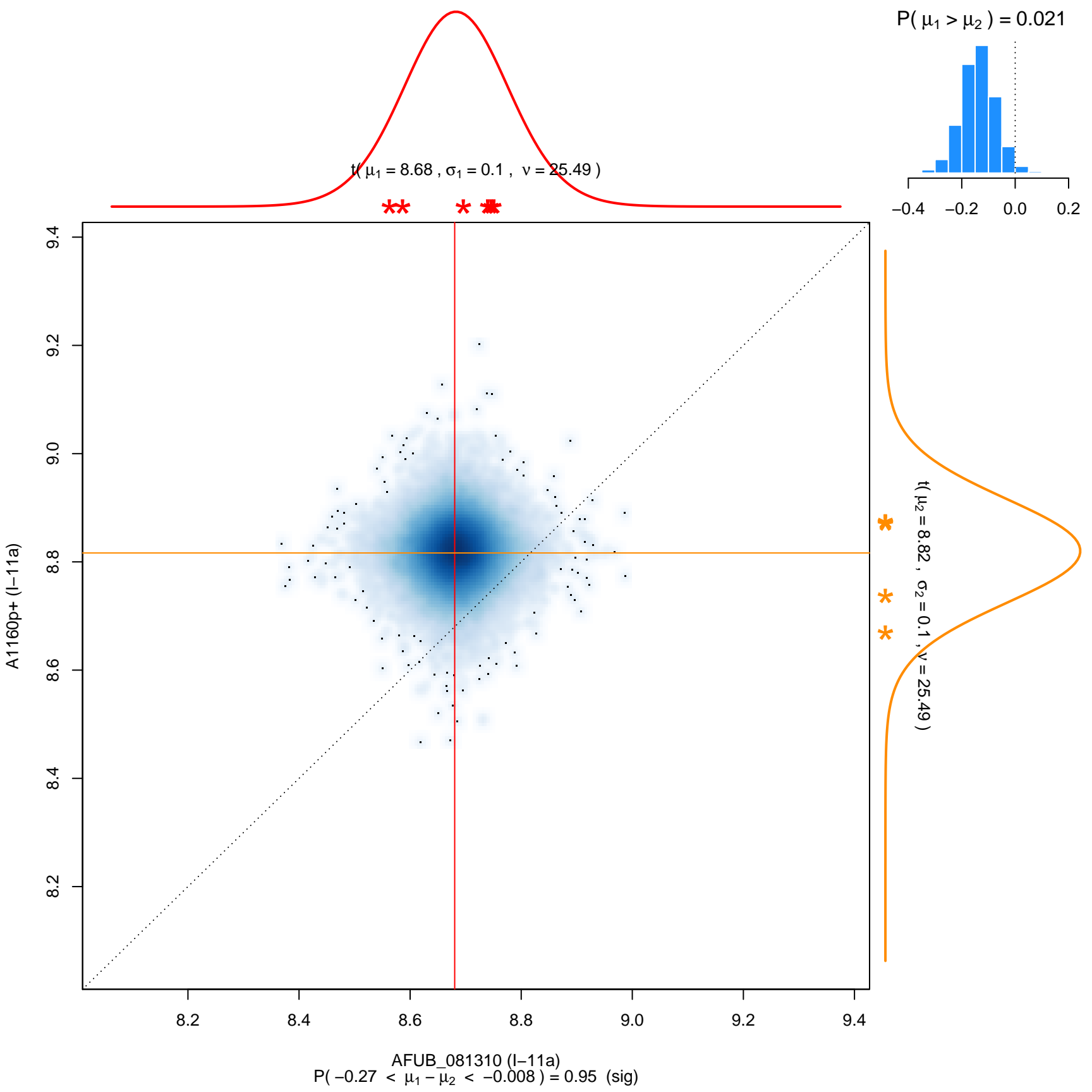

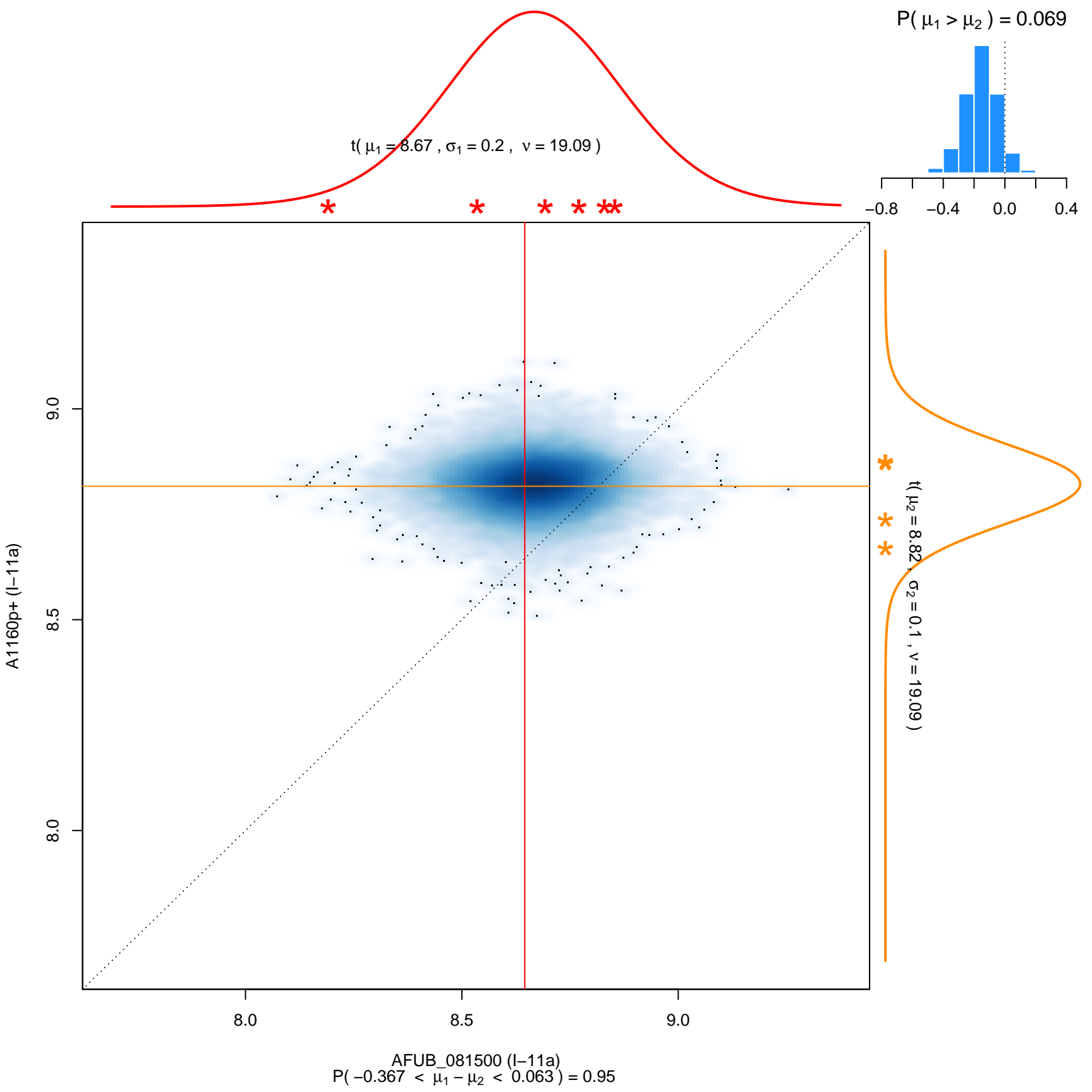

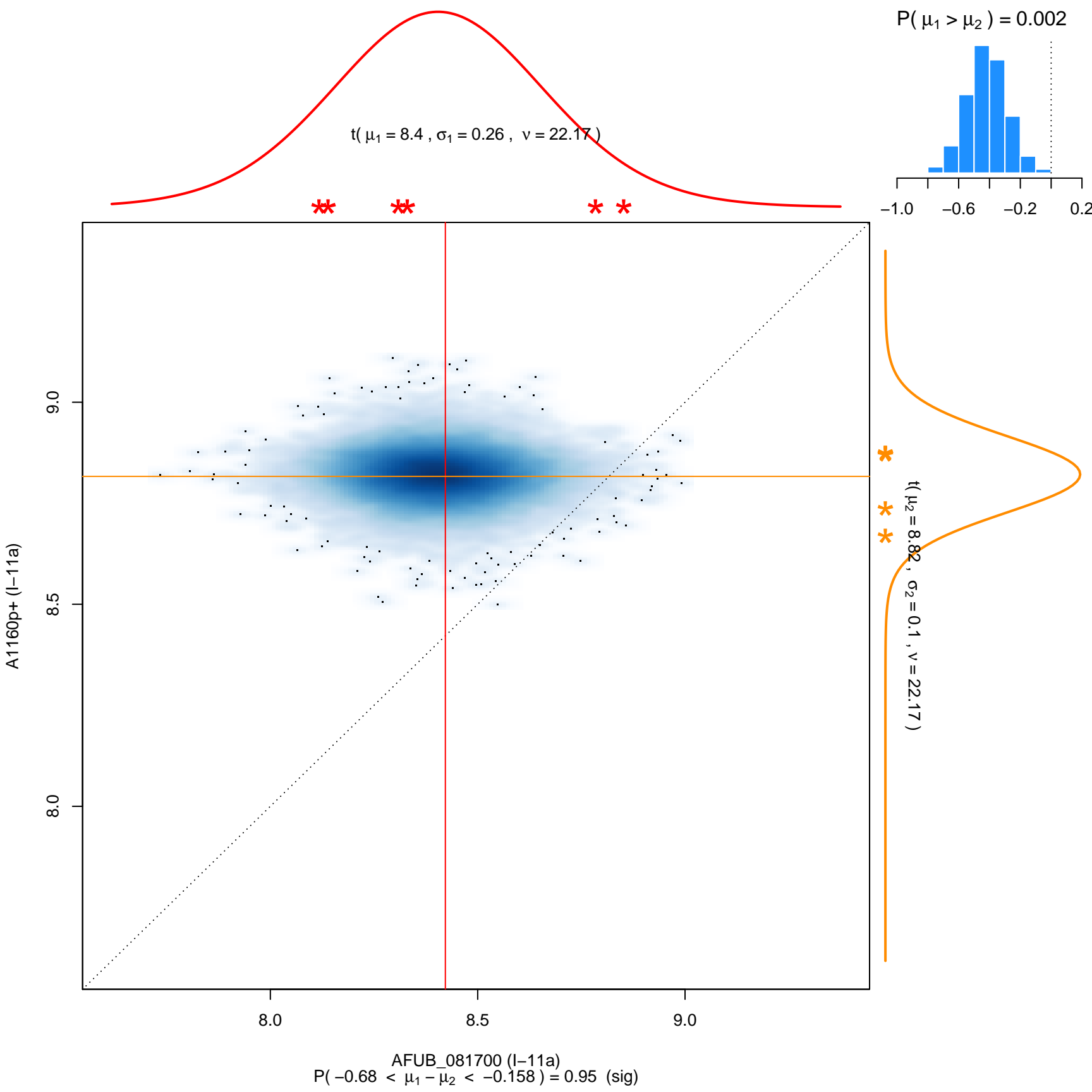

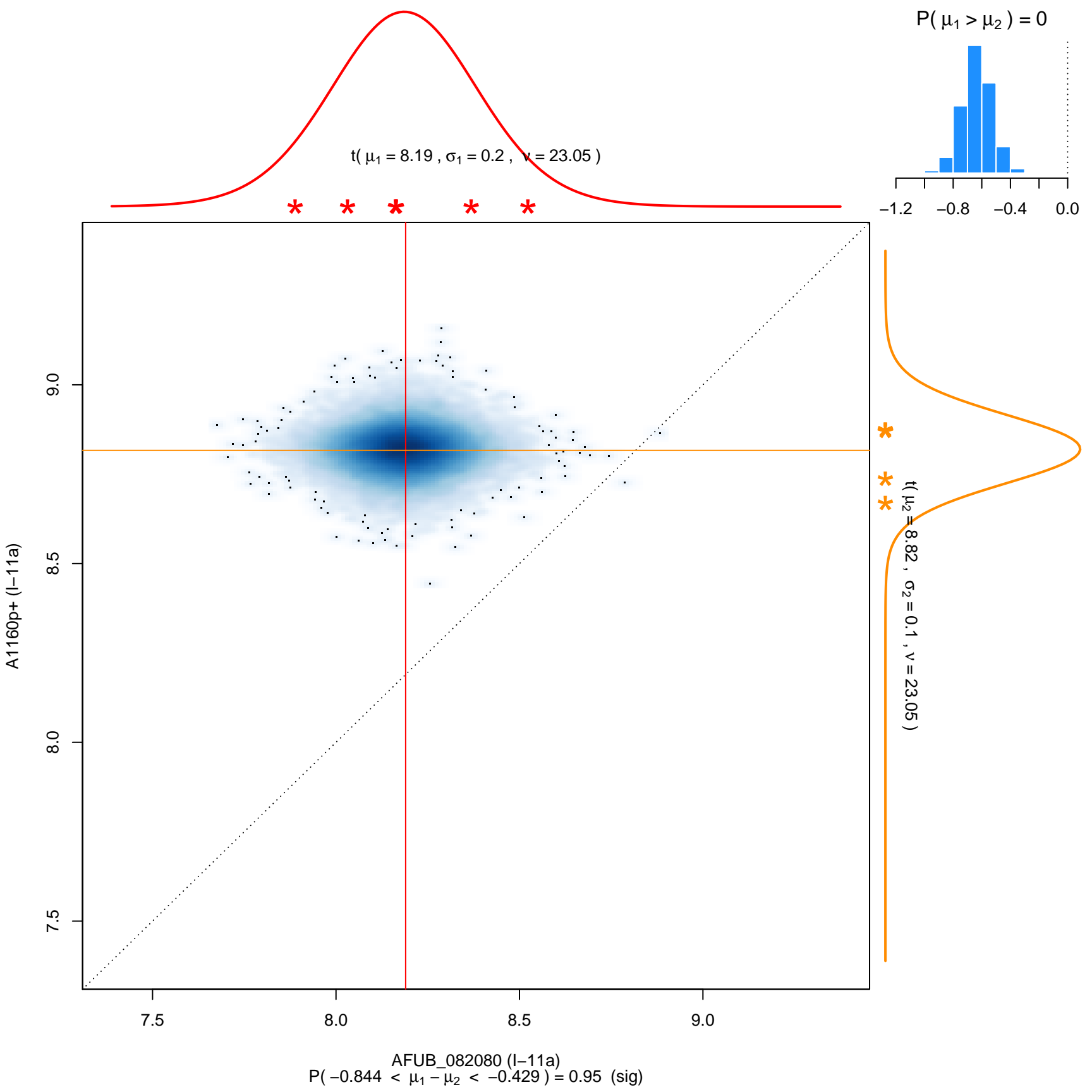

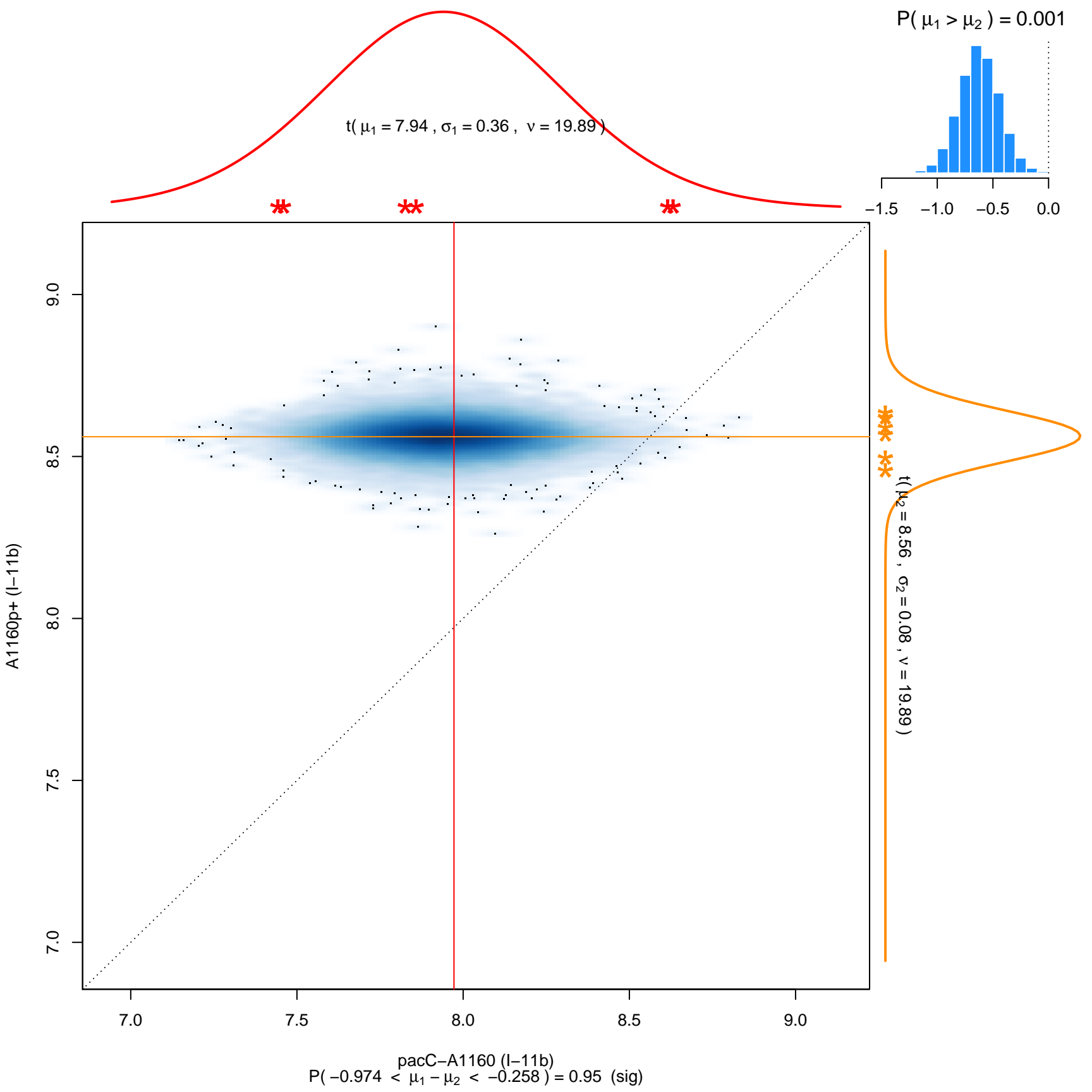

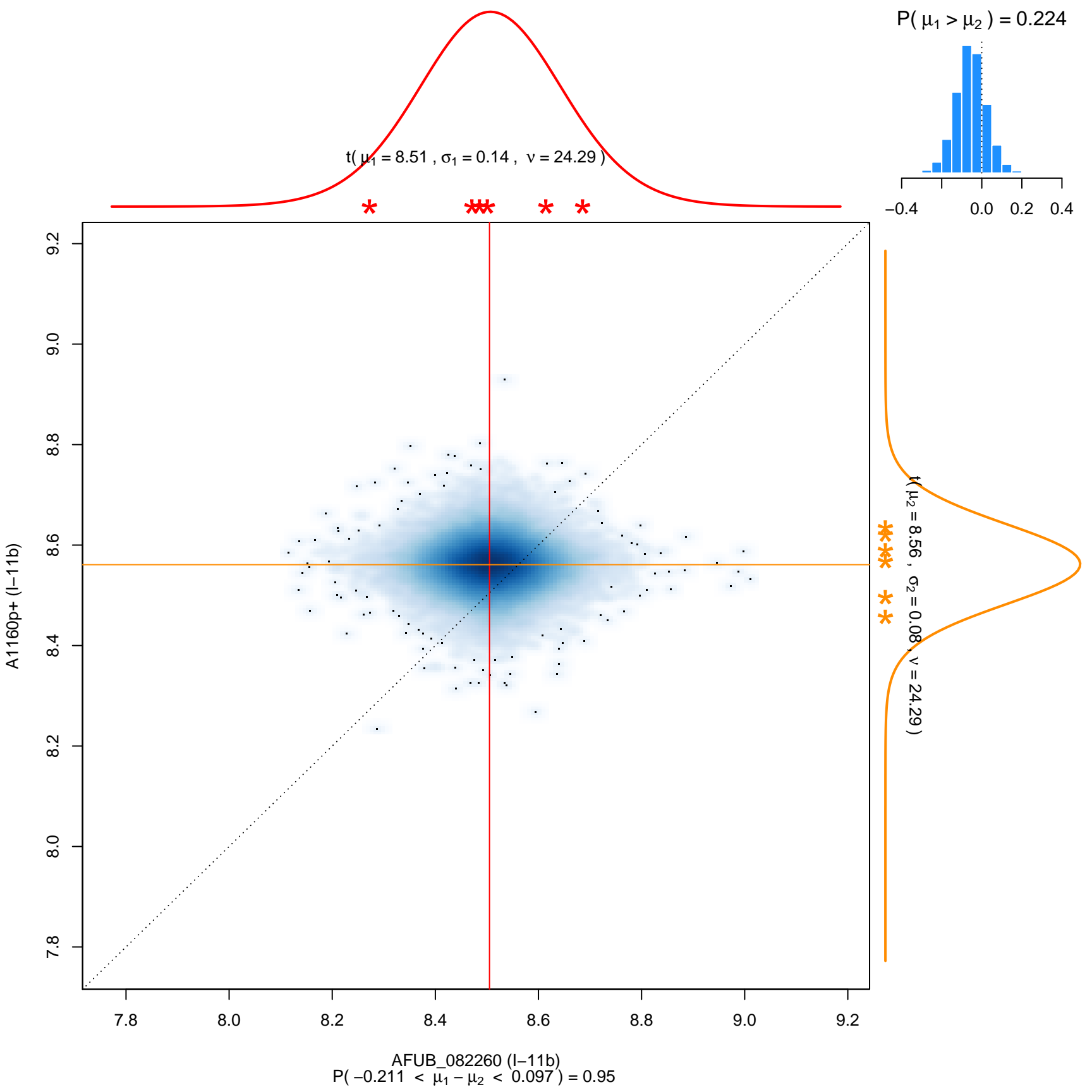

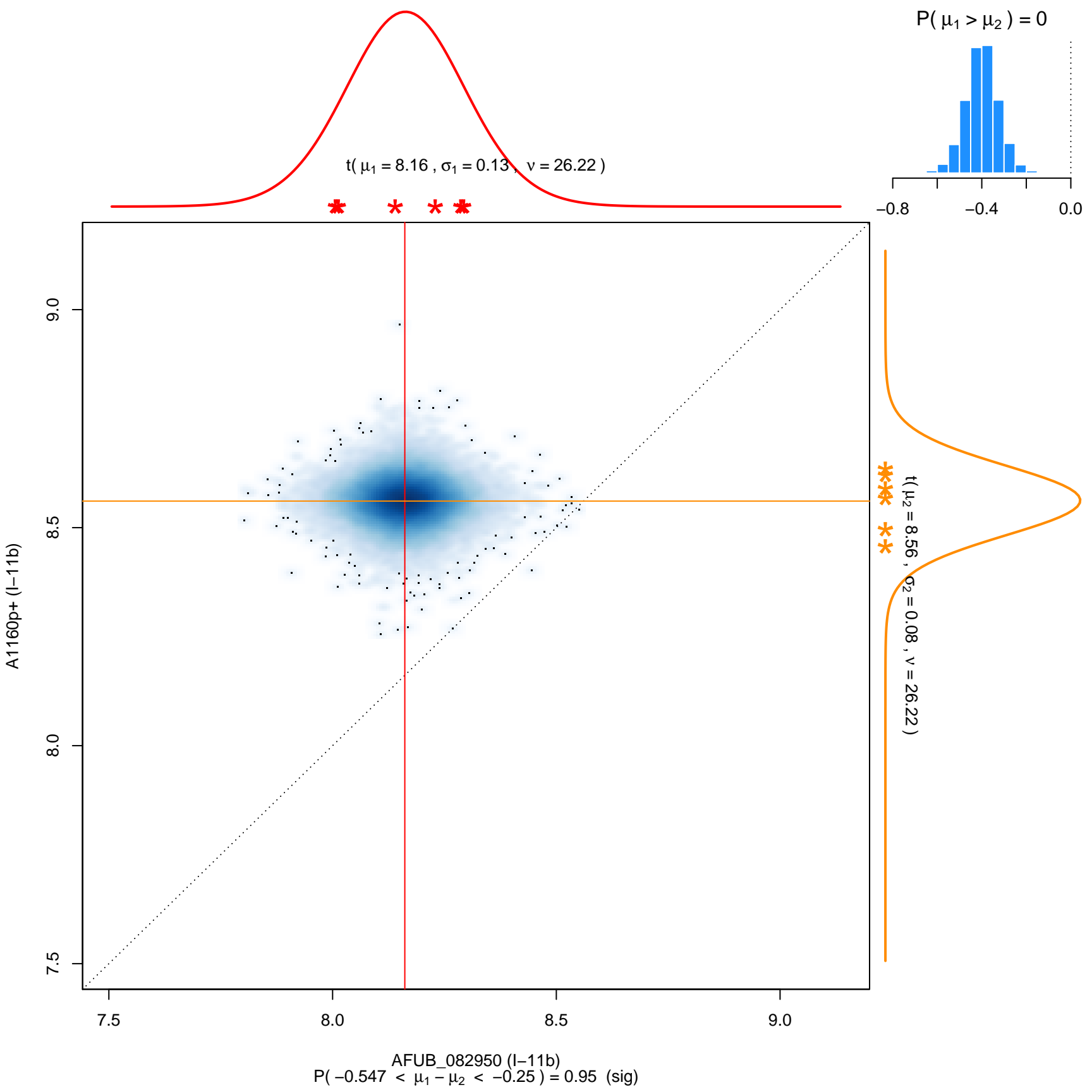

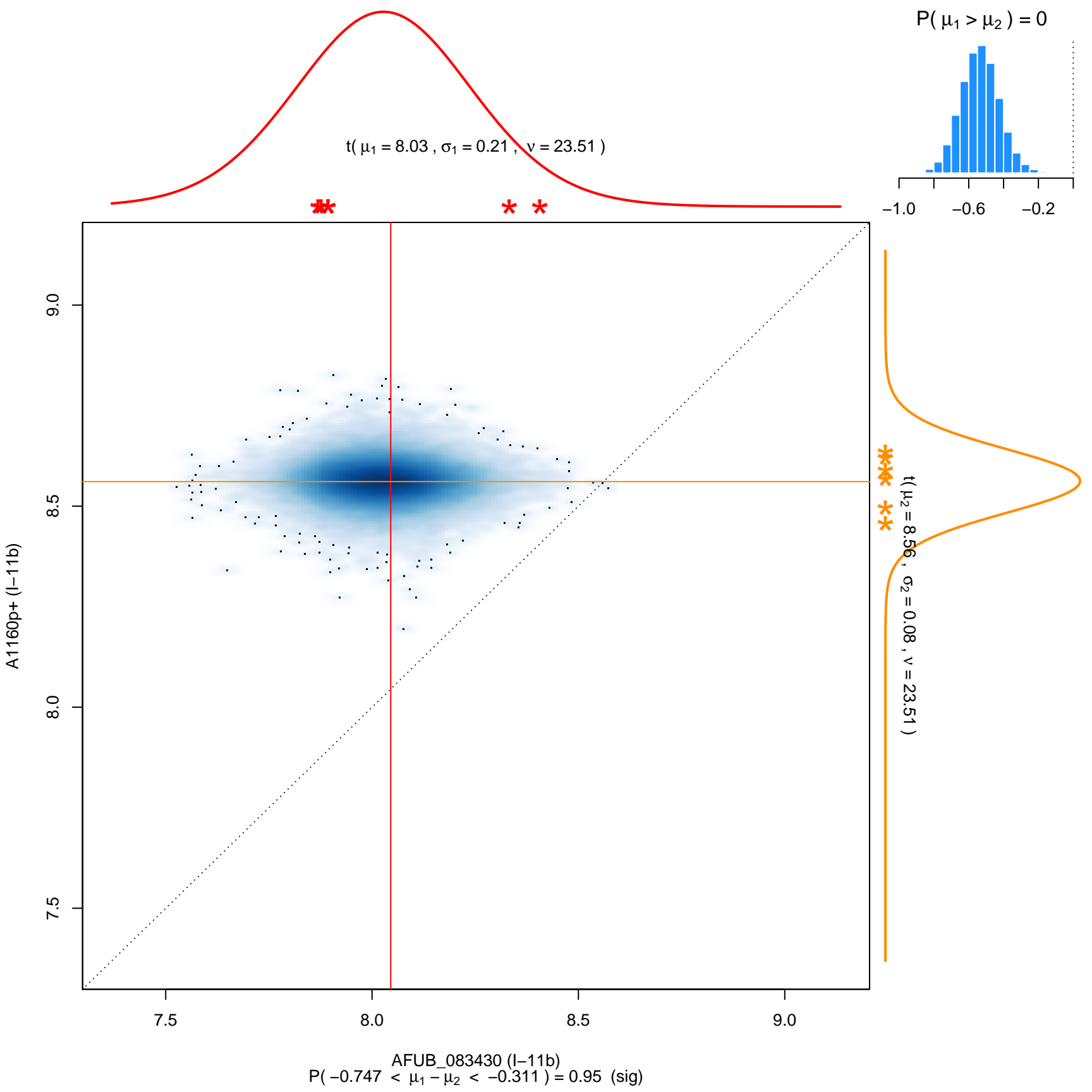

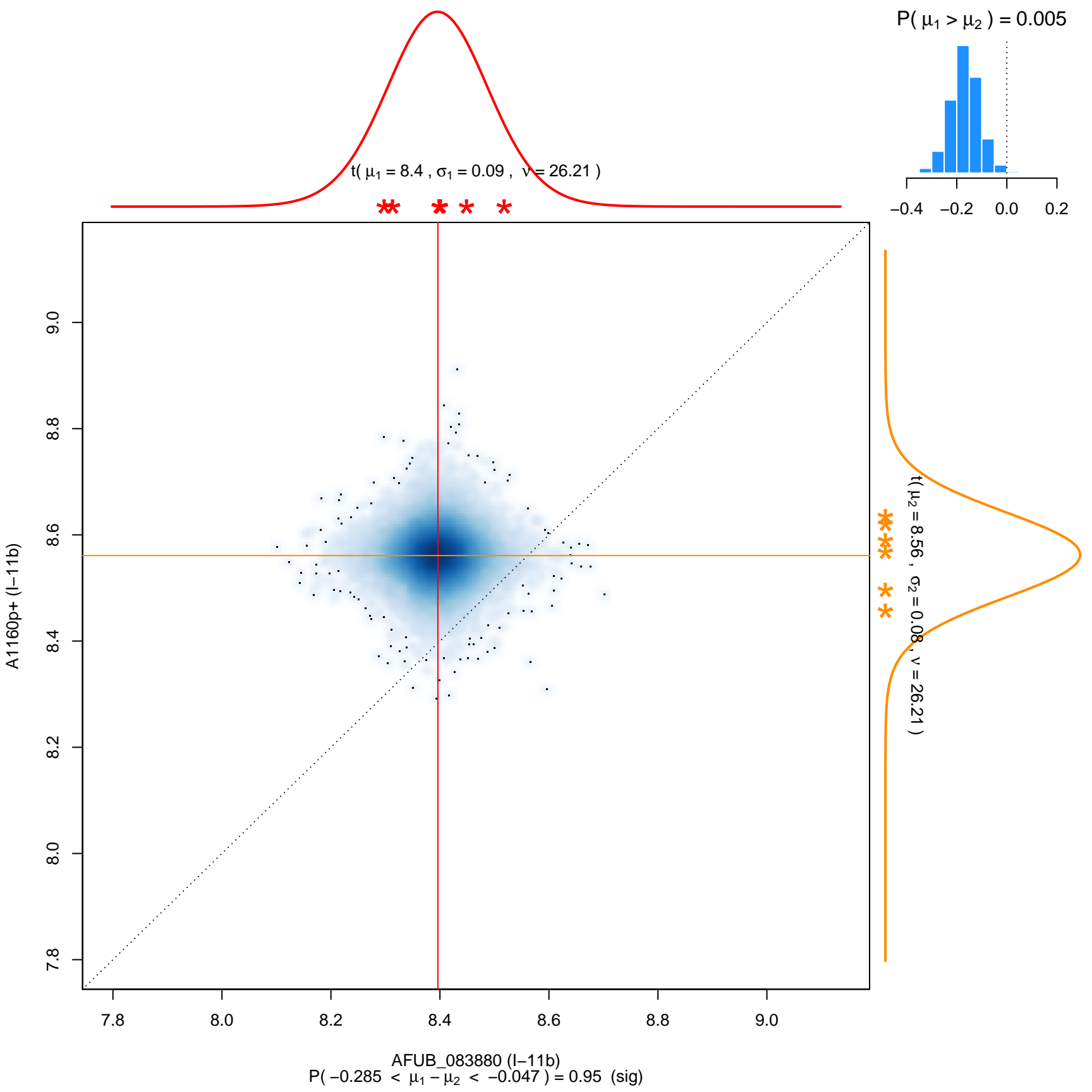

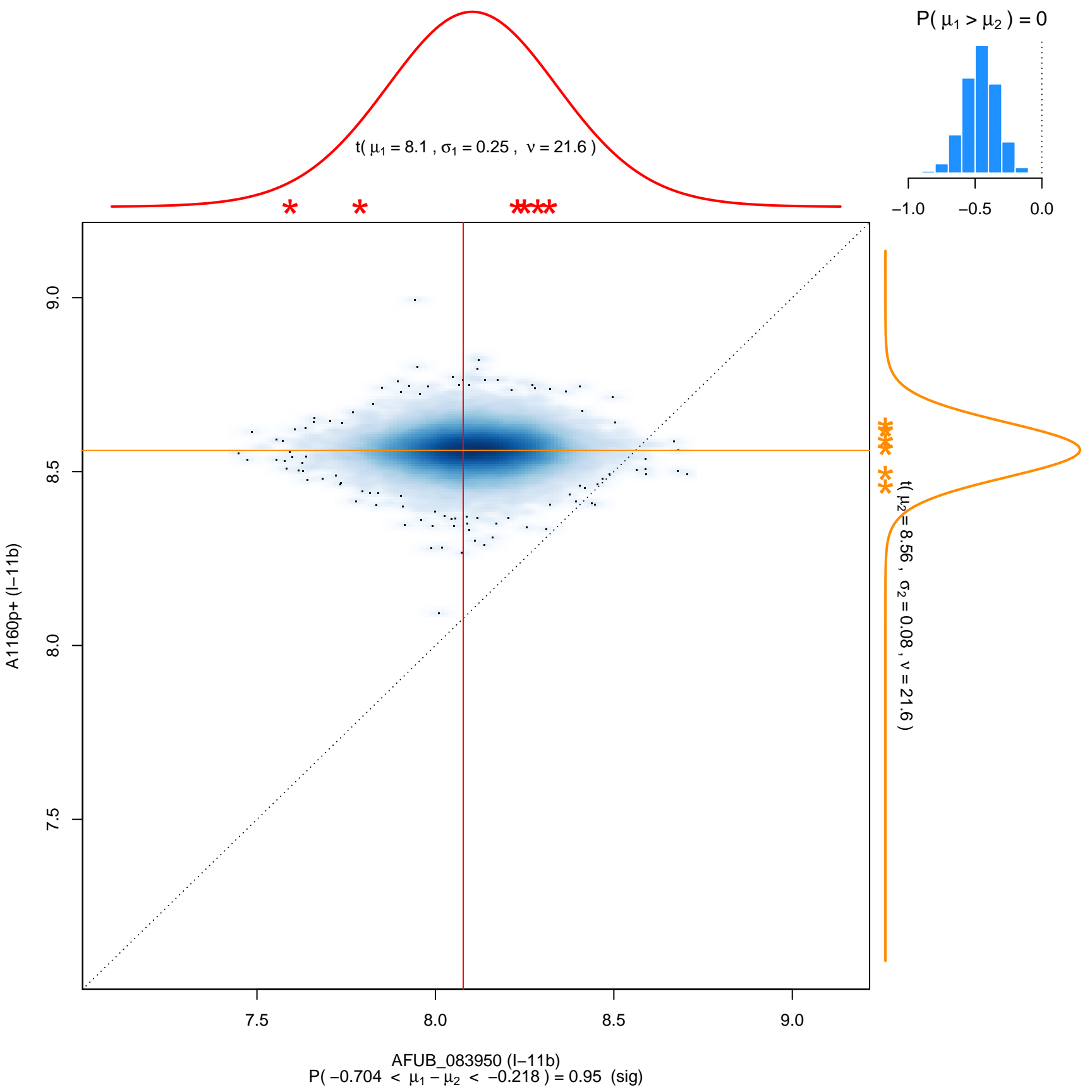

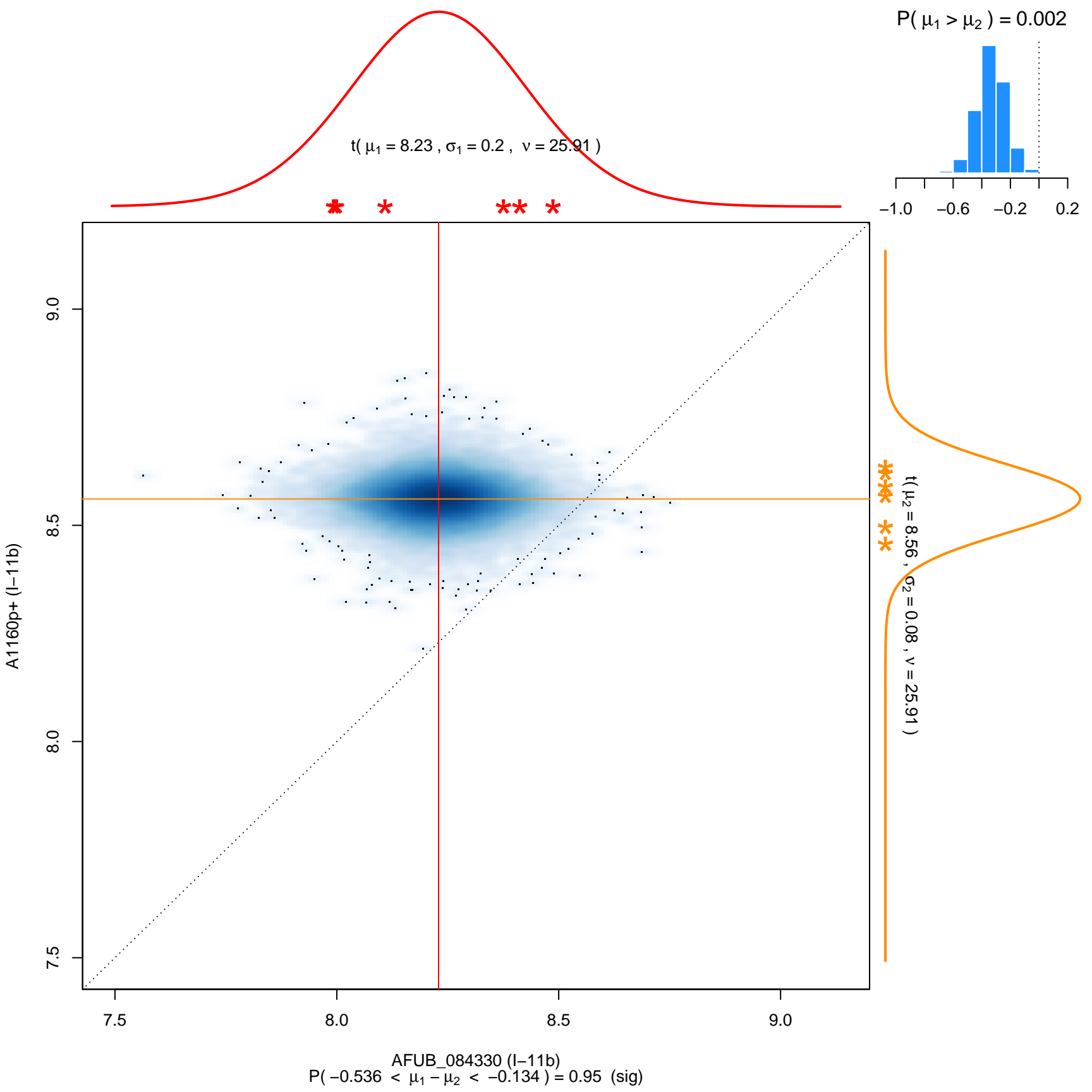

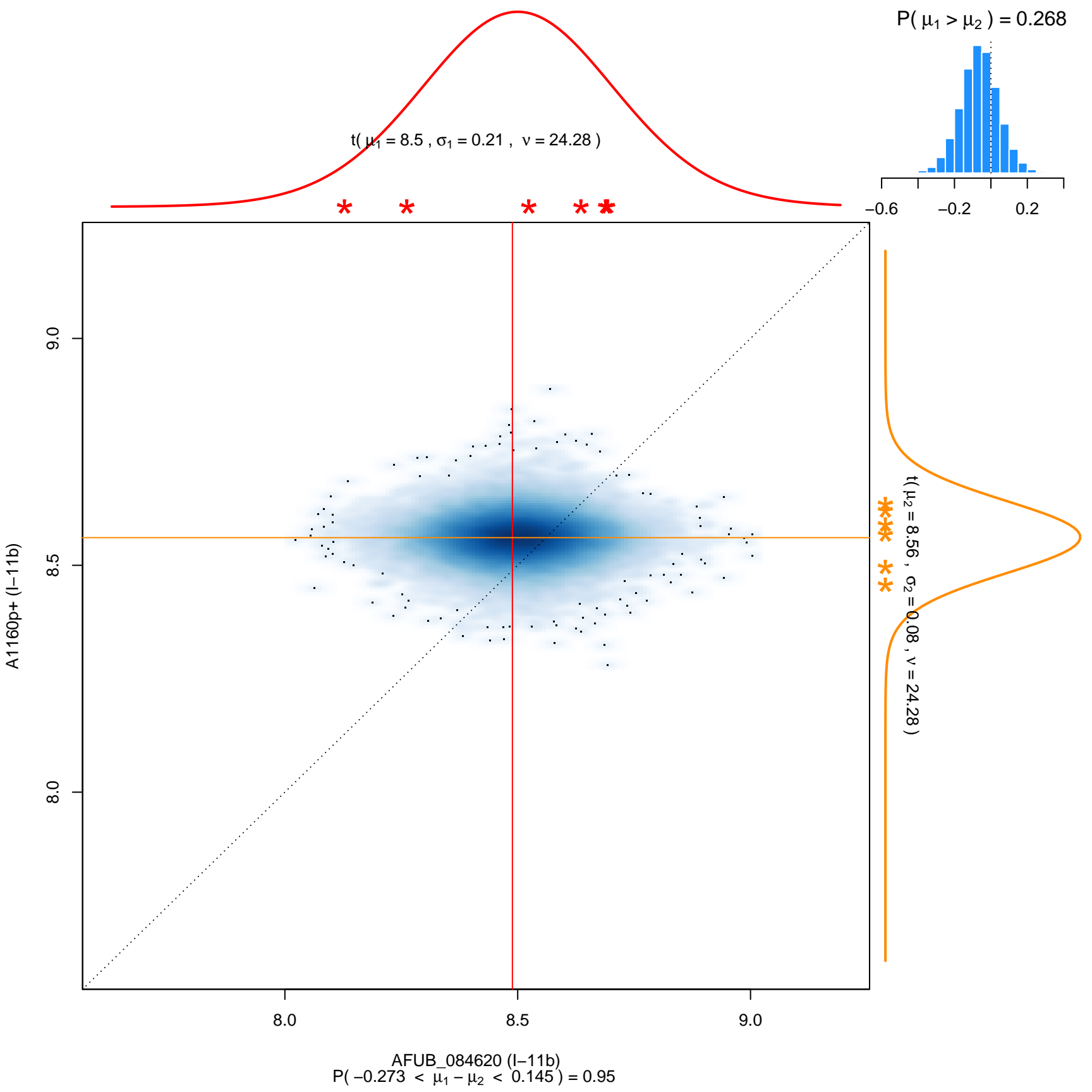

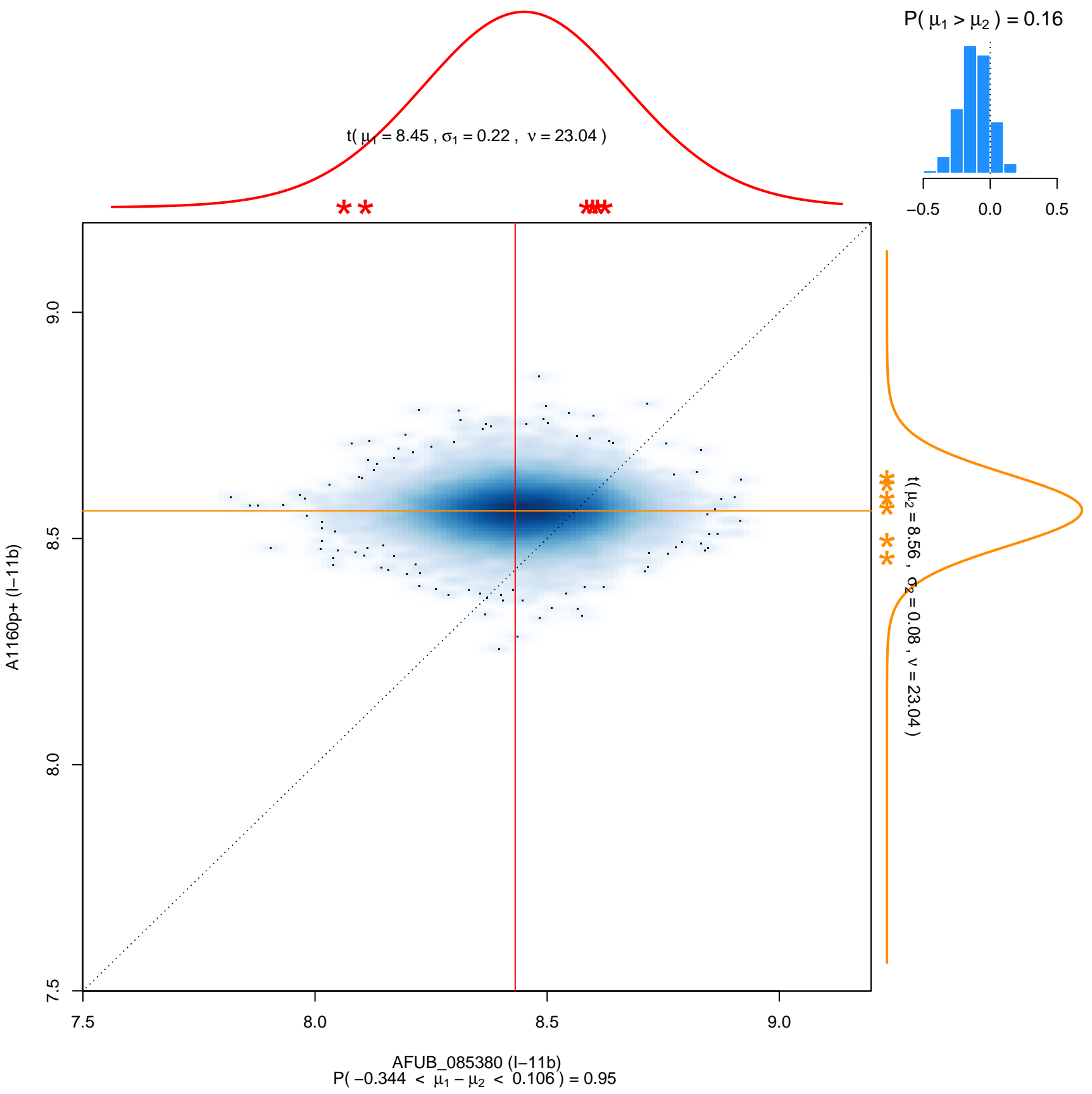

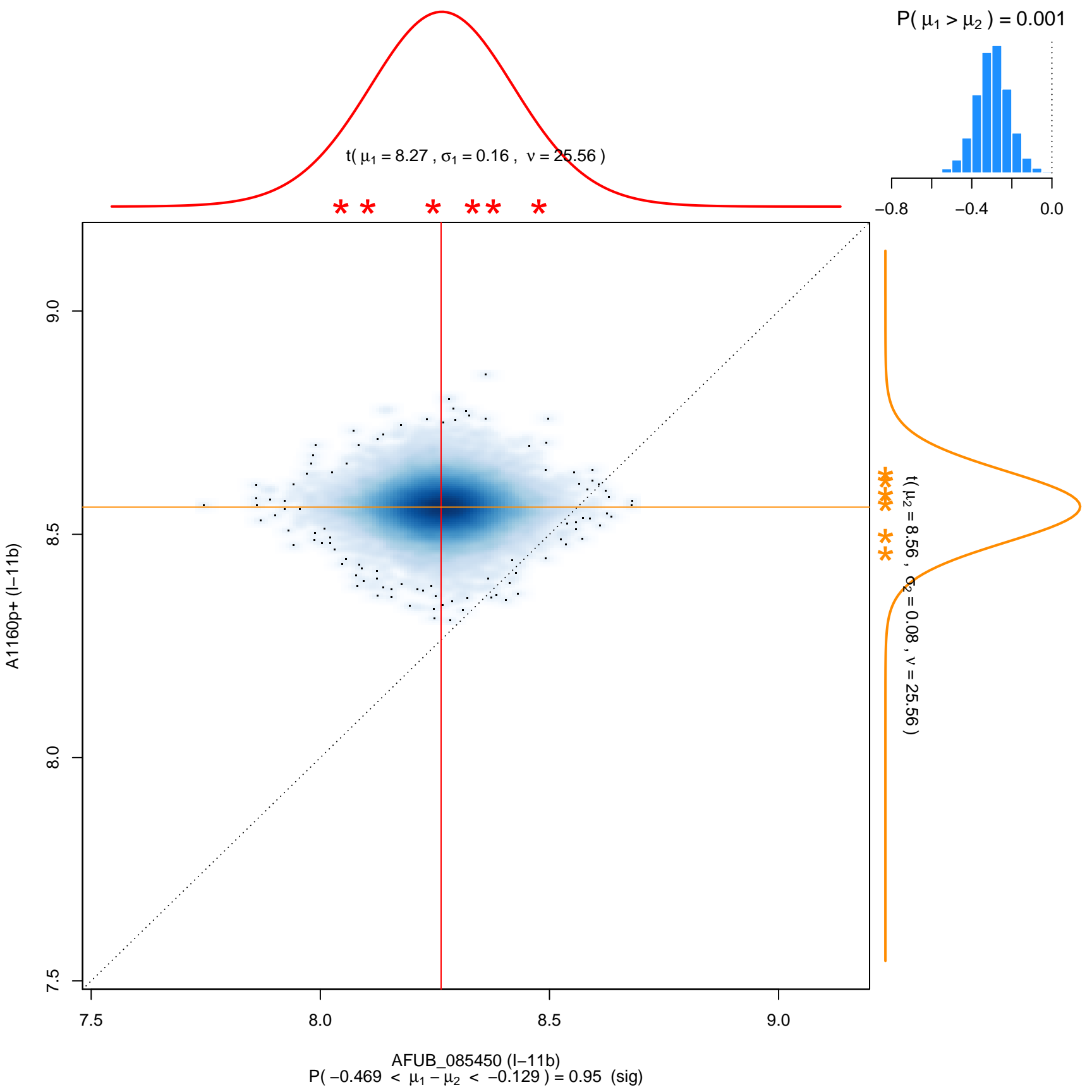

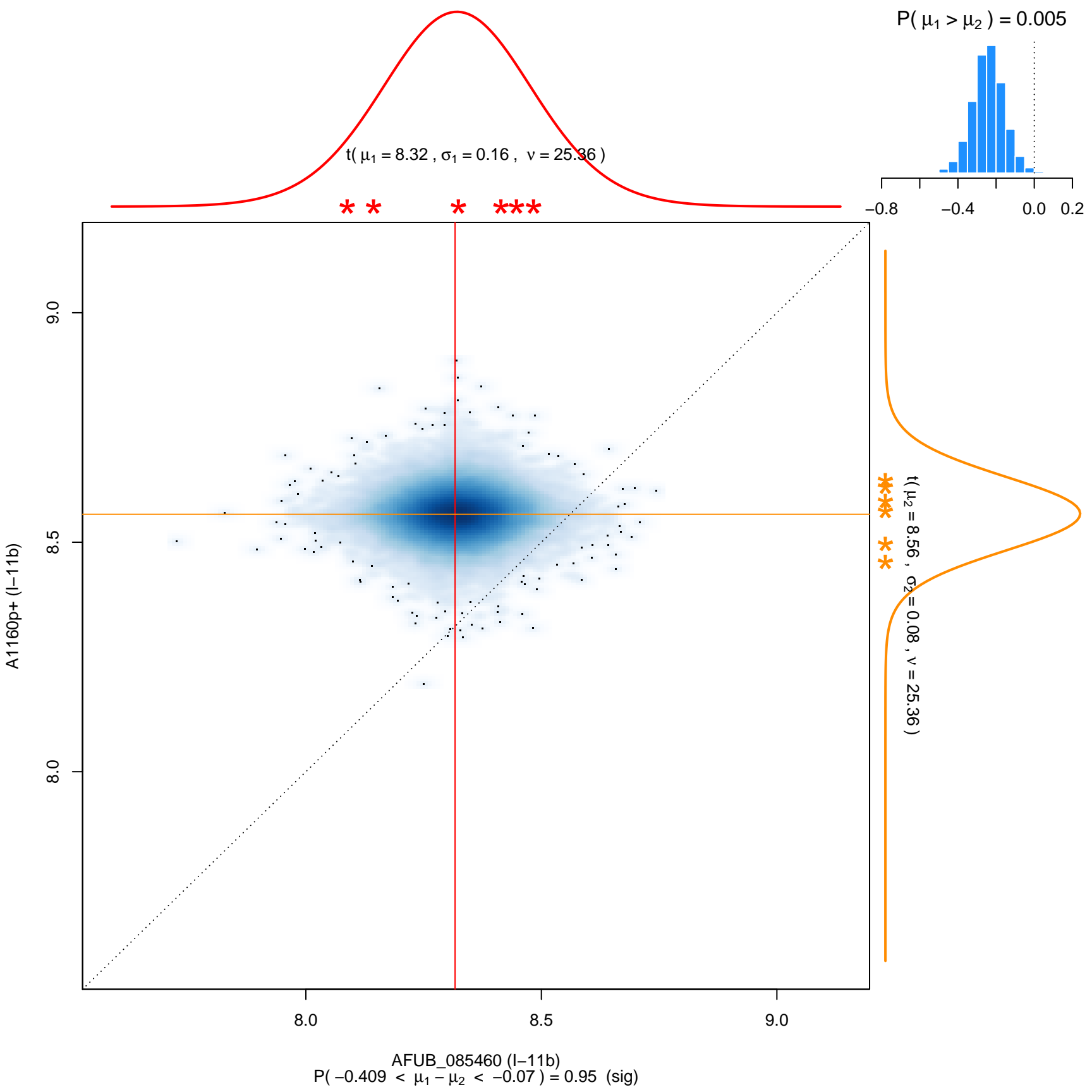

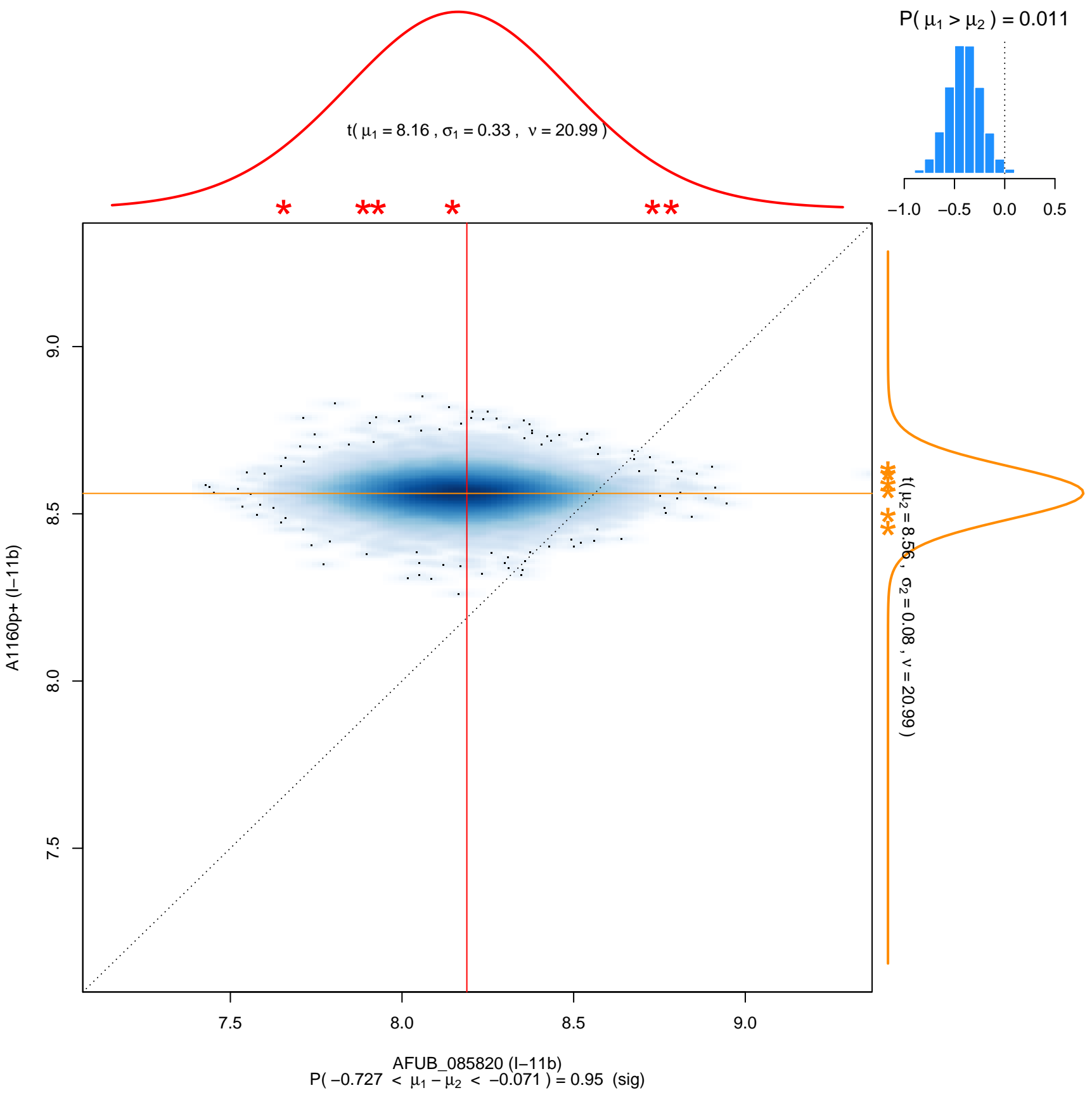

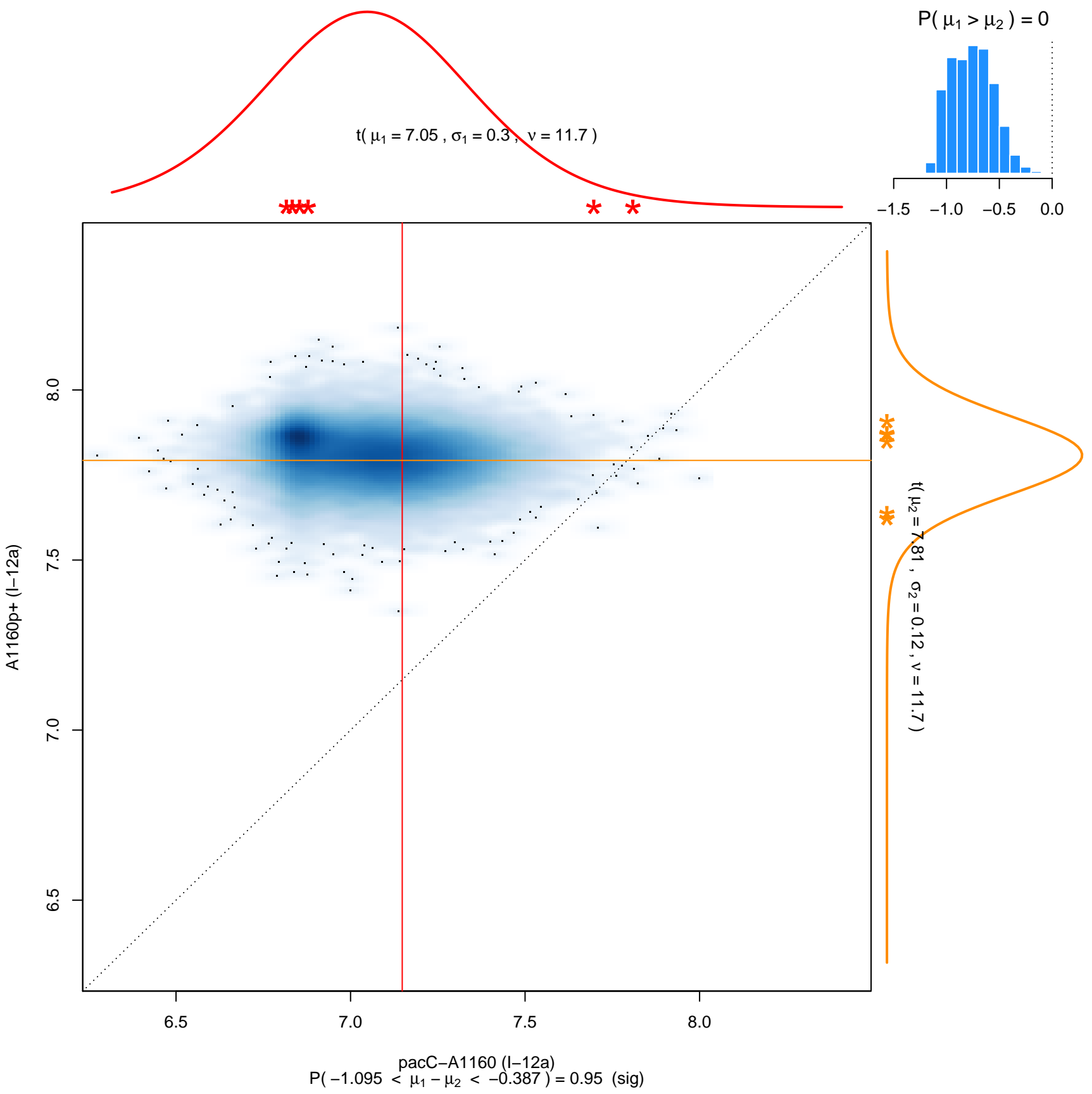

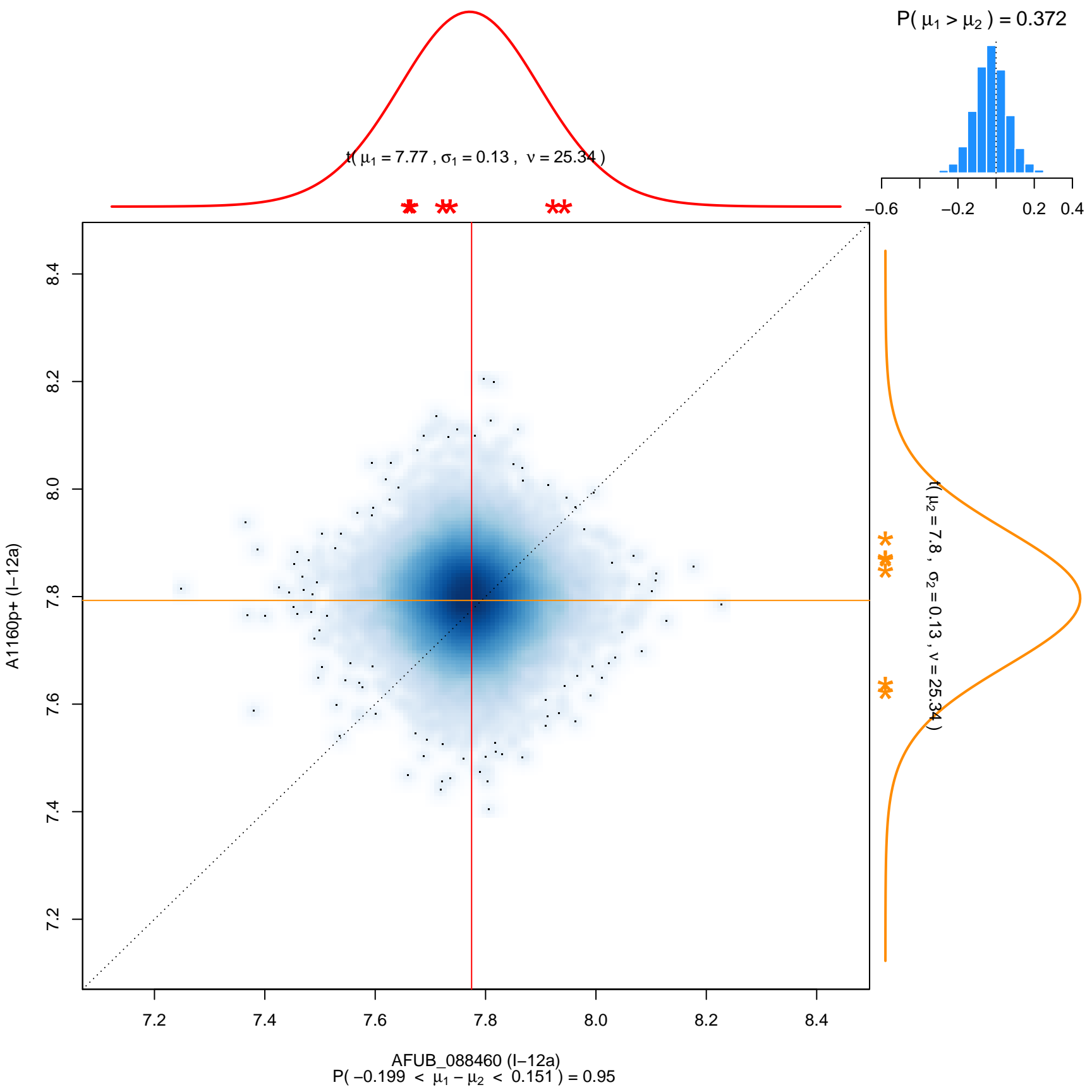

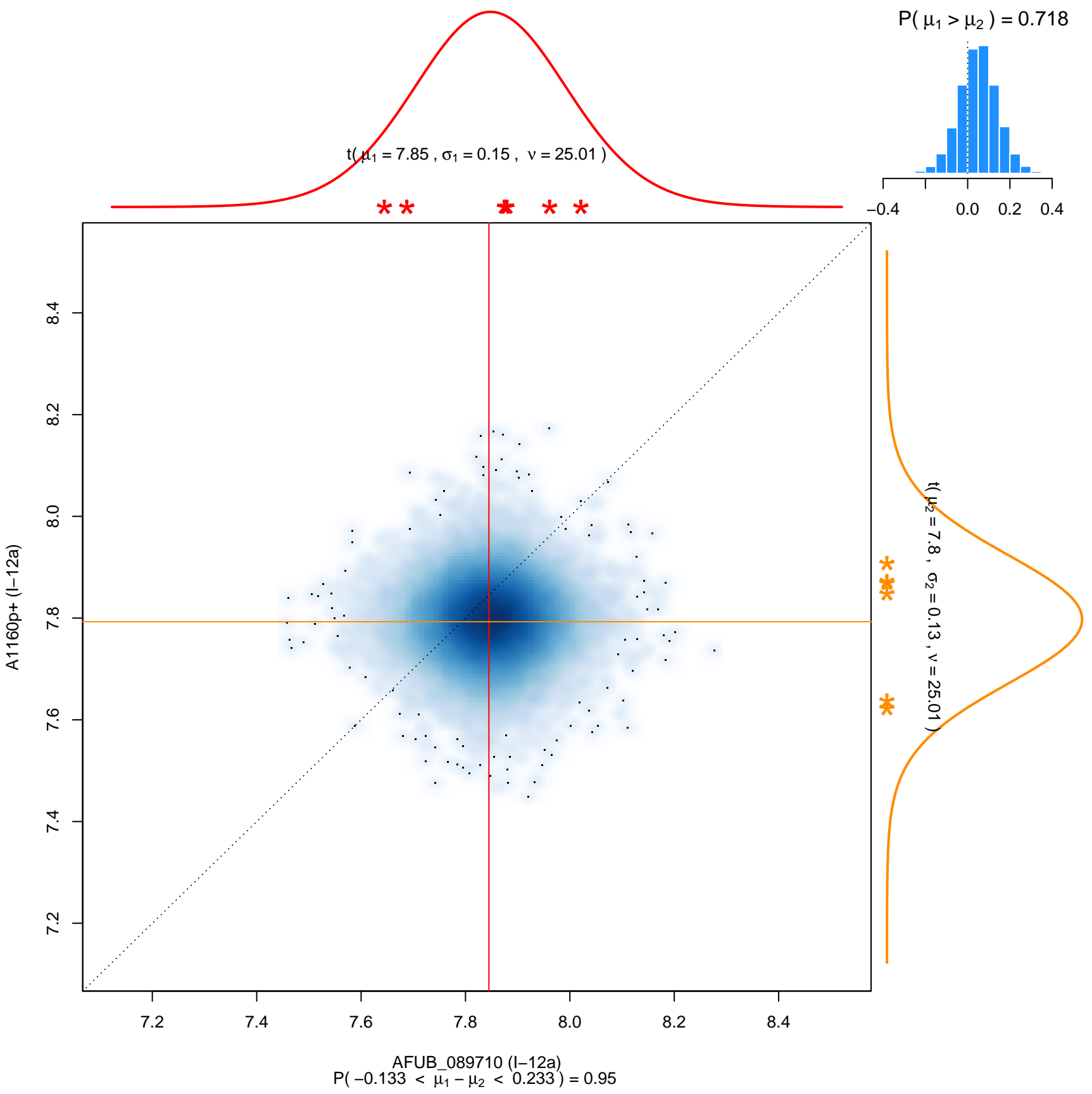

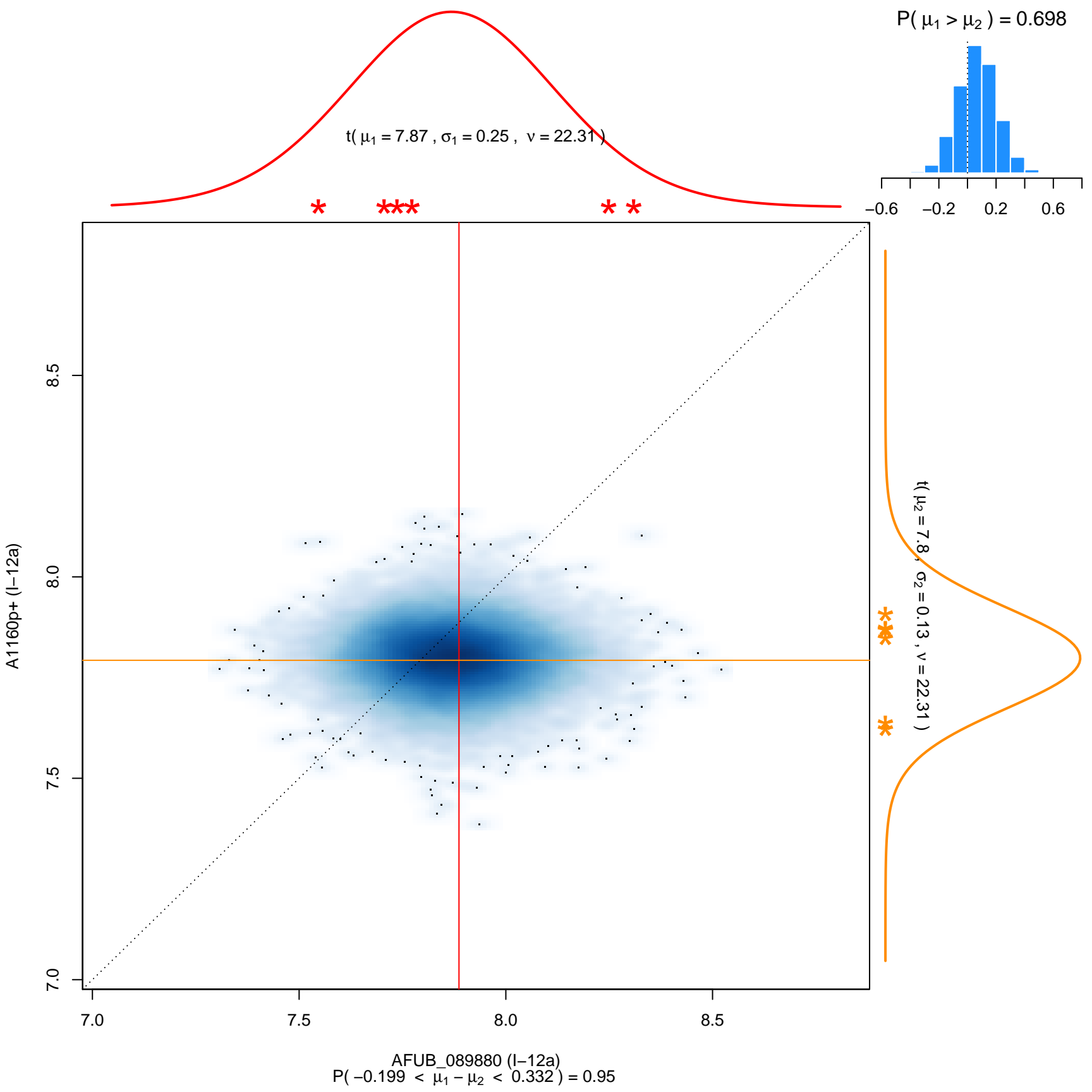

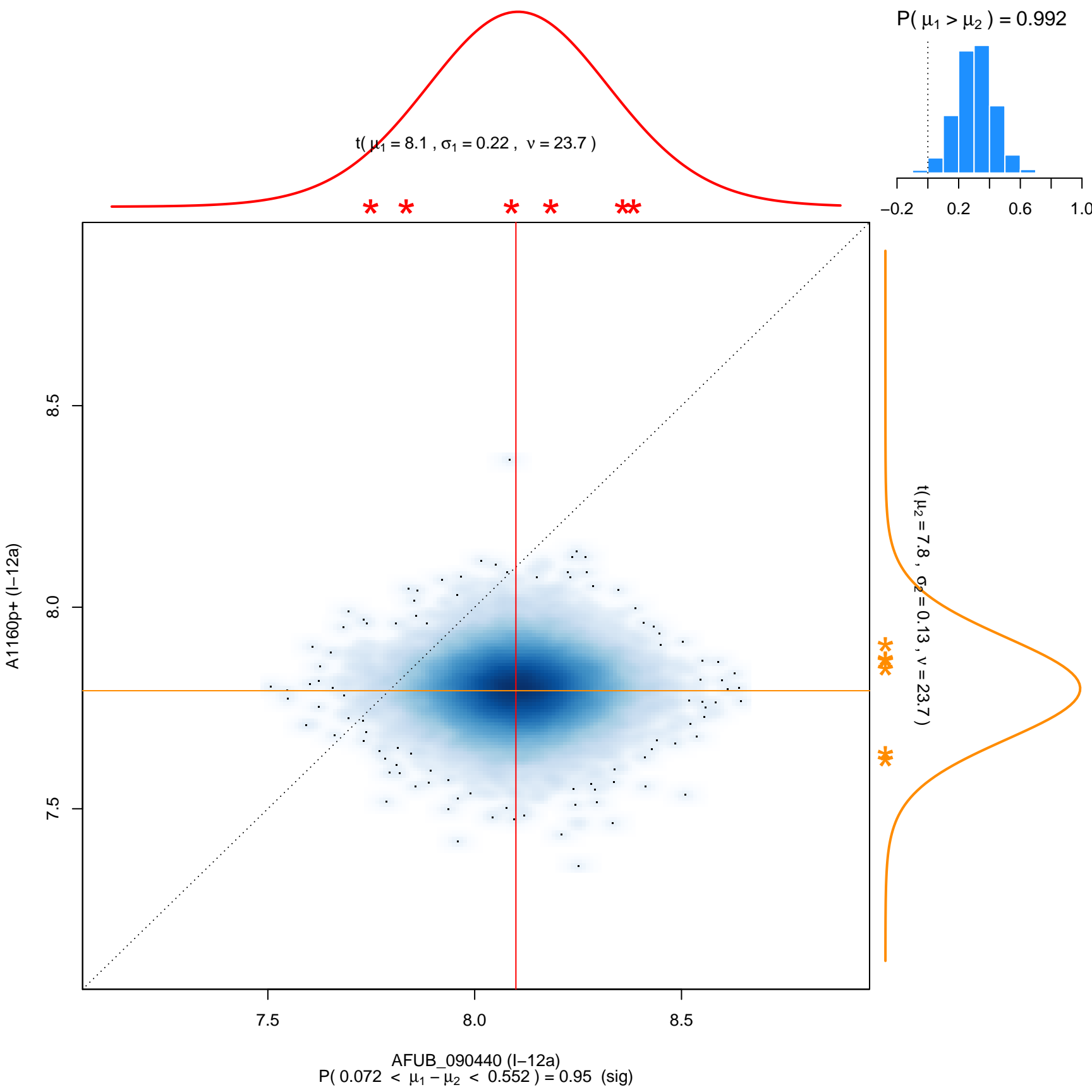

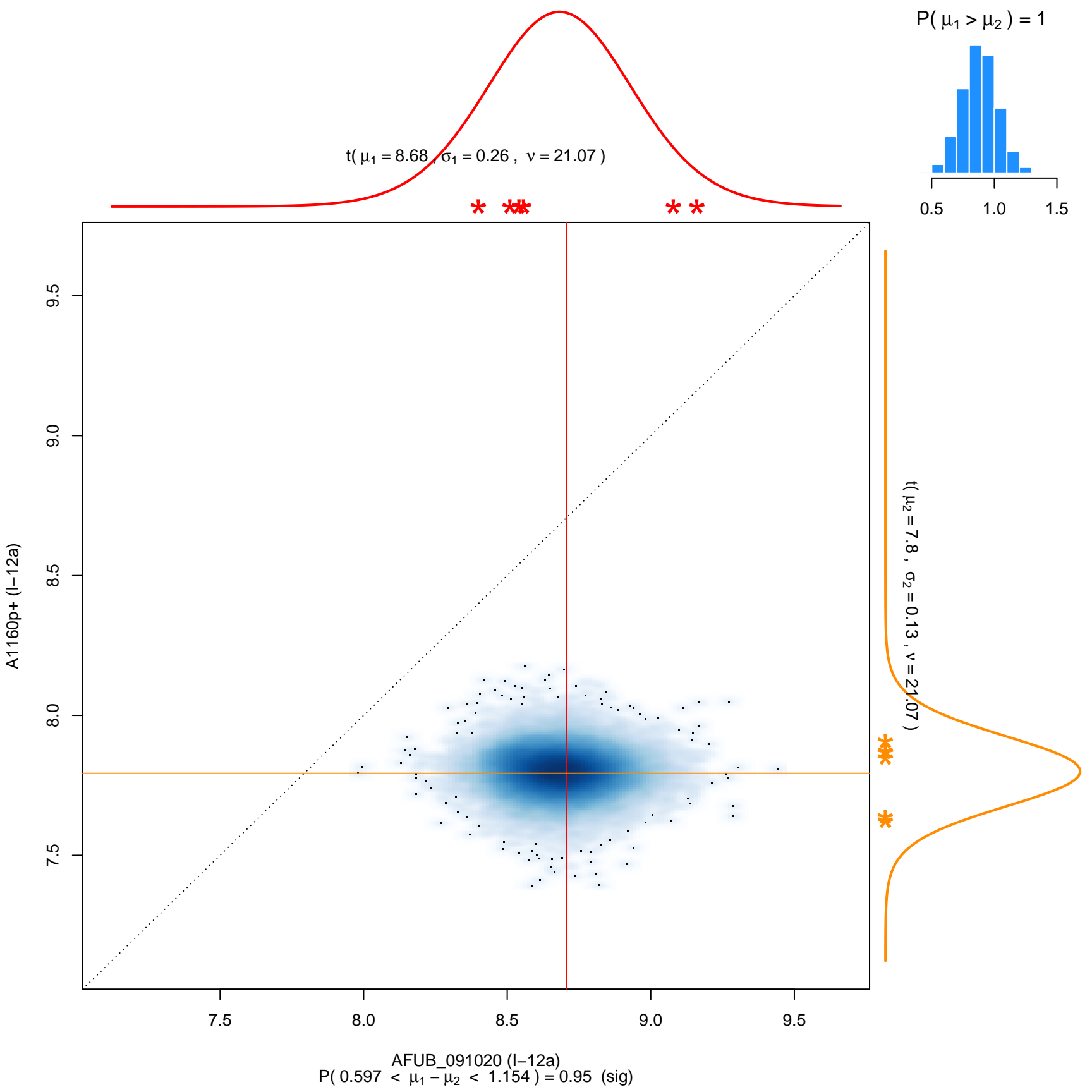

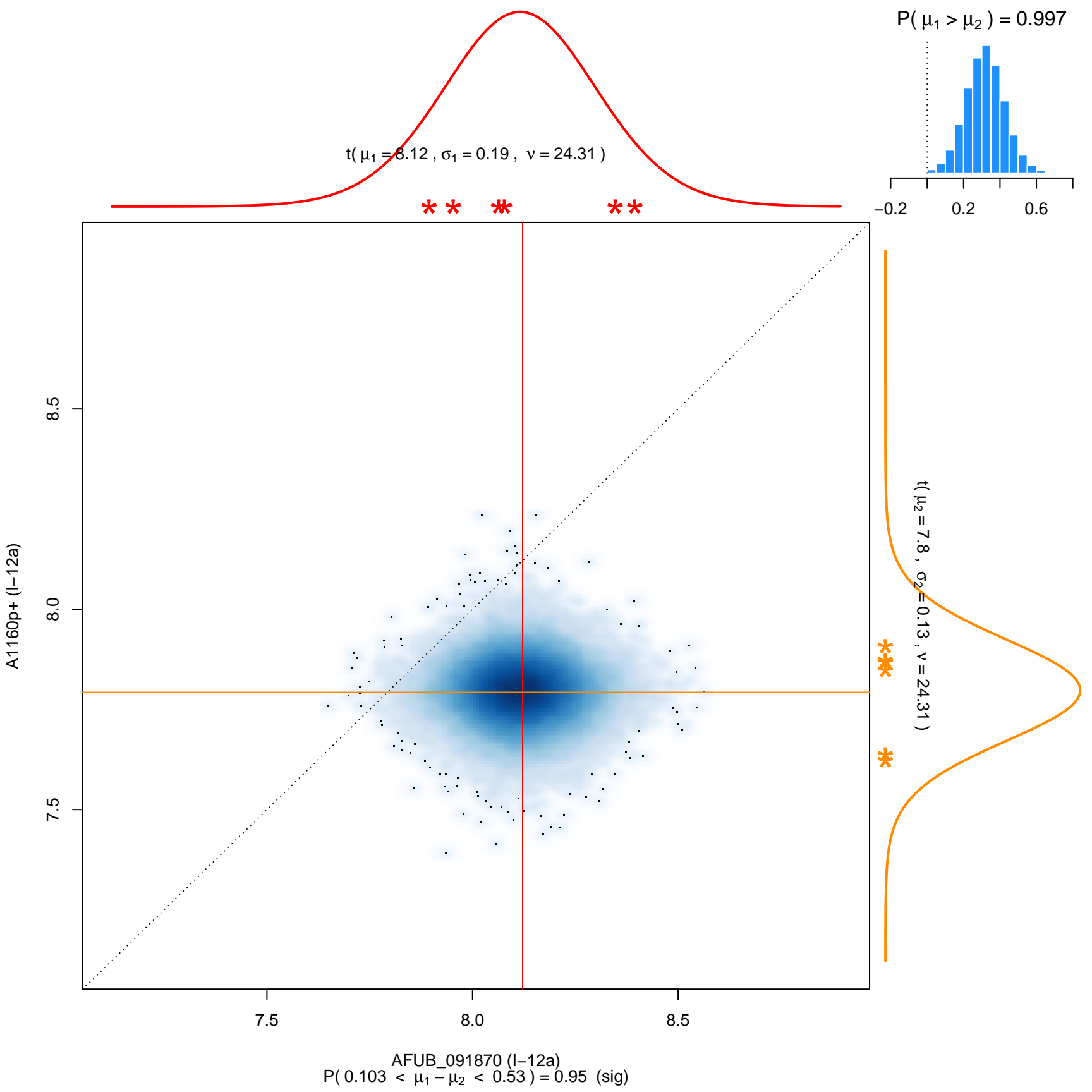

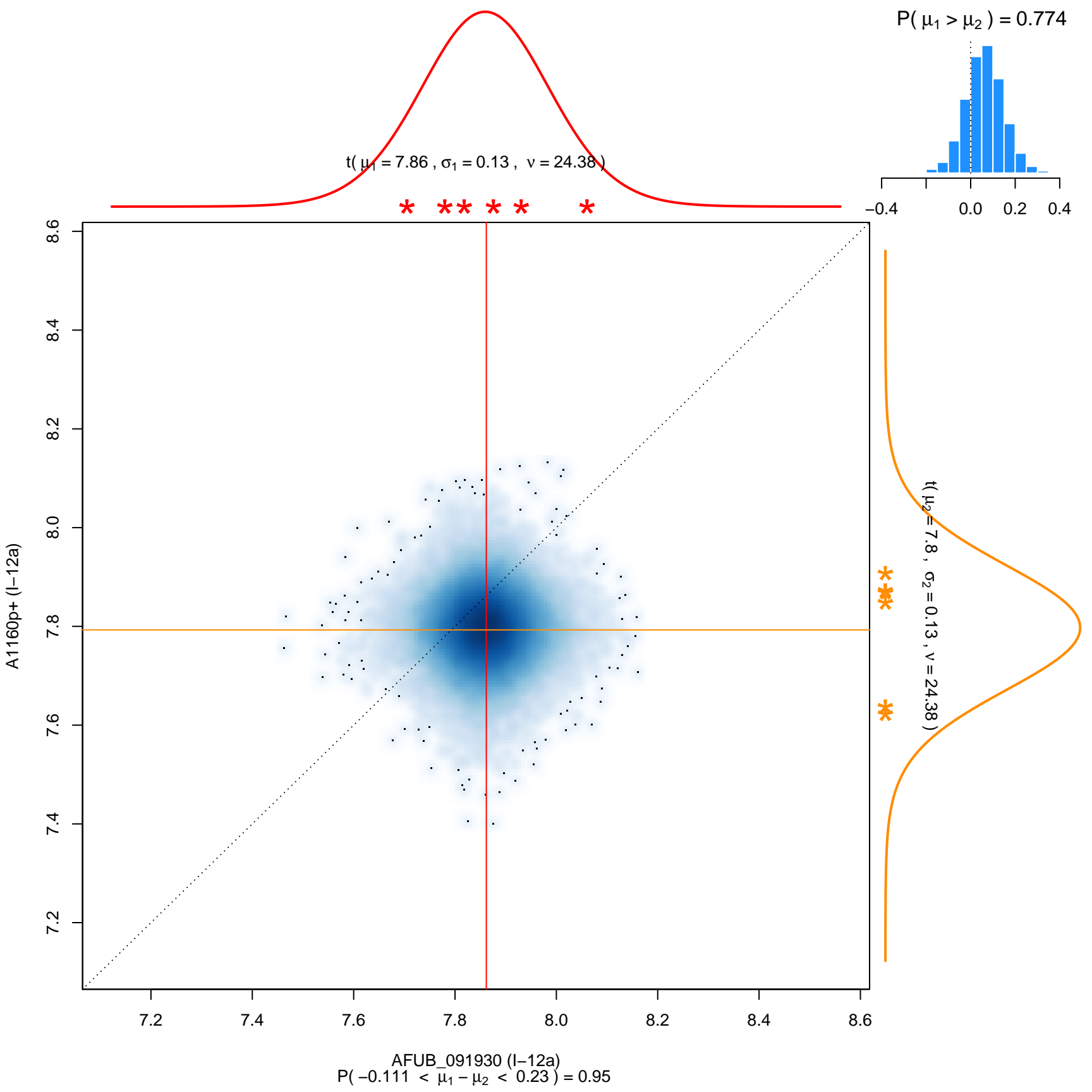

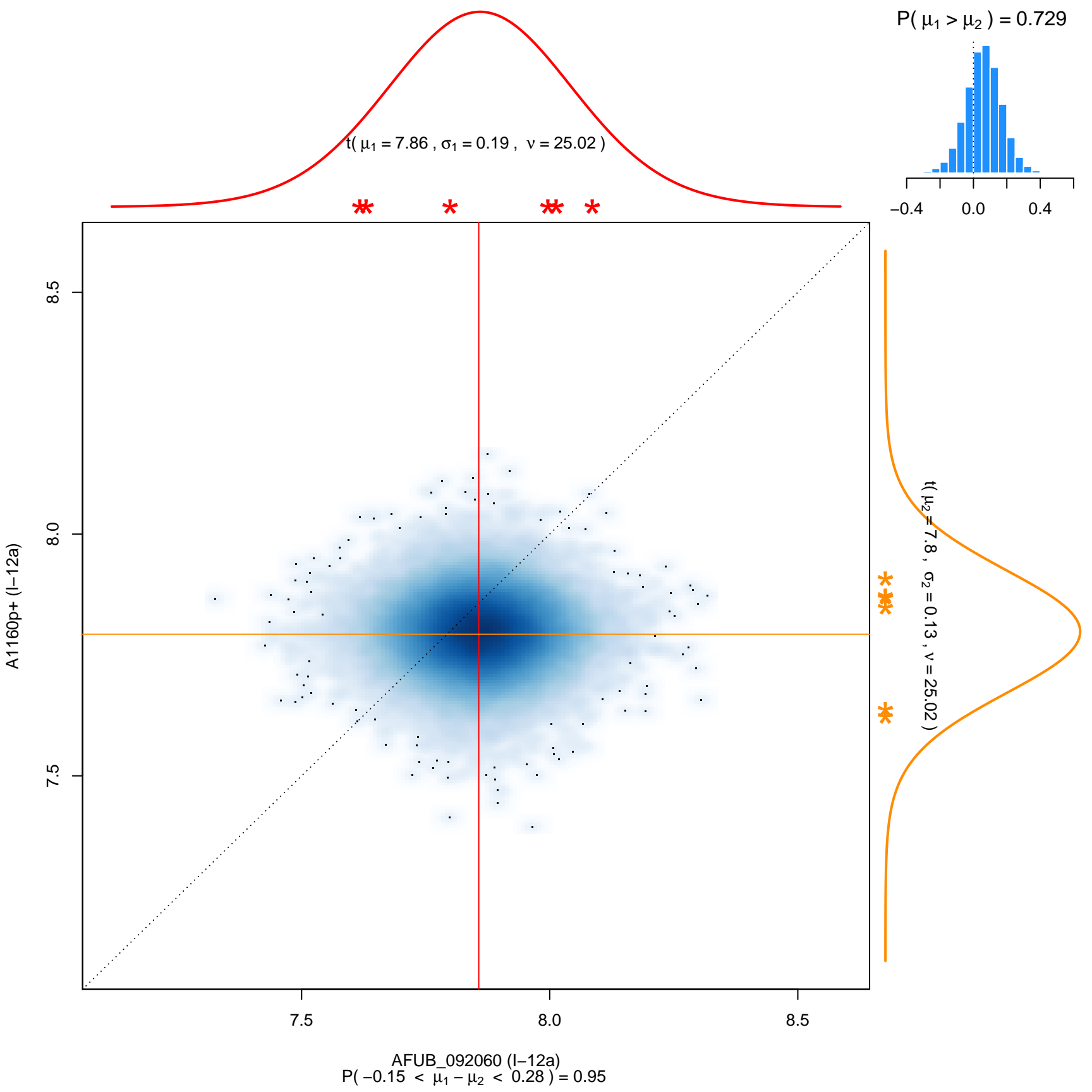

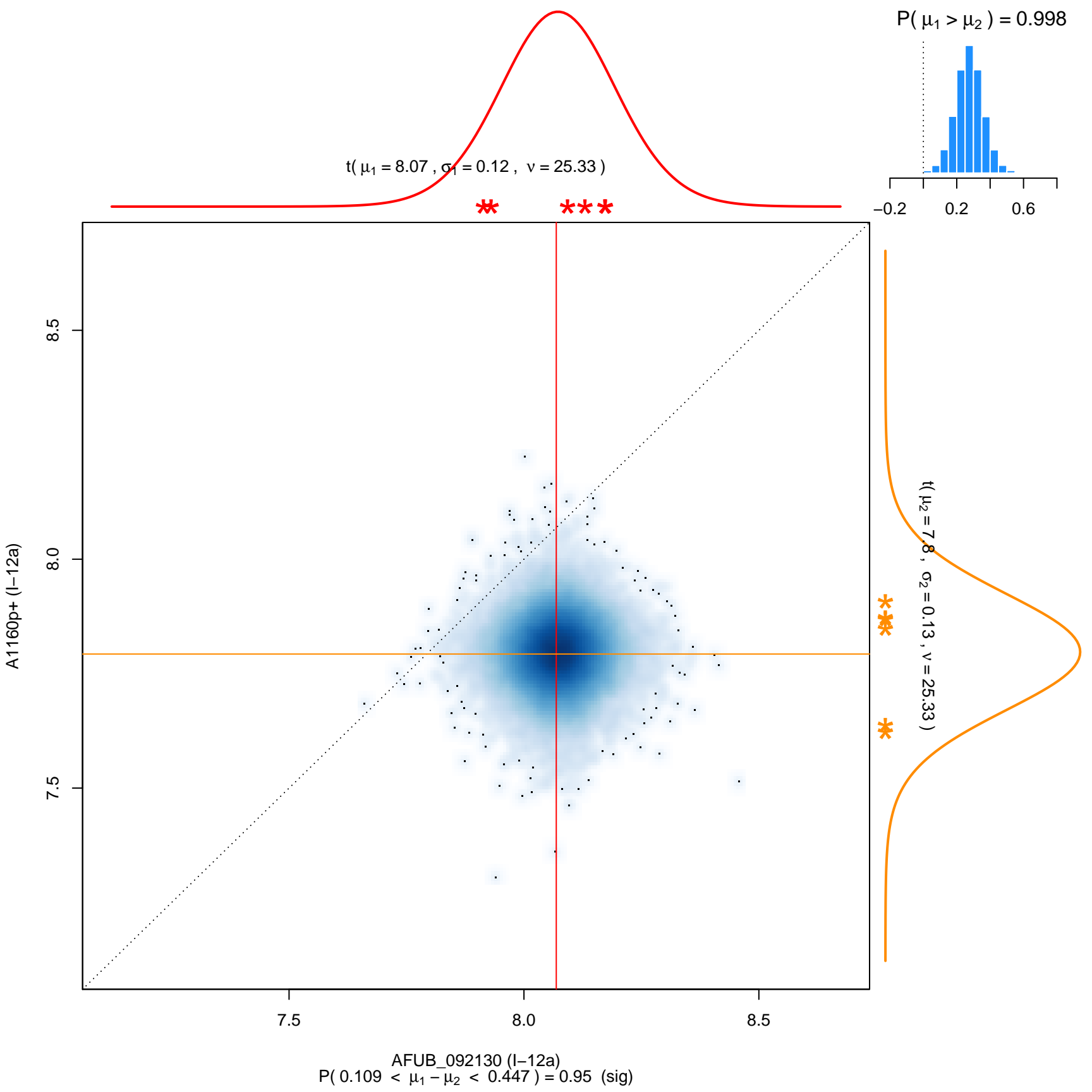

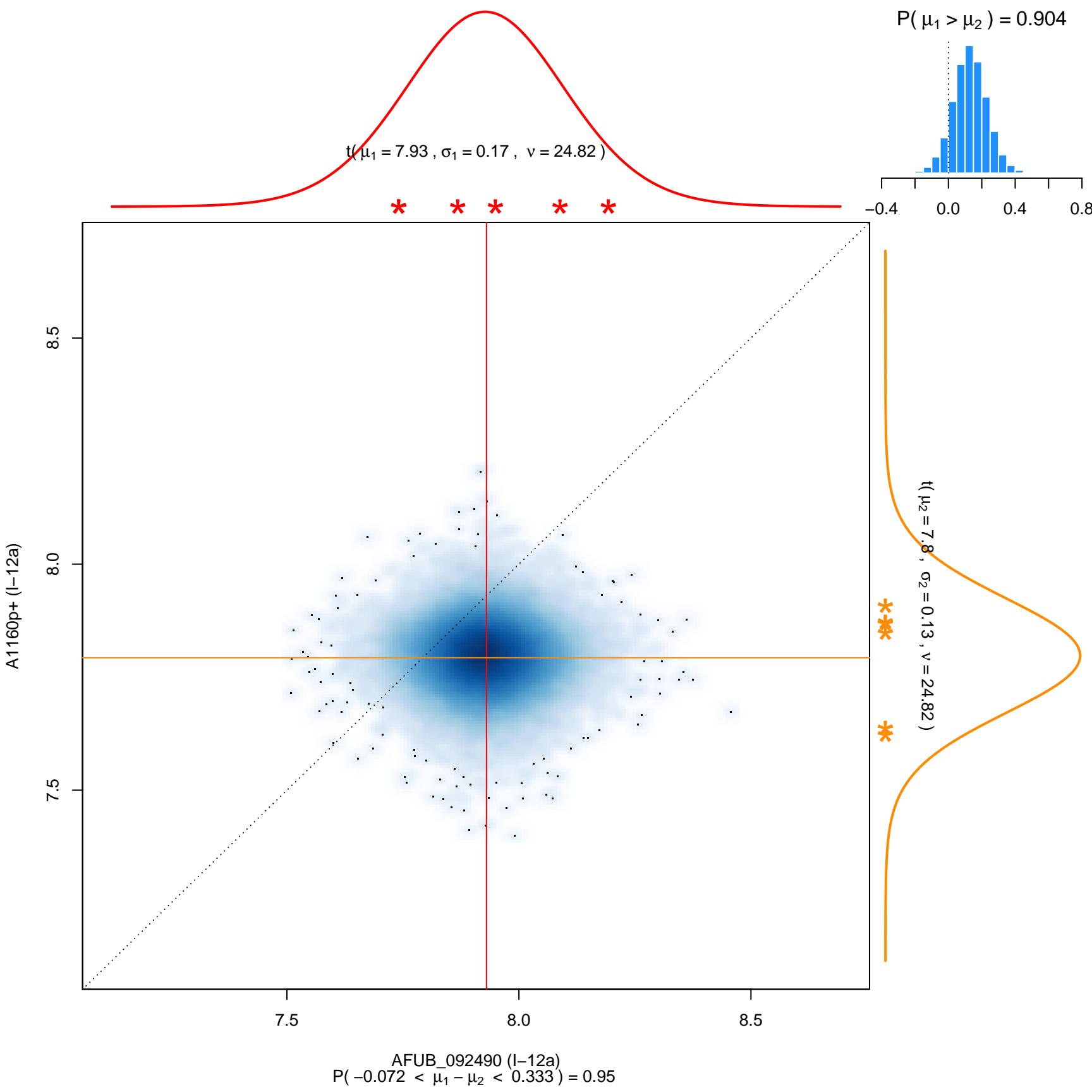

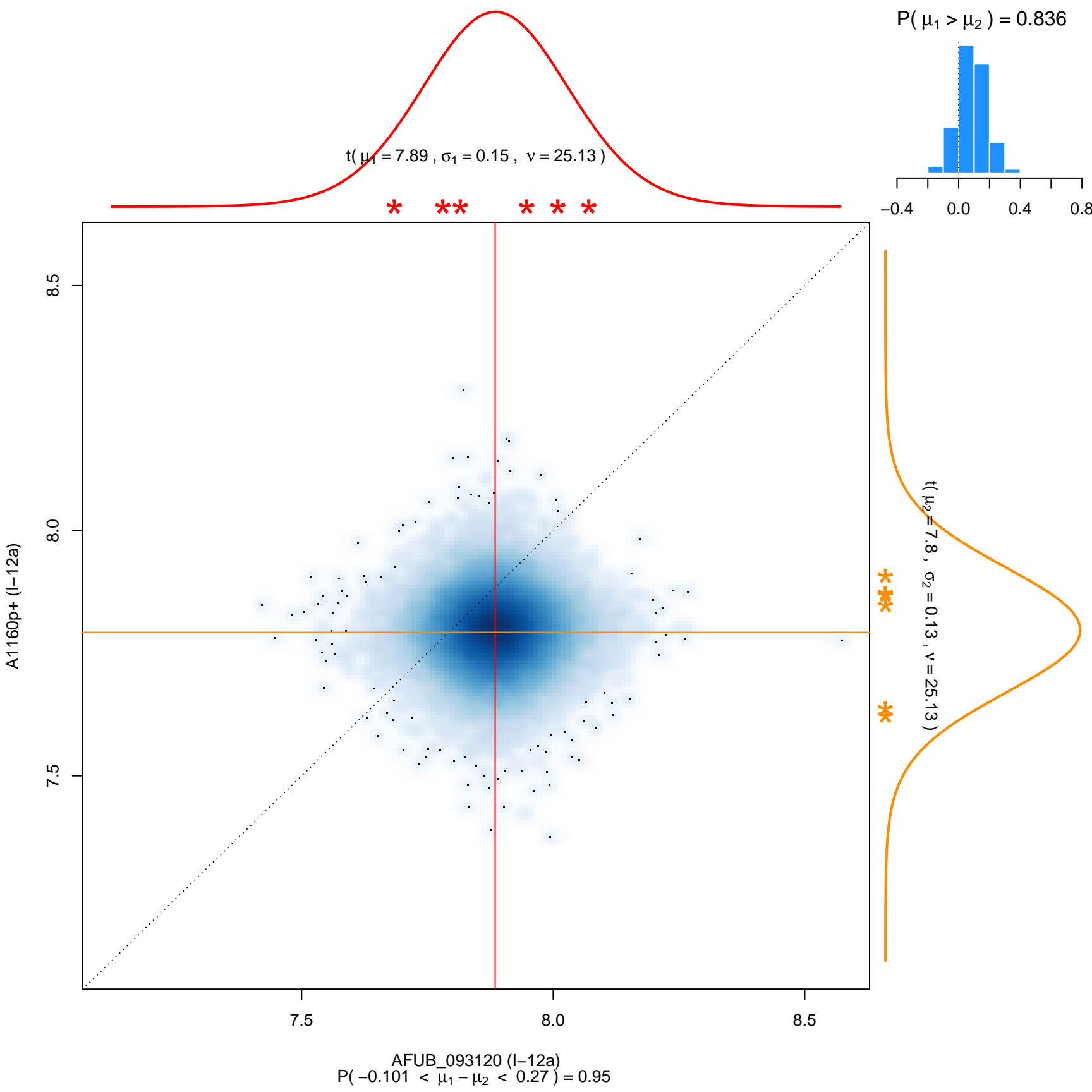

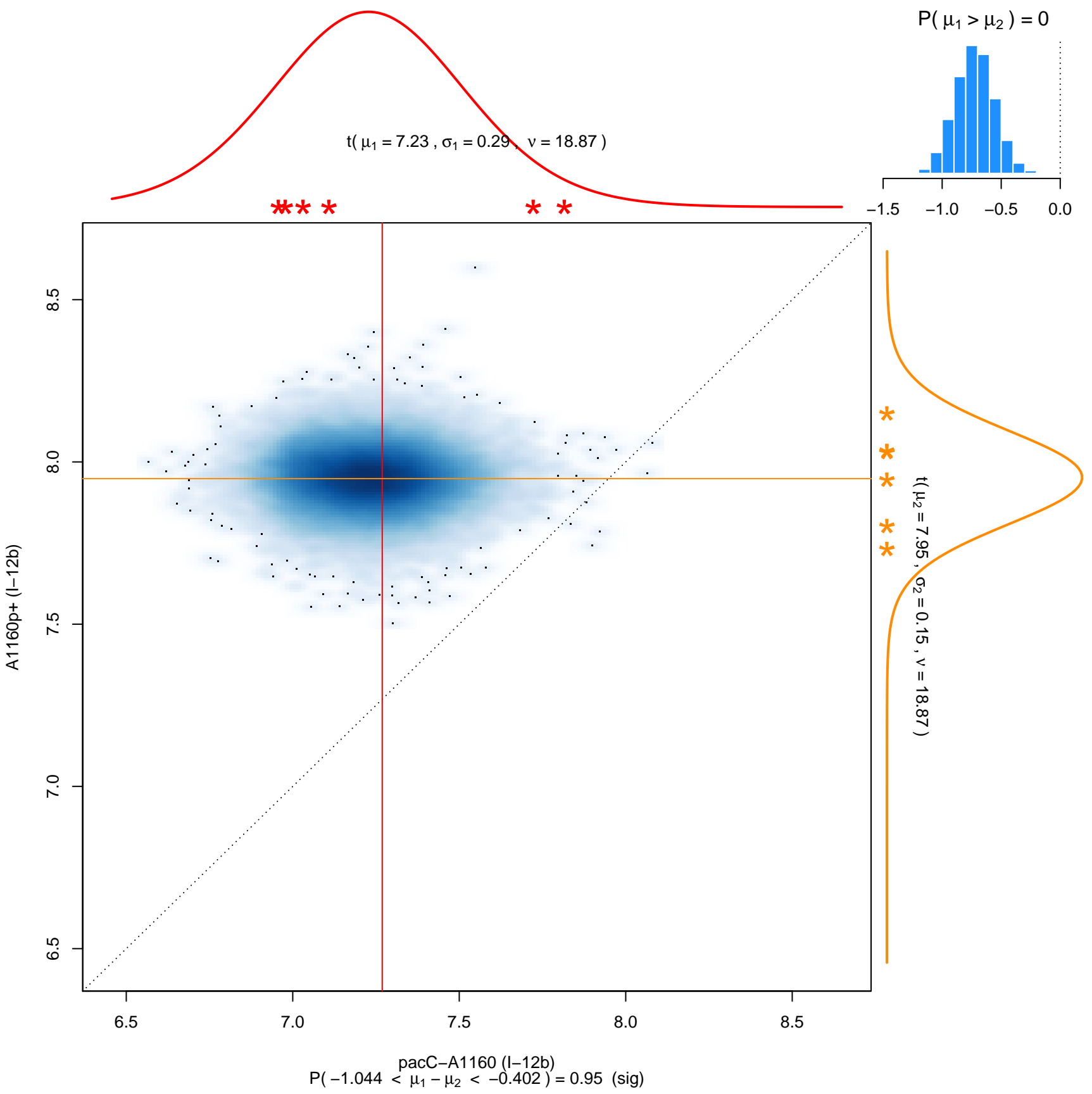

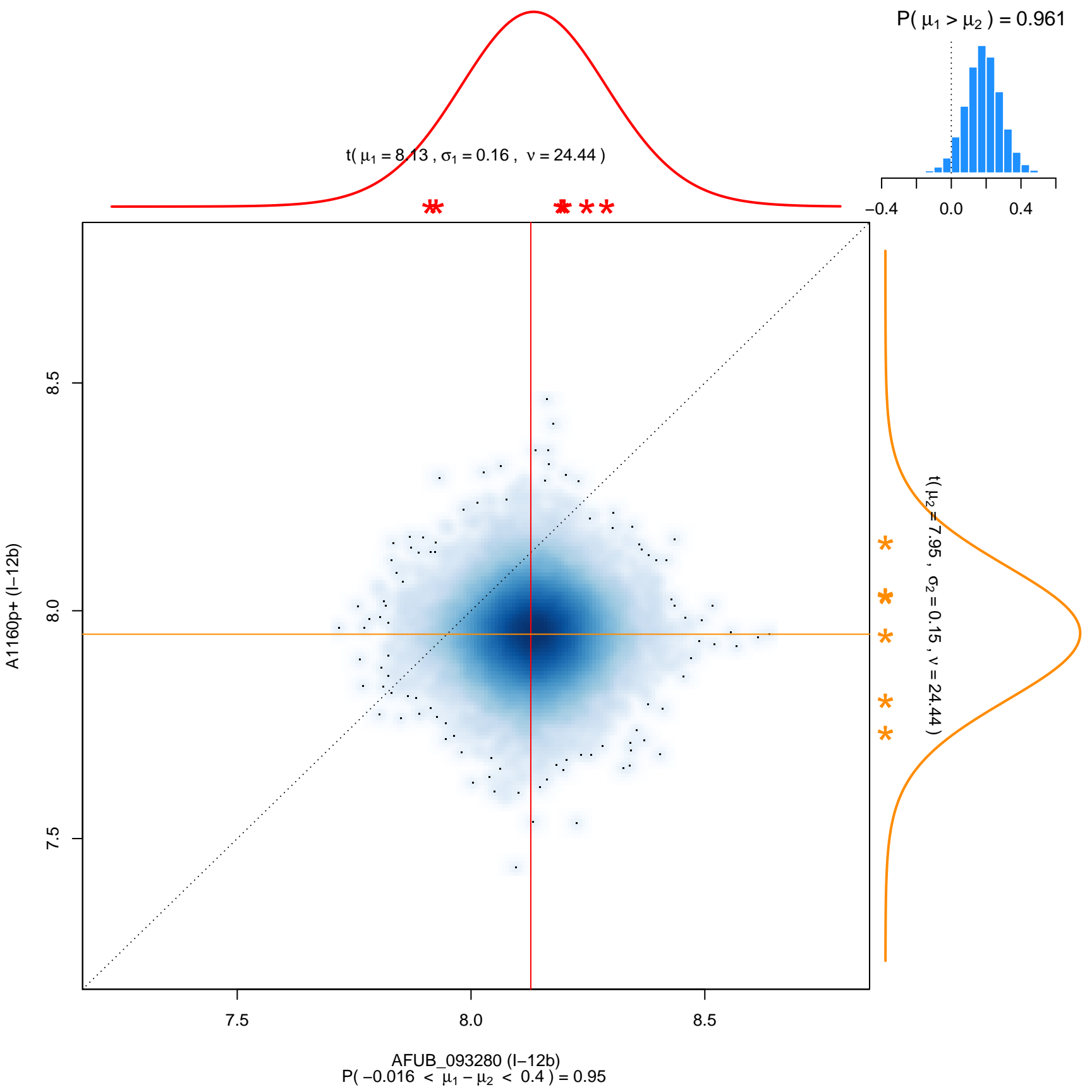

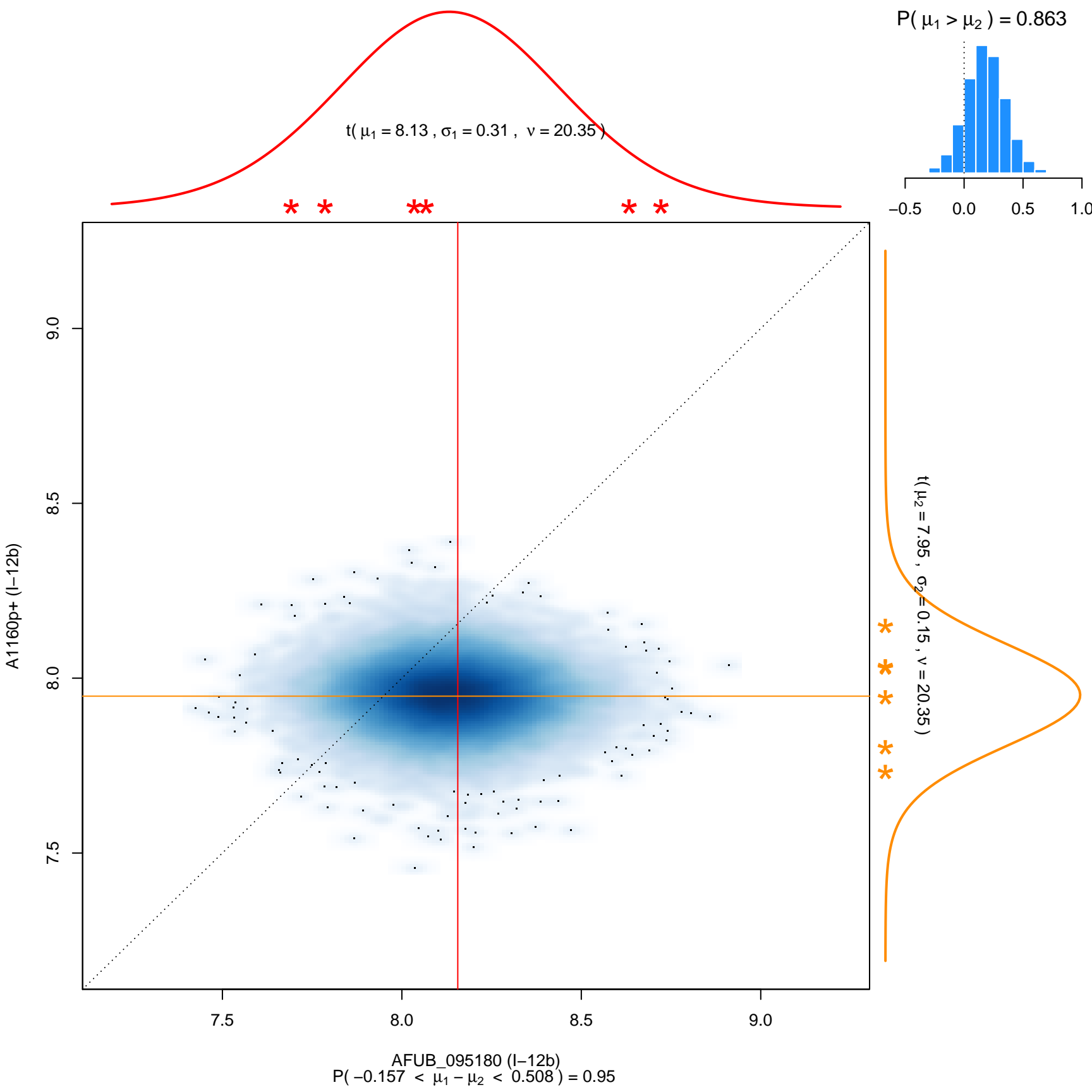

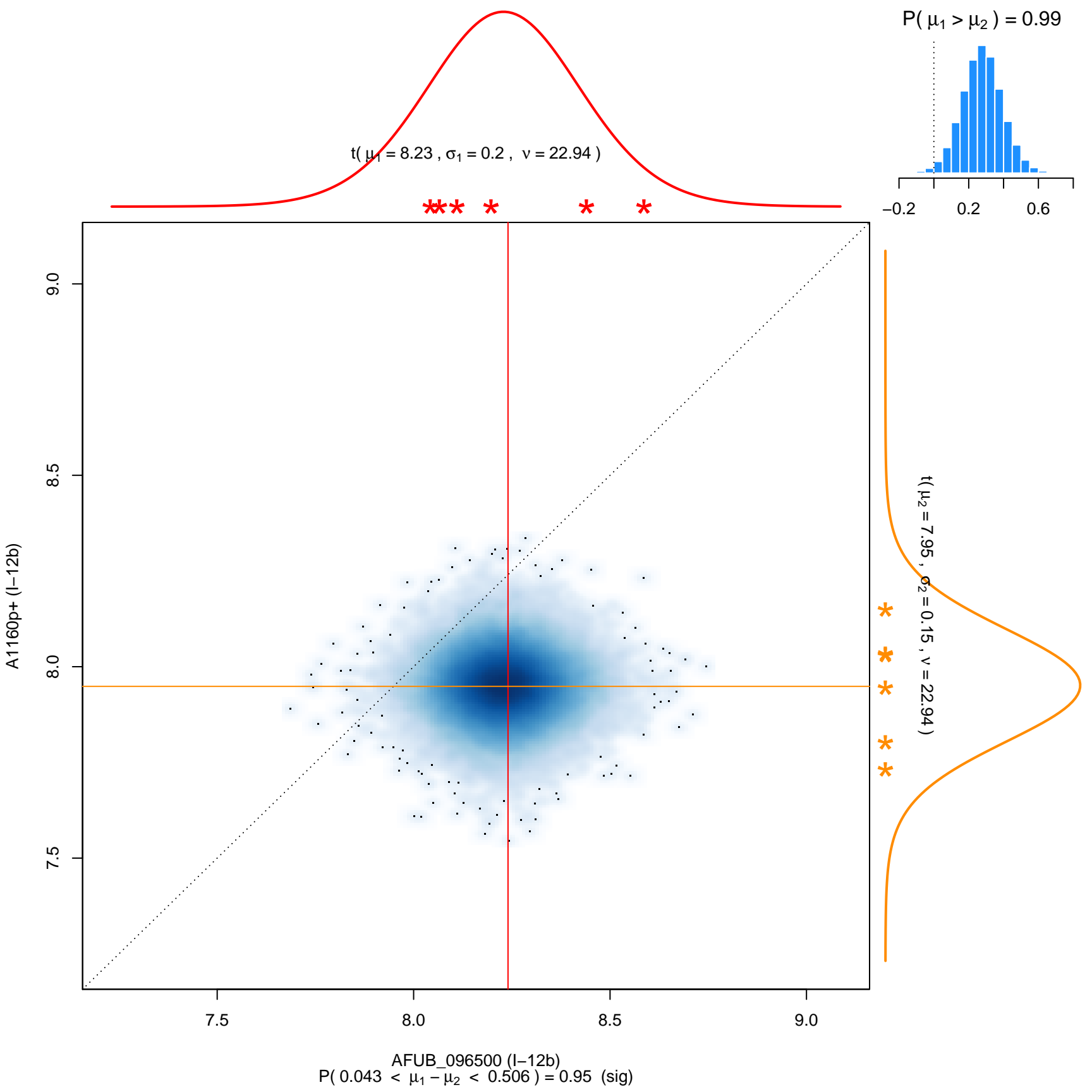

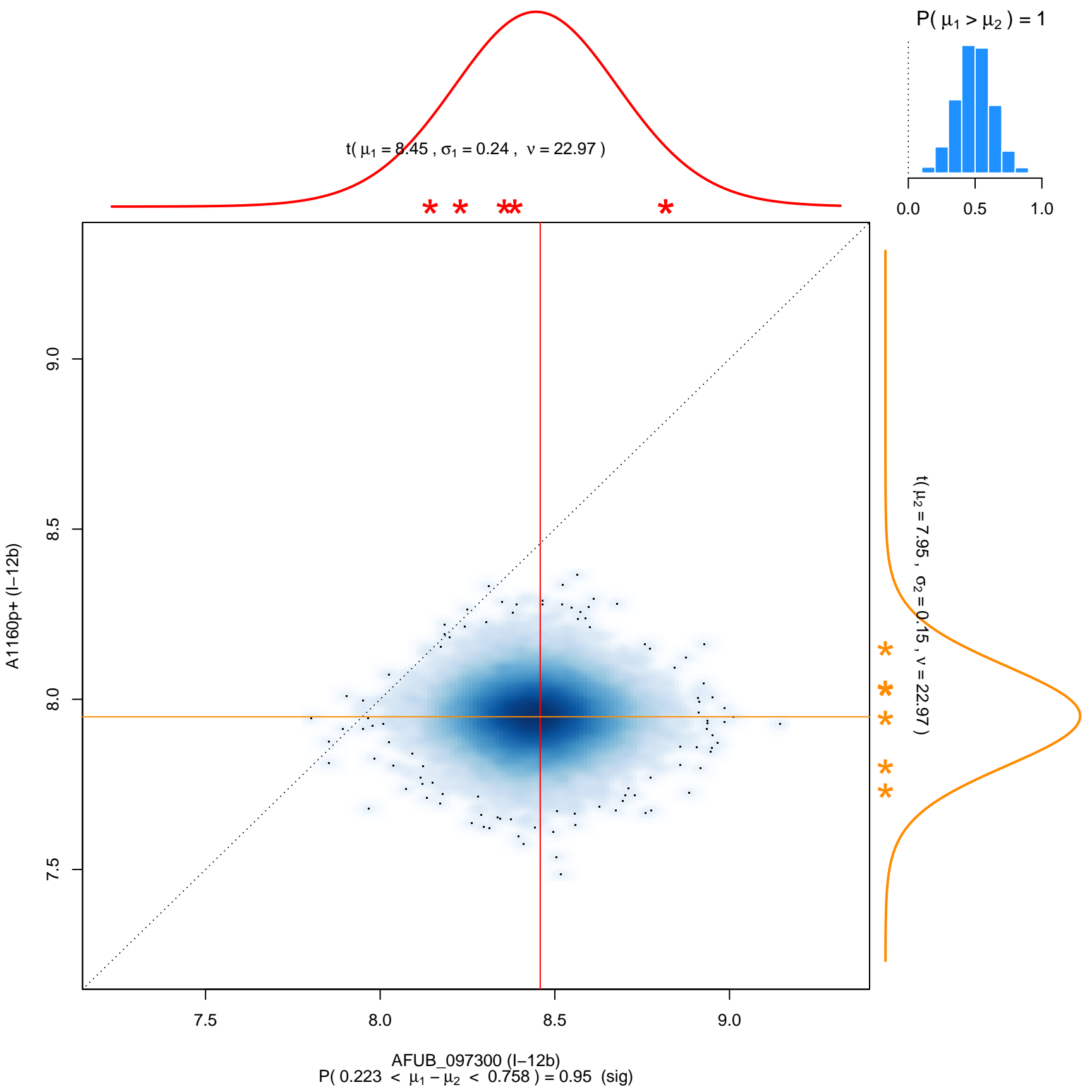

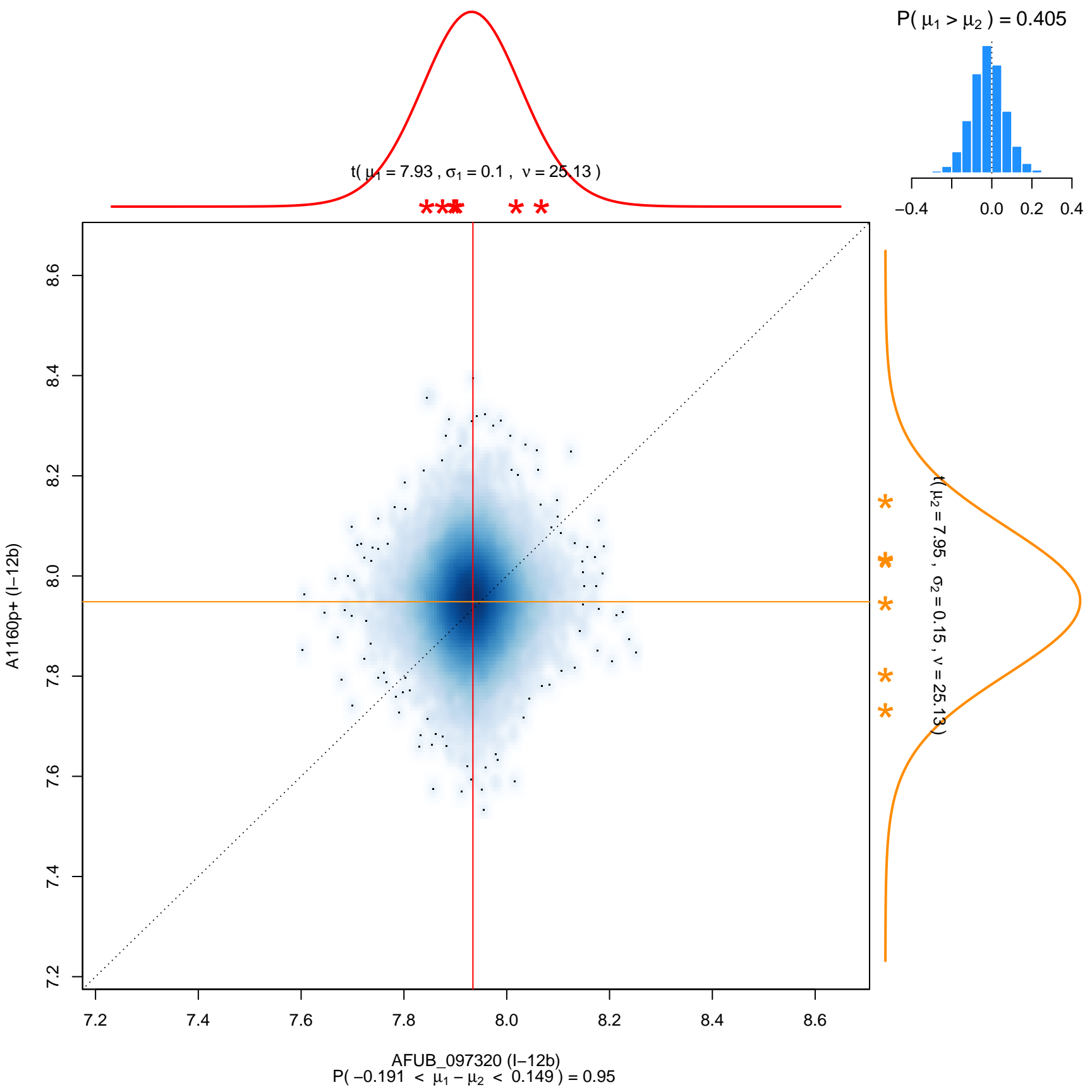

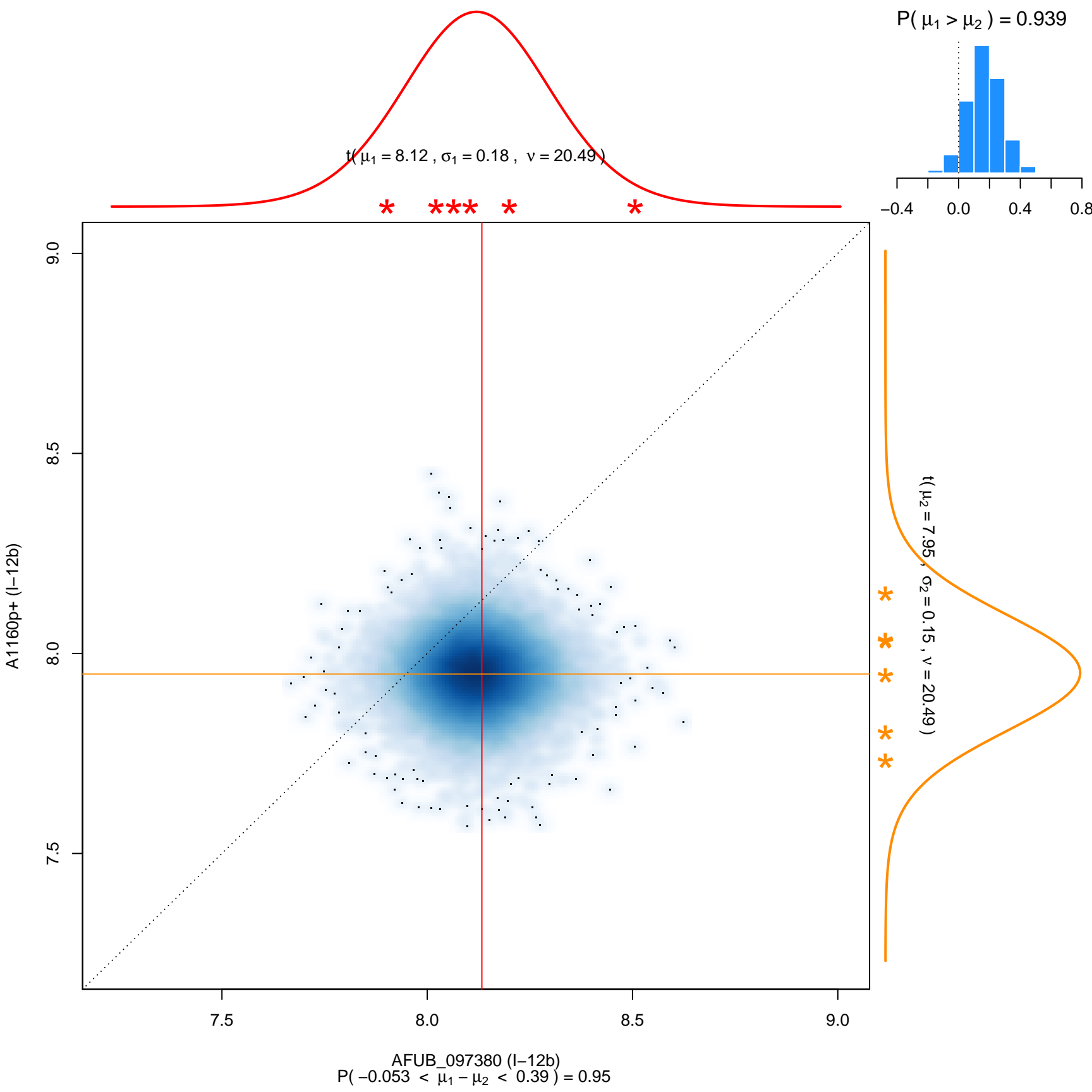

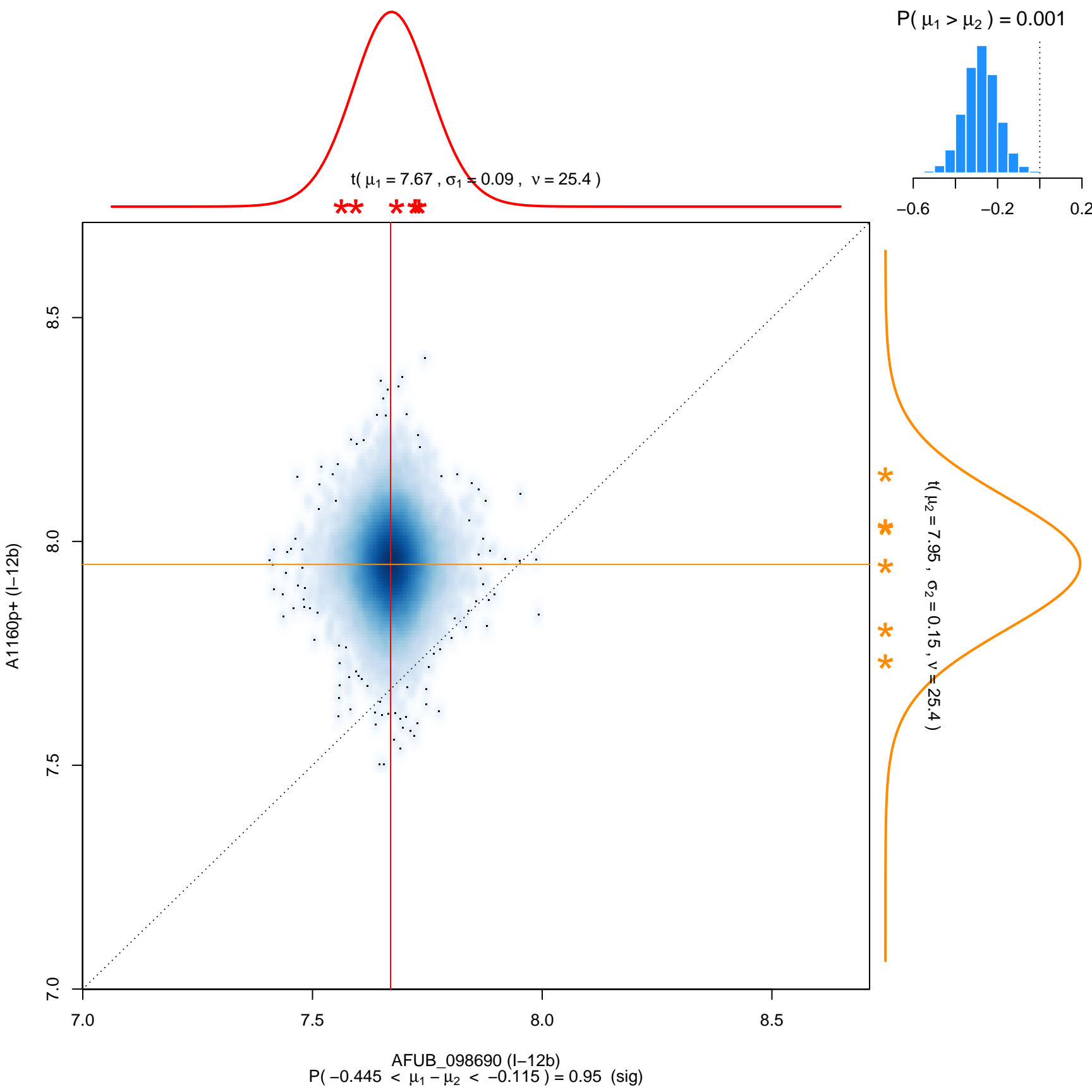

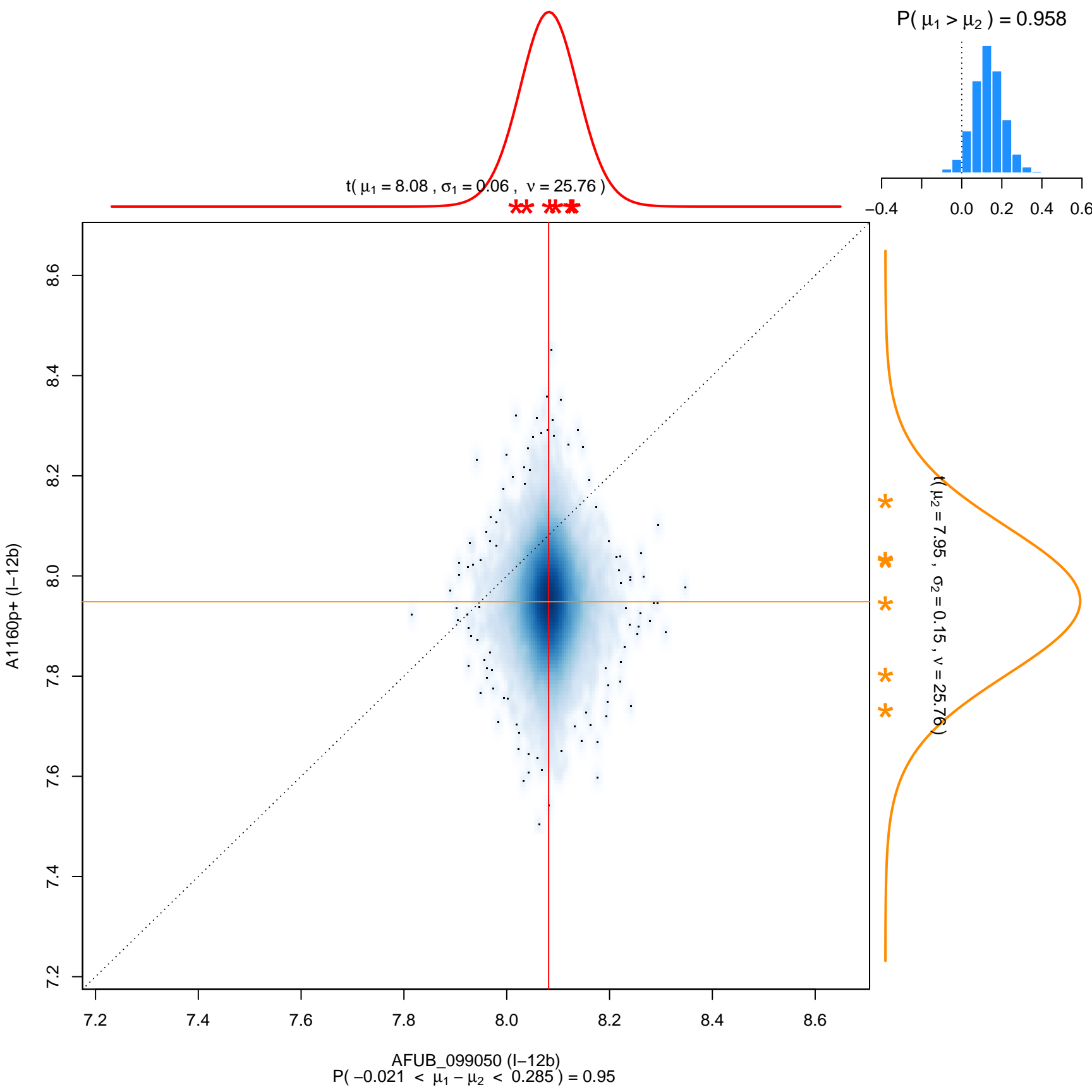

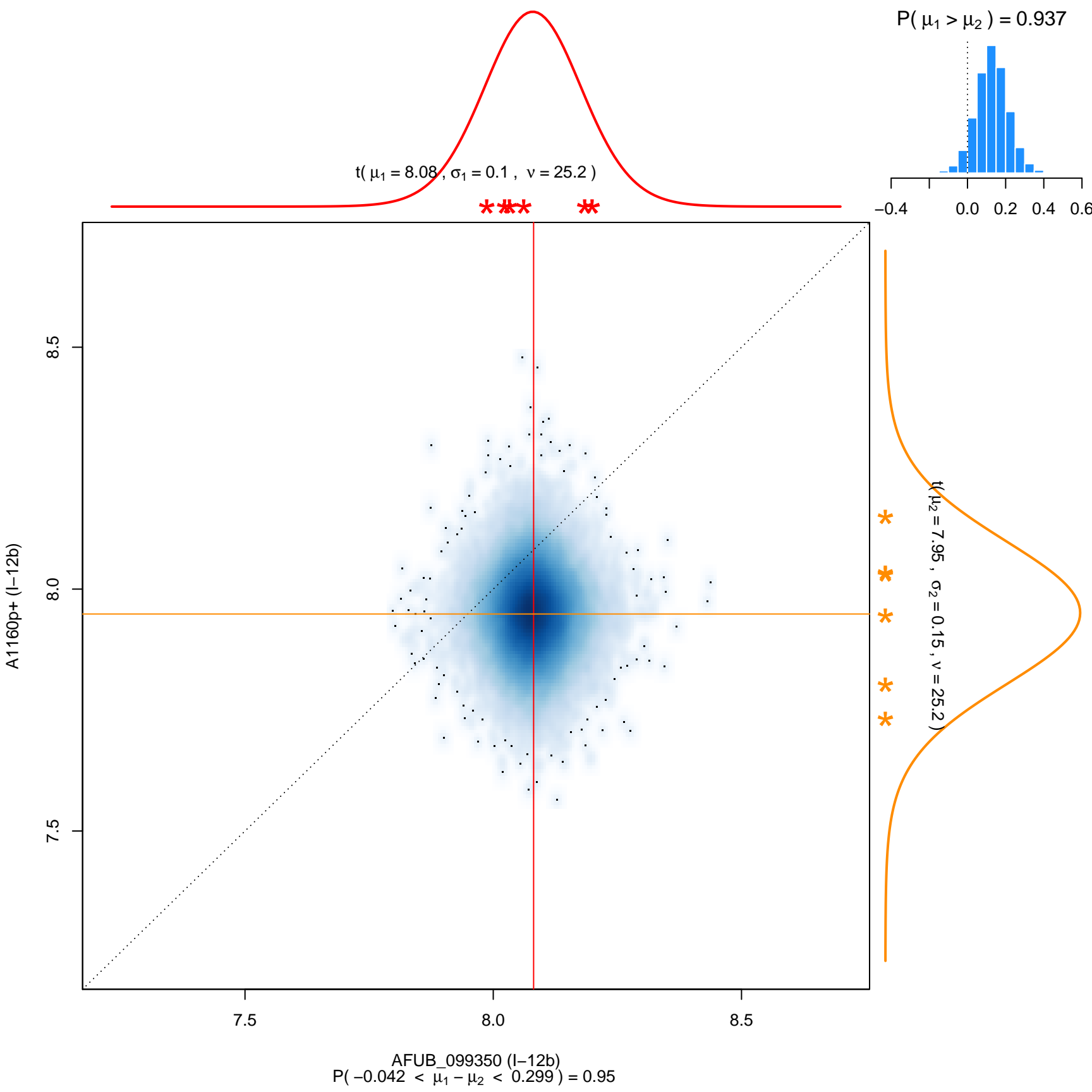

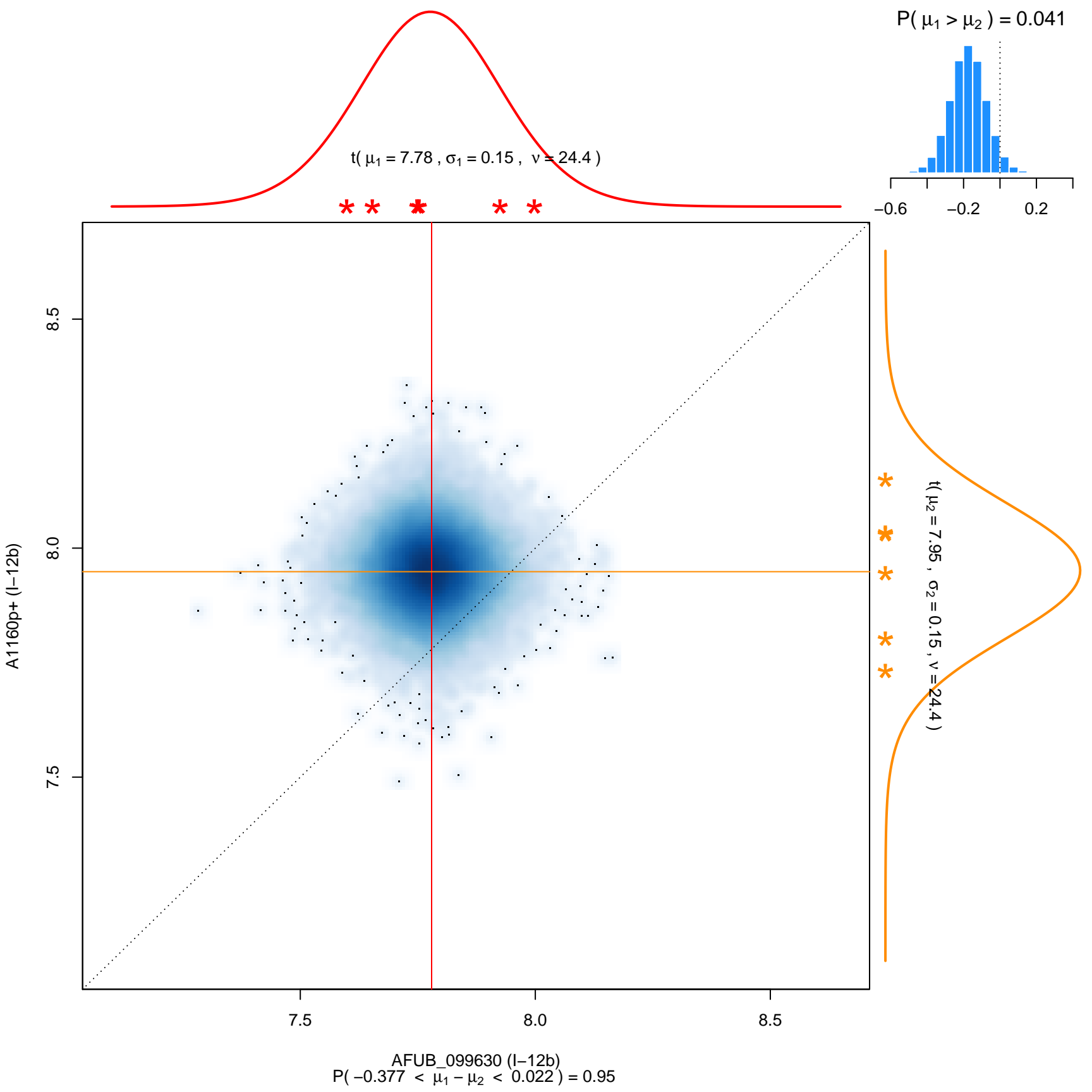

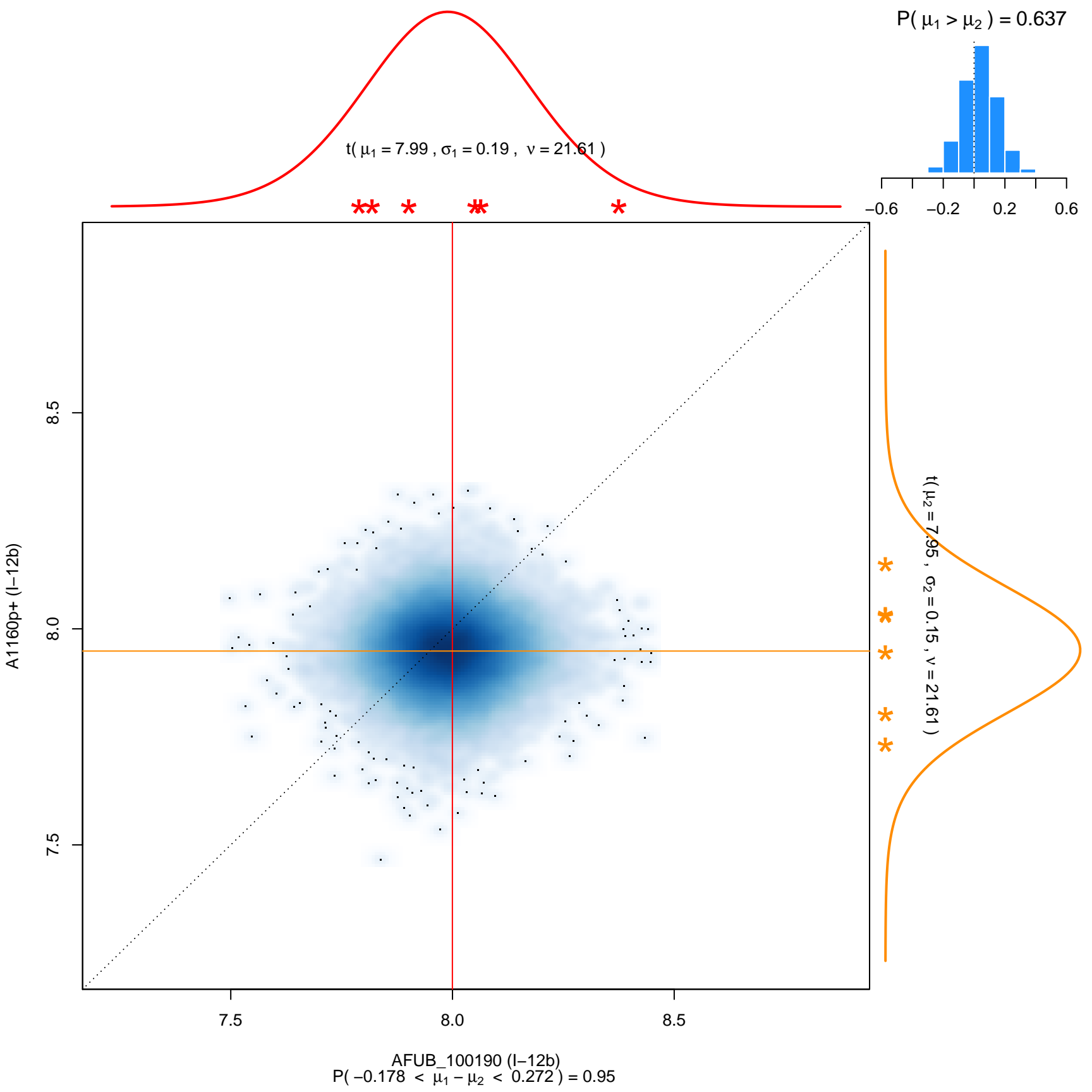

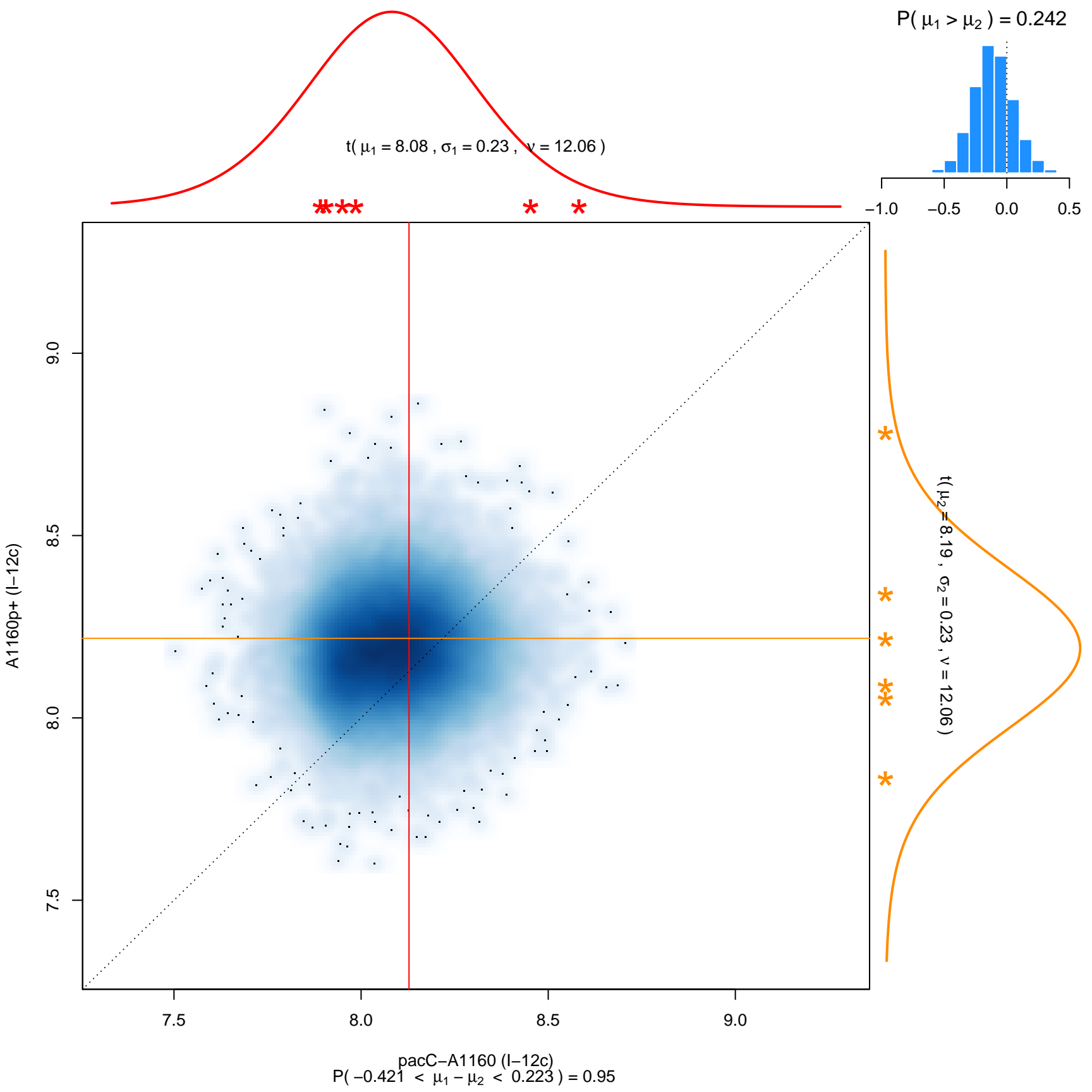

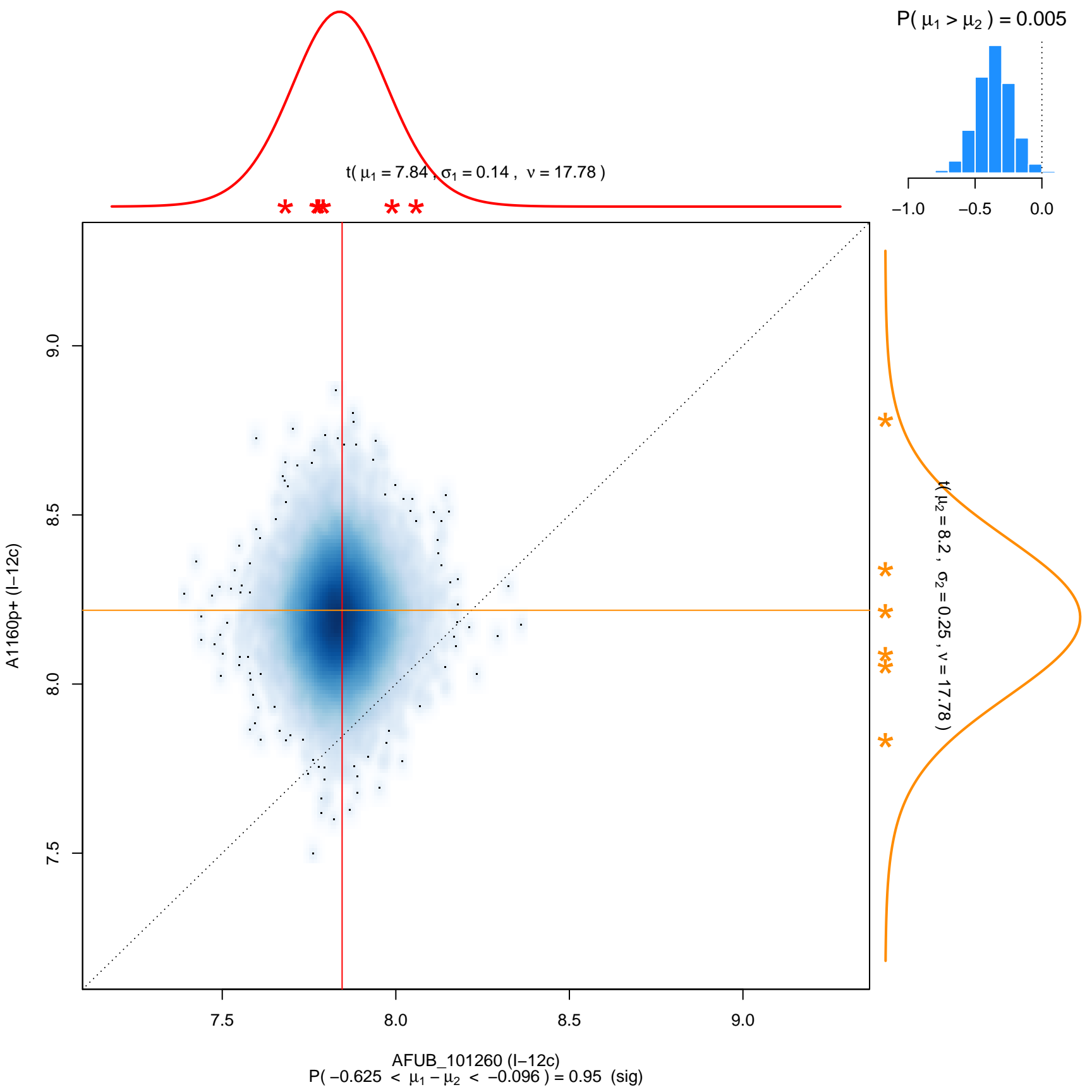

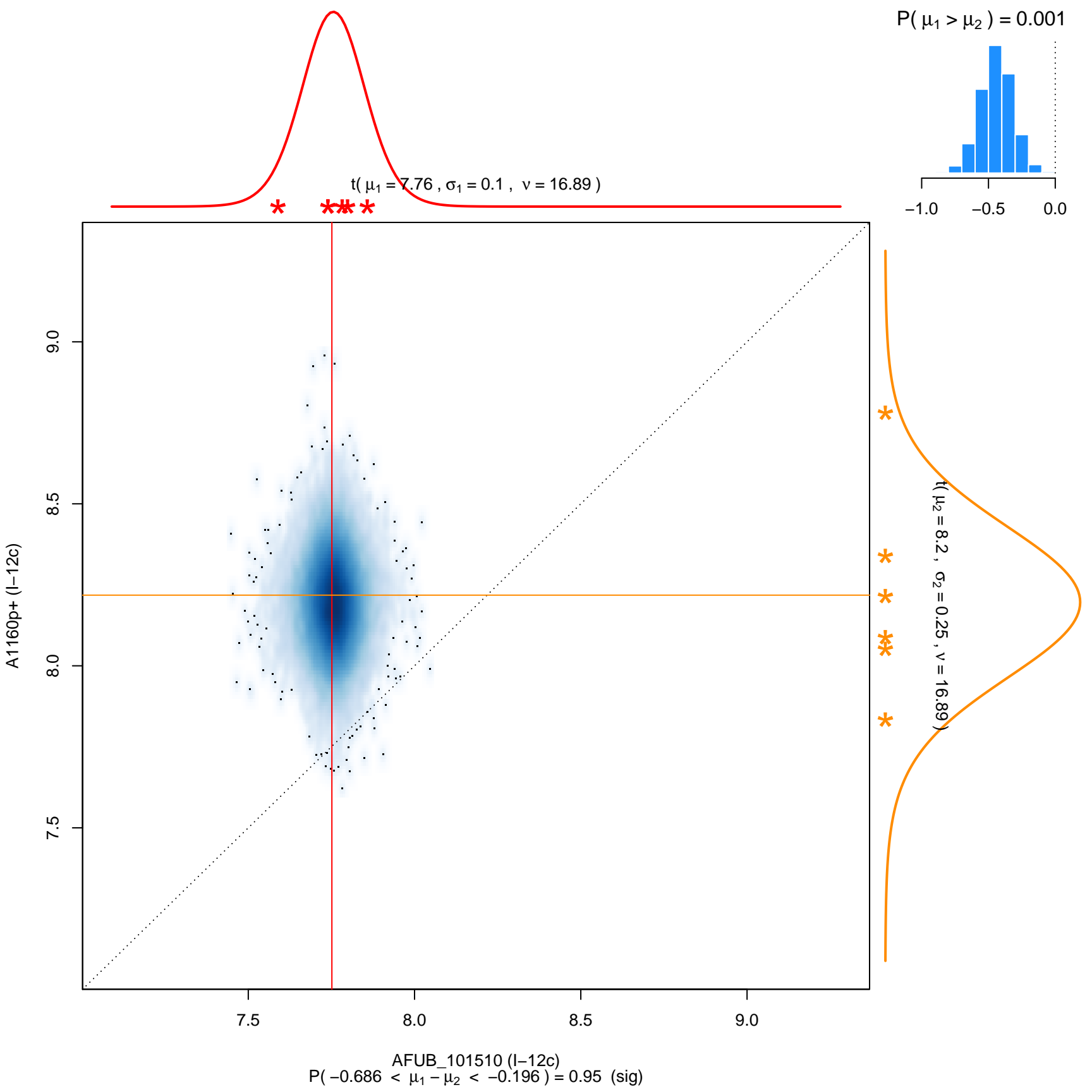

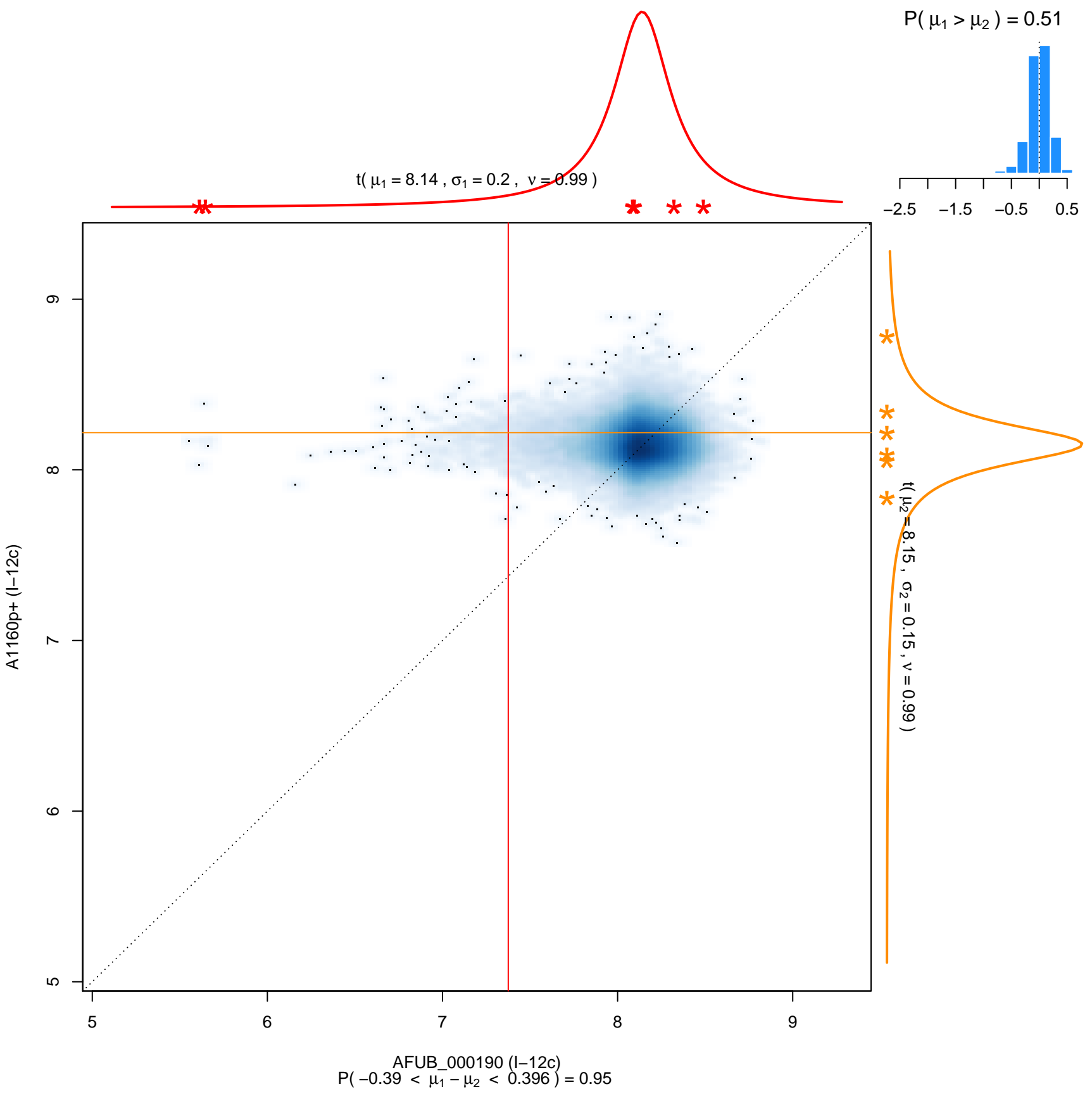

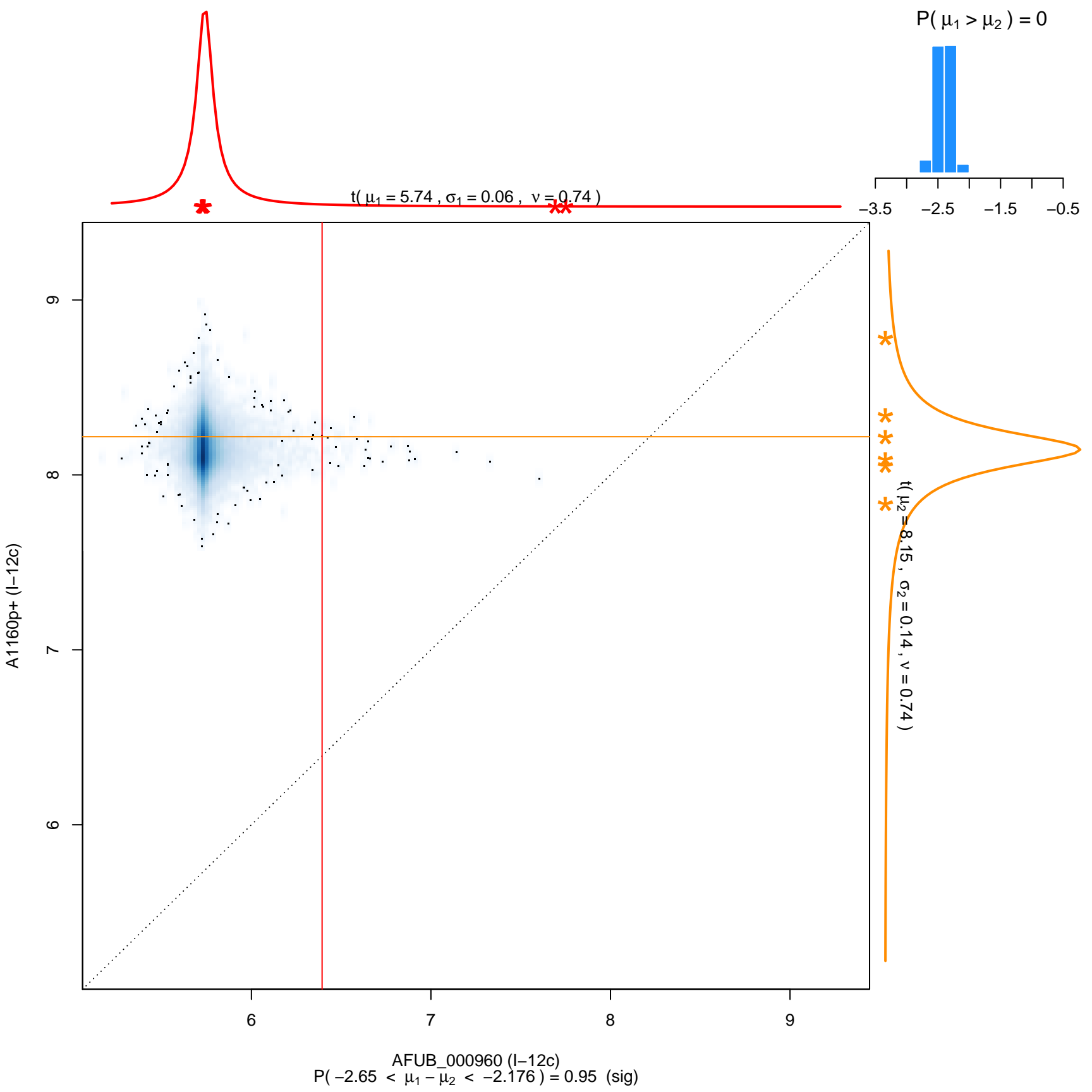

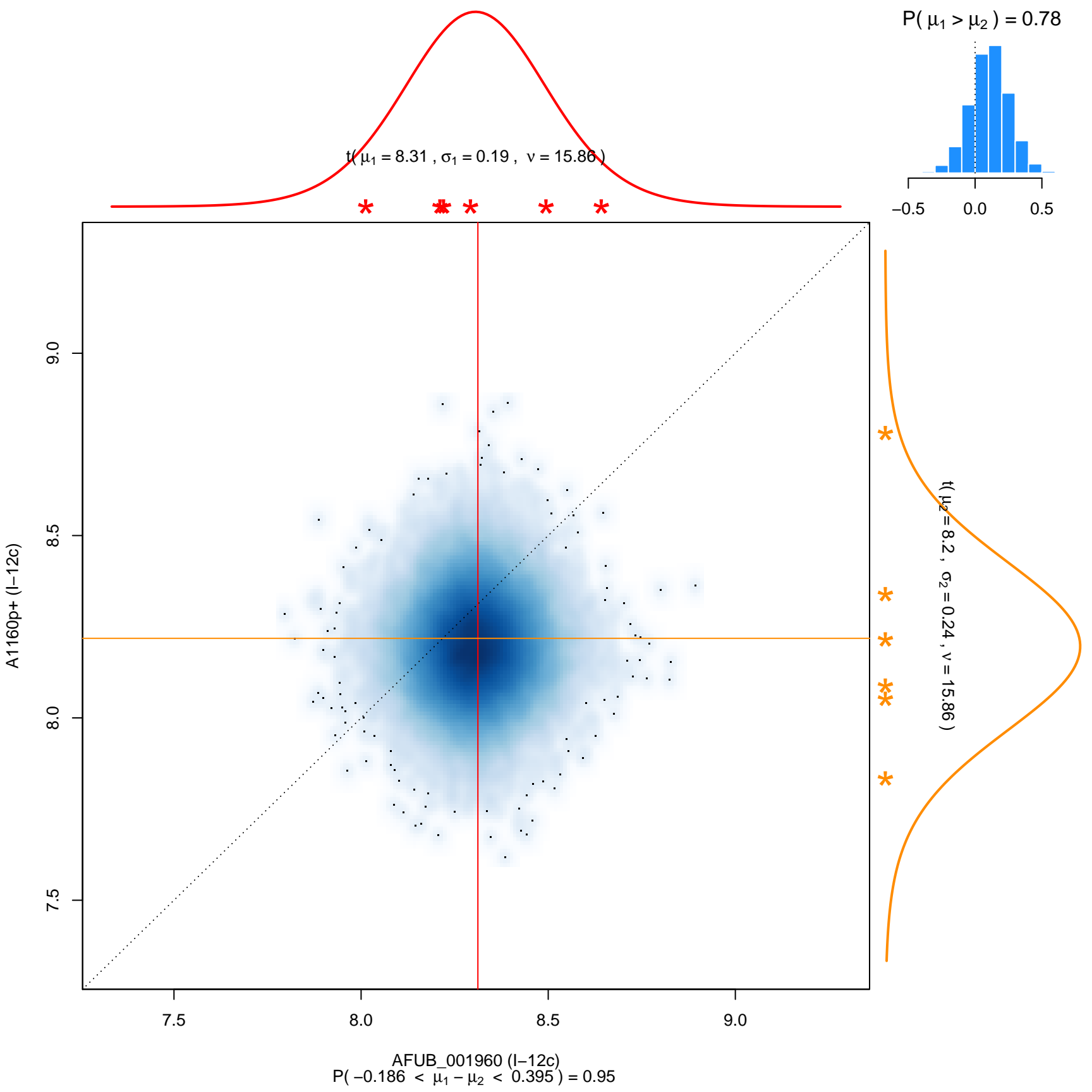

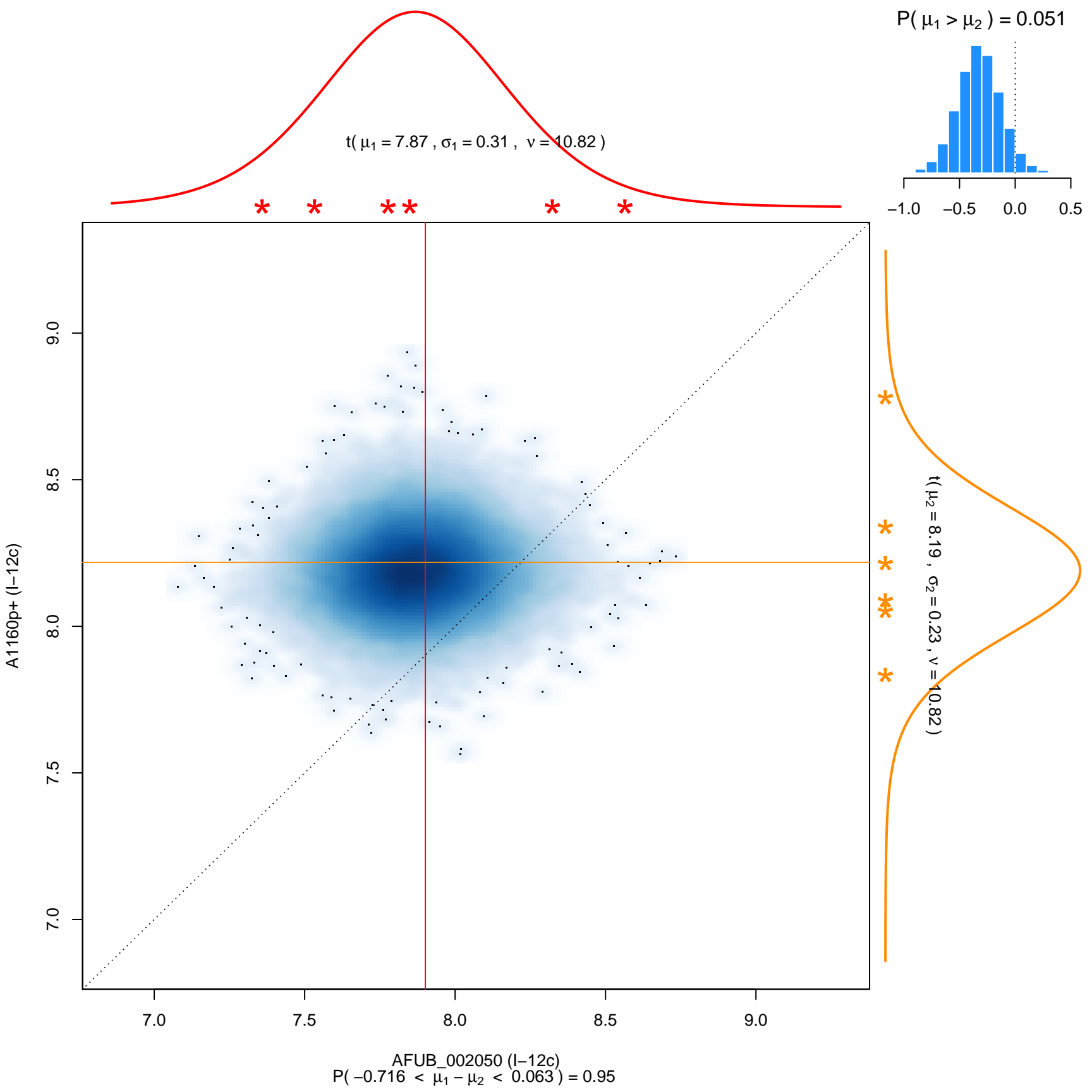

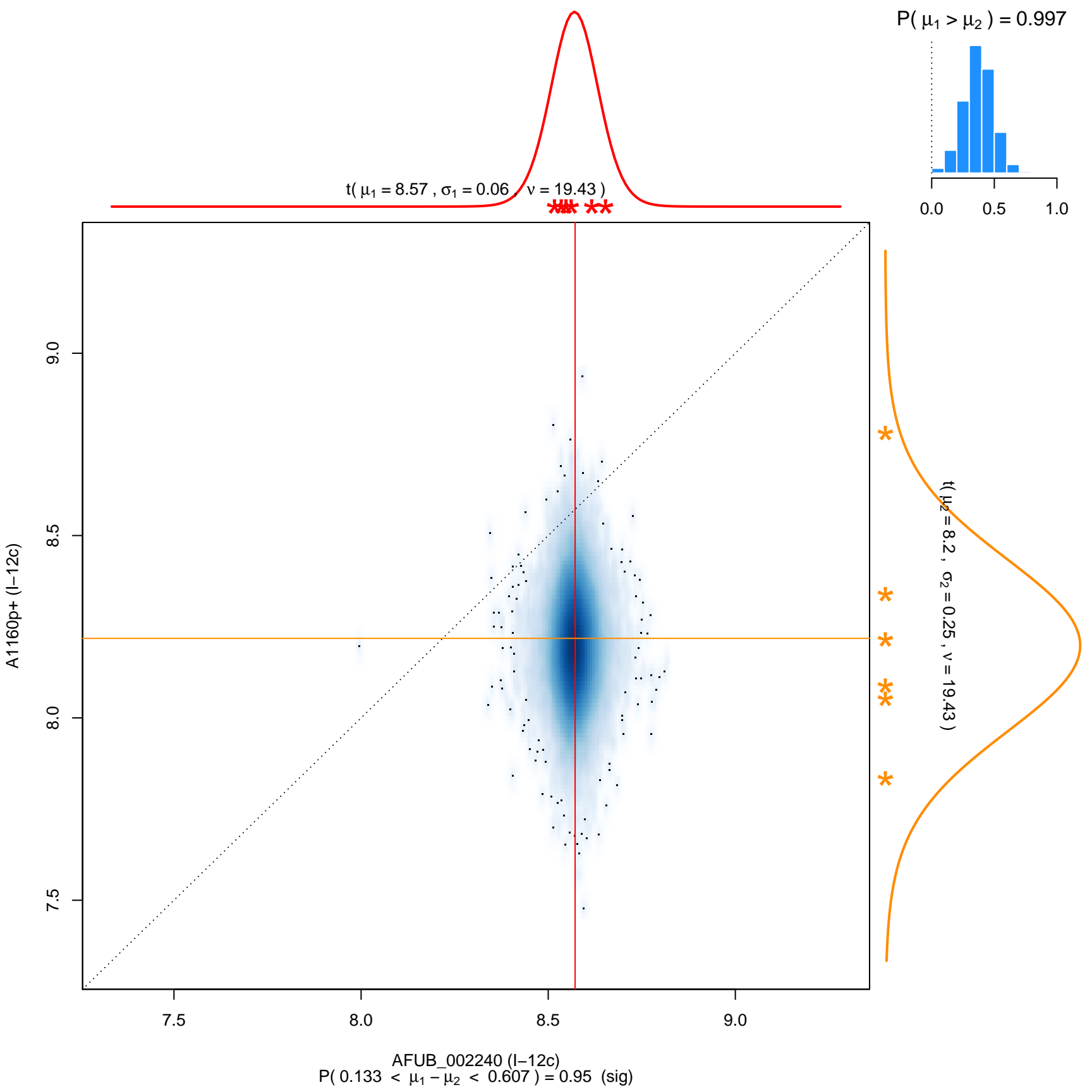

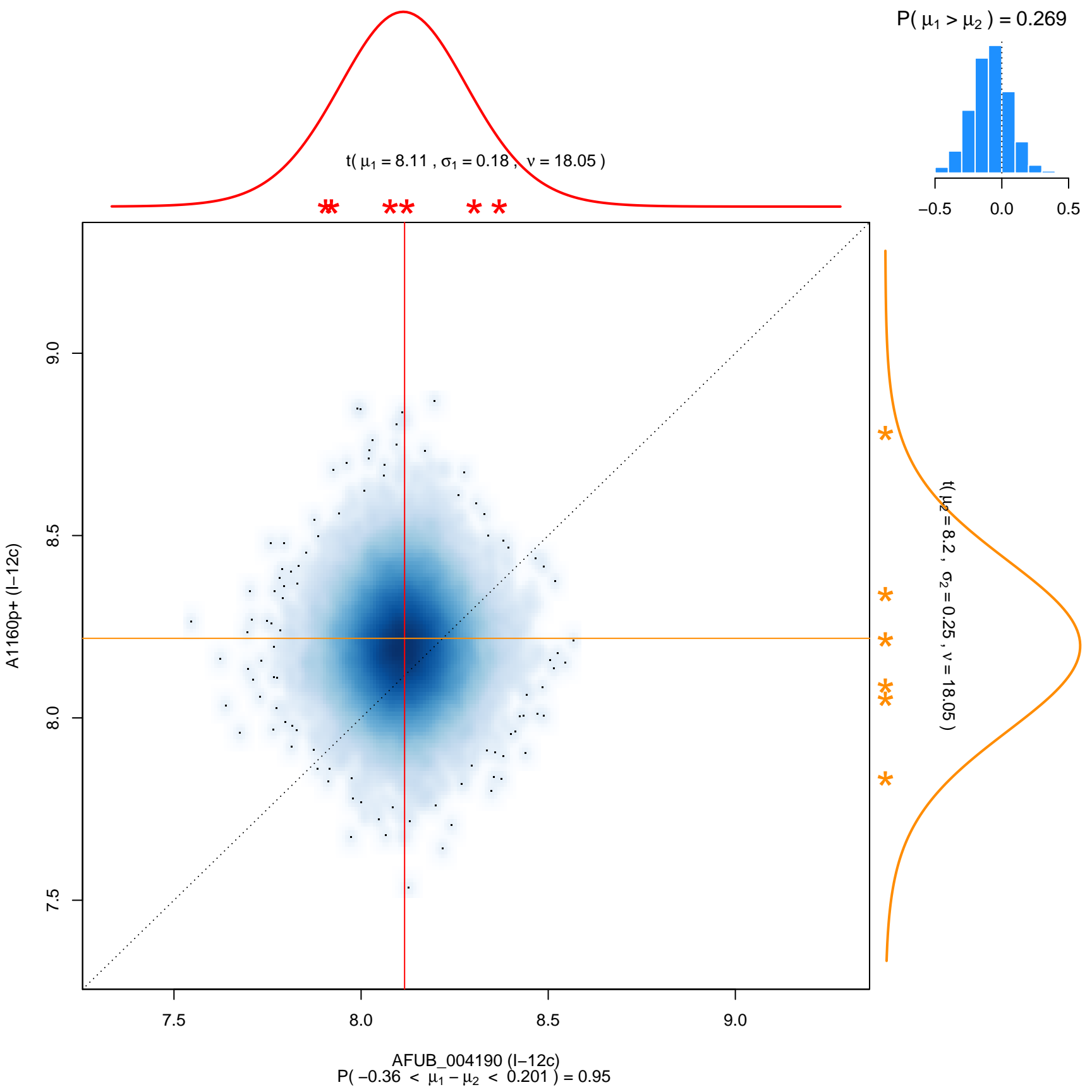

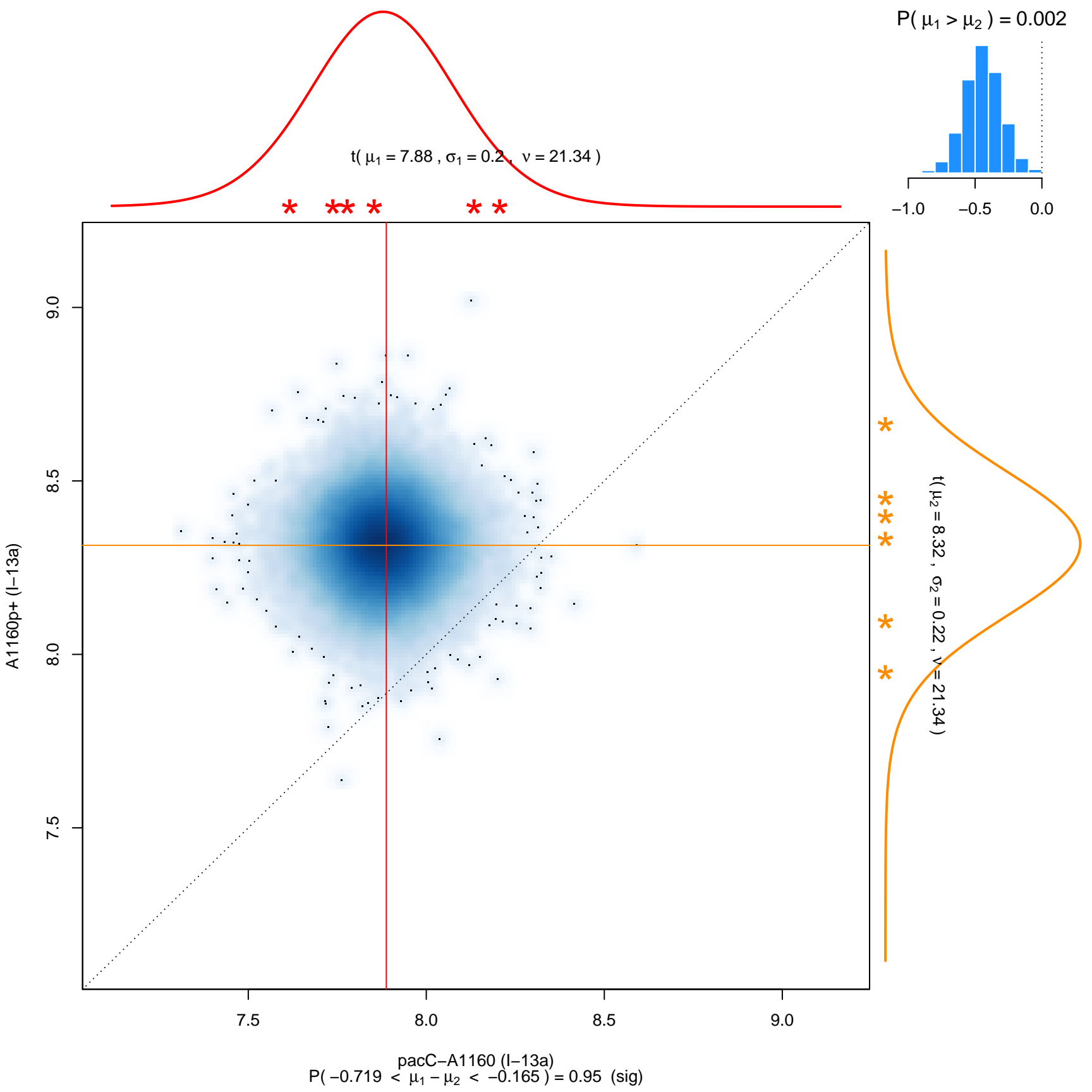

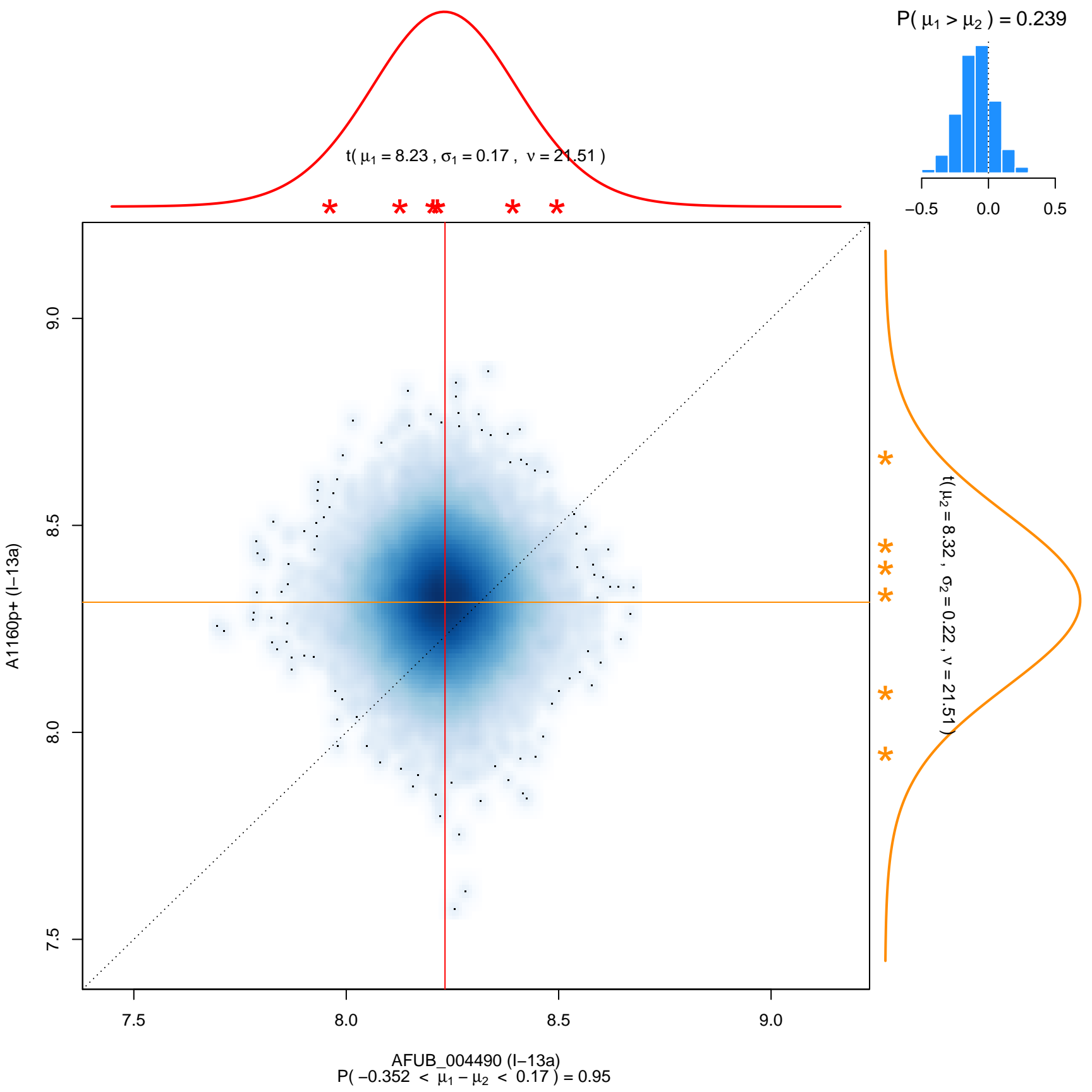

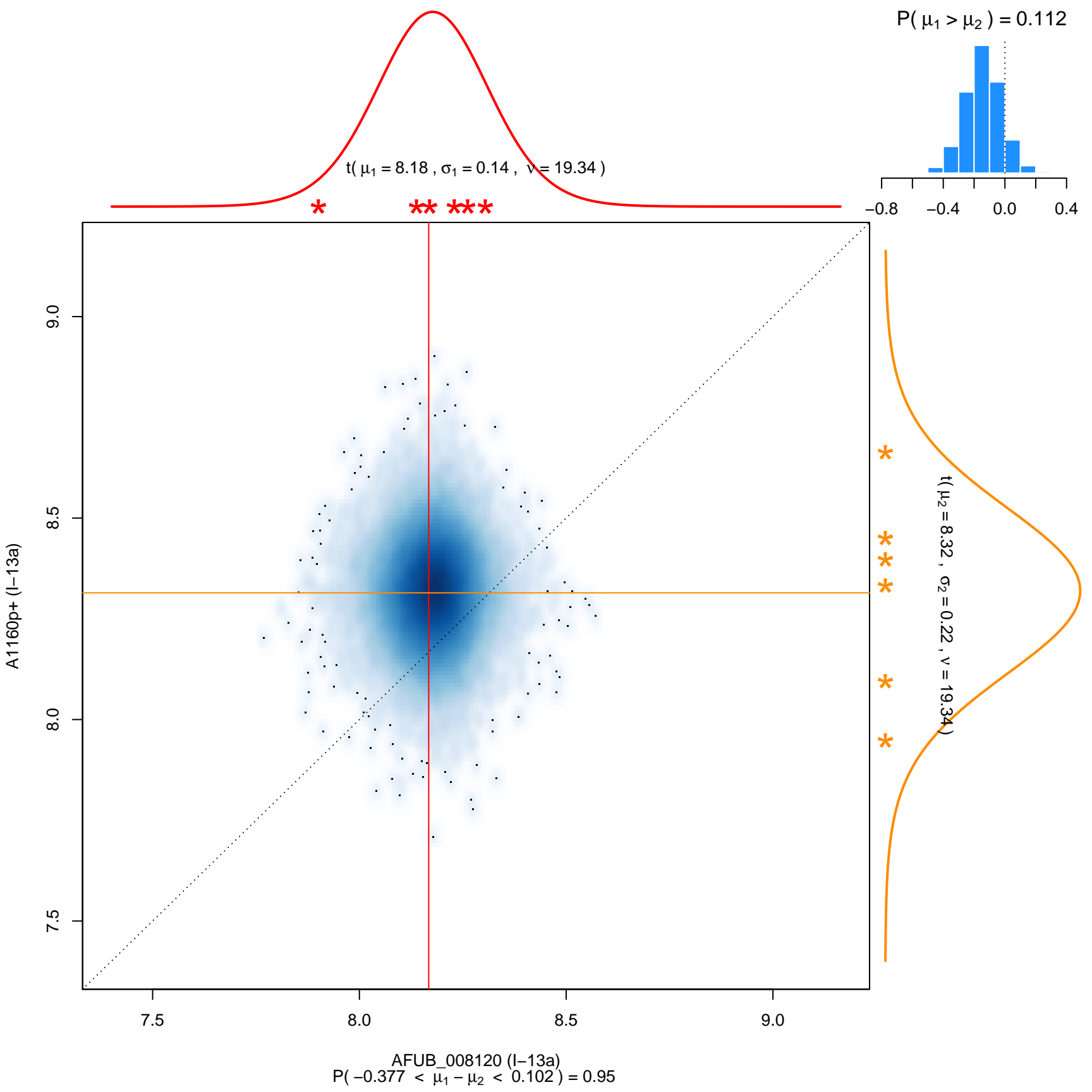

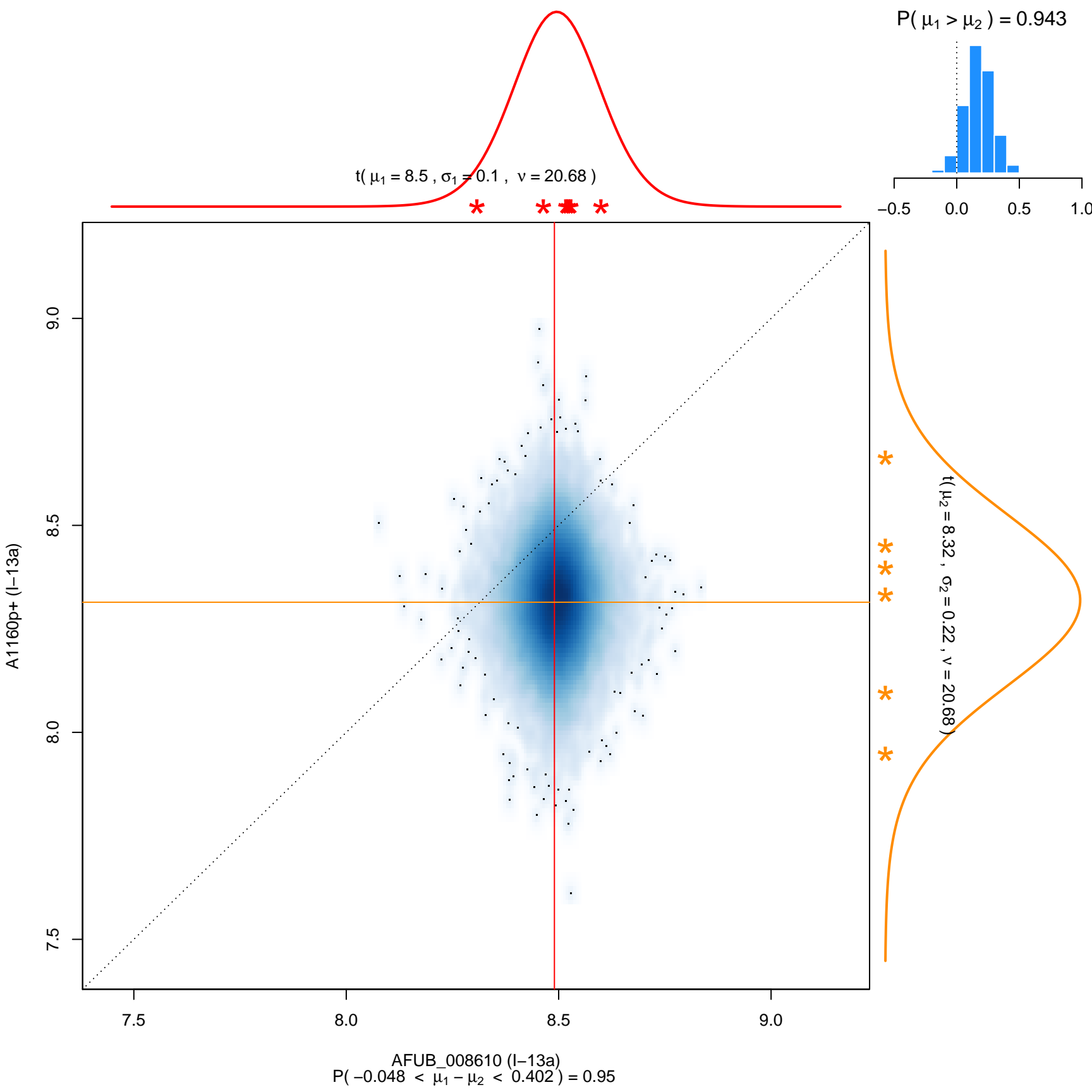

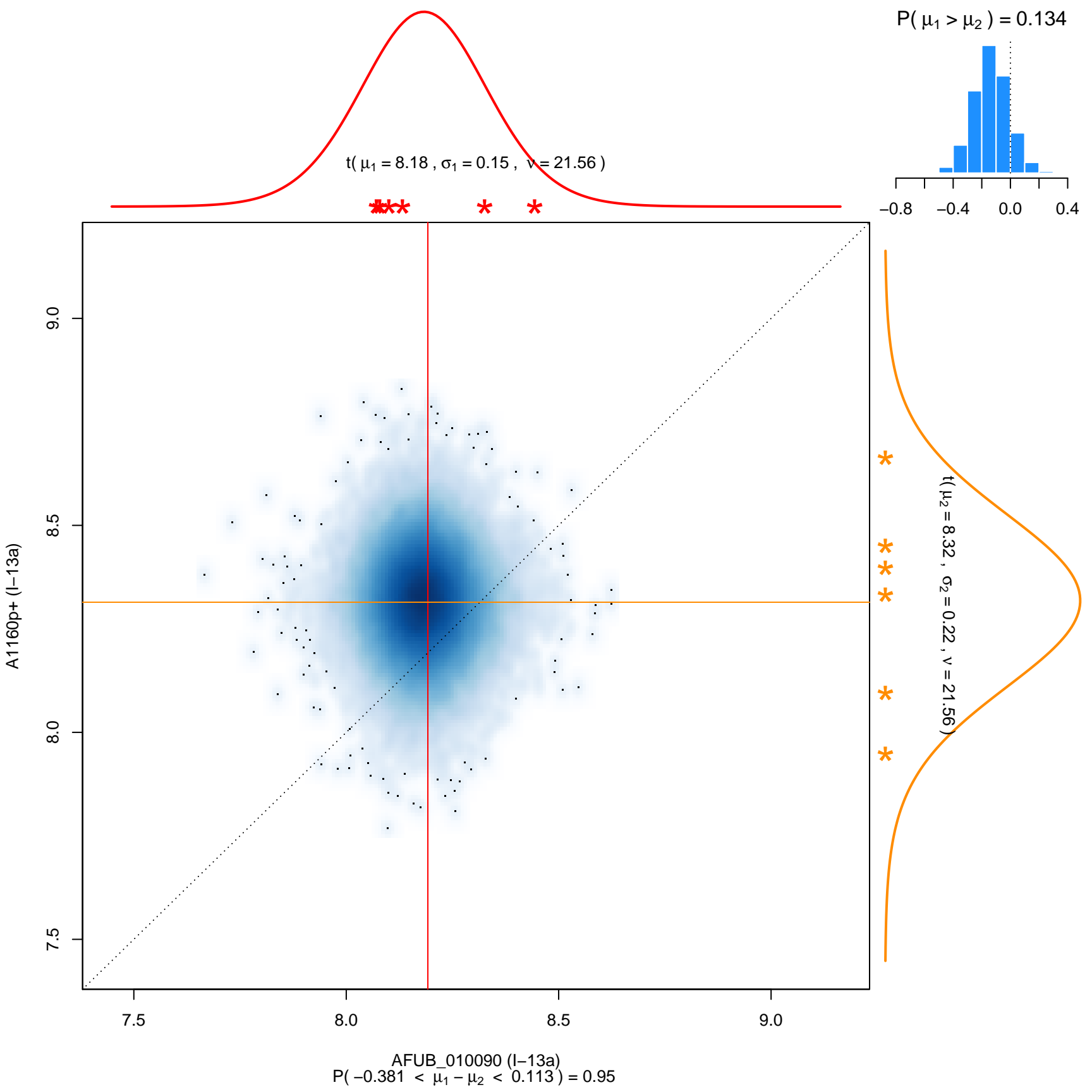

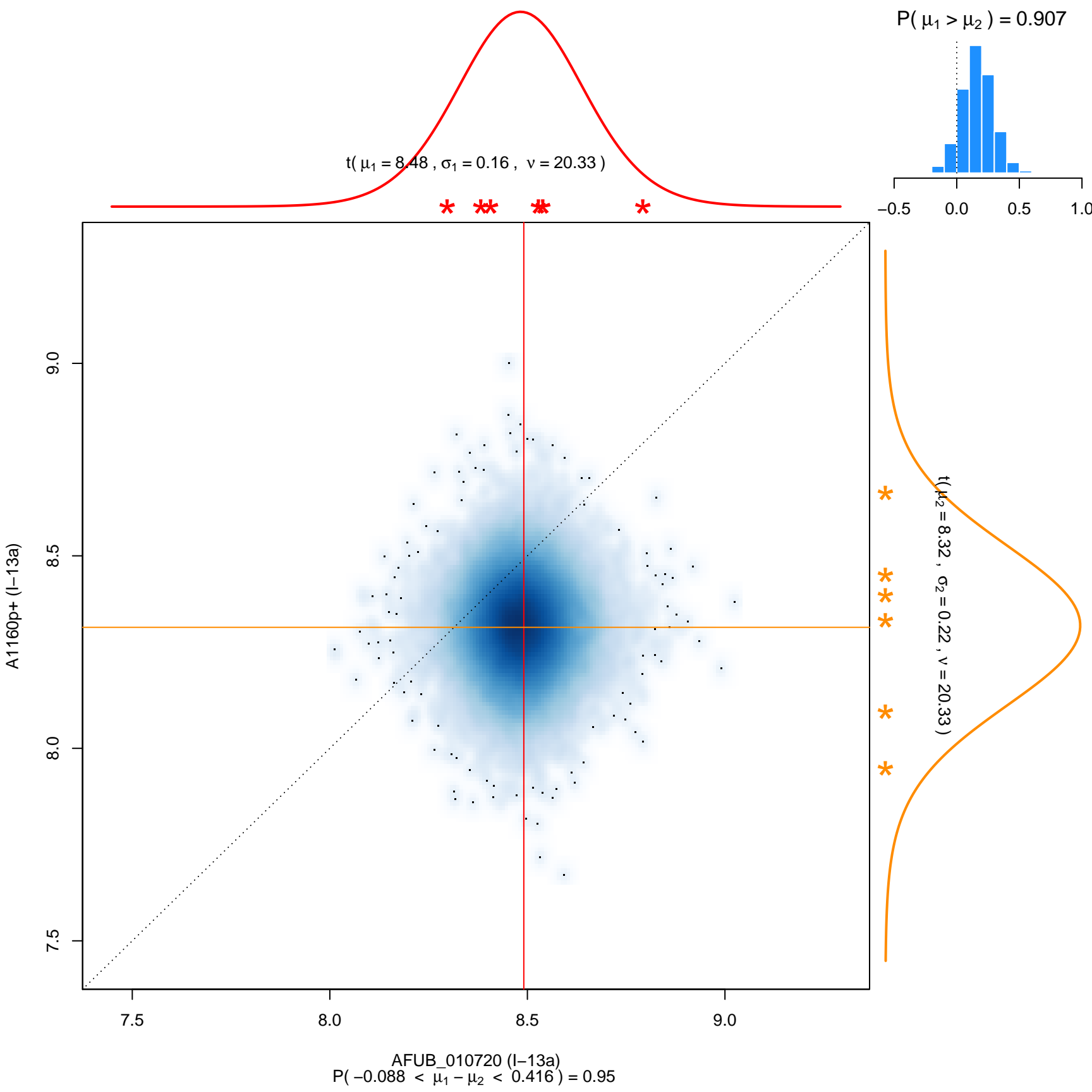

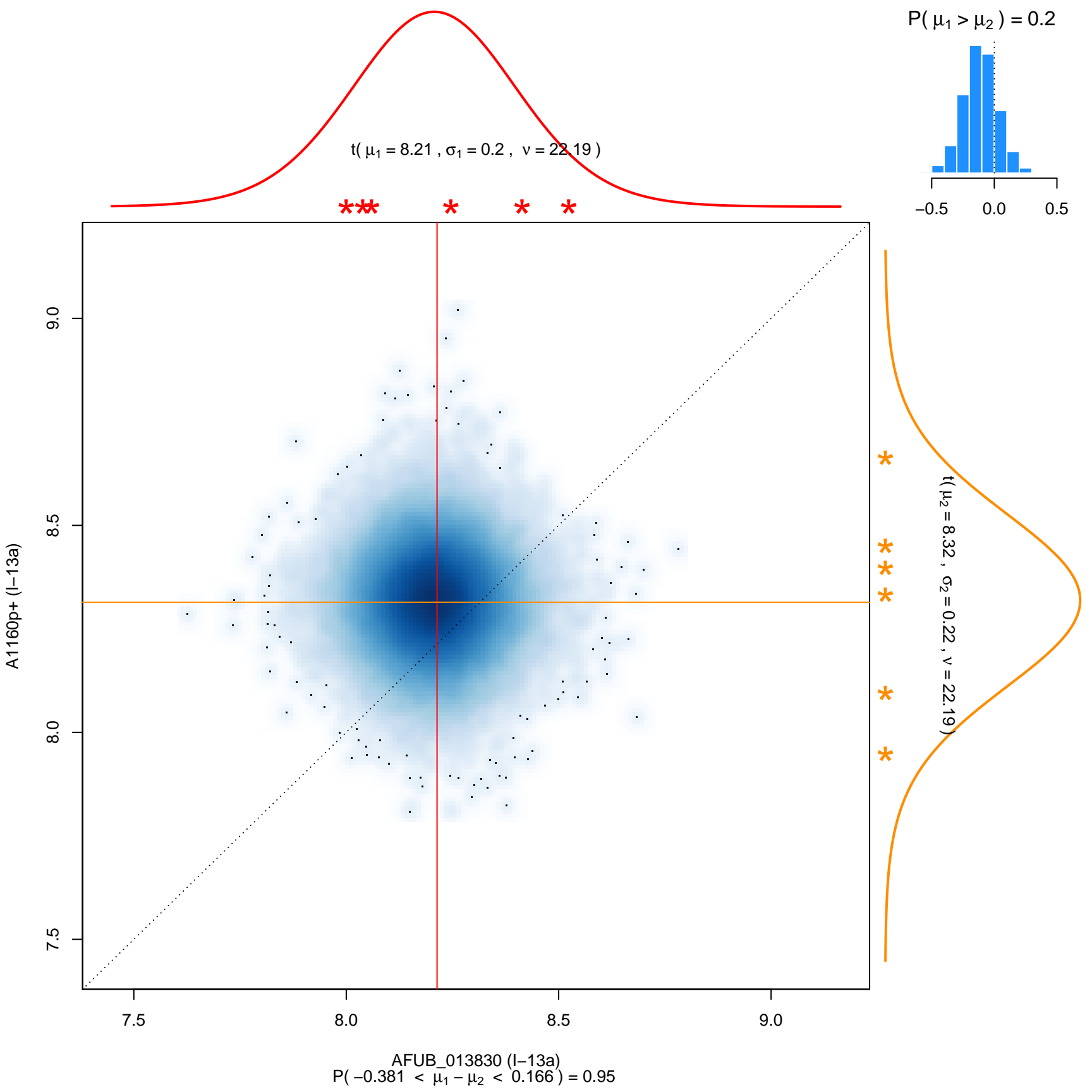

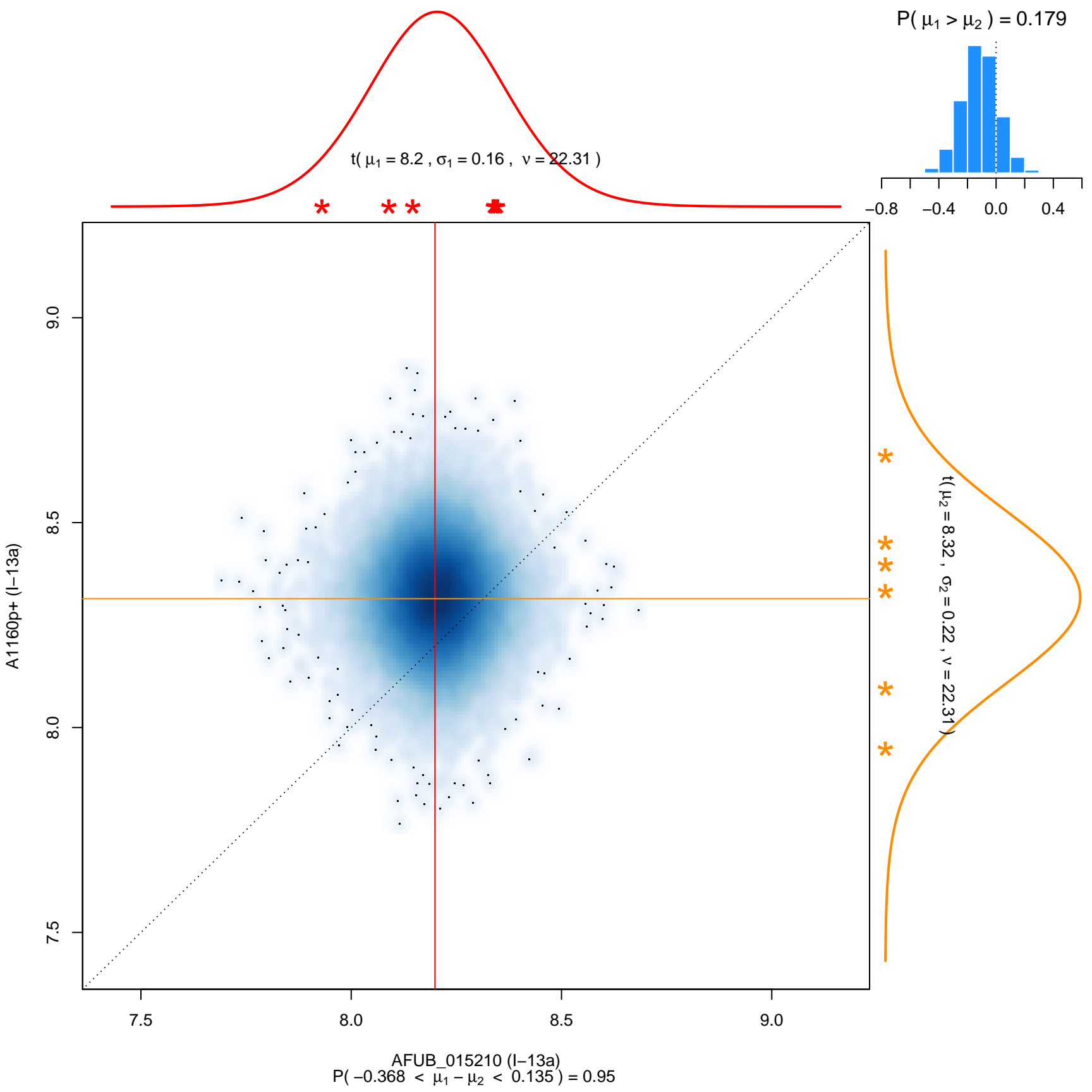

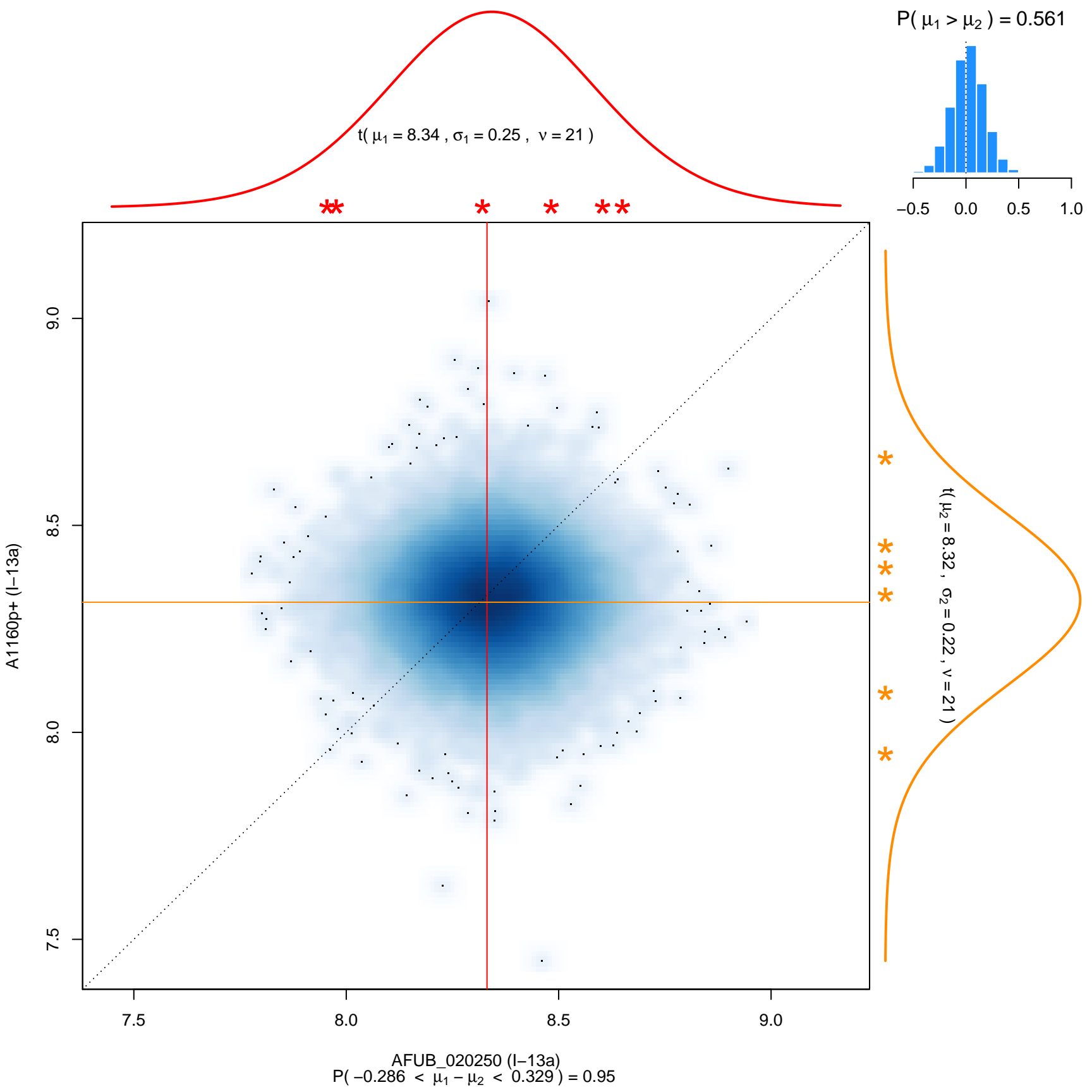

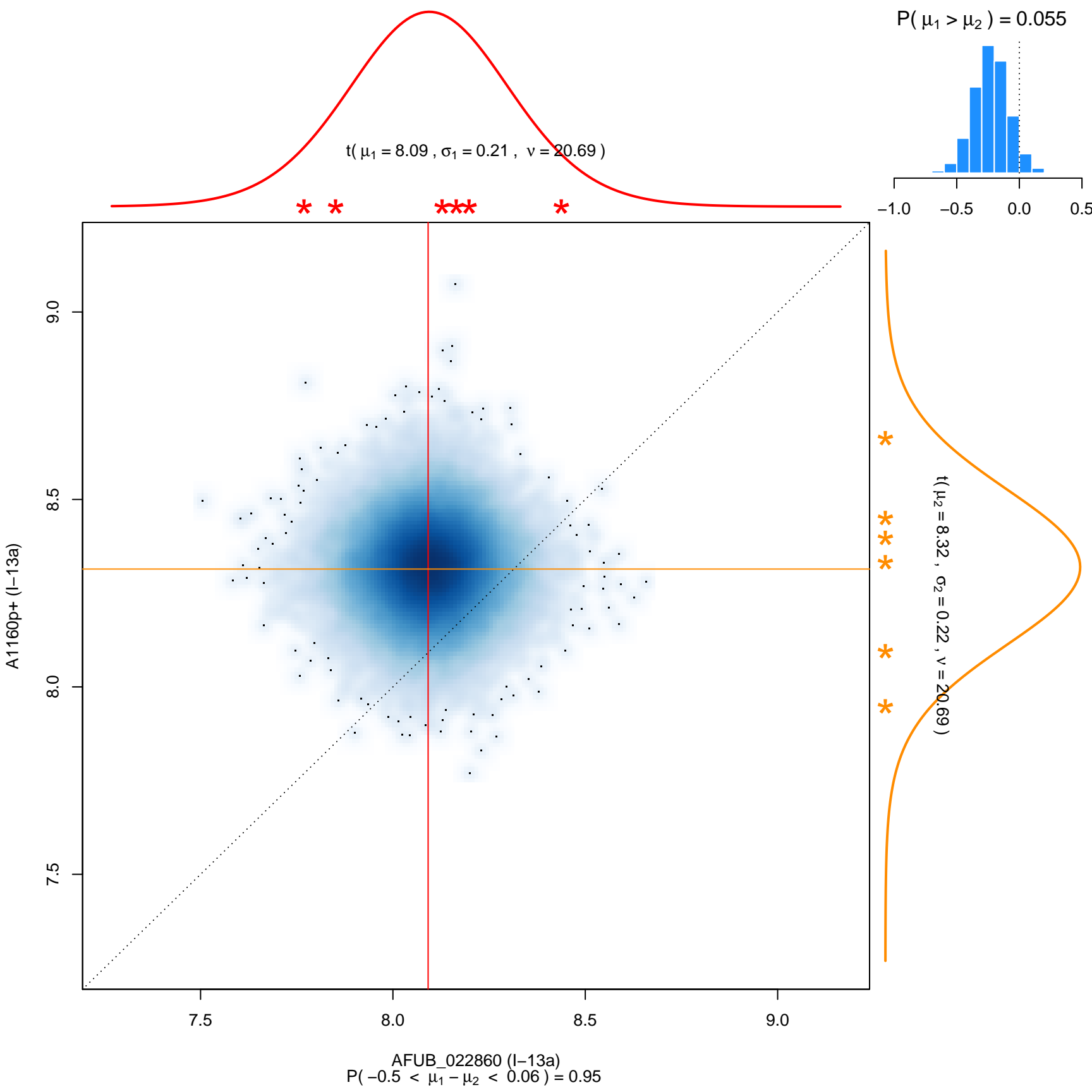

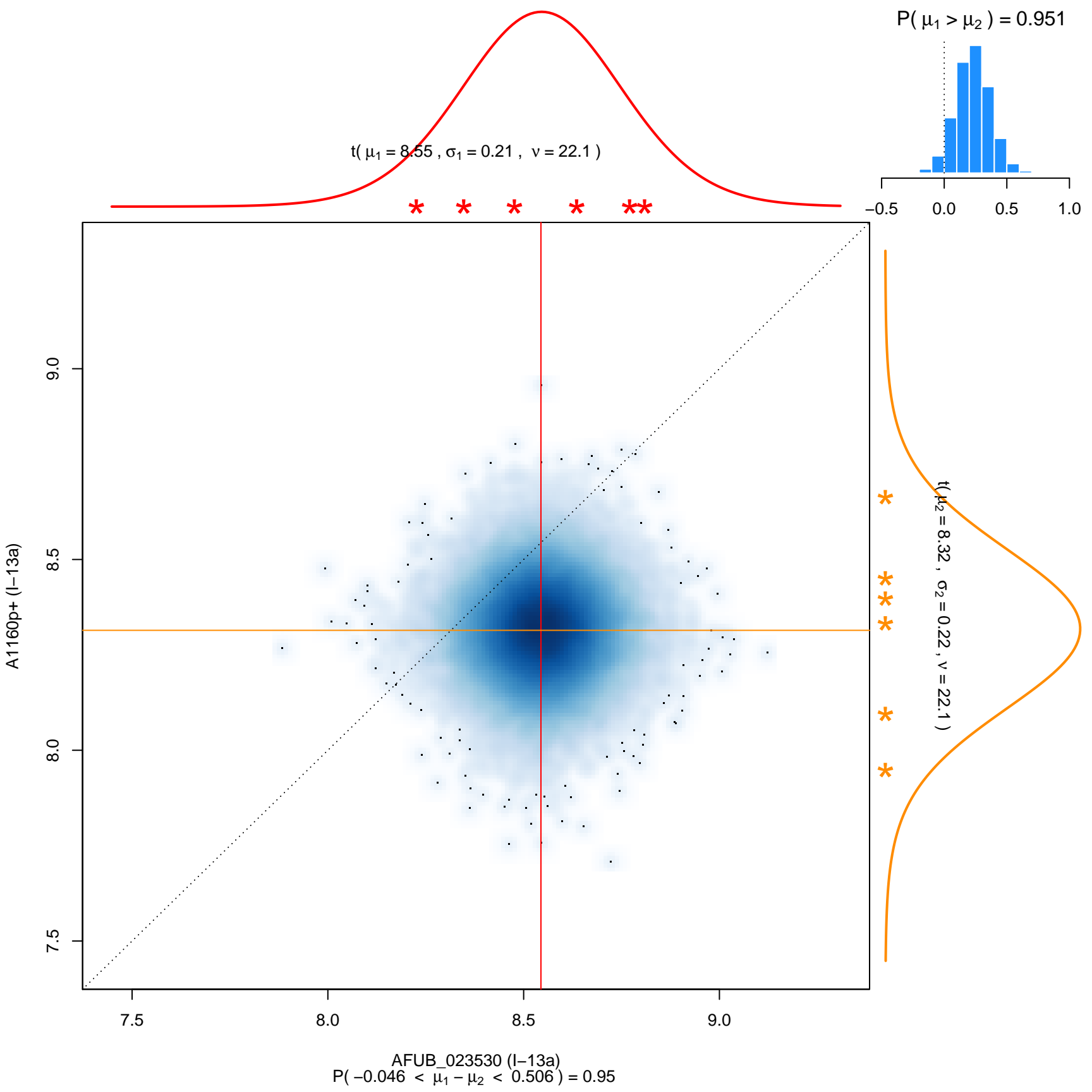

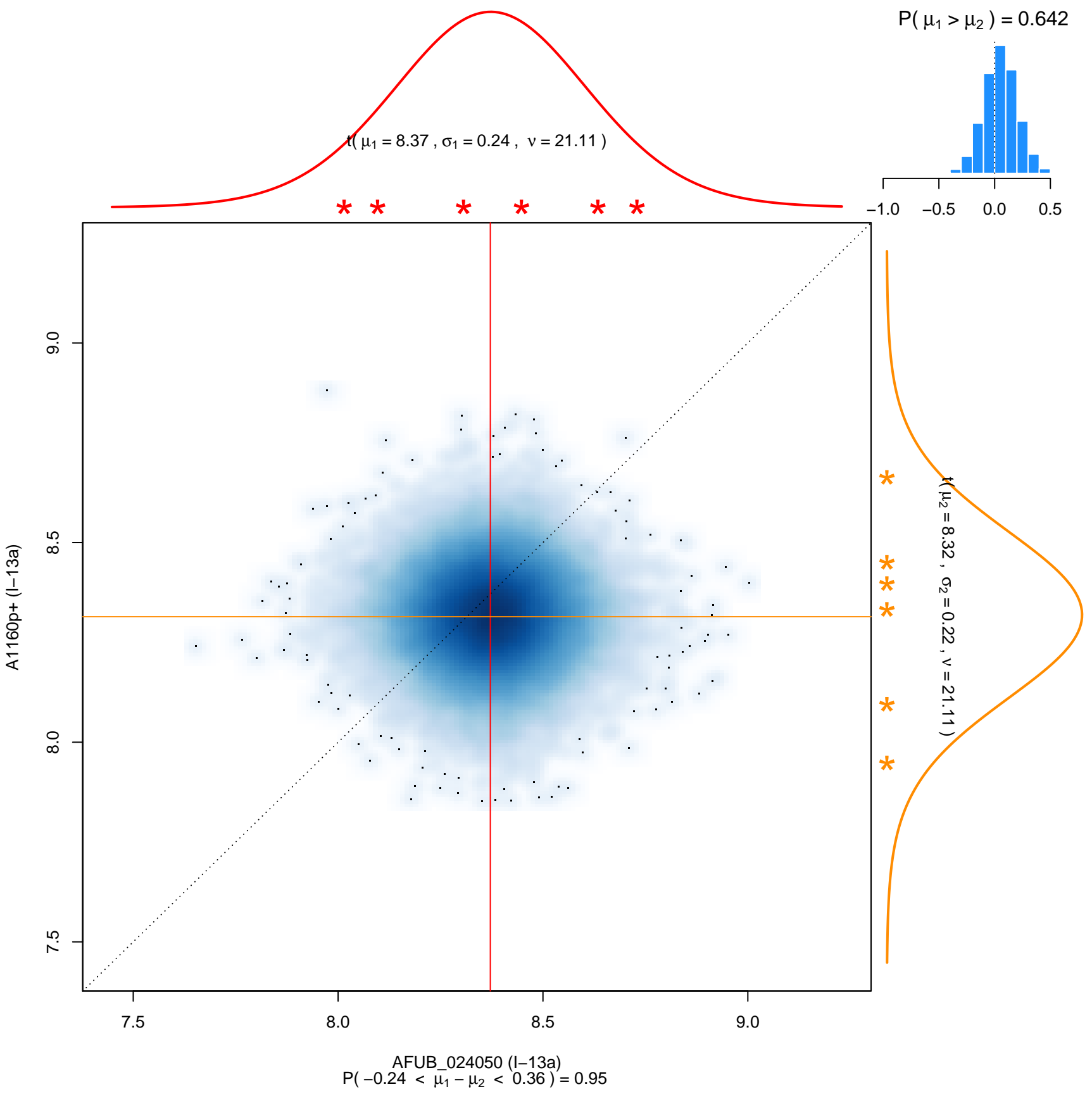

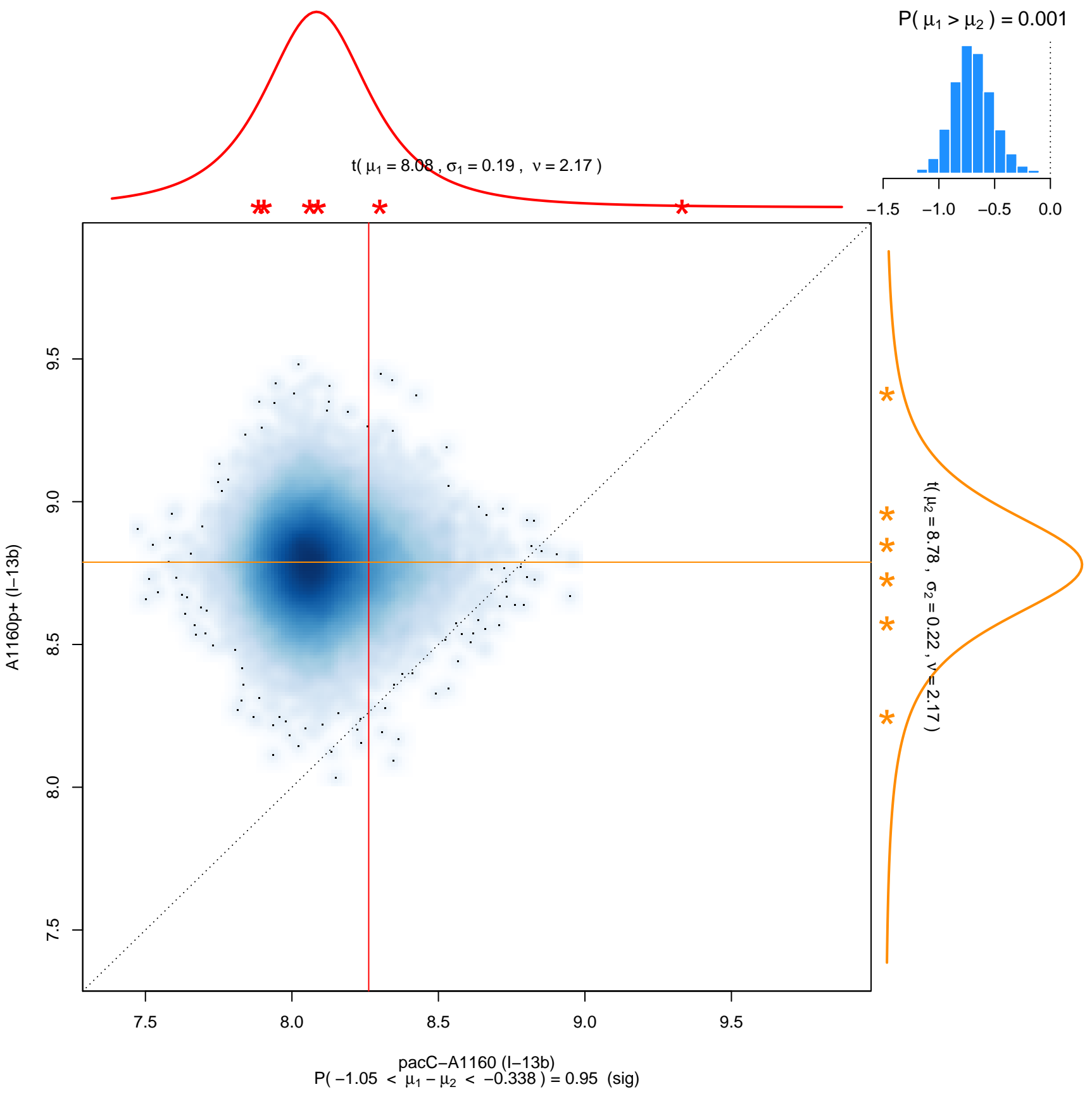

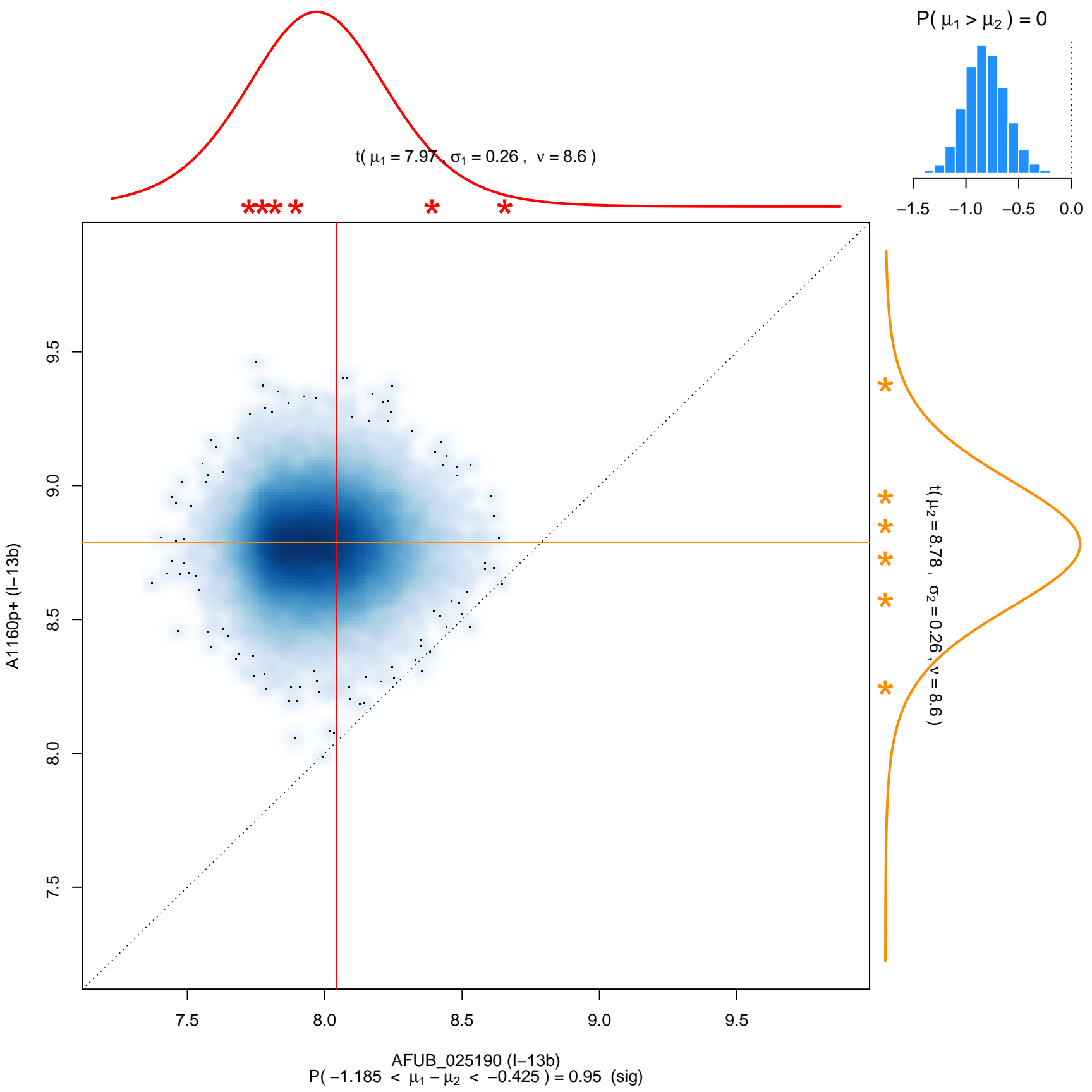

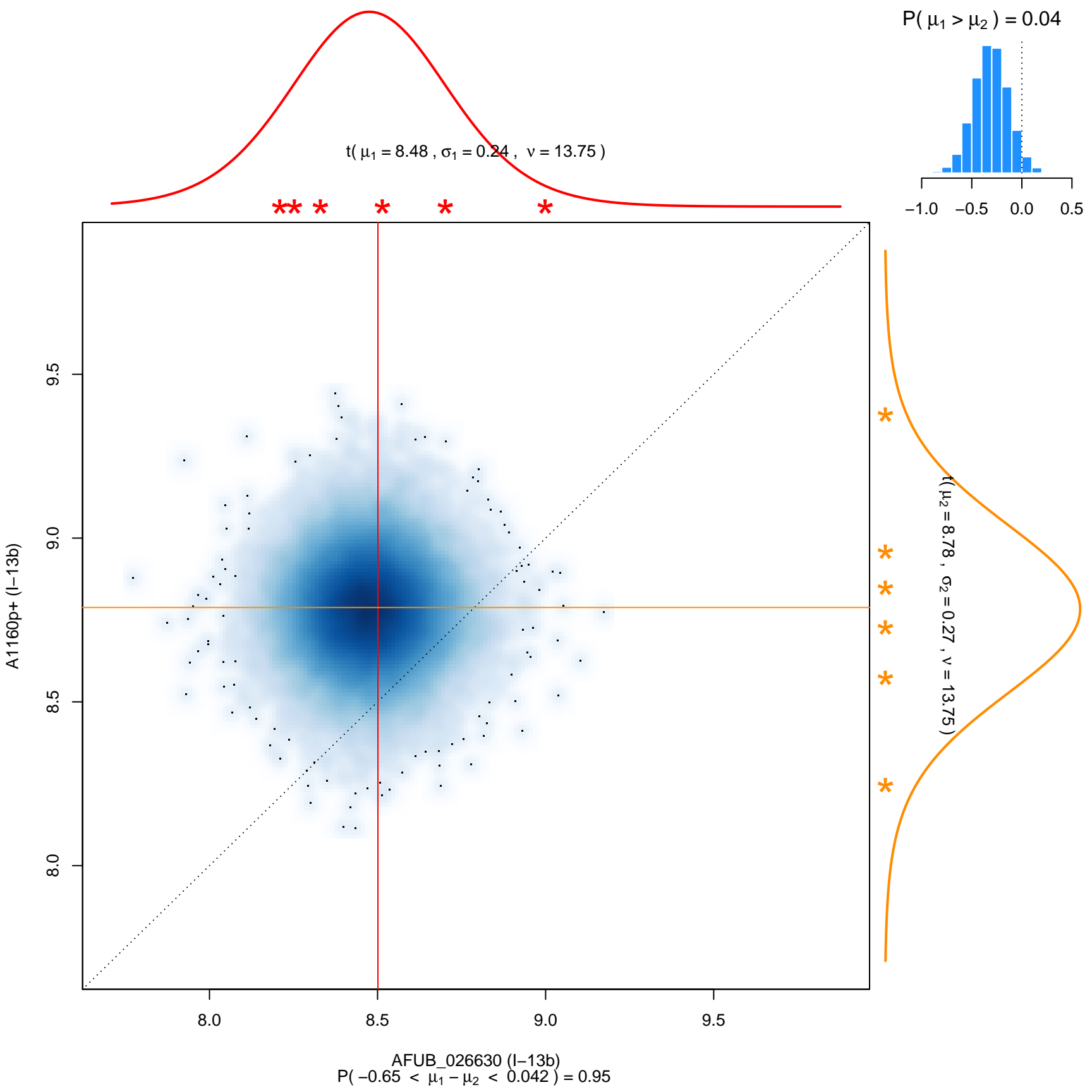

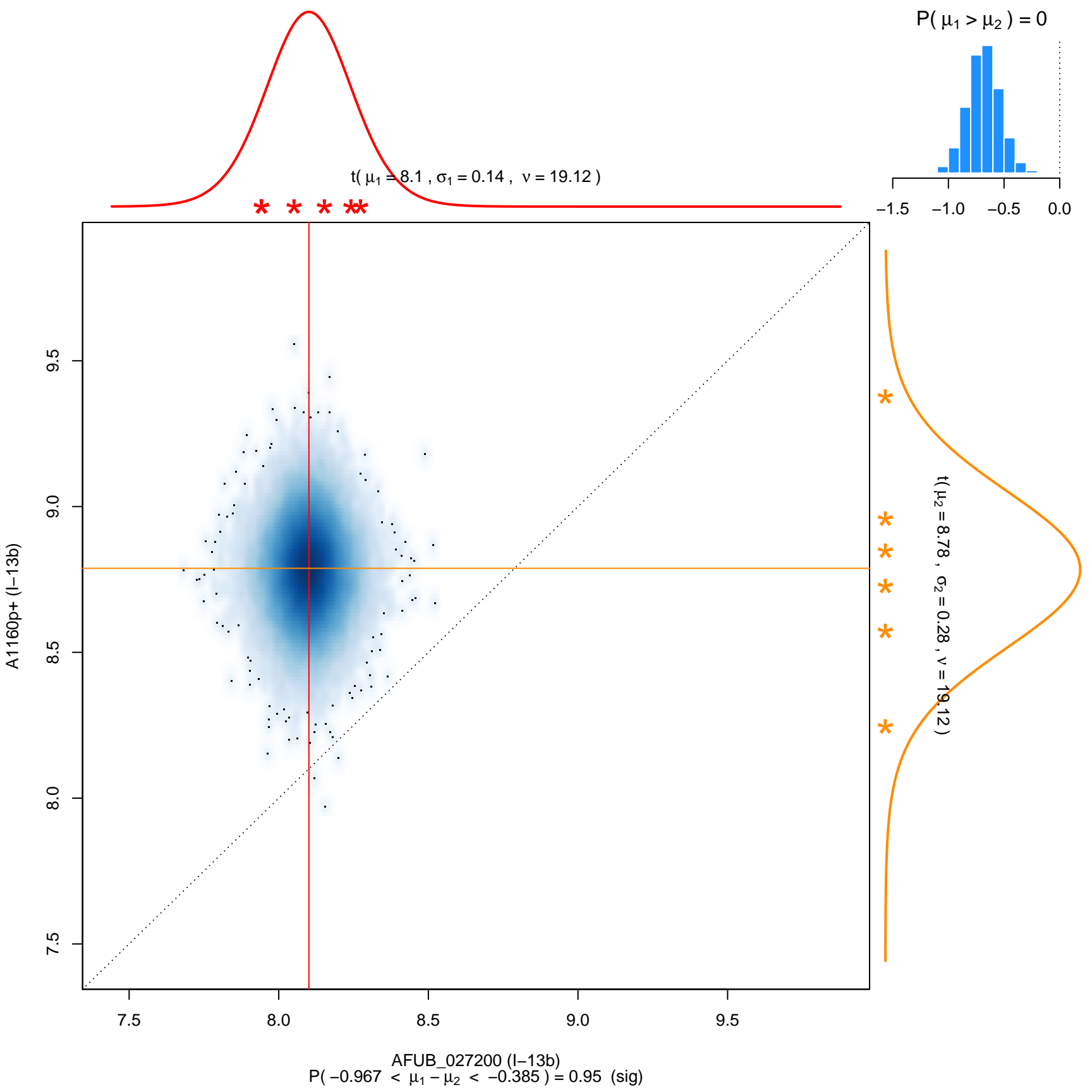

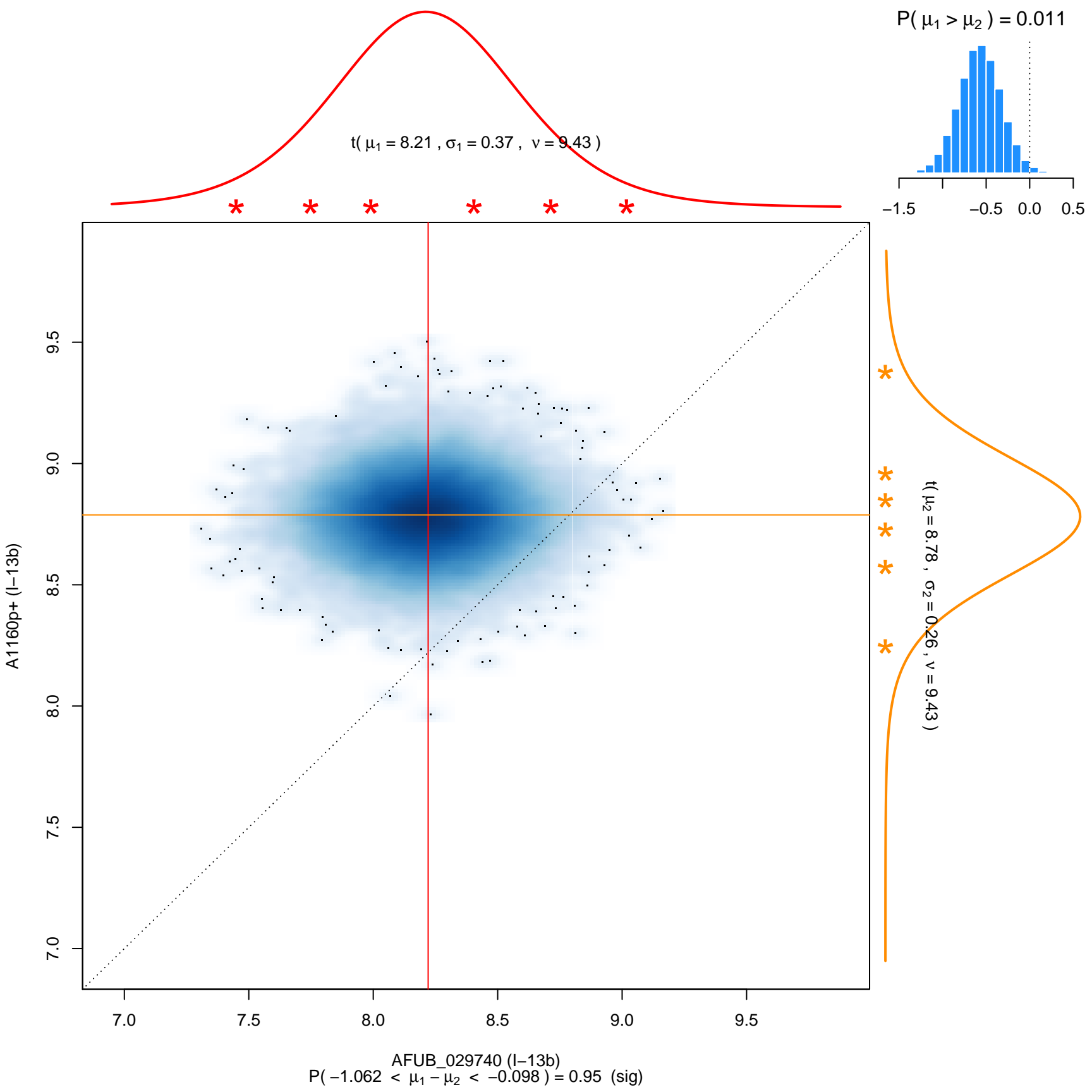

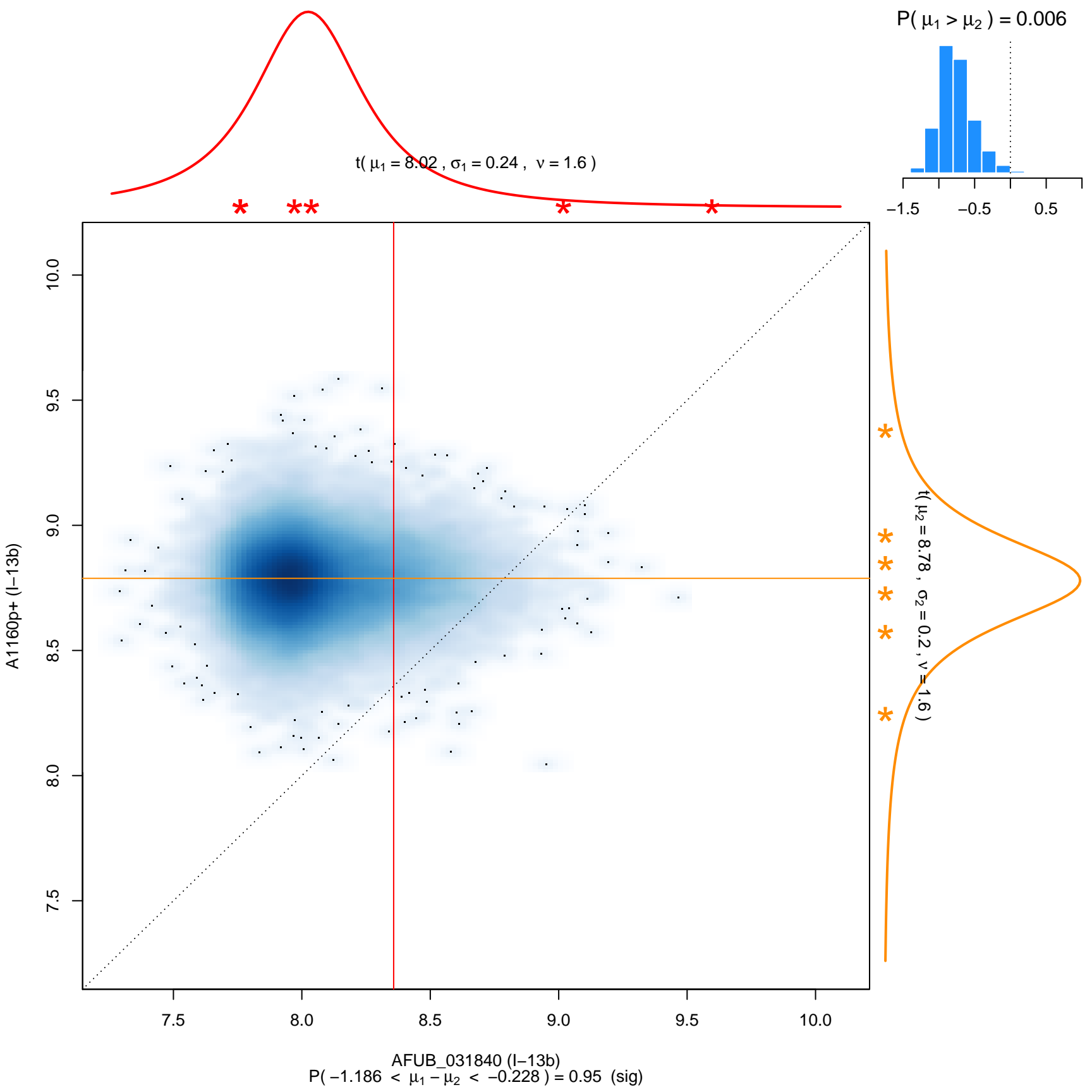

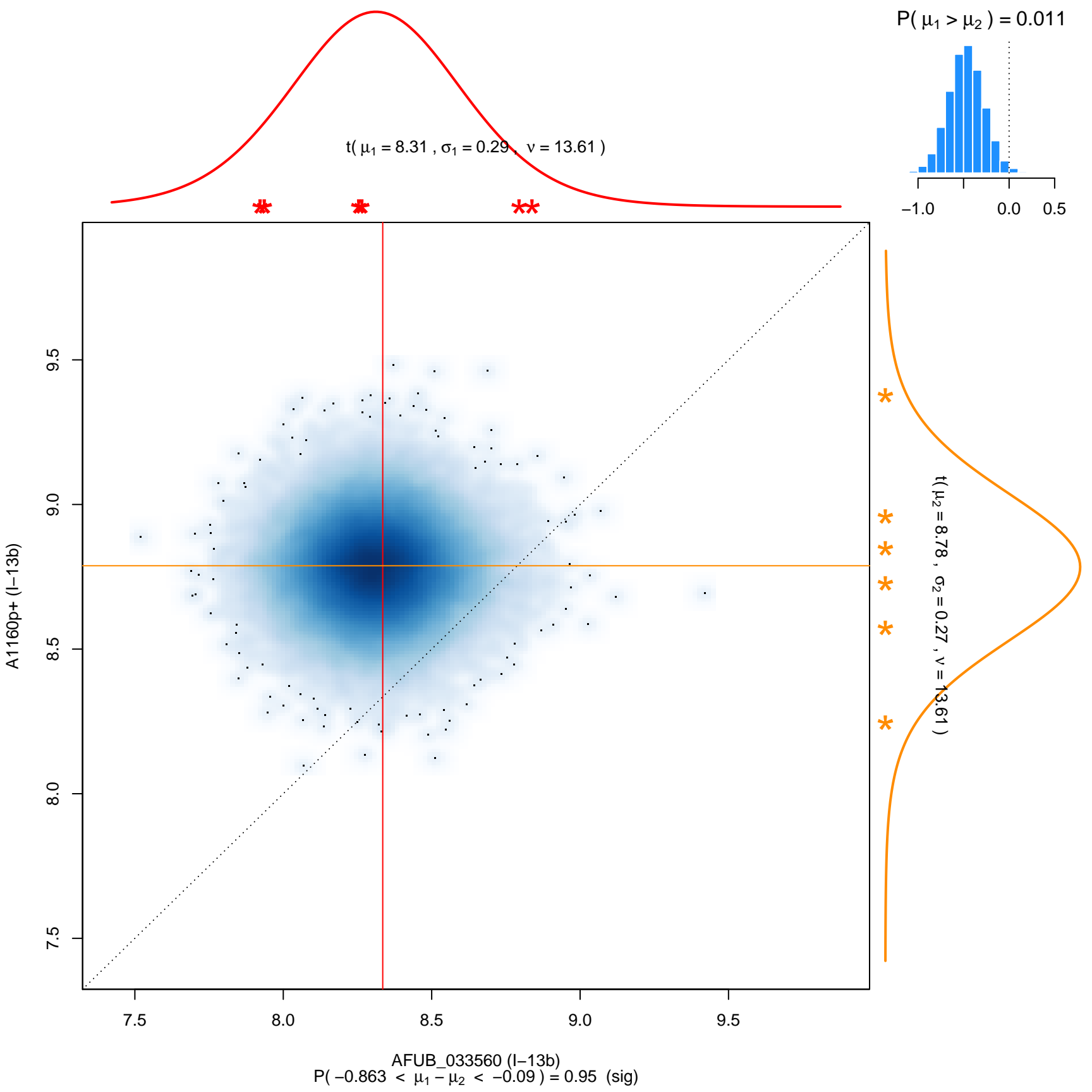

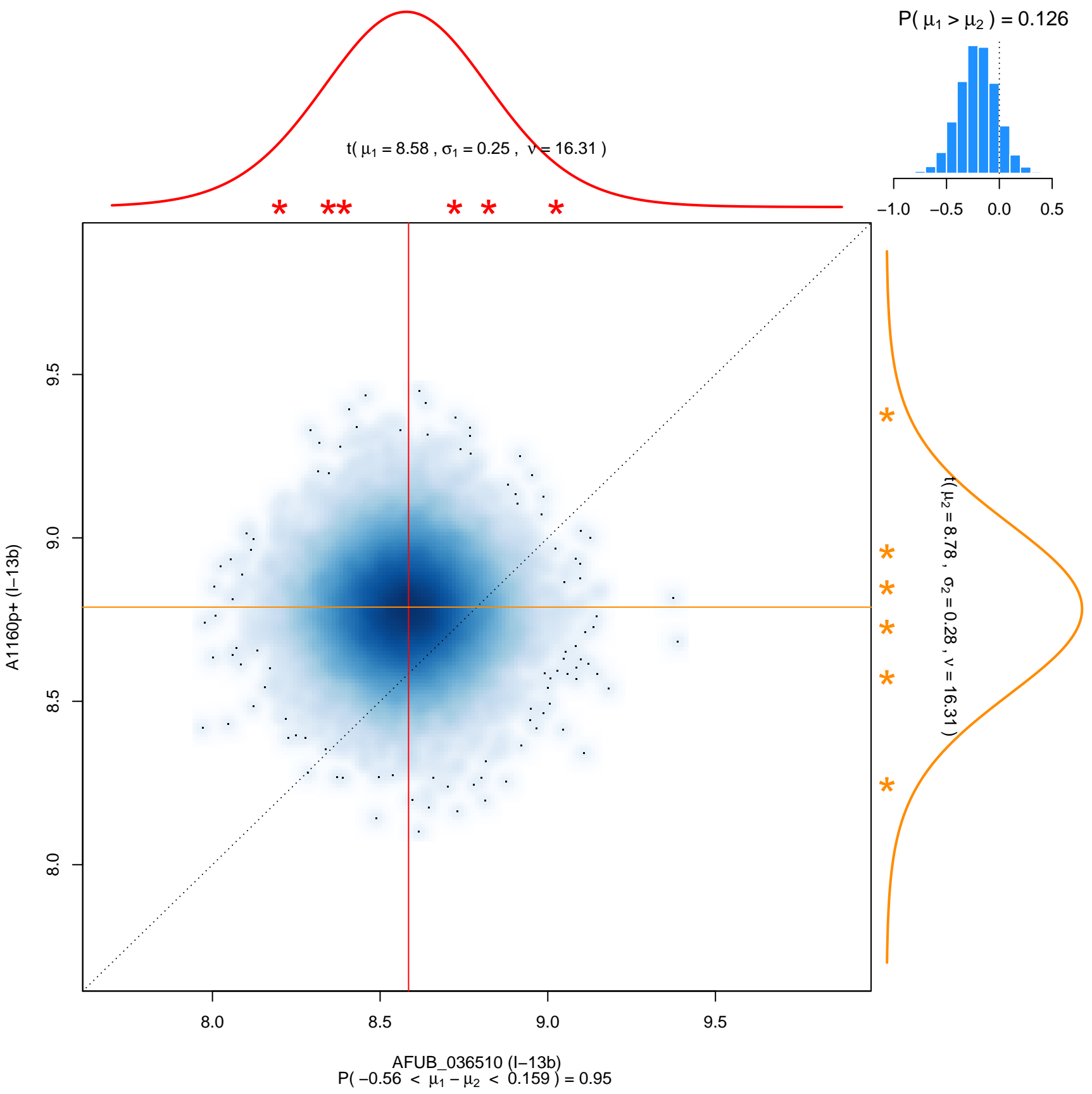

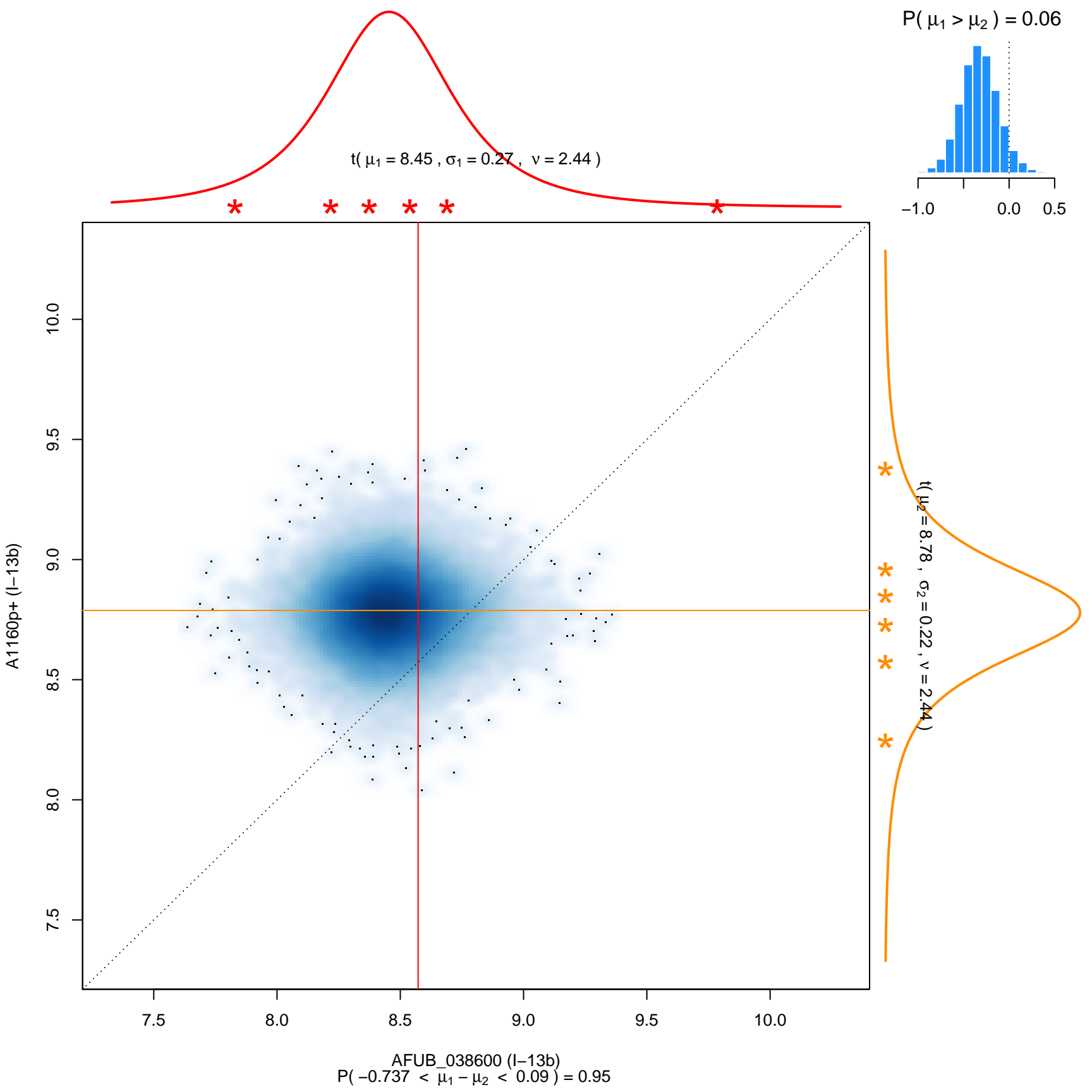

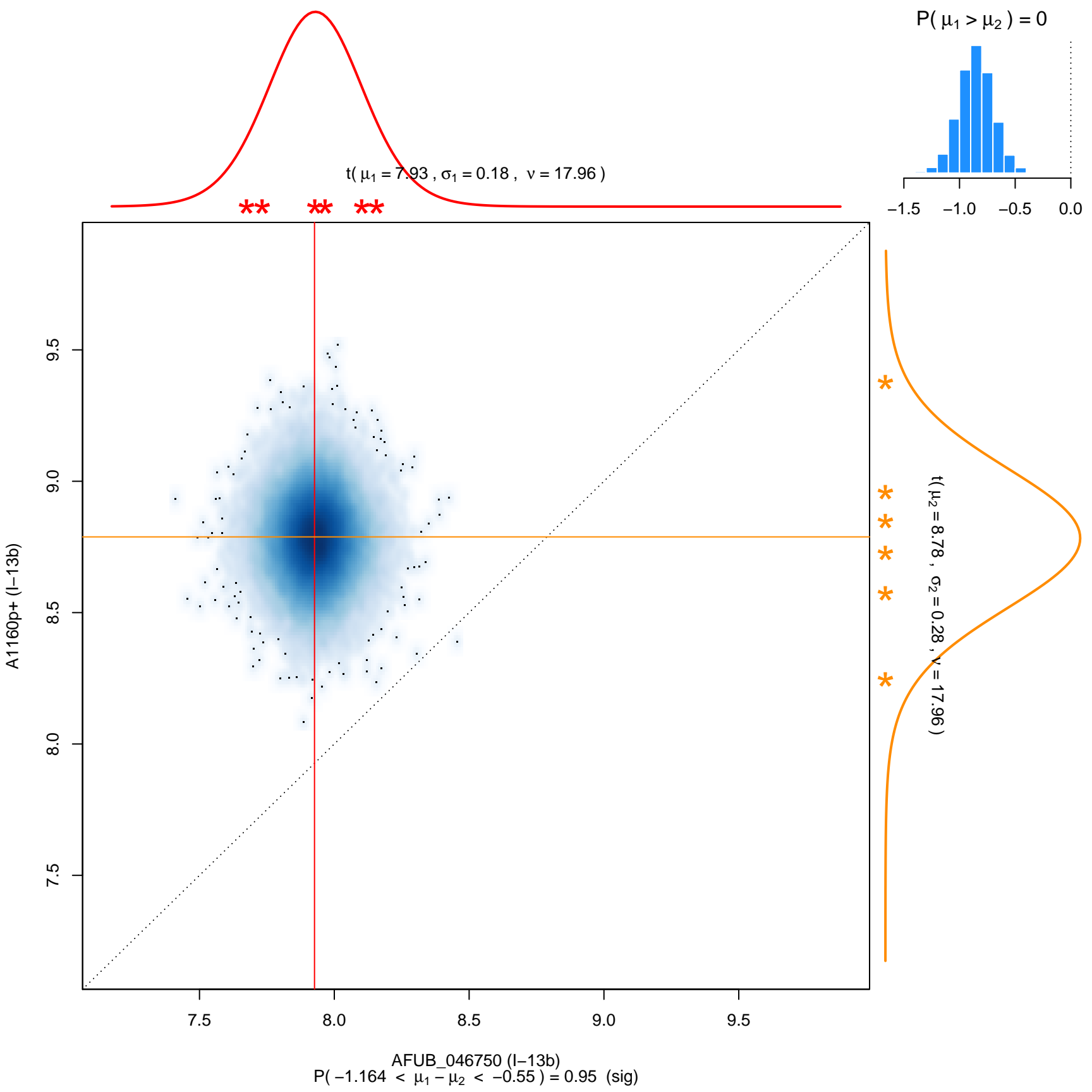

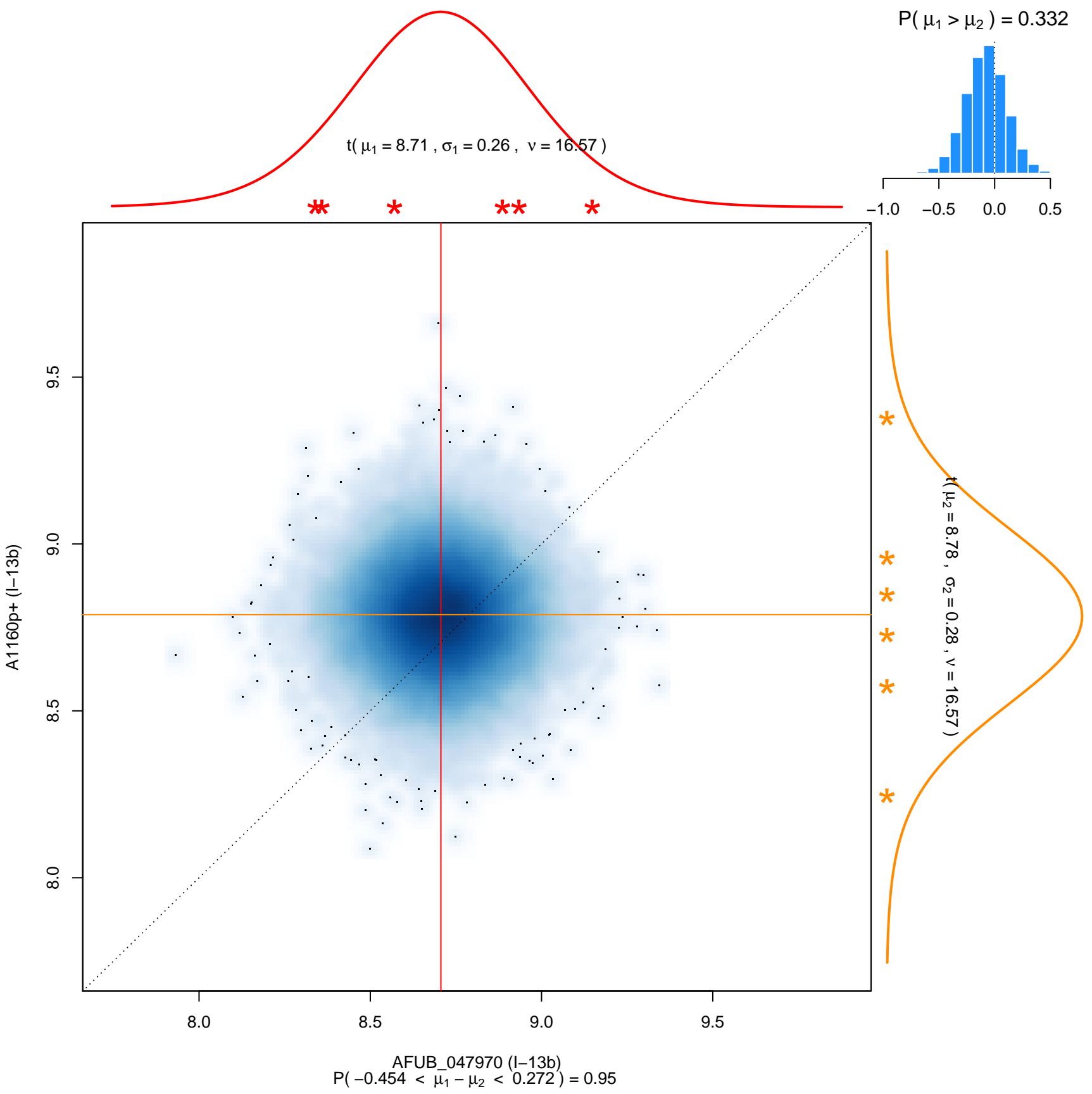

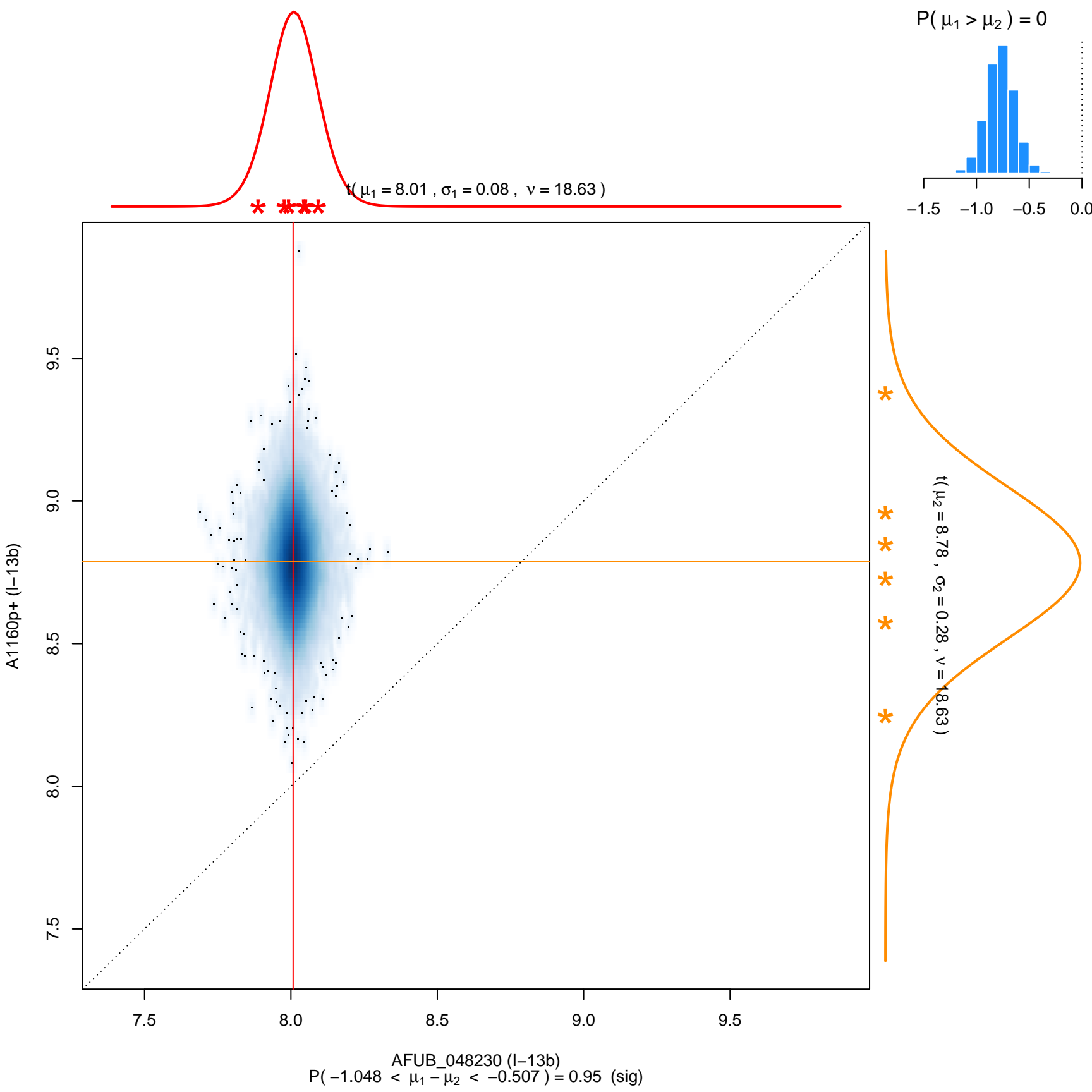

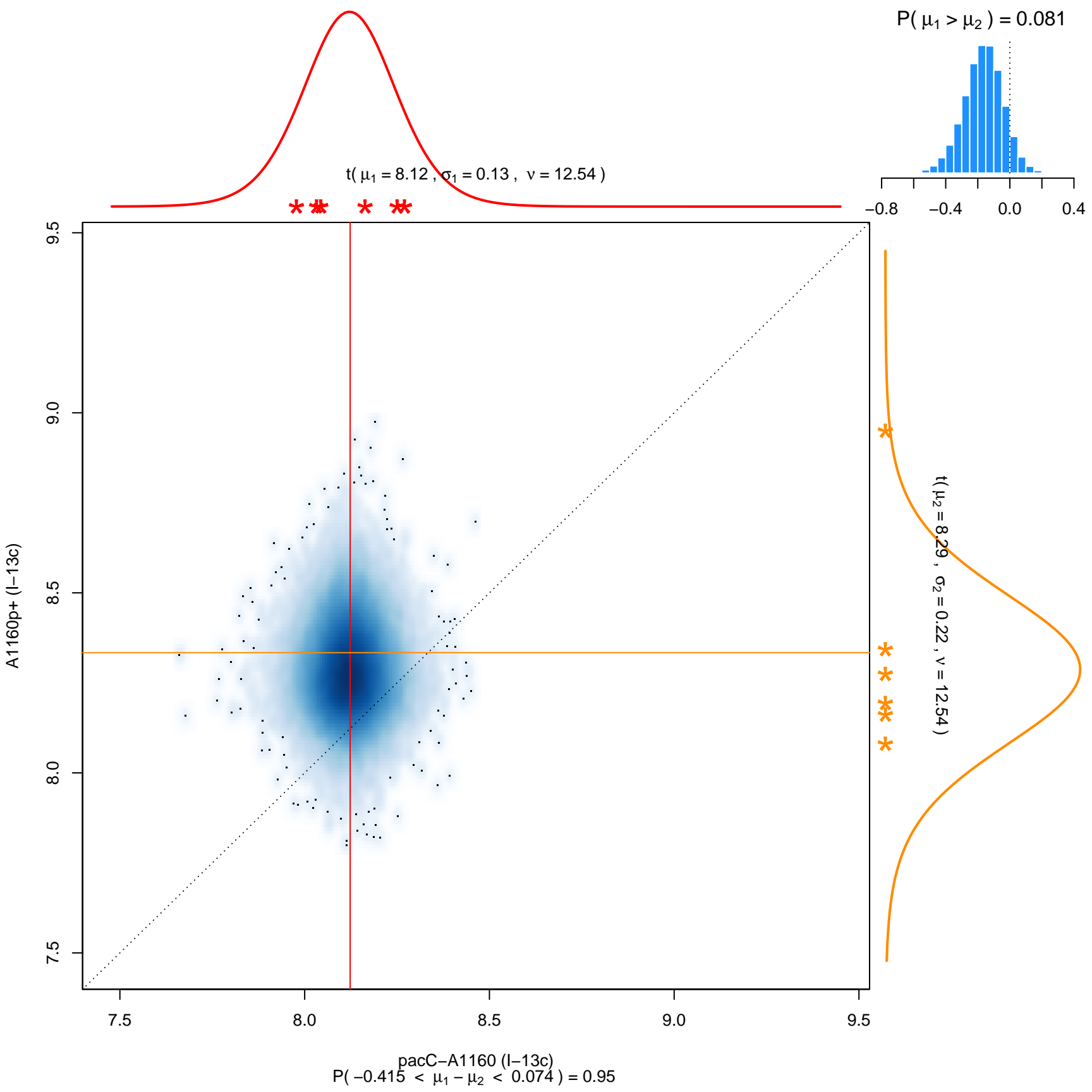

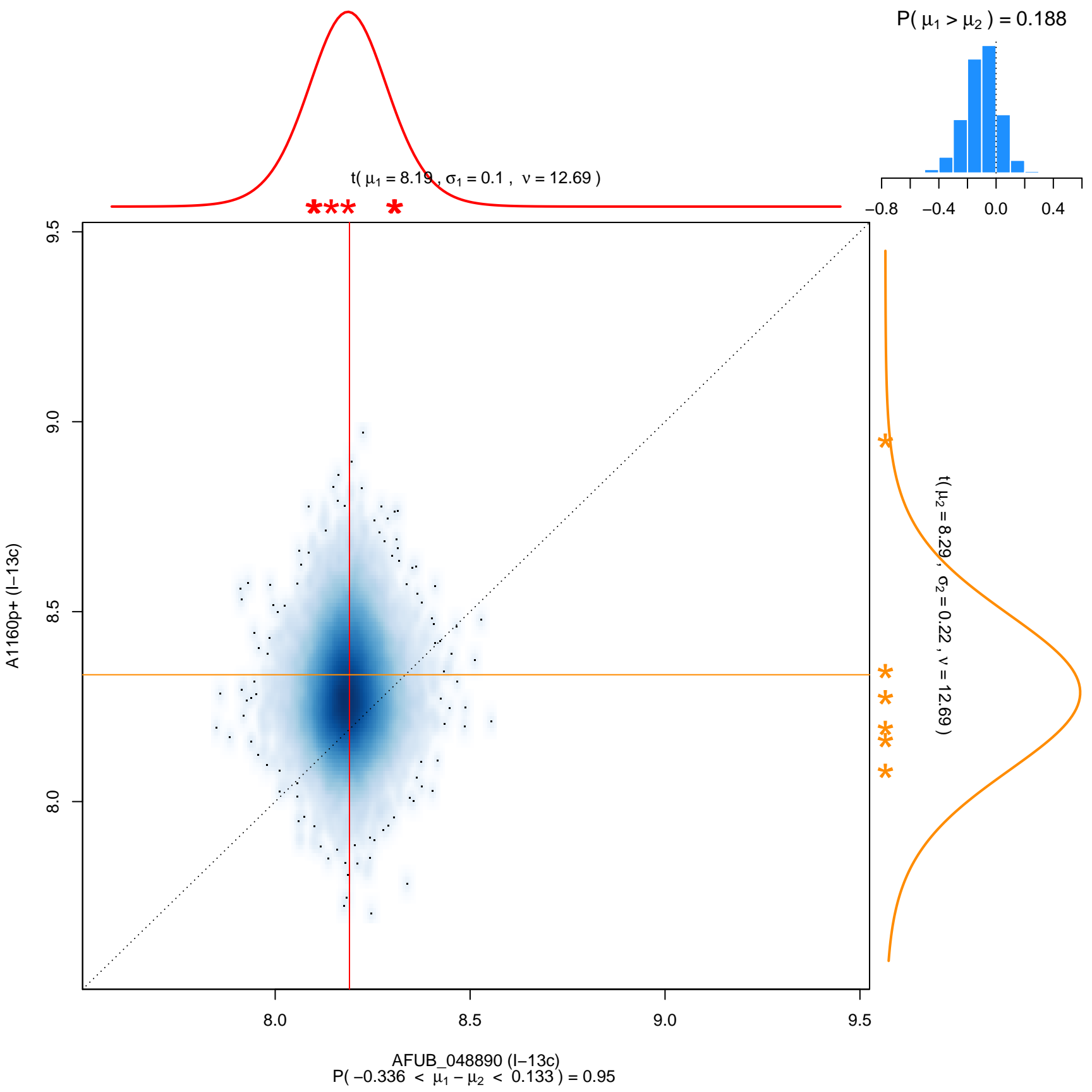

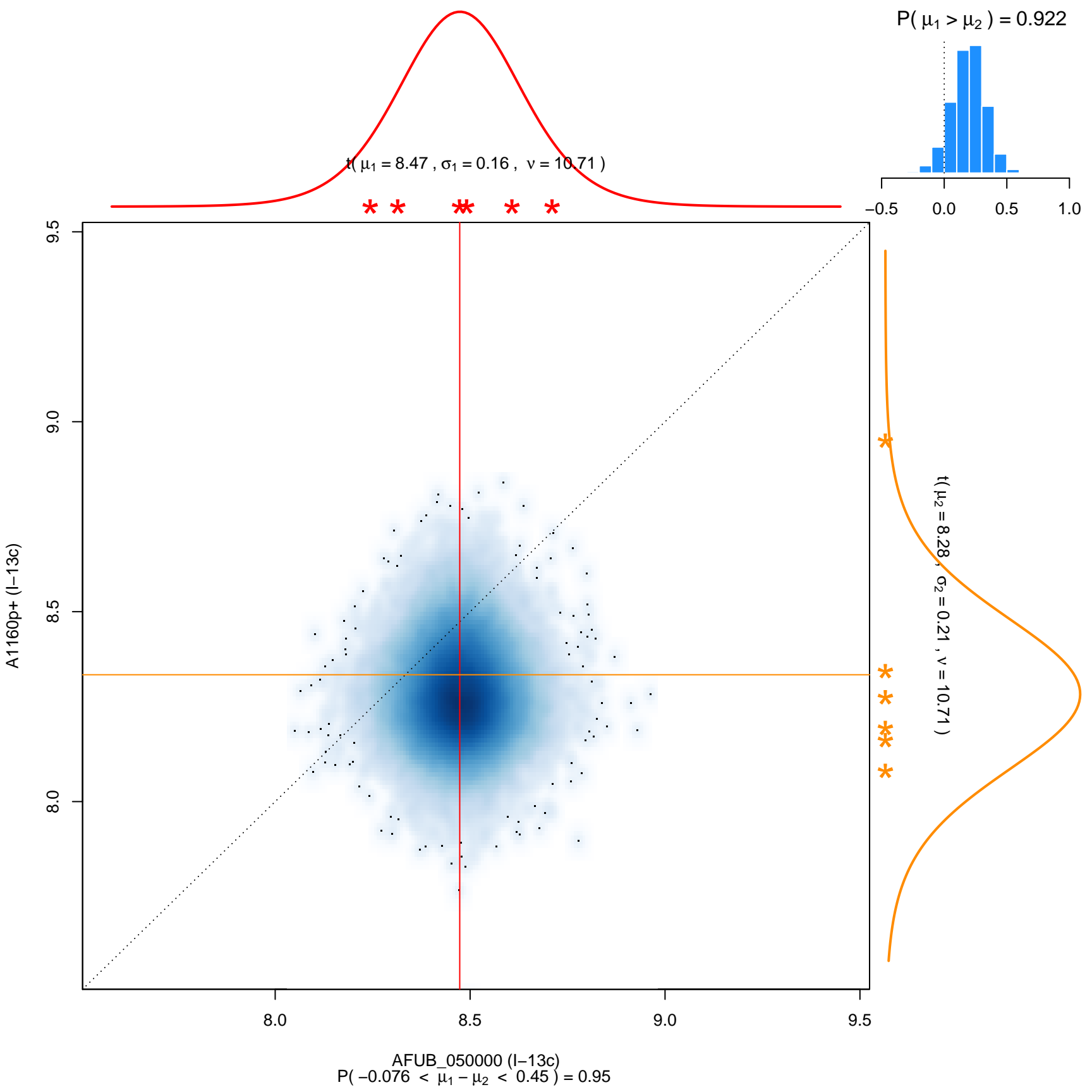

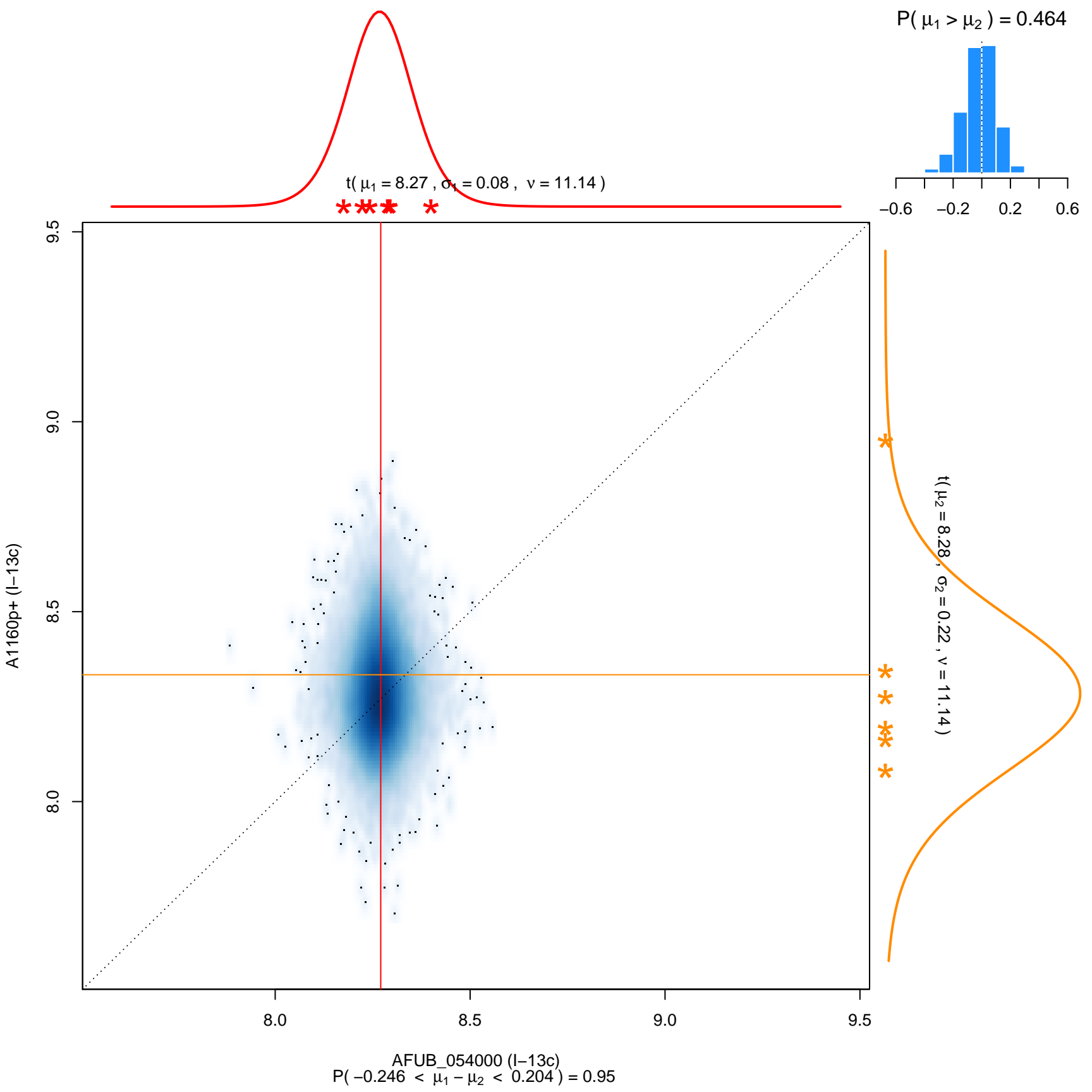

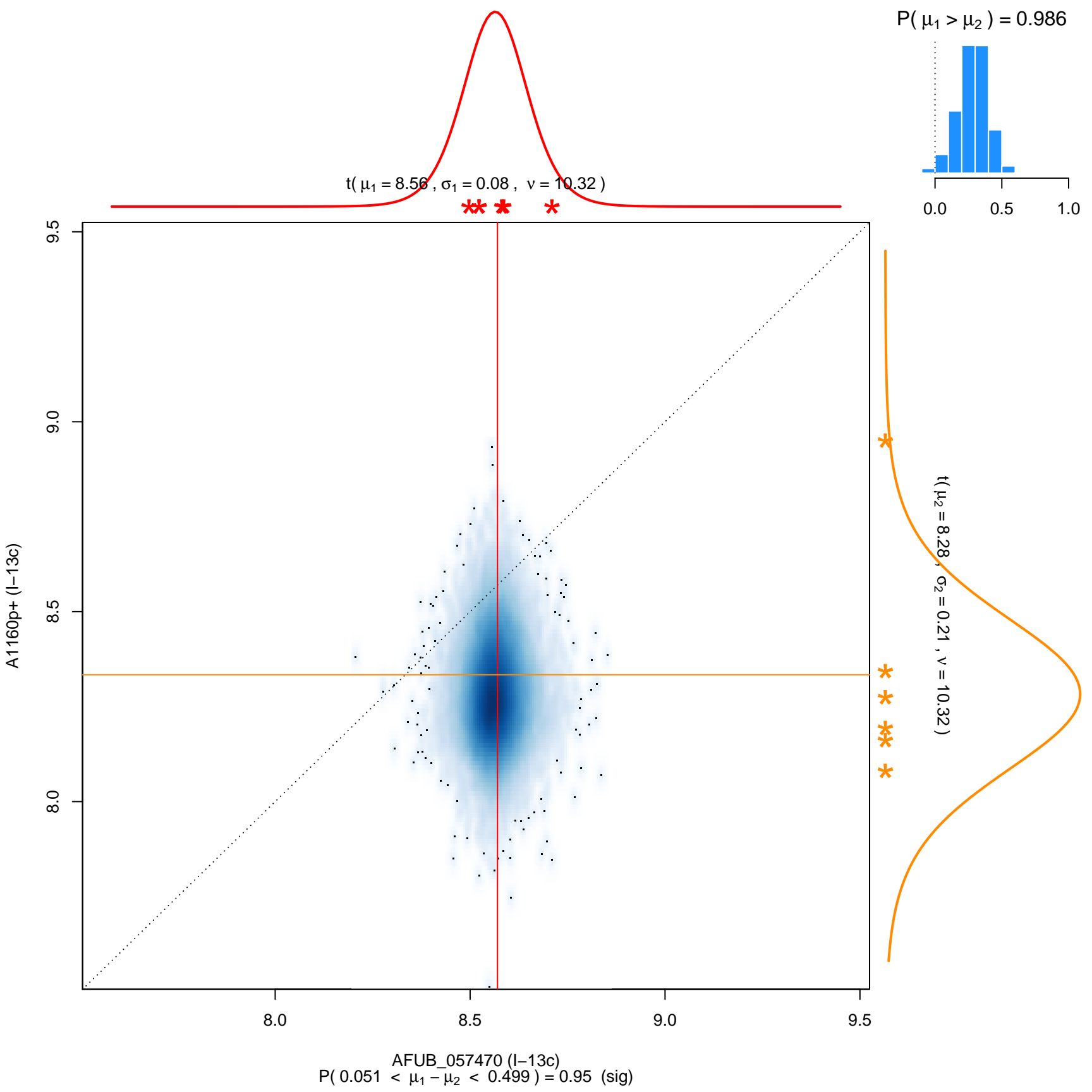

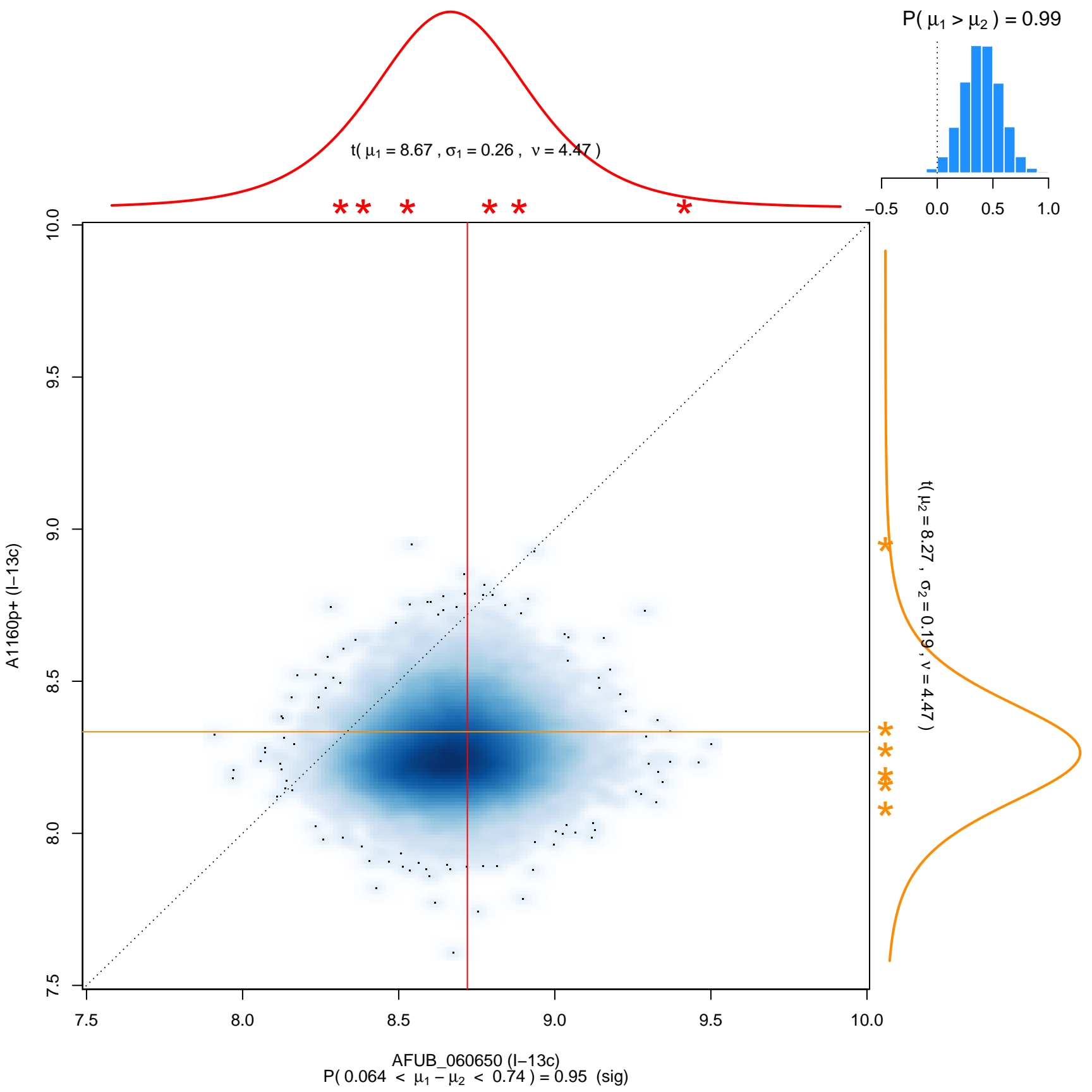

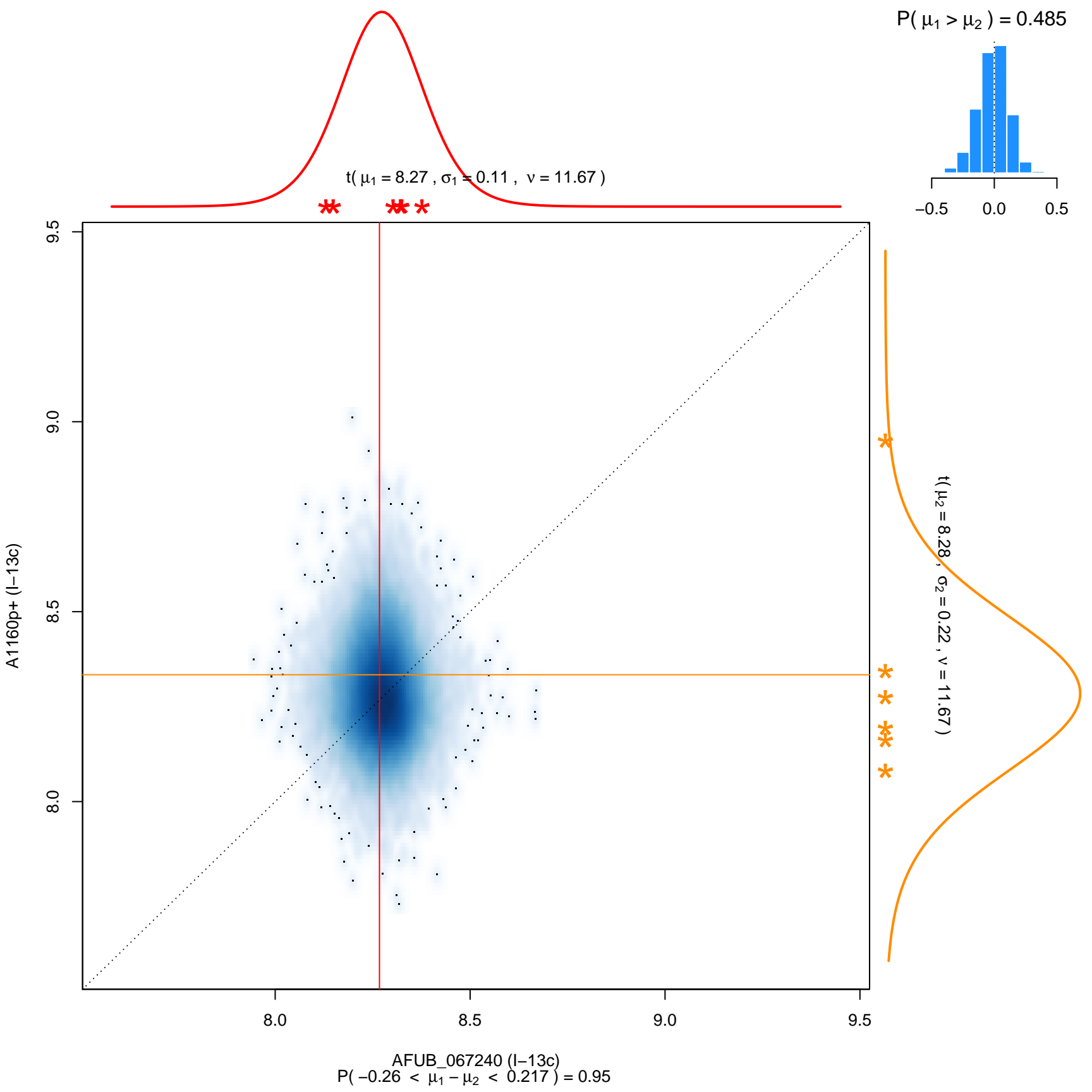

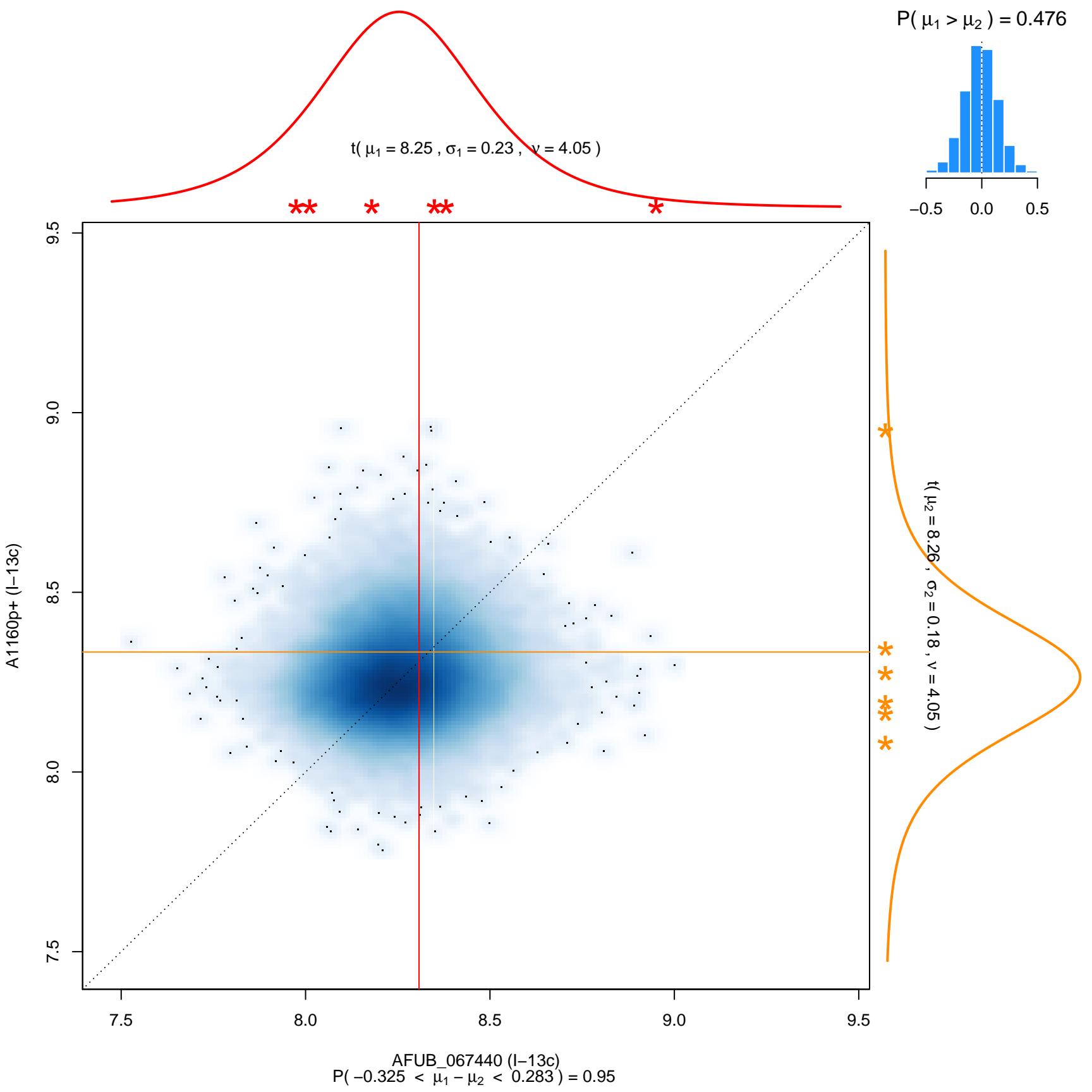

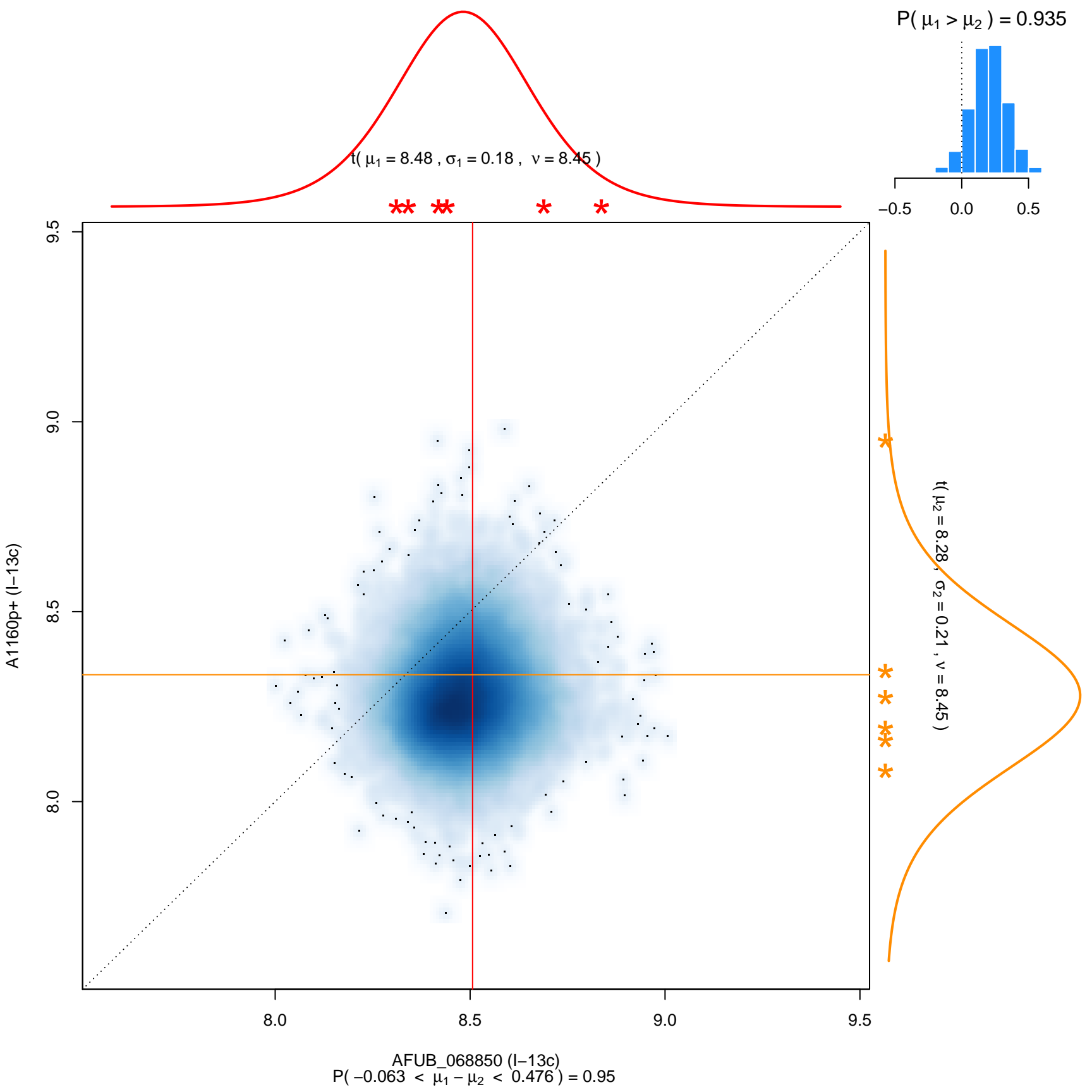

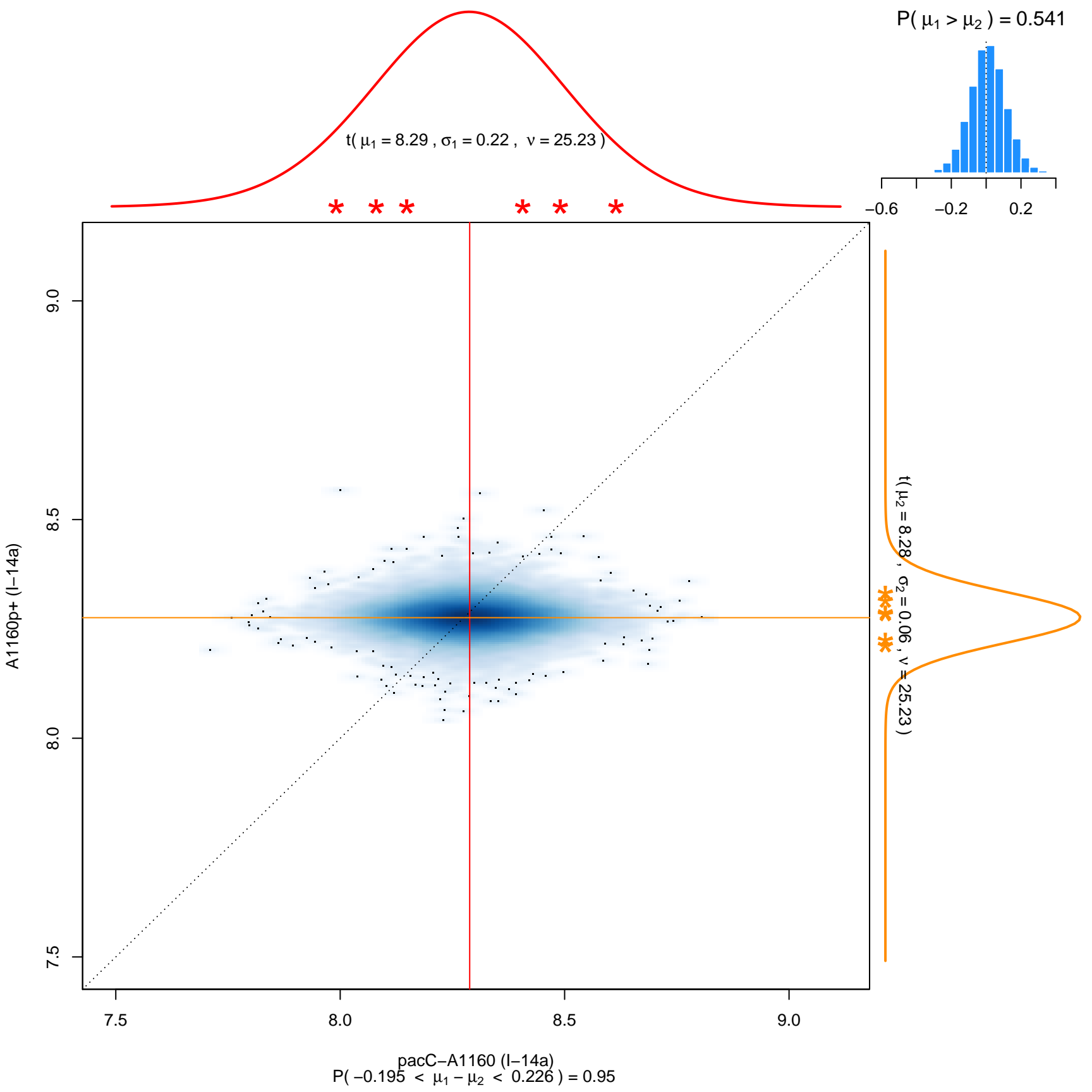

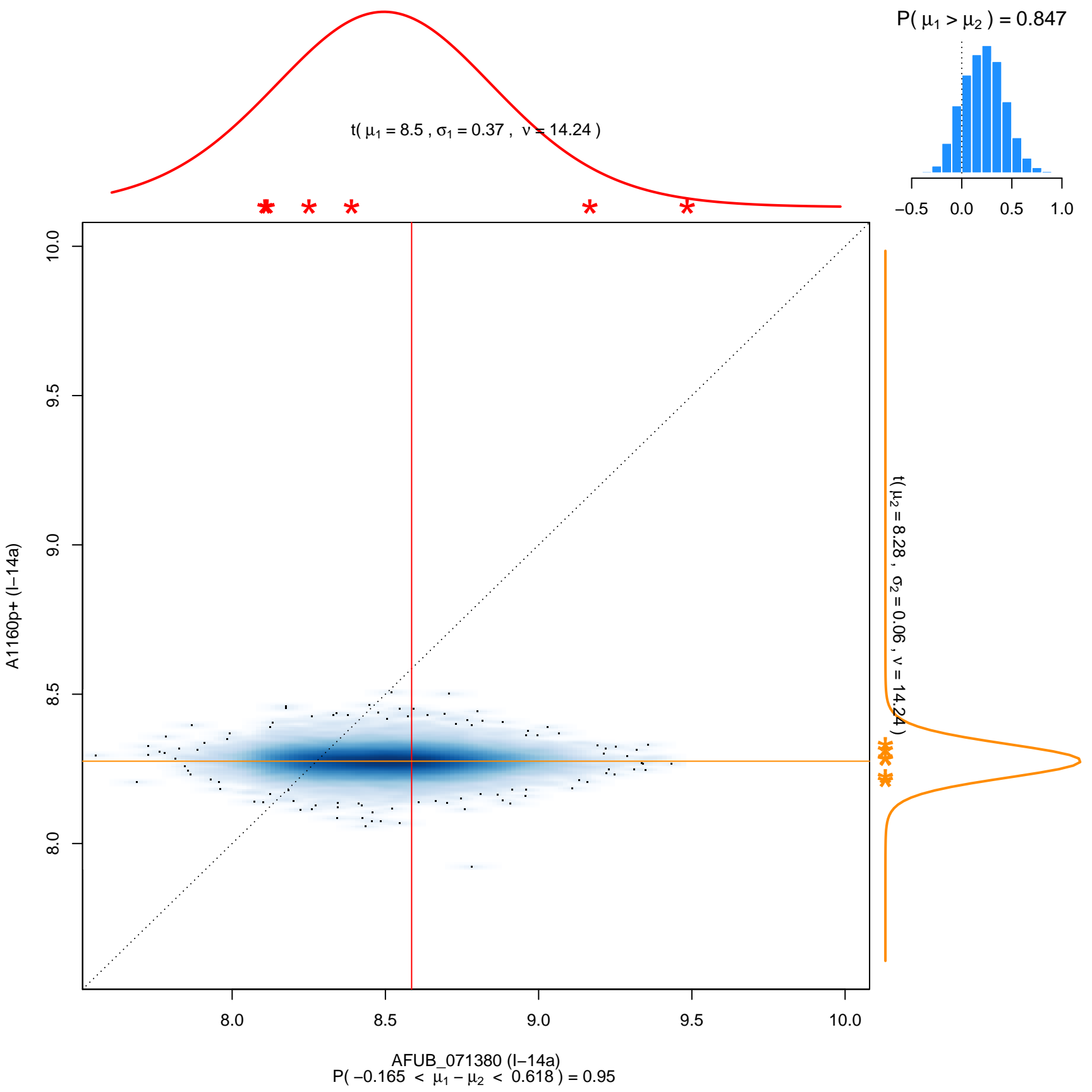

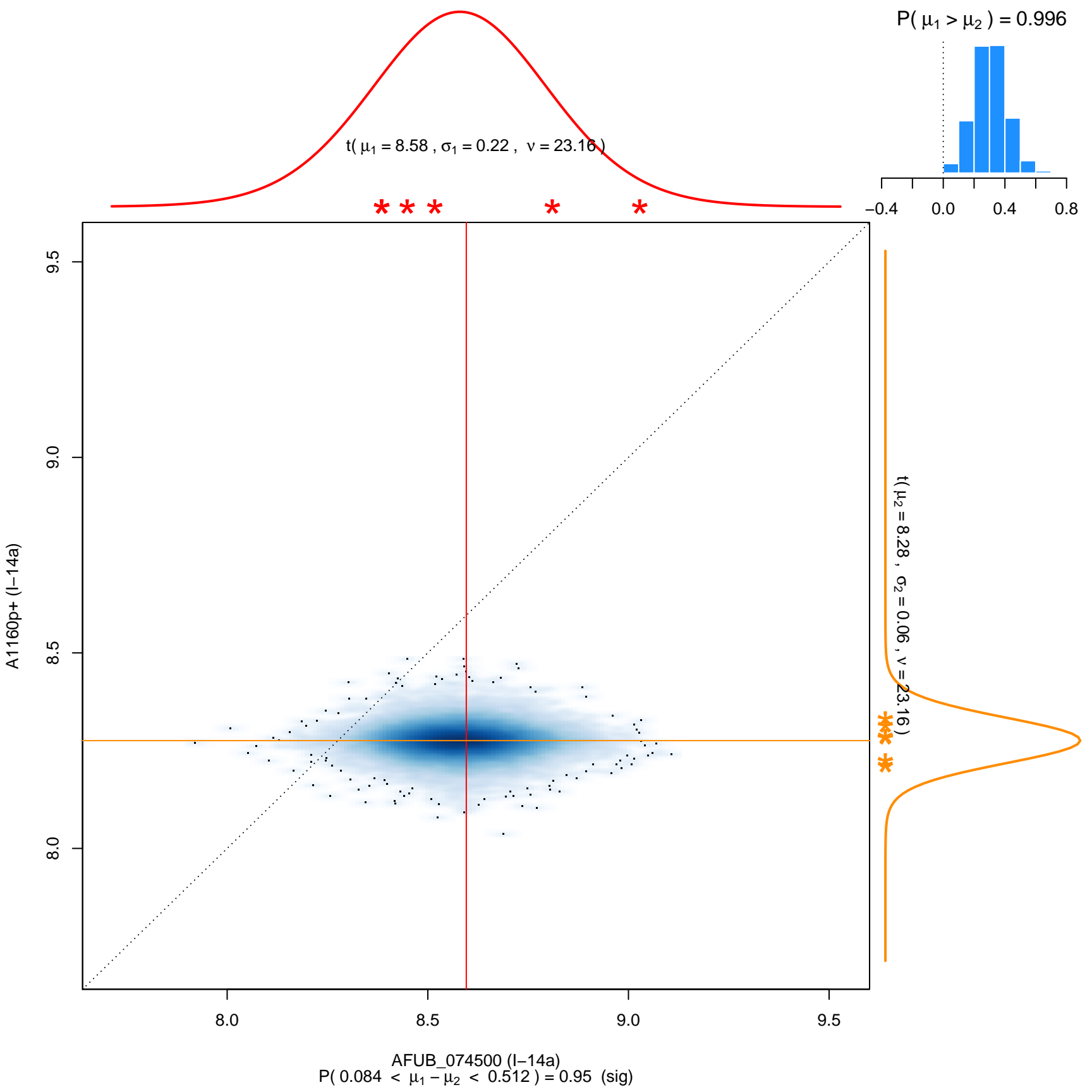

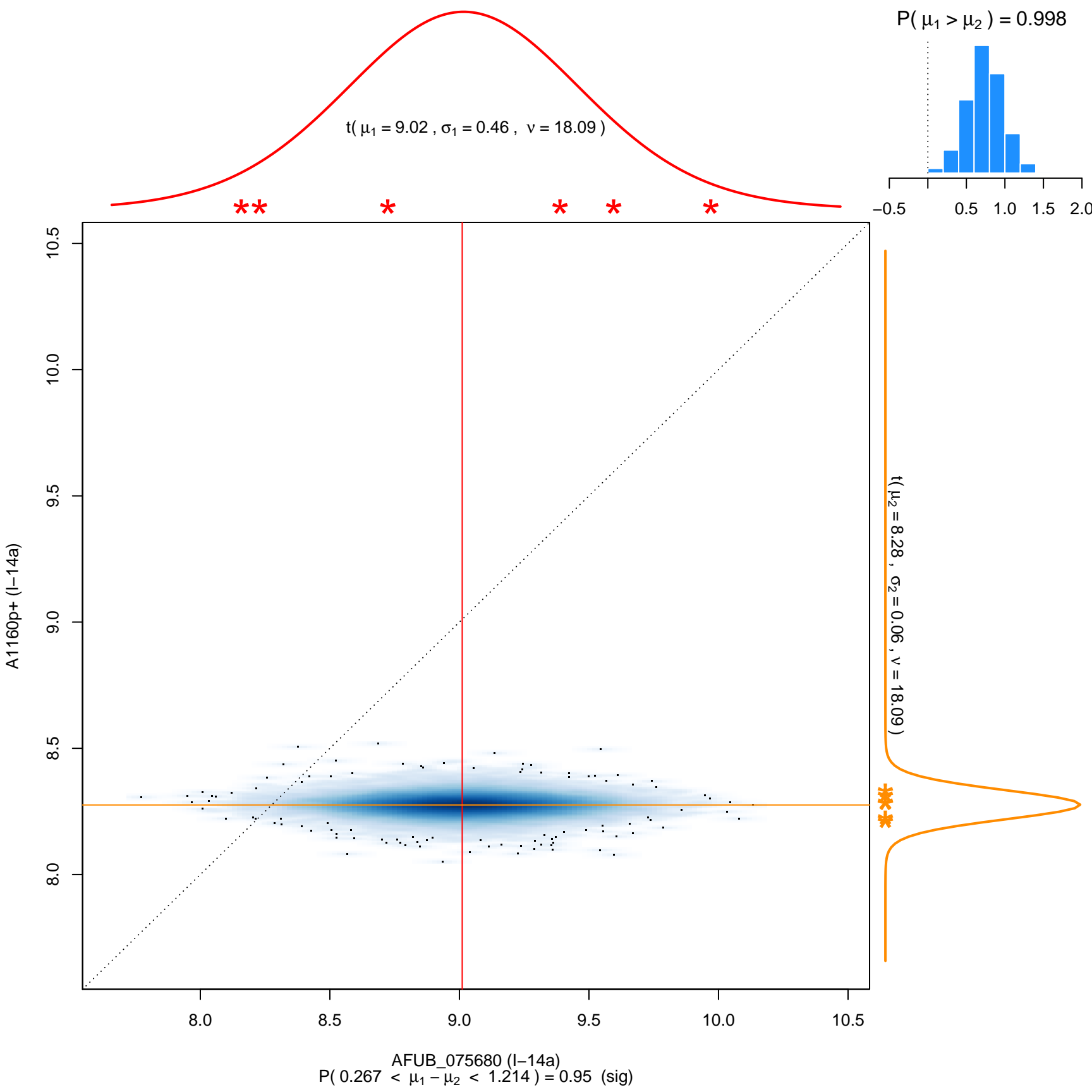

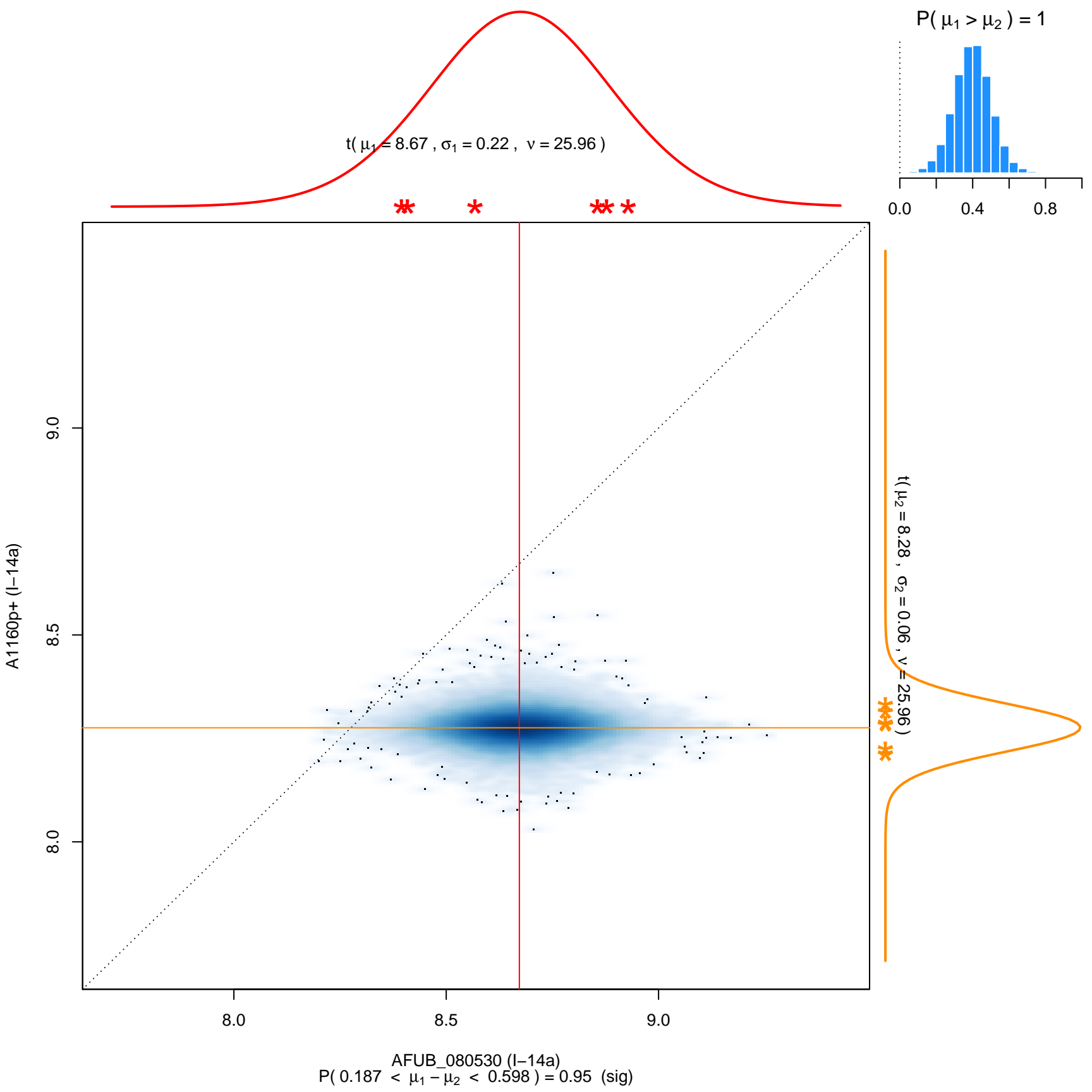

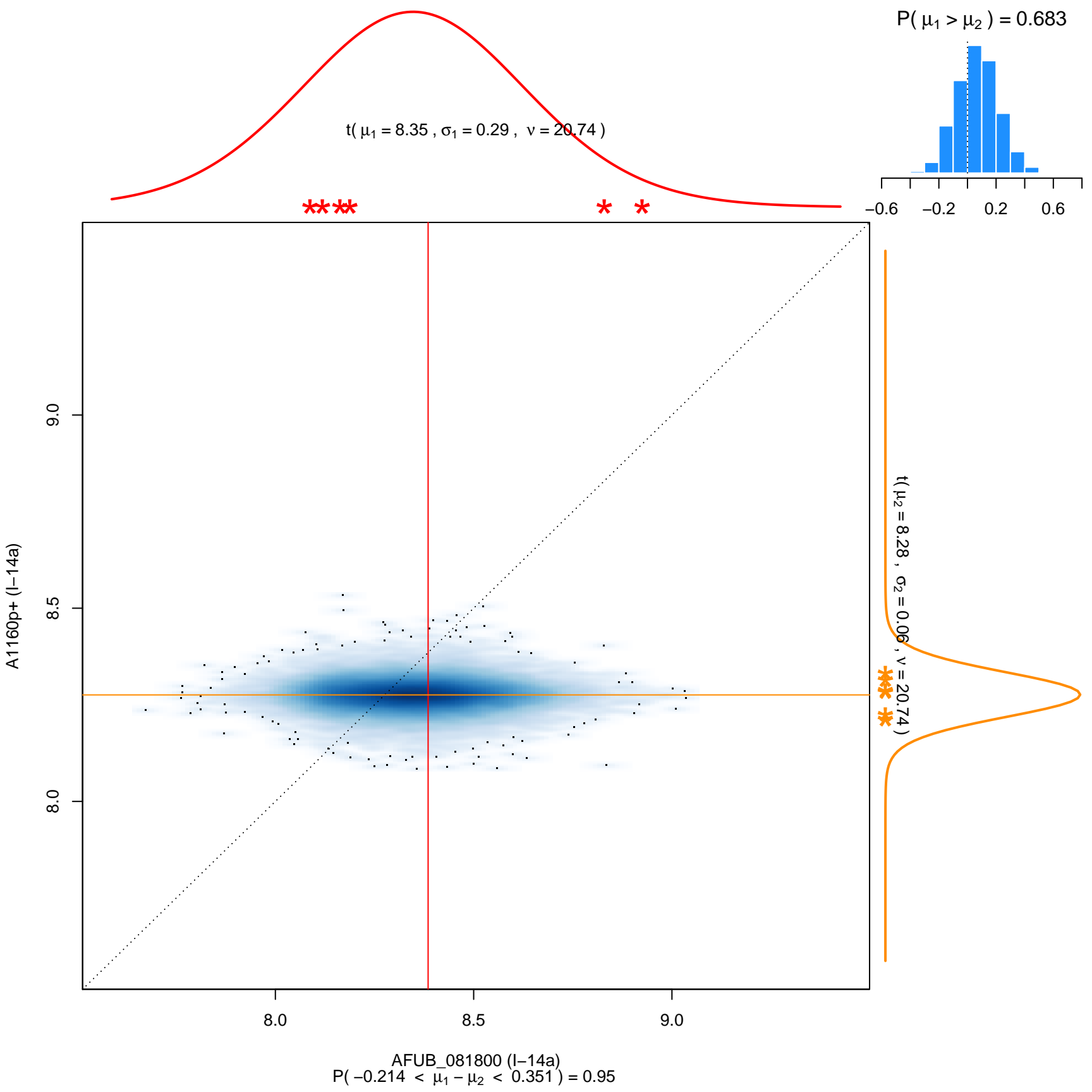

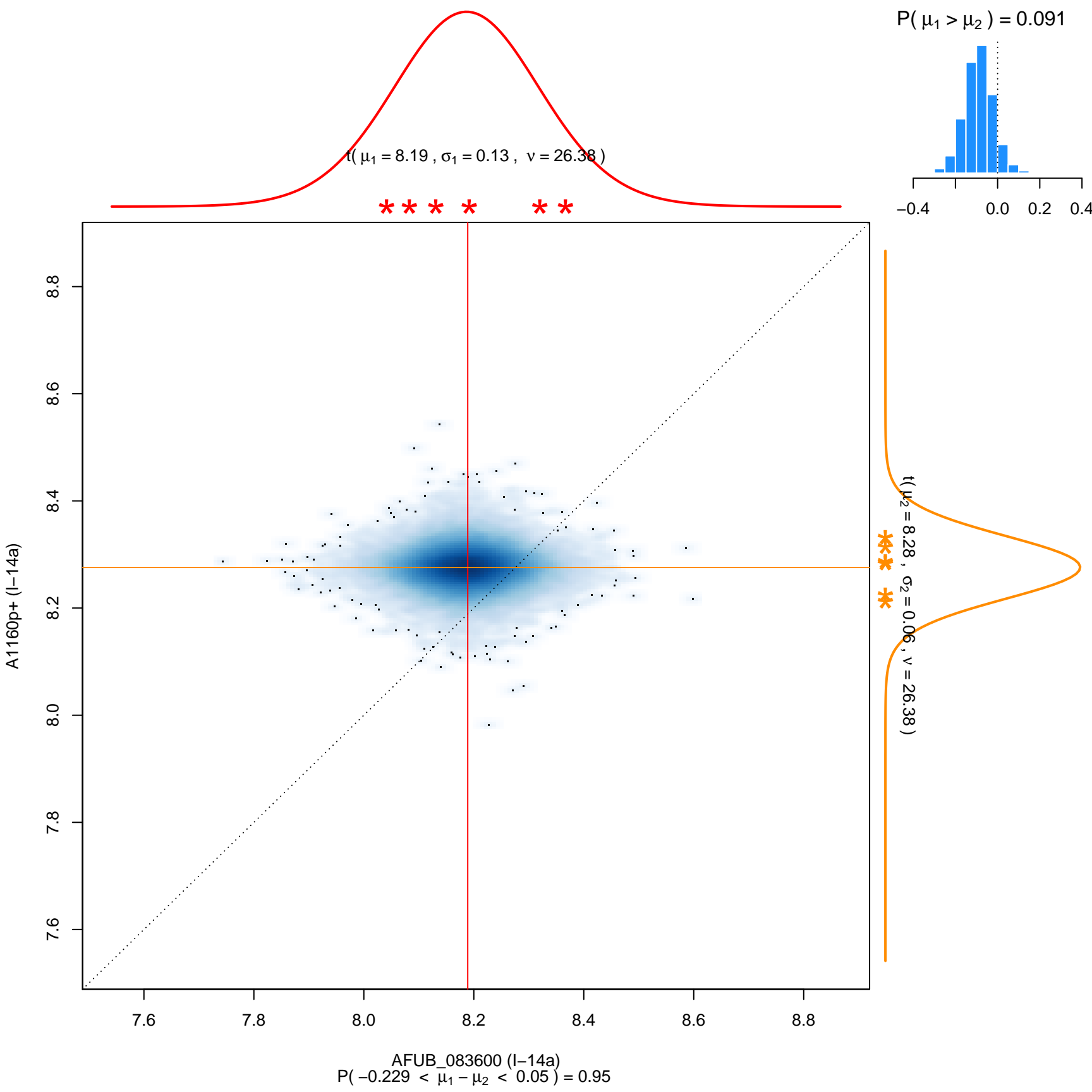

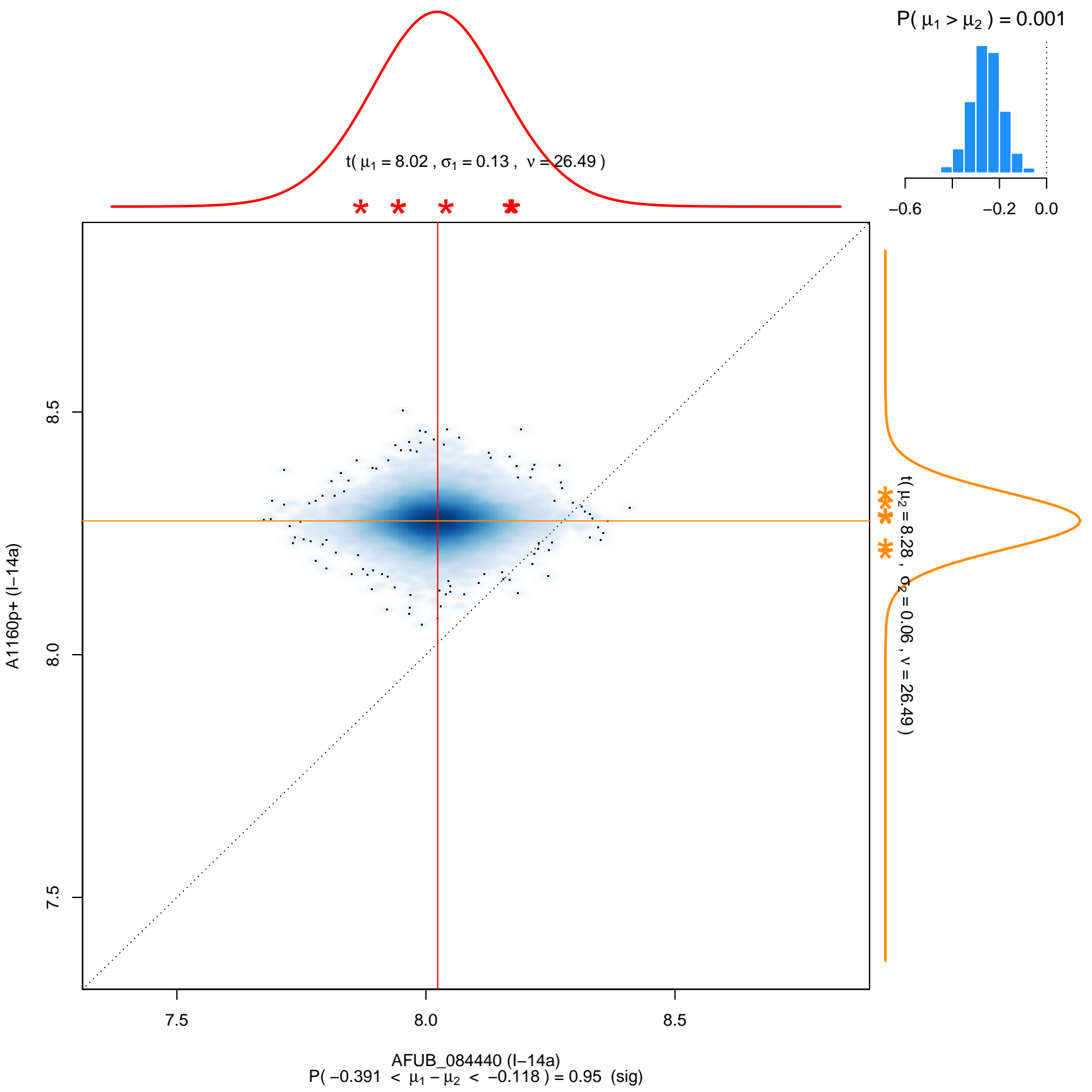

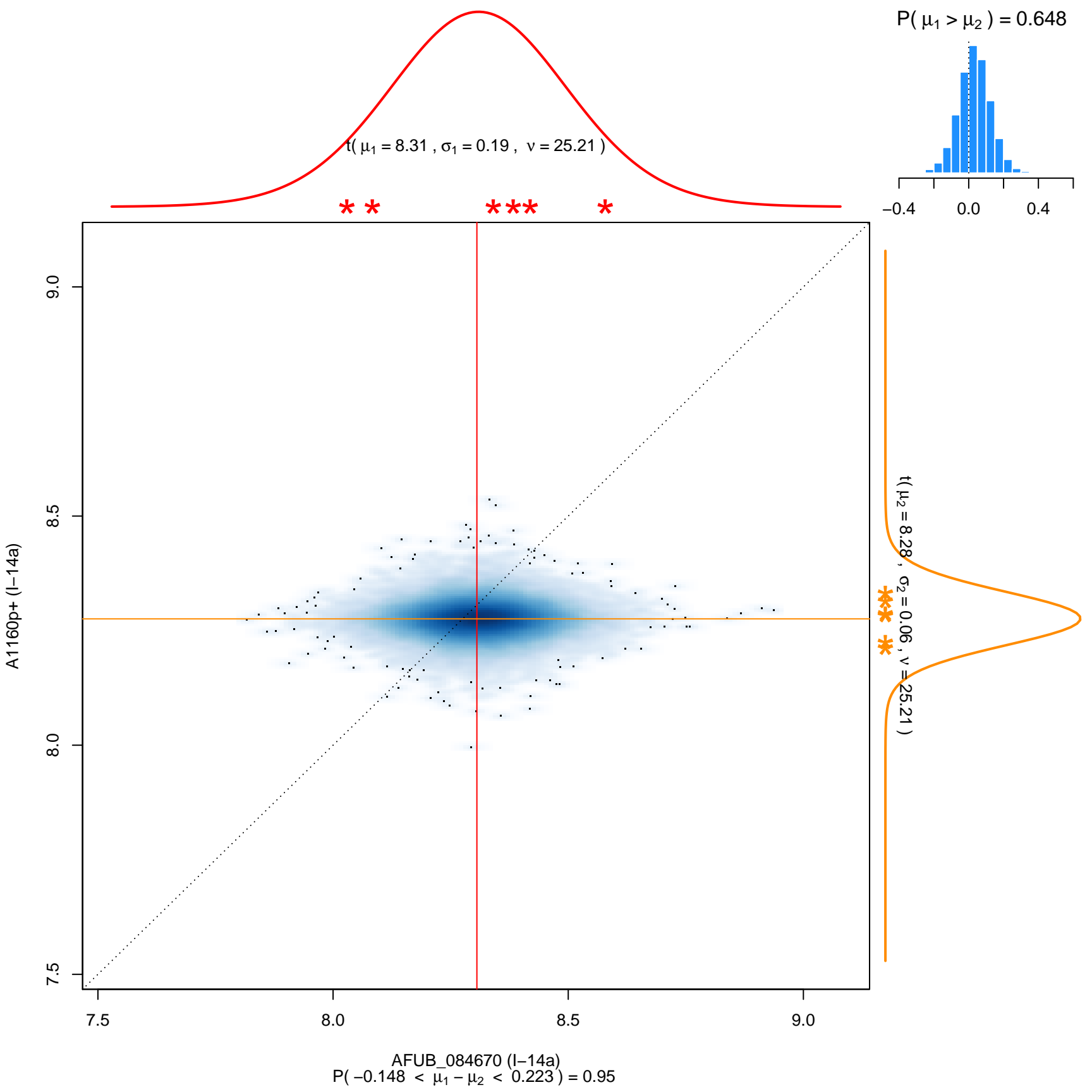

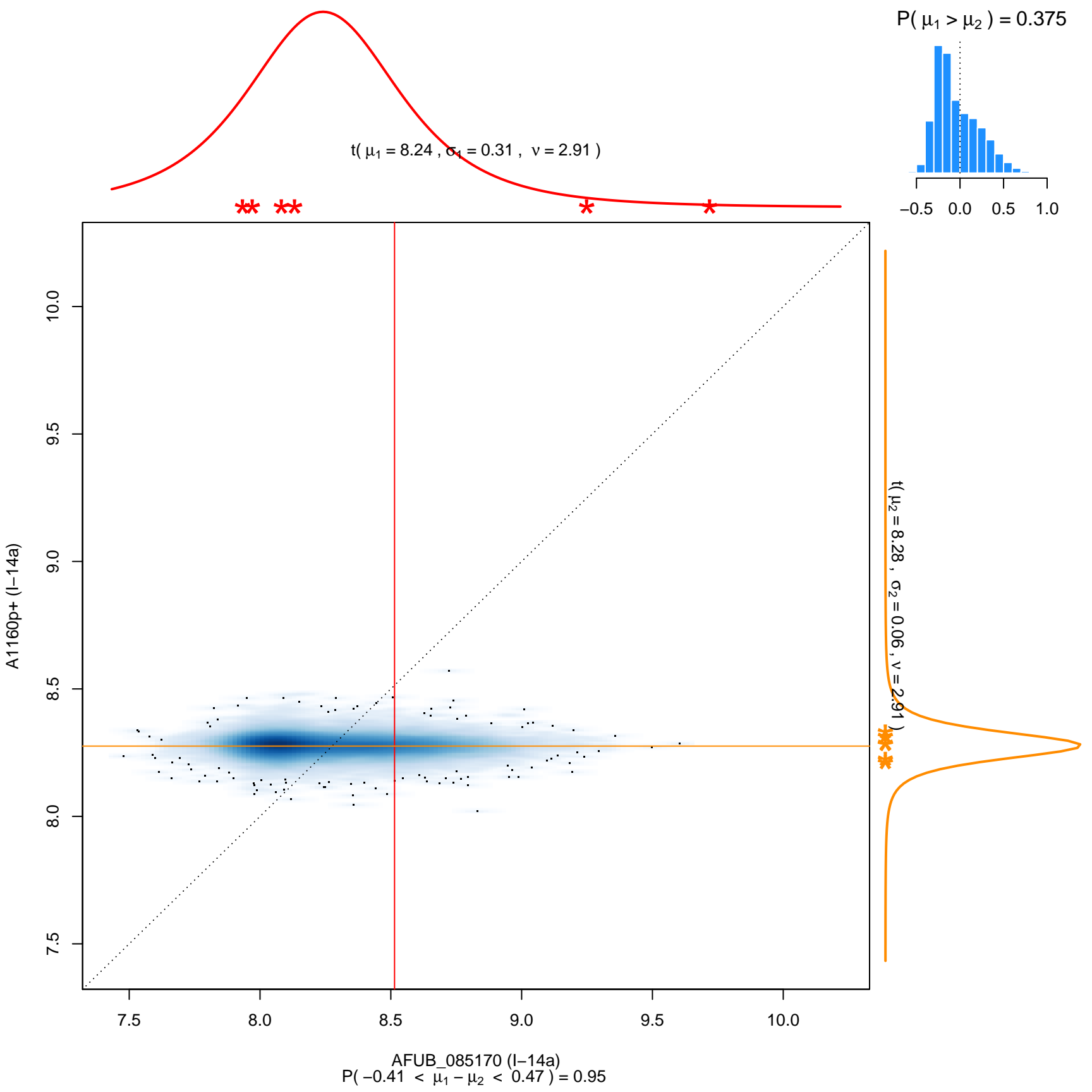

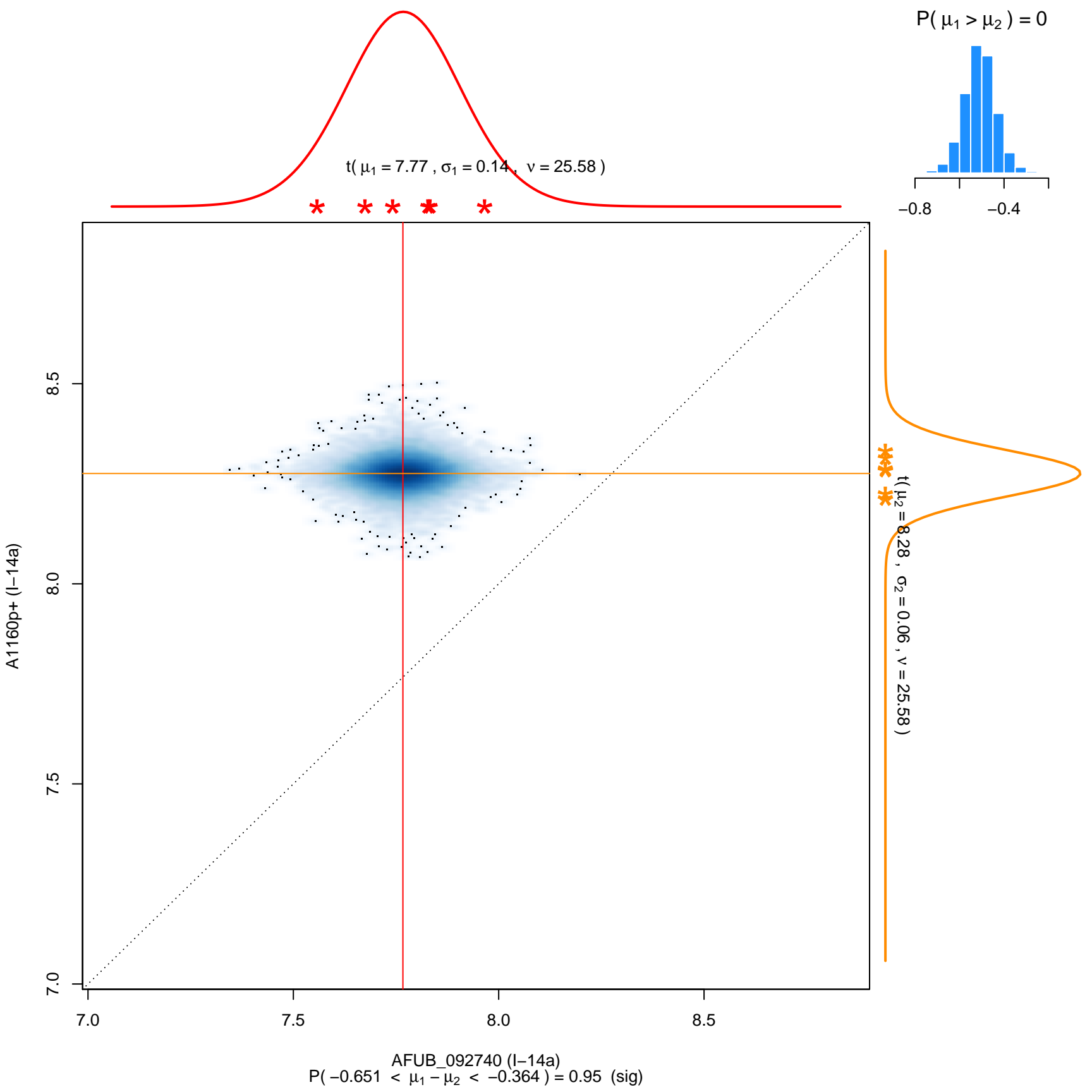

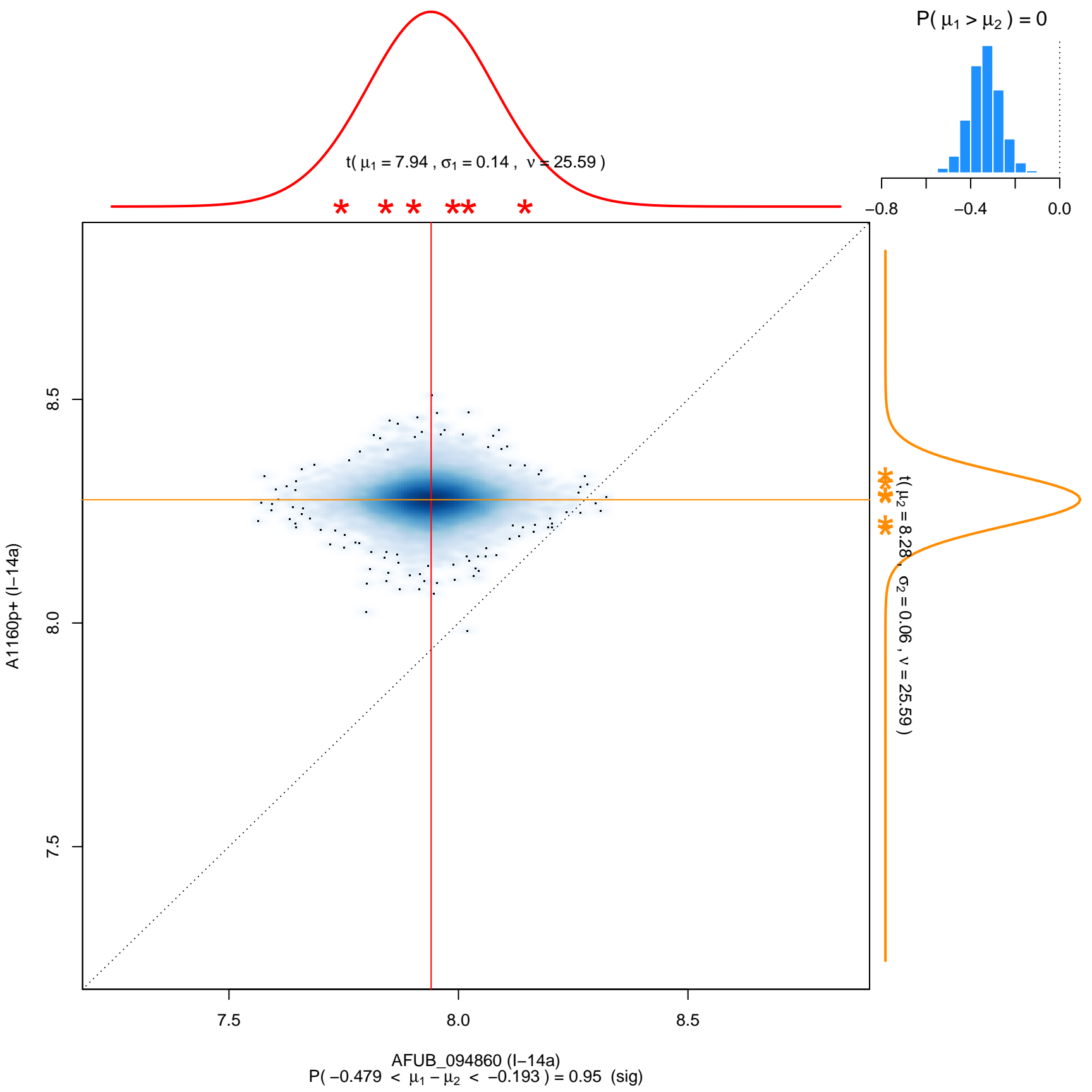

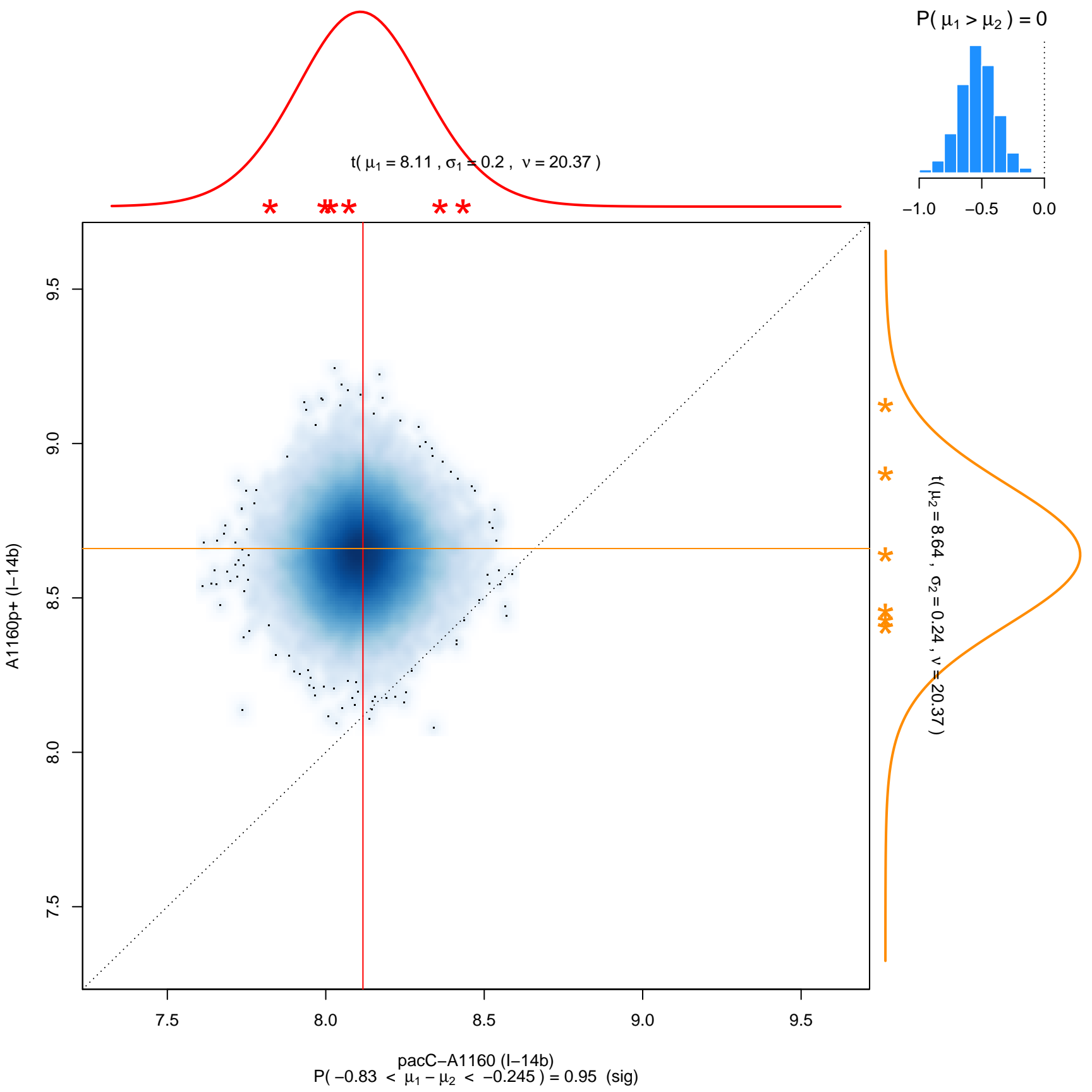

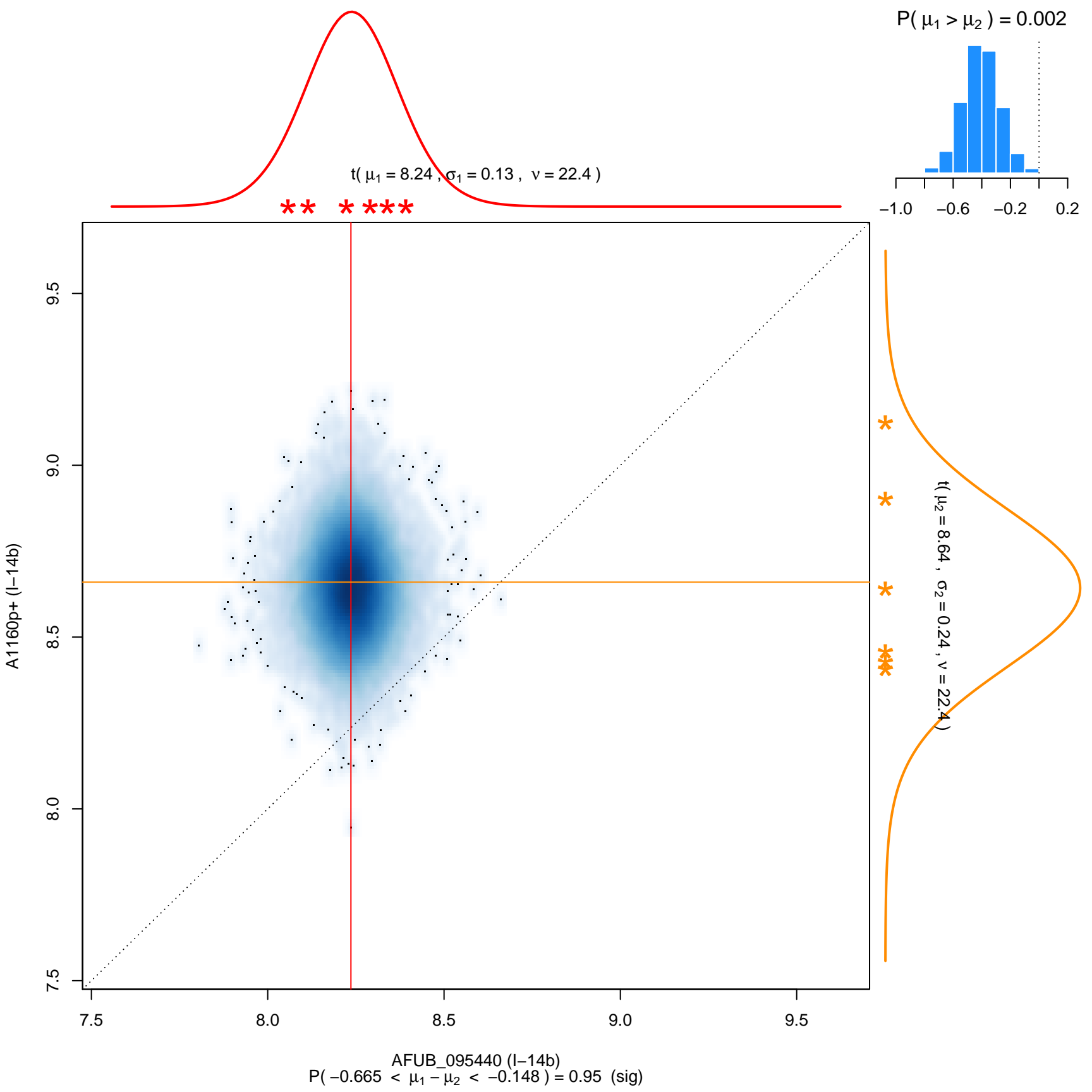

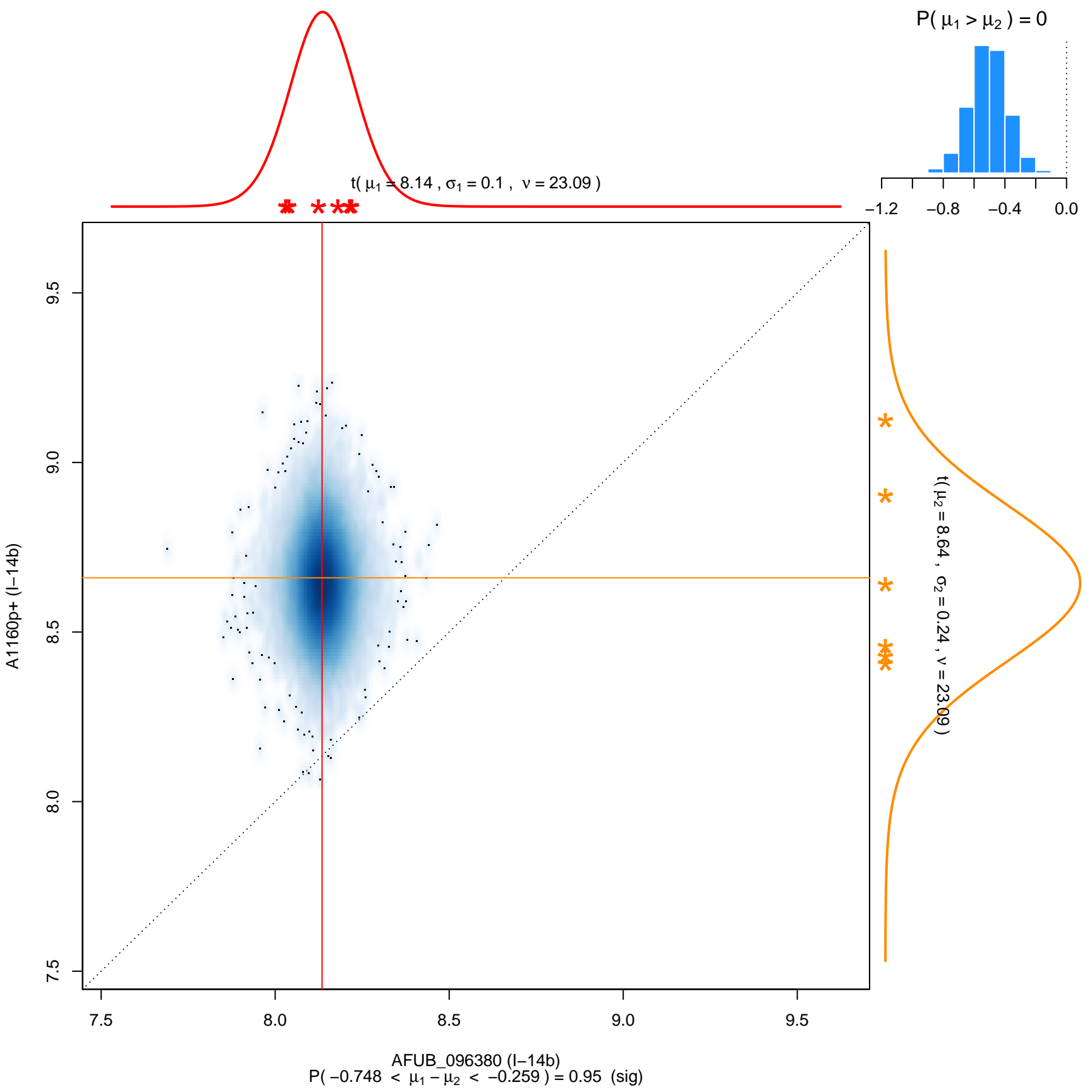

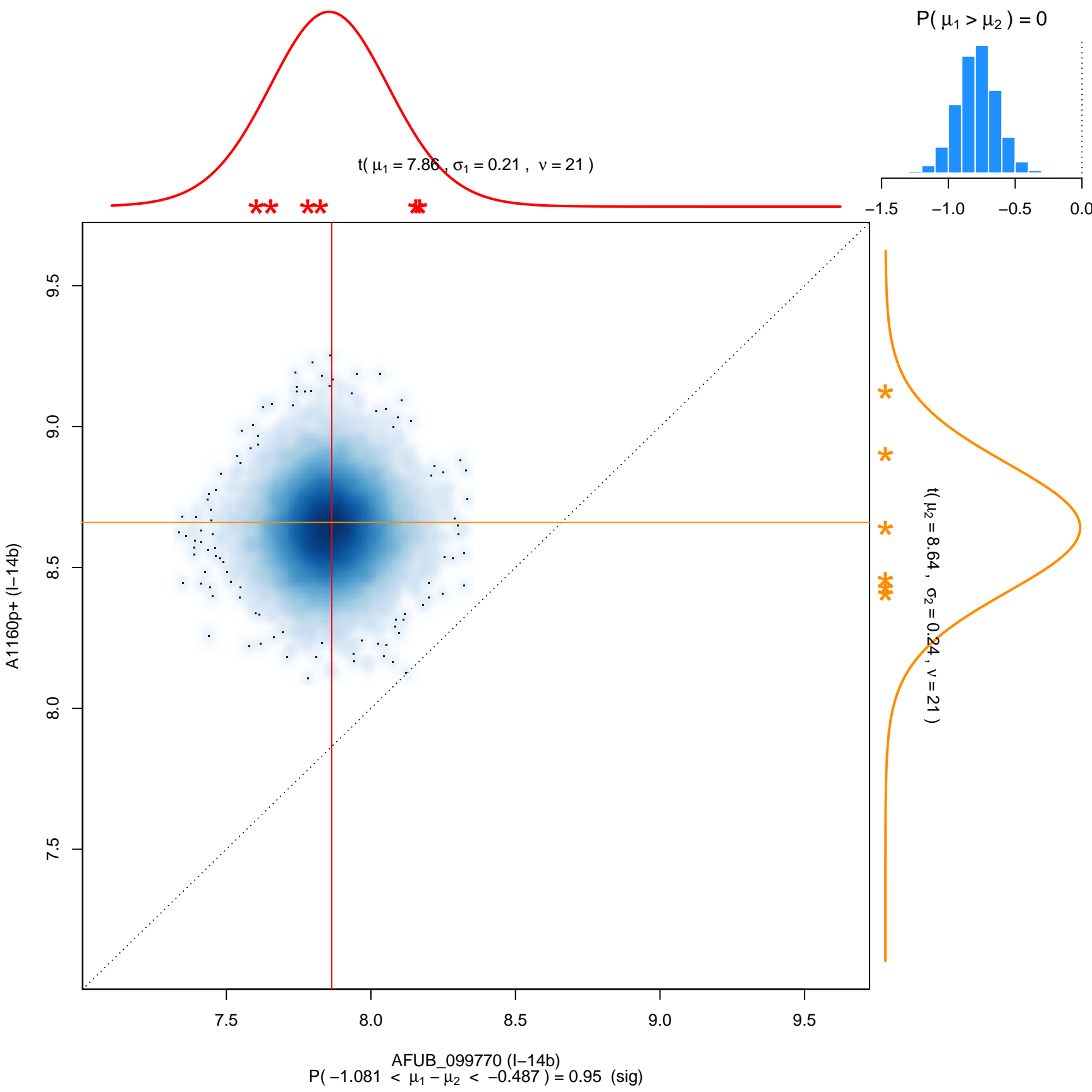

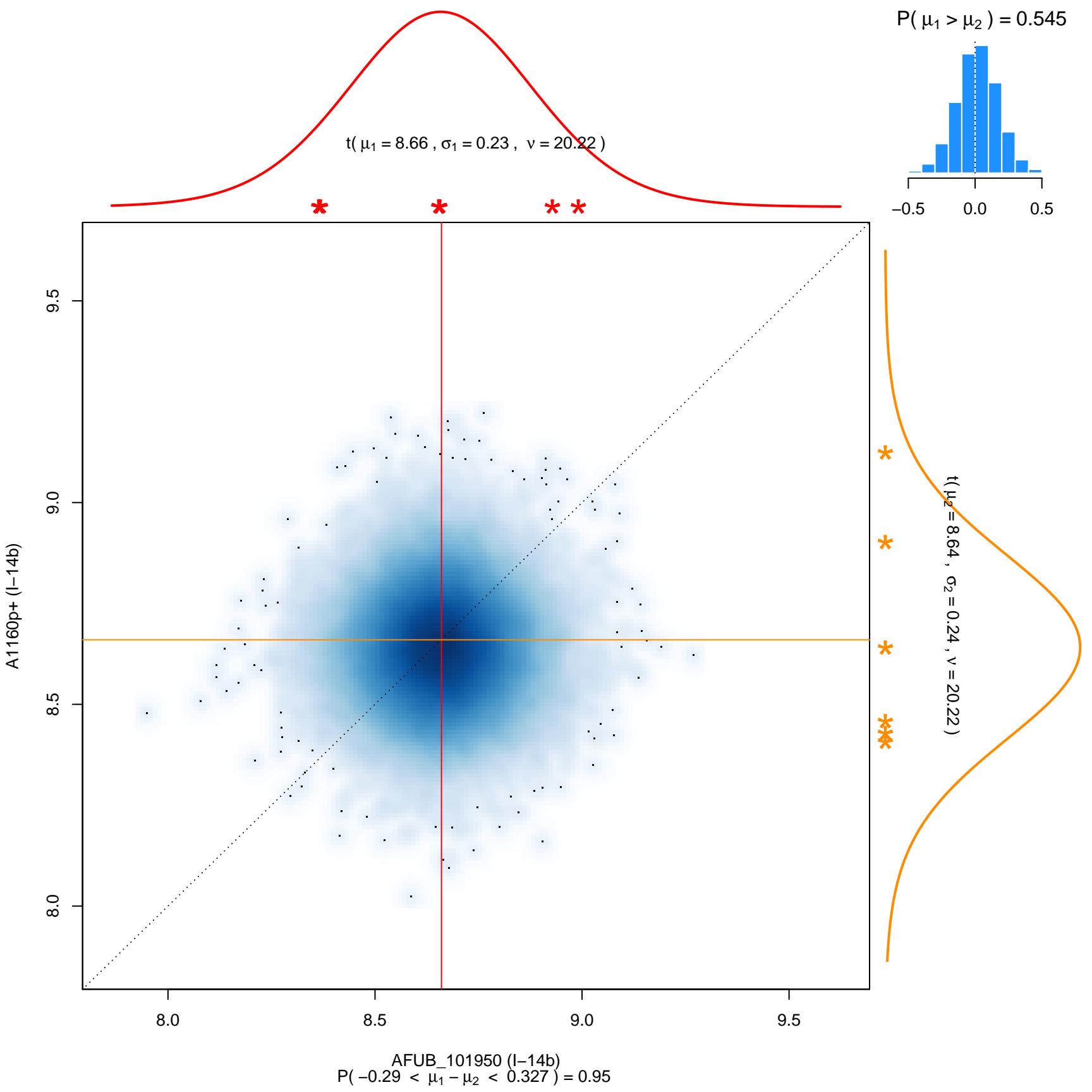

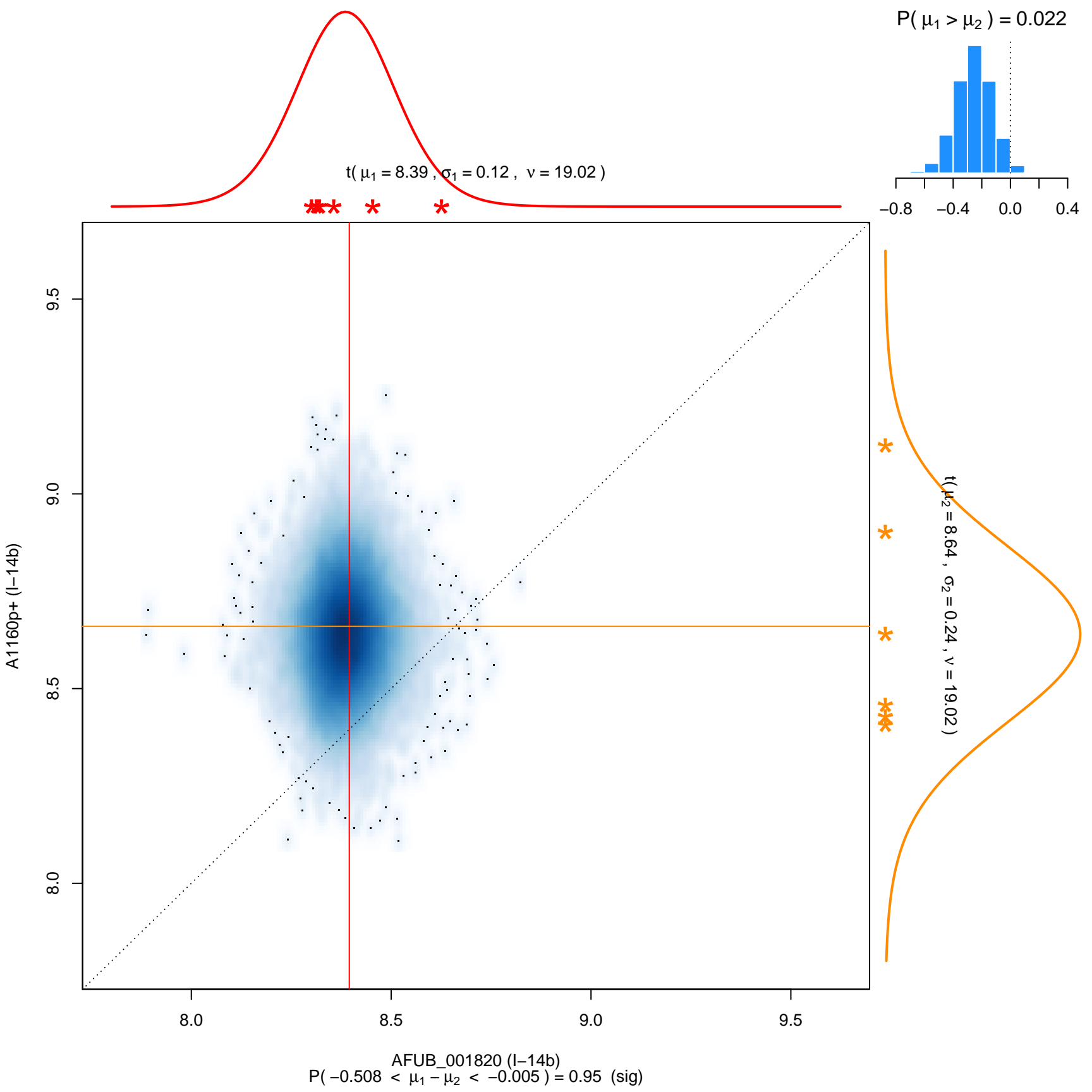

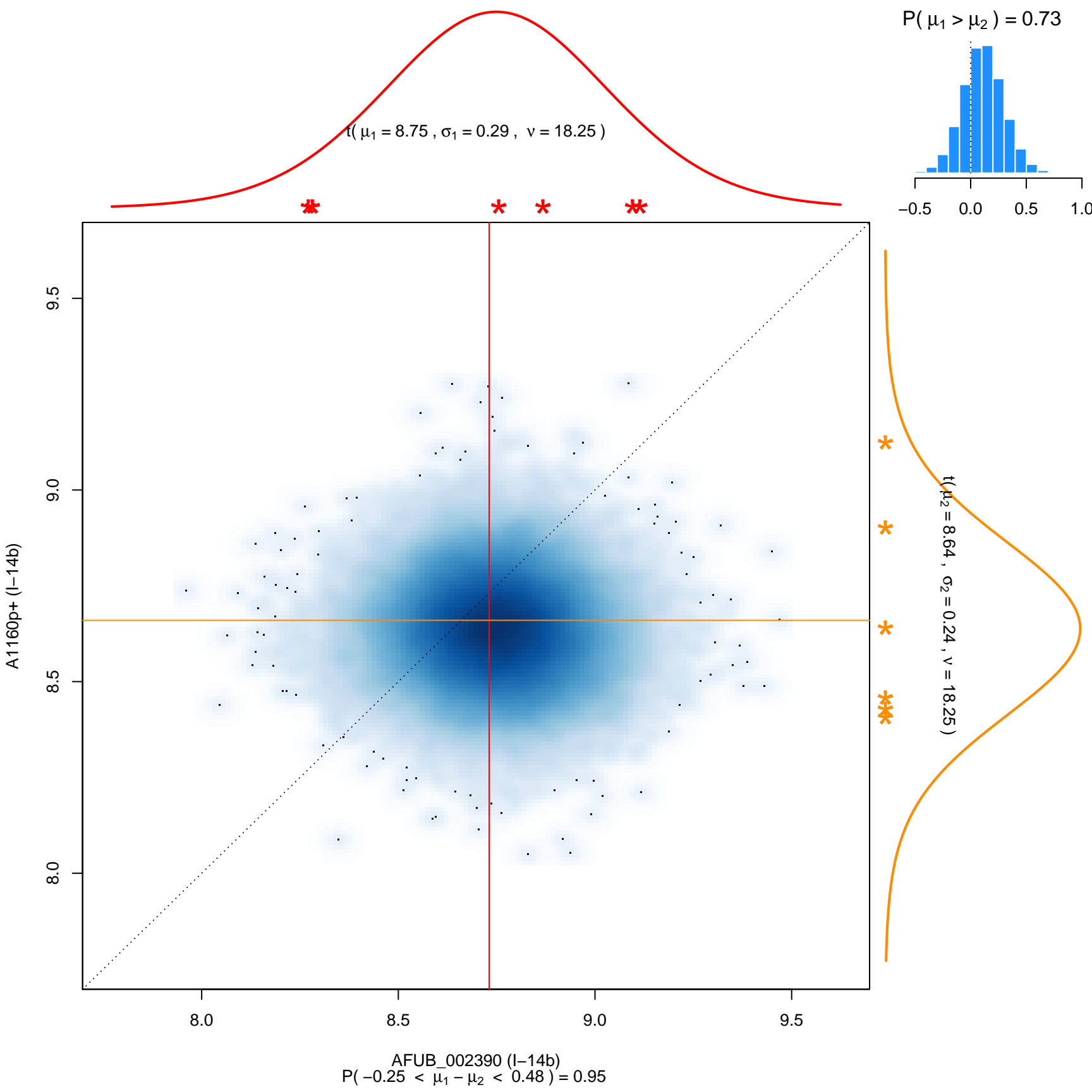

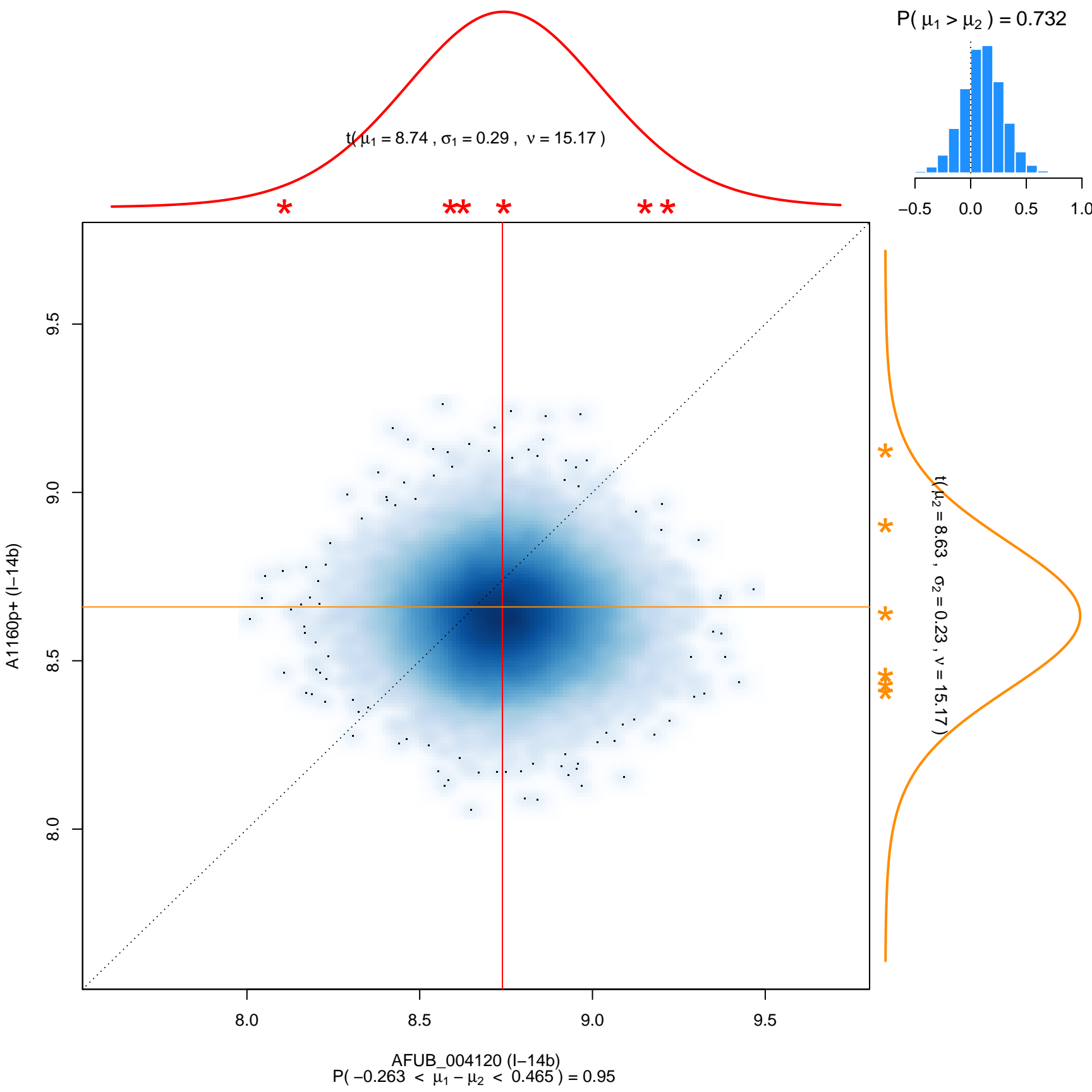

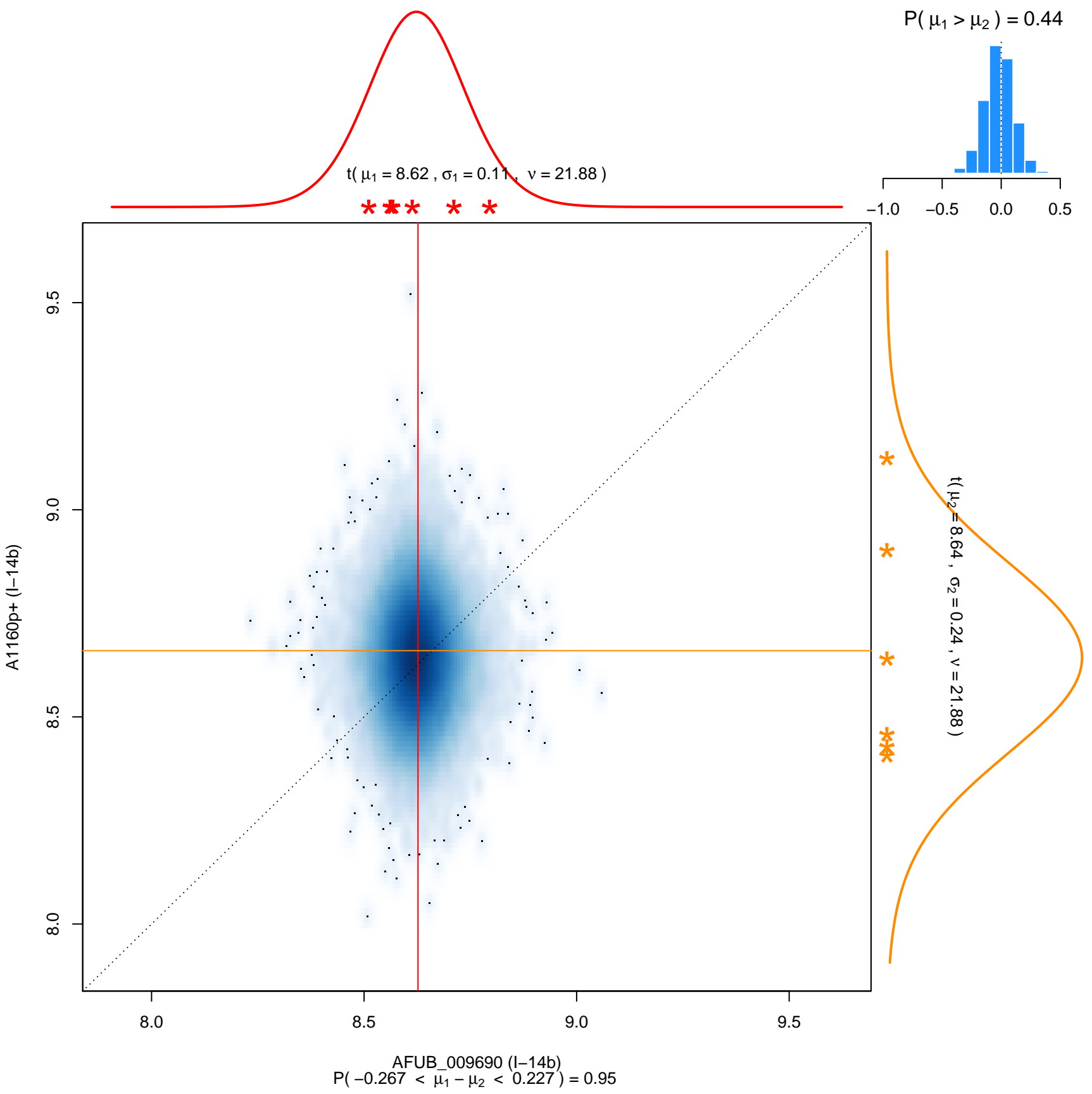

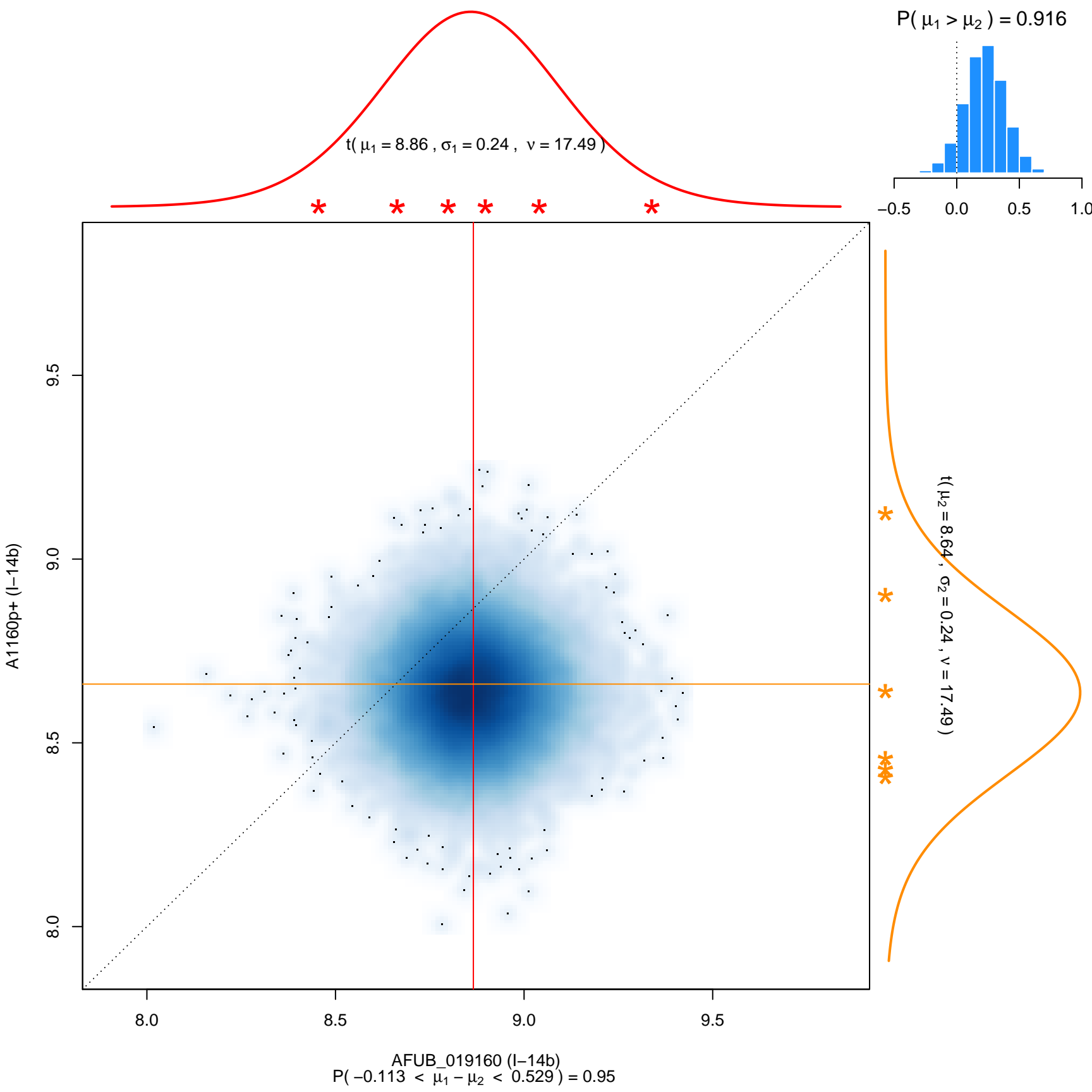

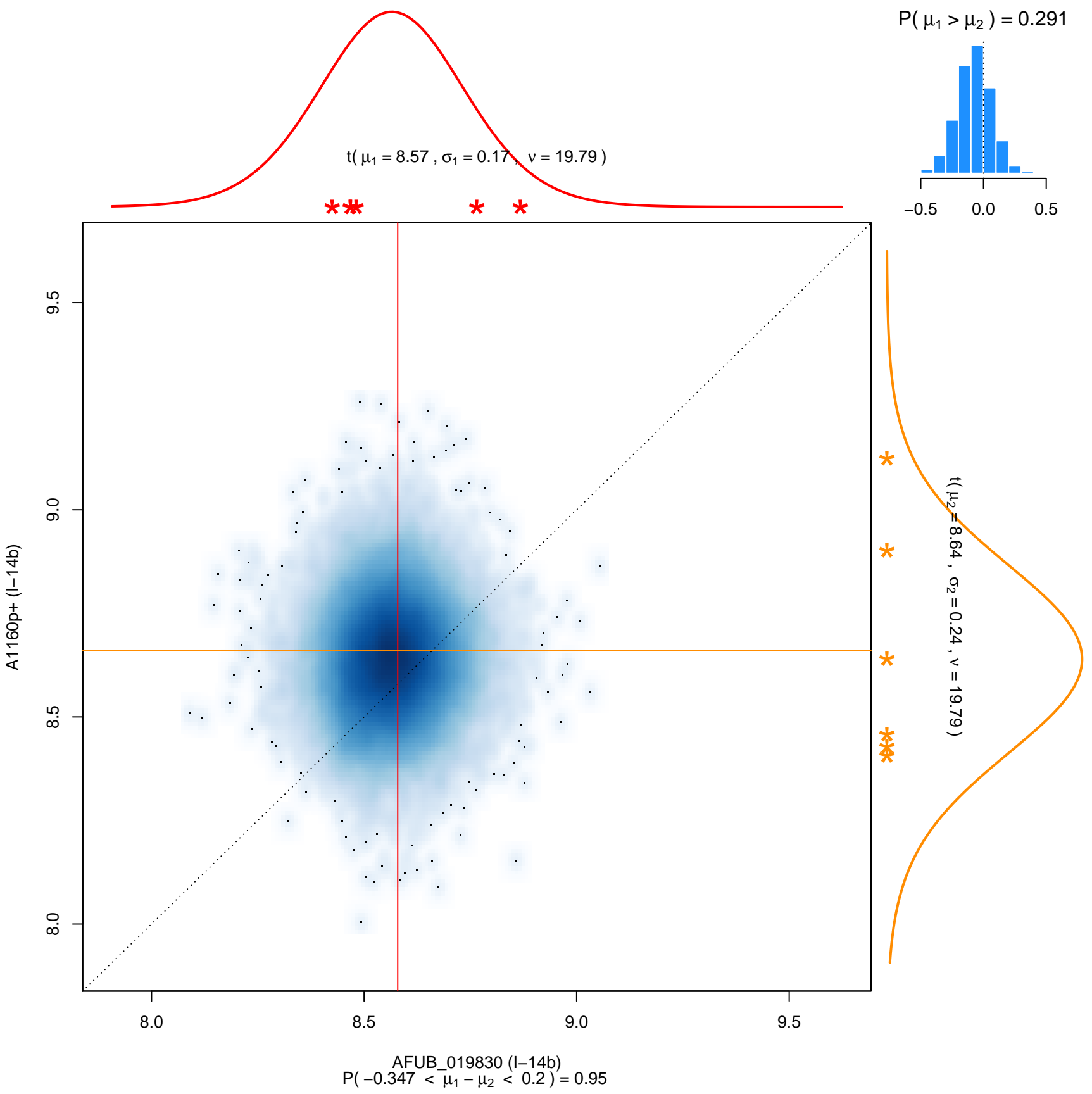

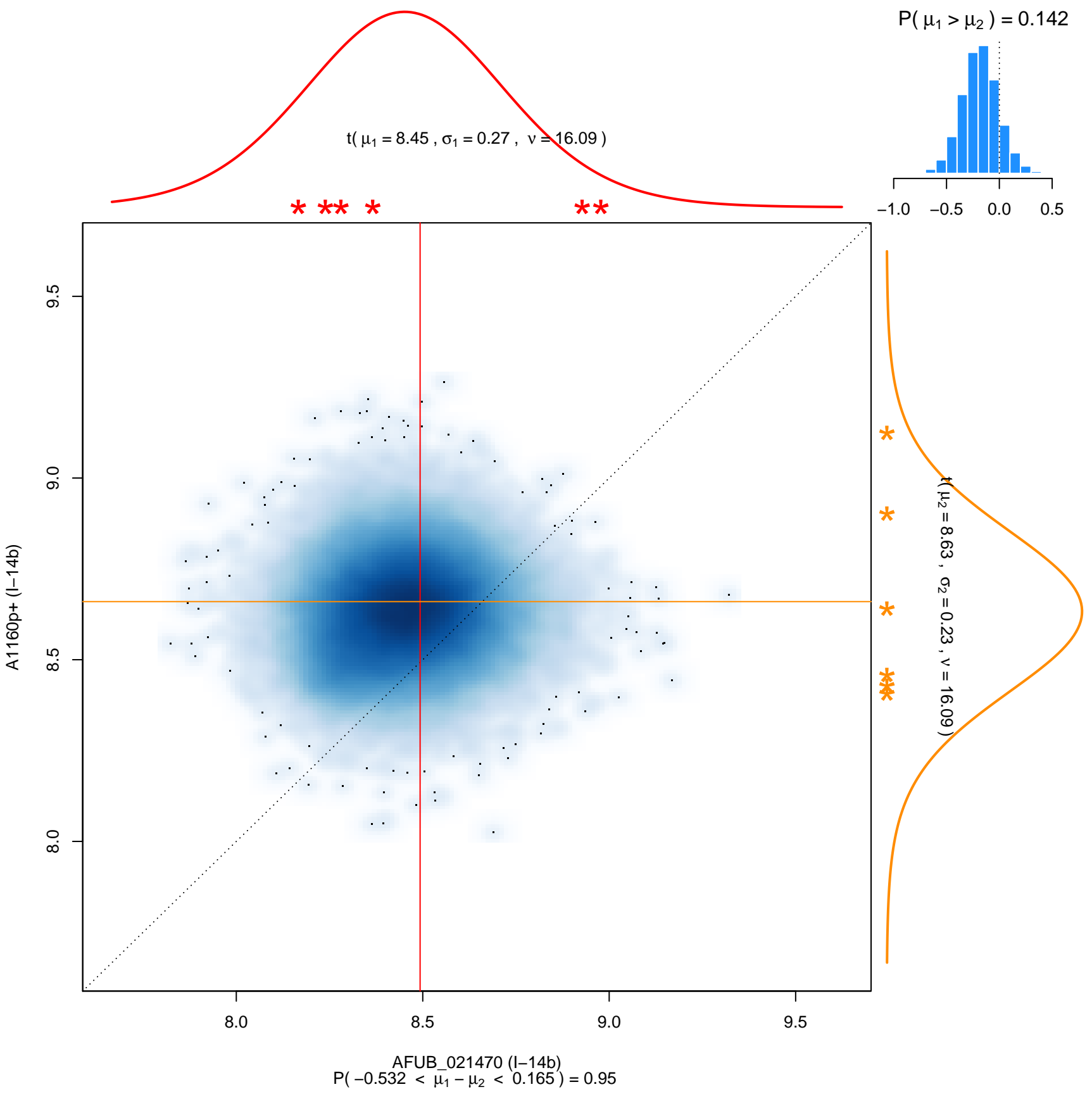

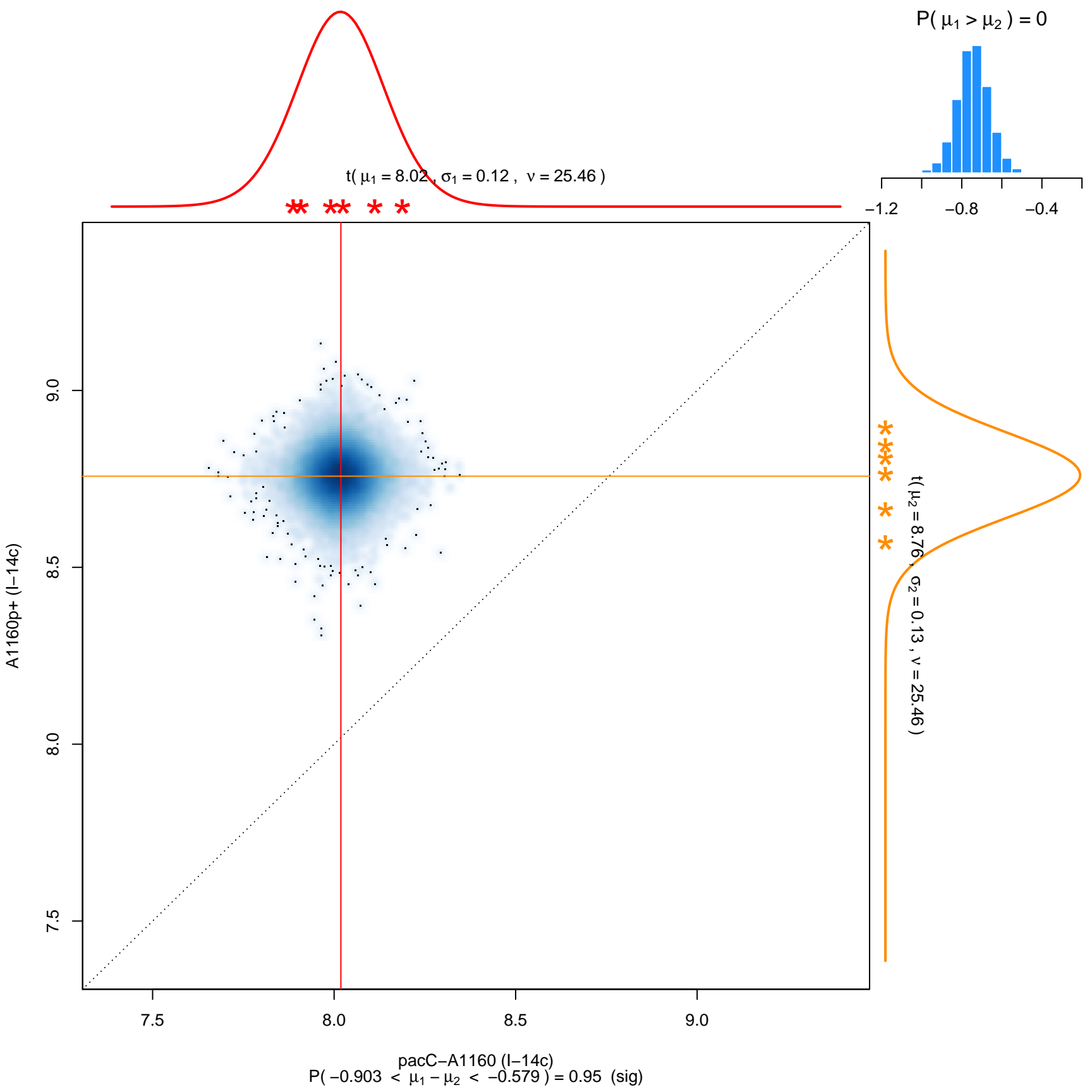

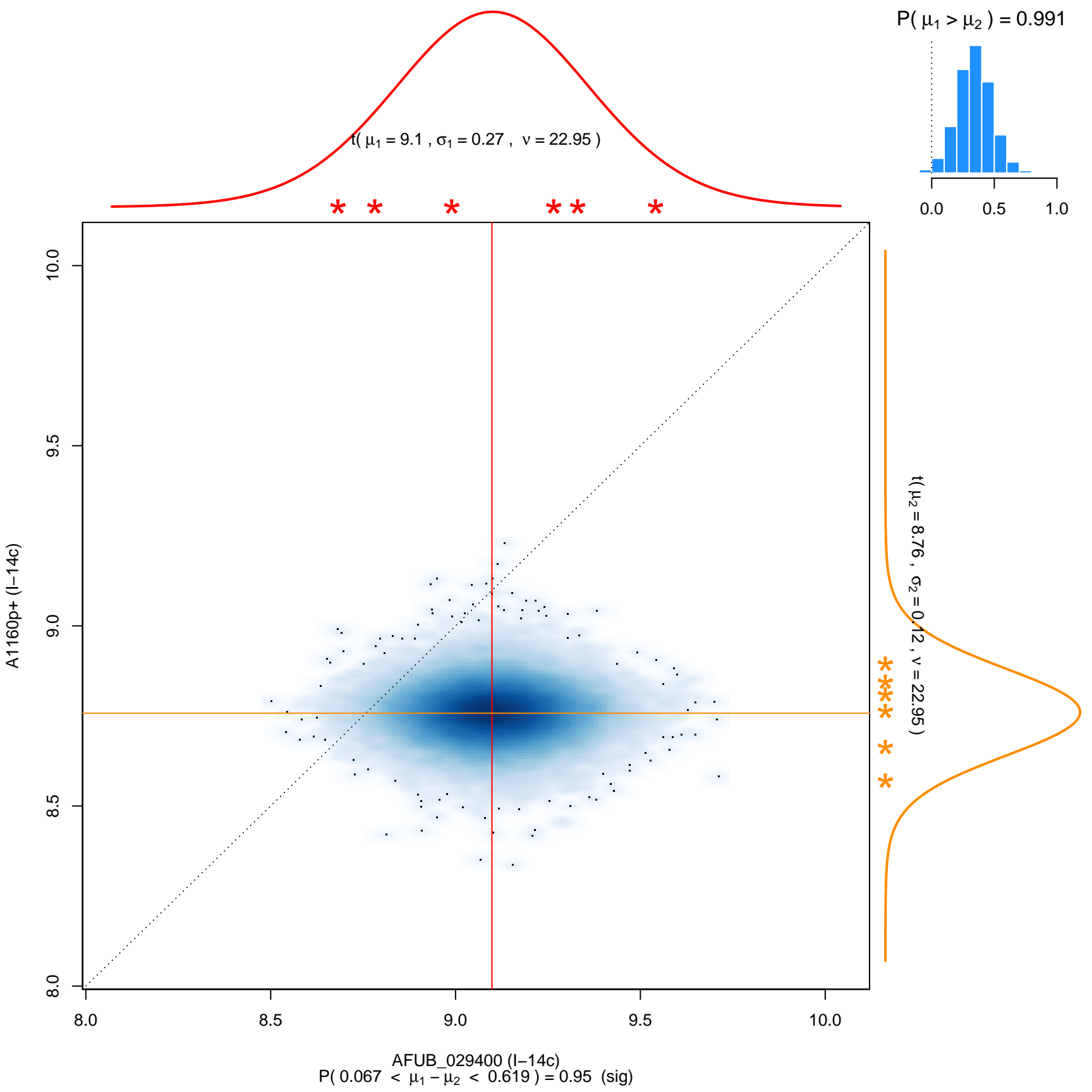

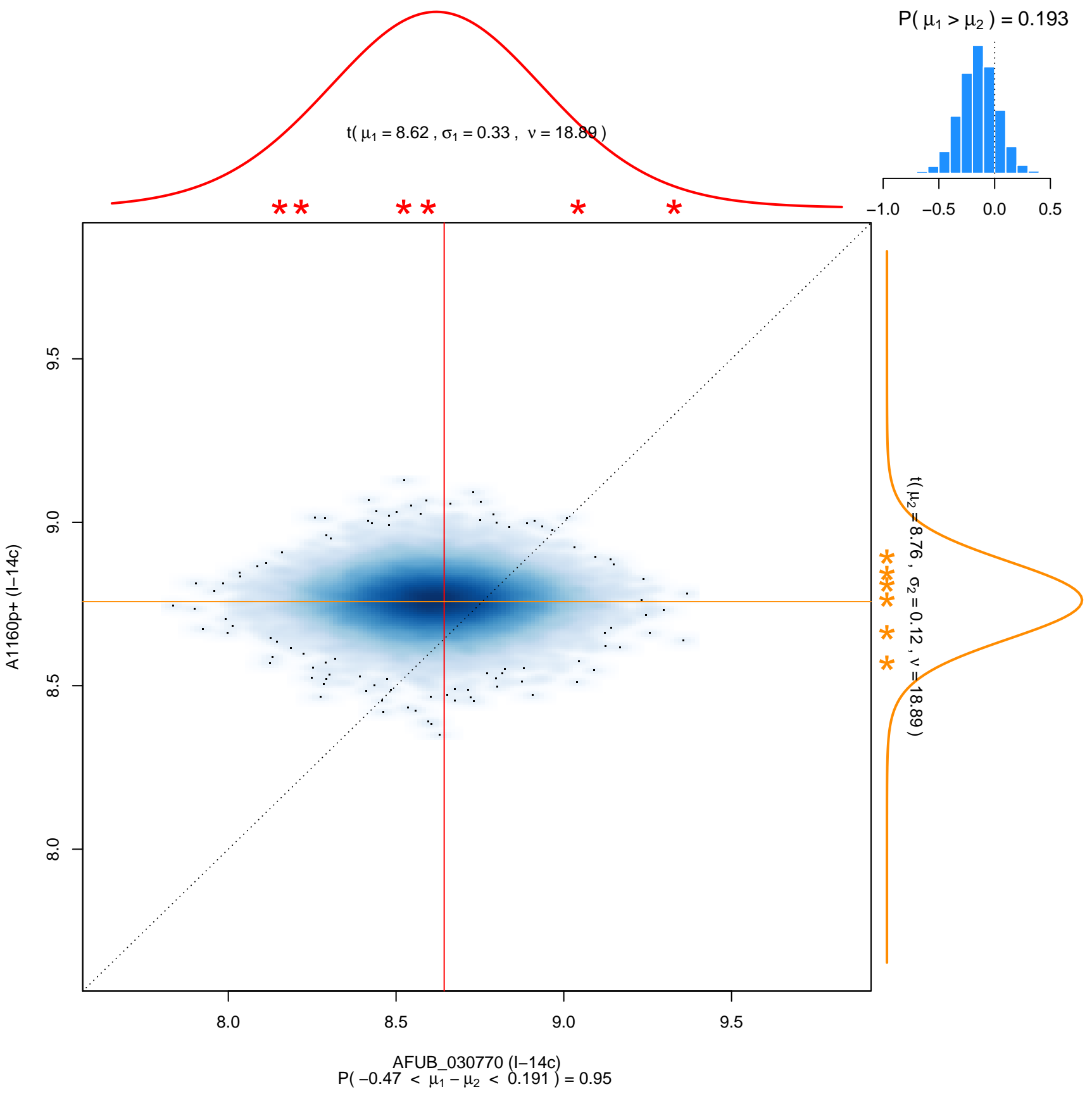

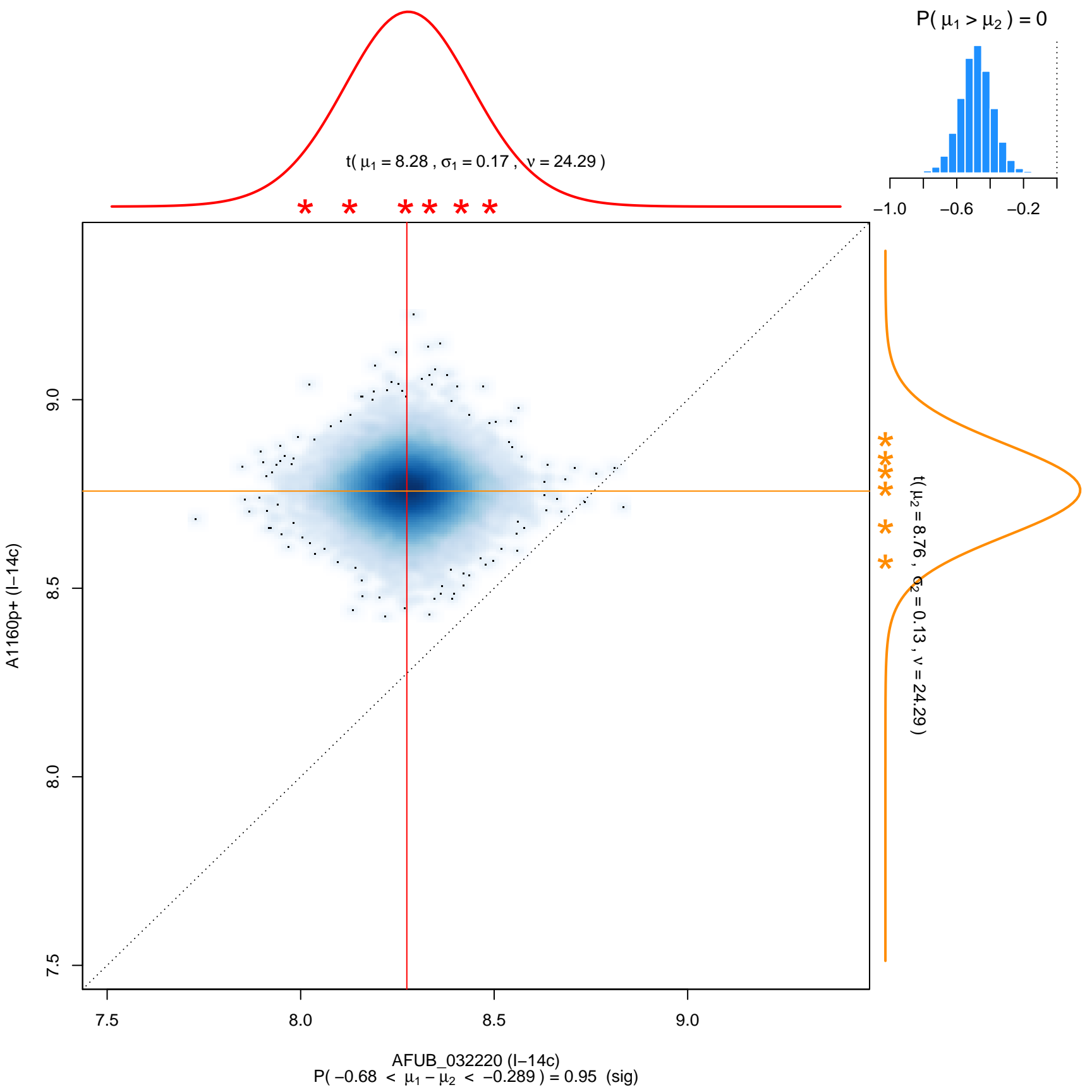

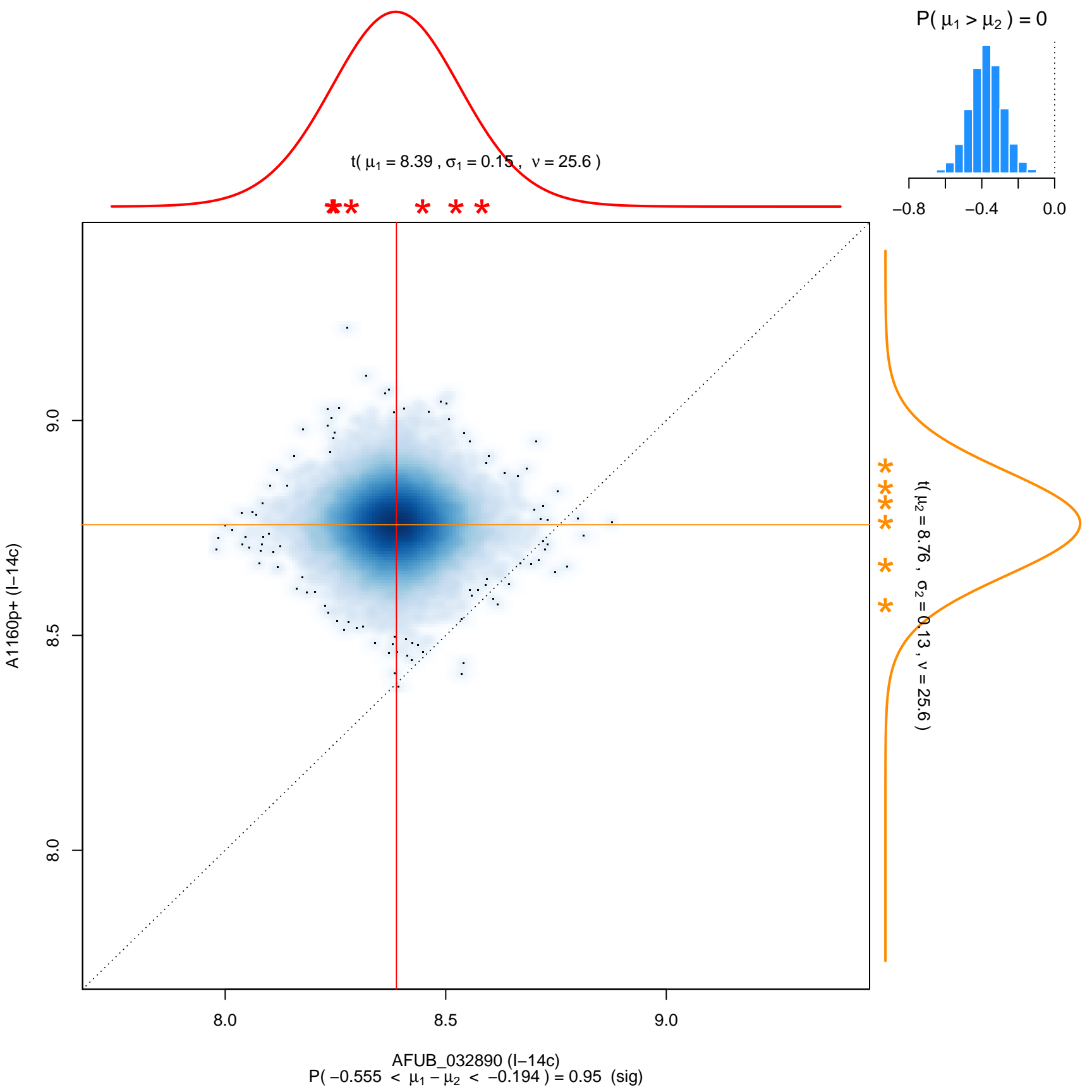

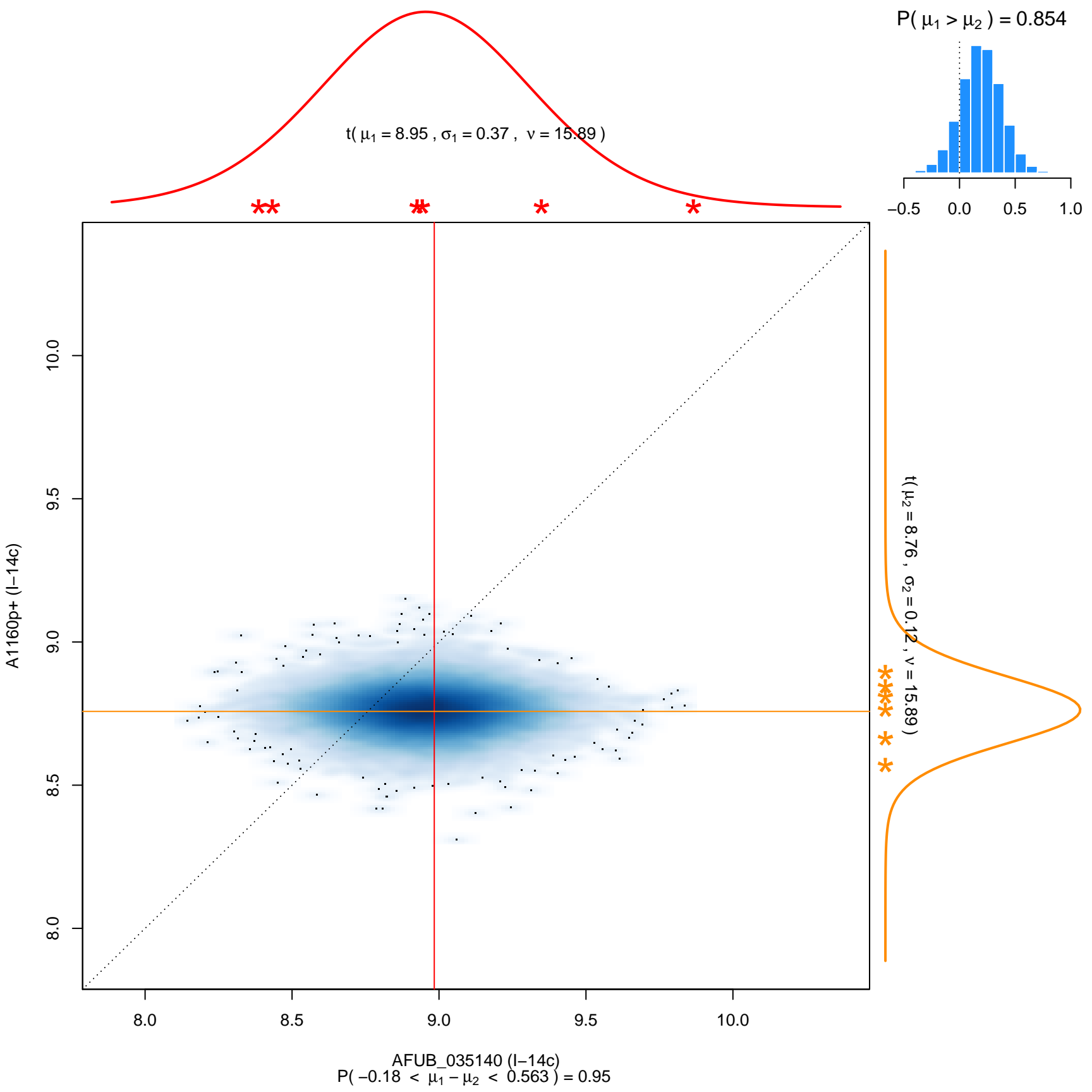

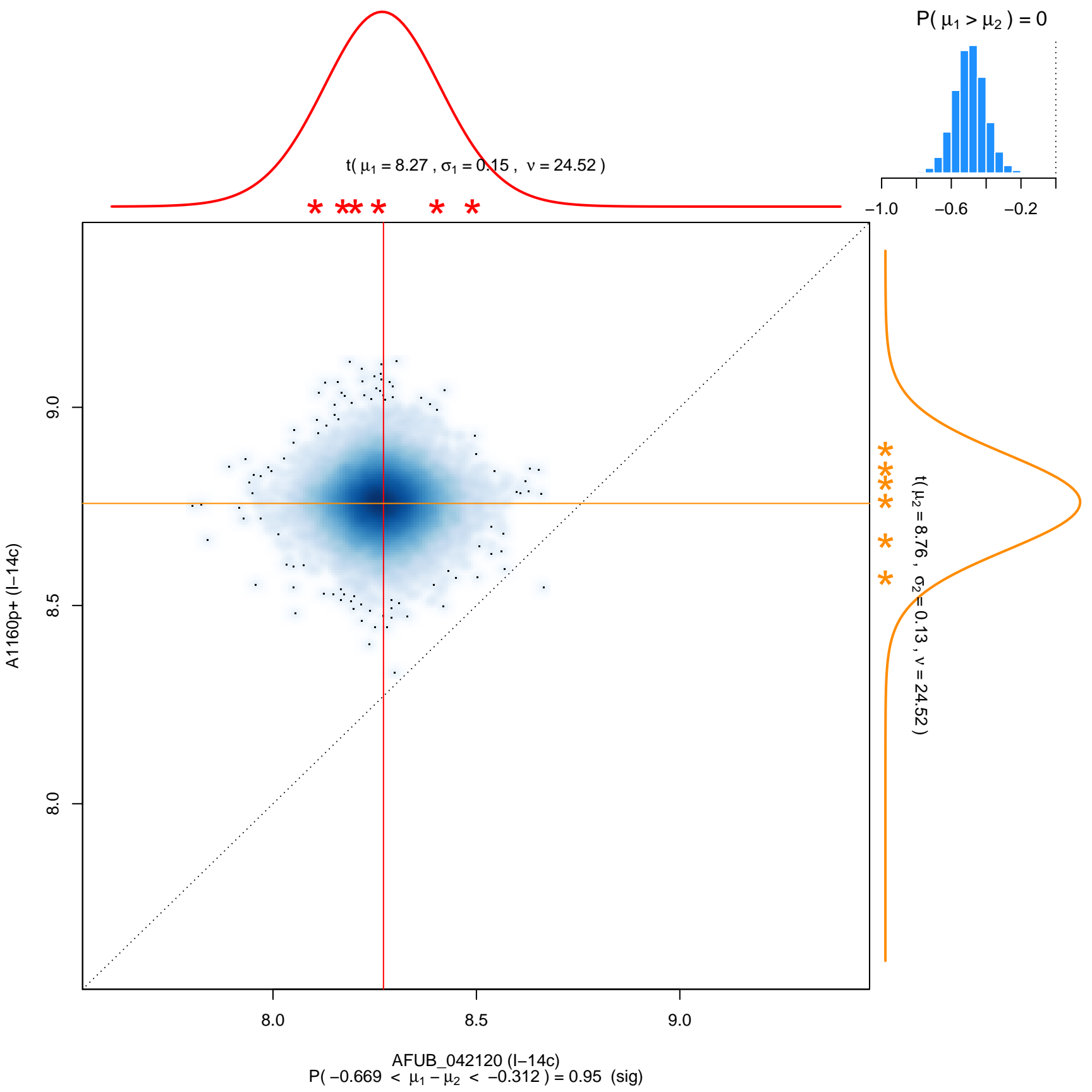

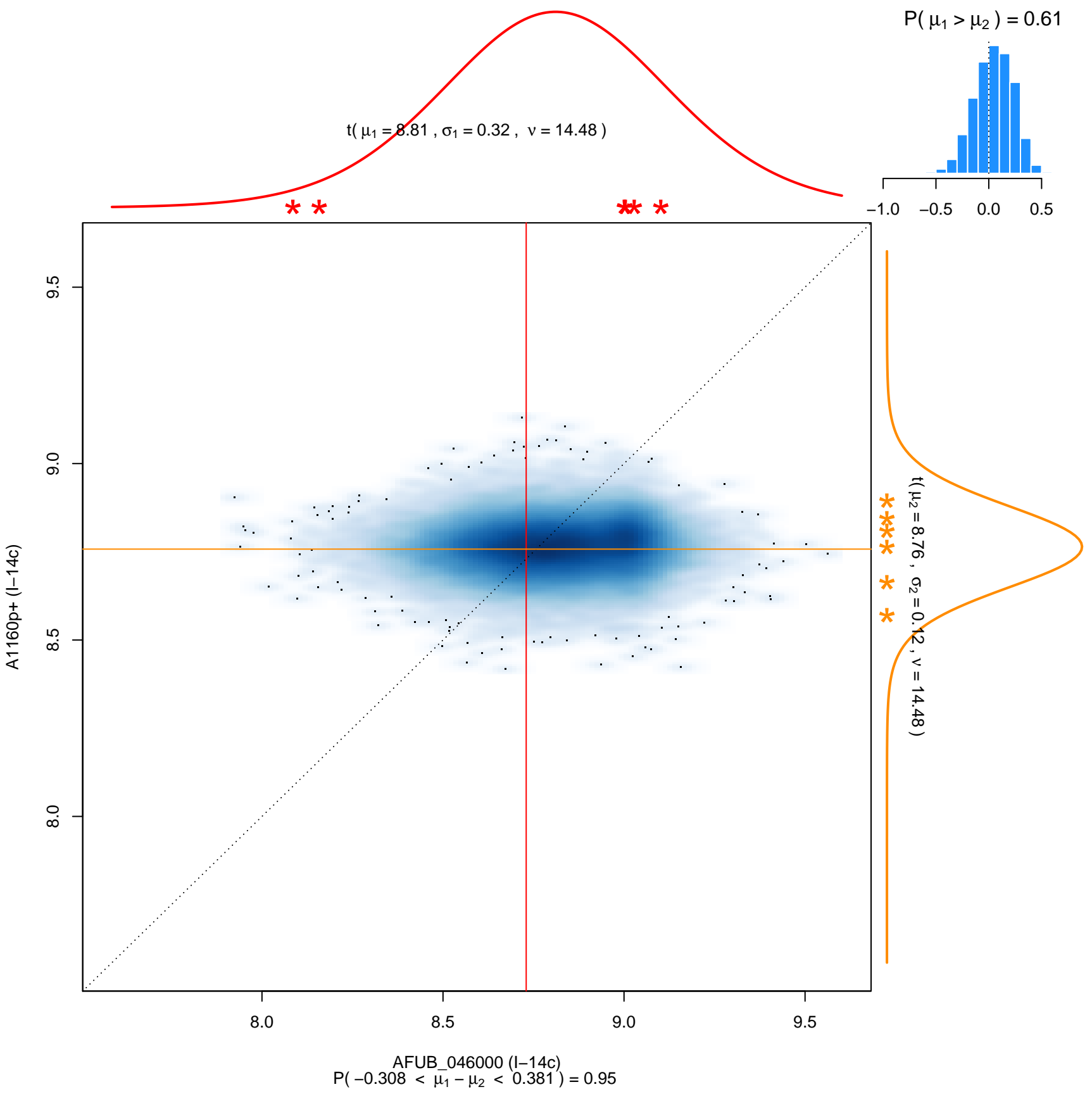

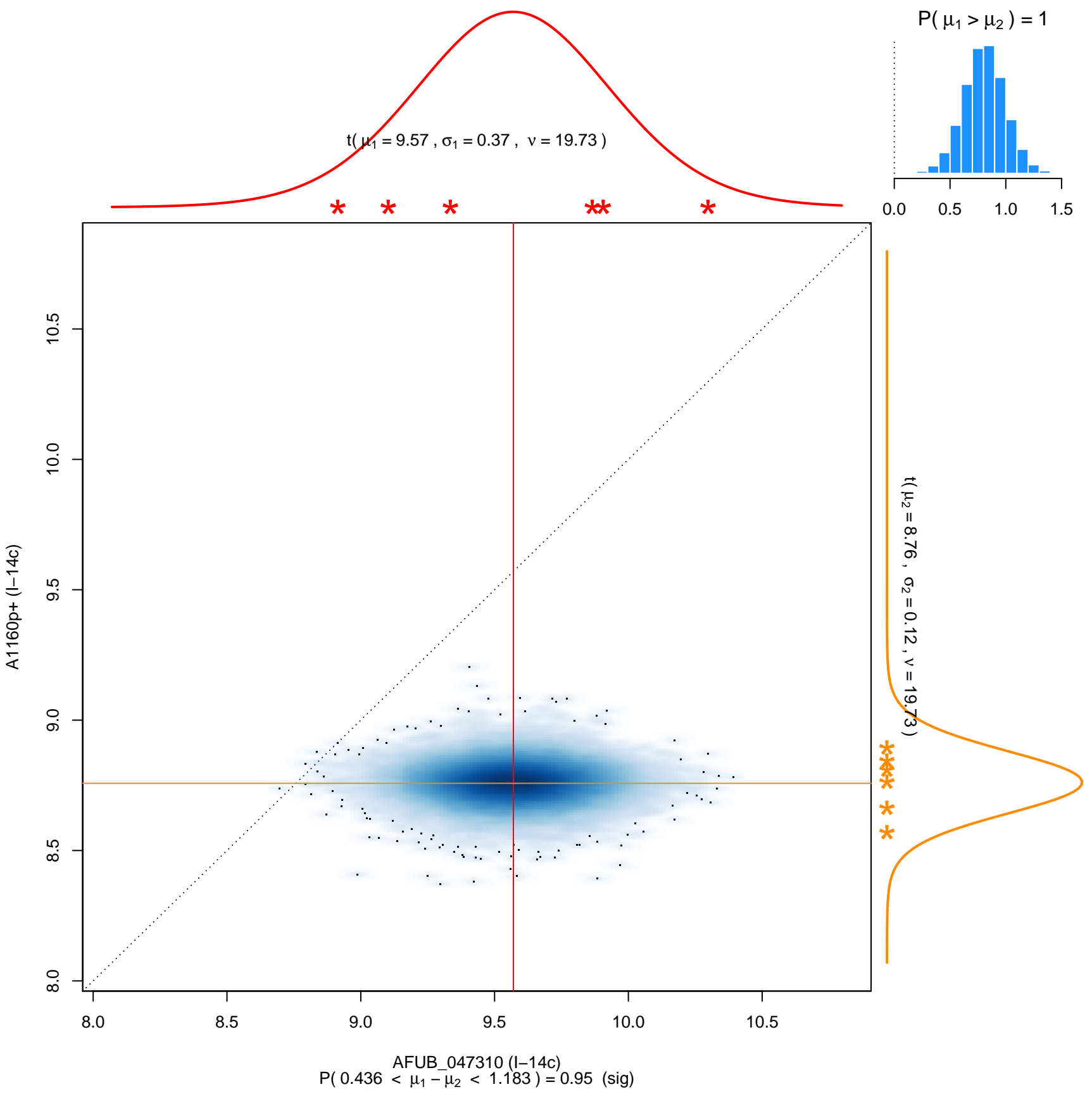

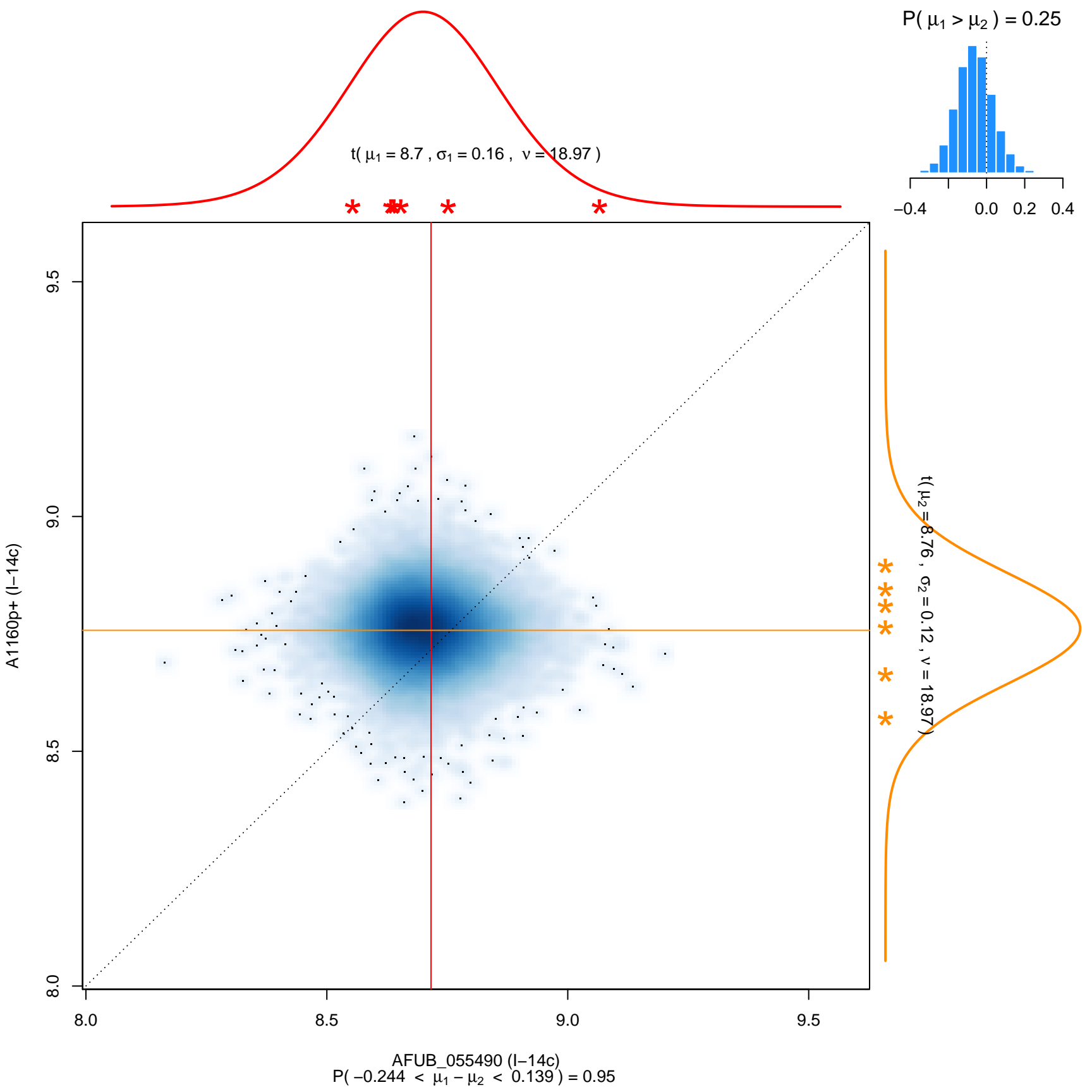

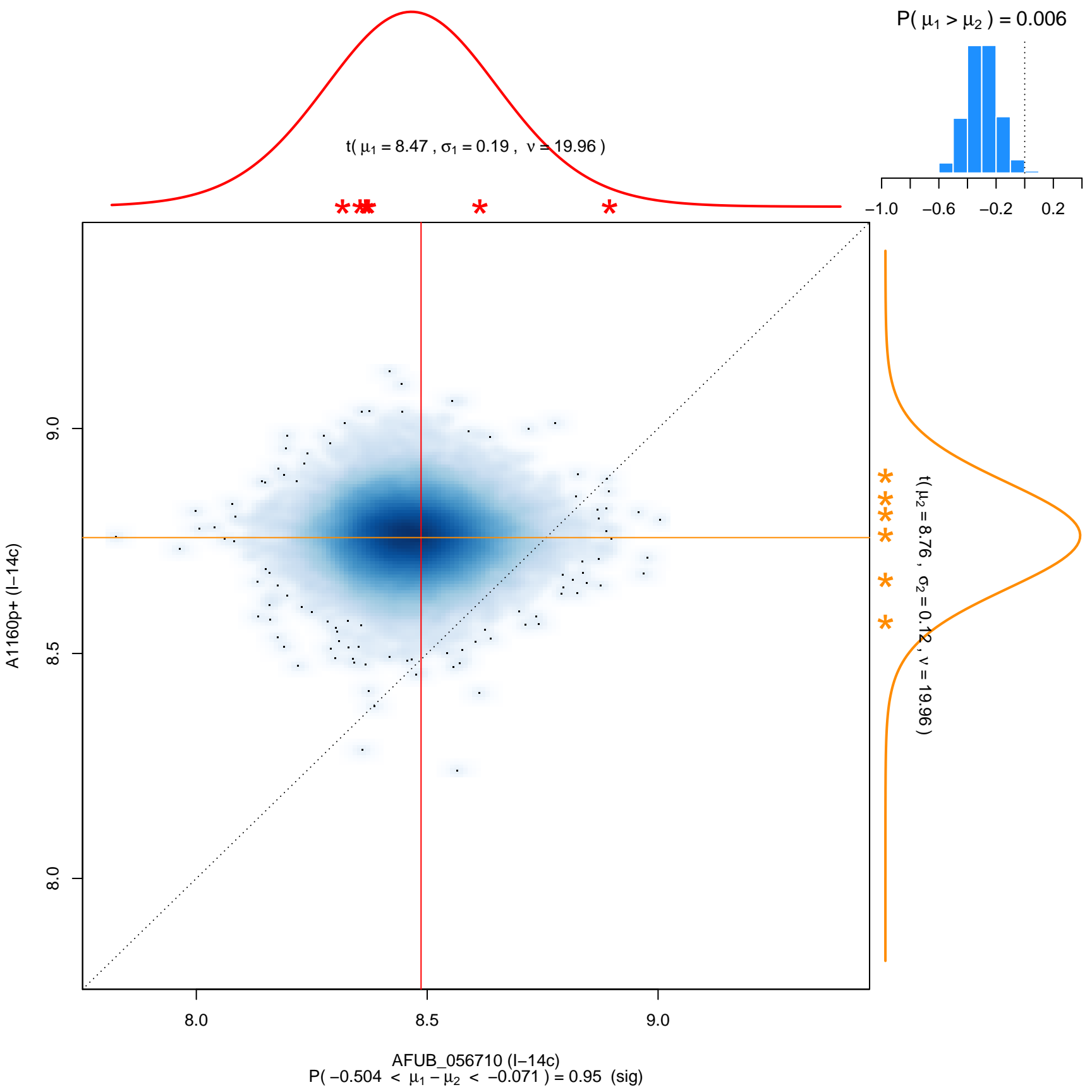

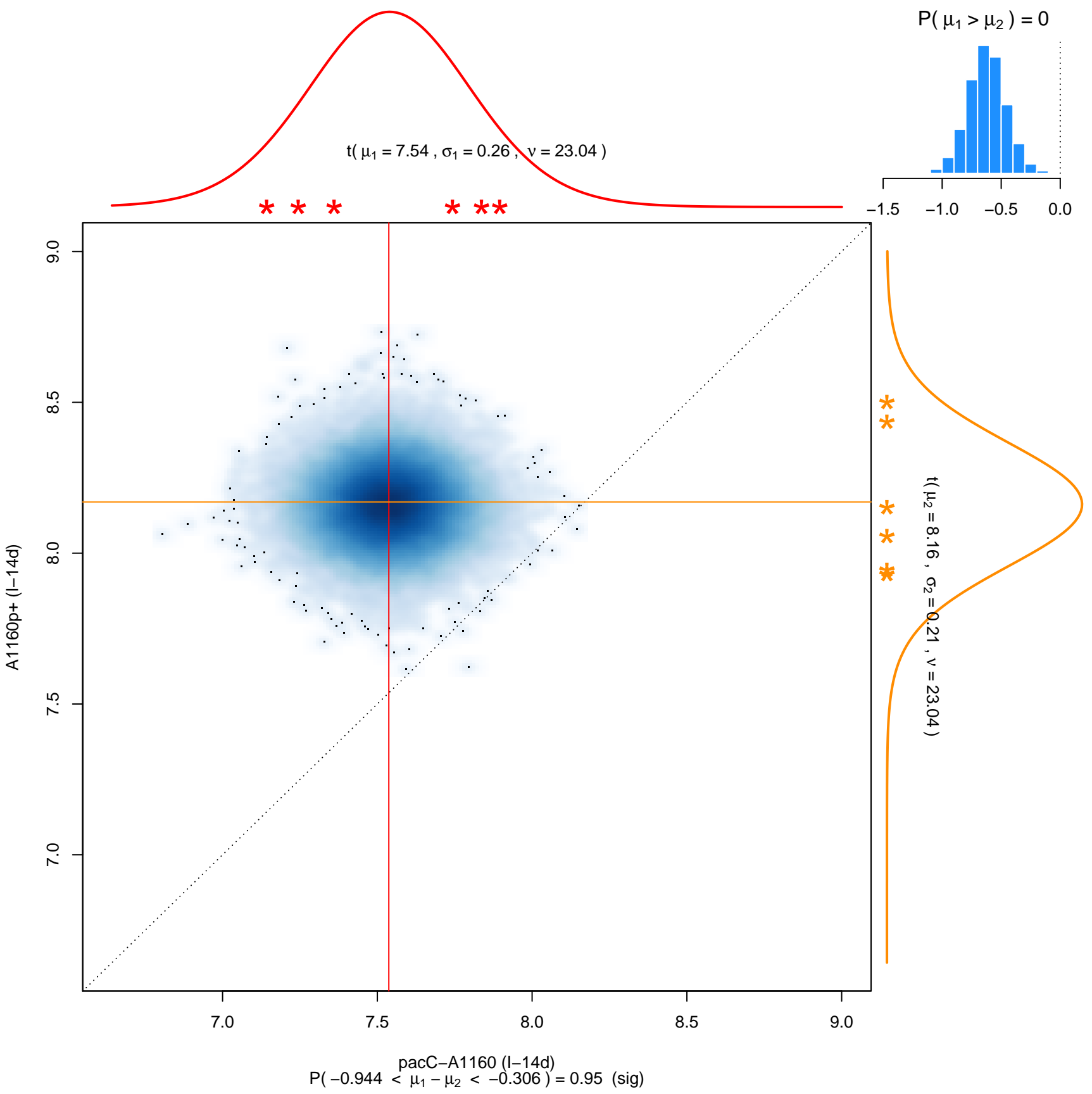

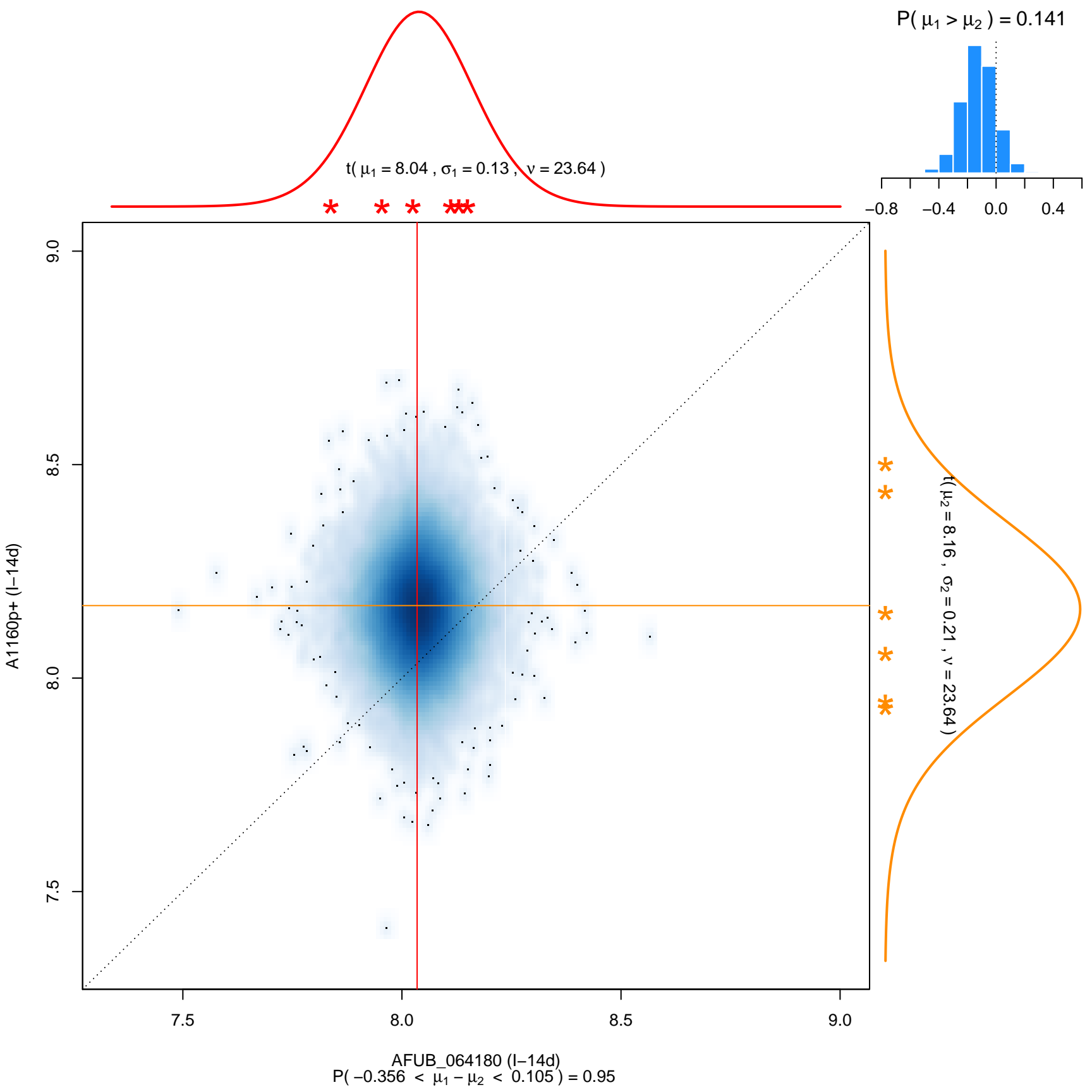

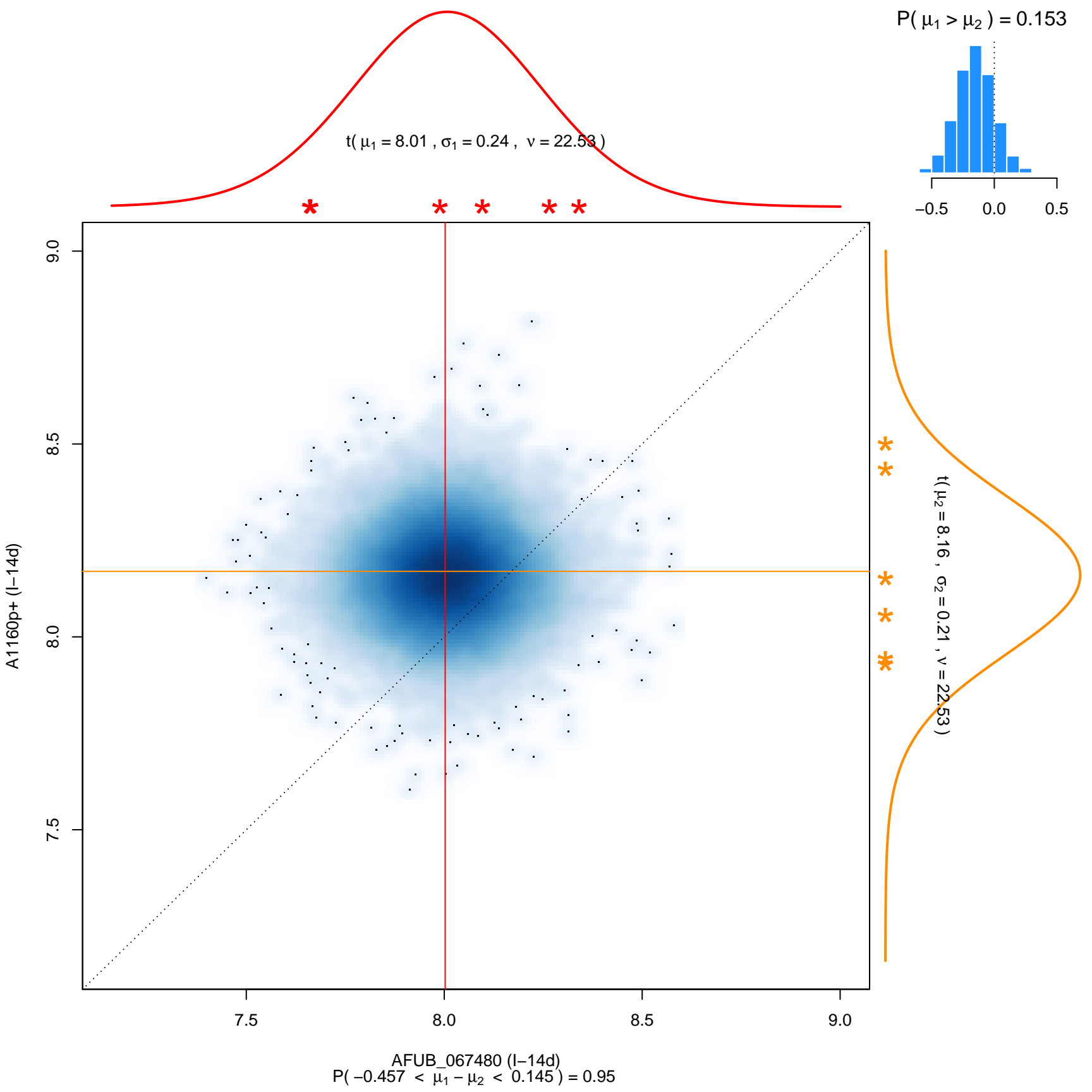

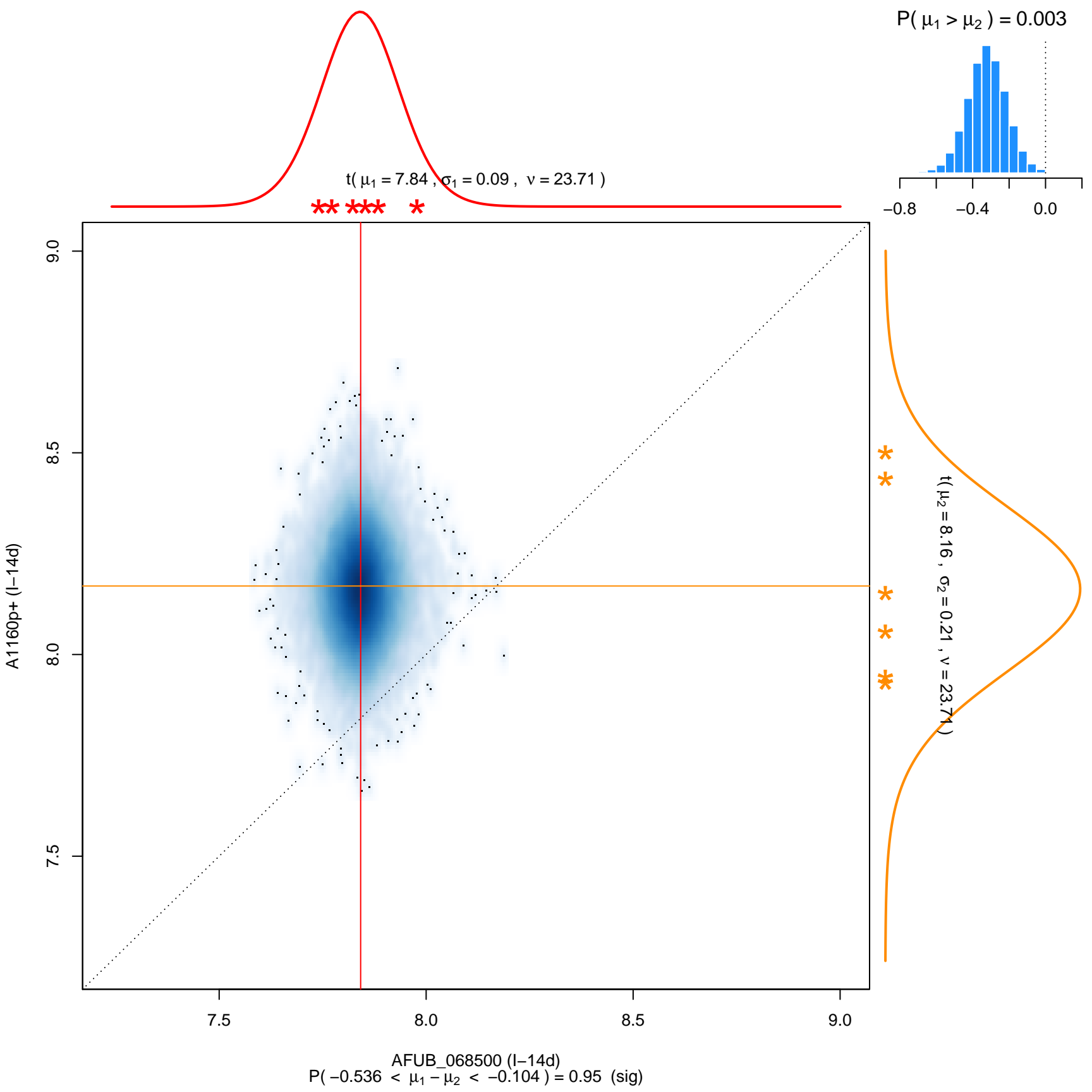

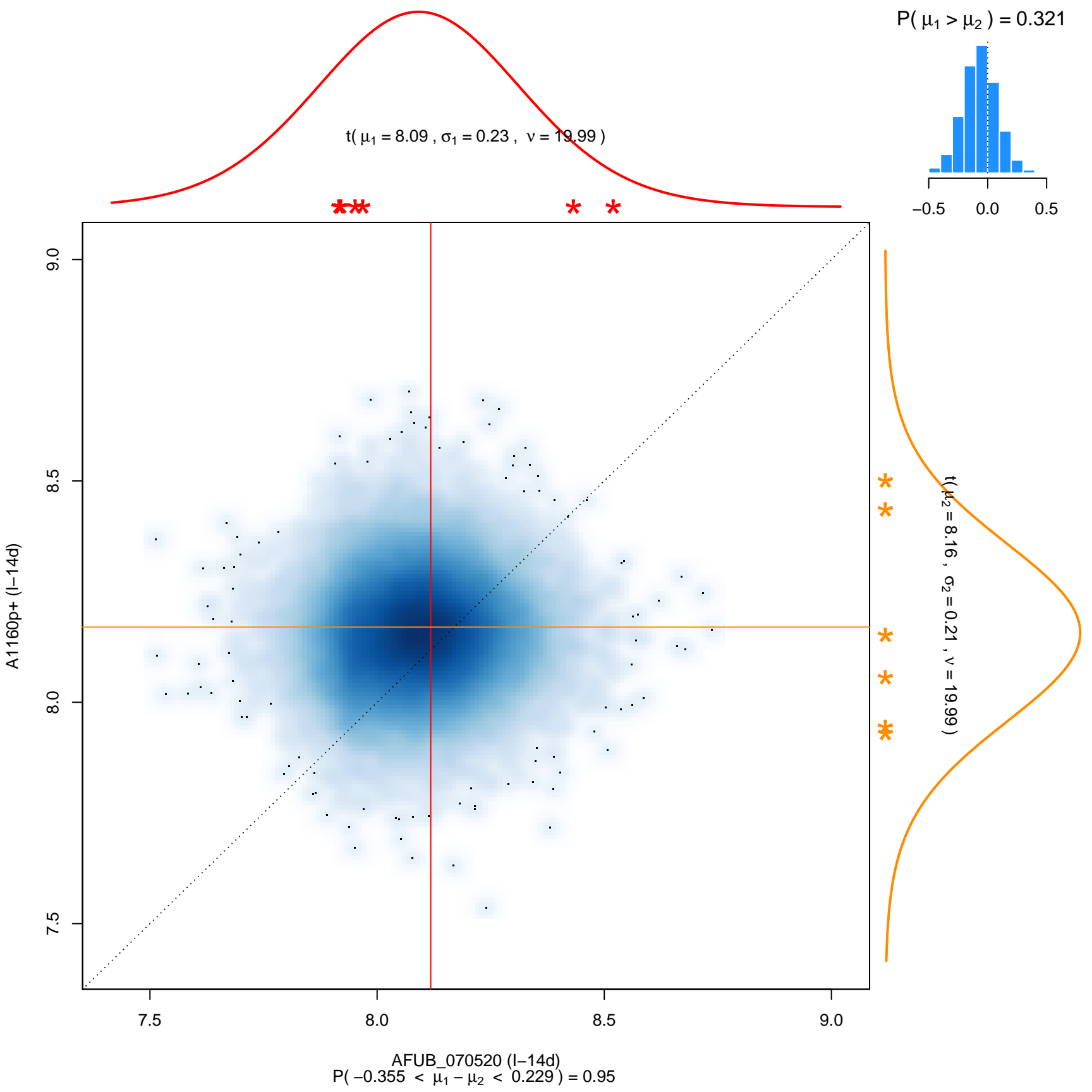

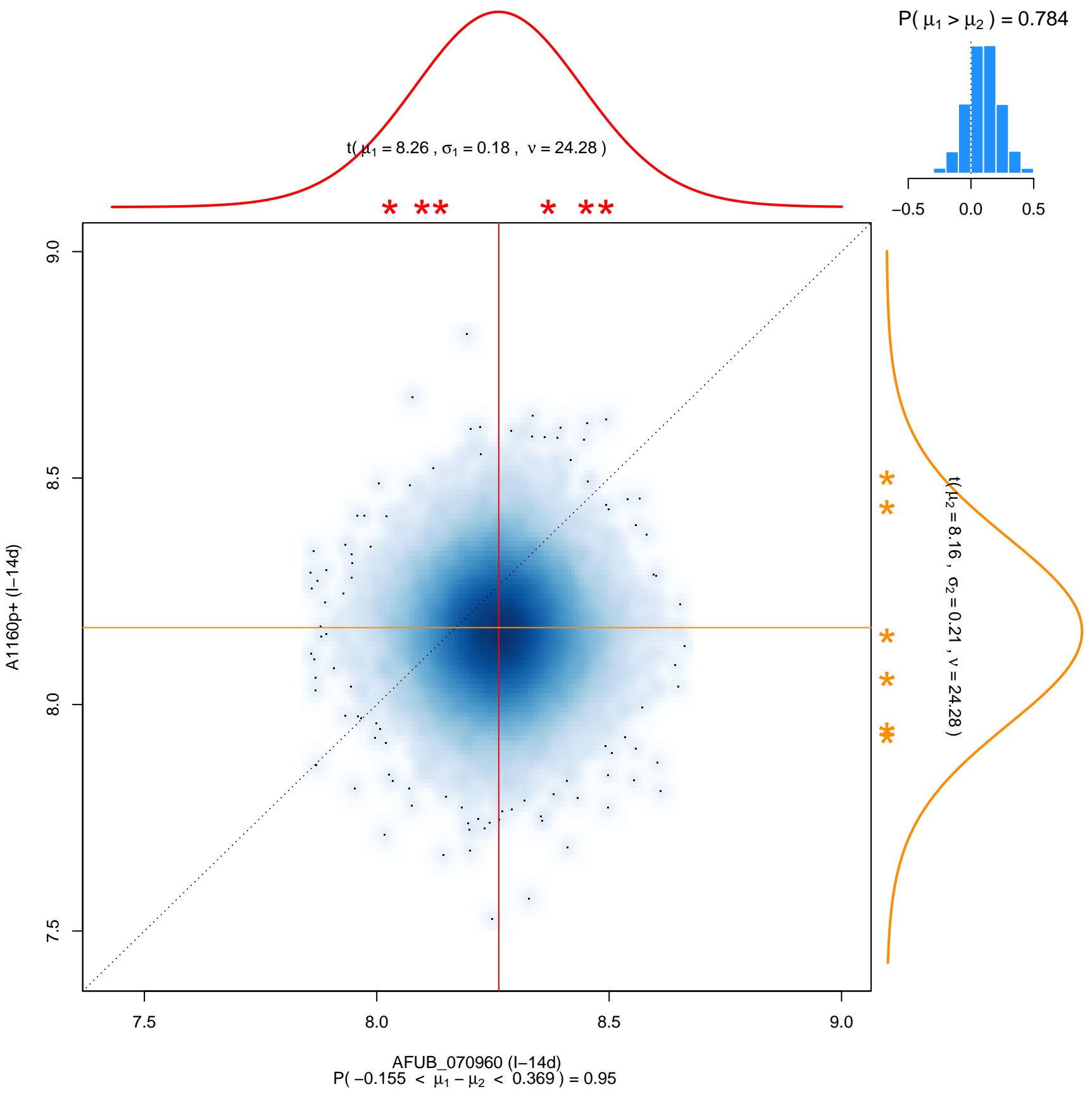

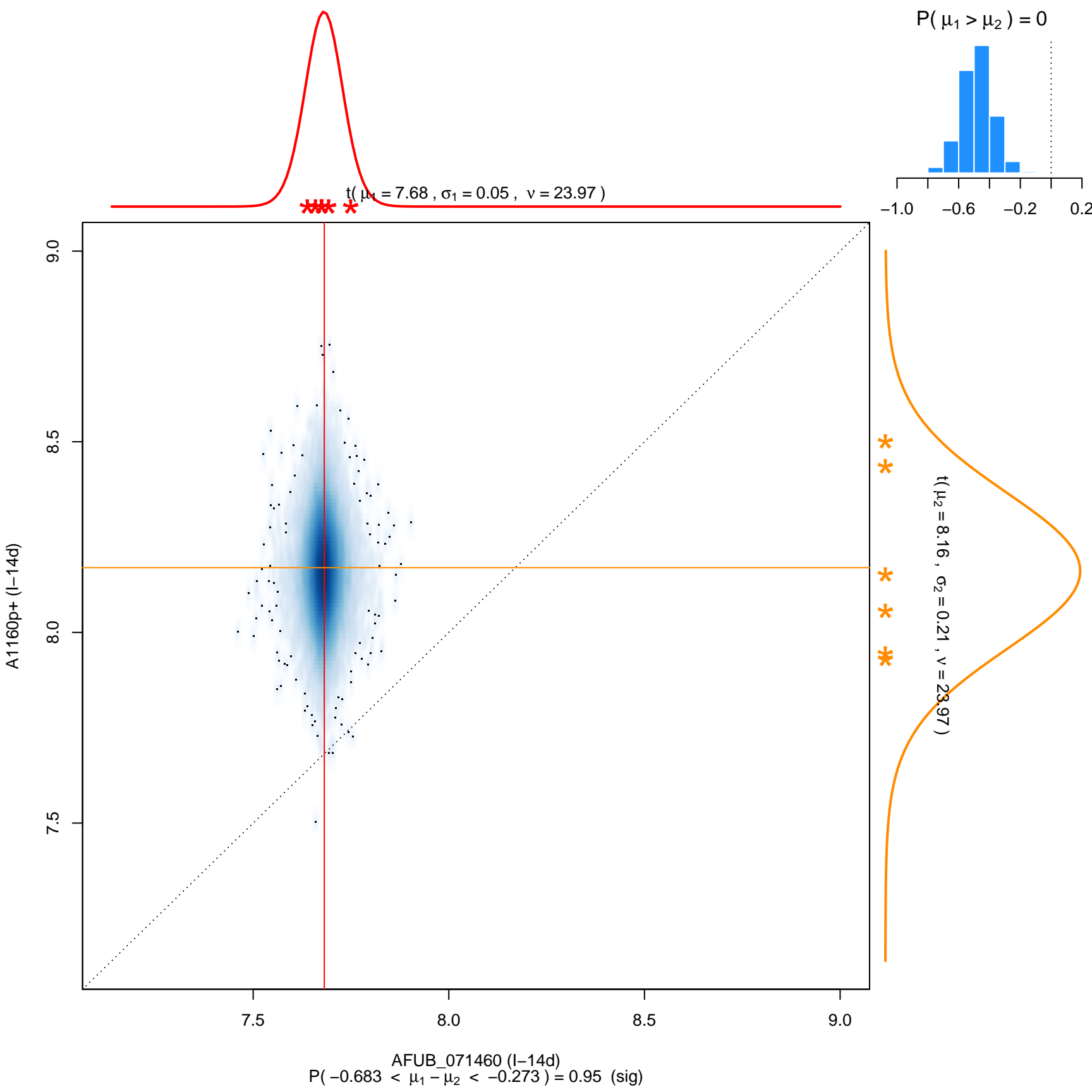

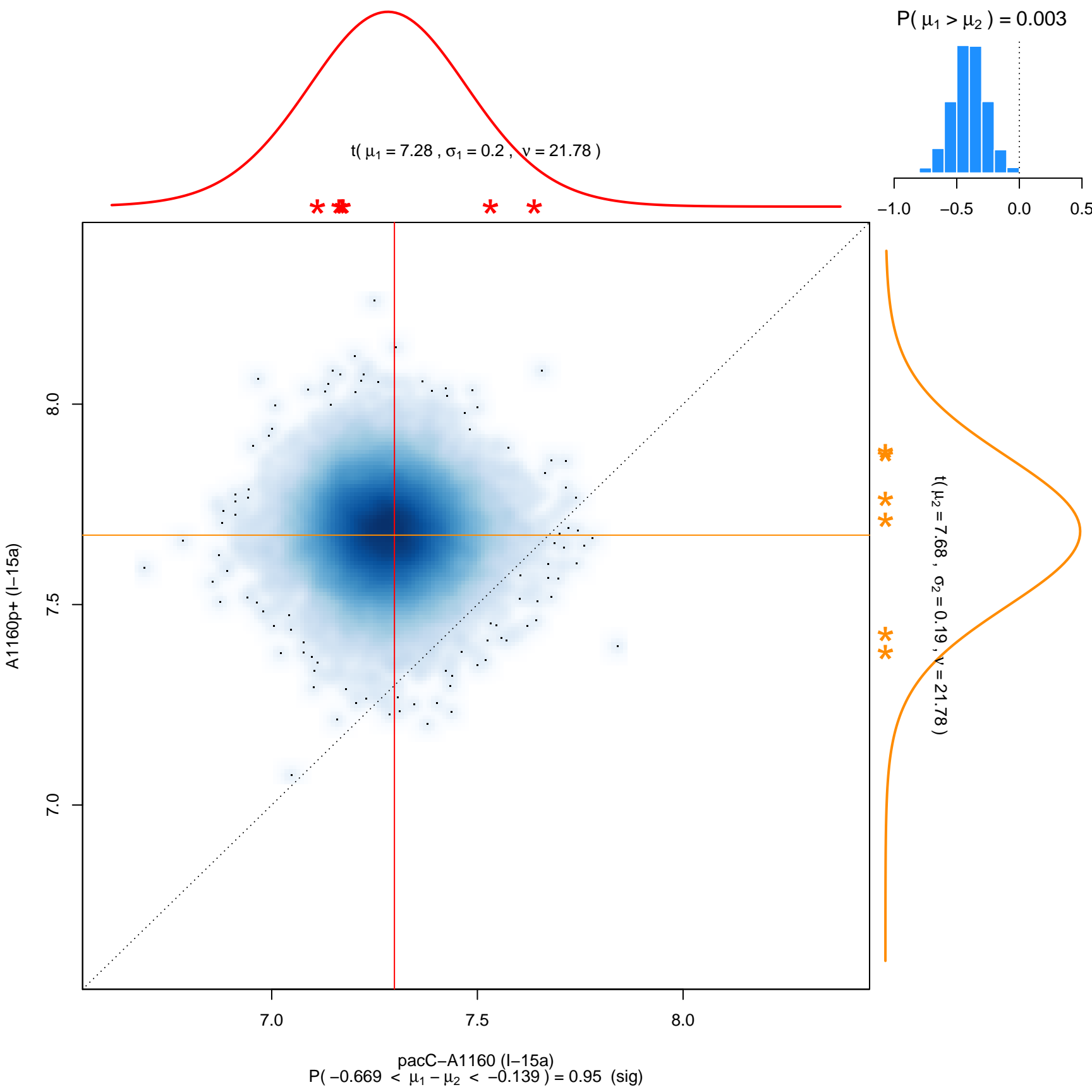

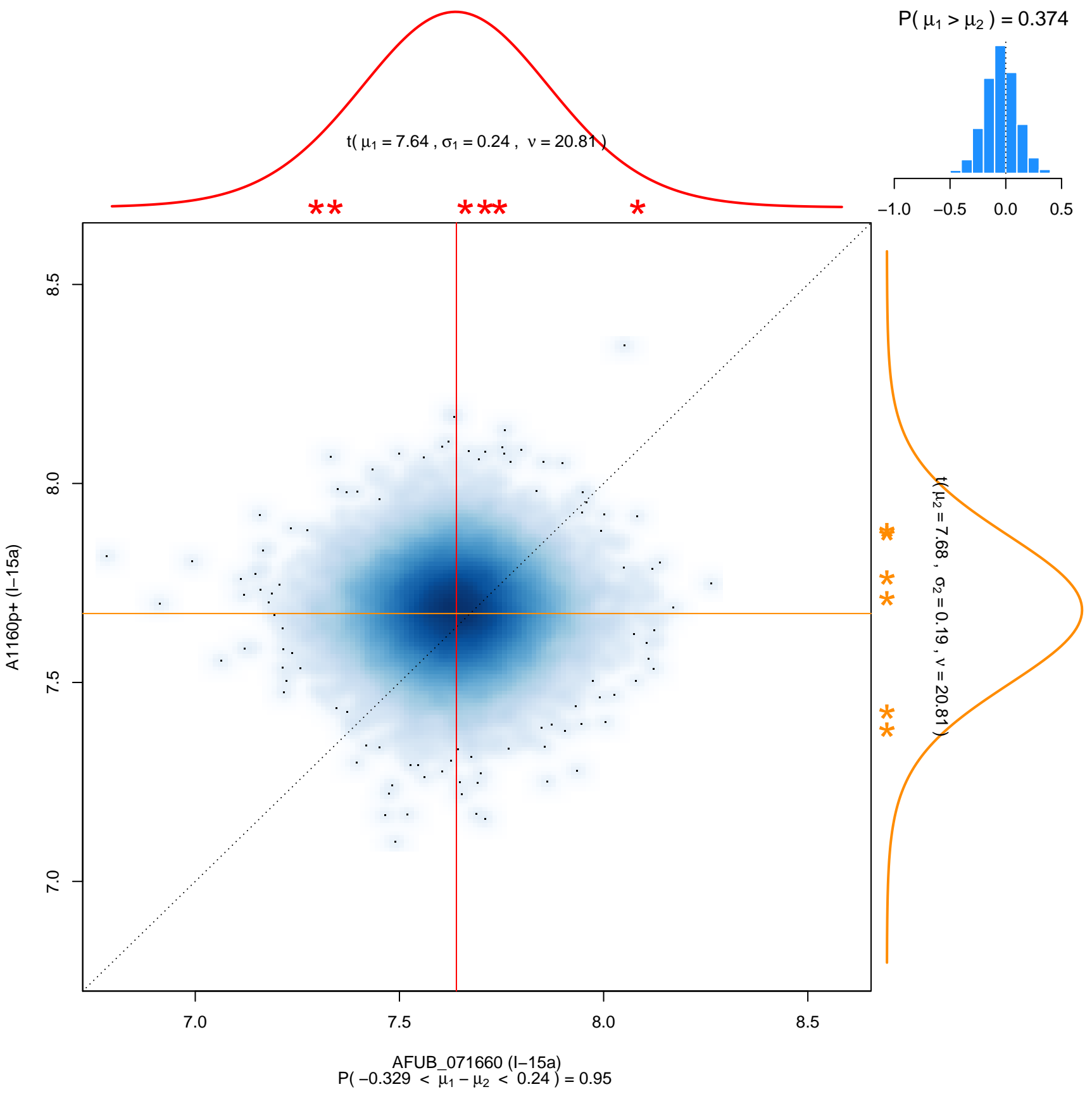

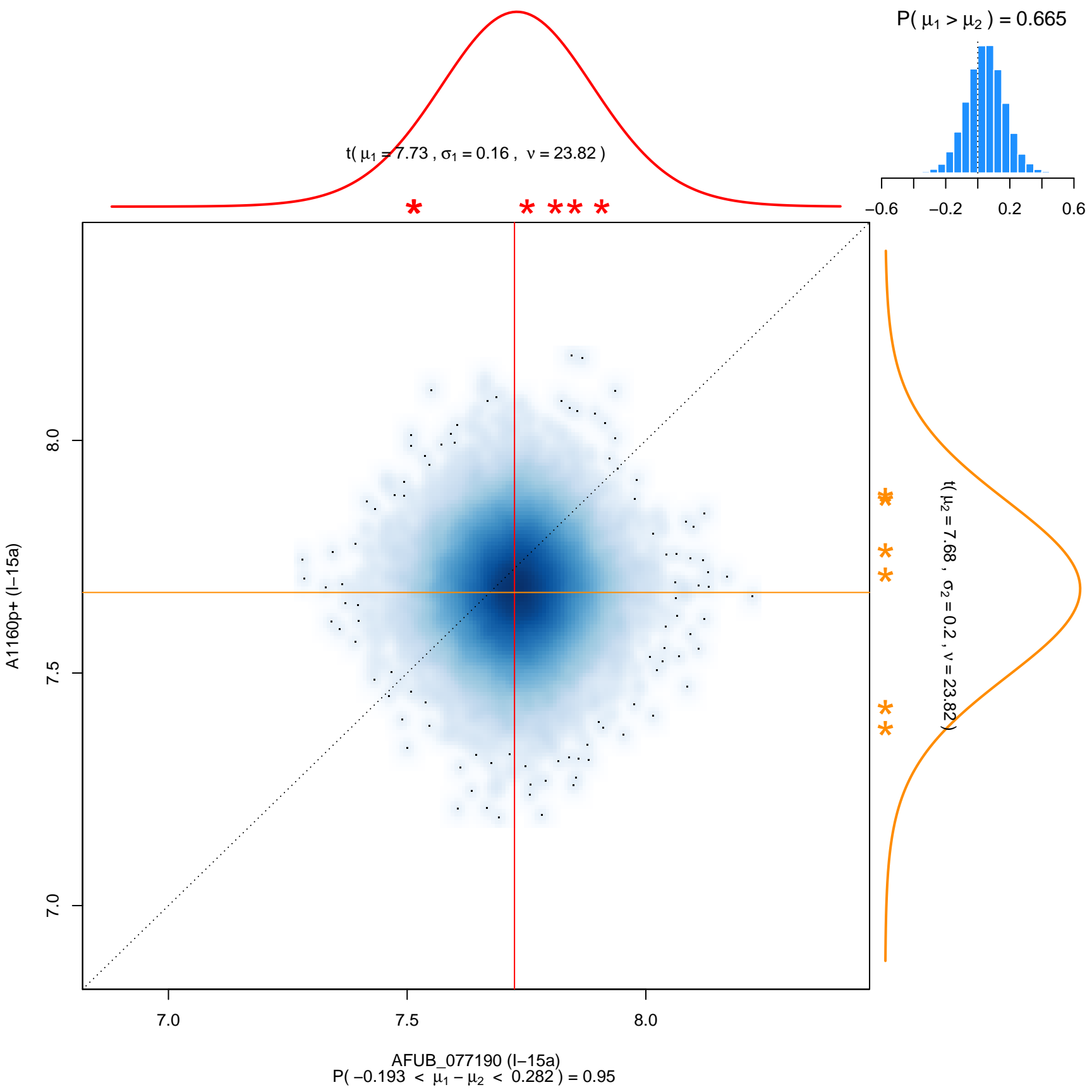

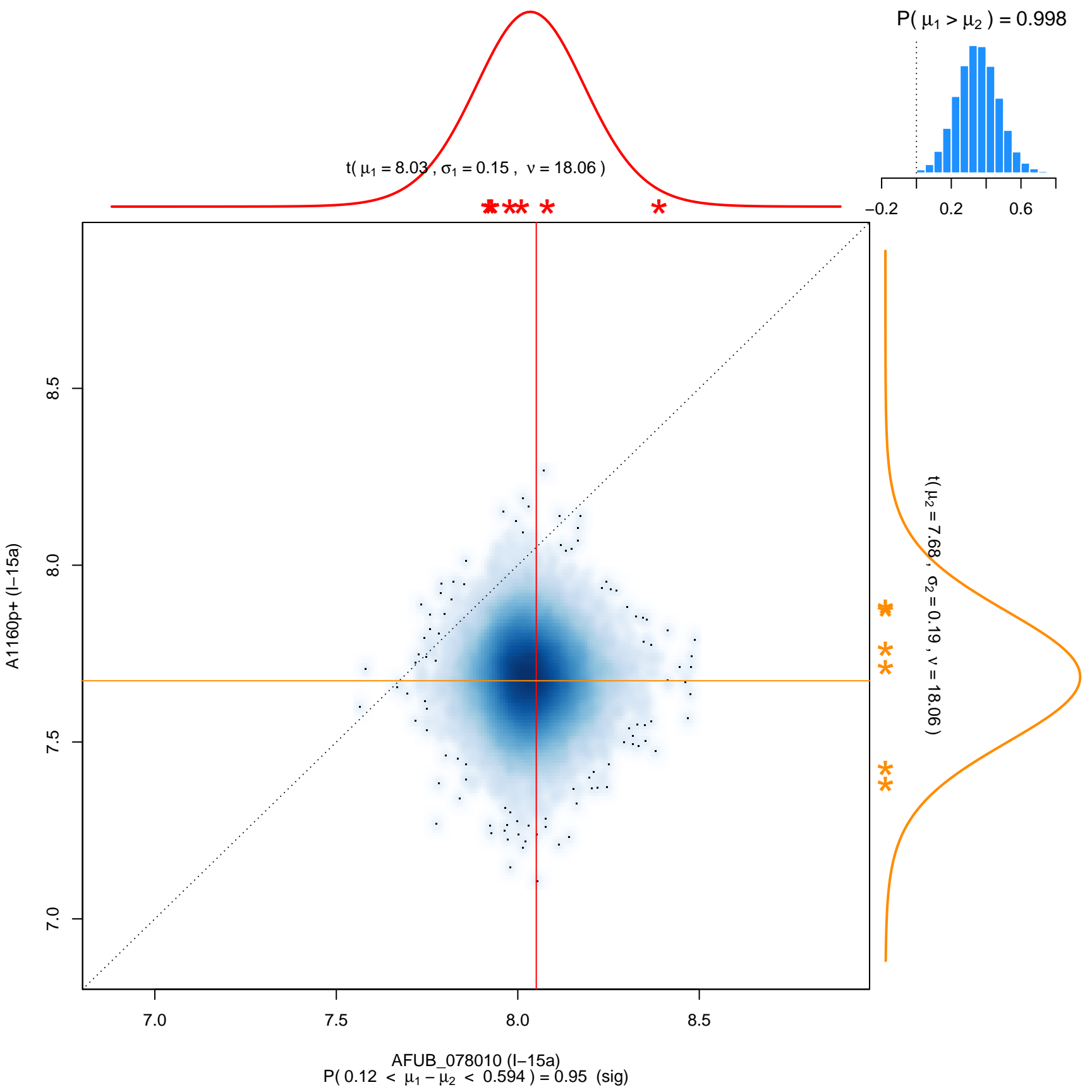

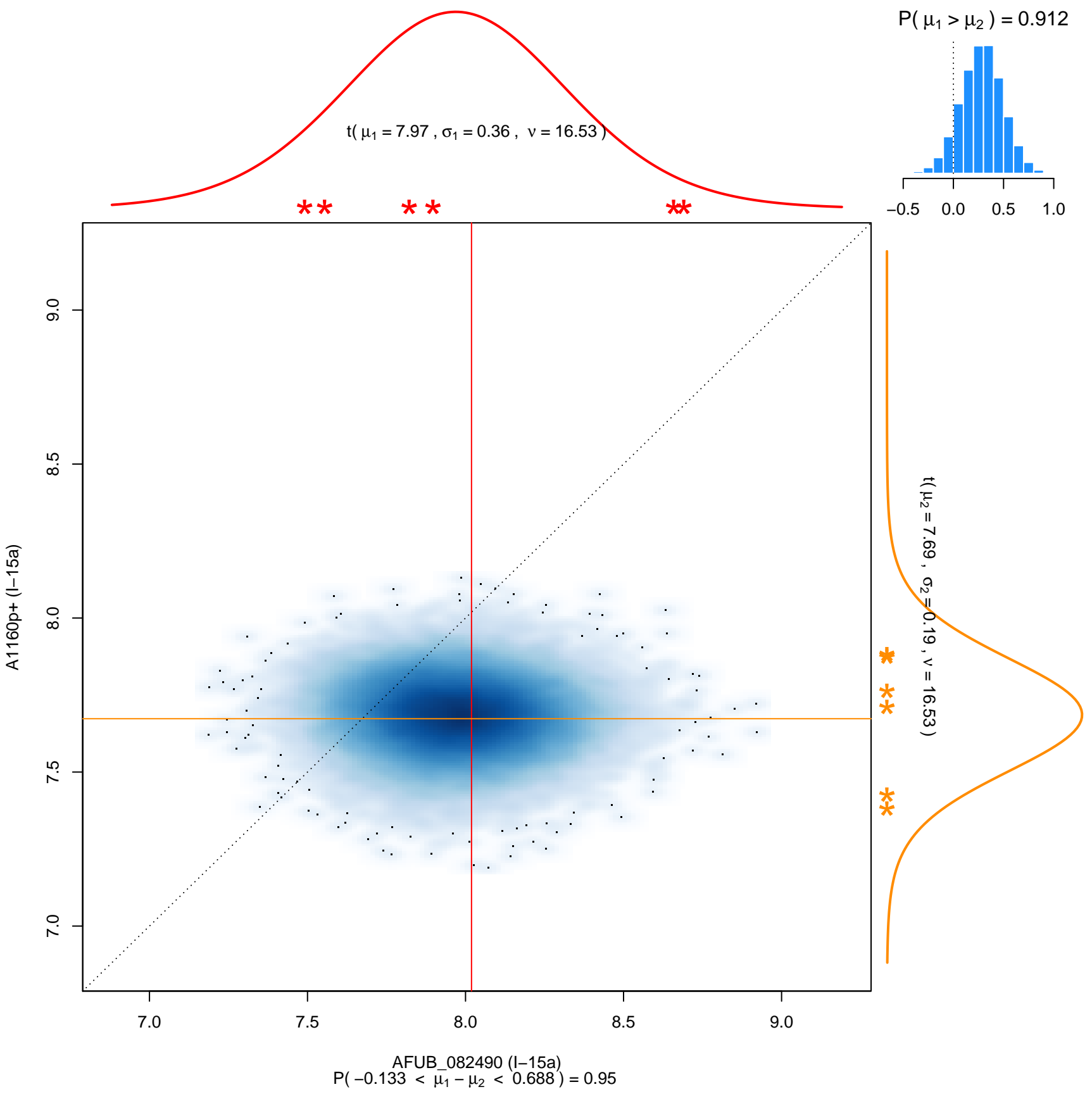

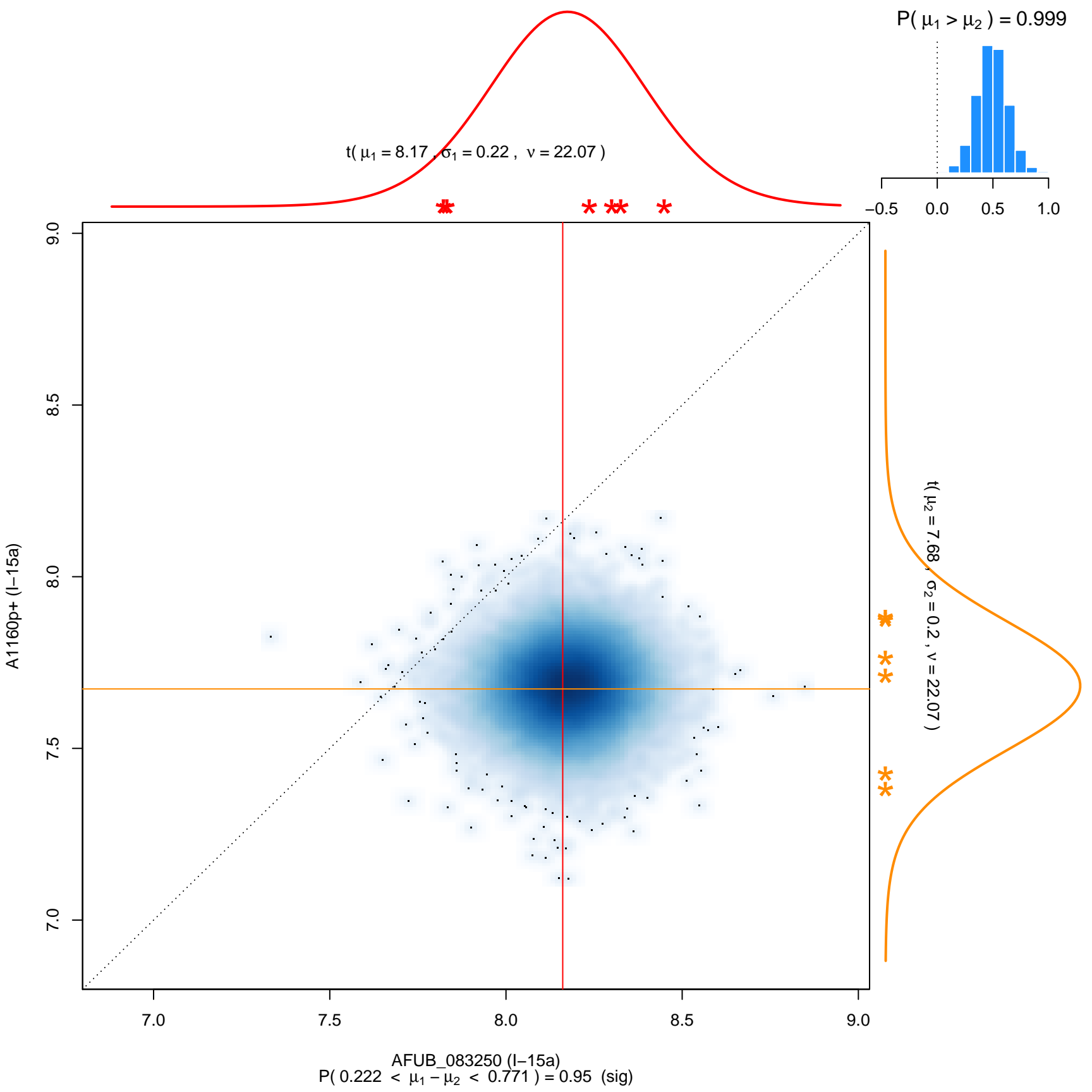

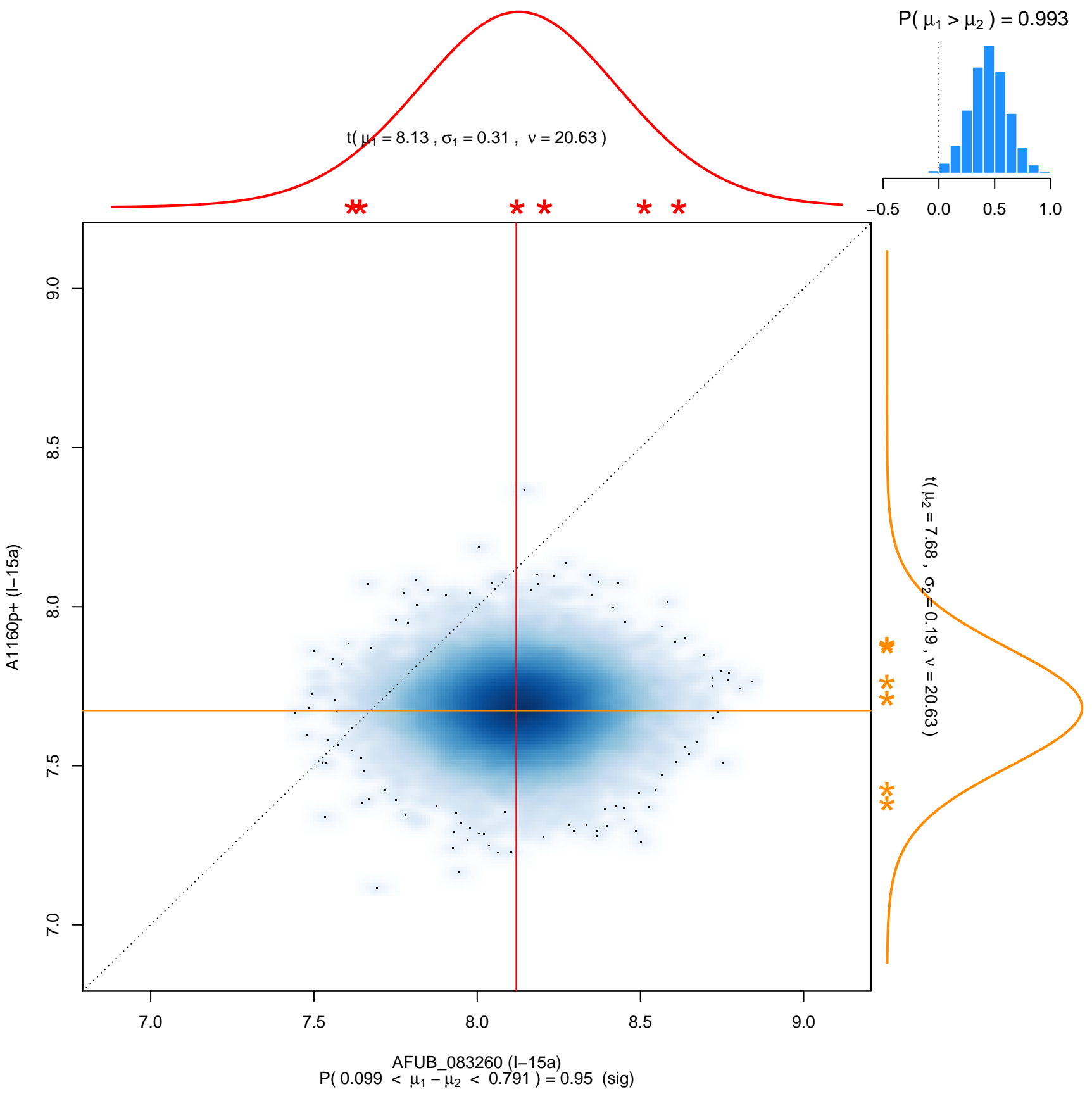

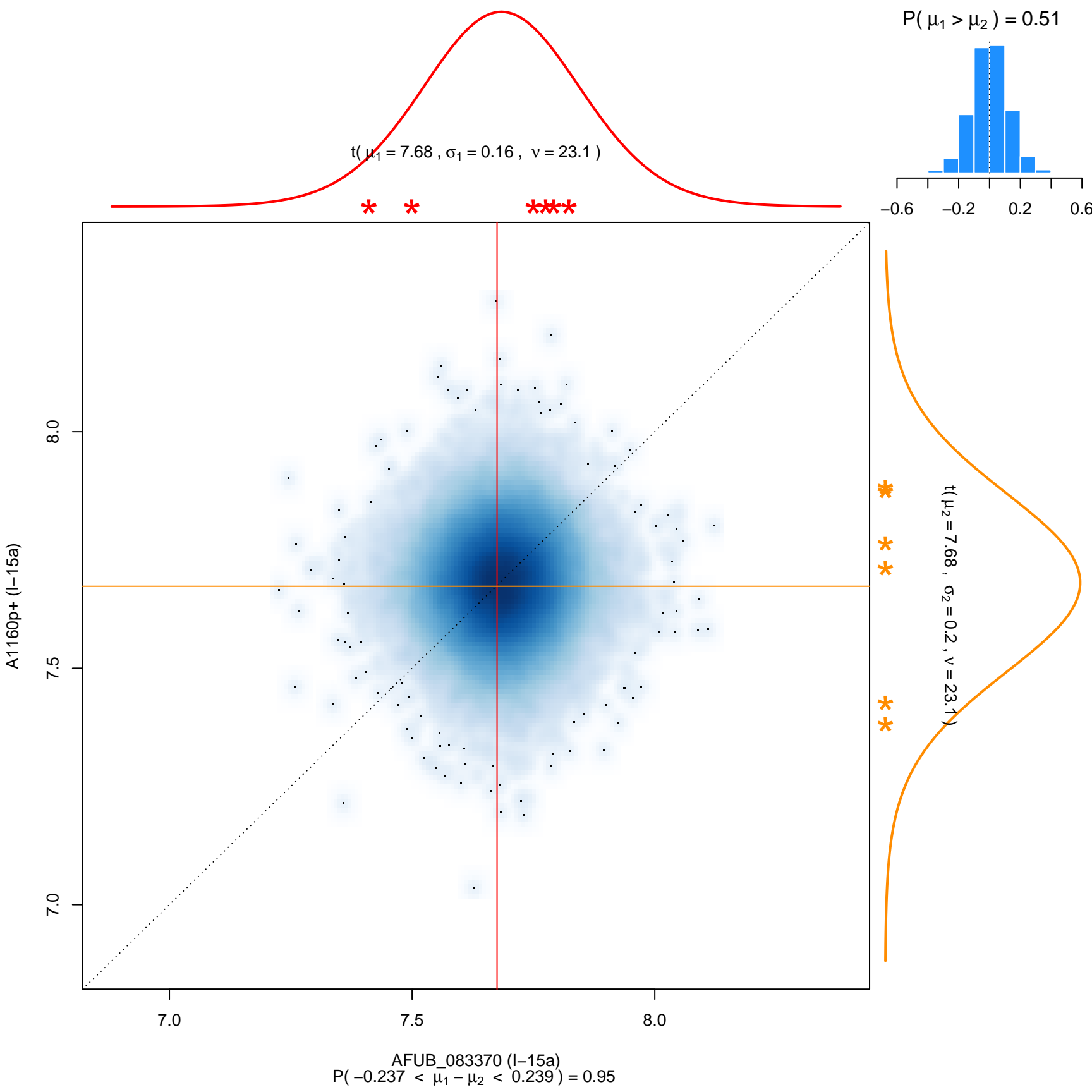

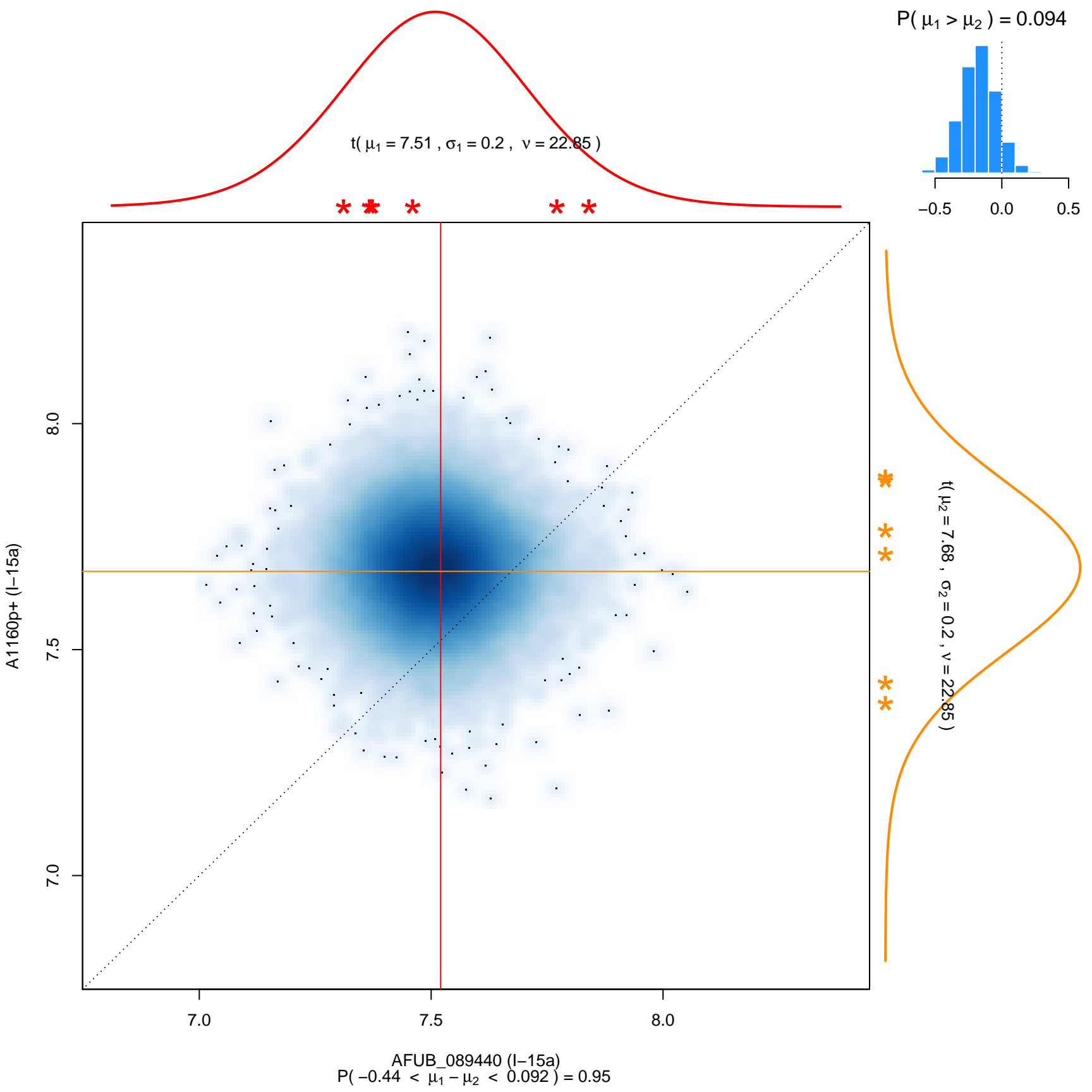

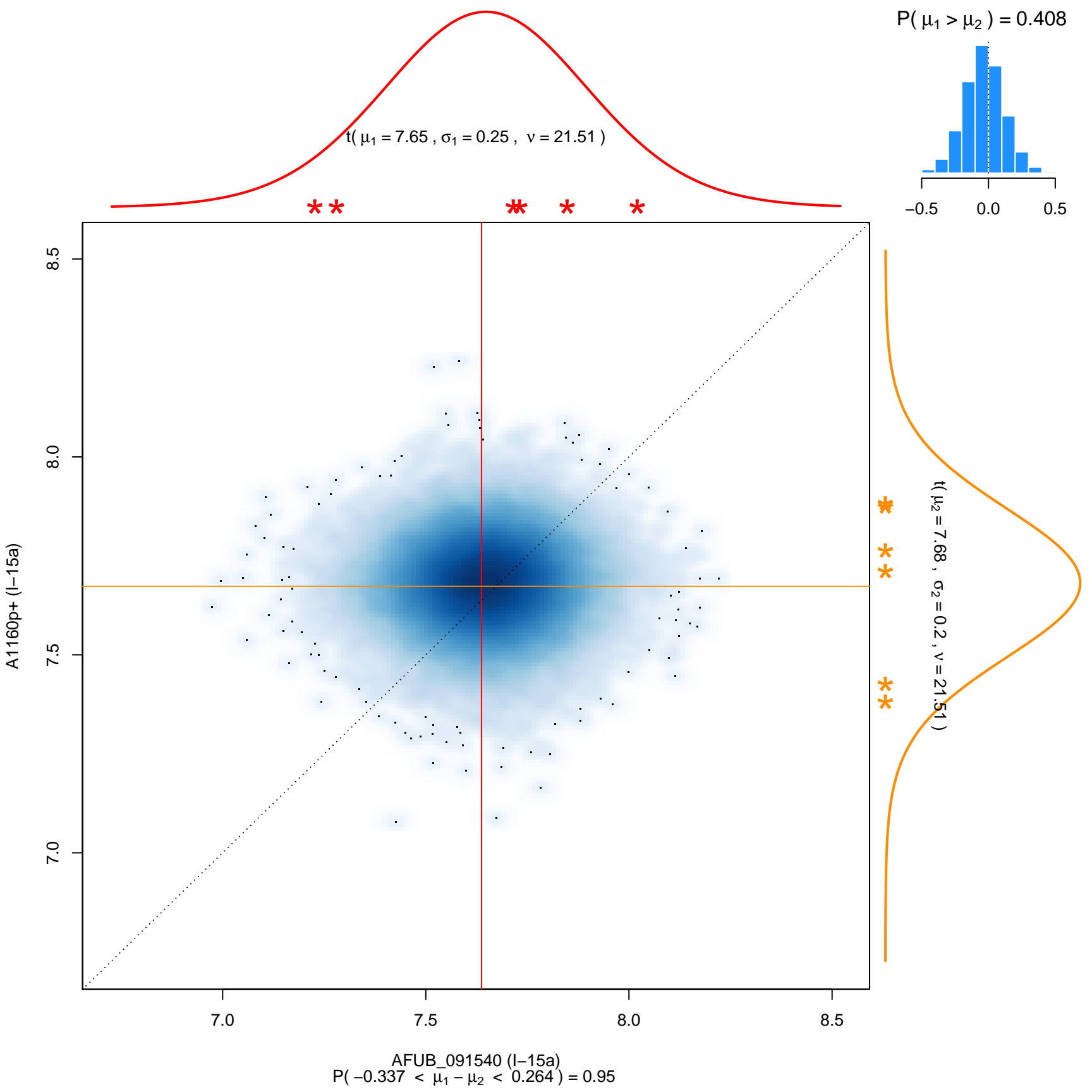

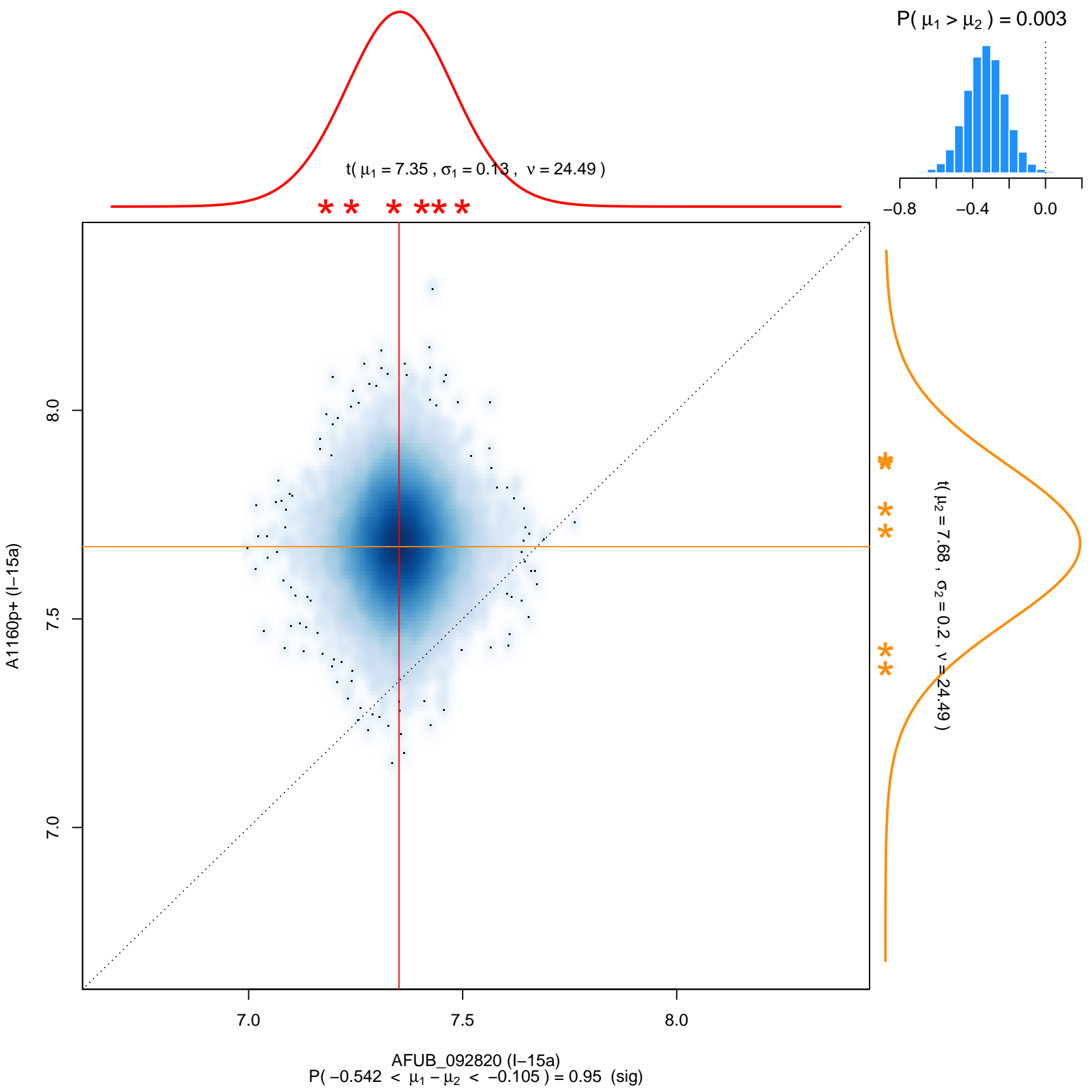

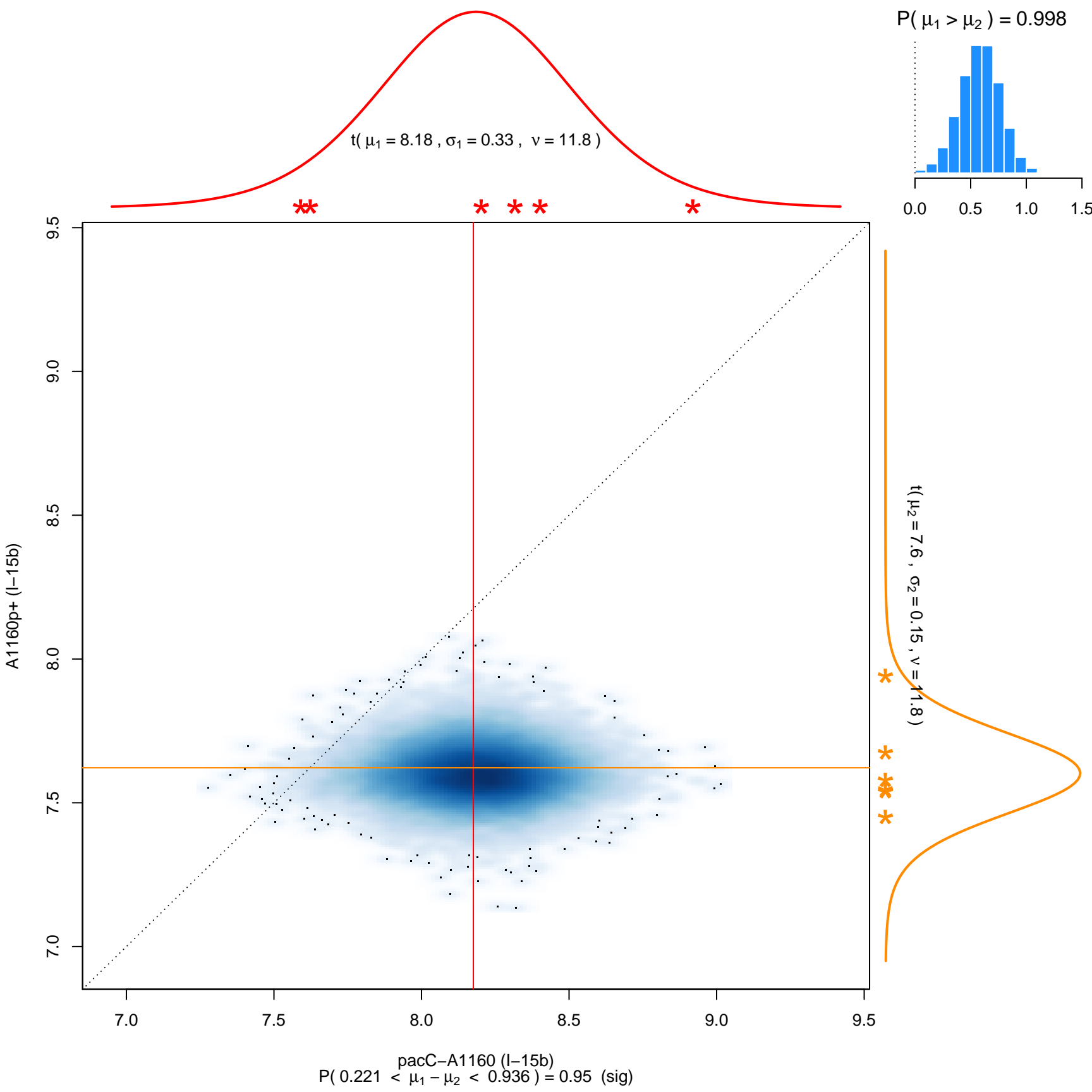

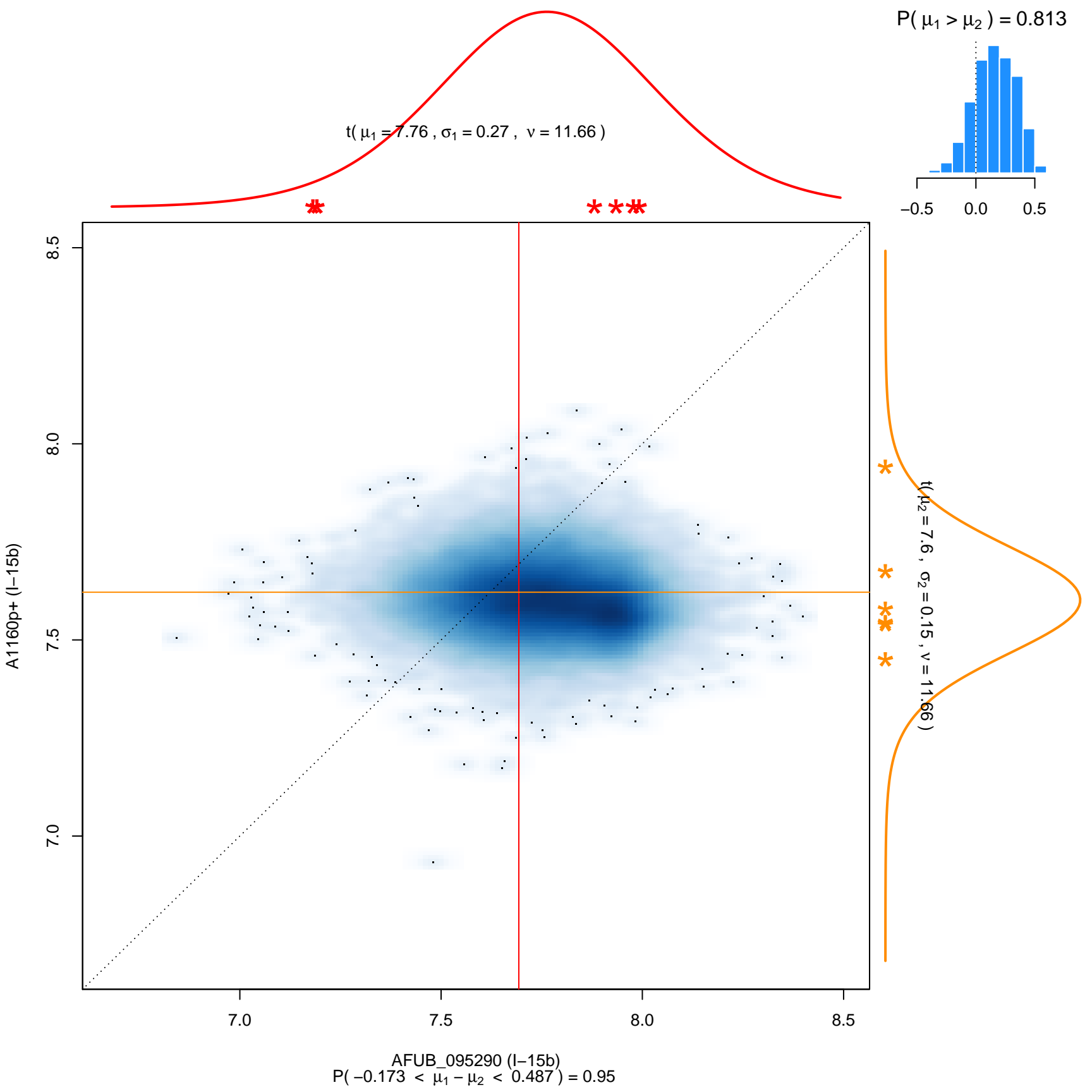

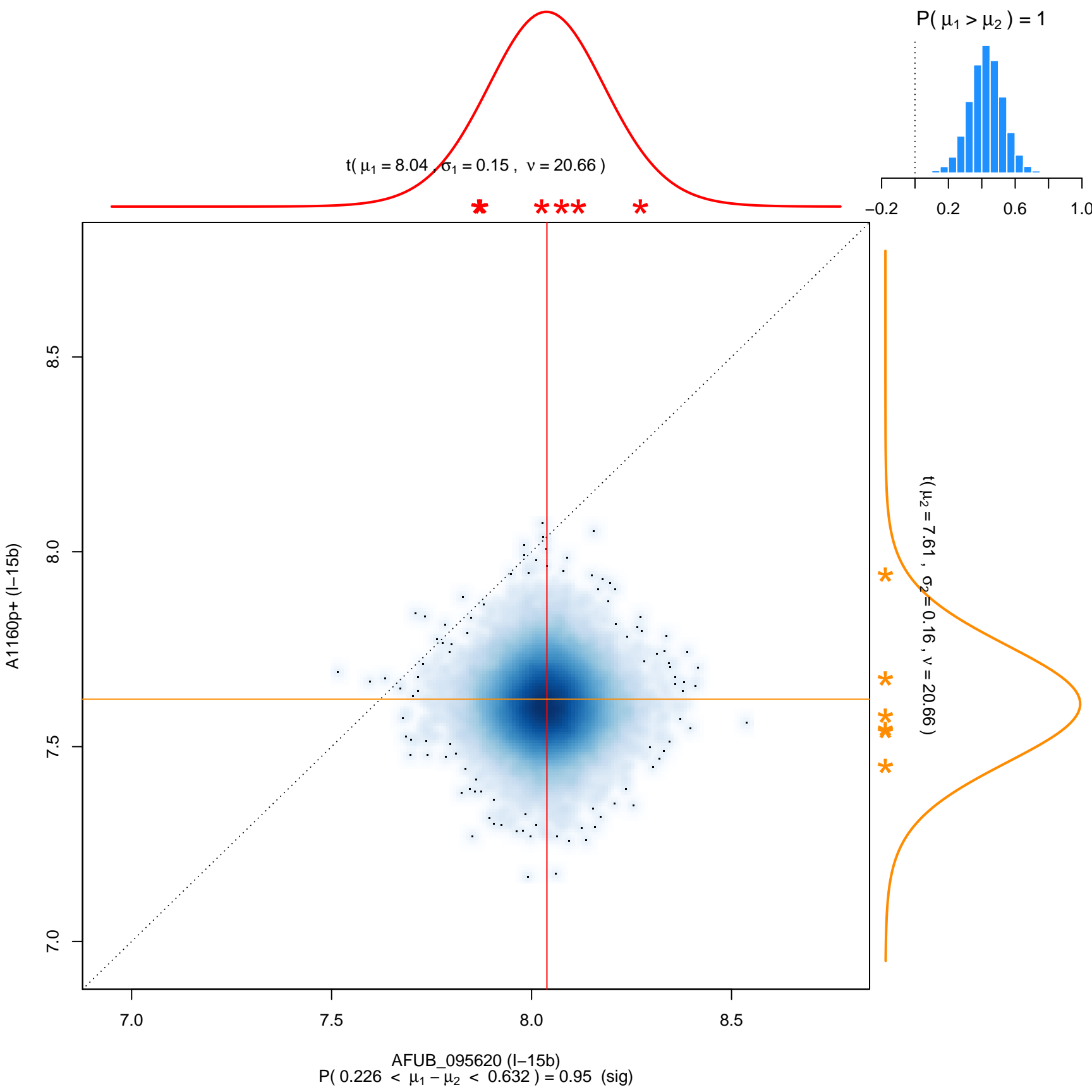

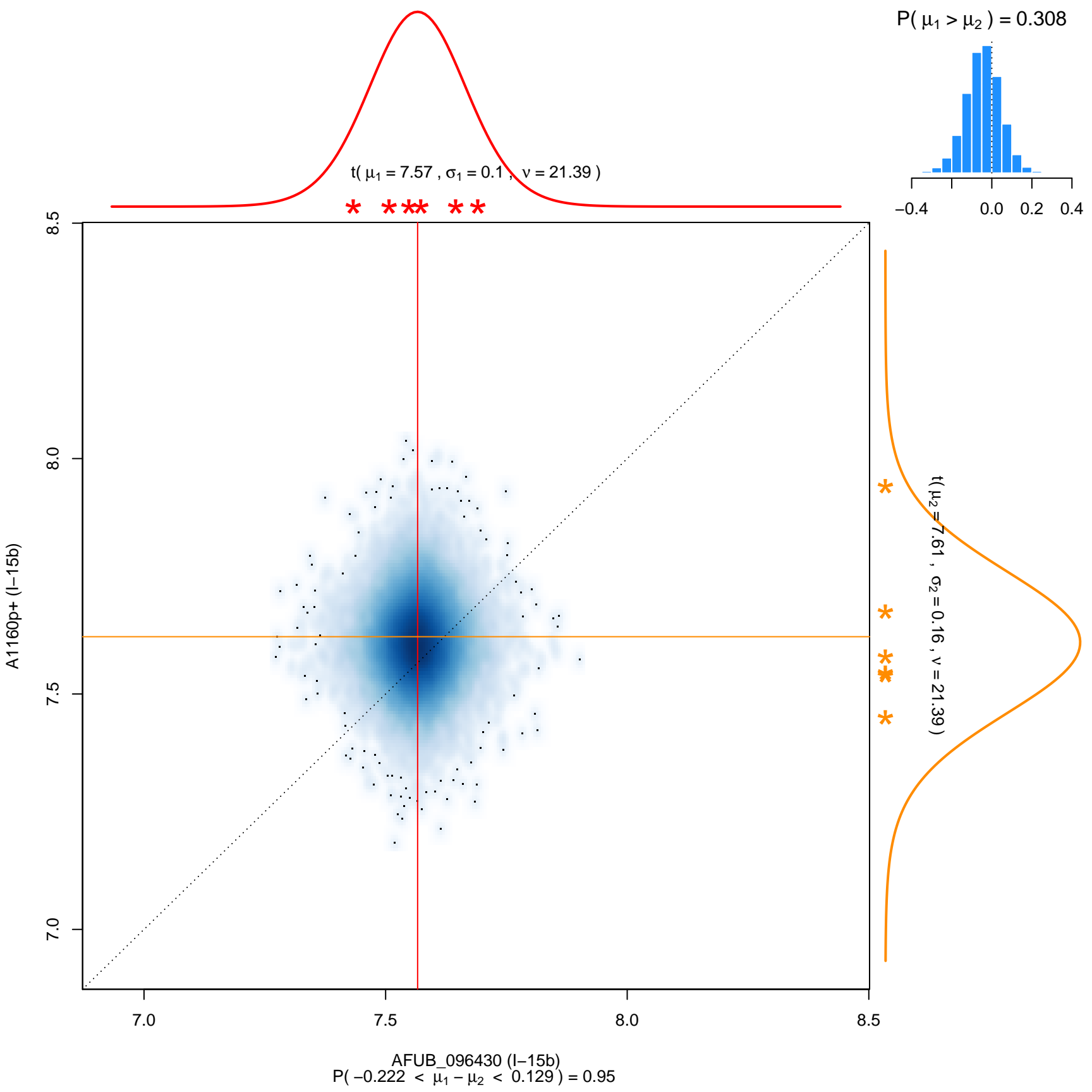

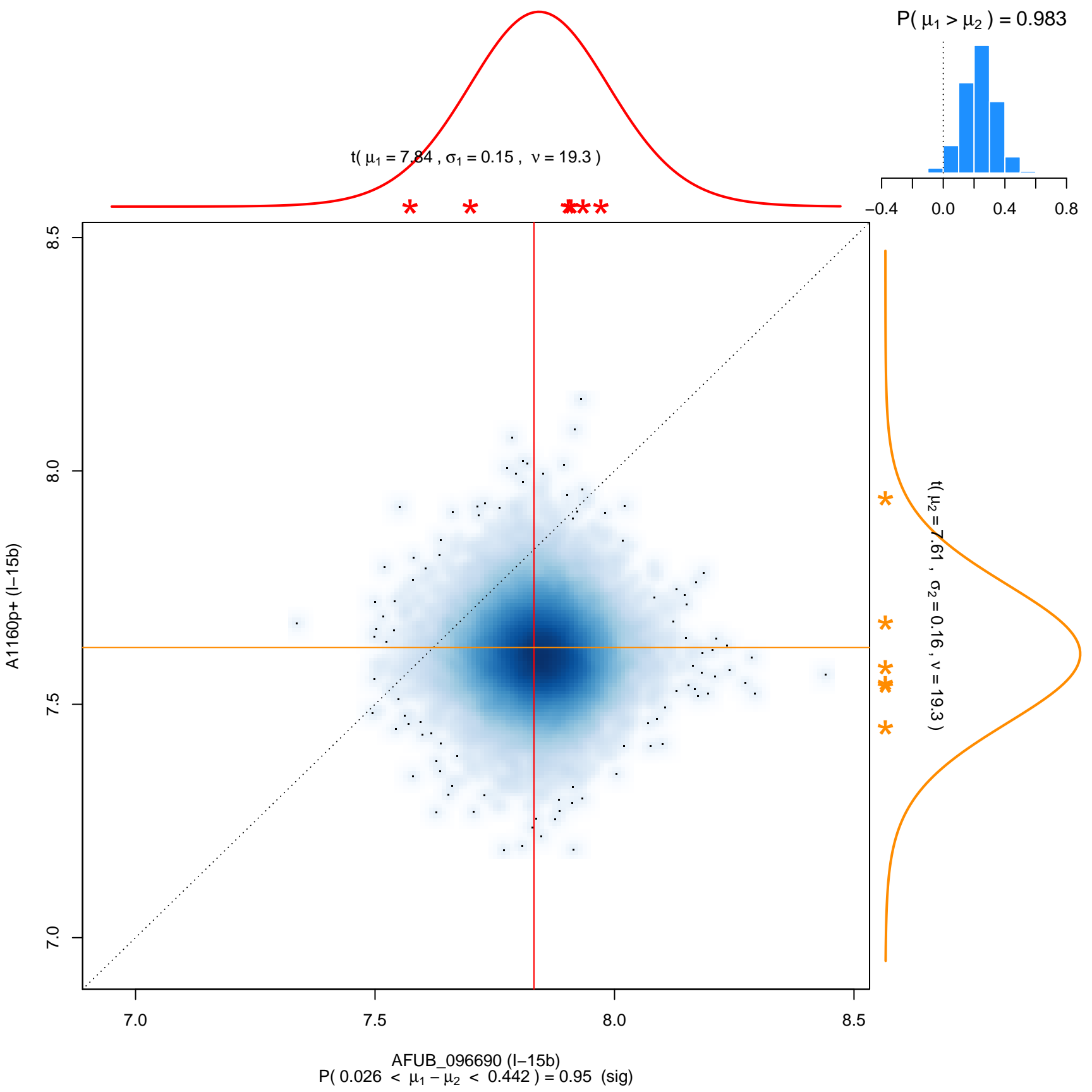

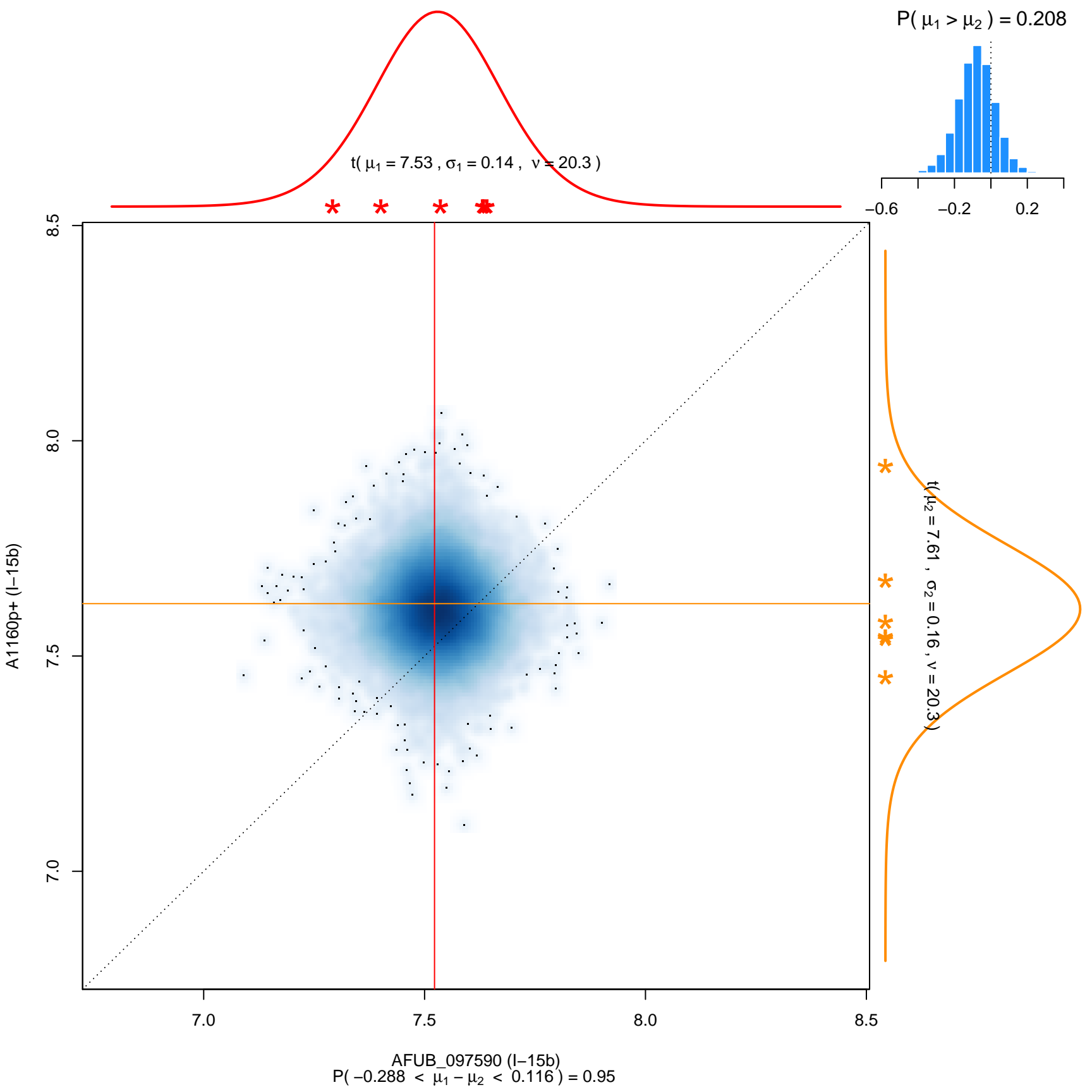

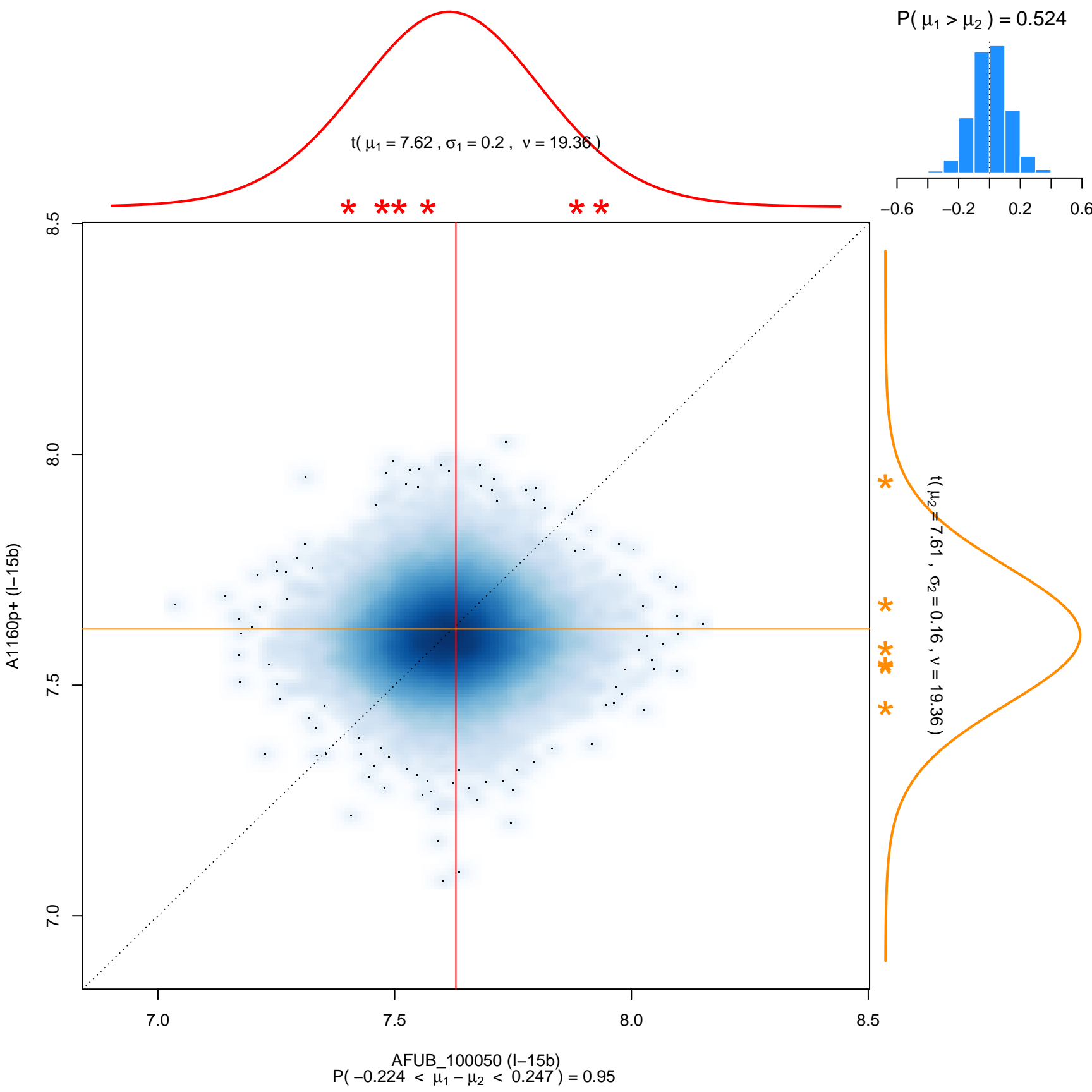

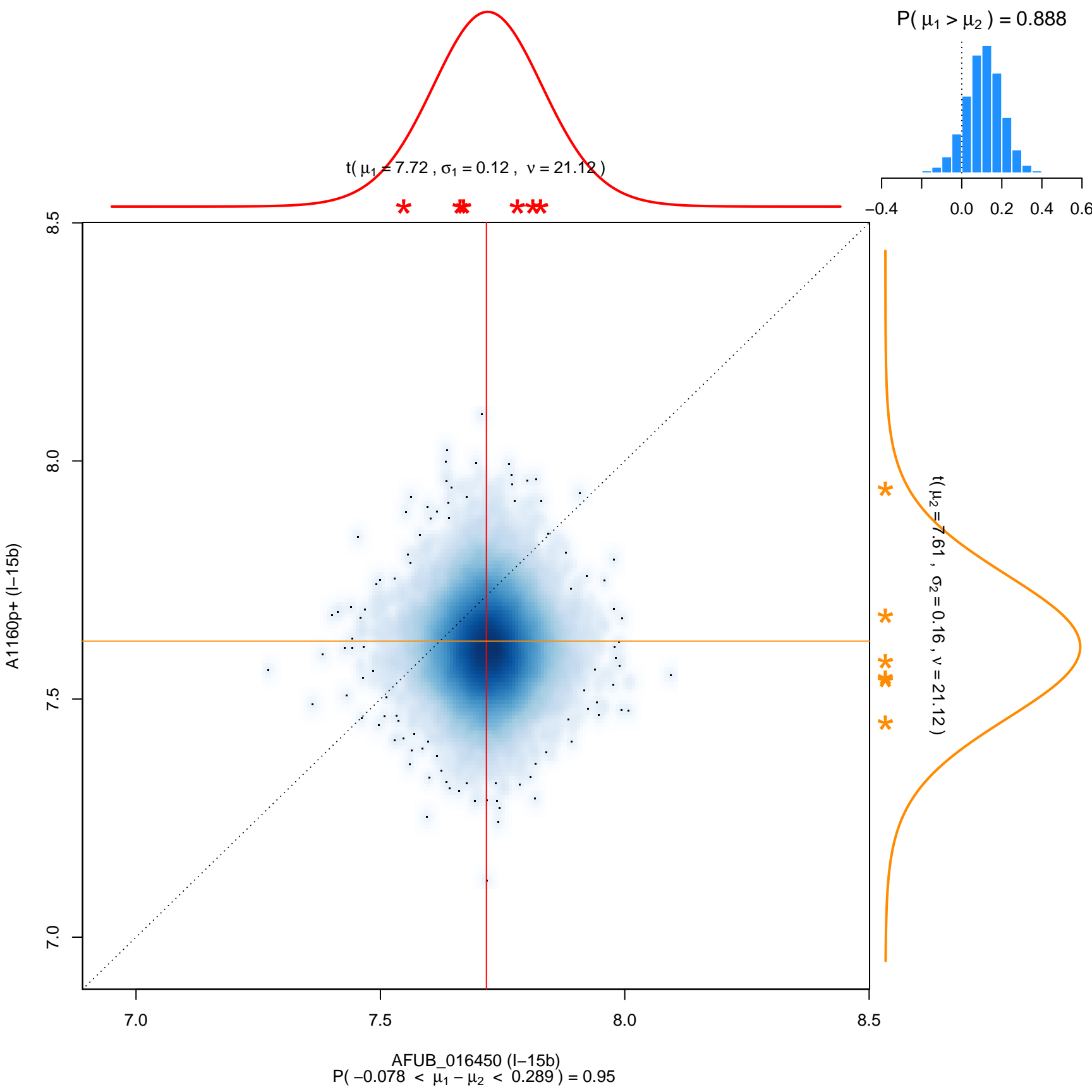

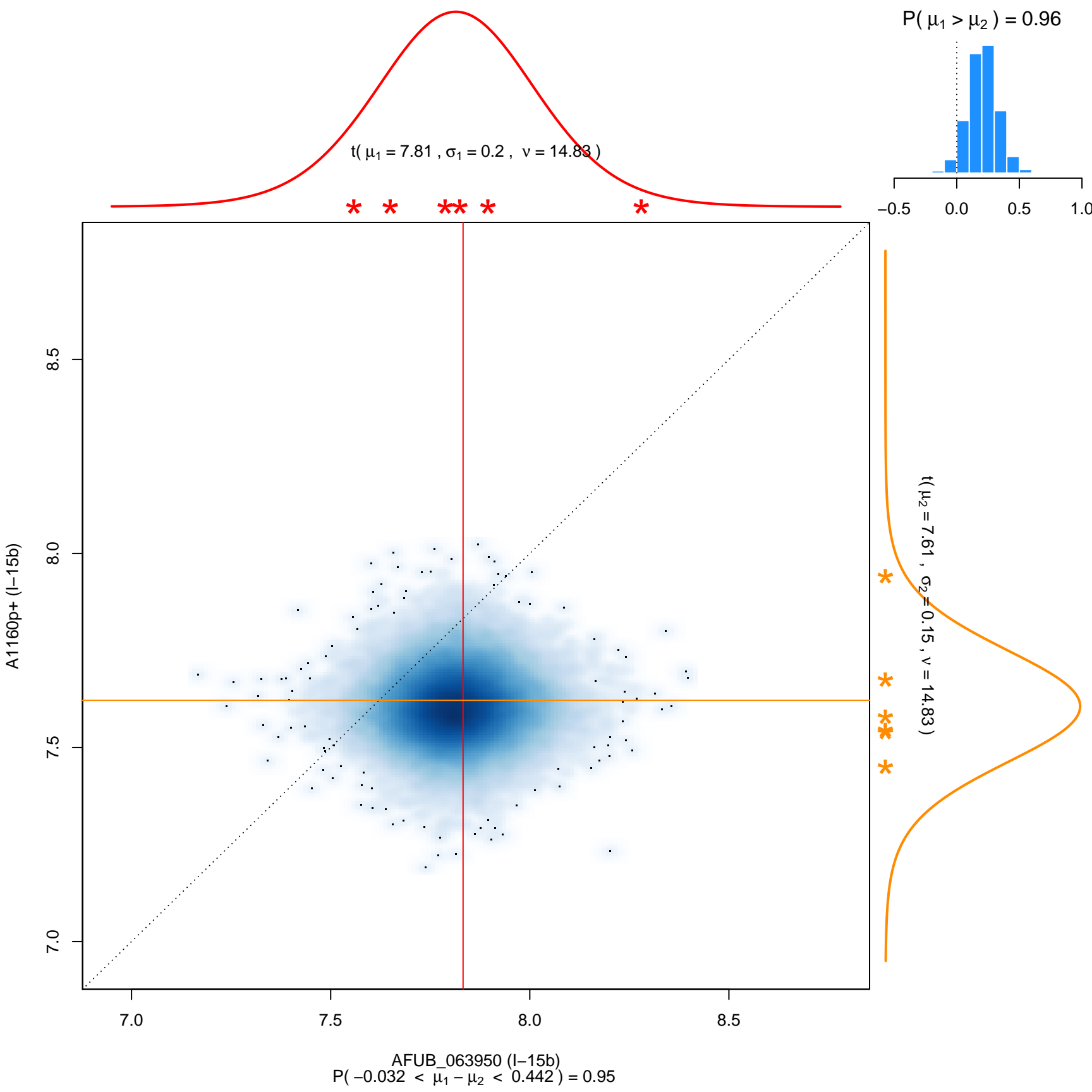

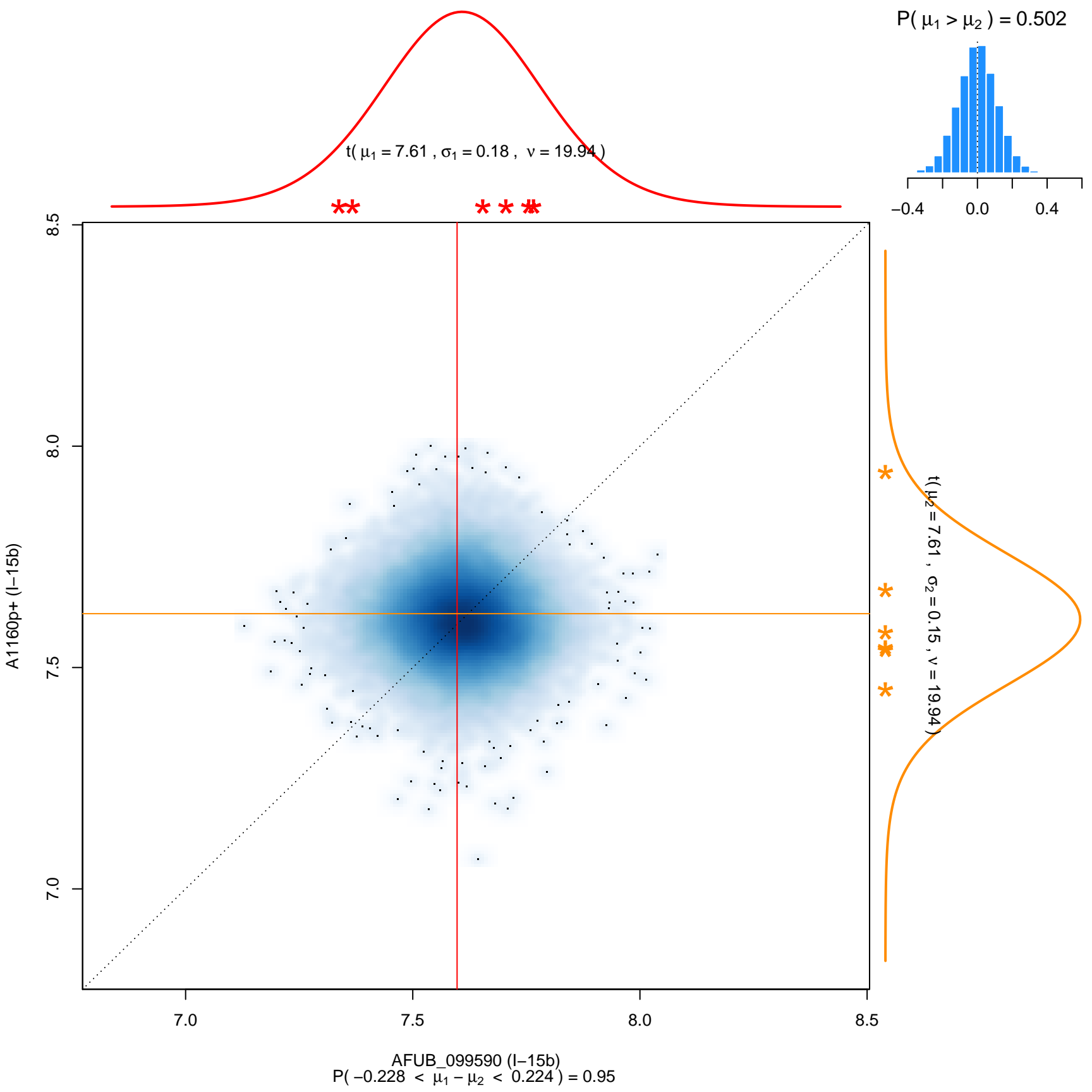

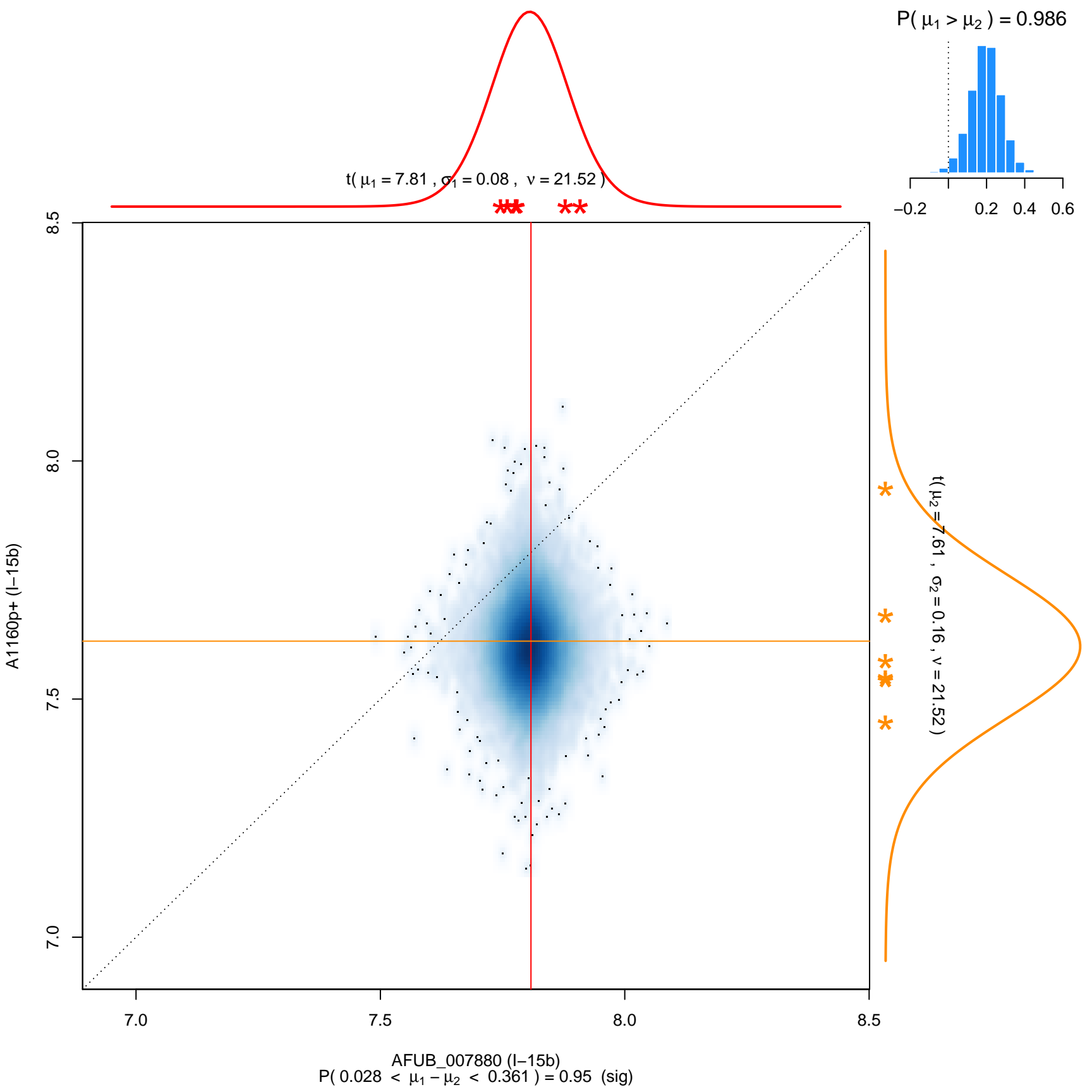

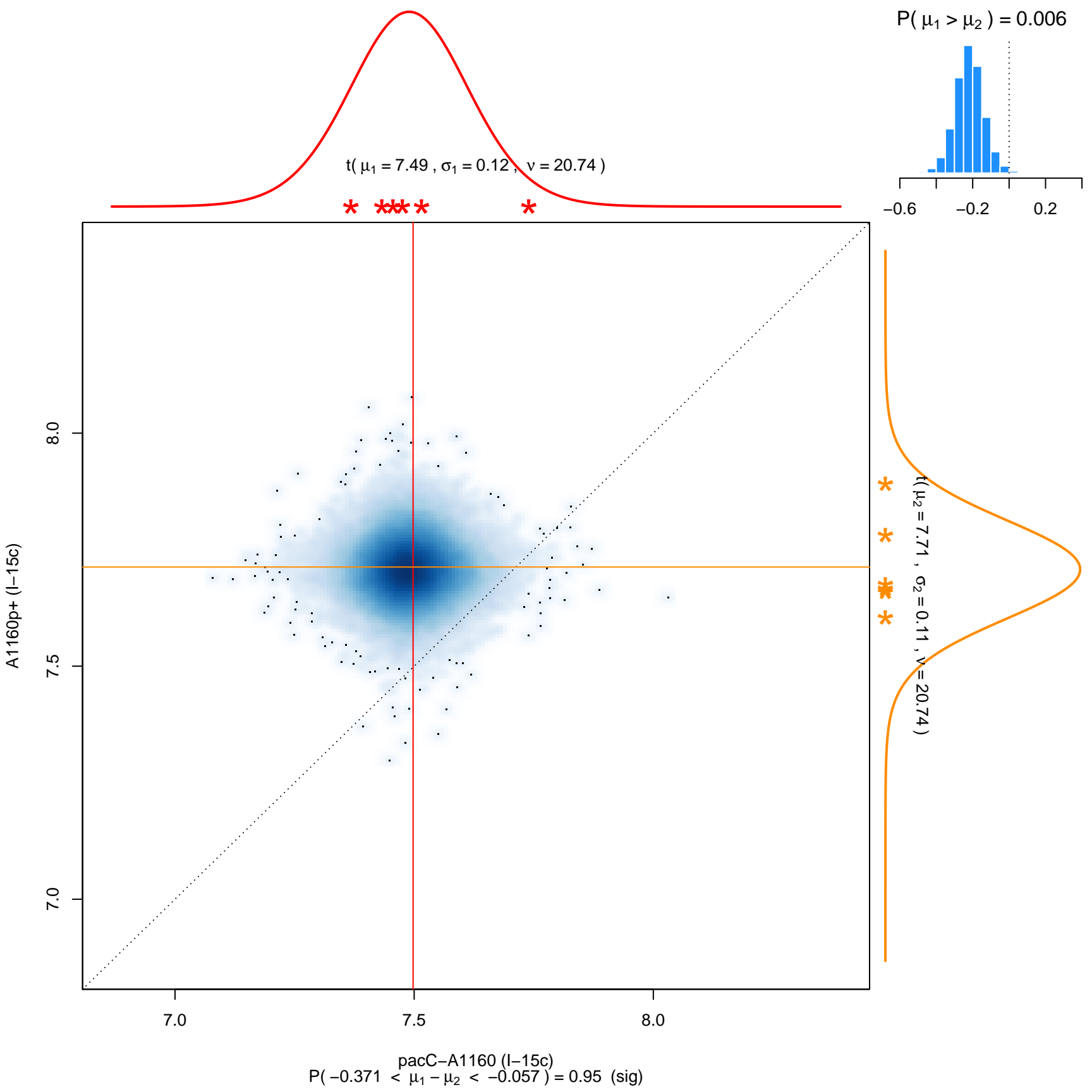

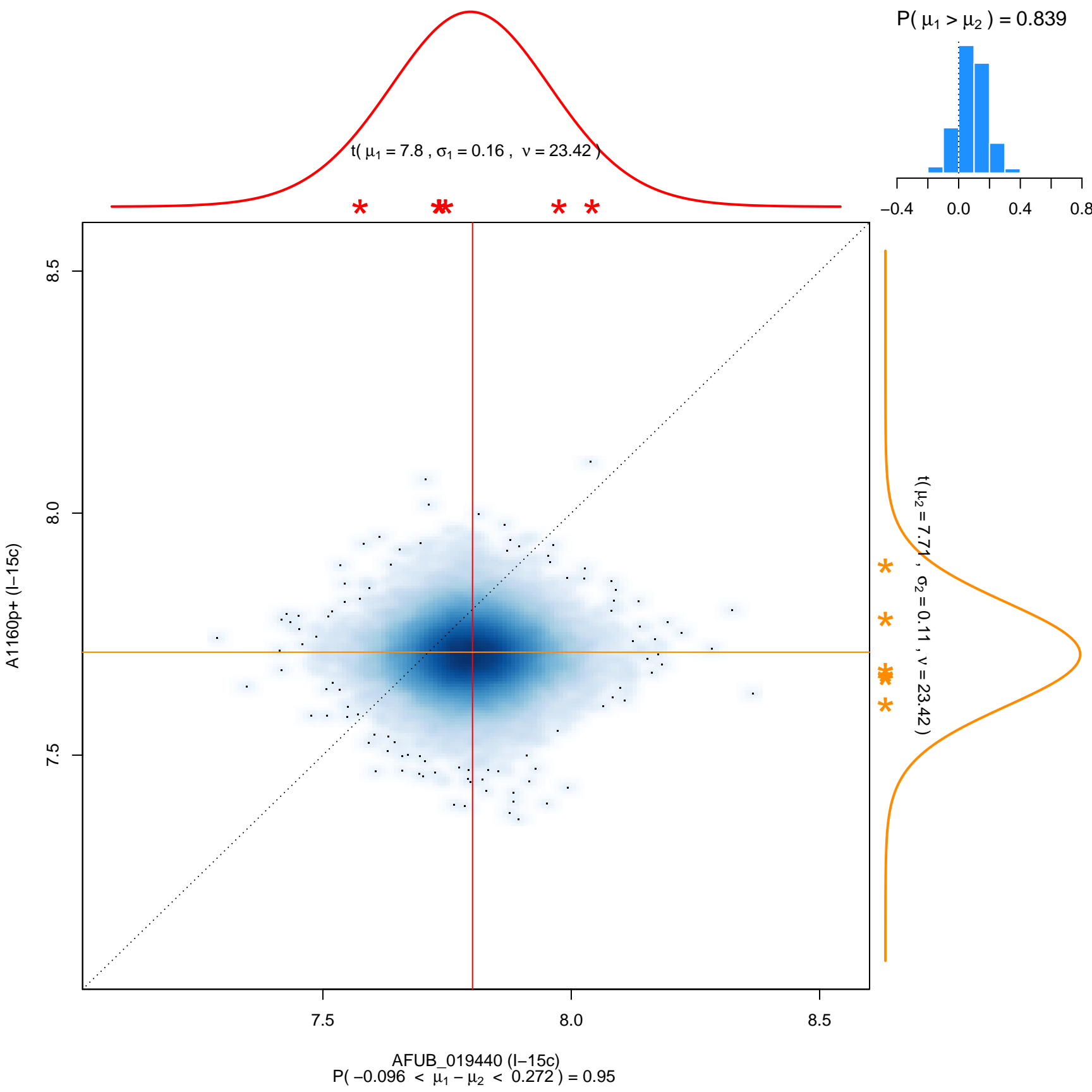

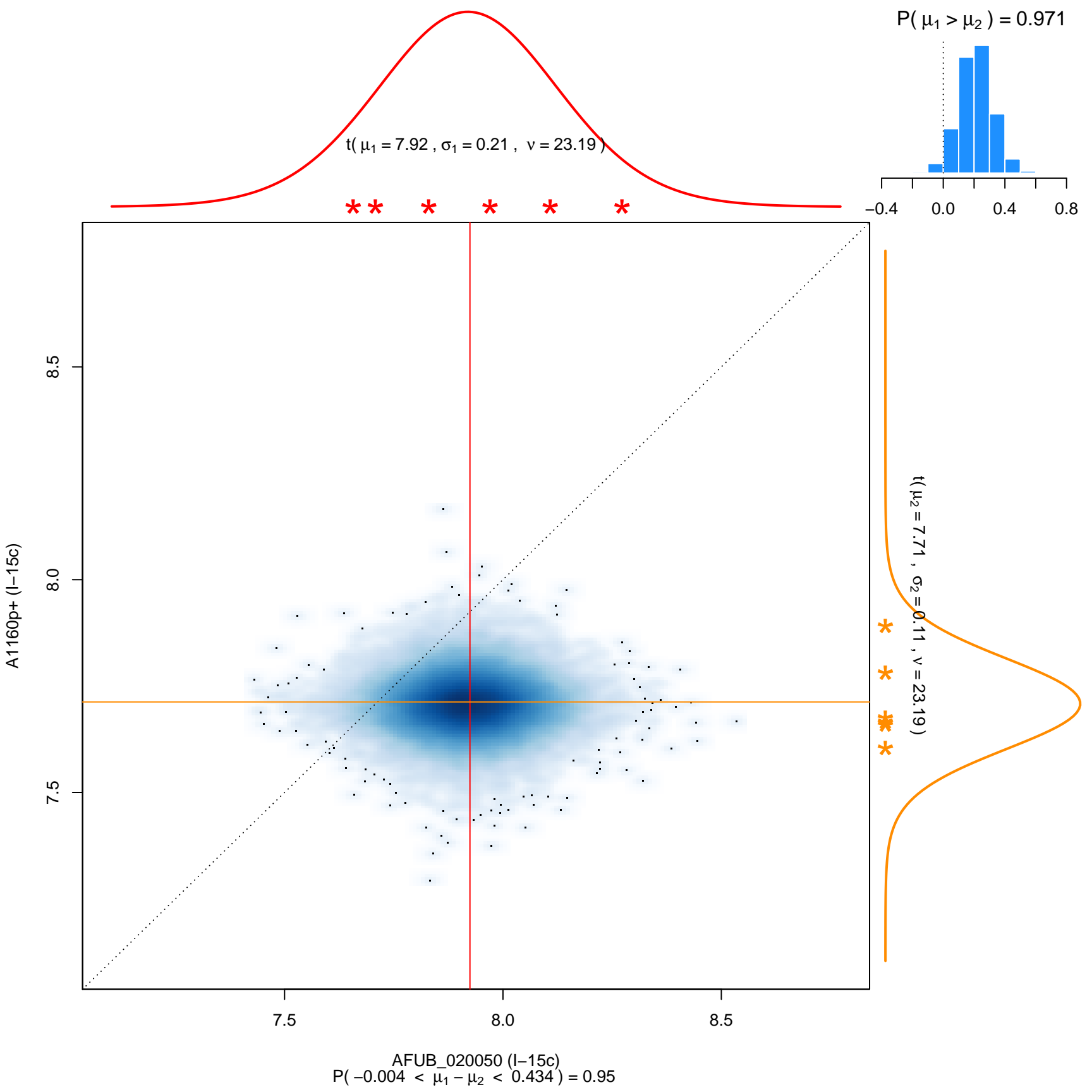

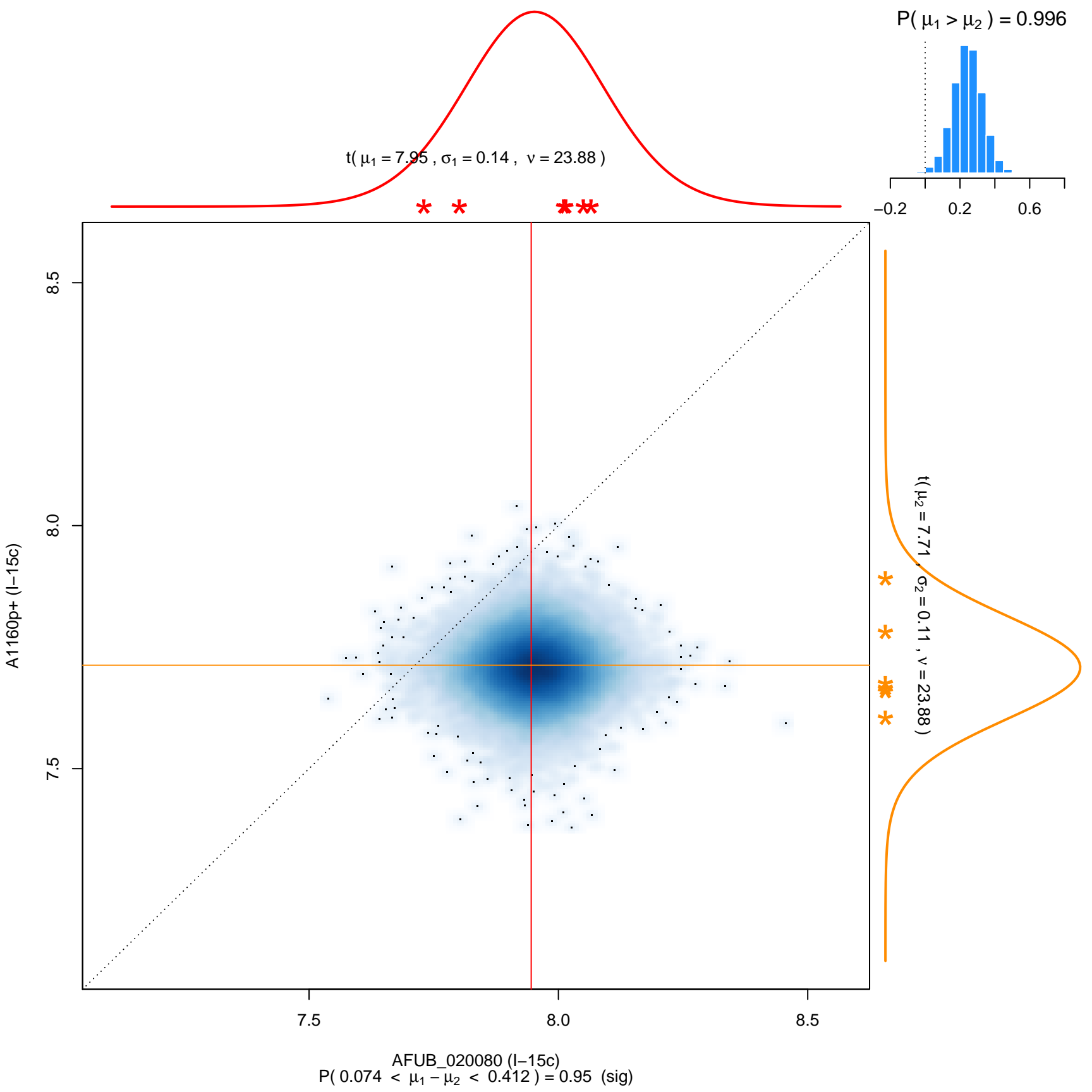

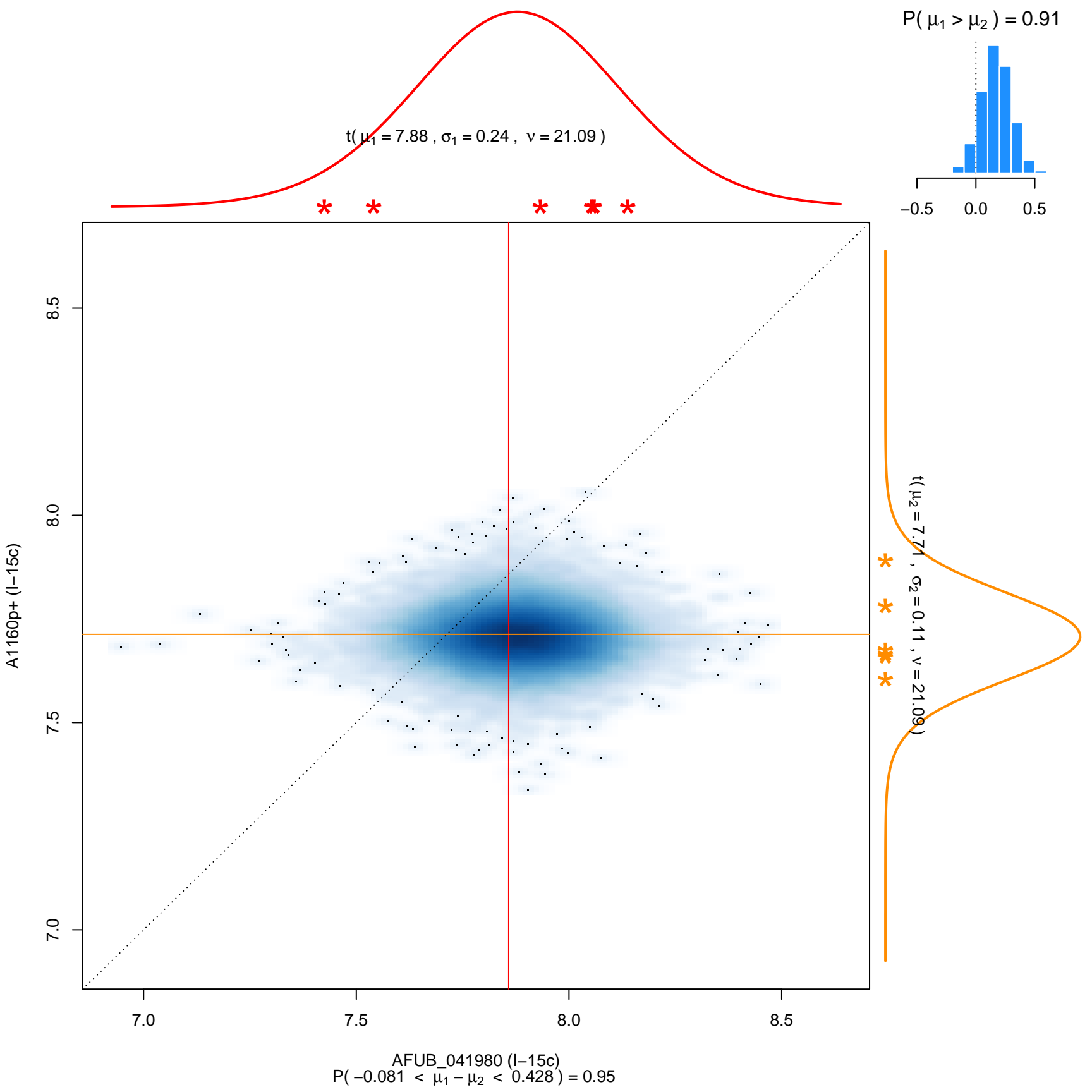

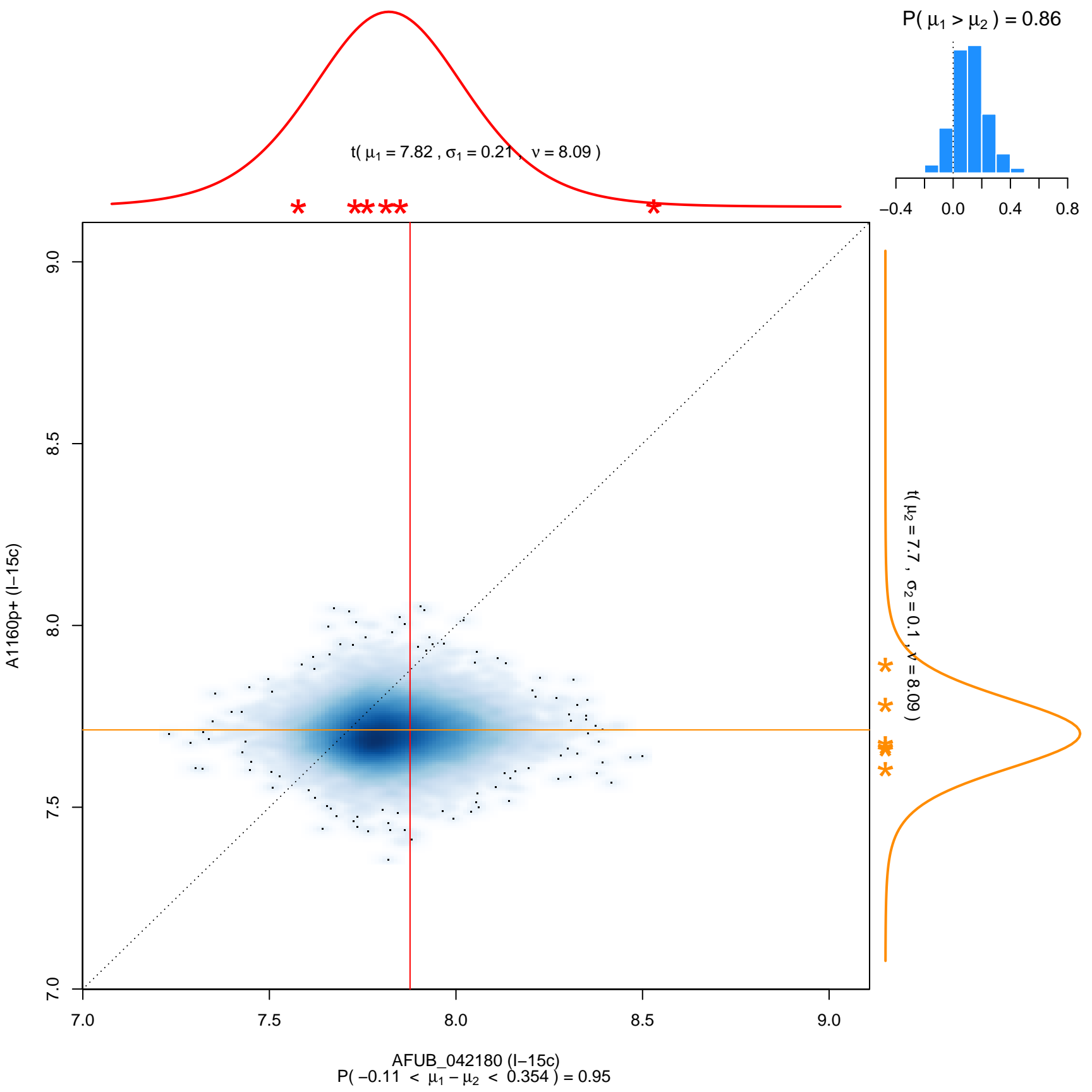

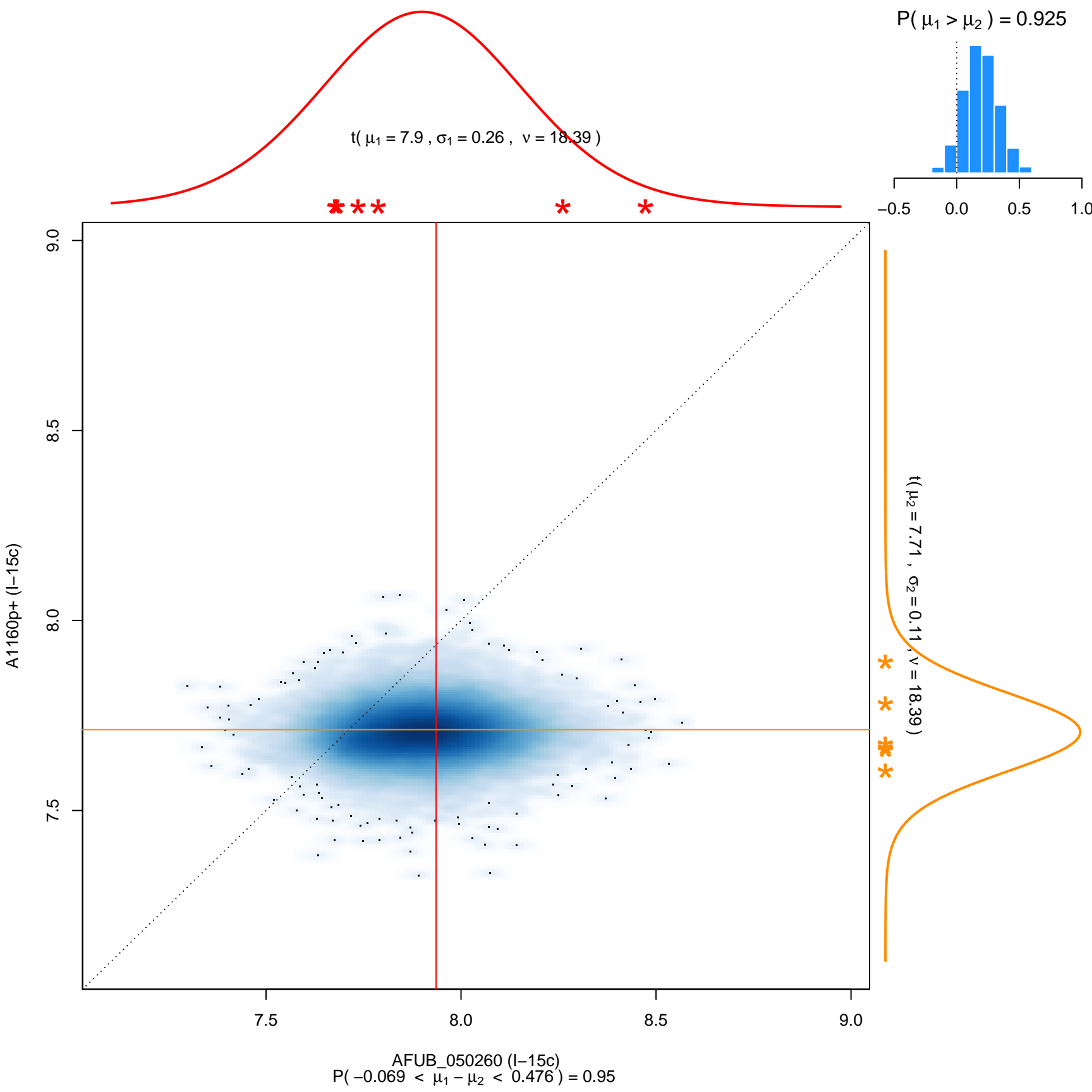

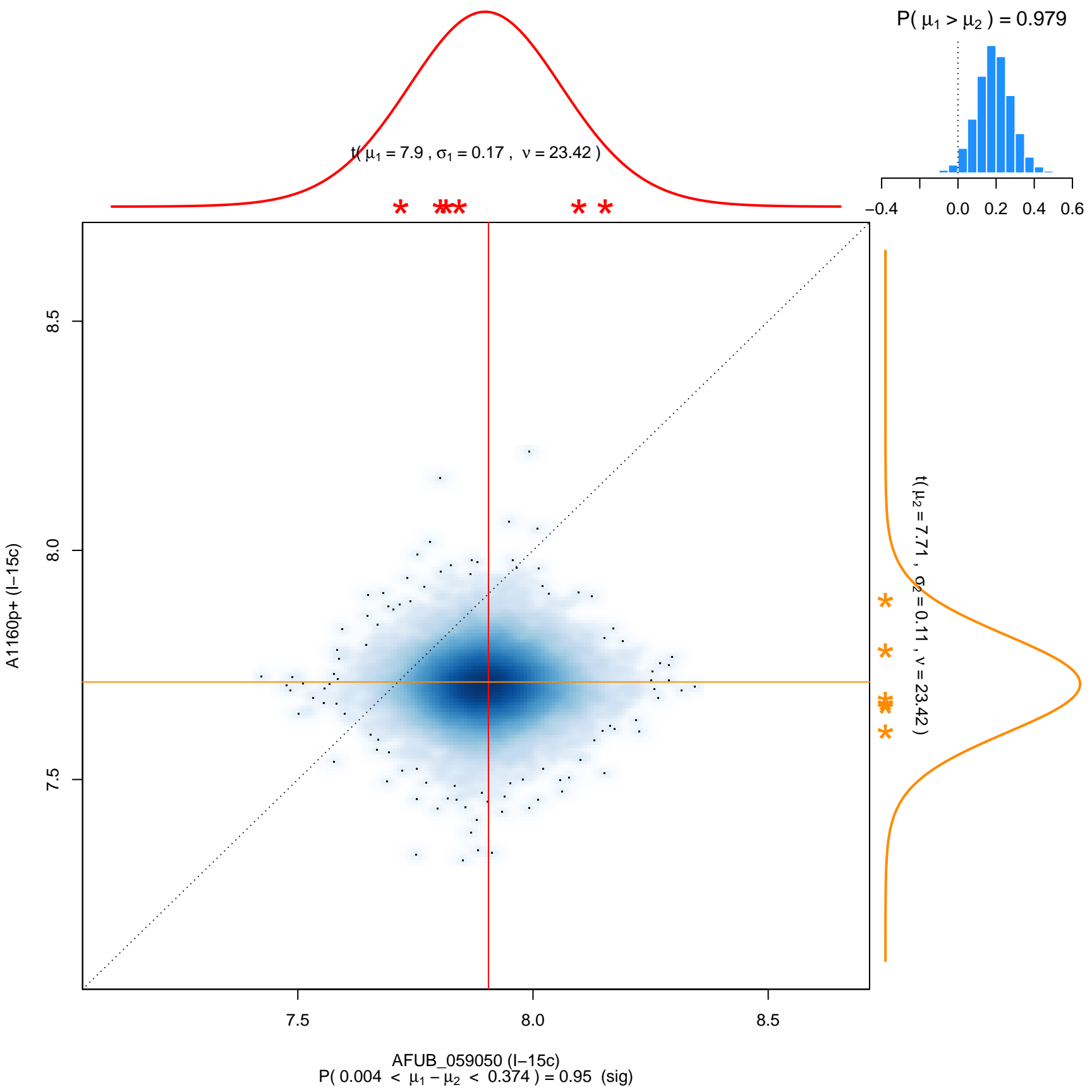

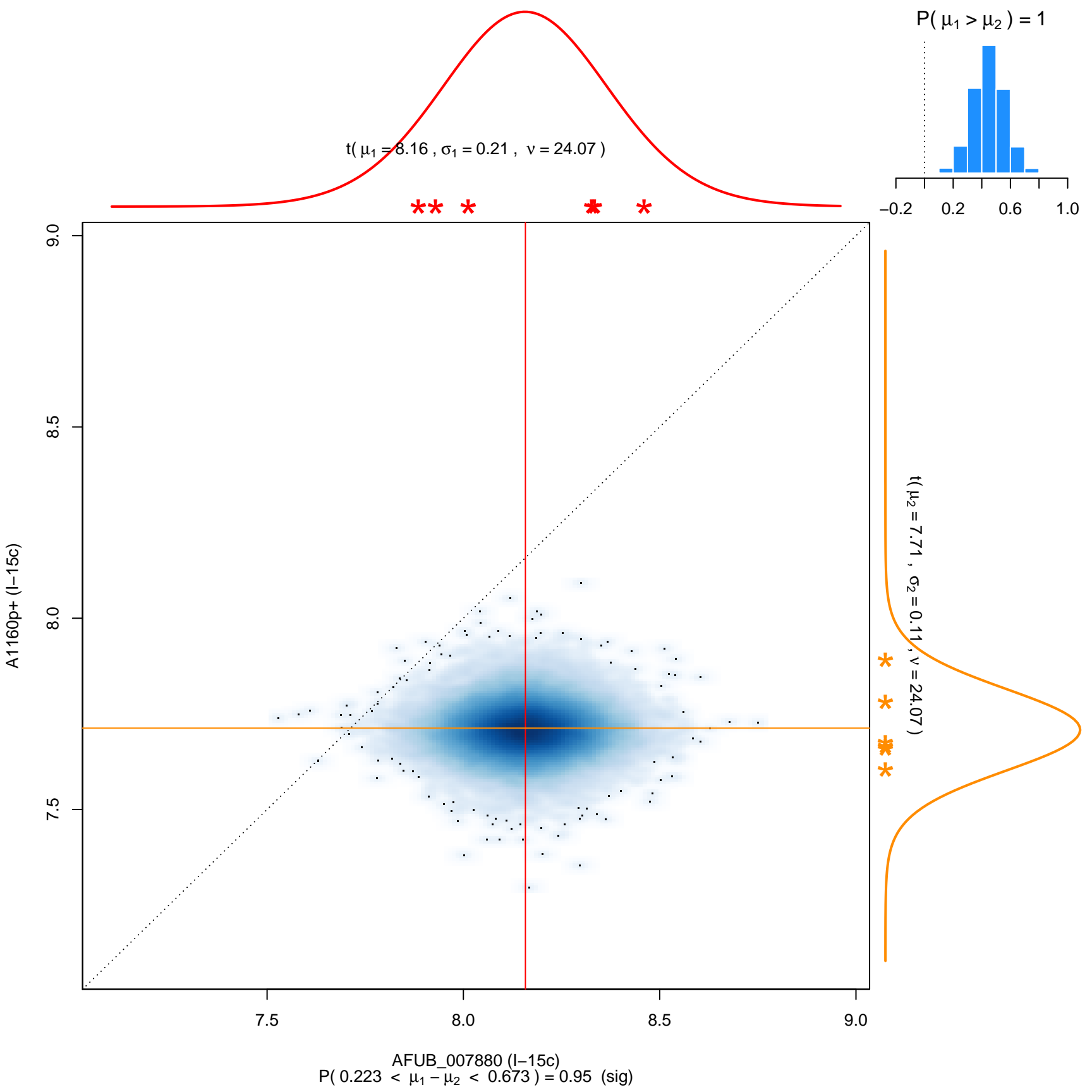

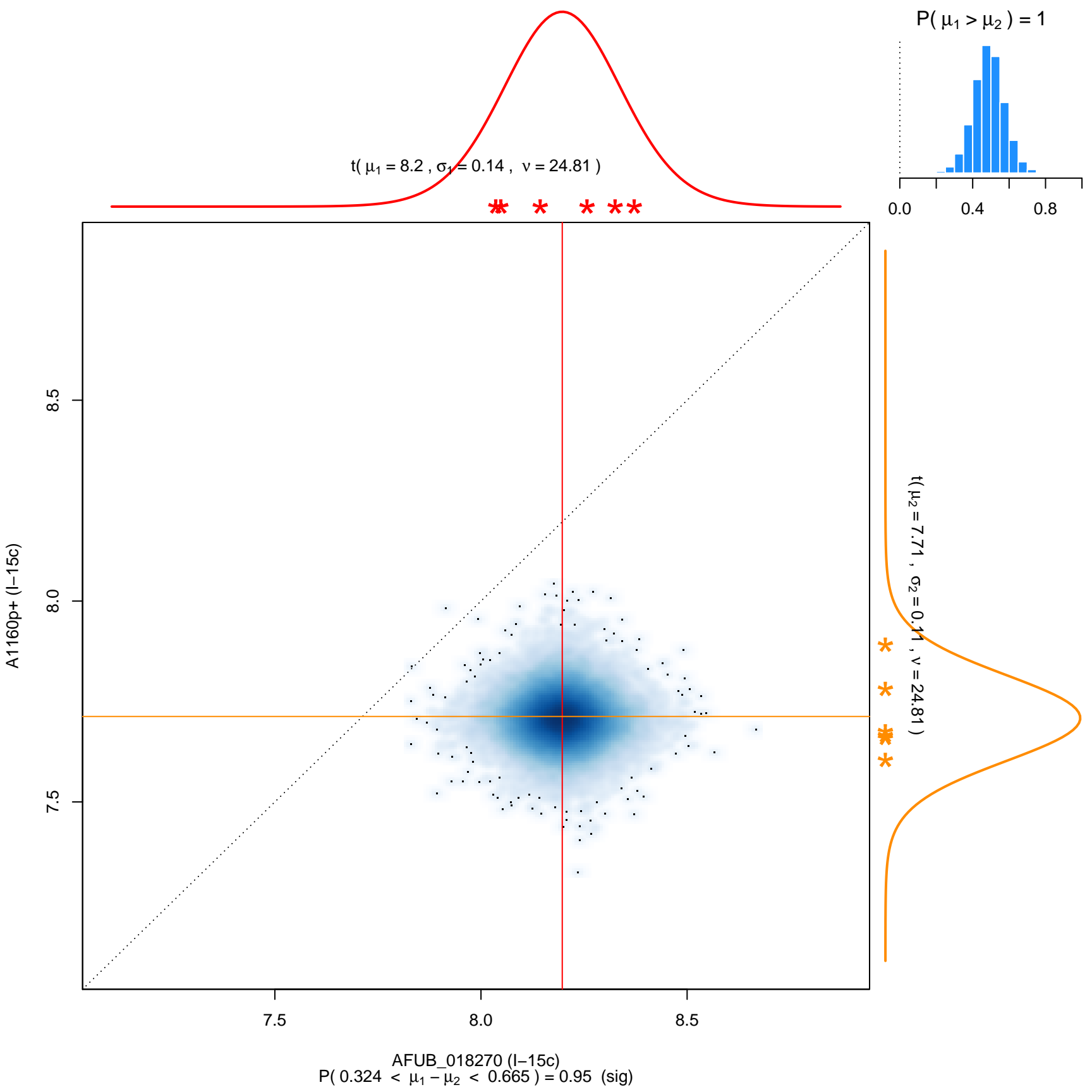

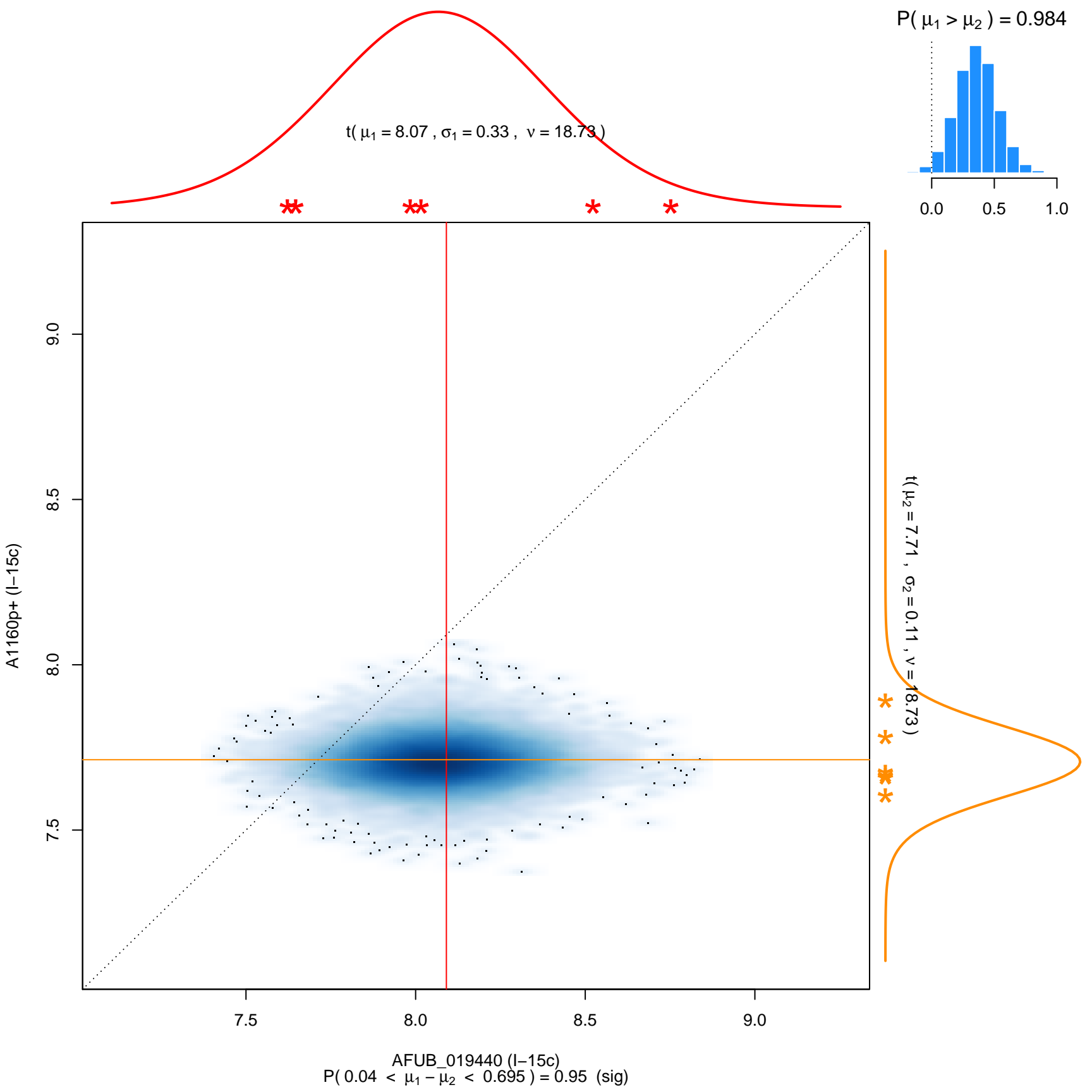

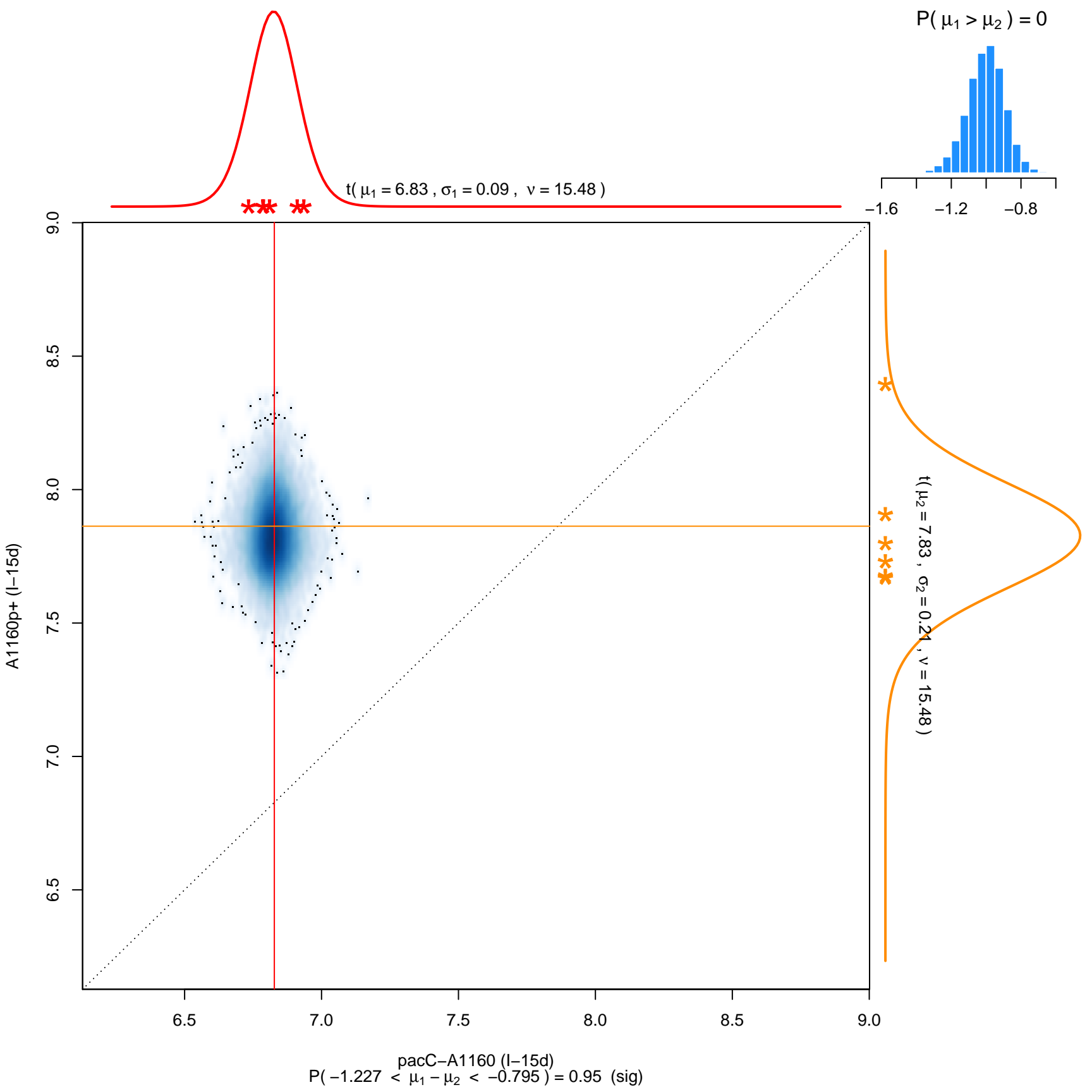

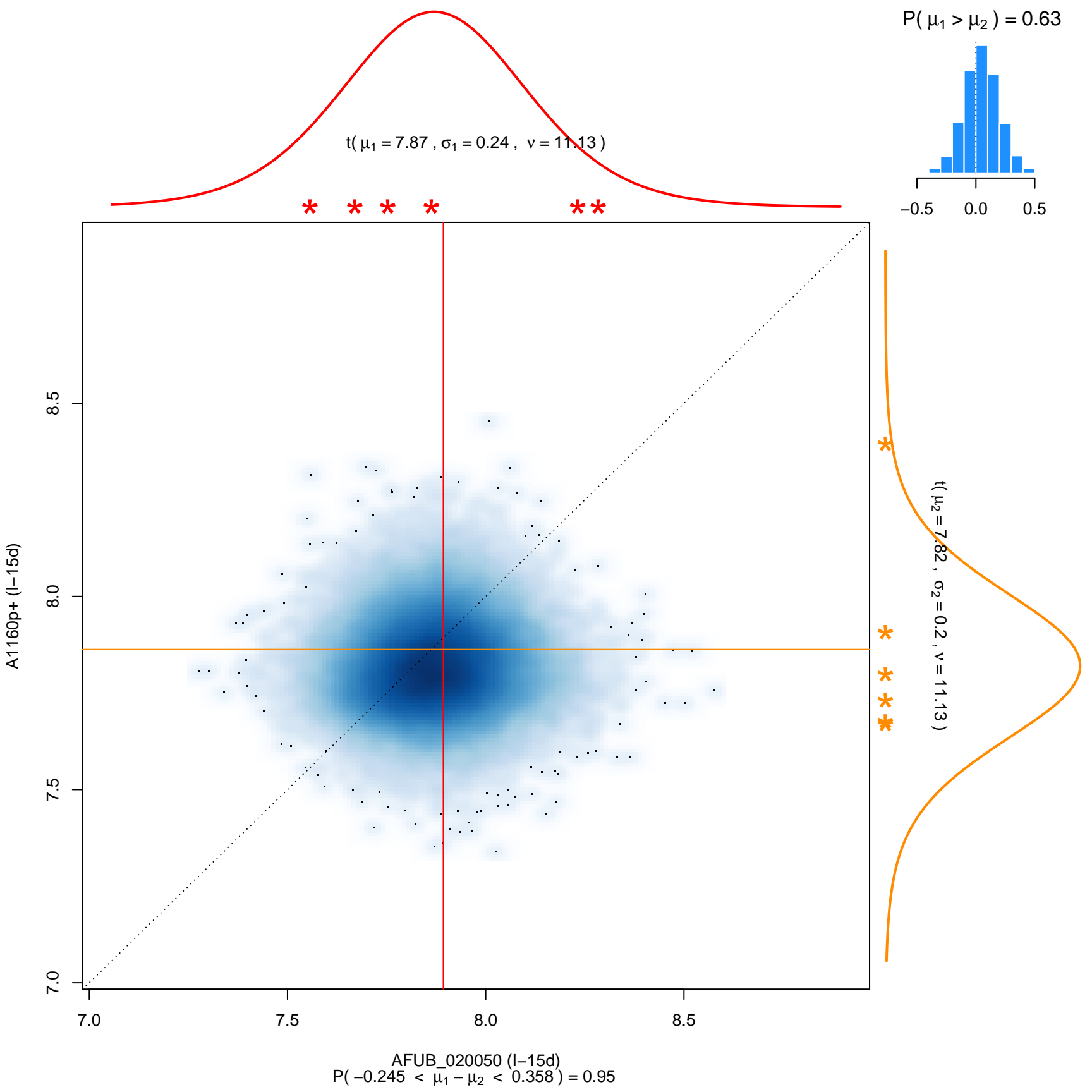

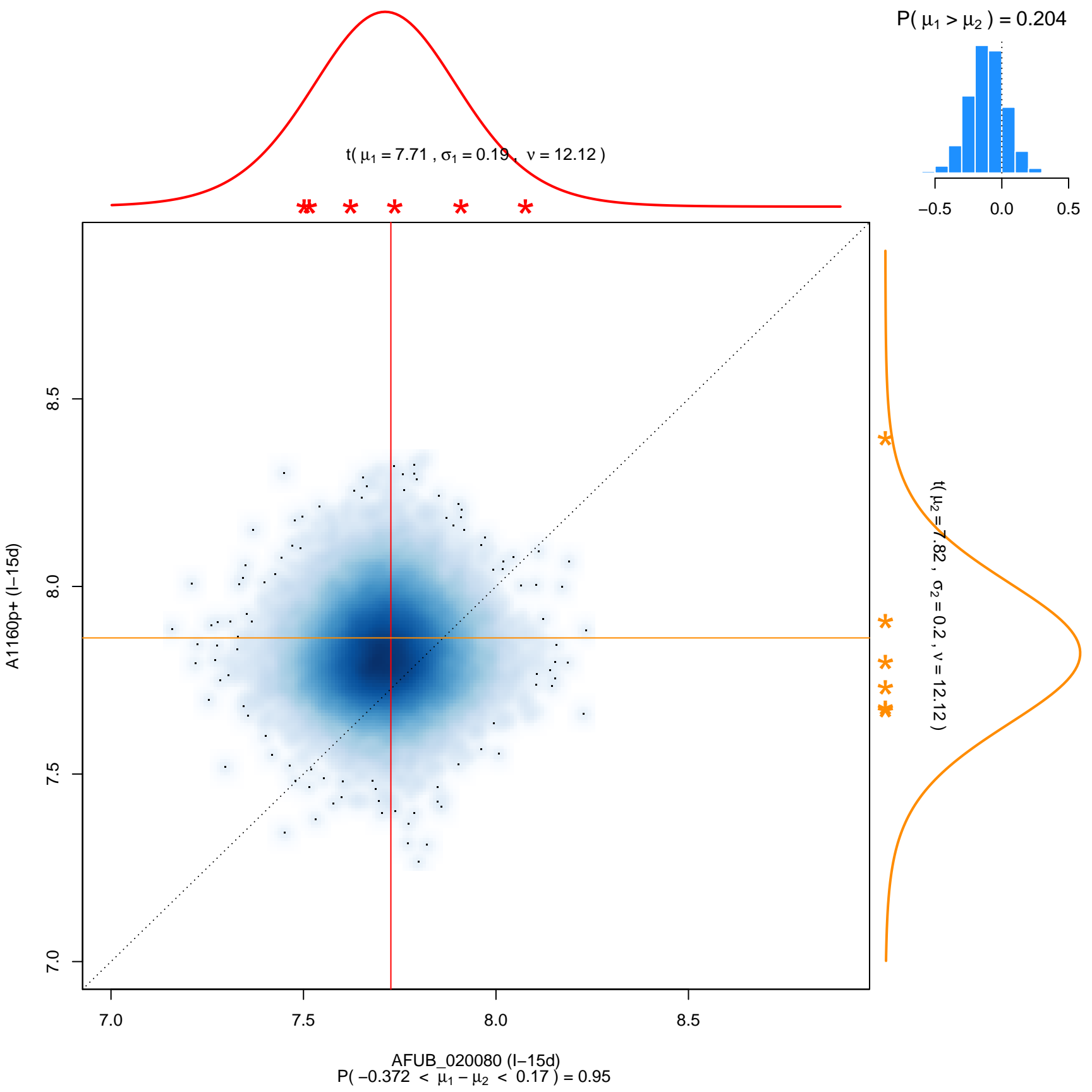

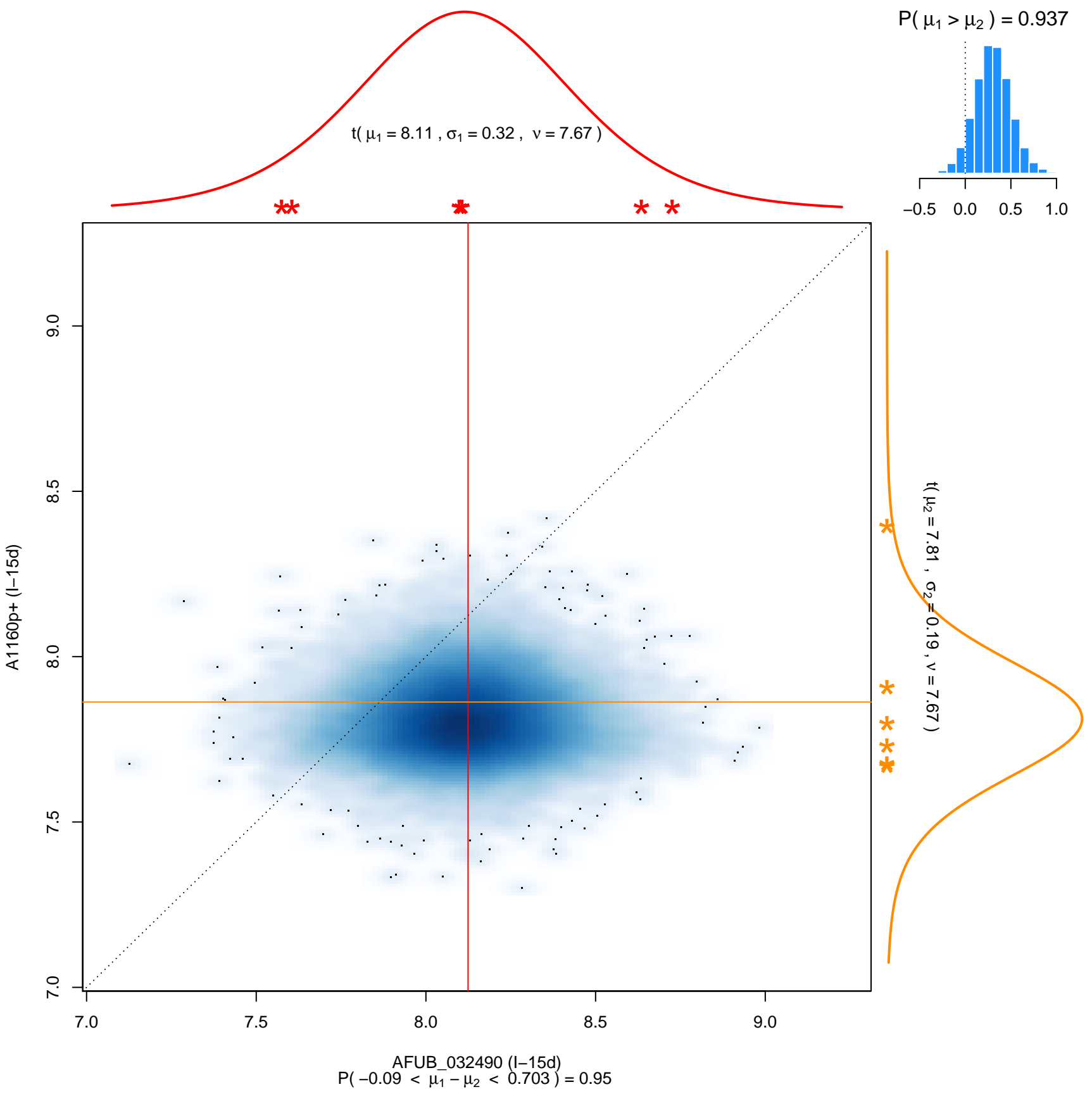

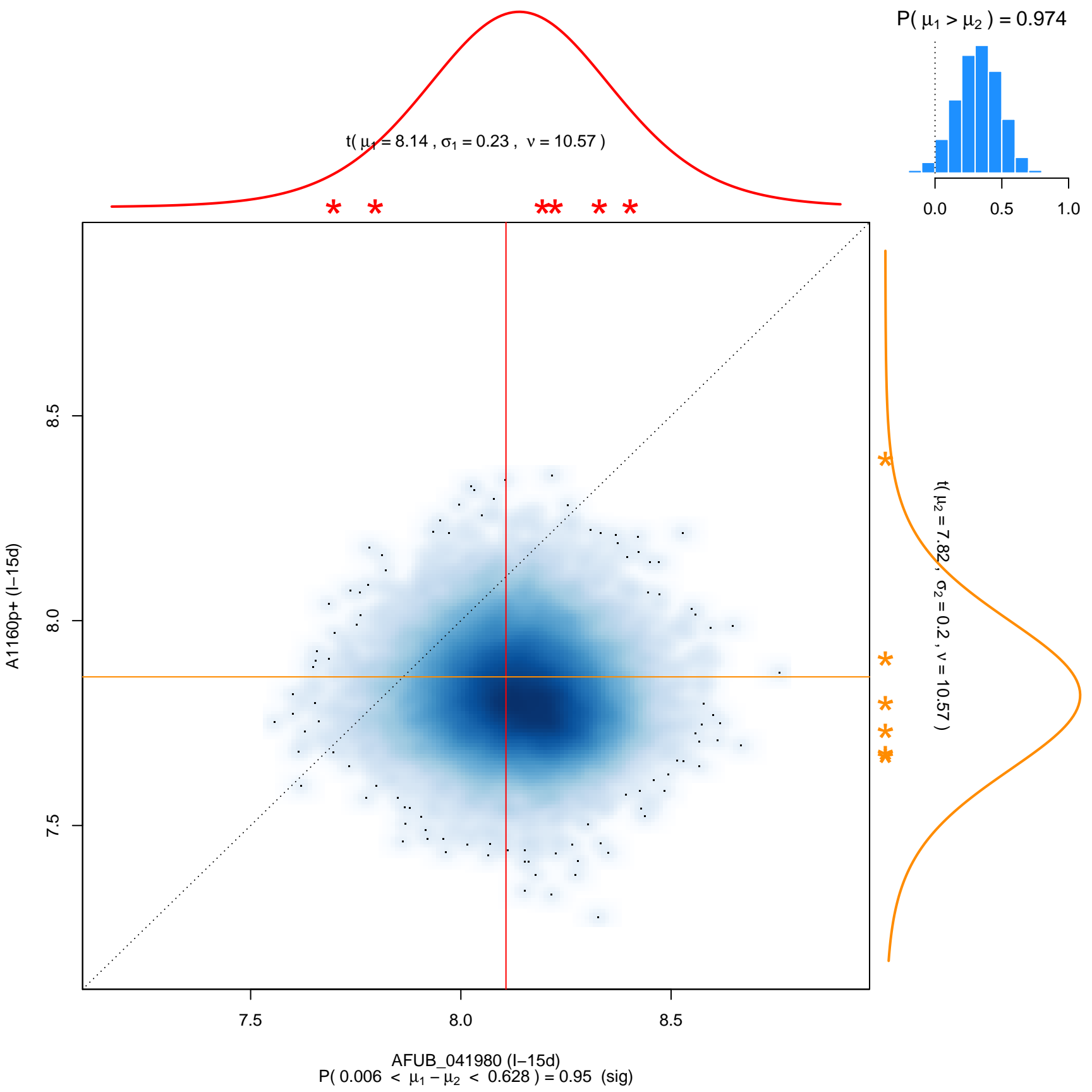

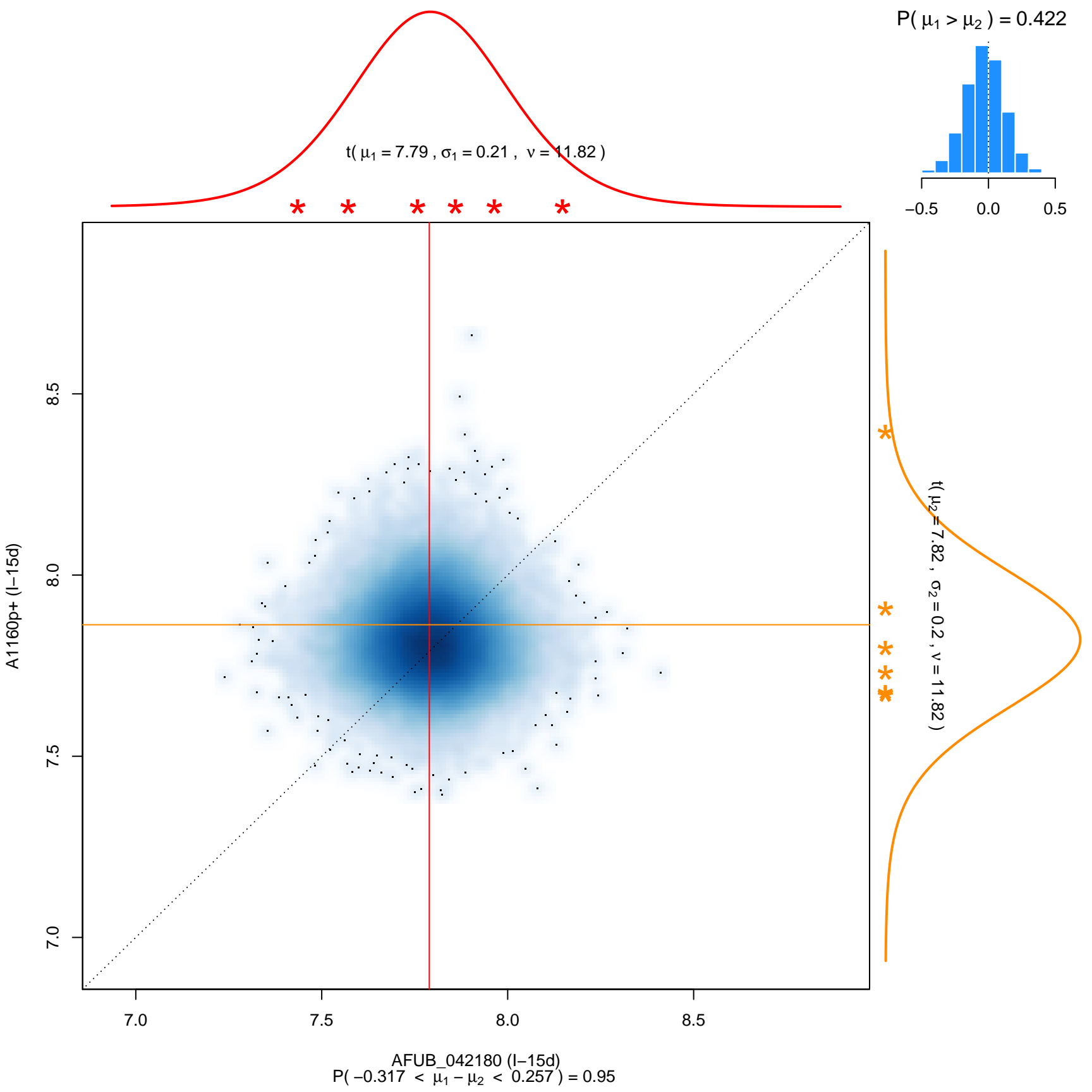

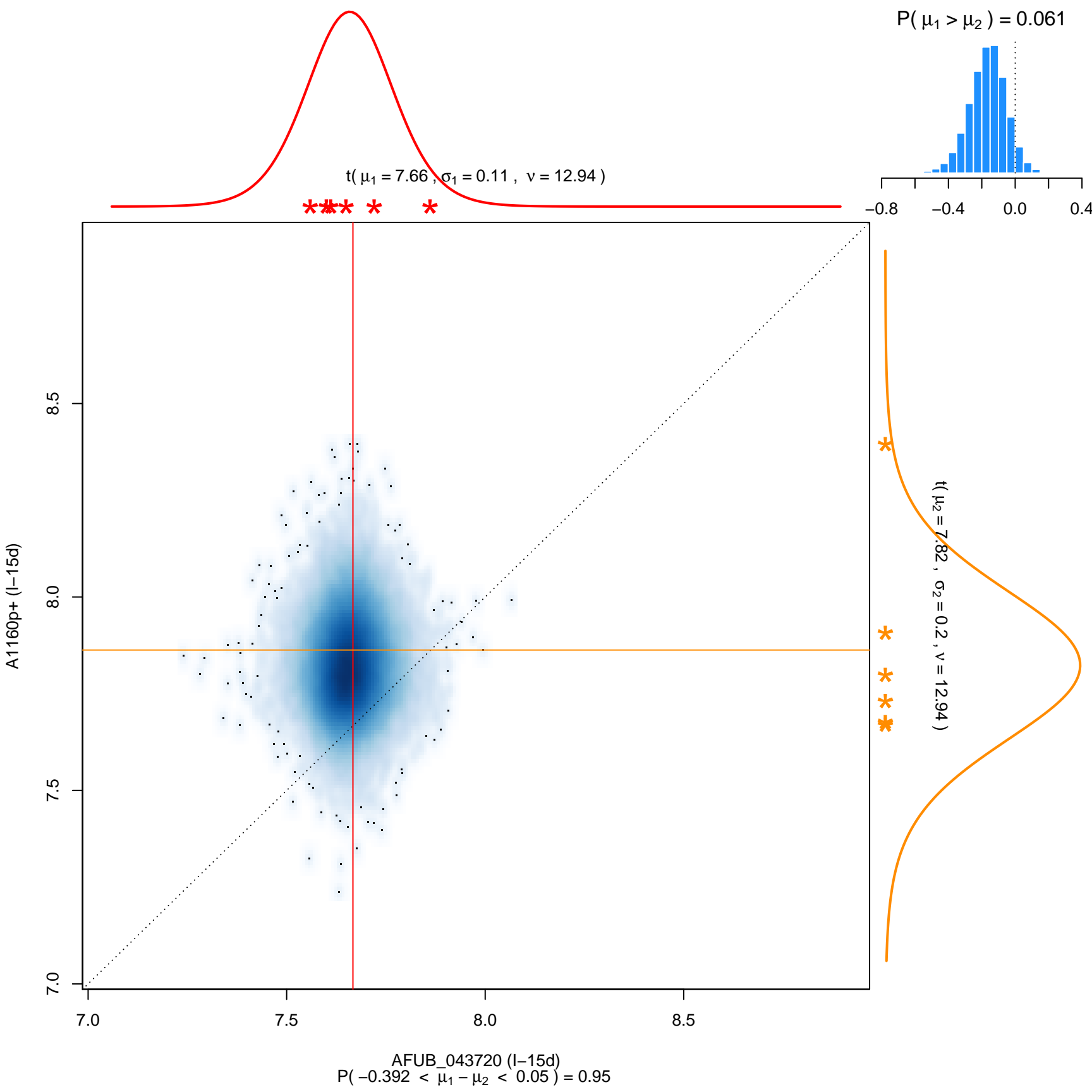

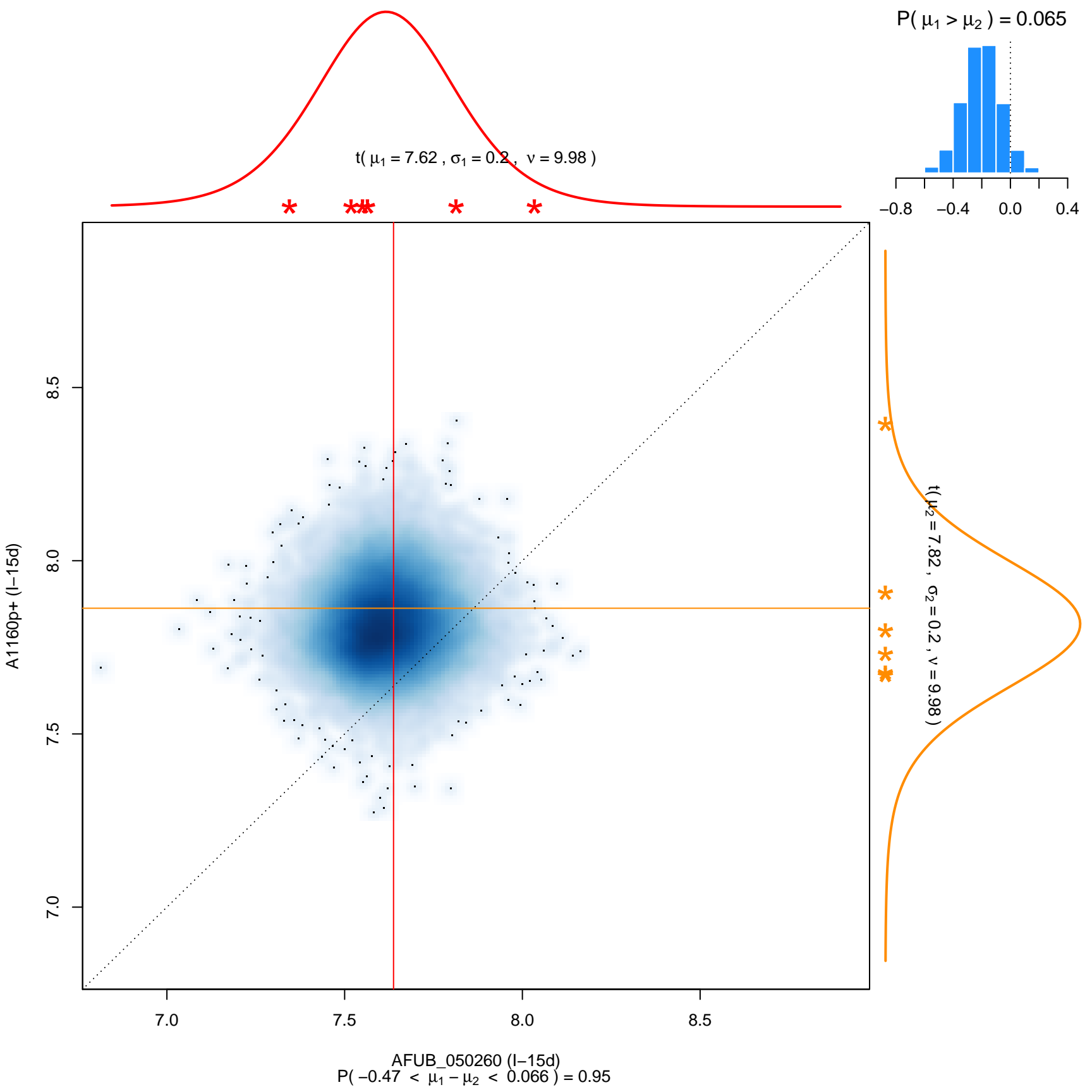

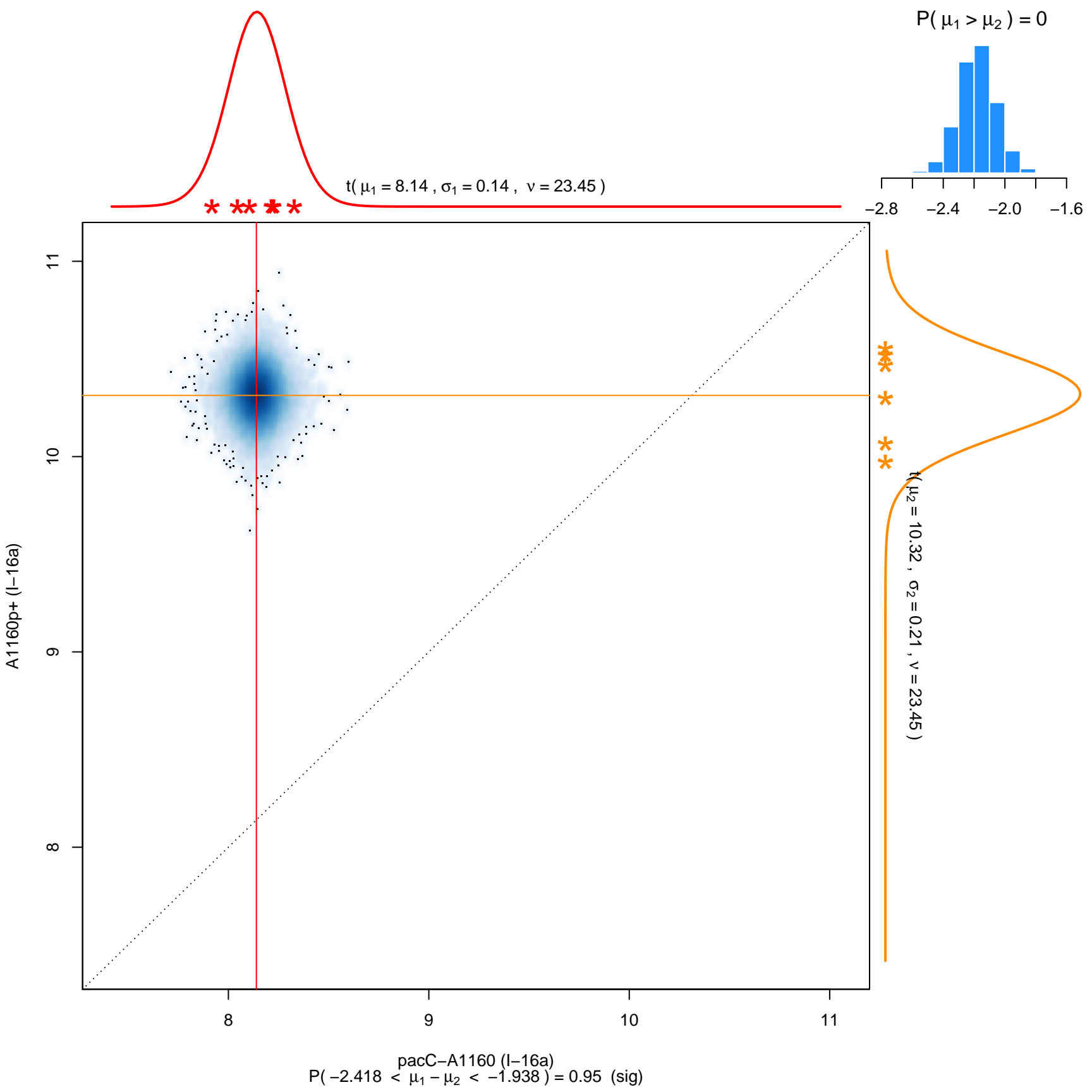

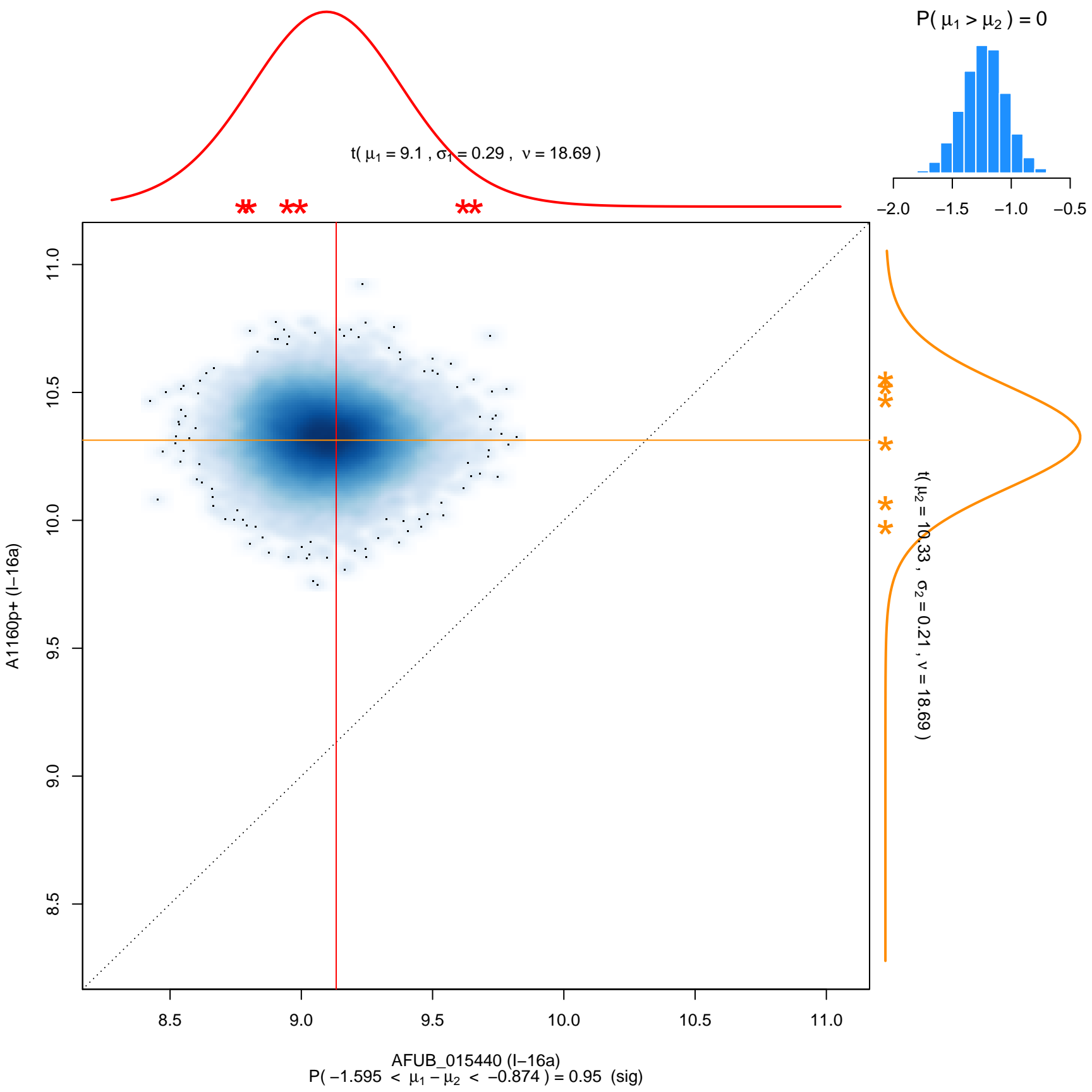

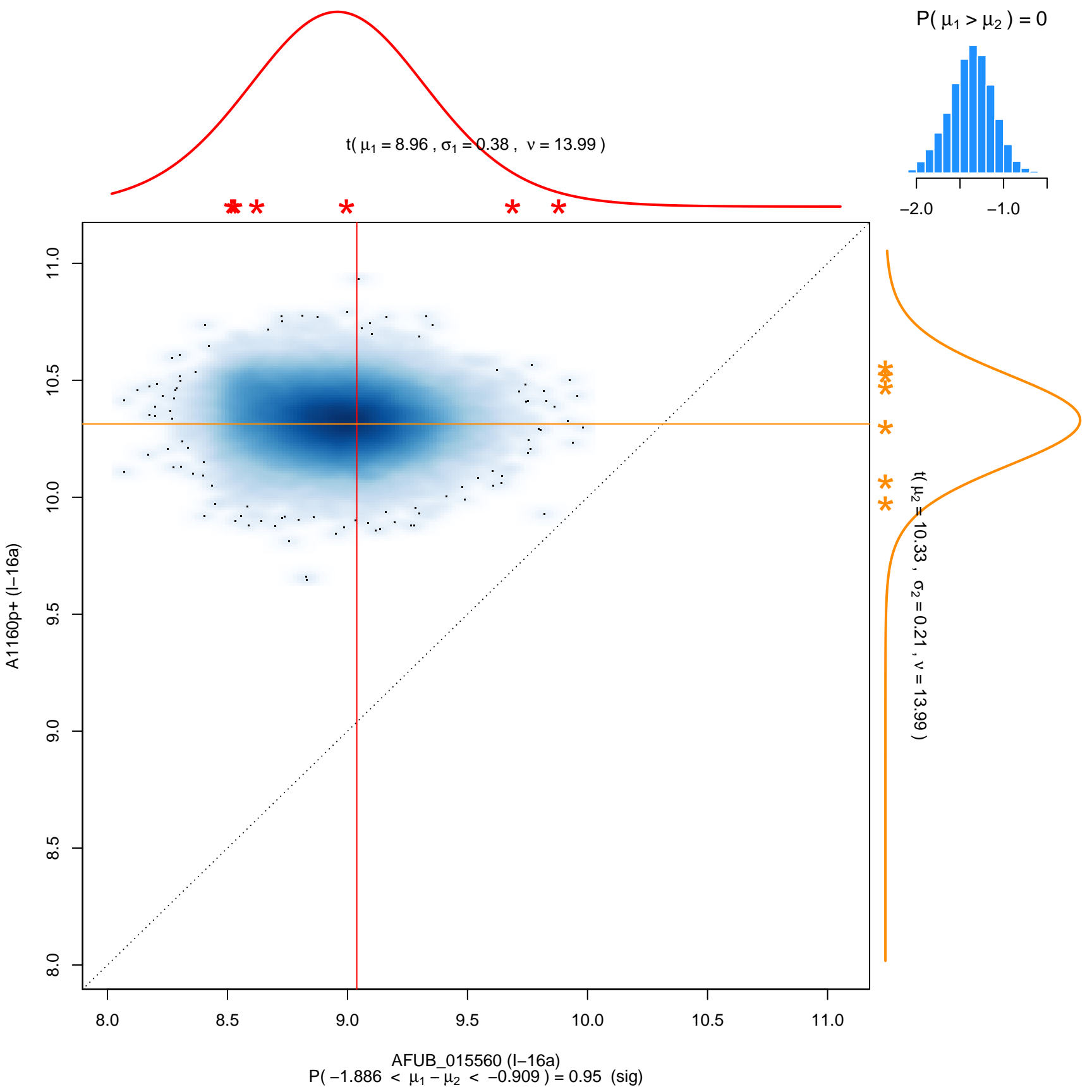

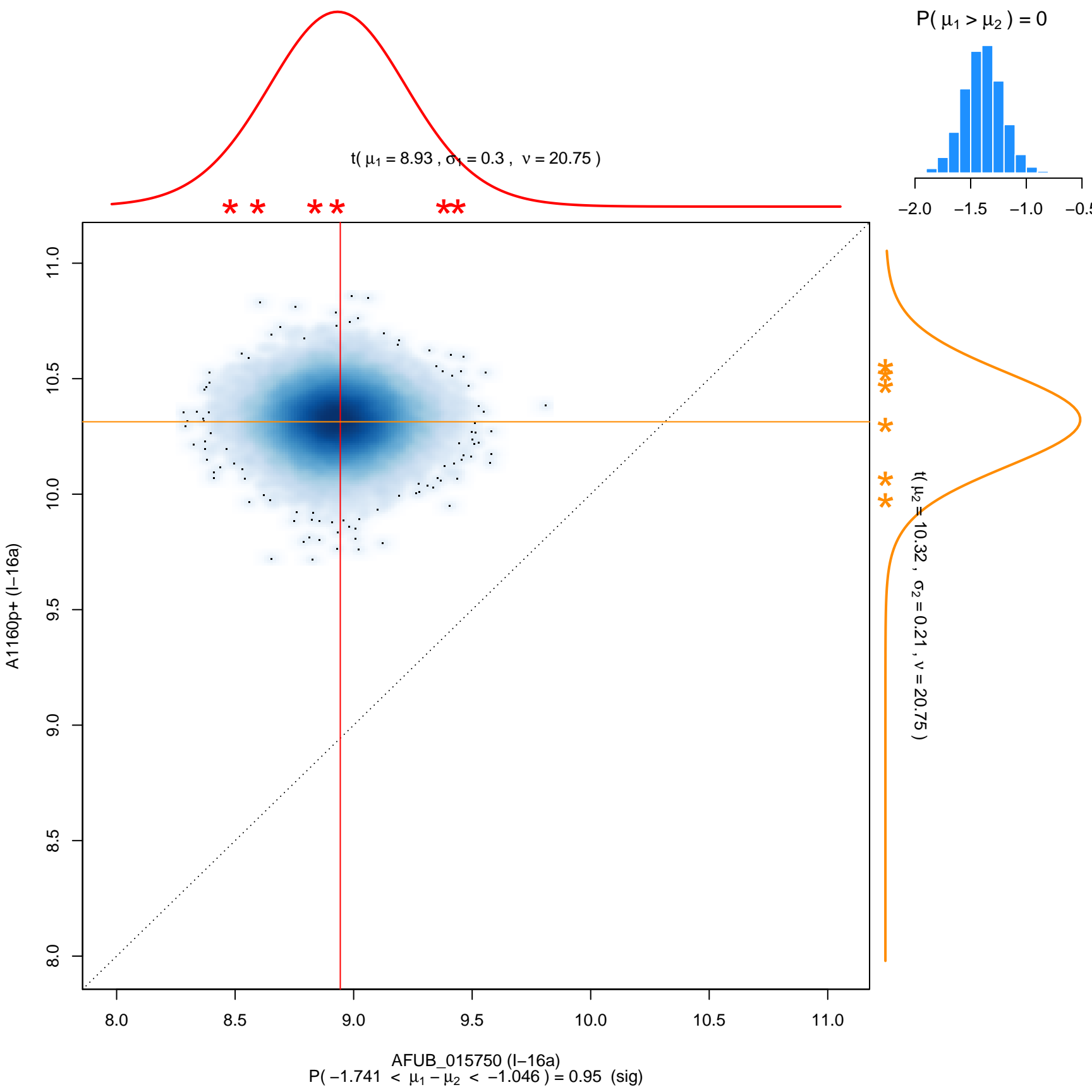

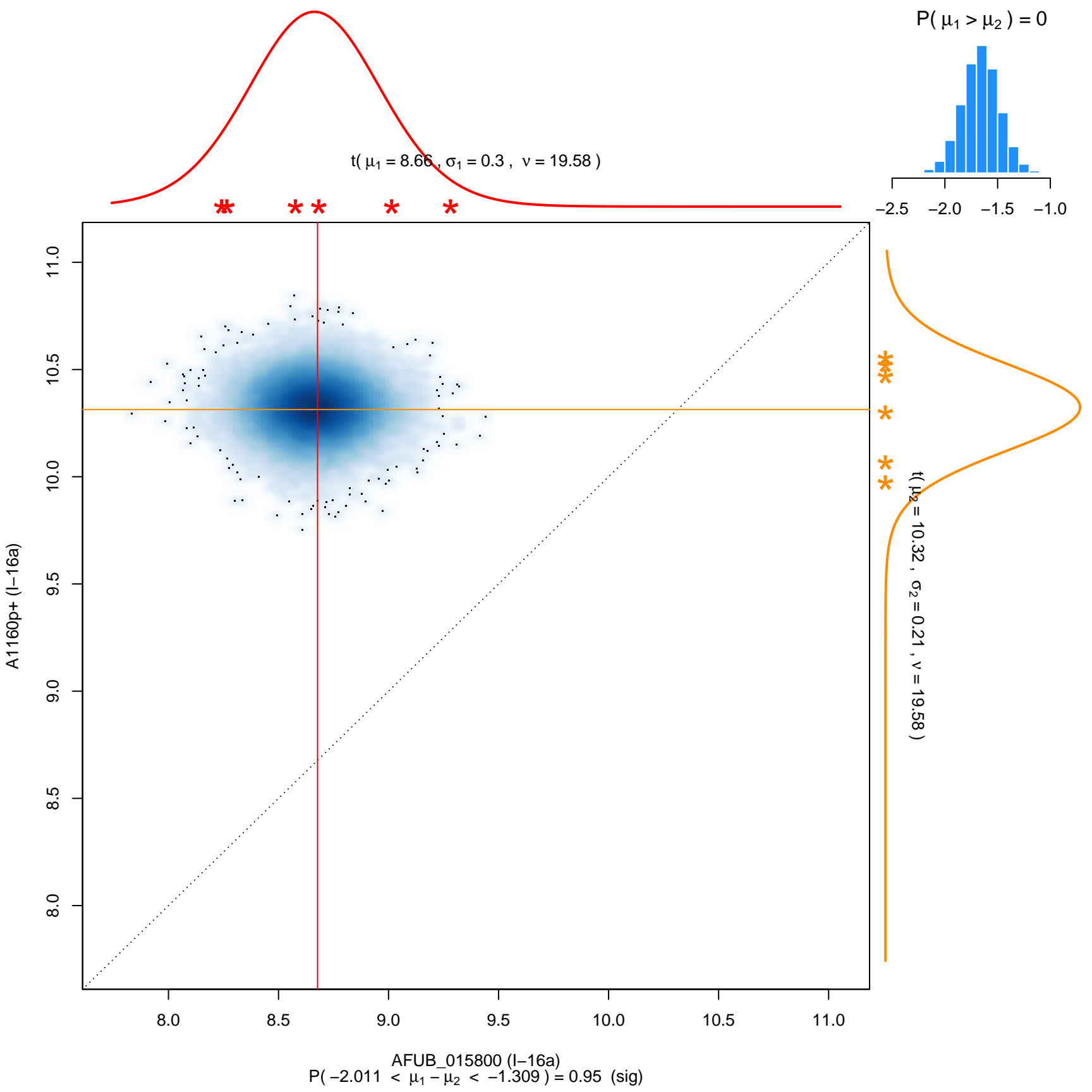

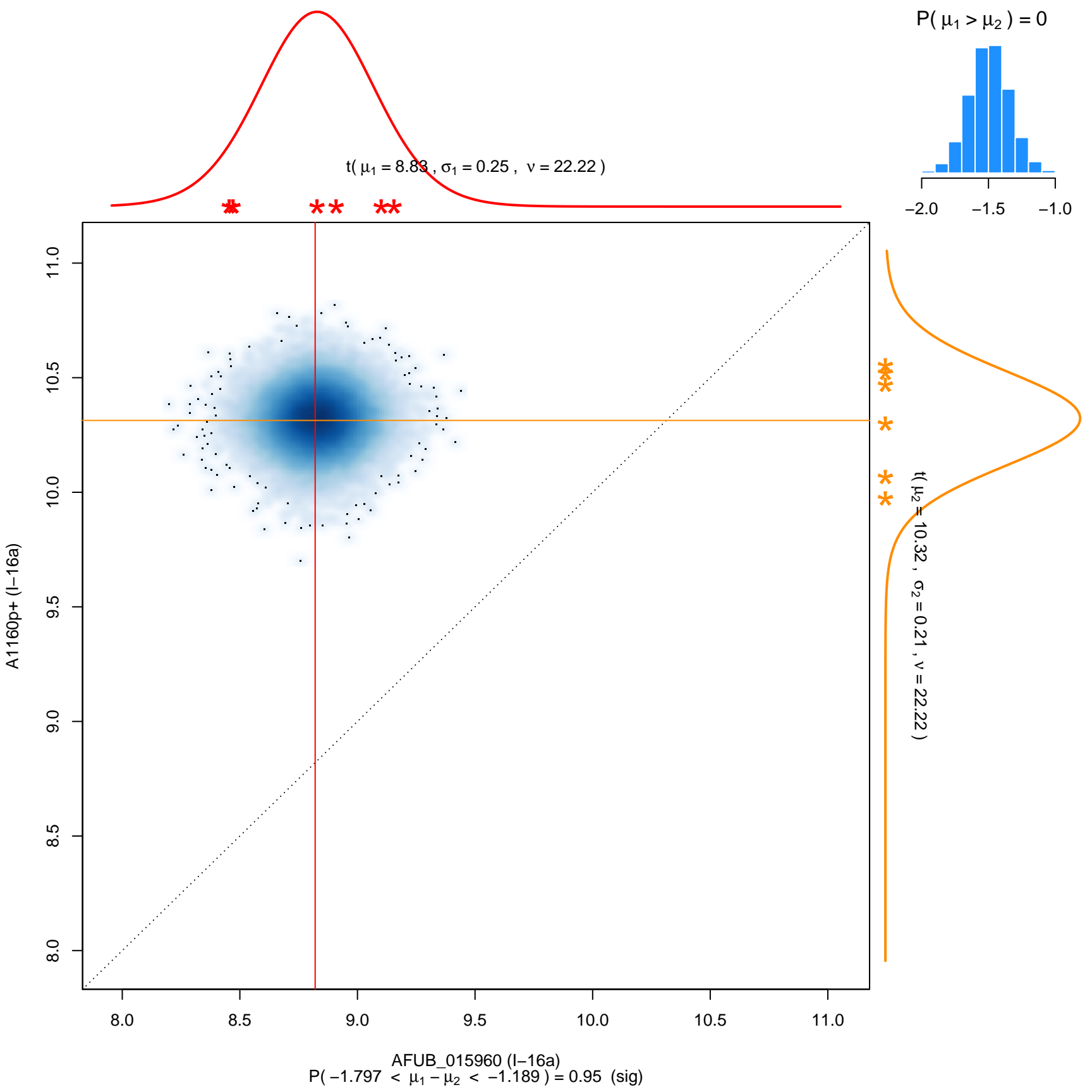

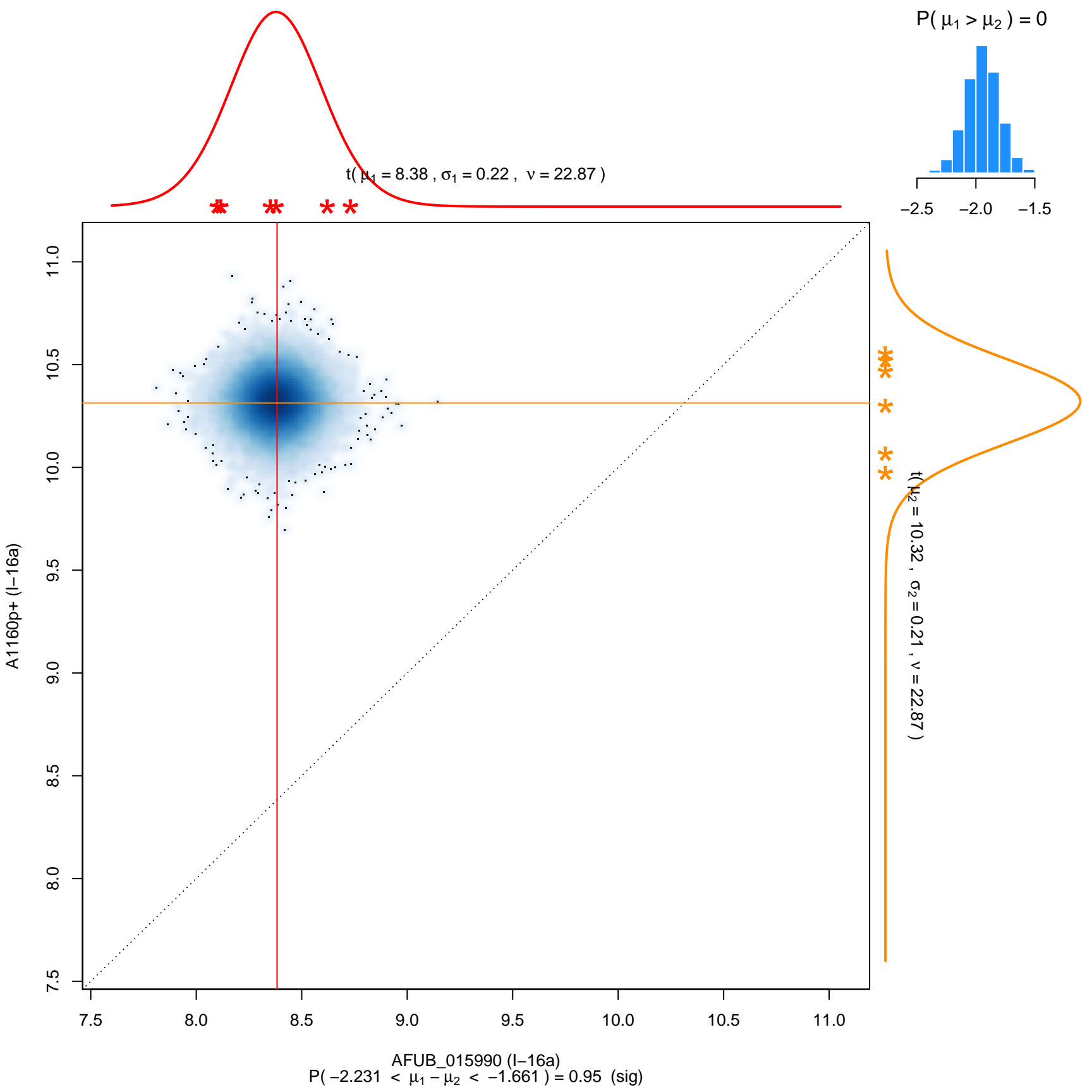

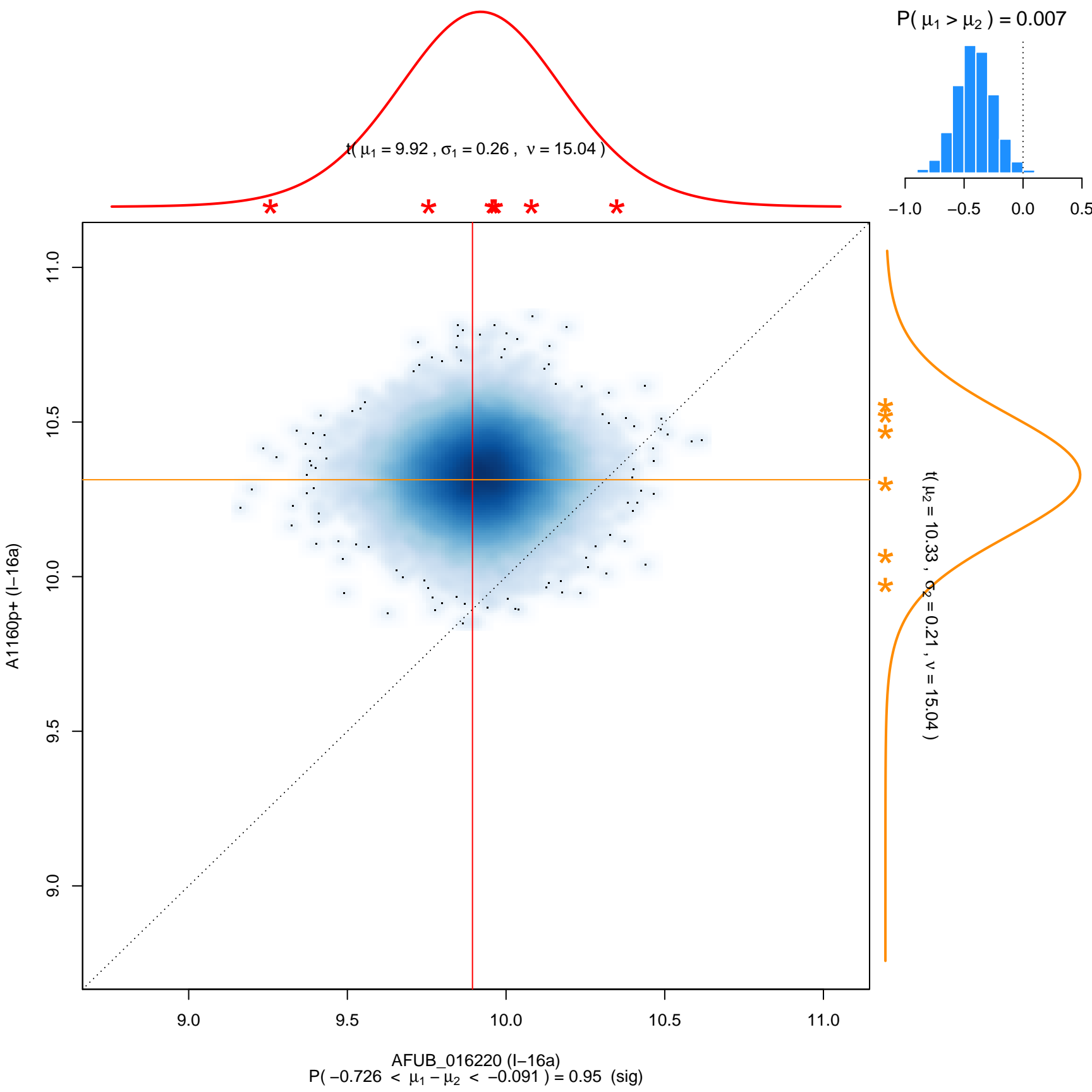

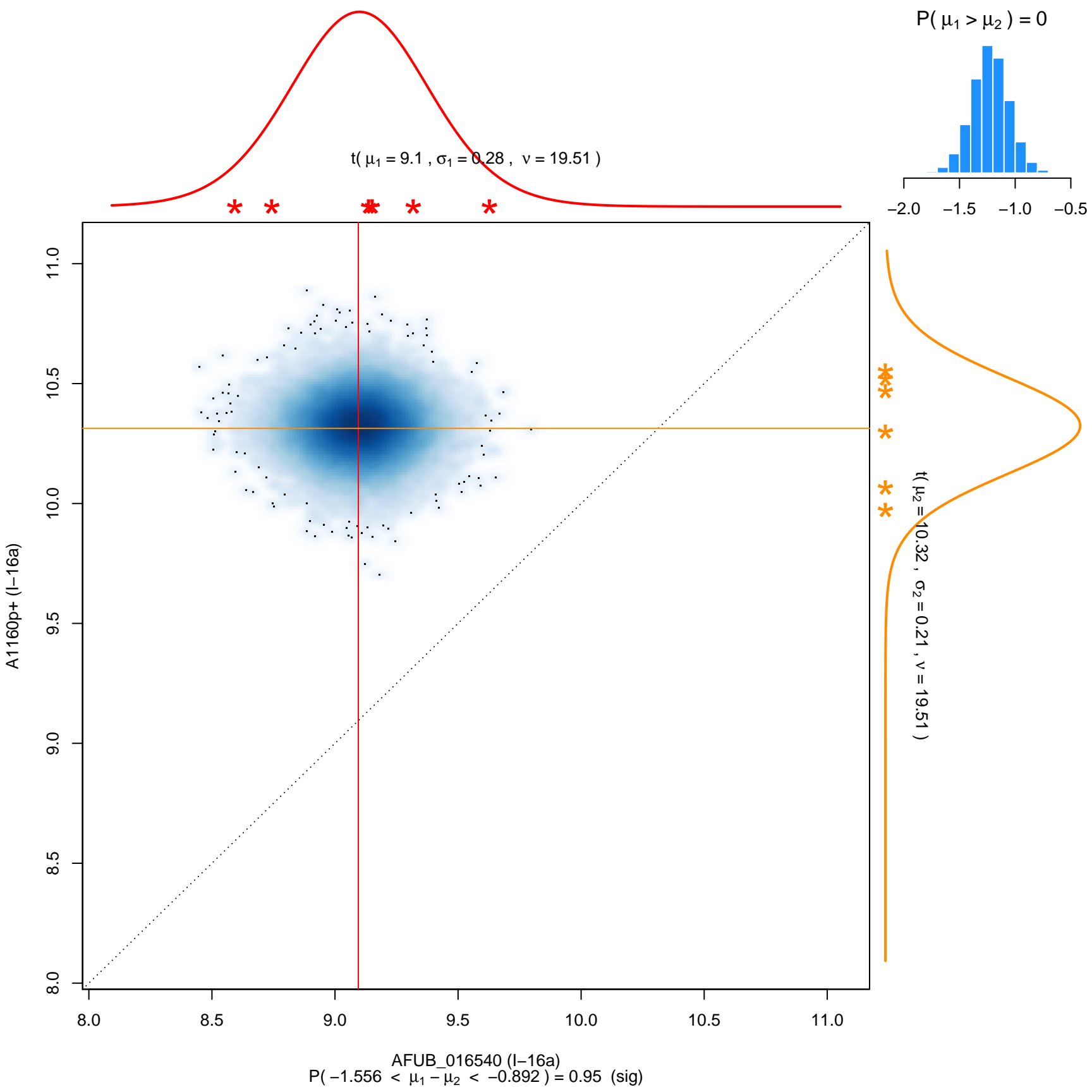

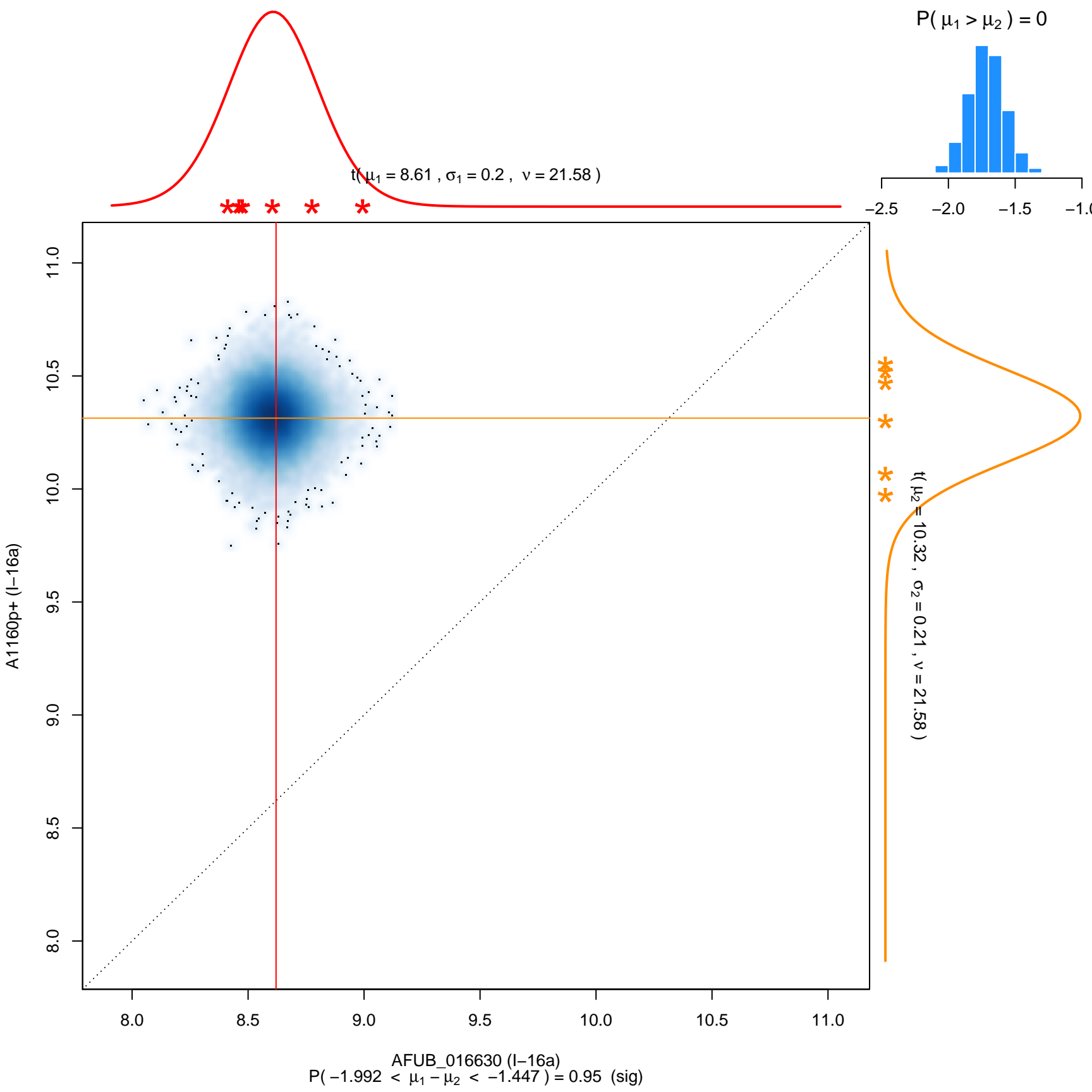

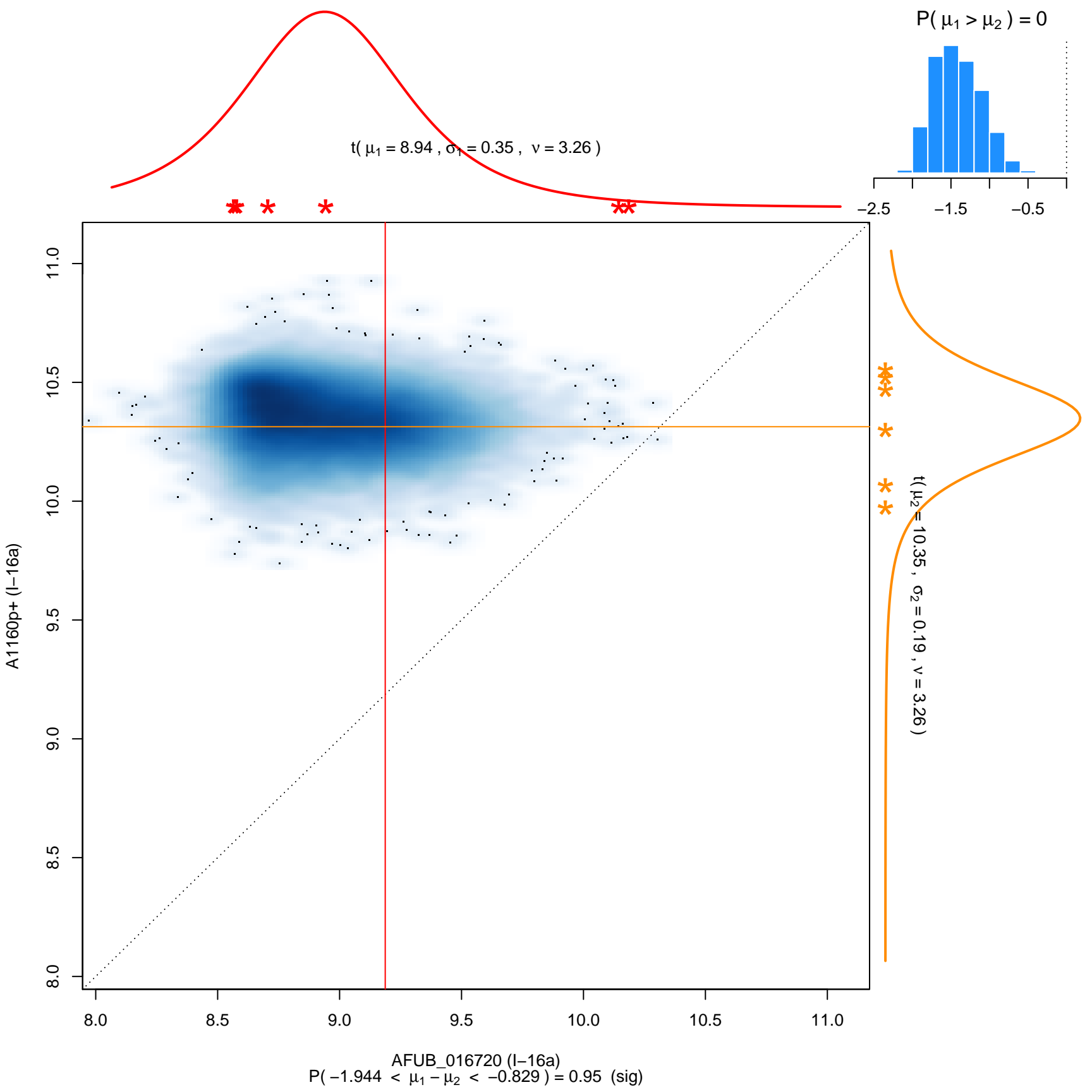

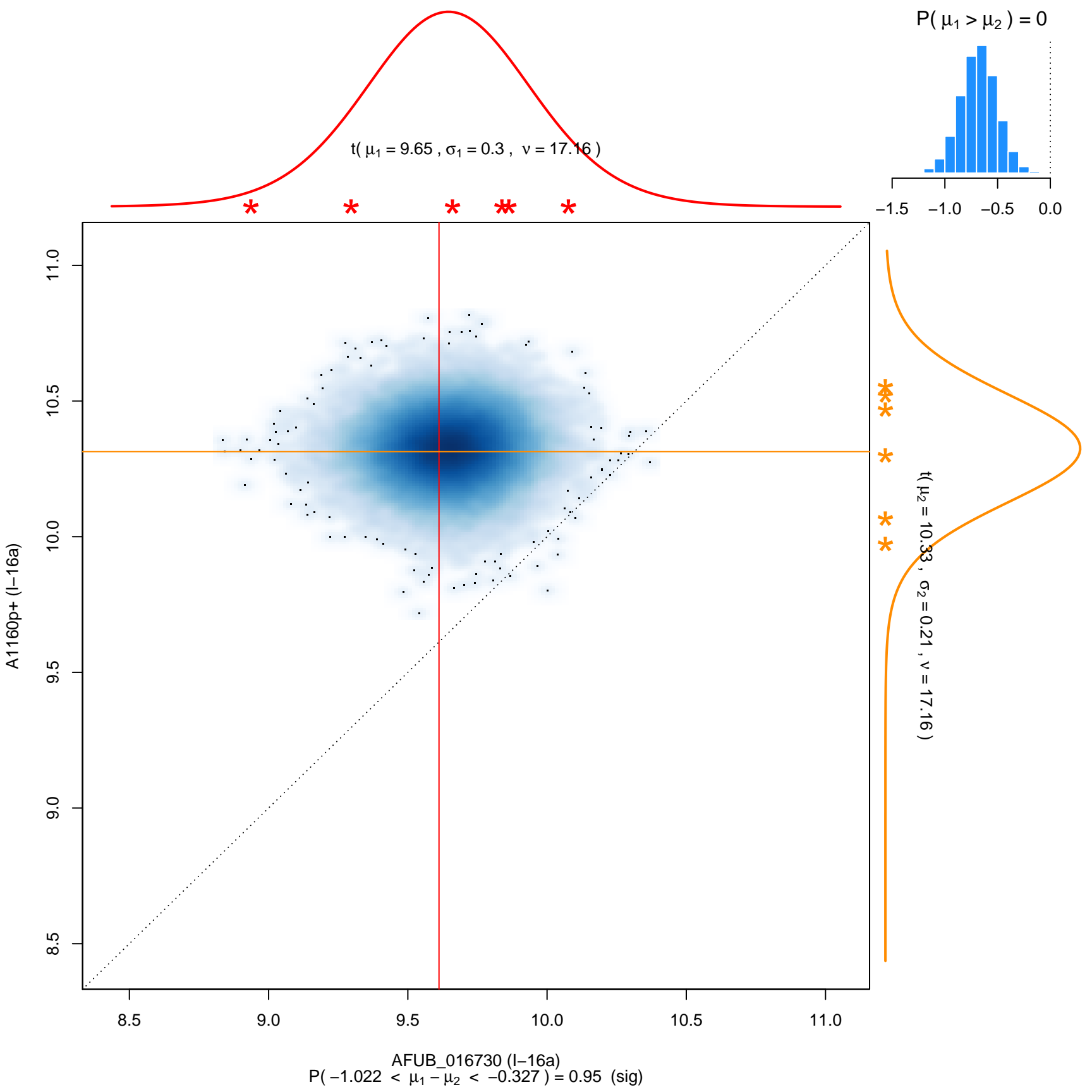

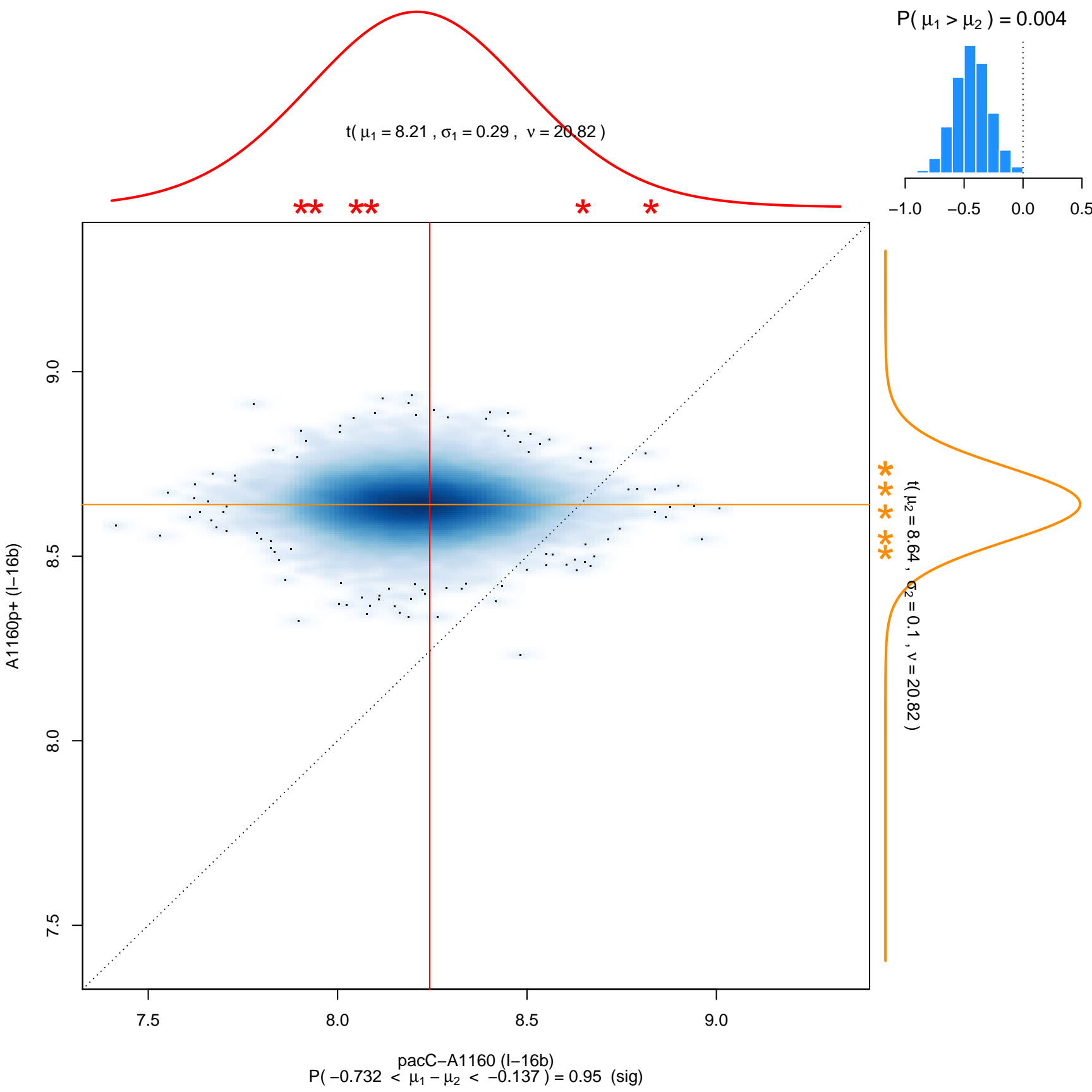

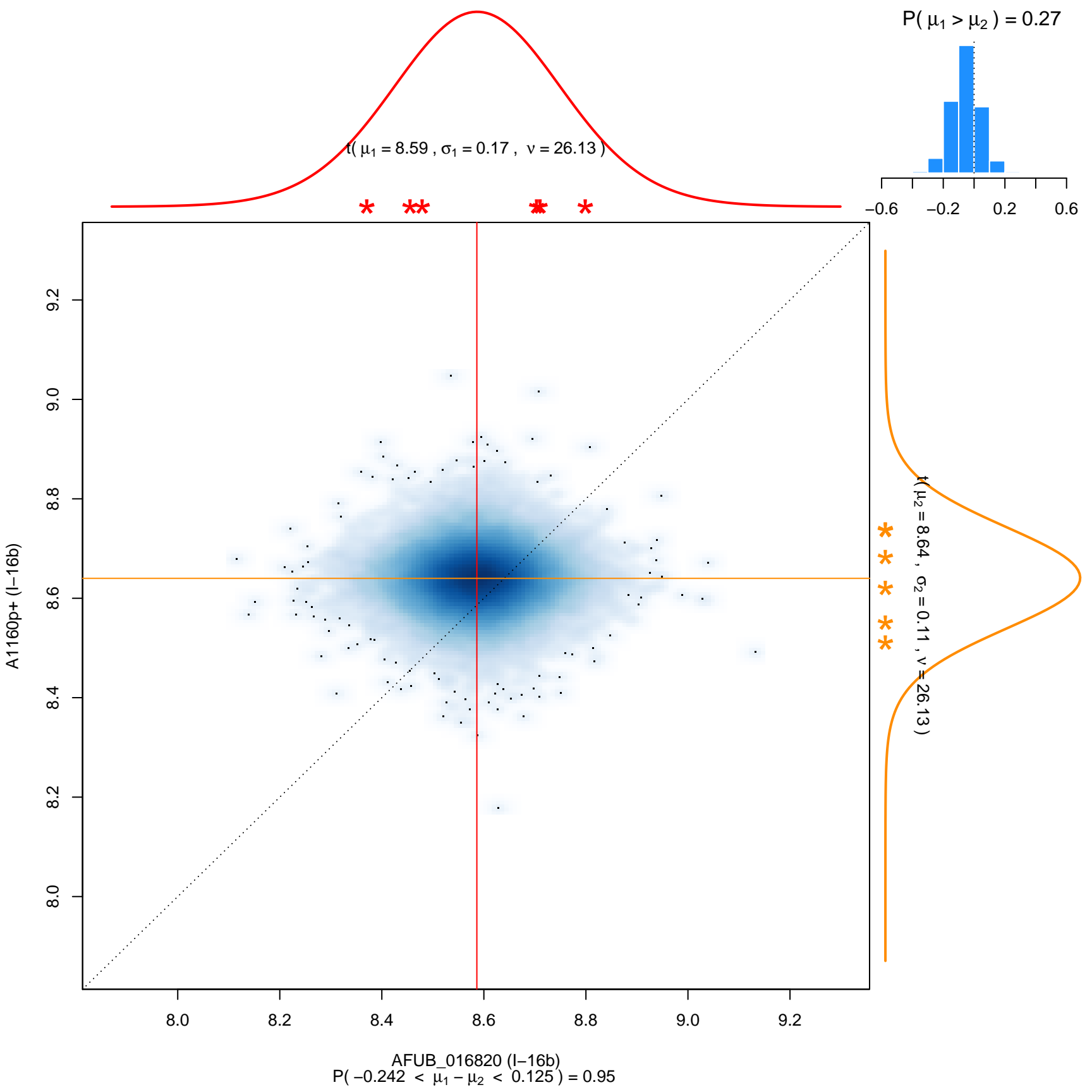

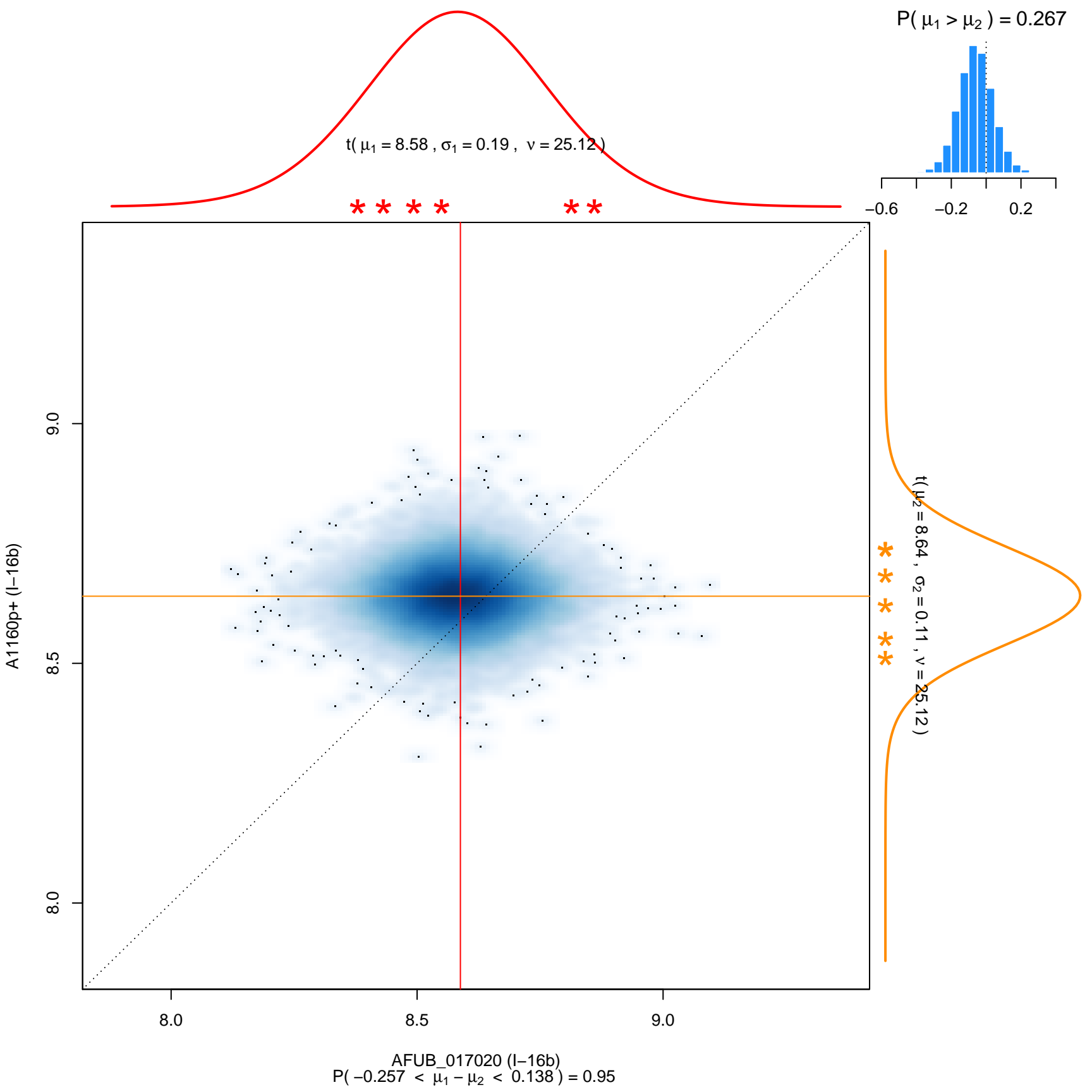

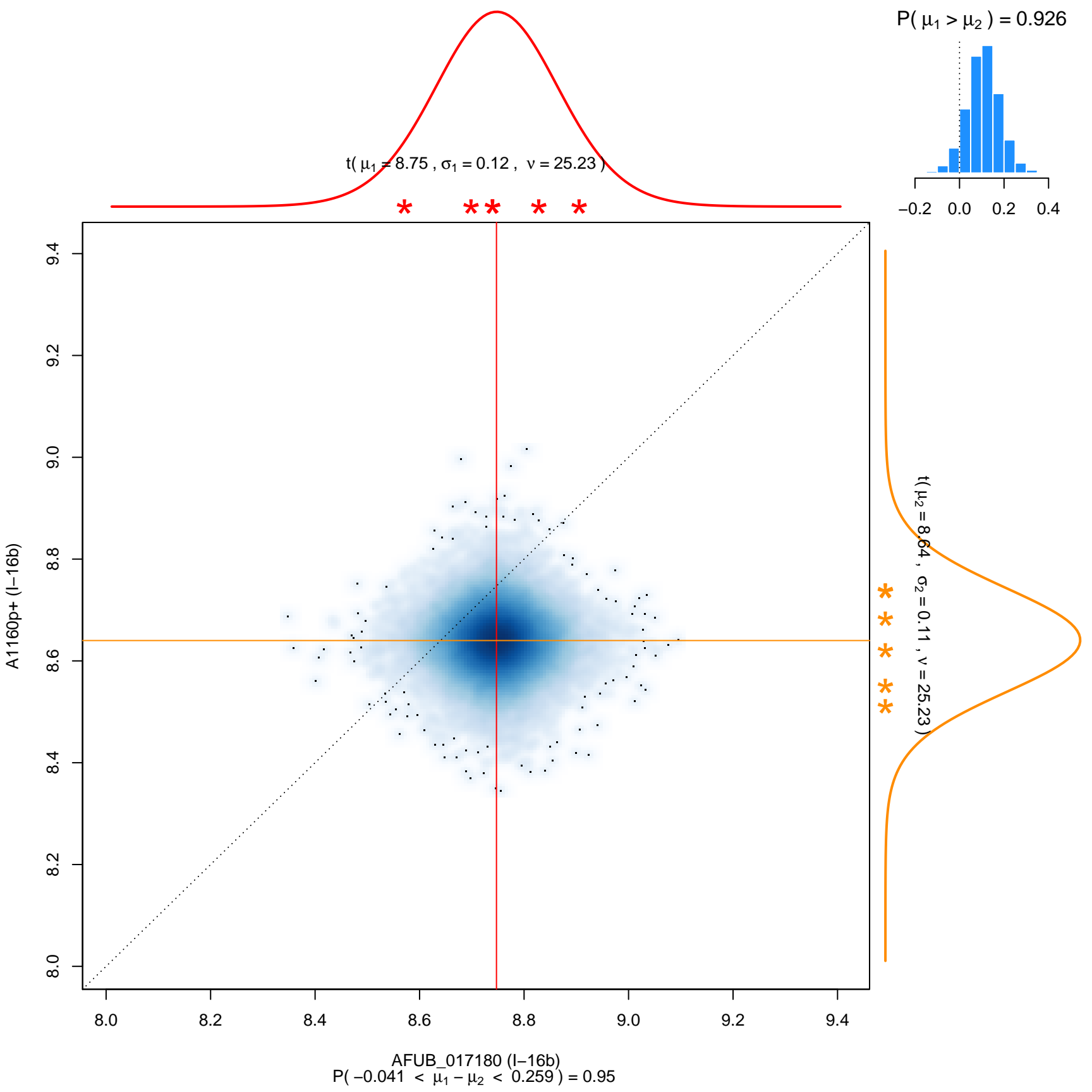

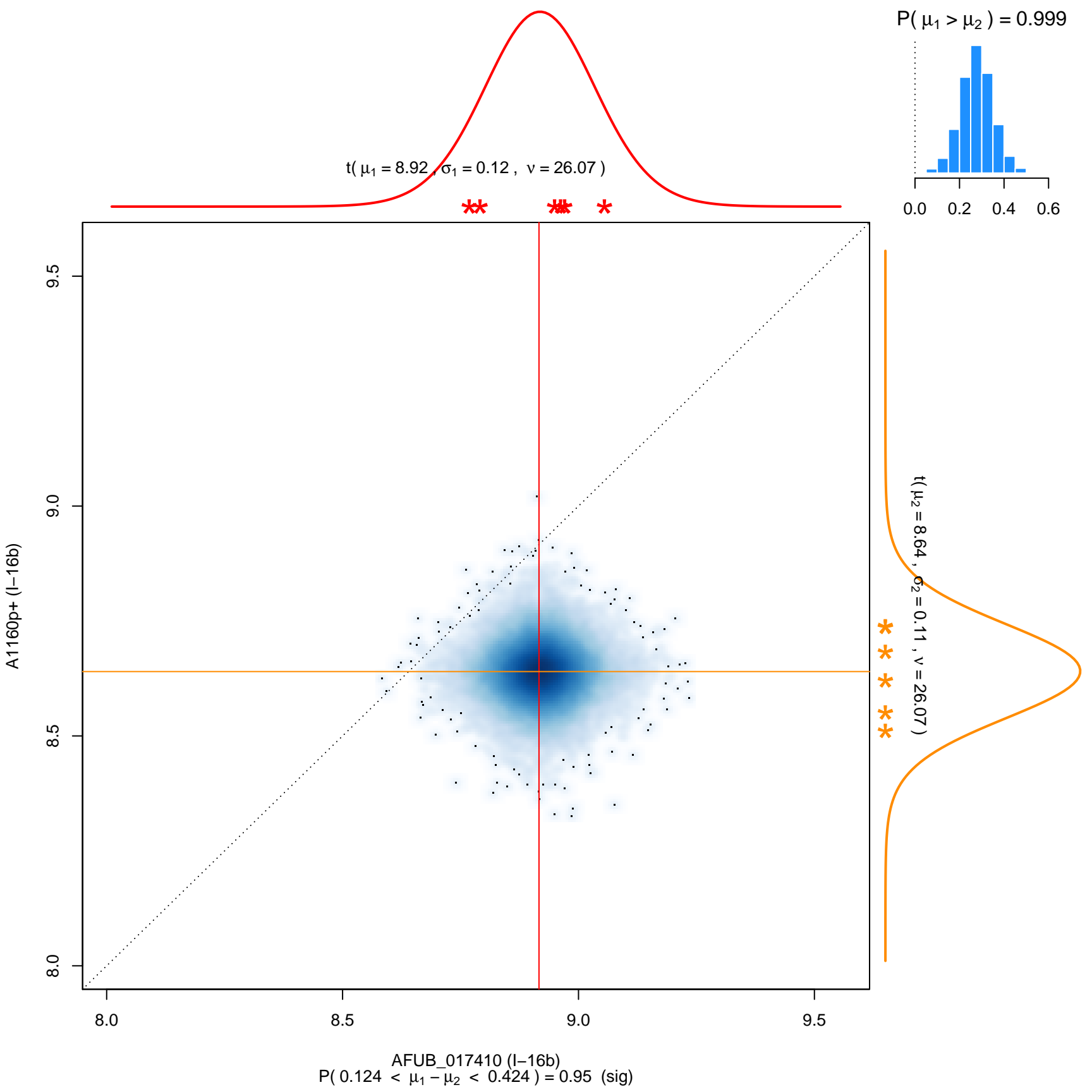

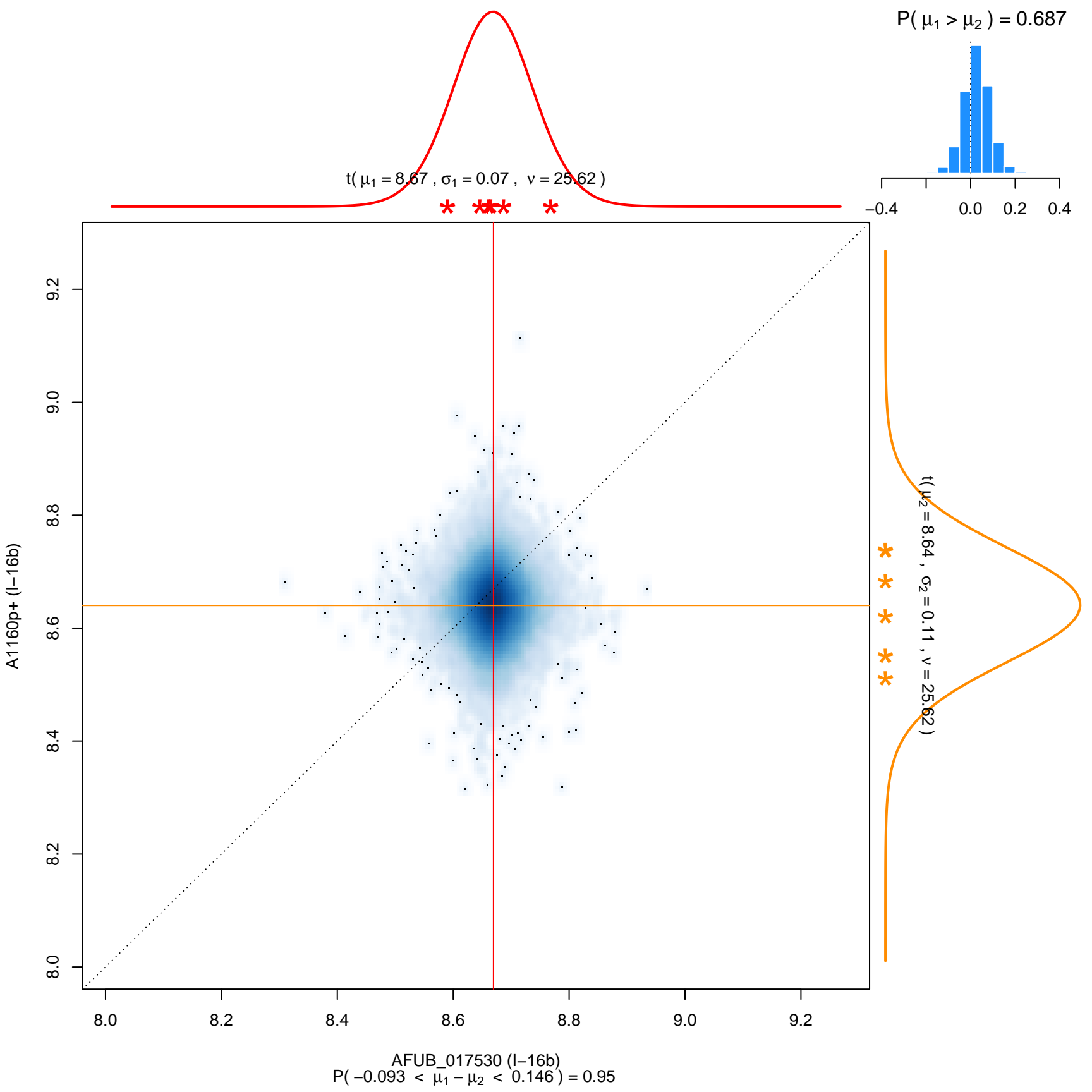

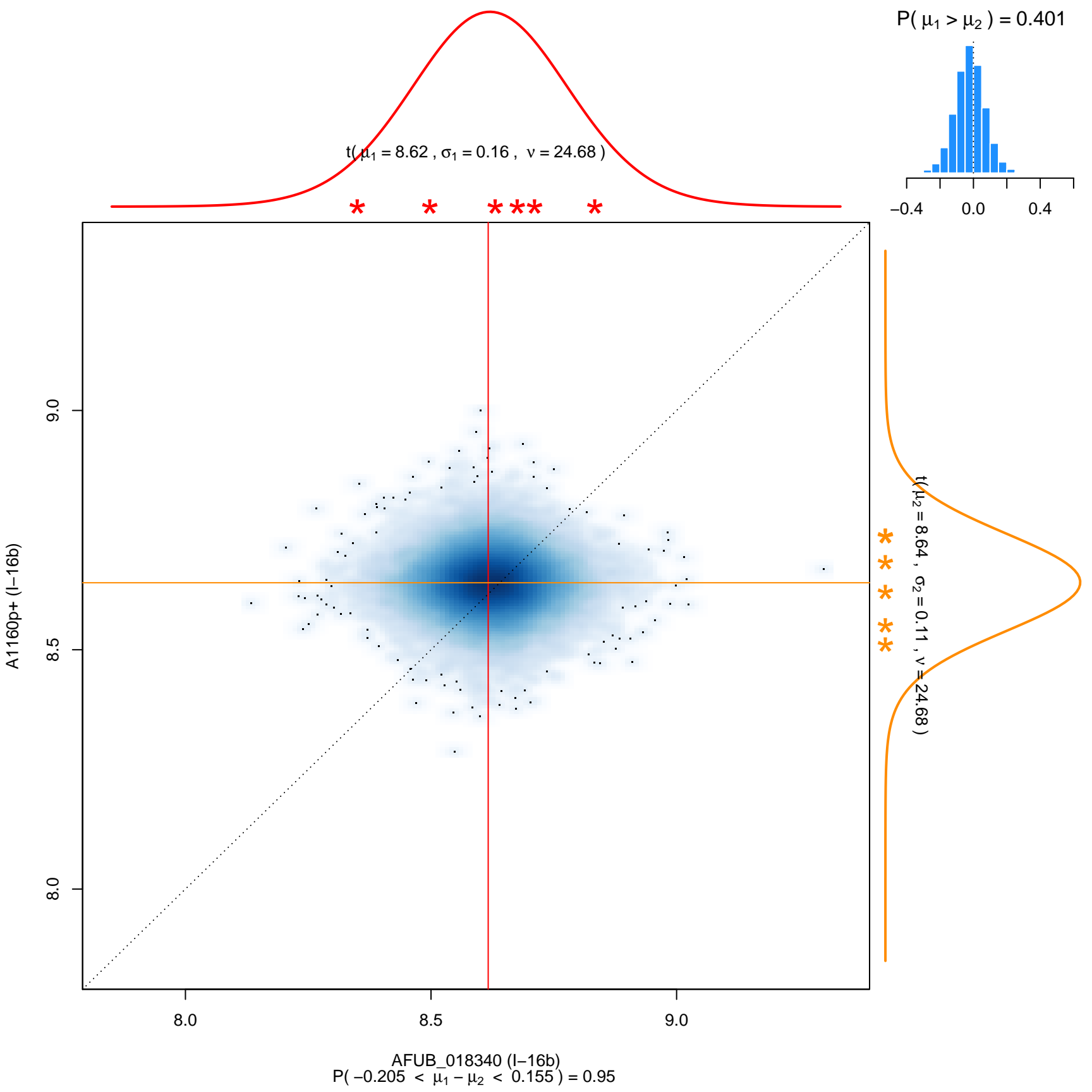

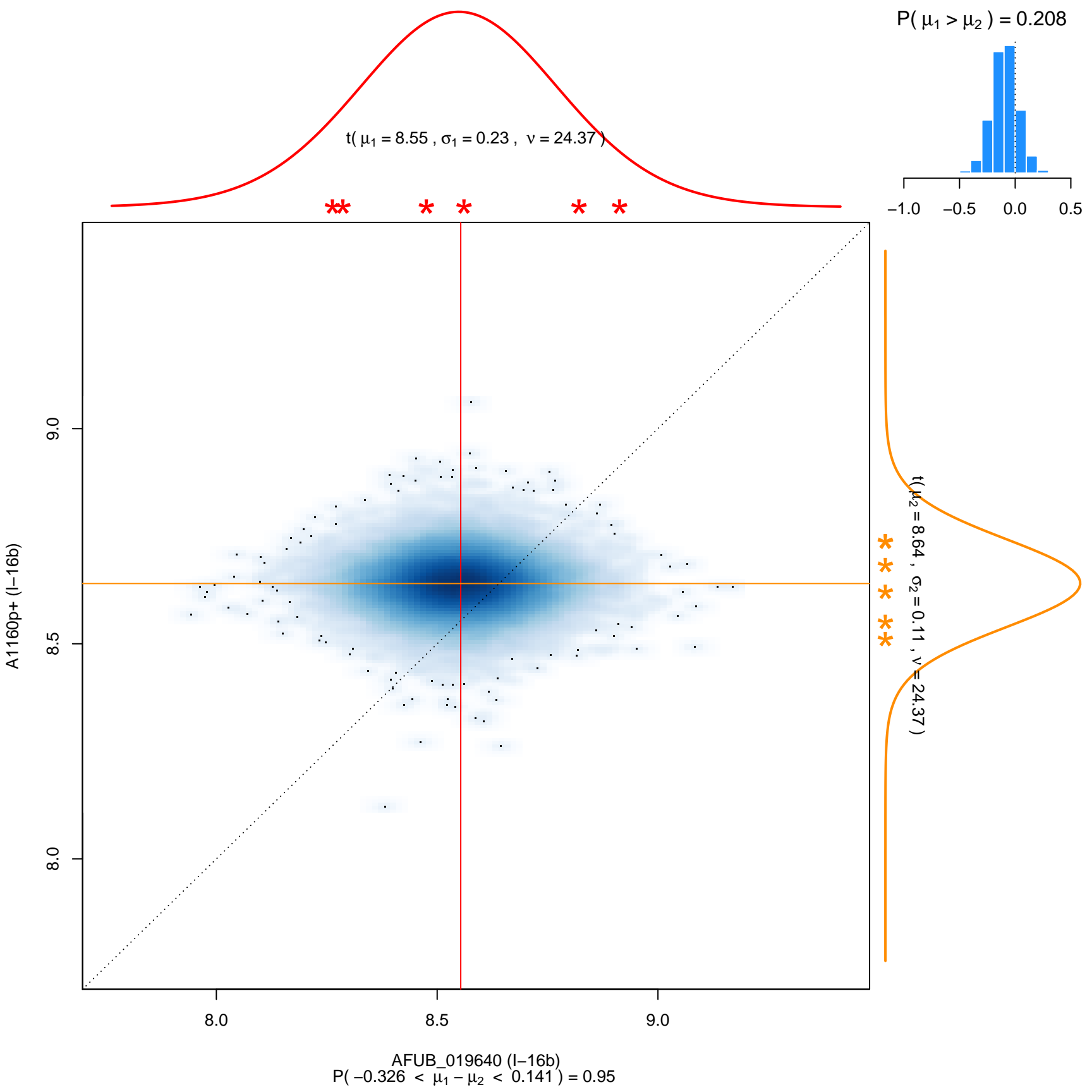

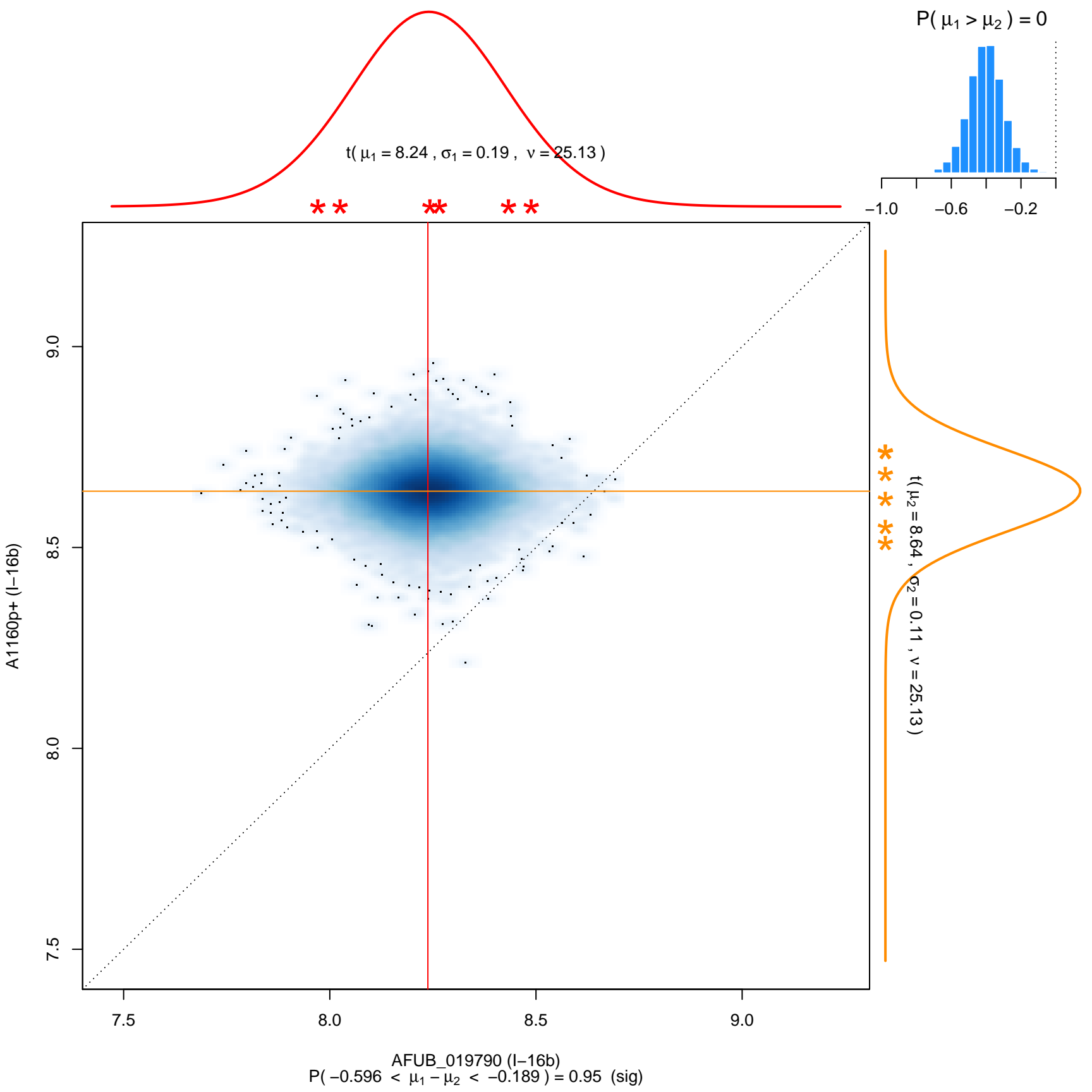

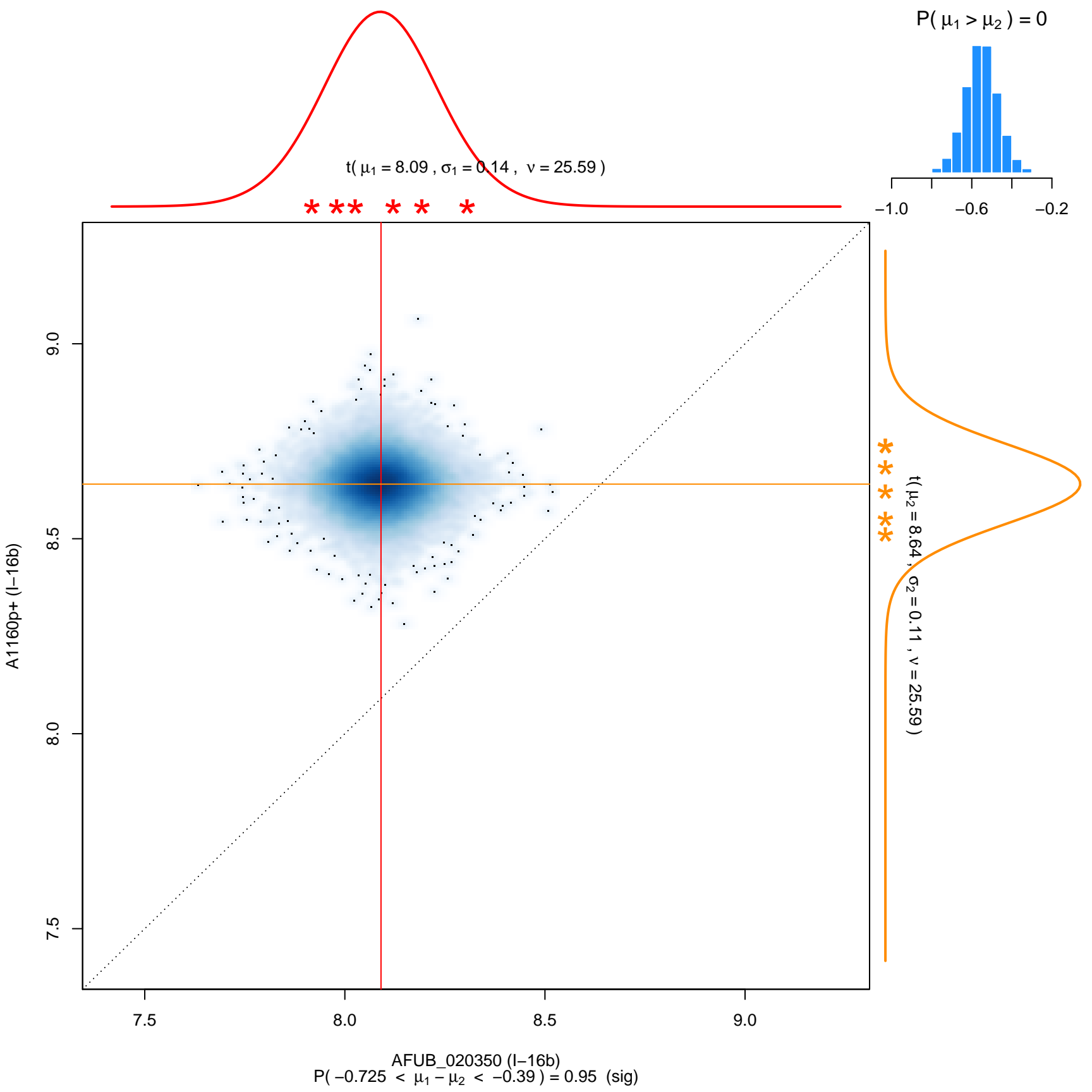

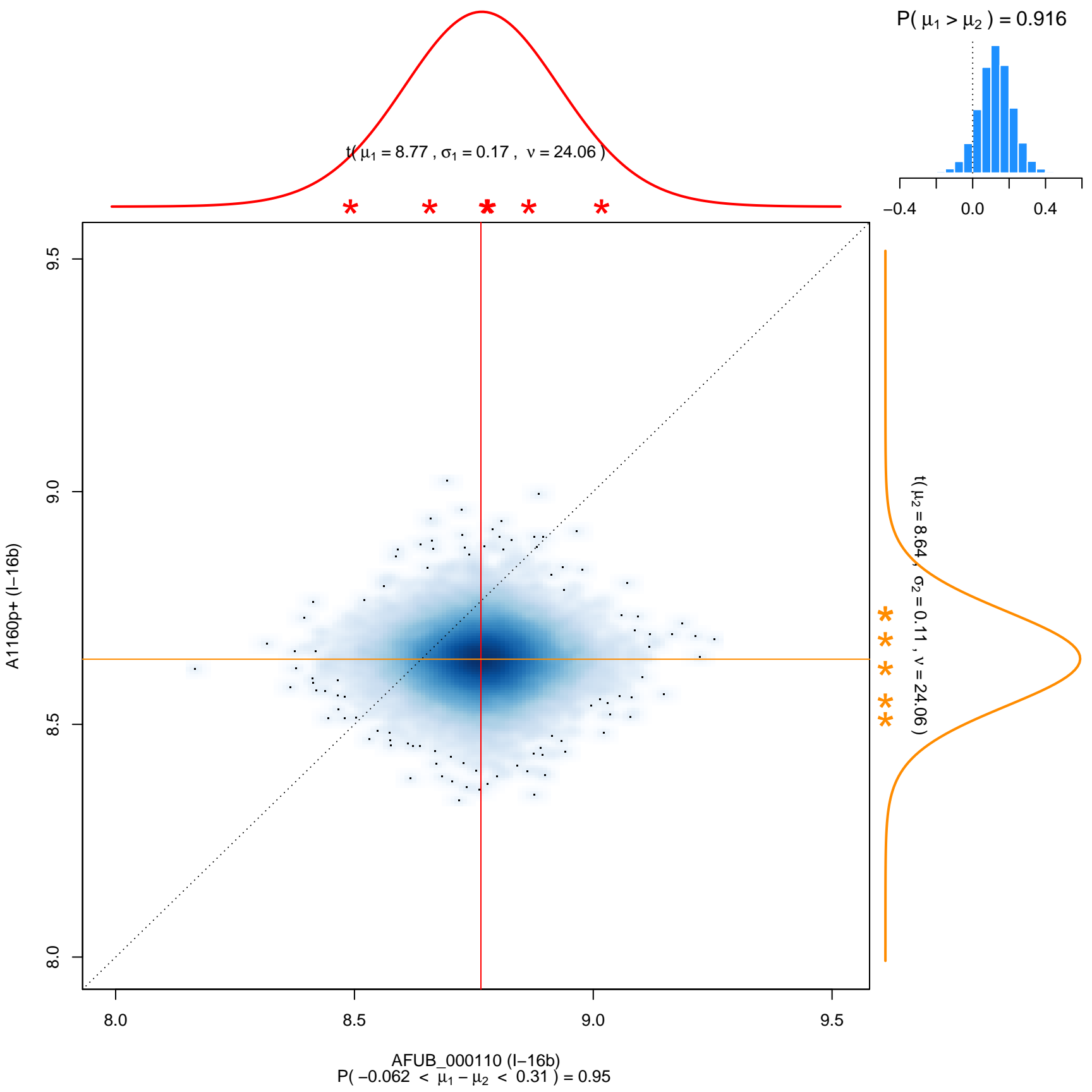

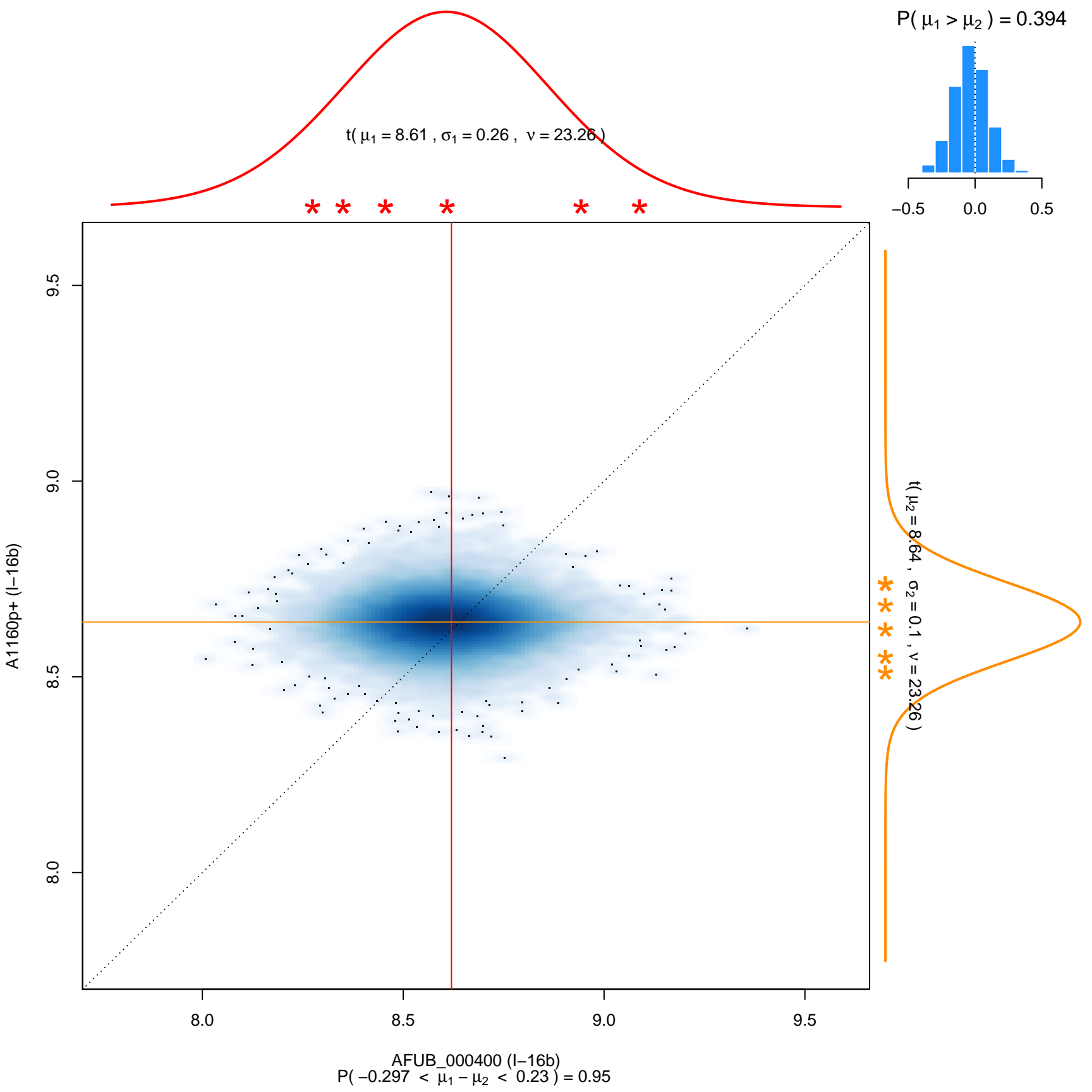

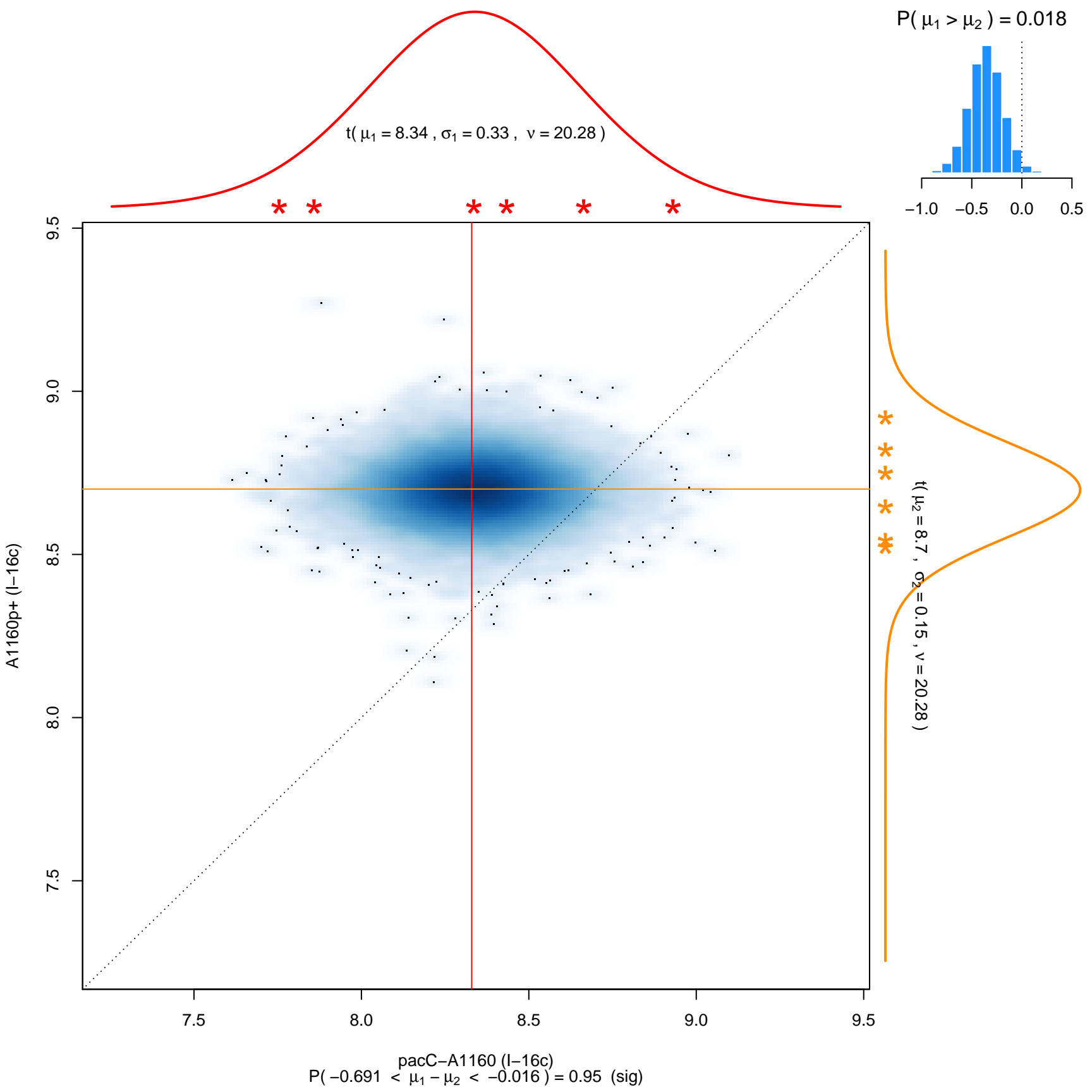

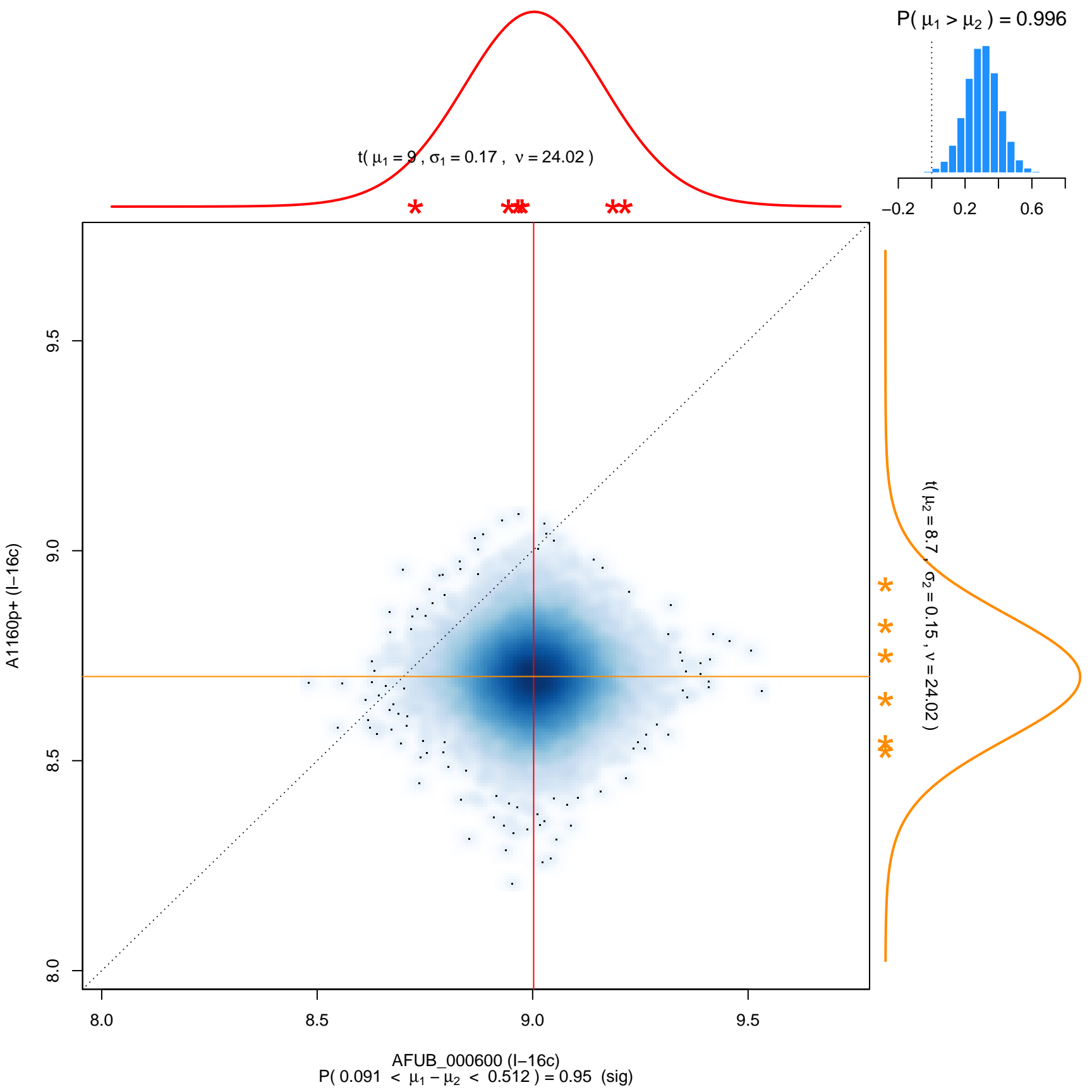

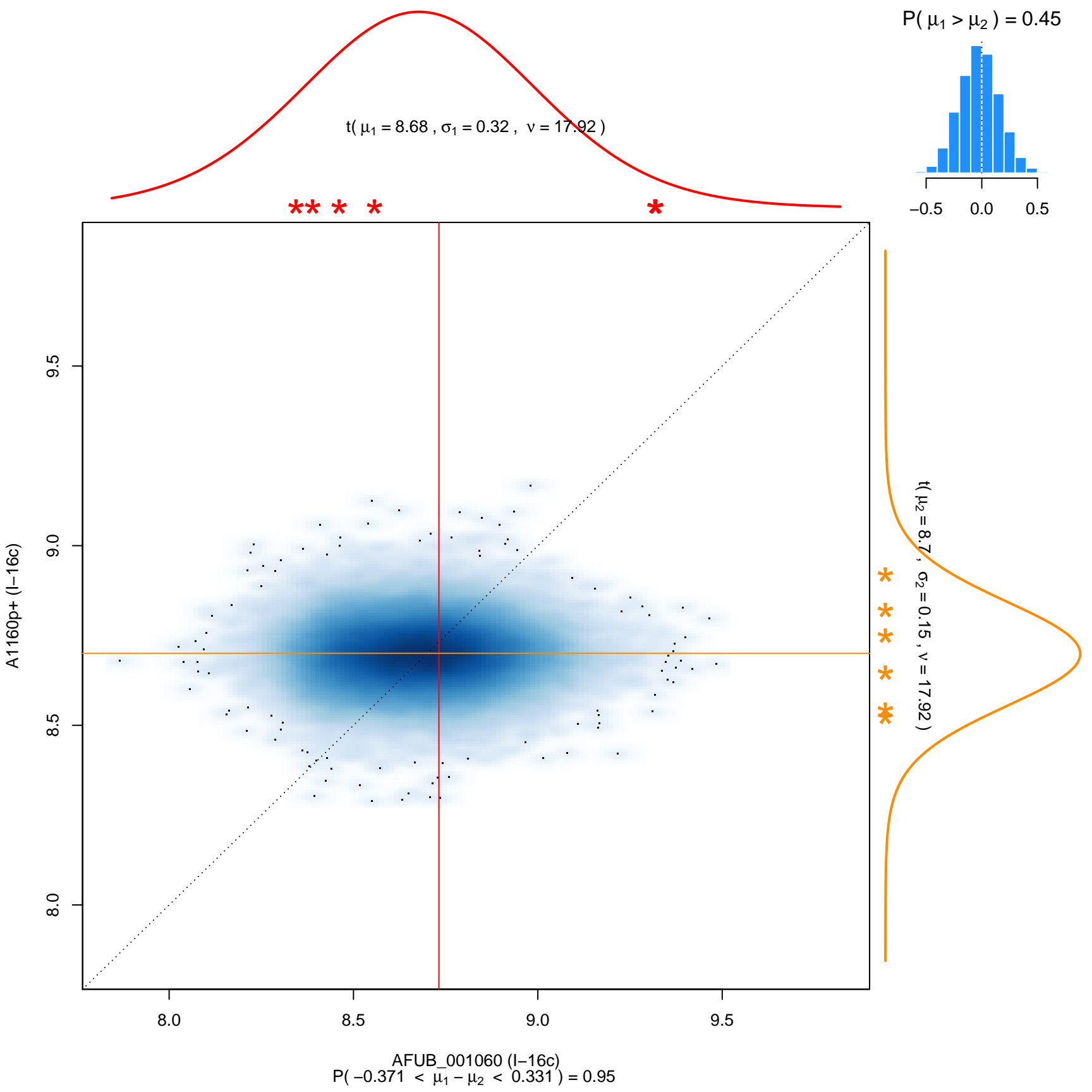

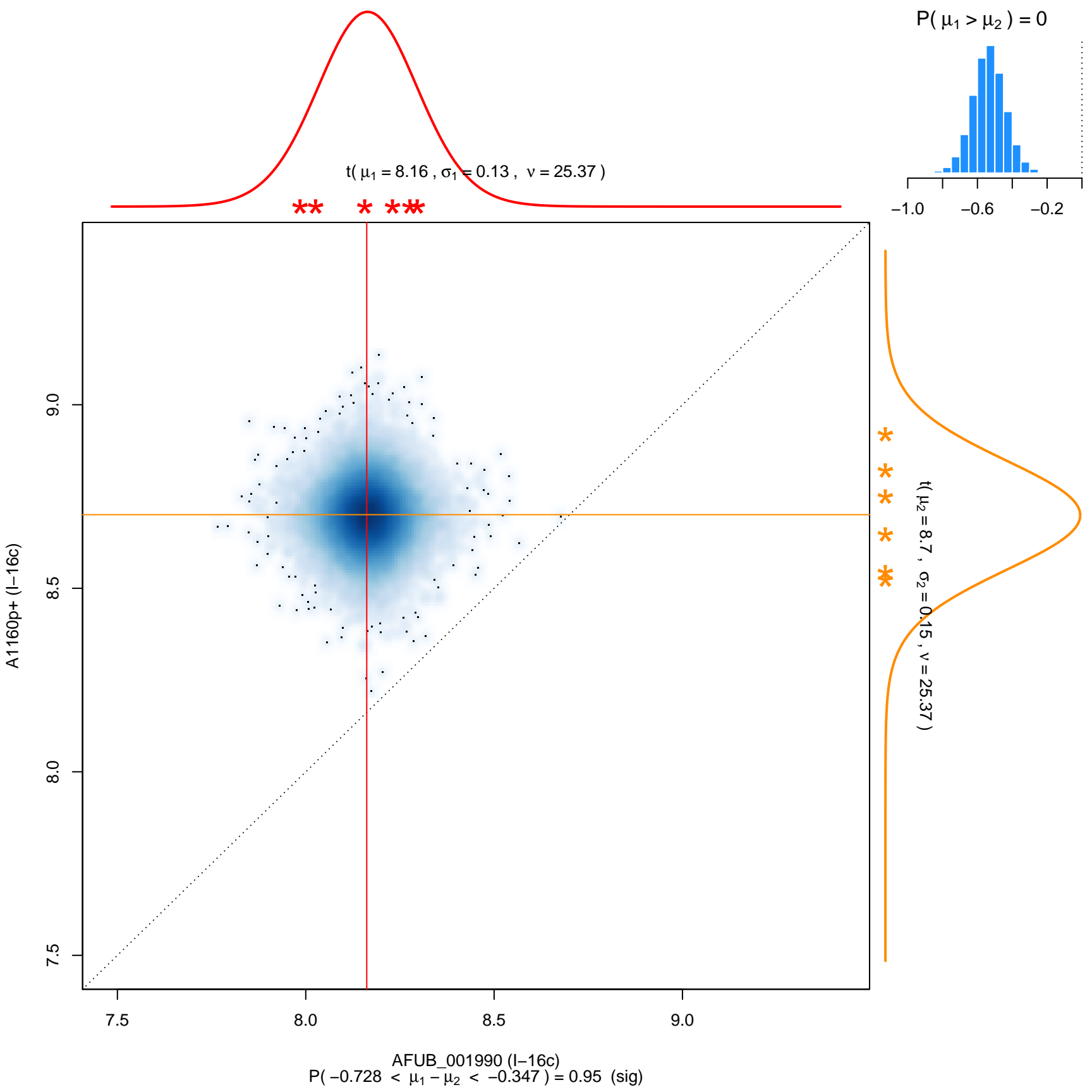

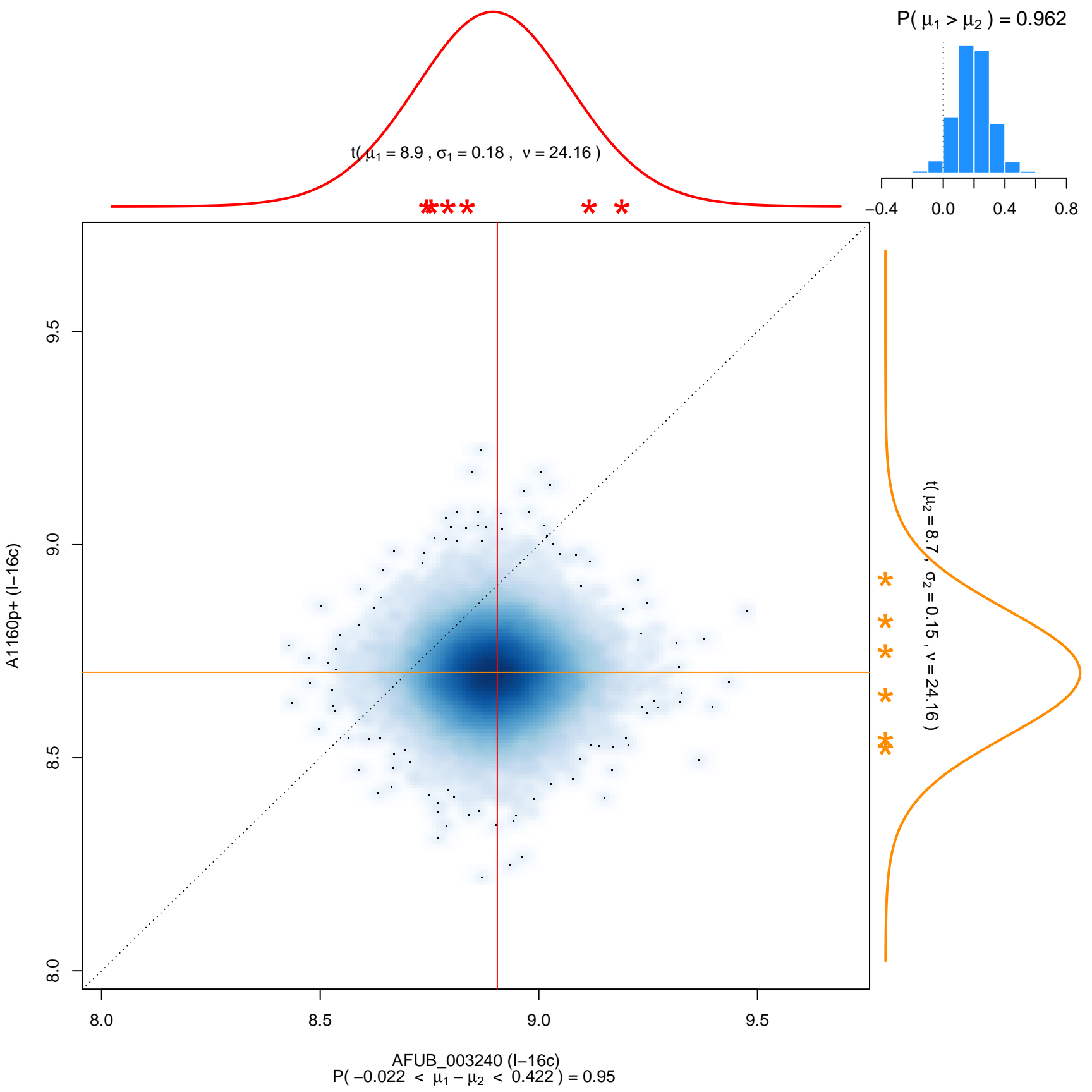

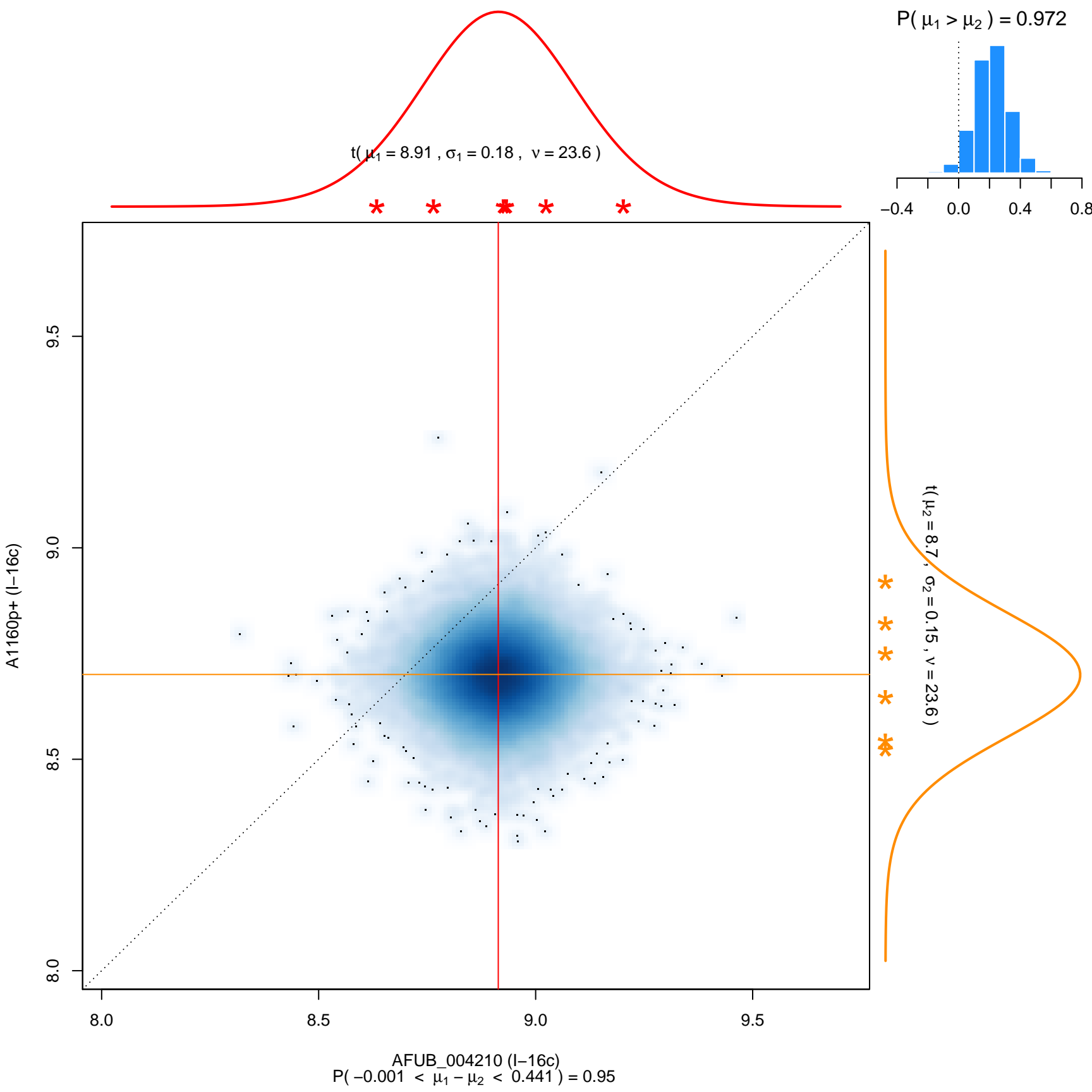

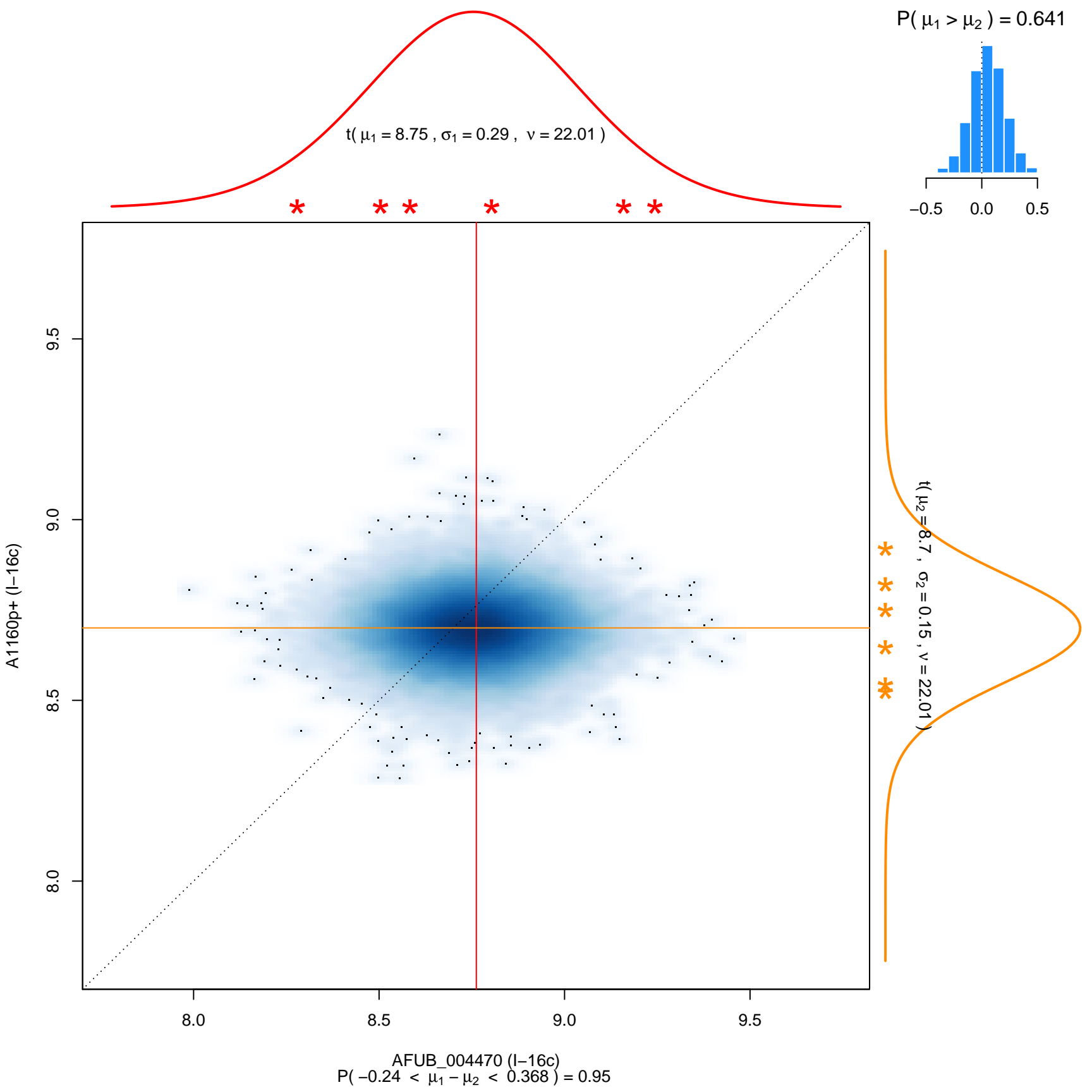

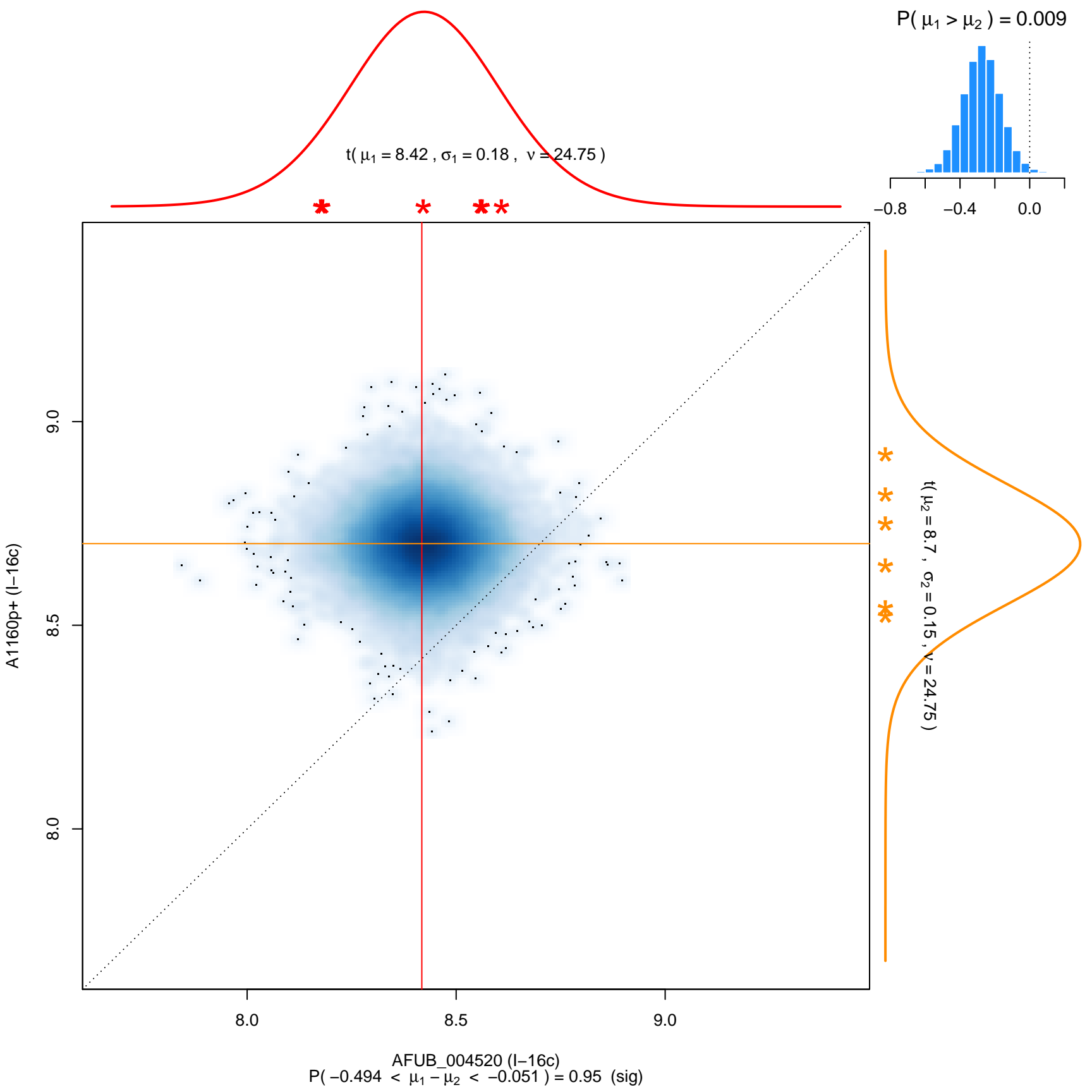

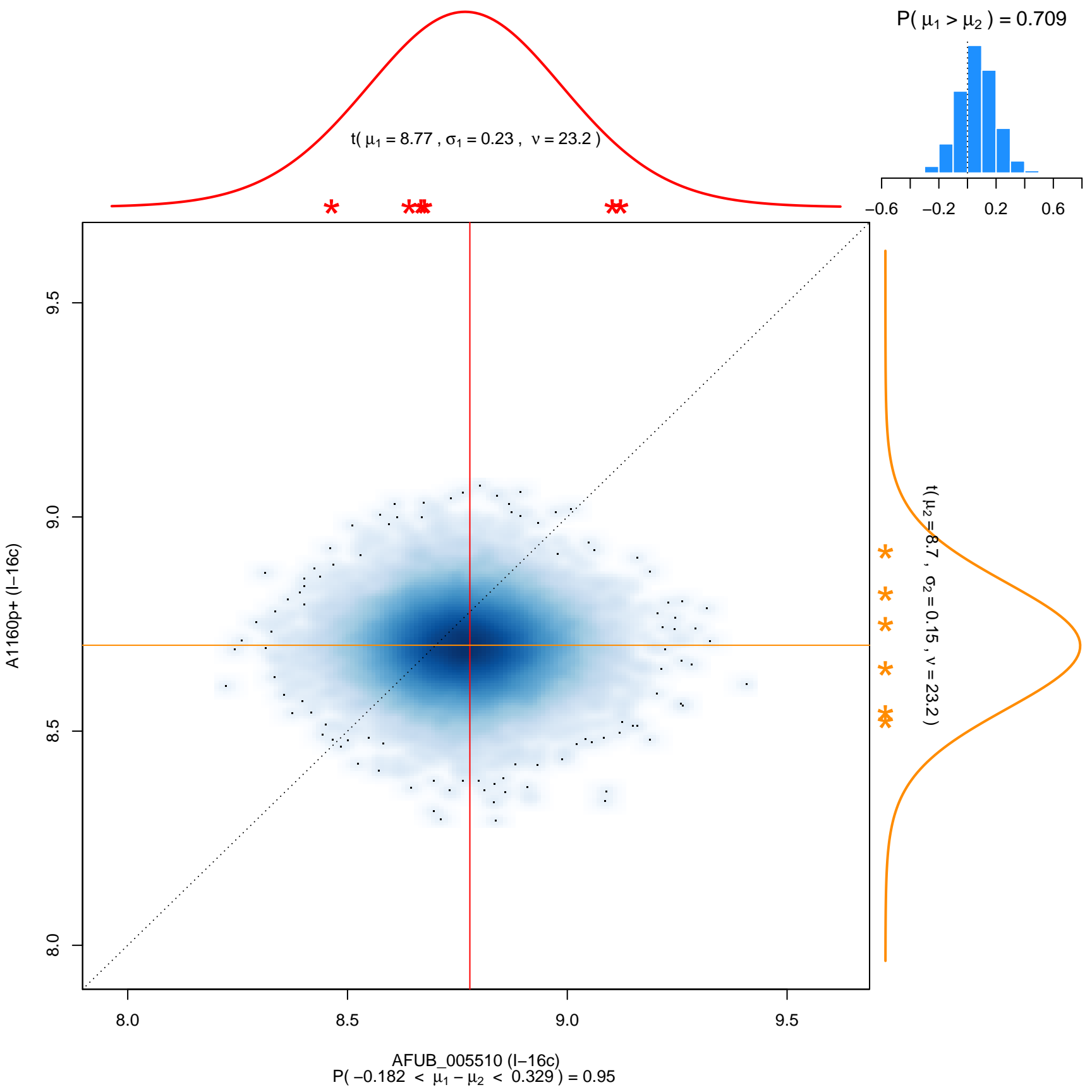

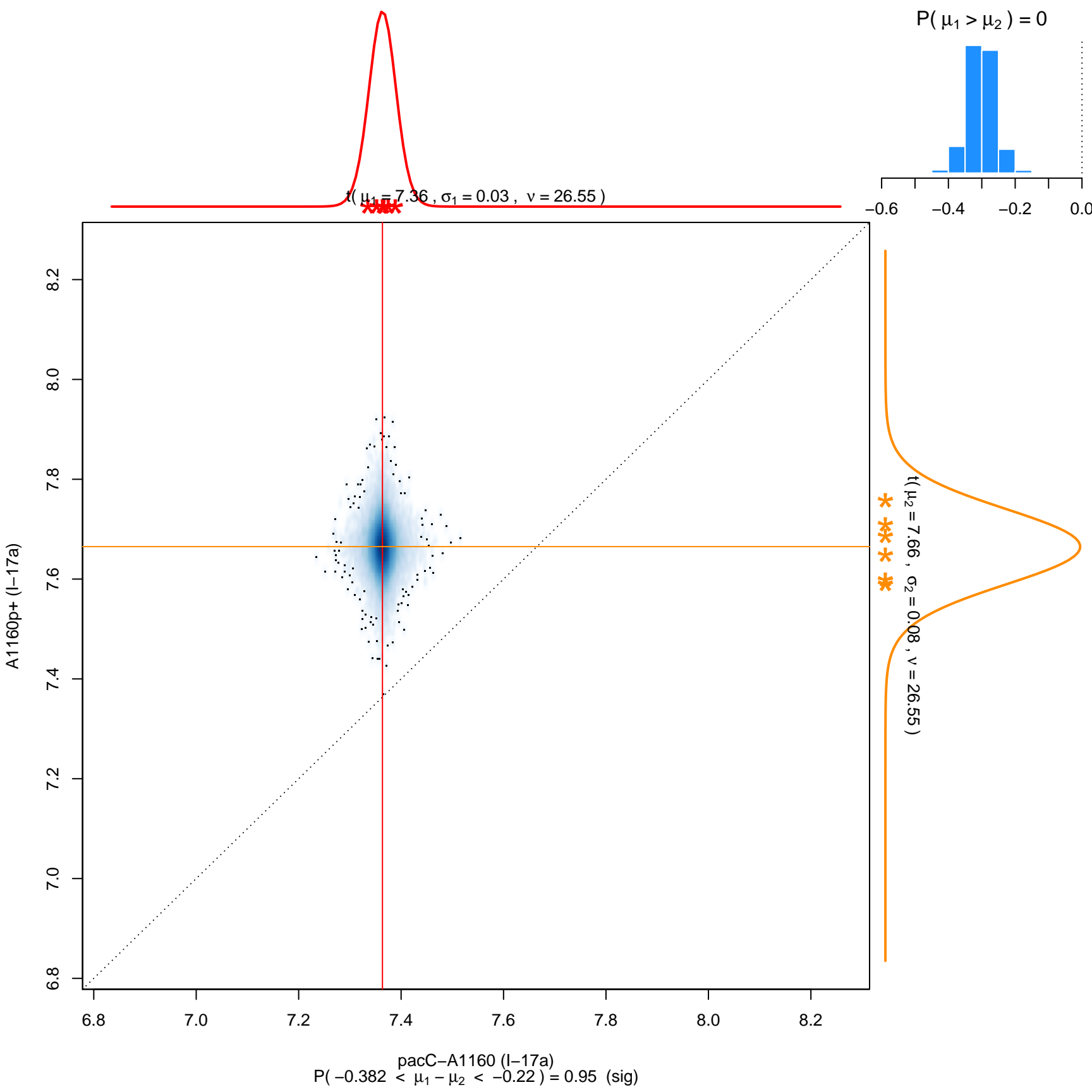

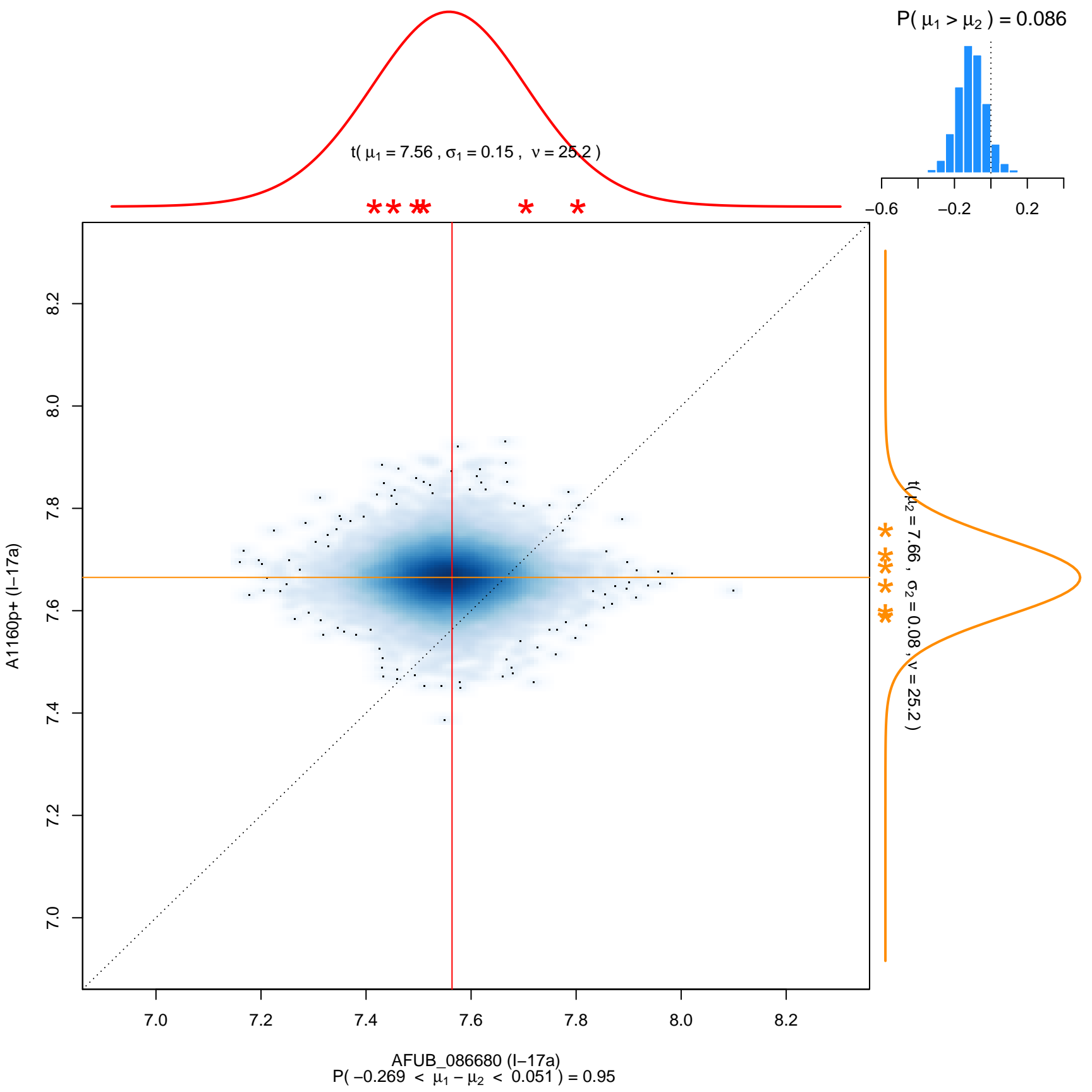

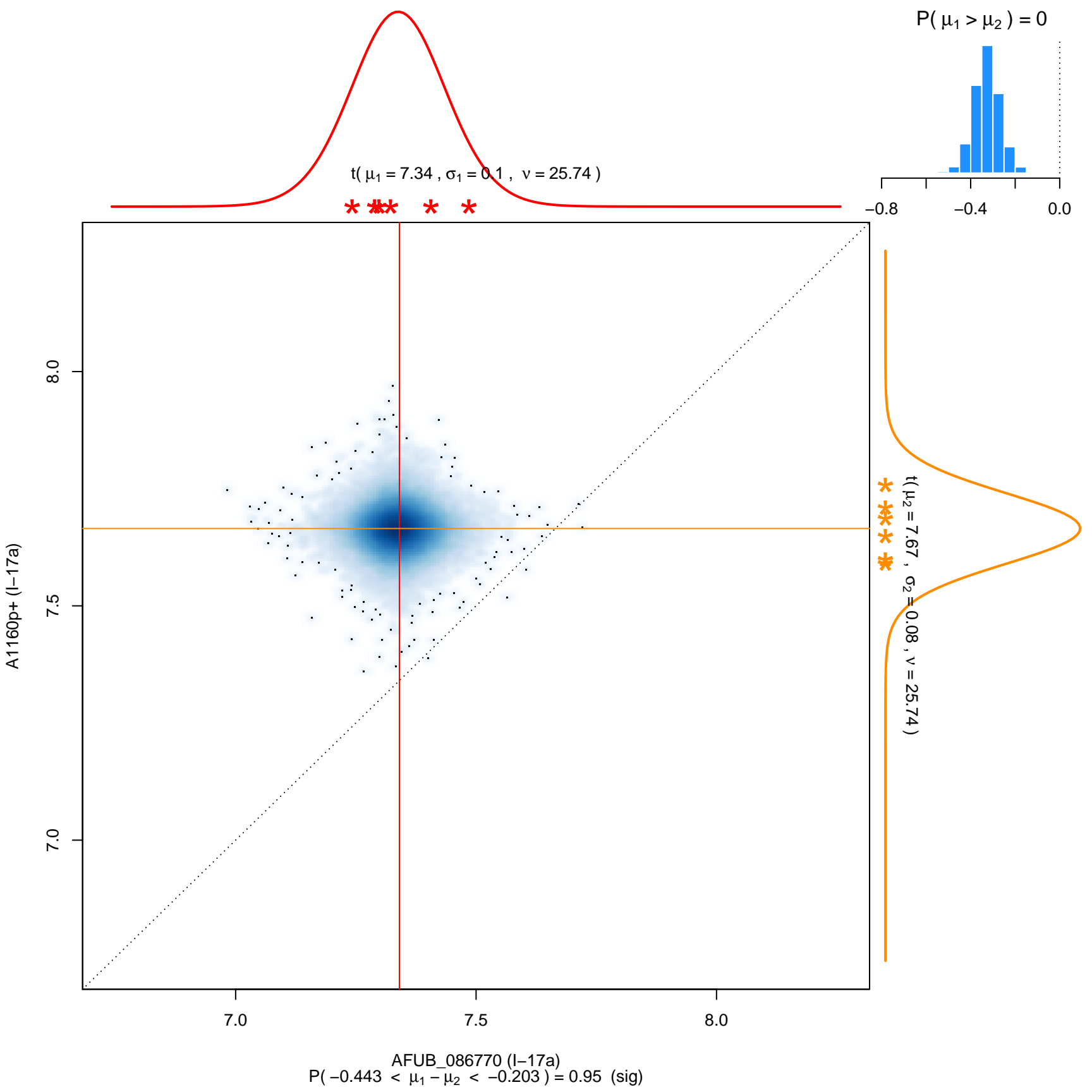

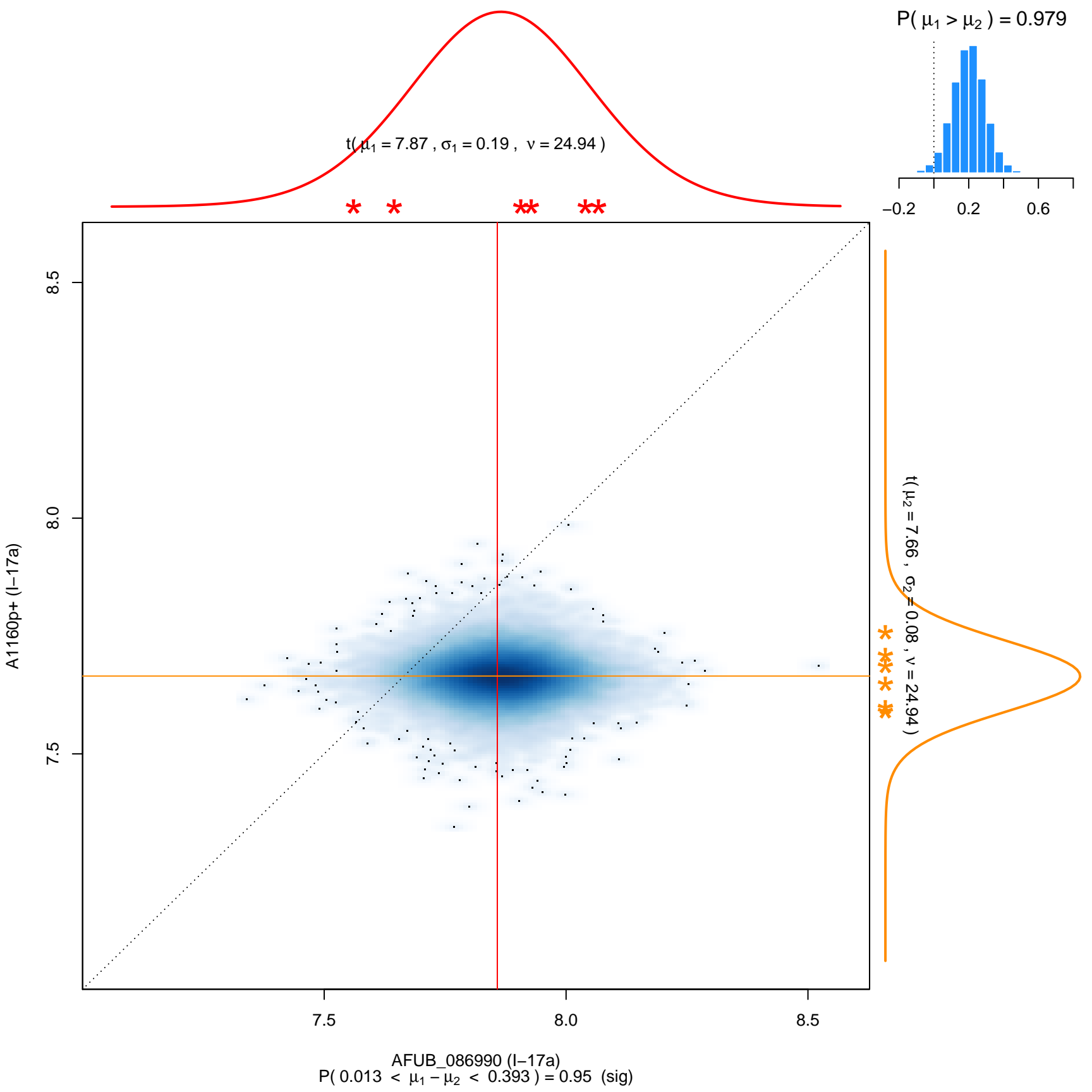

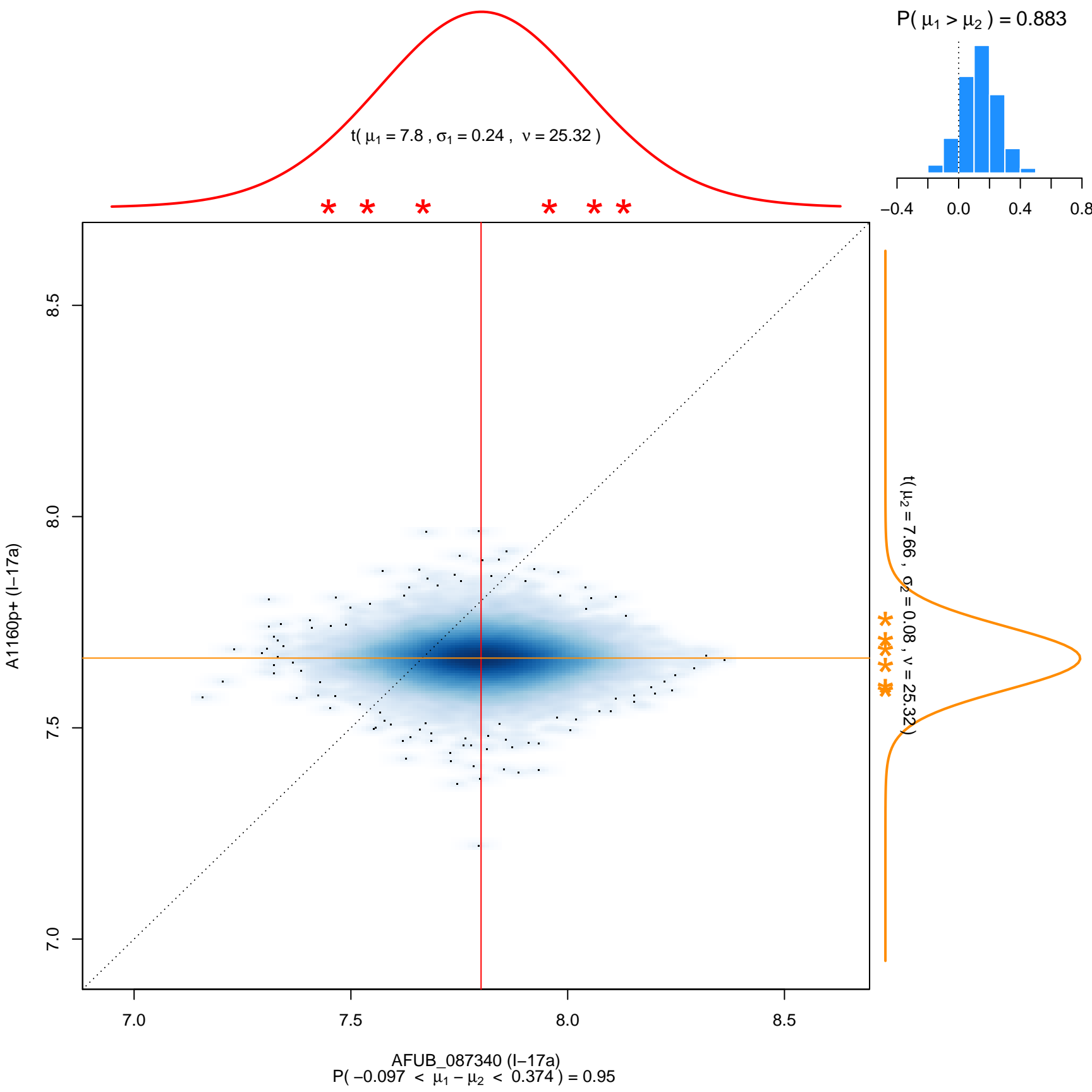

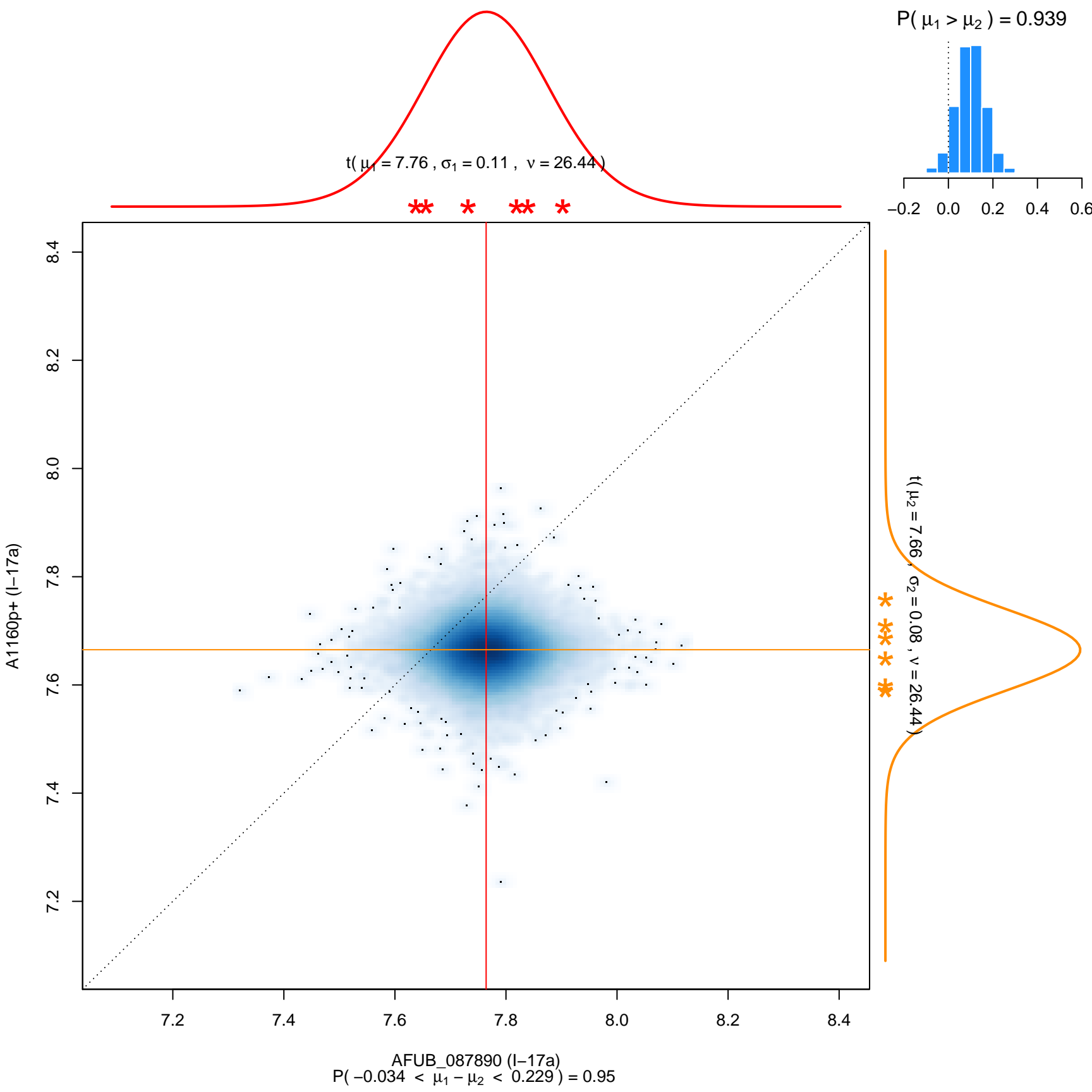

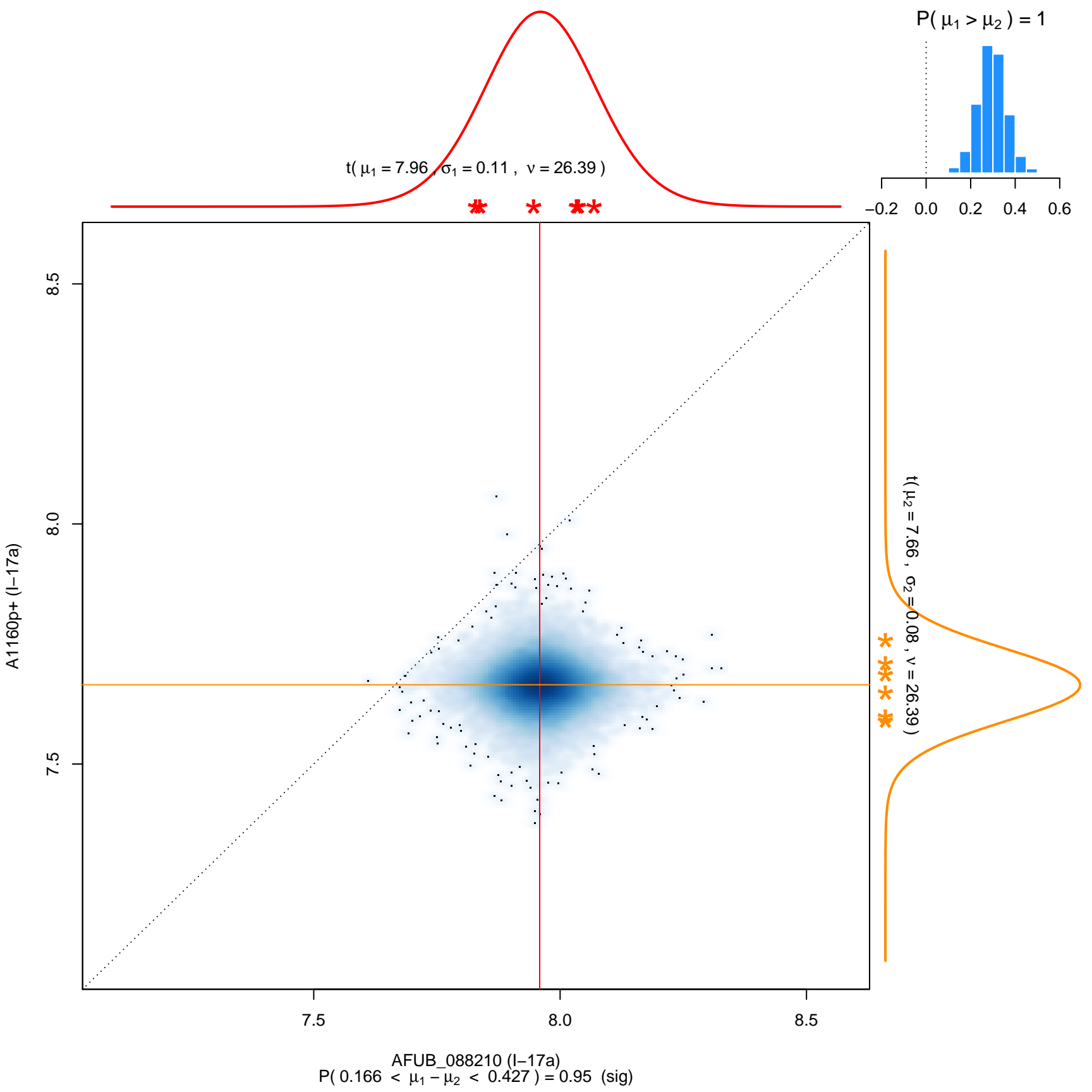

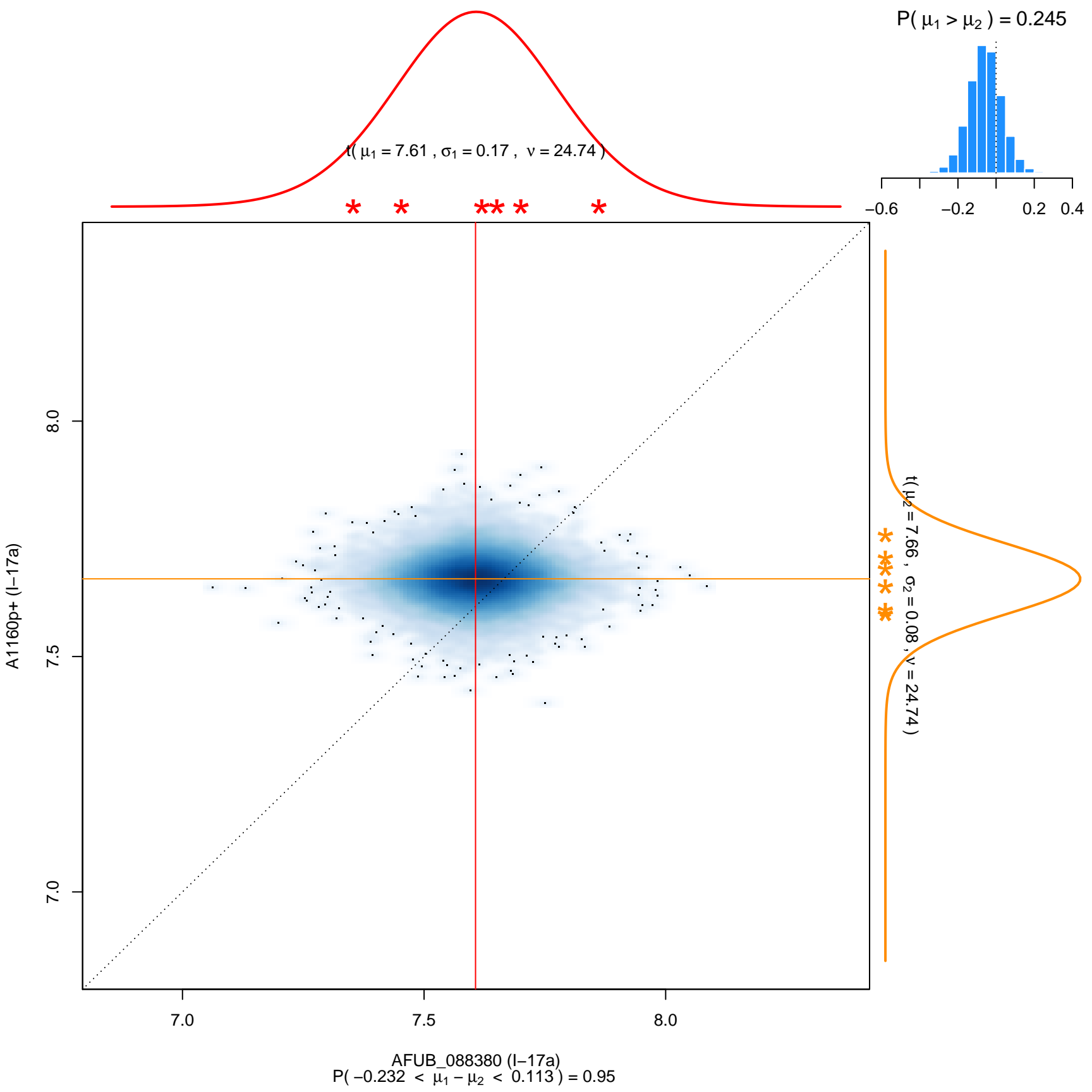

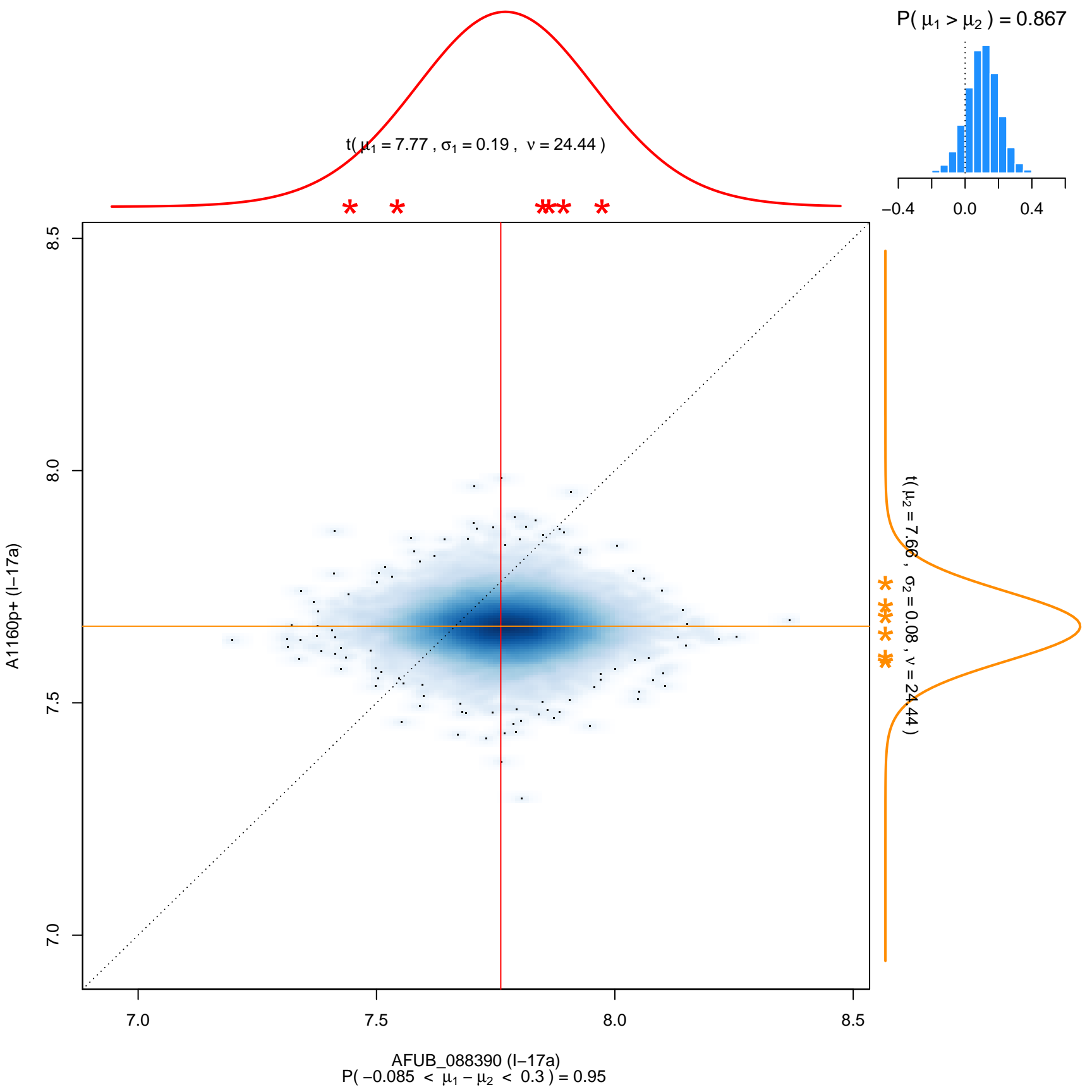

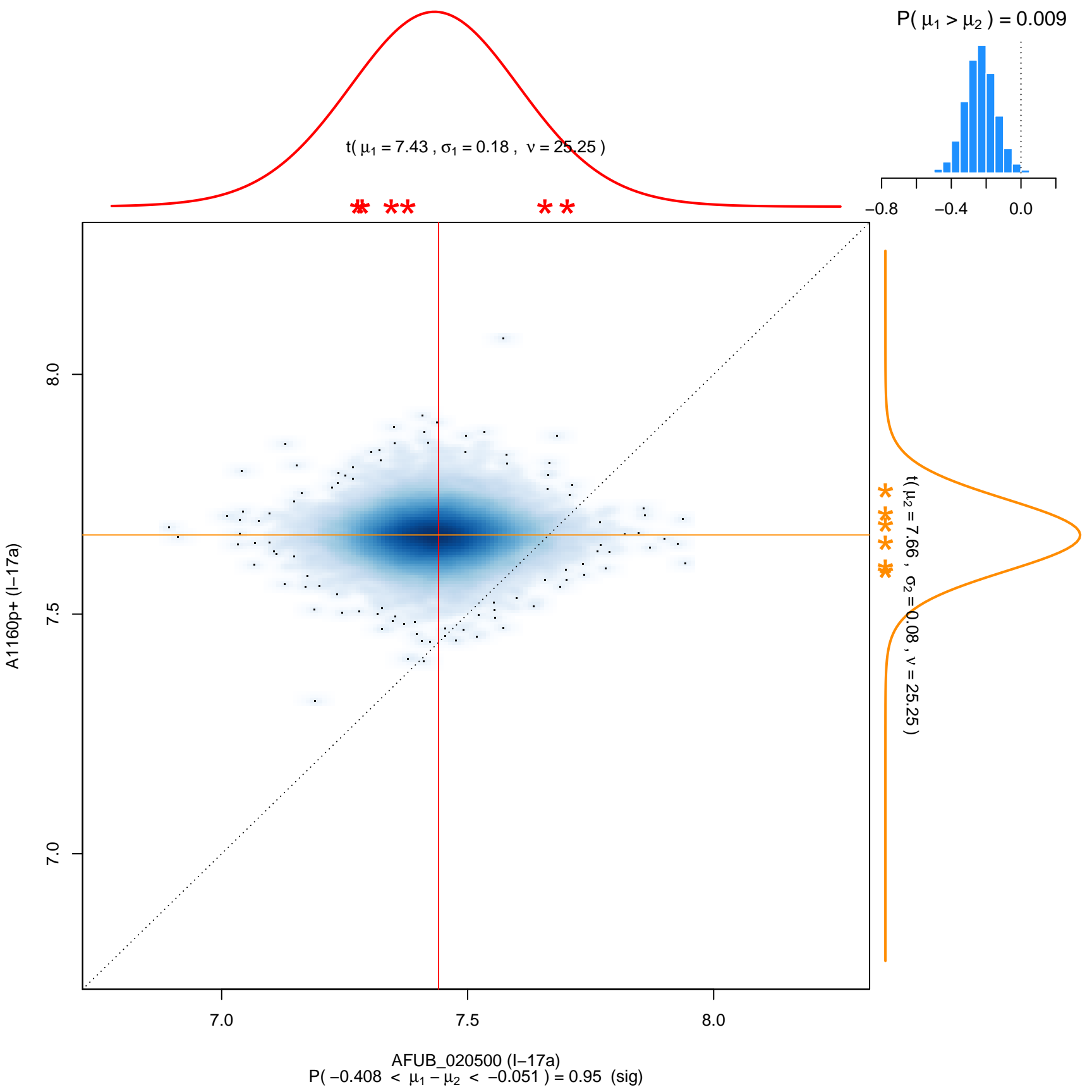

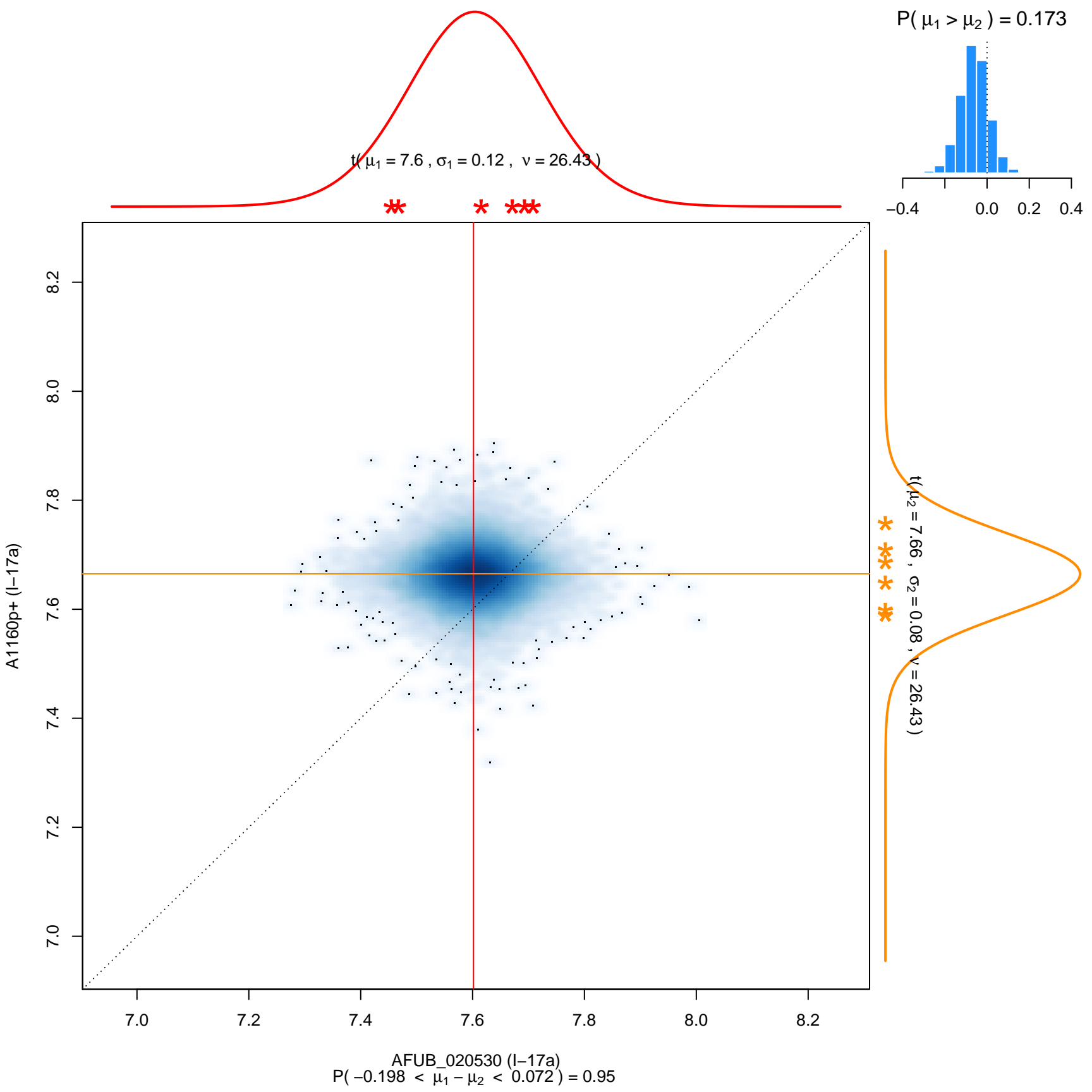

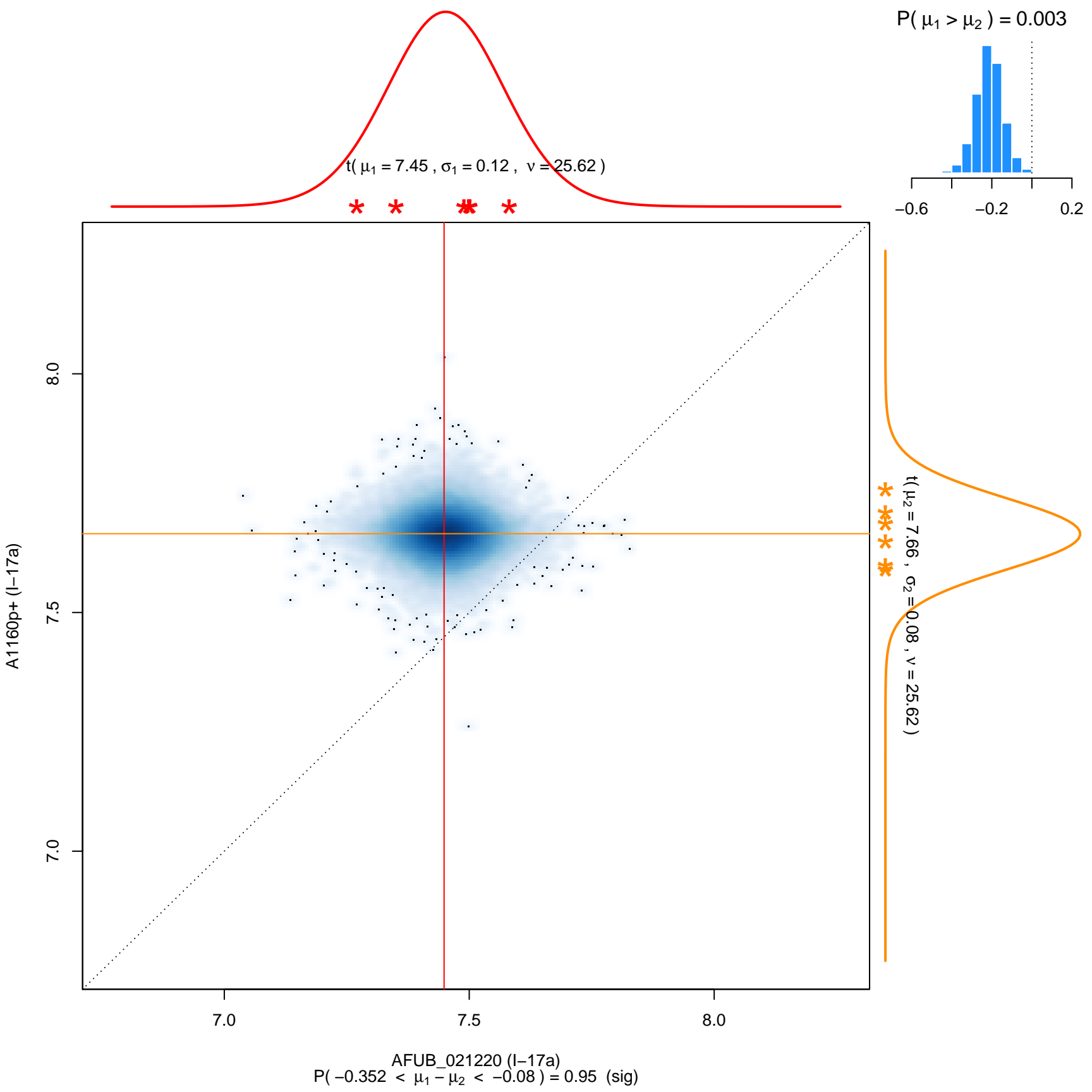

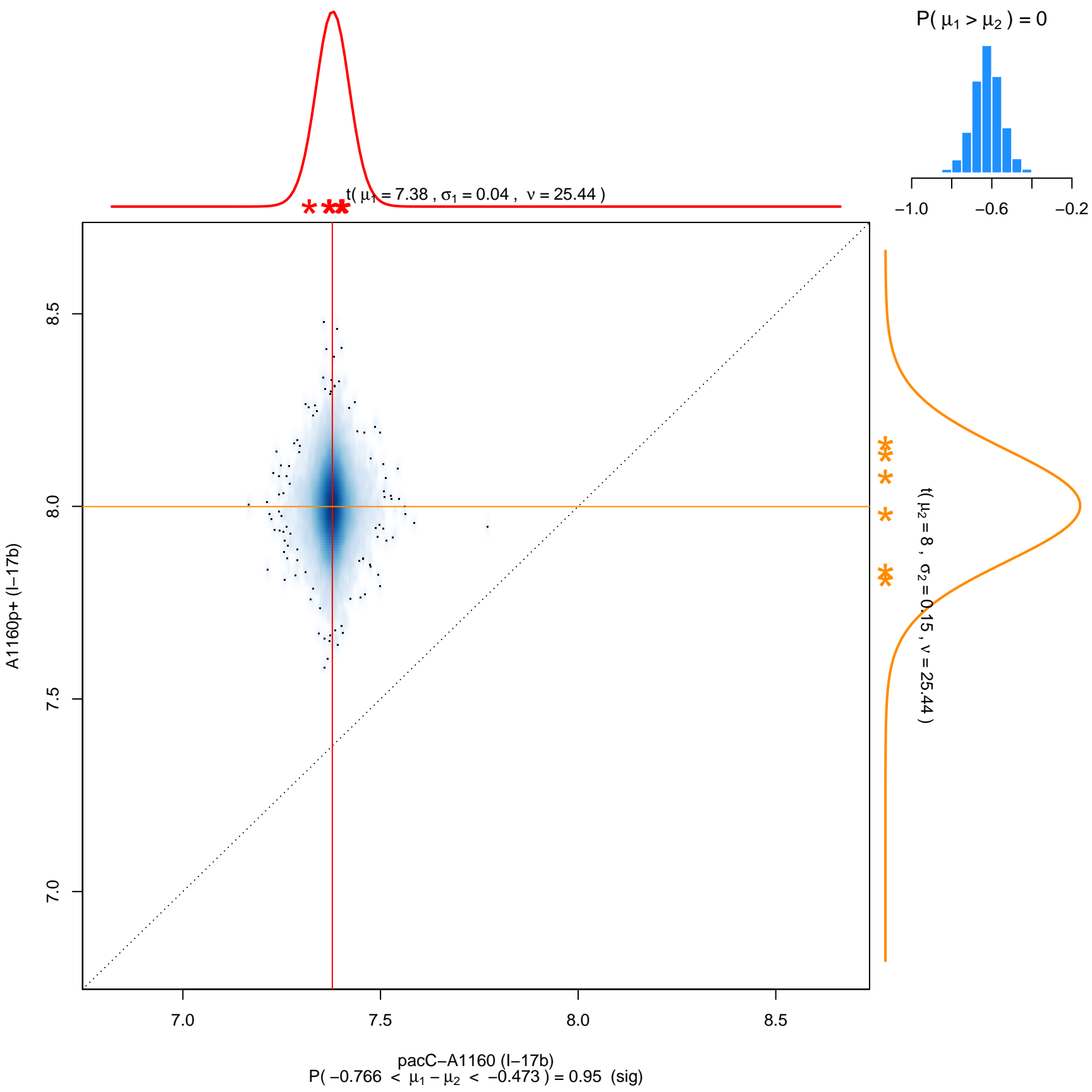

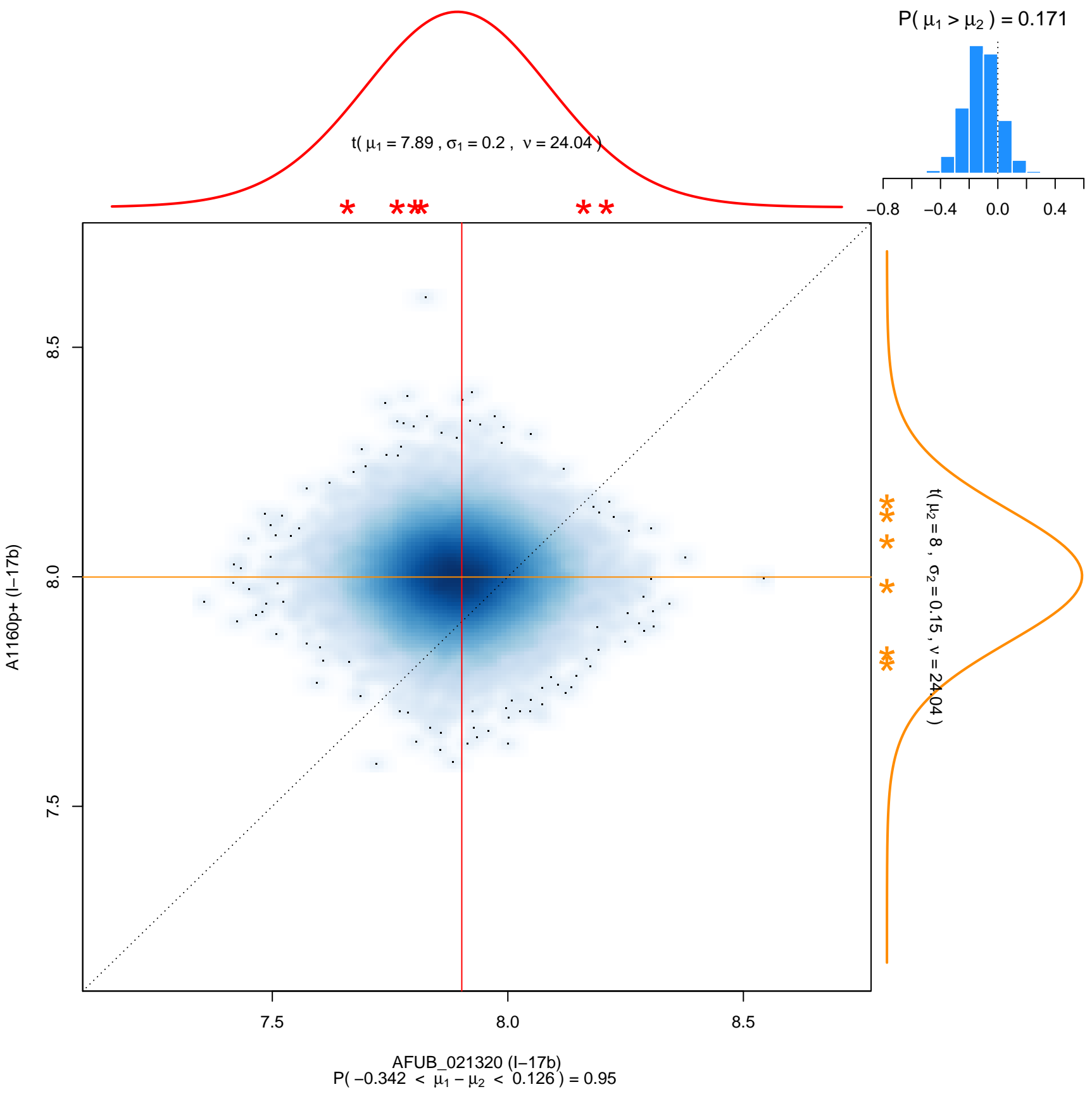

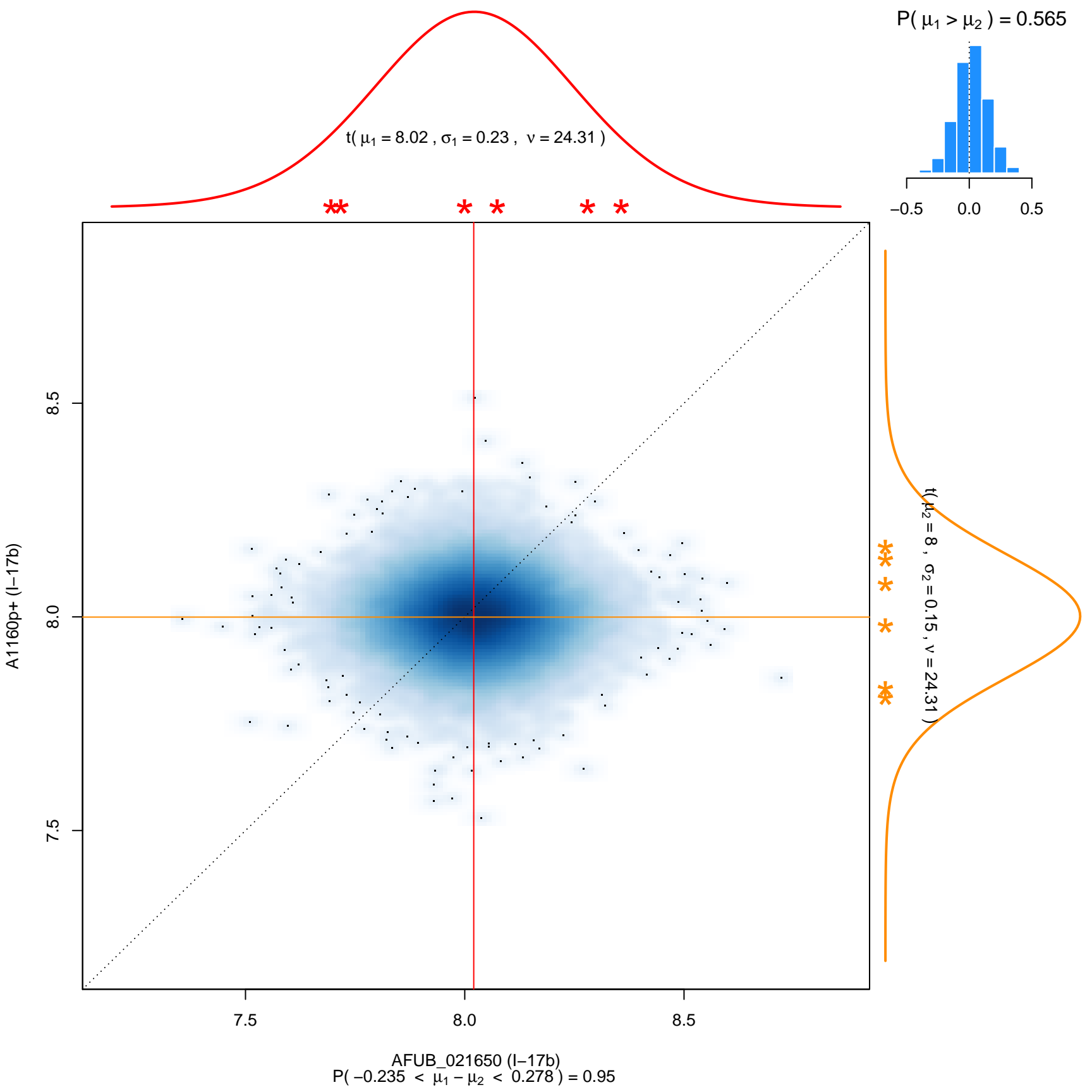

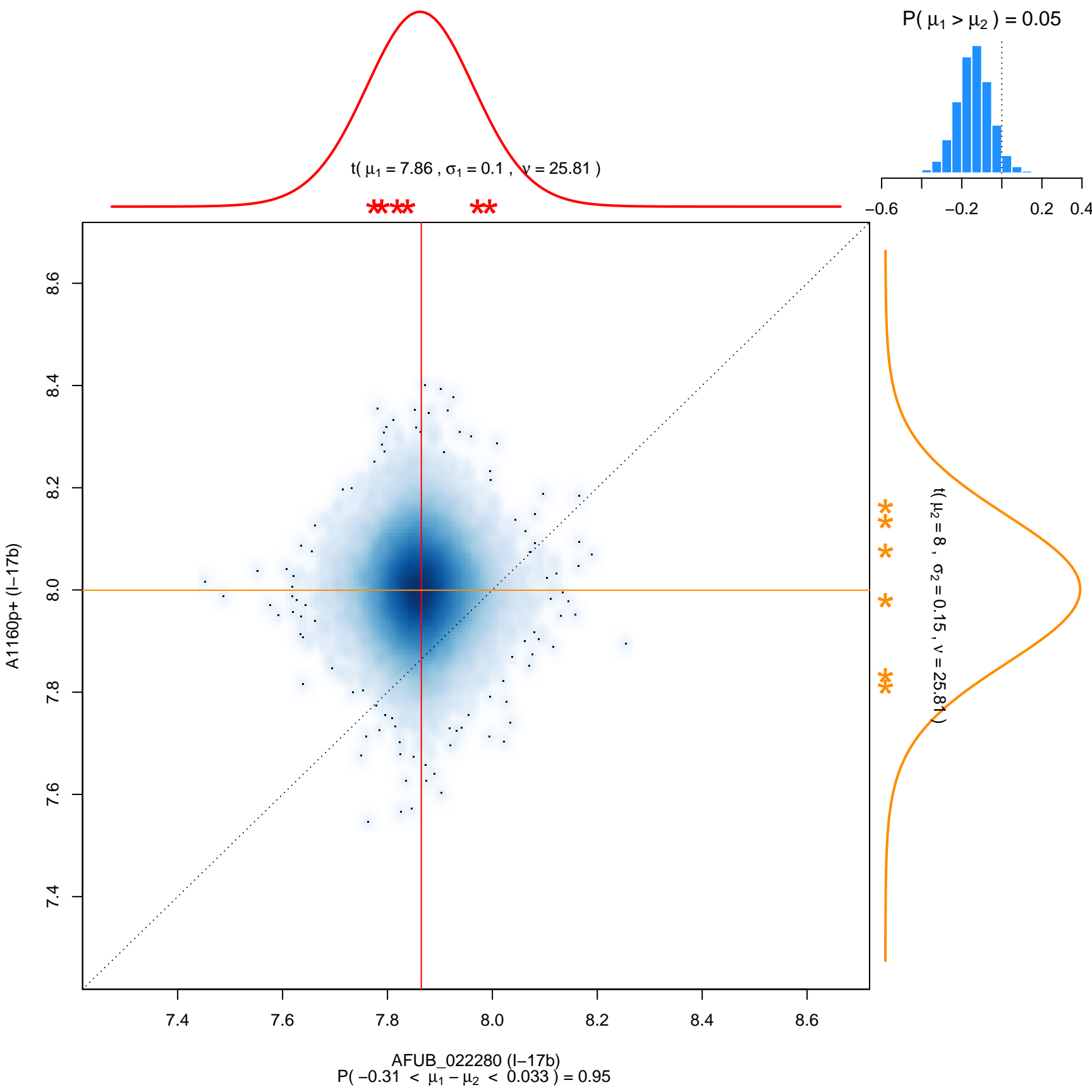

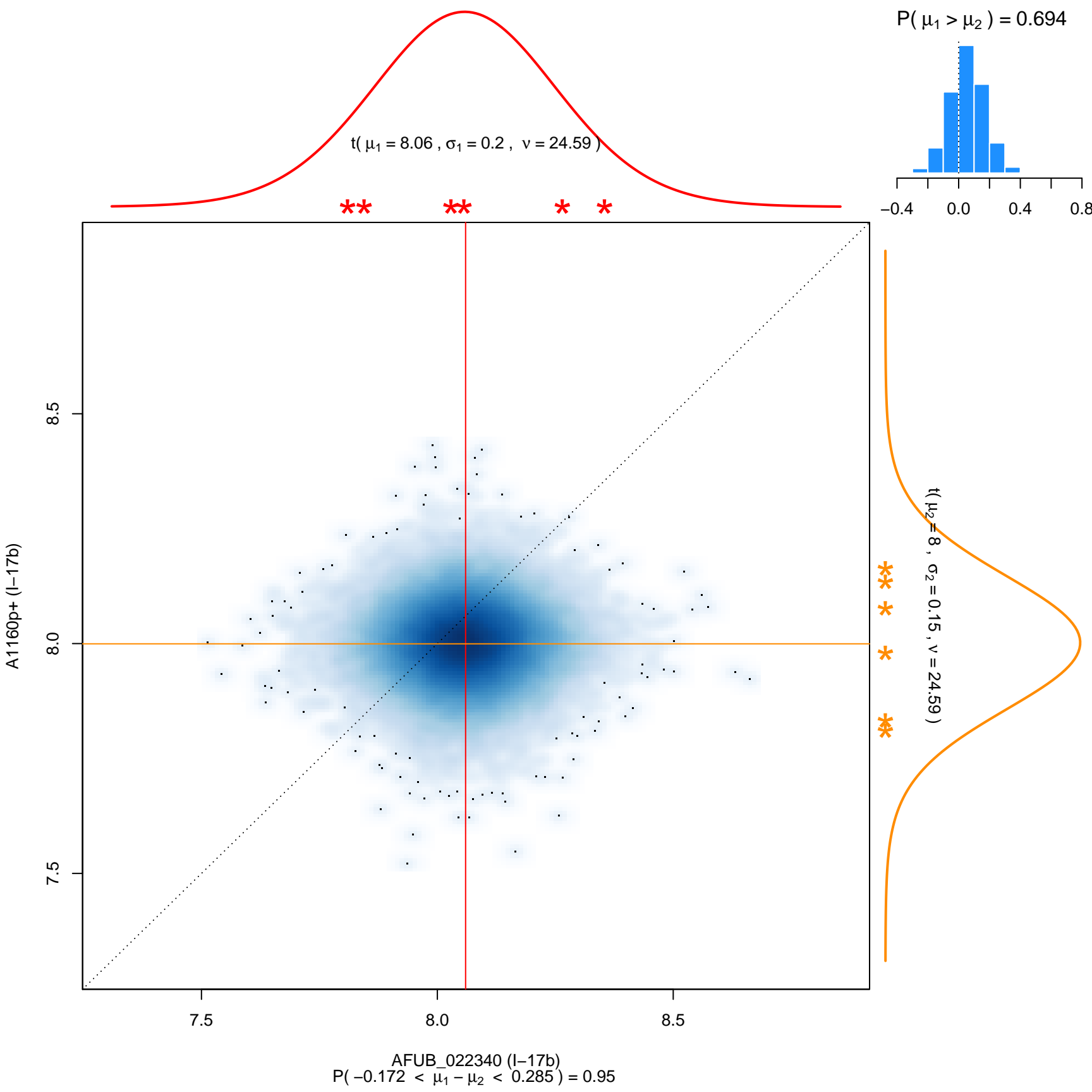

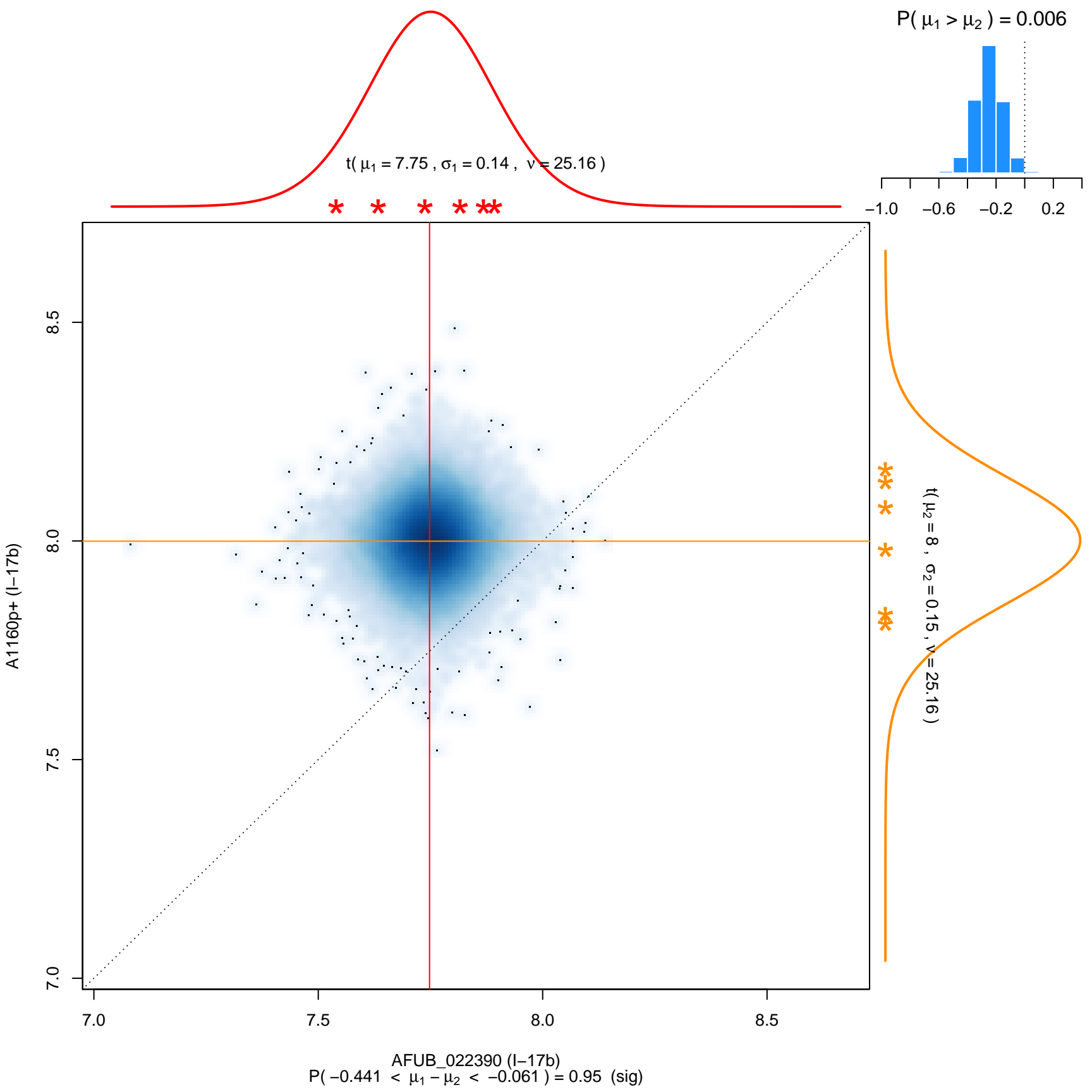

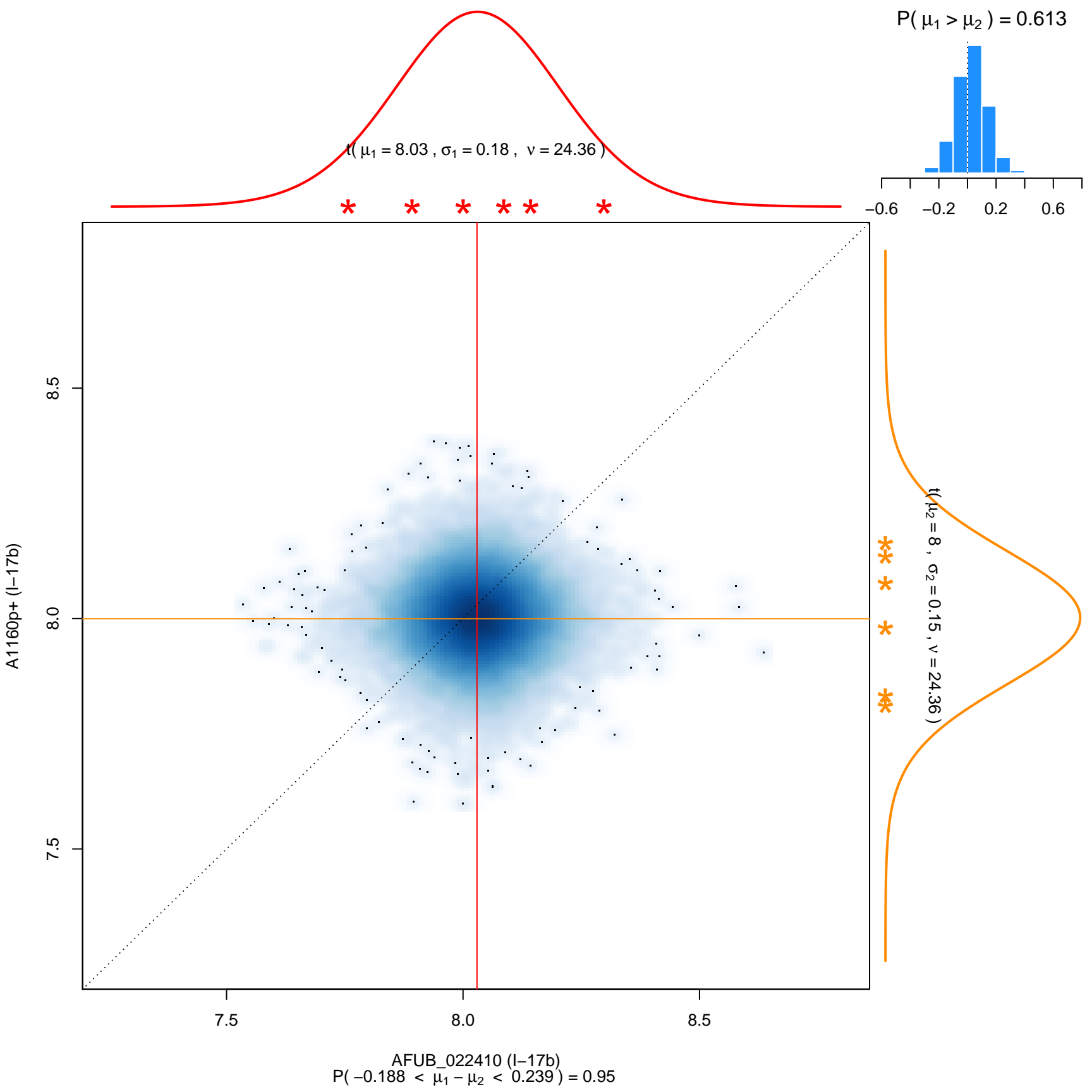

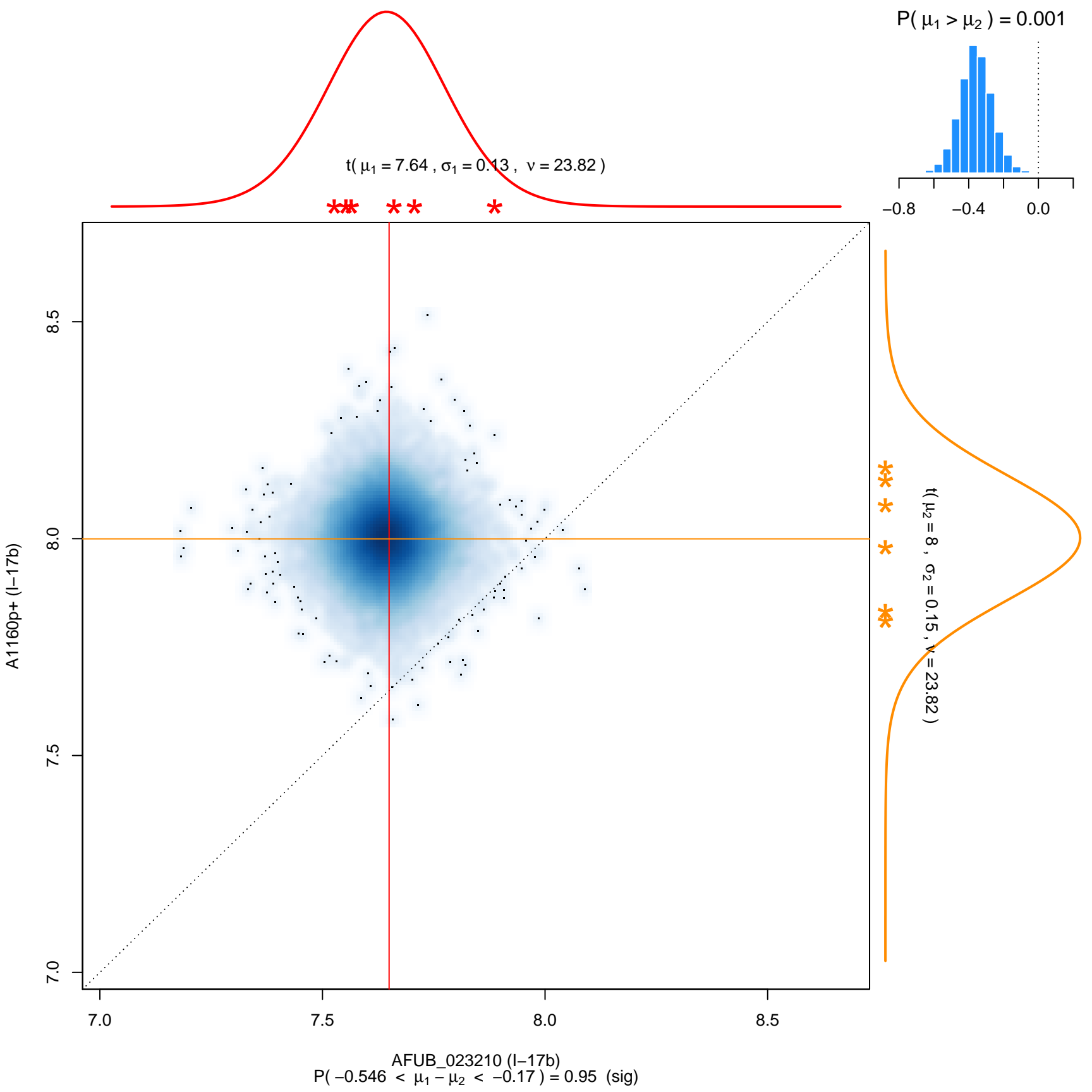

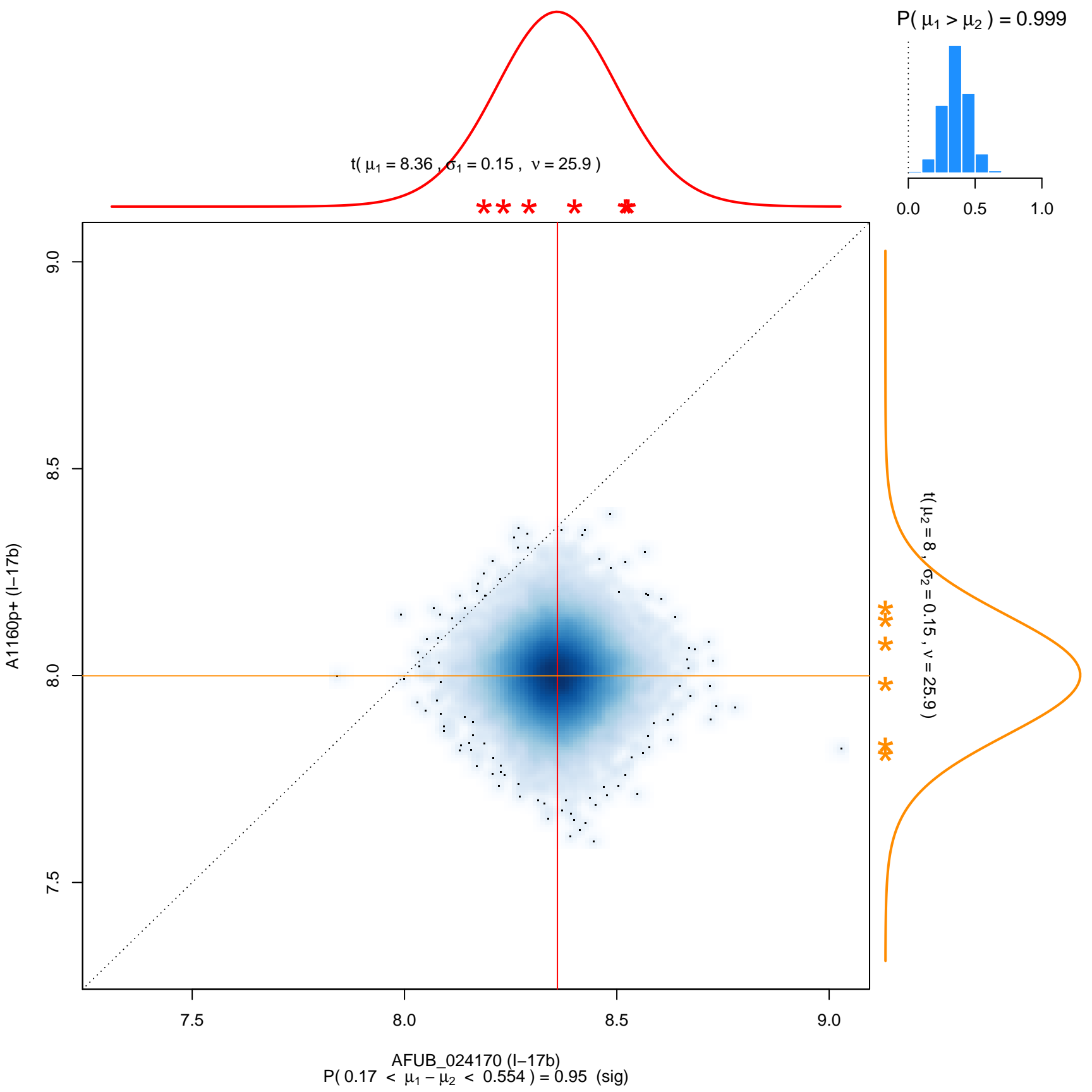

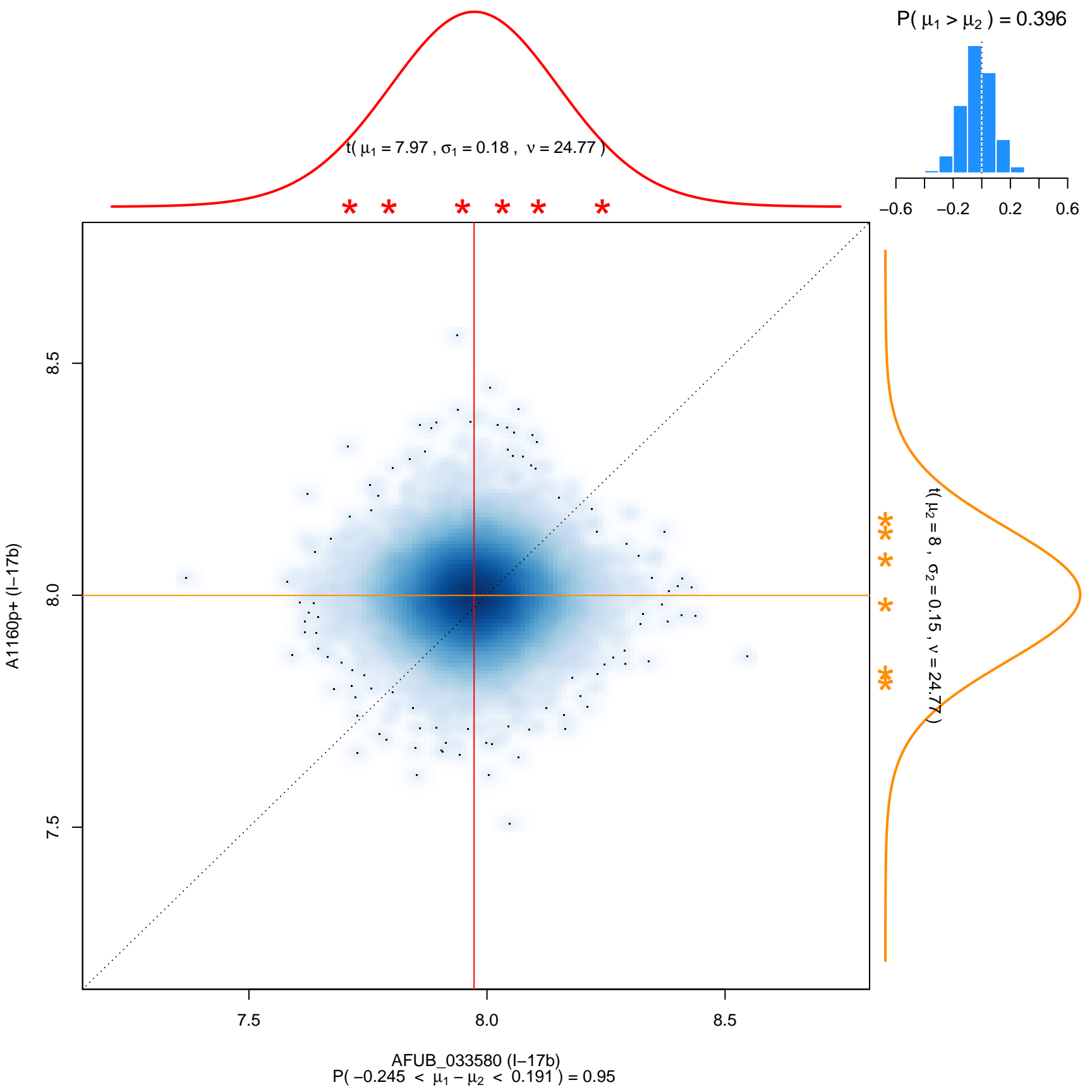

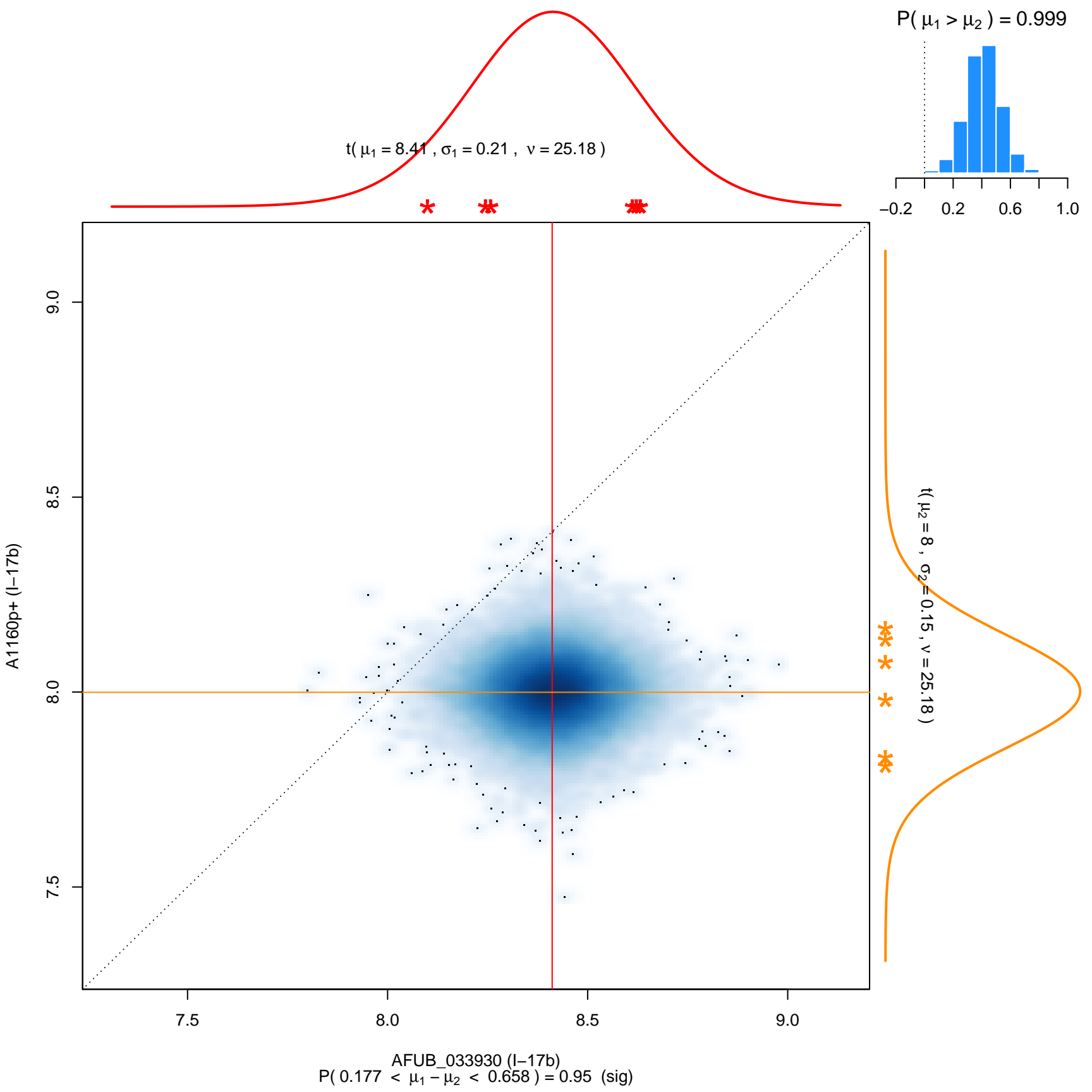

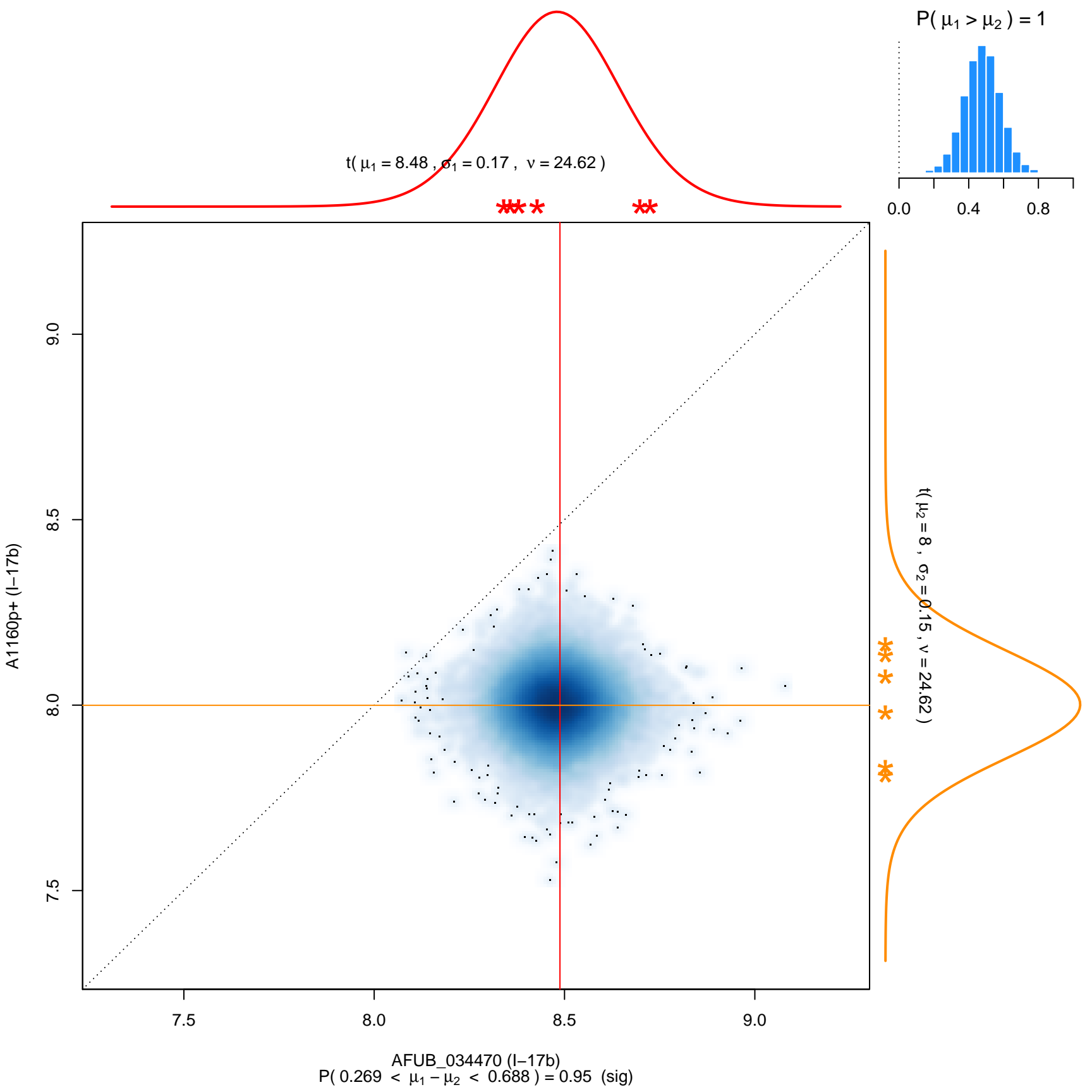

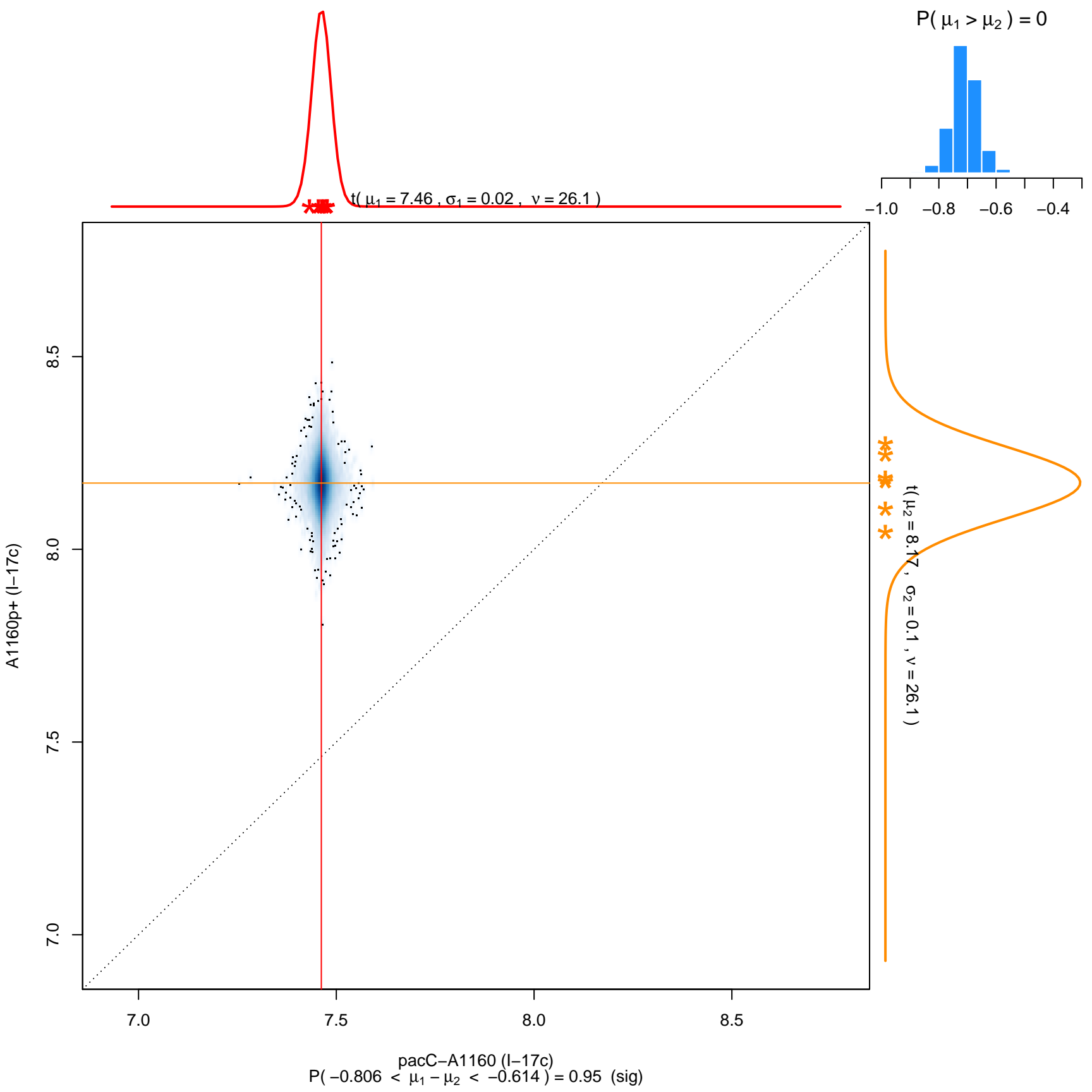

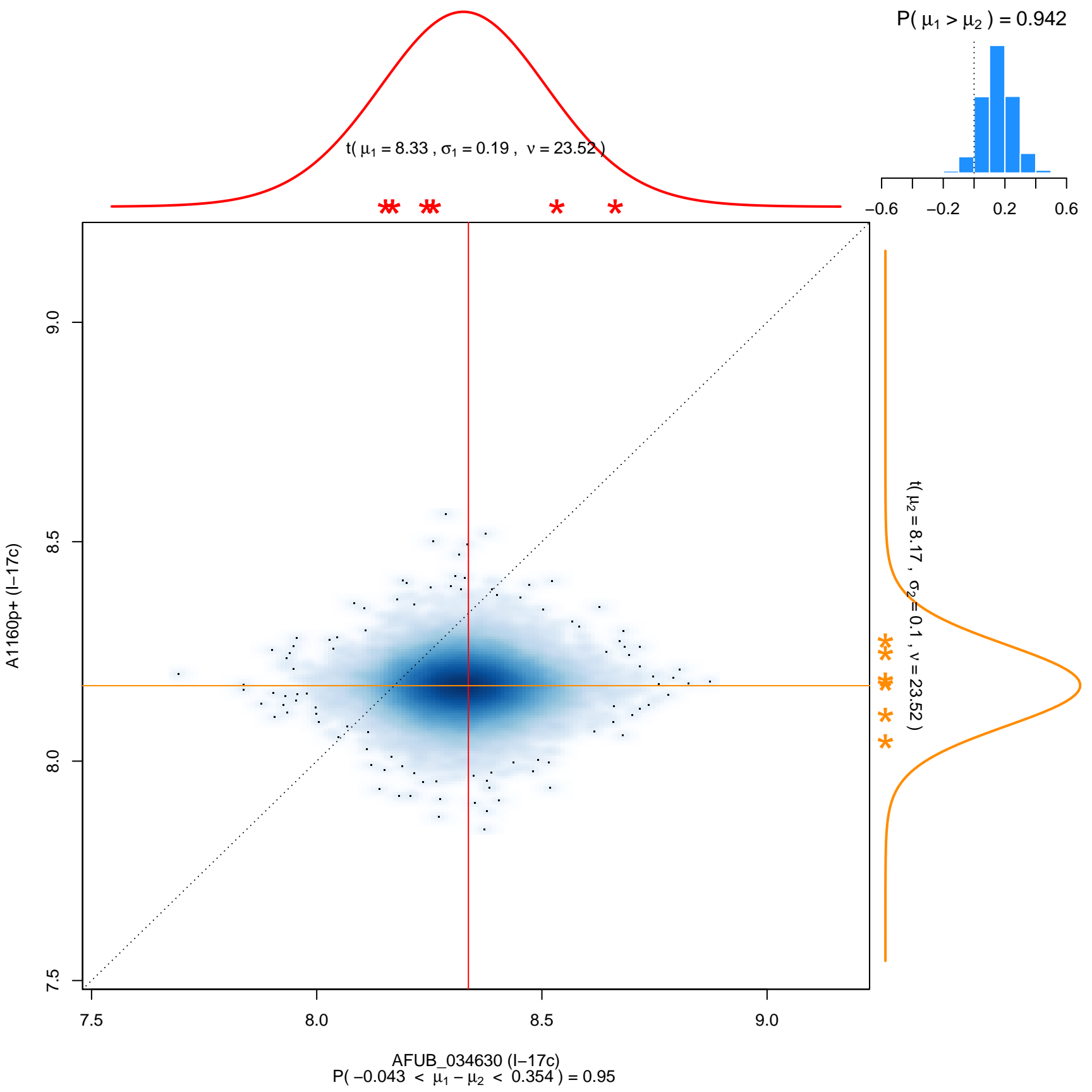

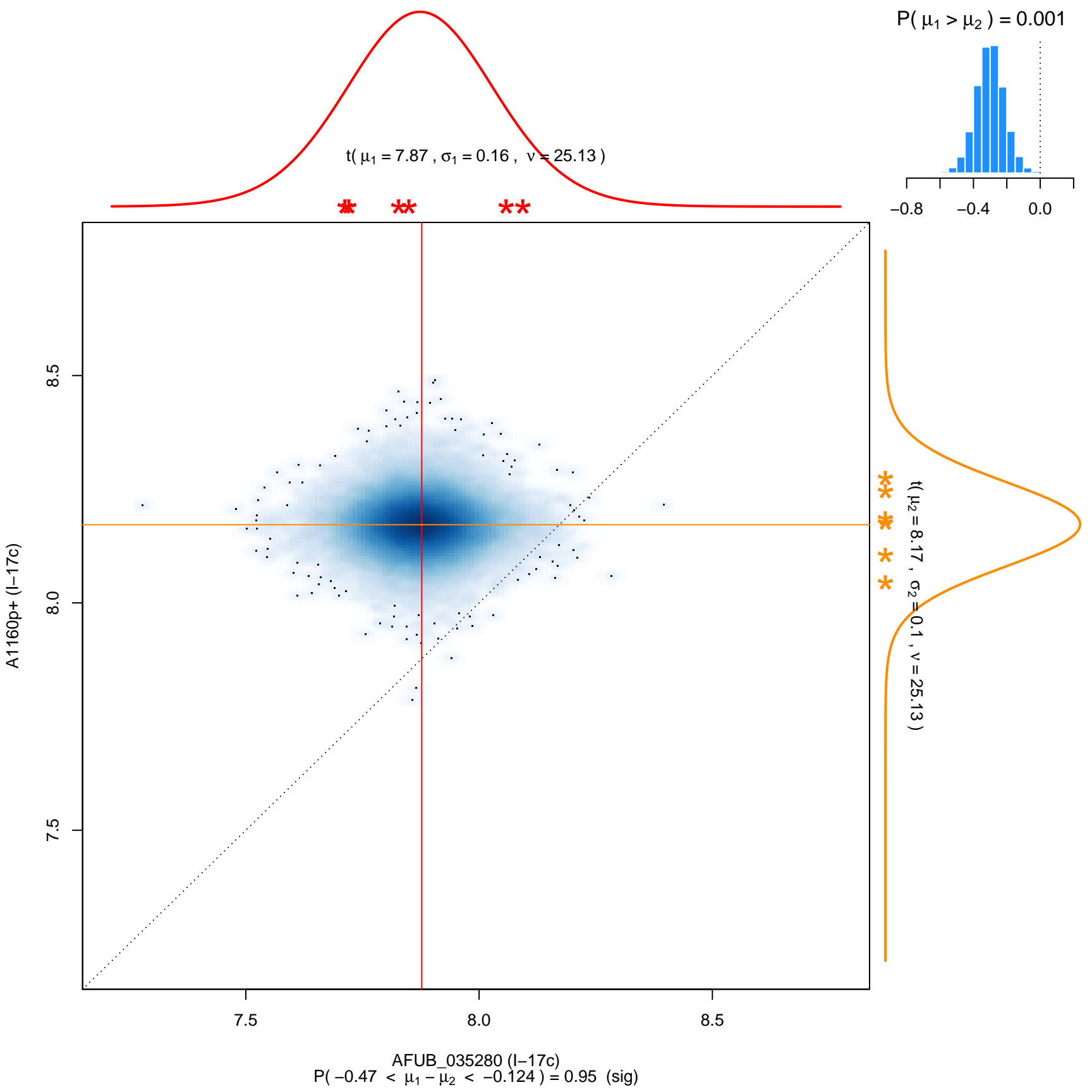

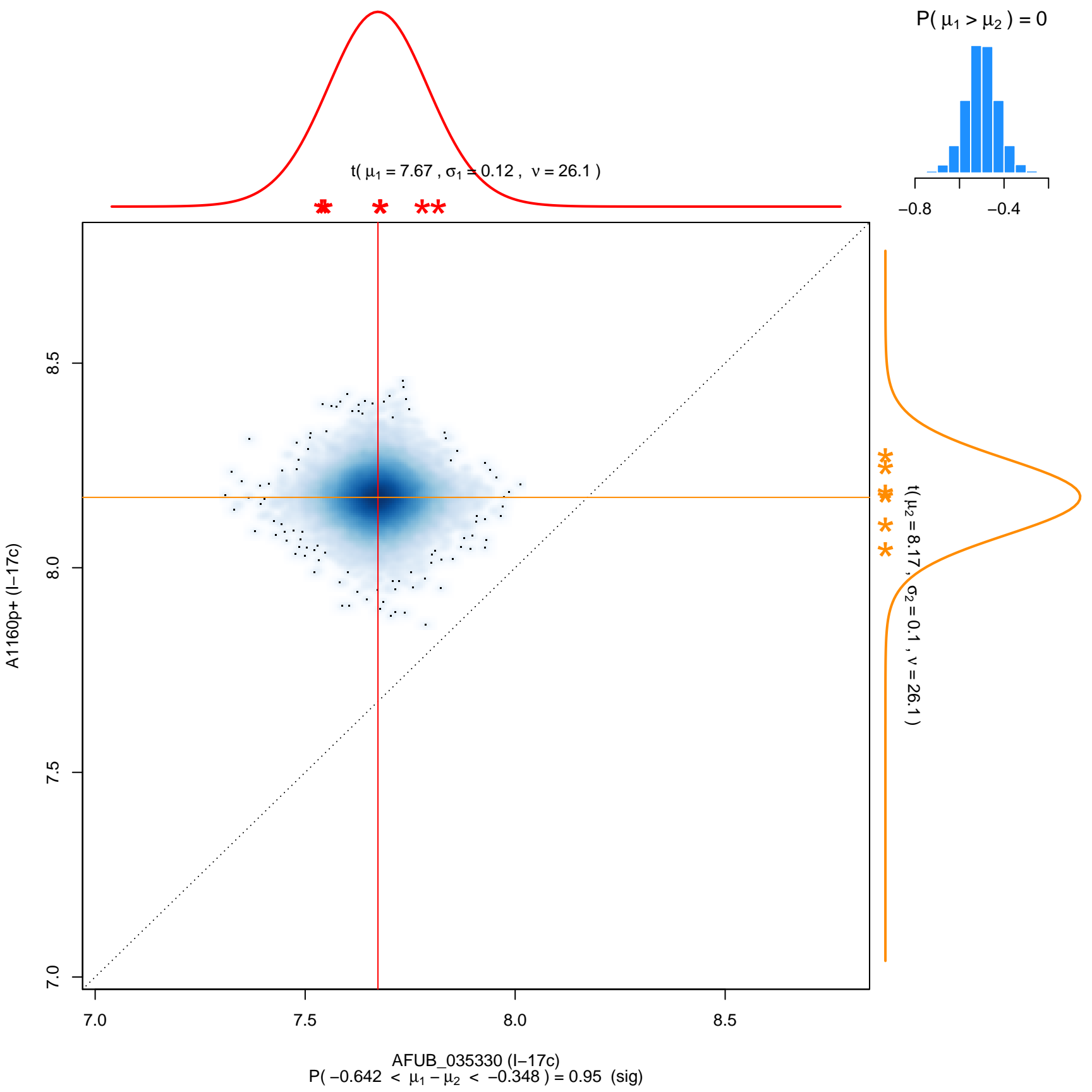

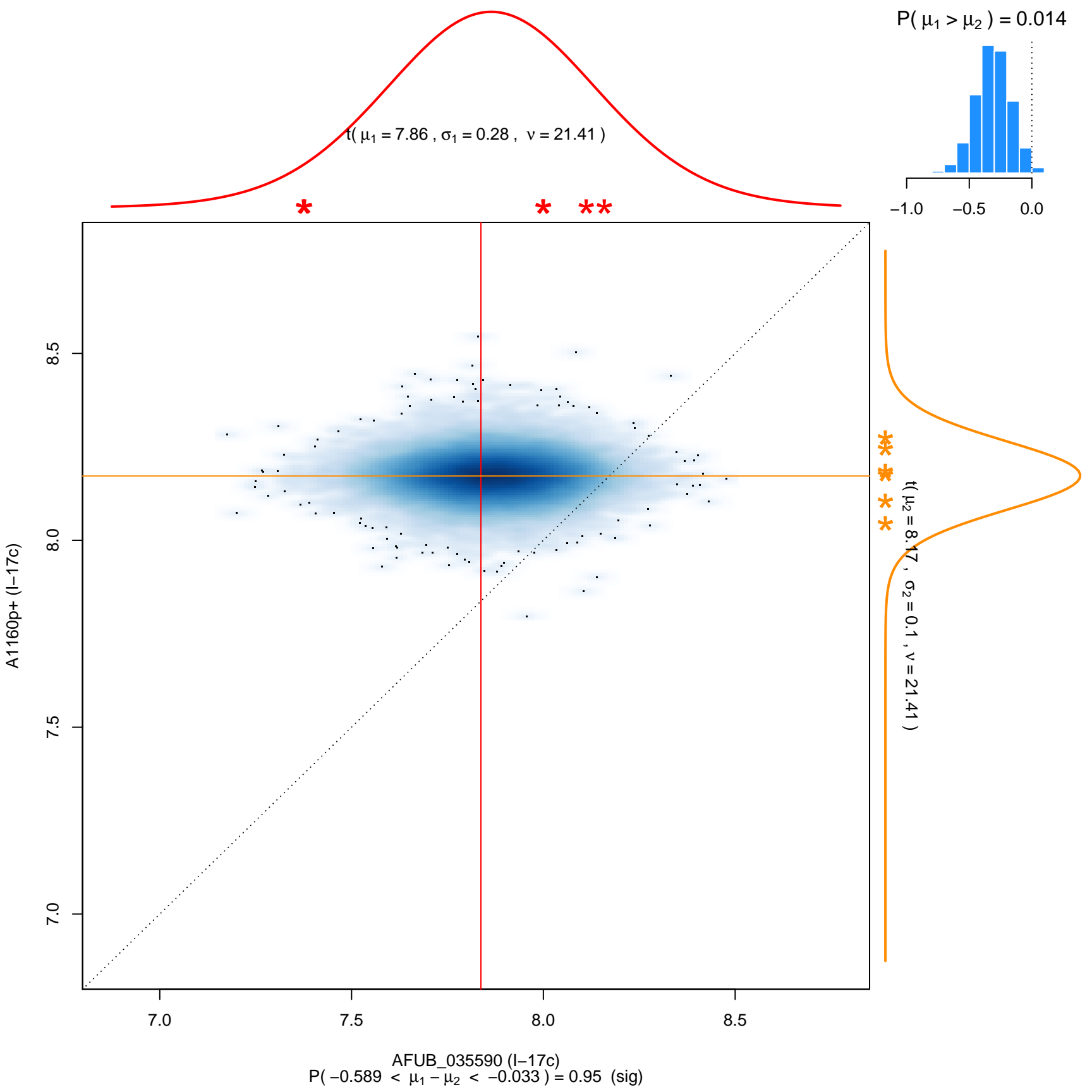

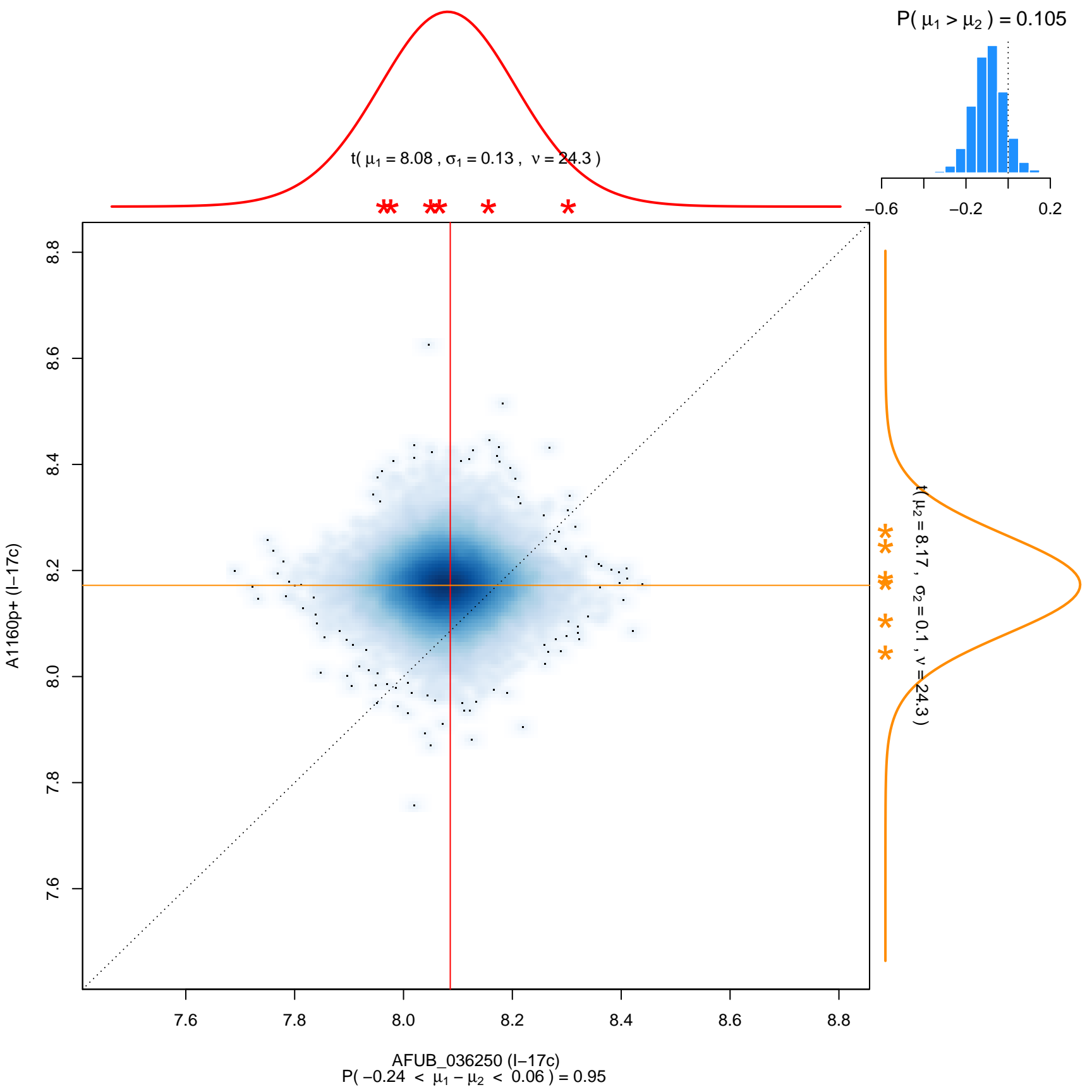

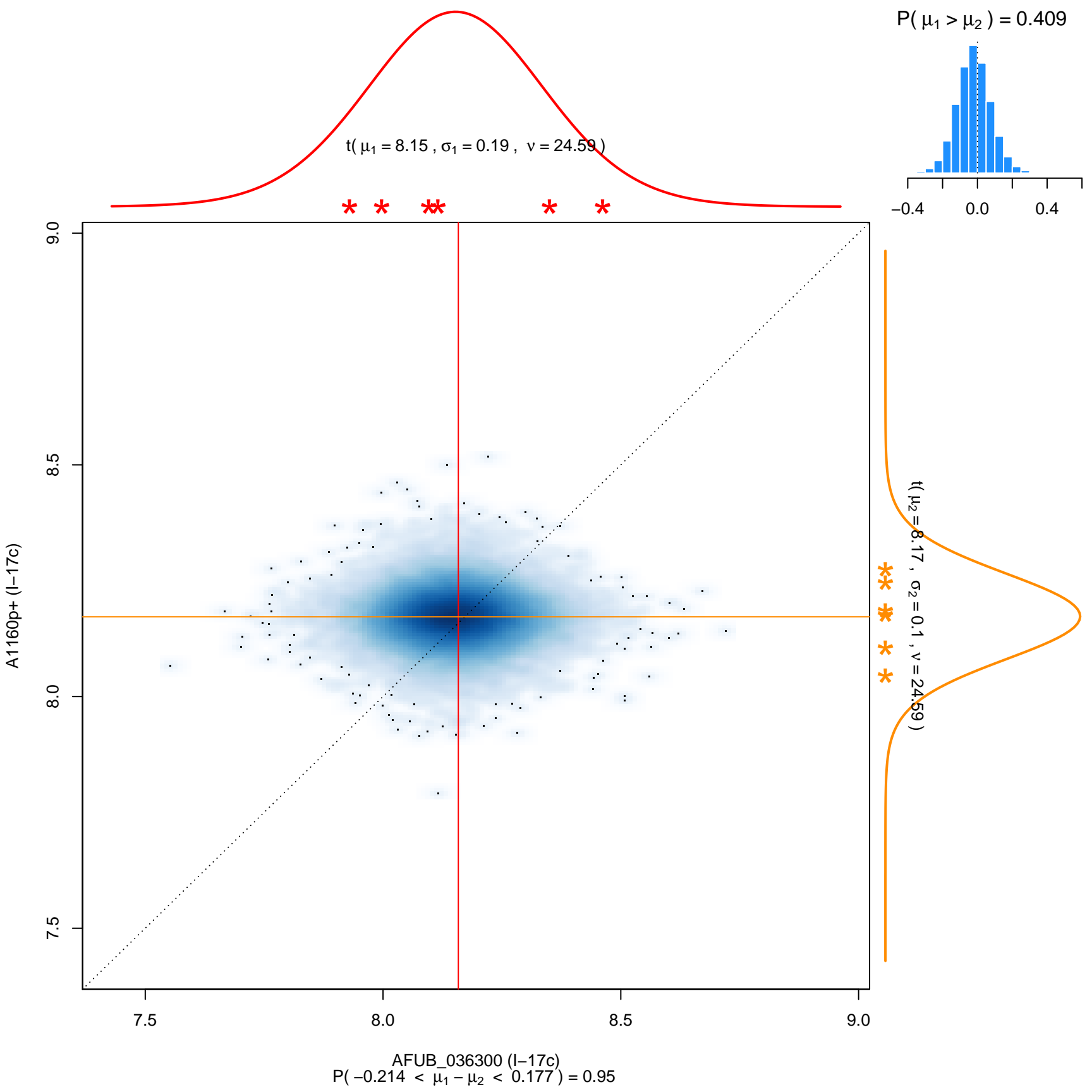

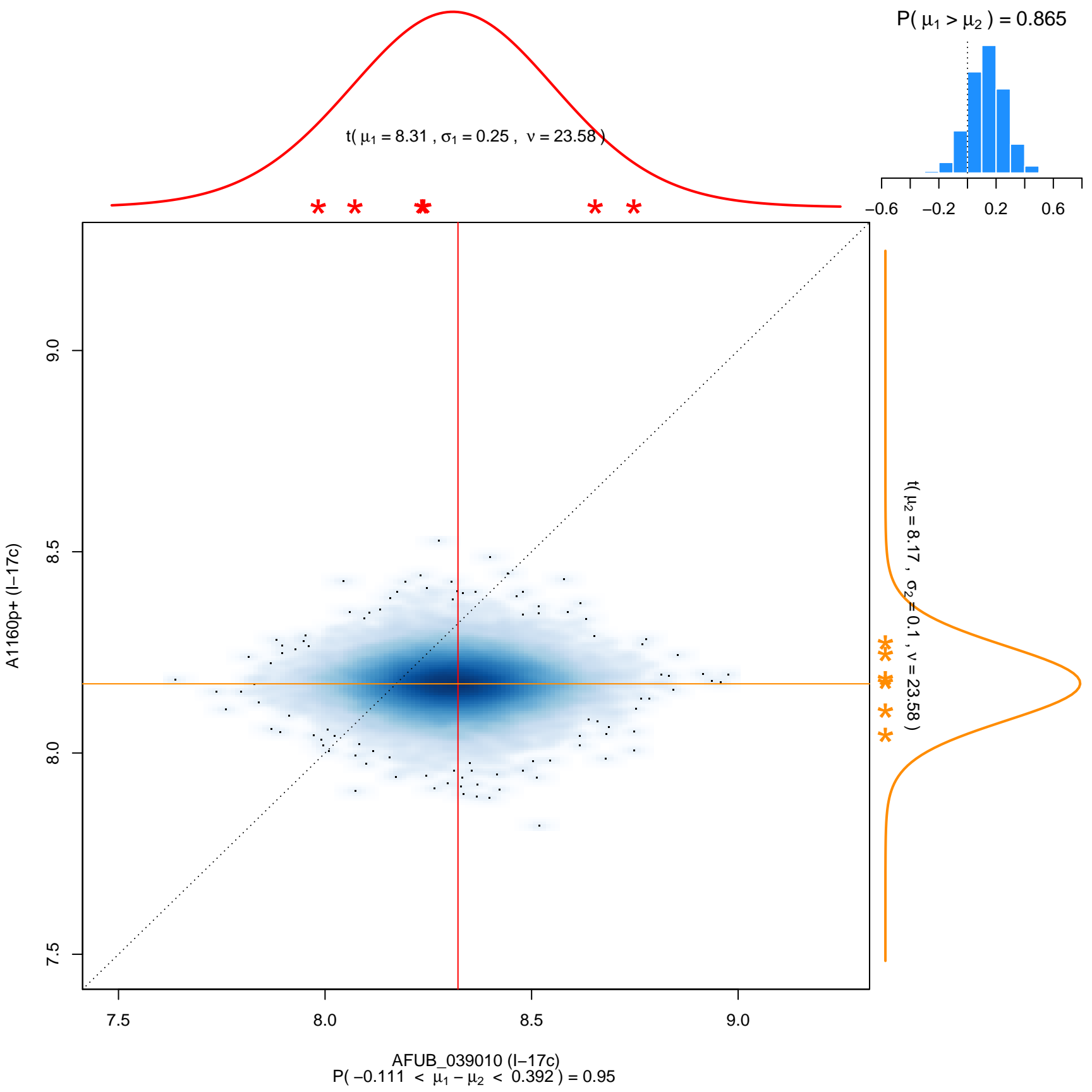

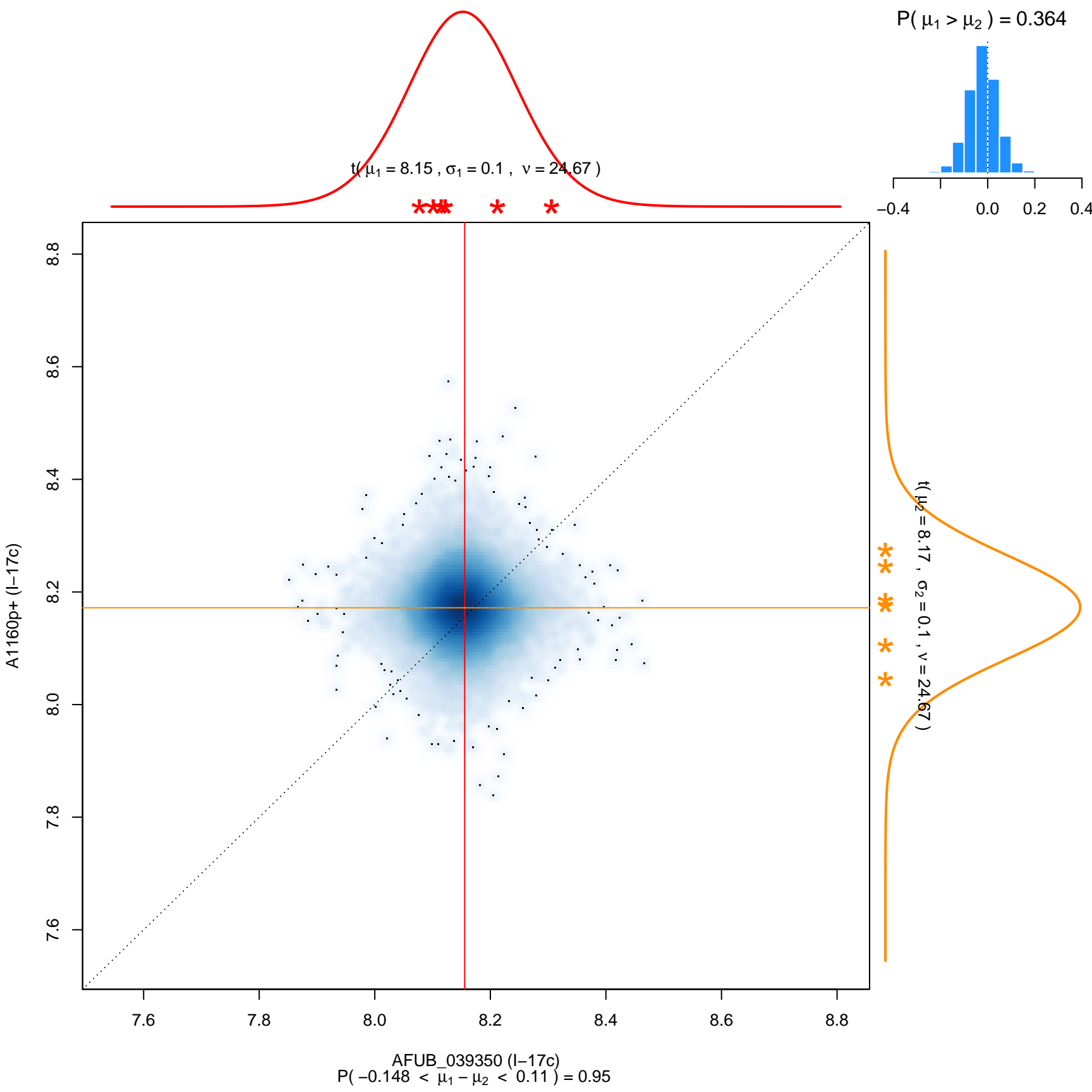

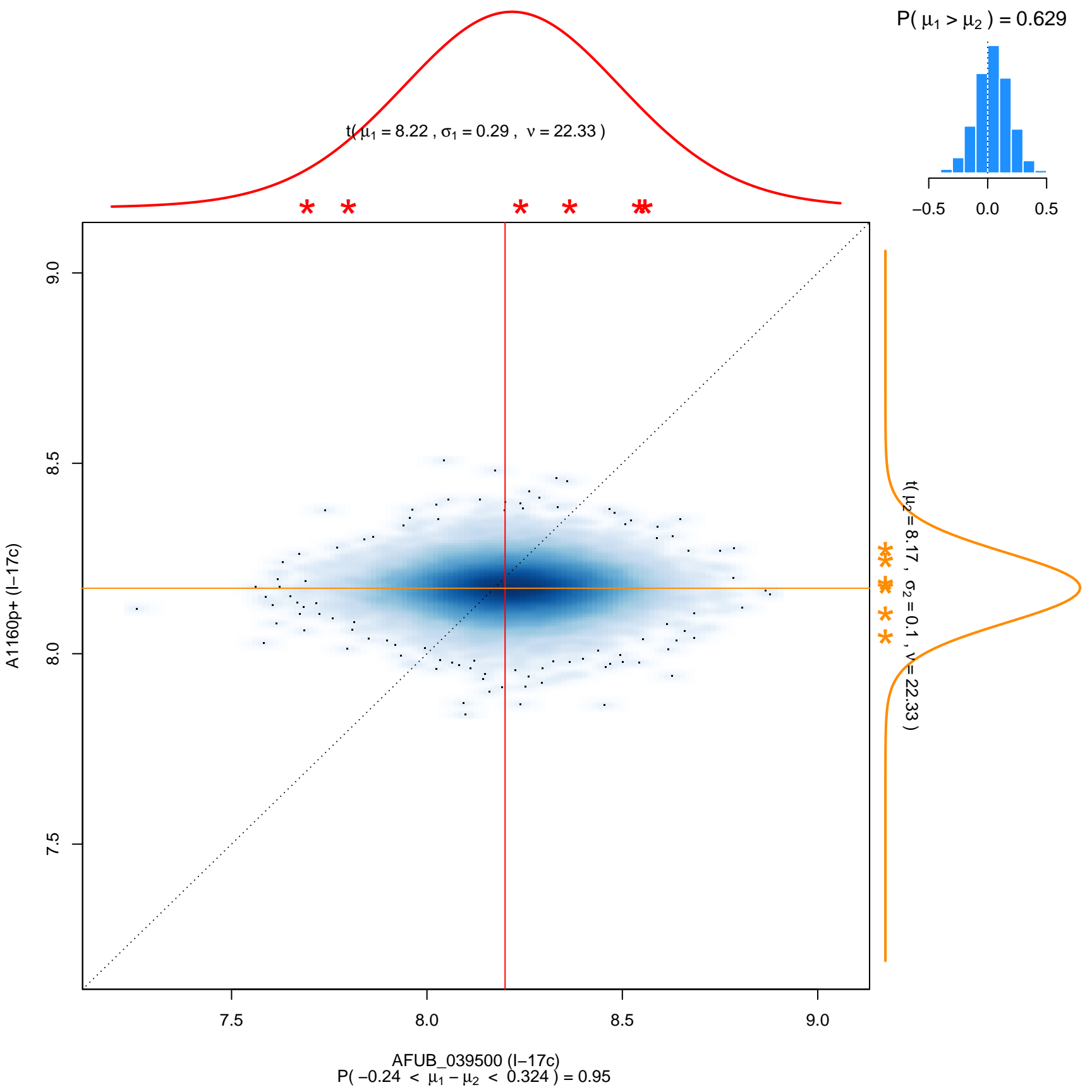

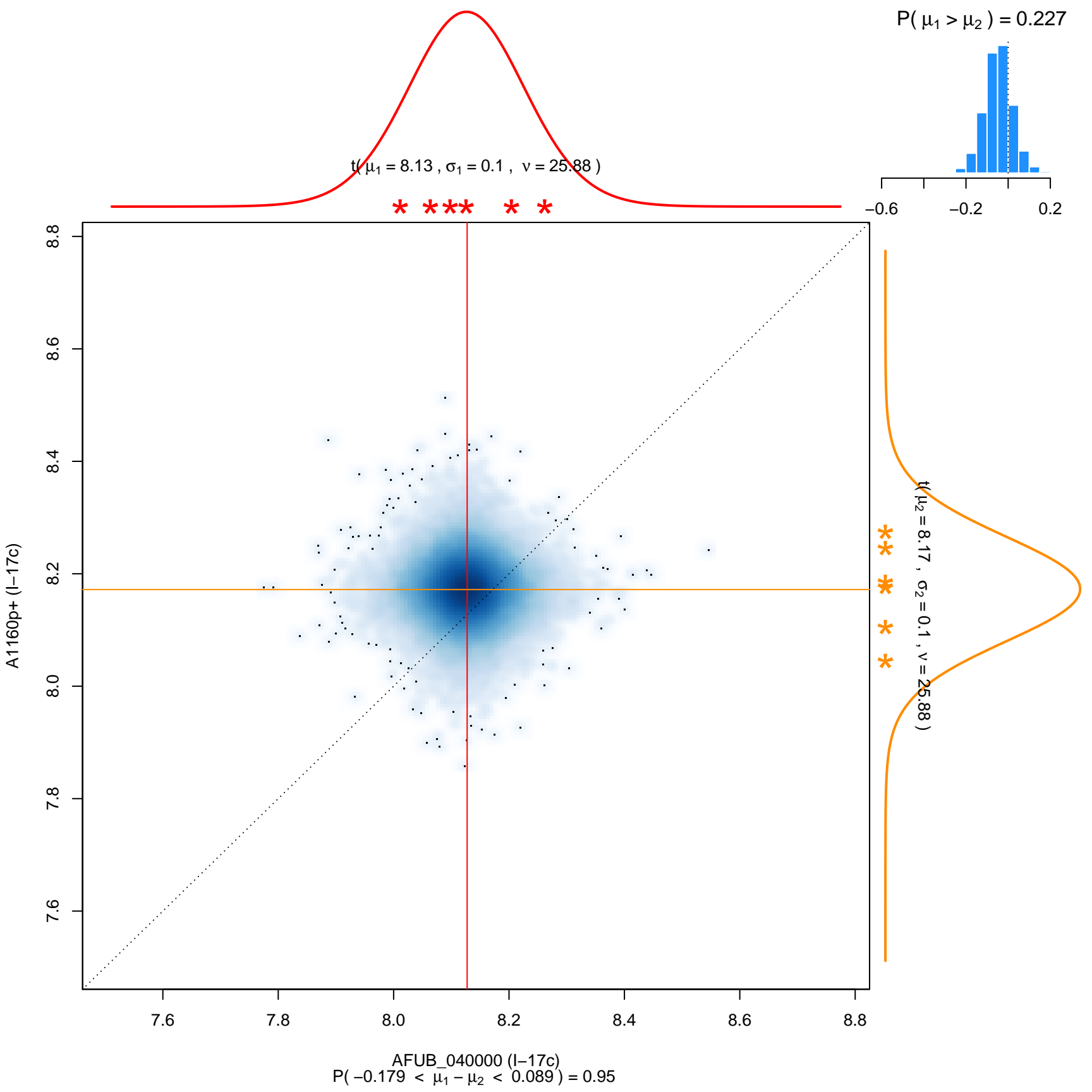

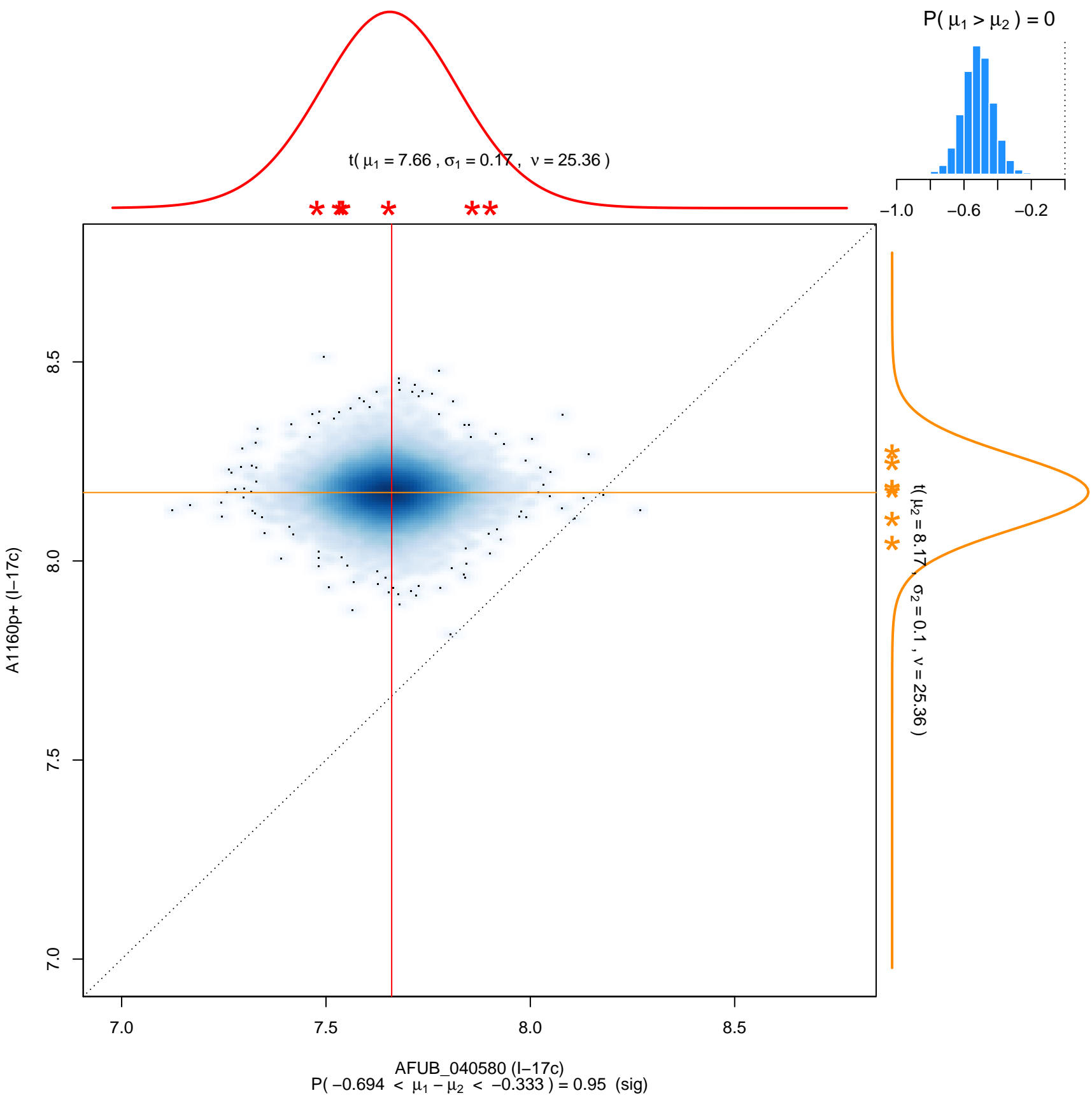

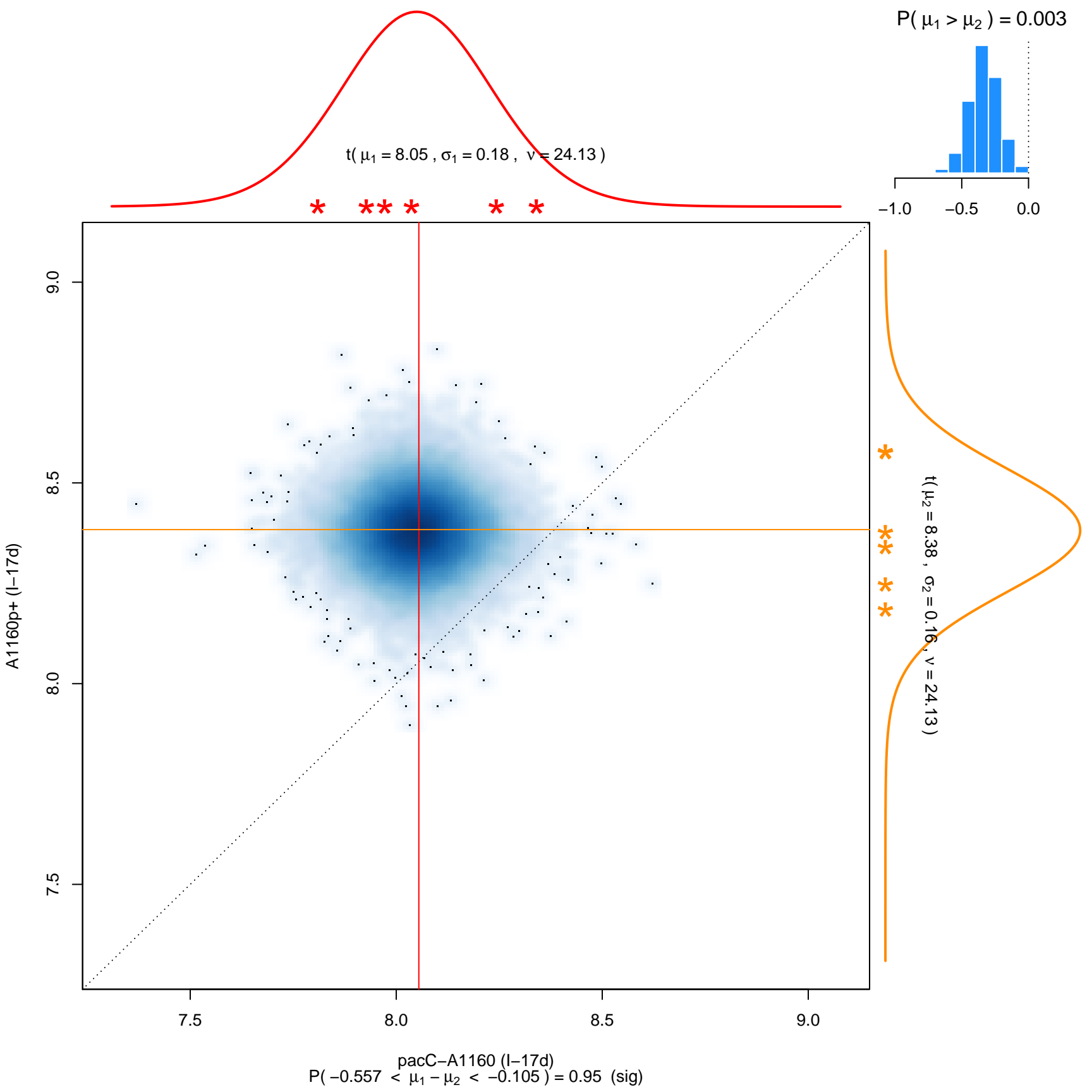

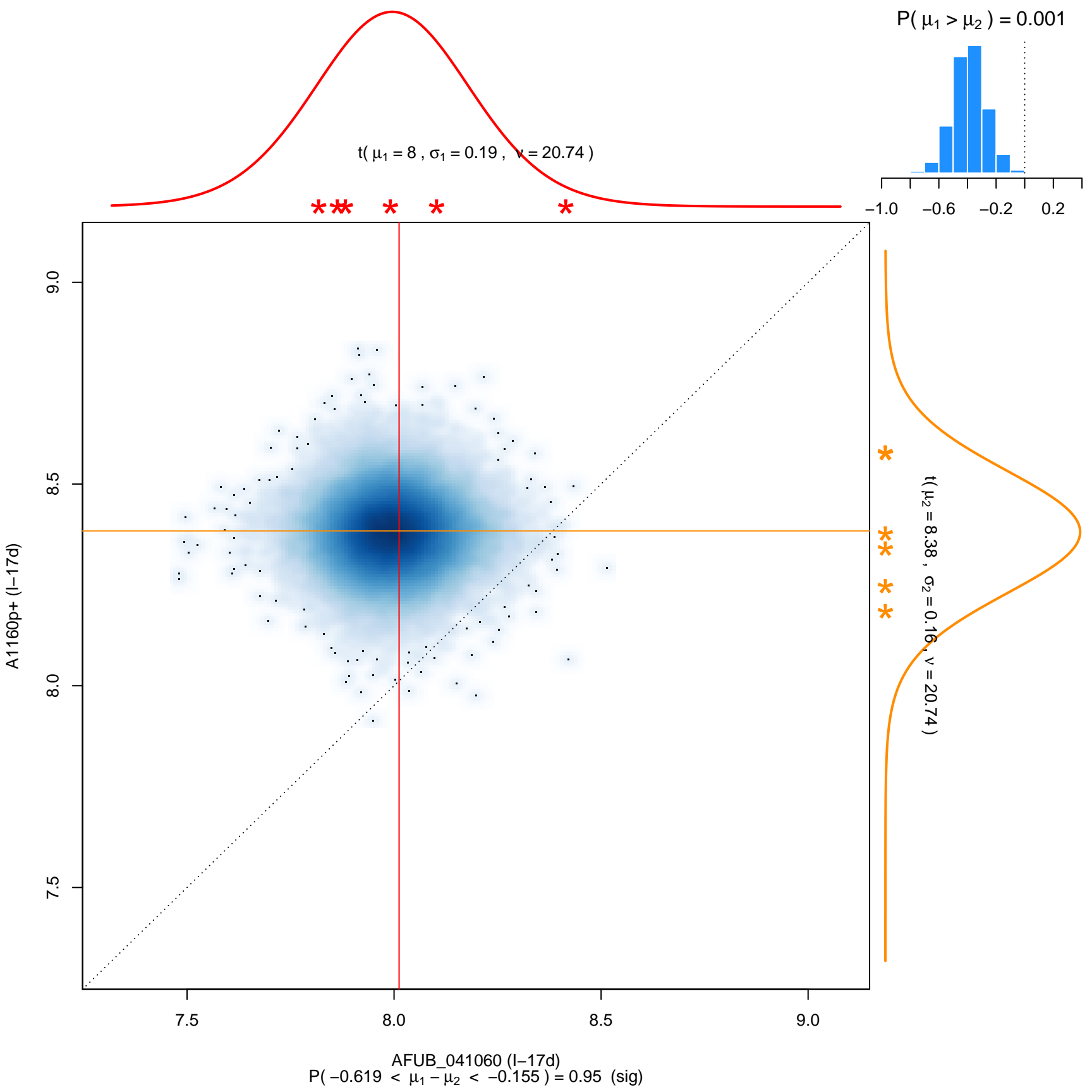

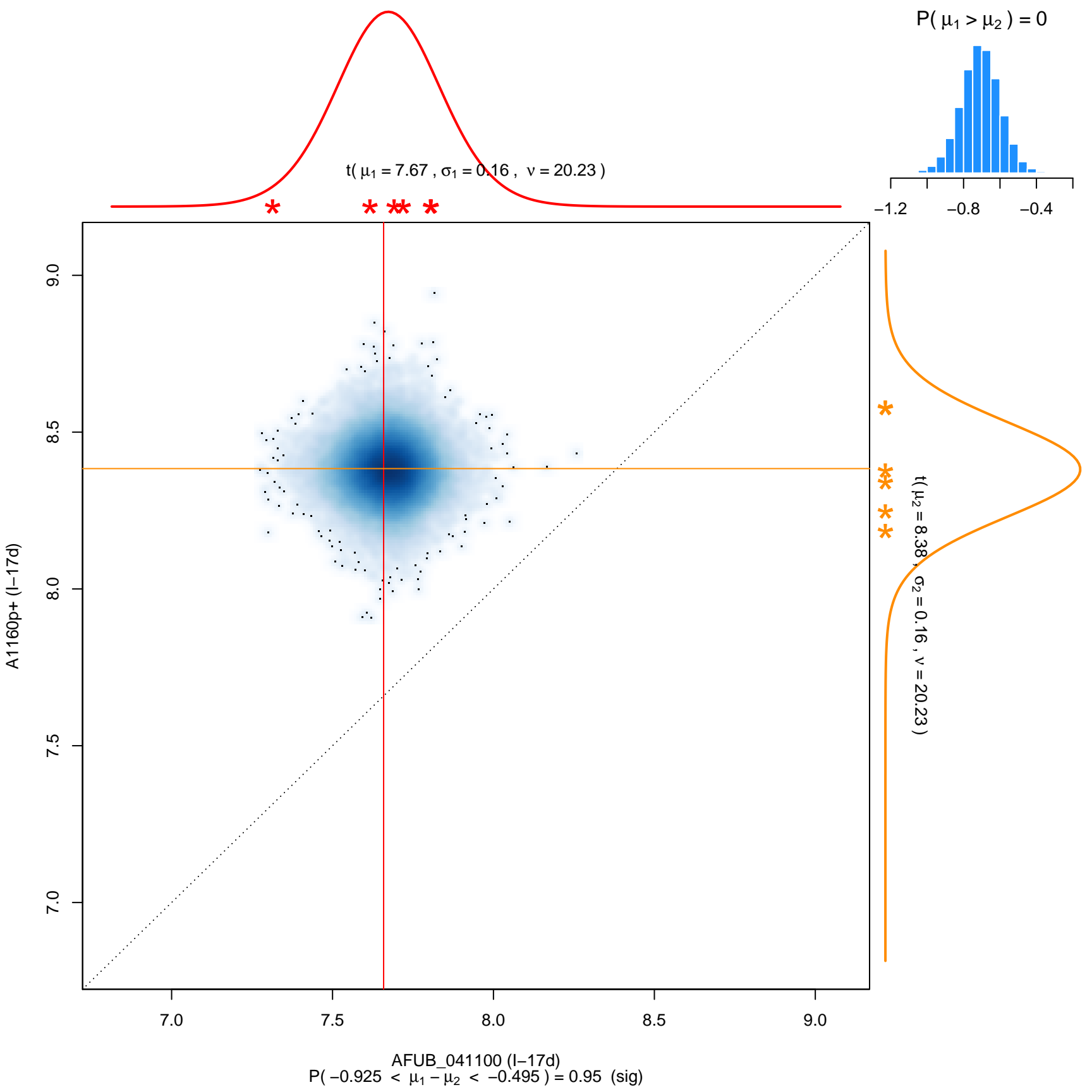

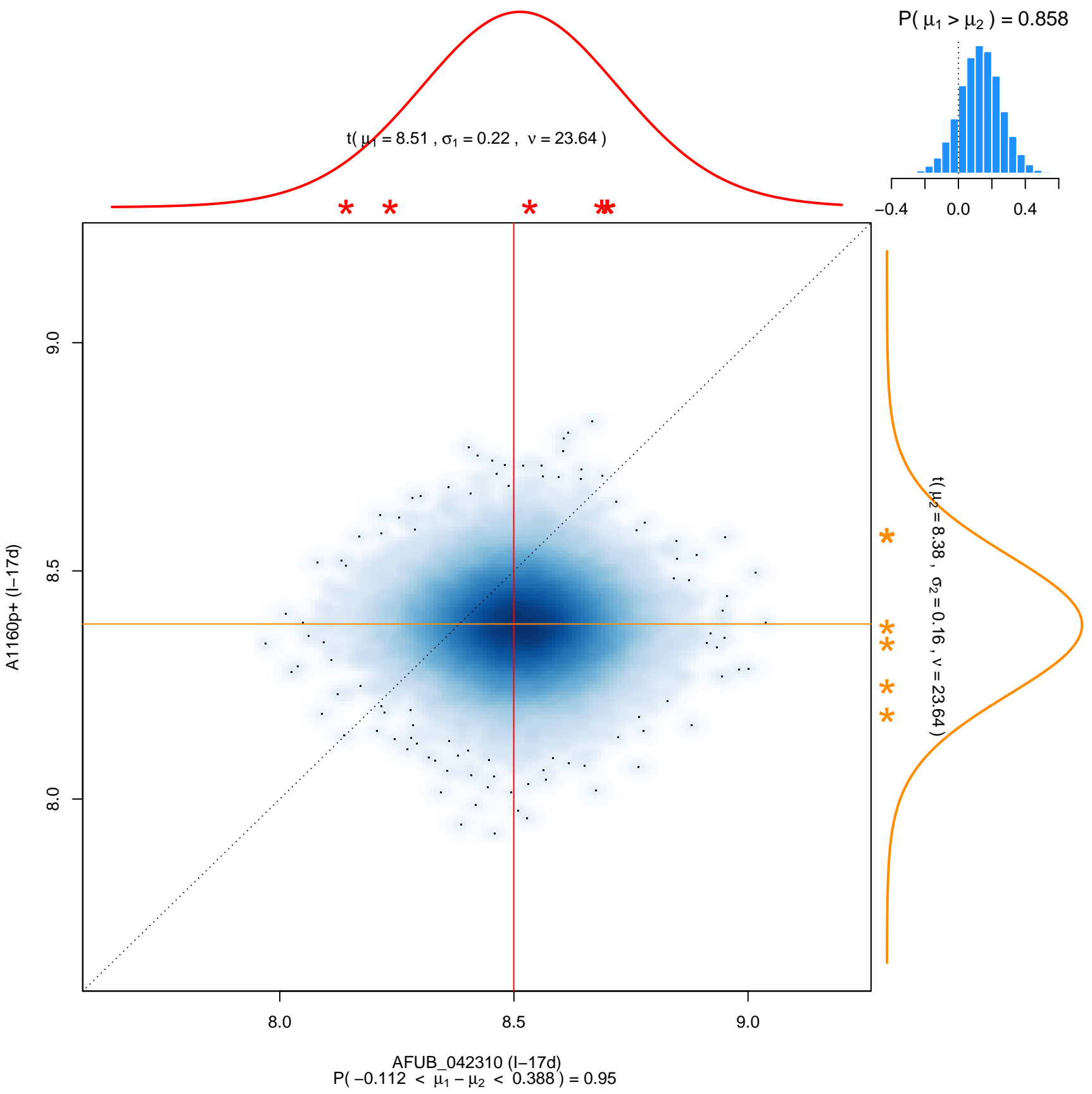

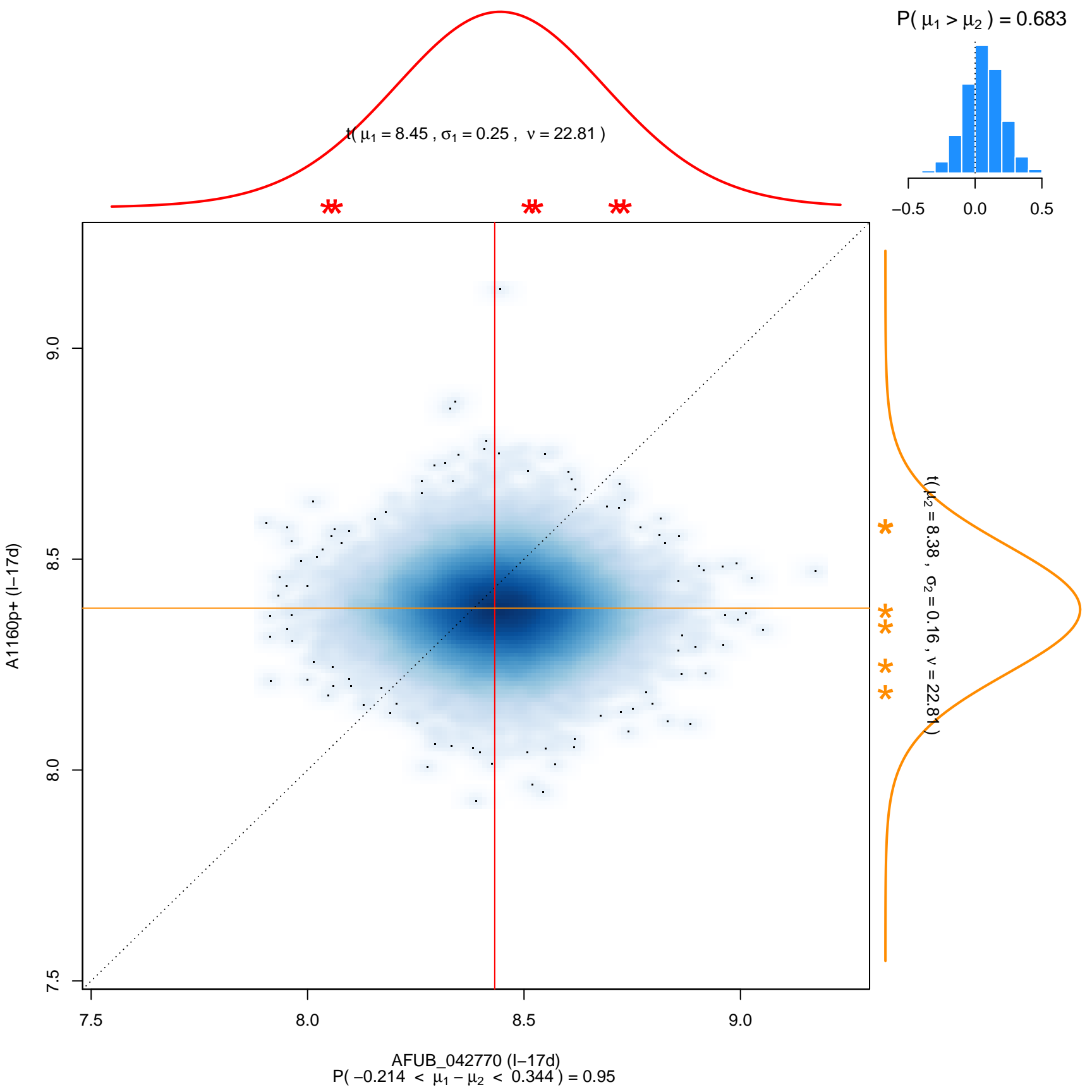

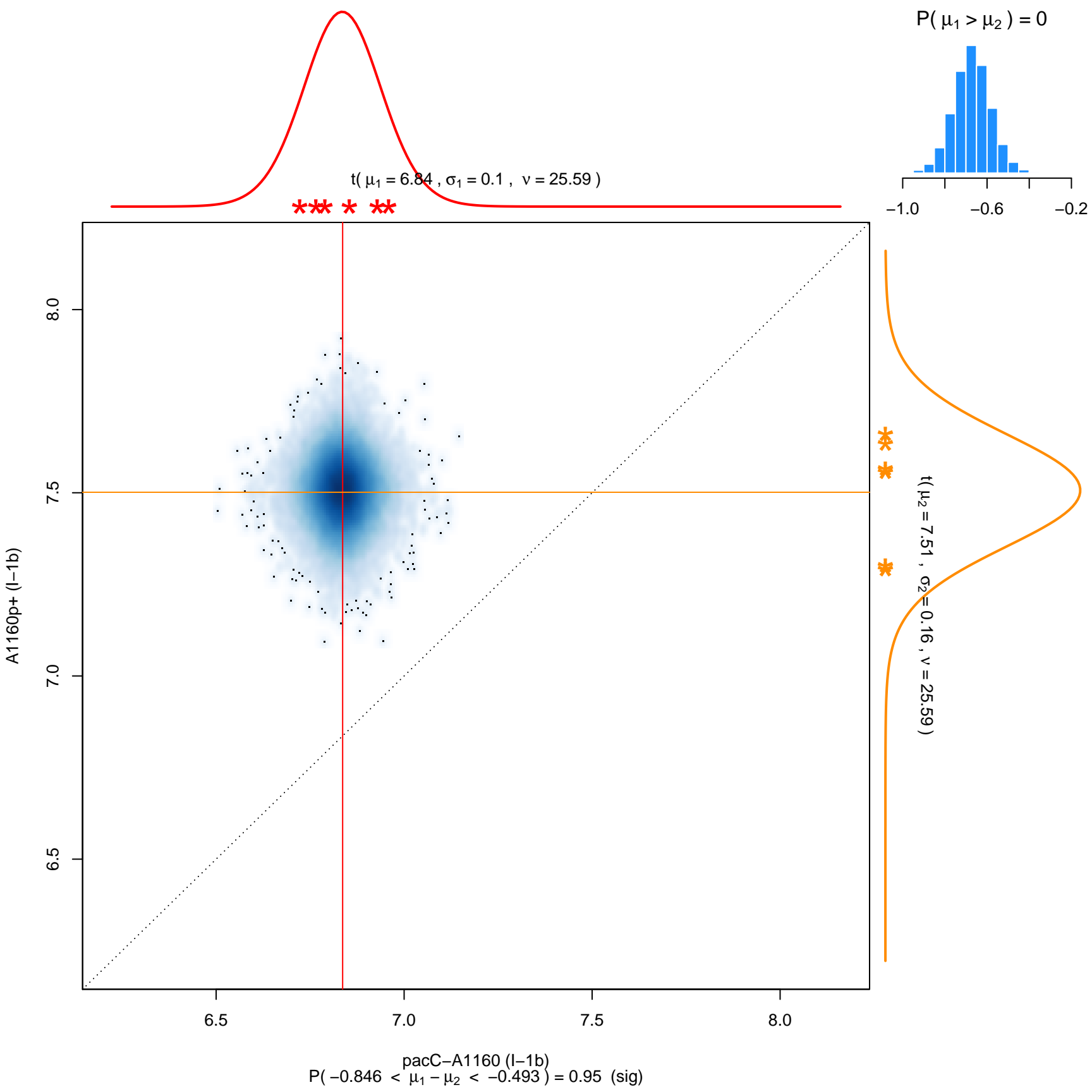

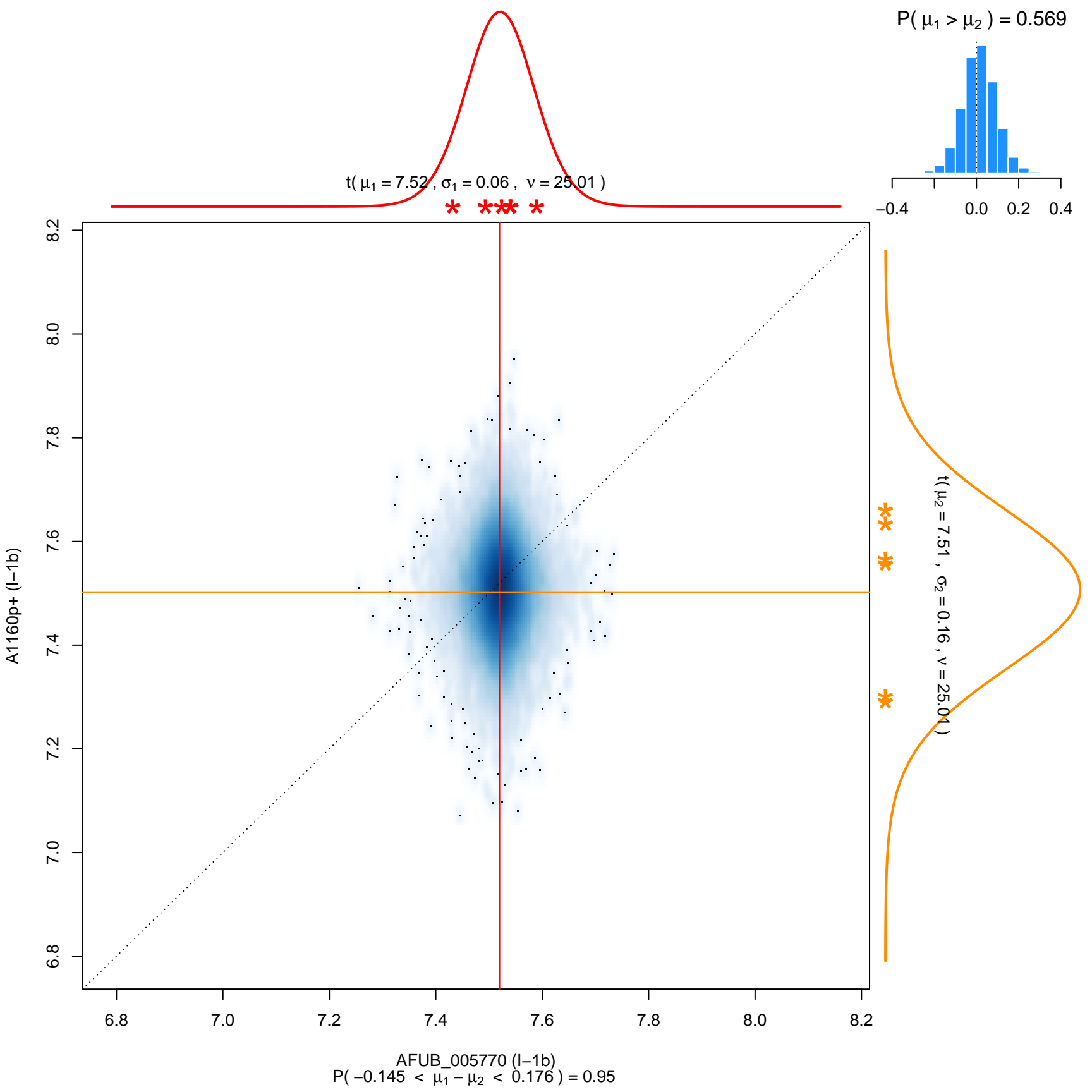

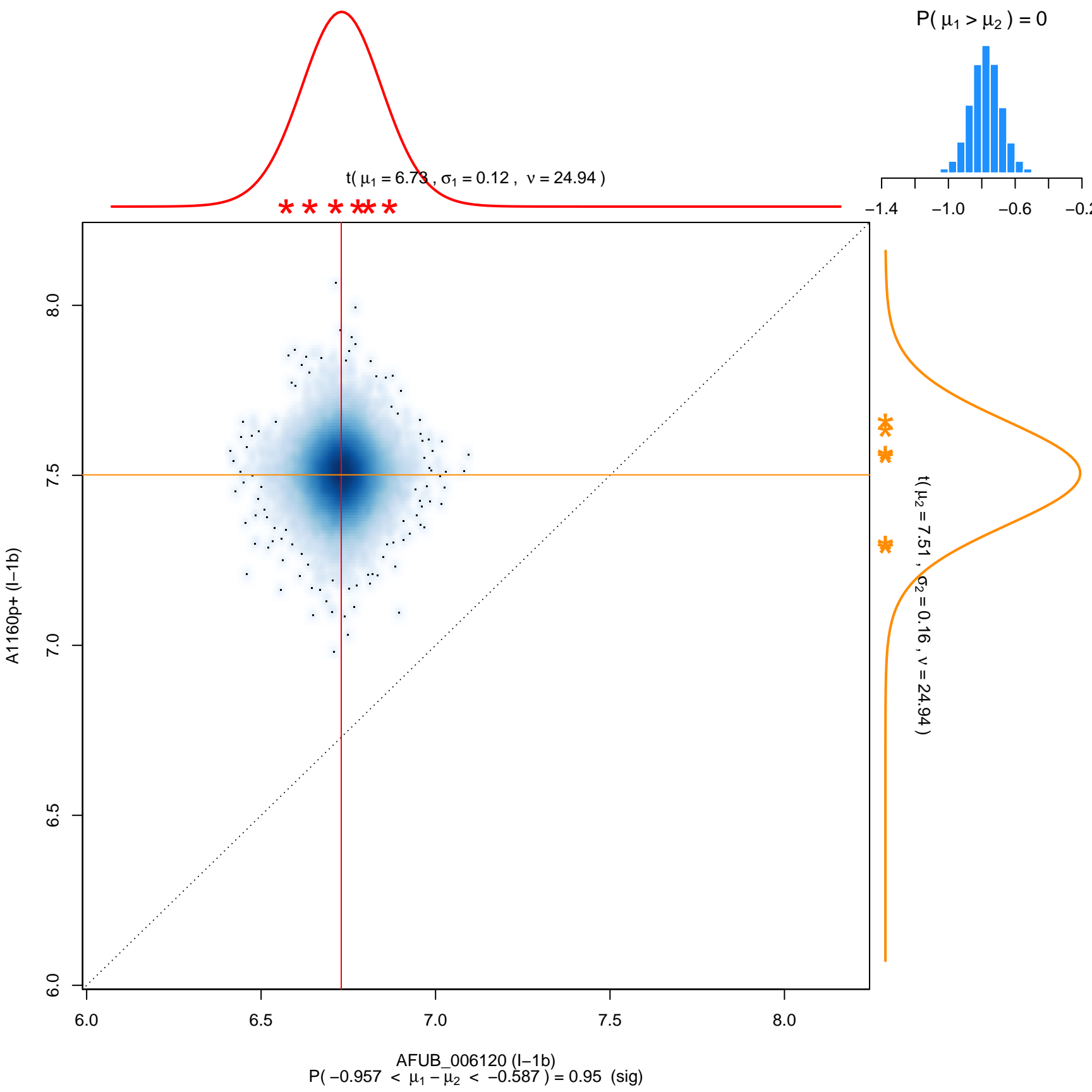

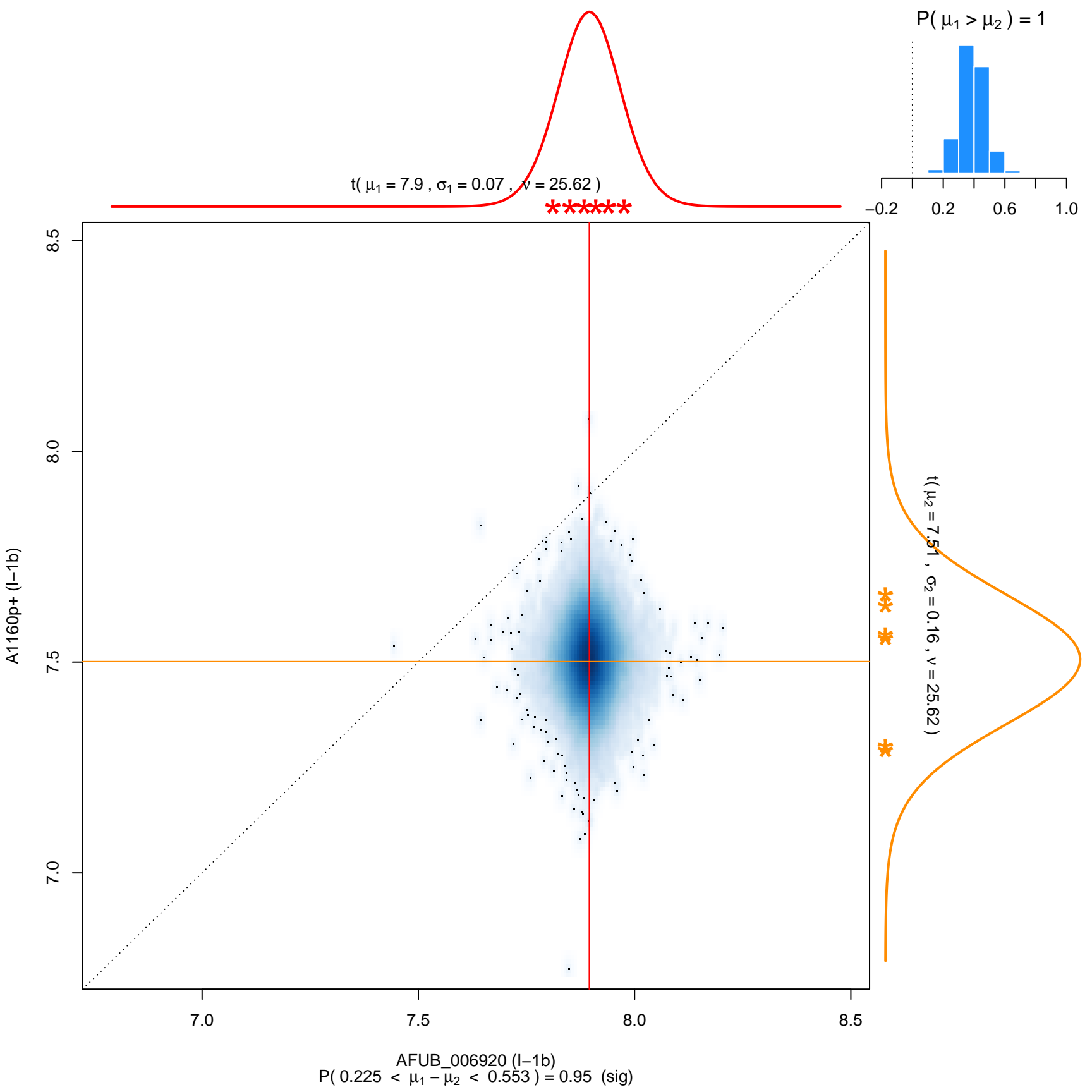

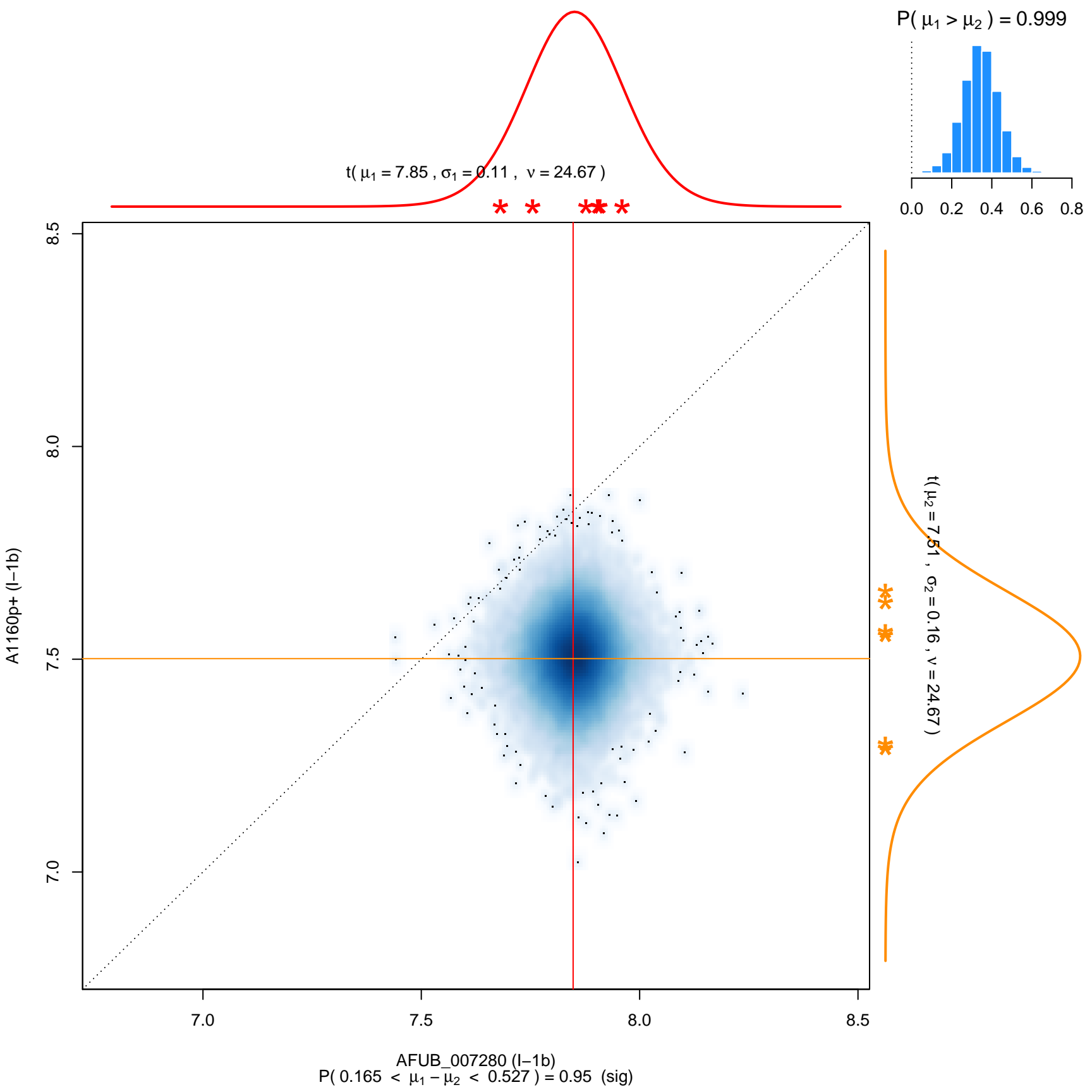

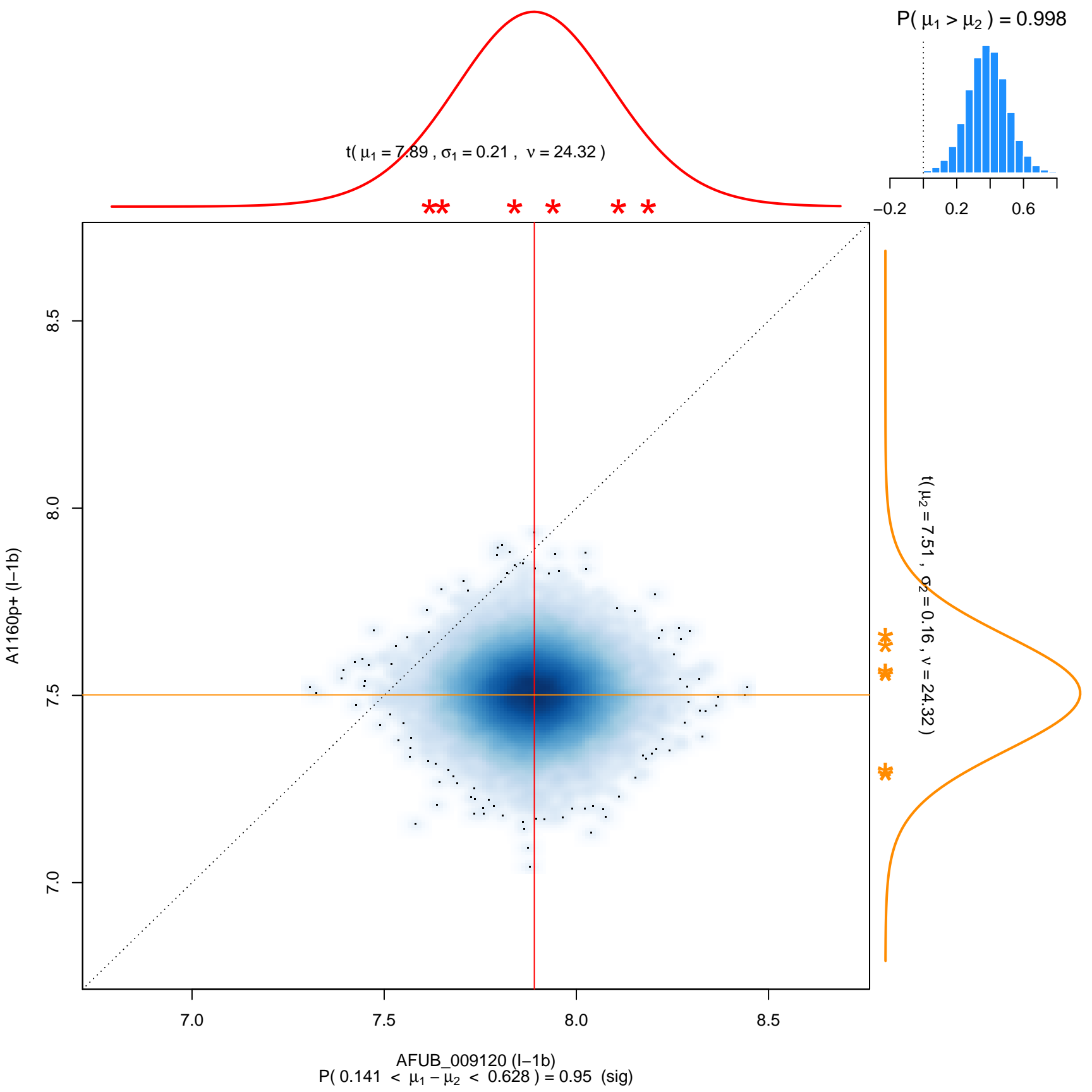

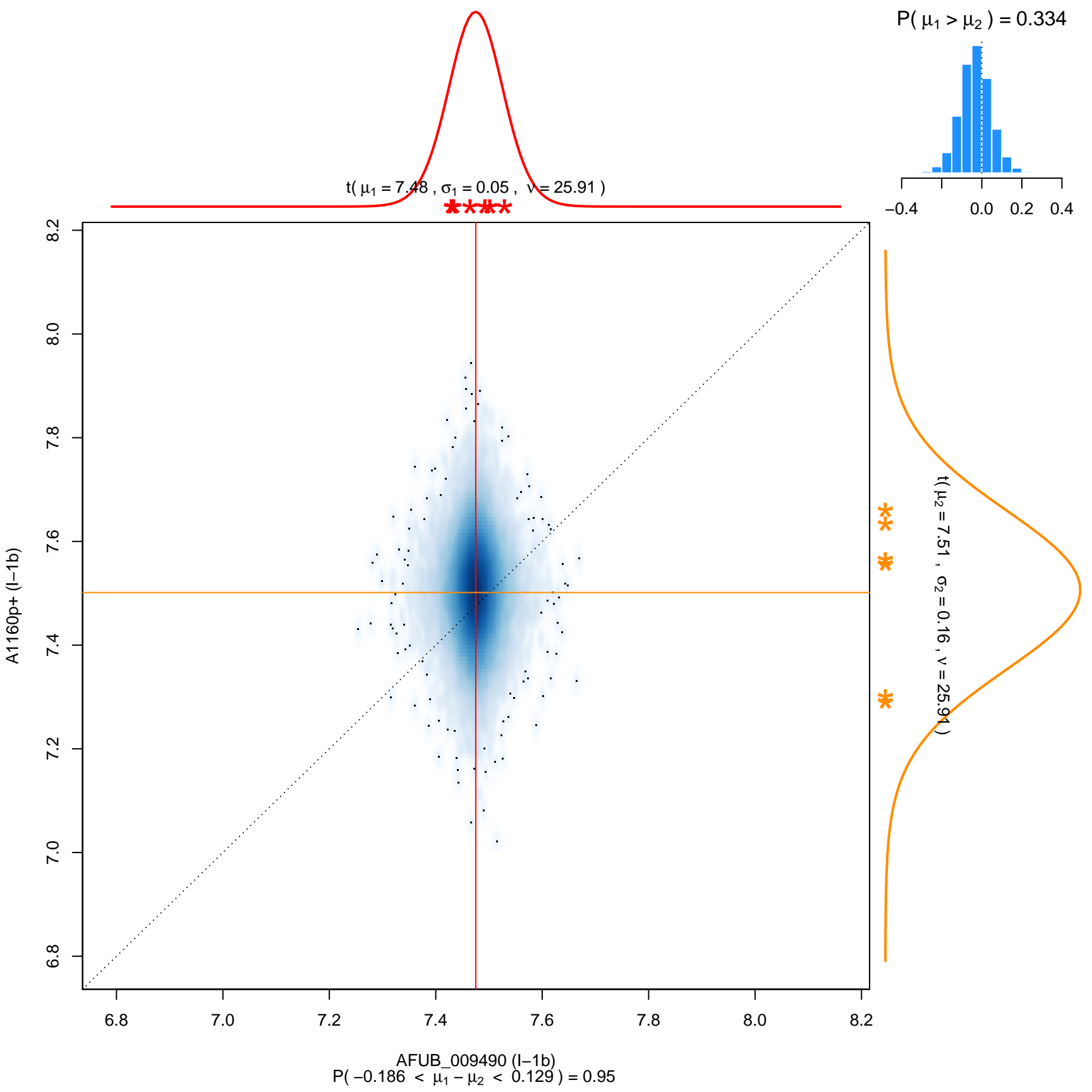

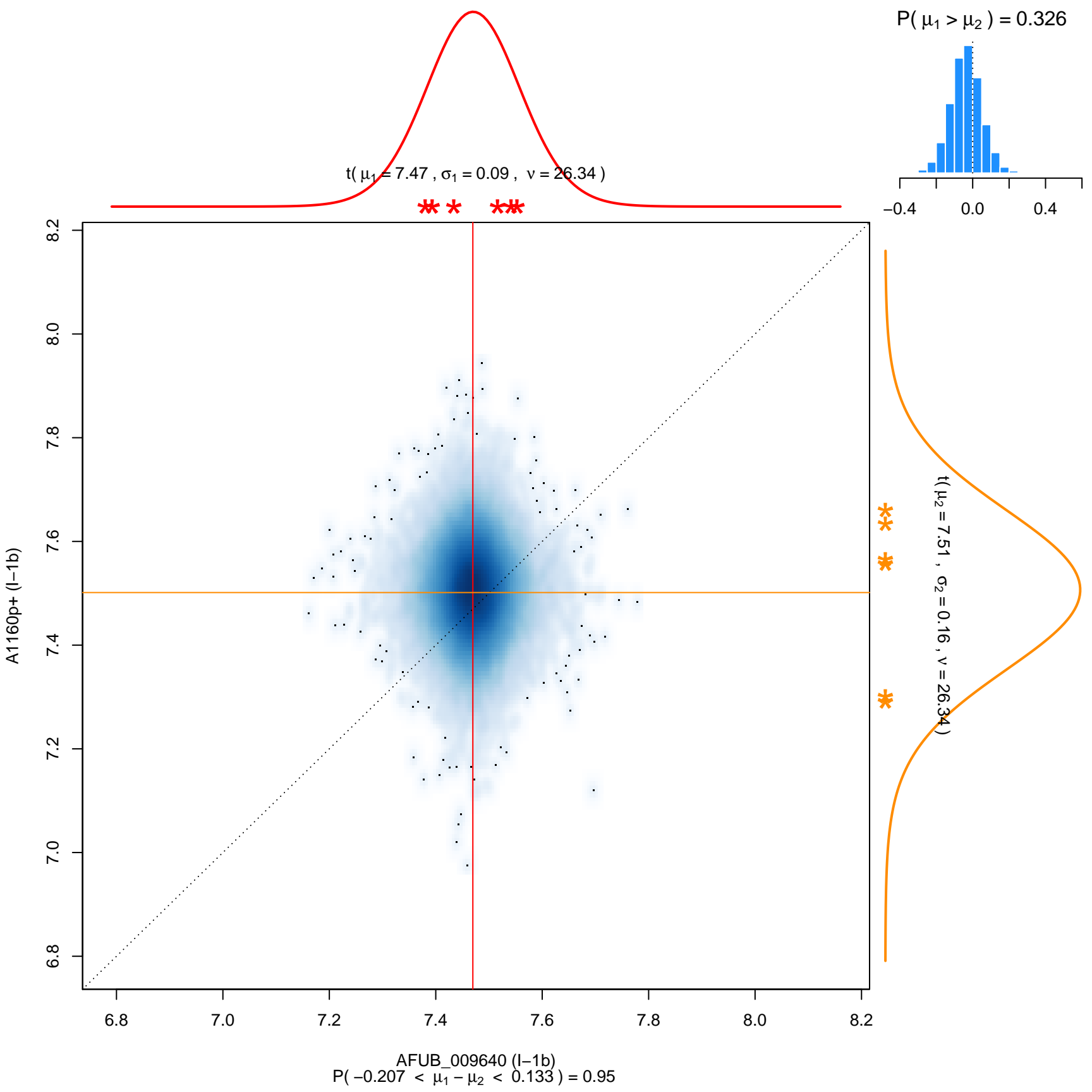

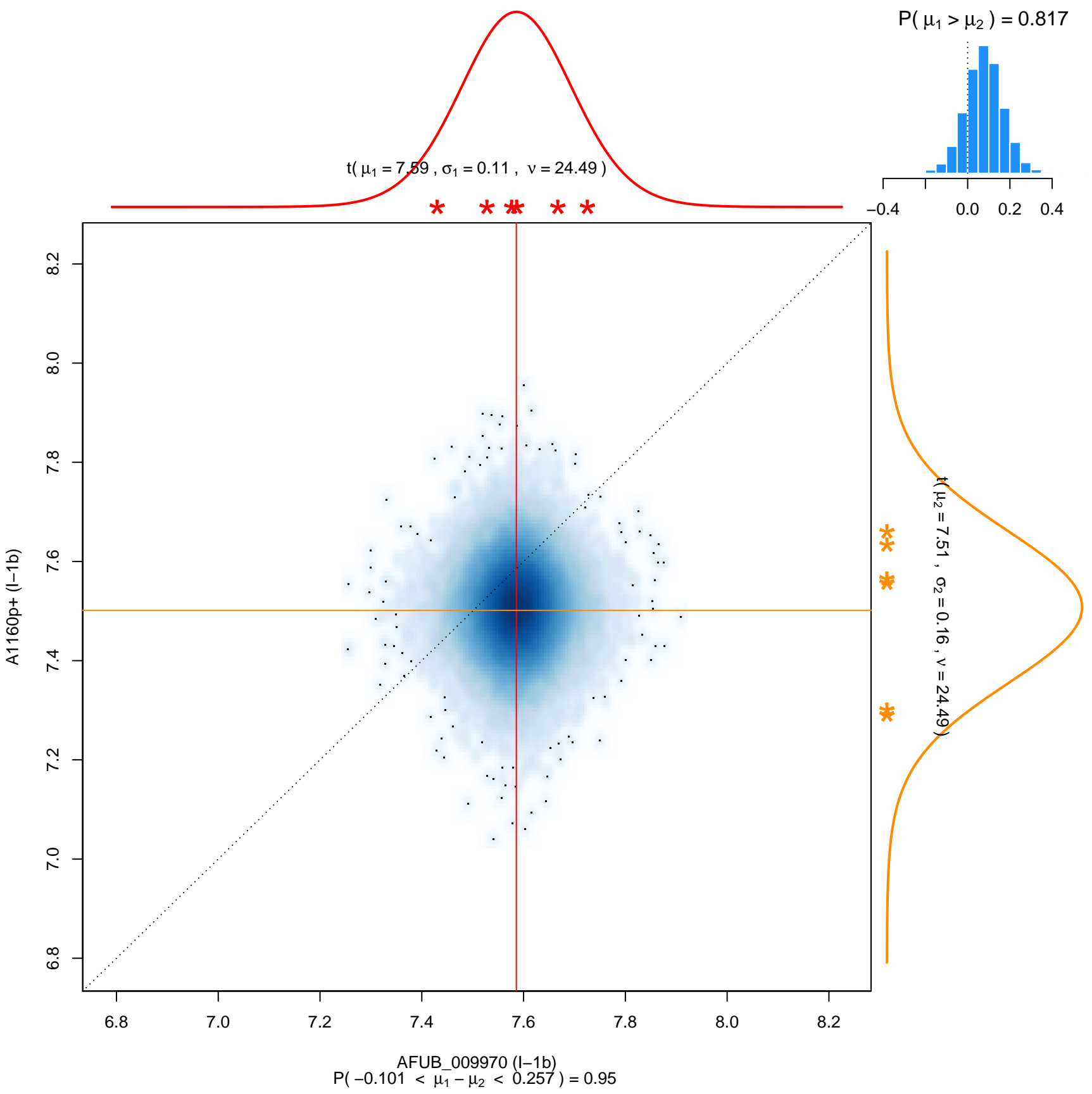

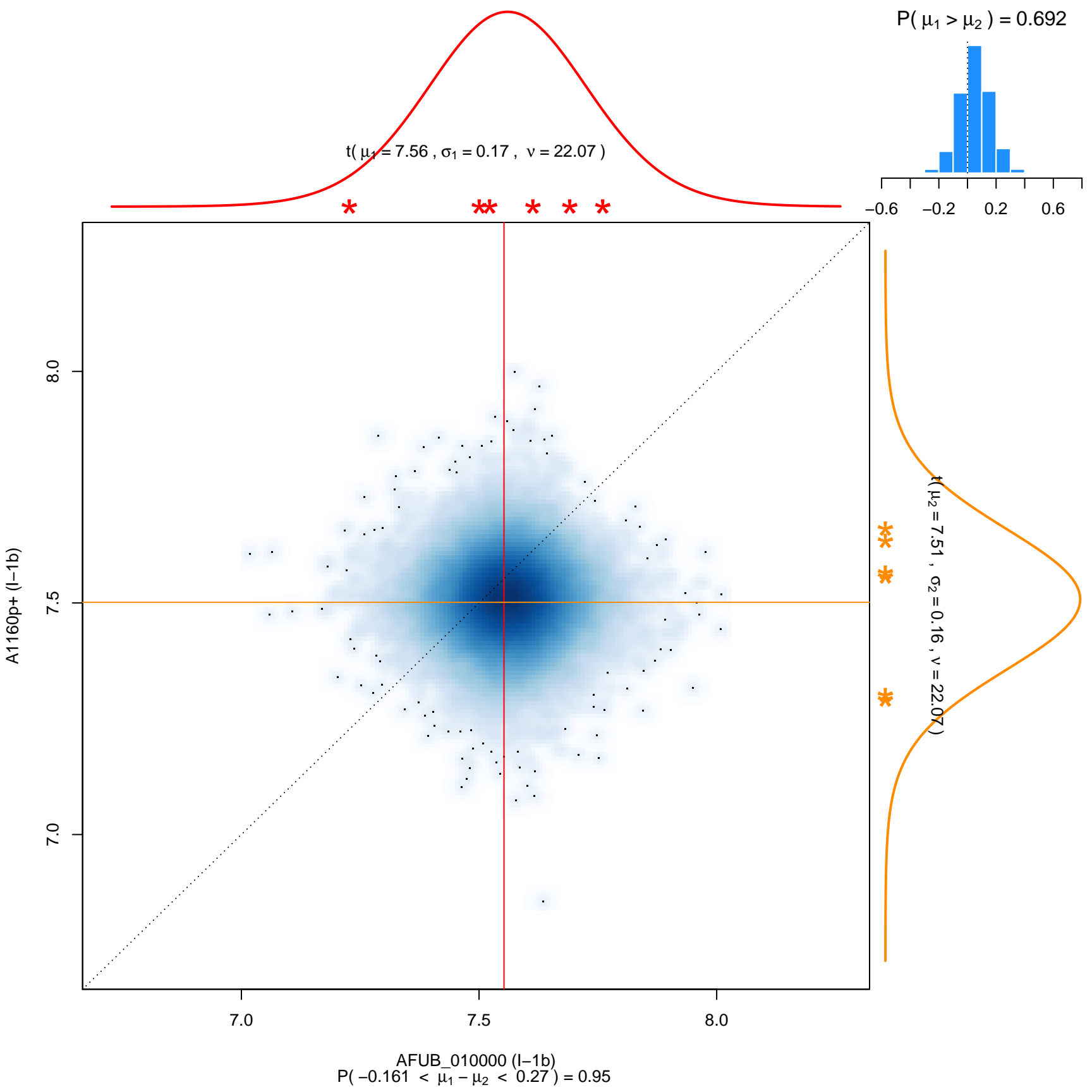

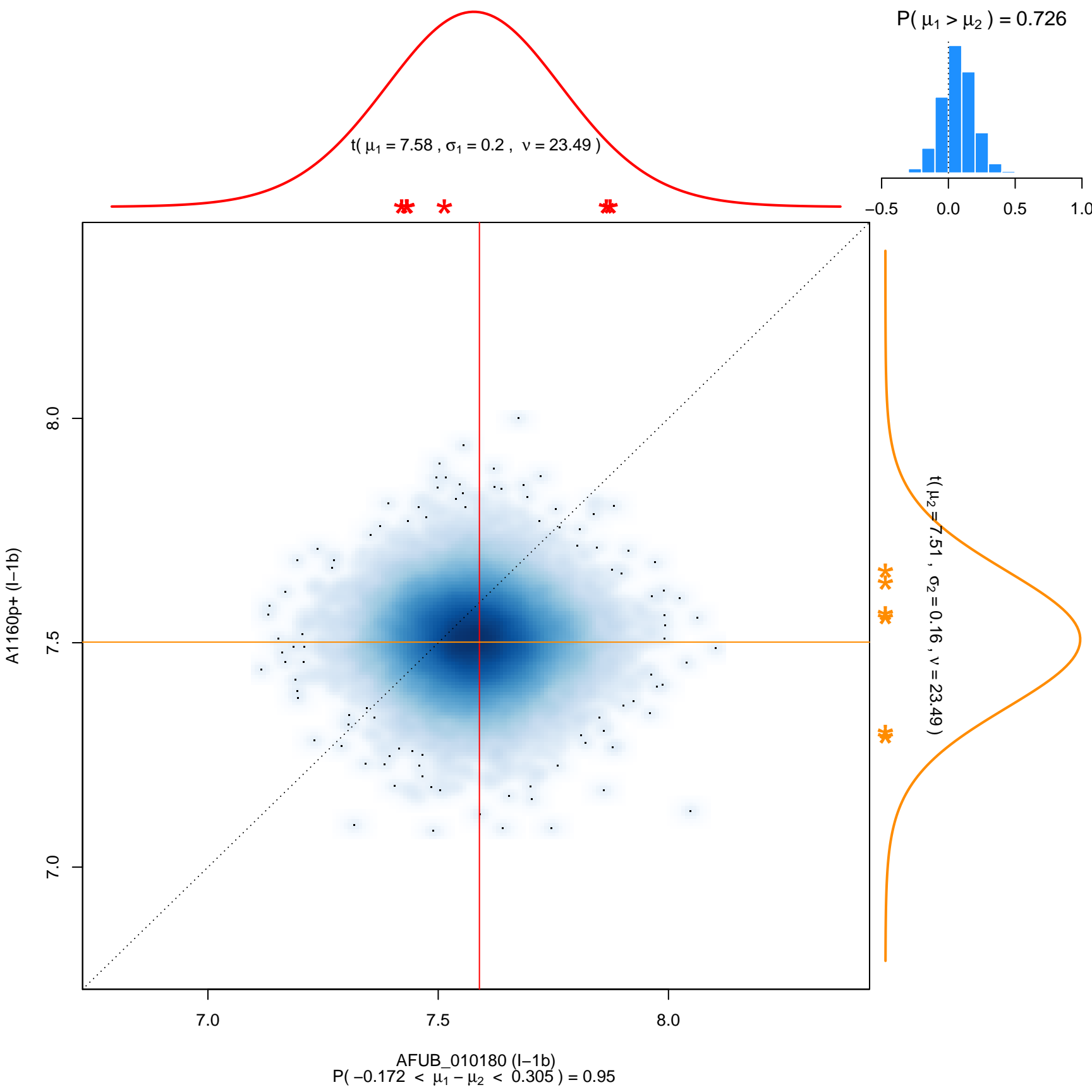

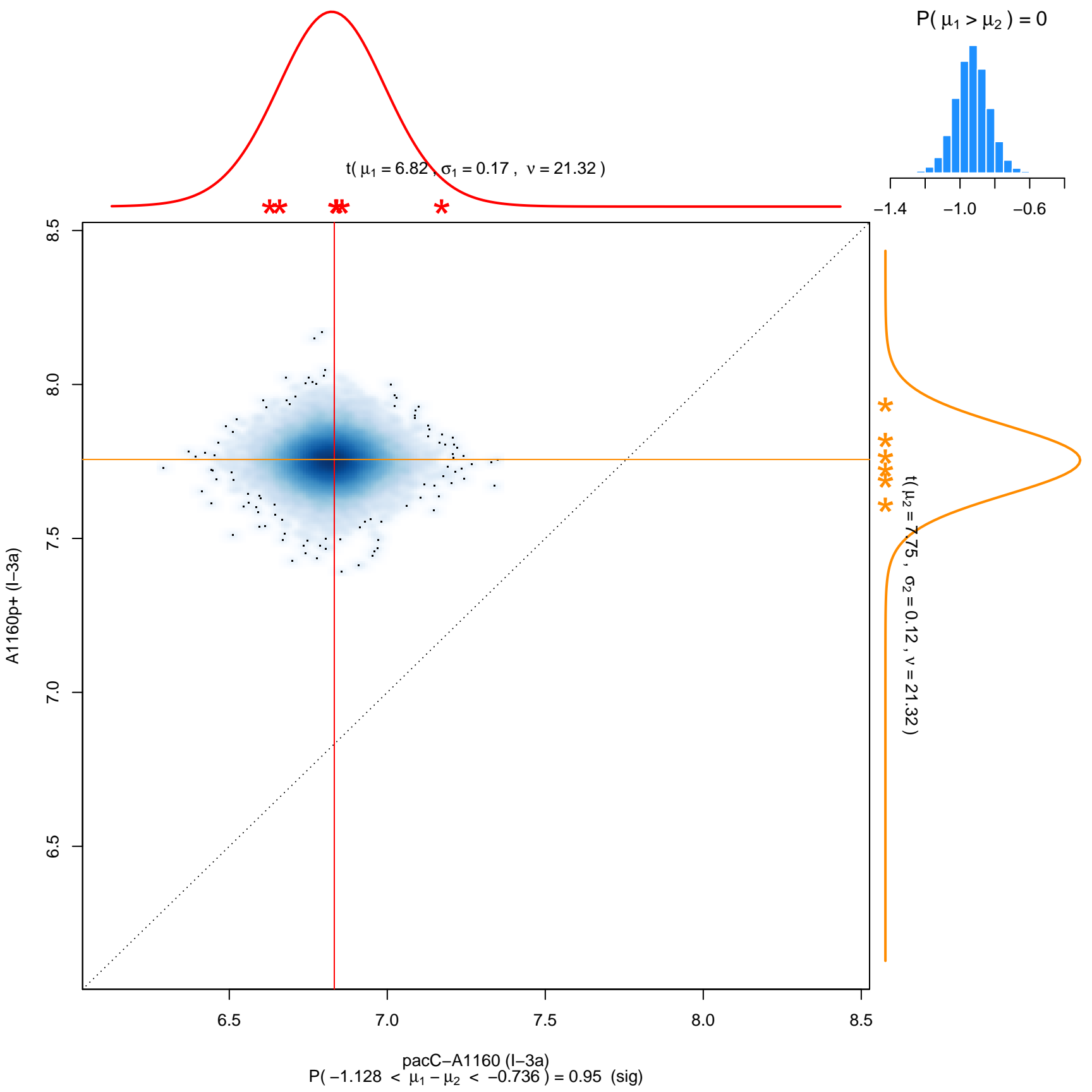

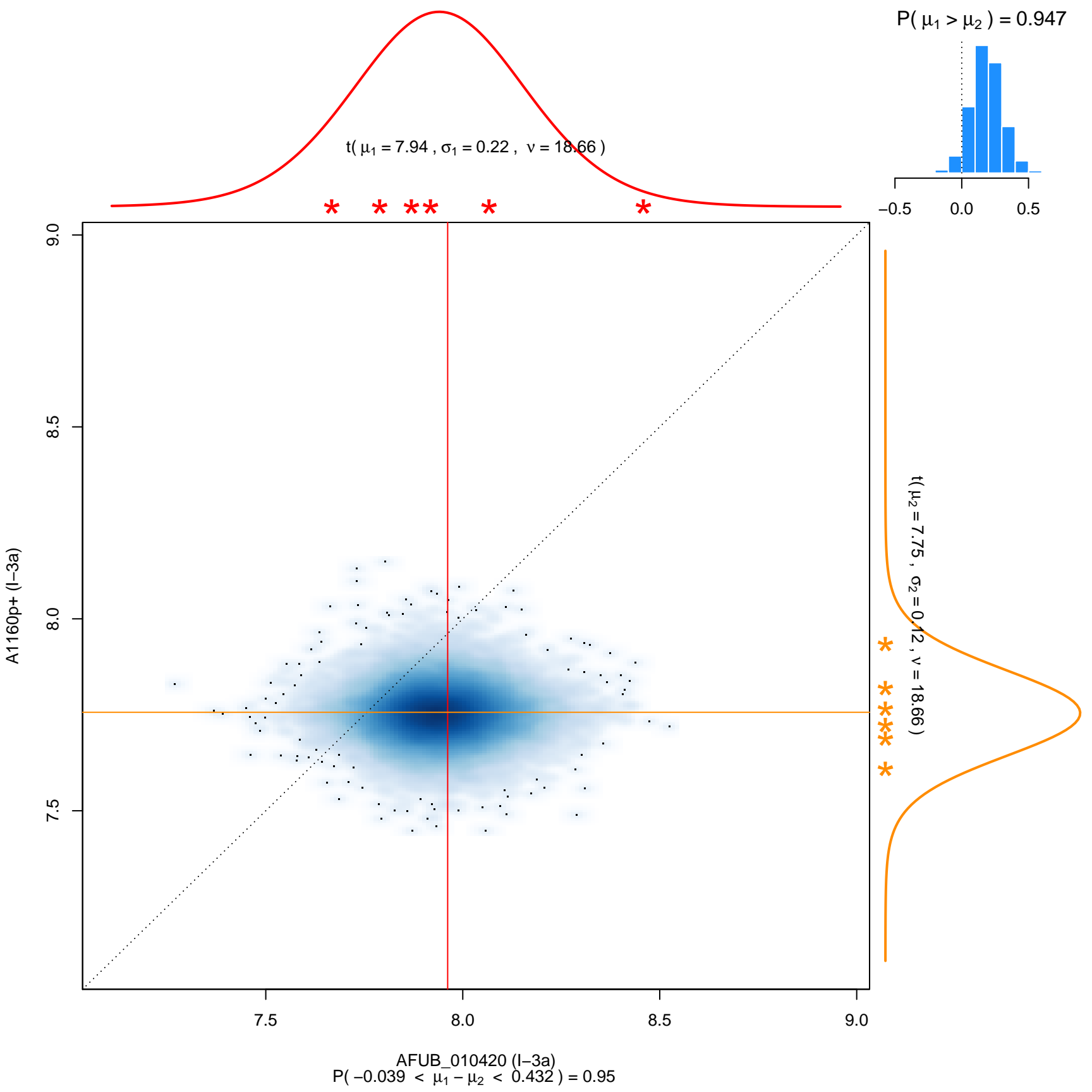

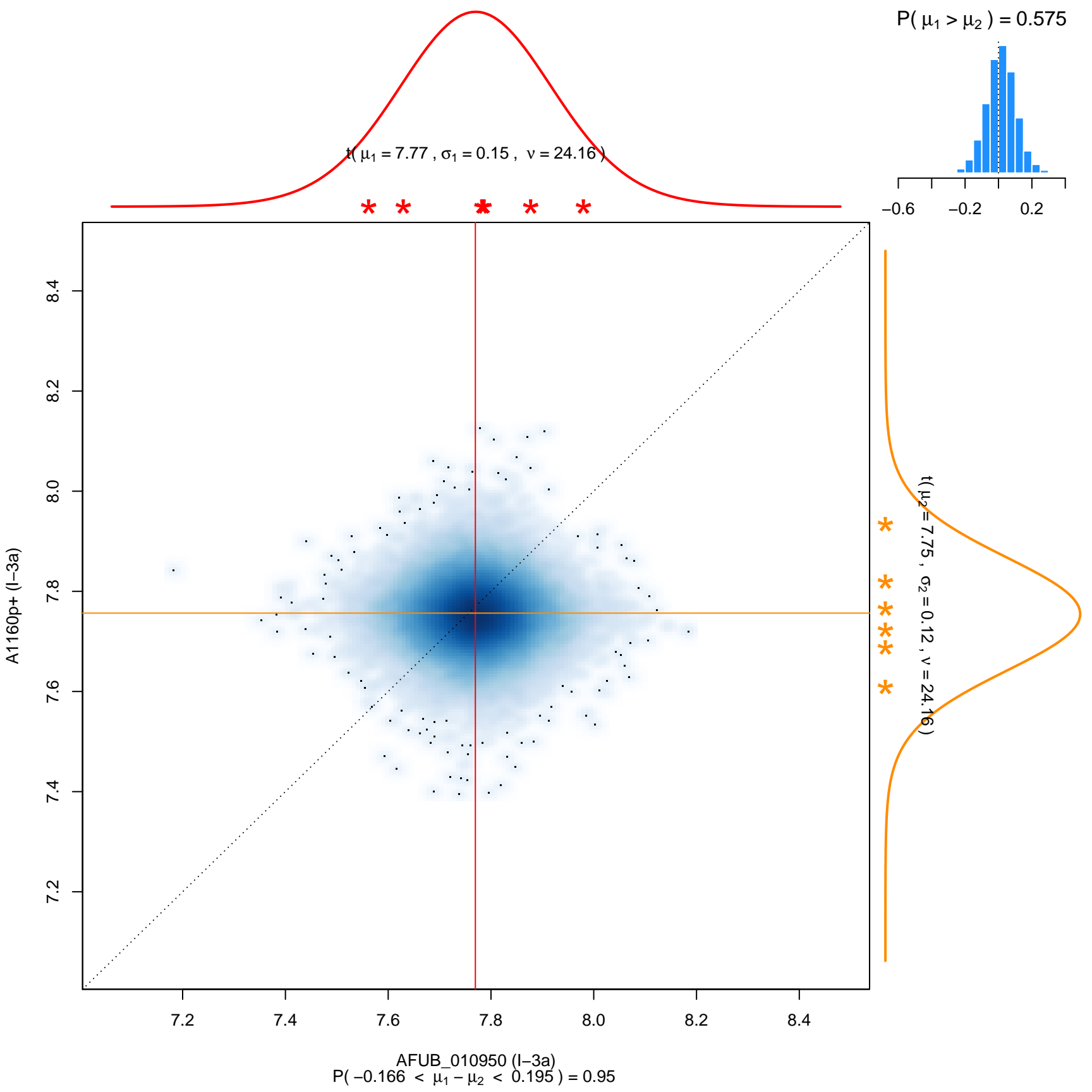

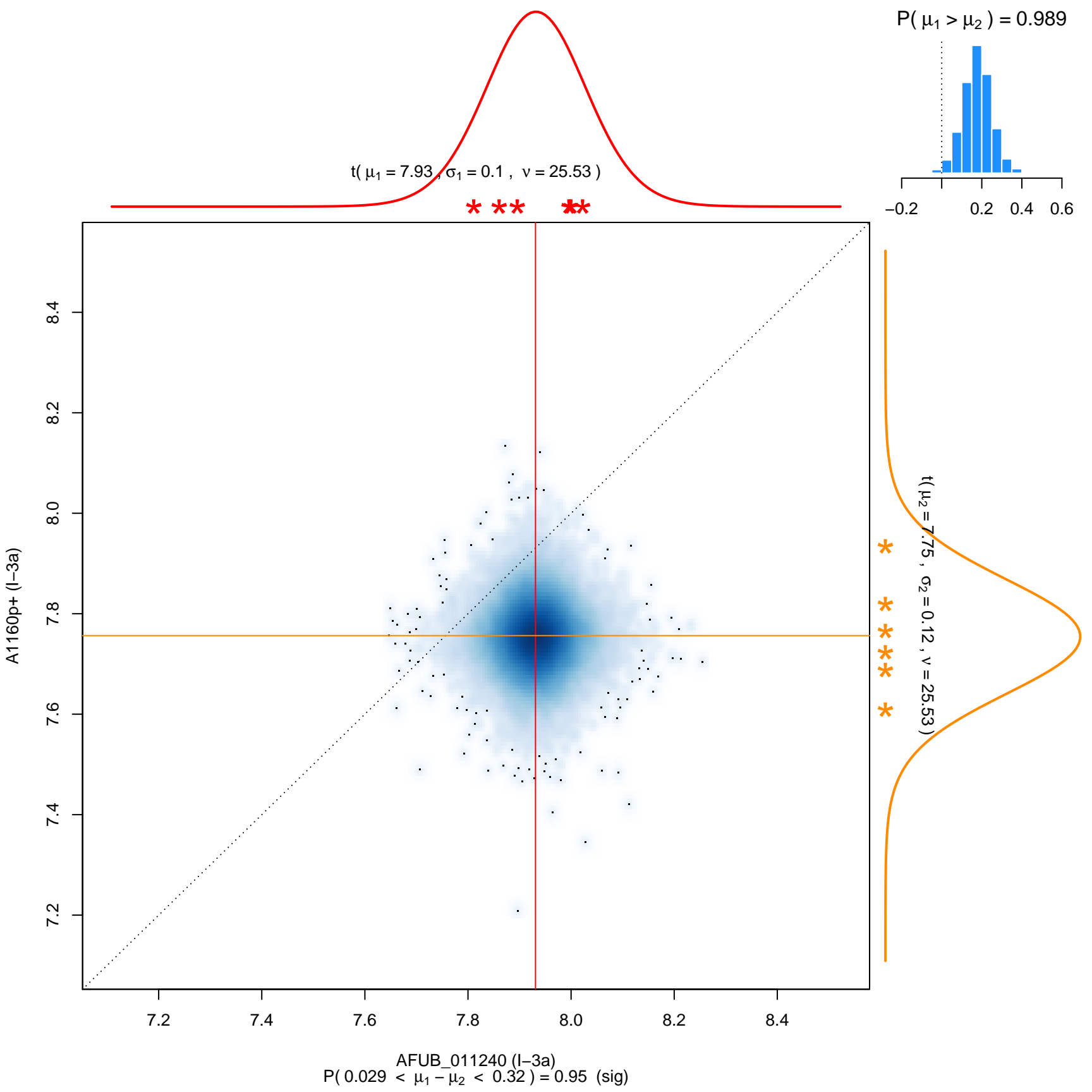

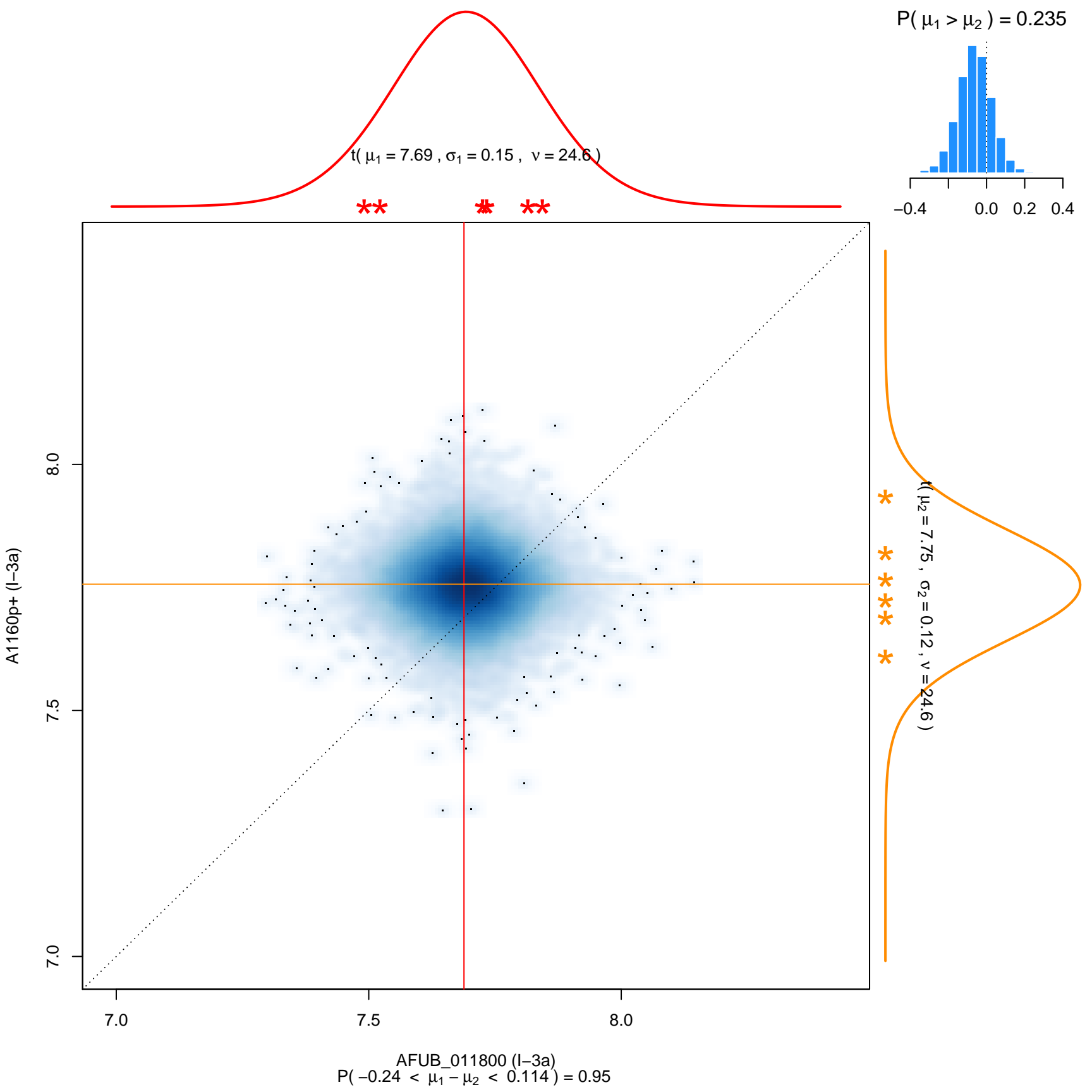

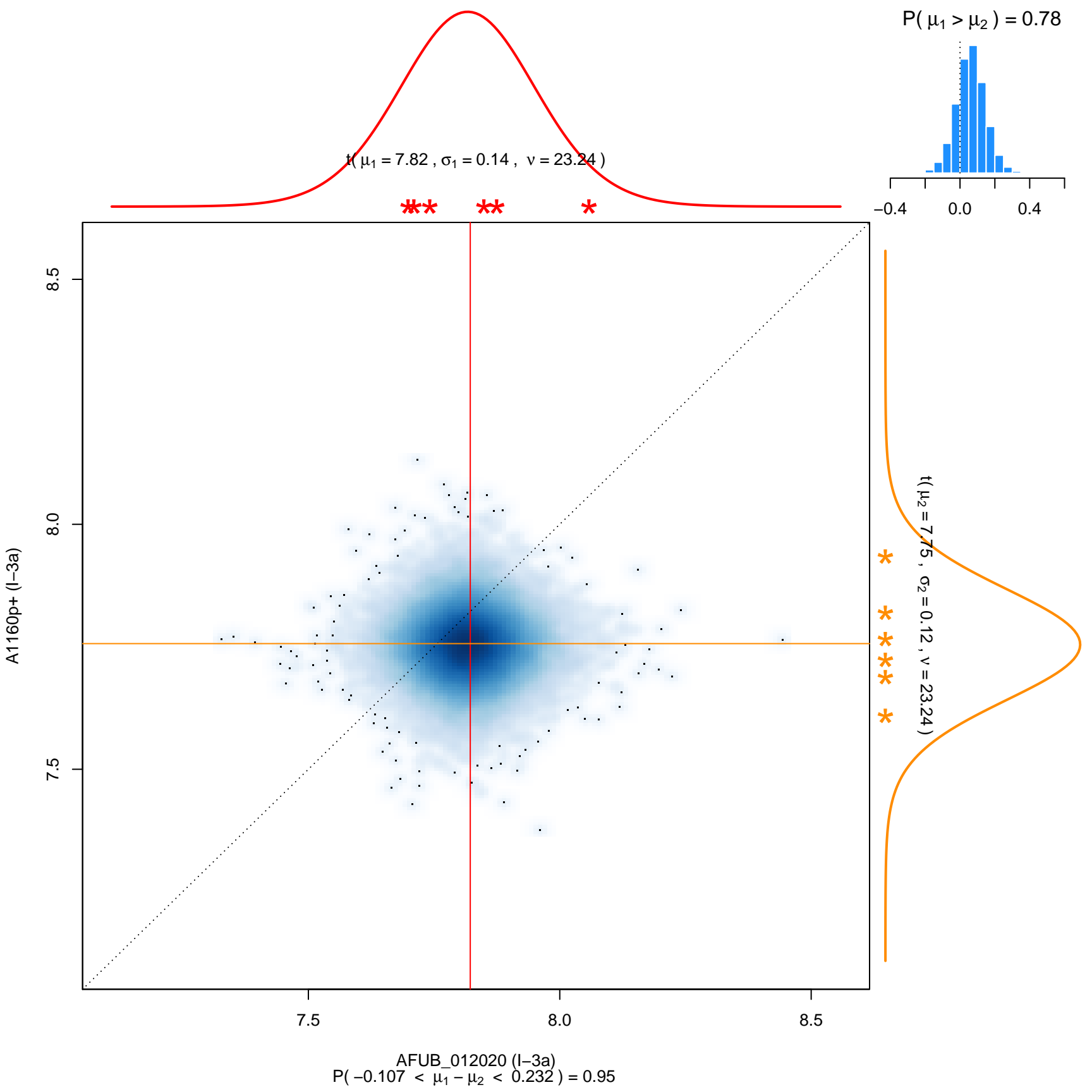

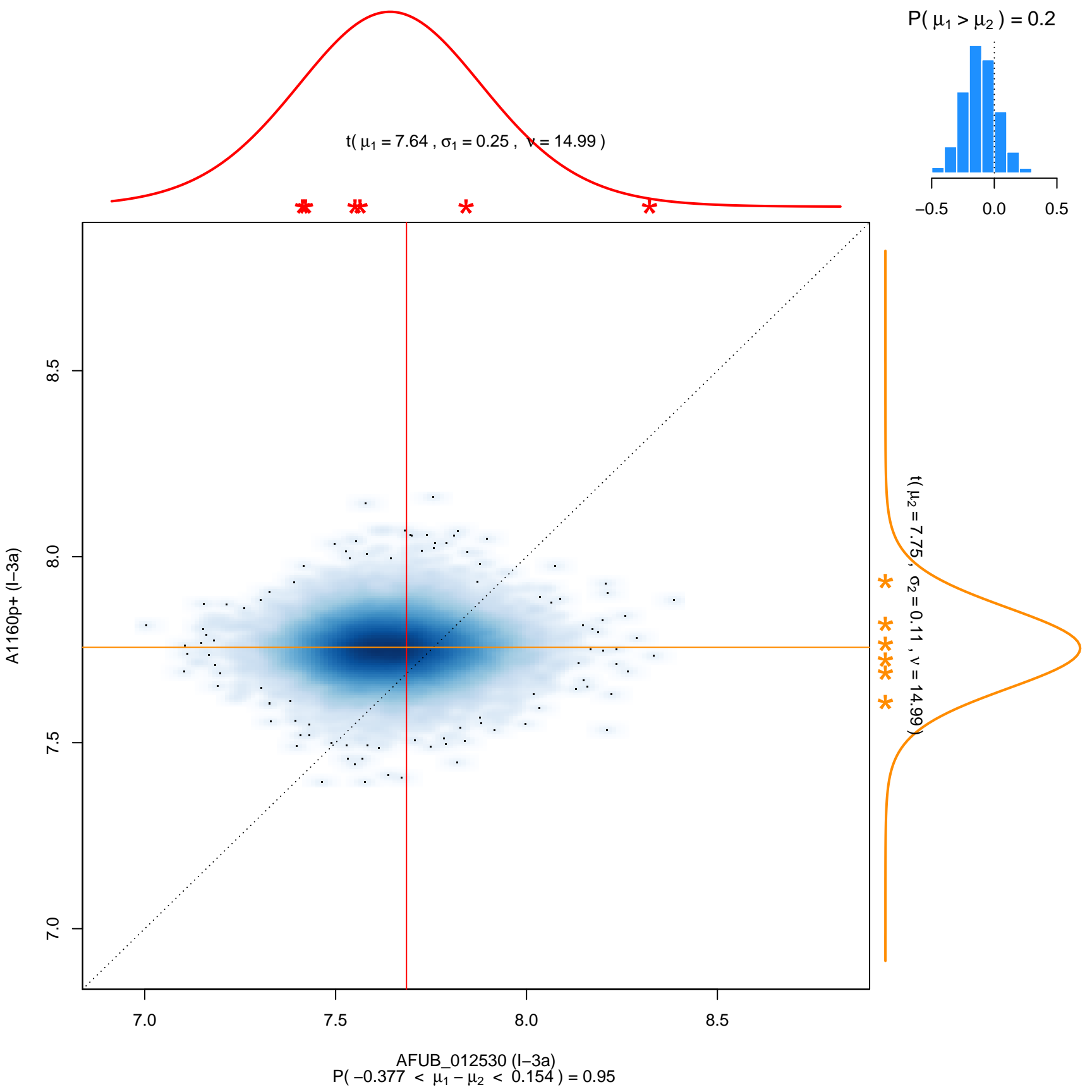

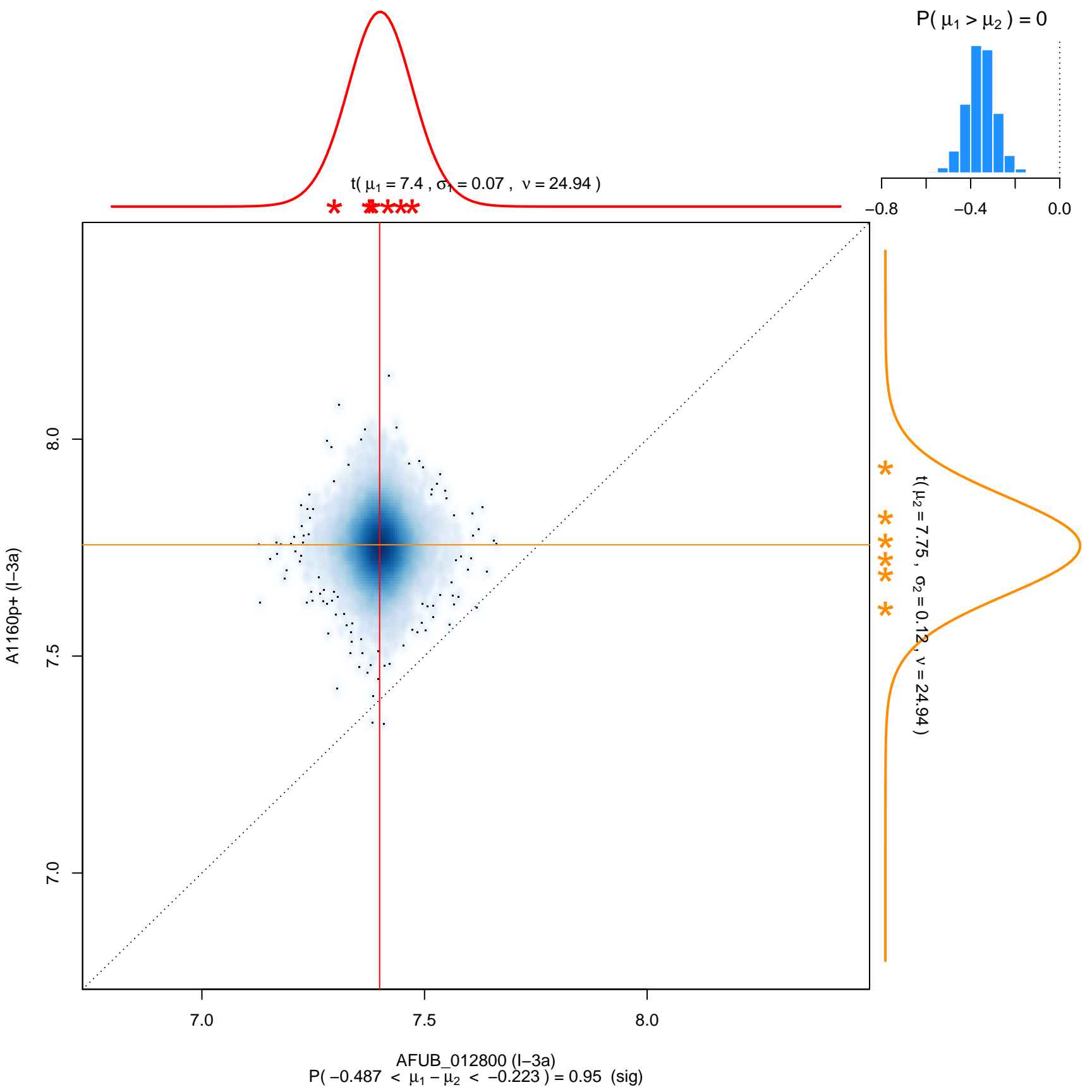

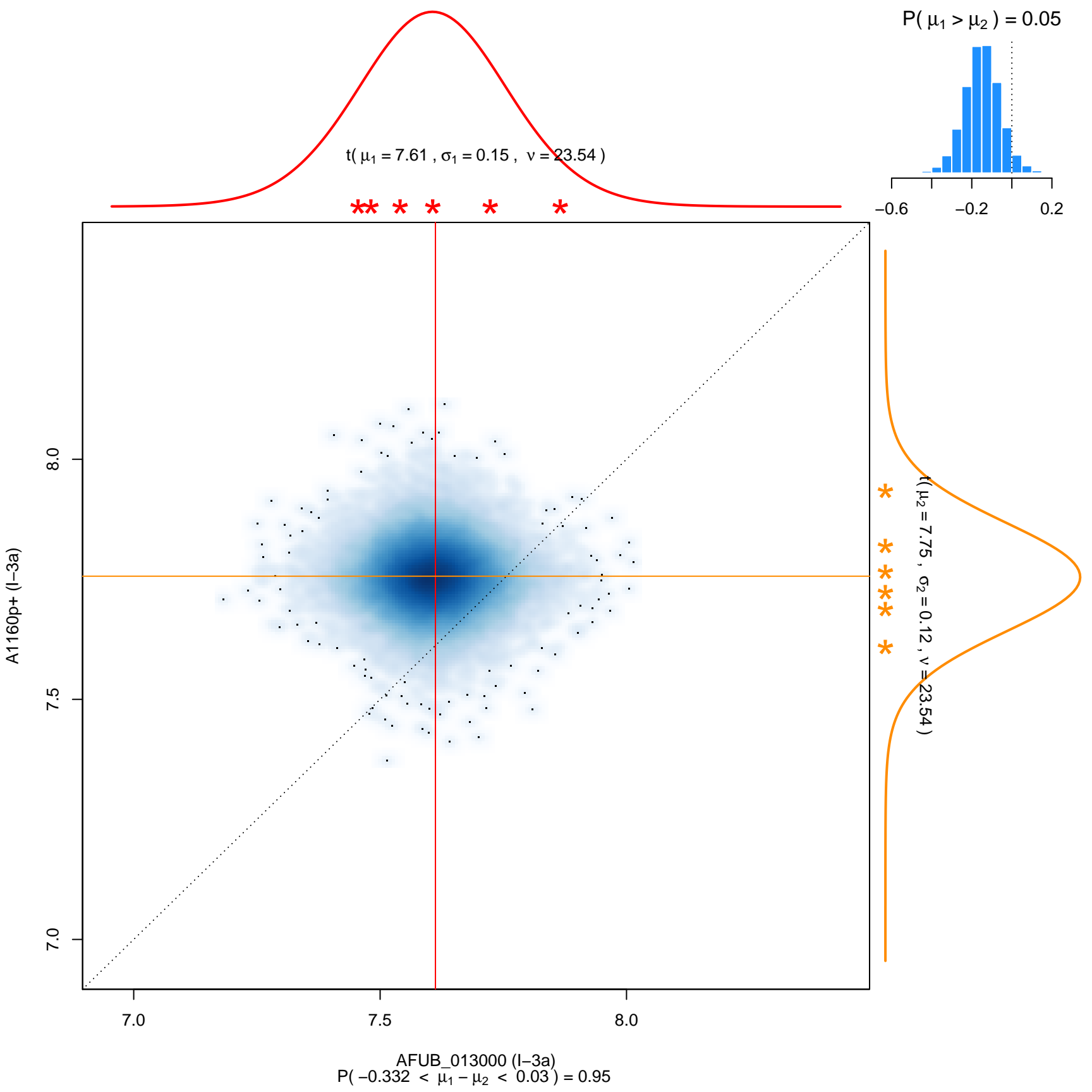

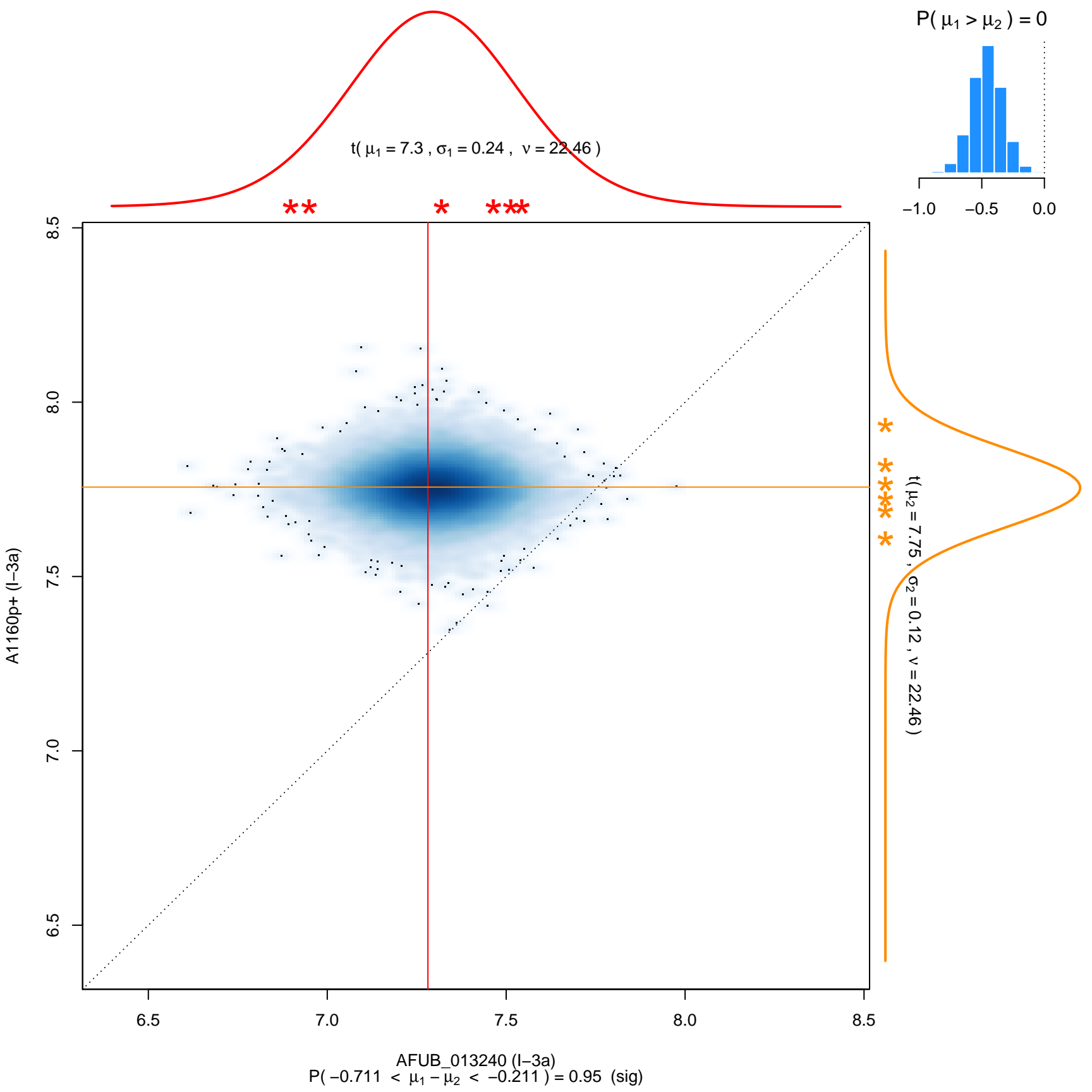

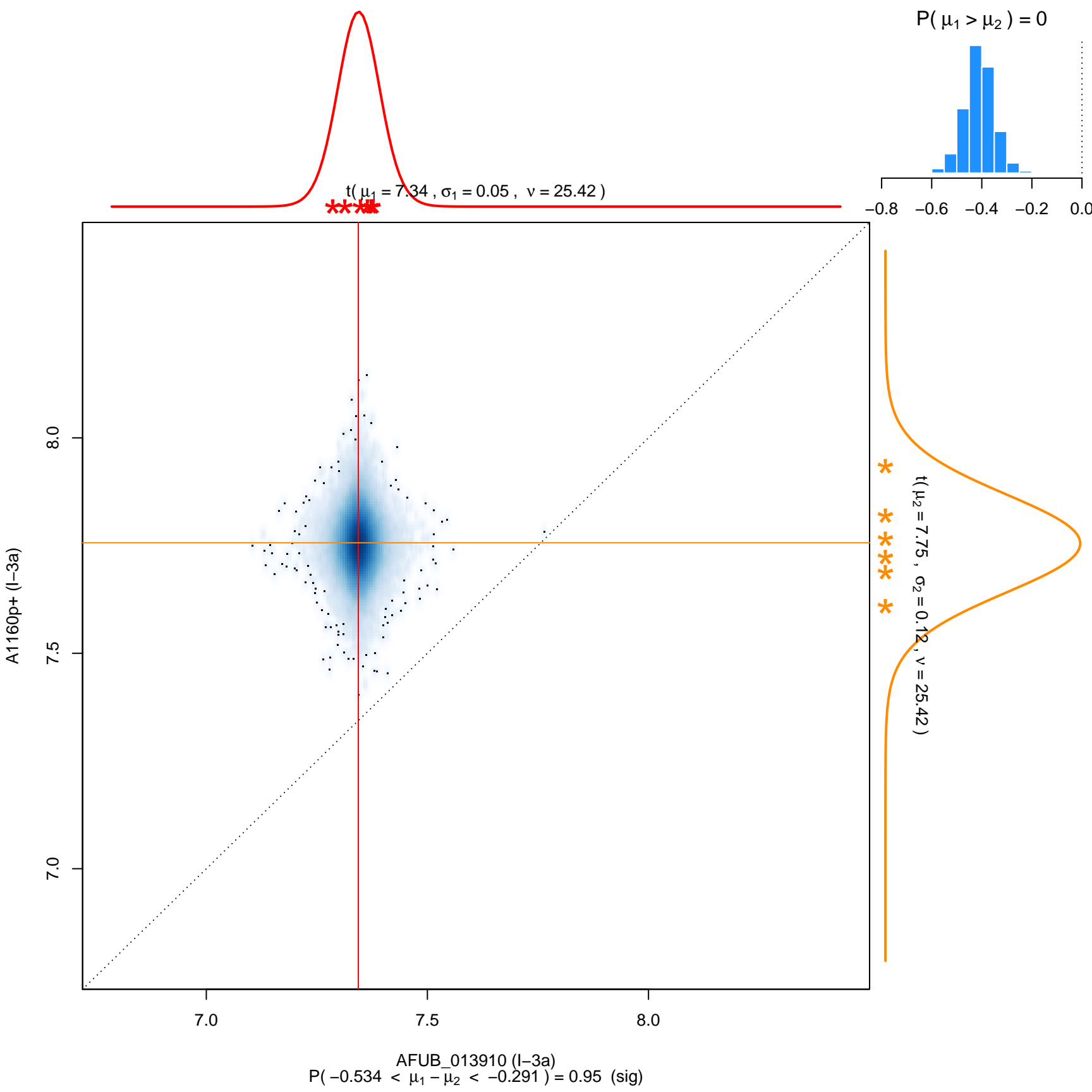

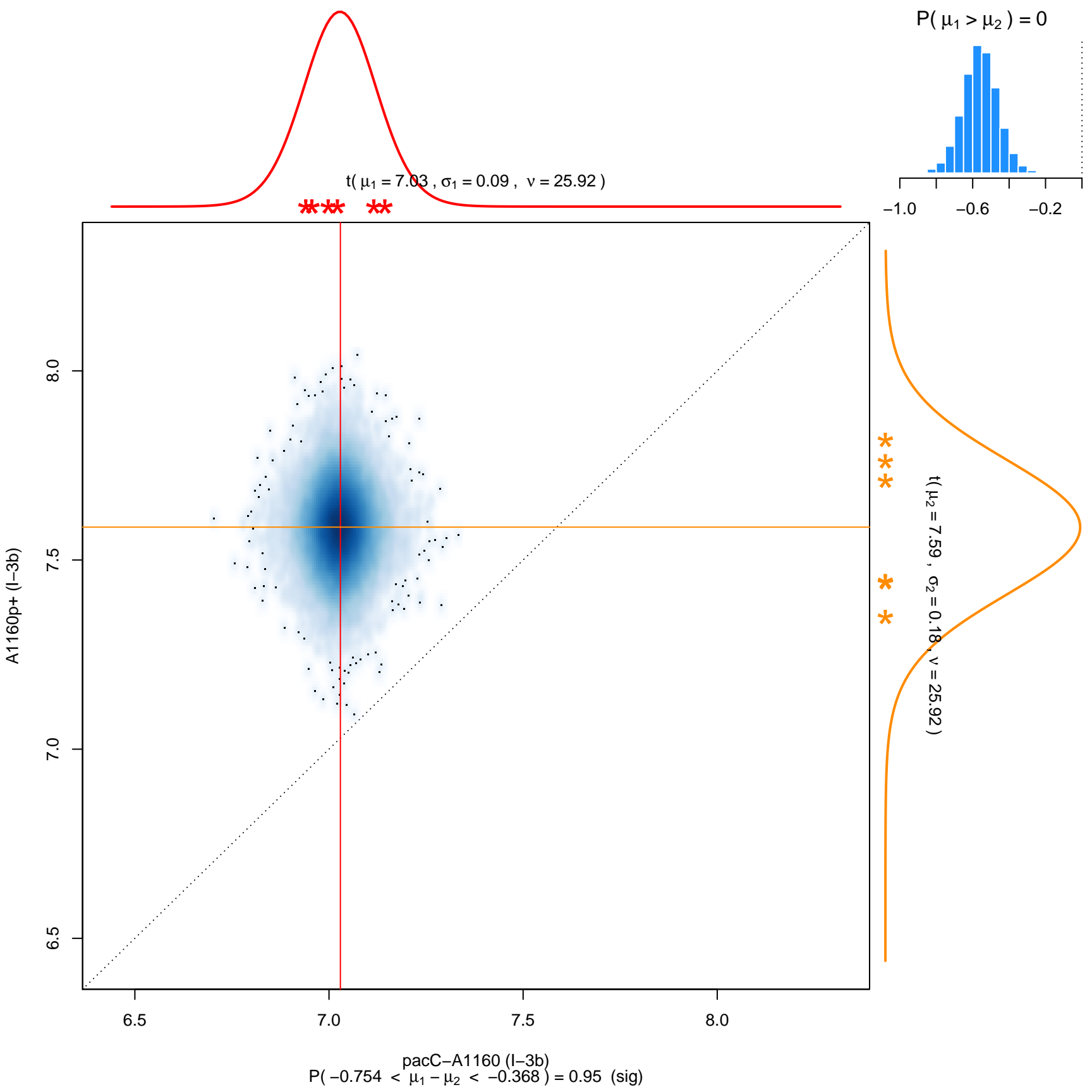

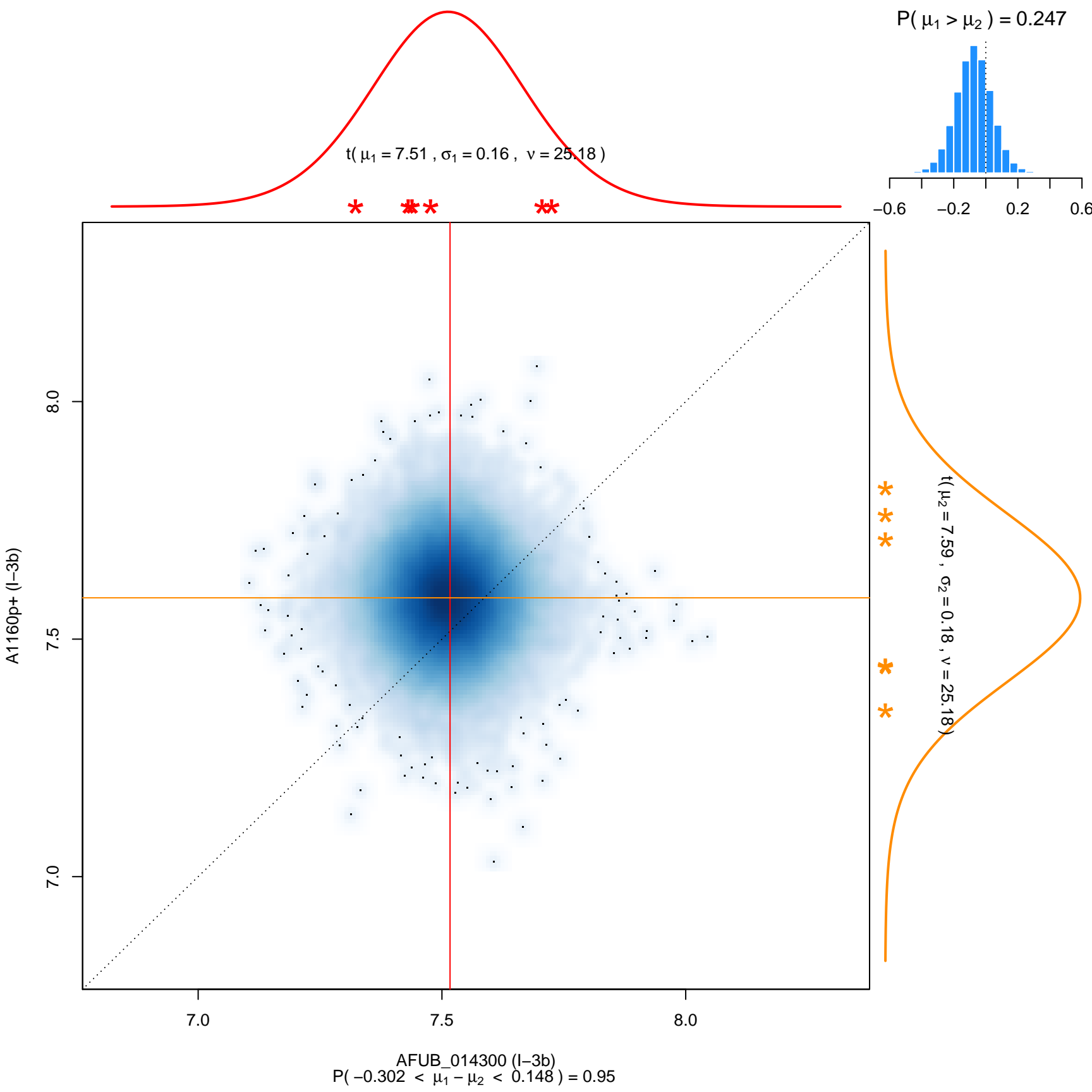

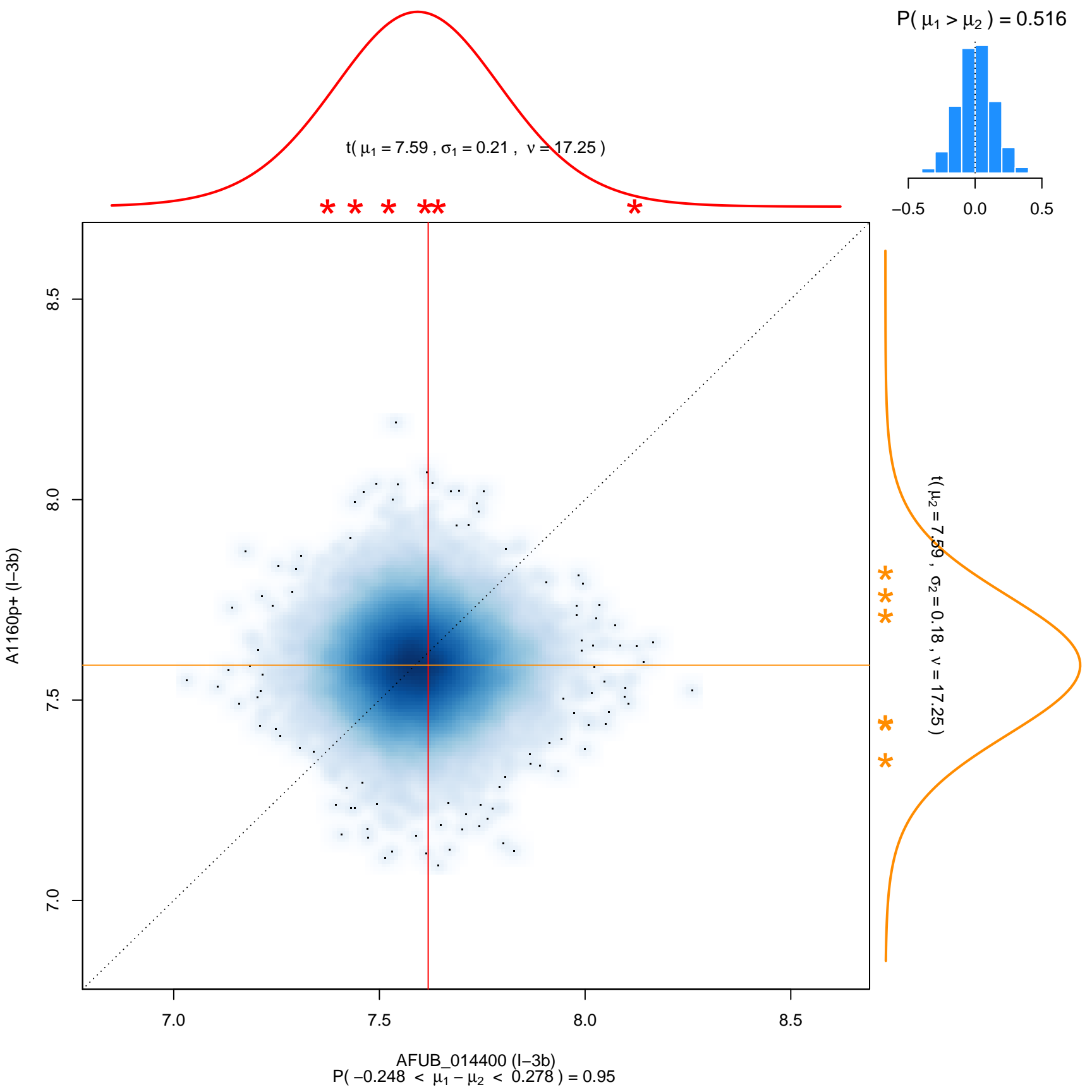

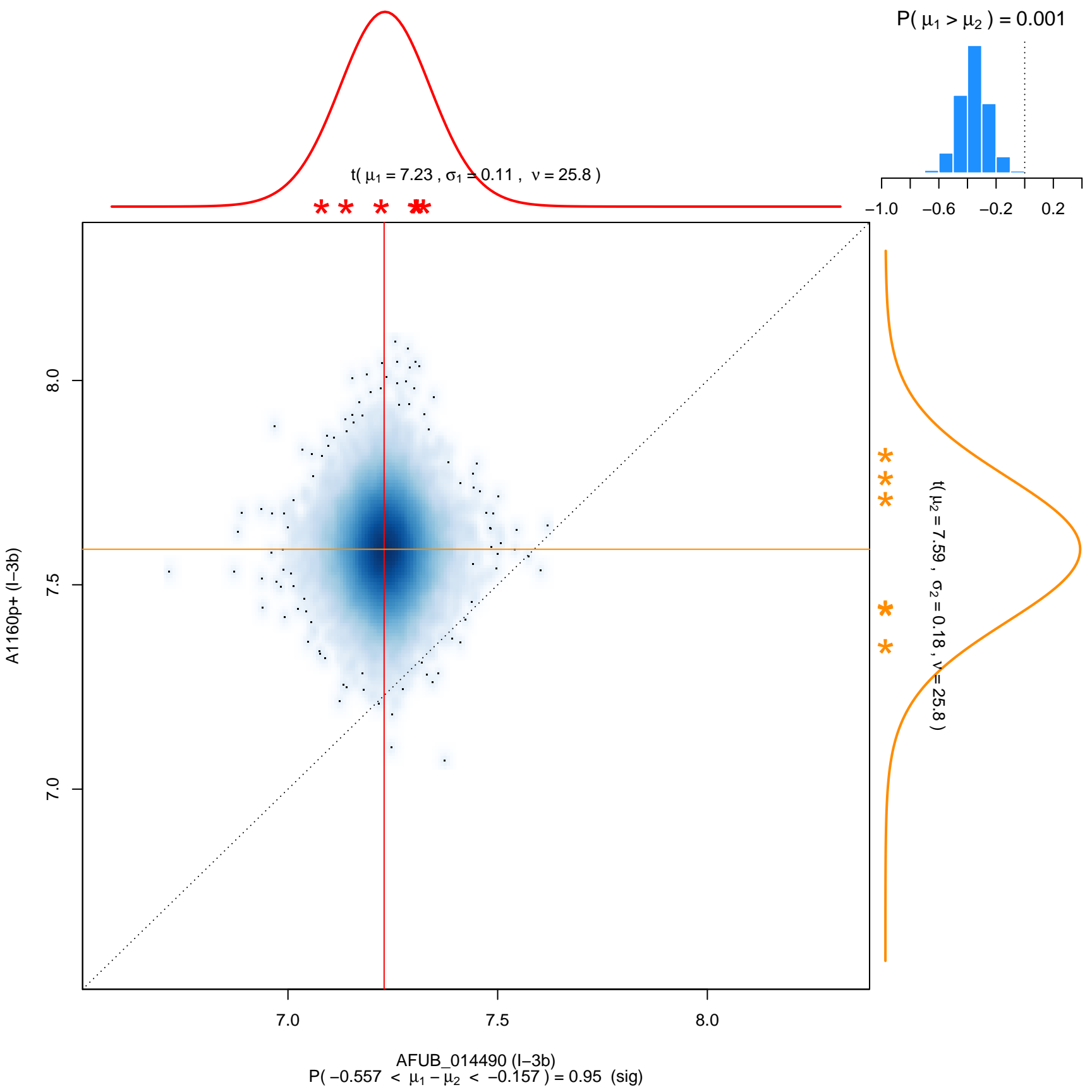

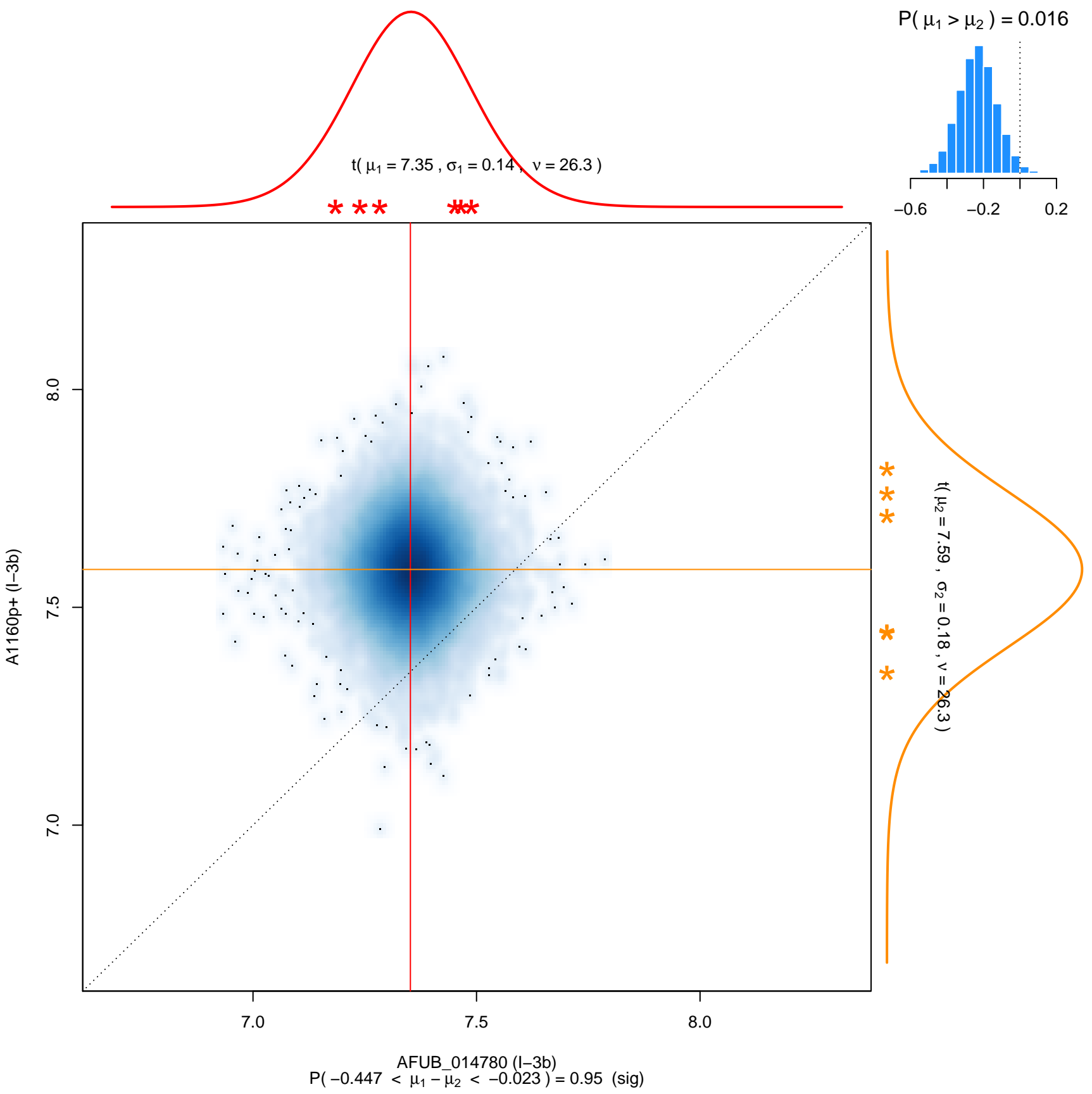

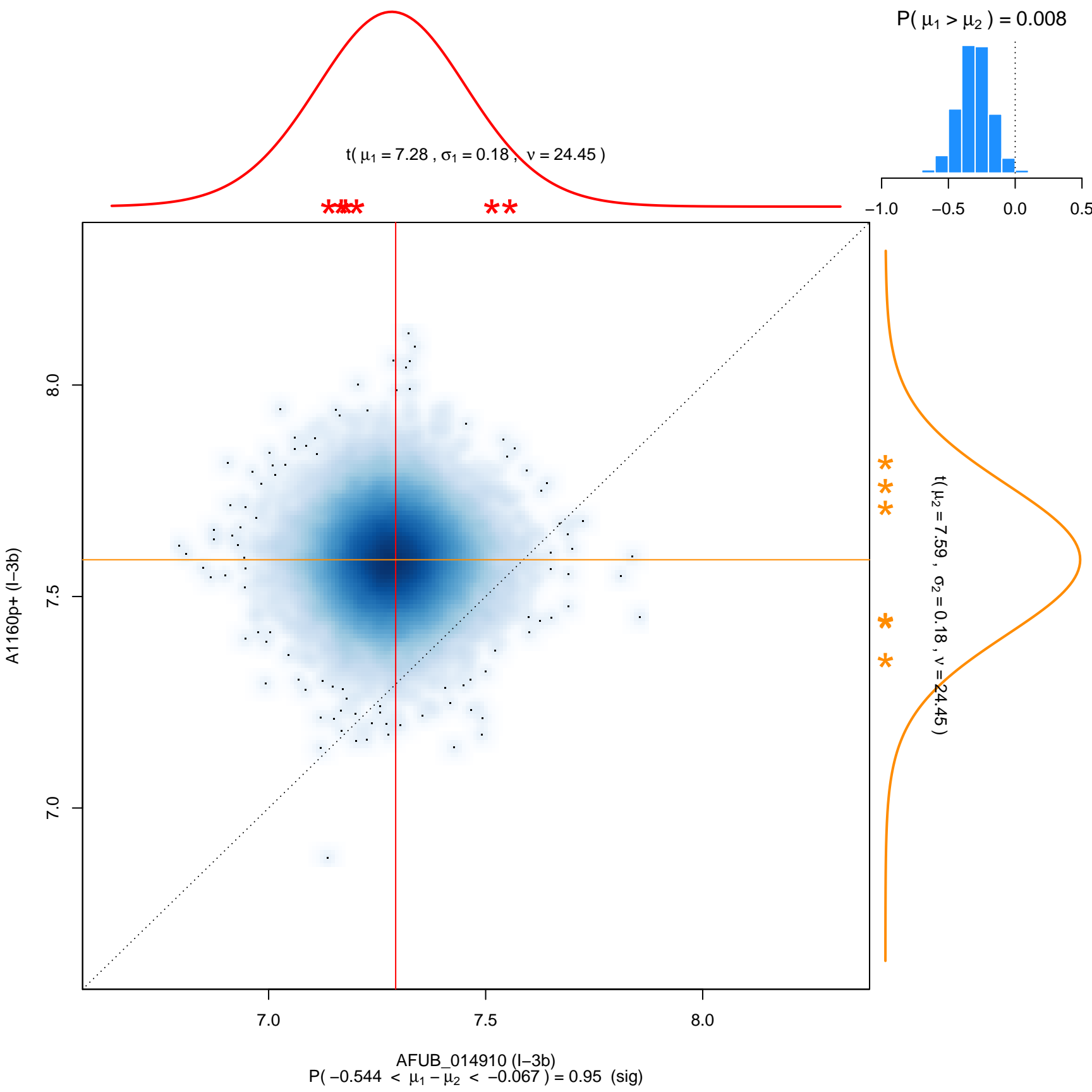

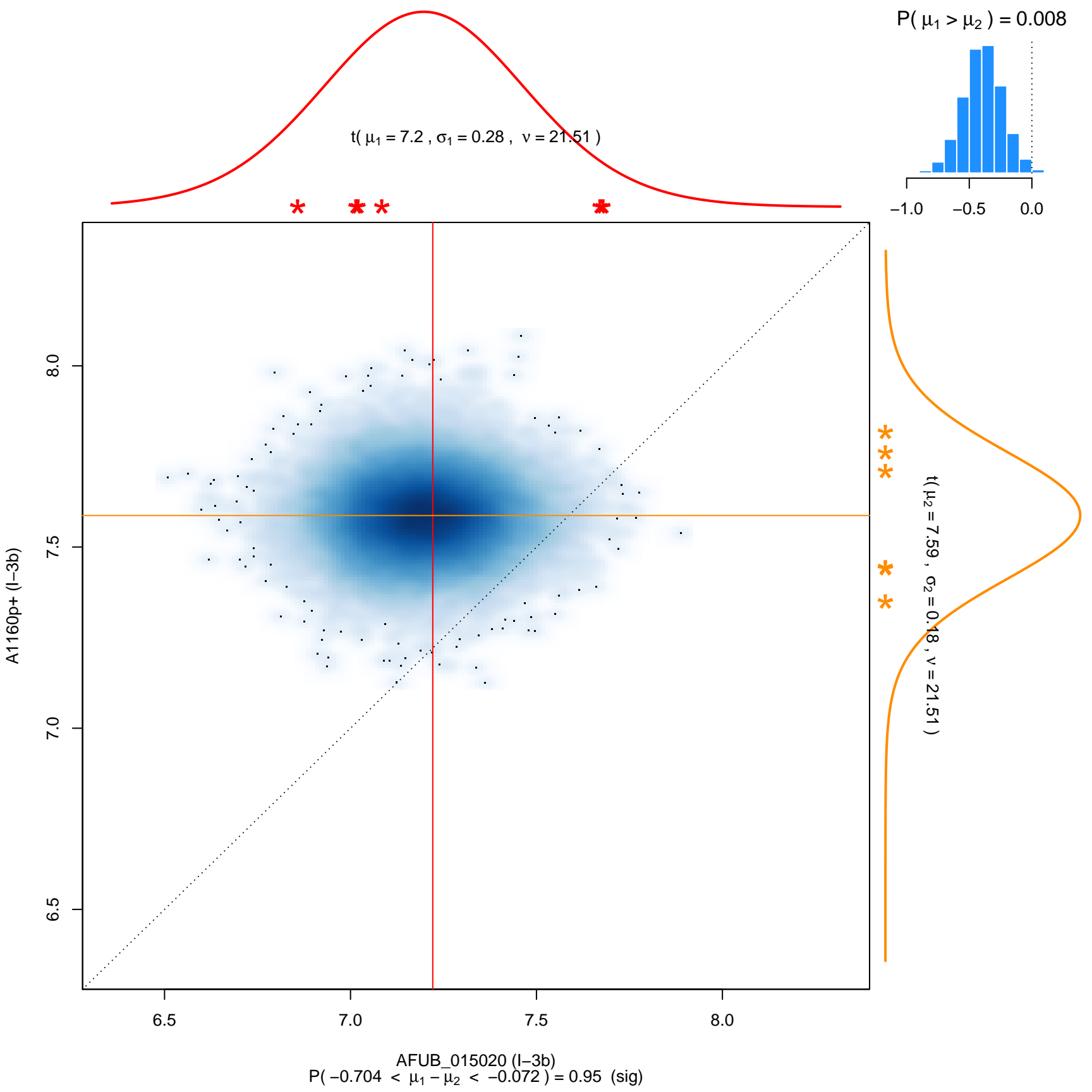

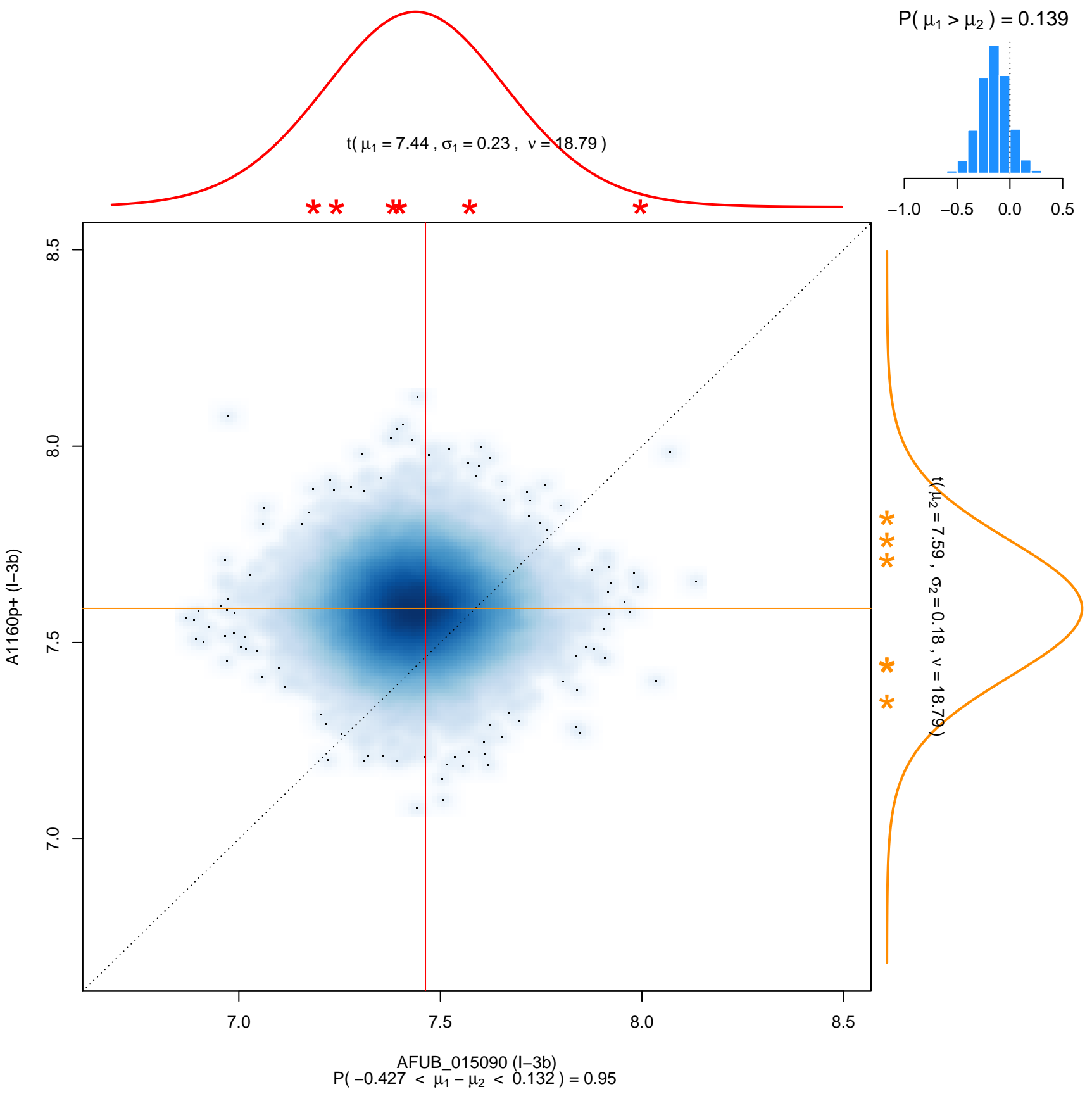

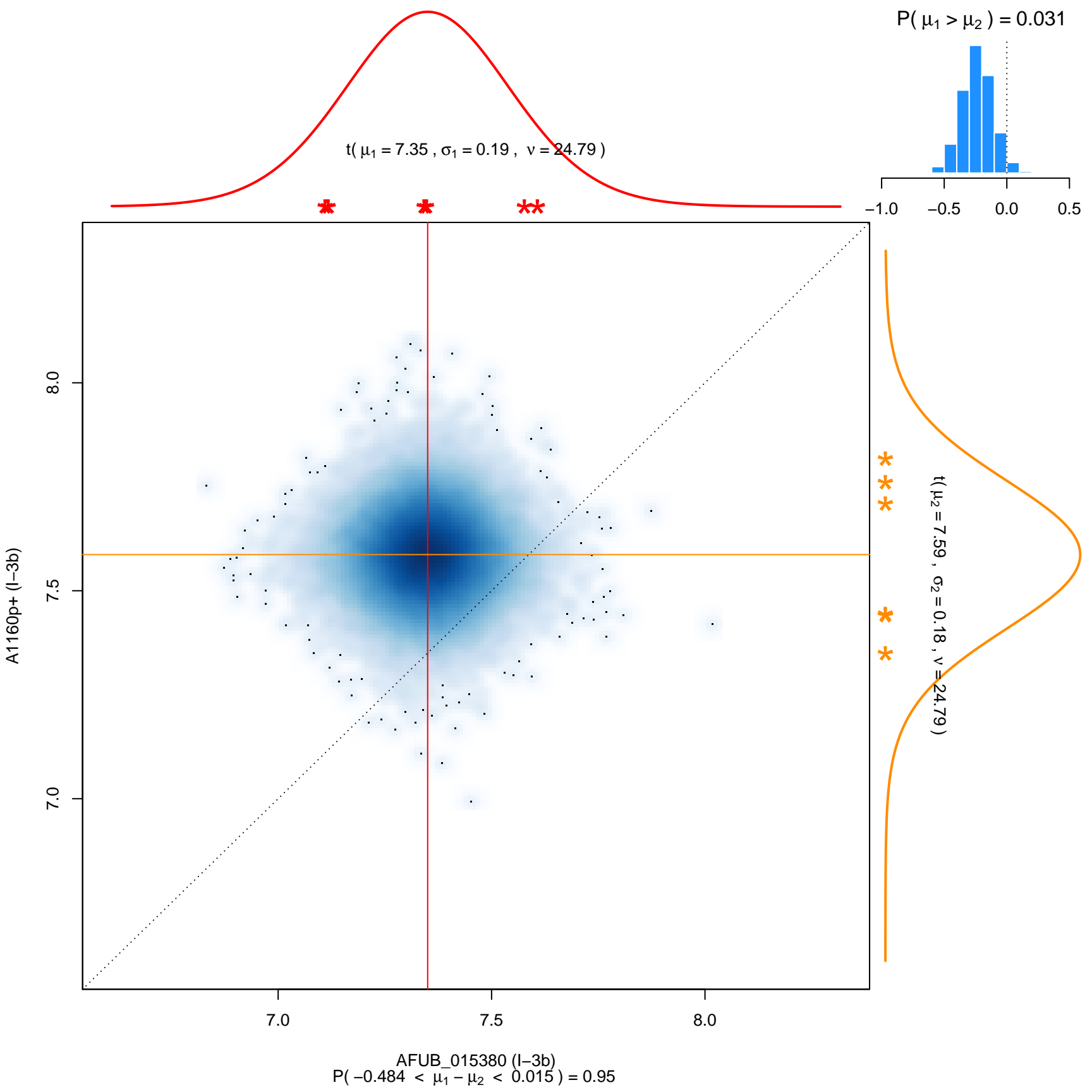

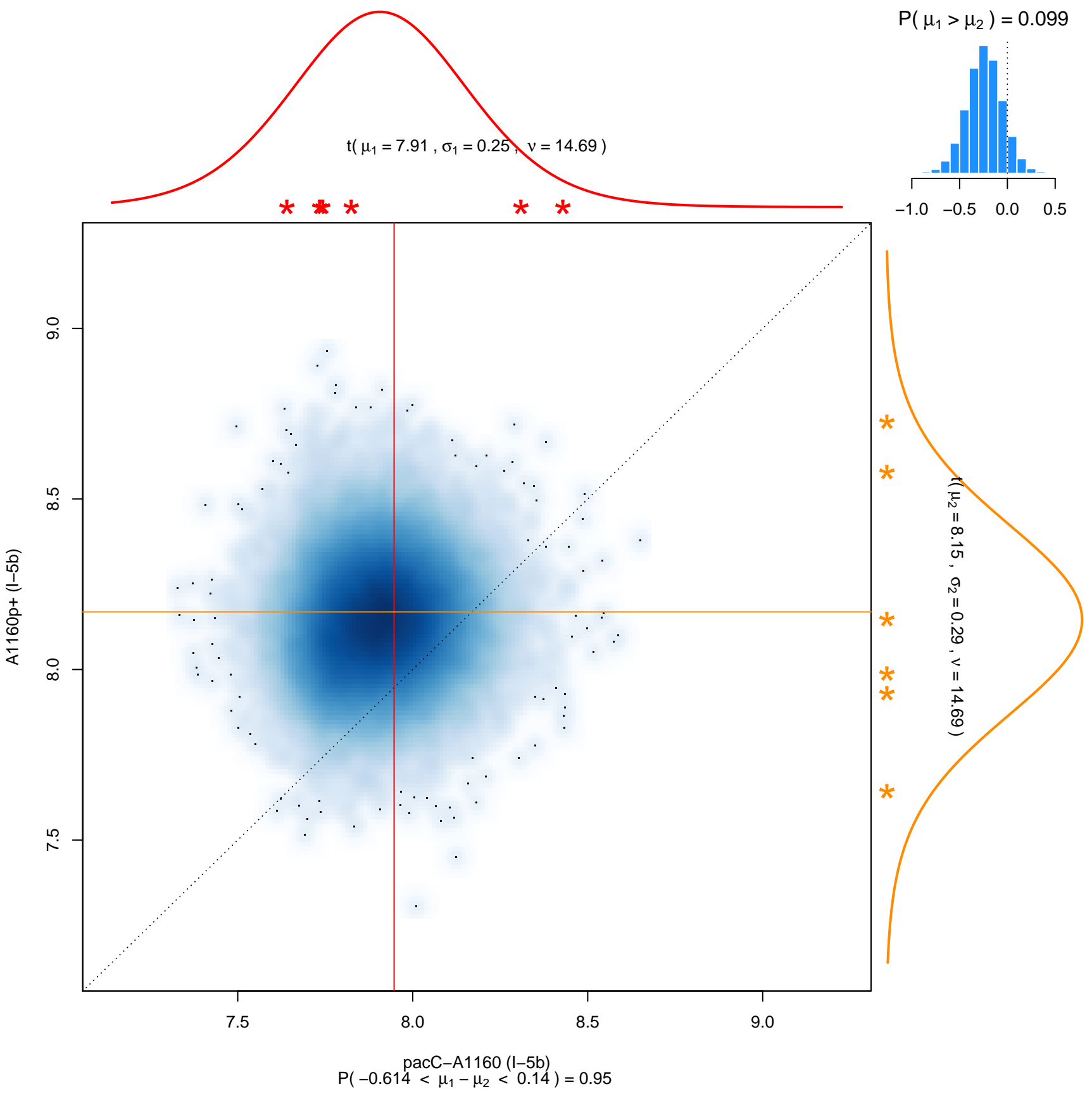

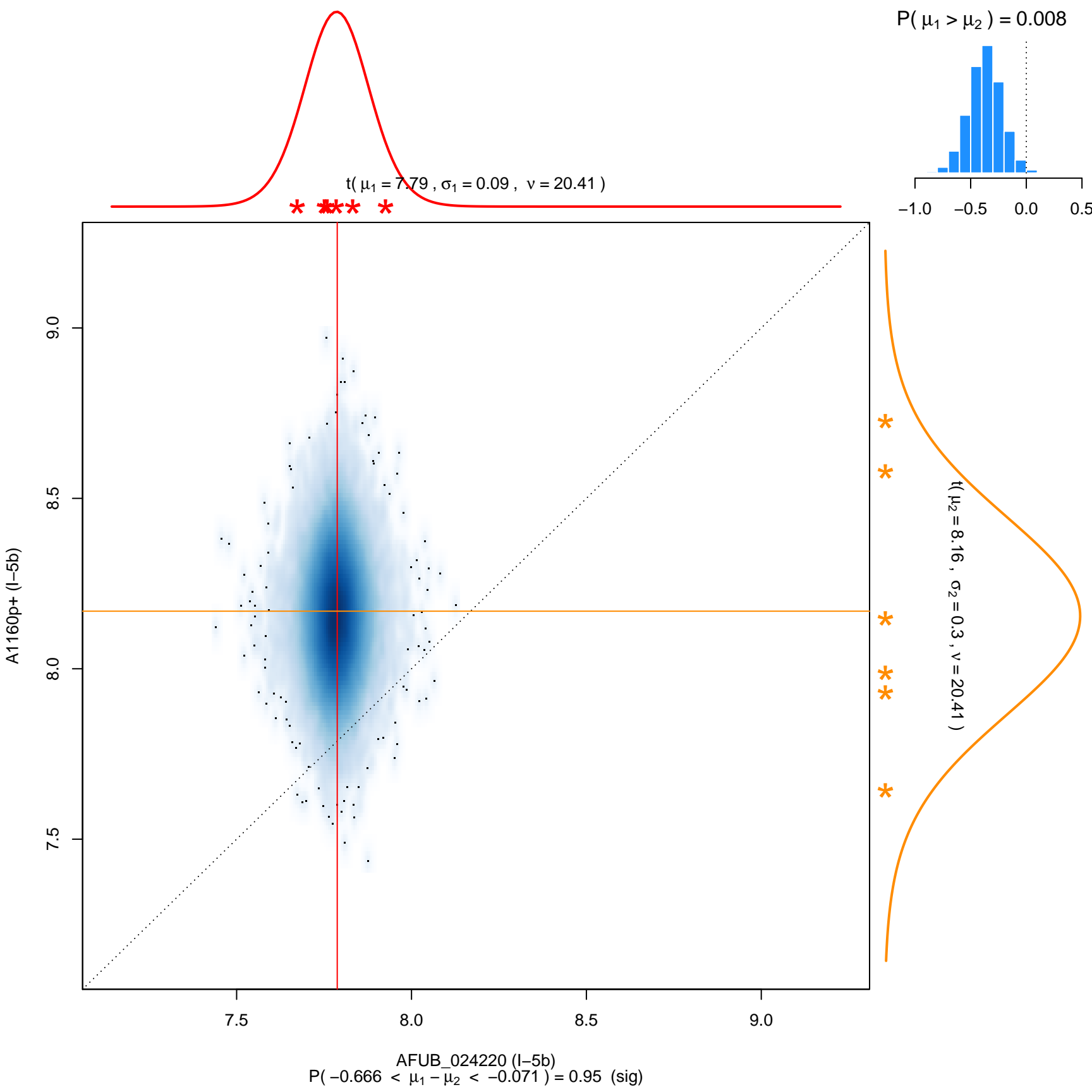

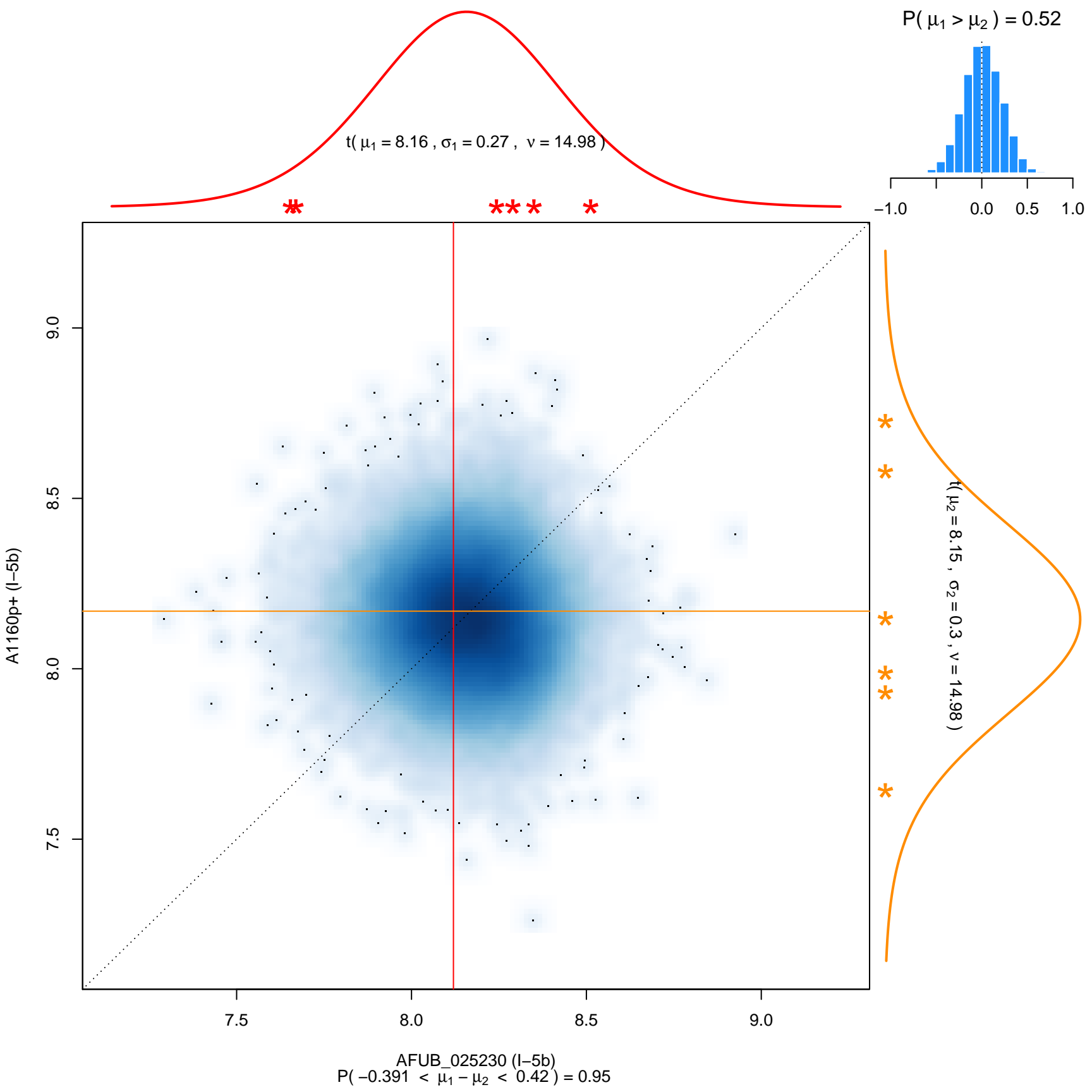

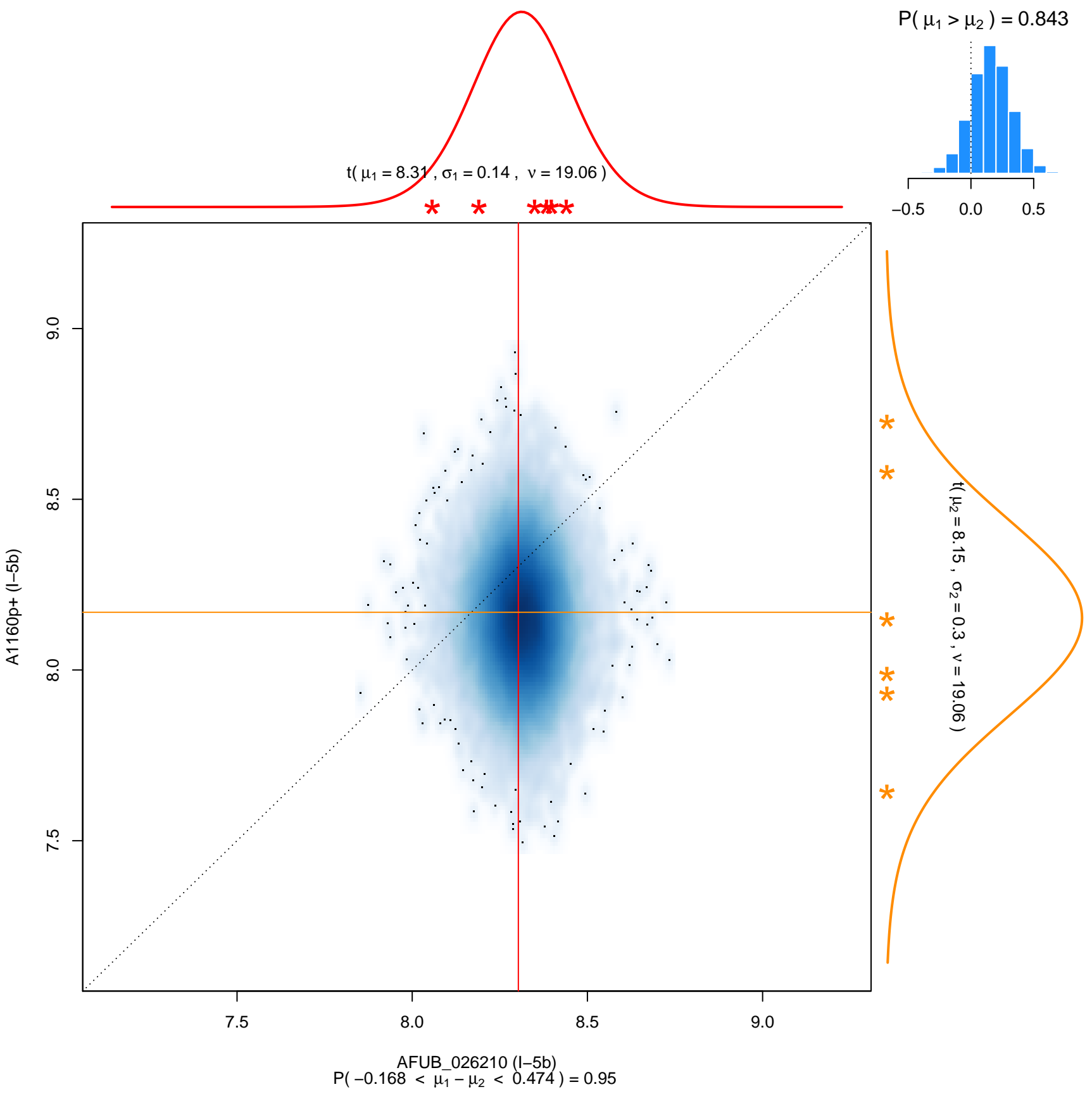

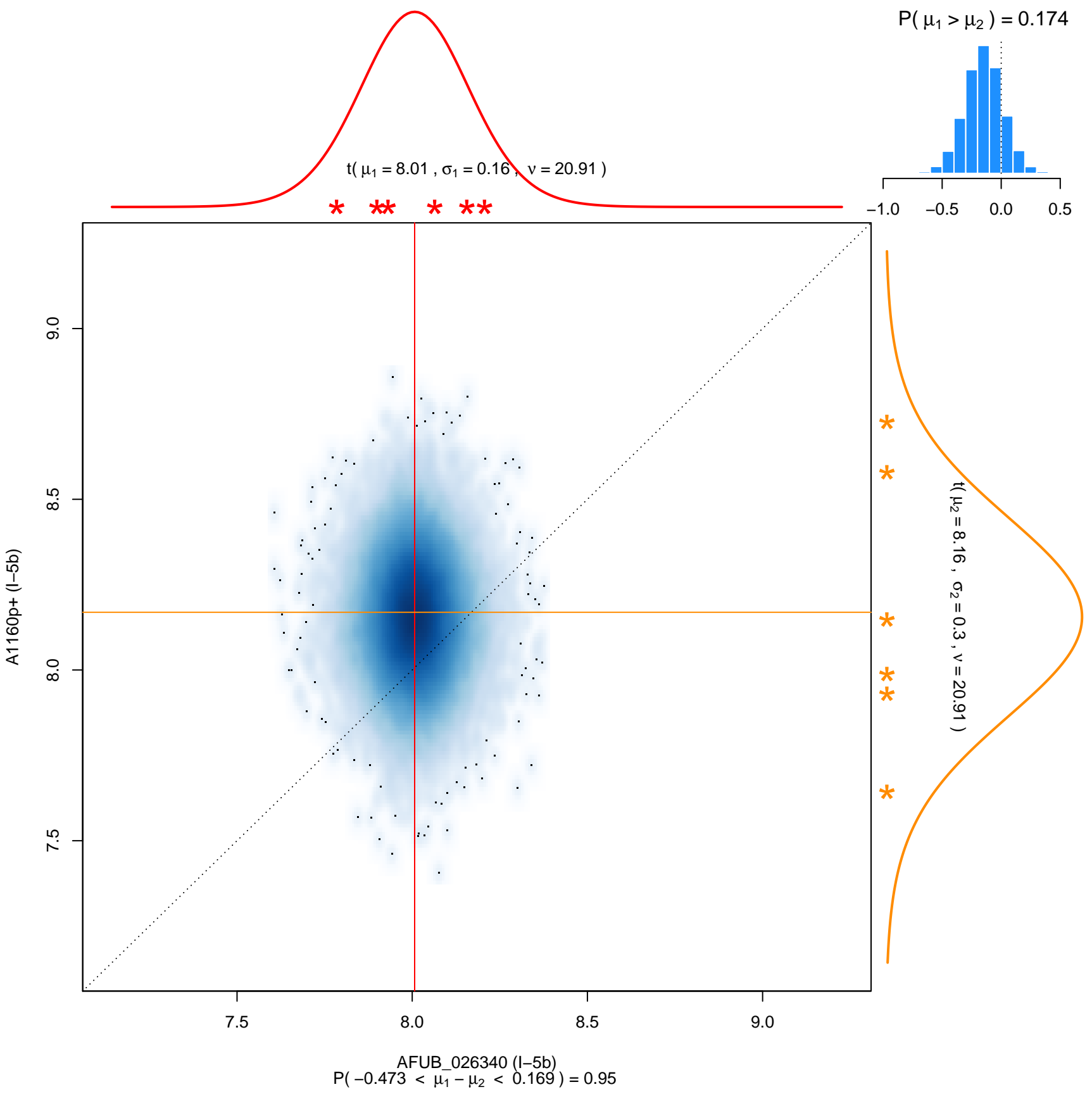

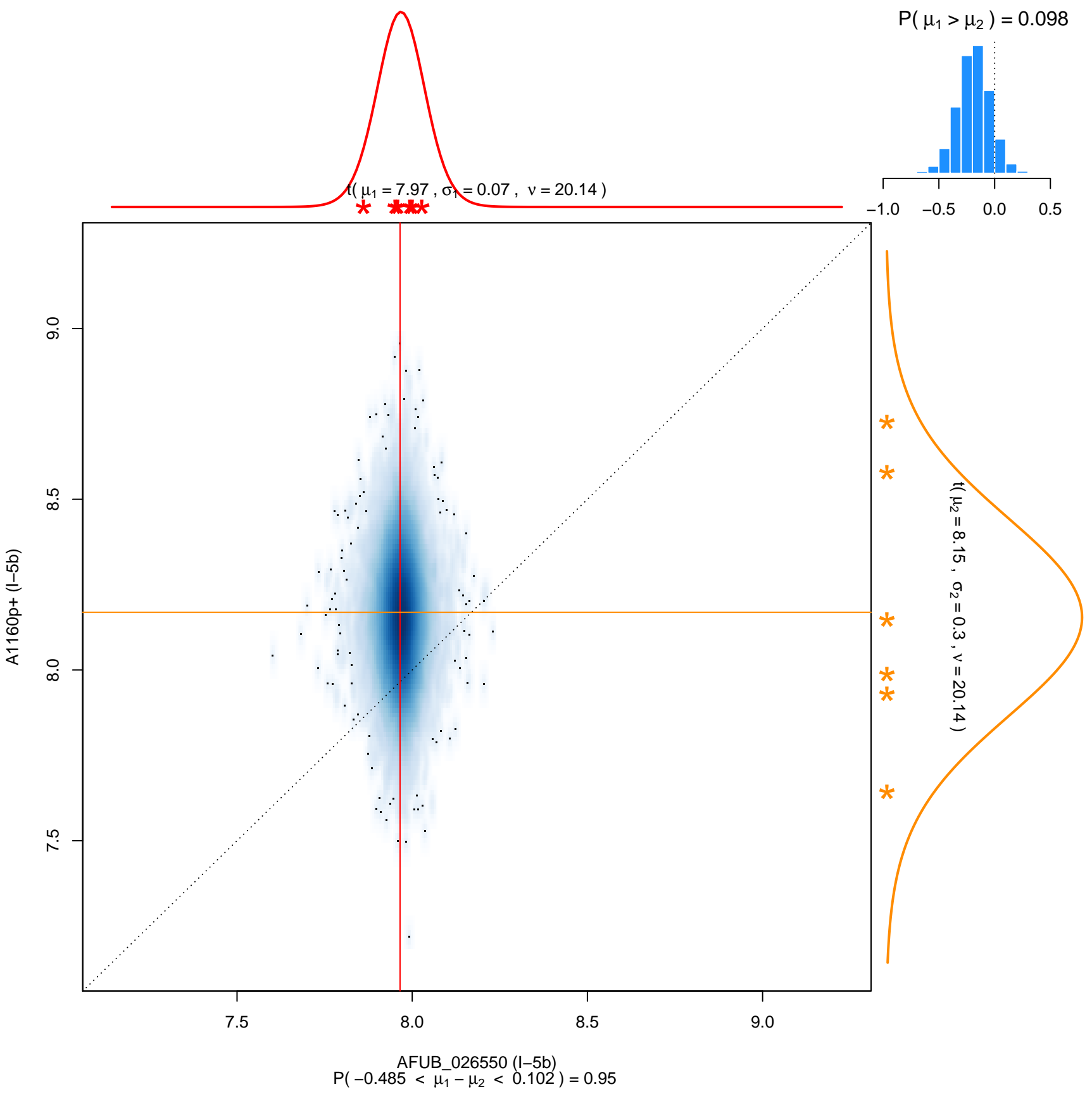

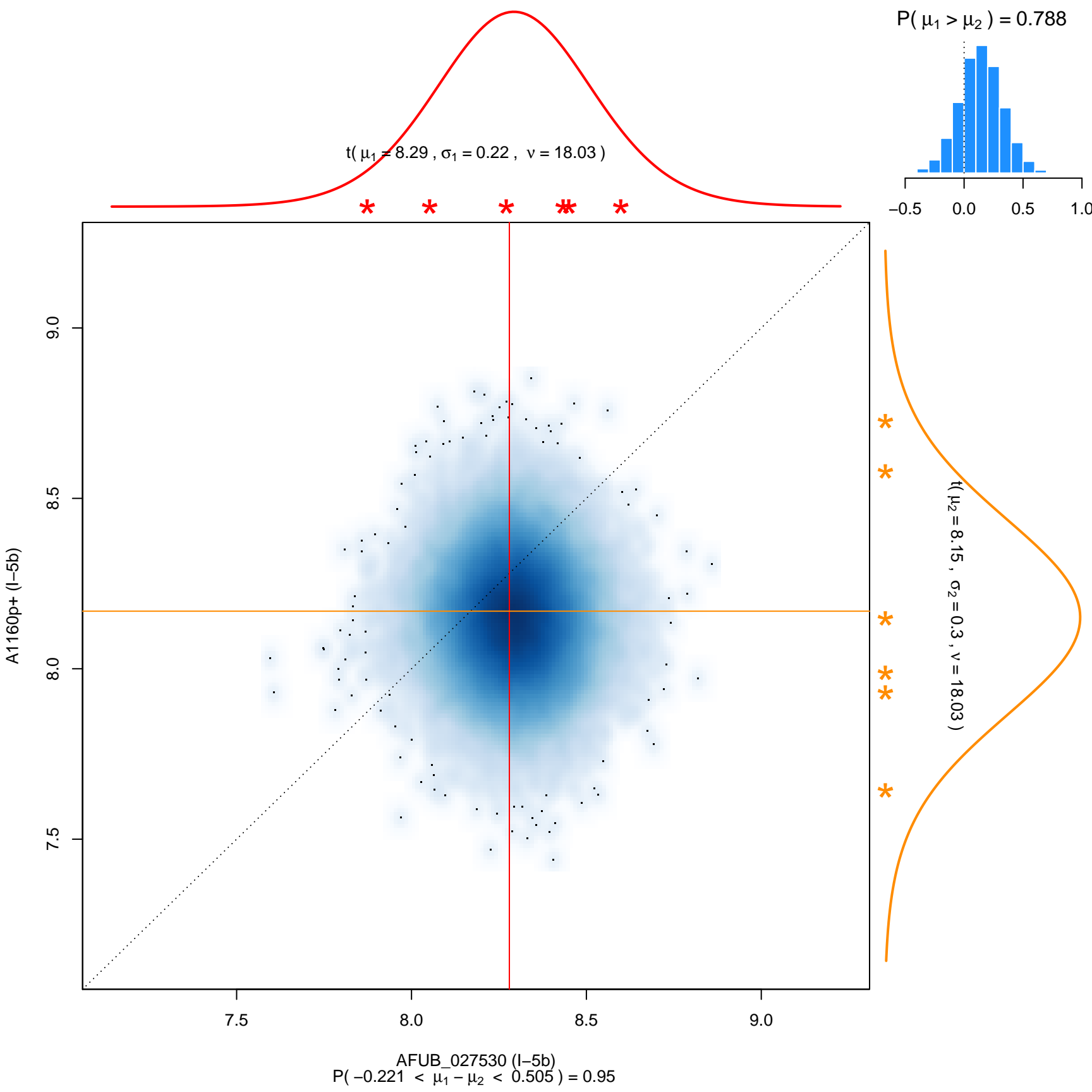

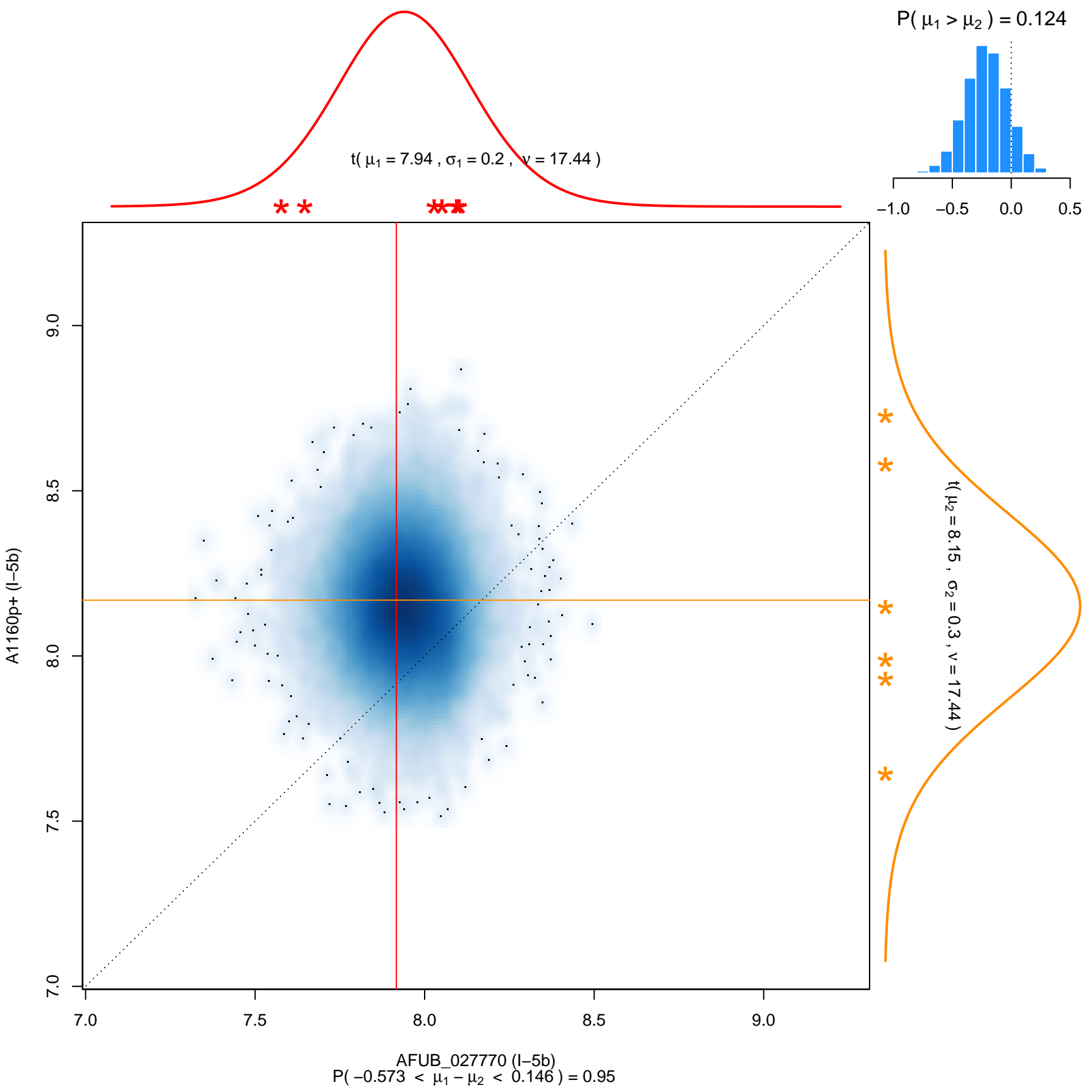

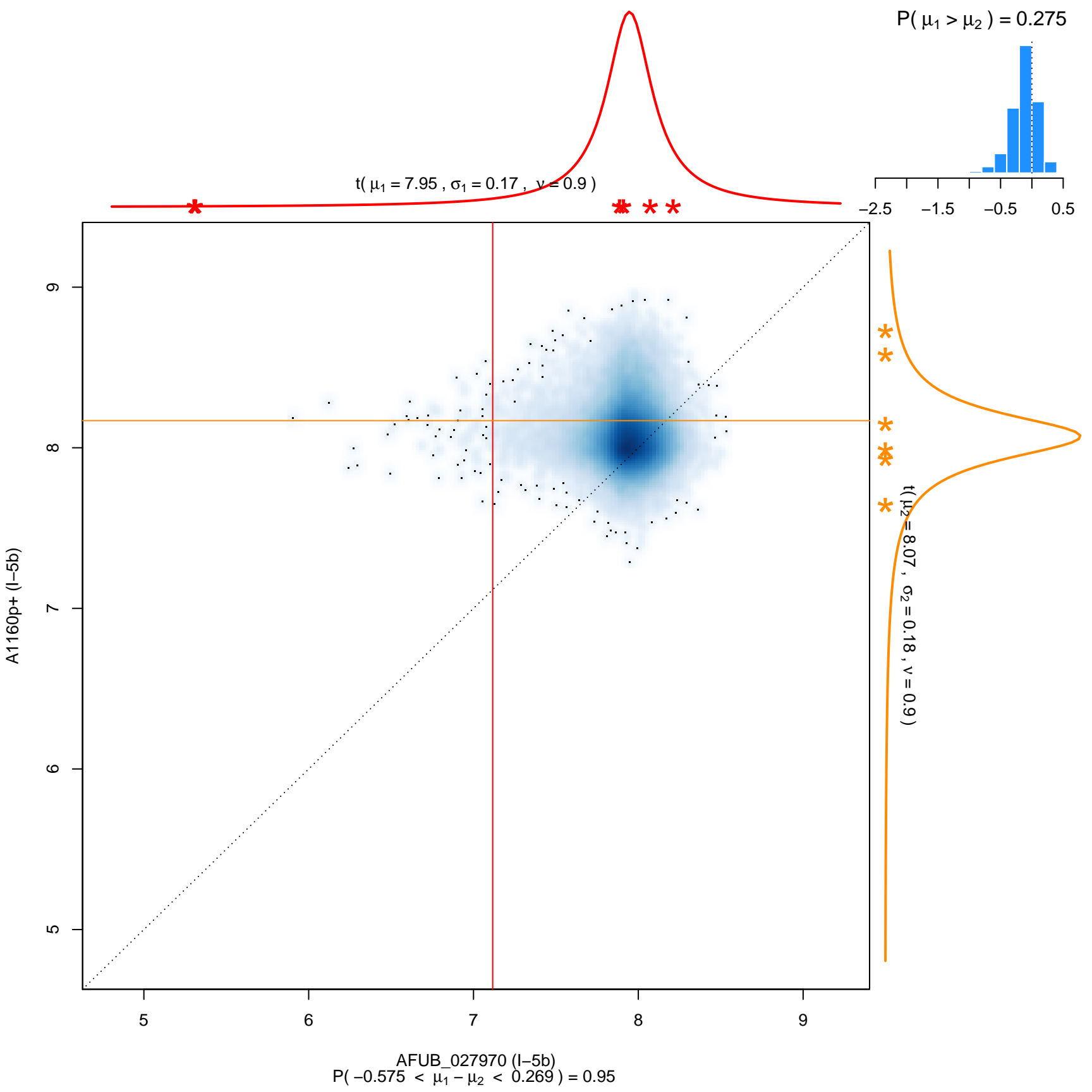

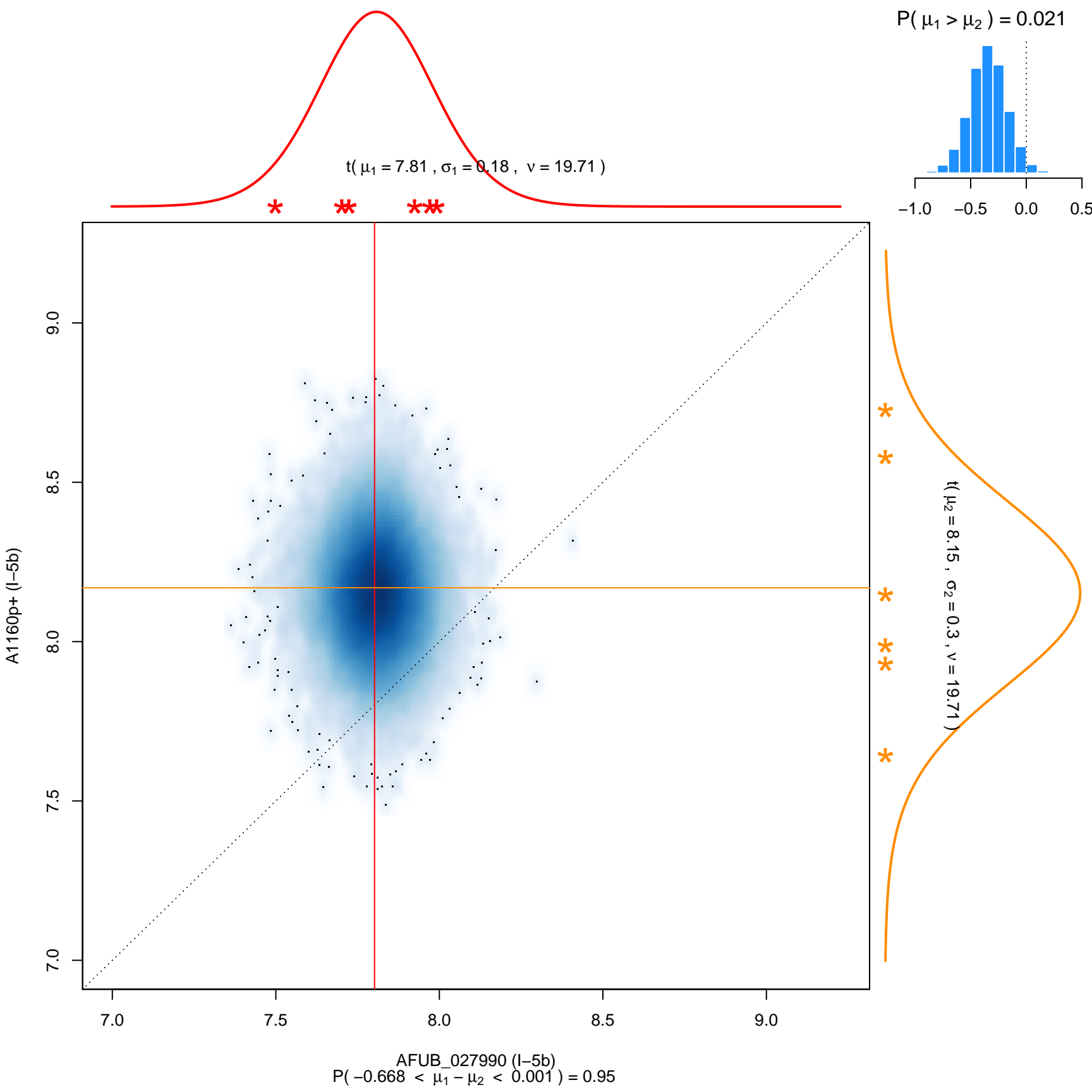

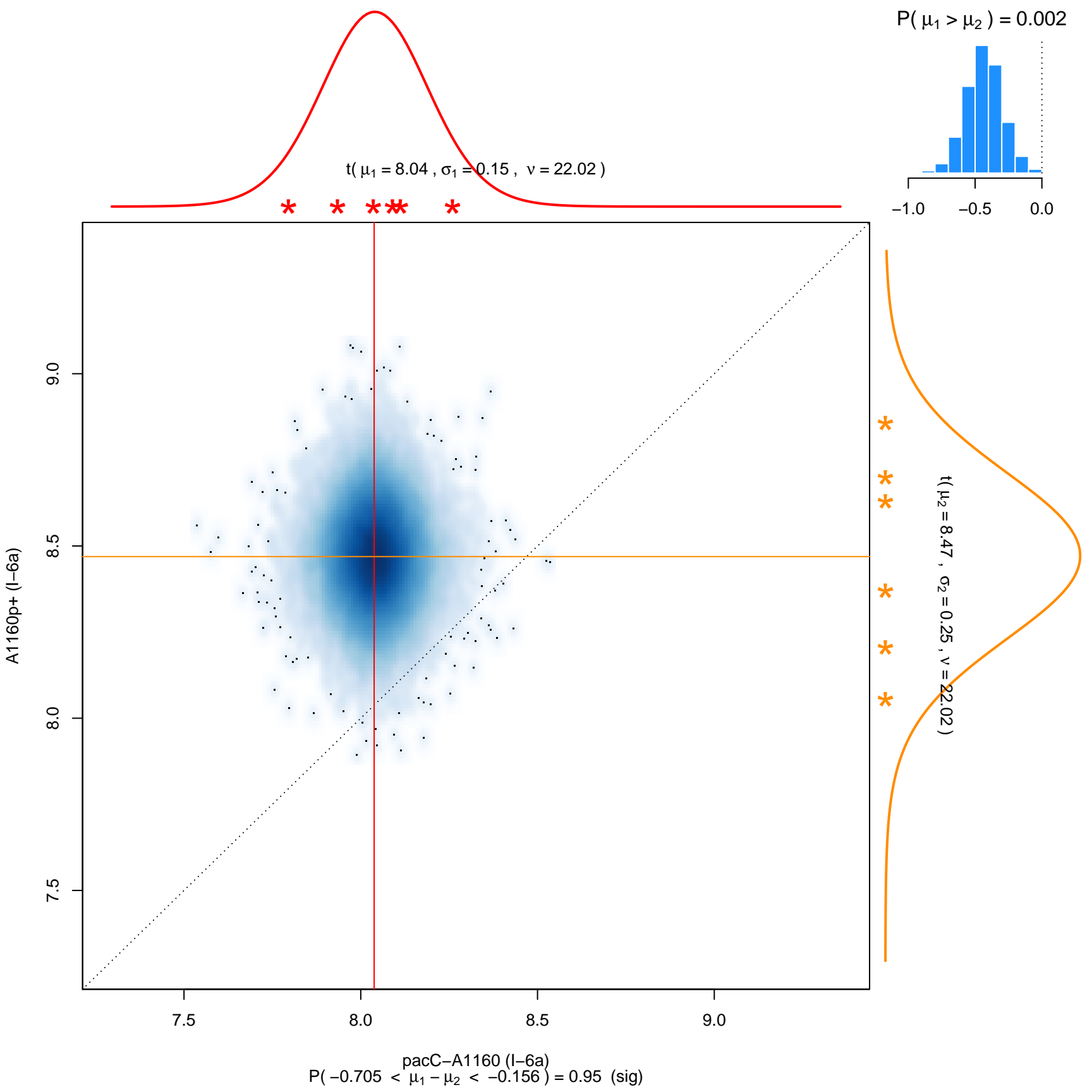

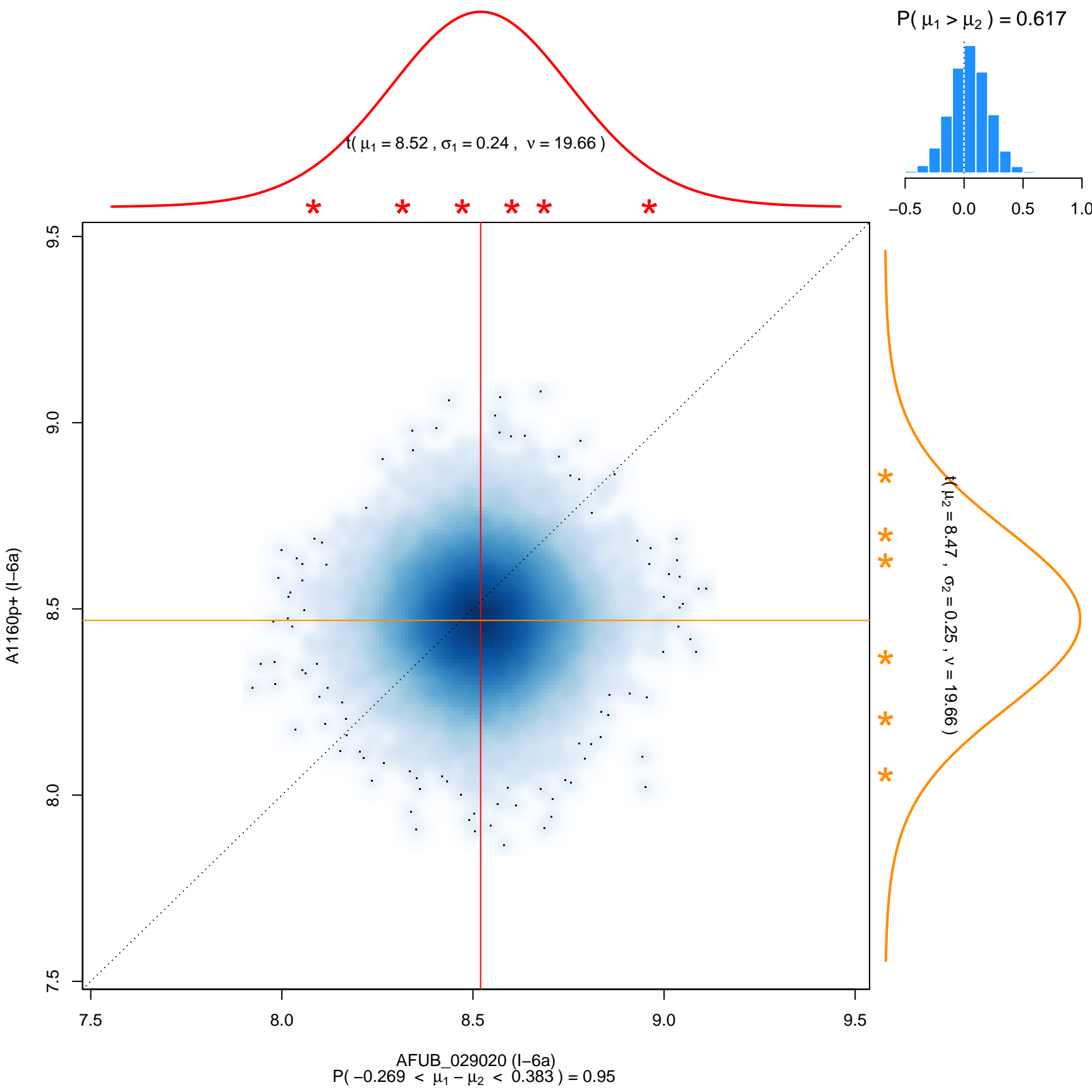

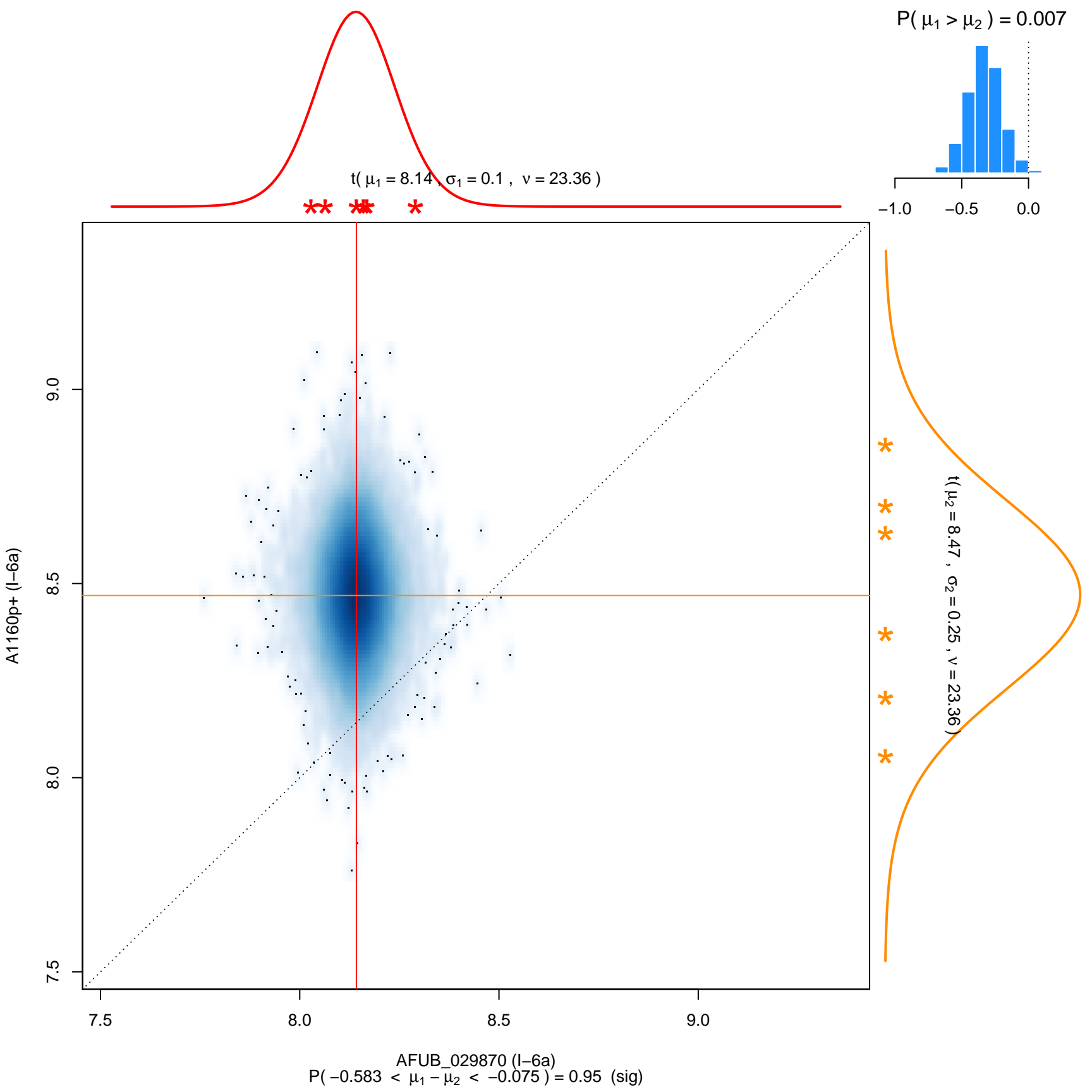

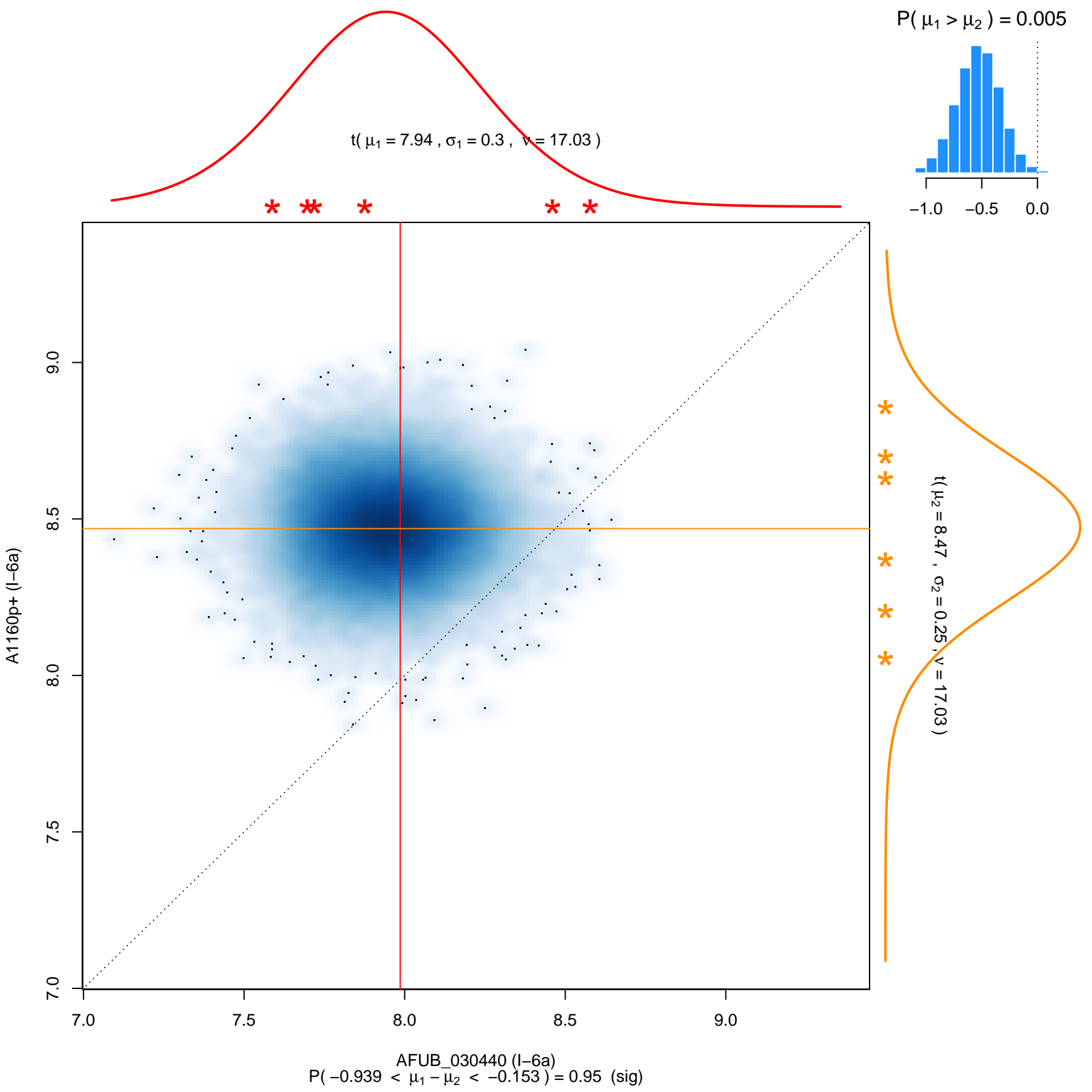

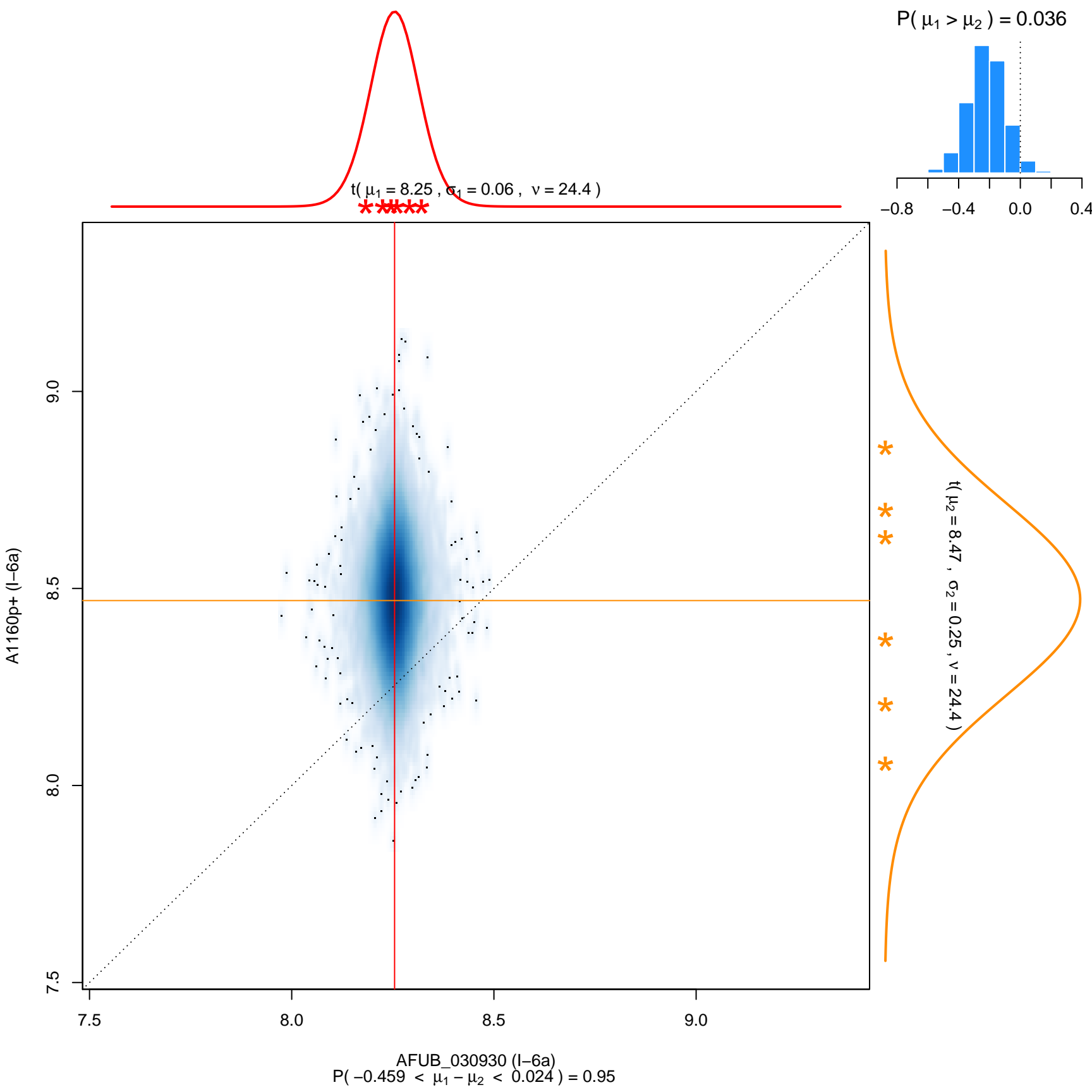

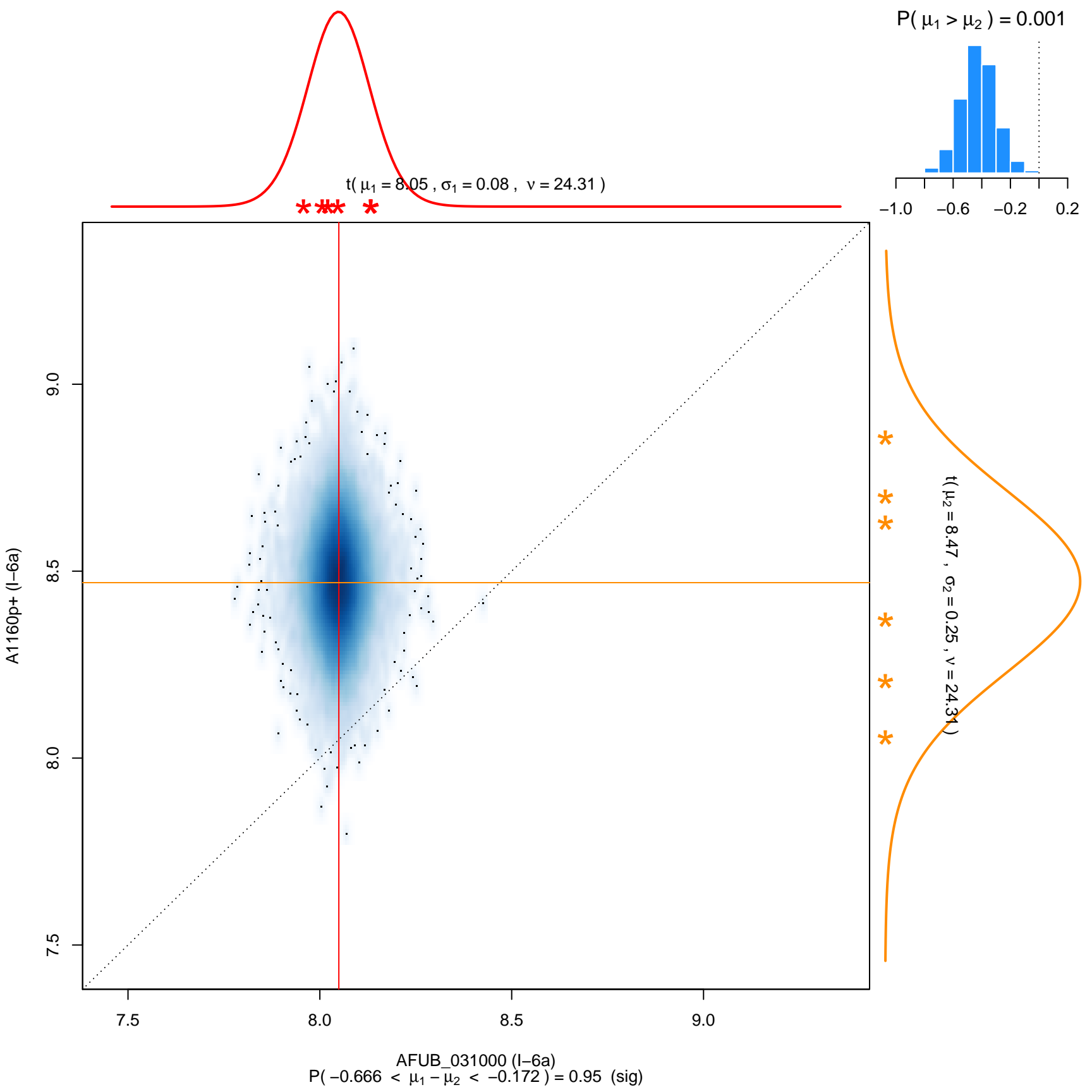

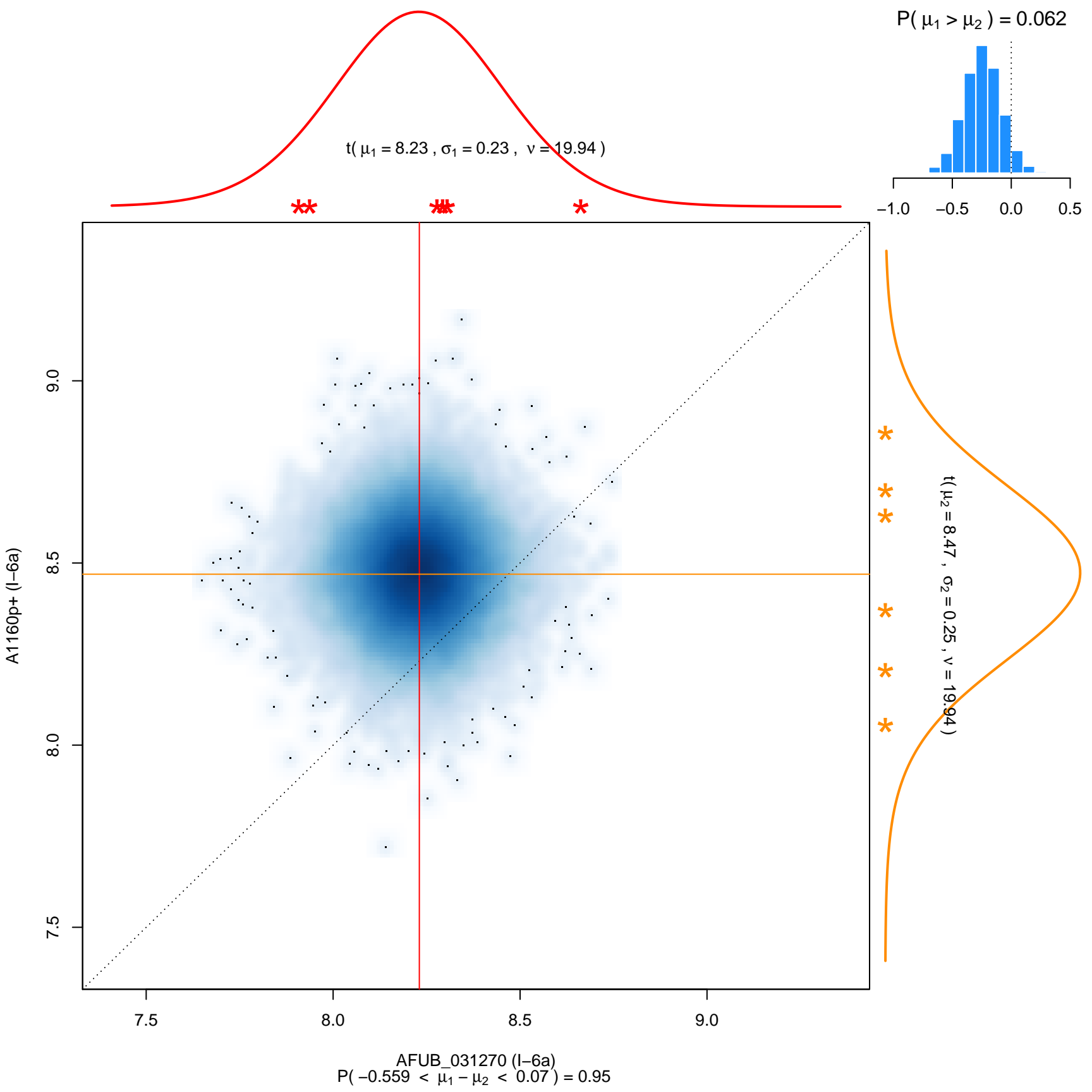

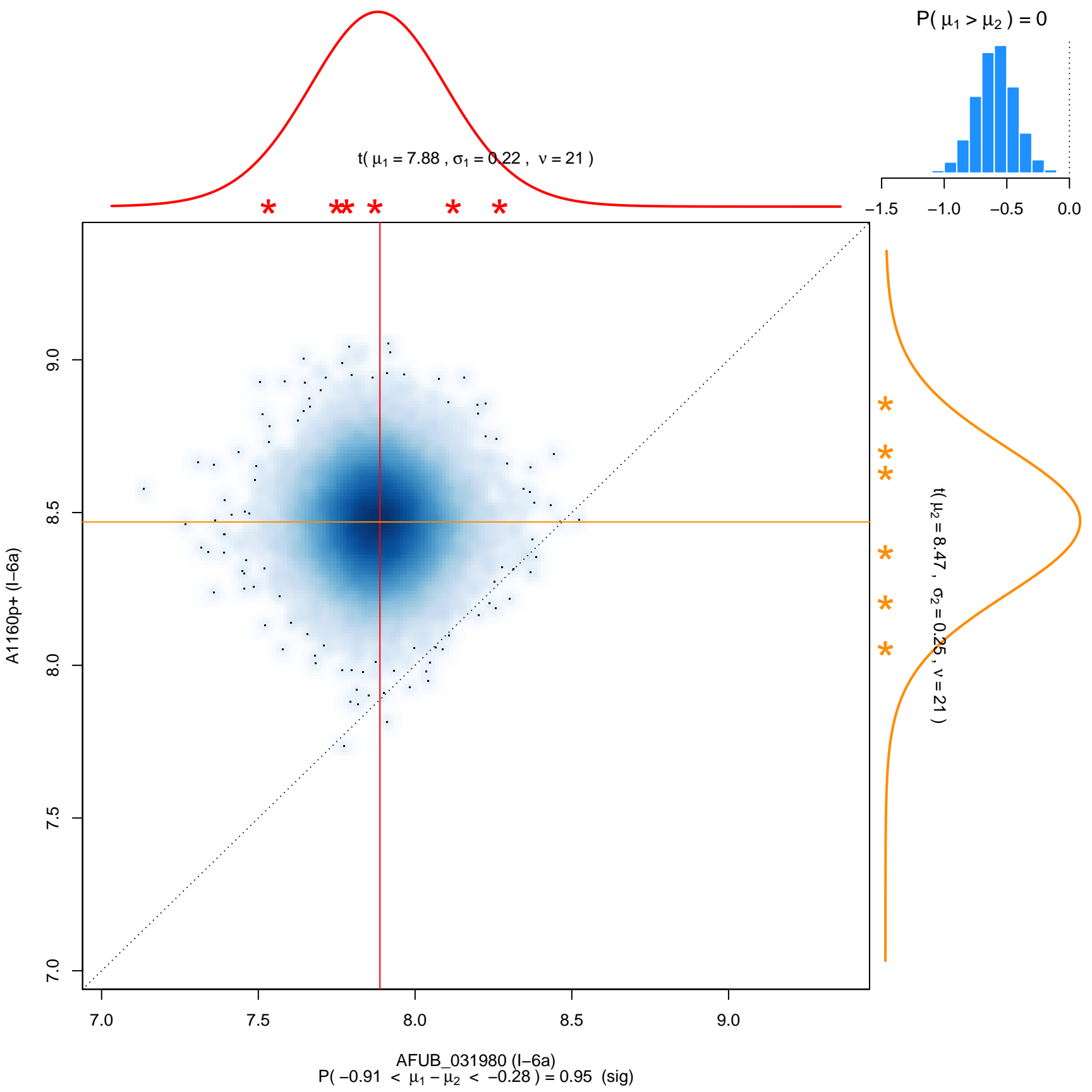

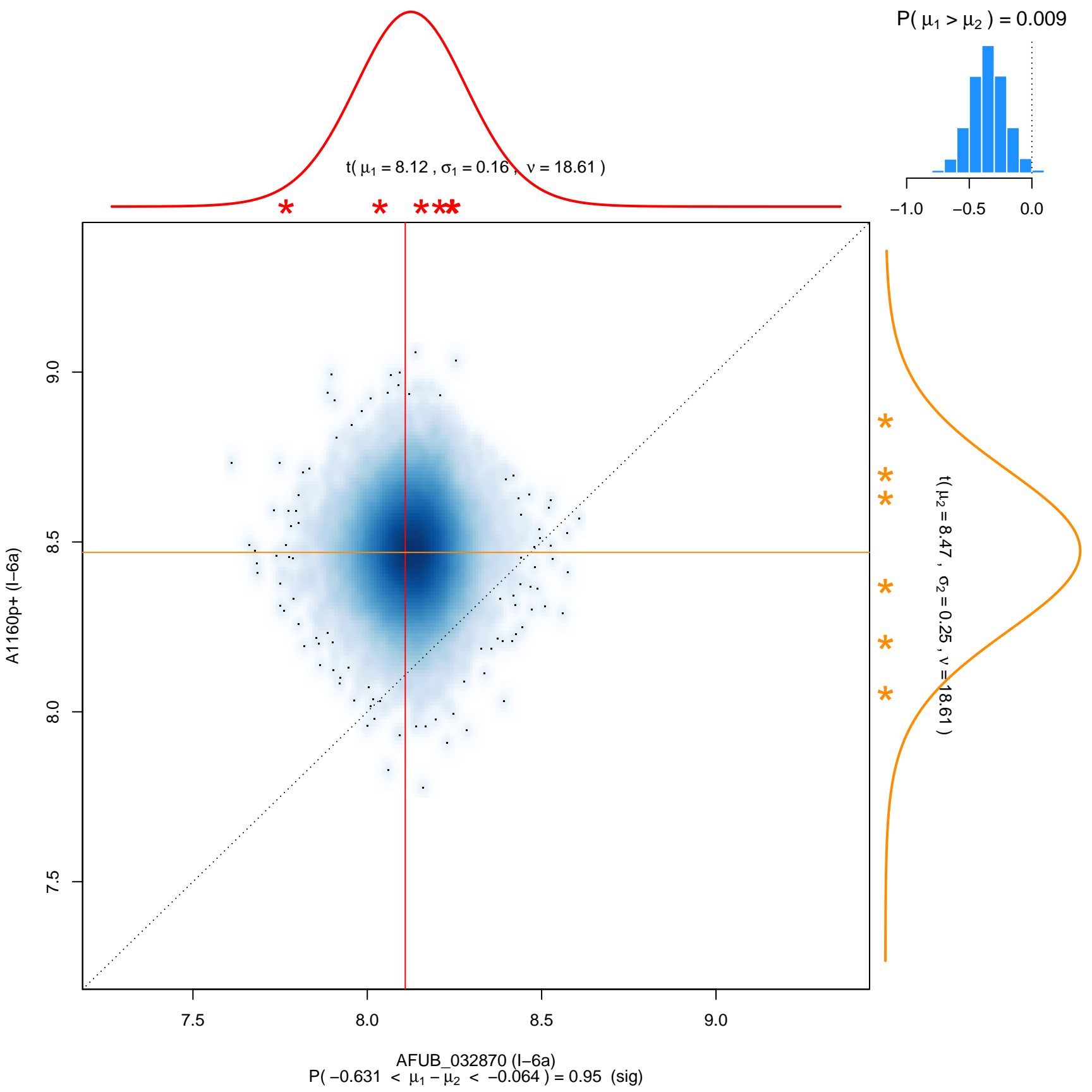

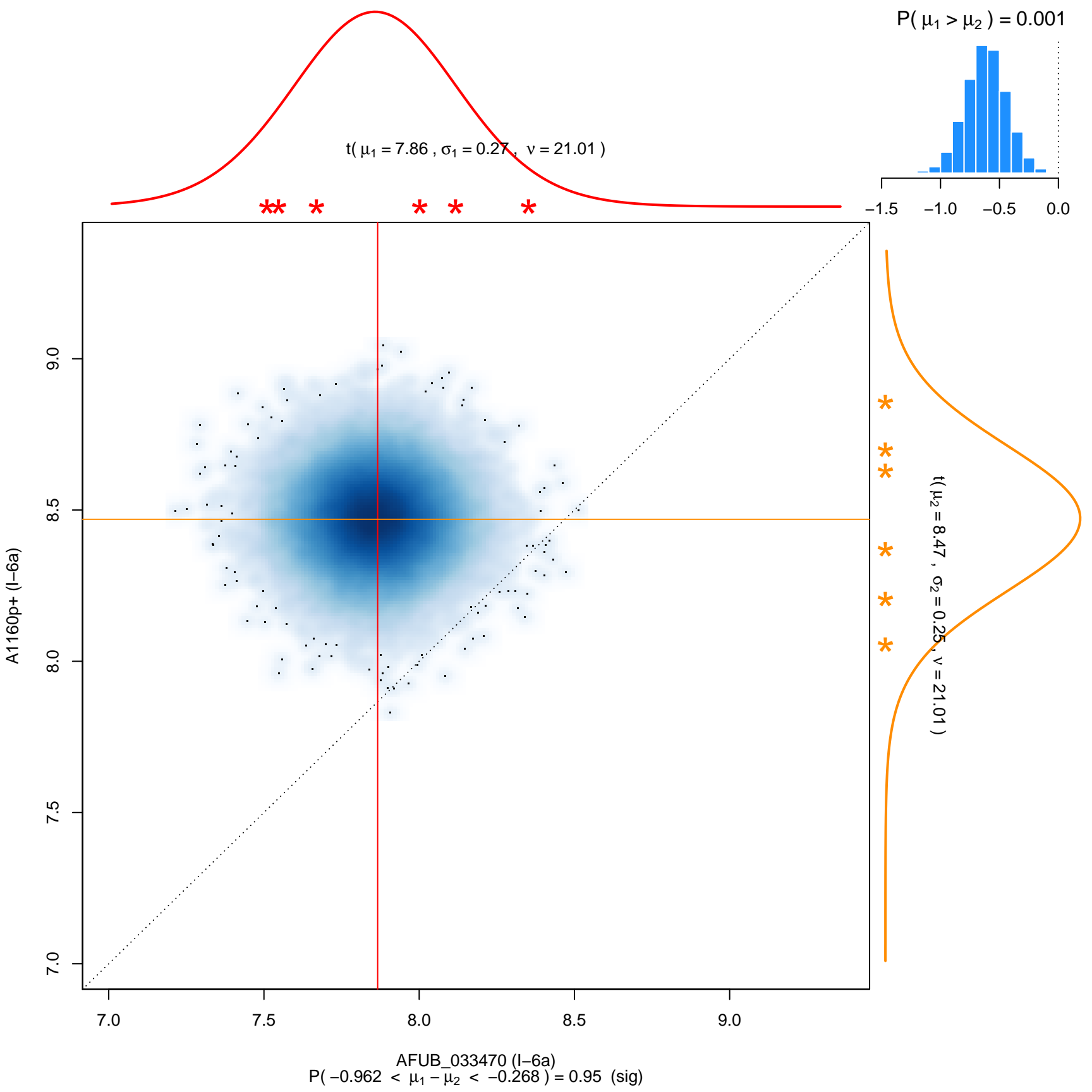

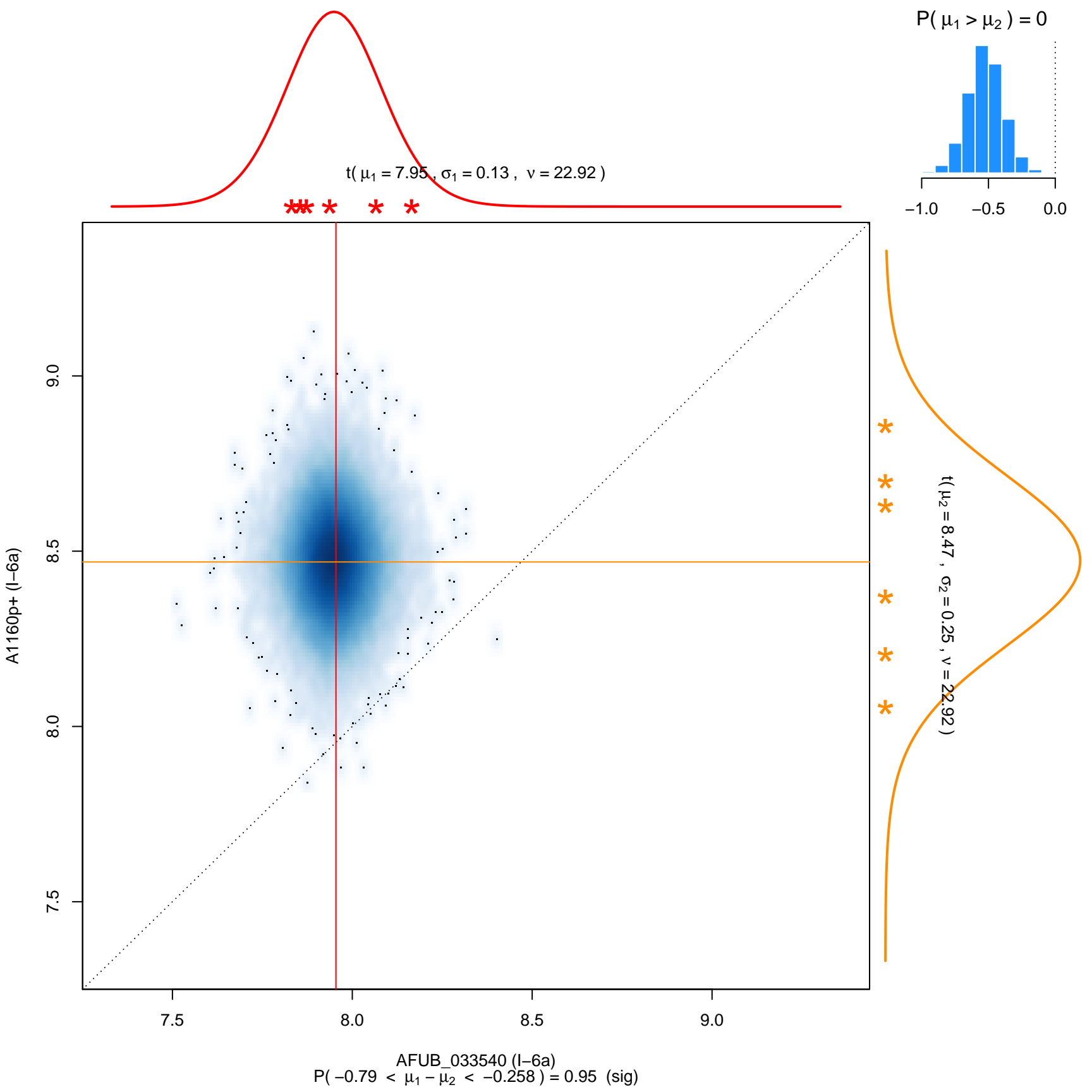

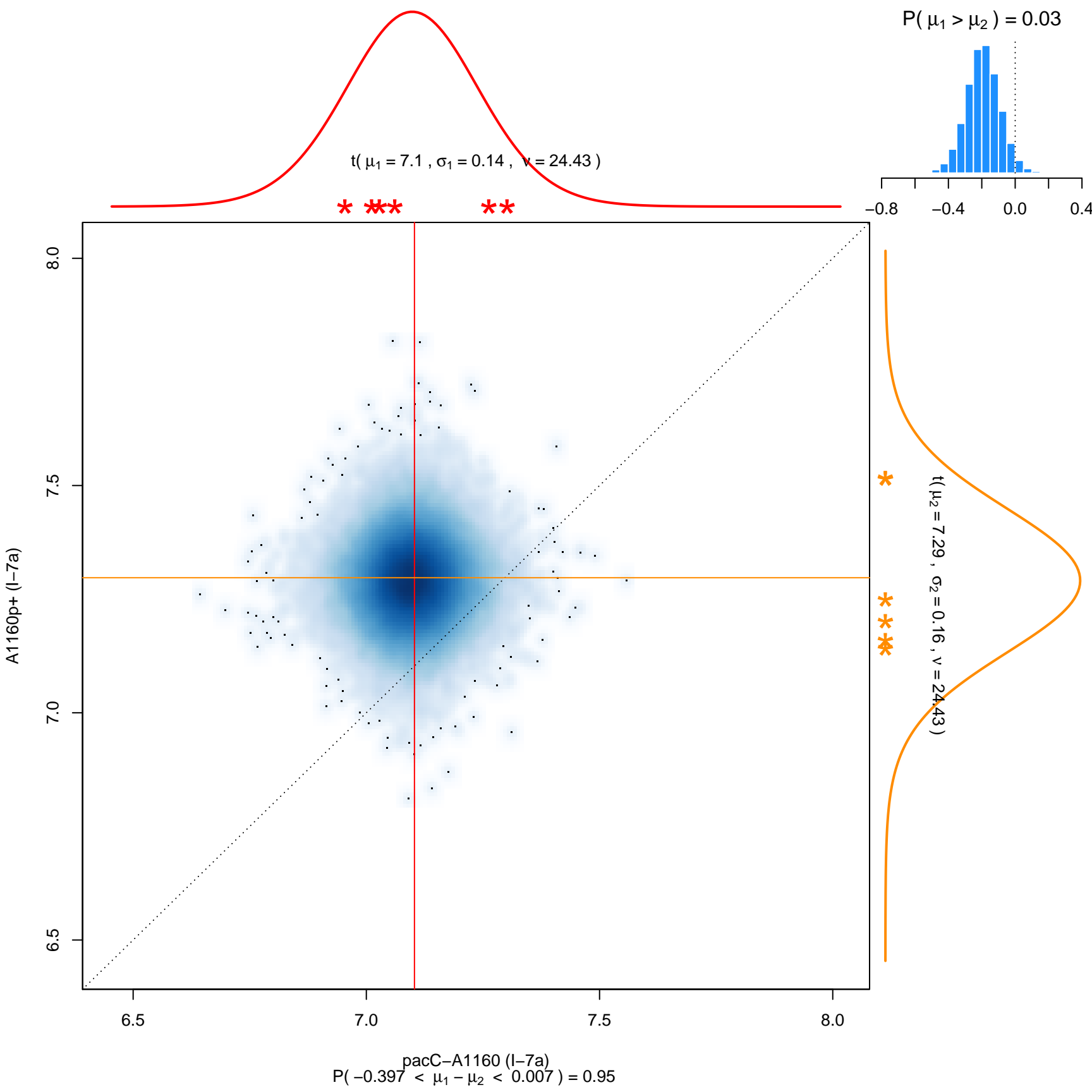

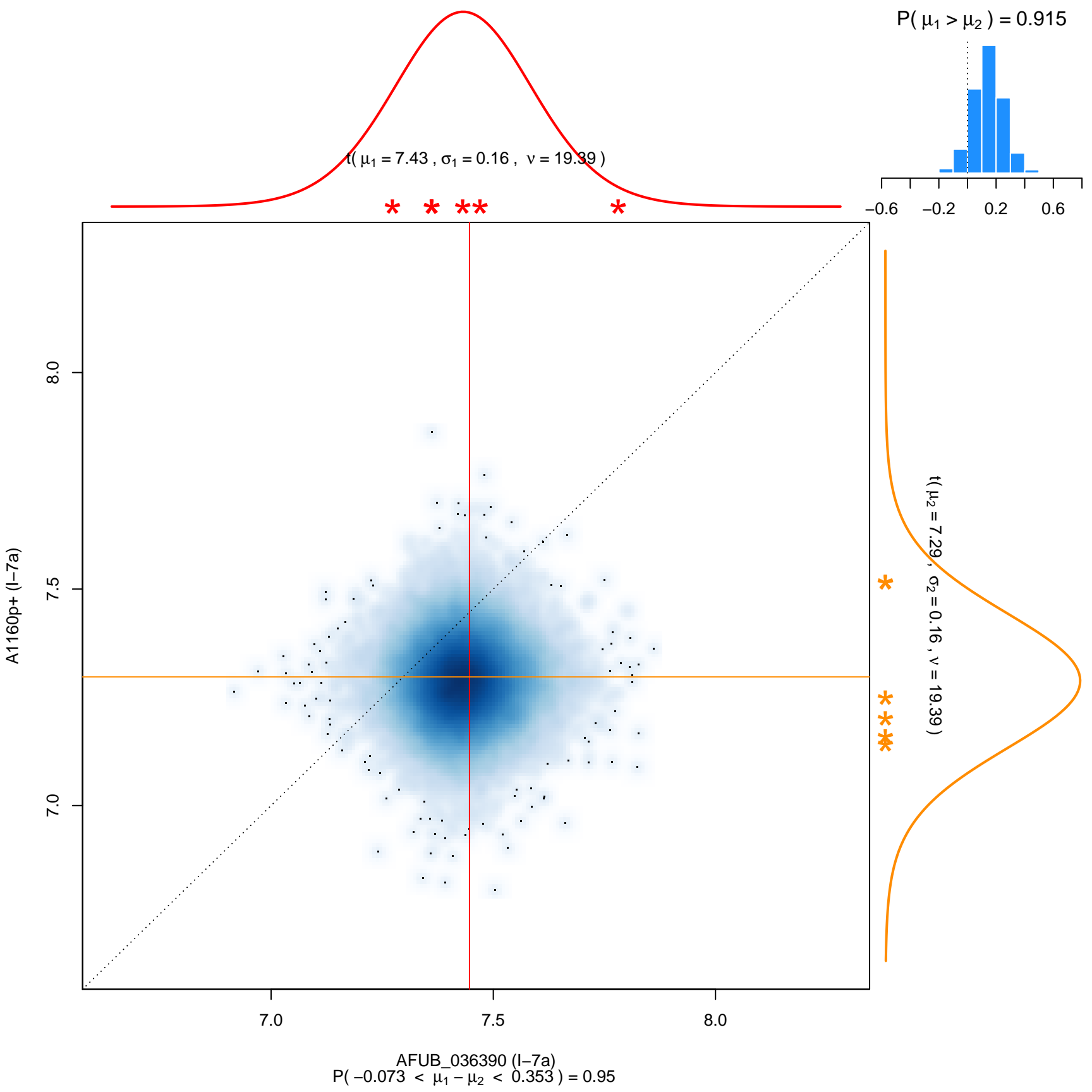

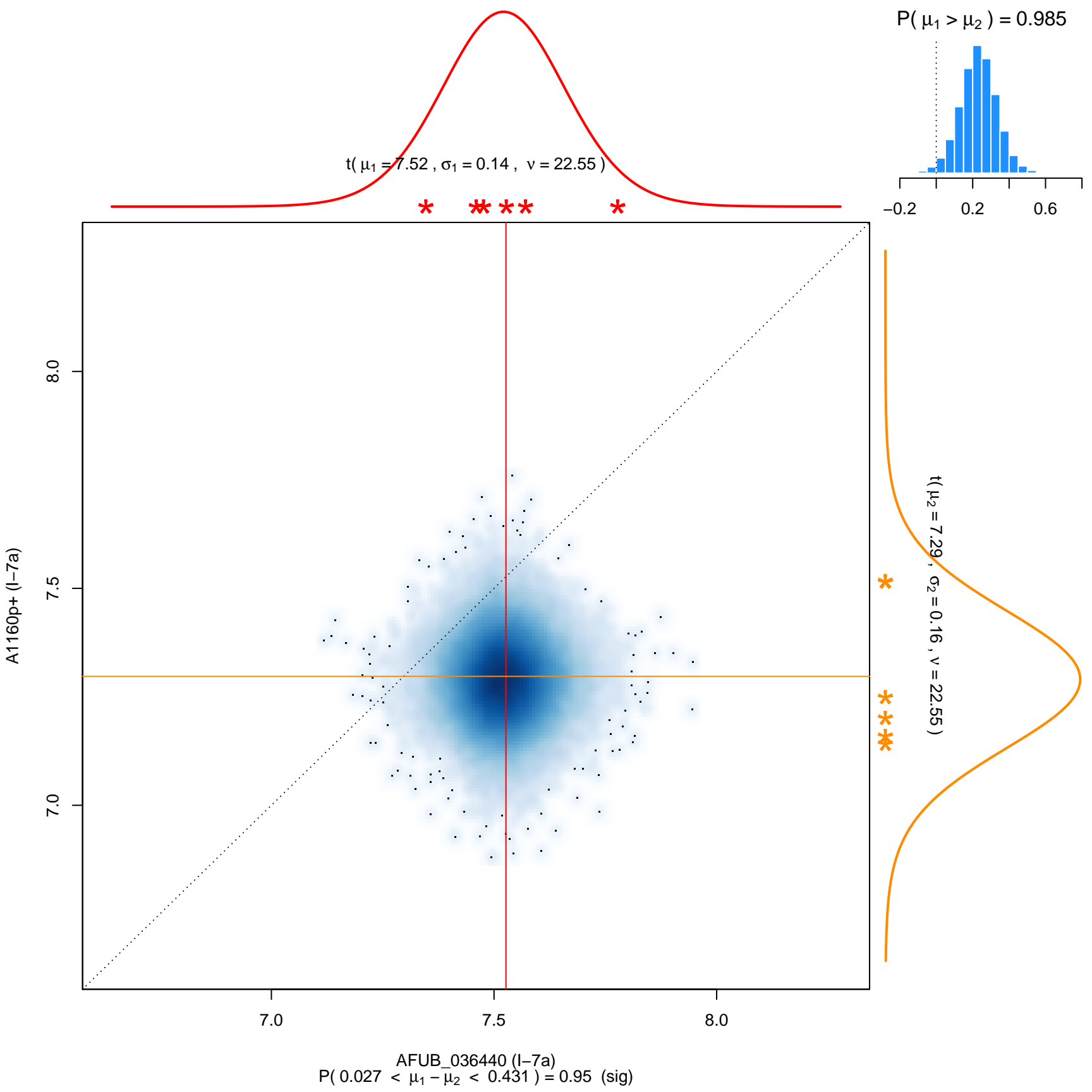

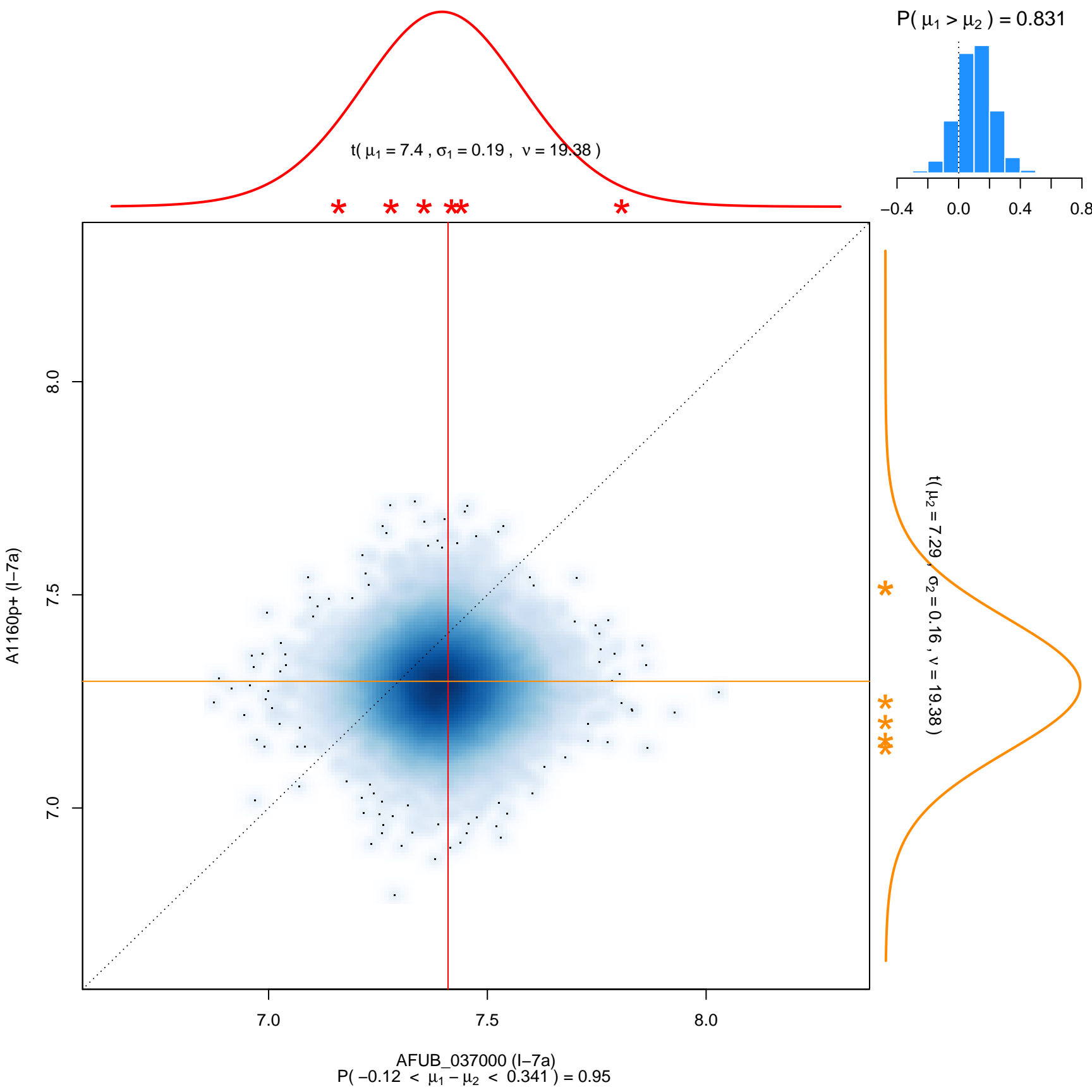

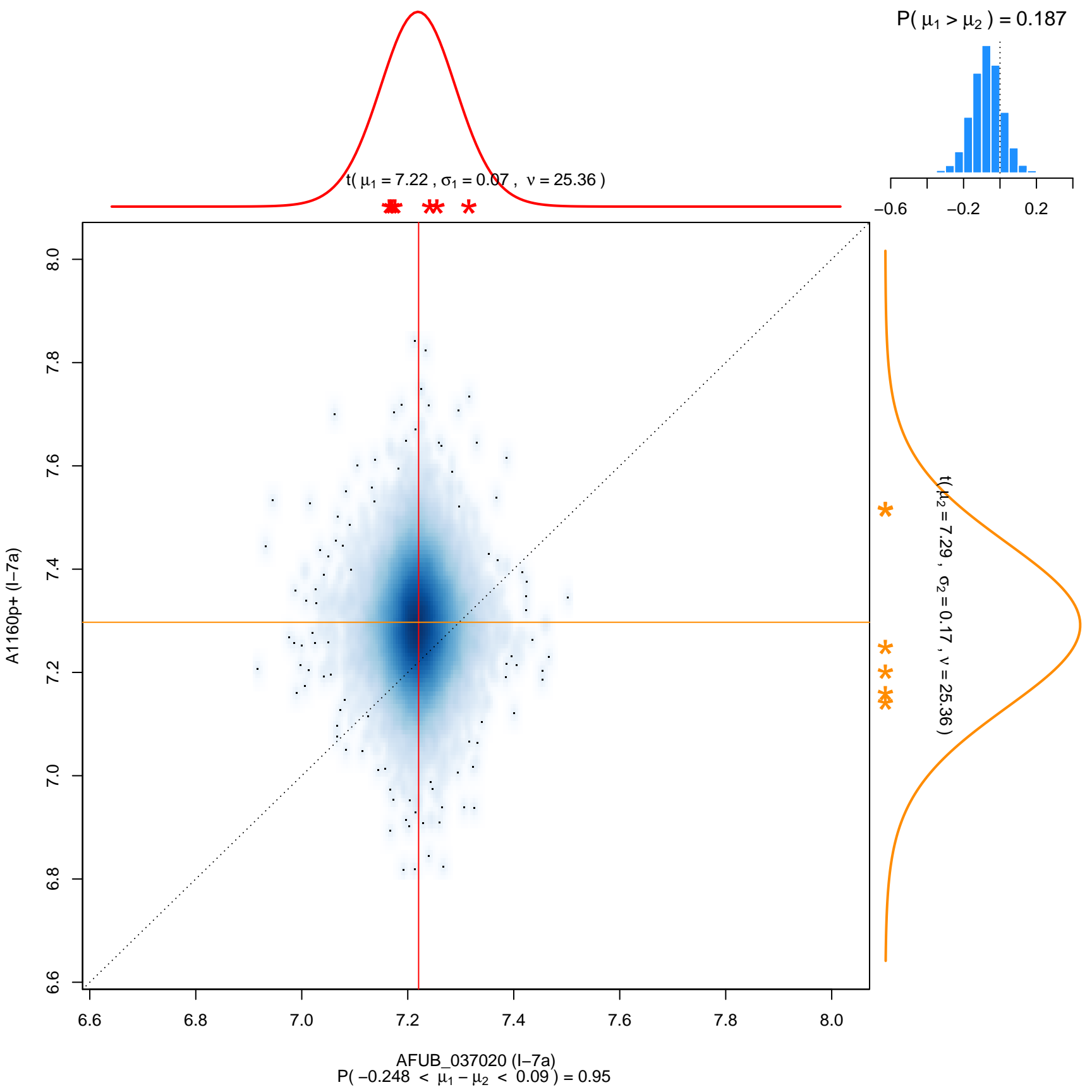

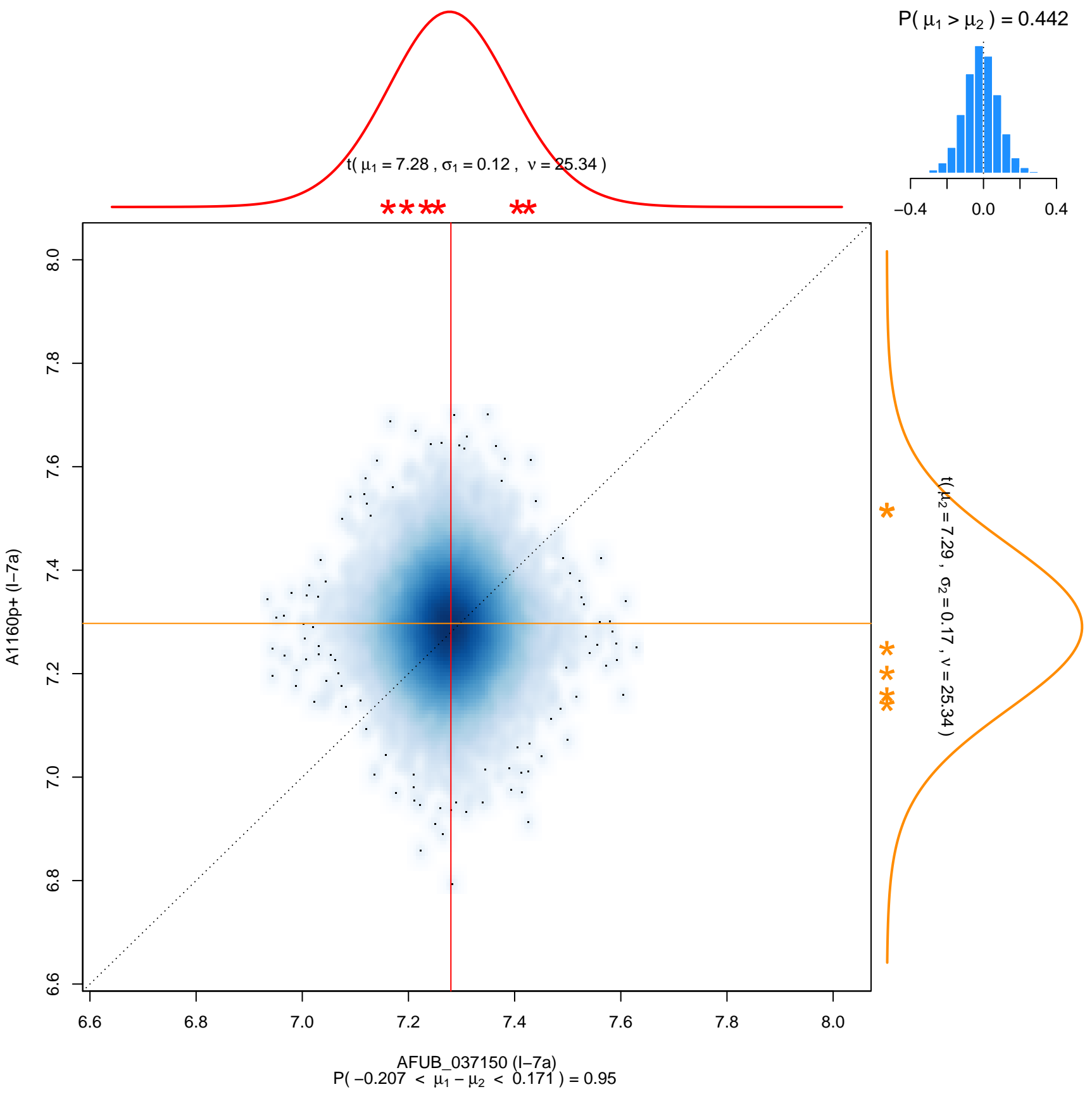

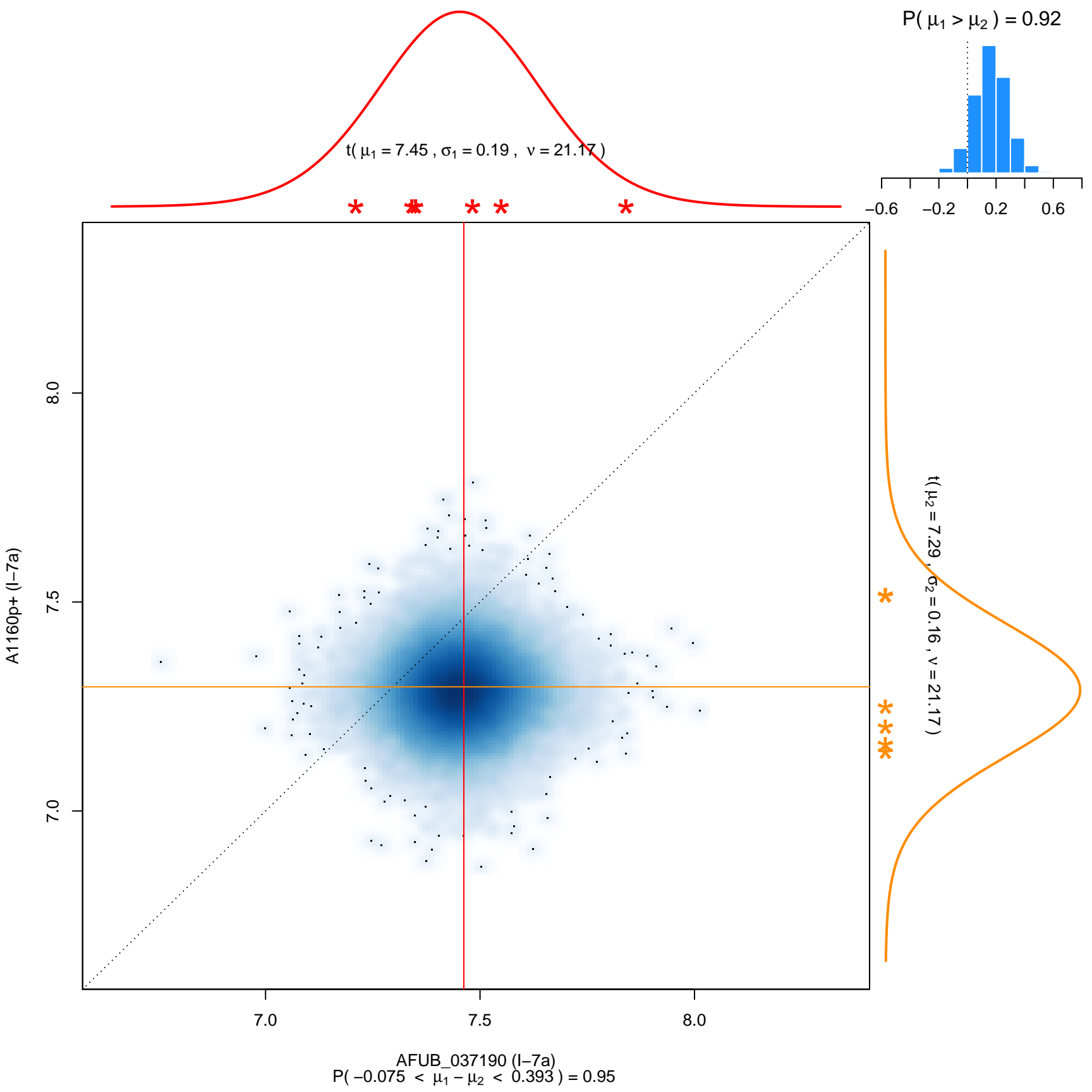

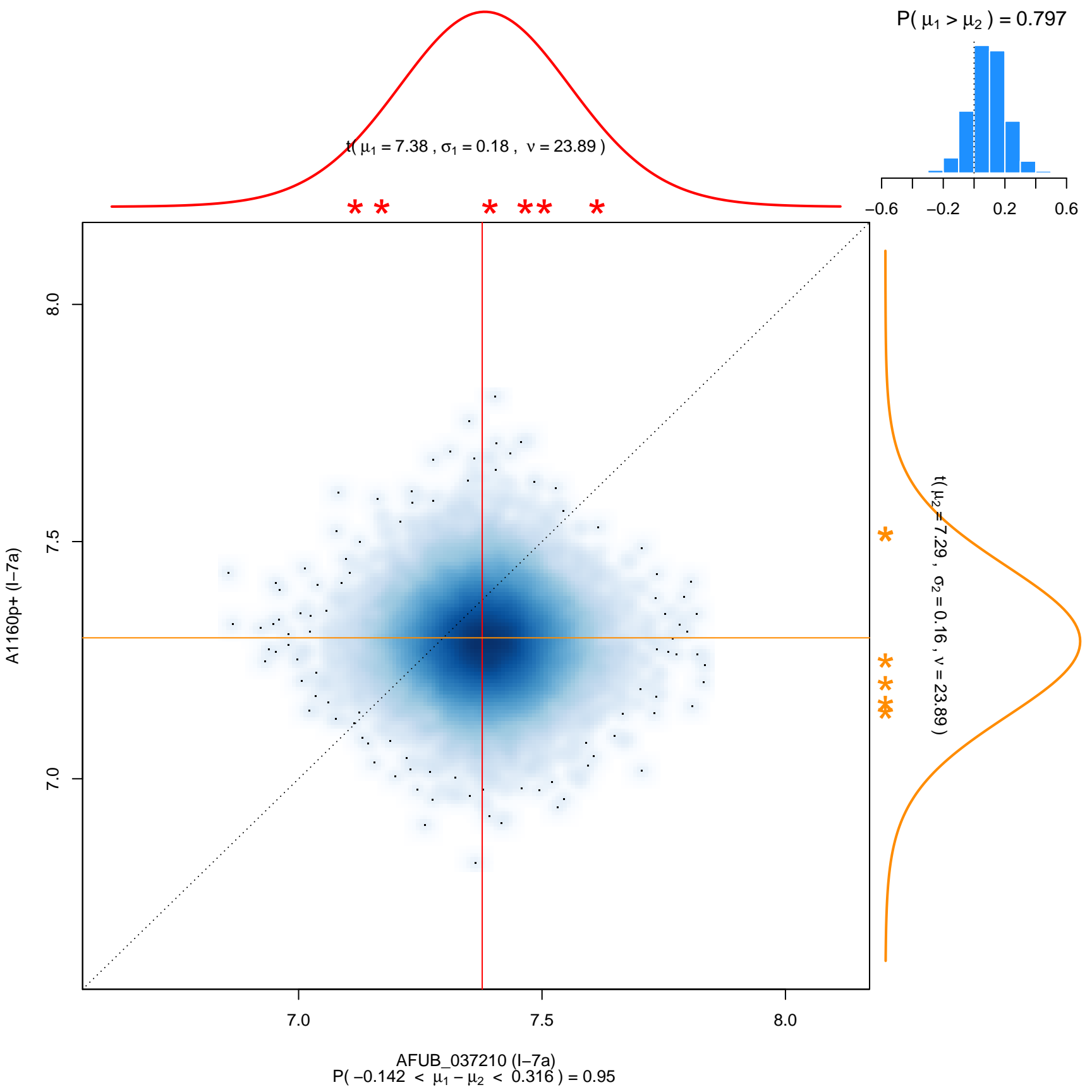

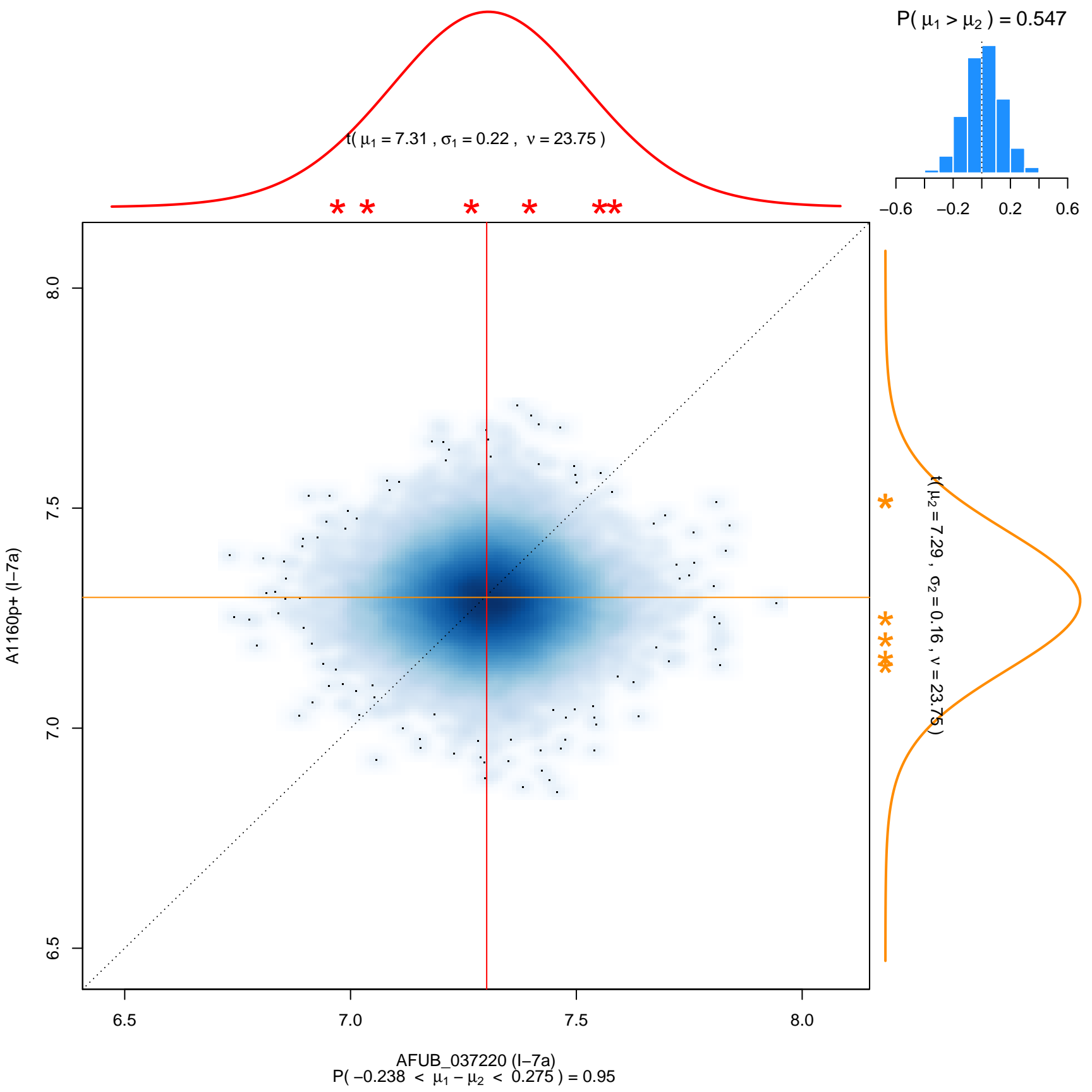

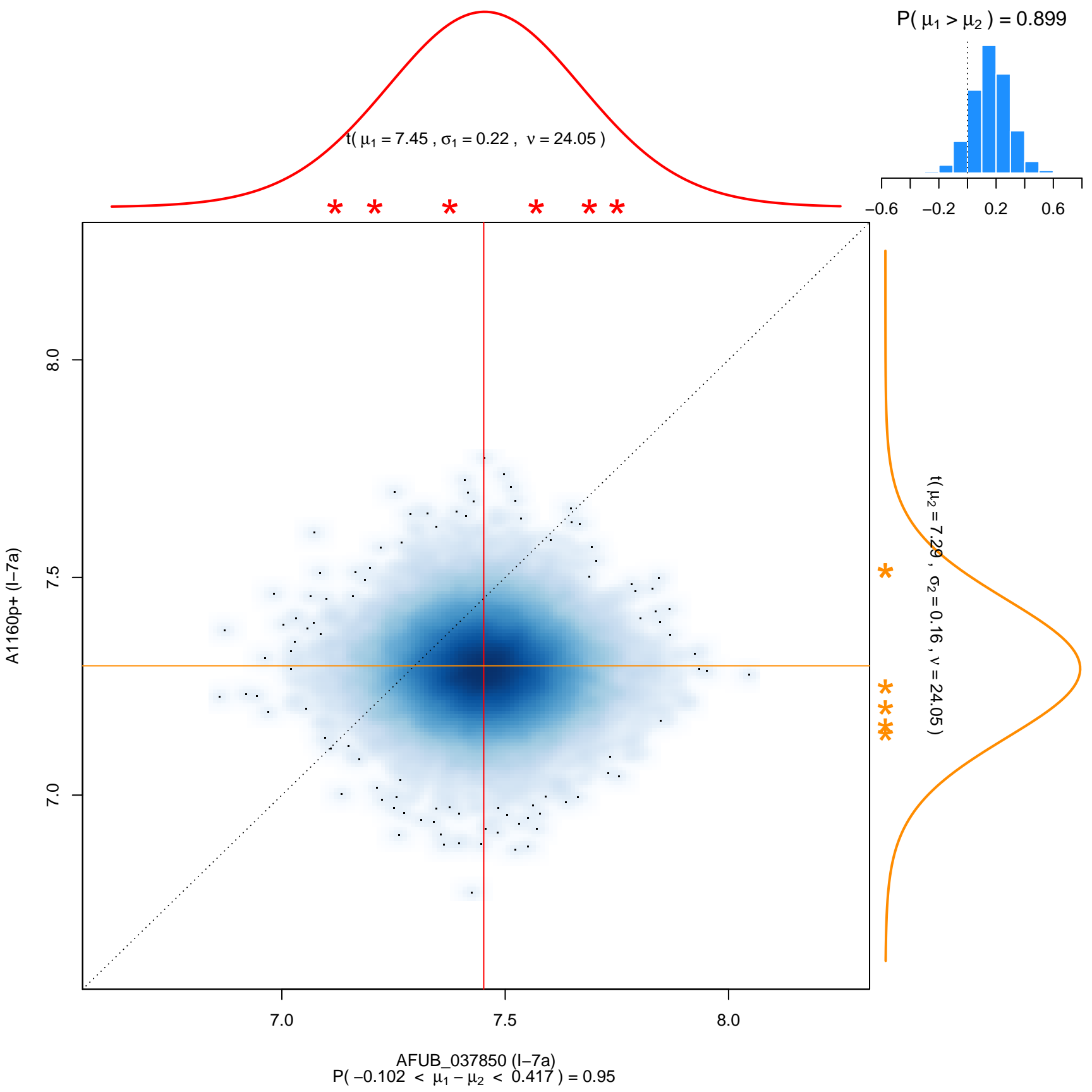

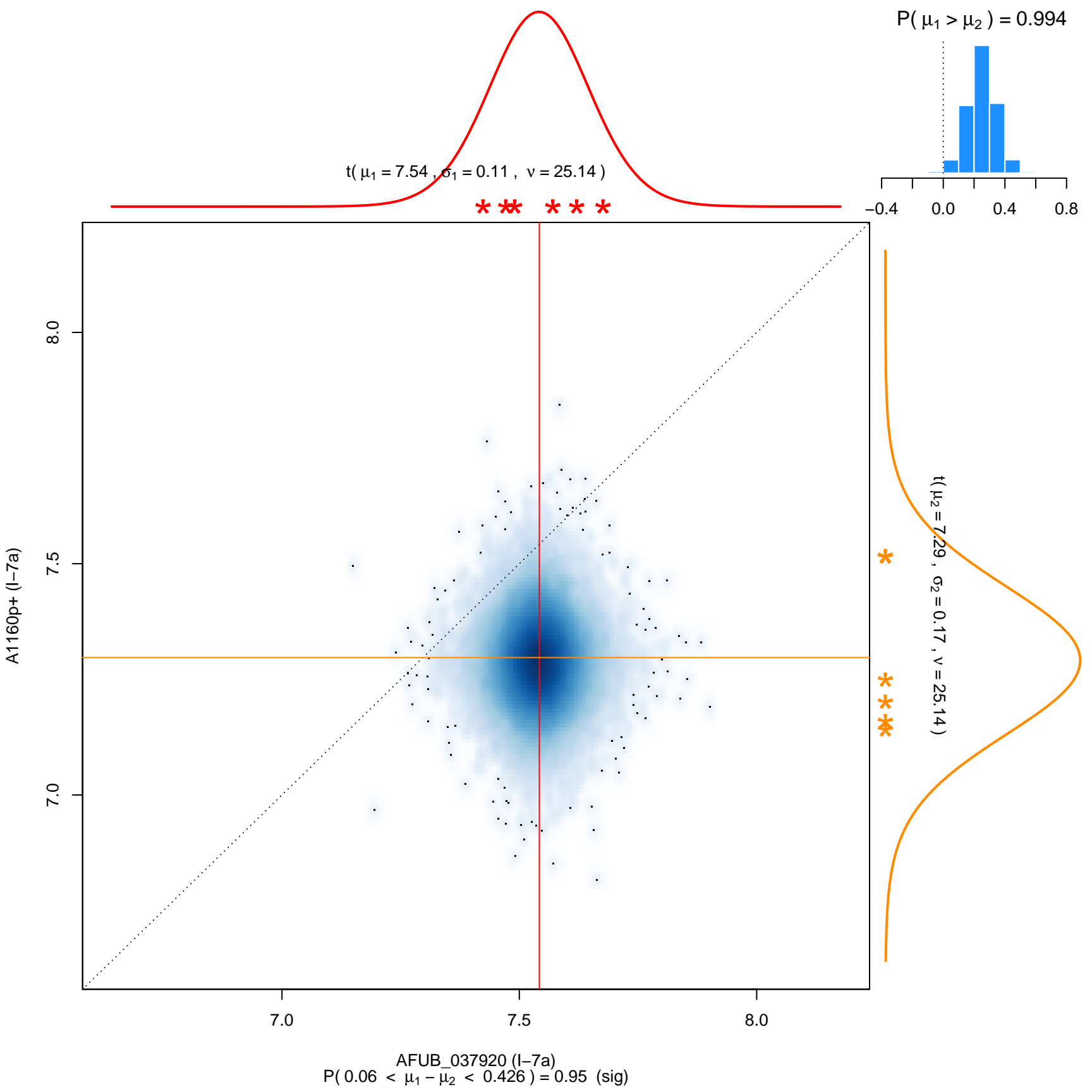

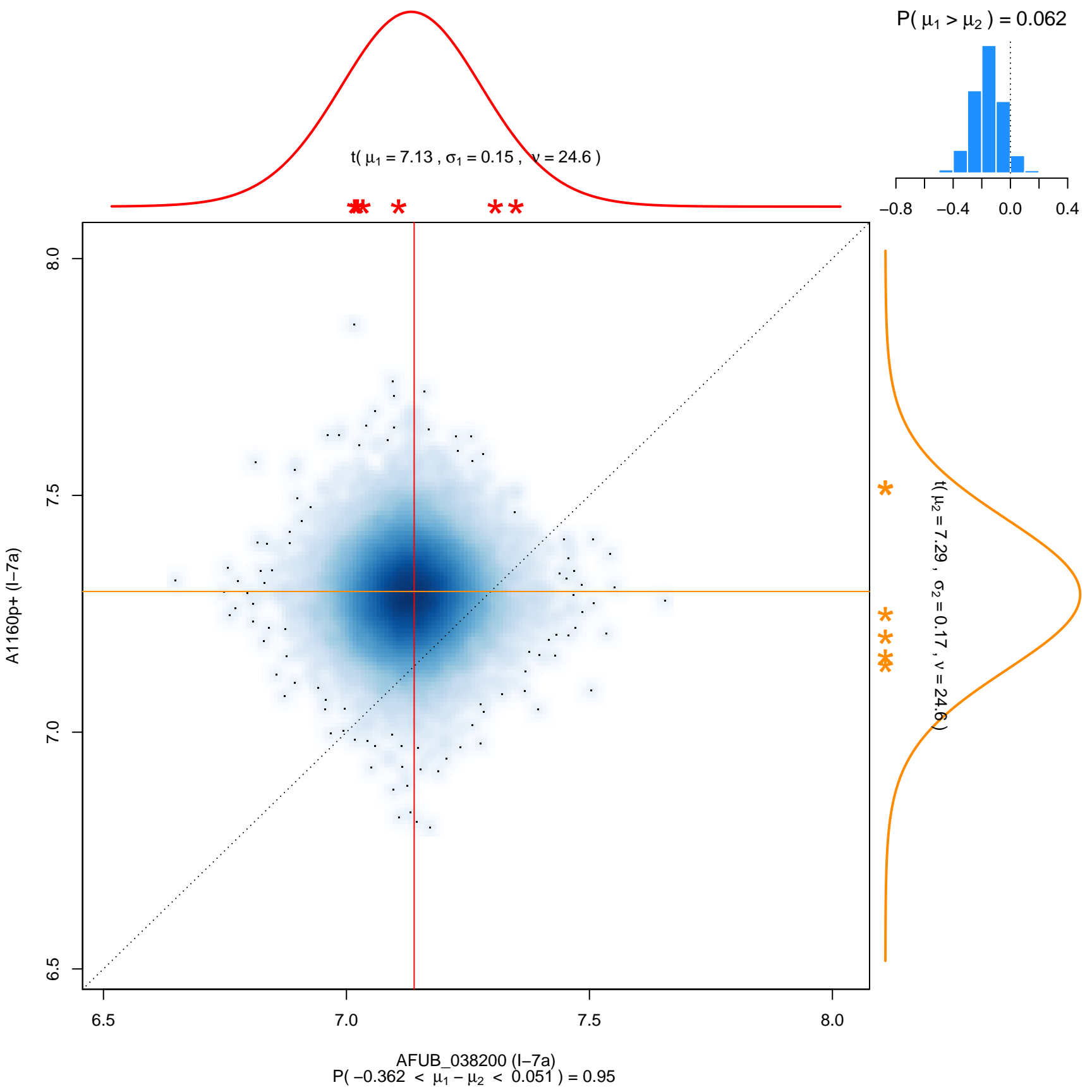

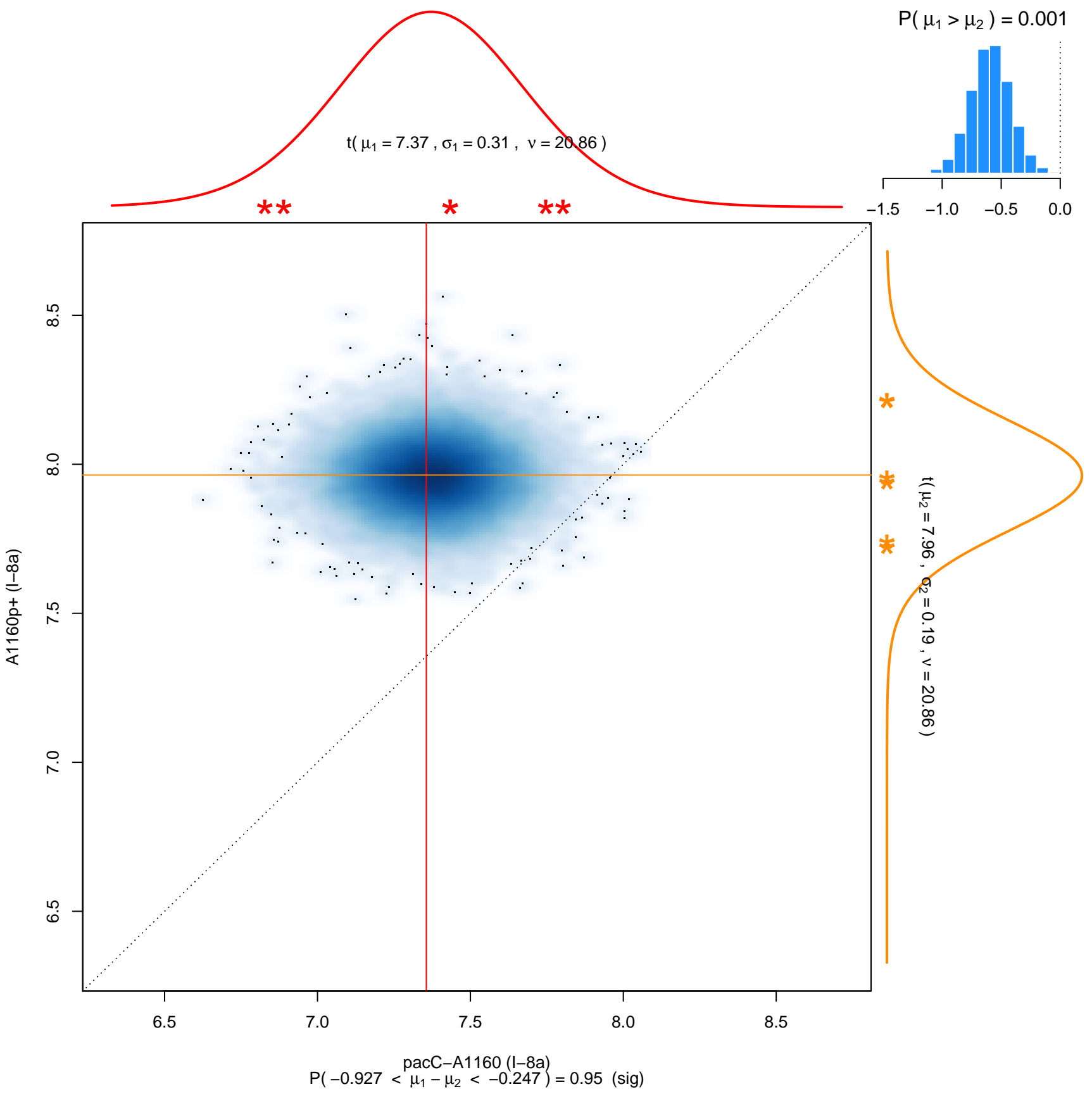

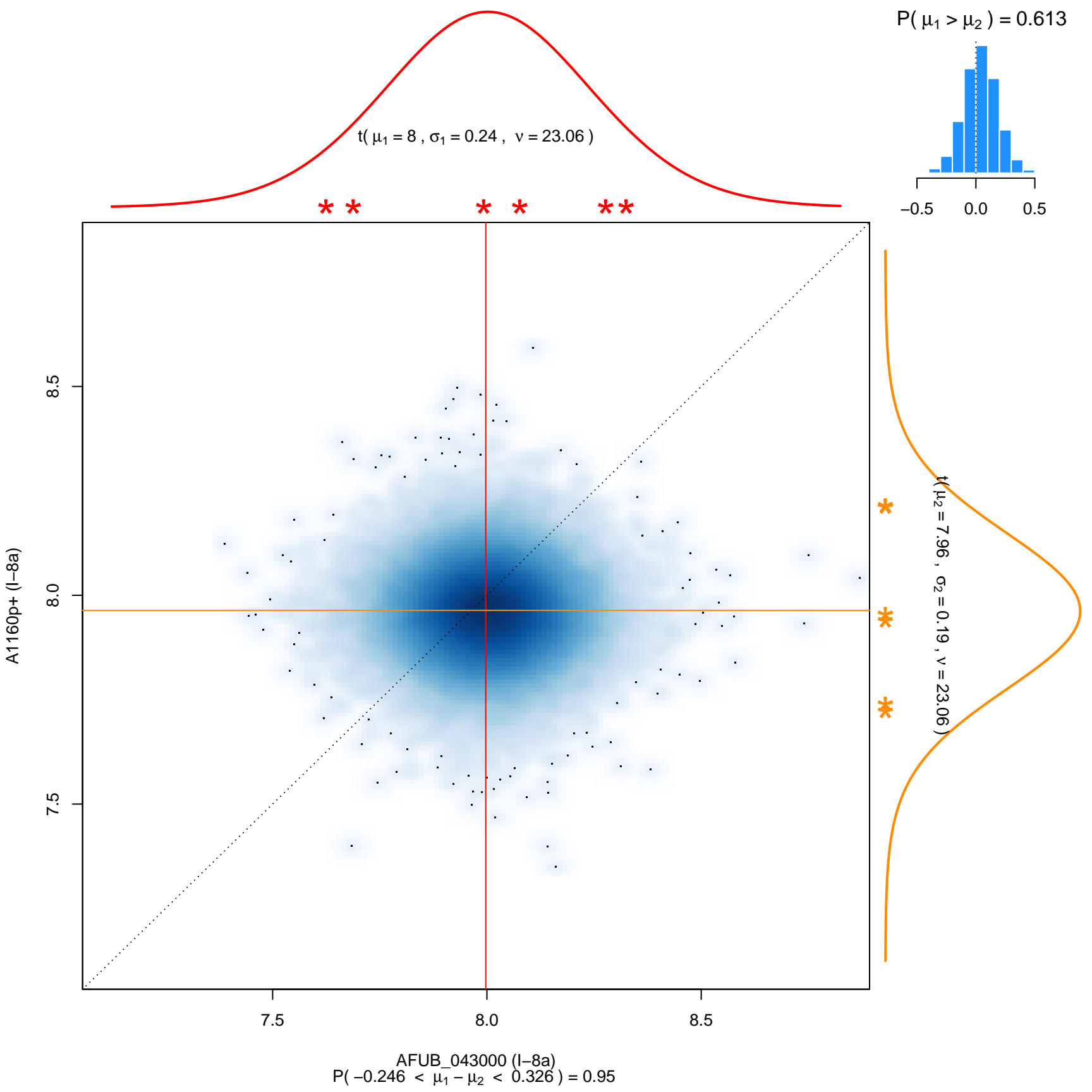

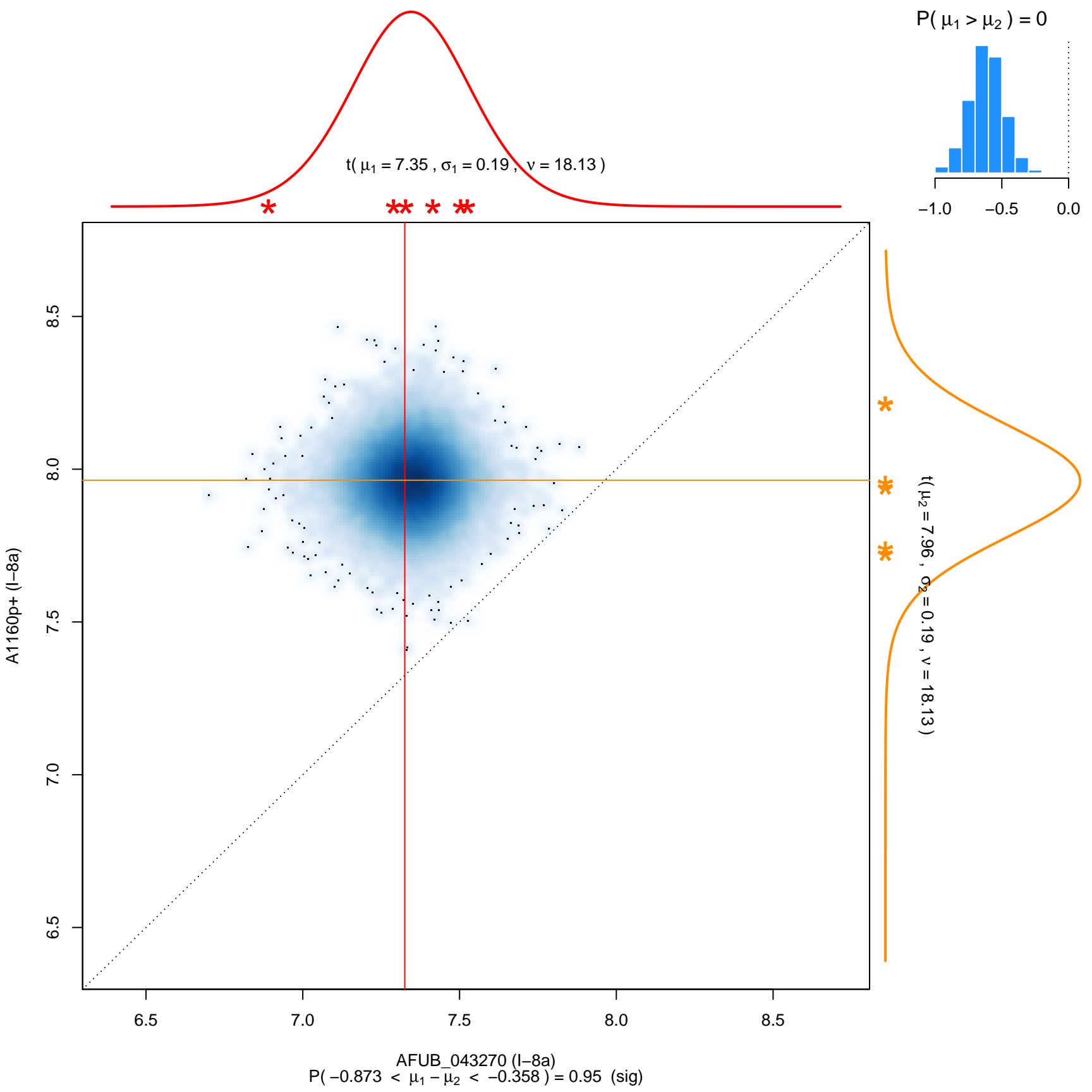

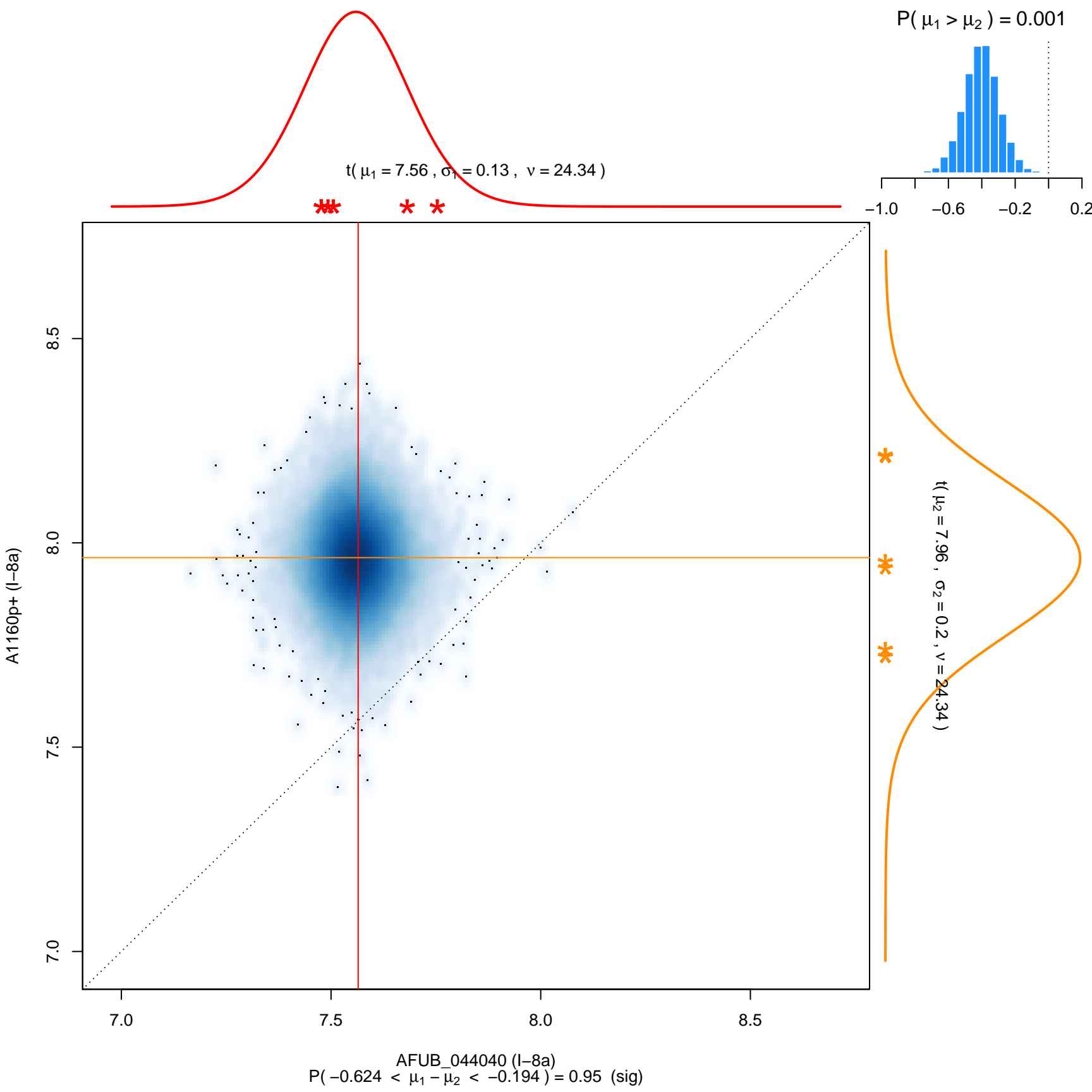

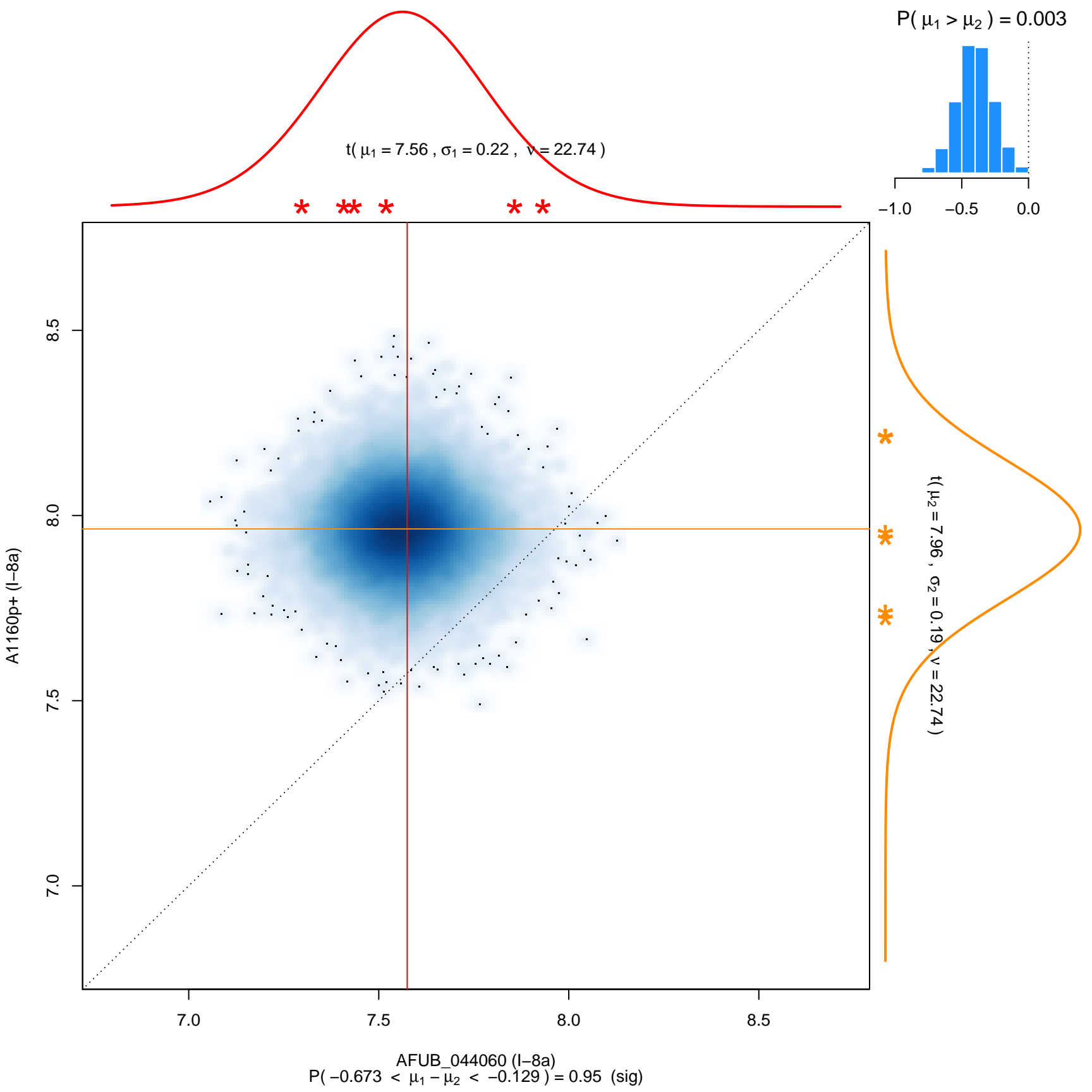

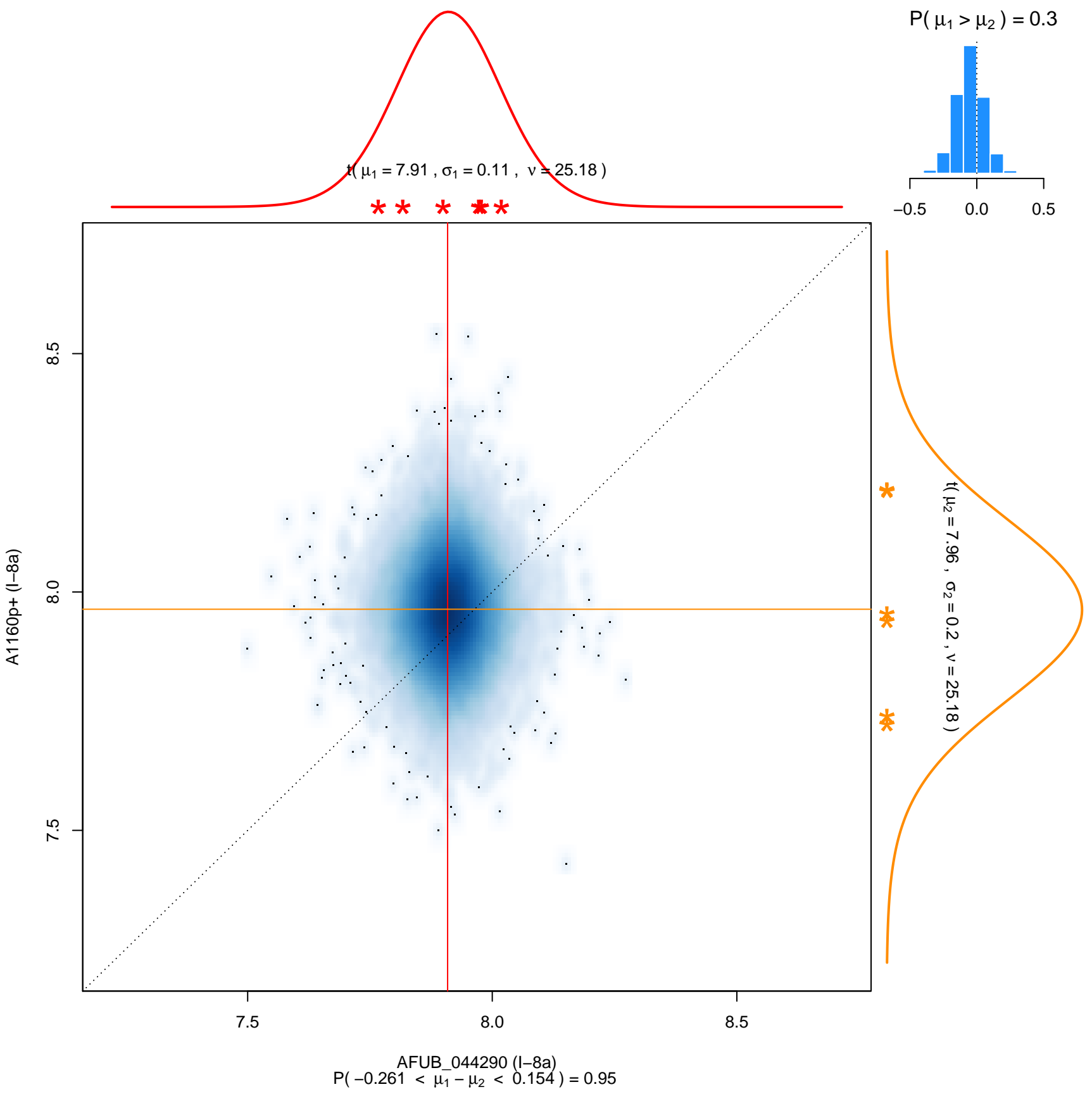

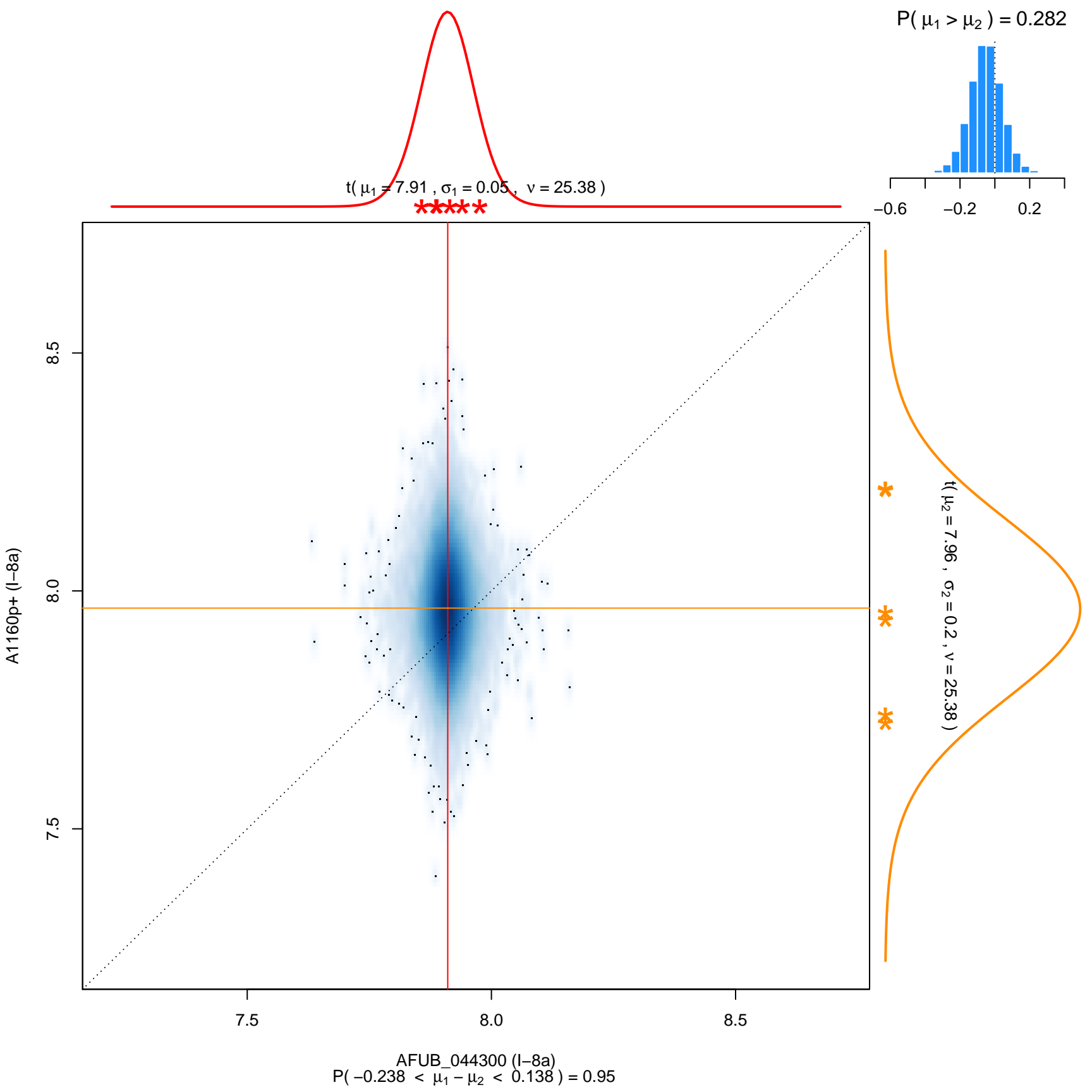

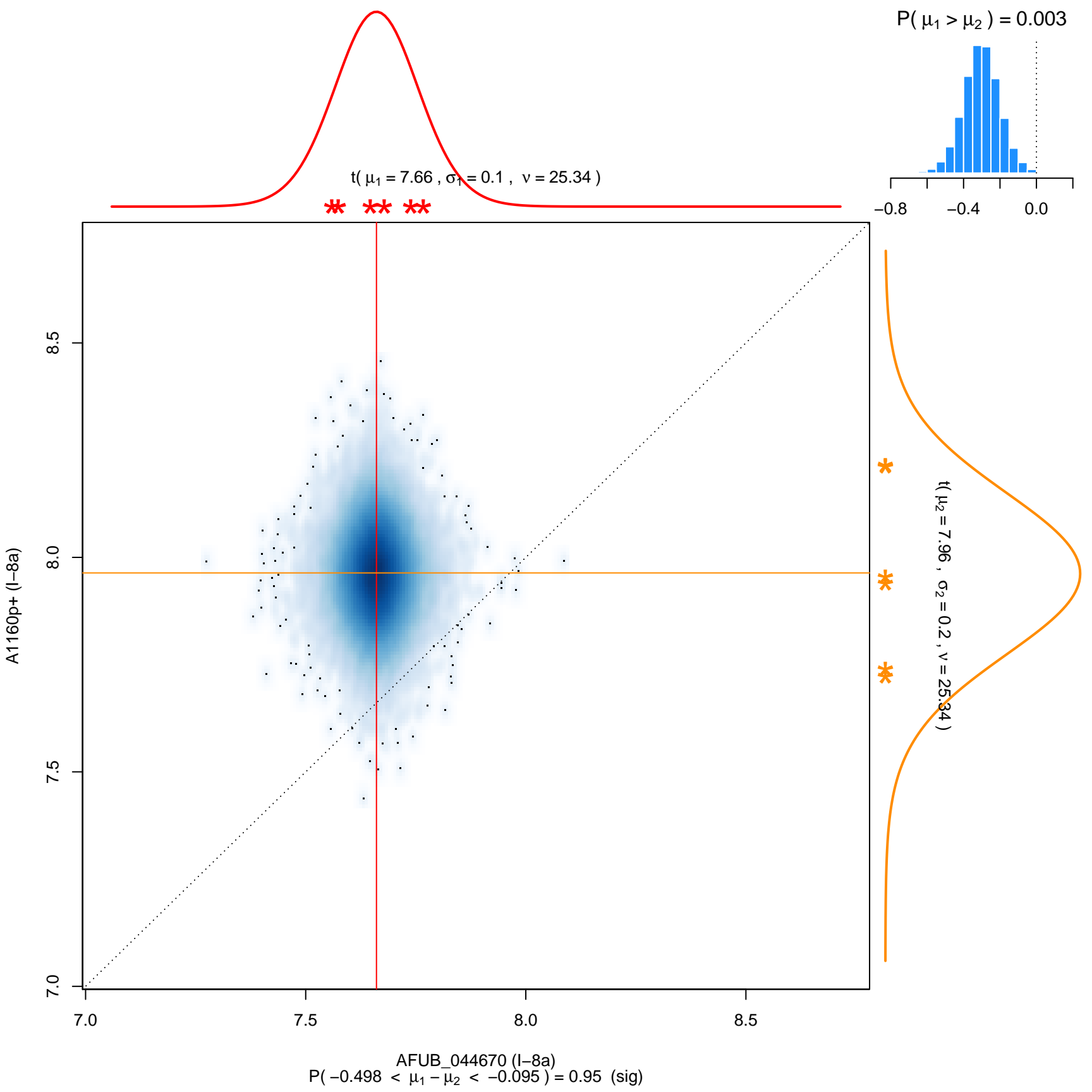

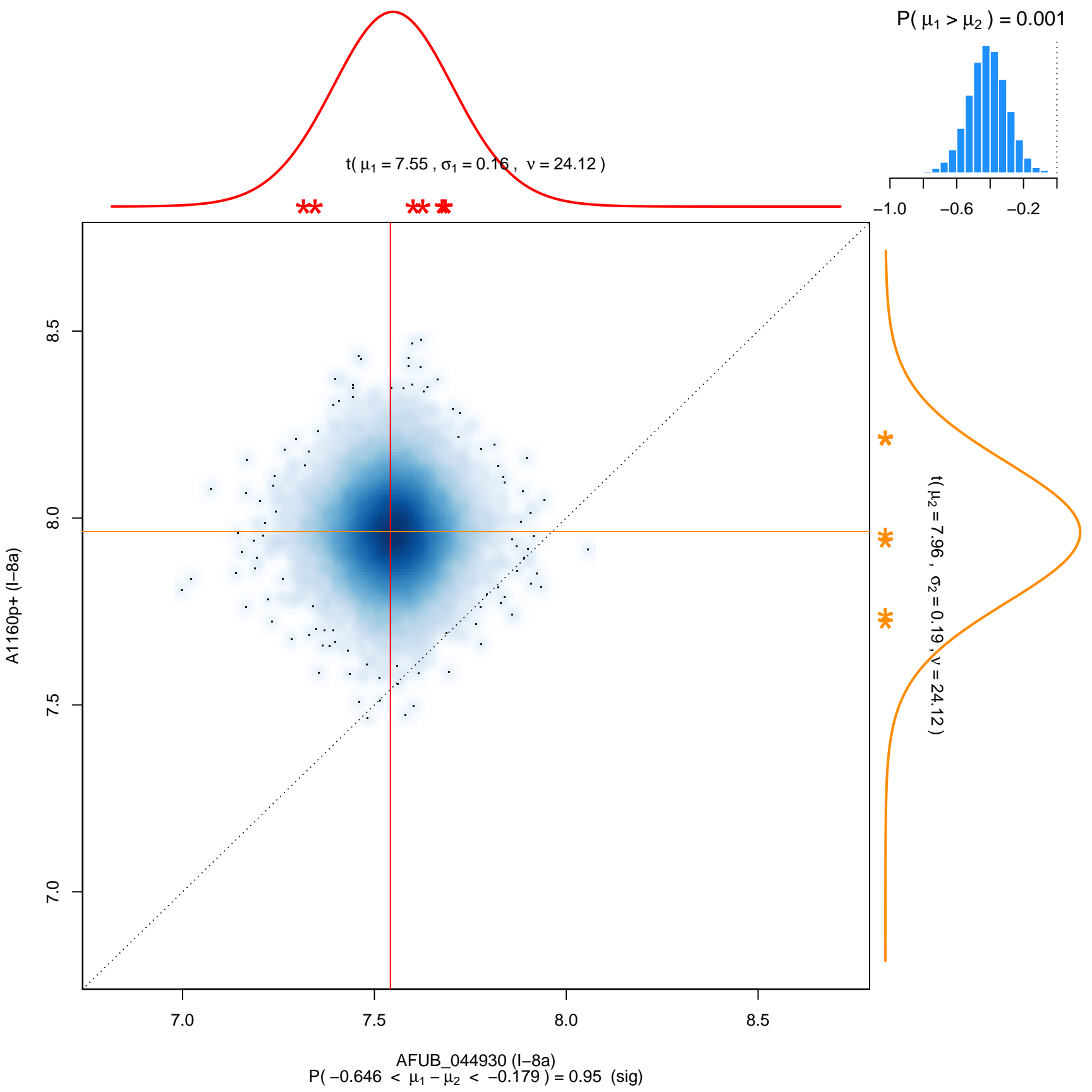

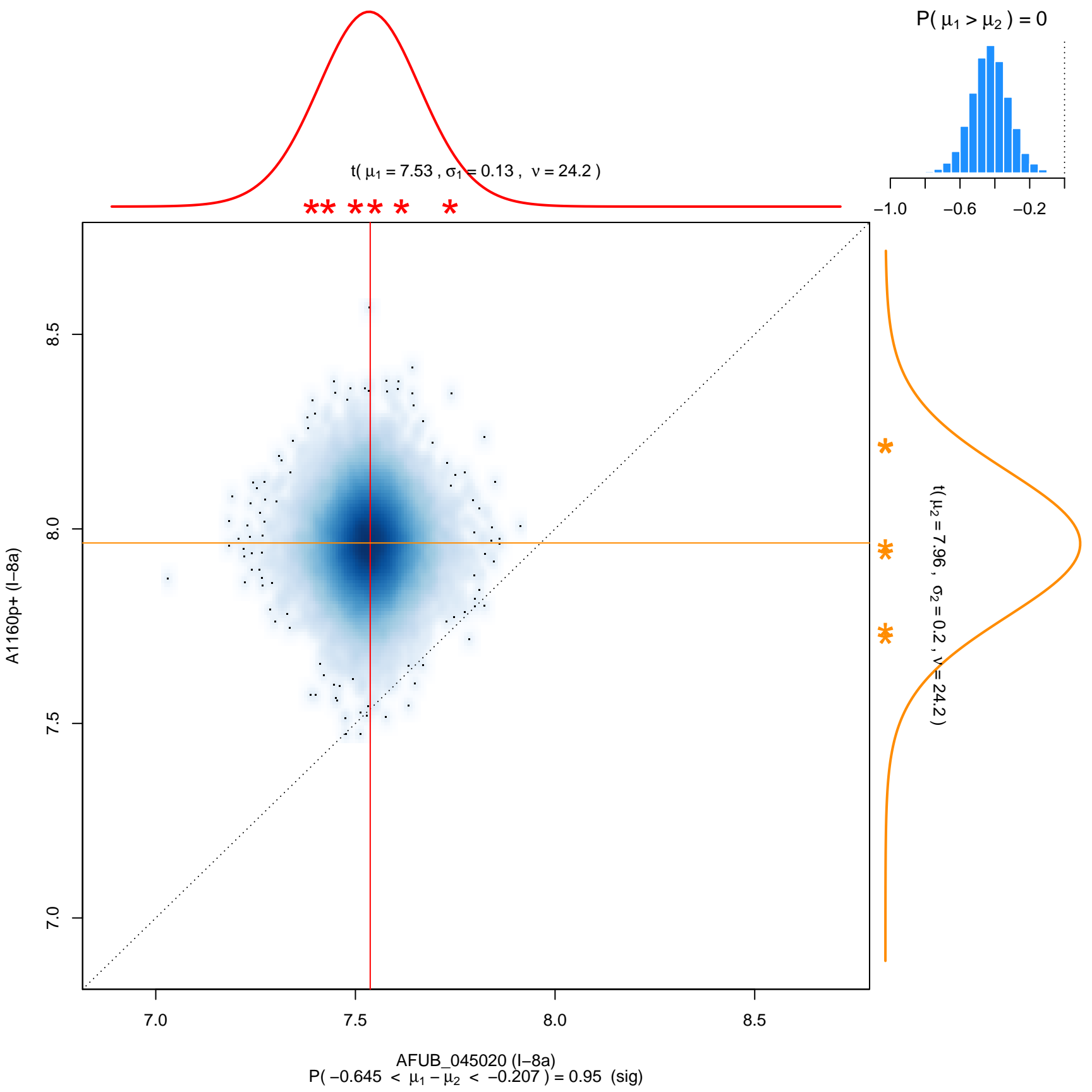

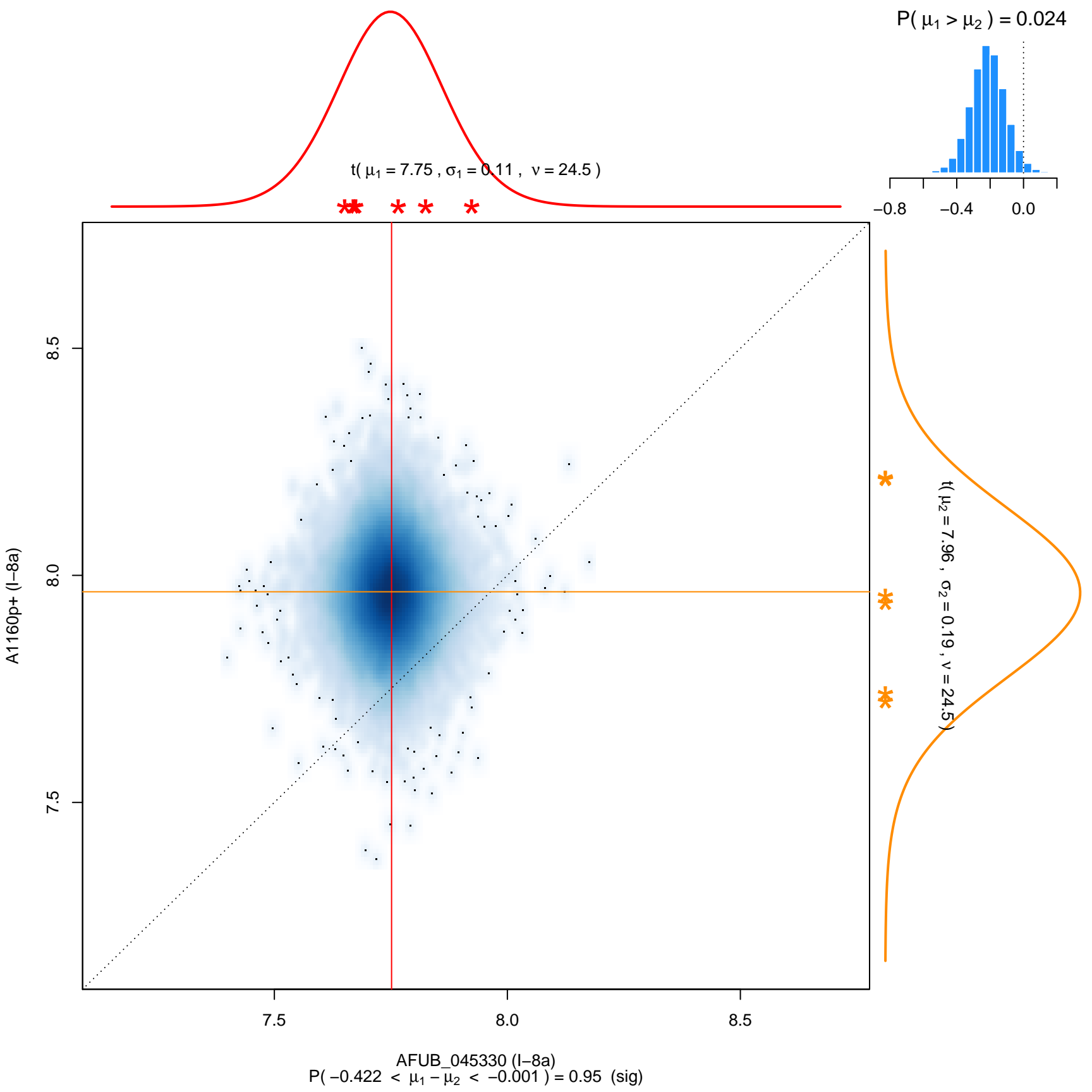

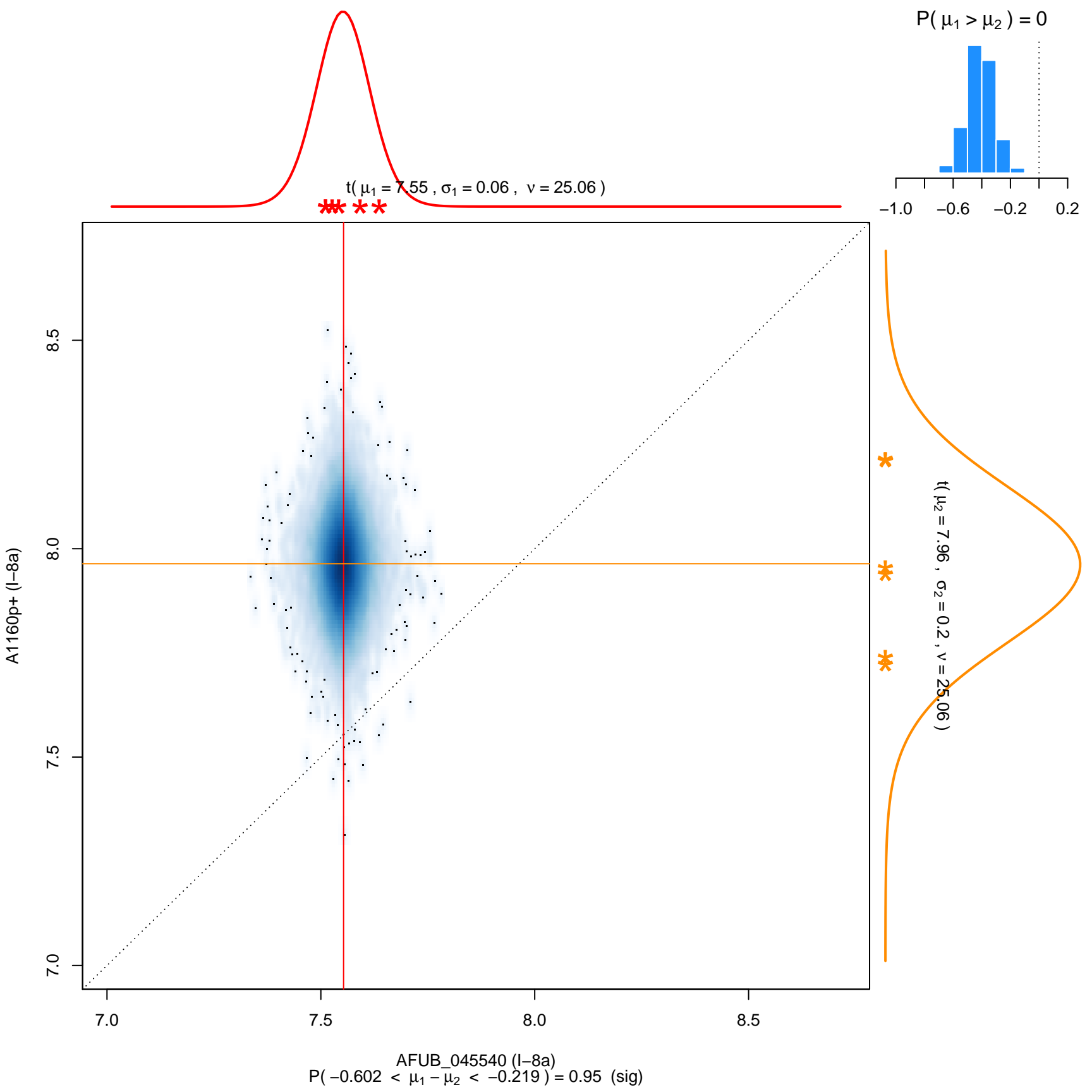

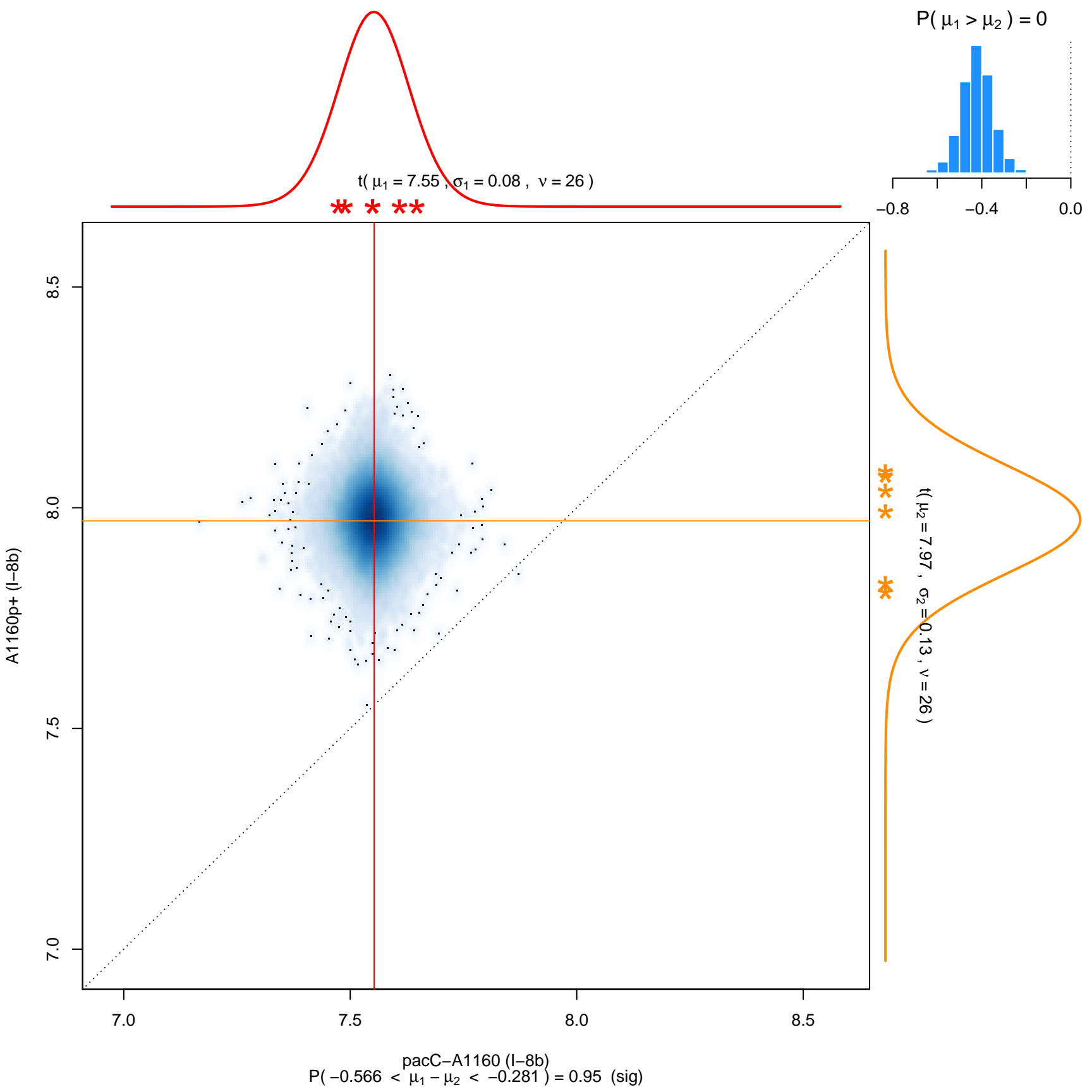

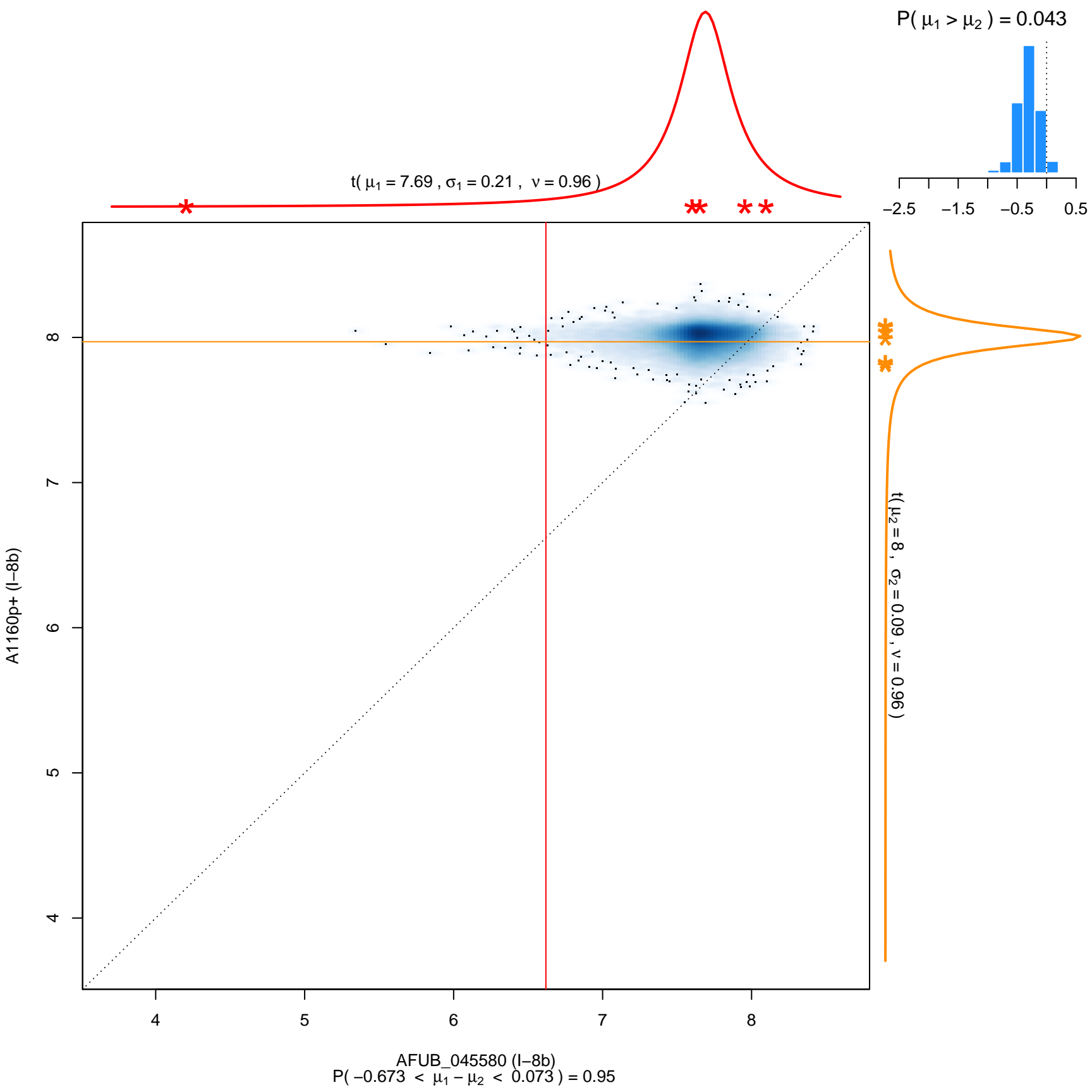

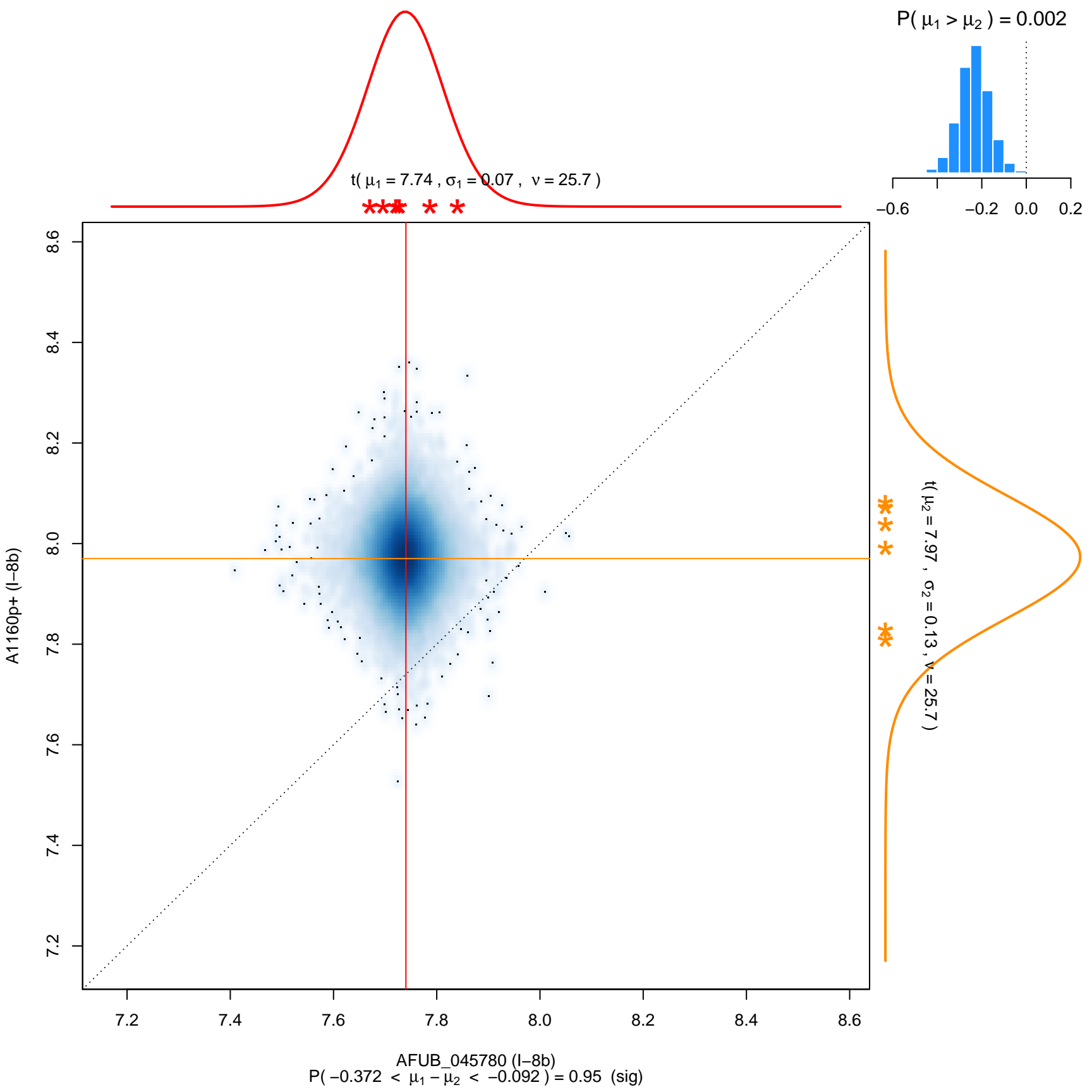

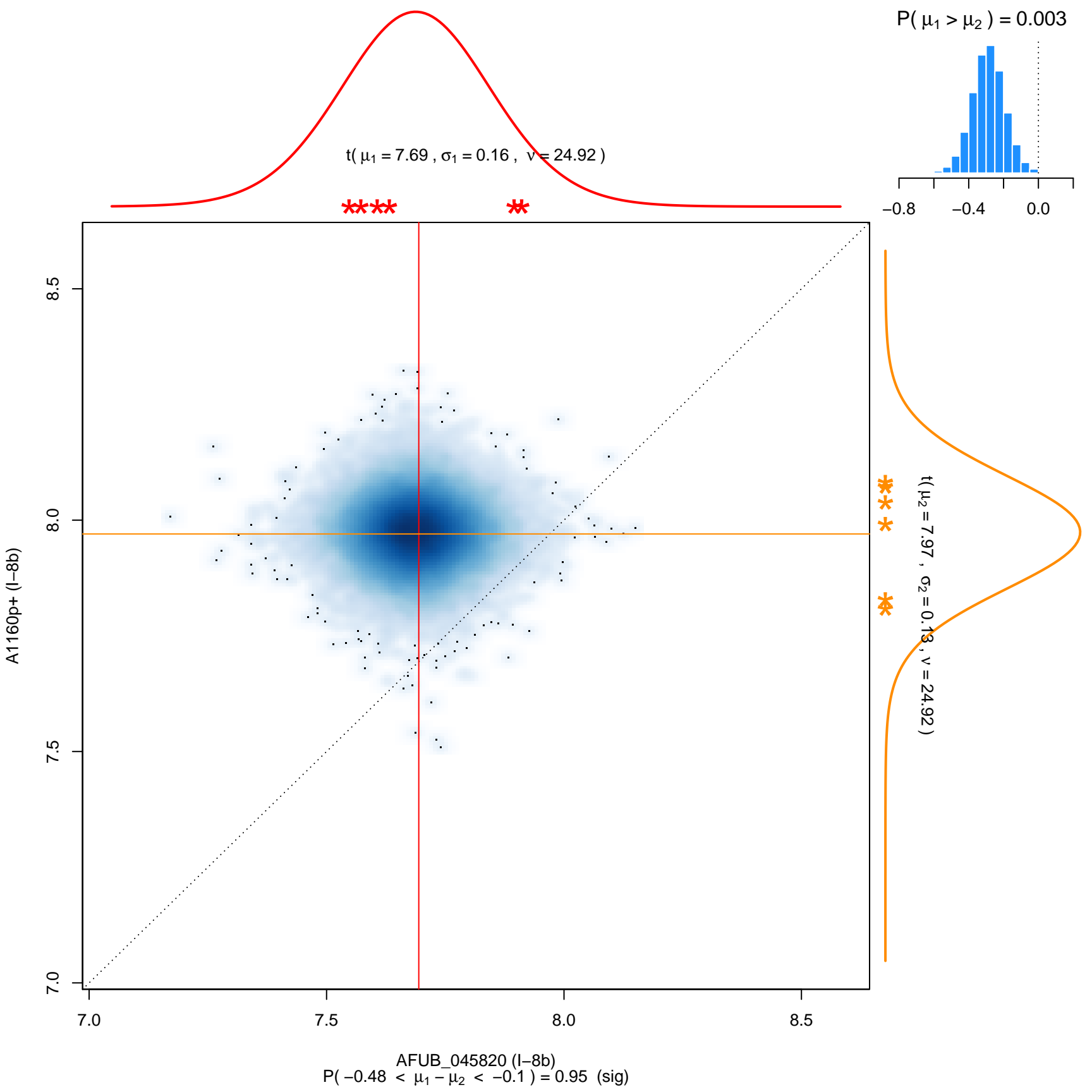

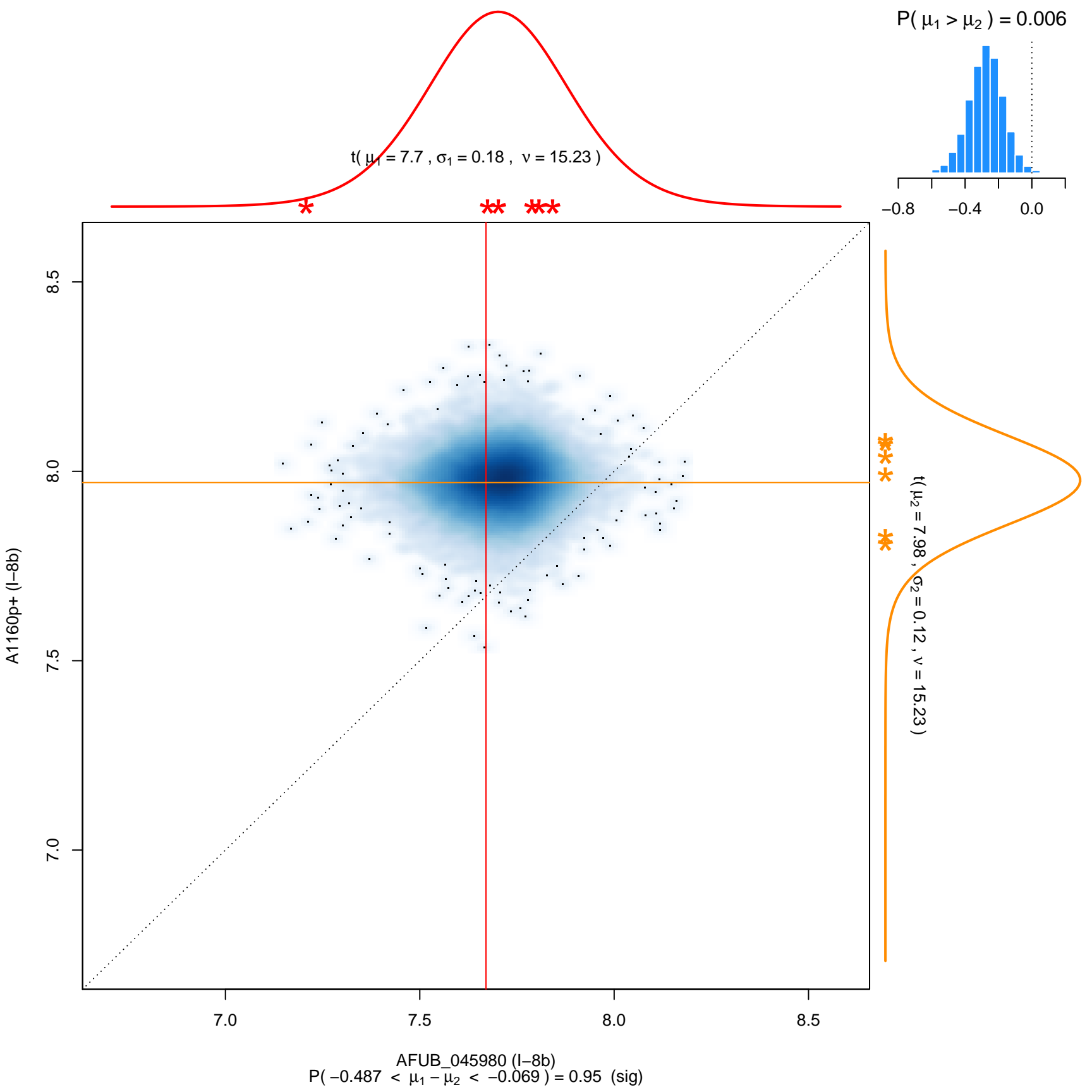

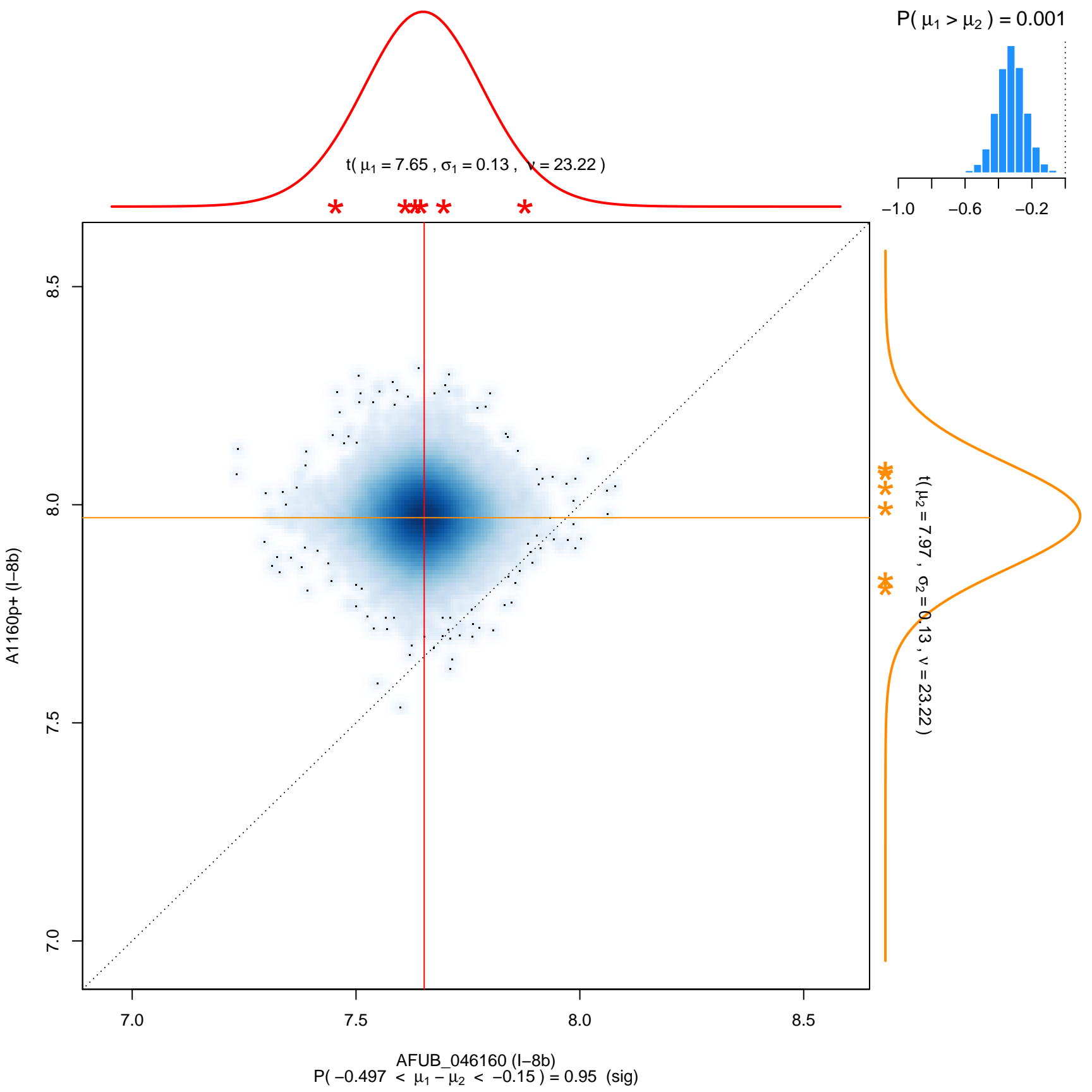

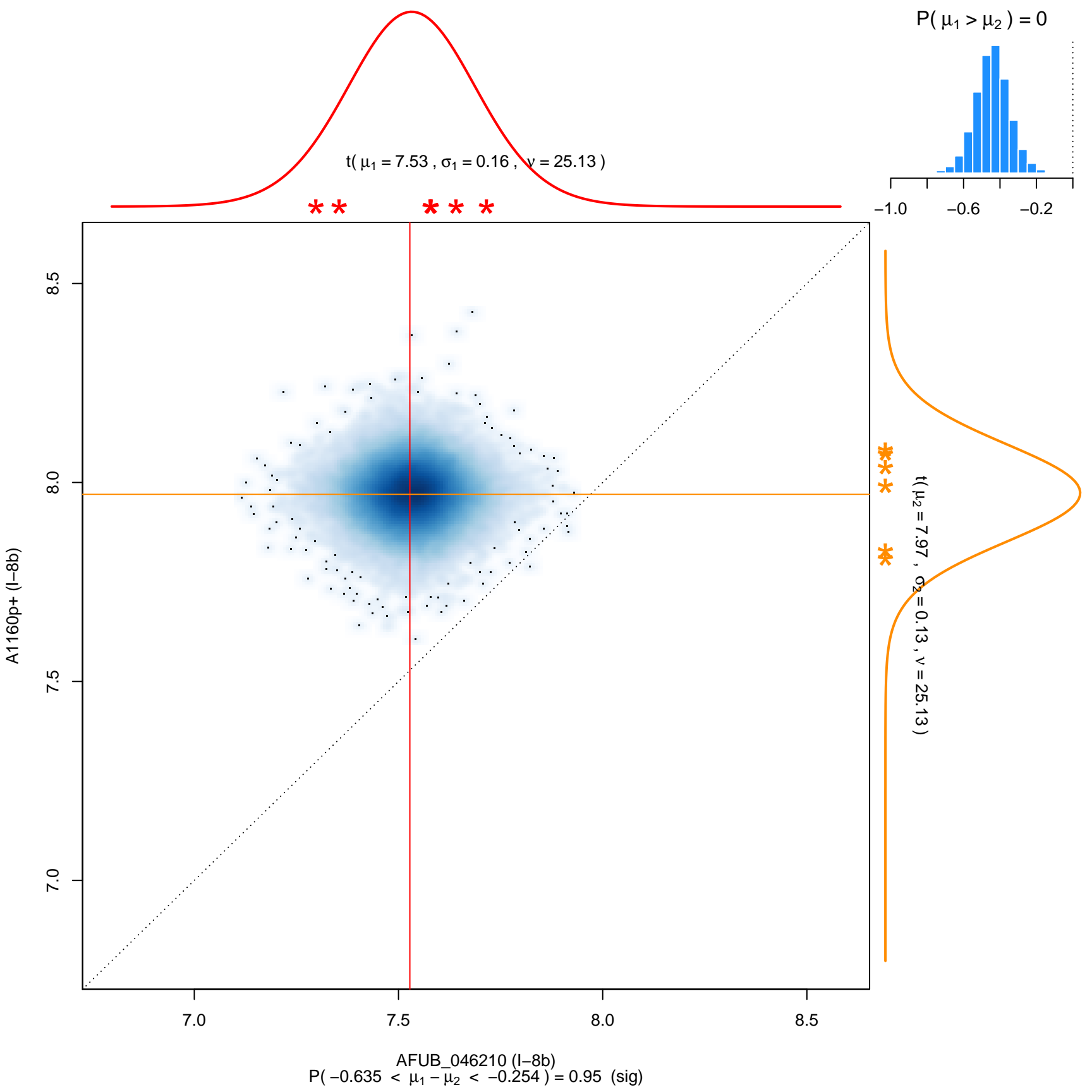

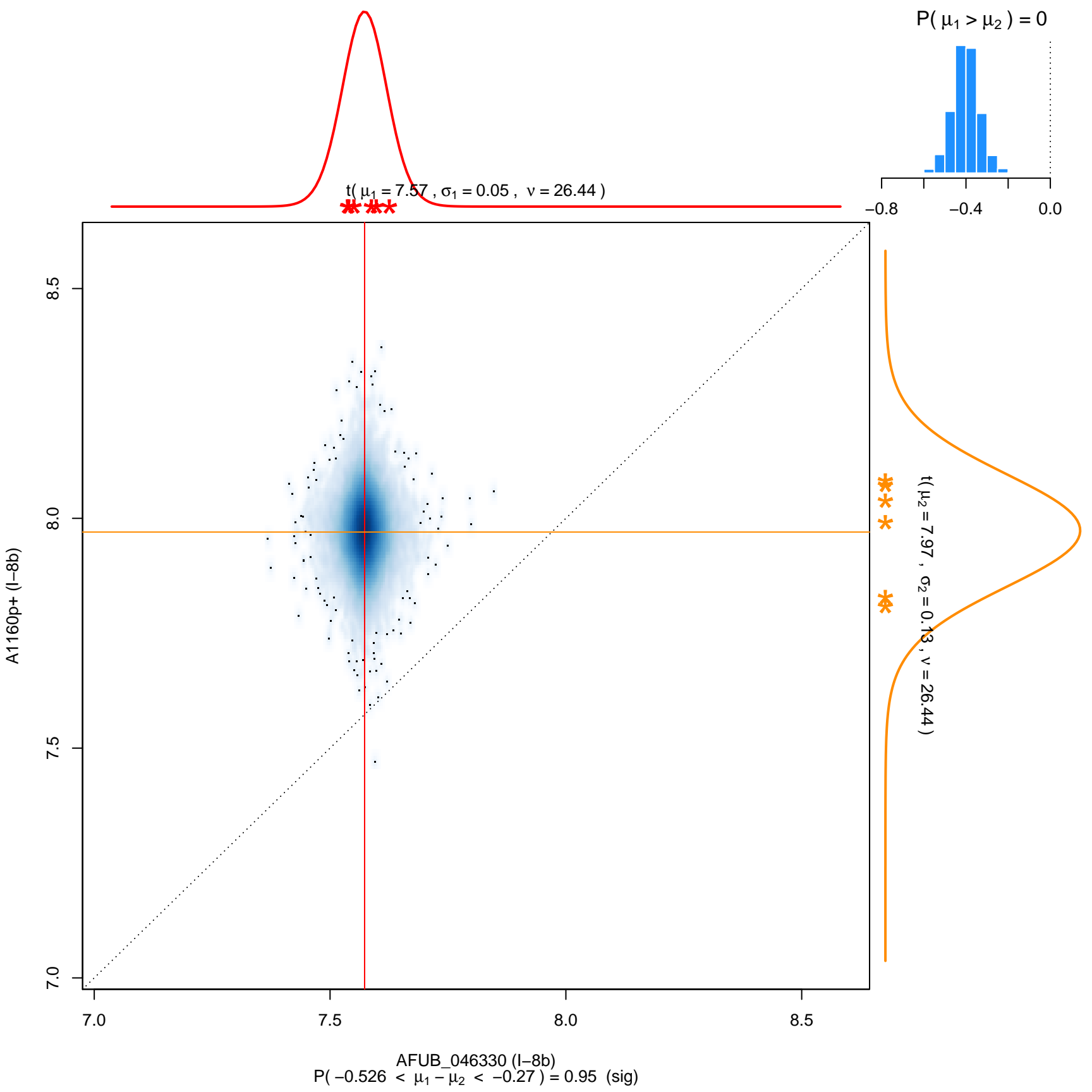

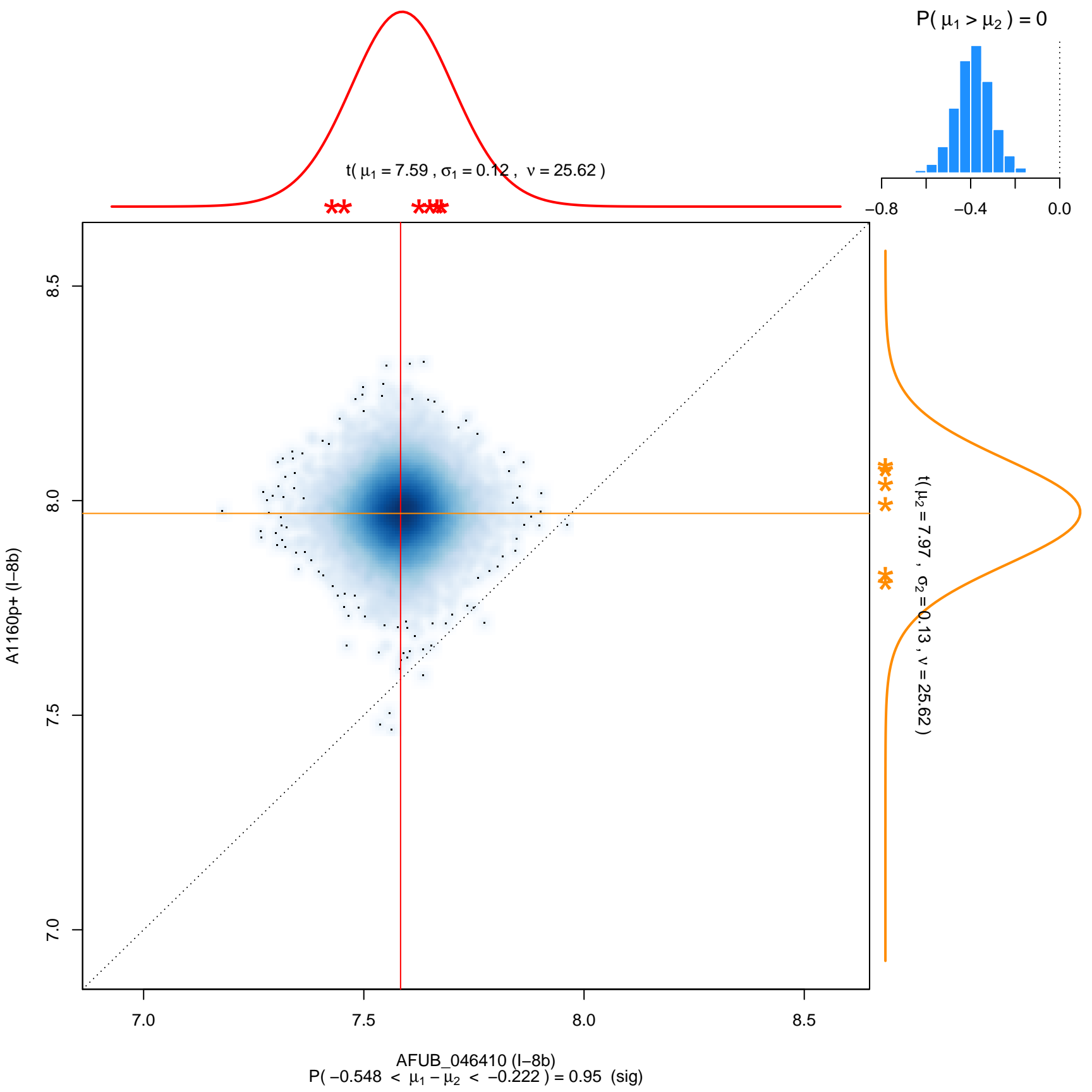

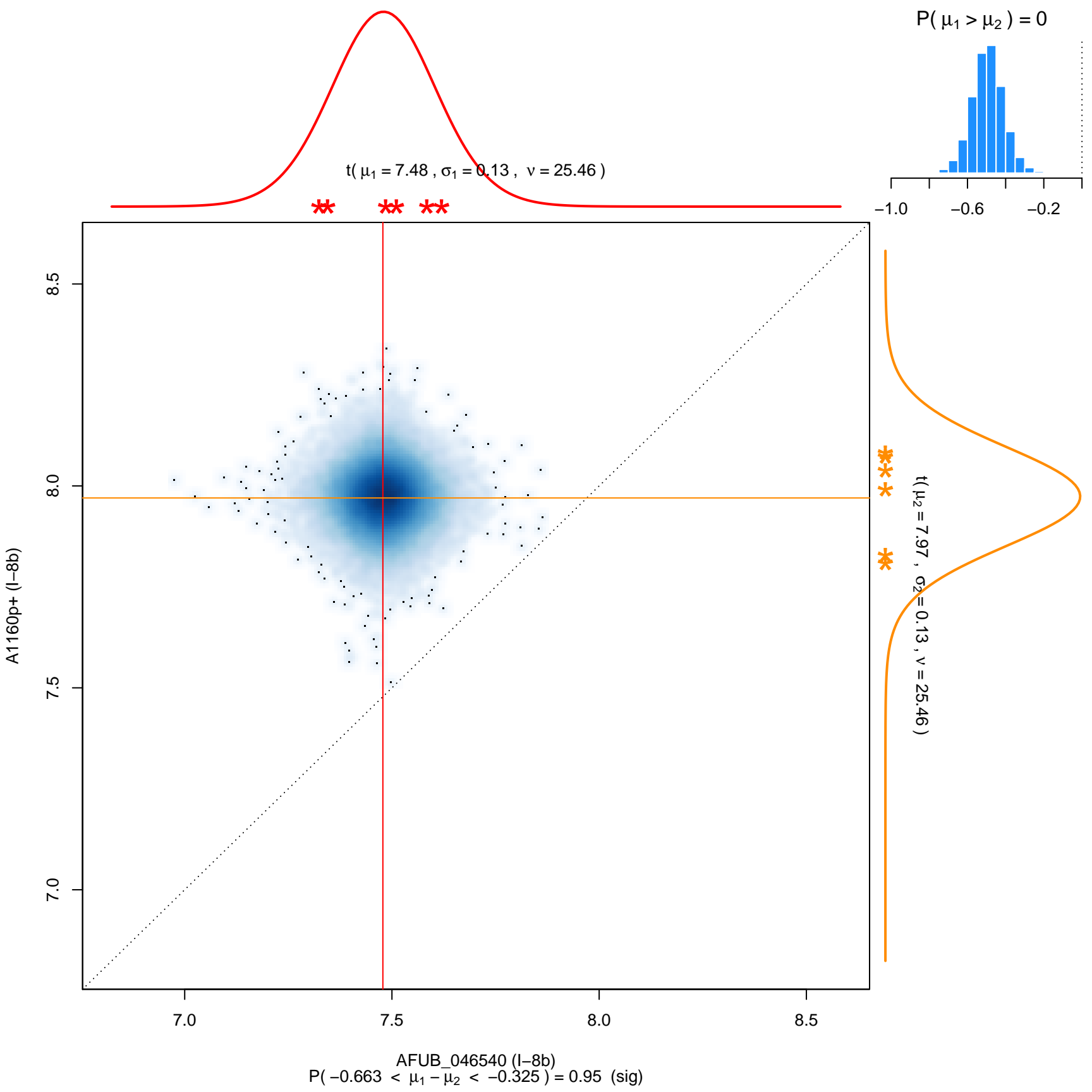

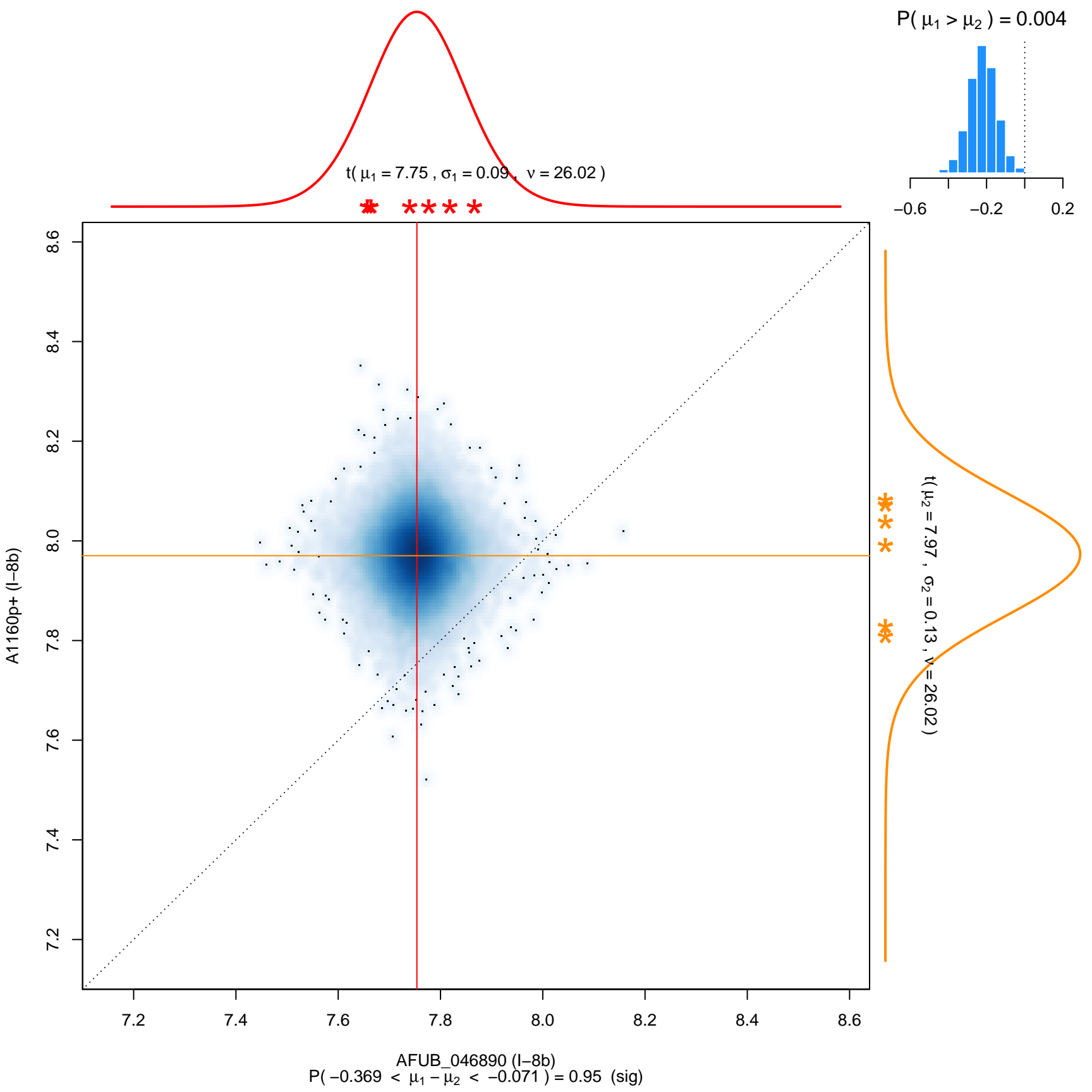

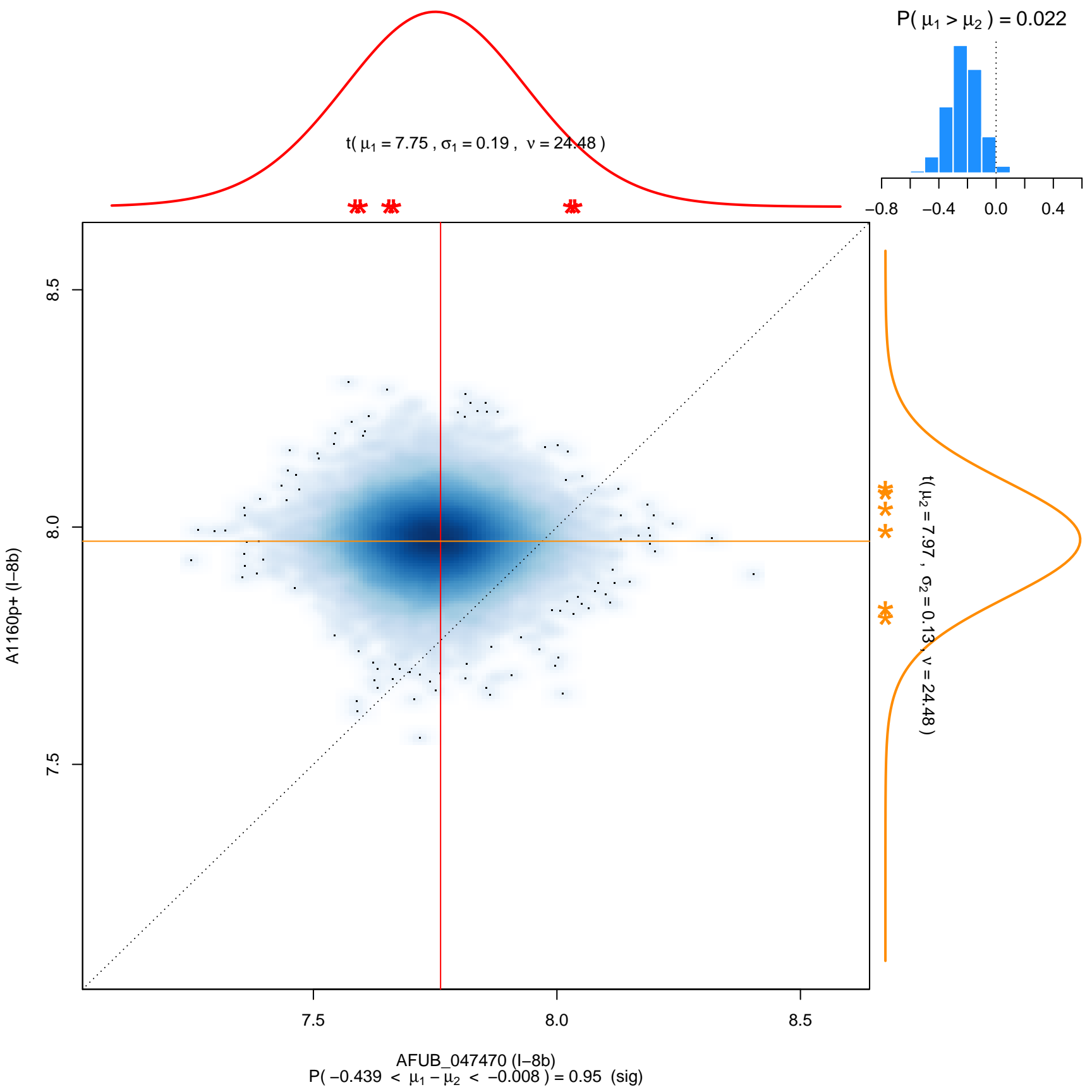

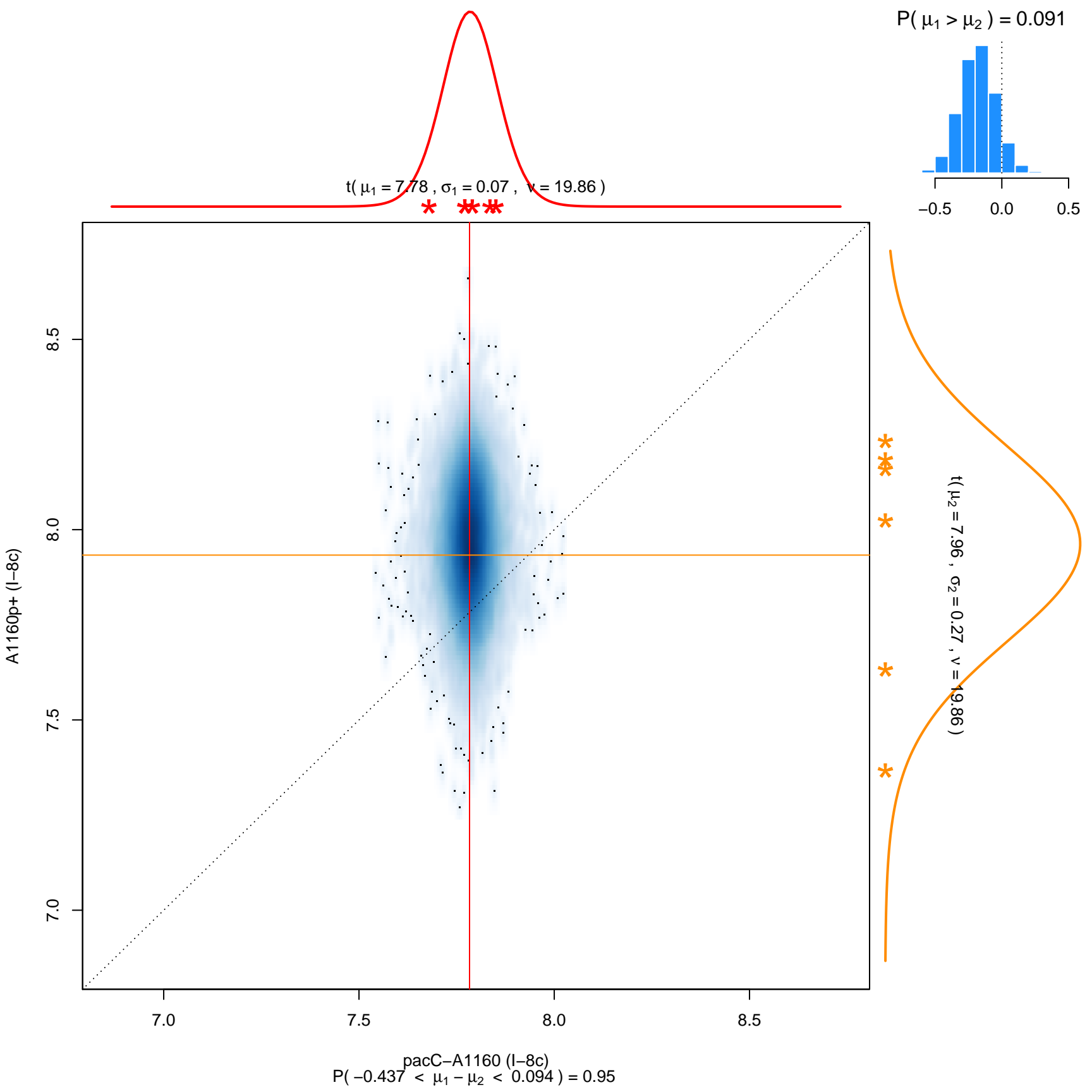

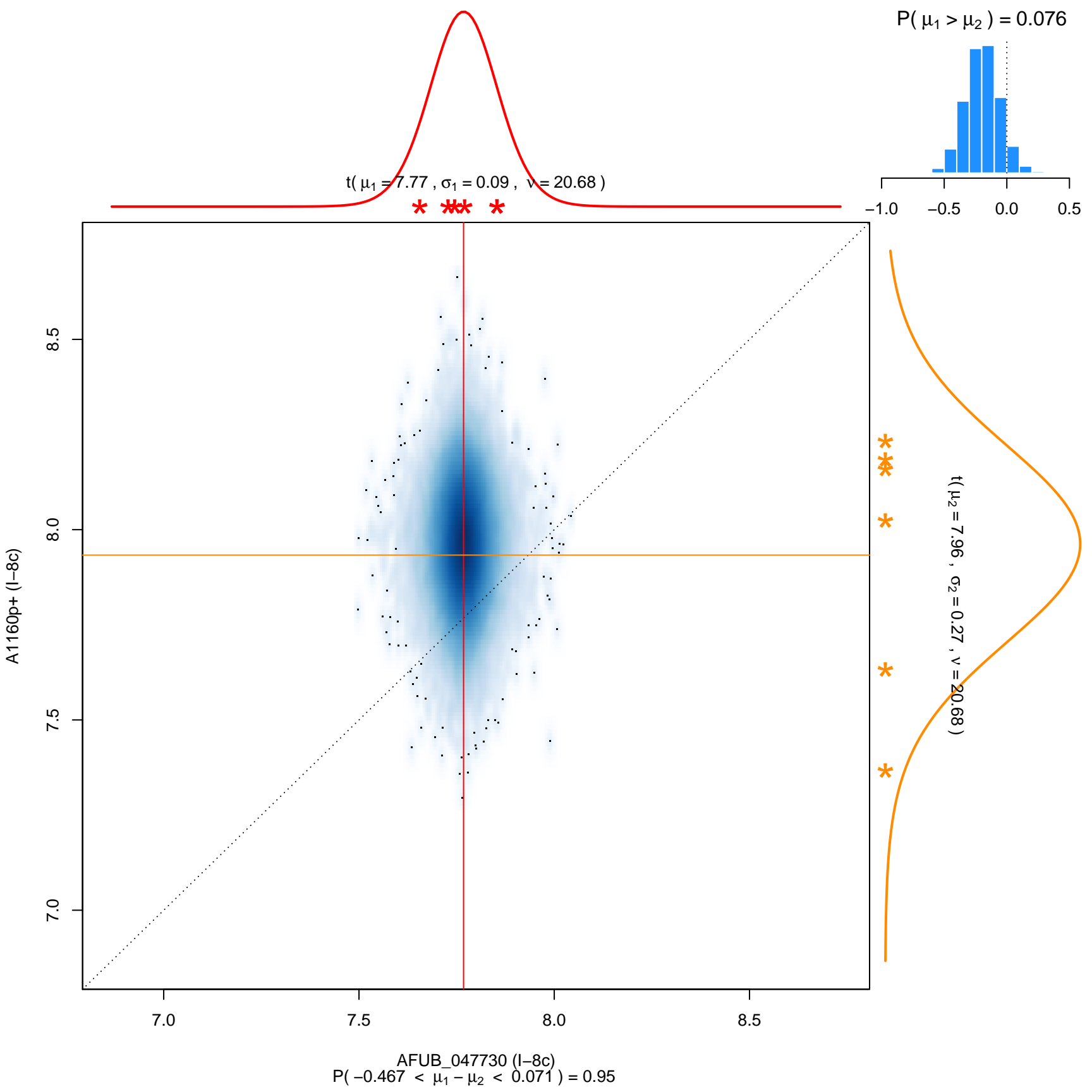

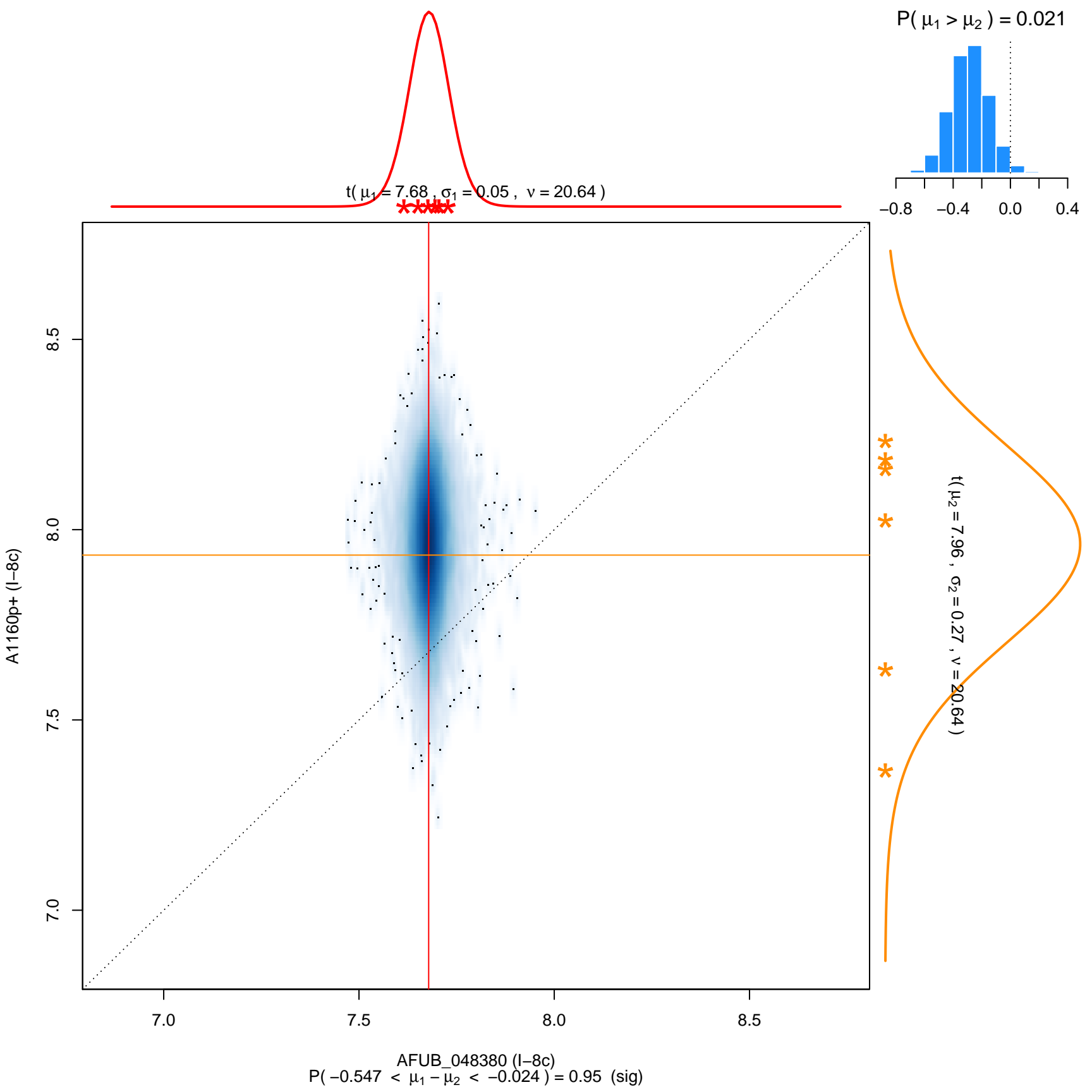

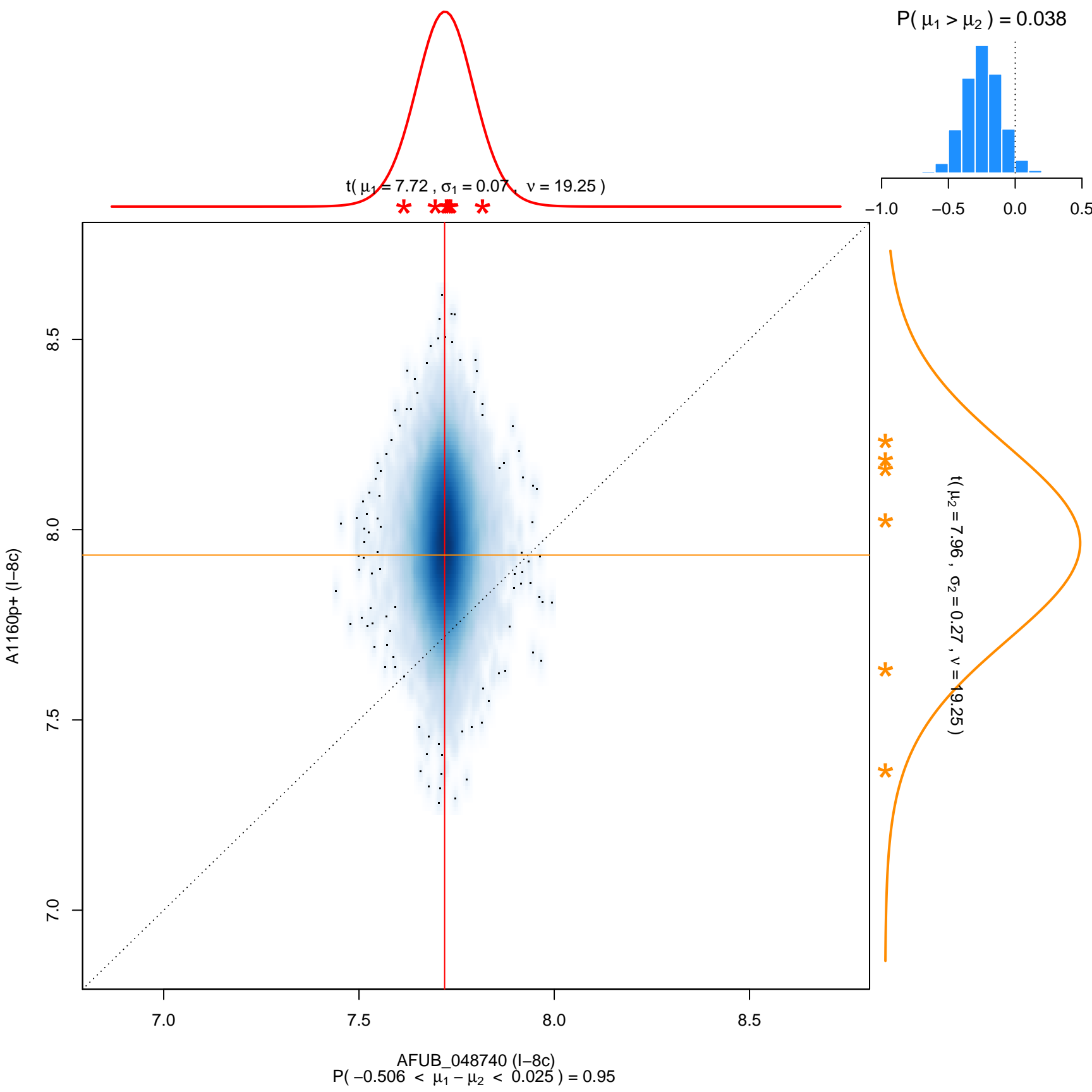

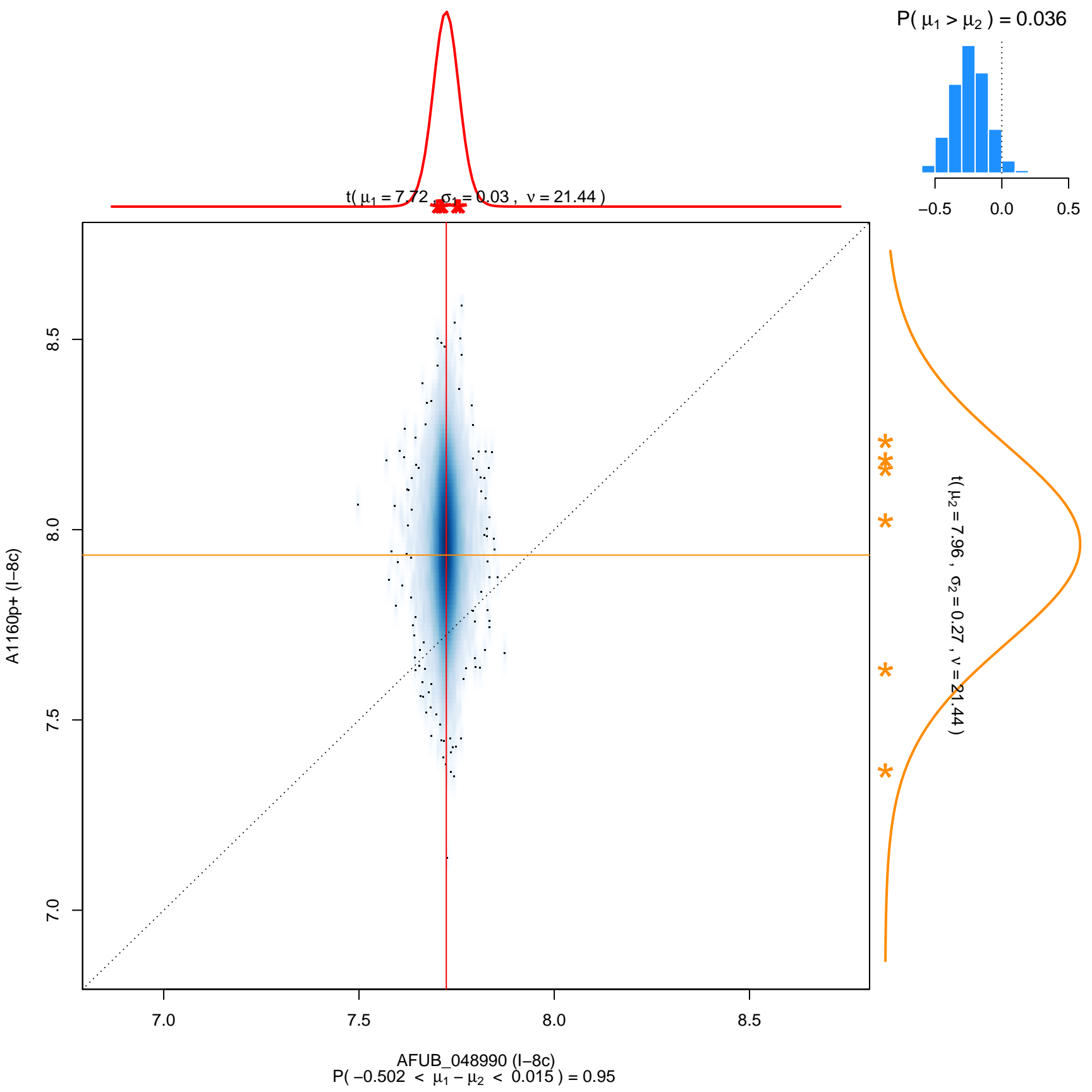

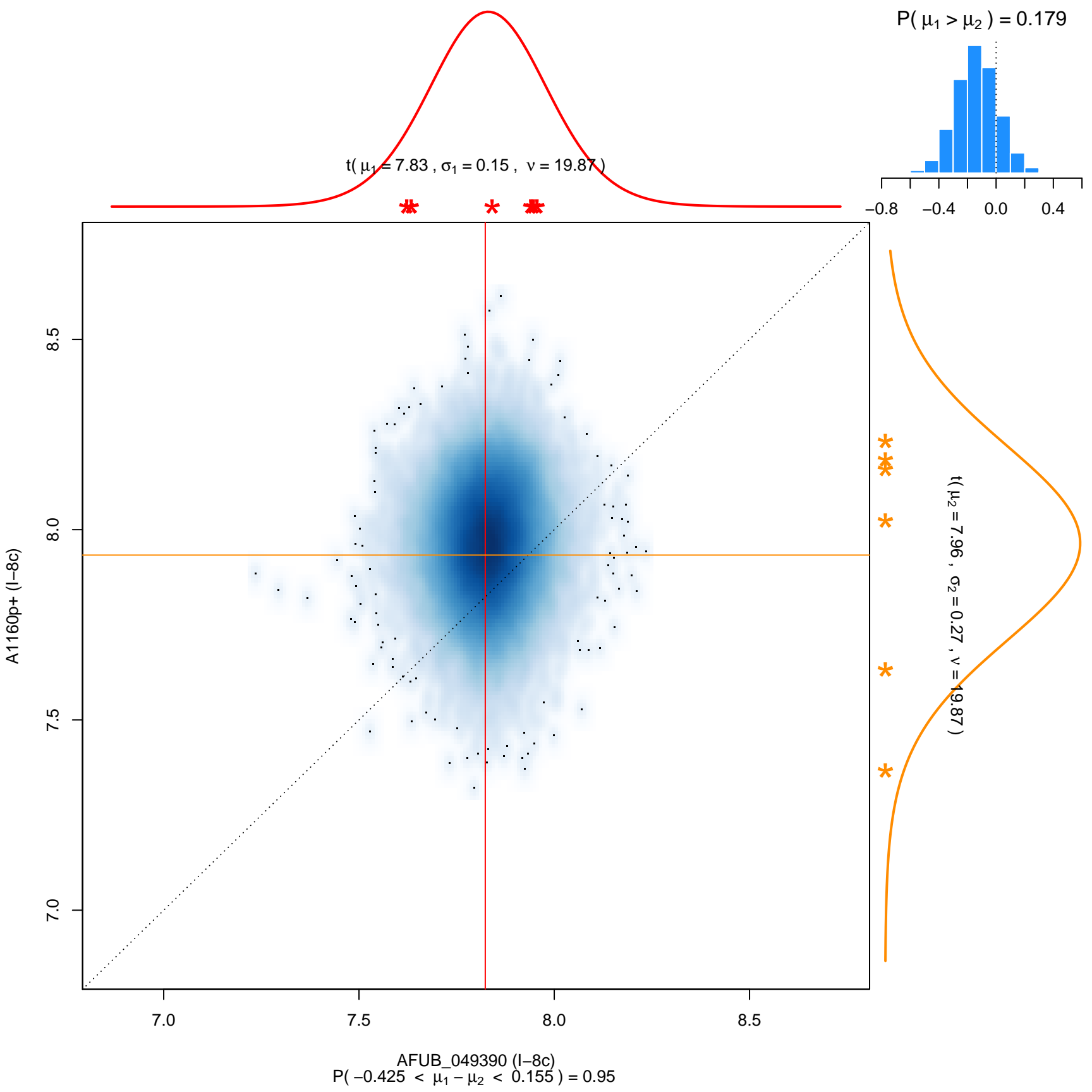

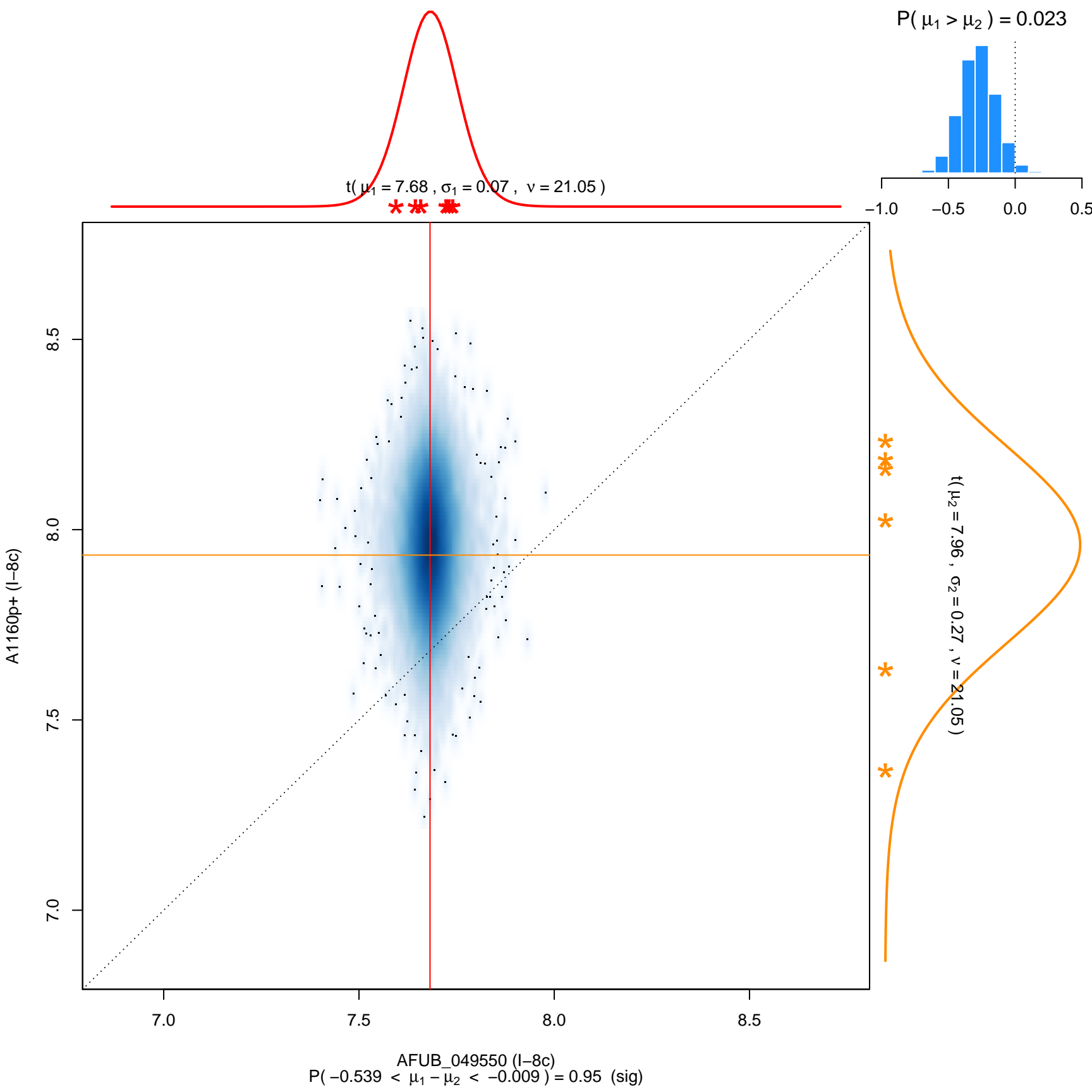

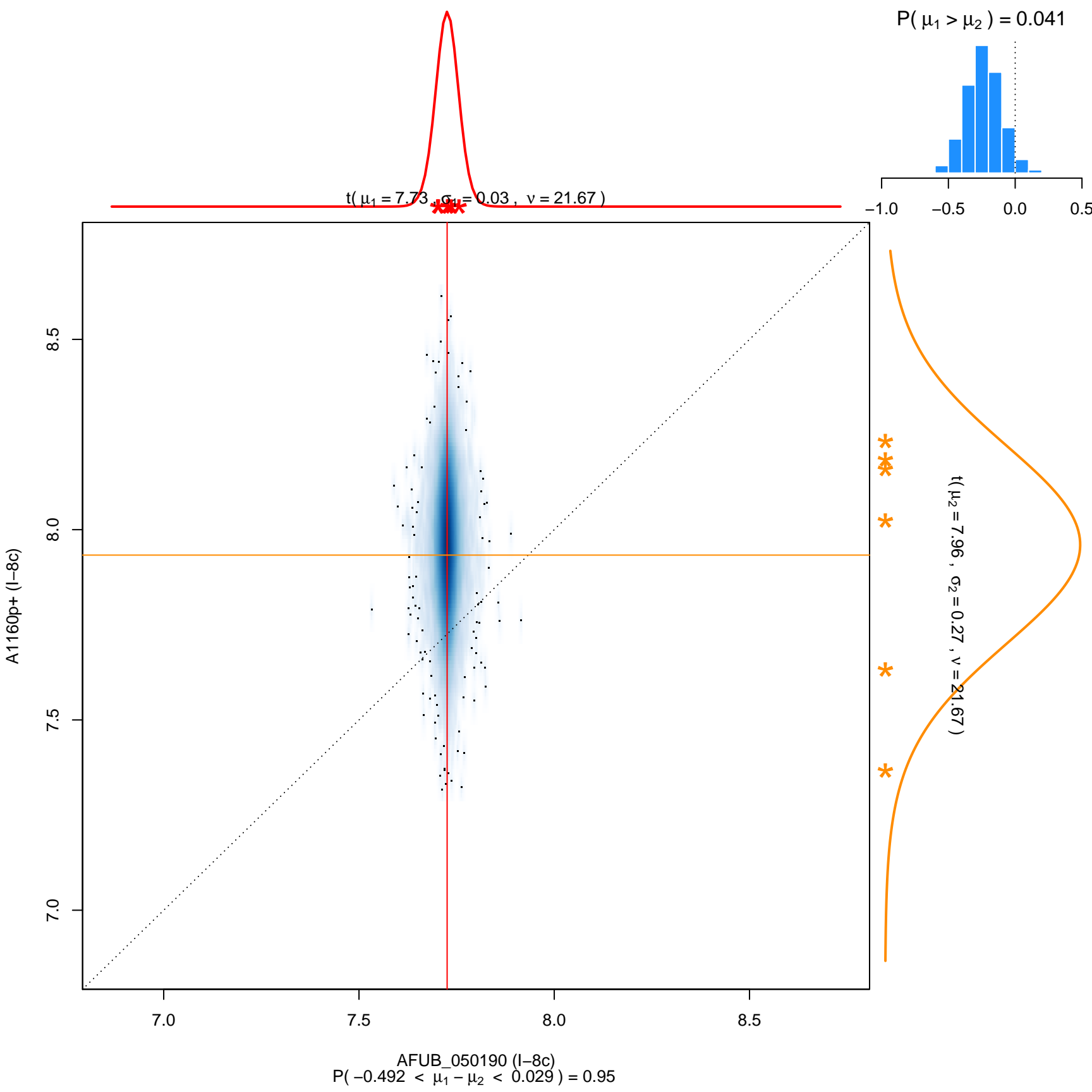

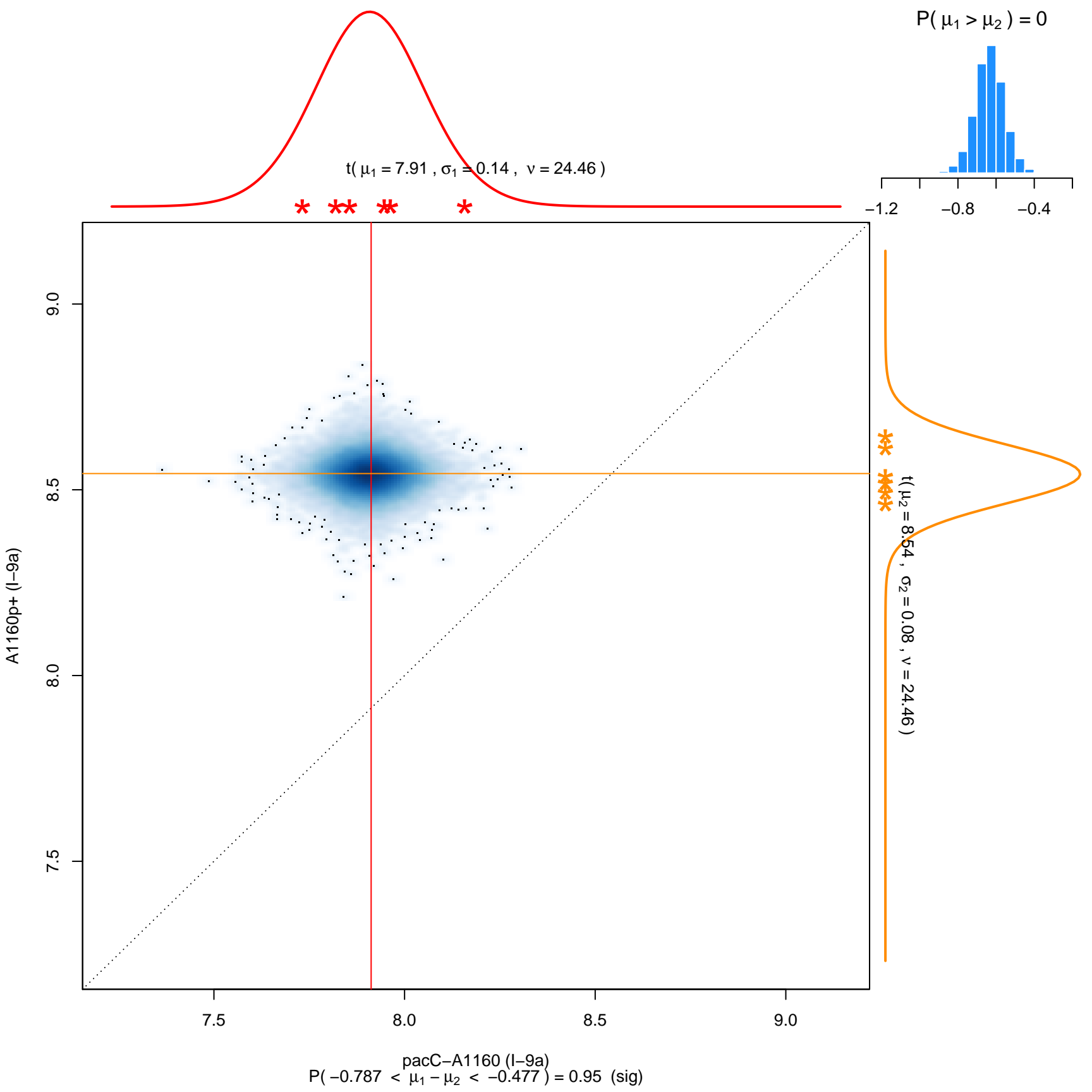

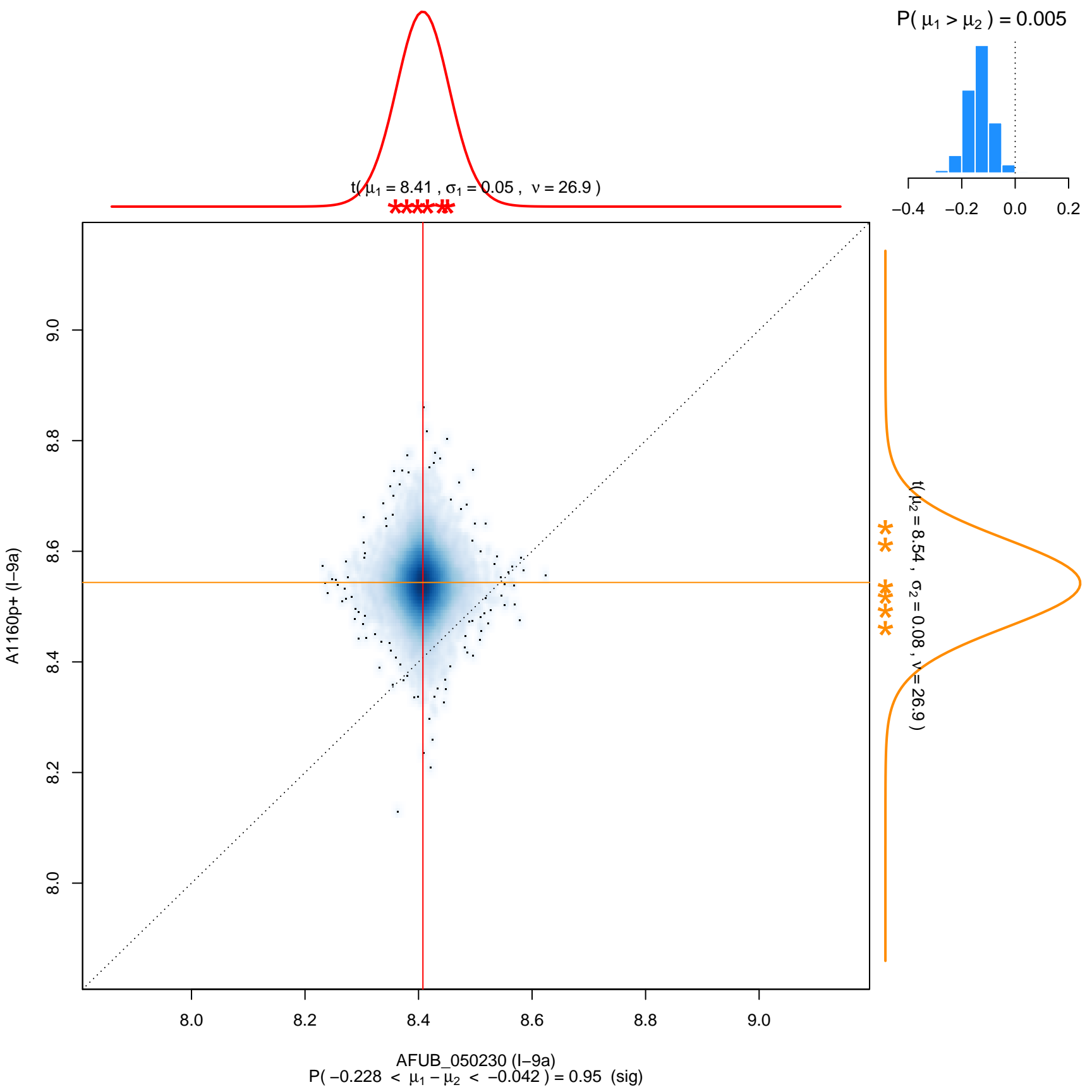

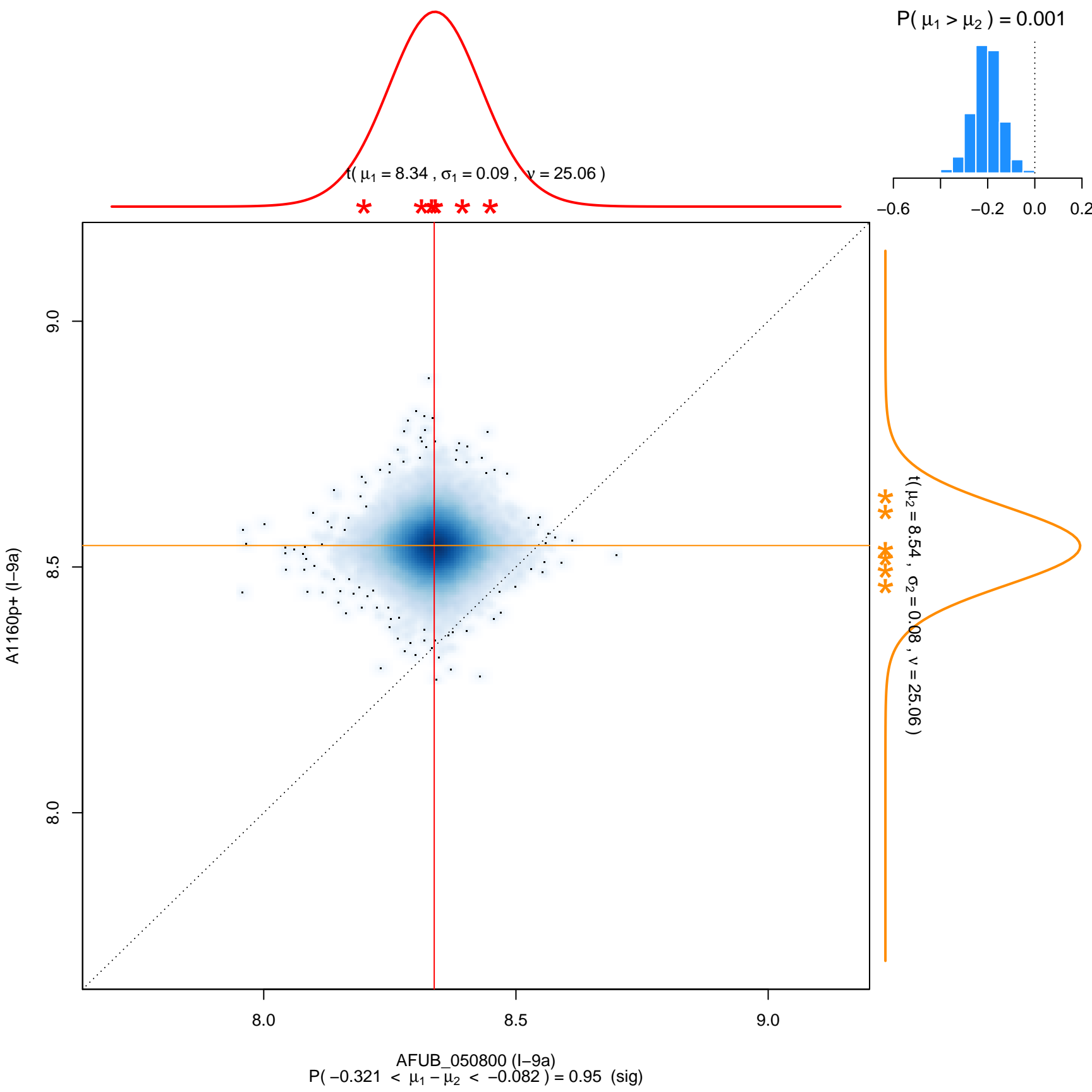

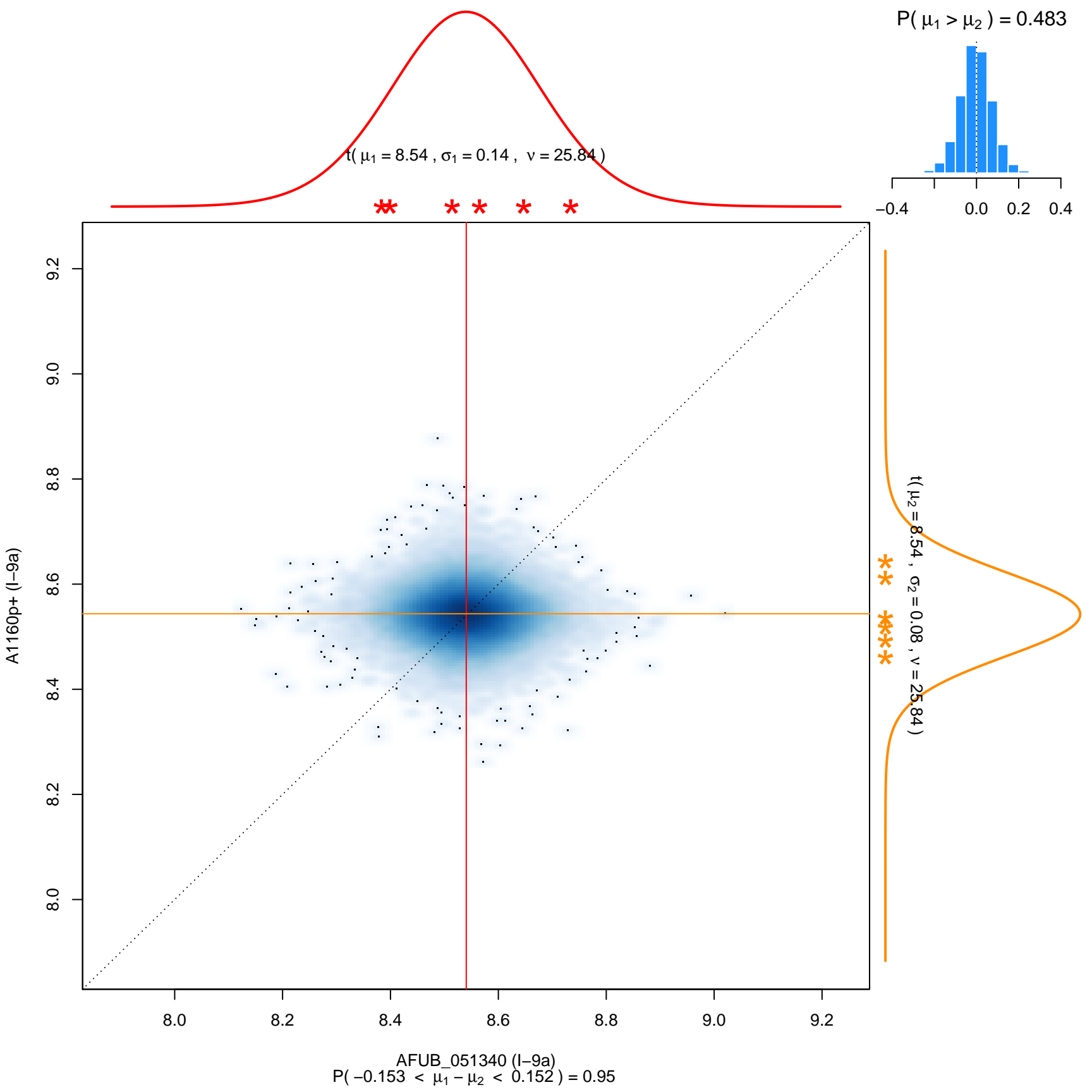

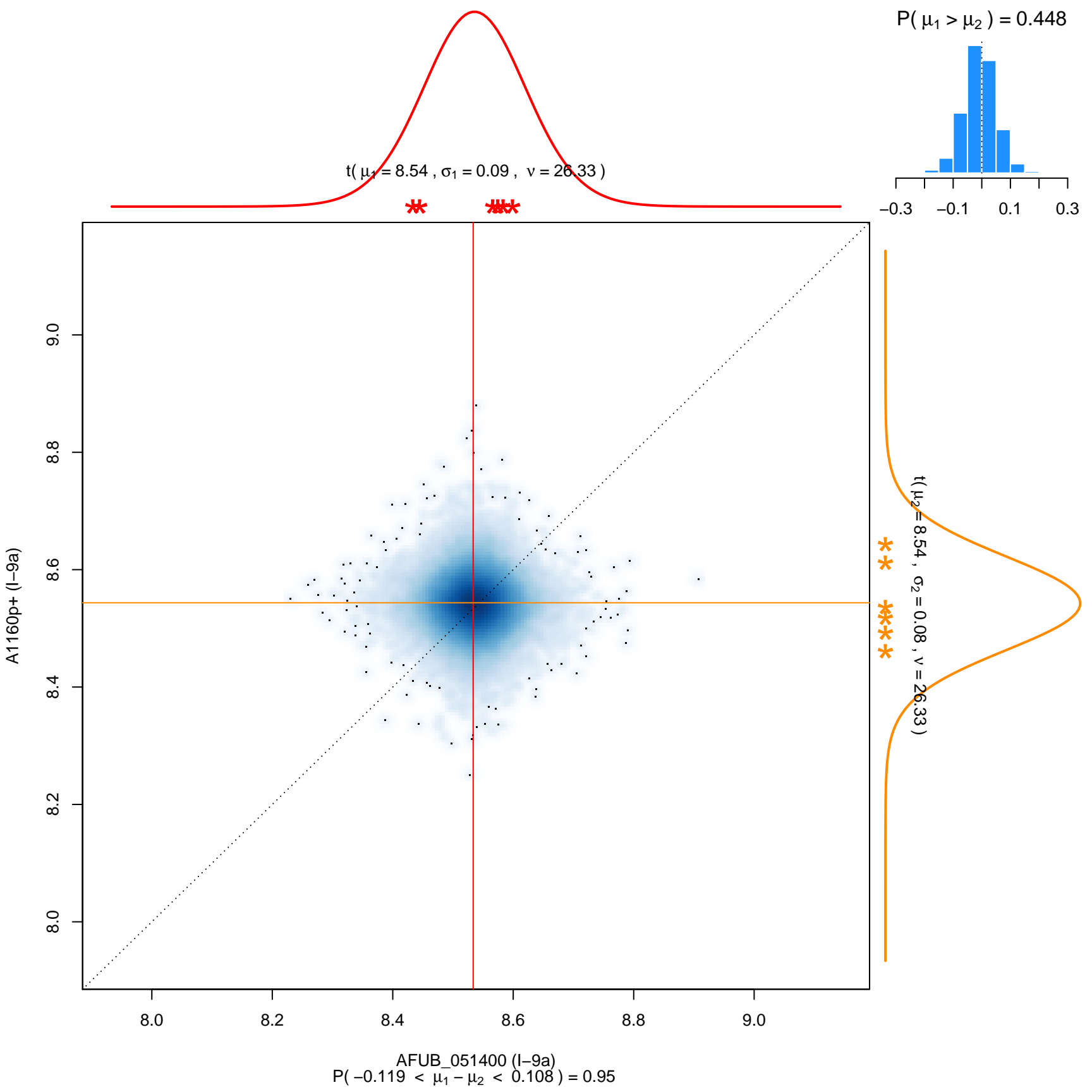

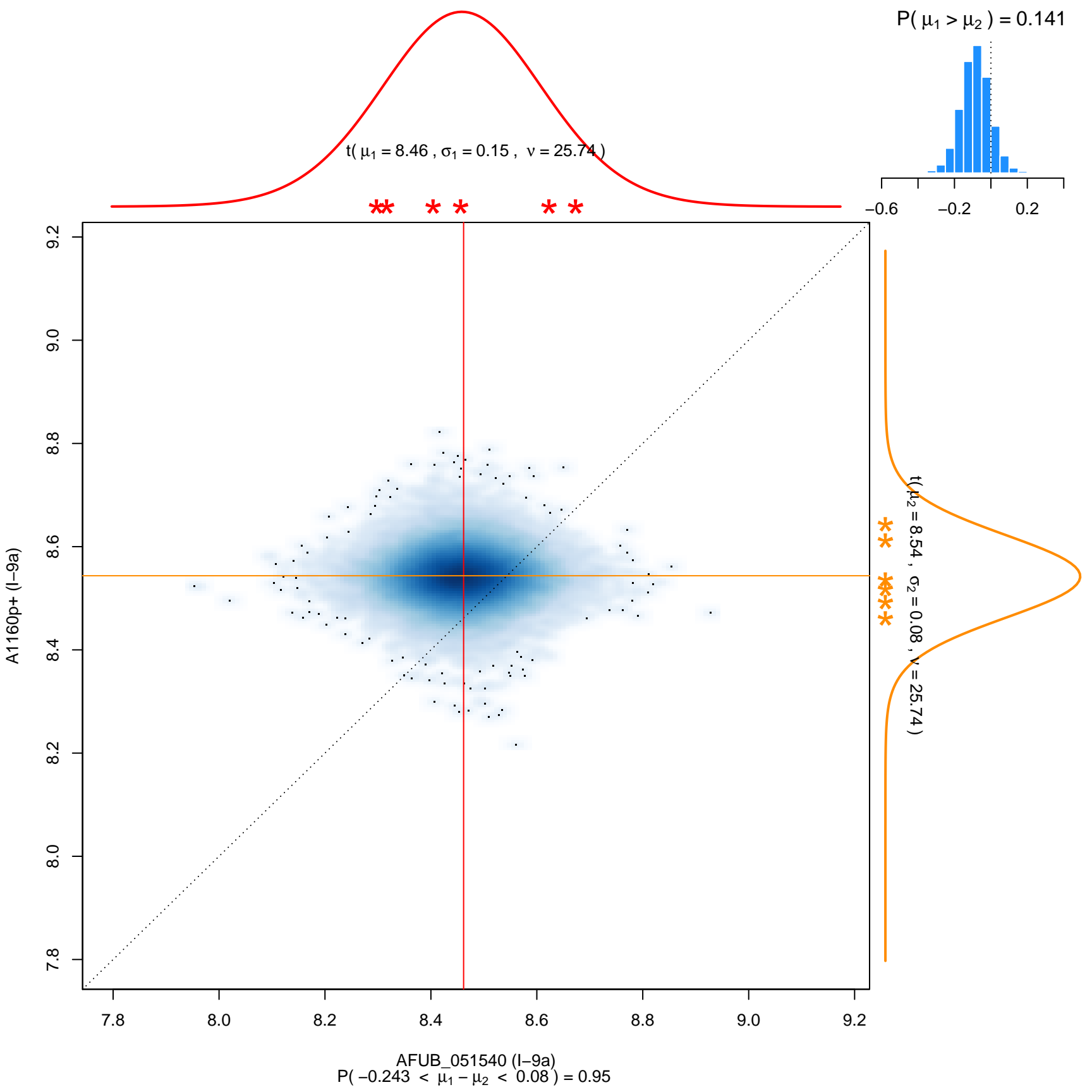

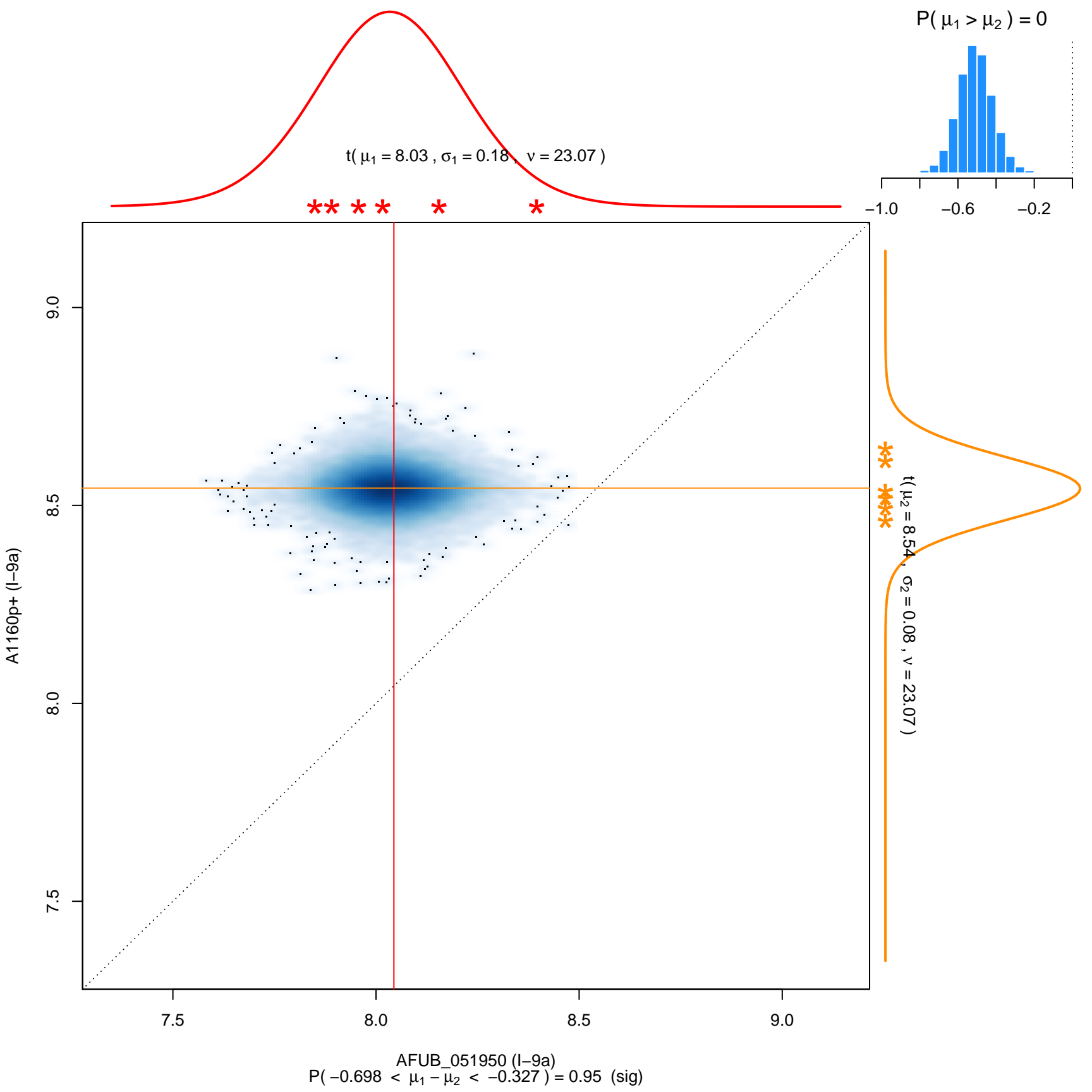

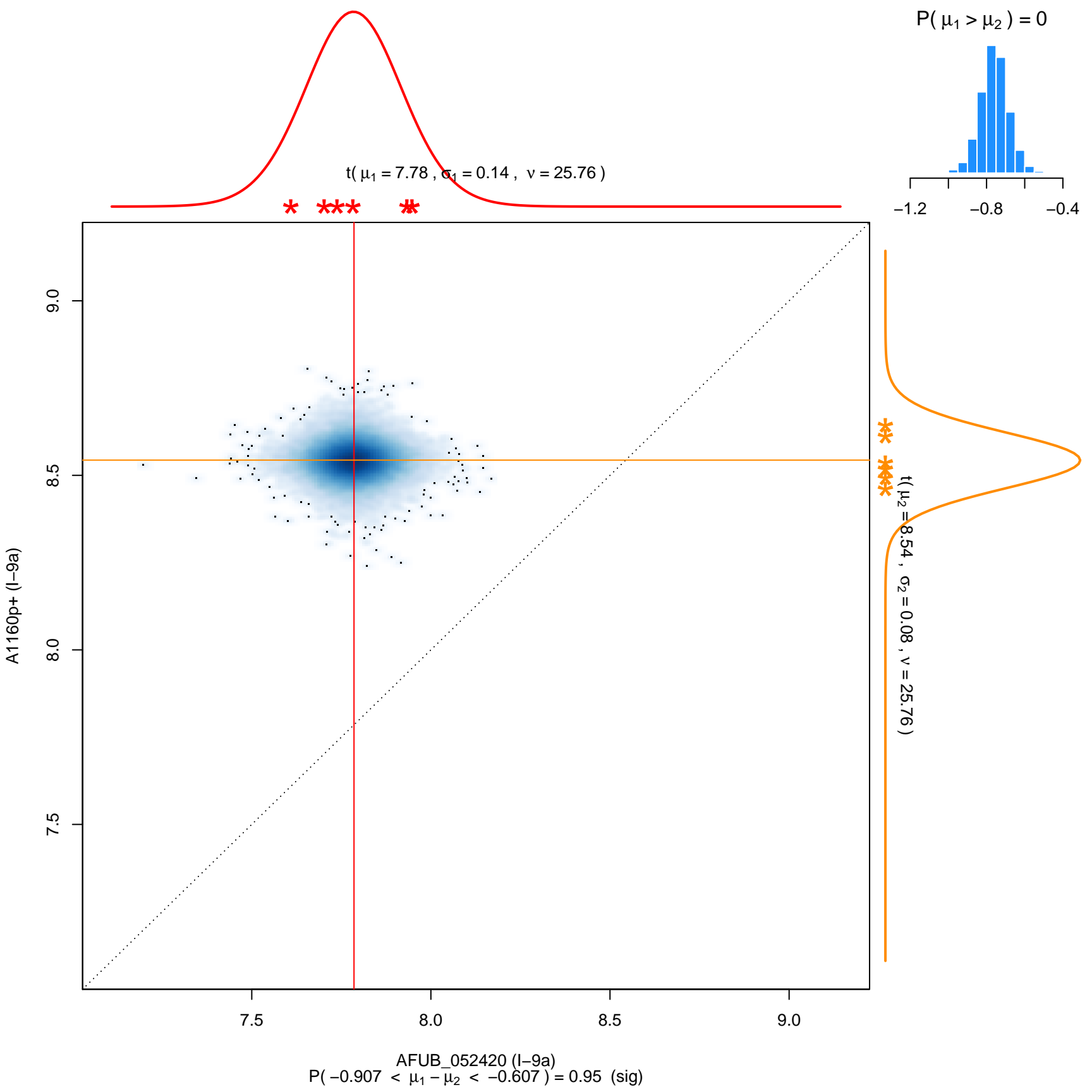

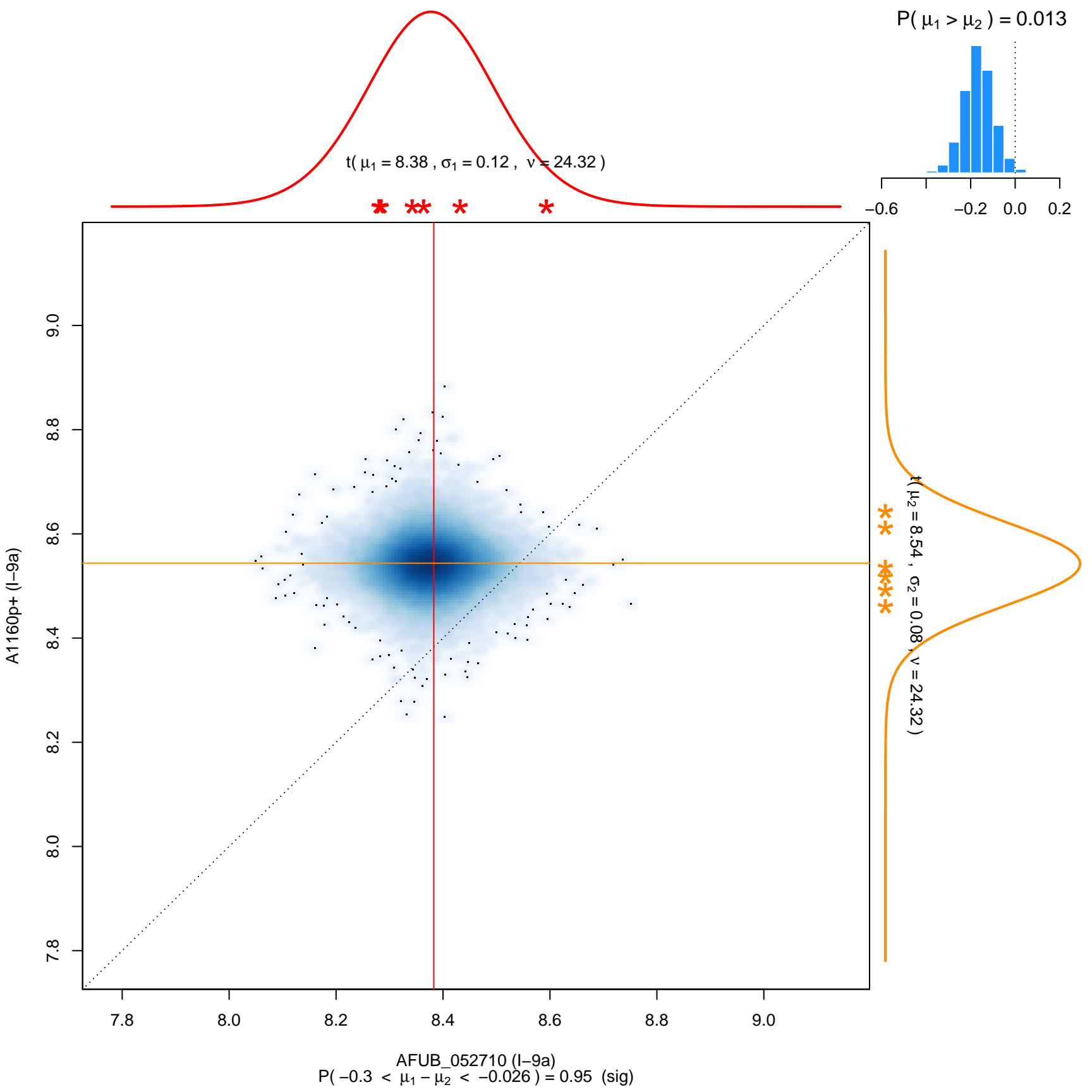

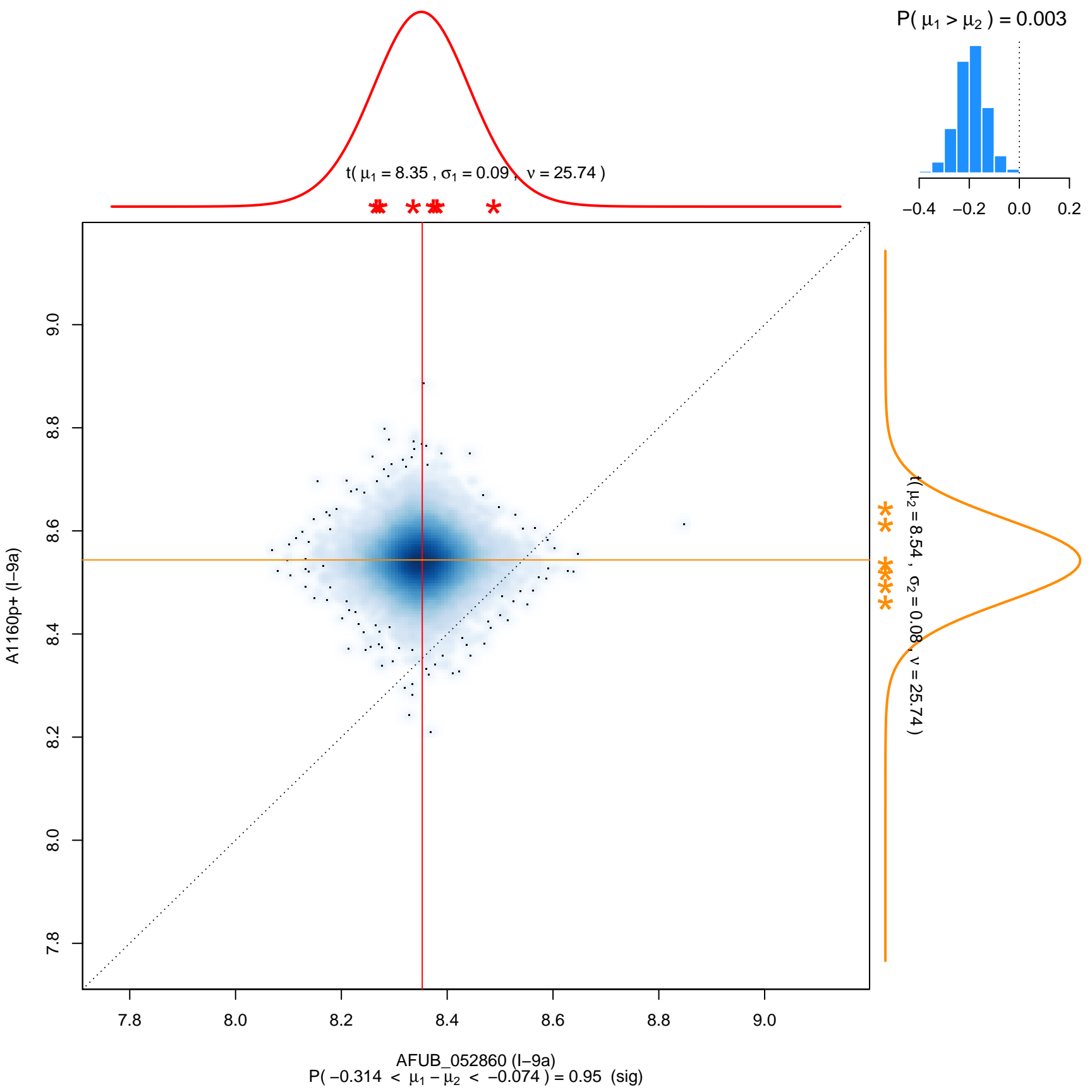

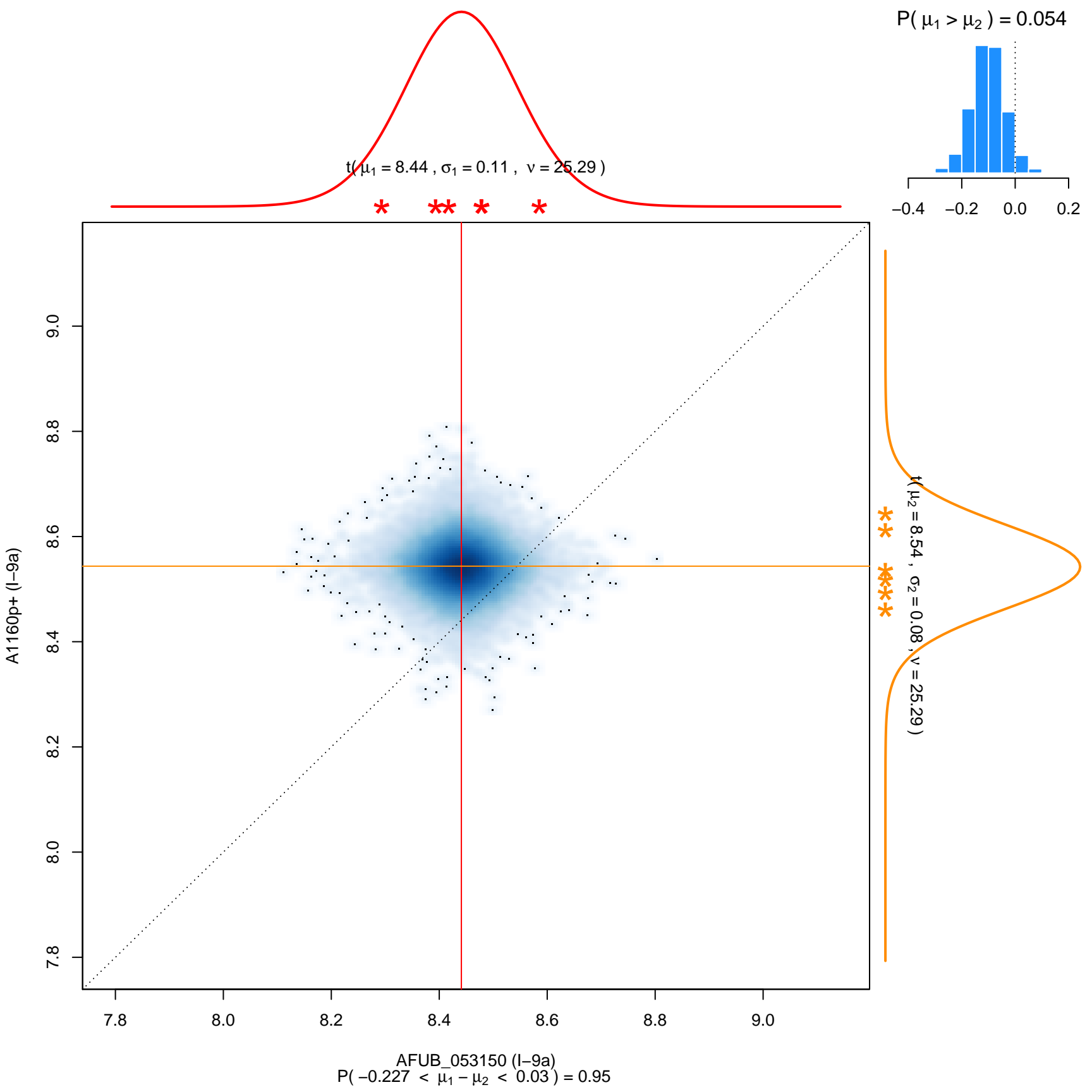

Supplement: Supplementary Table 2 — Output of the Boolean analysis performed for the LDH epithelial cell lysis screening for the TFKO mutants. [file DataSheet_2.pdf]

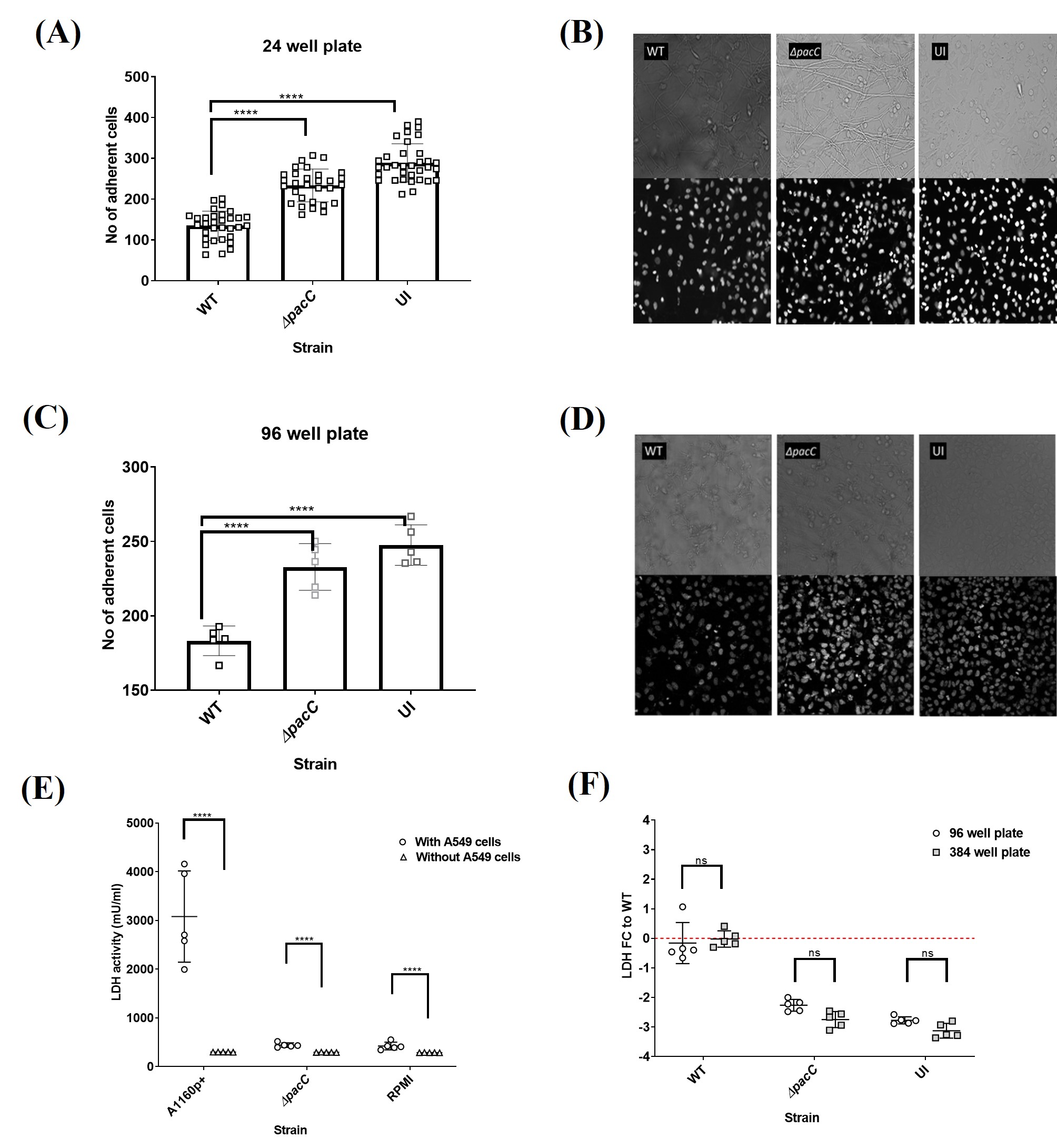

Supplement: Supplementary Figure 1 — Optimisation of epithelial damage assays for high throughput screening. Performance of detachment assay in a low throughput (A, B) and high throughput plate format (C, D) (A) Number of adherent cells per image enumerated in a 24 well plate. The total number of adherent cells per field of view were enumerated using a FIJI macro counting DAPI stained cells after A. fumigatus A1160+ and ΔpacC strains infection. Data was analysed by multiple t-tests. ****P<0.0001. (B) Representative images A1160p+ or ΔpacC after 16-hour infection of A549 monolayer in a 24 well plate. Images captured manually at 20 x magnification using NIKON TE-2000E (C) Number of adherent cells per image enumerated in a 96 well plate. The total number of adherent cells per image were enumerated using a FIJI macro counting DAPI stained cells after A. fumigatus A1160+ and ΔpacC strains infection. Data was analysed by multiple t-tests. ****P<0.0001. (D) Representative images A1160p+ and ΔpacC strains after 16-hour infection of A549 monolayer in a 96 well plate. Images captured automatedly at 40x magnification using confocal microscope. Suitability of LDH assay for high-throughput cell cytotoxicity screening (E, F) (E) A. fumigatus does not produce measurable LDH enzyme at 24 hours. Absolute LDH released by 500,000 spores of A1160p+, ΔpacC and un-infected in a 24 well plate after 24 hours challenge with and without A549 cells. (F) Performance of LDH assay in 96 versus 384 well format. LDH assay performed in a 96 well and a 384 well plate showing LDH fold change. Error bars show ± SD. Data was analysed by one-way ANOVA with Fisher’s LSD multiple comparisons test. UI=Un-infected. ***P<0.001, ****P<0.0001. [file Image_1.jpeg]

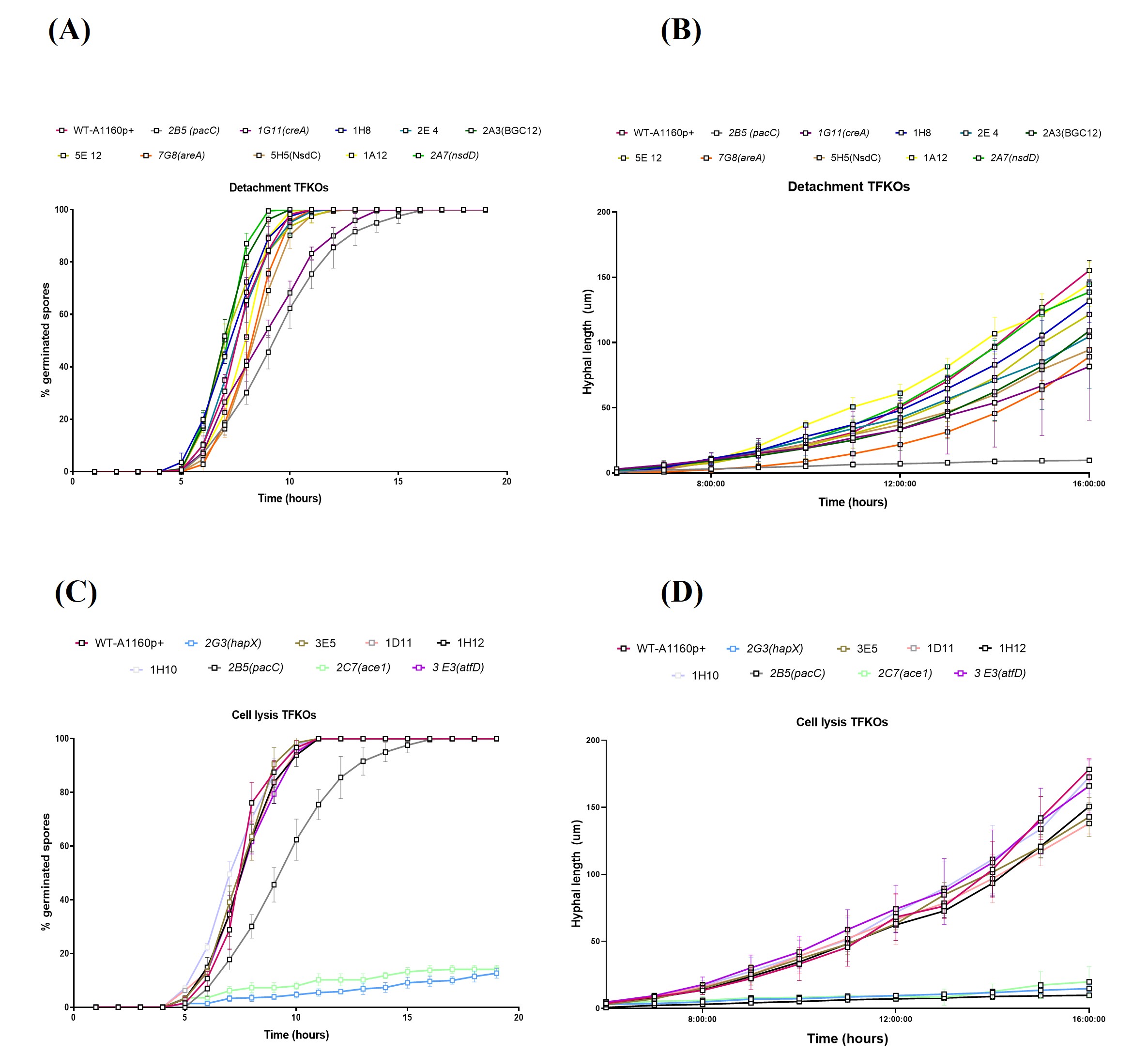

Supplement: Supplementary Figure 2 — Hyphal extension rate and germination potential of the A. fumigatus TFKOS measured by assessing growth using time-lapse confocal microscopy. (A) Percentage germination of the cell detachment mutants. (B) Hyphal extension rate of cell detachment mutants germination of the eRegs (cell detachment) mutants. (C) Percentage germination of the cell detachment mutants. (D) Hyphal extension rate of the cell lysis mutants. WT=A1160p+. Data was analysed by one-way ANOVA with Fisher’s LSD multiple comparisons test. Error bars show ± SD. *P<0.1, **P<0.01 ***P<0.001, ****P<0.0001. [file Image_2.jpeg]

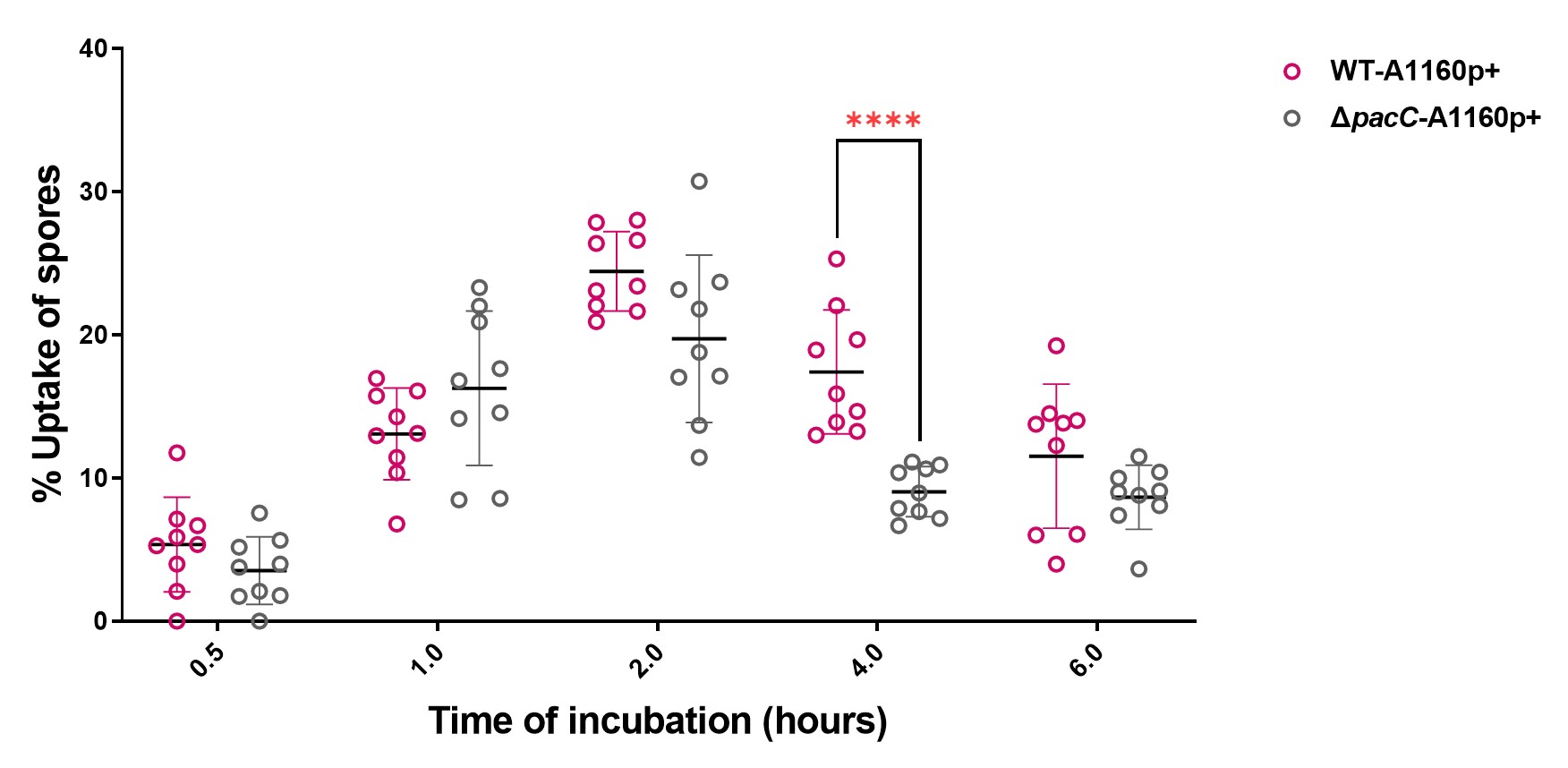

Supplement: Supplementary Figure 3 — Uptake capacity of A549 cells to the A. fumigatus parental strain (A1160p+) and ΔpacC strain over time. FITC stained spores incubated with A549 cells for 30min, 1 hour, 2 hours and 4 hours to enable A549 internalisation, after which the infected cells, were washed with PBS to stain the externally adherent spores with calcofluor white. Images were captured using the TCS-SP8 confocal microscope at 40x magnification Error bars show ± SD. Data was analysed by two-way ANOVA Sidak’s comparisons test of means. ***P<0.001, ****P<0.0001. Data shown is 3 technical reps with three biological replicates. [file Image_3.jpeg]
